# A high quality genome of the common swamp pitcher plant (*Nepenthes mirabilis*) using PacBio HiFi sequencing

**DOI:** 10.1371/journal.pone.0322885

**Published:** 2025-07-10

**Authors:** Christopher J. Jackson, Todd G.B. McLay, Gareth D. Holmes, Theodore R. Allnutt, Alastair S. Robinson

**Affiliations:** 1 Royal Botanic Gardens Victoria, Melbourne, Victoria, Australia; 2 School of BioSciences, The University of Melbourne, Parkville, Victoria, Australia; University of the Faroe Islands: Frodskaparsetur Foroya, FAROE ISLANDS

## Abstract

The genus *Nepenthes*, known commonly as tropical pitcher plants, are amongst the most recognisable carnivorous plants, capturing and digesting organic material in highly modified ‘pitcher’ leaves to acquire nitrogen and other valuable nutrients that are naturally scarce in their habitats. Here, PacBio HiFi long-read sequencing was used to assemble a near chromosome-level genome for a female specimen of *Nepenthes mirabilis*. We compare the genome organisation and gene content with a recently published chromosome-level assembly from a male specimen of the closely related *N. gracilis*, generated using Oxford Nanopore long-read sequencing and Hi-C scaffolding. We identify putative centromeres and large repeats in some *N. mirabilis* chromosomes that are absent from the *N. gracilis* assembly and examine the differences between the X and Y chromosomes, identifying a massive nuclear ribosomal repeat array in the *N. mirabilis* X-chromosome.

## Introduction

The field of plant genomics is currently undergoing a rapid and remarkable transformation. To date, genomes of 1,482 distinct plant species have been sequenced [1] but in the past three years alone (2021–2023), 2,373 genomes from 1,031 plant species, including 793 newly sequenced species, have been assembled [2]. The complexity of plant genomes, including frequent polyploidisation events and highly repetitive genomes, has historically posed significant challenges for genome assembly. Recent developments in sequencing technologies have enabled the generation of telomere-to-telomere (T2T) references for both simple and complex genomes [3]. These advancements reflect significant technological progress in sequencing and genome assembly, particularly the shift towards third-generation sequencing technologies, such as Pacific Biosciences’ continuous long reads (CLR) and Oxford Nanopore Technologies (ONT) sequencing platforms, which offer longer reads and greater accuracy, as well as genome scaffolding technologies such as Hi-C [4–6]. The assembly of T2T genomes has been particularly impactful in understanding the molecular mechanisms of character variation in agriculturally important plants, with ongoing improvements in the quality and completeness of these genomes [7,8]. Projects like the Earth BioGenome Project, which aims to sequence every named eukaryotic organism on the planet, highlight the potential of these advancements to address long-standing questions in phylogenetics, ecology, conservation, and agriculture, and enable deeper insights into the genetic underpinnings of plant adaptation and evolution [9–12].

Plant carnivory has long fascinated botanists as a rare and extreme adaptation to nutrient-poor habitats. Genomes of variable completeness are available for several carnivorous plant species, including examples from *Drosera* [13] and *Dionaea* [14] in the order Caryophyllales, and from non-Caryophyllales lineages such as *Cephalotus* (Oxalidales; [15]) and *Utricularia* (Lamiales; [16]), and these resources are valuable for comparative genomic studies across different trapping strategies and other related plant lineages. Further research on plant carnivory has the potential to provide insight into the molecular mechanisms underlying nutrient acquisition and the evolution of plant-animal interactions. Comparative genetic studies, including transcriptomics, proteomics, and genomics have revealed significant insights into carnivore evolution and the genetic basis of the carnivorous syndrome in plants, and identified a suite of proteins and related genes associated with the carnivorous syndrome [17,18].

*Nepenthes* Linnaeus ([19]: 955), known commonly as tropical pitcher plants, are amongst the most recognisable carnivorous plants, capturing and digesting organic material in highly modified ‘pitcher’ leaves to acquire nitrogen and other valuable nutrients that are naturally scarce in their habitats [20]. The monogeneric family Nepenthaceae comprises over 165 species [21,22] of dioecious, insect-pollinated lianas and sub-shrubs distributed primarily within the Malesian and Papuasian biogeographic regions—with centres of diversity on Borneo, Mindanao, and Sumatra—and outlying species that occur in Madagascar, the Seychelles, Sri Lanka, Meghalaya (India), and New Caledonia [23,24]. Recently, high-throughput sequencing methods—genome skimming, and DNA target capture and sequencing—have given rise to well-resolved phylogenies that resolve most *Nepenthes* species into major clades, and furthermore provide evidence of historic hybridisation events as well as an indication of the crown diversification of the genus to ca. 20 Mya [25,26]. Nepenthaceae sits within Caryophyllales, a diverse order of ca. 39 families [27] and ca. 12,500 species known for their cosmopolitan distribution and extreme adaptations, including drought-, cold- and halo-tolerance, CAM photosynthesis and, of course, carnivory [28]. The families are broadly divided into two major clades, the core Caryophyllales and the non-core Caryophyllales or ‘carnivore-clade’. Of the ca. 850 species of carnivorous plants recognised to date worldwide, ca. 53% occur in the carnivore-clade of Caryophyllales. Understanding the evolution of plant carnivory may lie within the genomes of members of the Caryophyllales.

Here, PacBio HiFi long-read sequencing was used to assemble a near chromosome-level genome for *Nepenthes mirabilis* (Lour.) Druce [29]. *Nepenthes mirabilis* is the most widely distributed species in the genus, ranging from continental Southeast Asia east to New Guinea, the Caroline Islands, and Far North Queensland, Australia [30]. A phyllode (= expanded petiole) transcriptome was also generated from Illumina short-read data and used to annotate the genome assembly. Owing to the increased rate of plant genome sequencing and the biological interest in *Nepenthes,* during our study highly fragmented genome assemblies from short-read data were published for *N. mirabilis* [31] and *N. alata* [32] (scaffold number and N50 are 159,555/ 10.2 kb and 170,420/ 6.8 kb, respectively), as well as a chromosome-level assembly from the closely related *N. gracilis,* generated using Oxford Nanopore long-read sequencing and Hi-C scaffolding [33]. The latter study presented a comprehensive investigation into the evolution of the *Nepenthes* genome, including discovery of a decaploid genome with subgenome dominance (rejecting previous hypotheses of an octoploidal or hexadecaploidal genome [34]), as well as investigations of the role of polyploidy and subsequent gene diversification in carnivory-related trait development. Moreover, whereas most plants are functional hermaphrodites, *Nepenthes* is dioecious – that is, individual plants are either male or female. In *Nepenthes*, this sex determination involves an XY sex chromosome system [35]. The *N. gracilis* study identified the male sex-chromosome and putative genes involved in dioecy on the Y-chromosome. Rather than repeating these investigations with our assembly (and comparatively more limited dataset), we set out to investigate differences between the two genome assemblies based on different sequencing technologies and assembly approaches; here we present a comparison between the chromosome-level assemblies from our *N. mirabilis* assembly and *N. gracilis* [33]. Through this process we identified putative centromeres and large repeats in some chromosomes that are absent from the *N. gracilis* assembly. We also examined the differences between the X and Y chromosomes and identified a massive nuclear ribosomal repeat array in the X-chromosome assembly of *N. mirabilis*.

## Materials & methods

### Plant materials, DNA & RNA extraction, and sequencing

Material for sequencing was harvested from a *N. mirabilis* accession from the Babinda area, Queensland, Australia. The plant was sourced from Cairns Botanic Gardens and cultivated under glasshouse conditions at Royal Botanic Gardens Victoria, Melbourne; no permits were required as part of this work. A voucher of the female accession used for sequencing is lodged at the Australian Tropical Herbarium (CNS 148197.1). For DNA isolation, approximately 20 g of young pitcher (= leaf) and phyllode (= expanded petiole) material was cleaned and surface sterilised before being ground under liquid nitrogen. DNA was isolated from the powdered material based on the method described by McLay et al. (2022) including two pre-washes in D-sorbitol buffer, a CTAB lysis buffer with 1.4 M NaCl, and a final elution with 100 µl ultrapure H_2_0. The resultant DNA isolate was assessed for concentration with a Qubit 3 fluorometer (Invitrogen, USA), purity with a NanoDrop Lite spectrophotometer (Thermo Scientific, USA), and integrity using a TapeStation 4150 (Agilent, USA).

For PacBio HiFi sequencing, approximately 10 μg of high molecular weight (HMW) DNA was used as input for SMRT bell template preparation, following enzymatic clean-up, mechanical shearing, DNA repair, and Blue Pippin clean-up for fragment size enrichment. Genomic DNA sequencing was performed by Genomics WA, Perth, Australia. For RNA isolation, tissue from a young phyllode was washed, then placed in chilled RNAlater (Invitrogen) immediately after harvest. The material was then ground under liquid nitrogen. A NucleoSpin RNA Plant and Fungi kit (Macherey-Nagel, Germany) was used to isolate RNA from 100 mg of tissue following the manufacturer’s protocol, but with a modified lysis buffer which included 400 µl buffer PFL and 50 µl buffer PFR (manufacturer supplied), 100 µl Fruit-mate for RNA Purification (Takara Bio, USA) and 2% (v/v) β-mercaptoethanol. The resultant isolate was purified with a NucleoSpin RNA Clean-up Mini kit (Macherey-Nagel) and assessed for purity, concentration and integrity as described above. A library was prepared using an mRNA library preparation kit and sequenced on a NovaSeq SP 2x150 bp flow cell at the Australian Genome Research Facility (AGRF), Melbourne, Australia.

### Long read genome assembly

Hifiasm version 0.16.1-r375 [36,37] was used to assemble PacBio CCS reads, generating 832 contigs totalling 1,260,180,061 bases with an N50 of ~18.32 Mb (see S3 Table in S2 File for further details). Then, purge_dups v0.0.3 [38] was used to remove haplotigs and heterozygous contig overlaps using PacBio CCS reads. In addition, a script to calculate Shannon’s entropy was used to identify 6 contigs that consisted only of simple repeats which were also excluded. Finally, contaminant contigs originating from bacteria or fungi were removed (see section “Genome quality control” below for methods), along with contigs of mitochondrial or plastid genome origin, leaving 67 contigs totalling 1,003,663,055 bases with an N50 of ~20.08 Mb. CCS data were used to estimate the genome size with JellyFish [39] version 2.3.0 and GenomeScope [40] version 1.0.0.

For details of bioinformatic commands, settings, and scripts, see the GitHub repository at https://github.com/chrisjackson-pellicle/nepenthes_genome_manuscript.

### Repetitive elements annotation

A non-redundant transposable element (TE) library was generated using the EDTA pipeline version 1.8.4 [41]. To assist in filtering out gene-related sequences from the final TE library, EDTA was provided with nucleotide transcript sequences from the closely related taxon *Beta vulgaris* (see [42]). The TE library output contained 3,892 sequences. TEs were then classified using the Transposon Classifier “RFSB” tool from TransposonUltimate version 1.0 [43], with the option [-mode classify]. Custom Python scripts were used to relabel the EDTA TE library sequences with the TransposonUltimate classification, with the following amendment: in cases where the TransposonUltimate classification probability of a sequence to either Class I (retrotransposons) or Class II (DNA transposons) was less than 0.5, the sequence was labelled as ‘unclassified’. The relabelled TE library was used to soft-mask the genome assembly using RepeatMasker version 4.1.0 [44], and the output file produced by RepeatMasker was used to generate an annotation table (Supplementary Table S4 File) using the RepeatMasker script “buildSummary.pl”. To further investigate repeats, heatmaps of Fourier spectra showing locations of repeats (including their length and how perfectly they repeat), together with plots of the rolling sum of repeat abundance and the Shannon diversity values for repeats, were produced using RepeatOBSserverV1 [45]

### Gene prediction and functional annotation

Structural gene annotation of the TE-masked genome assembly was performed using the BRAKER2 pipeline [46]. ETP-Mode was used, which accepts evidence hints in the form of spliced RNAseq alignments and spliced protein alignments. RNA sequence reads were trimmed and filtered using Trimmomatic [47] v0.39 (see Supplementary Table S2 File for filtering statistics). To generate RNA-seq spliced alignment hints, quality filtered RNAseq data were aligned to the soft-masked genome using STAR [48], and the resulting BAM file was supplied to BRAKER2. To generate a database of proteins for BRAKER2 input, the OrthoDB v10.1 [49] catalogue of orthologous protein-coding genes was filtered for Viridiplantae sequences (NCBI taxon ID 33090) and the filtered protein families were supplied to BRAKER2. To remove putative transposons (i.e., those that were not identified with the EDTA pipeline described above) from the resulting gene set, Pfam domains were identified in the gene nucleotide sequences, and corresponding domain text descriptions were extracted from the Pfam website (http://pfam.xfam.org/). For each gene, Pfam descriptions were searched against a list of transposon-related terms (transcriptase, transposase, gag, env, transposon, repetitive element, RNA-directed DNA polymerase, pol protein, non-LTR retrotransposon, mobile element, retroelement, retrovirus, Retroviral, group-specific antigen). Where more than half of the Pfam domains in a gene had matches to one of these terms, the gene was flagged as a potential transposon and removed from the BRAKER2 predicted gene set. Finally, any gene that has no external support (i.e., RNAseq or OrthoDB protein alignment evidence) during BRAKER2 gene prediction, and lacked a functional annotation (see below), was removed; see S8 File for the corresponding sequences. See the GitHub repository for full methods. Completeness of the resulting predicted protein-coding gene set was assessed using BUSCO [50] v4.0.4 searching against the eudicotyledons_odb10_odb10 (2,121 genes) database.

The predicted genes were assigned functions using three methods. Firstly, Pfam domains were determined by searching the protein dataset against v33.1 of the PfamA.hmm database [51] using the hmmsearch program from HMMER v3.2.1 [52] (S5 Table in S2 File). Secondly, KEGG Orthology (KO) annotation of the filtered BRAKER2 predicted gene set (29,806 genes) was performed using the BLAST algorithm implemented in BlastKOALA [53] via the KEGG website (https://www.kegg.jp/blastkoala/), see S6 Table in S2 File. Finally, the filtered BRAKER2 predicted gene set was annotated using InterProScan [54] version 5.50–84.0 (see S3 File). A Venn diagram to compare the genes functionally annotated by each methodology was produced using TbTools [55].

### Genome quality control

To remove contaminant contigs originating from bacteria or fungi, the following steps were taken. First, the amino acid sequences for BRAKER-predicted genes were recovered from each contig and compared to the NCBI nr database using NCBI or DIAMOND BLAST. The lineage for the top BLAST hit for each gene was then recovered. For each contig, the BLAST hit lineages were manually examined; if these lineages were predominantly bacterial or fungal, the corresponding contig was removed from the *Nepenthes mirabilis* genome assembly. To identify contigs of mitochondrial or plastid genome origin, HiFi reads were mapped against this filtered *N. mirabilis* assembly using minimap2 [56] and the resulting *.bam file was used as input to MetaBAT [57]. Contigs with the highest read depth were identified from the per-contig read depth report produced; the top 14 contigs had a total average read depth ranging from ~411 – 3368x, whereas the contig with the next highest read depth was ~ 58x. Each of the 14 high read depth contigs was identified as being of mitochondrial or plastid genome origin by manual BLAST searches against the publicly available *N. mirabilis* plastid genome (accession NC_041271) and the *N. ventricosa* *×* *N. alata* mitochondrial genome (accession MH798871); these contigs were removed from the assembly, leaving 67 contigs. The full gene prediction pipeline was then re-run on the 67-contig assembly as described above, beginning with repetitive element annotation using EDTA.

For each stage of the genome assembly, statistics were generated using the software assembly stats (https://github.com/sanger-pathogens/assembly-stats). A k-mer spectra plot was generated from the final 67 contig assembly and the PacBio CCS data using KMC [58] (see Fig S1A in S1 File).

The presence of putative telomeres in the 67 contig assembly was assessed using the software tidk [59]. A candidate telomeric repeat sequence was identified using the `explore` module, and the location and repeat number within each contig was assessed with the `search` module. Tandem repeats in all contigs were identified using Tandem Repeats Finder v0.0.3 [60] with the parameters ‘2 3 3 80 10 500 2000’. All-*vs*-all contig alignments were generated for contig pairs using LASTZ [61] version 1.04.22 in MAF format using parameters --step = 20 --strand=both --hspthresh = 75000, and alignments were visualised as dot plots.

### Organelle genome assembly and NUPT/NUMT identification

HiFi reads were mapped to published organelle genomes of close relatives using minimap2 v2.24 in ‘map-hifi’ mode (plastome: *Nepenthes khasiana* NC_051455, *N.* *×* *ventrata* MH923233, *N. ventricosa* MK758110, and a previously published *N. mirabilis* NC_044185; Mitochondria: *N.* *×* *ventrata* MH798871, and *N. ventricosa* *×* *N. alata* NC_039531). Mapped reads were sub-sampled to ~100X depth and assembled using Hifiasm. Assembled organelle genomes were compared to published genomes using BLAST and Mauve [62] within Geneious Prime v2023.0.4 (http://www.geneious.com/).

Plastid contigs were annotated using Chloe (https://github.com/ian-small/chloe). Annotated protein encoding genes were BLAST (blastp) aligned to a database of all Caryophyllales plastid RefSeq genes (June 2024, NCBI) and the mean and maximum % alignment length among all top-hit genera (mean of means within all genera – 20 hits per query) recorded. If a gene’s mean or maximum alignment length was < 75% it was manually examined in Geneious and recorded as a pseudogene; in the absence of 3D structure and function assessments for these partial genes, which is beyond the scope of this paper, this pseudogene assignment is arbitrary. Mitochondrial contigs were annotated using GeSeq [63] with BlatX, BlatN, tRNAscan and NCBI RefSeq references: all Caryophyllales mitochondria (June, 2024; 25 genomes). As above for plastid genes, mitochondrial genes were aligned to a BLAST RefSeq database to check for pseudogenisation. The mitochondrial contigs were also annotated with plastid genes using Chloe in the same way as the plastid genome.

Organelle genome to nuclear genome transfers were classified into four categories: NUMTs – nuclear mitochondrial transfers; NUPTs – nuclear plastid transfers; NUM/PTs - transfers of genome regions which could not be distinguished as either plastid or mitochondrial, e.g., originating from genes present in both organelle genomes; and NUMPTs – complex transfers containing mitochondrial and plastid regions in tandem. To identify each type in the nuclear genome assembly, the assembled mtDNA and plDNA genomes were used as a query in BLAST searches of each nuclear scaffold. BLAST hits were filtered to include only those with an alignment length greater than 100 bp and with a minimum identity of 85%. Nested hits were concatenated into single NUPT/ NUMT/ NUMPT region where there was less than 300 bp between BLAST hits. HiFi reads were mapped to NUMT/ NUPT containing contigs to identify if any insertion site borders coincided with read ends or low coverage (therefore indicating that the insertions could be artifacts of assembly).

### Comparison to the *Nepenthes gracilis* genome assembly

Synteny between the *Nepenthes mirabilis* genome reported here and the published *N. gracilis* genome [33] was investigated as follows. The male *N. gracilis* assembly annotation data were filtered to retain annotations corresponding to the 40 chromosome-scale scaffolds, and the filtered data was converted from gff3 to bed format. The predicted CDS sequences from the male *N. gracilis* assembly were also filtered to retain genes from the 40 scaffolds and translated to produce predicted protein sequences. Genome annotation data for the 67 contig *N. mirabilis* genome were converted from gff3 to bed format, and bed annotations and predicted protein data from both taxa were used as input to GENESPACE version 1.4 [64] to produce a riparian plot. Close syntenic matches between *N. gracilis* scaffolds and *N. mirabilis* contigs were identified from the plot, nucleotide alignments were generated for contig pairs using LASTZ version 1.04.22 in MAF format using parameters --step = 20 --strand=both --hspthresh = 75000, and alignments were graphed as dot plots. In addition to comparisons with the closely related *Nepenthes gracilis* described above, synteny was examined with two other chromosome-scale Caryophyllales genomes: *Beta vulgaris and Fagopyrum tataricum* (GenBank accessions: GCF_000511025.2 and GCA_002319775.1 respectively). GENESPACE (v1.2.3) was used to find syntenic blocks between chromosomes/ contigs and produce a riparian plot [64].

### Identification of orthologous gene families

**Fig 1:**
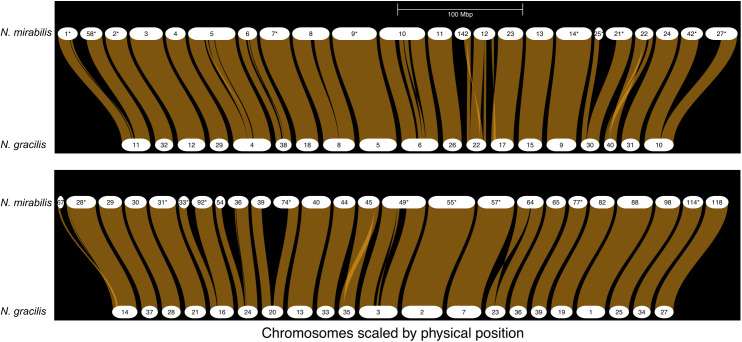
Schematic of gene order synteny on nuclear genome contigs from *N. mirabilis* (top) and *N. gracilis* (bottom). Only the 48 (of 67) *N. mirabilis* contigs with detected syntenic matches to *N. gracilis* contigs are shown. White horizontal bars represent contigs (*N. mirabilis*) or scaffolds (*N. gracilis*). Numbers labelling the *N. mirabilis* contigs have been truncated from the full contig name (e.g., “39” corresponds to “ptg000039l_1”) for clarity. An asterisk after a *N. mirabilis* contig number indicates that it has been reverse-complemented to match the orientation of the corresponding *N. gracilis* contig. Light brown braids connecting contigs represent regions of gene synteny.

To compare the diversity and abundance of *Nepenthes mirabilis* gene families to other Caryophyllales species and angiosperms more broadly, gene families (orthogroups) were calculated using OrthoFinder v2.3.12 [65]. Eighteen species including *N. mirabilis* were included in the analysis (S7 Table in S2 File); protein sets containing a single isoform for each gene were used. OrthoFinder was run using default settings. Visualisations of selected OrthoFinder results were generated using a modified version of the script Fig_1_ResultsOverview.py, originally available at https://zenodo.org/record/1481147#.X5ognVlxXUI, see also the GitHub repository for this study.

## Results and discussion

### Genome sequencing and assembly of the *Nepenthes mirabilis* nuclear genome

To produce a genome for *Nepenthes mirabilis*, approximately 33.3 Gb of PacBio CCS genomic sequence data was generated (see S1 Table in S2 File for Circular Consensus Sequence (CCS) statistics). In addition, 68.9 Gb of RNAseq Illumina paired-end read sequence data were produced. The haploid genome size of *N. mirabilis* was estimated to be 0.93 Gb using GenomeScope with the PacBio CCS data, with an estimated heterozygosity of 2.15% (including a repeat length of 258 Mb, see S8 Table in S2 File). The data for this study have been deposited in the European Nucleotide Archive (ENA) at EMBL-EBI under accession number PRJEB86749 (https://www.ebi.ac.uk/ena/browser/view/PRJEB86749).

The initial long-read assembly was 1.26 Gb with an N50 of 18.3 Mb. Haplotigs and heterozygous overlaps were removed, reducing the total assembly size to 1.13 Gb. Contaminant contigs from fungal and bacterial sources were then removed, along with contigs derived from the *N. mirabilis* organelle genomes. The final assembly consisted of 67 contigs totalling ~1 Gb, with an N50 of ~20 Mb and ~27x read coverage (see S9 Table in S2 File for a comparison with the other carnivorous plant genomes used in this study). While this genome size correlates well with the *N. gracilis* genome assembly (see below for a detailed comparison), it is approximately four times the size estimated for the *N. pervillei* genome using flow cytometry [66]. The reason for this discrepancy is unclear; although *N. pervillei* is sister to the rest of the genus [26] it shares the same chromosome number with all other *Nepenthes* species examined [34], and so either the flow cytometry estimate is incorrect or there has been large-scale reduction of the *N. pervillei* chromosomes following its divergence (or, alternatively, large-scale expansion in other *Nepenthes*).

### Comparison of *N. mirabilis* and *N. gracilis* assemblies

As this study was being conducted, a genome assembly for *Nepenthes gracilis* was published [33], generated from Hi-C scaffolding of contigs assembled from Oxford Nanopore long-read data. Of the 176 scaffolds produced by Saul et al. [33], 40 are chromosome scale, consistent with the number of chromosomes reported for this species; the number of individual contigs in the 40 scaffolds ranges from 6–53. Internal genome synteny analyses revealed that *N. gracilis* is decaploid (i.e., with 5 subgenomes), with a basic chromosome number of x = 8, and that the gene content is highly fractionated. Homoeologous chromosome groups showed a clear distinction between one dominant, gene-rich chromosome and four recessive, gene-poor chromosomes, indicating fractionation bias [33]. To investigate biological differences between the 67 contigs in our *N. mirabilis* assembly and the 40 *N. gracilis* scaffolds, as well as differences potentially arising from the different sequencing technologies and assembly approaches used, we examined both gene-order and nucleotide-alignment synteny (Fig 1 and Fig S1D in S1 File, respectively).

In total, 48 *N. mirabilis* contigs show strong synteny with *N. gracilis* scaffolds, with all 40 *N. gracilis* scaffolds having at least one match. Of the 48 *N. mirabilis* contigs, 33 (~69%) have a 1:1 relationship (i.e., a single *N. mirabilis* contig has a full-length match to a single *N. gracilis* scaffold, see Figures E and D a in S1 File). In most cases (25/33) no rearrangements are observed, whereas *N. mirabilis* contig ptg000064l_1 appears to have an inversion of one chromosome arm relative to *N. gracilis* scaffold 23; in other cases, more complex rearrangements are observed (e.g., *N. mirabilis* contig ptg000022l_1). A further 12 *N. mirabilis* contigs are matched in pairs to a single *N. gracilis* scaffold each. As shown in S1D (b) and S1F Figs in S1 File, synteny of each contig pair is lost towards the centre of the corresponding *N. gracilis* scaffold. Except for *N. mirabilis* contigs ptg000039l_1 and ptg000074l_1, which correspond to the *N. gracilis* sex-chromosome (see below), these non-syntenic regions correspond to tandem repeat arrays with an identical or near identical 36 bp repeat unit (Fig S1H in S1 File, green dots). Given that genomic regions with long repeats are more difficult for genome assemblers to reconstruct, it is likely that these *N. mirabilis* contig pairs represent each arm of a contiguous chromosome *in vivo*, and that the assembler software was unable to fully reconstruct the long repeat-rich sequence that connects the chromosome arms. Examination of the corresponding regions in the homologous *N. gracilis* scaffolds did not detect similar repeat arrays, either in terms of length or repeat unit size. In four of five cases, the *N. gracilis* scaffold contained one or more contig junctions in these regions, and homology between the *N. mirabilis* contig arms and the *N. gracilis* scaffold did not extend across these junction(s), perhaps due to the equivalent repeats not being assembled and/or scaffolded in the *N. mirabilis* scaffolds. Given the difficulties of bioinformatic reconstruction of such regions, the number of repeat units in the *N. mirabilis* contigs should be viewed with caution, and the true repeat number *in vivo* will require further investigation. Nonetheless, the partial reconstruction of these repeat regions highlights the ability of PacBio HiFi reads and appropriate assembly software to reveal additional insights into genome structure. Overall, it appears there is remarkable similarity between the *N. mirabilis* and *N. gracilis* genomes both at the gene synteny and primary nucleotide sequence level and, consistent with the stable chromosome number observed across *Nepenthes* species [33], *N. mirabilis* also contains a decaploid genome with five subgenomes. In contrast, gene synteny analysis comparing *N. mirabilis* to the Caryophyllales taxa *Beta vulgaris* and *Fagopyrum tataricum* synteny show high levels of rearrangement (Fig S1N in S1 File).

To further assess the completeness of our genome assembly, we searched the *N. mirabilis* contigs for telomeric repeats, as the presence of telomeres at both termini of a given contig likely indicates near end-to-end reconstruction of the corresponding chromosome. Typical plant-type telomeres with the repeat sequence ‘TTTAGGG’ [67] were detected—the repeat number and location of the telomeric sequence across the 67 *N. mirabilis* contigs is shown in Fig S1C in S1 File. Of the 33 contigs that have a 1:1 relationship with a *N. gracilis* chromosome scaffold, telomeric repeats were found at both termini of 18 contigs and at one terminus of 13 contigs. Further, telomeres were observed at the distal end of an additional 12 contigs representing putative chromosome arm pairs. As these pairs likely comprise complete sequence except for a repeat array (see above), these data suggest that the *N. mirabilis* assembly contains 30 complete or near complete telomere-to-telomere chromosomes, and likely near complete sequence (i.e., missing one telomere) for an additional 13 chromosomes. The relatively complete and contiguous contigs in the *N. mirabilis* assembly (and the full or partial assembly of large repeats arrays that are absent in the *N. gracilis* scaffolds) account for the difference observed in the sizes of *N. mirabilis* and *N. gracilis* assemblies (~973 Mb and ~747 Mb, respectively, for the main 48 and 40 contigs/scaffolds).

The large proportion of contiguous and complete chromosomes in the *Nepenthes mirabilis* assembly also facilitated identification of putative centromeres; these often-repetitive regions can be missing or only partly reconstructed in genome assemblies comprising scaffolds. Plant centromeres can exhibit extraordinary diversity in size, structure and composition across different taxa [68], but in many plants they are found within long arrays of tandemly repeated sequence, called satellites [69]. The satellite monomer can differ in length, but often ranges from 100–400 bp, and satellite arrays can be megabases in scale. Arrays interspersed with Long Terminal Repeat (LTR) transposons have also been detected [70,71]. To identify candidate centromeres in our *N. mirabilis* contigs, we used several approaches: firstly, given the highly repetitive nature of many plant centromeres, we searched for long regions of repetitive sequence shared across all putative full-chromosome contigs by generating all-vs-all alignment dot plots. For many contig dot plots we observed a distinct rectangular region filled with alignment matches, which is typical of low-complexity repetitive regions. This rectangle usually occurs towards the middle of a given *N. mirabilis* reference chromosome, and the nucleotide coordinates of the rectangle relative to the reference are consistent across contig comparisons (see Fig S1I in S1 File for an example, and S4 File for full results). These results indicate that many contigs contain a large, low-complexity region with strong sequence similarity between different contigs (at least at the subregion level, if not contiguous sequence identity). Examination of alignments from these dot plot regions revealed that they correspond to a subset of *N. mirabilis* transposons, comprised largely of LTR ‘Gypsy’ retrotransposons, that are mainly localised to the large low-complexity regions and occur in multiple (often fragmented) copies (see Fig S1J in S1 File for an example, S5 File for full results, and S12 Table in S2 File). The low complexity regions are also gene-poor (S5 File, blue dots), consistent with the rare single/low copy genes observed in the centromeres of other plants [71]. We therefore hypothesise that these regions represent centromeres or centromere-adjacent regions in the *N. mirabilis* contigs. Interestingly, several contigs exhibit multiple distinct dot plot rectangles (e.g., ptg000005l_1, ptg000049l_1), and these regions all contain transposons from the subset identified above. All-vs-all dot plots of the *N. gracilis* scaffolds show similar distinct dot plot rectangles for some chromosomes (e.g., scaffold12, scaffold19, see S7 File), but these are often smaller than their counterparts on syntenic *N. mirabilis* contigs, or absent, perhaps indicating only partial reconstruction of these repetitive regions in *N. gracilis*.

To search for tandem-repeat satellite arrays that might be associated with putative centromere regions in *Nepenthes mirabilis*, we identified and mapped tandem repeats across all contigs, filtered to remove repeats that overlapped with transposons (S5 File). While no consistent pattern was observed across all contigs, some contigs exhibited distinct features. For example, in contig ptg000002l_1 the ~ 0.5 Mb region upstream of the putative centromere-associated transposon area comprises dense arrays of tandem-repeat satellite DNA with different repeat unit and array sizes. In contig ptg000011l_1, on the other hand, the centromere-associated transposons are interspersed with multiple satellite arrays with different, but short (<100 bp) repeat units, and overlapping and continuing downstream of the transposon region is a region with multiple repeat arrays with long repeat units (>1000 bp) but fewer unit copies. In four sets of paired contigs corresponding to chromosome arms (ptg000001l_1/ptg000058l_1, ptg000042l_1/ptg000027l_1, ptg000033l_1/ptg000092l_1, ptg000054l_1/ptg000036l_1), the centromere-associated transposon region is interrupted by a long satellite repeat, and the 36-base repeat unit is identical (or near identical) in these three chromosomes. A fourth contig pair also contains this satellite repeat, but it occurs interspersed between multiple blocks of centromere-associated transposons (contigs ptg000025l_1 and ptg000021l_1).

To further investigate the putative centromere regions in *N. mirabilis* we used RepeatOBServerV1 [45] to generate heatmaps showing the locations of repeats (including their length and how perfectly they repeat) within each contig, as well Shannon diversity value plots and overall repeat abundance across each contig (S6 File). Overall, results are consistent with the centromere positions identified by the dot plot rectangles described above (S6 File and Table S17 in S2 File). Some contigs exhibit a bright vertically blurred region in their heatmaps at these contig positions, together with a maximum peak in their repeat abundance plots (e.g., ptg000002l_1, ptg000004l_1, ptg000006_1); these results indicate clusters of non-tandem repetitive elements such as retrotransposons [45]. Almost all contigs have a minimum valley in their Shannon diversity plots at the same contig location as their corresponding dot plot rectangles, indicating low diversity of repeat lengths in these regions. Such minima often correspond to regions of dense tandem repeats [45]; further investigation will be required to clarify the relationship between the RepeatOBServer results and the consistent clusters of retrotransposons detected using the dot plot approach. Overall, it appears that centromeres in *N. mirabilis* exhibit some common patterns but also substantial variation, consistent with centromeres observed in other organisms [70].

### The *Nepenthes mirabilis* female sex chromosome and rDNA operon tandem repeat

**Fig 2:**
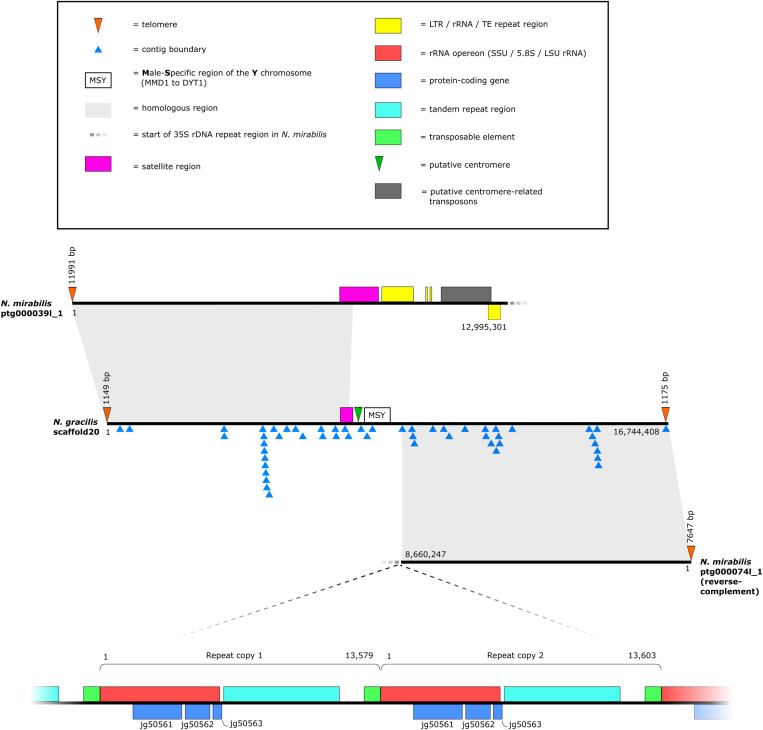
Schematic of the *N. gracilis* Y-chromosome and the *N. mirabilis* X-chromosome.

Whereas most plants are functional hermaphrodites, *Nepenthes* is dioecious—that is, individual plants are either male or female. This sex determination is under genetic control in *Nepenthes* and involves an XY sex chromosome system [35]. A scaffold corresponding to the male sex (Y) chromosome was identified in *N. gracilis* [33], and contains a sex-specific, fully non-recombining region termed the male-specific region of the Y, or MSY. The MSY region encompasses ~1 Mb in the scaffold, and contains three genes with known function: DYSFUNCTIONAL TAPETUM 1 (DYT1), the only fully male-linked gene known to date in *Nepenthes*; an ortholog of MALE MEIOCYTE DEATH 1 (MMD1), which encodes a PHD-finger transcription factor whose loss causes male meiotic defects; and a male-specific copy (LFY-Y) of the LEAFY (LFY) gene which, in hermaphroditic angiosperms, encodes a plant-specific transcription factor that assigns the floral fate of meristems. Notably, the MSY encompasses two contig junctions within the *N. gracilis* scaffold (Fig 2, blue triangles), and so the exact size of the region requires further verification.

In our genome assembly from a female *N. mirabilis* plant, we identified two contigs corresponding to the *N. gracilis* Y scaffold, which together represent the female X-chromosome. As shown in Fig 2, as well as in gene synteny and dot plot analyses (Figs S1D (b) and S1F in S1 File), the regions of homology between the two *N. mirabilis* contigs and the *N. gracilis* scaffold correspond to each arm of the scaffold, but as expected they do not encompass the *N. gracilis* MSY region, further corroborating this position as the sex-determining region in this genus. *Nepenthes mirabilis* contig ptg000039l_1 corresponds to the ‘left’ chromosome arm relative to the *N. gracilis* scaffold, and the reverse complement of contig ptg000074l_1 corresponds to the ‘right’ arm. Telomeric repeats are present at the left and right termini of *N. mirabilis* contigs ptg000039l_1 and ptg000074l_1, respectively. A telomeric repeat is also present at the ‘left’ terminus of the *N. gracilis* scaffold, whereas at the ‘right’ terminus a telomeric repeat occurs at a contig terminus and is followed by a final ~73 kb contig; this latter contig may represent a misplacement during Hi-C scaffolding.

For *Nepenthes mirabilis* contig ptg000074l_1, the boundary of homology proximal to the *N. gracilis* MSY occurs at a contig terminus in the *N. gracilis* scaffold. We also examined homology between contig ptg000074l_1 and a *N. gracilis* X-chromosome scaffold that was generated by scaffolding contigs from a female plant assembly using male Hi-C data [33]; while this extends the region of homology by ~6.5 kb, homology is again lost at a contig terminus in the *N. gracilis* female scaffold, perhaps indicating that the genome assembly software had difficulty extending contigs beyond this point during *N. gracilis* assembly (see below for a discussion of potential causes). Following the region of homology with the *N. gracilis* Y scaffold, contig ptg000074l_1 contains a stretch of ~28 kb that is rich in LTR retrotransposons, and finally terminates with a very long (11.4 Mb) repeat region with a repeat unit containing the 35S rRNA operon (see below).

For *Nepenthes mirabilis* contig ptg000039l_1, the precise boundary of the homologous region proximal to the *N. gracilis* MSY is difficult to determine as it occurs within a satellite region that in *N. gracilis* comprises multiple Hi-C-scaffolded contigs (Fig 2, blue triangles). Following the satellite region, contig ptg000039l_1 contains three clusters (~1 Mb, 65 kb and 380 kb, respectively) with multiple copies of the 35S rRNA operon genes, but these are not arrayed in highly similar tandem repeats as in true 35S rRNA operon arrays (see below). Instead, they often occur as fragments that are spaced relatively sparsely; sequence identity is divergent compared to copies in the true operon arrays, and they can occur as subclusters on both forward and reverse strands. The two smaller clusters are separated by a ~ 1.7 Mb region containing the putative centromere-related transposons as observed in most other chromosomes (see above). Twenty-seven annotated genes occur scattered throughout these regions, but closer examination suggests that they are largely pseudogene fragments (see S13 Table in S2 File). As in contig ptg000074l_1, the non-telomere terminus of contig ptg000039l_1 terminates with a rRNA operon repeat region ~2.4 Mb long. Together, these data suggest that the *N. mirabilis* contigs provide telomere-to-telomere coverage of most of the X-chromosome, but that the X-chromosome contains a long tandem repeat of the rRNA operon that prevented the genome assembler from producing a single contiguous chromosome. Interestingly, the rRNA operon array in contig ptg000074l_1 is interrupted by a ~ 50 kb NUMPT insertion containing both mtDNA genes and plDNA gene fragments.

In eukaryotes, ribosomal DNA (rDNA) comprises genes for four structural ribosomal RNAs (rRNA)—the 5S, 5.8S, 18S and 28S/26S/25S rRNAs. The 5S rRNA is encoded by a 5S DNA unit, whereas the 5.8S, 18S and 28S/26S/25S rRNAs are encoded by a single rDNA operon referred to as the 35S rDNA in plants [72]. Both the 5S and 35S rDNAs are typically arranged as tandem repeats in the genome, with the copy number varying from several to many thousands, and these tandem repeats can occur at one or many genomic loci. The 5S genes are often physically separated from the 35S (the S-type, or Separate arrangement), although they can be linked (the L-type, or Linked arrangement) [72]. In the *Nepenthes mirabilis* X chromosome contigs, the 35S rDNA tandem repeat comprises ~1200 copies, with ~200 in contig ptg000039l_1 and ~1000 in contig ptg000074l_1, although these numbers should be taken as provisional both because the repeat region is not fully assembled, and because genome assemblers can have difficulty in accurately assembling such repeat areas. We searched the *N. gracilis* Y-chromosome scaffold for evidence of a similar 35S rDNA repeat, but only a single repeat unit was detected (containing only a fragment of the 28S/26S/25S rRNA) located on the contig upstream of the MMD1 gene in the MSY region. Given the highly fragmented nature of the *N. gracilis* scaffold around this area (i.e., it comprises multiple contigs scaffolded with Hi-C data, Fig 2, blue triangles) it is difficult to know whether the 35S rDNA tandem repeat is absent in the *N. gracilis* Y-chromosome, or whether the genome assembler failed to assemble the region (that is, if present and correctly assembled it would be located between one of the contig junctions). Previous karyotyping in *Nepenthes* suggests that the sex chromosomes are homomorphic [34] indicating the latter scenario is more likely. Notably, however, a heterogeneous location of the 35S rRNA operon in XY sex chromosomes has been previously detected in the dioecious angiosperm *Spinacia oleracea* [73], the liverwort *Marchantia polymorpha* [74], and is also known in some animals [75–78]; in these organisms, the rRNA operon was detected on the X chromosome but not the Y.

To search for candidate rDNA operons in *Nepenthes gracilis* and to further characterise the distribution of rDNA tandem repeats in *Nepenthes* more broadly, we searched the complete *N. mirabilis* and *N. gracilis* genome assemblies for rRNA genes. In *N. mirabilis*, two main additional regions containing multiple 35S rRNA genes were detected (See Tables S15 and S16 in S2 File for full details). The first of these (ptg000080l_1) corresponds to a short ~215 kb contig comprised entirely of 35S rDNA repeats (18 copies in total with a length range of ~10–12.5 kb) which is not part of the main chromosome-scale contig assembly. The second rRNA repeat region occurs in contig ptg000049l_1, which corresponds to *N. gracilis* scaffold3. The repeats occur in two distinct regions ~72 kb and ~1.7 Mb long, respectively. While these subregions contain some copies of the full canonical 35S rDNA unit, the overall pattern of rRNA gene organisation differs from the *N. mirabilis* X-chromosome repeat, with many direct gene repeats, gene fragments, and longer intergenic regions. In *N. gracilis* three main candidate 35S rDNA operon regions were detected in scaffold41, scaffold3 and scaffold17. Scaffold41 is ~ 1.4 Mb long and is not included in the 40 chromosome-scale scaffolds—the rRNA genes in this scaffold occur in two regions, and the majority are not organised in canonical 35S rDNA repeats. Similarly, the rRNA genes in scaffold3 and scaffold17 do not occur in typical 35S rDNA repeats; scaffold17 largely matches *N. mirabilis* contig ptg000023l_1, but no rDNA genes were detected in the latter. Overall, it appears that the X-chromosome contains the principal 35S rRNA operon repeat locus in *N. mirabilis*, whereas no candidate for a typical 35S rRNA operon repeat was detected in *N. gracilis*. The location of this operon array in *N. mirabilis* close to the putative centromere is relatively uncommon, with a recent study reporting a terminal or subterminal location in most taxa with data available (~90%), while ~18% are located interstitially and only ~13% appear close to centromeres [79].

In addition to genes for the 18S, 5.8S and 28S/26S/25S RNAs, the 35S rDNA contains internal transcribed spacers 1 and 2 (ITS1 and ITS2, flanking the 5.8S rDNA), and an intergenic spacer (IGS). The individual repeat units are thought to evolve together (i.e., concerted evolution) resulting in high similarity or identical rRNA genes across the repeat units, although variation can occur. In contrast, the IGS is rapidly evolving with many species exhibiting variation in IGS lengths [80]. A recent examination of IGS regions across 12 species (including five plants) showed that the IGS largely comprises short direct repeats and multiple long tandem repeats that likely originated from the insertion and imprecise excision of transposons [80]. In *N. mirabilis*, the X-chromosome 35S repeat unit varies in length and occasional unit fragments are present, but most unit lengths are centred around ~11.4 kb and ~13.7 kb (Fig S1K in S1 File). A schematic of two example repeat units is shown in Fig 2. Consistent with the IGS studies above, the majority of the IGS in *N. mirabilis* consists of a tandem repeat; a fragment of a putative Gypsy LTR transposon is also present but overlaps the 5’ ETS region immediately upstream of the 18S rRNA, and hence might be an erroneous annotation. Further, comparisons with shorter and longer examples of the repeat unit show that variation in length is largely due to differences in the number of tandem repeats in the IGS, as found in other plants. An alignment of an example IGS region from *N. mirabilis* with the region downstream of the 28S/26S/25S RNA in *N. gracilis* scaffold41 (see above) shows some conservation of sequence identity (Fig S1L in S1 File), consistent with the observation that at least sections of the IGS region exhibit signals of vertical inheritance within a species or genus, but above this taxonomic level sequence similarity rapidly declines [80].

Interestingly, three annotated protein-coding genes occur within the *N. mirabilis* 35S (Fig 2 and S14 Table in S2 File). The first of these, jg50561, contains an exon with high similarity to *TAR1* (Transcript Antisense to Ribosomal RNA), whereas the second gene jg50562 contains an exon with high similarity to *RRT15* (Regulator of rDNA transcription protein 15); both these exons are completely encapsulated within the 23S rRNA gene, but occur on the opposite strand. *TAR1* was first identified in yeast and is potentially involved in regulation of respiratory metabolism [https://string-db.org/network/4932.YLR154W-C]. The precise role of *RRT15* is unknown, but in yeast it may influence rRNA transcription [81]. The third gene, jg50563, contains an exon with hits to hypothetical proteins from other plants, and overlaps the 3’ terminus of the 23S rRNA on the opposite strand.

Finally, we searched for tandem repeats of the 5S rDNA. Multiple loci containing repeat arrays were detected in both species. The largest array occurs on a homologous chromosome (*Nepenthes mirabilis* contig ptg000022l_1 and *N. gracilis* scaffold40), and has a repeat unit length of ~310 bp. The array size is ~ 850 kb in *N. mirabilis* and ~60 kb in *N. gracilis*; *N. mirabilis* scaffold40 contains a contig junction within the array, and so the *in vivo* array size in the contiguous chromosome cannot be determined. A second, much shorter repeat array is found for both species on a different homologous chromosome (*N. mirabilis* contig ptg000014l_1 and *N. gracilis* scaffold9, ~ 5.7 kb and 935 kb respectively), as well as three arrays (~11 kb, 5.5 kb and 1.7 kb) within a ~ 45 kb region of *N. gracilis* scaffold30 that are absent from the homologous *N. mirabilis* contig ptg000025l_1. Although copies of the 35S rDNA genes are detected on the same chromosomes as 5S rDNA copies, the 35S rDNA genes are often fragments and none occur in tandem repeats of linked 35S – 5S arrays, indicating that the *Nepenthes* 5S rDNA is largely or fully separated from the 35S rRNA (i.e., an S-type organisation).

### *Nepenthes mirabilis* genome characterisation, annotation, and gene family clustering

Transposable elements (TEs) comprised ~65% of the total genome sequence (S4 Table in S2 File). Most of the transposable elements belonged to long terminal repeat (LTR) retrotransposons (44.04% of the total genome, with 35.52% classified as Gypsy-type LTRs), followed by DNA transposable elements (15.29%). Comparing the proportion of TEs in the genomes of Caryophyllales species sequenced to date indicates that while the ‘core Caryophyllales’ have similar repetitive genome components (e.g., *Beta vulgaris* 42.3%; *Amaranthus hypochondriacus* 48%; *Phiambolia similis* 47%), there is a wide variation in TE components amongst the non-core species (e.g., *Fagopyrum esculentum* 71%) and the ‘carnivore-clade’ (e.g., *Drosera spatulata* 6%; *Aldrovanda vesiculosa* 20%; *N. mirabilis* 65%; *Dionaea muscipula* 80%). Such a wide variation is also found in non-Caryophyllales carnivores (e.g., *Utricularia gibba* 32%; *Utricularia reniformis* 56%; *Cephalotus follicularis* 77%).

Nuclear gene models were predicted using the BRAKER2 pipeline, followed by additional filtering to remove putative TEs and genes with little or no support (see Methods). In total, 29,806 genes remained after filtering. This number is comparable to most other carnivorous plant taxa examine in this study (S10 Table in S2 File). Of the 29,806 predicted genes, 28,143 (~94.4%) were functionally annotated by at least one source (InterProScan = ~94.4%; KEGG = ~32%; Pfam = 64%; see Fig S1B in S1 File). The completeness of the predicted proteome was assessed using BUSCO analyses. The predicted gene set contained complete sequences for 93.6% of the 2,121 Eudicotyledons BUSCO genes, with only 3% missing entirely (see S10 Table in S2 File for full BUSCO results and a comparison with other carnivorous plant taxa used in this study).

**Fig 3:**
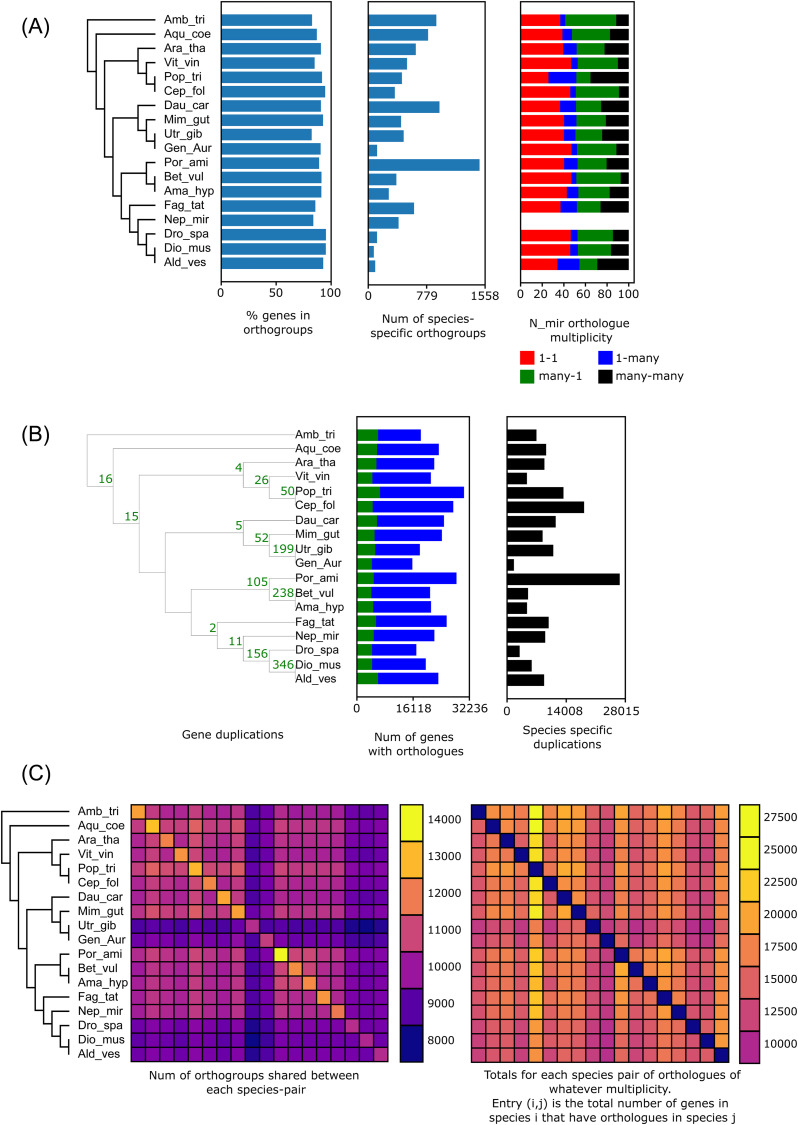
Plots of OrthoFinder statistics from broad angiosperms sampling. (A) Genes in orthogroups, number of species specific orthogroups, and ortholog multiplicity of all samples relative to *N*. *mirabilis*. (B) Estimated gene duplications on phylogeny, genes with orthologues, number of species-specific orthogroups. (C) Orthogroup overlap between species pairs. On-diagonal values in the left panel correspond to the total number of orthogroups present for each species. On-diagonal values in the right panel all equal zero; note that this heatmap is not a mirror image, as species i might have many more copies of a given ortholog than species j.

OrthoFinder analysis of a set of 18 angiosperm species assigned 25,457 (85.4%) of the *N. mirabilis* genes to one of the 26,109 identified orthogroups (Fig 3, S11 Table in S2 File). A total of 22,259 (74.7%) *N. mirabilis* genes were present in an orthogroup containing an ortholog from at least one other angiosperm species. 3,198 (10.7%) genes present in one of the 404 orthogroups containing *N. mirabilis* sequences only; 4,349 predicted *N. mirabilis* genes did not have any identified orthologs.

### Organelle genome assemblies and NUPT/NUMT identification

The organelle genome assemblies consisted of one plastome contig (156,374 bp), and two mitochondrial contigs (269,272 bp and 221,233 bp). The structure and gene content of the *Nepenthes mirabilis* plastome is similar to plastomes from *N.* *×* *ventrata* [82] and a previously sequenced *N. mirabilis* [83], following the typical angiosperm structure of a large single copy region, a small single copy region, and two inverted repeats. This contrasts with other carnivore-clade Caryophyllales such as *Drosera* and *Aldrovanda*, in which the plastomes exhibit both structural rearrangements and gene loss or pseudogenisation [84,85]. In *N. mirabilis*, only one plastid pseudogene was found, *ccsA*, and no genes of mitochondrial origin were found in the plastome. The sequence and structure of plastomes are typically stable amongst angiosperms that utilise photosynthesis as a main source of carbon acquisition, but as more studies are undertaken an emerging general pattern of plastome degradation and rearrangement is apparent in plants that have evolved different strategies for nutrition/life history. Such drastic plastome evolution has been discovered in parasitic plants, mycoheterotrophs, and carnivores. Given our growing knowledge in plastome evolution in plants with alternative lifestyles, it is somewhat surprising that the *Nepenthes* lineage appears to maintain fully functional plastome sequences [82,83], especially considering the gene loss and rearrangements discovered in other members of the Caryophyllales carnivore-clade.

The mitochondrial assembly comprised two contigs with a total length 490,505 bp. Alignments using Mauve showed that these contigs aligned well to the published mitochondrial genome of *N. *×* ventrata* ([86], GenBank accession MH798871.1, length = 520,764 bp), but with many rearrangements (see Fig S1M in S1 File). This finding aligns with previous investigations into mitochondrial genome rearrangements between closely related plant species (i.e., the same genus; [87,88]). Both mitochondrial contigs were found to have identical start and end repeat regions (22,847 and 17,468 bp respectively), indicating they are both possibly circular molecules. Organelle genome annotations showed that the plastome contained 78 protein encoding genes, one pseudogene (*ccsA*), eight rRNAs, and 45 tRNAs. The mitome contained 36 protein encoding genes, five pseudogenes, three rRNAs, and 27 tRNAs. No genes of mitochondrial origin were found in the plastome. Thirteen protein encoding genes of plastid origin (*atpE*, *pbf1*, *psbB*, *psbM*, *psbZ*, *infA*, *rpl14*, *rpl16*, *rpl2*, *rpl23*, *rpl36*, *rps19*, and *rps8*) and 14 plastid pseudogenes (*clpP1*, *ndhC*, *ndhJ*, *pafI*, *petB*, *petD*, *petG*, *psaB*, *psbC*, *psbT*, *psbA*, *rbcL*, *rpoA*, *rps11*) were found in the mitome. The transfer of plastid genes to mitomes is a well-established occurrence [89].

The organelle genome assemblies were used to identify putative transfers from the organelle genomes to the nuclear genome. A total of 2,129 Nuclear-mitochondrial DNA (NUMTs; total length 841,650 bp), 1,446 Nuclear-plastid DNA (NUPTs, total length 471,030 bp), 186 NUM/PTS (ambiguous identity, total length 58,792 bp), and 113 NUMPTS (complex tandem NUMT–NUPT, total length 88,913 bp) were observed (see S9 File for annotation in GFF3 format). All identified NUMTs/NUPTs had mapped HiFi reads covering at least 50 bp of the insert and nuclear genome junction, indicating they were not assembly artifacts. As more nuclear and organellar genomes are sequenced for plants, we will gain a better understanding of how genes move from one genome to another, and whether there are evolutionary consequences to doing so.

## Conclusion

In this study we assembled a high quality draft genome of *Nepenthes mirabilis*, comprising 67 main contigs with an N50 of ~20 Mb, and totalling ~1 Gb in length. The annotated genome includes 29,806 genes, of which 94.4% were functionally annotated; 66% of the genome was determined to be transposable elements. Despite not using any genome scaffolding method (such as HiC), we obtained a close-to-chromosome scale assembly (67 contigs for 40 chromosomes) using a single HiFi flow-cell, demonstrating the ability to assemble a good reference for ~1 Gb genomes for less than AU $10,000. However, it is important to note that this approach is only feasible for genomes that are relatively non-repetitive, and it requires high-quality DNA as an input. For genomes with high levels of repetitive DNA, or plants with difficult to remove secondary compounds, more expensive and specialised techniques may be required.

## Supporting information

S1 File Supporting information Figures S1A – S1N. (DOCX)

S2 File Tables S1 – S17. (XLSX)

S3 File Table of InterProScan annotations for the 29,806 *N. mirabilis* predicted genes. (ZIP)

S4 File All-vs-all dot plots of *N. mirabilis* putative full-chromosome contigs. (ZIP)

S5 File Plot of the location of genes, tandem repeats and transposons mapped against individual *N. mirabilis* contigs. (PDF)

S6 File RepeatOBserver plots for *N. mirabilis* contigs. (PDF)

S7 File All-vs-all dot plots of *N. gracilis* putative full-chromosome contigs. (PDF)

S8 File Putative *N. mirabilis* predicted gene sequences that were removed from final gene set due to lack of external evidence or functional annotation, in FASTA format. (ZIP)

S9 File NUPT/NUMT annotation of *N. mirabilis* nuDNA contigs, in GFF3 format. (GFF3)
