# Supplementary material for: A high quality genome of the common swamp pitcher plant (Nepenthes mirabilis) using PacBio HiFi sequencing
Source: PLoS One. 2025 Jul 10;20(7):e0322885. doi: 10.1371/journal.pone.0322885 (PMC12244726; doi:10.1371/journal.pone.0322885)
Supplement: S1 File — (DOCX) [file pone.0322885.s001.docx]

**Supporting Information File S1**

**Contents:**

S1 A KMC spectra plot.

S1 B Functional annotations Venn diagram comparing the overlap of the *N. mirabilis* proteome annotated using Pfam, InterProScan, and KEGG.

S1 C Plot of telomere repeat sequence counts across each *N. mirabilis* contig.

S1 D Nucleotide alignment dot plots of *N. mirabilis* contigs matching *N. gracilis* scaffolds.

S1 E GENESPACE Riparian plot showing gene order synteny for *N. mirabilis* contigs matching a single *N. gracilis* scaffold.

S1 F GENESPACE Riparian plot showing gene order synteny for paired *N. mirabilis* contigs matching a single *N. gracilis* scaffold.

S1 G GENESPACE Riparian plot showing gene order synteny for *N. mirabilis* contigs with more complex hits against a *N. gracilis* scaffold(s).

S1 H Pairs of *N. mirabilis* contigs matching a single *N. gracilis* scaffold, showing the location of genes and the location of tandem repeats grouped by repeat unit size.

S1 I Alignment dot plots of *N. mirabilis* contig ptg000002l_1 vs ptg000003l_1, ptg000007l_1, ptg000024l_1 and ptg00003136l_1.

S1 J Plot of the location of genes, tandem repeats and transposons mapped against *N. mirabilis* contigs ptg000002l_1 and ptg000003l_1.

S1 K Bar graph showing the length distribution of the 35S rRNA operon repeat unit in the *N. mirabilis* X-chromosome.

S1 L Alignment of the three-prime end of the 28S/26S/25S RNA in *N. gracilis* scaffold41 with the same region in the 35S rRNA operon repeat unit in the *N. mirabilis* X-chromosome.

S1 M Mauve alignment of *N. mirabilis* mtDNA contigs with the *N. ventricosa* × *N. alata* mtDNA.

S1 N GENESPACE Riparian plot showing gene order synteny for *N. mirabilis* contigs vs *Beta vulgaris* and *Fagopyrum tataricum*.

**S1 A. KMC spectra plot**


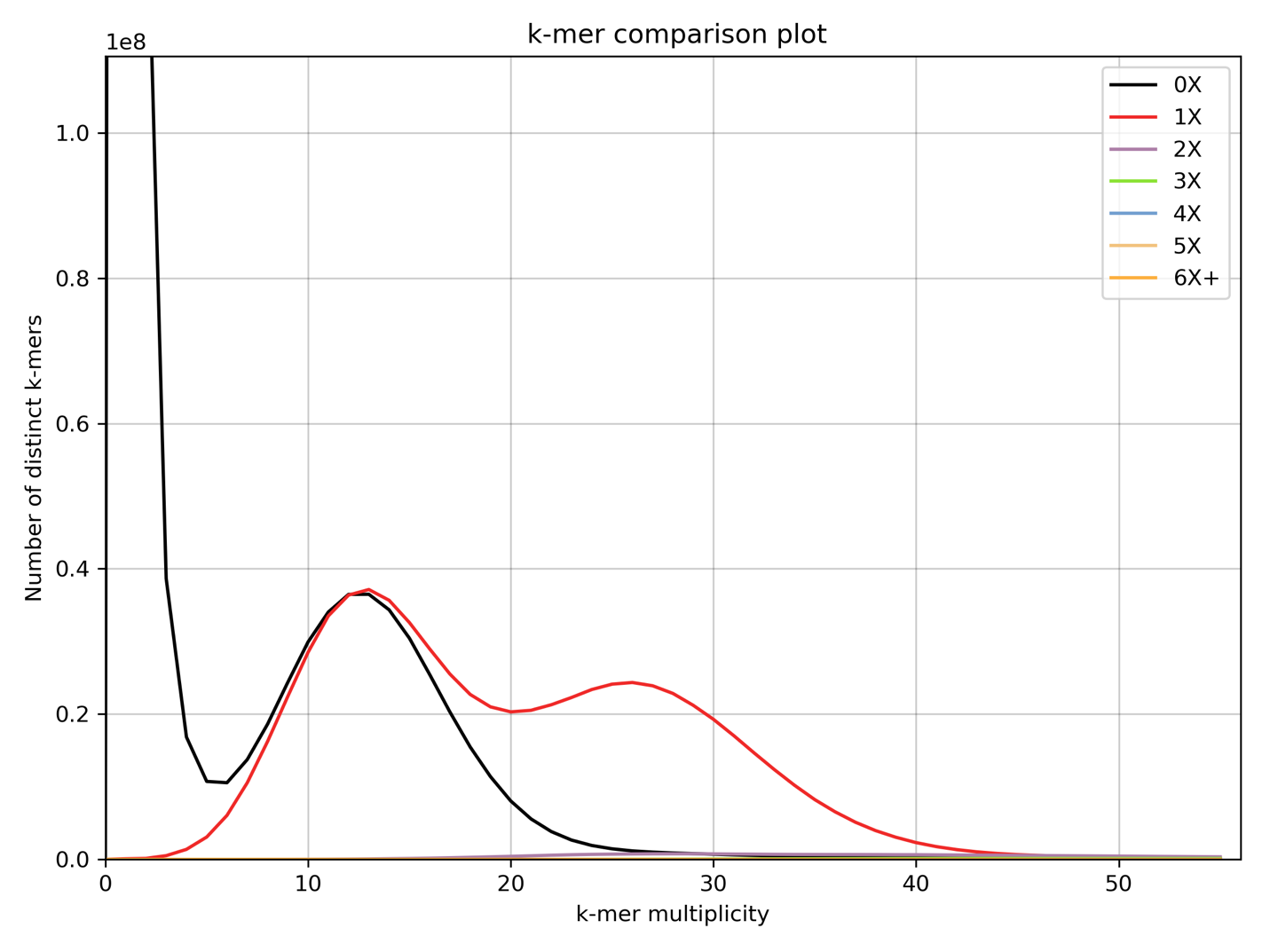


A copy-number spectrum plot of k-mers found in the PacBio CCS read set and the 67-contig assembly, with the latter coloured based on their count in the assembly. k-mers found only in the read set are shown in black; those at low frequency (i.e., multiplicity) likely represent errors in the read set, whereas those at higher frequencies (e.g., black k-mers within the one or two-copy peaks at ~13x and ~27x multiplicity, respectively) indicate sequences missing from the assembly. The red peak at the 2-copy region (~27x multiplicity) represents homozygous two-copy k-mers that occur only once in the assembly, suggesting that Hifiasm followed by purge-dups has largely succeeded in producing a pseudo-haploid assembly. The purple peak at the same region represents homozygous two-copy k-mers that still occur twice in the assembly, indicating that a small fraction of the assembly is still in diploid form.

**S2 B Functional annotations Venn diagram comparing the overlap of the *Nepenthes mirabilis* proteome annotated using Pfam, InterProScan, and KEGG.**


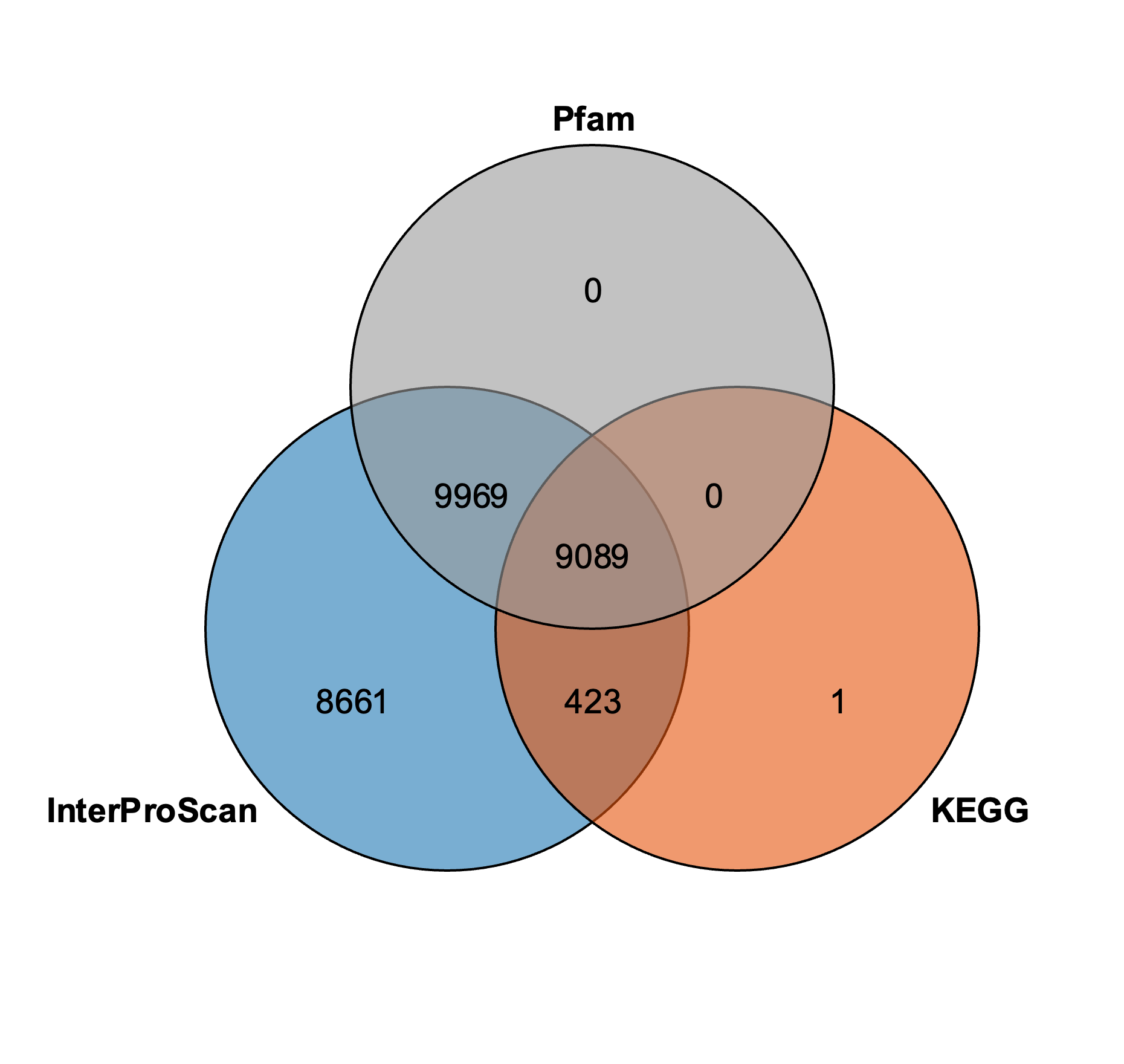


**S1 C Plot of telomere repeat sequence counts across each *Nepenthes mirabilis* contig. Output corresponds to the telomere repeat sequence ‘AAACCCT’, which is equivalent to the typical plant-type telomere sequence ‘TTTAGGG’.**

1. ***N. mirabilis* contigs with 1:1 full-length synteny vs *N. gracilis* scaffolds**

1. **Paired *N. mirabilis* contigs with partial synteny vs a single *N. gracilis* scaffold**

1. ***N. mirabilis* contigs with more complex syntenic matches against a *N. gracilis* scaffold(s)**

1. ***N. mirabilis* contigs containing predicted genes but no syntenic matches against a *N. gracilis* scaffold(s):**

1. ***N. mirabilis* contigs with no genes and no syntenic matches against a *N. gracilis* scaffold(s)**

**S1 D Nucleotide alignment dot plots of *Nepenthes mirabilis* contigs matching *N. gracilis* scaffolds.** Note that some *N. mirabilis* contigs have been reverse complemented relative to their orientation in the genome assembly to best match the corresponding *N. gracilis* scaffold. Blue dot/lines represent matches in the forward orientation, whereas red dots/line represent matches to the reverse complement of the reference sequence.

1. ***N. mirabilis* contigs with 1:1 full-length synteny vs *N. gracilis* scaffolds**

1. **Paired *N. mirabilis* contigs with partial synteny vs a single *N. gracilis* scaffold**

1. ***N. mirabilis* contigs with more complex syntenic matches against a *N. gracilis* scaffold(s)**

**S1 E GENESPACE Riparian plot showing gene order synteny for *N. mirabilis* contigs matching a single *N. gracilis* scaffold.**

**S1 F GENESPACE Riparian plot showing gene order synteny for paired *N. mirabilis* contigs matching a single *N. gracilis* scaffold.**

**S1 G GENESPACE Riparian plot showing gene order synteny for *N. mirabilis* contigs with more complex hits against a *N. gracilis* scaffold(s).**

**S1 H Pairs of *N. mirabilis* contigs matching a single *N. gracilis* scaffold, showing the location of genes (blue dots) and the location of tandem repeats grouped by repeat unit size (pink/purple dots).** Red horizontal lines represent the contig. Green dots represent 36 bp tandem repeat units; the long arrays at the termini of the first five chromosome pairs have identical or near identical sequence identity in the 36 bp array repeat unit.


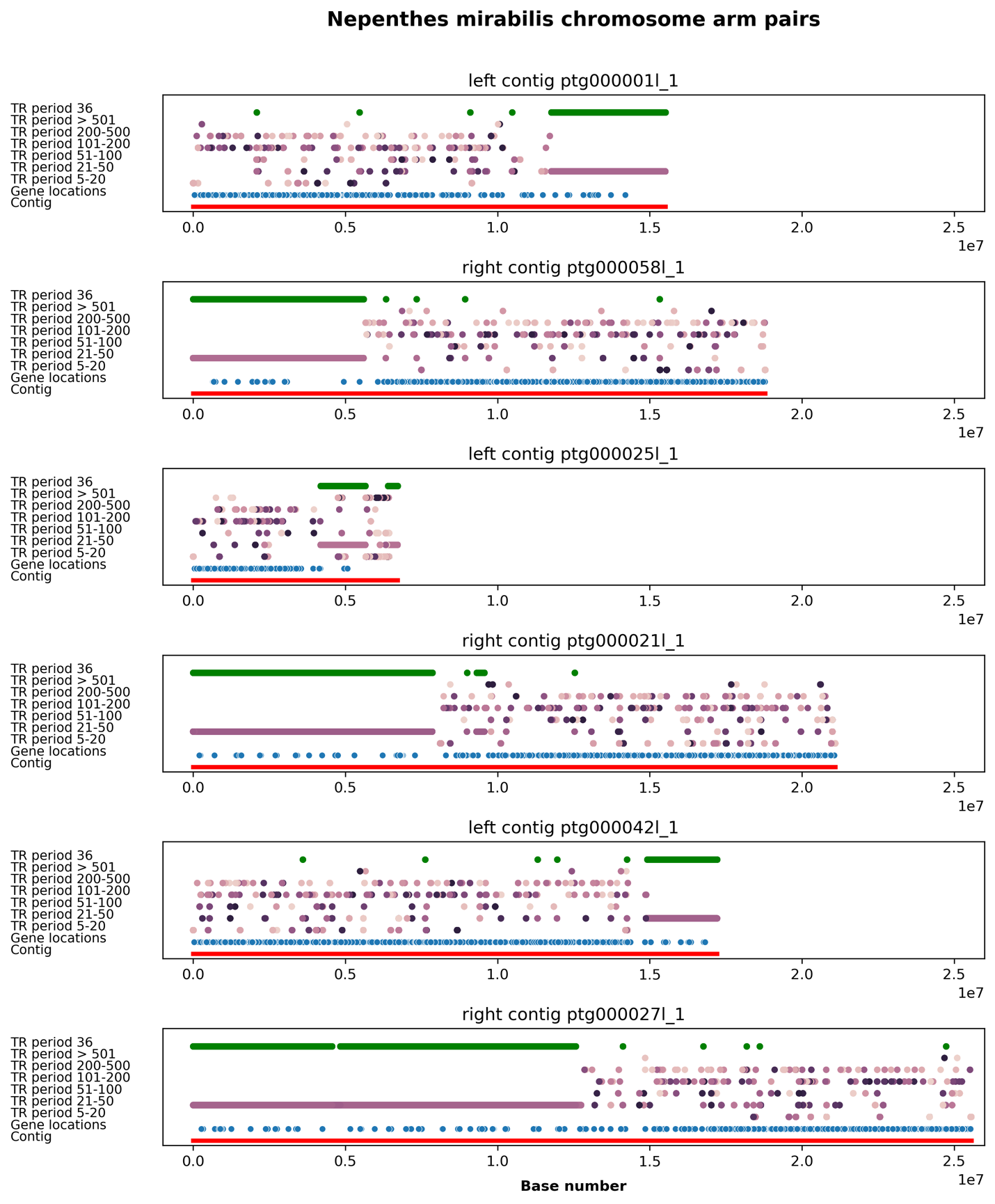


**
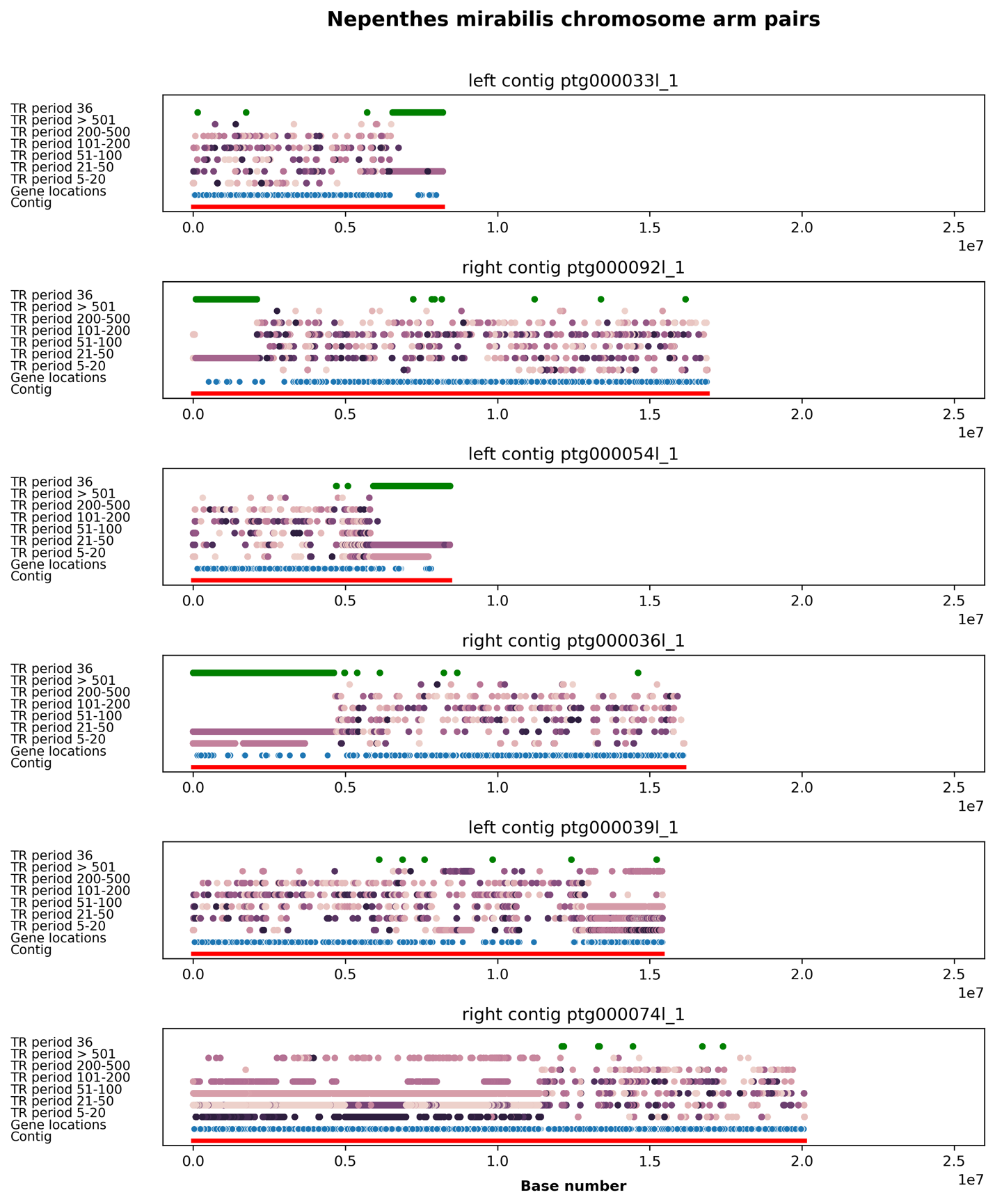
**

**S1 I Alignment dot plots of *N. mirabilis* contig ptg000002l_1 vs ptg000003l_1, ptg000007l_1, ptg000024l_1 and ptg000031l_1.** Each dot plot shows a rectangular region of alignment matches that for contig ptg000002l_1 consistently occurs within the nucleotide range ~7.8 x 10^6^ – 9 x 10^6^.

**S1 J** P**lot of the location of genes, tandem repeats and transposons mapped against *N. mirabilis* contigs ptg000002l_1 and ptg000003l_1.** Red horizontal bar: contig sequence. Grey horizontal bar: contig coordinates of all-vs-all contig dot plot rectangle hit regions (see manuscript text). Blue dots: location of individual genes. Horizontal lines of purple dots: tandem repeats grouped by period size range (from bottom to top: period 5–20, period 21–50, period 51–100, period 101–200, period 201–500, period >500). Green/yellow/orange/red dots: individual transposons, with each horizontal line of dots corresponding to multiple locations for a single transposon. Only transposons where >=70% of the copies occur within the all-vs-all contig dot plot rectangle hit region for >=10 contigs are shown. Dots are coloured by the number of contigs for which this threshold is true (e.g., in 27 of 33 contigs, >=70% of the copies occur within the rectangle hit region for a given contig). Green is the largest number of contigs (27), and red is the smallest (10); see also Supplementary File S2 Table S12).

**S1 K** **Bar graph showing the length distribution of the 35S rRNA operon repeat unit in the *N. mirabilis* X-chromosome.**

**
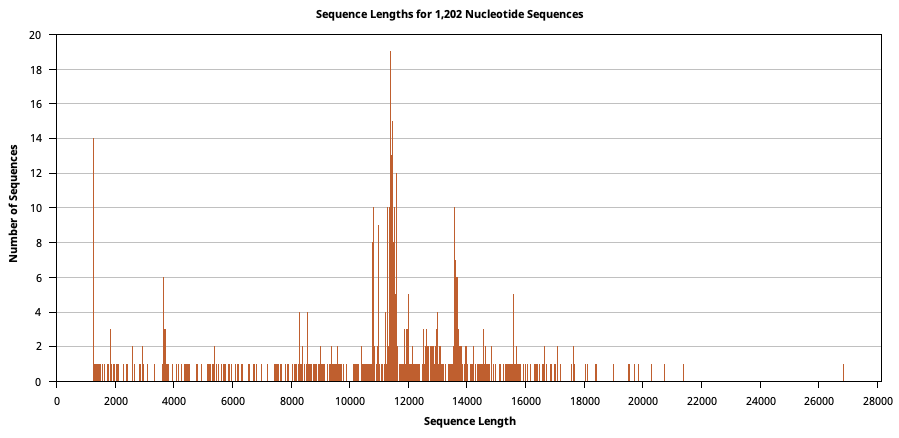
**

**S1 L** **Alignment of the three-prime end of the 28S/26S/25S RNA in *N. gracilis* scaffold41 with the same region in the 35S rRNA operon repeat unit in the *N. mirabilis* X-chromosome.** Black columns represent conserved nucleotide characters.

**
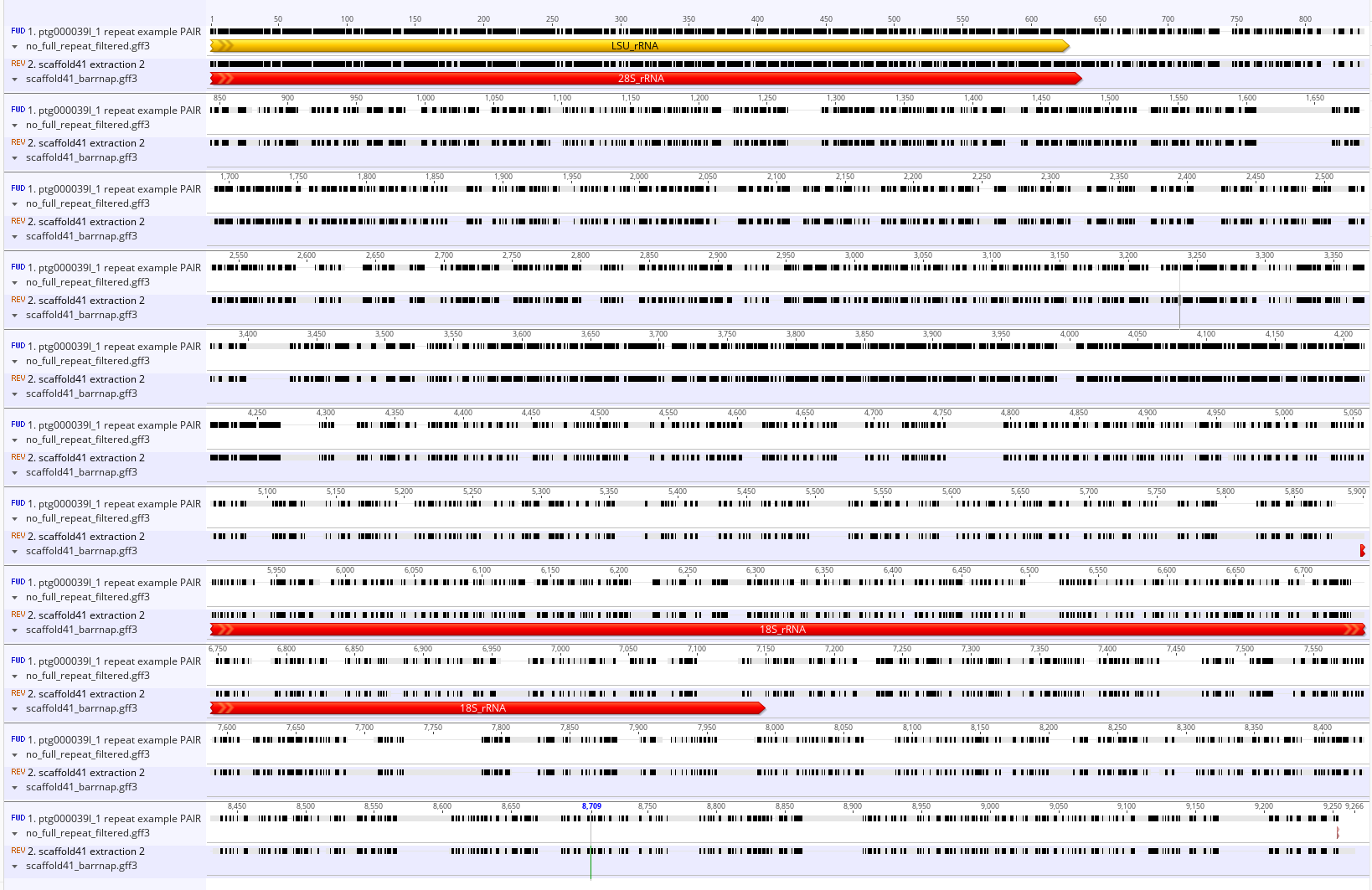
**

S1 M Mauve alignment of *N. mirabilis* mtDNA contigs (top) with the *N. ventricosa* × *N. alata* mtDNA (bottom, accession MH798871). Coloured blocks in the N. mirabilis contigs connected by lines to similarly colored blocks in the *N. ventricosa* × *N. alata* contig indicate regions in each genome that are homologous. The region of sequence covered by a coloured block is entirely collinear and homologous among the genomes. The boundaries of coloured blocks usually indicate the breakpoints of genome rearrangement (unless sequence has been gained or lost in the breakpoint region).

**
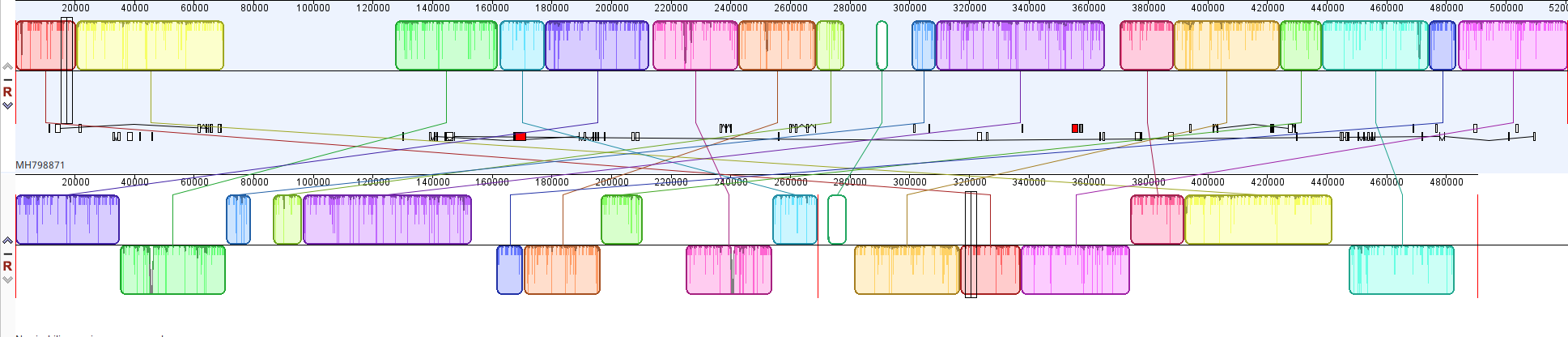
**

S1 N GENESPACE Riparian plot showing gene order synteny for *N. mirabilis* (nm) contigs vs *Beta vulgaris* (bt) and *Fagopyrum tataricum* (ft).
