# Supplementary figures and images for: A high quality genome of the common swamp pitcher plant (Nepenthes mirabilis) using PacBio HiFi sequencing
Source: PLoS One. 2025 Jul 10;20(7):e0322885. doi: 10.1371/journal.pone.0322885 (PMC12244726; doi:10.1371/journal.pone.0322885)

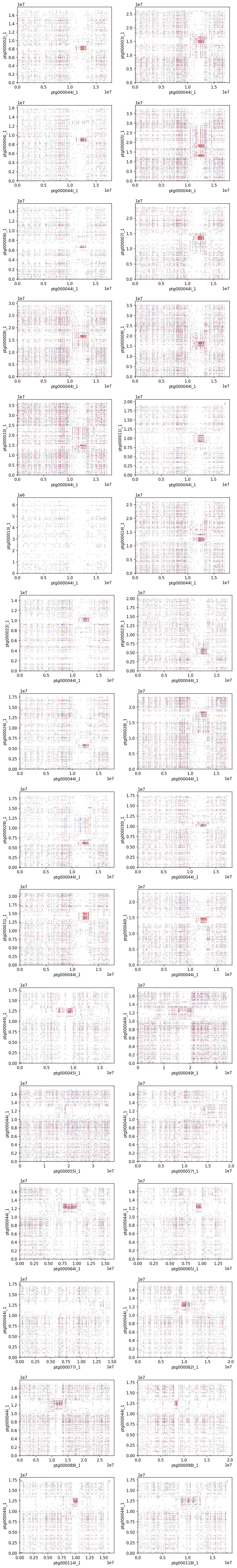

Supplement: S4 File — (ZIP) [file pone.0322885.s004.zip › File_S4/ptg000044l_1.jpg]

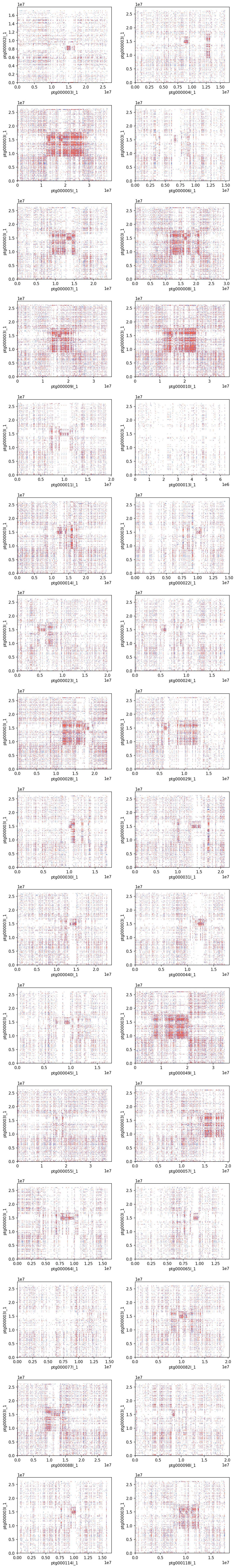

Supplement: S4 File — (ZIP) [file pone.0322885.s004.zip › File_S4/ptg000003l_1.jpg]

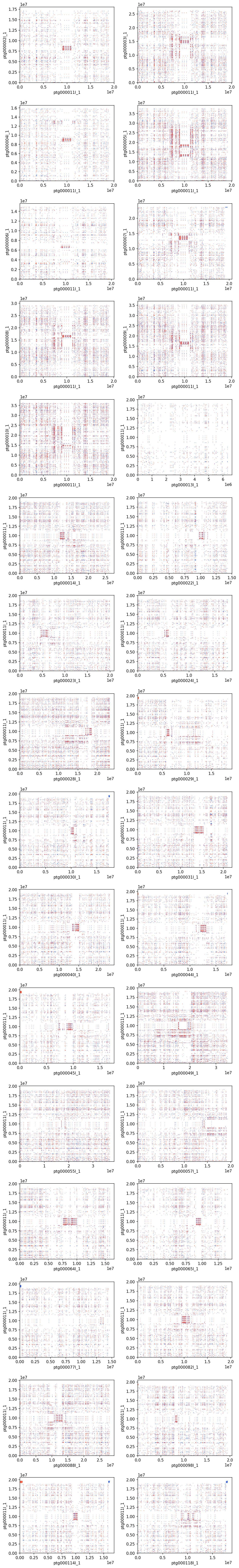

Supplement: S4 File — (ZIP) [file pone.0322885.s004.zip › File_S4/ptg000011l_1.jpg]

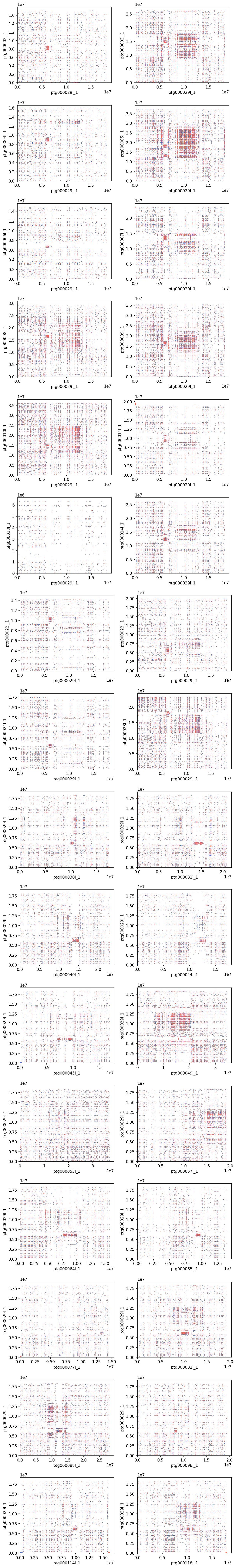

Supplement: S4 File — (ZIP) [file pone.0322885.s004.zip › File_S4/ptg000029l_1.jpg]

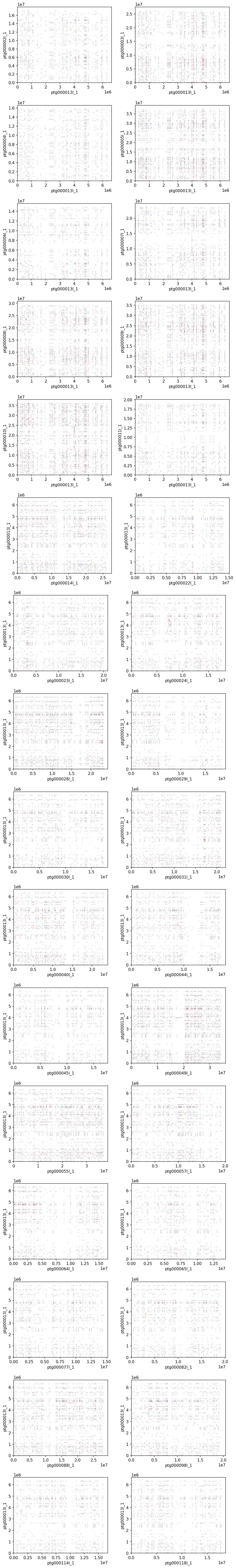

Supplement: S4 File — (ZIP) [file pone.0322885.s004.zip › File_S4/ptg000013l_1.jpg]

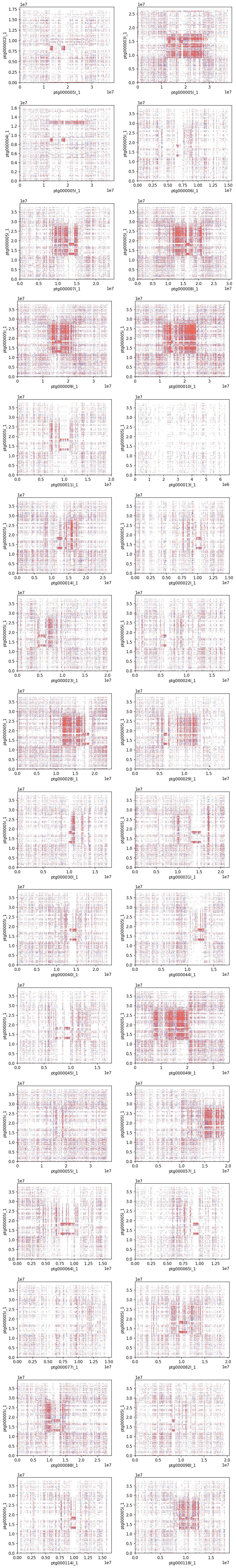

Supplement: S4 File — (ZIP) [file pone.0322885.s004.zip › File_S4/ptg000005l_1.jpg]

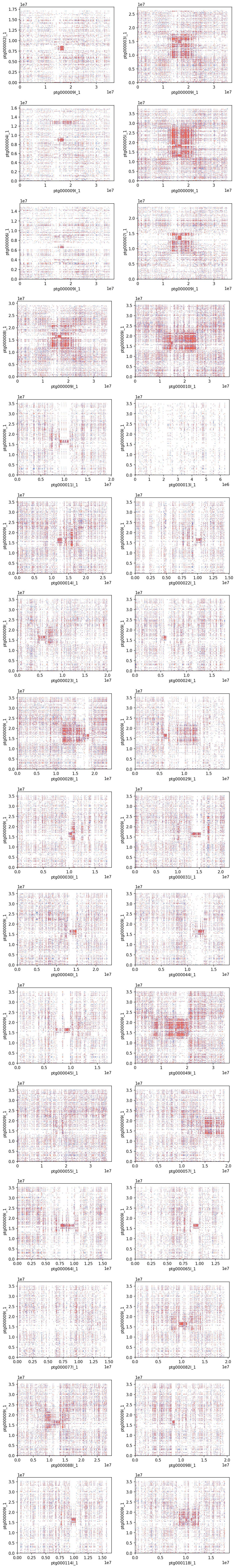

Supplement: S4 File — (ZIP) [file pone.0322885.s004.zip › File_S4/ptg000009l_1.jpg]

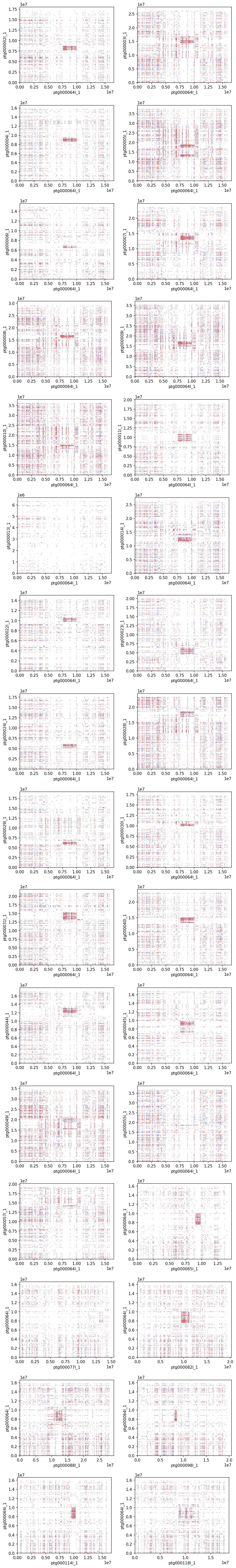

Supplement: S4 File — (ZIP) [file pone.0322885.s004.zip › File_S4/ptg000064l_1.jpg]

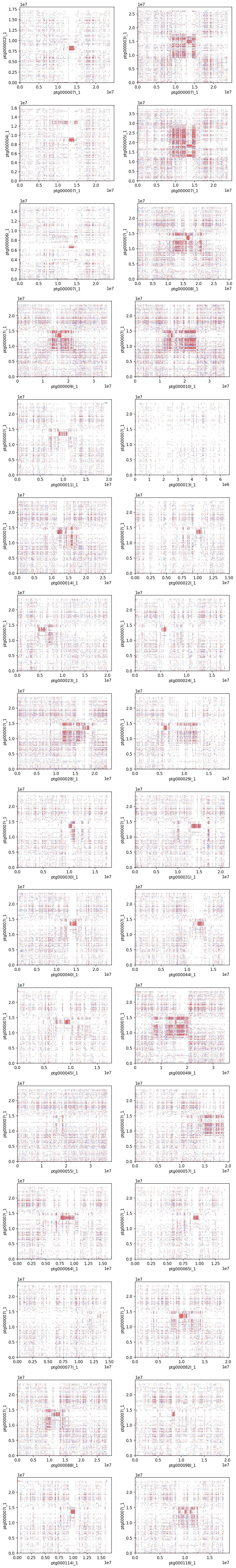

Supplement: S4 File — (ZIP) [file pone.0322885.s004.zip › File_S4/ptg000007l_1.jpg]

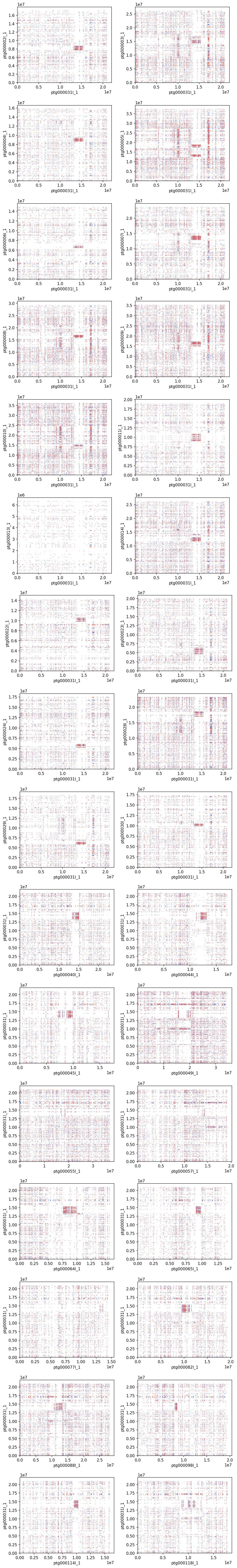

Supplement: S4 File — (ZIP) [file pone.0322885.s004.zip › File_S4/ptg000031l_1.jpg]

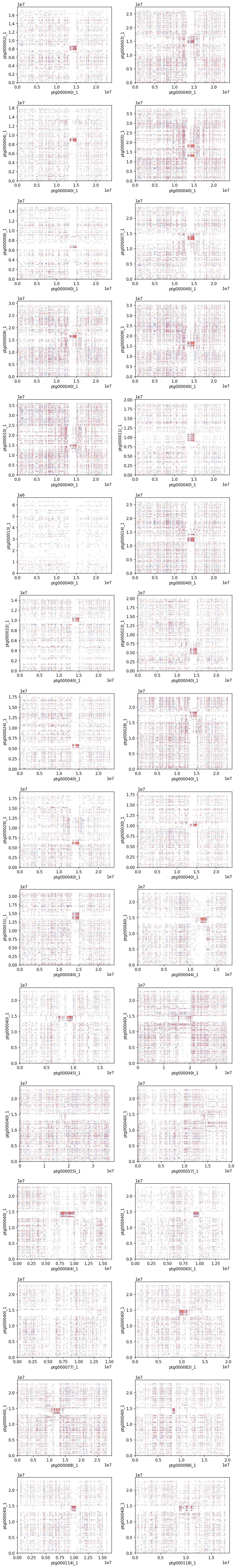

Supplement: S4 File — (ZIP) [file pone.0322885.s004.zip › File_S4/ptg000040l_1.jpg]

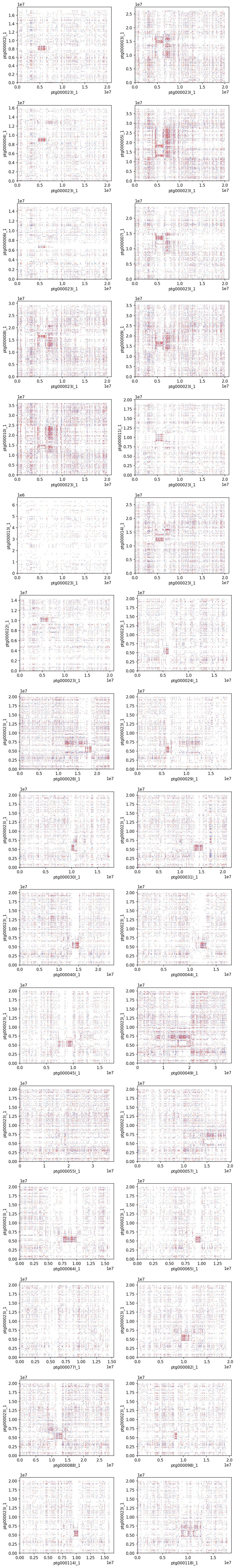

Supplement: S4 File — (ZIP) [file pone.0322885.s004.zip › File_S4/ptg000023l_1.jpg]

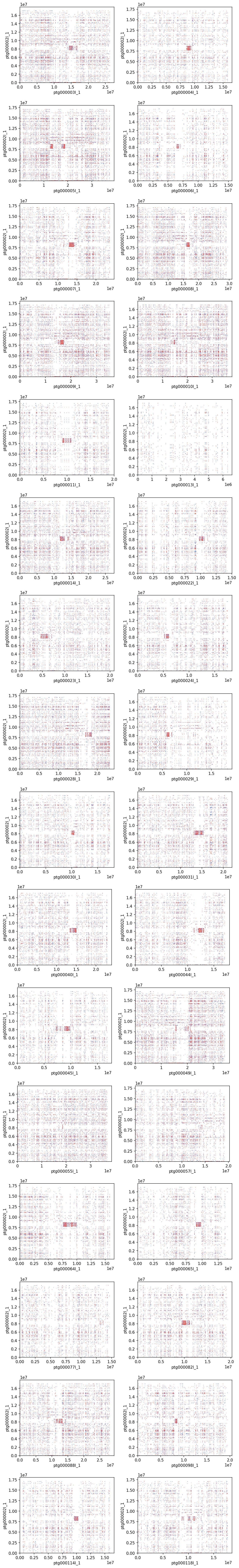

Supplement: S4 File — (ZIP) [file pone.0322885.s004.zip › File_S4/ptg000002l_1.jpg]

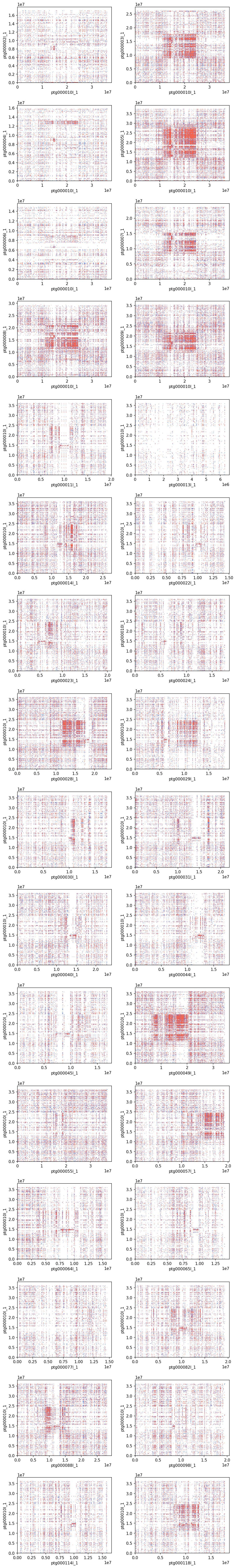

Supplement: S4 File — (ZIP) [file pone.0322885.s004.zip › File_S4/ptg000010l_1.jpg]

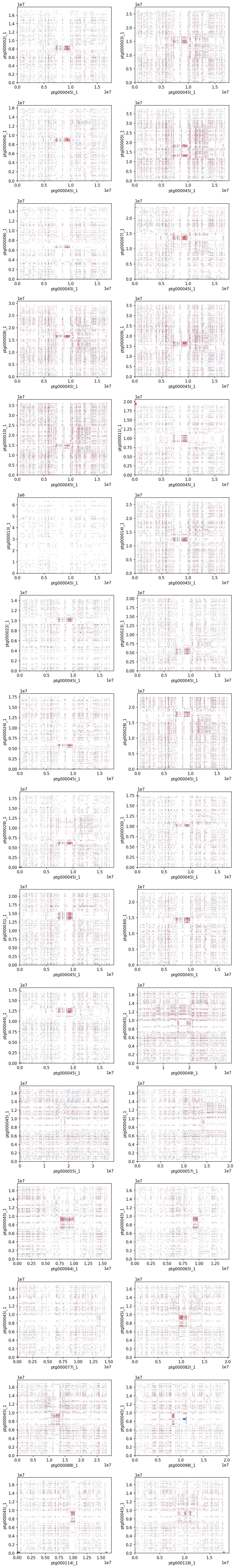

Supplement: S4 File — (ZIP) [file pone.0322885.s004.zip › File_S4/ptg000045l_1.jpg]

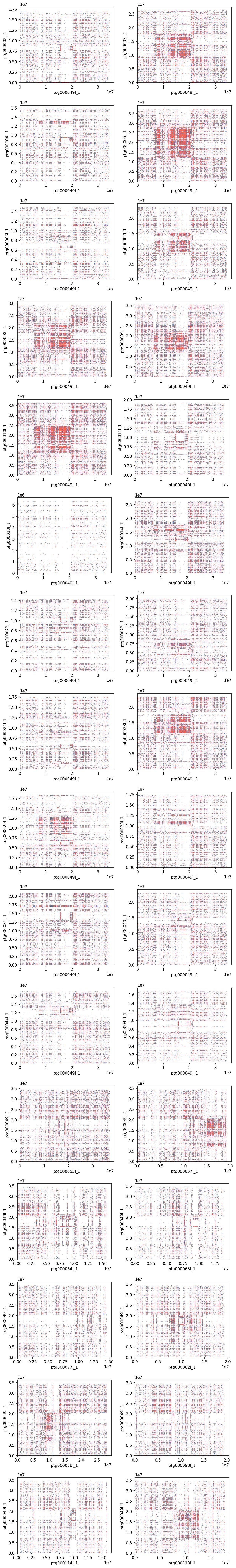

Supplement: S4 File — (ZIP) [file pone.0322885.s004.zip › File_S4/ptg000049l_1.jpg]

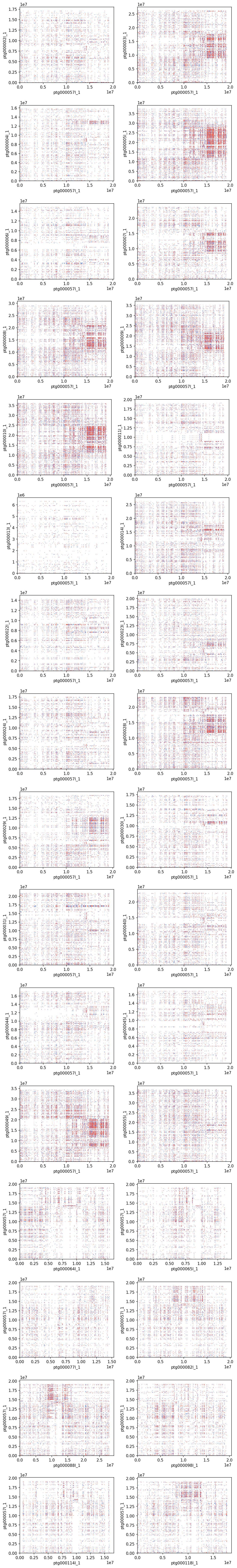

Supplement: S4 File — (ZIP) [file pone.0322885.s004.zip › File_S4/ptg000057l_1.jpg]

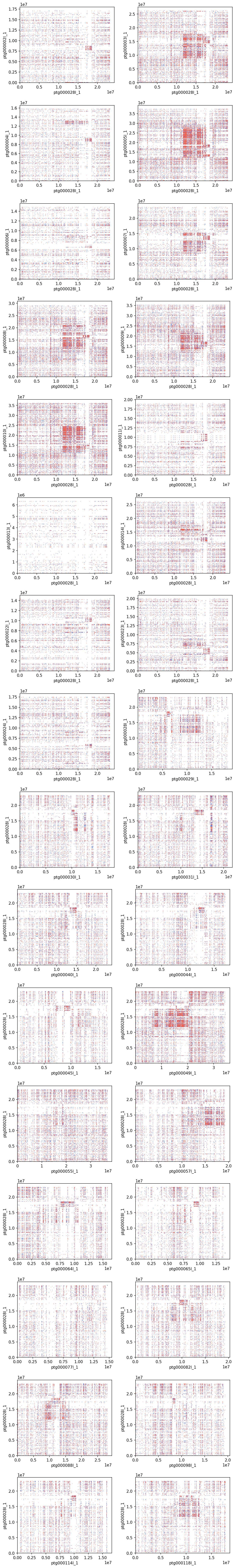

Supplement: S4 File — (ZIP) [file pone.0322885.s004.zip › File_S4/ptg000028l_1.jpg]

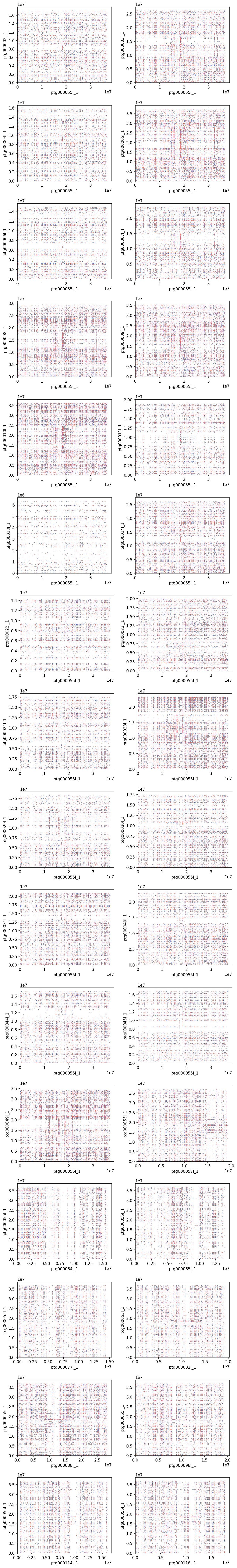

Supplement: S4 File — (ZIP) [file pone.0322885.s004.zip › File_S4/ptg000055l_1.jpg]

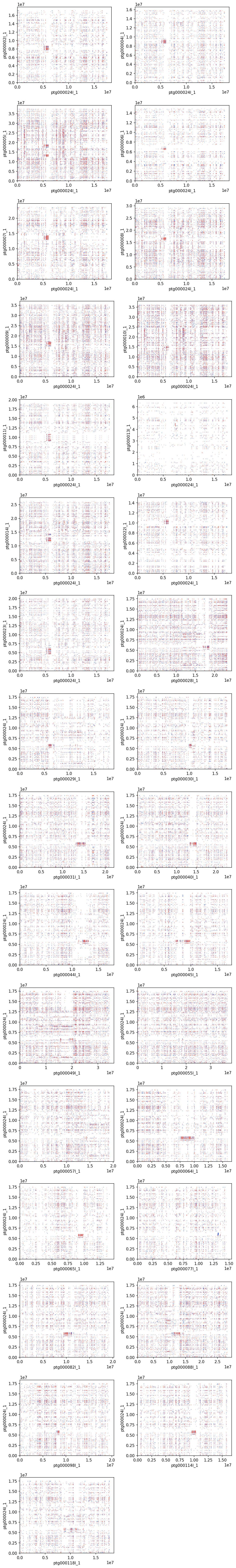

Supplement: S4 File — (ZIP) [file pone.0322885.s004.zip › File_S4/ptg000024l_1.jpg]

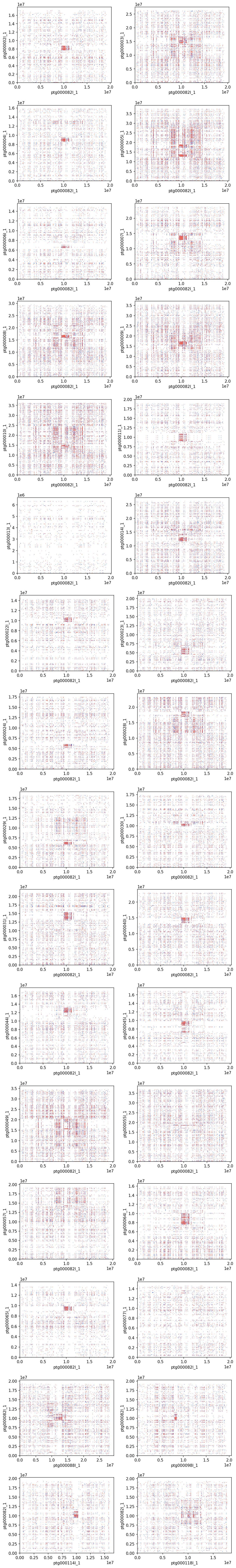

Supplement: S4 File — (ZIP) [file pone.0322885.s004.zip › File_S4/ptg000082l_1.jpg]

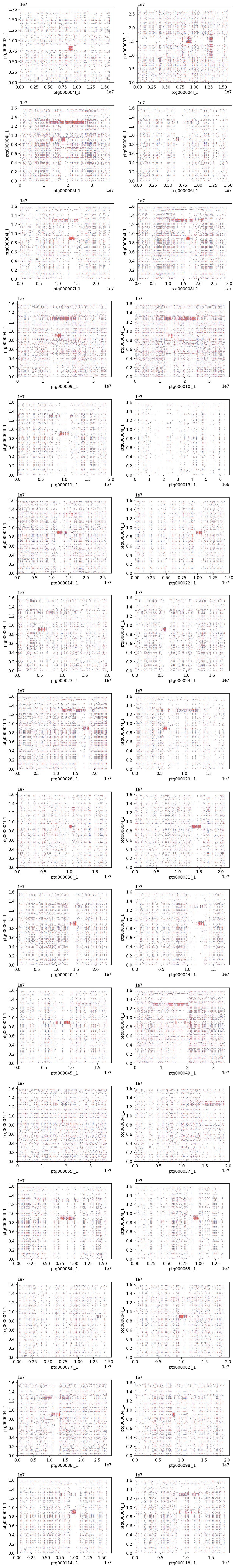

Supplement: S4 File — (ZIP) [file pone.0322885.s004.zip › File_S4/ptg000004l_1.jpg]

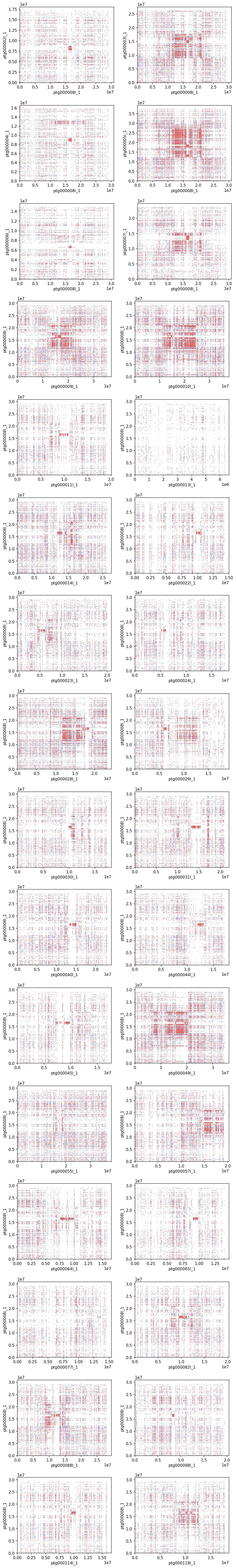

Supplement: S4 File — (ZIP) [file pone.0322885.s004.zip › File_S4/ptg000008l_1.jpg]

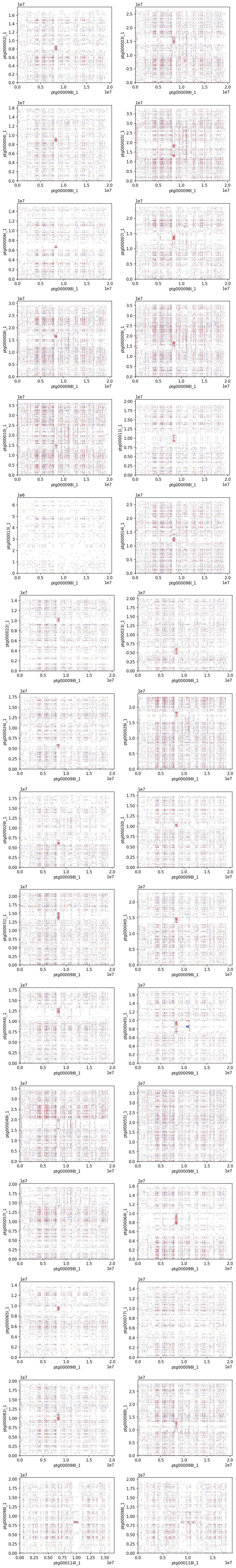

Supplement: S4 File — (ZIP) [file pone.0322885.s004.zip › File_S4/ptg000098l_1.jpg]

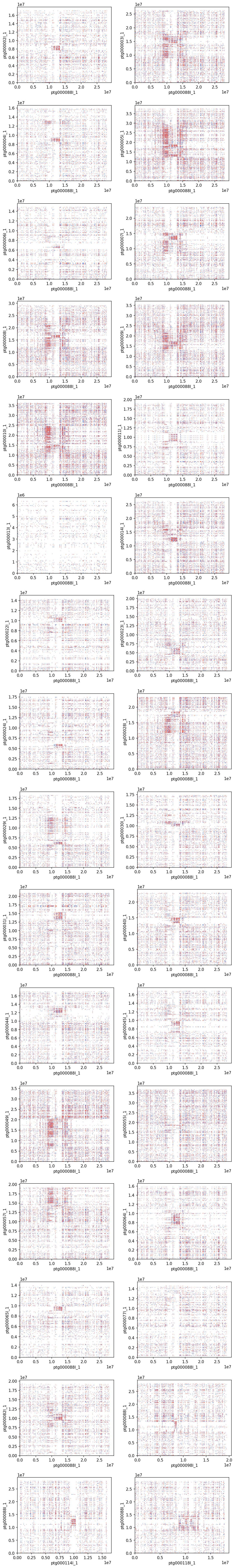

Supplement: S4 File — (ZIP) [file pone.0322885.s004.zip › File_S4/ptg000088l_1.jpg]

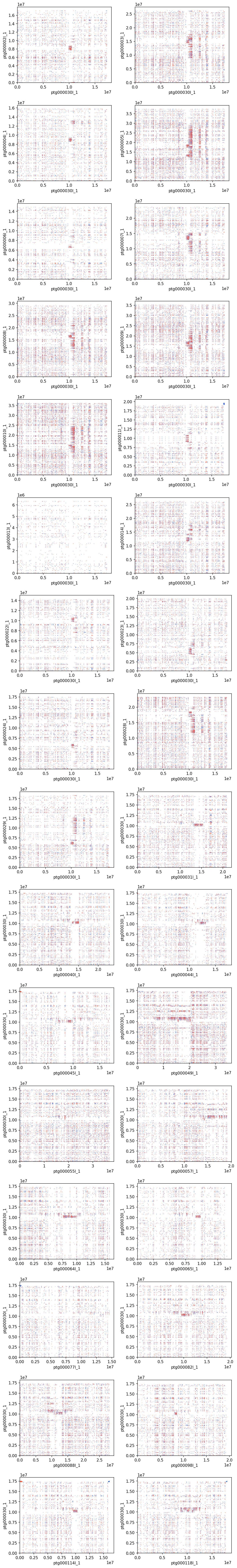

Supplement: S4 File — (ZIP) [file pone.0322885.s004.zip › File_S4/ptg000030l_1.jpg]

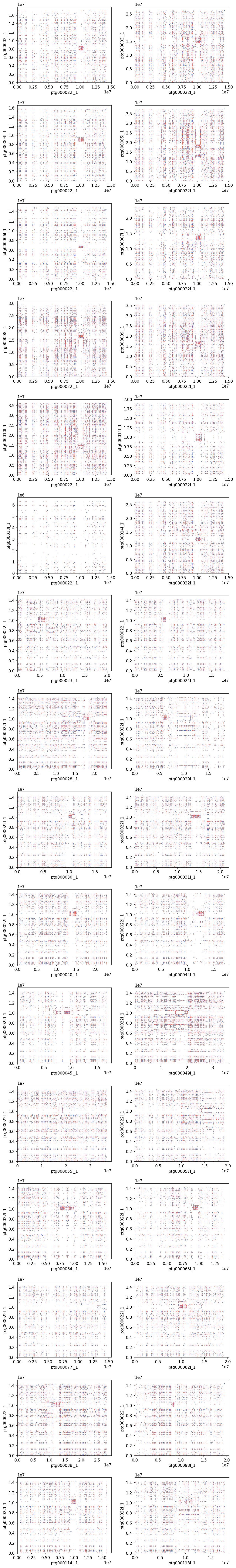

Supplement: S4 File — (ZIP) [file pone.0322885.s004.zip › File_S4/ptg000022l_1.jpg]

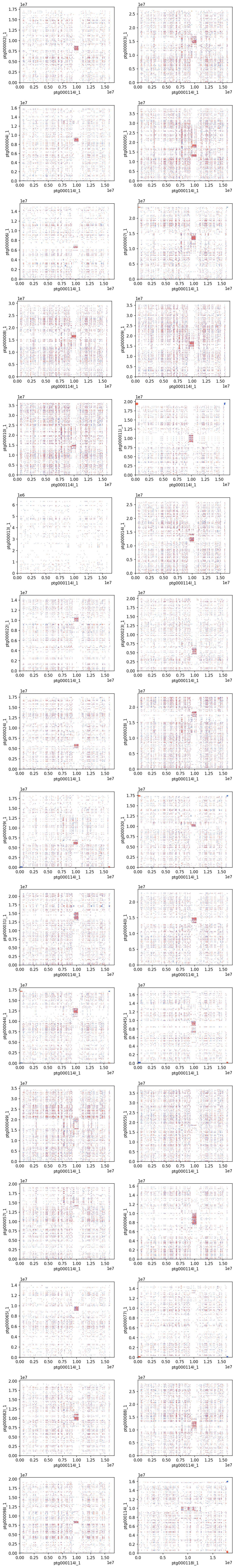

Supplement: S4 File — (ZIP) [file pone.0322885.s004.zip › File_S4/ptg000114l_1.jpg]

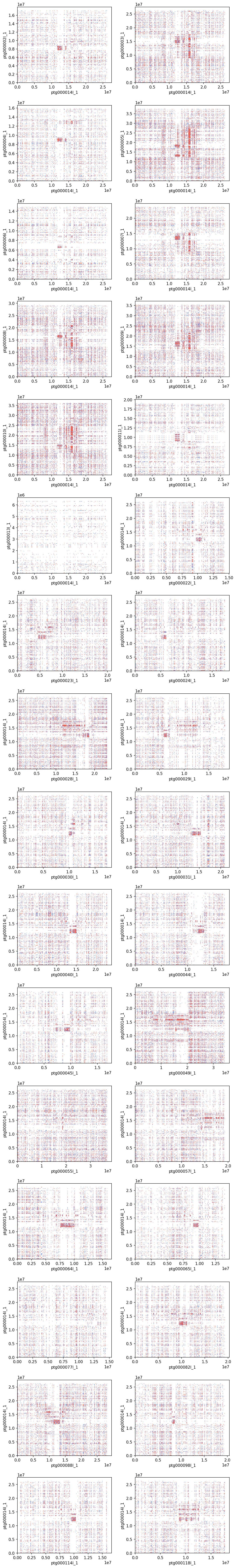

Supplement: S4 File — (ZIP) [file pone.0322885.s004.zip › File_S4/ptg000014l_1.jpg]

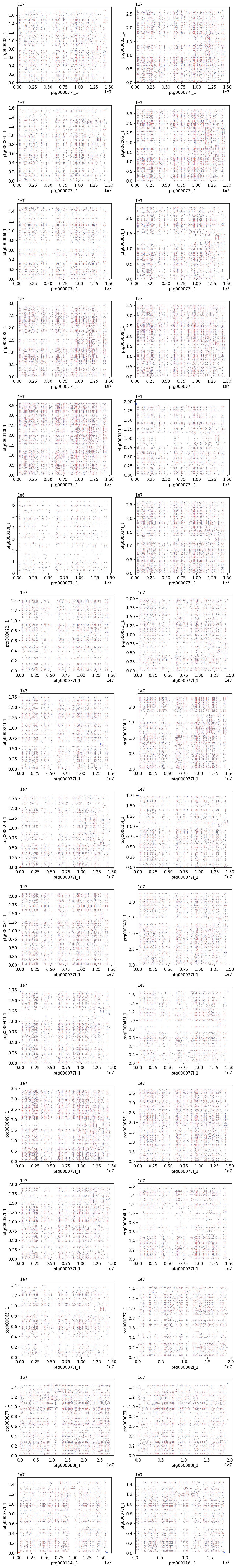

Supplement: S4 File — (ZIP) [file pone.0322885.s004.zip › File_S4/ptg000077l_1.jpg]

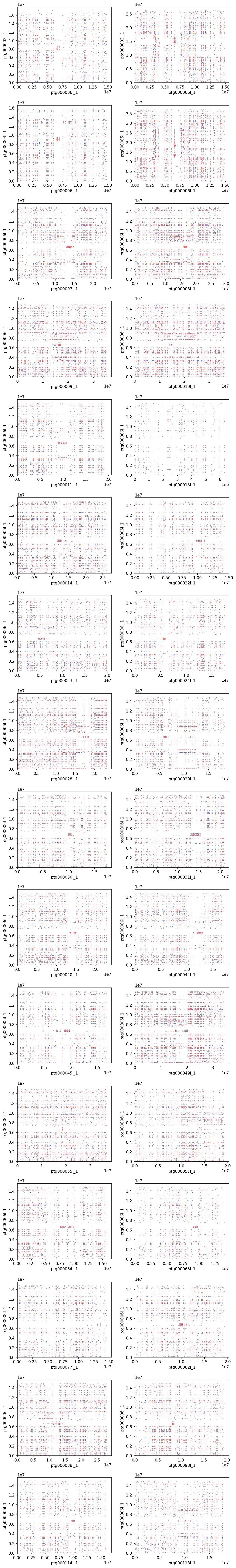

Supplement: S4 File — (ZIP) [file pone.0322885.s004.zip › File_S4/ptg000006l_1.jpg]

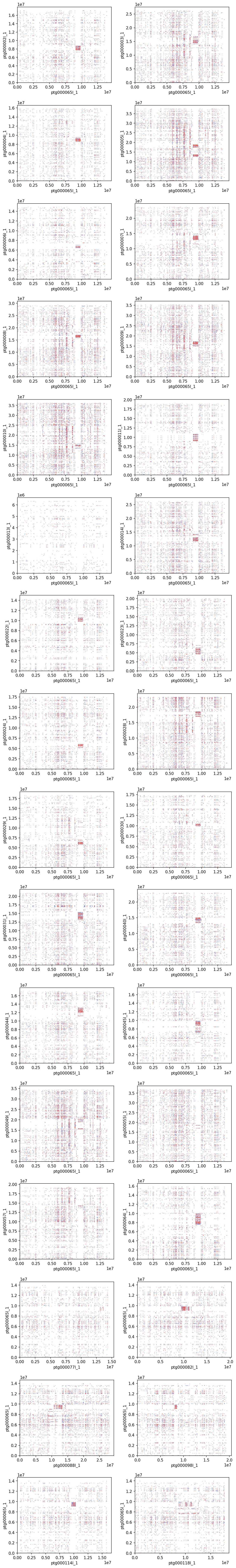

Supplement: S4 File — (ZIP) [file pone.0322885.s004.zip › File_S4/ptg000065l_1.jpg]

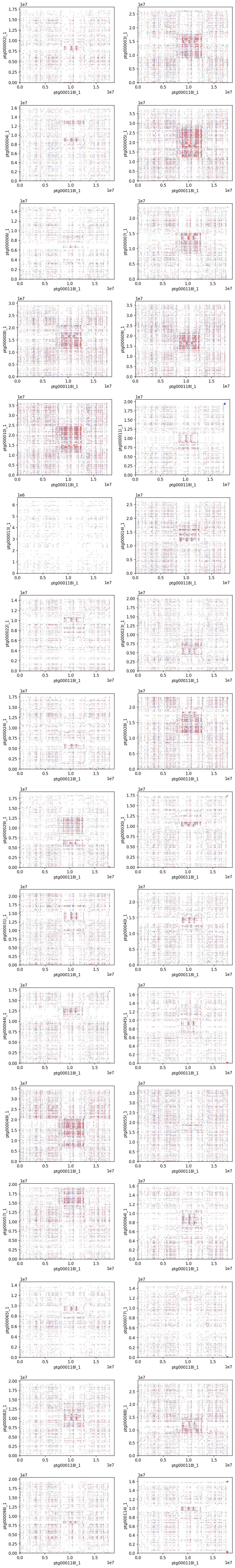

Supplement: S4 File — (ZIP) [file pone.0322885.s004.zip › File_S4/ptg000118l_1.jpg]

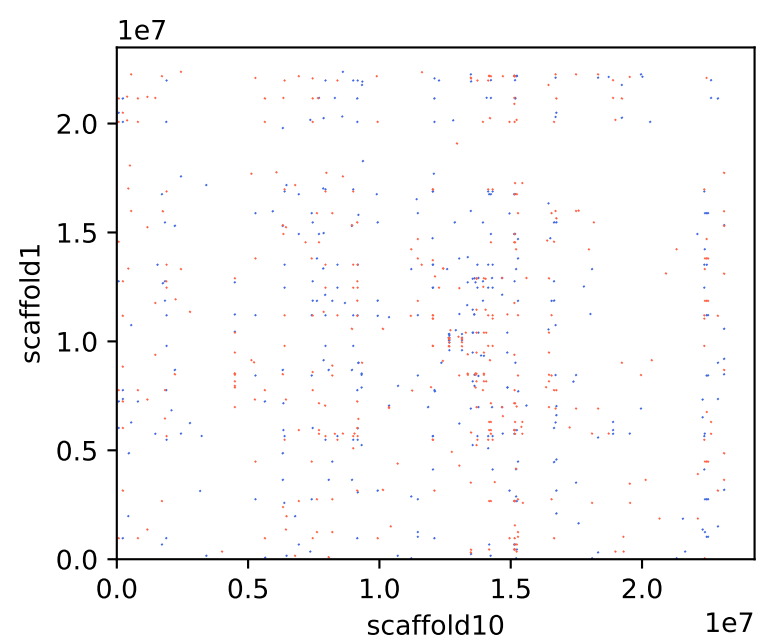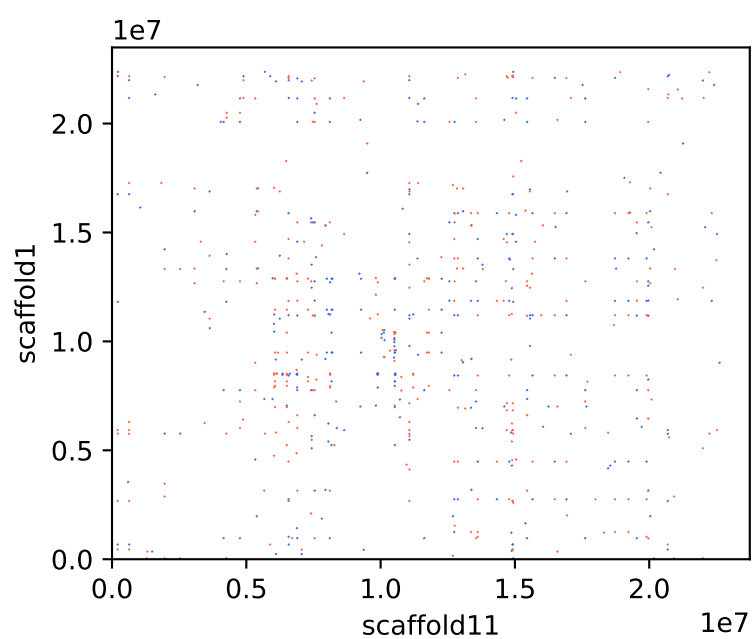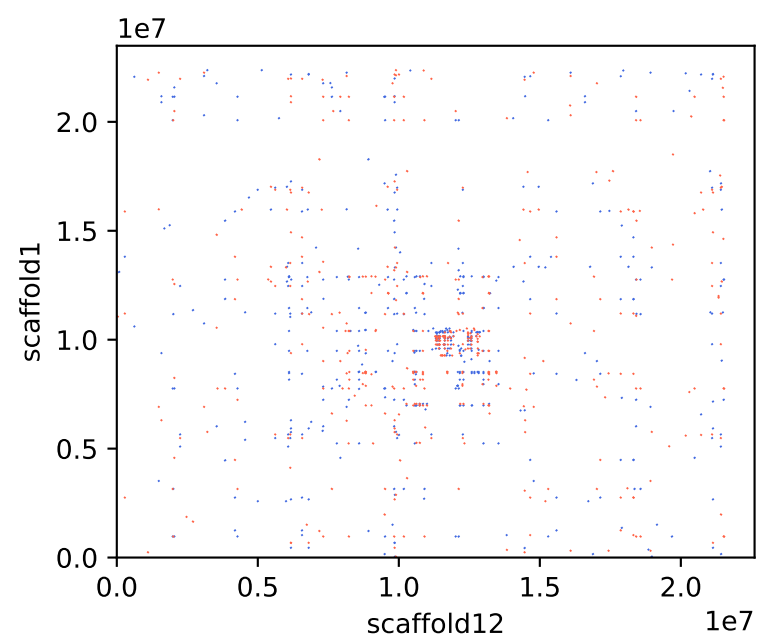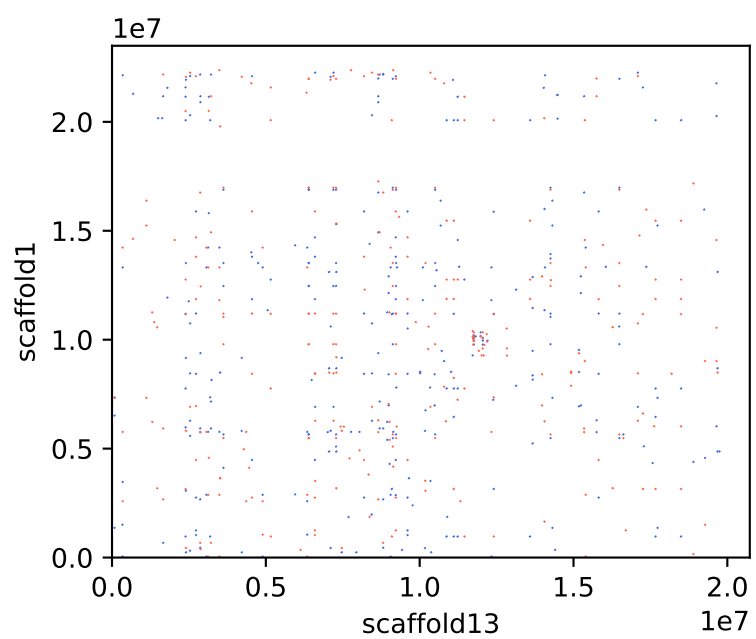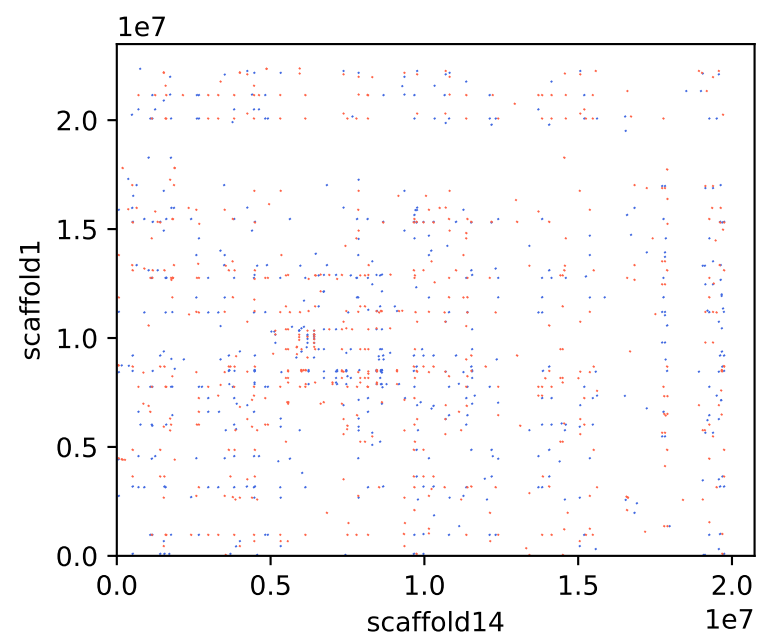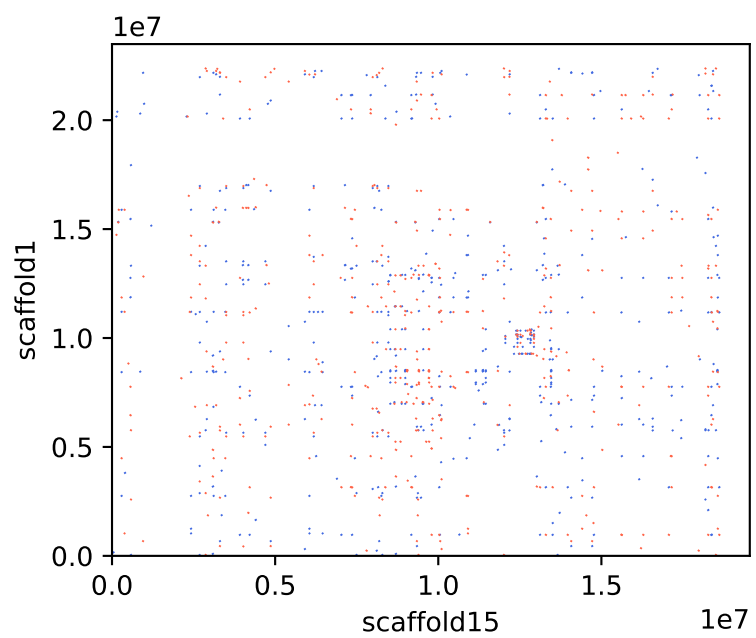

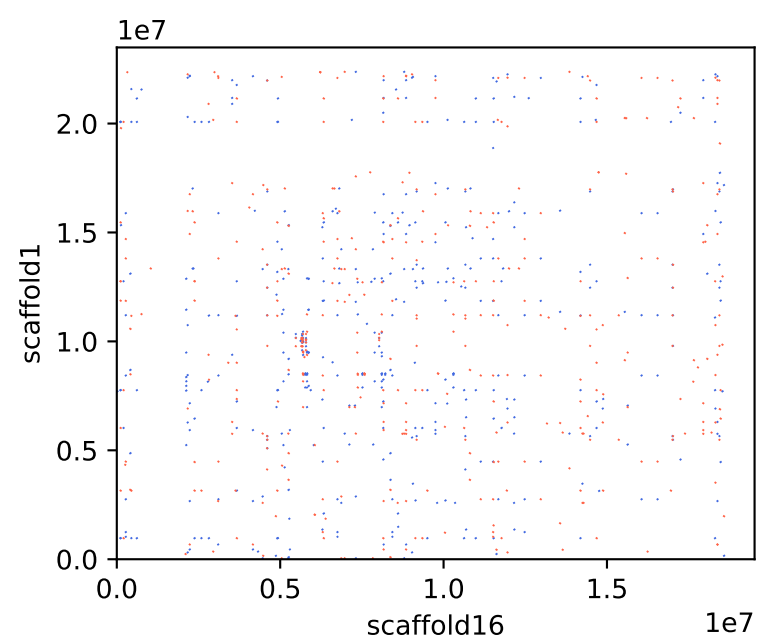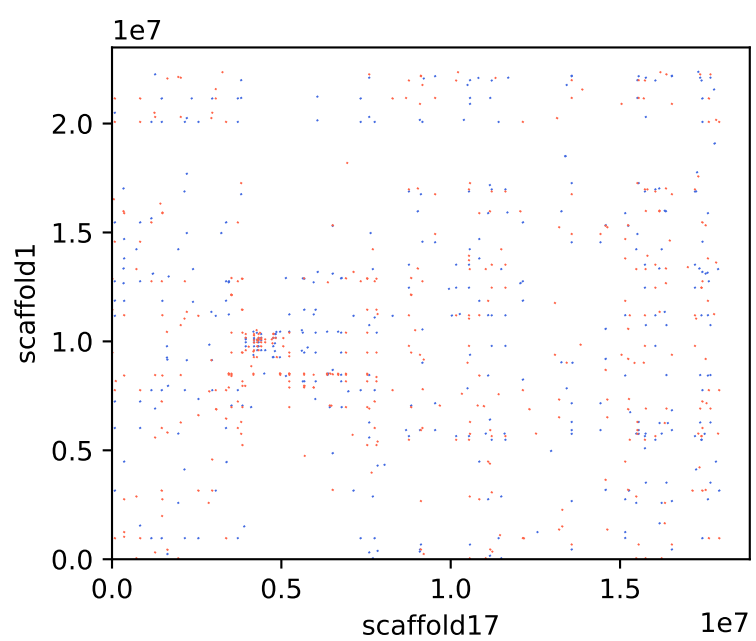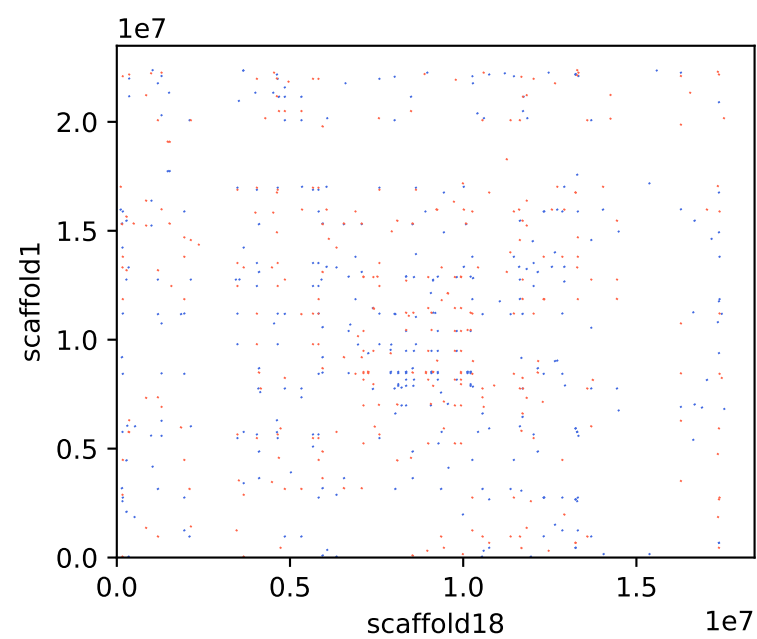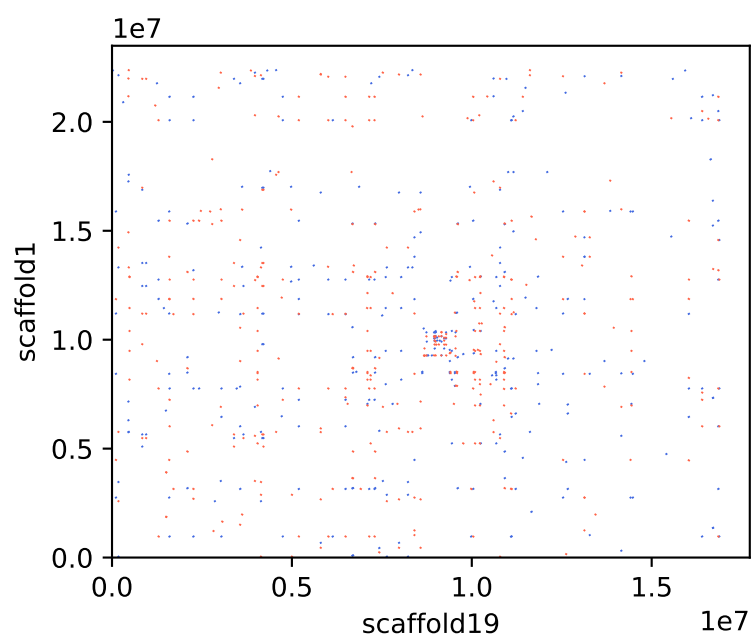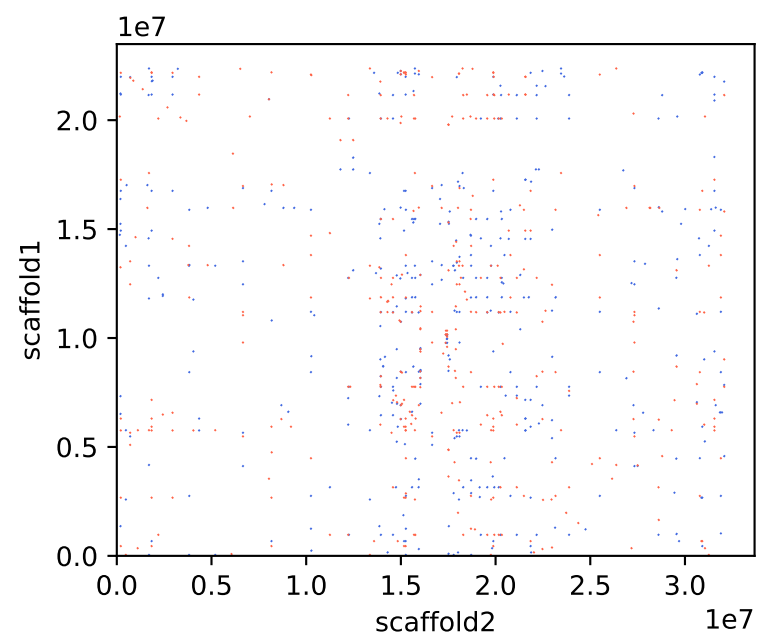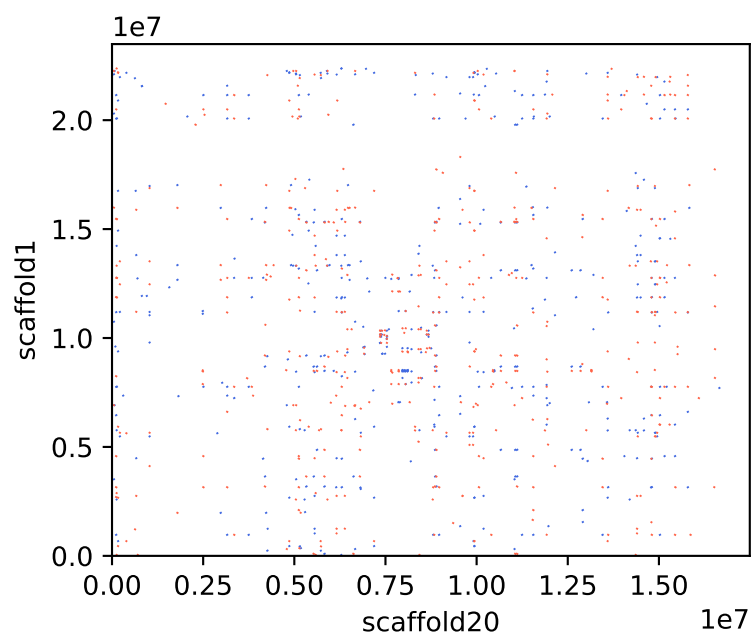

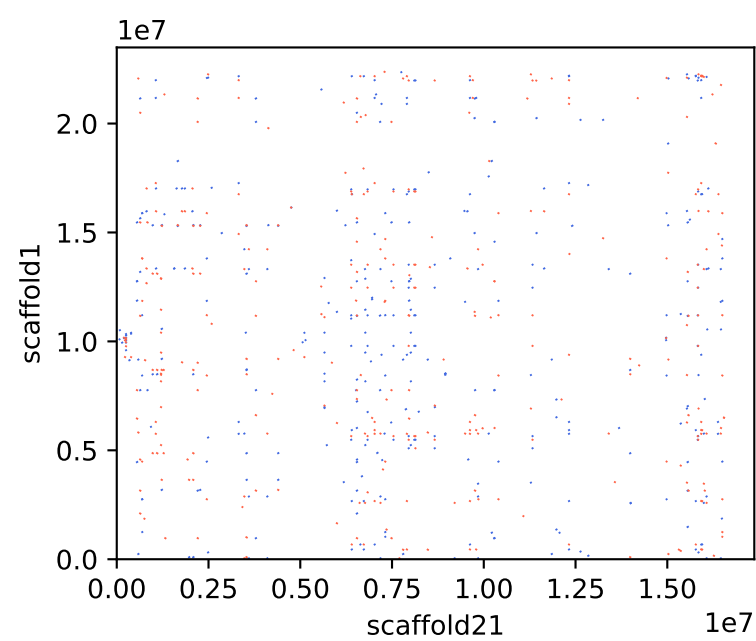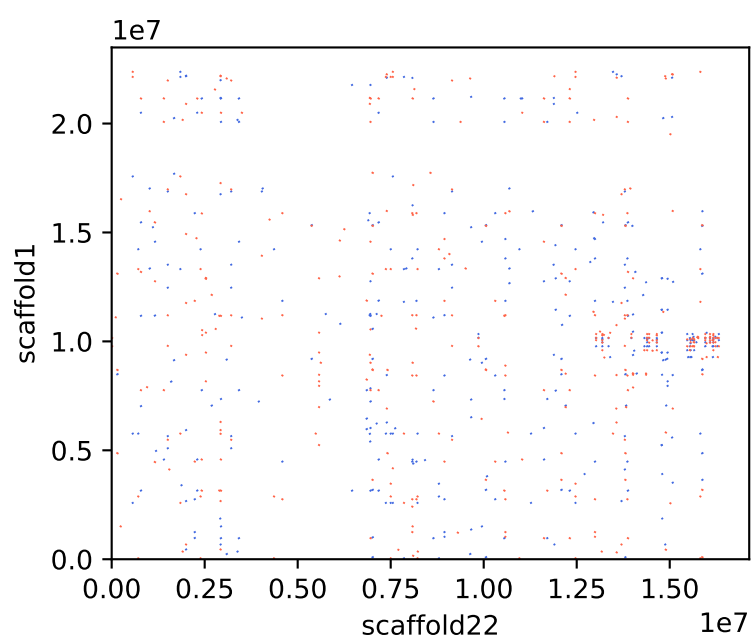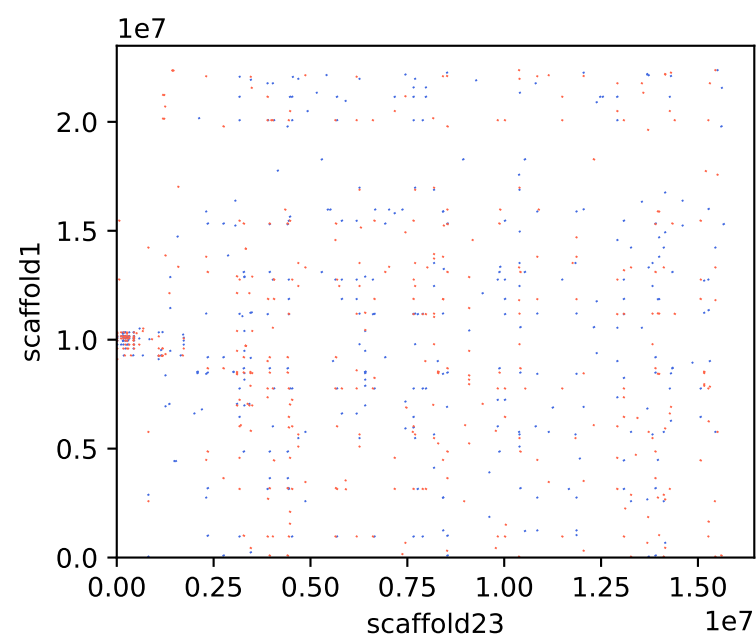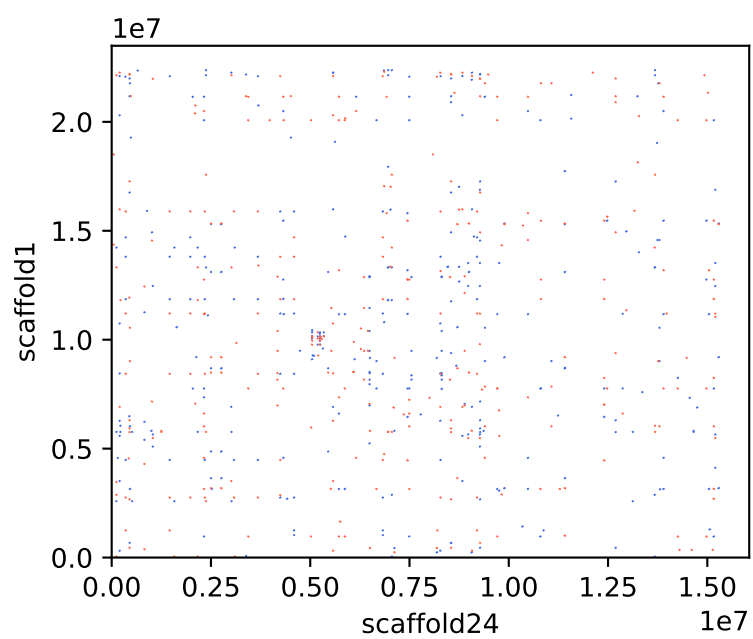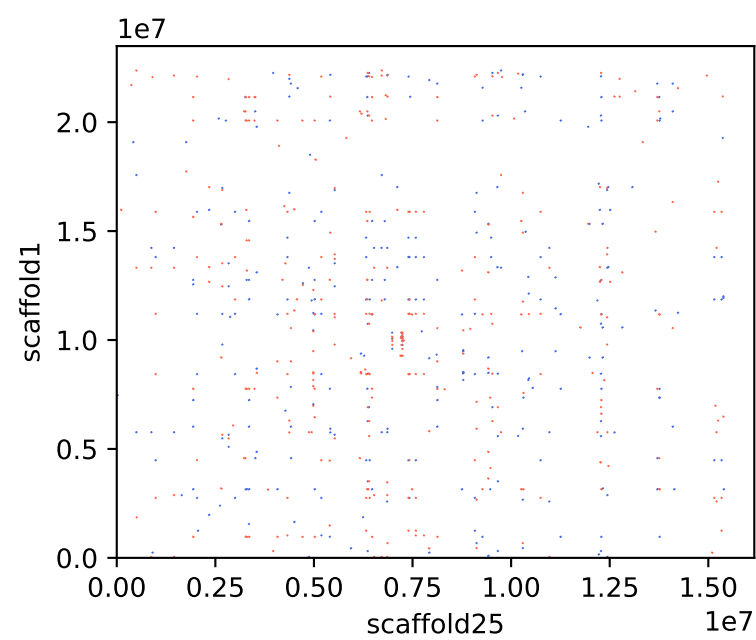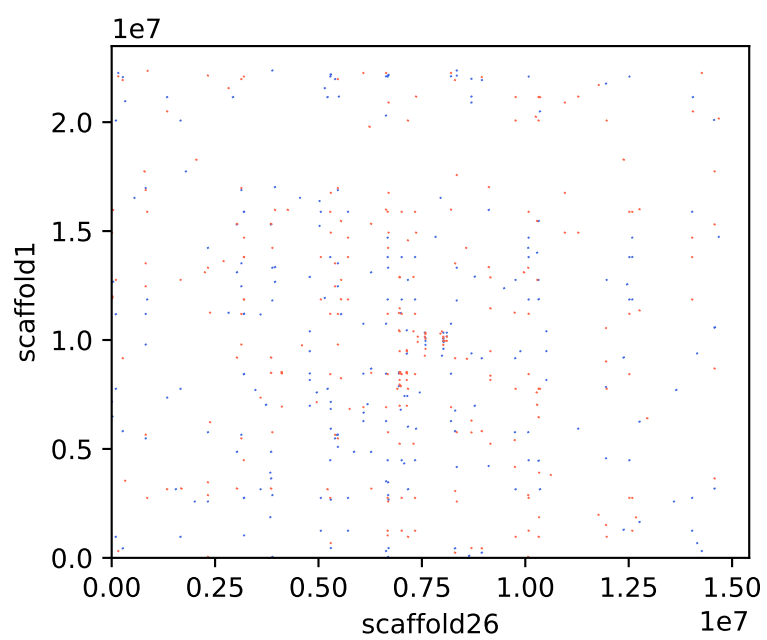

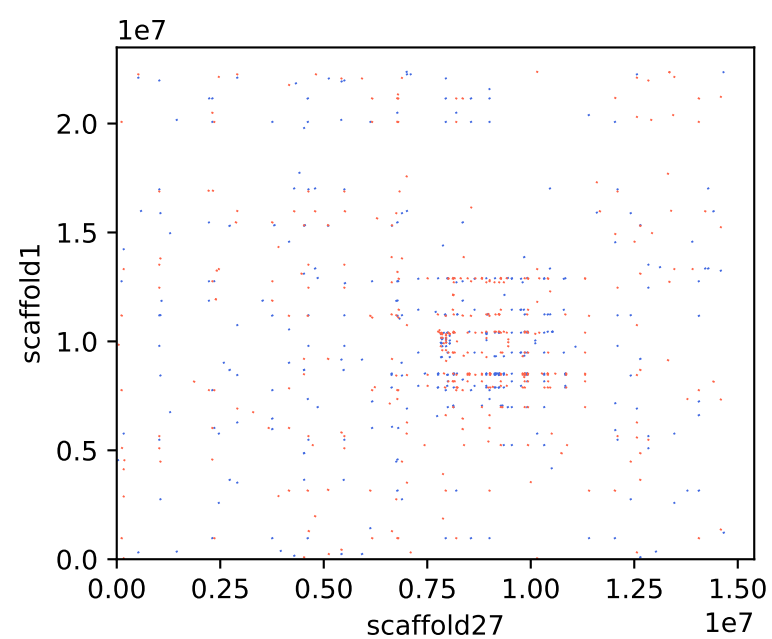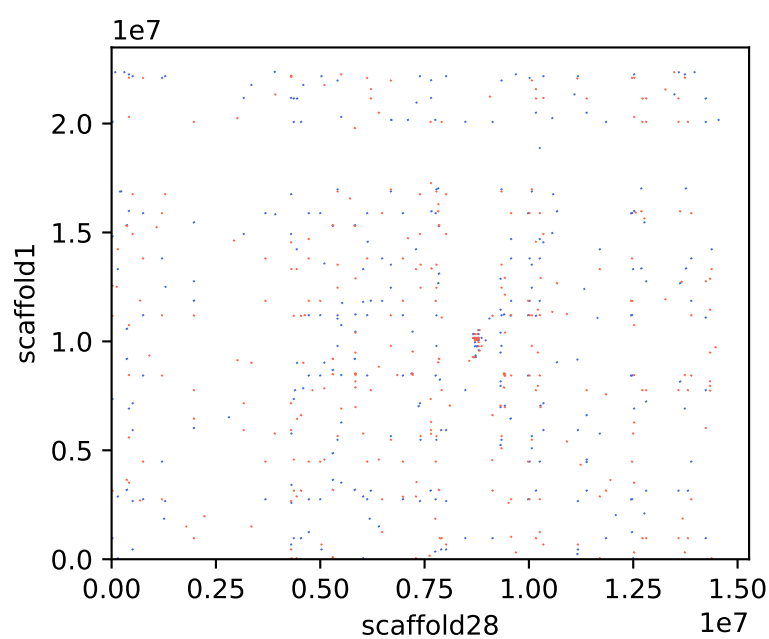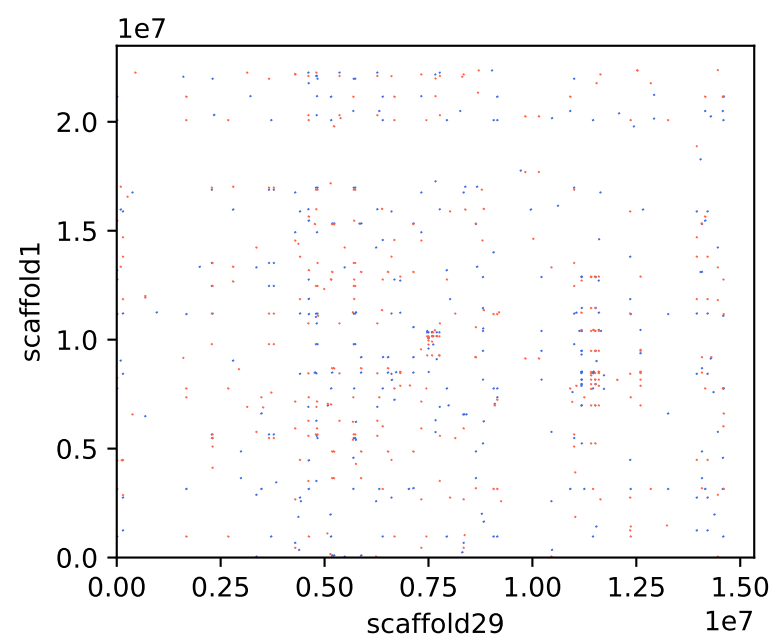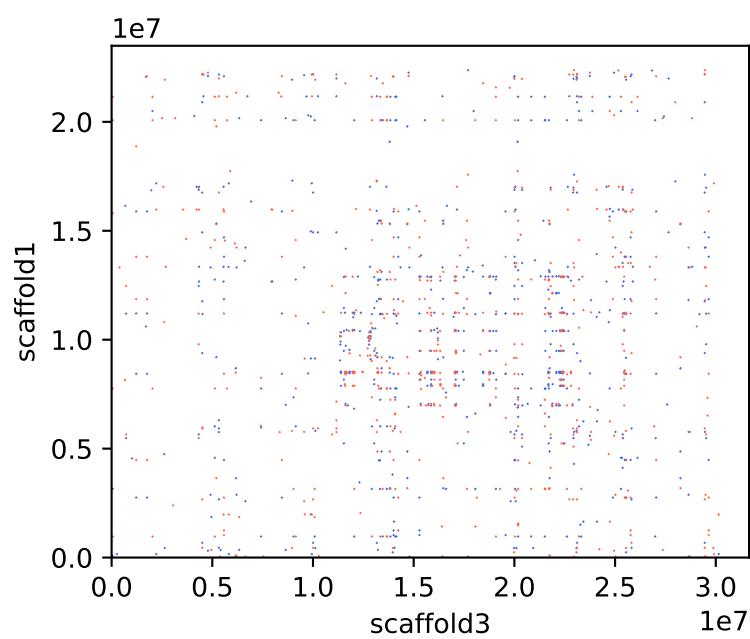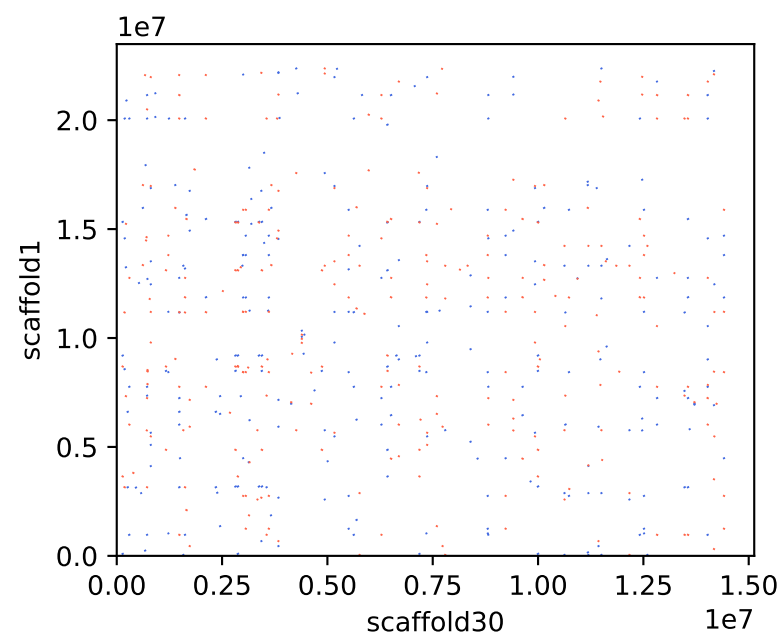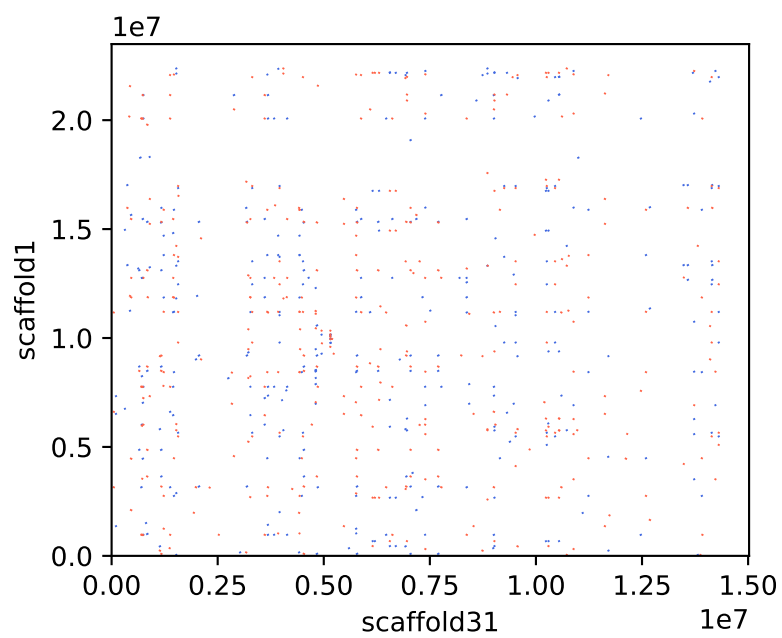

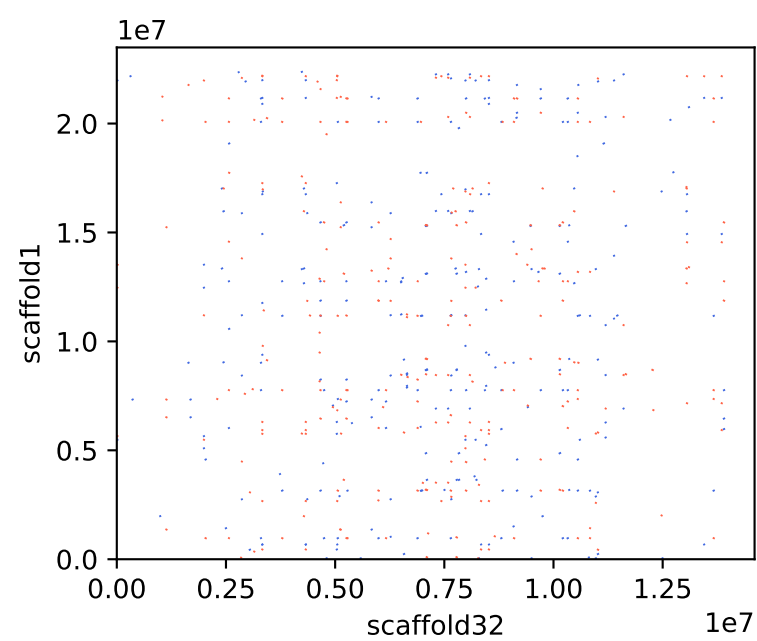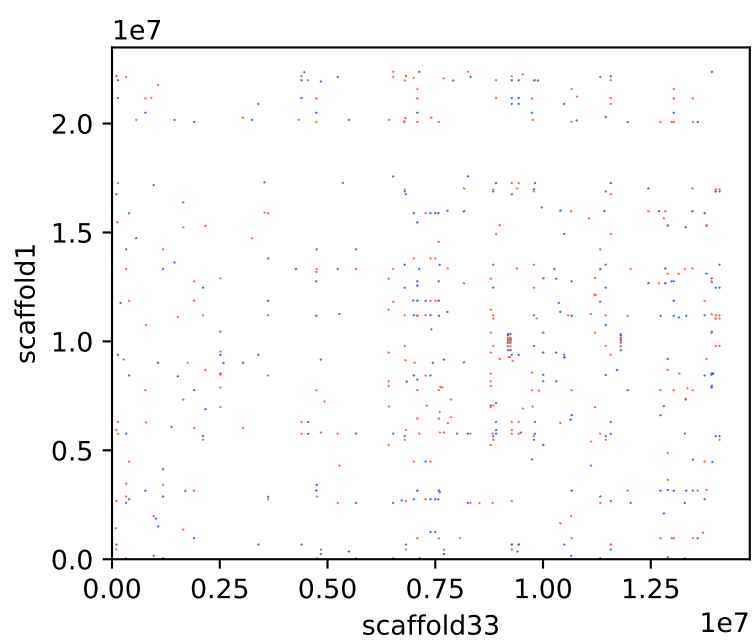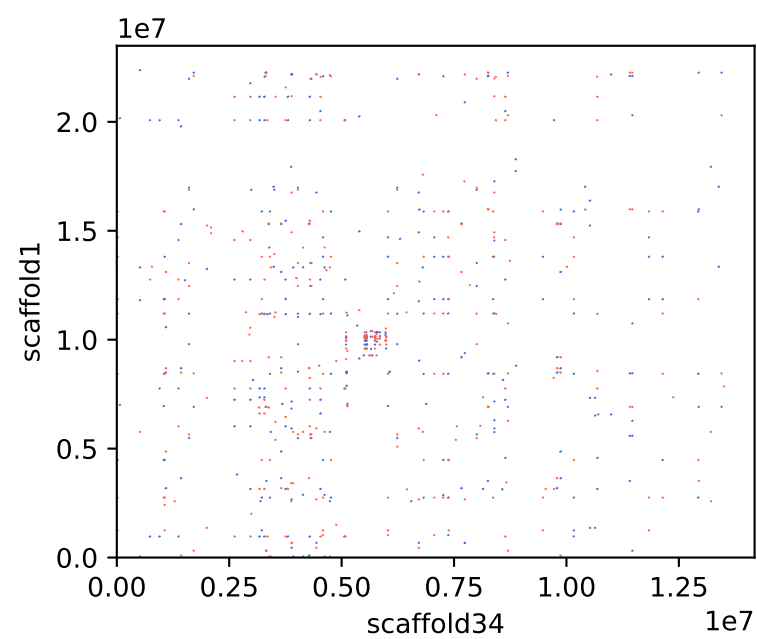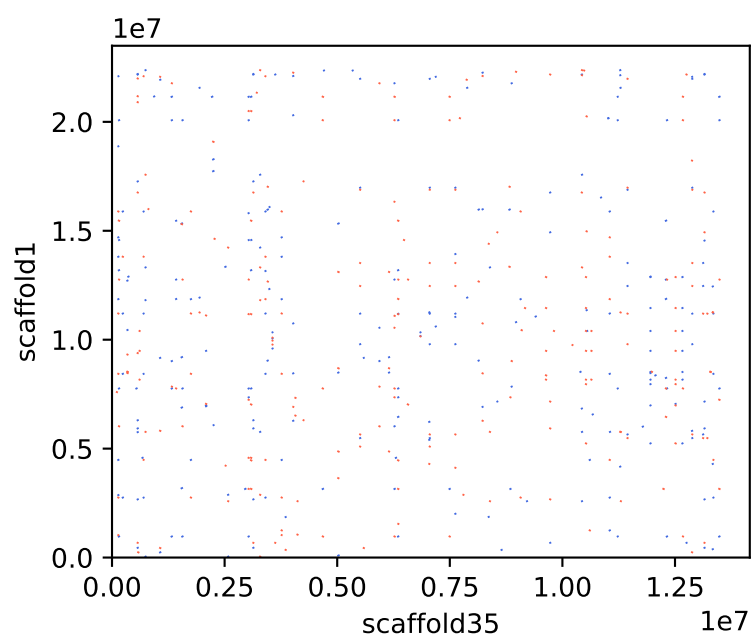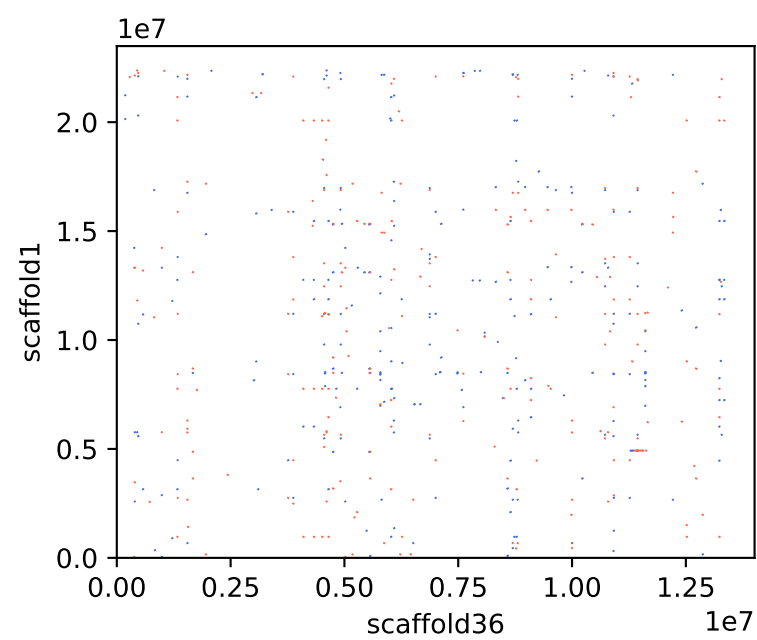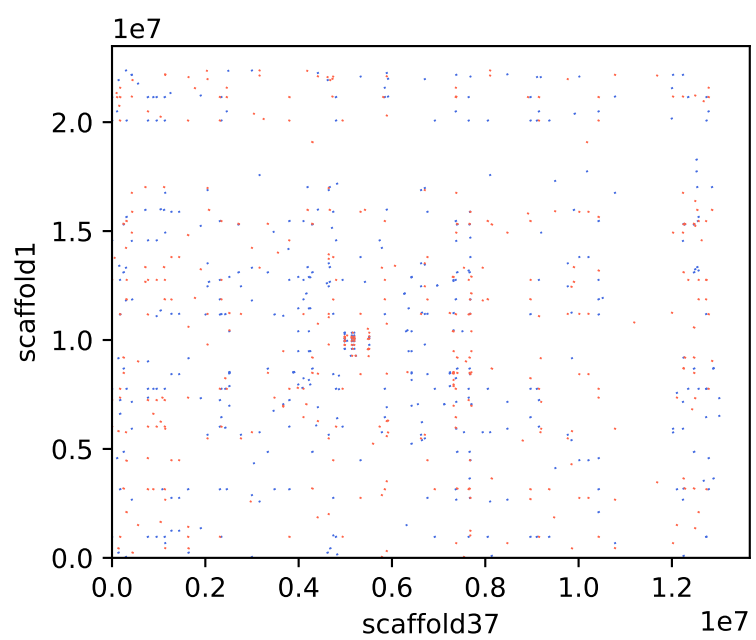

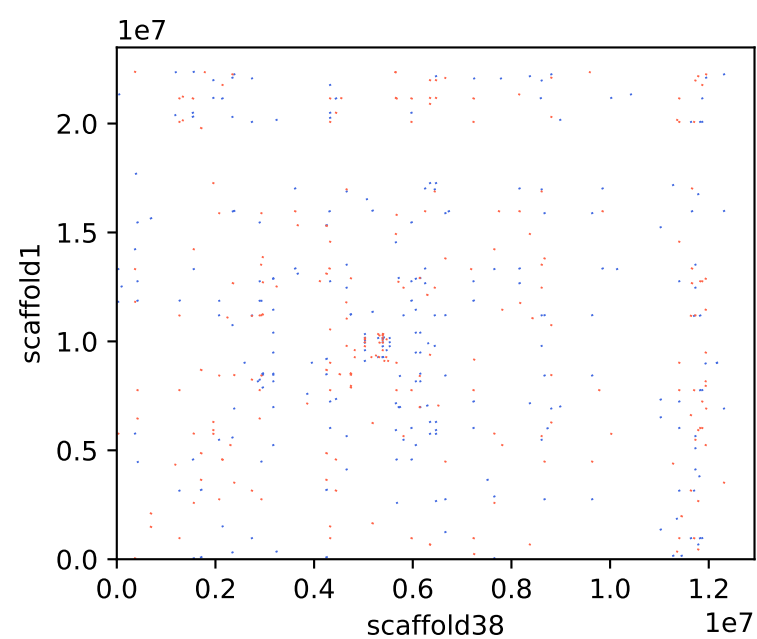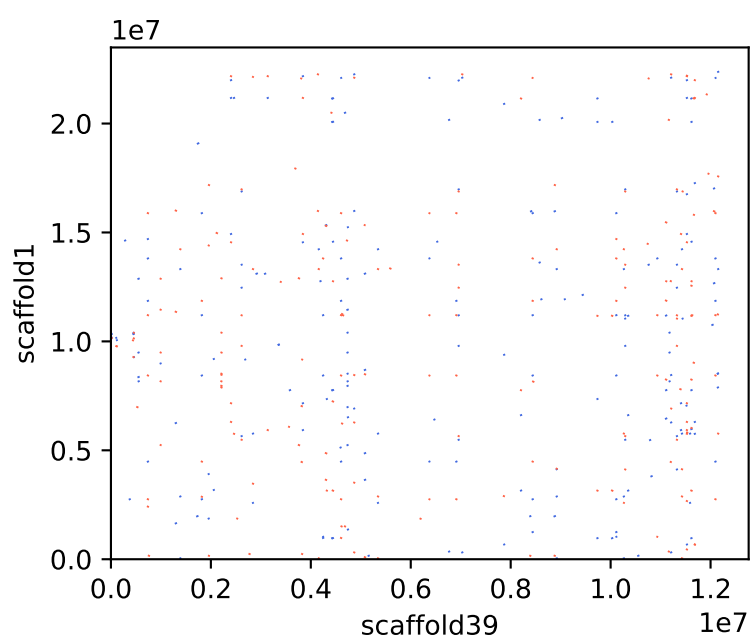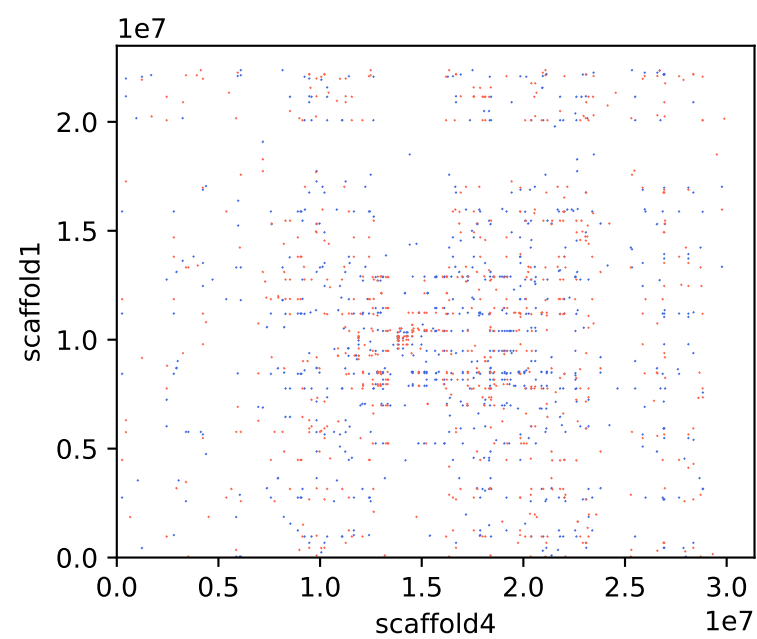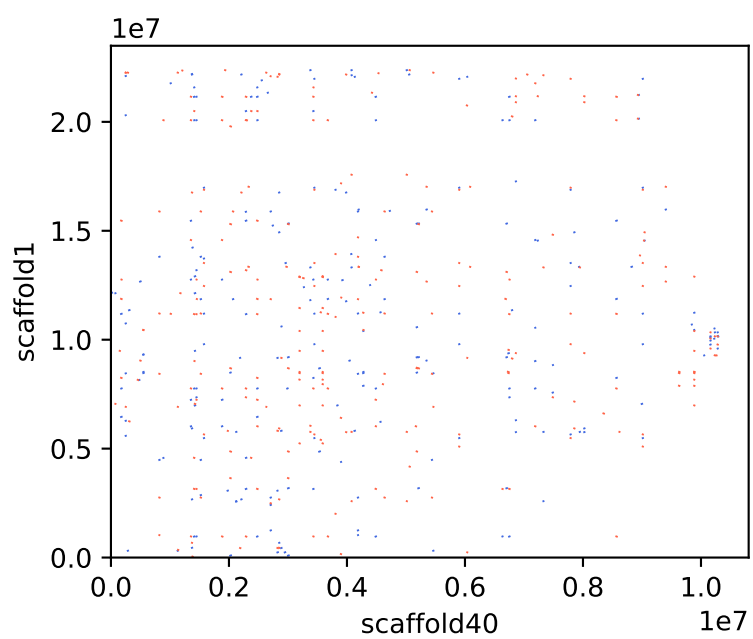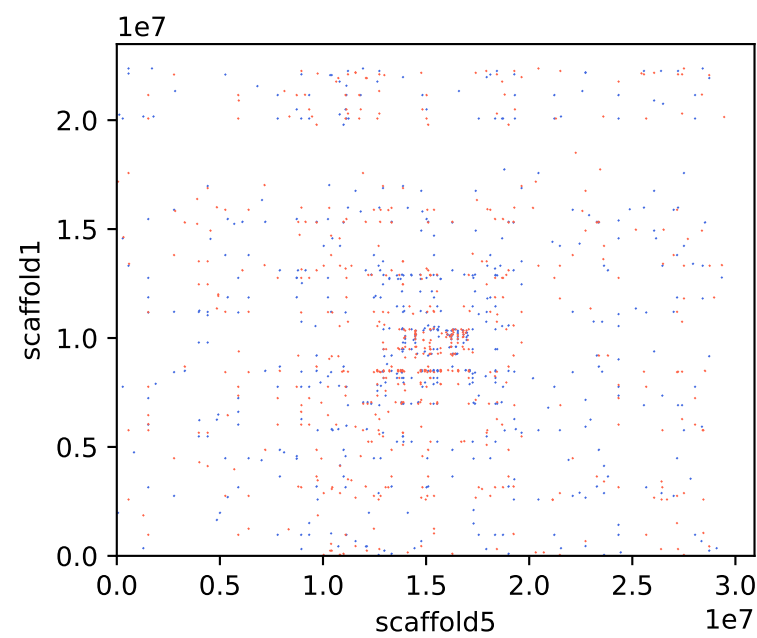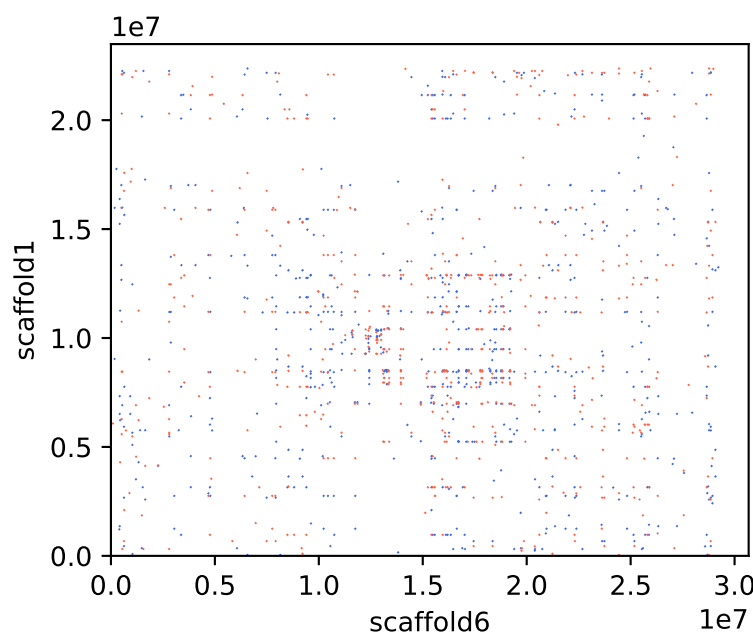

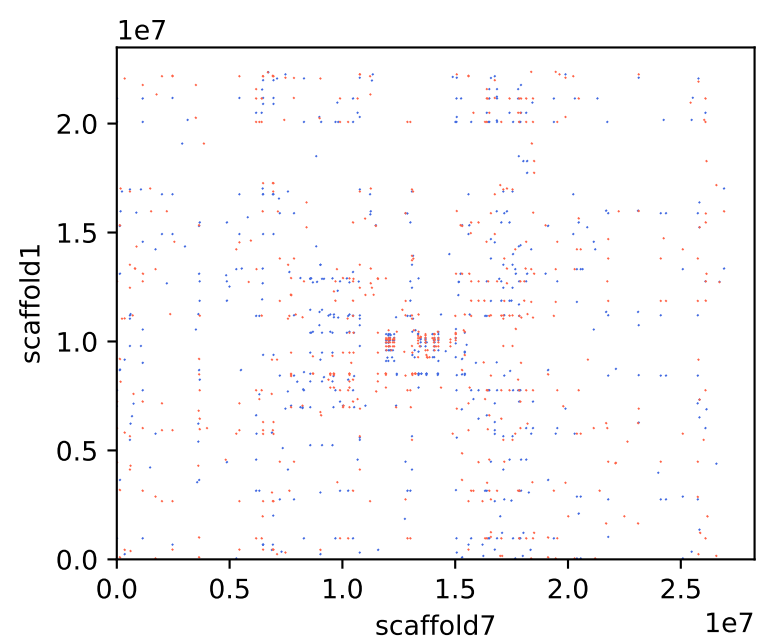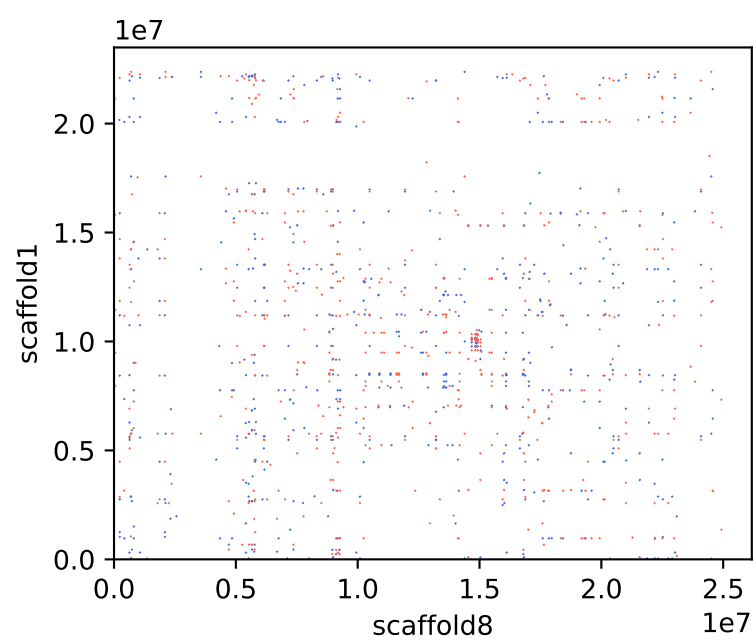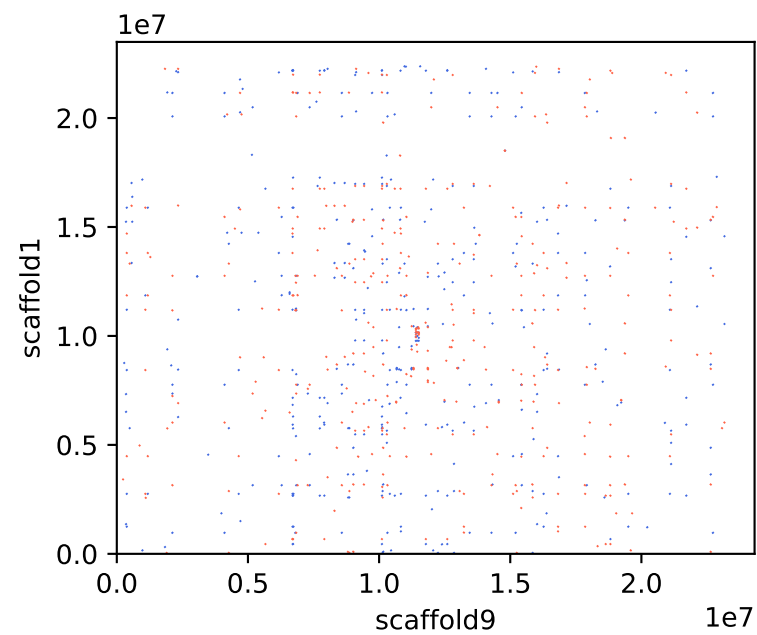

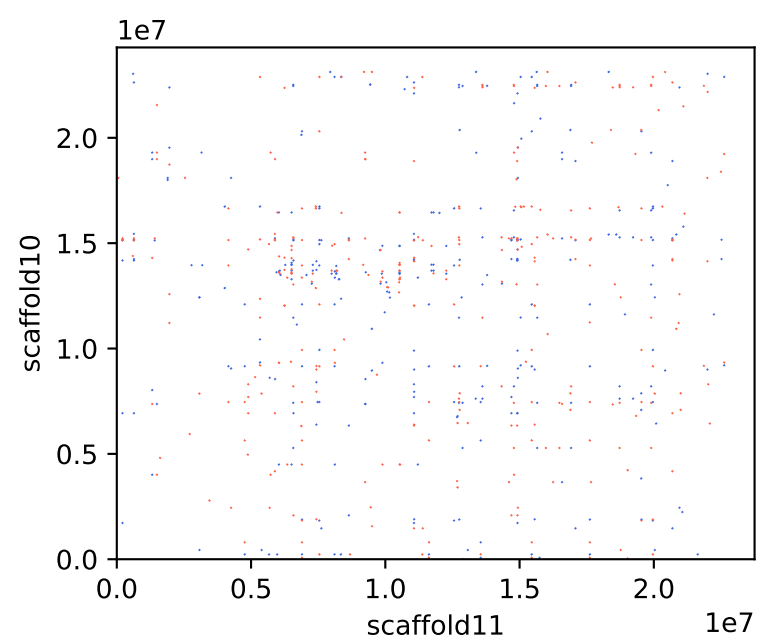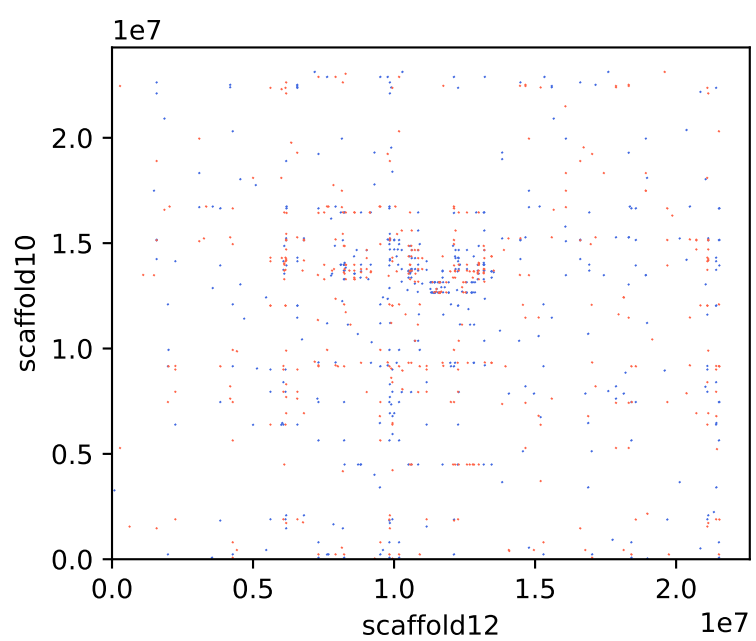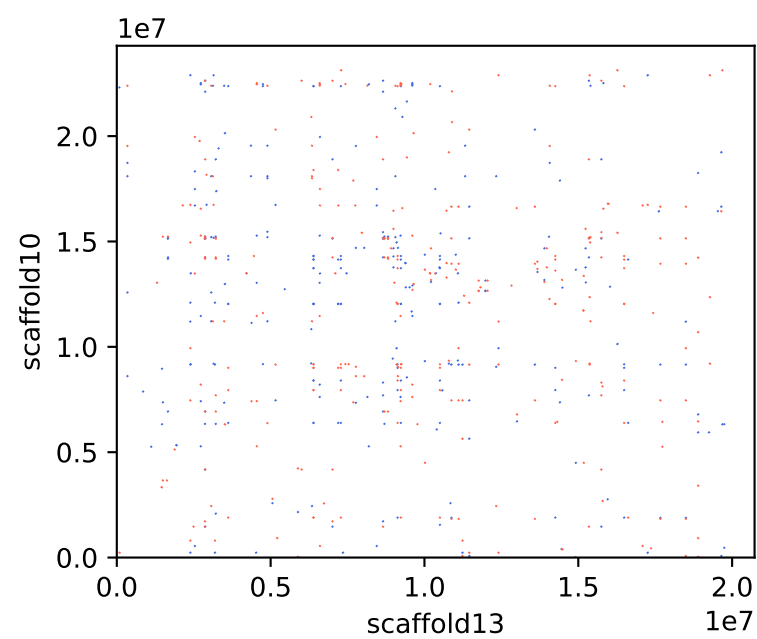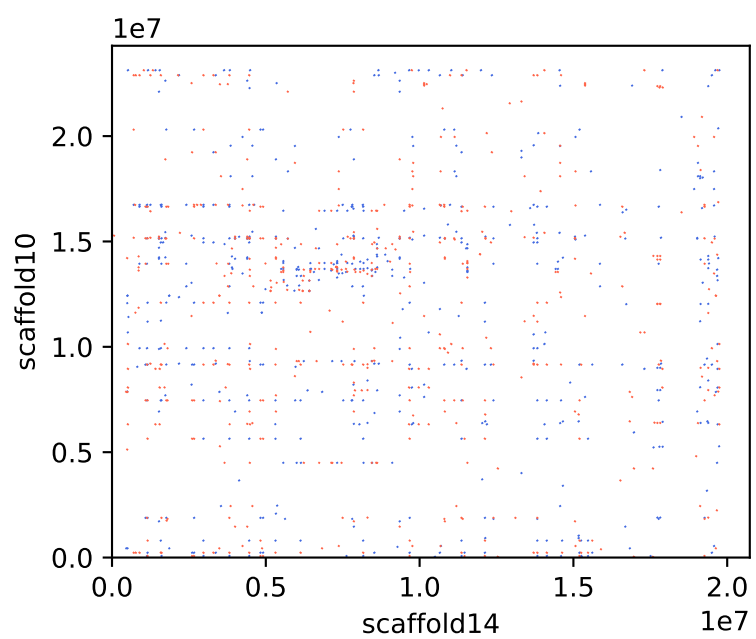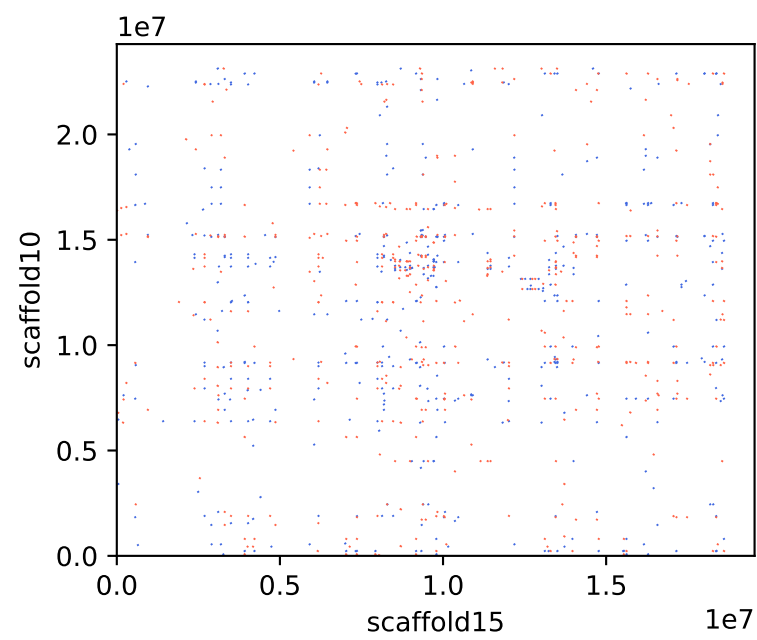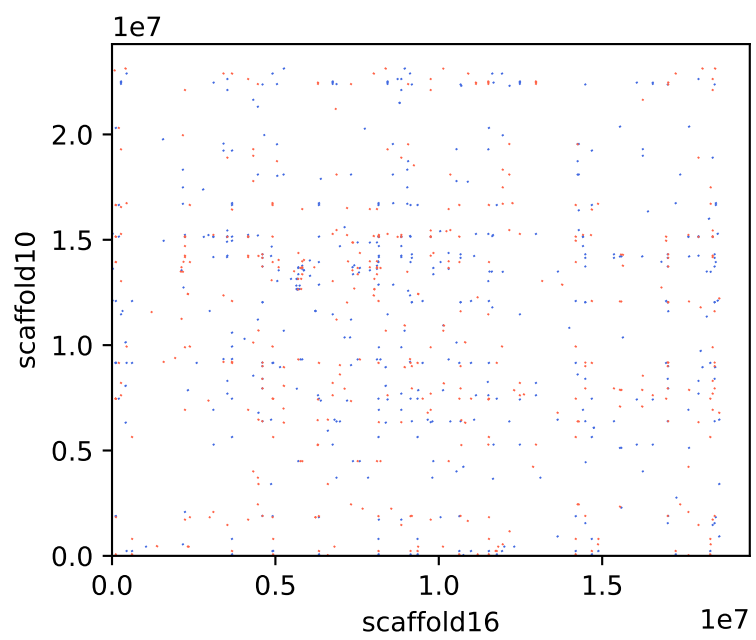

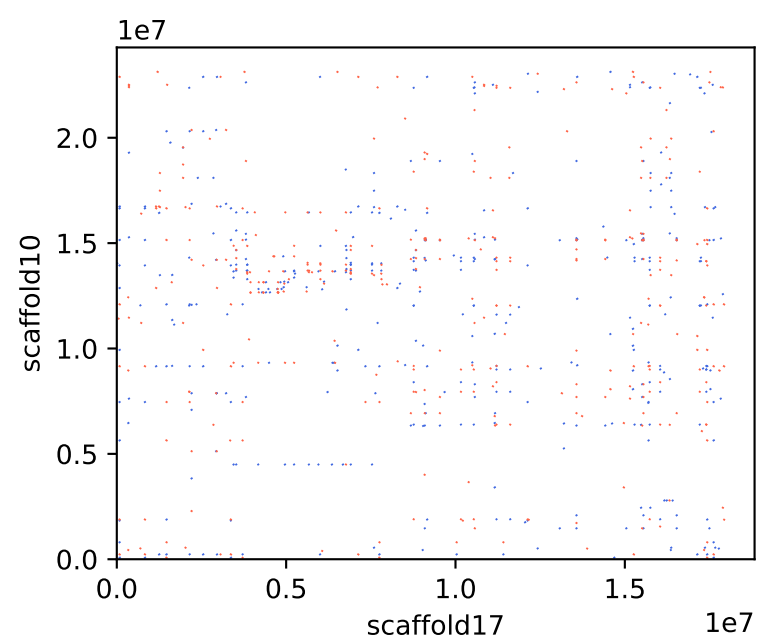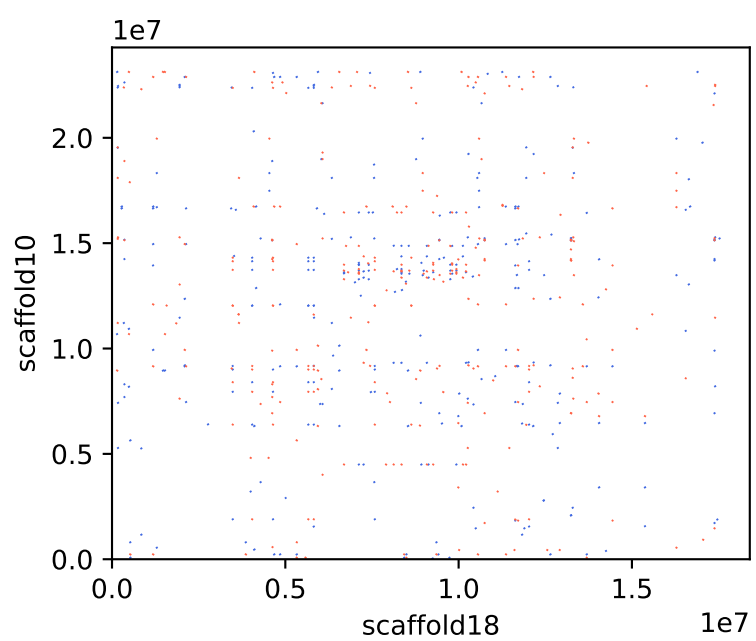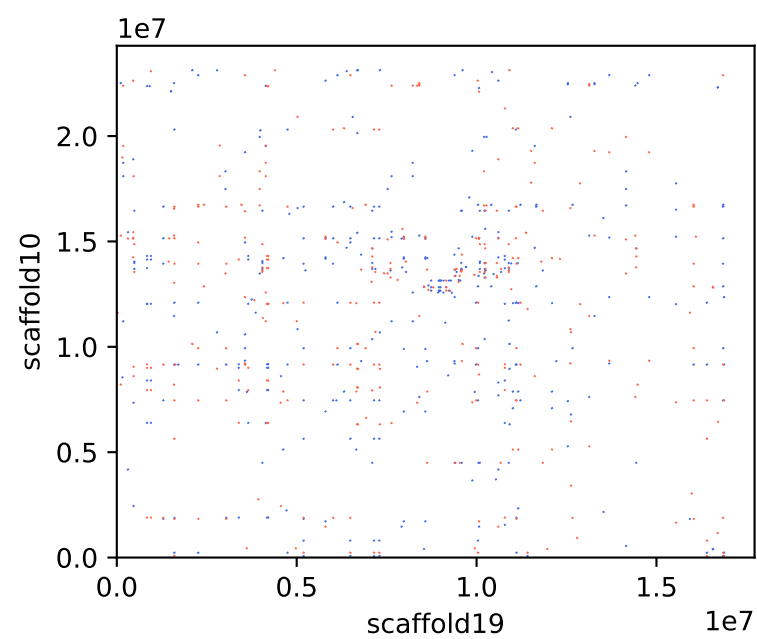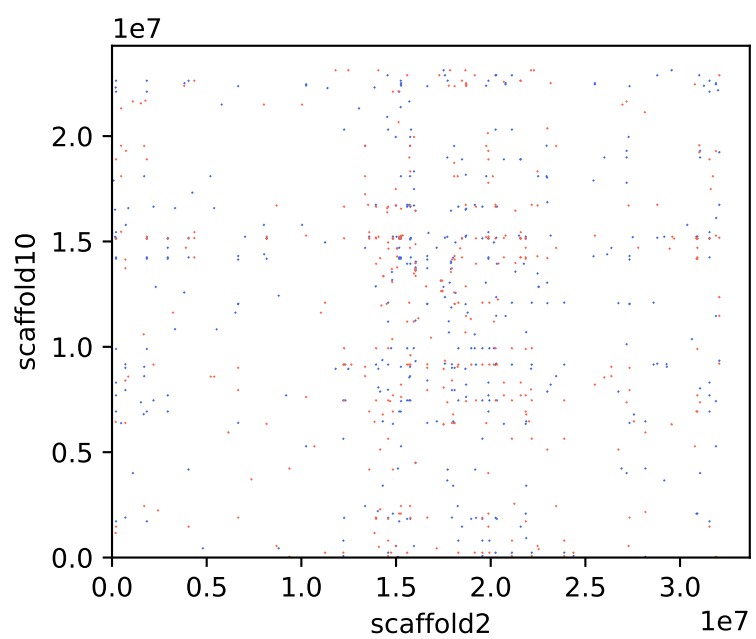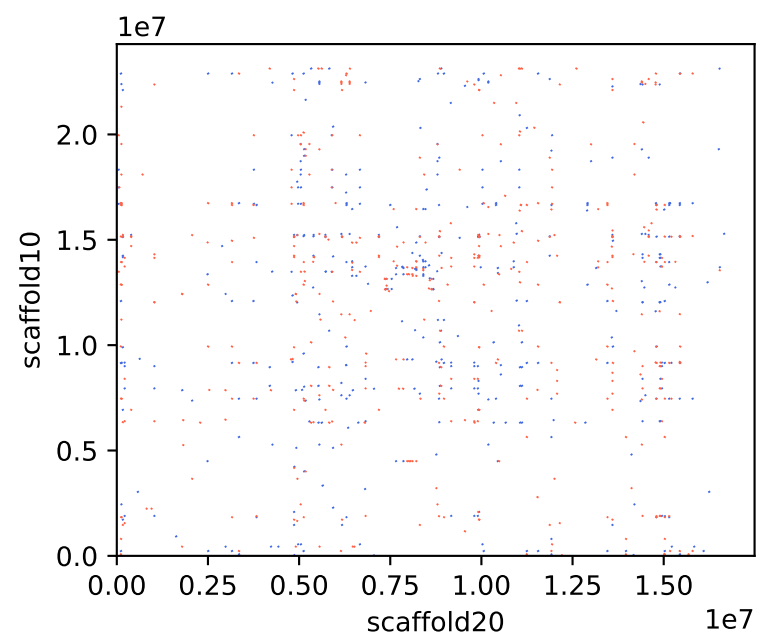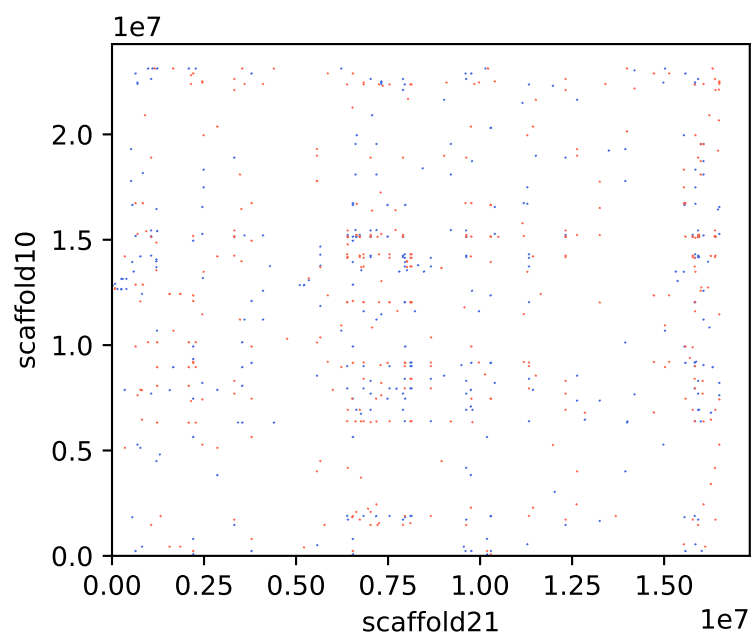

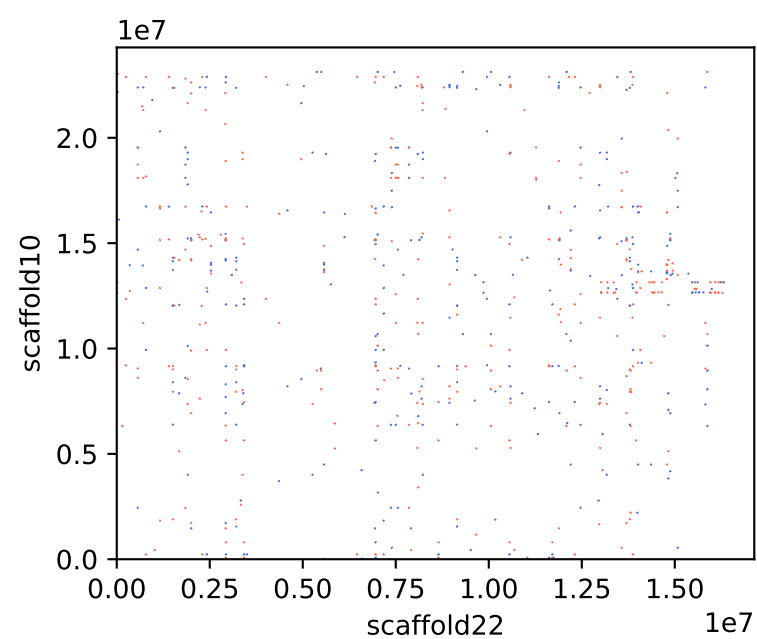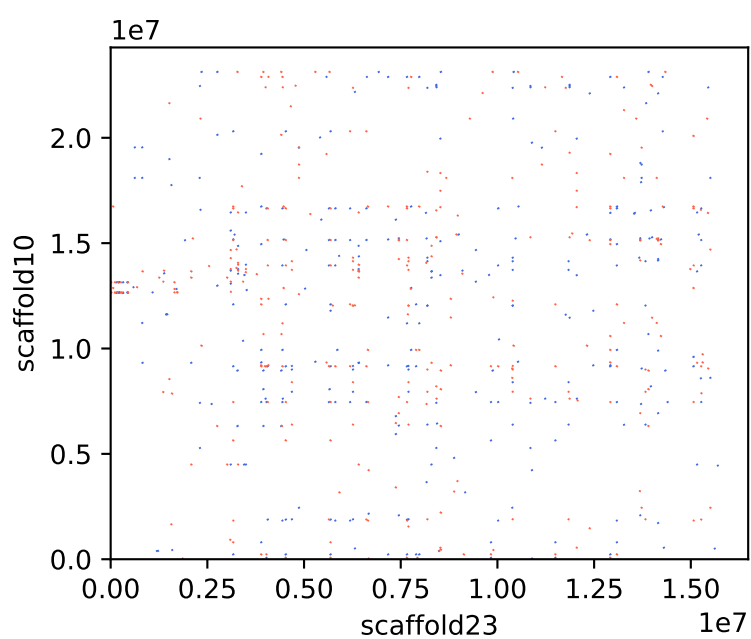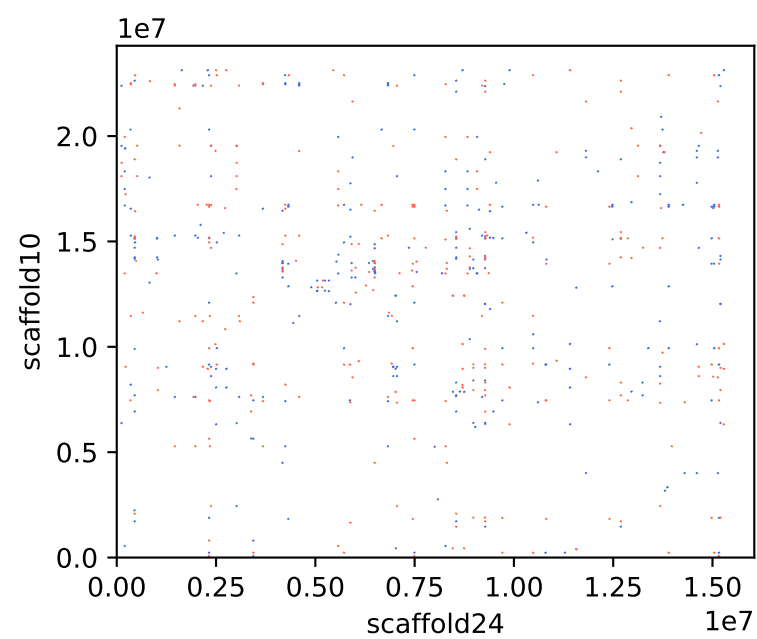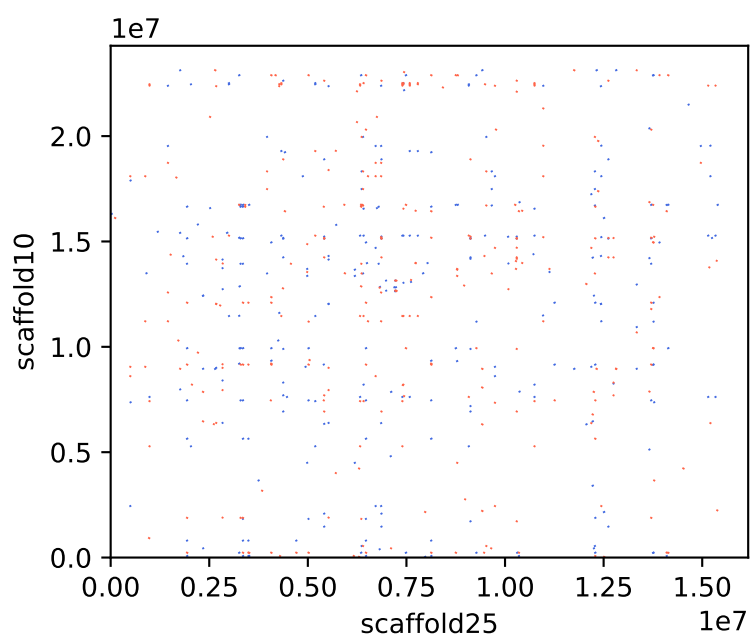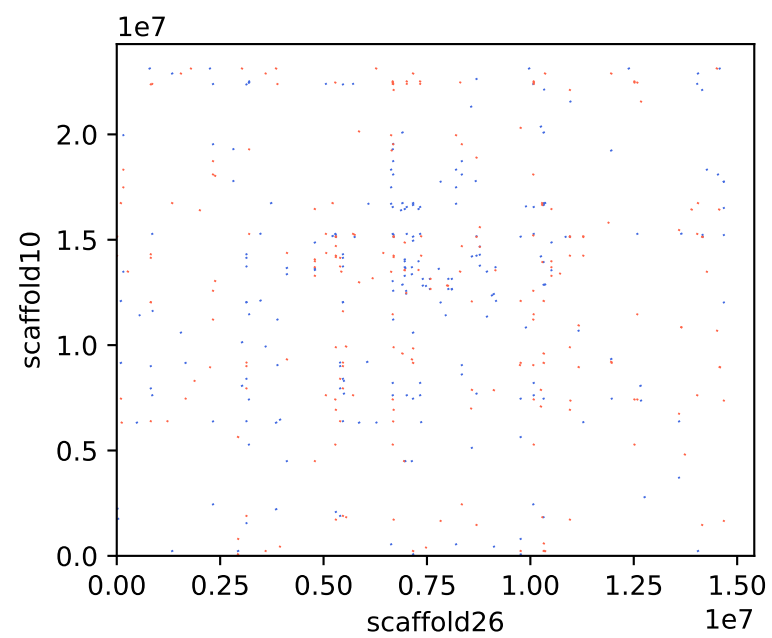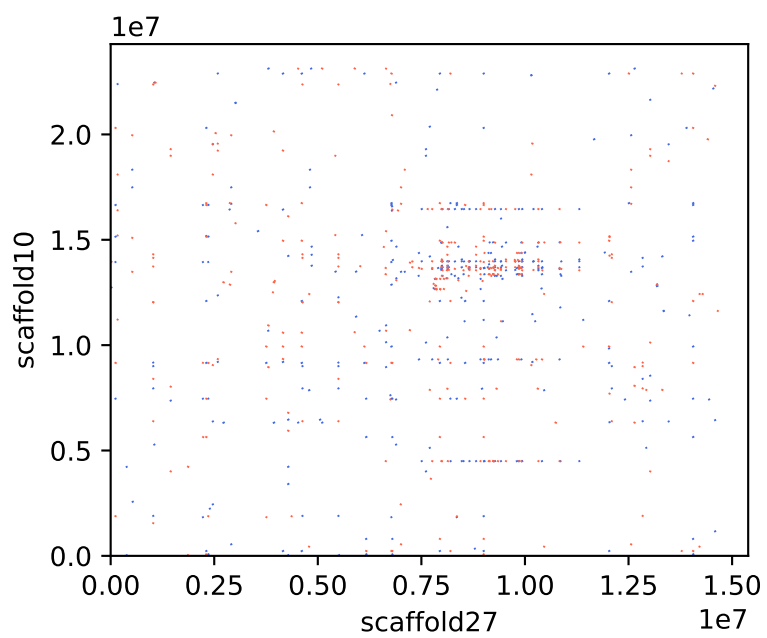

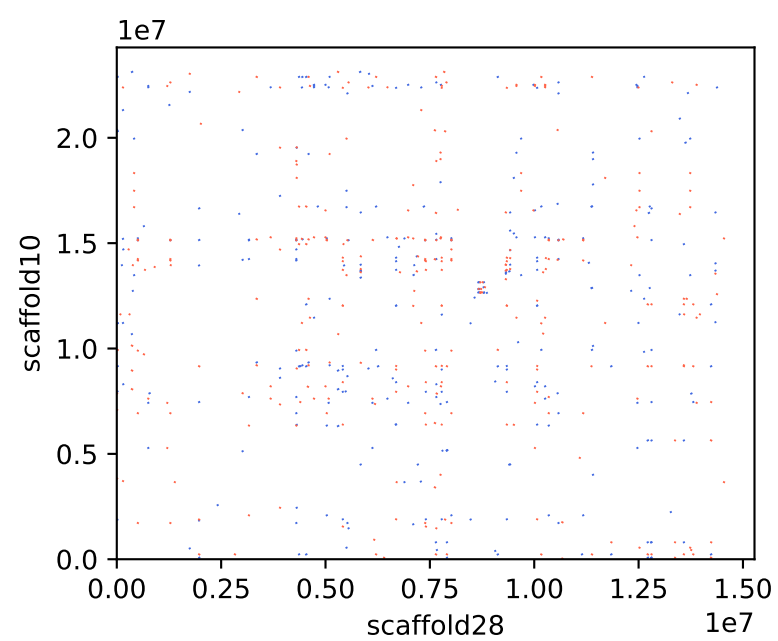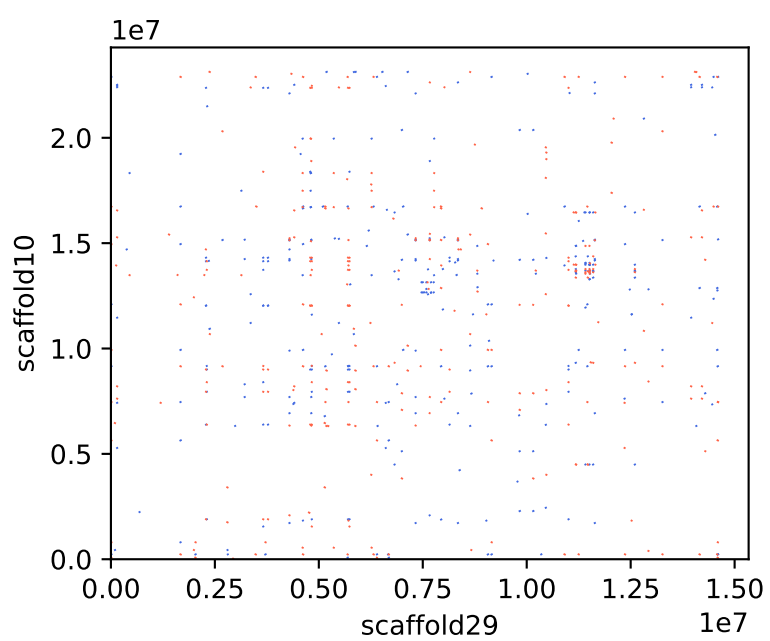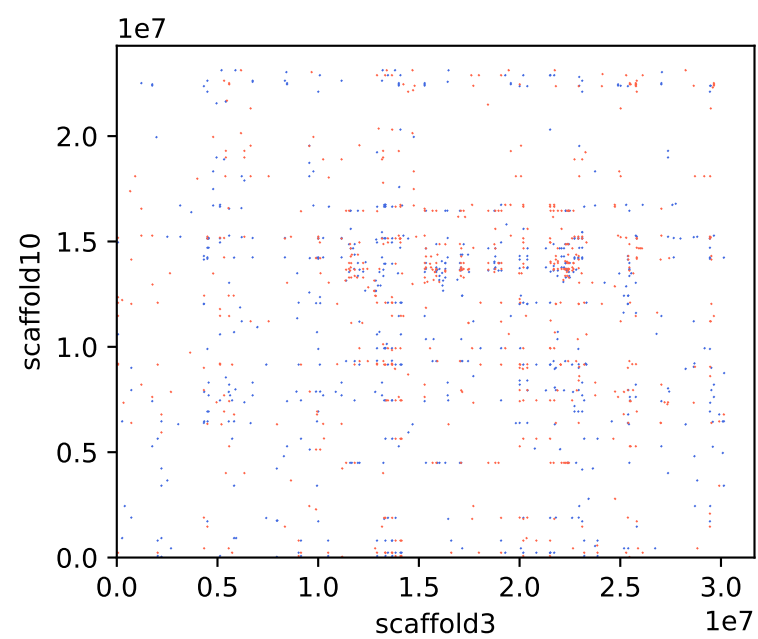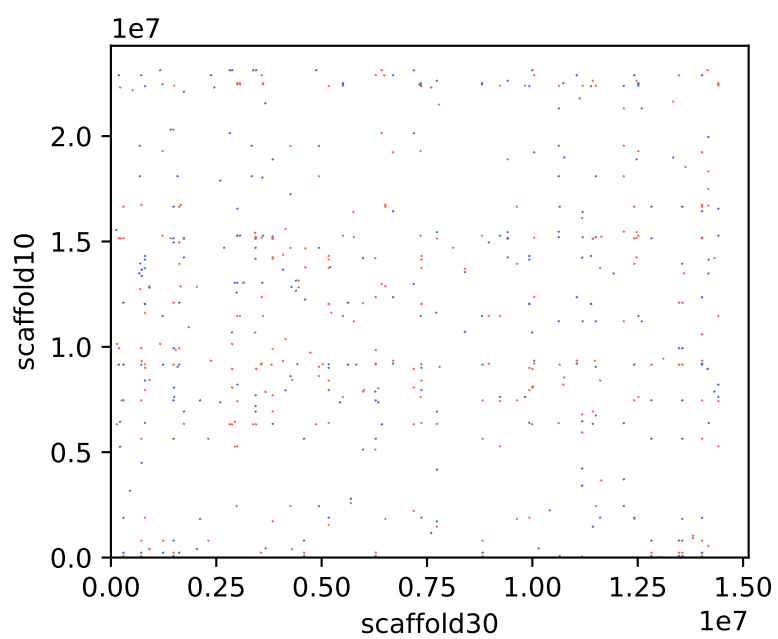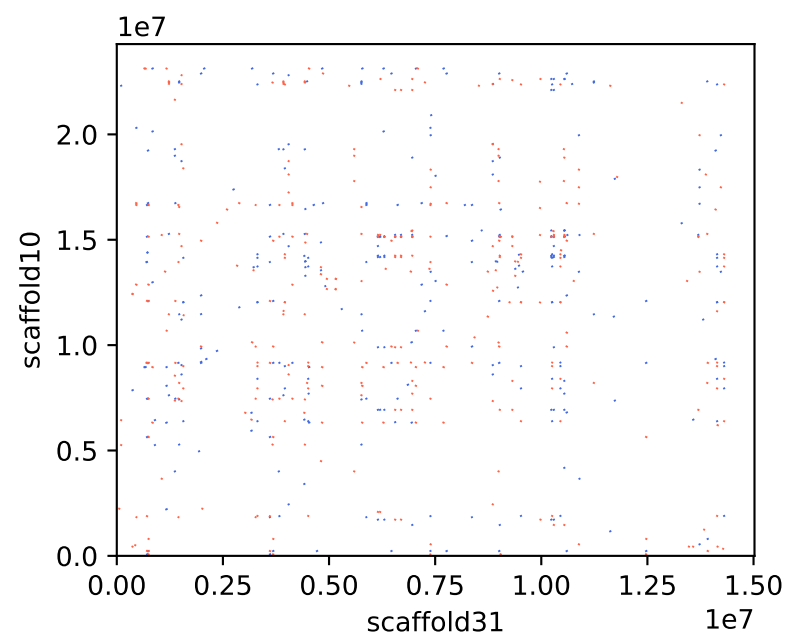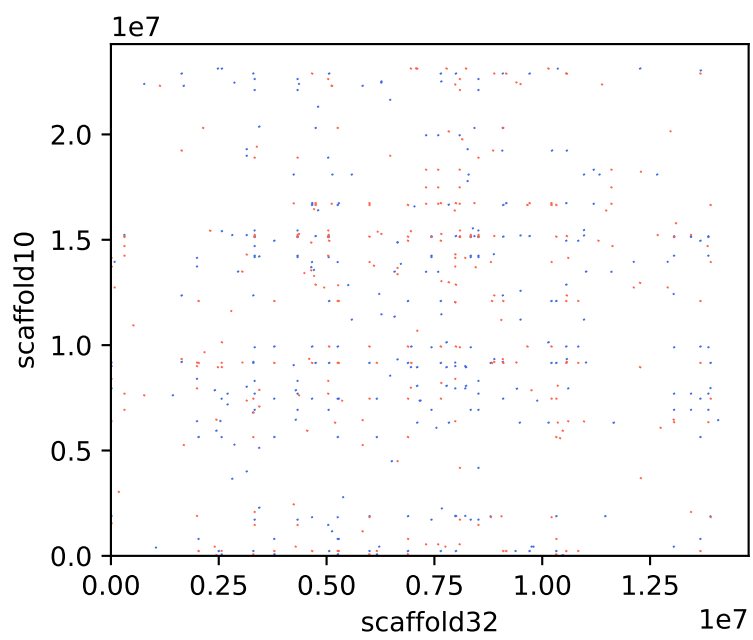

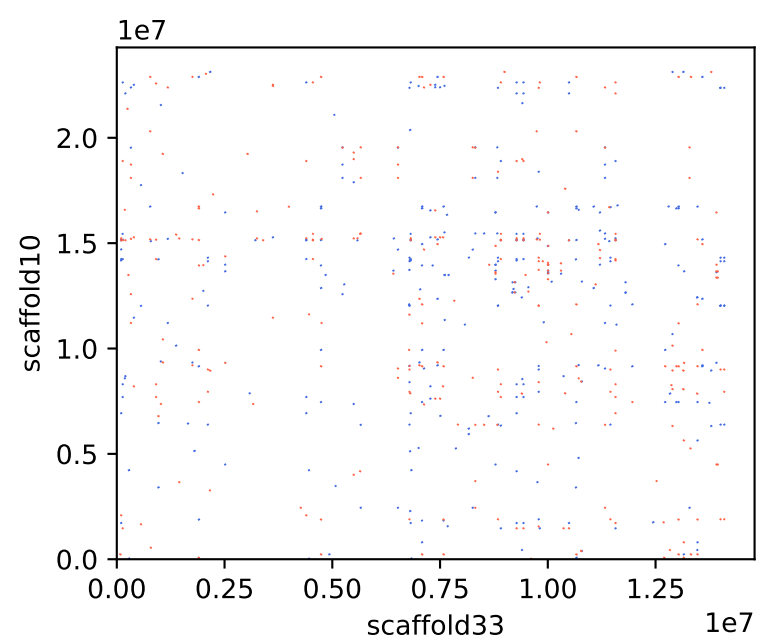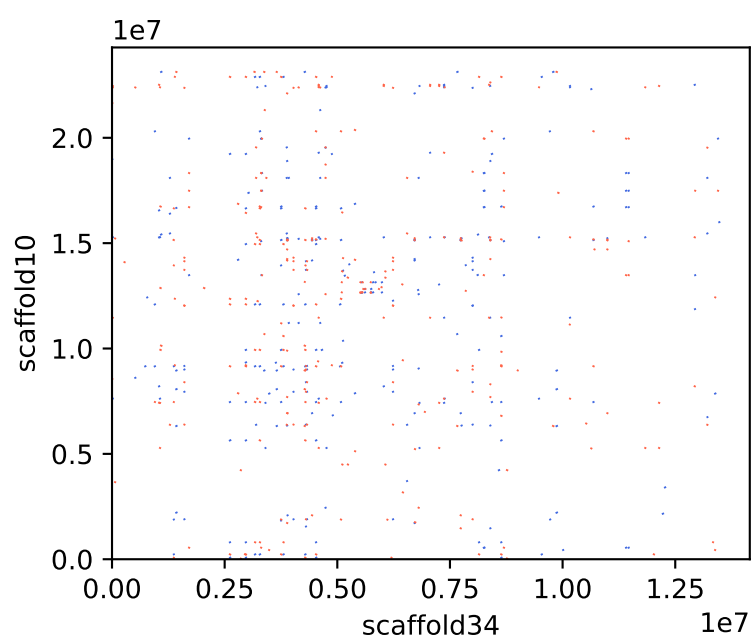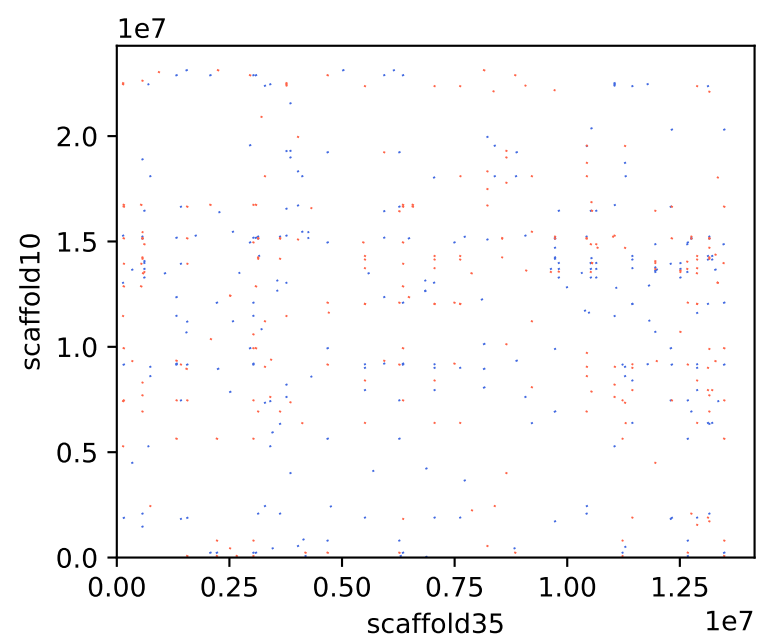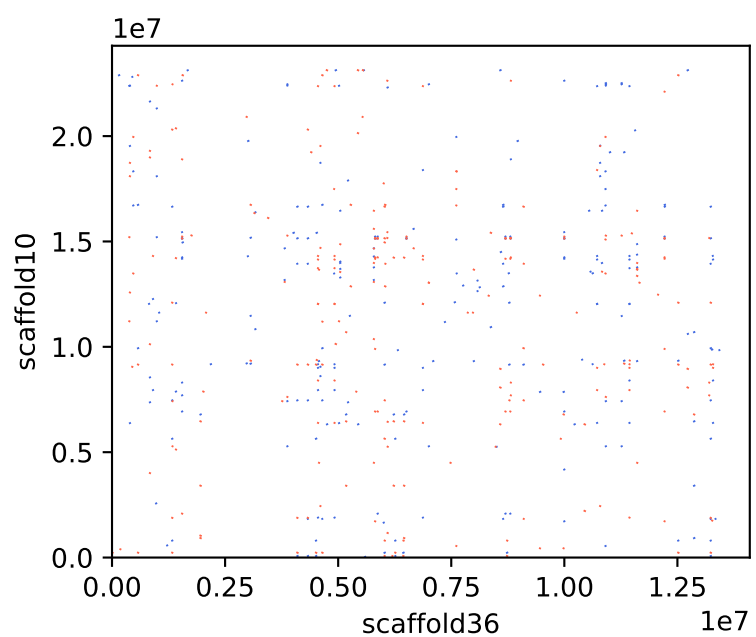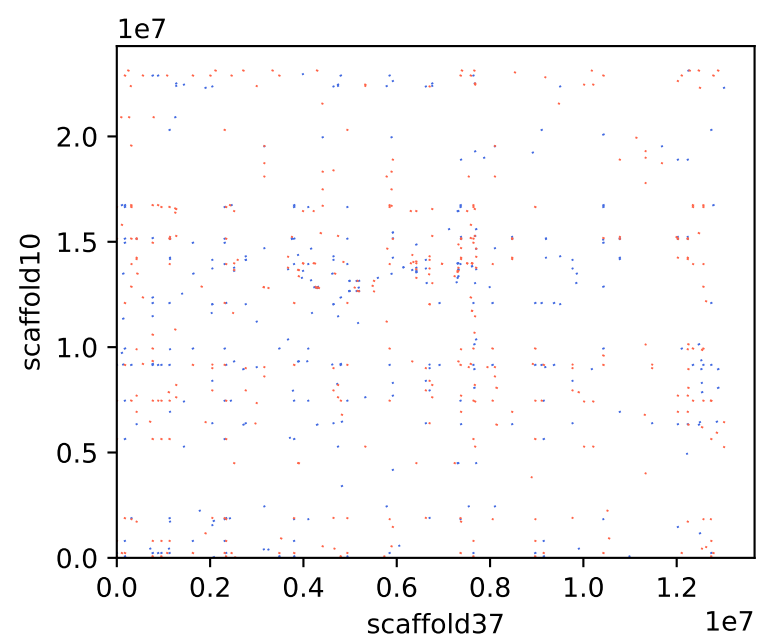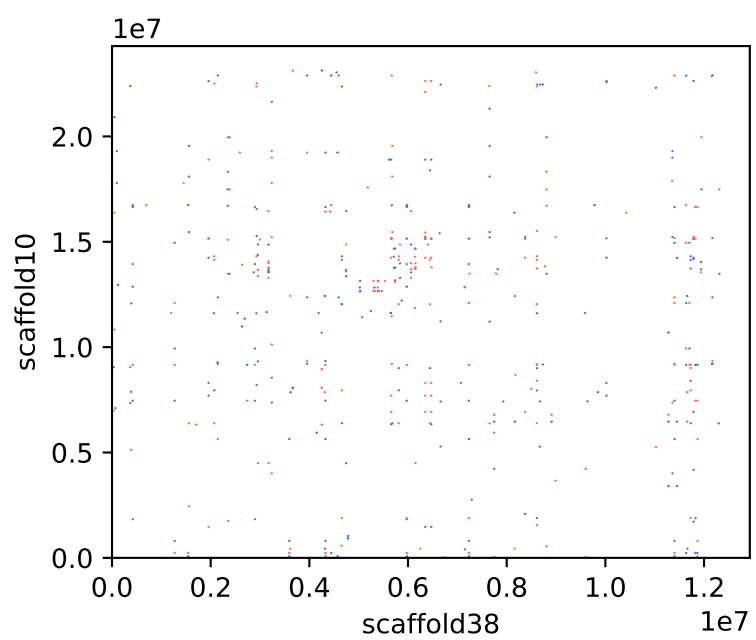

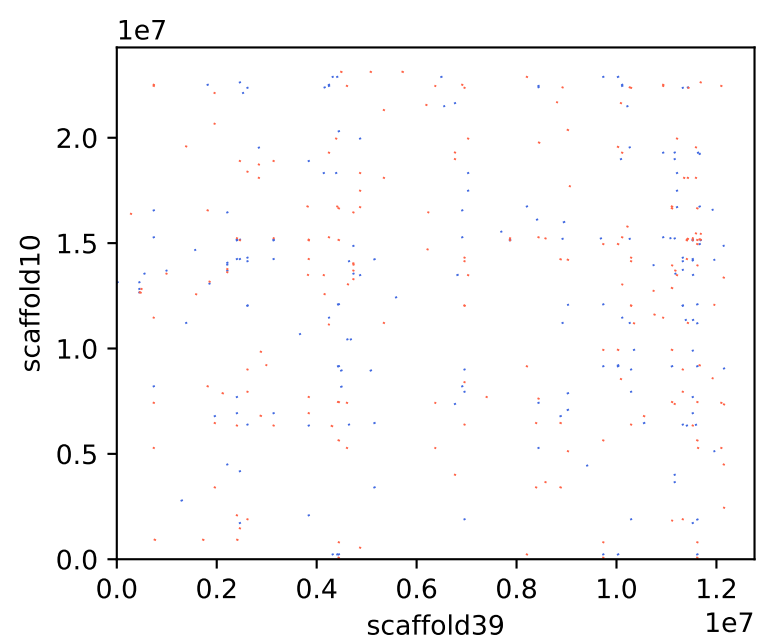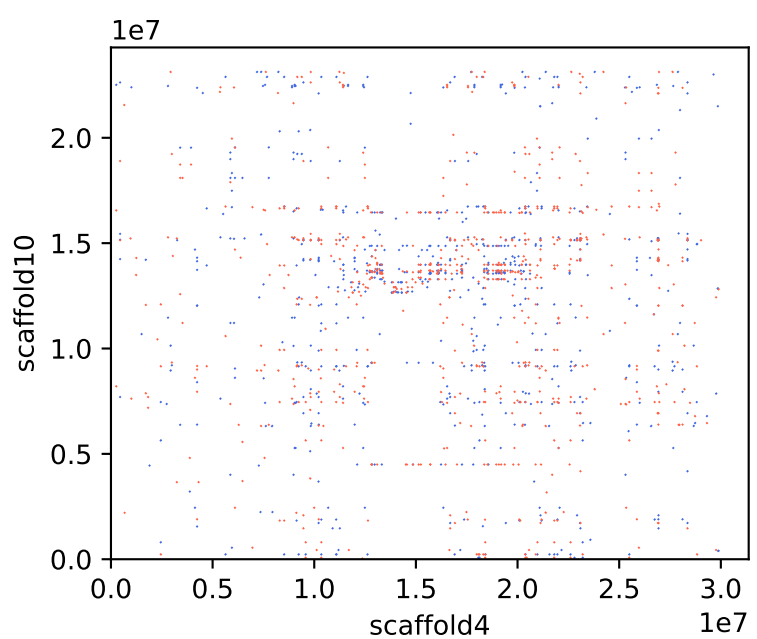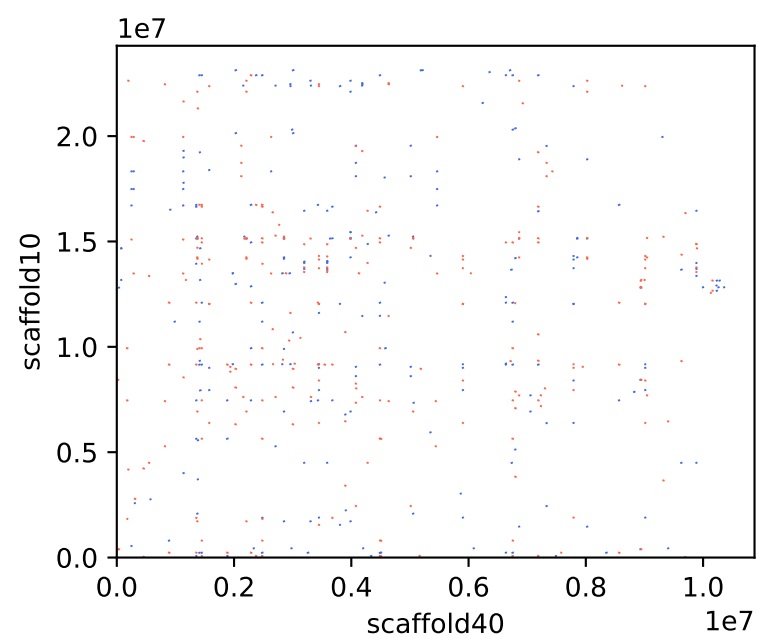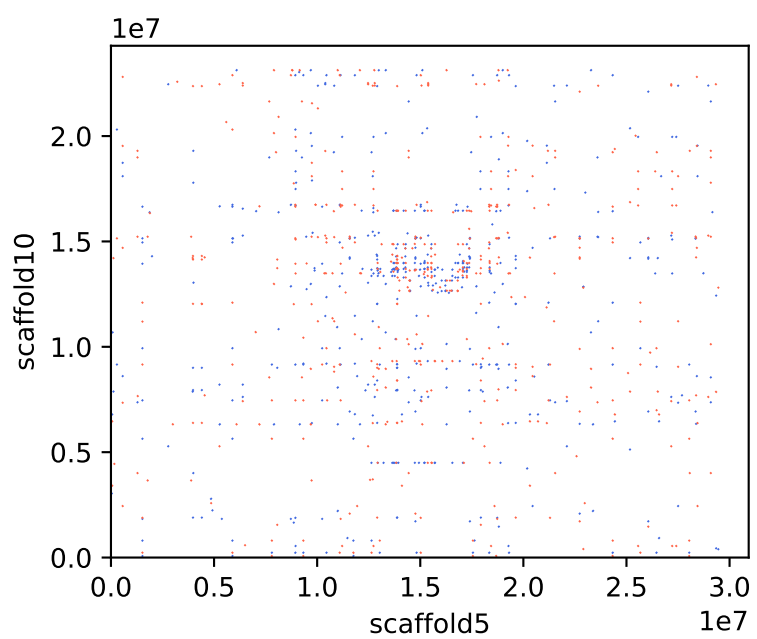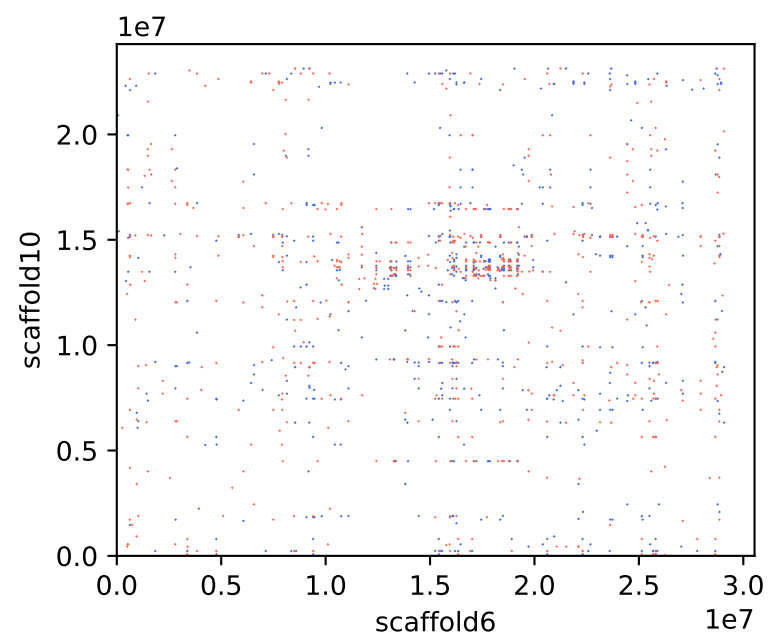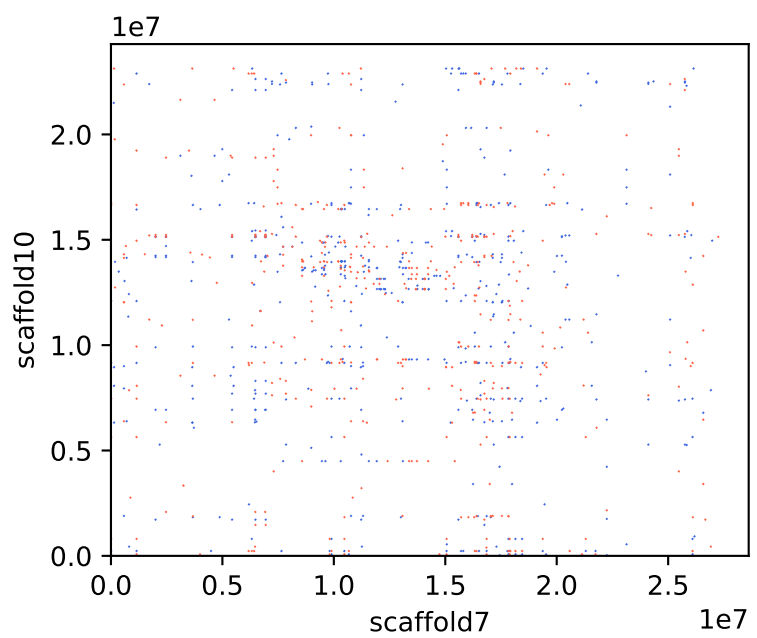

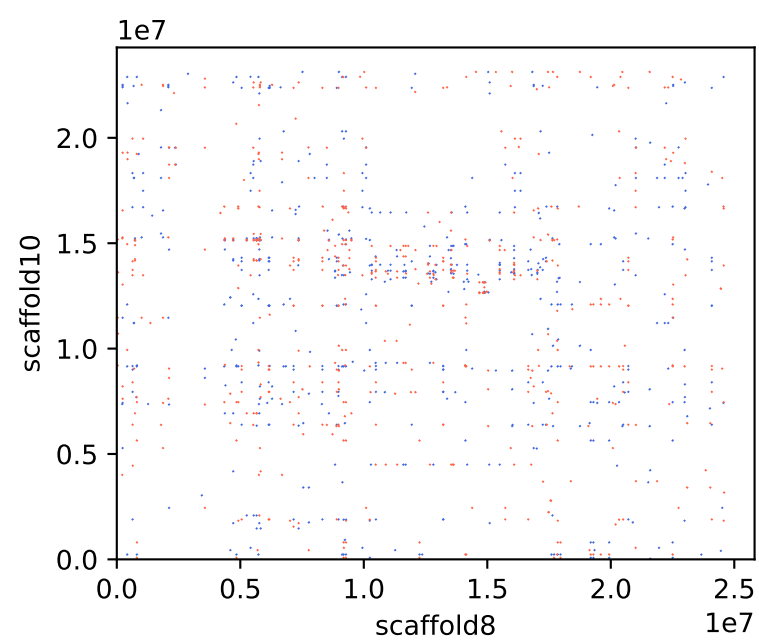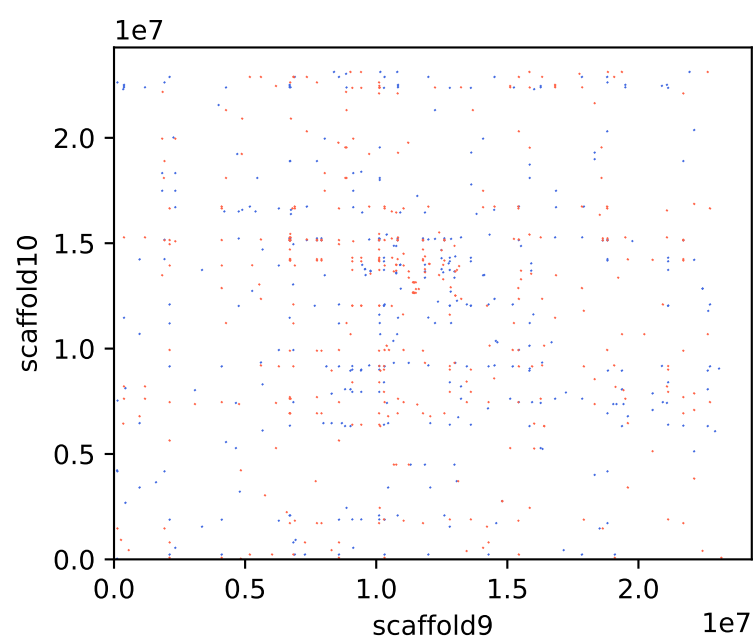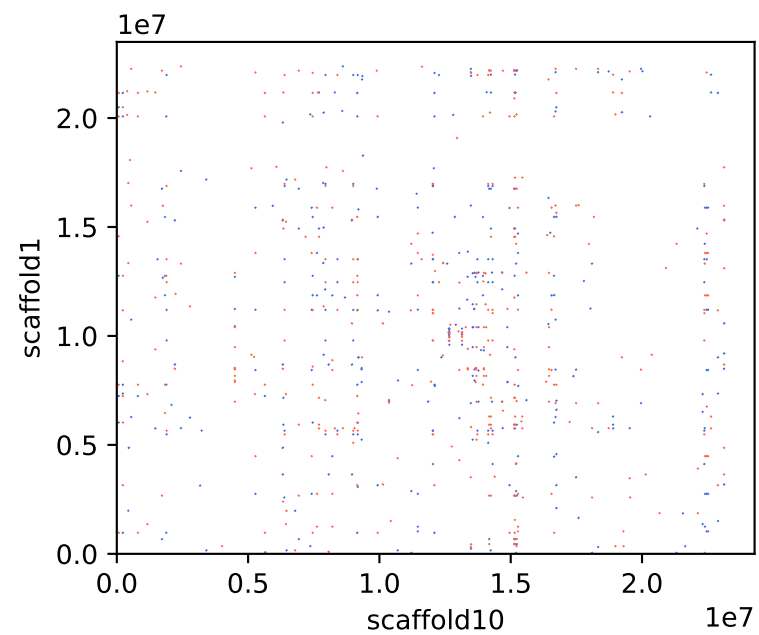

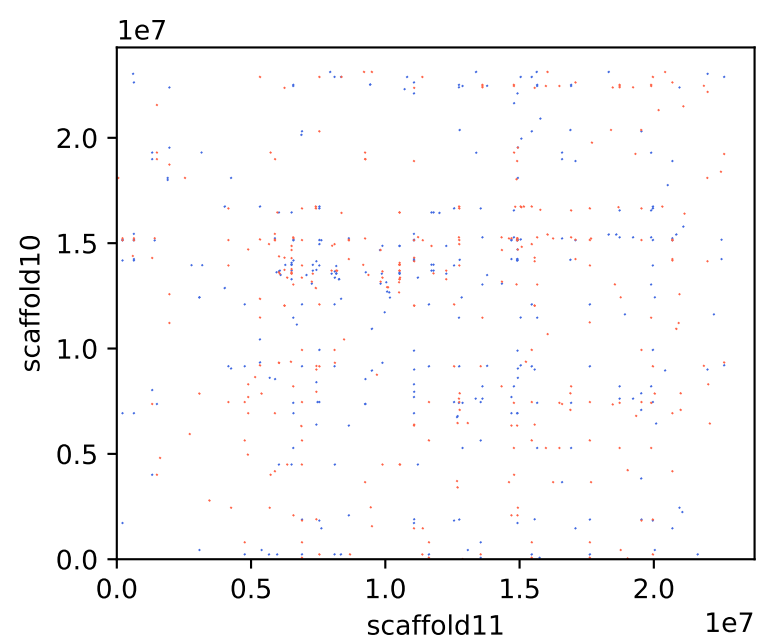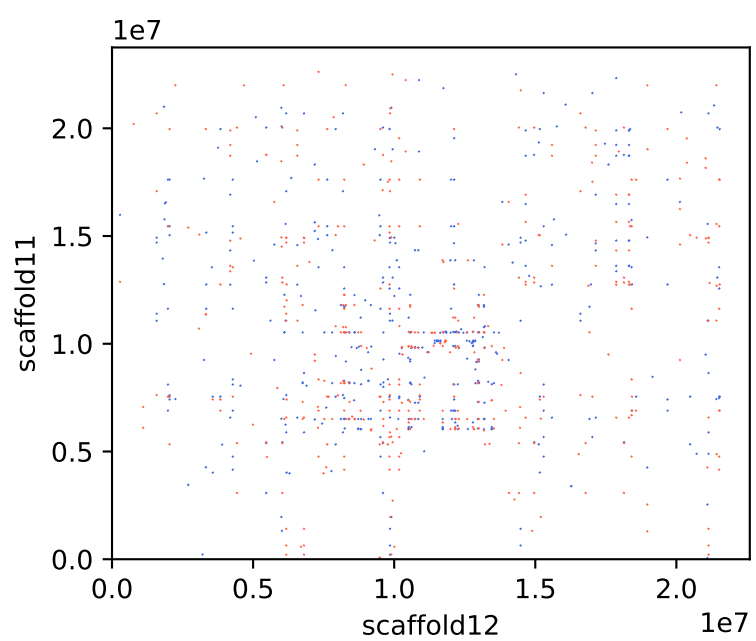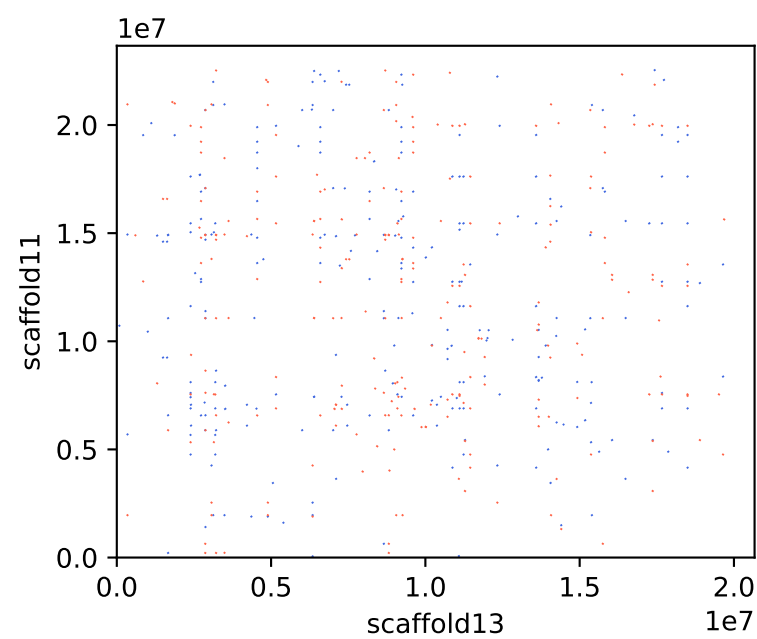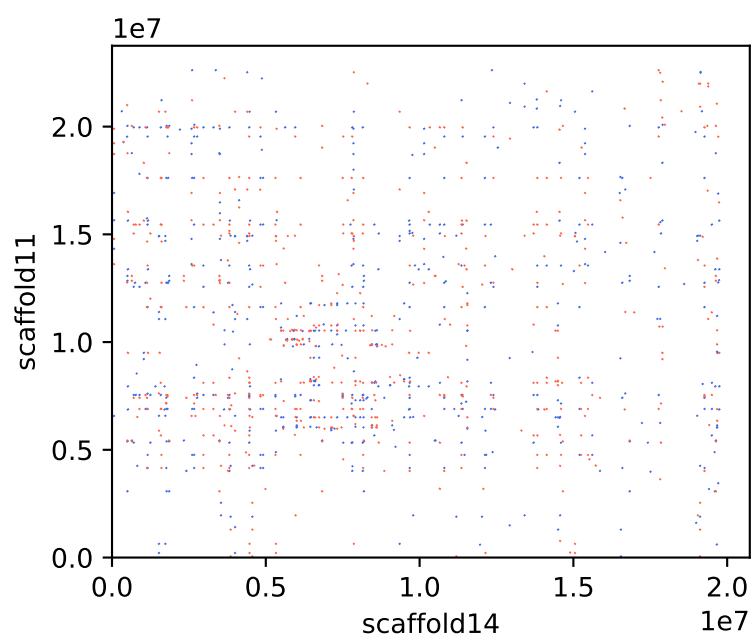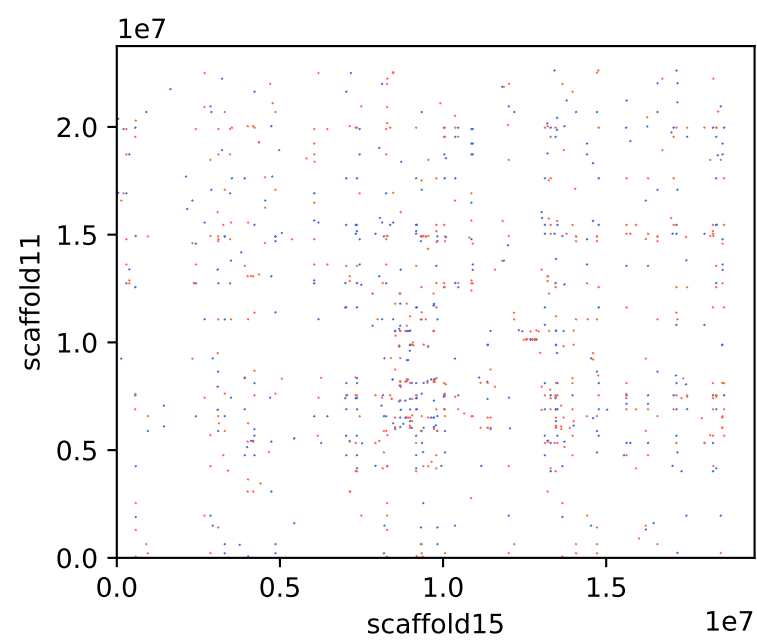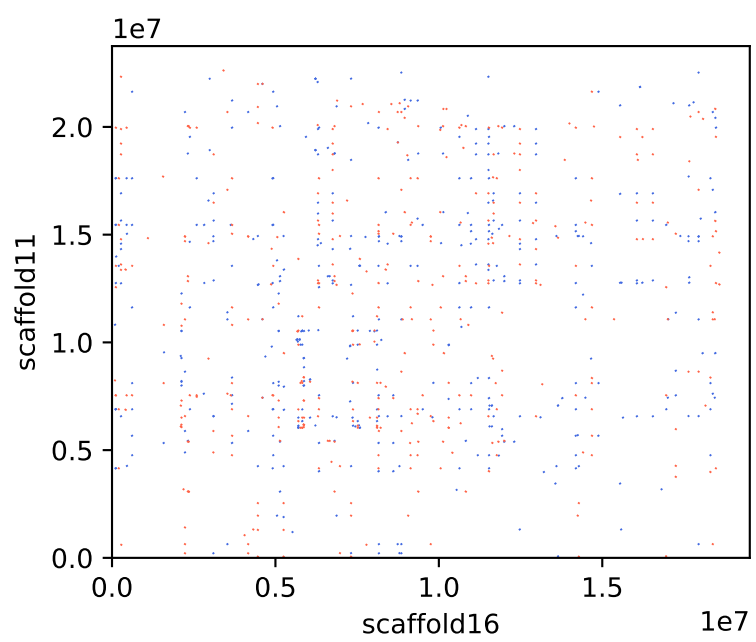

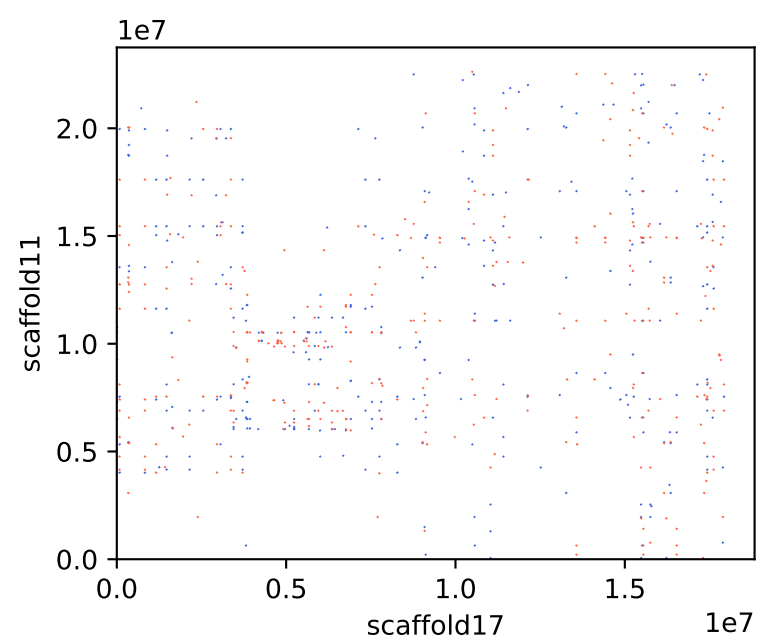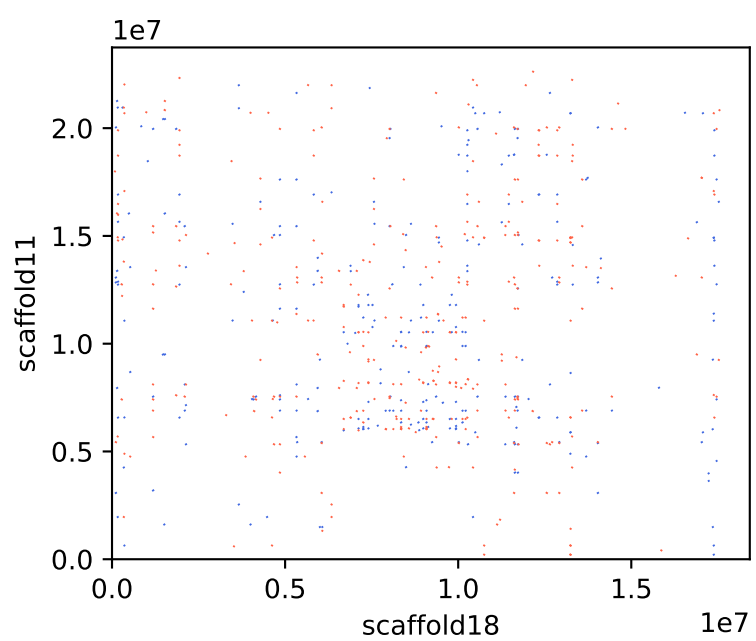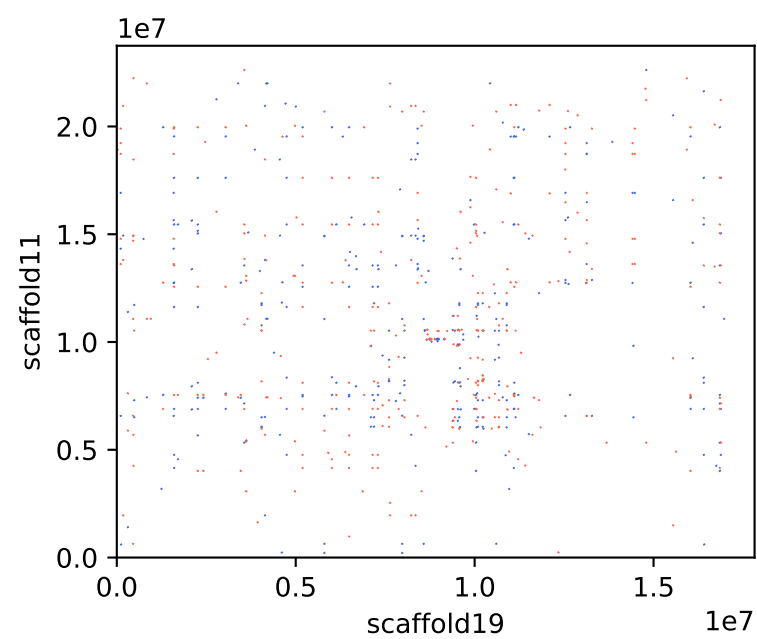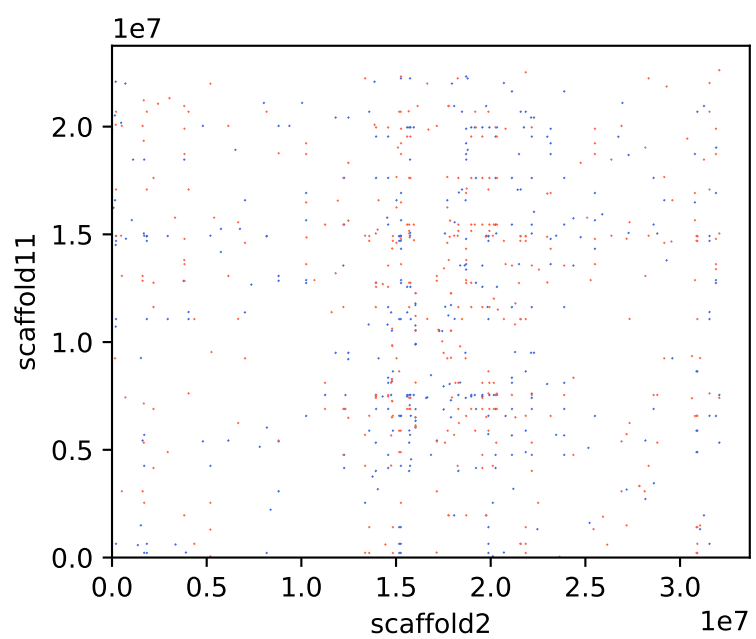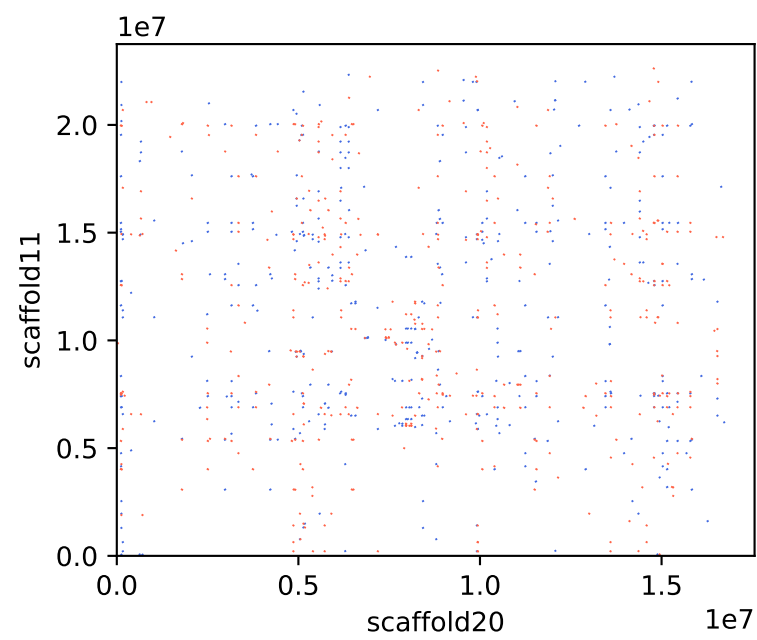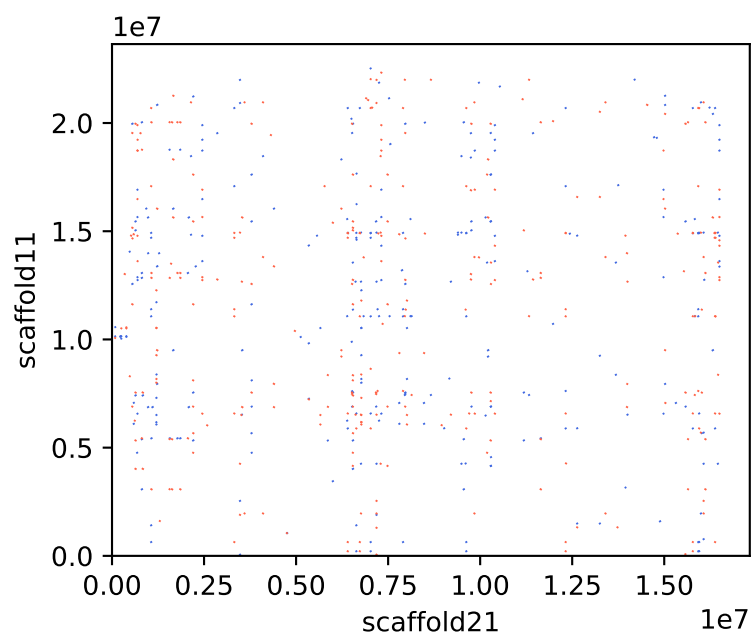

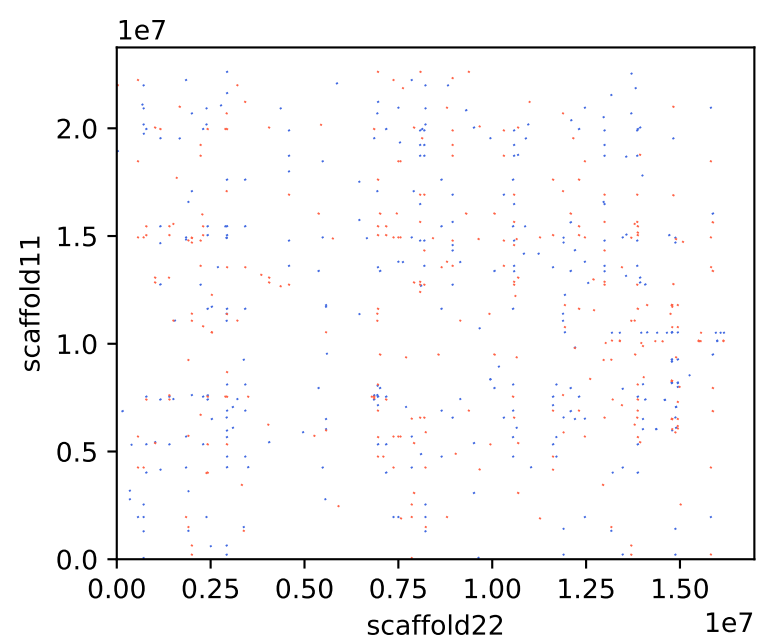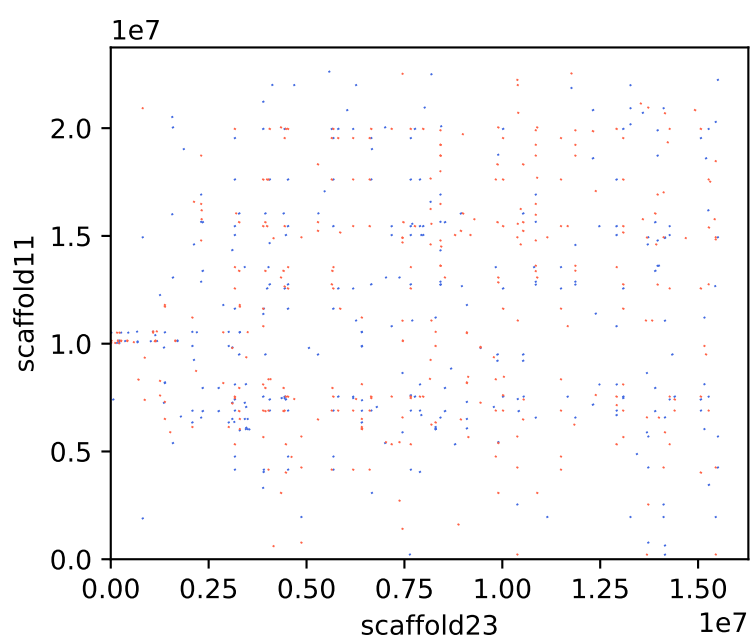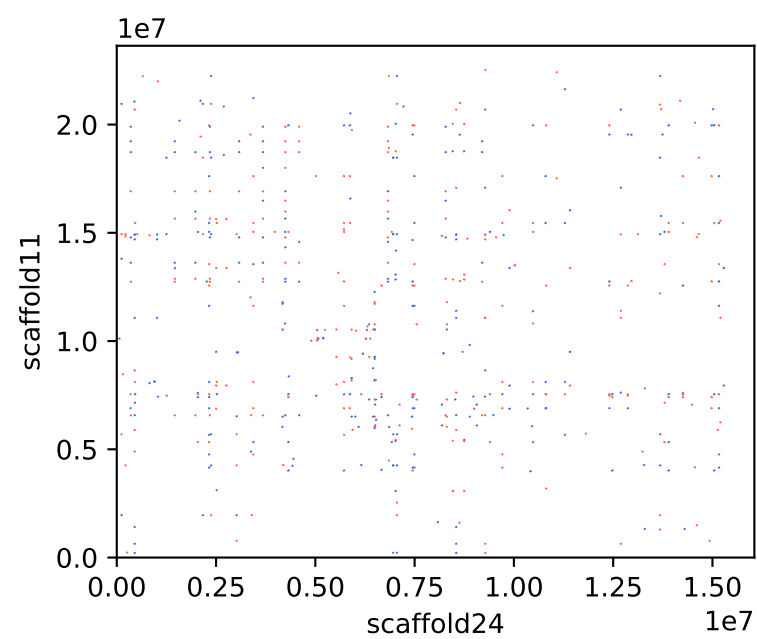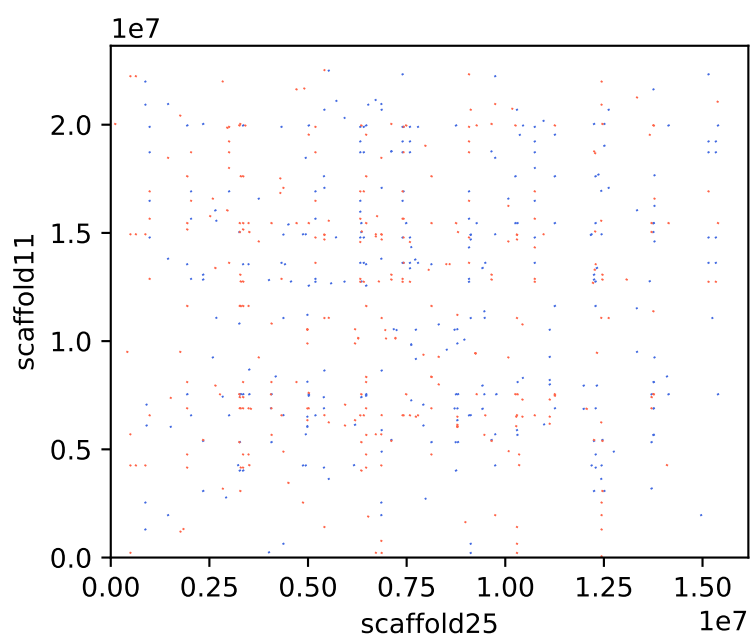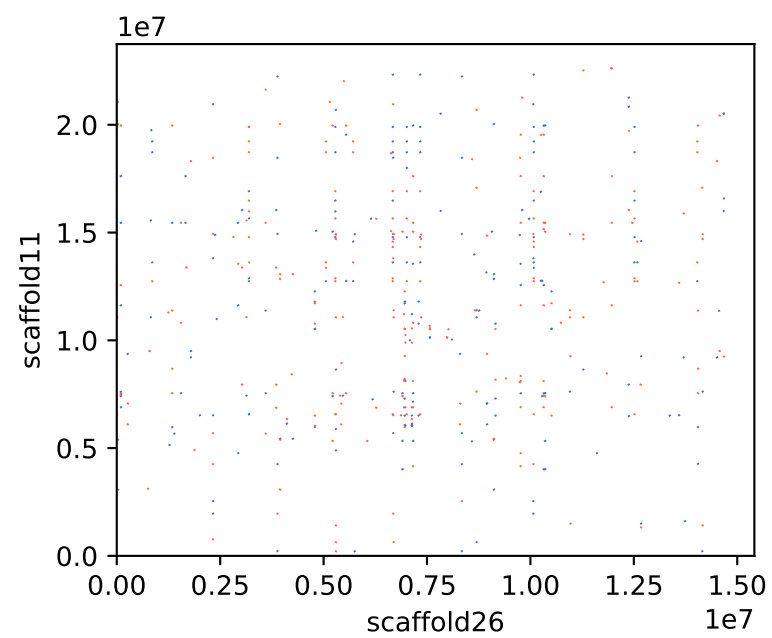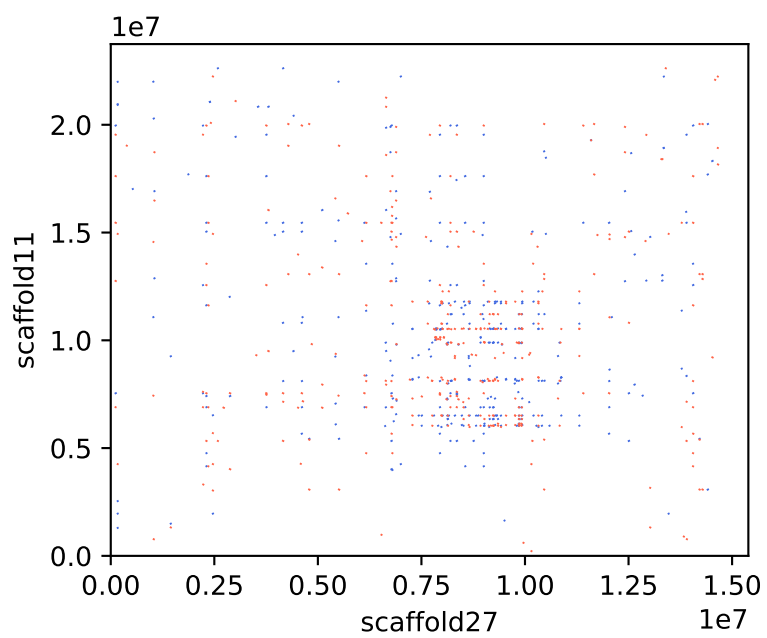

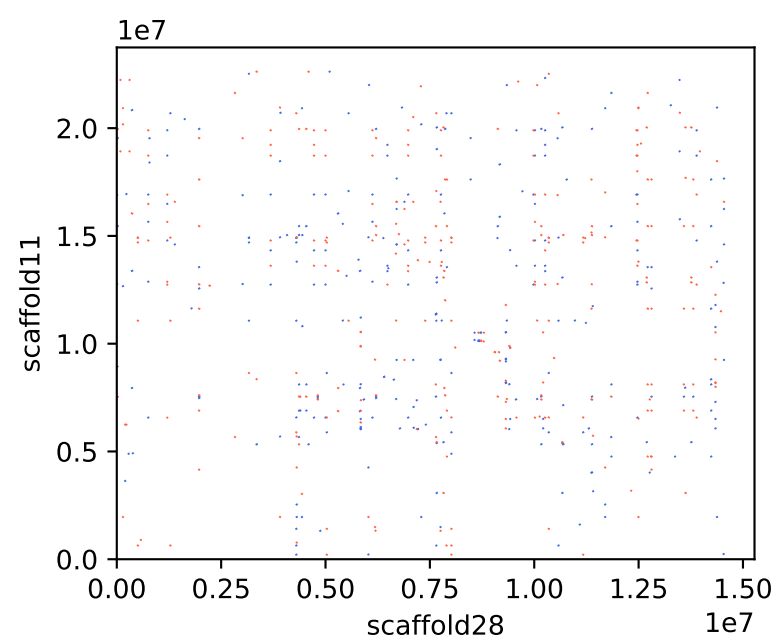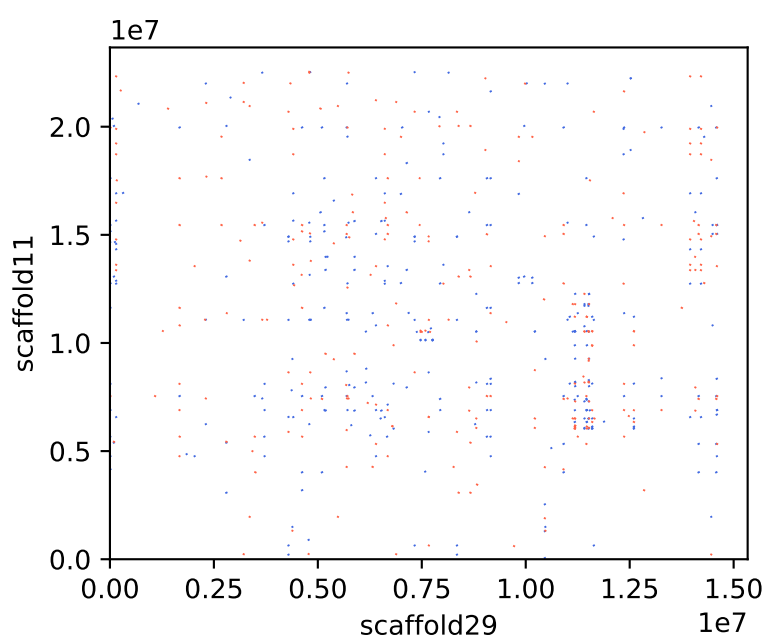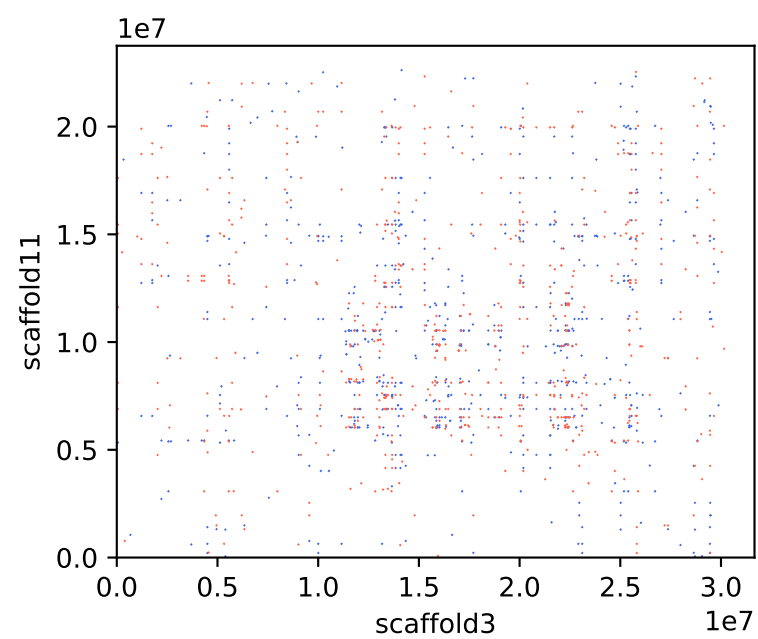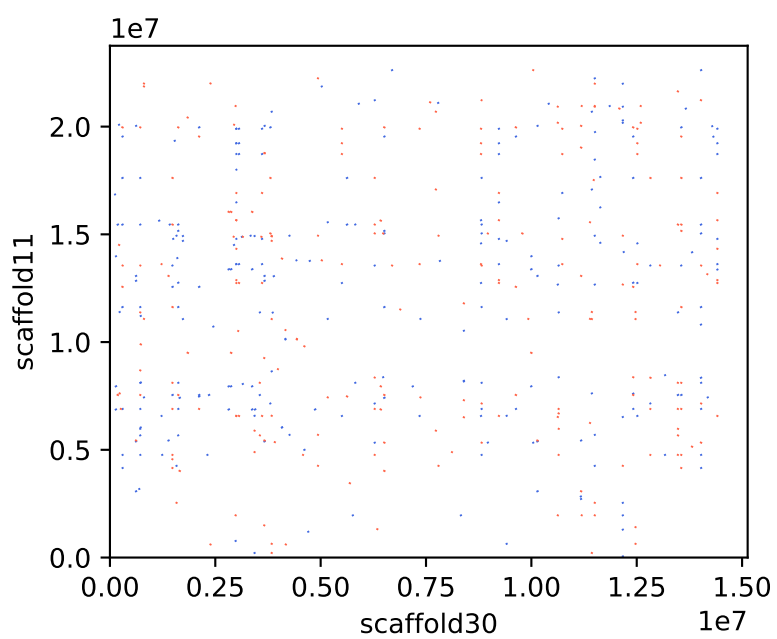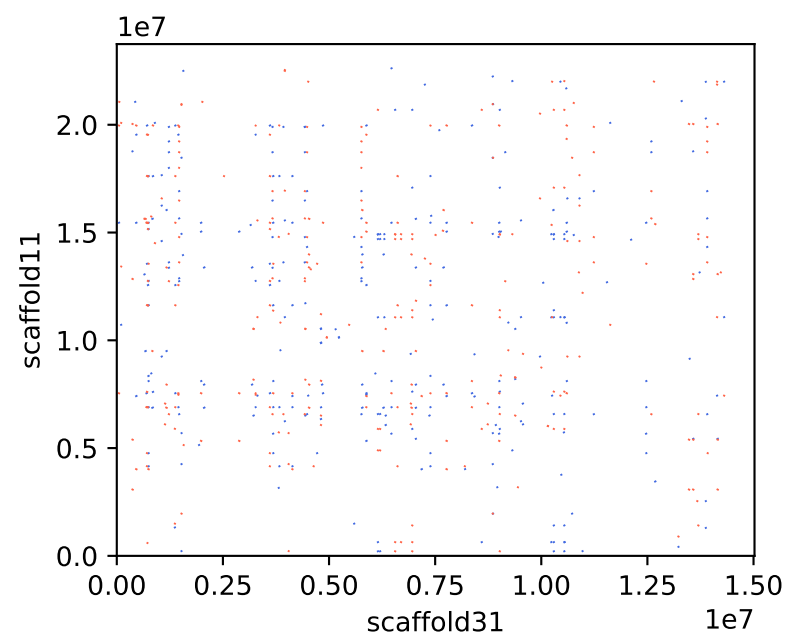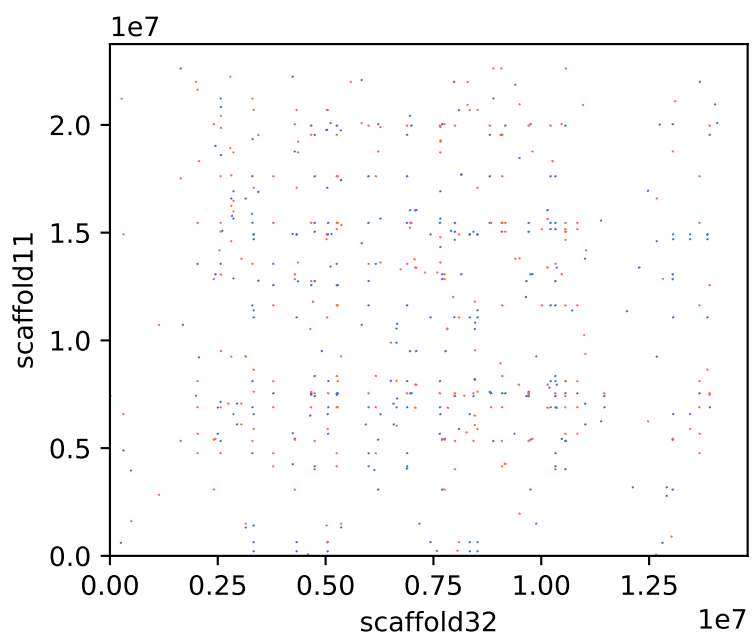

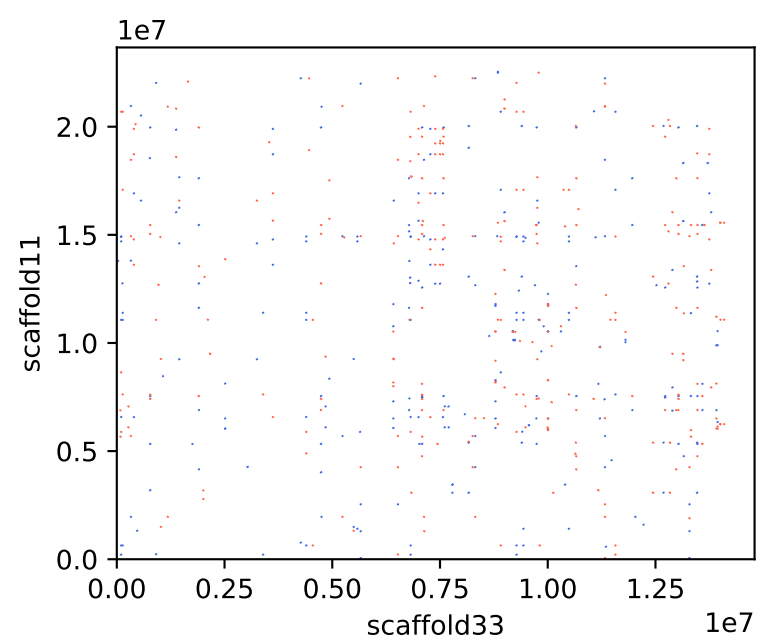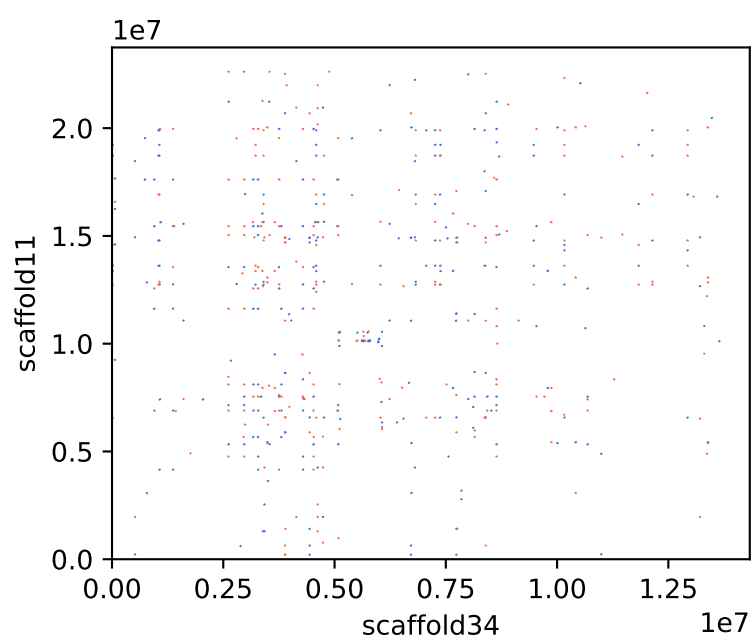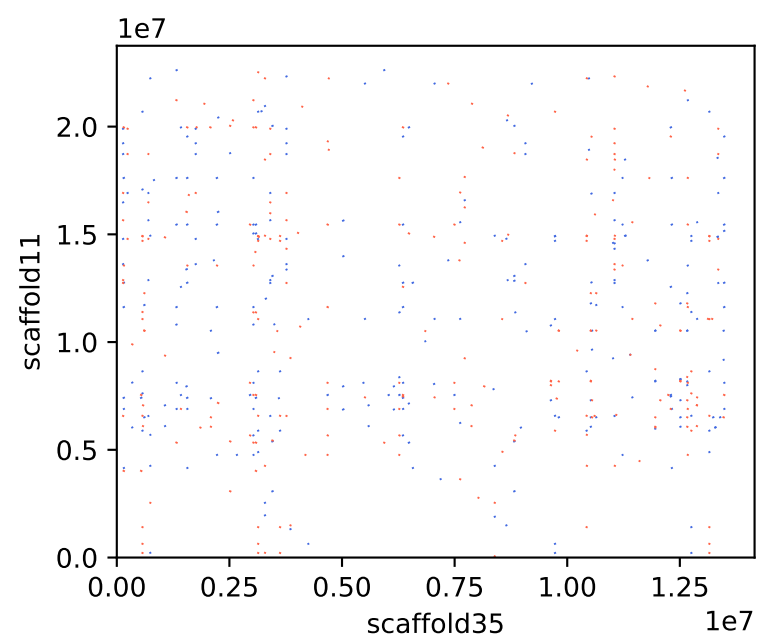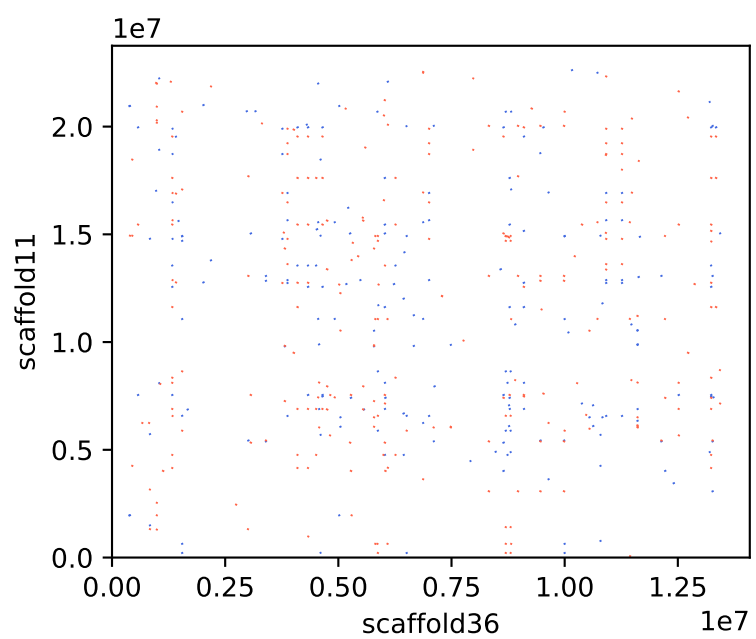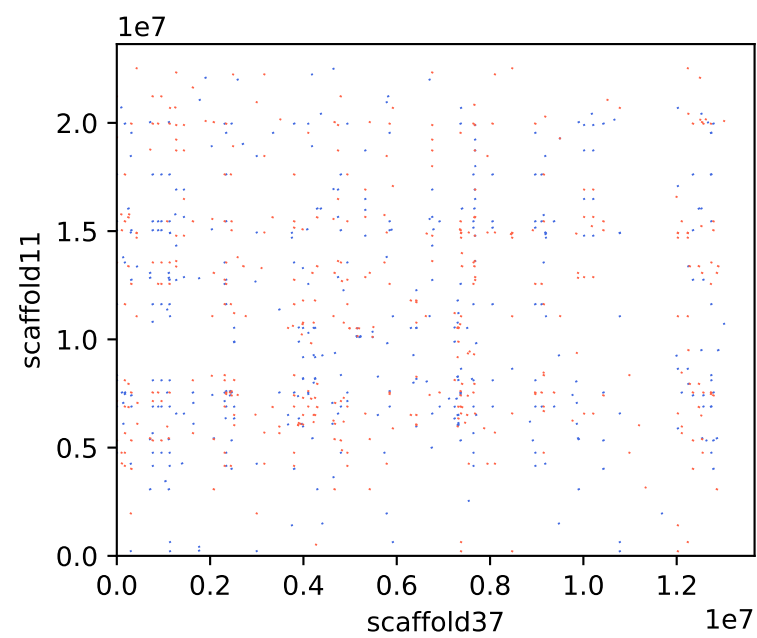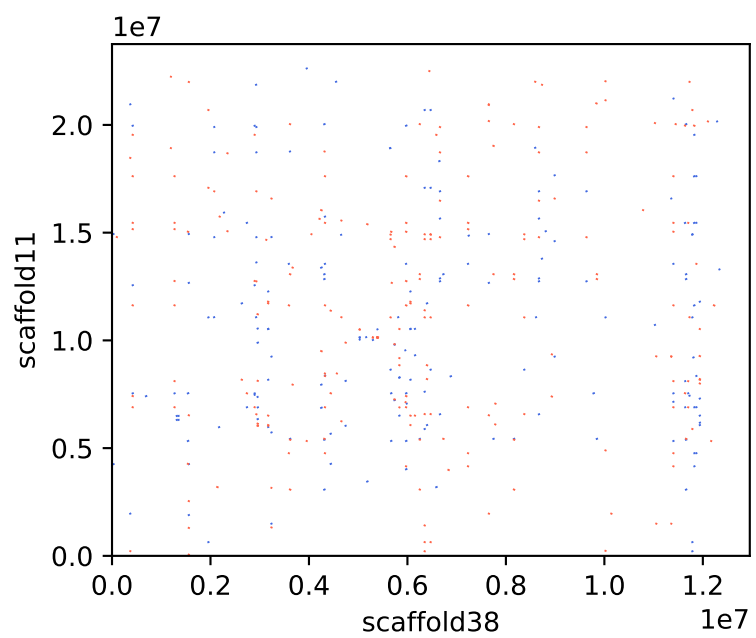

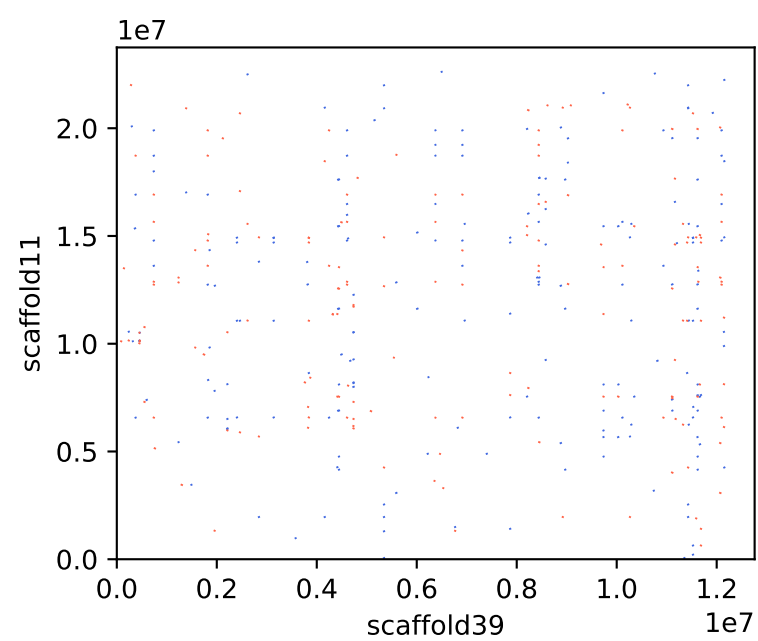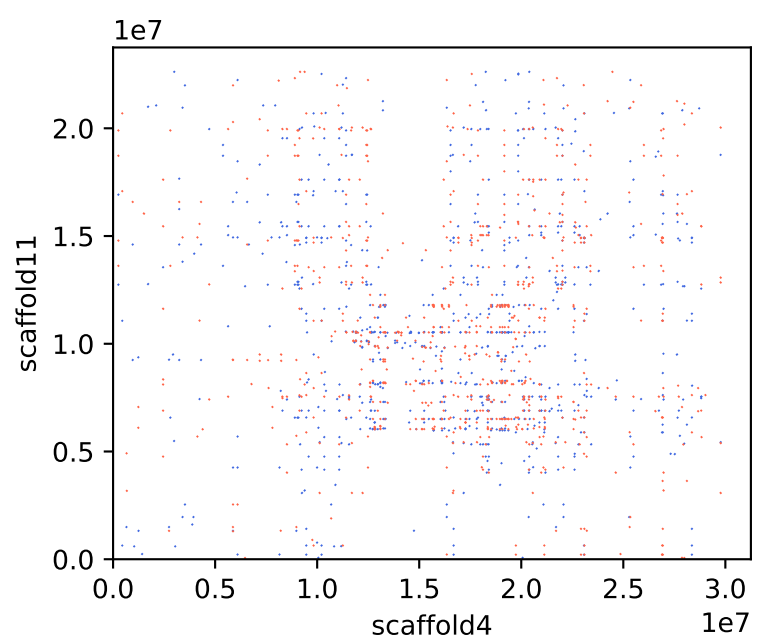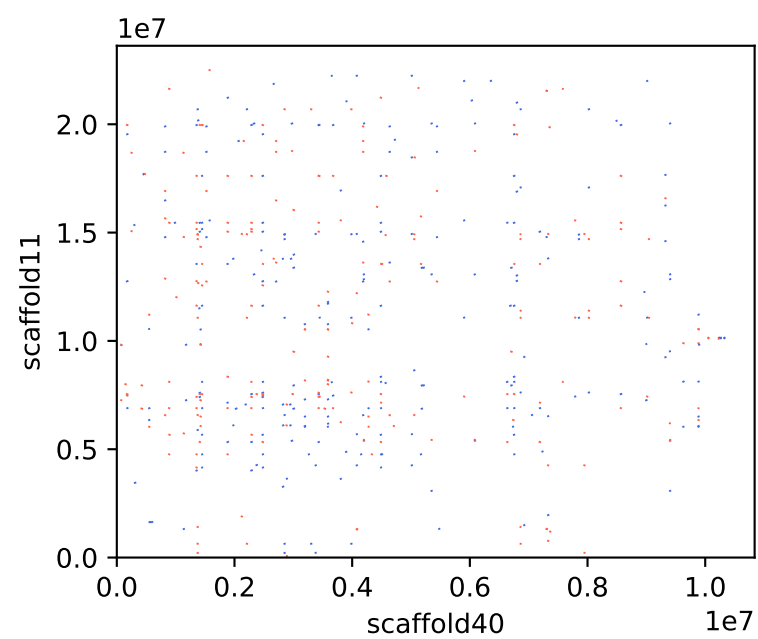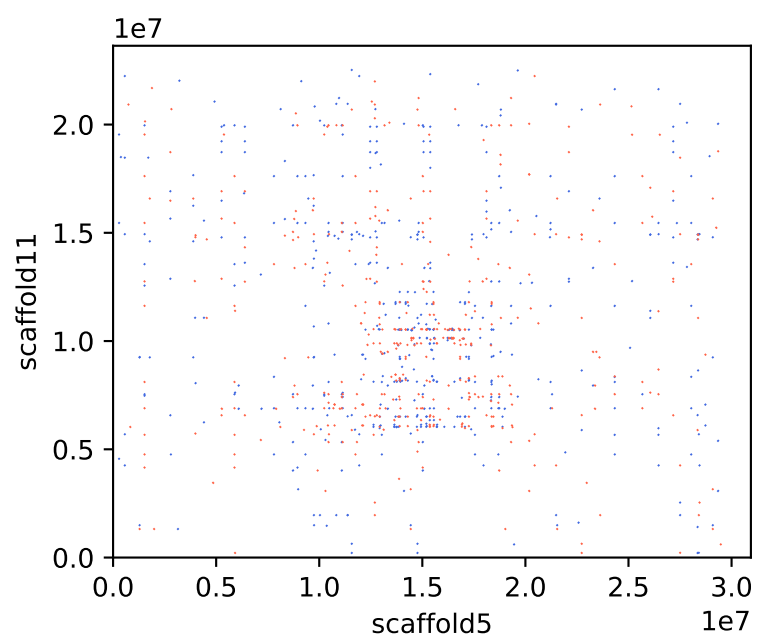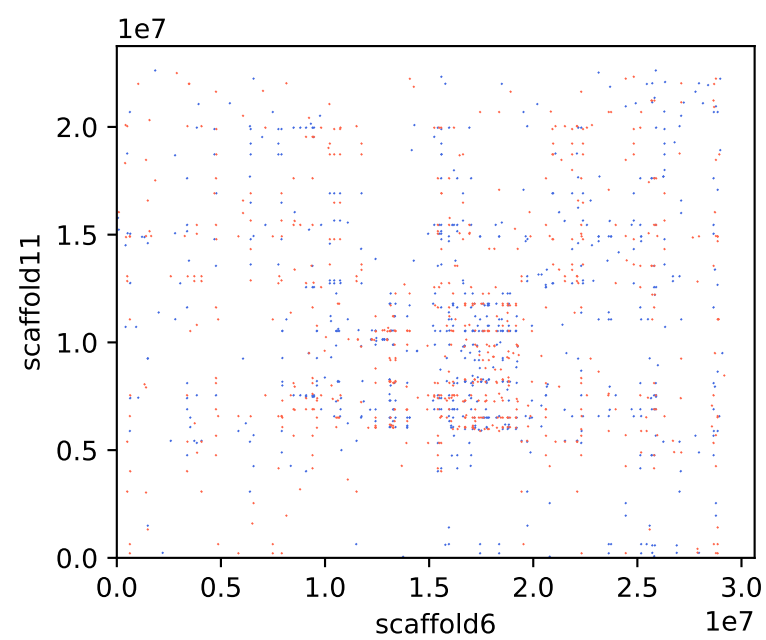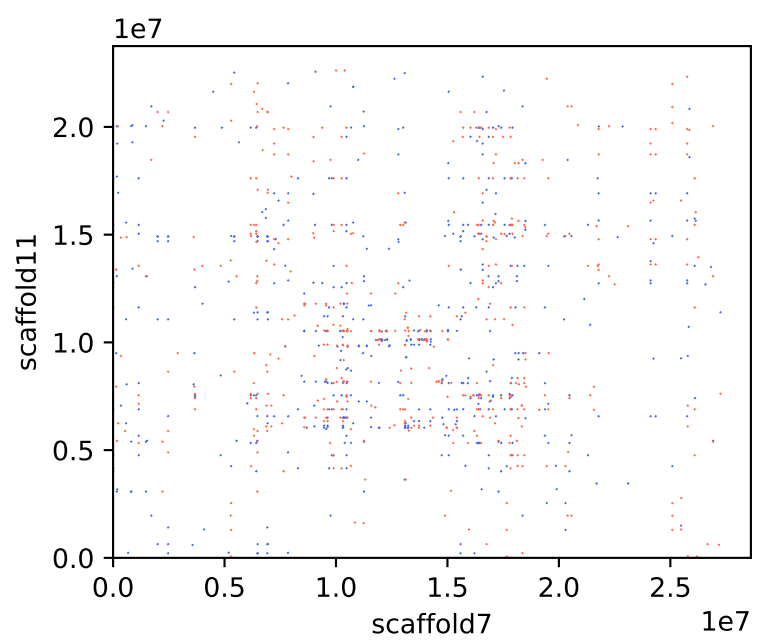

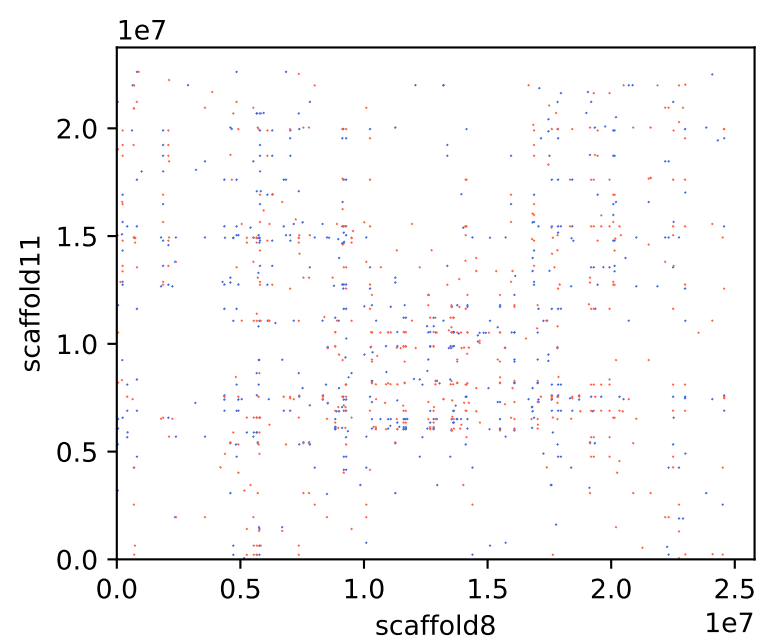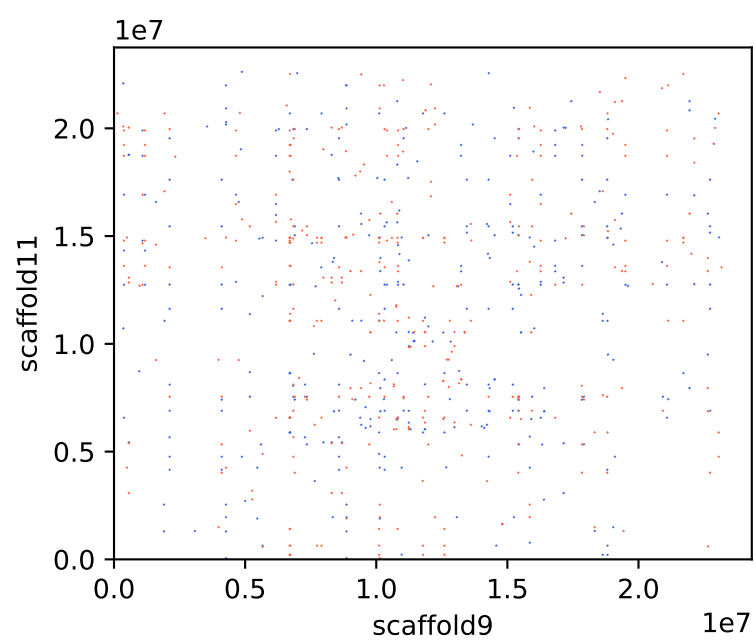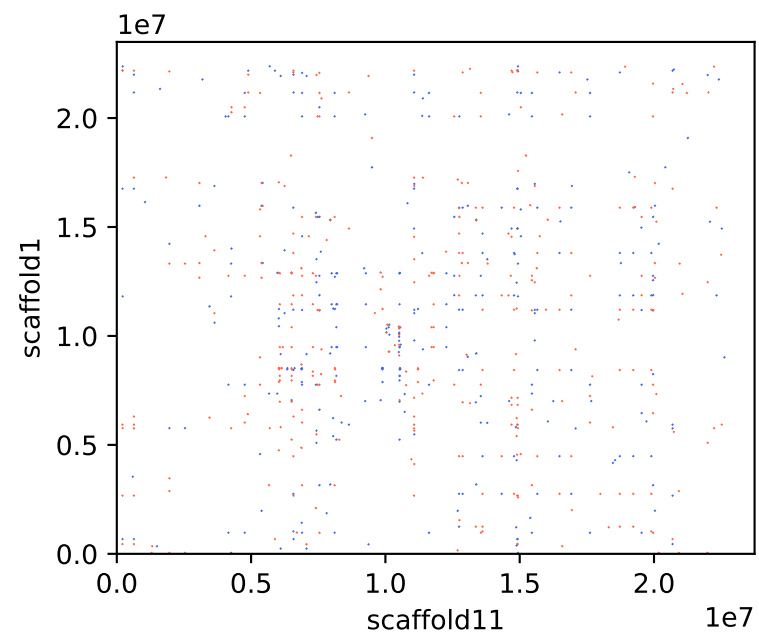

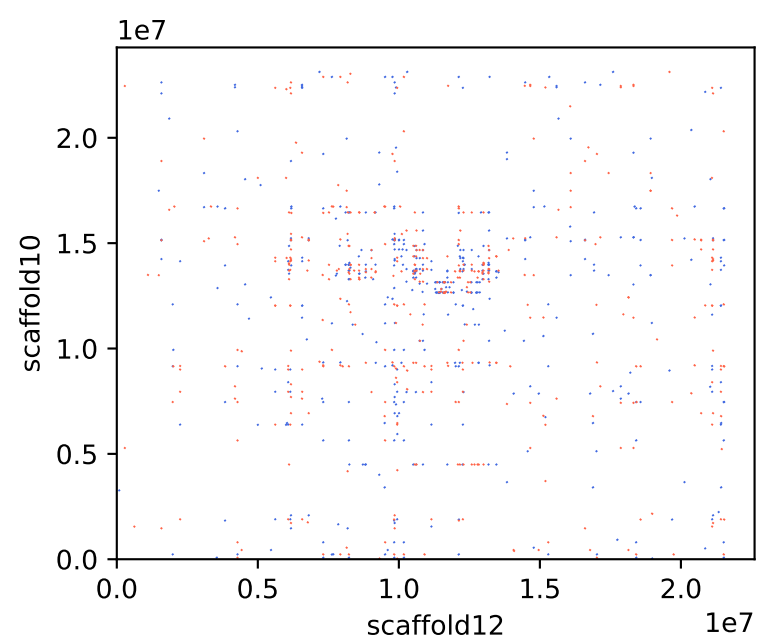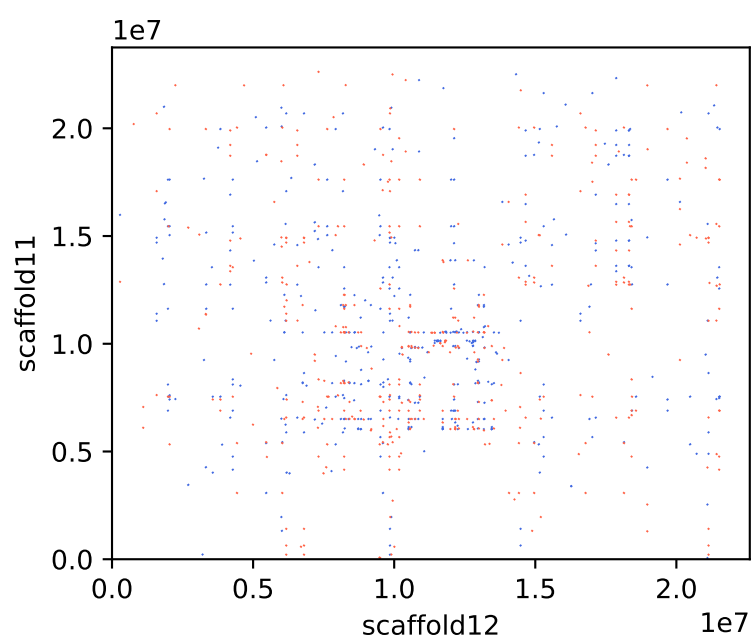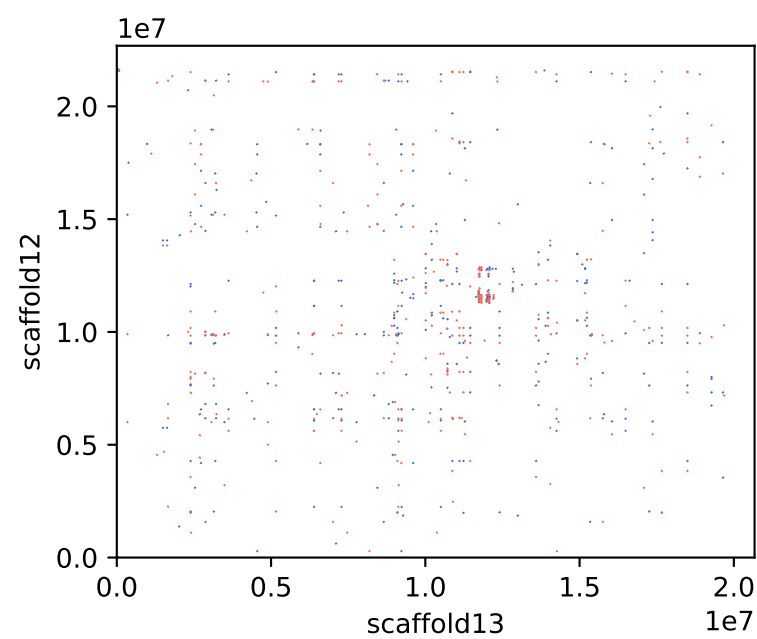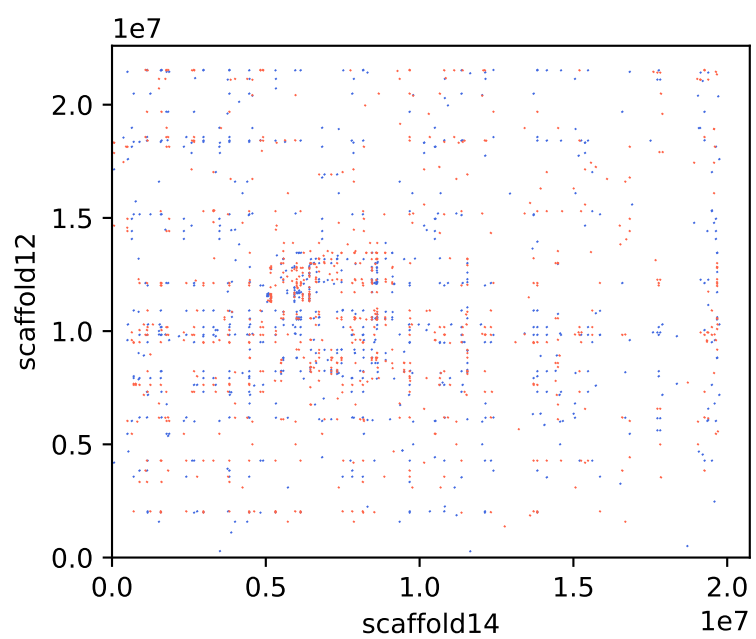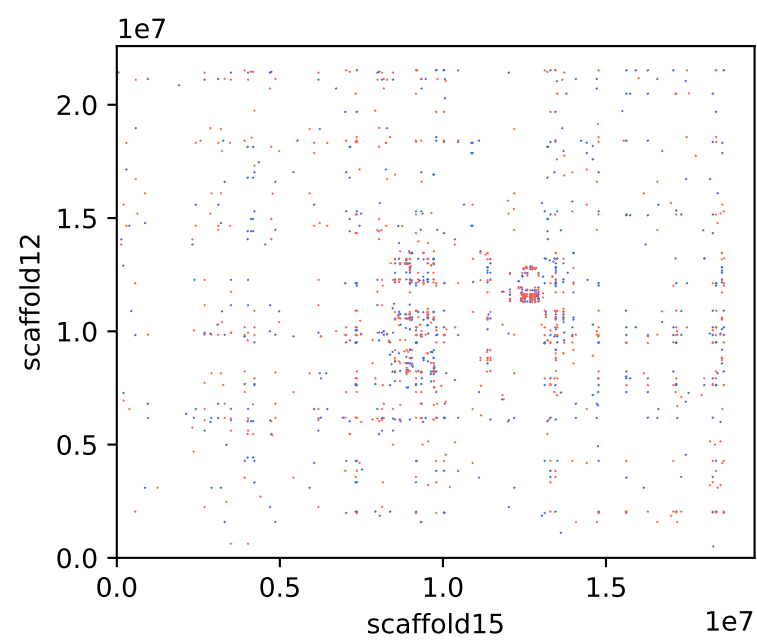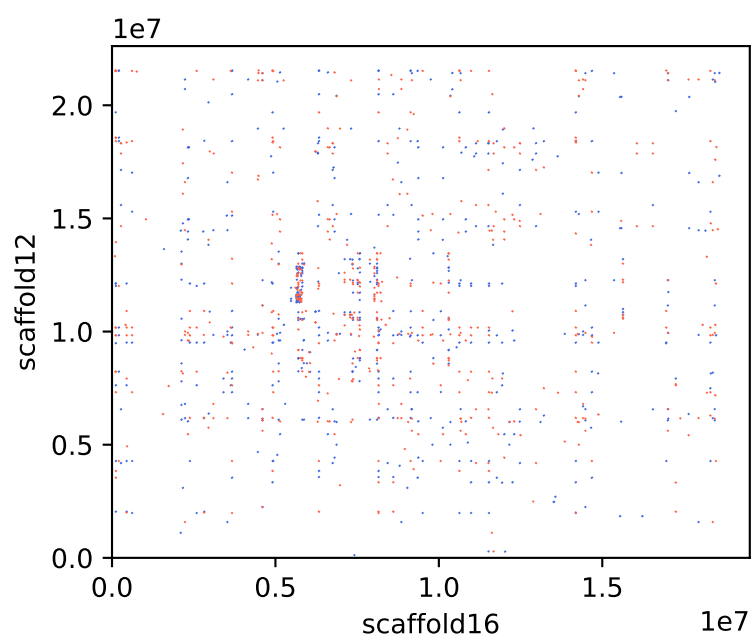

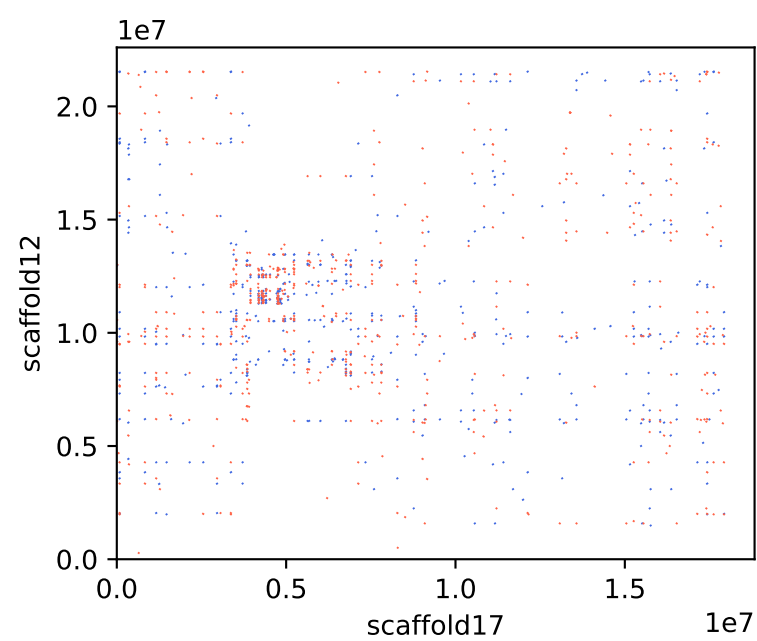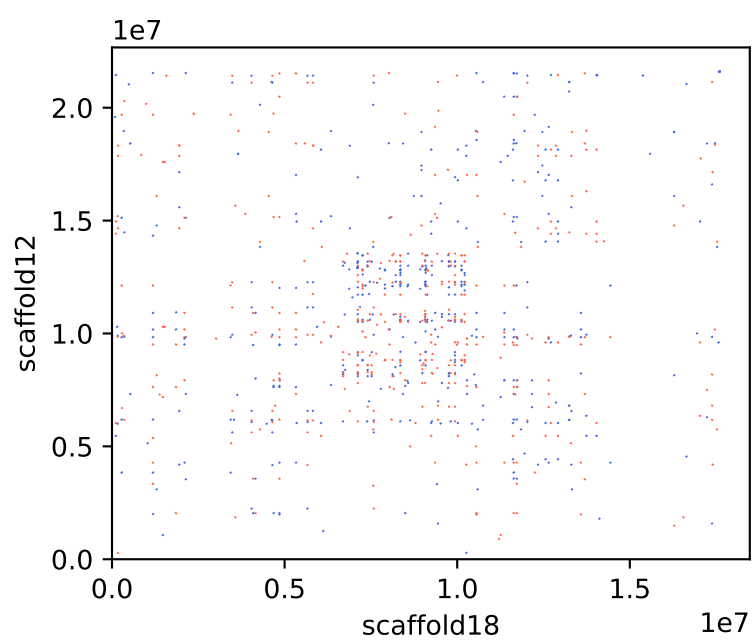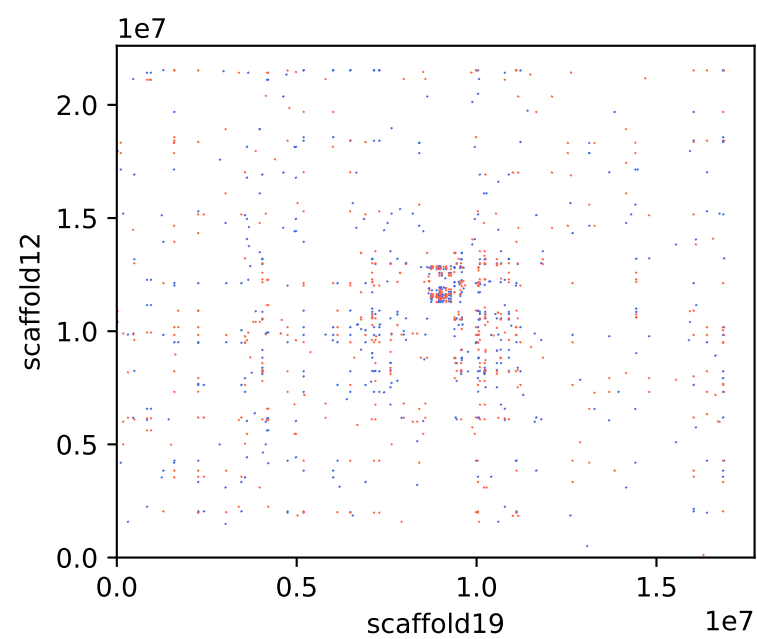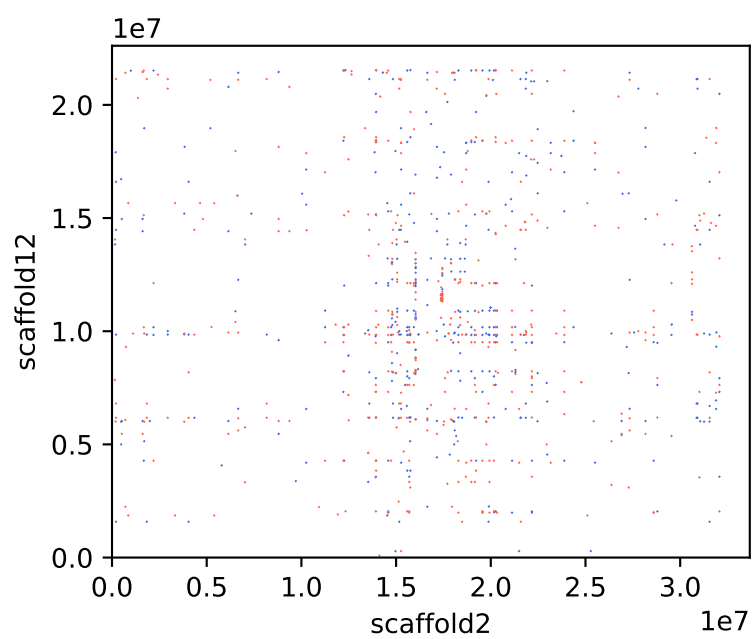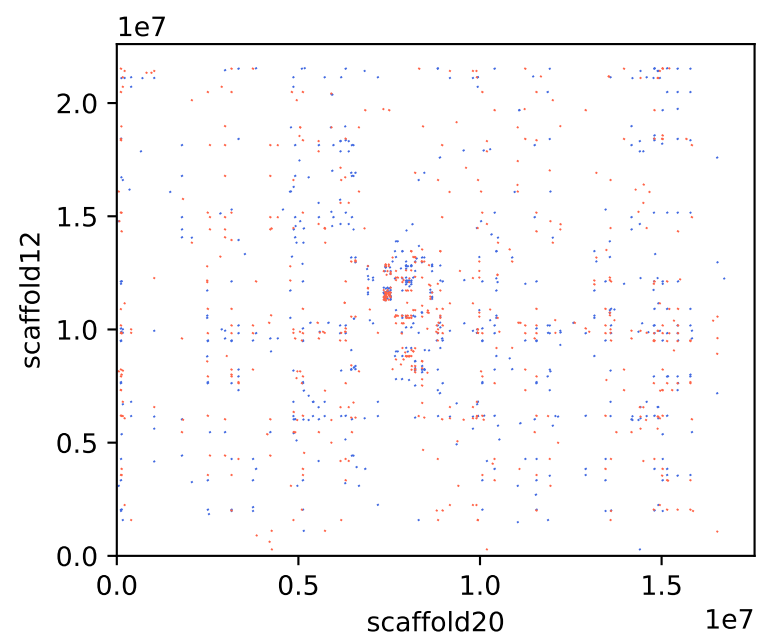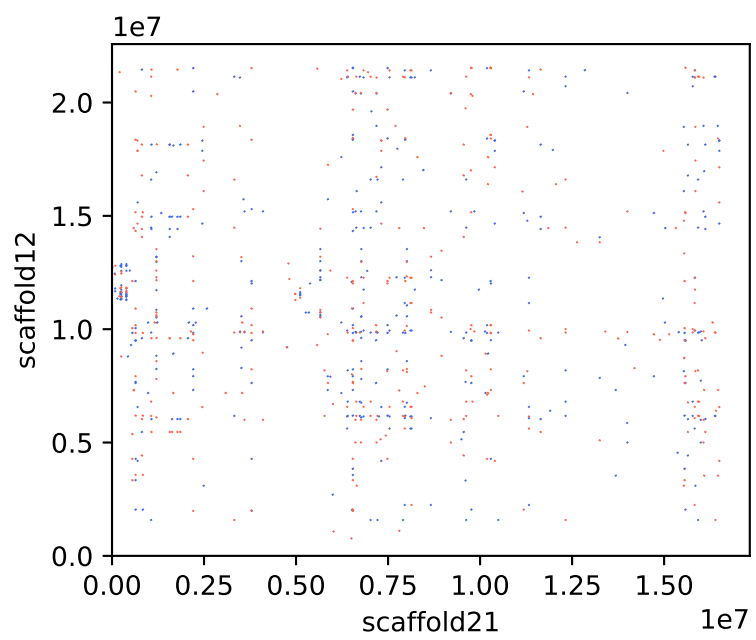

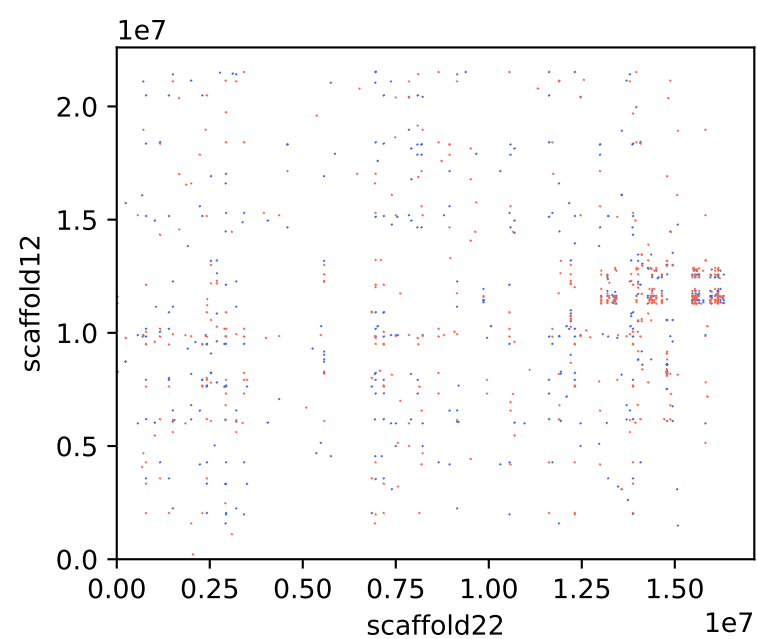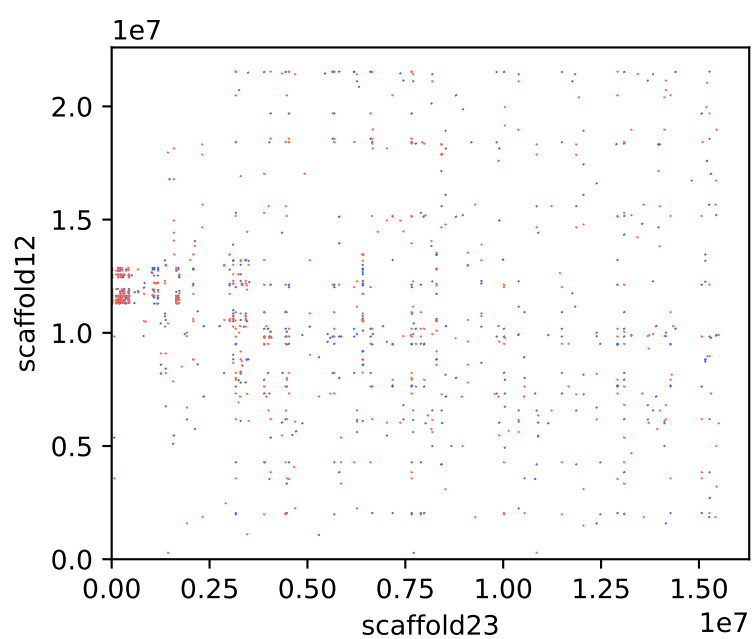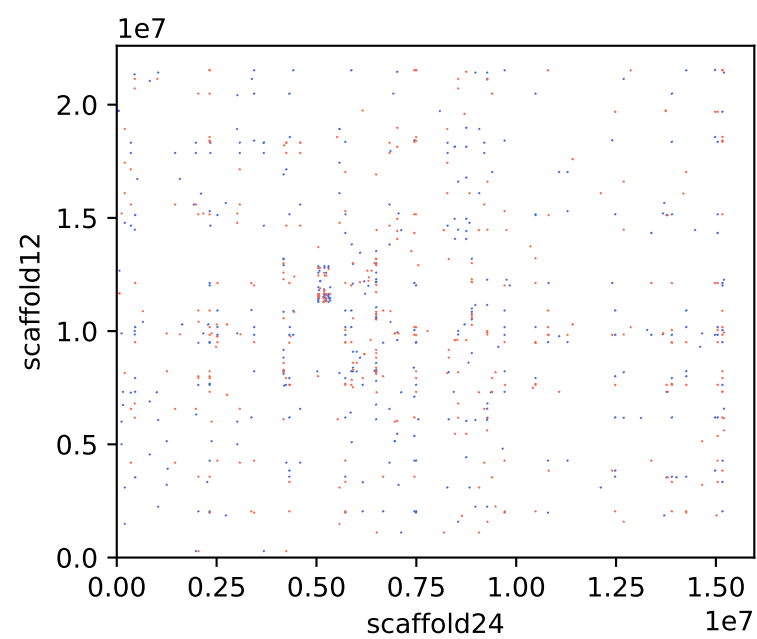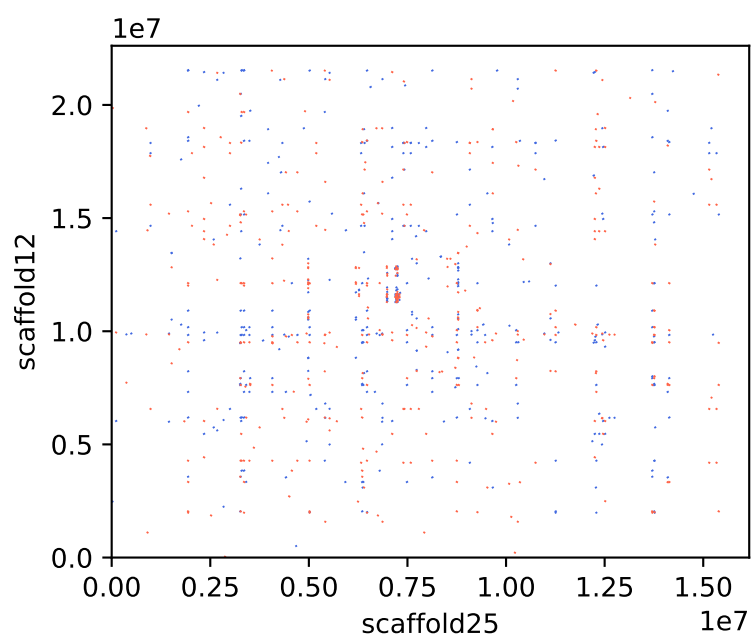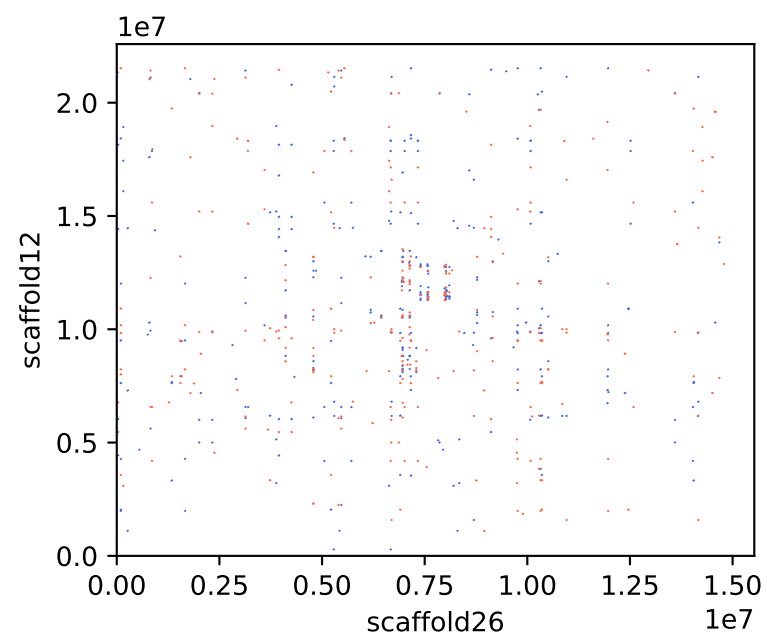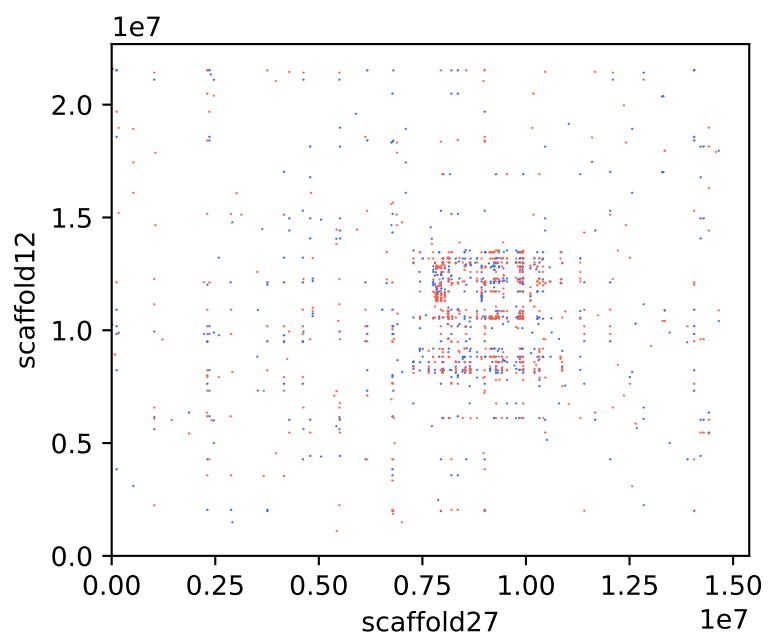

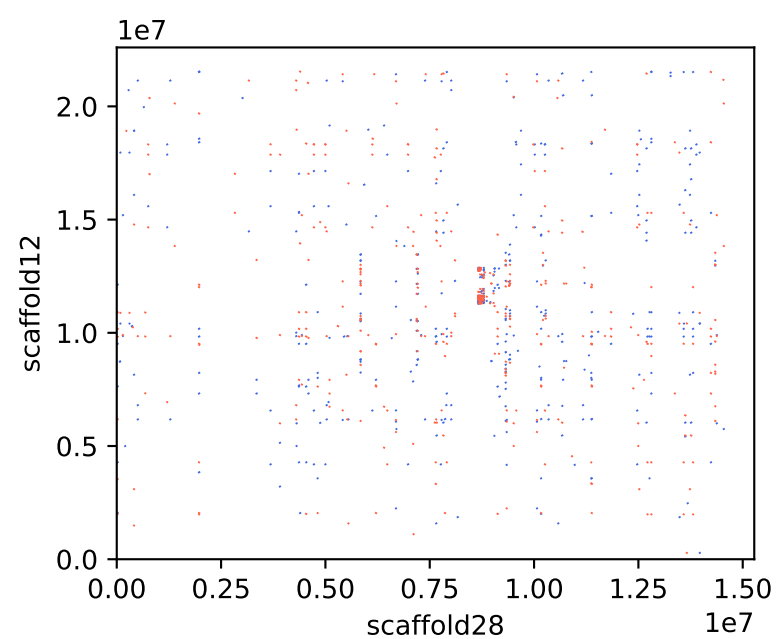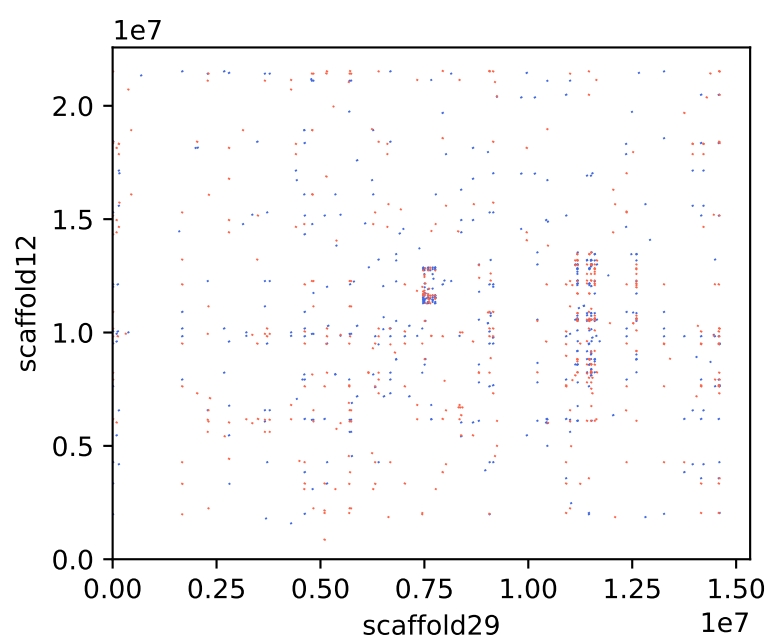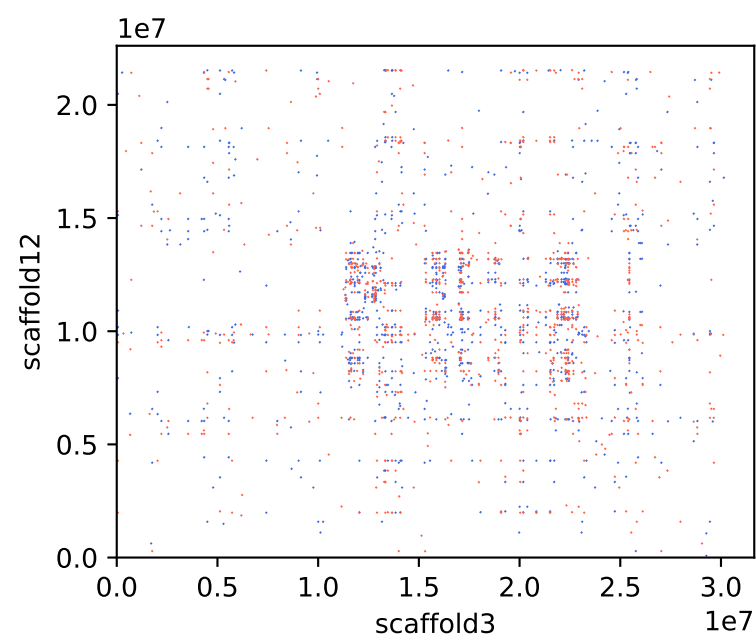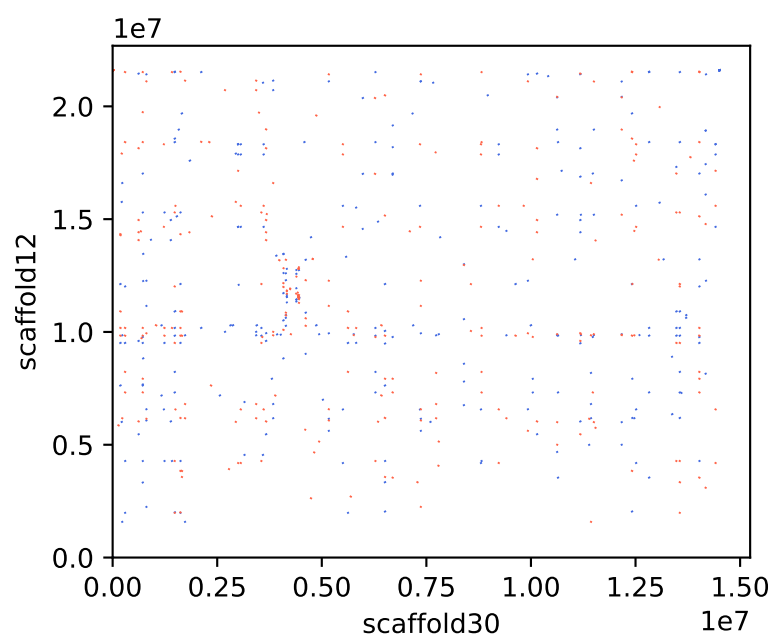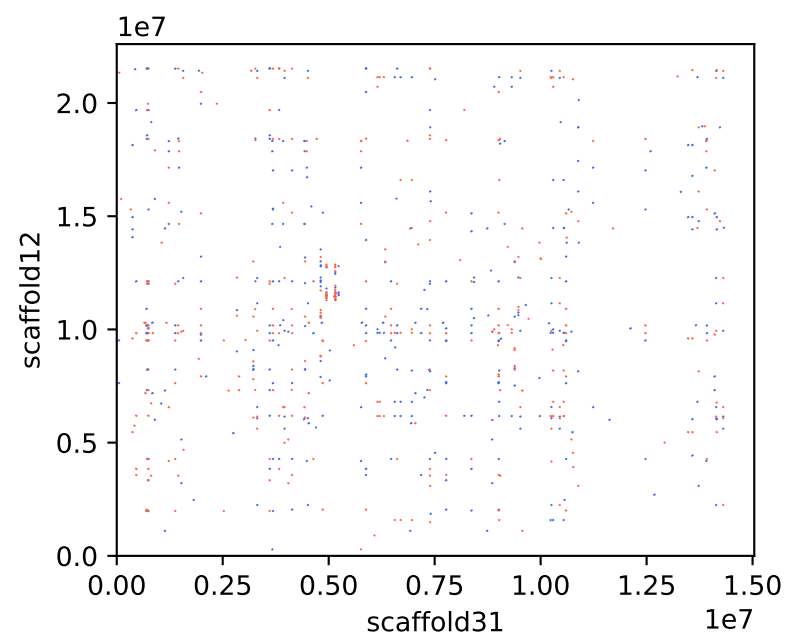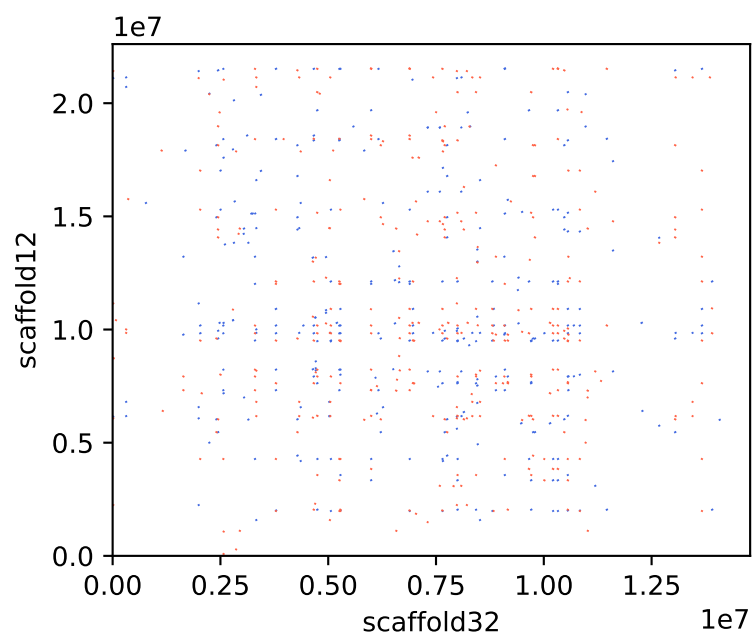

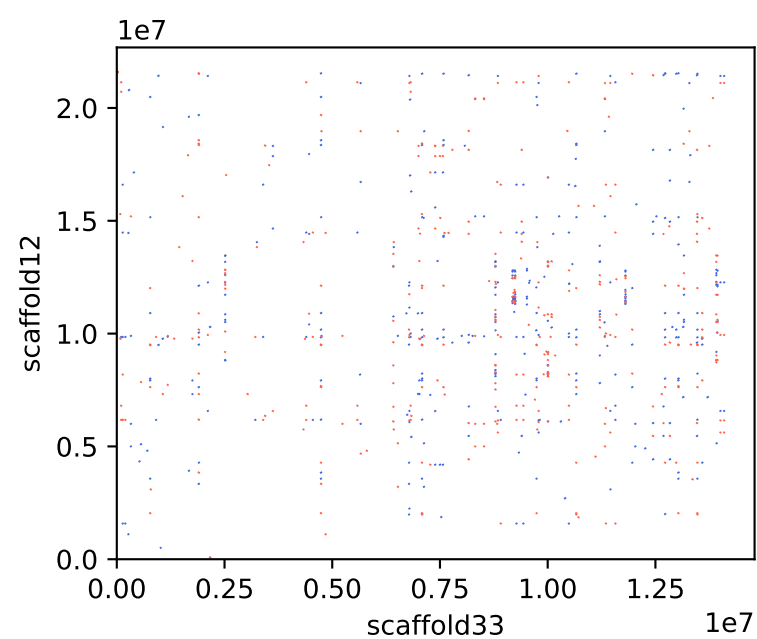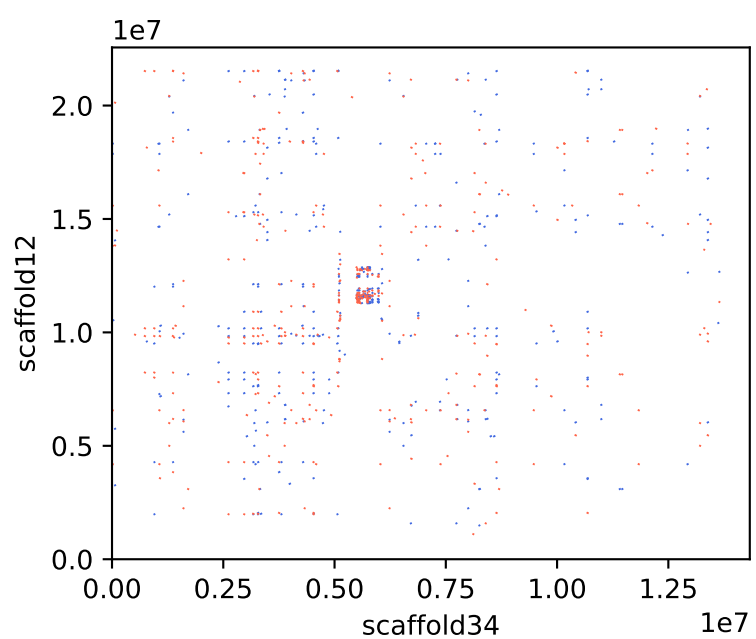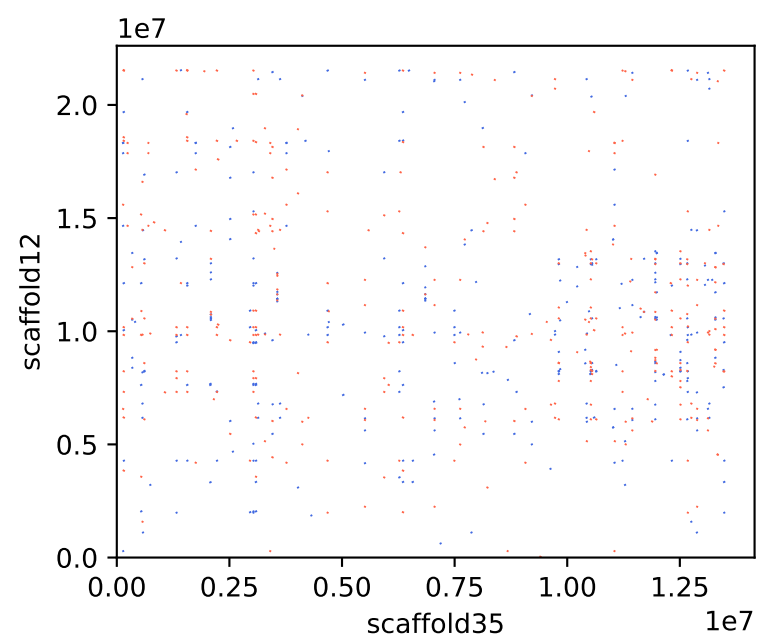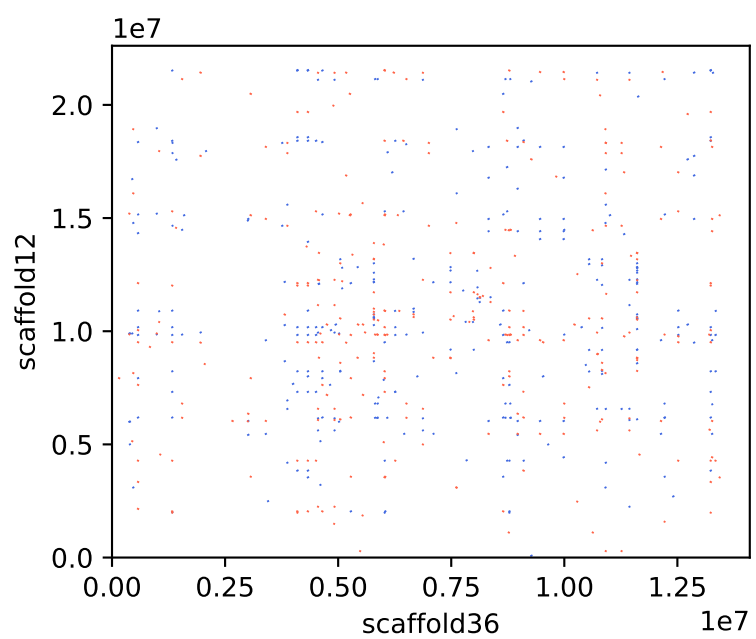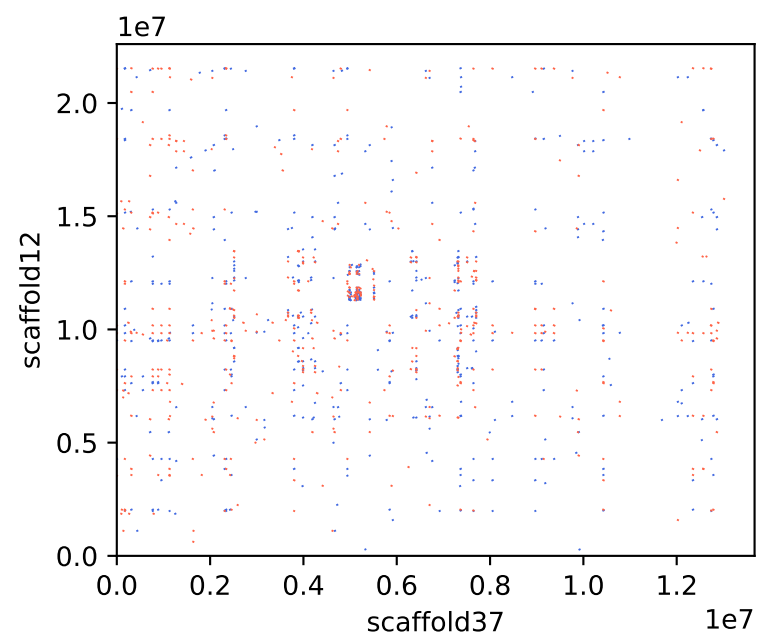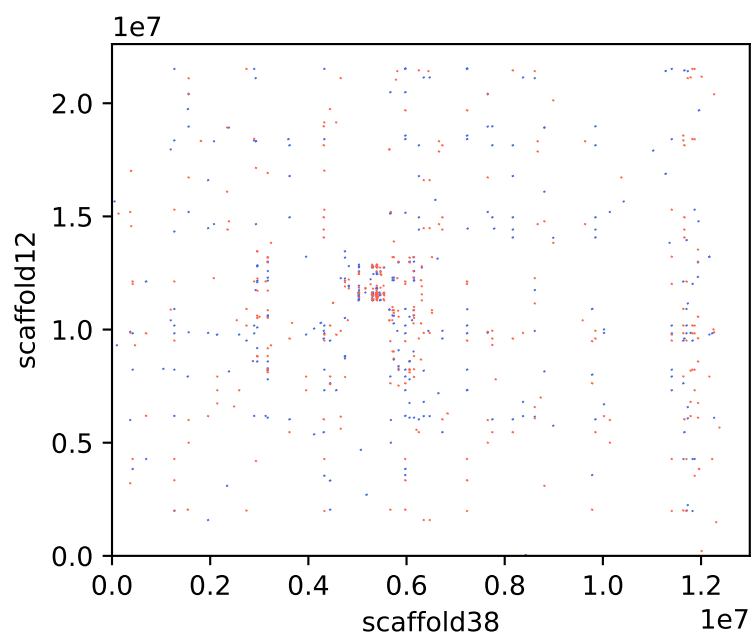

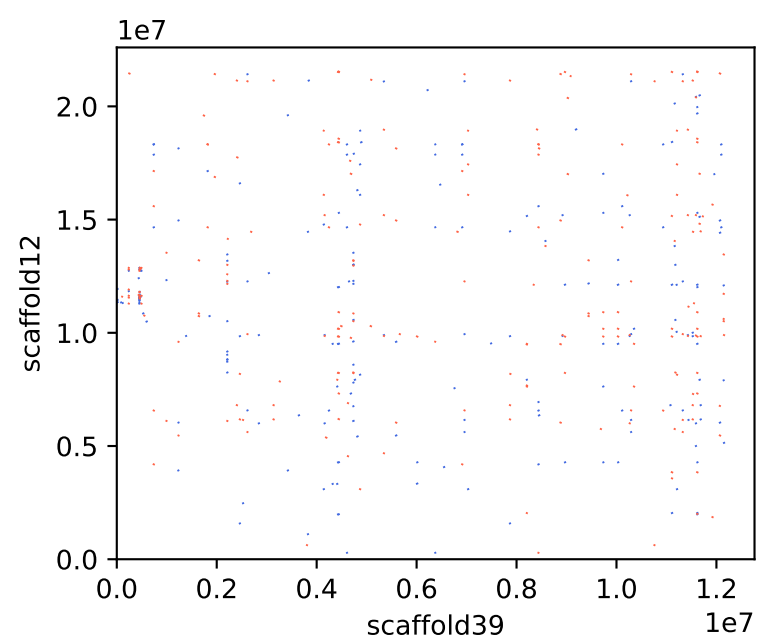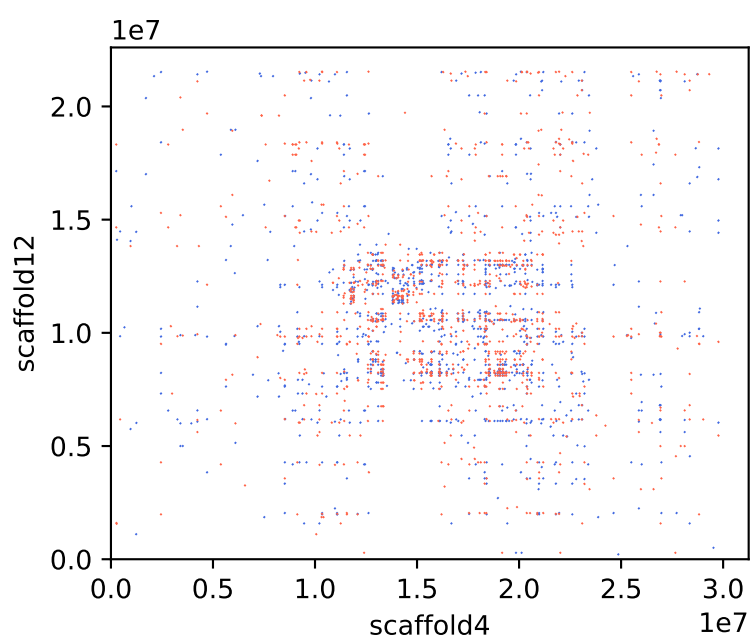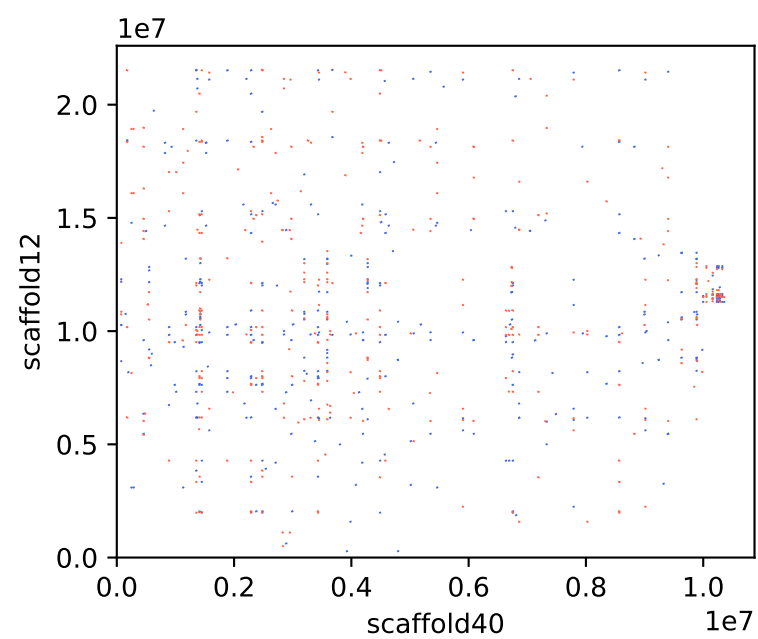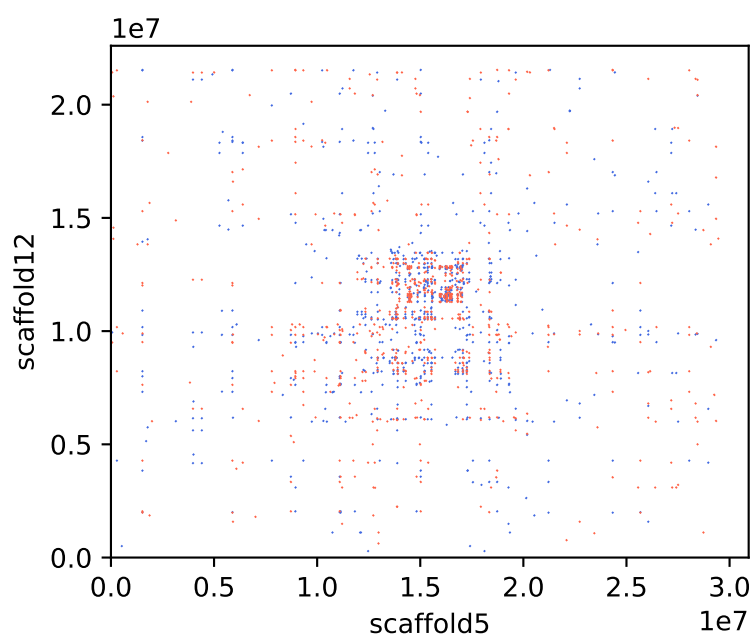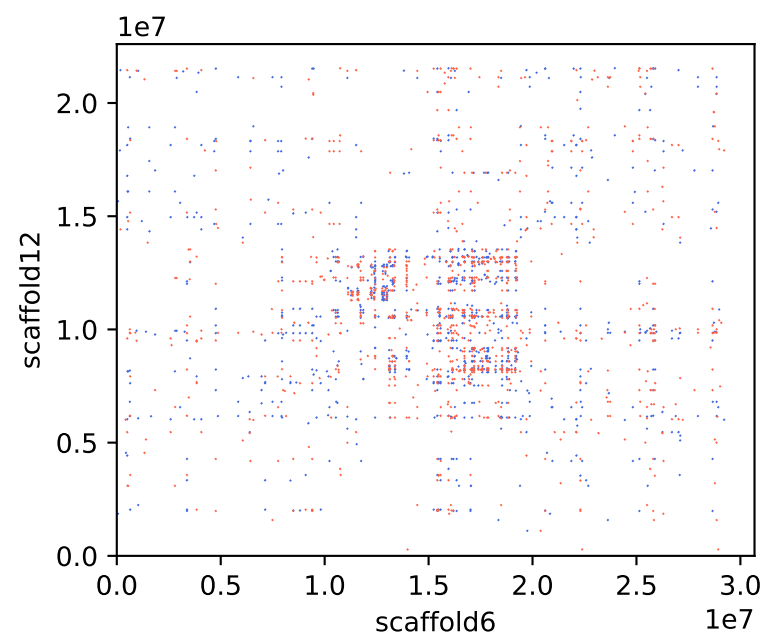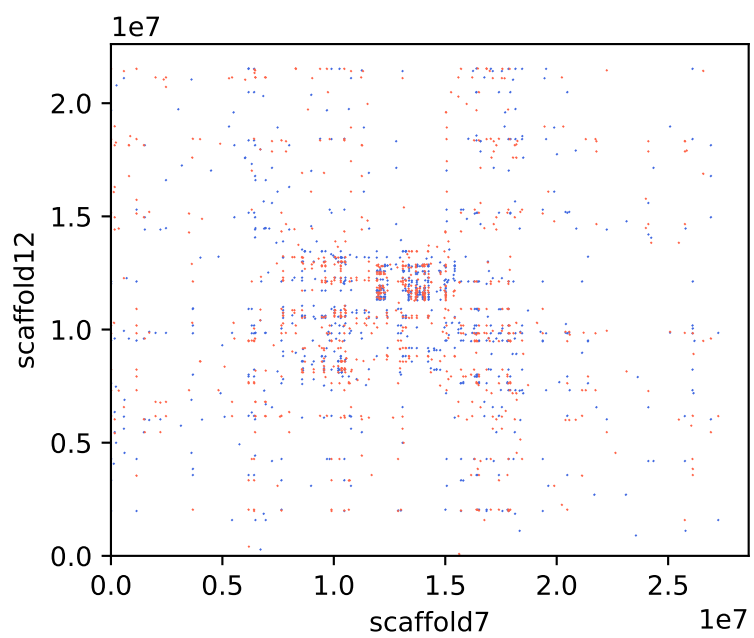

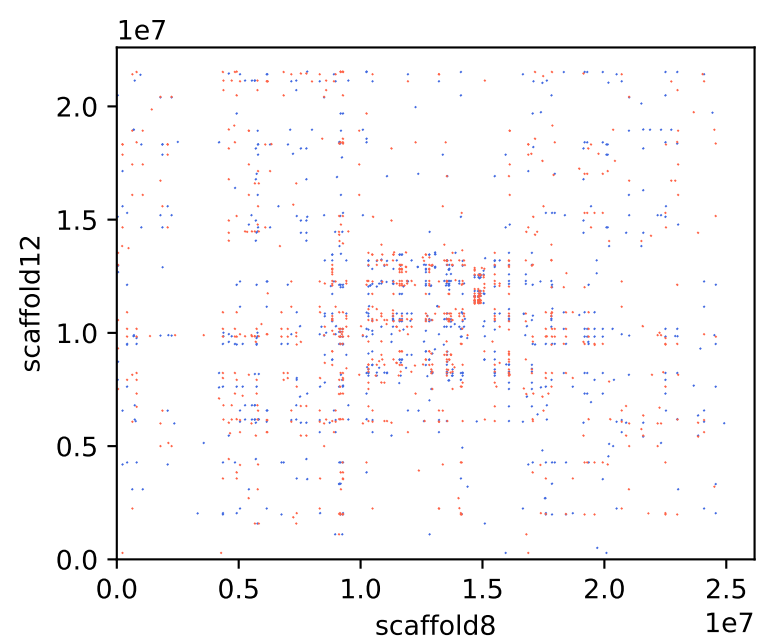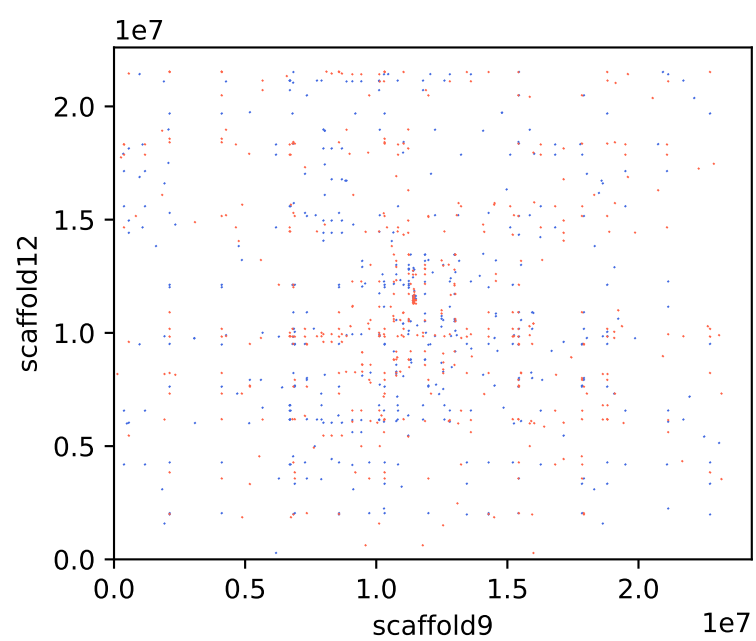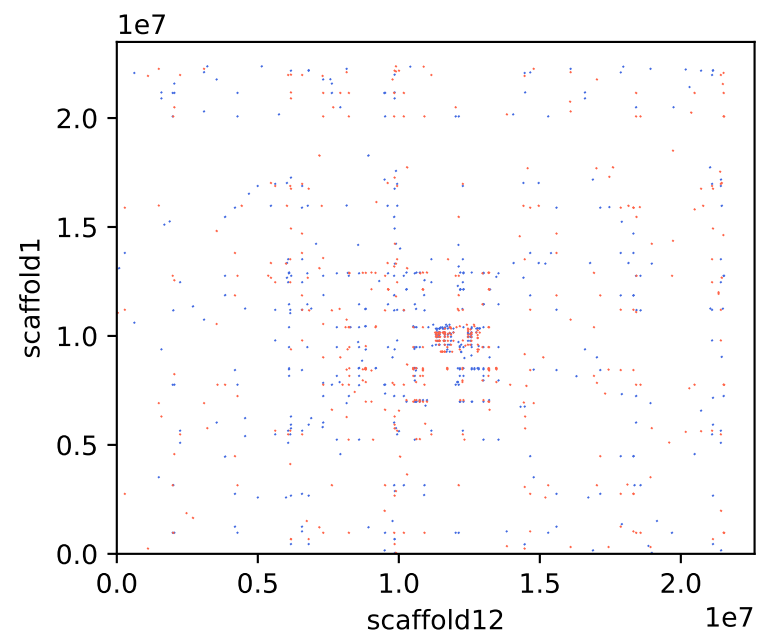

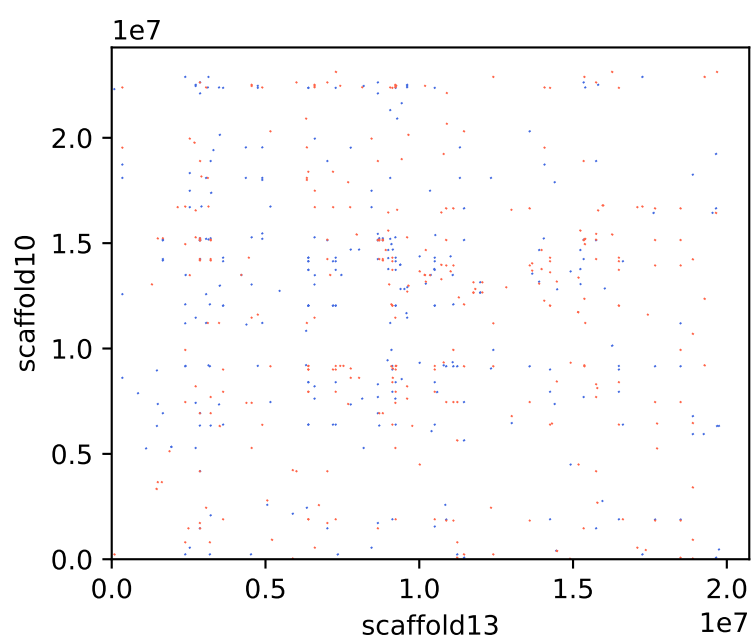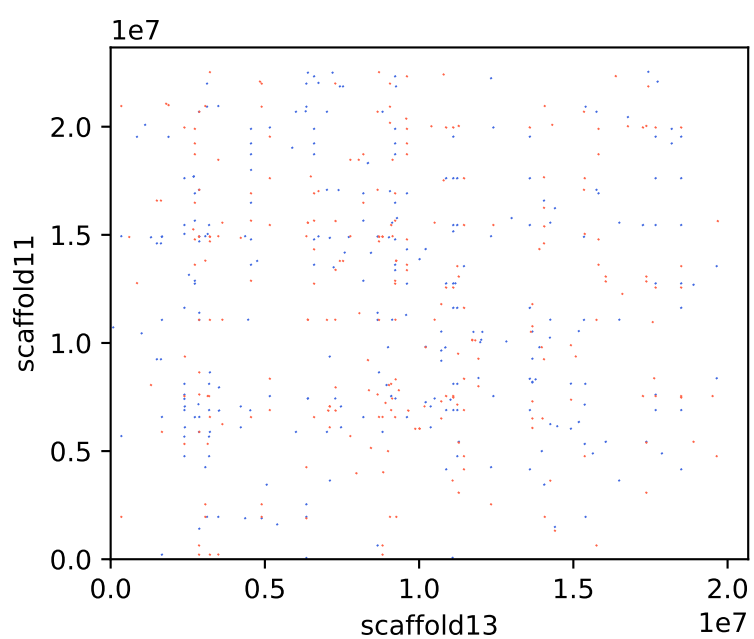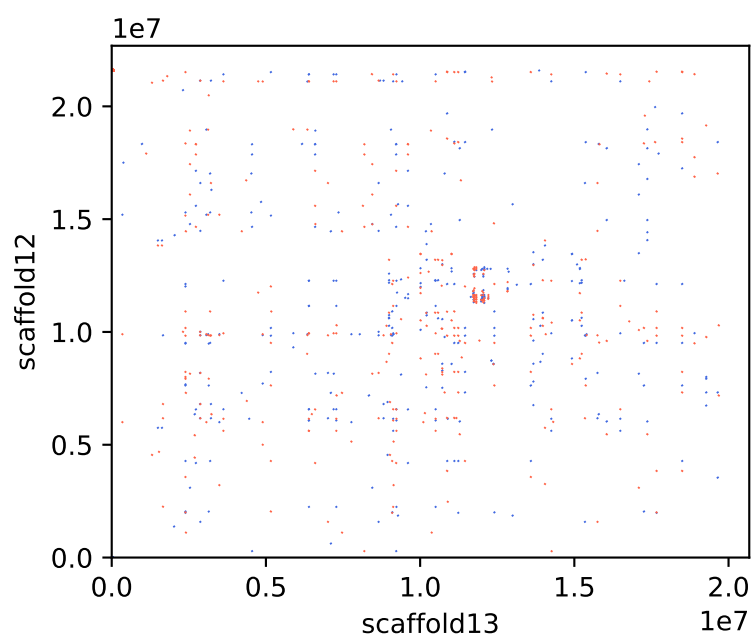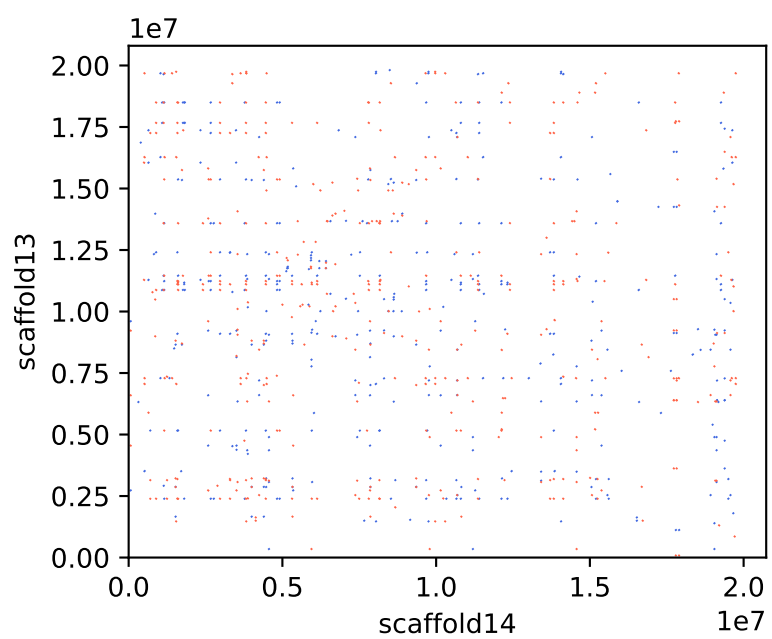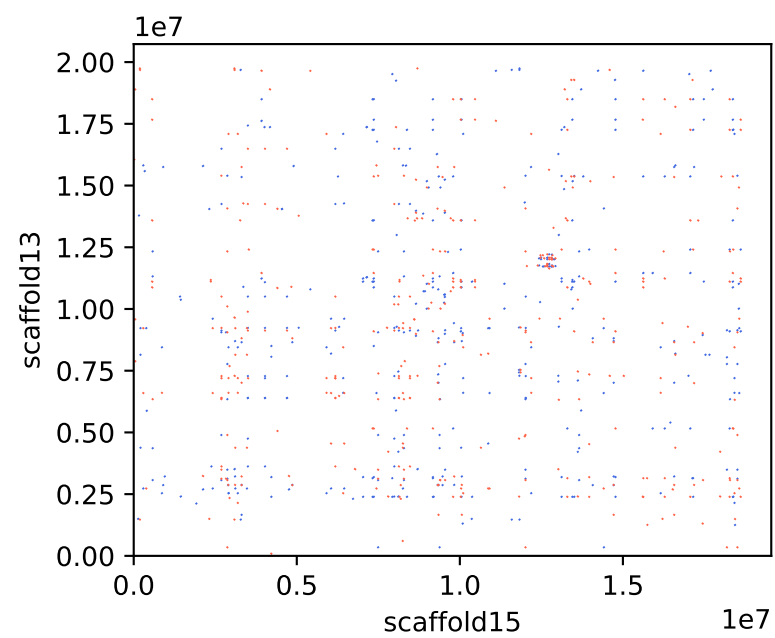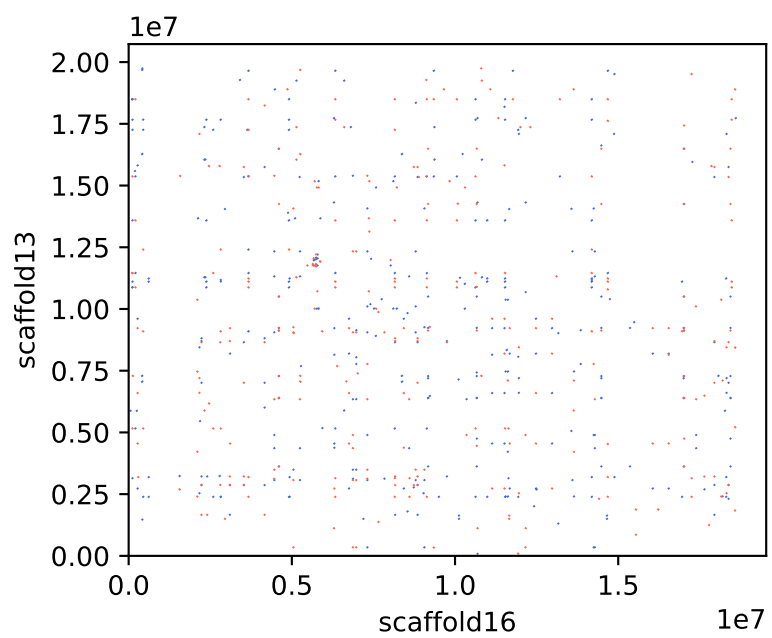

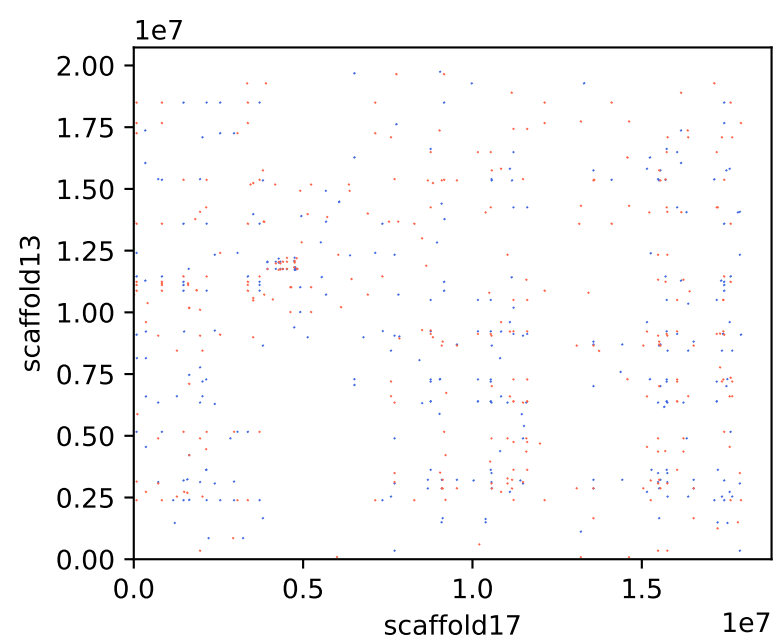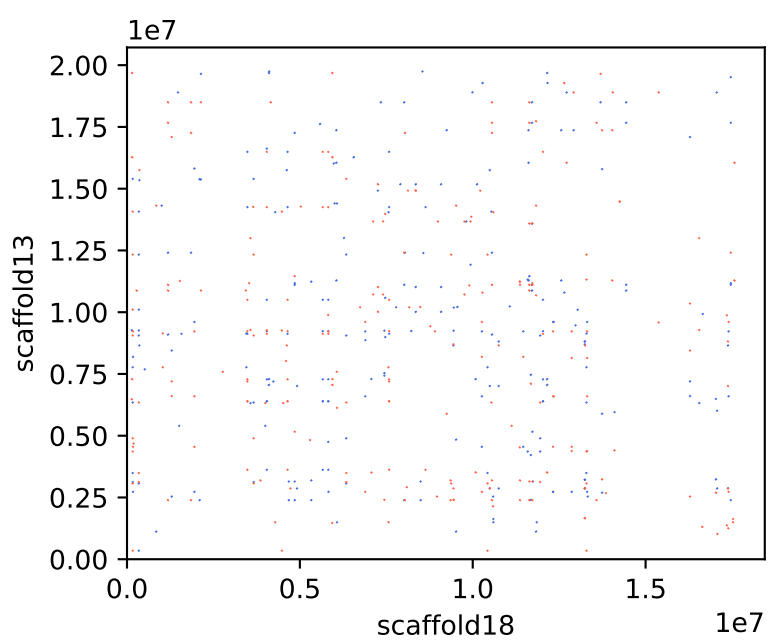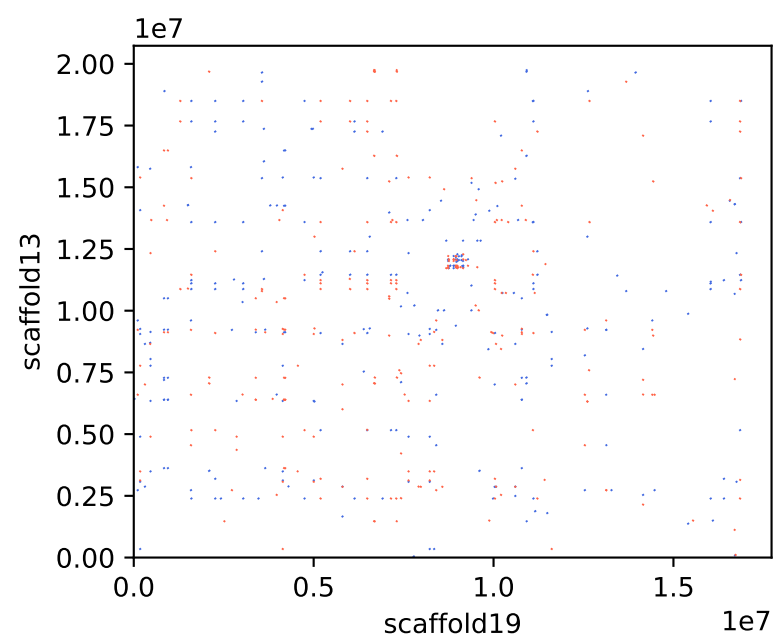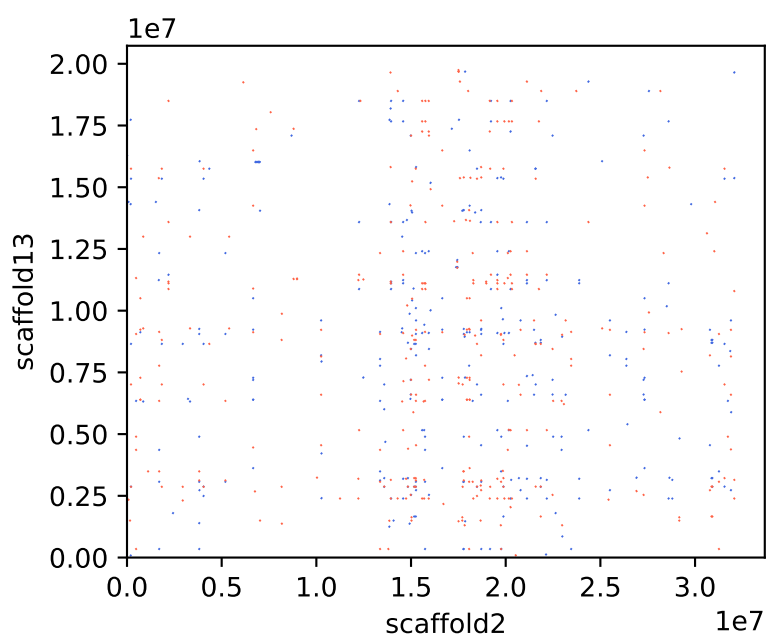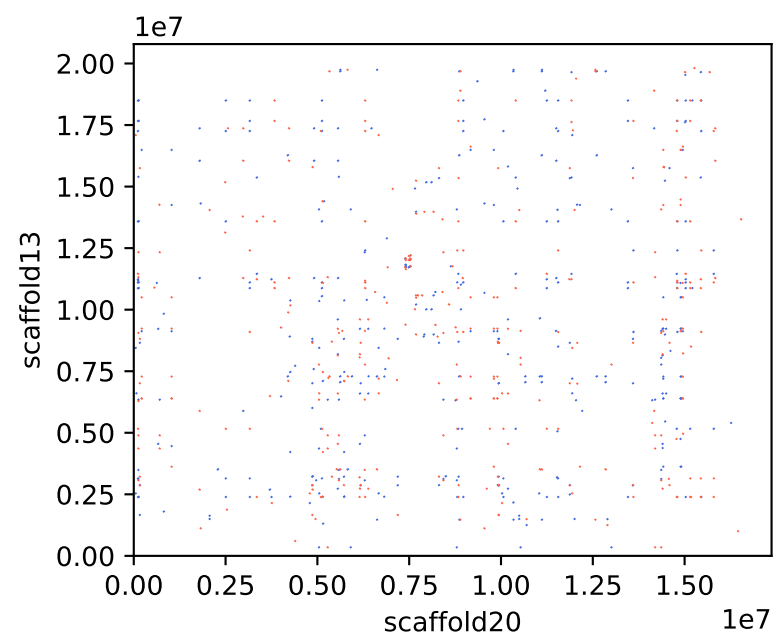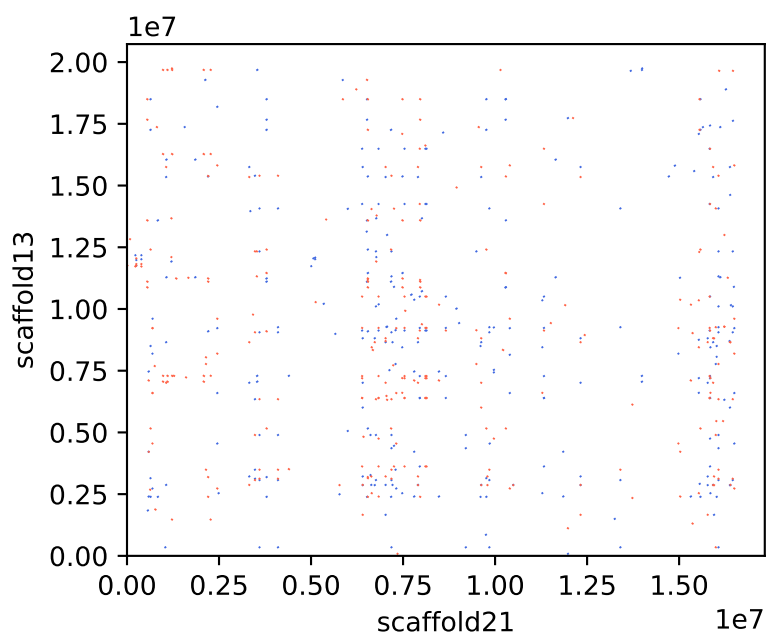

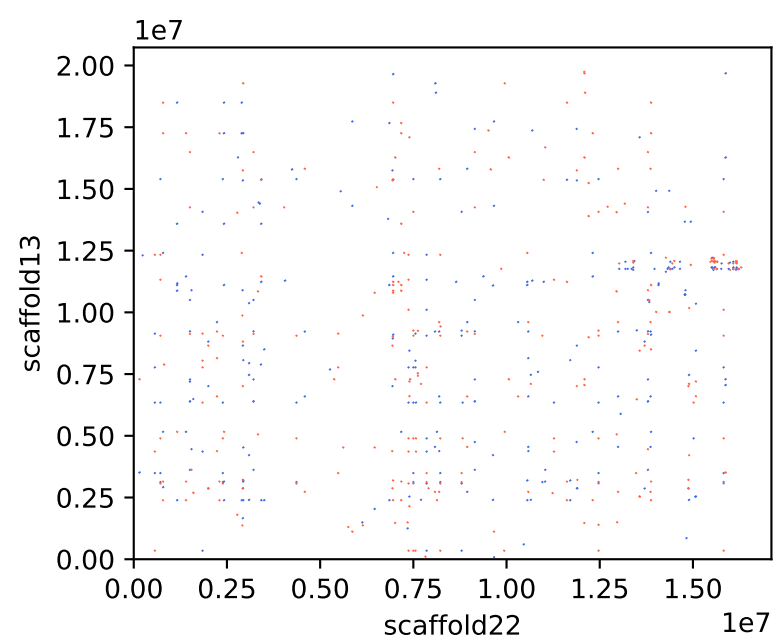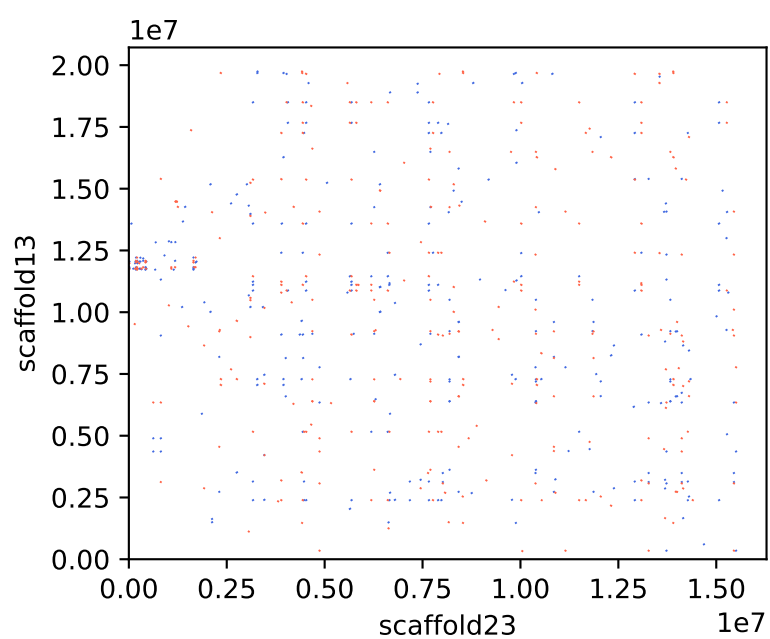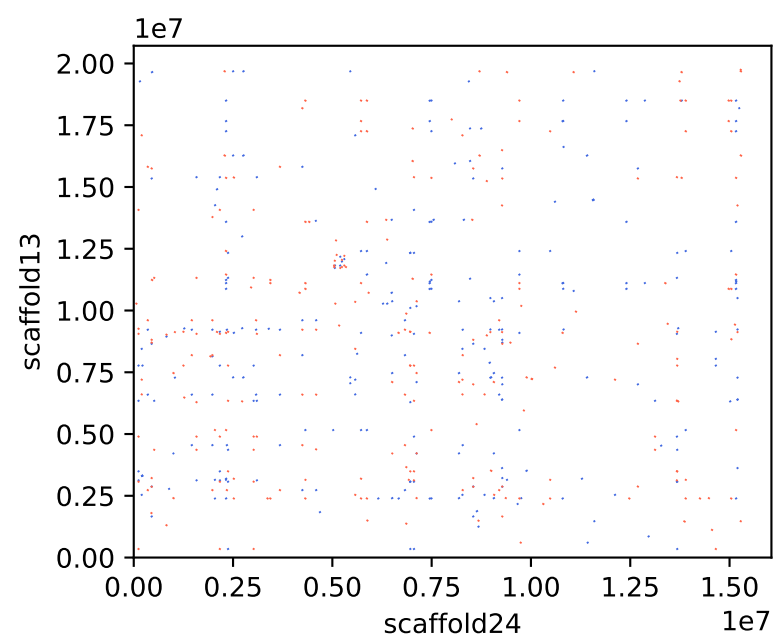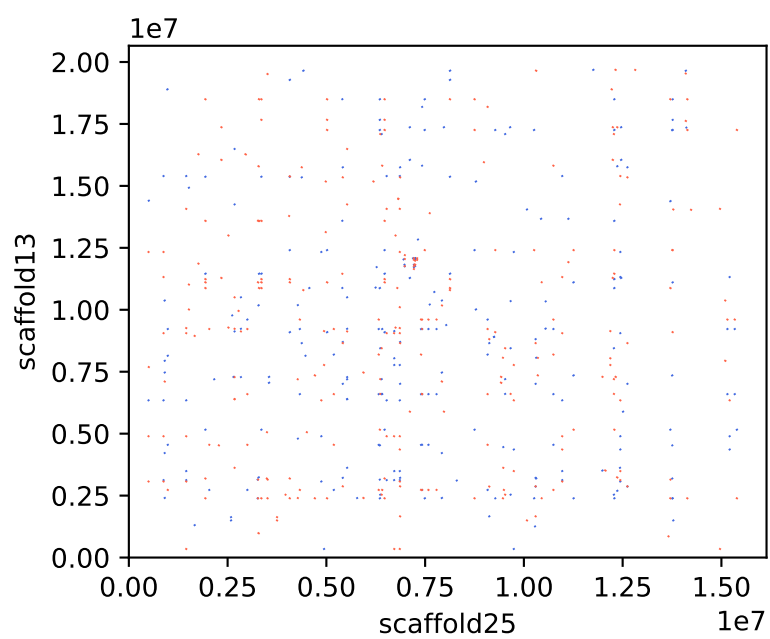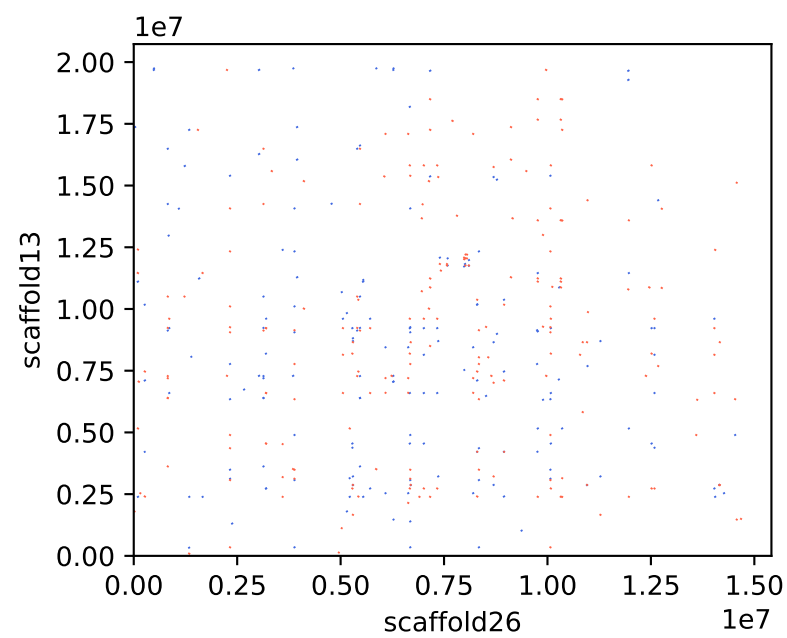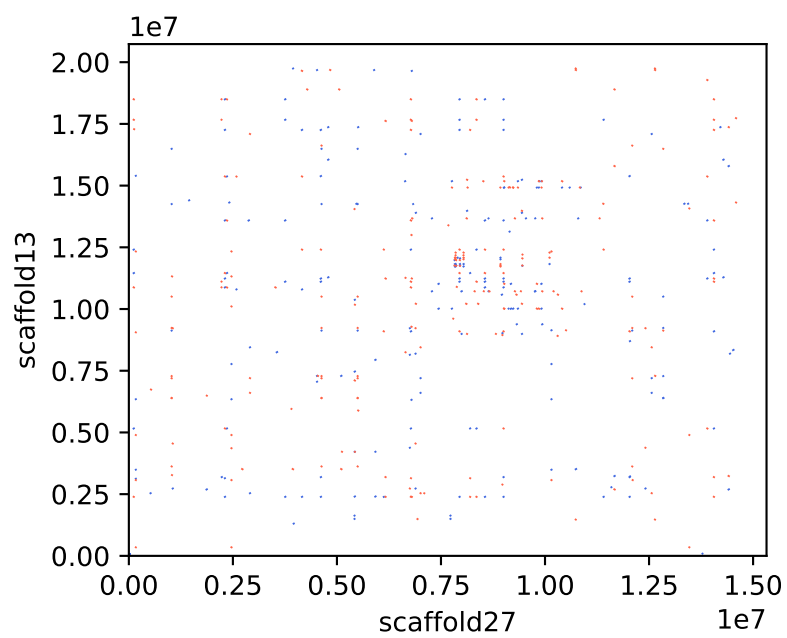

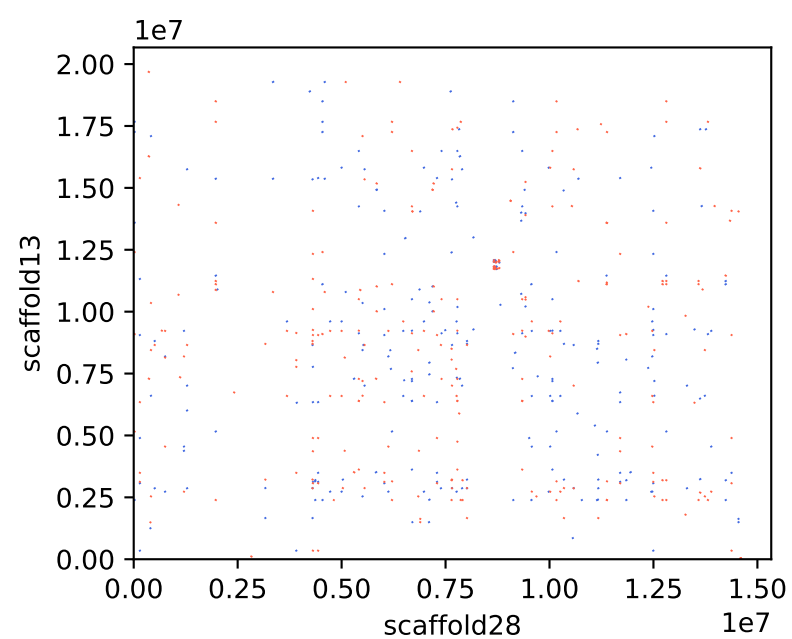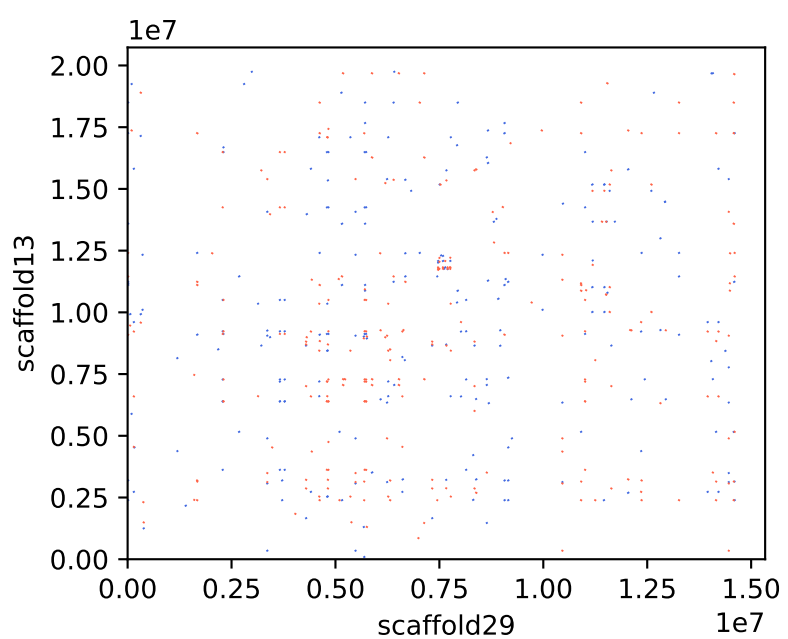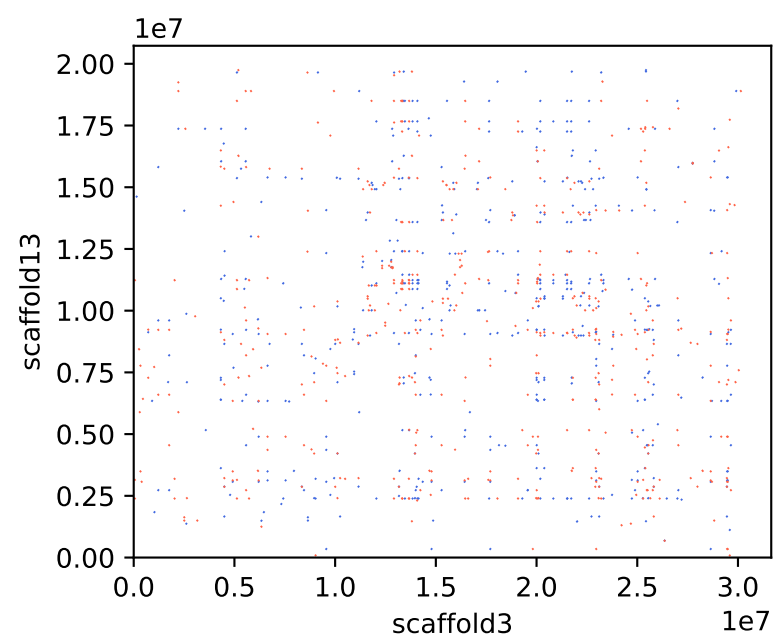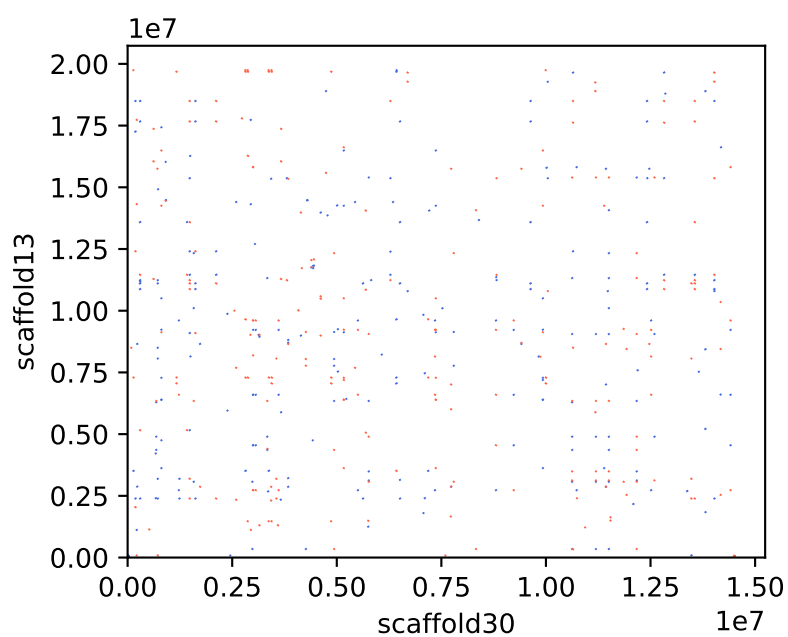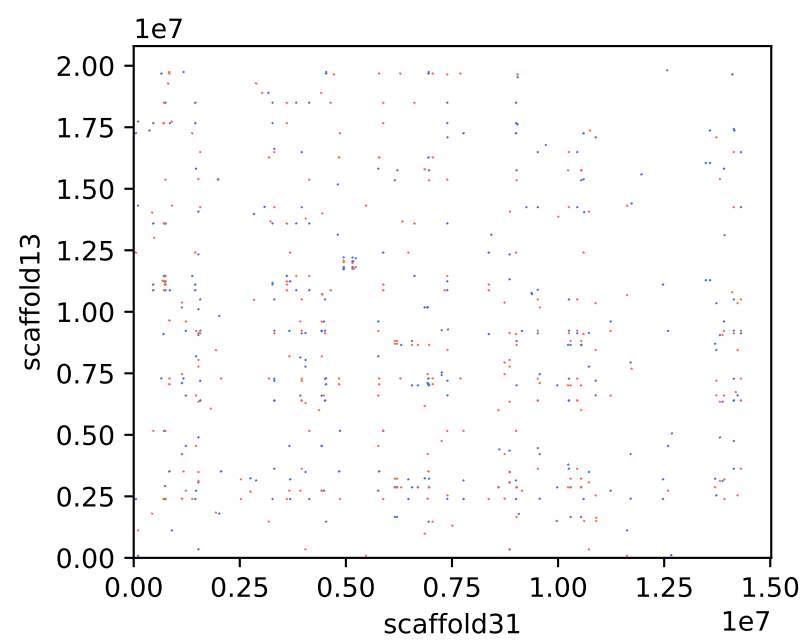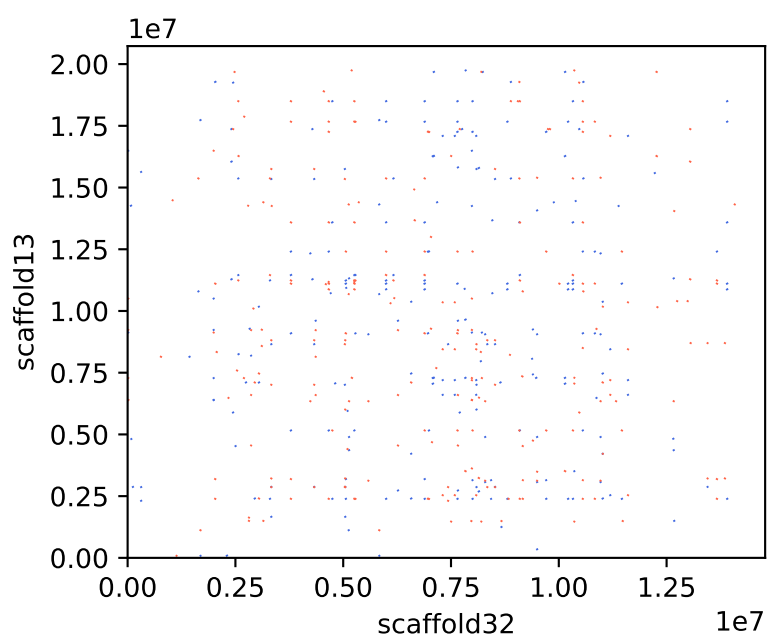

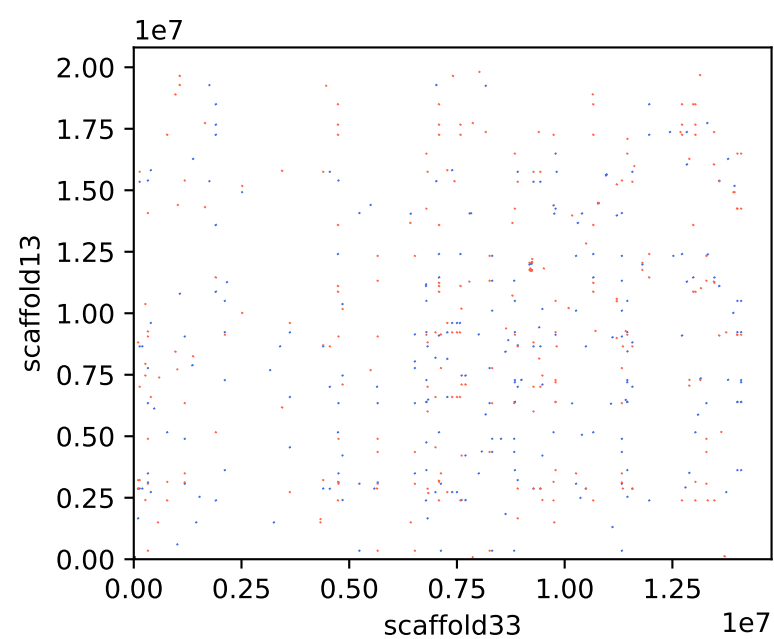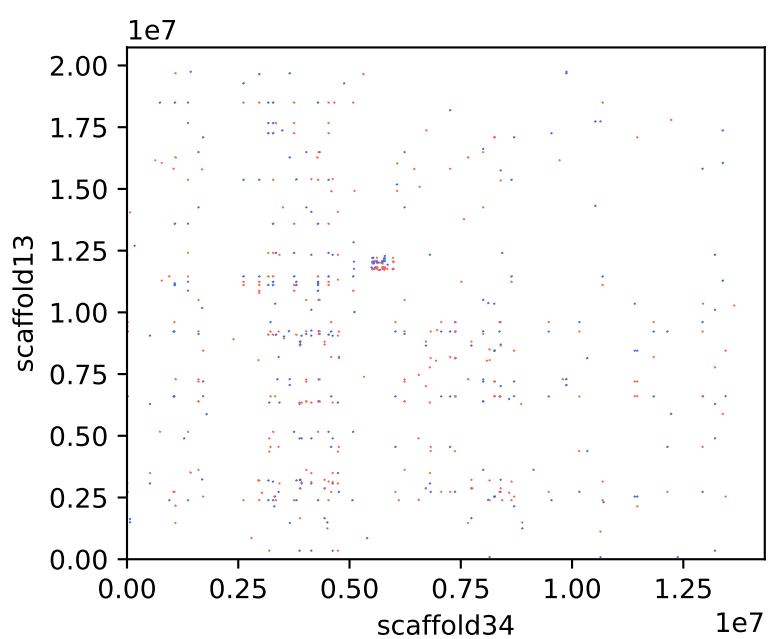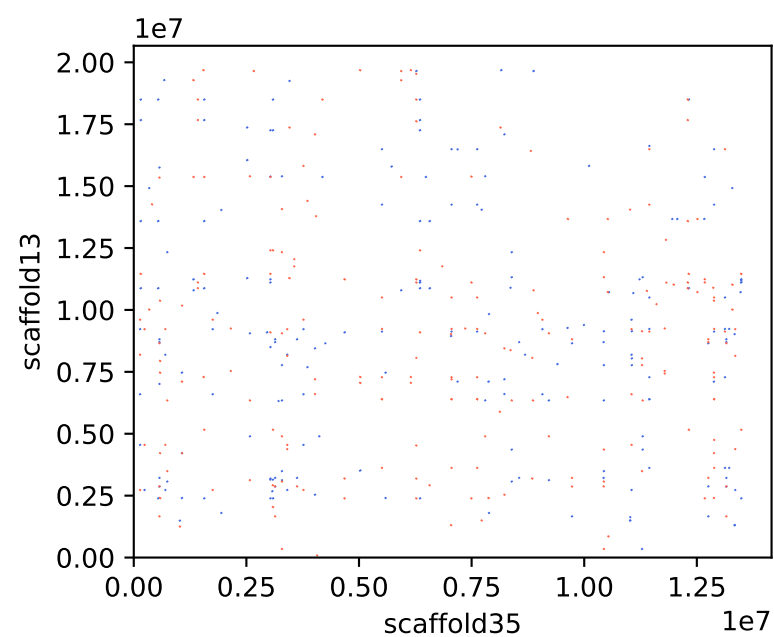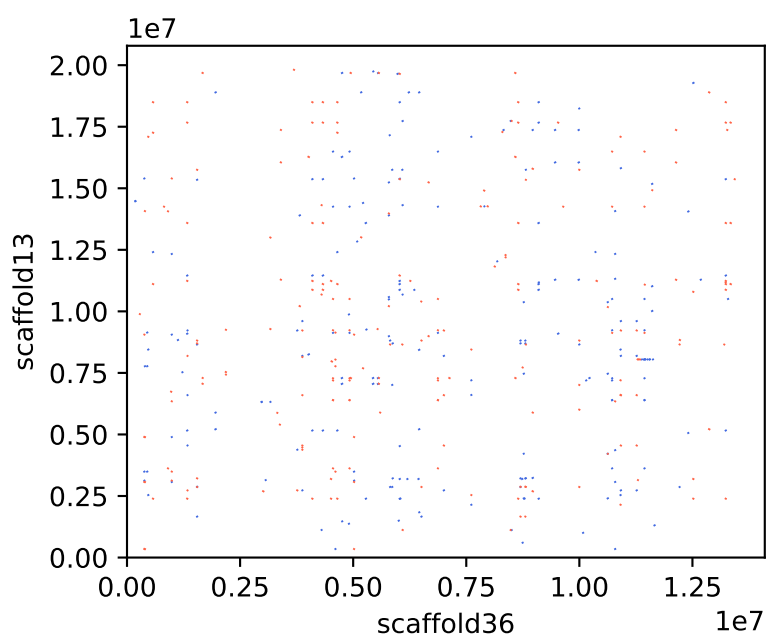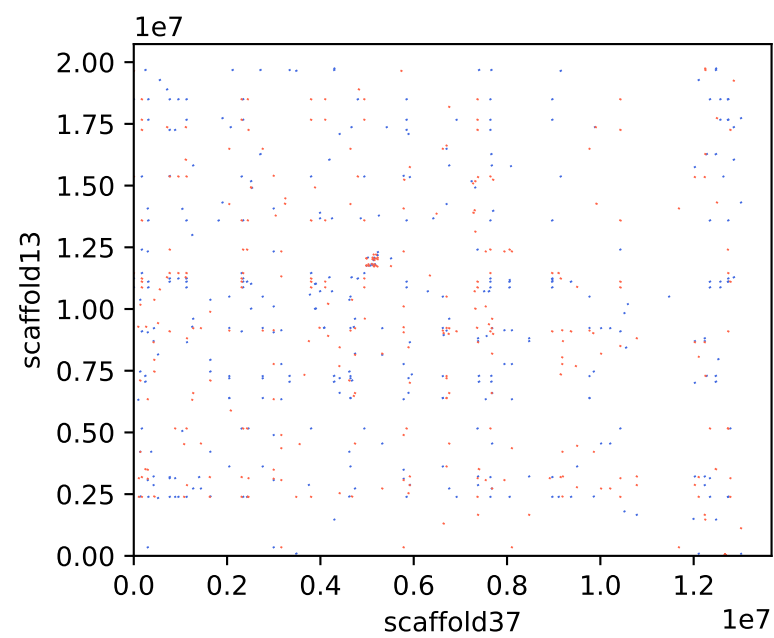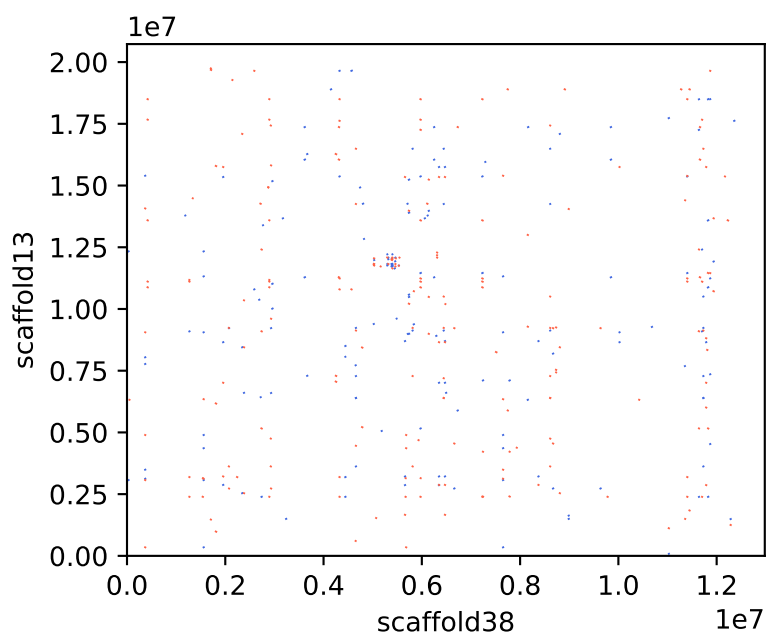

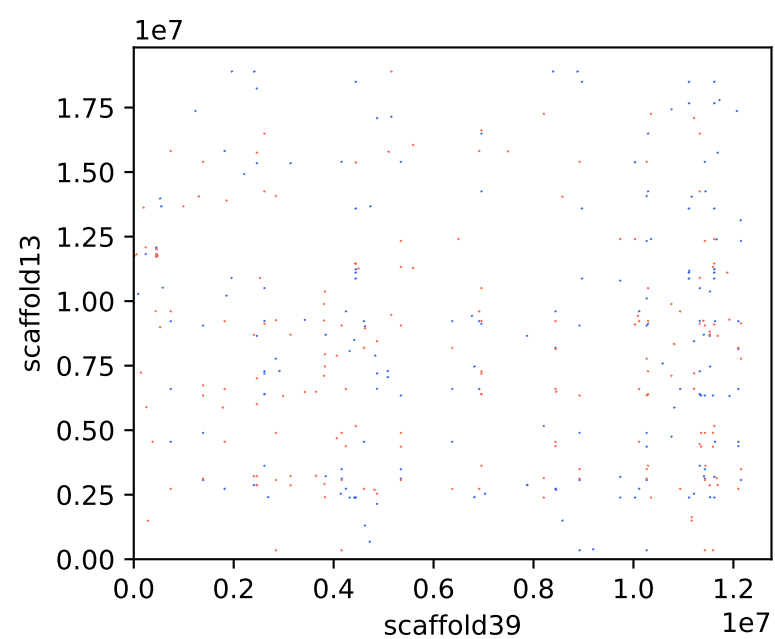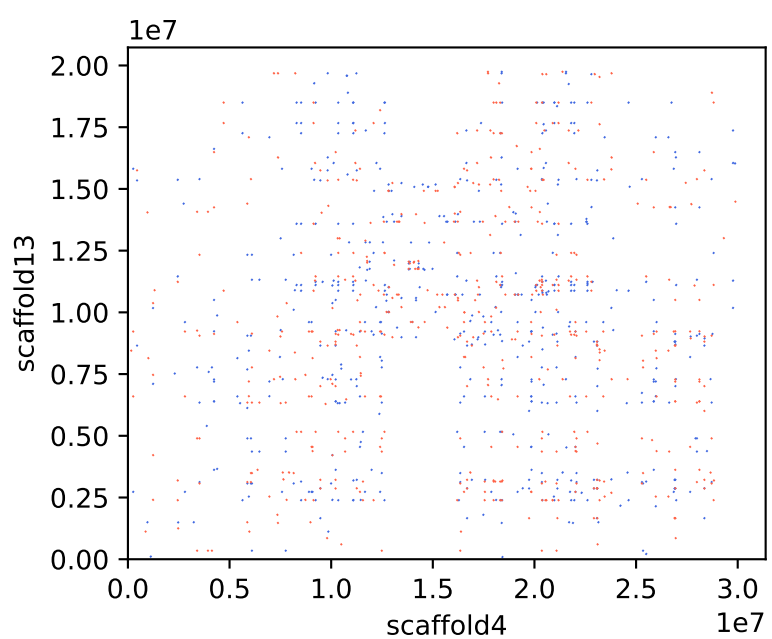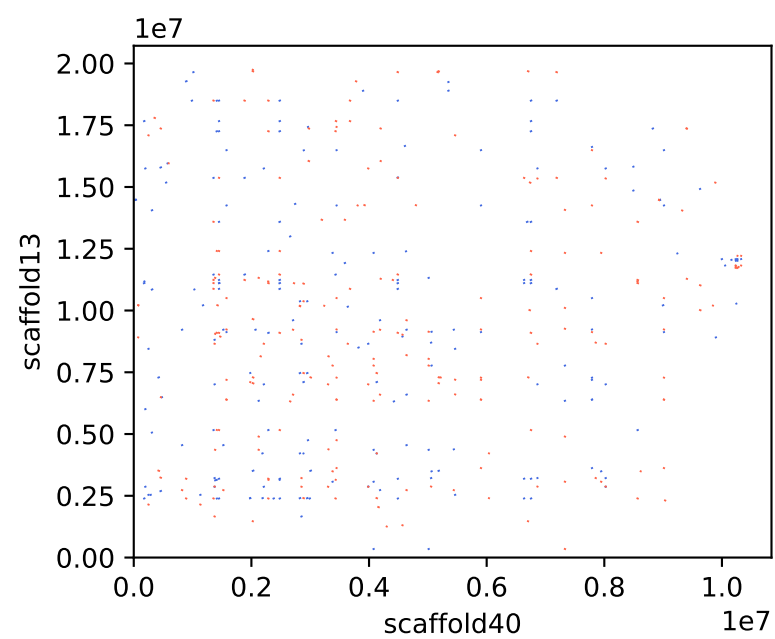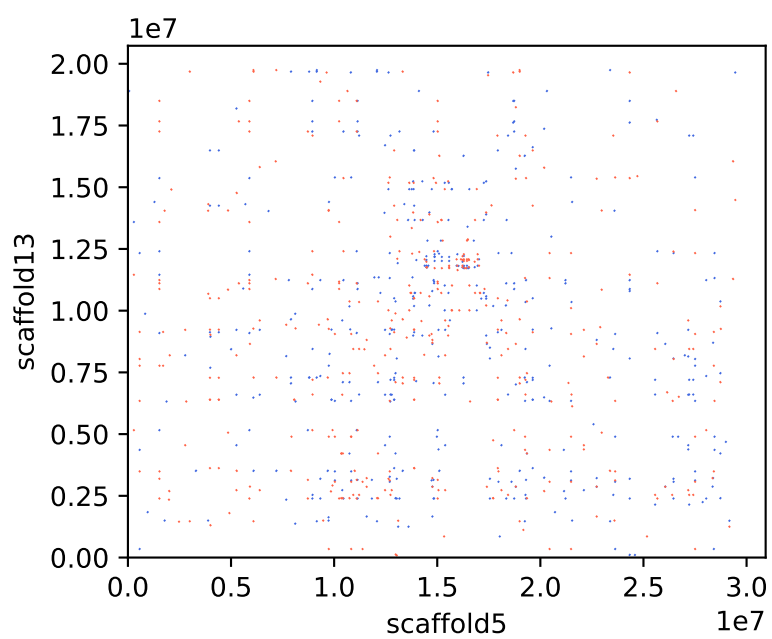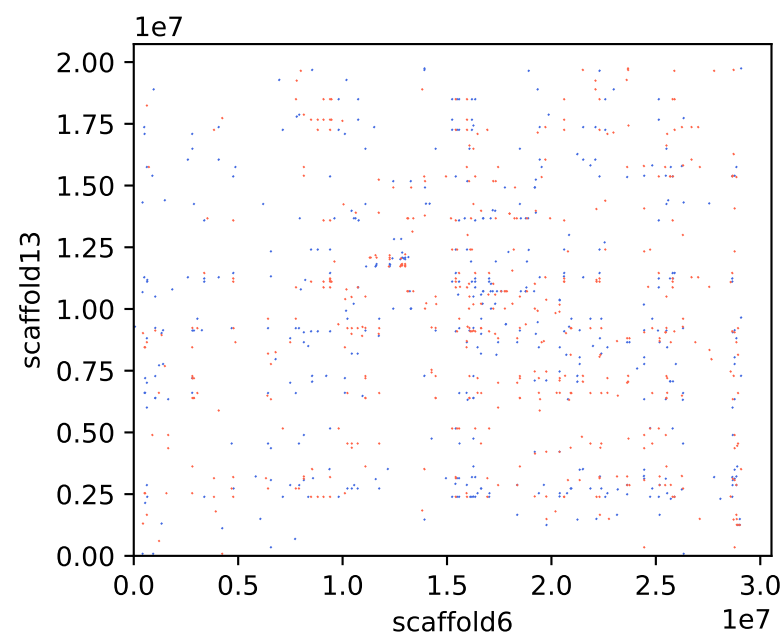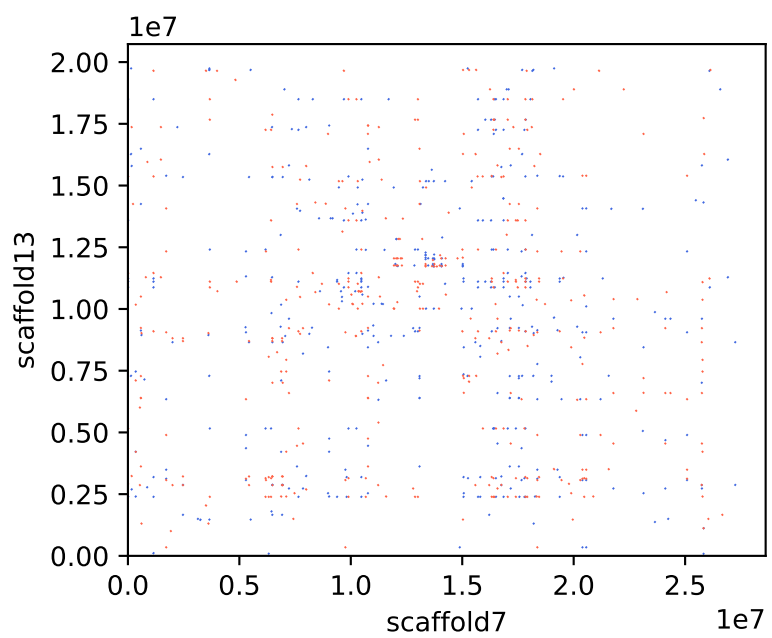

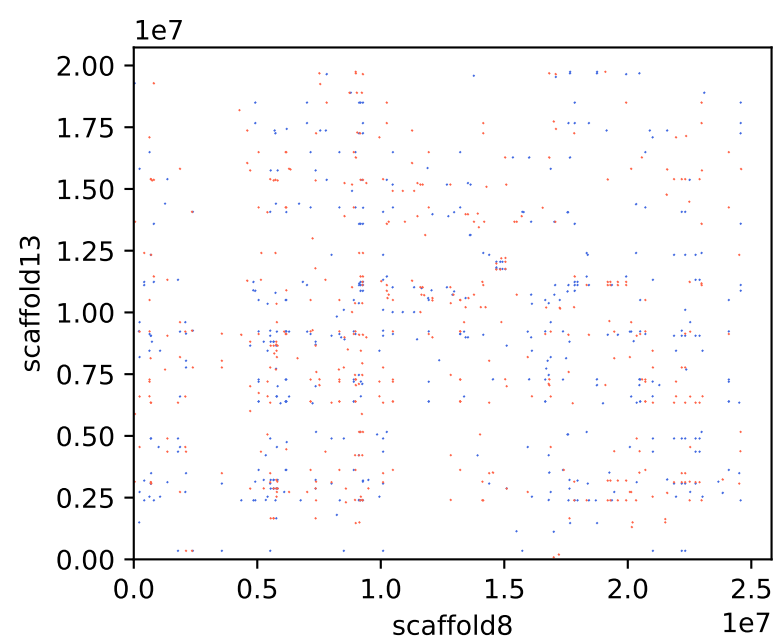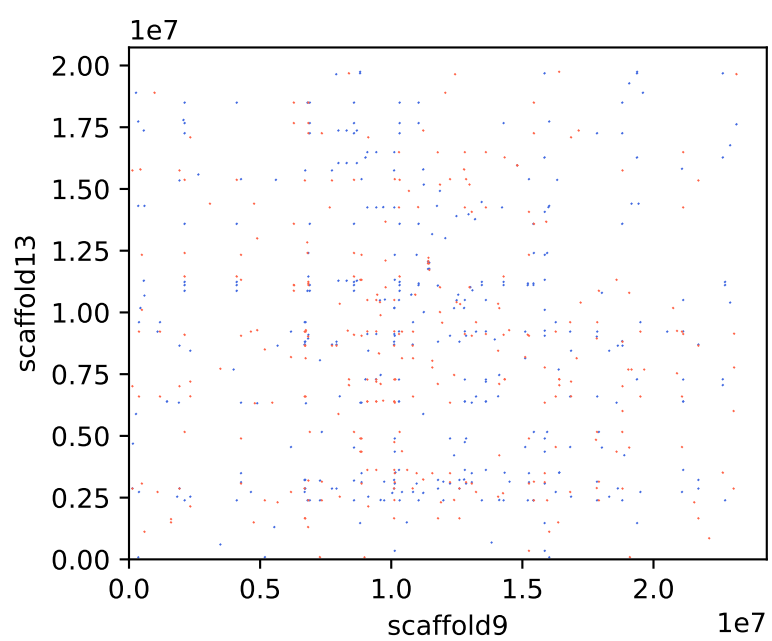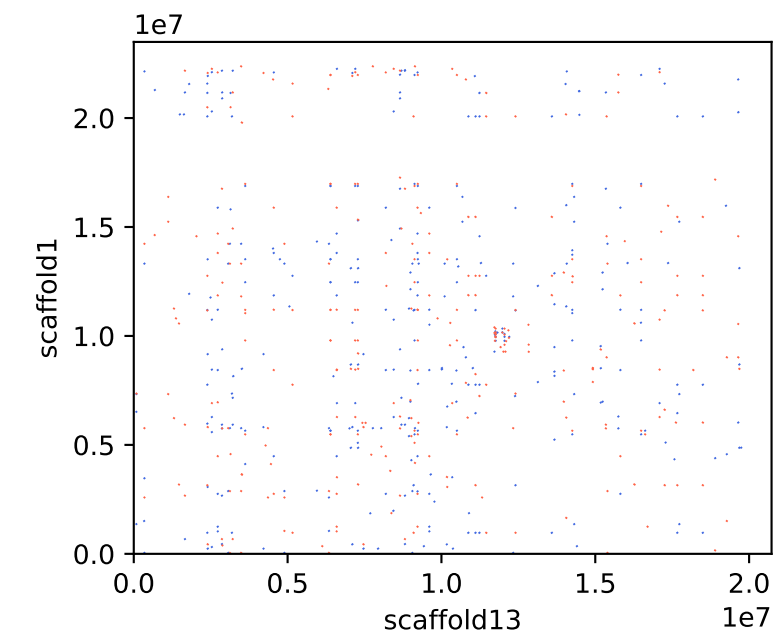

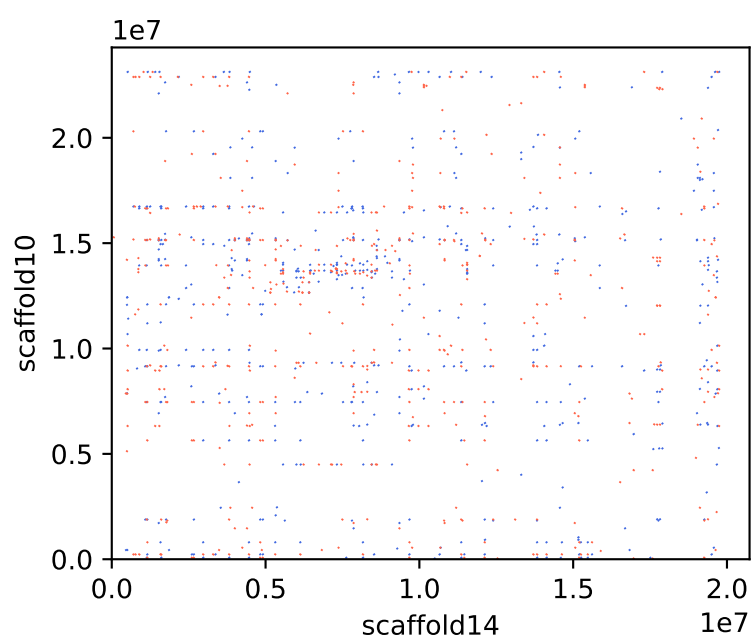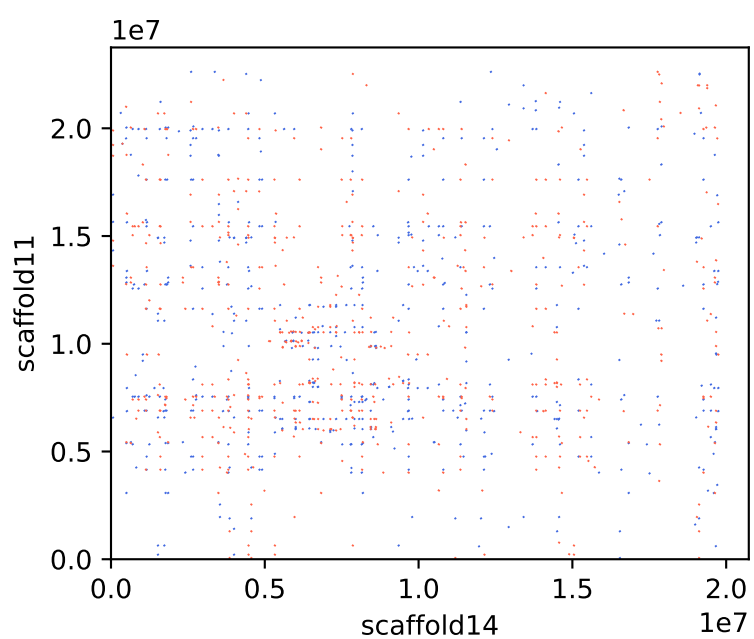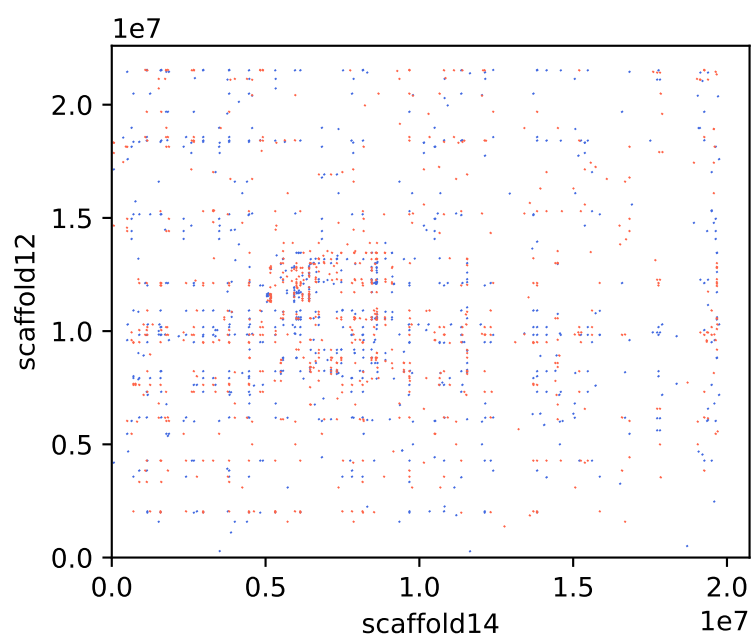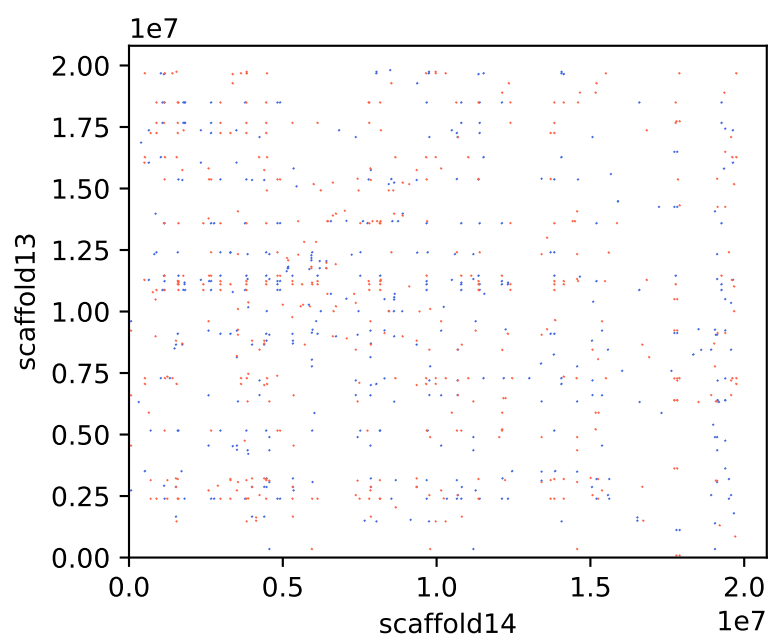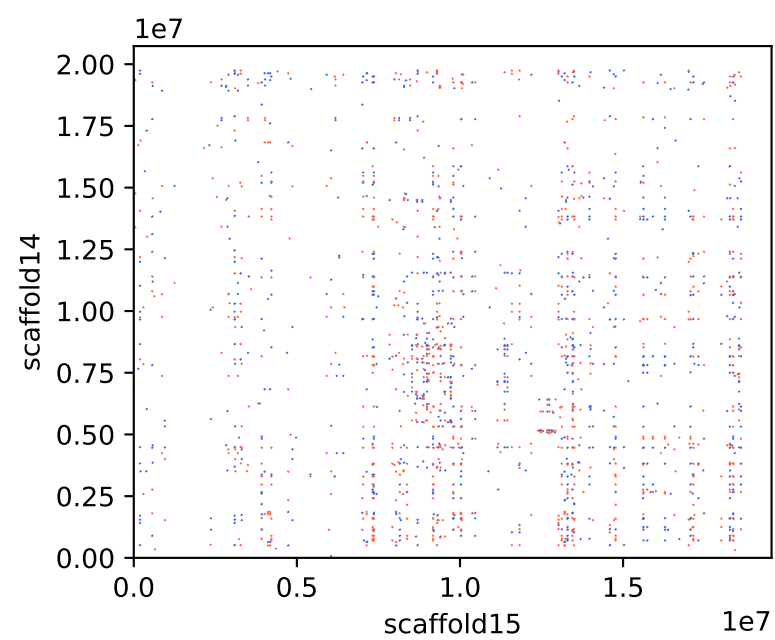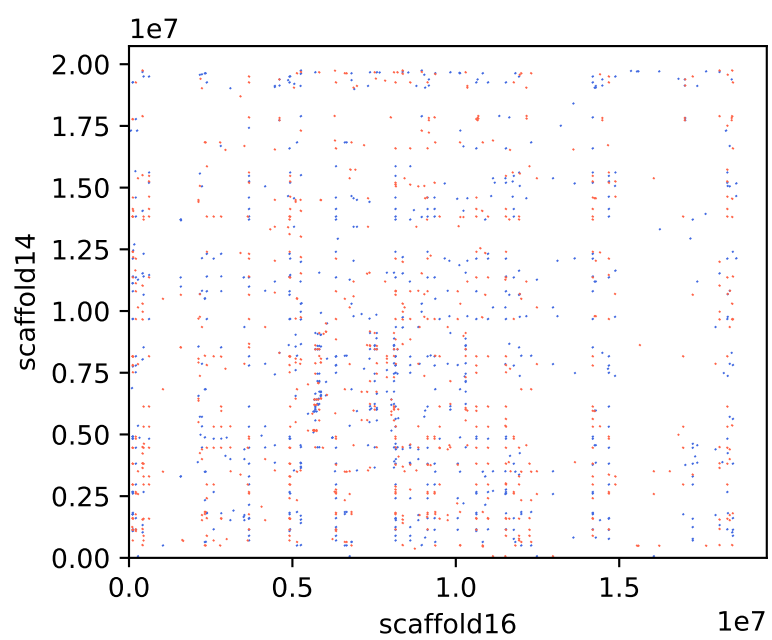

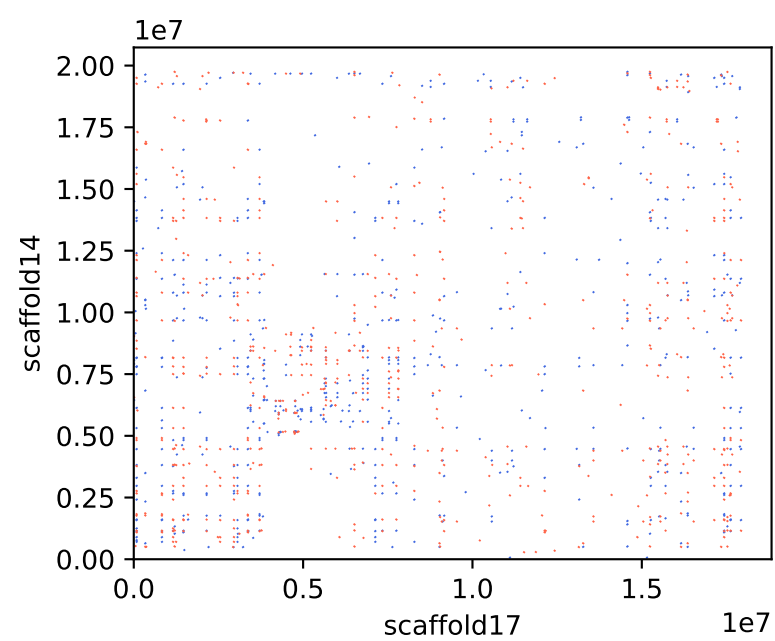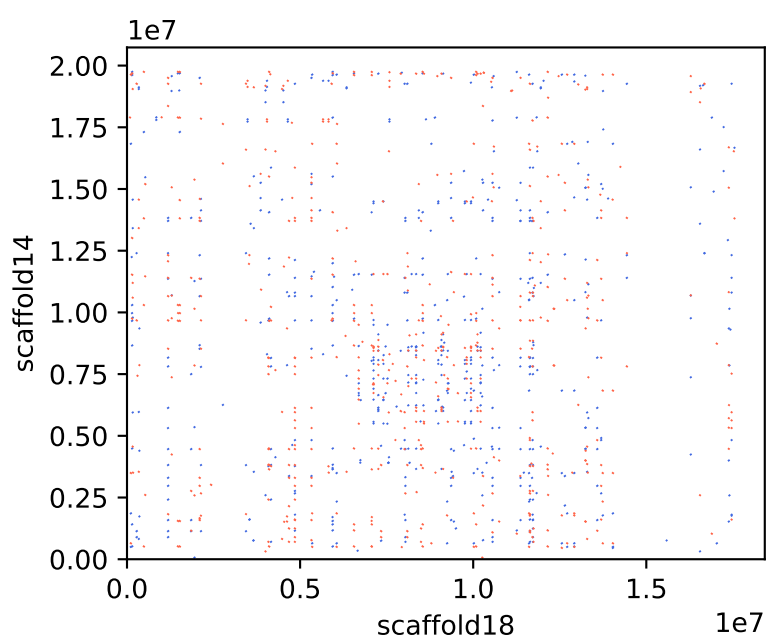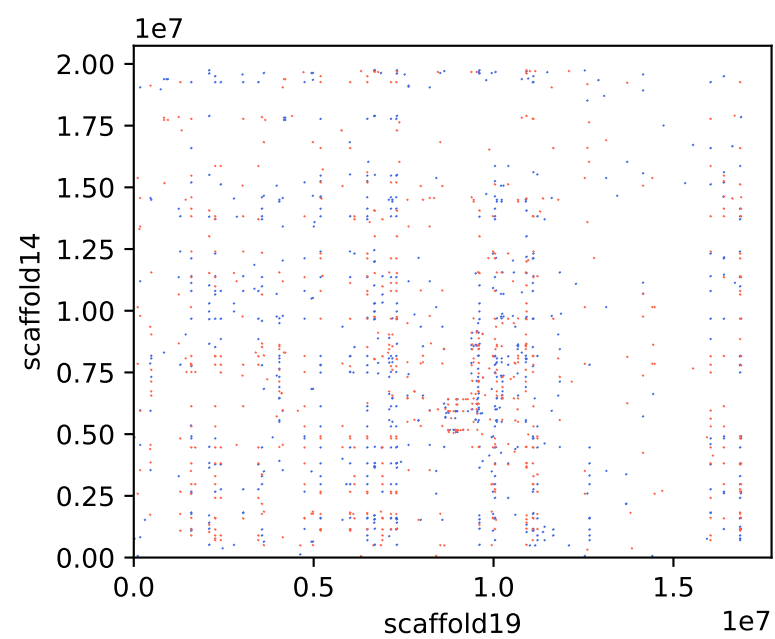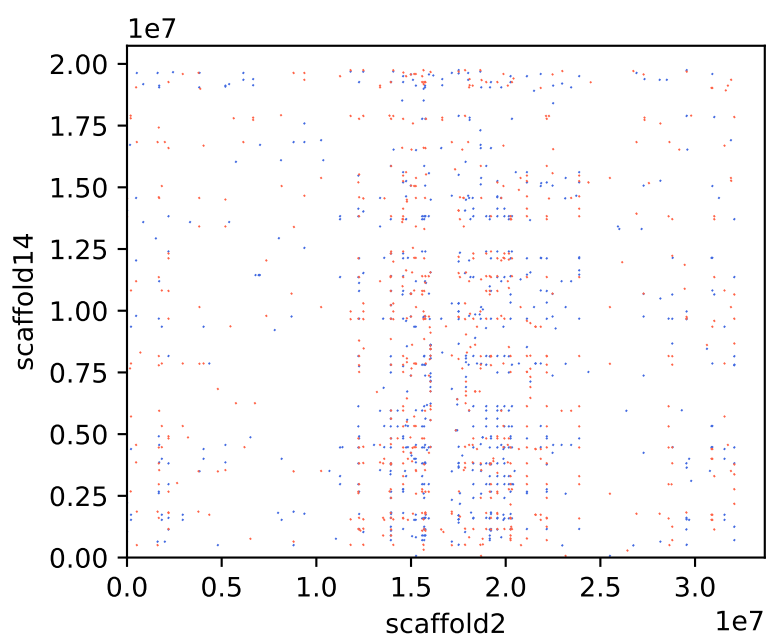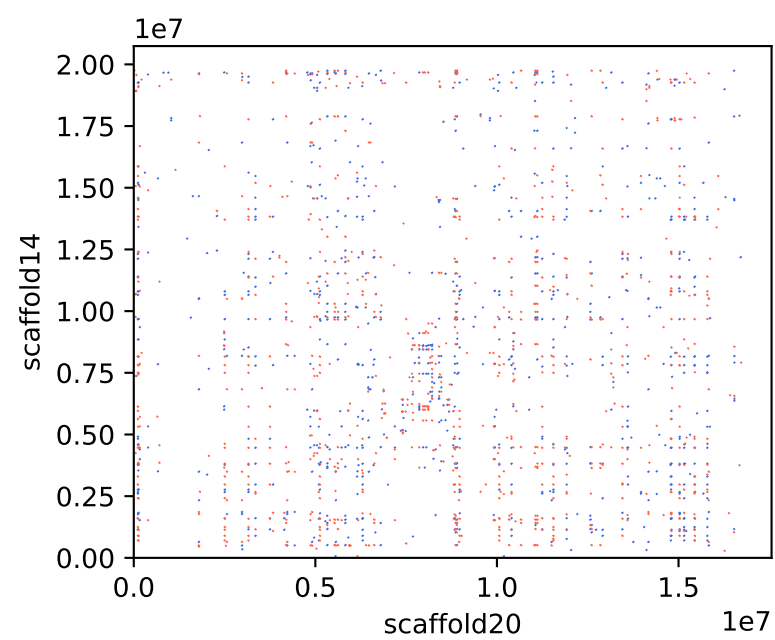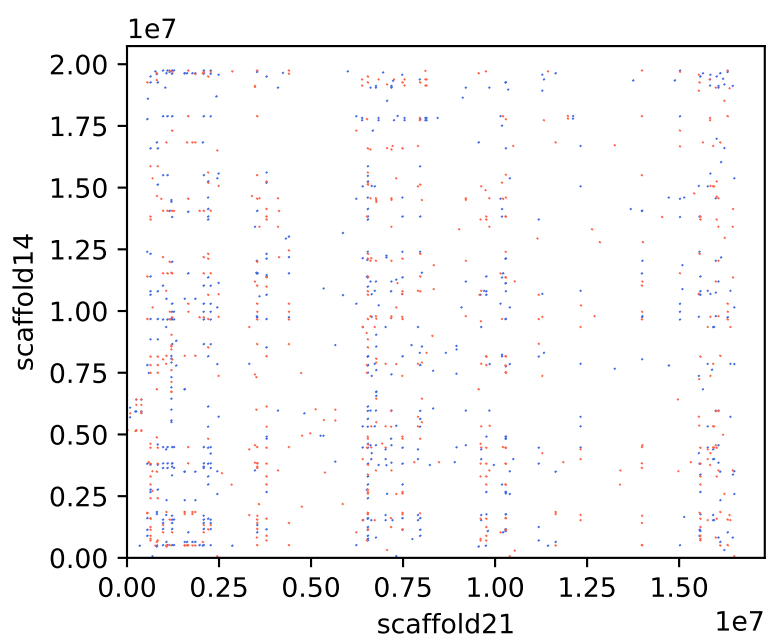

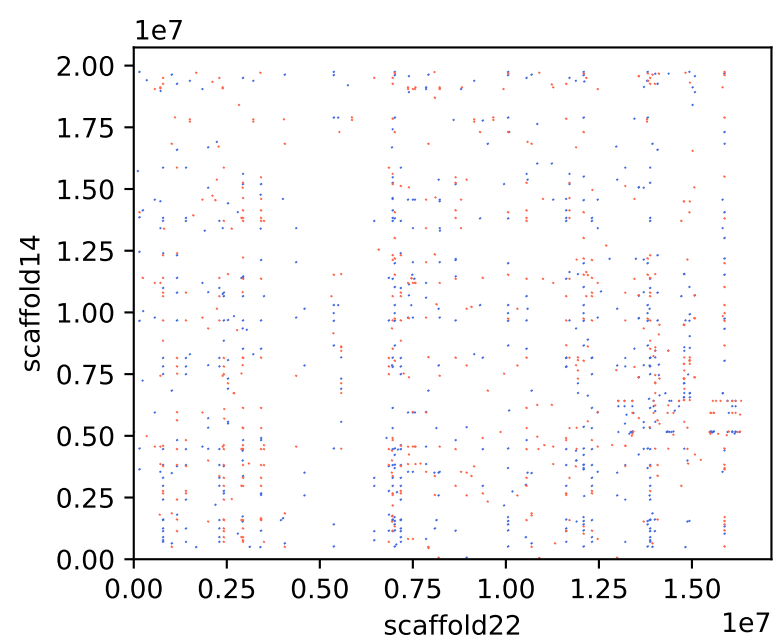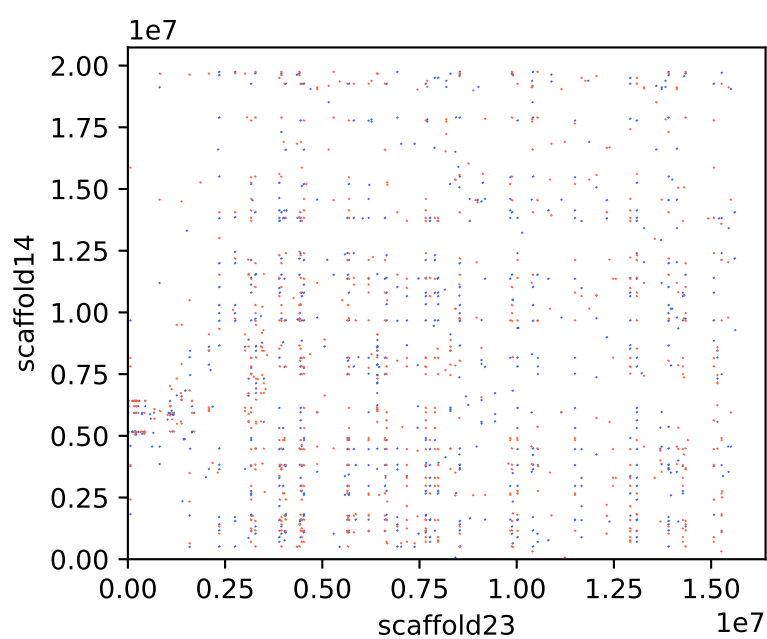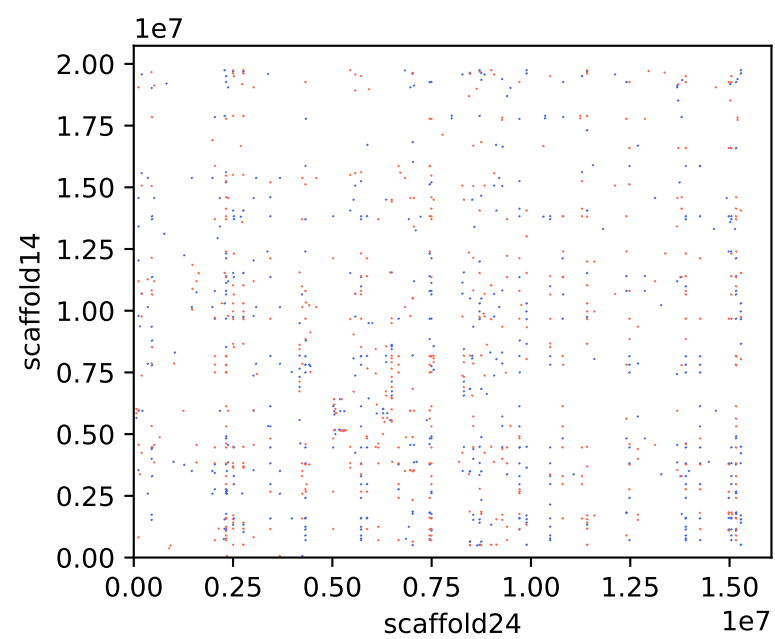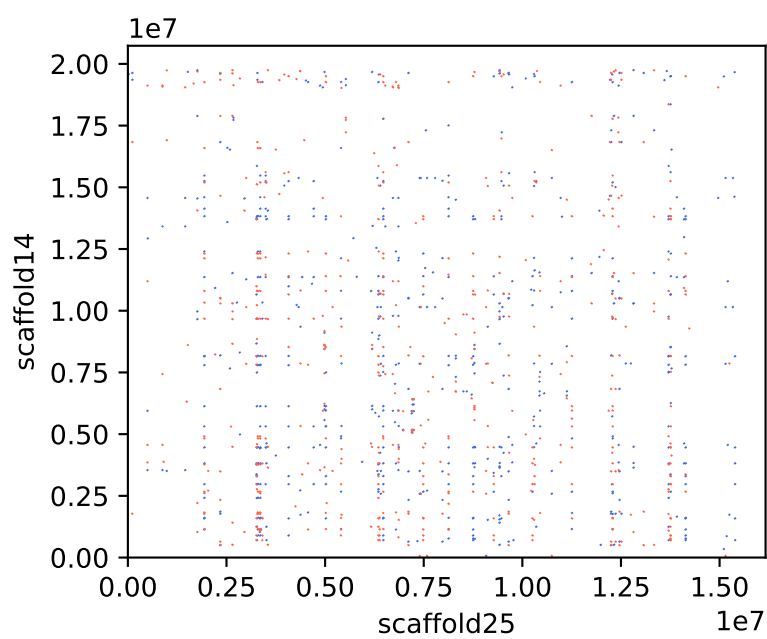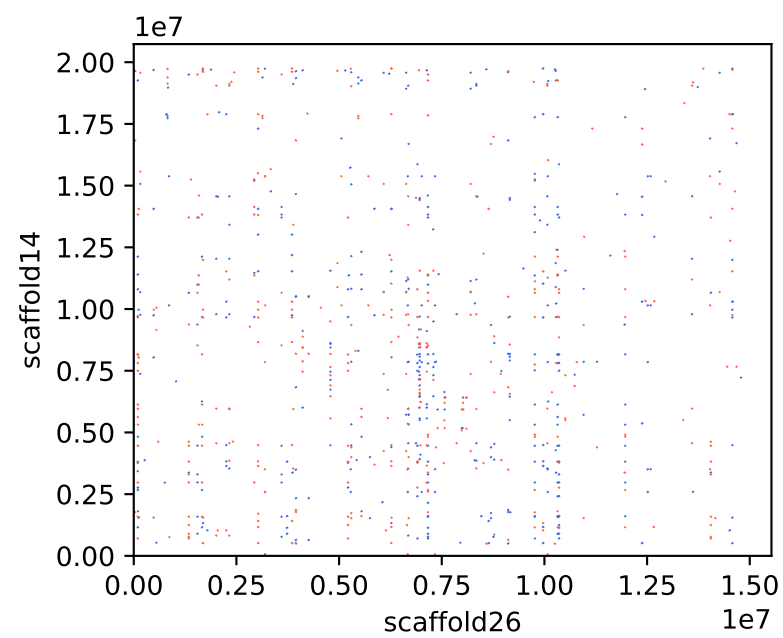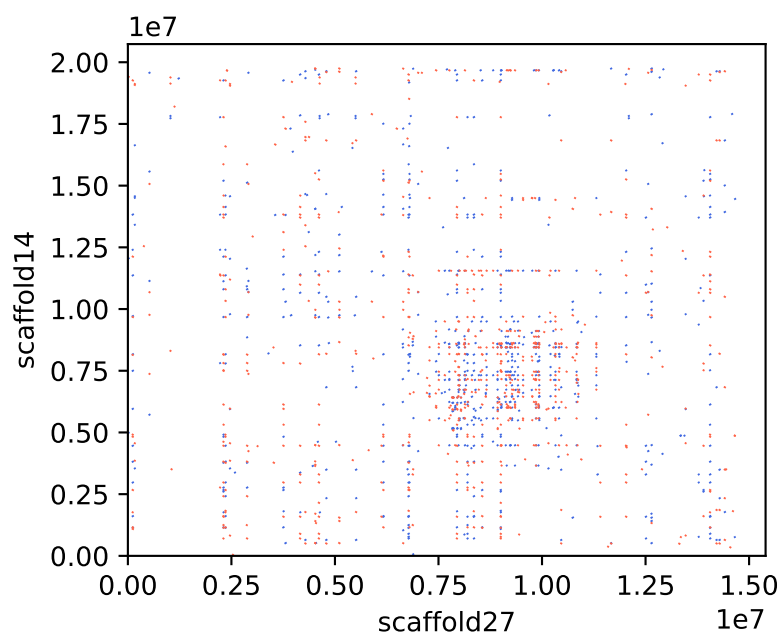

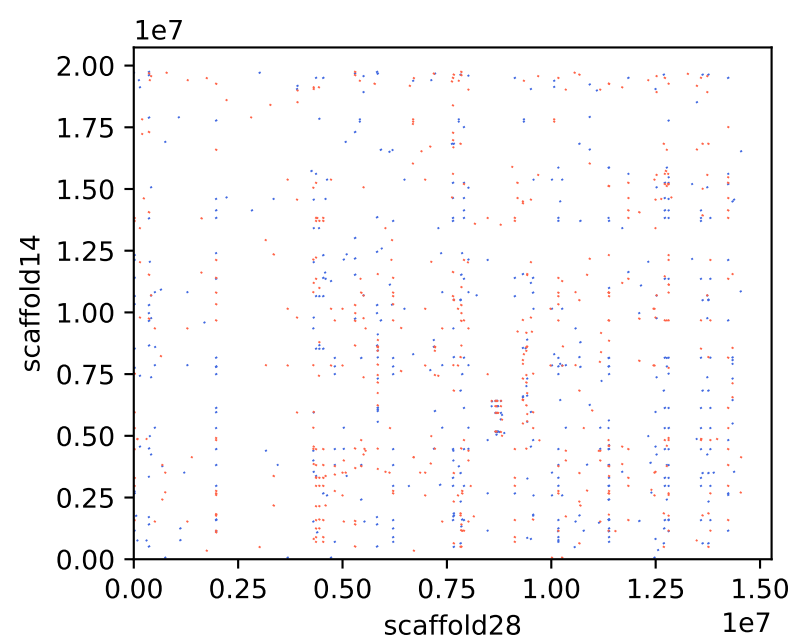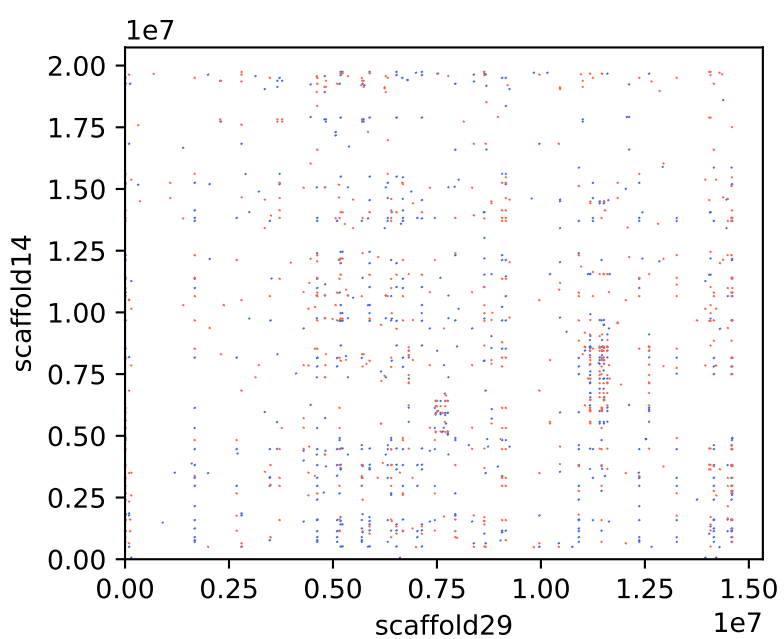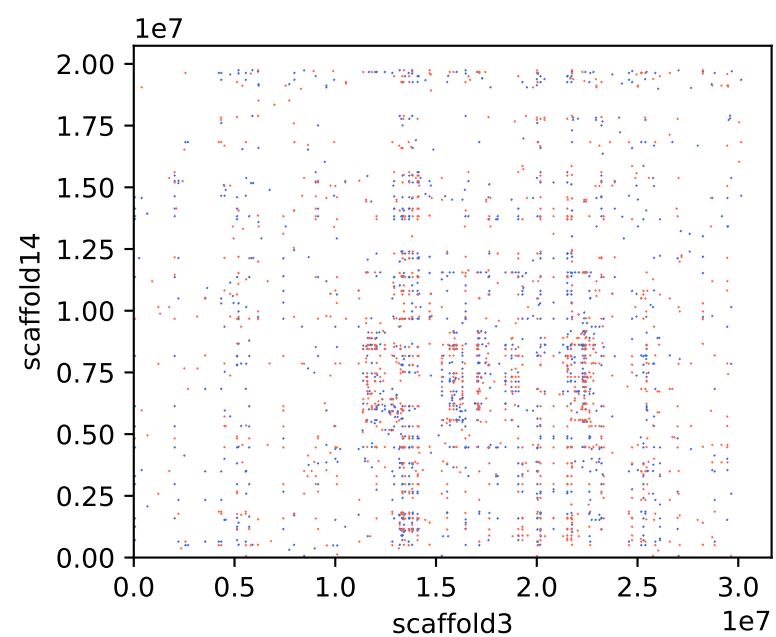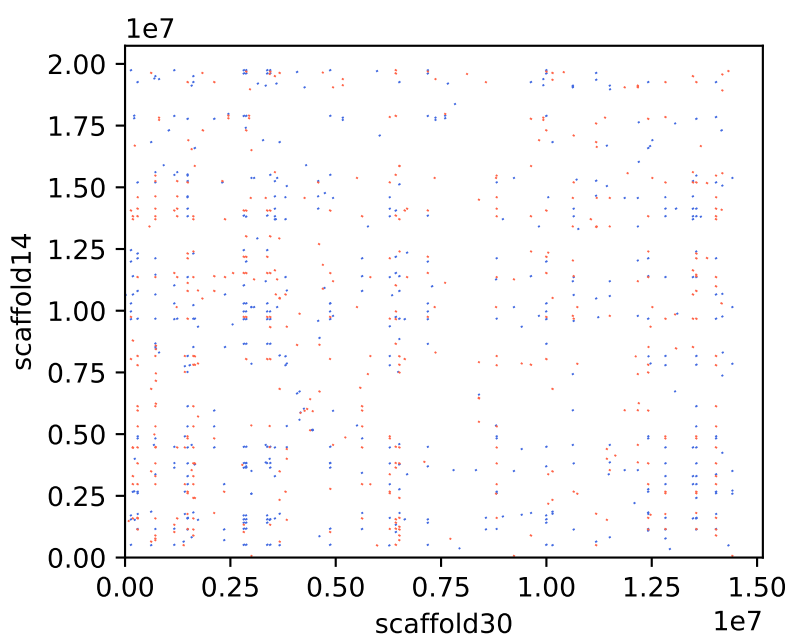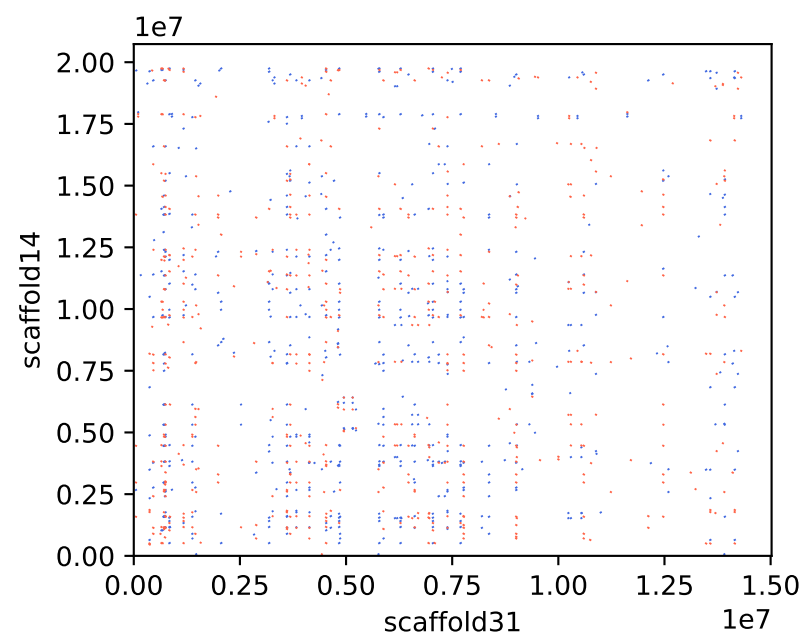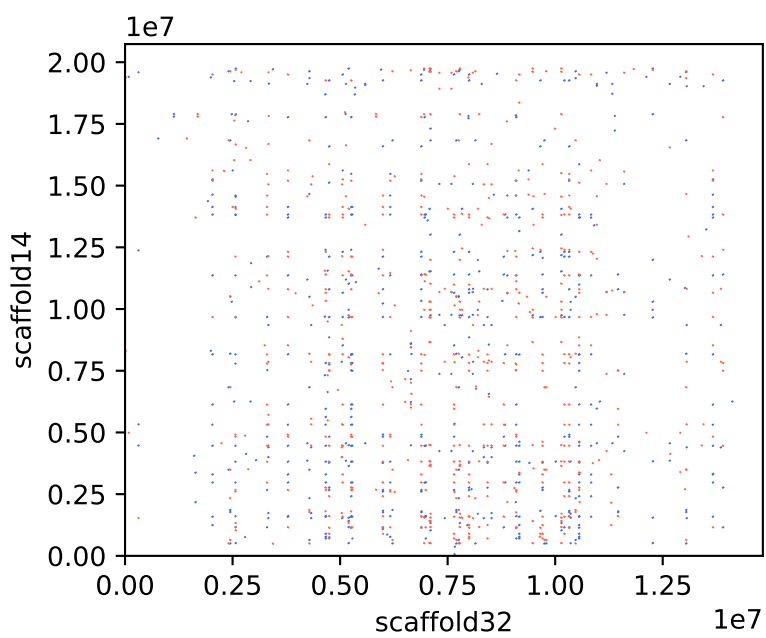

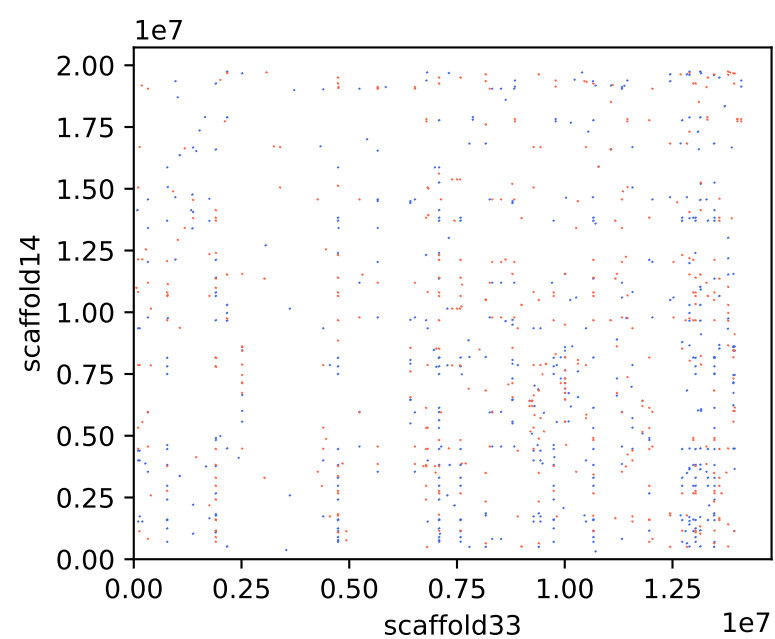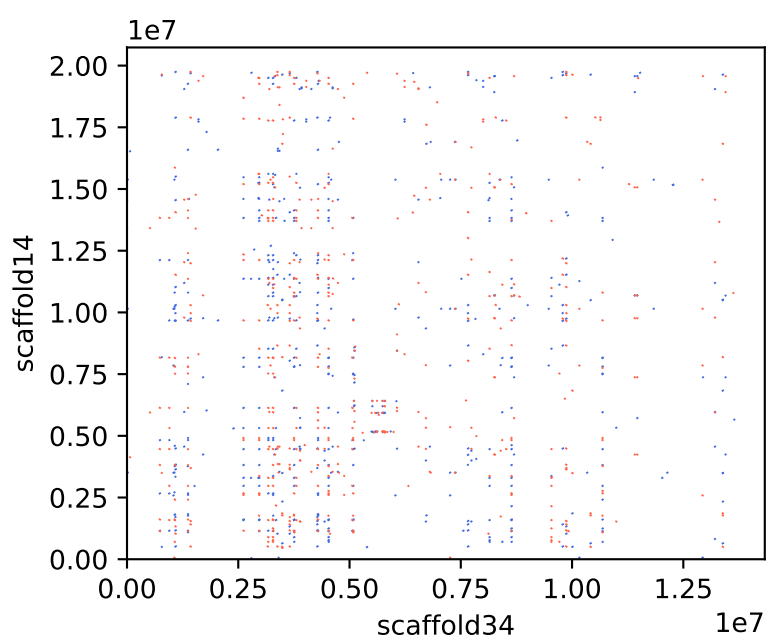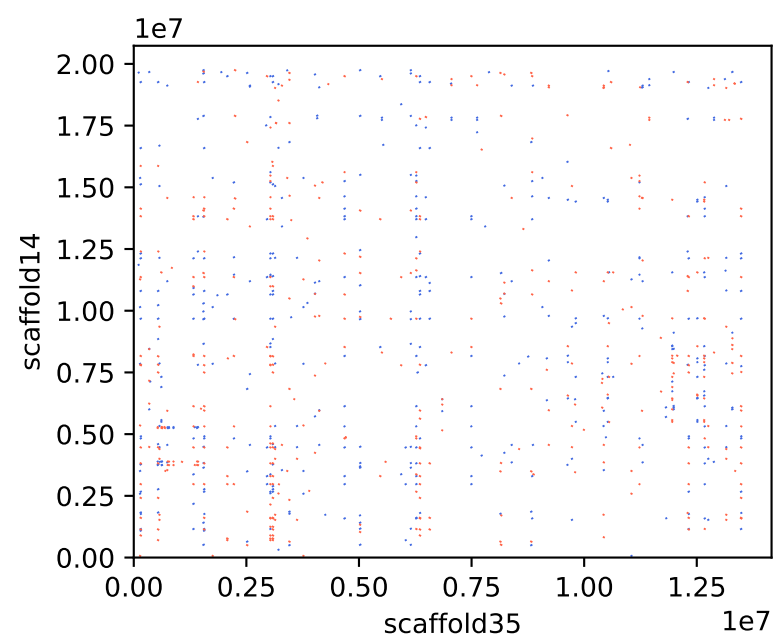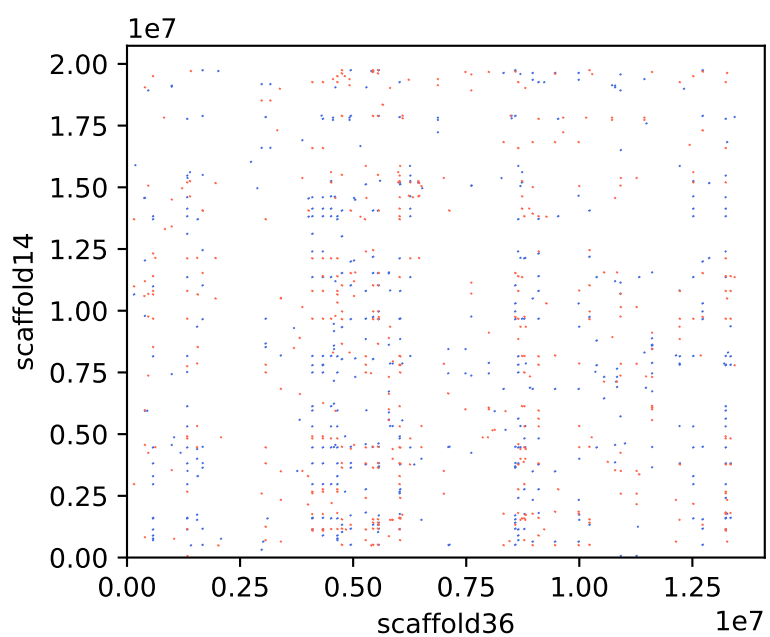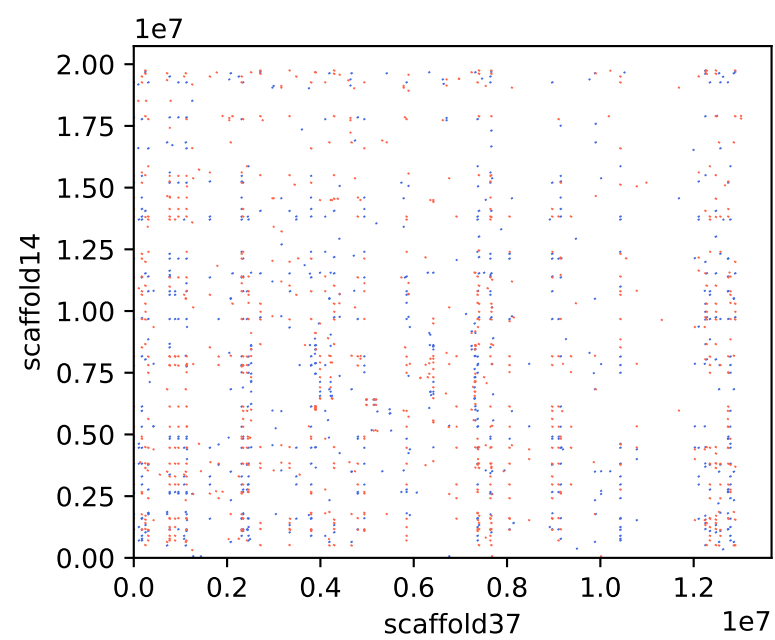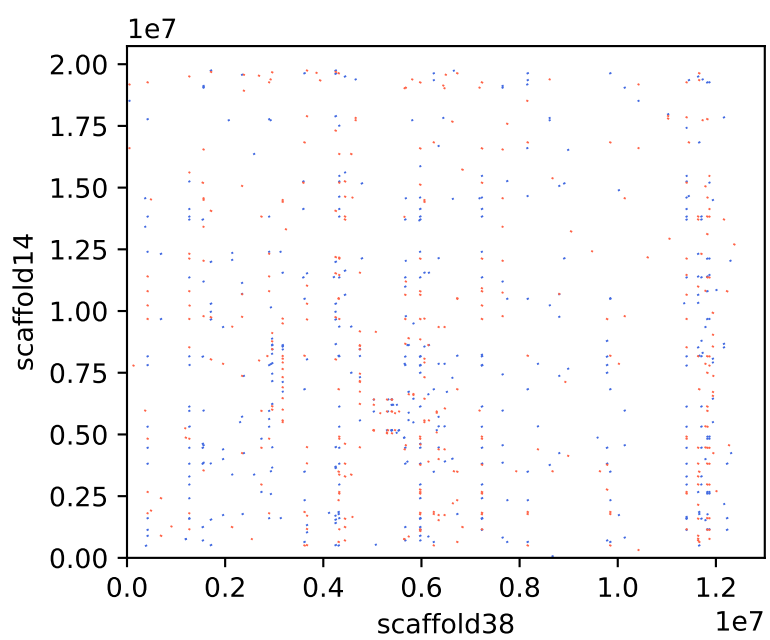

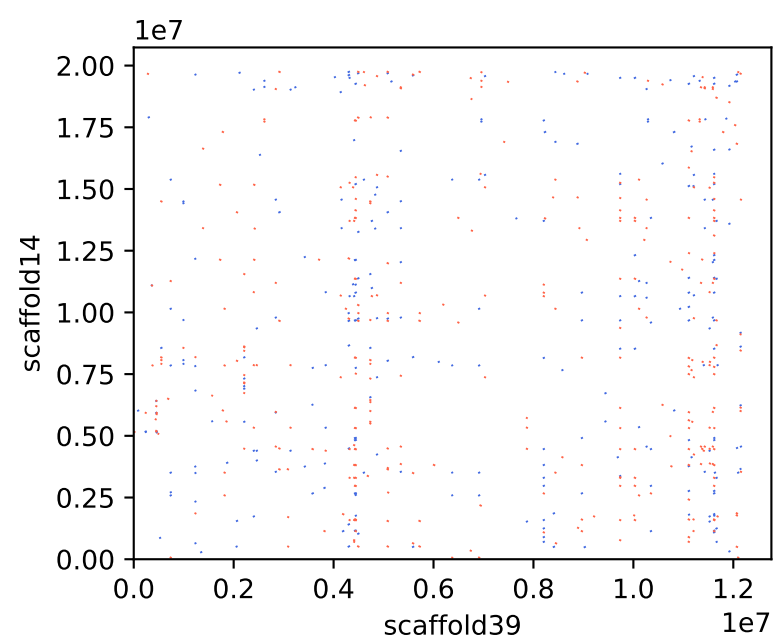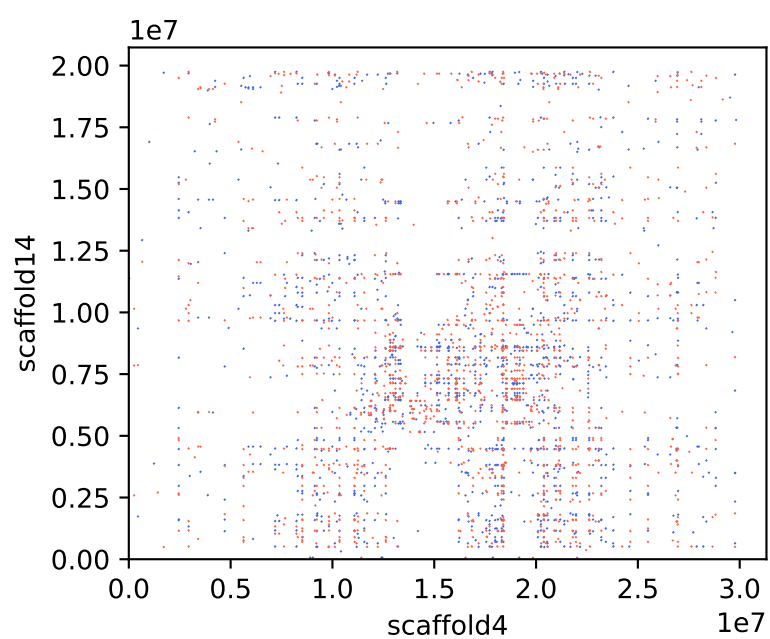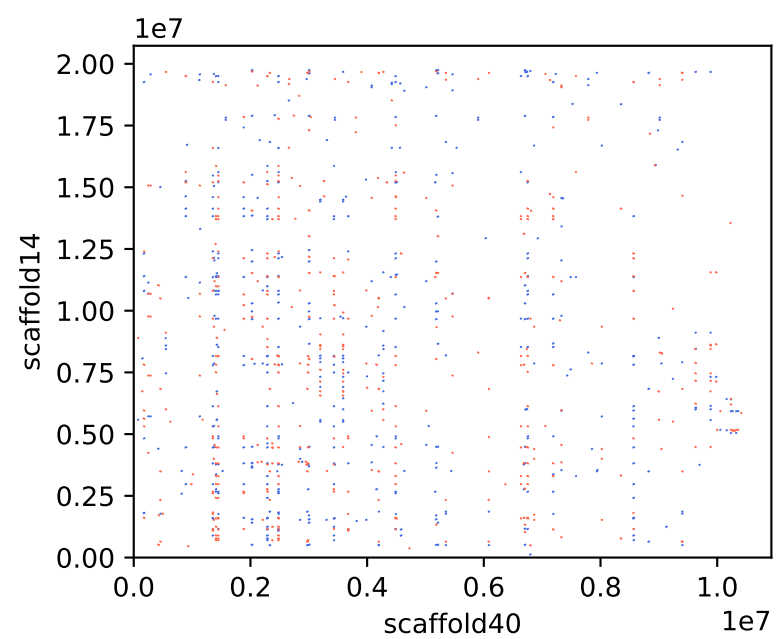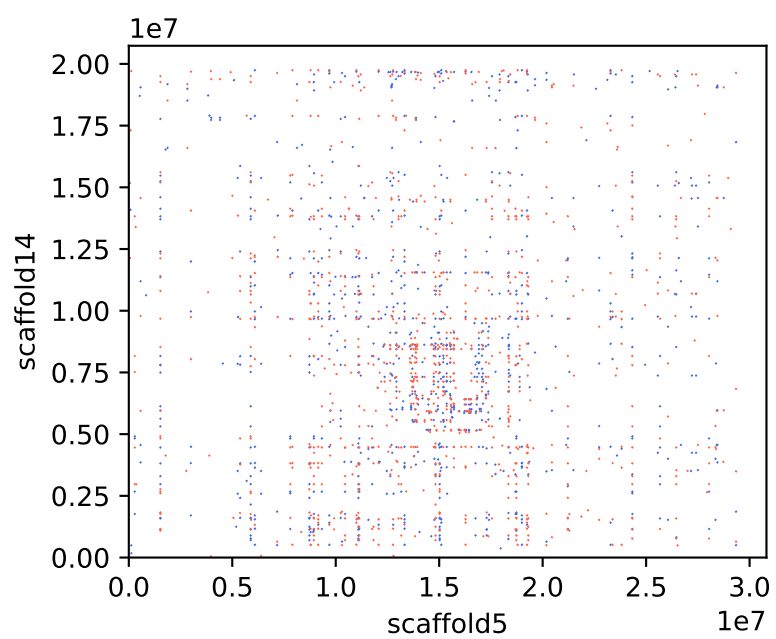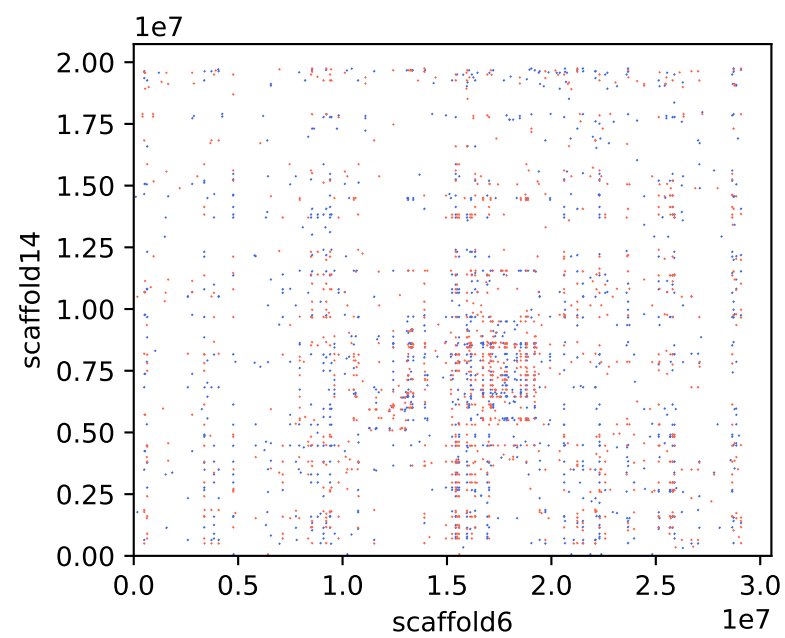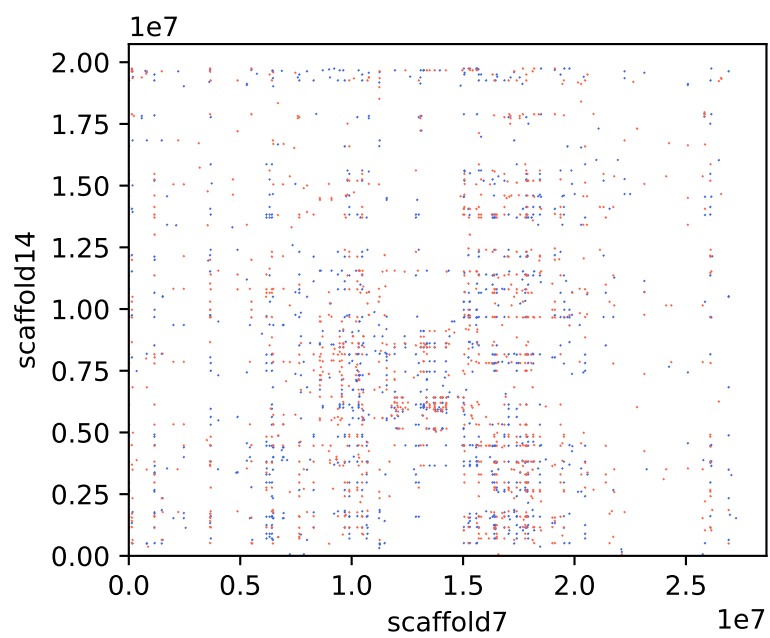

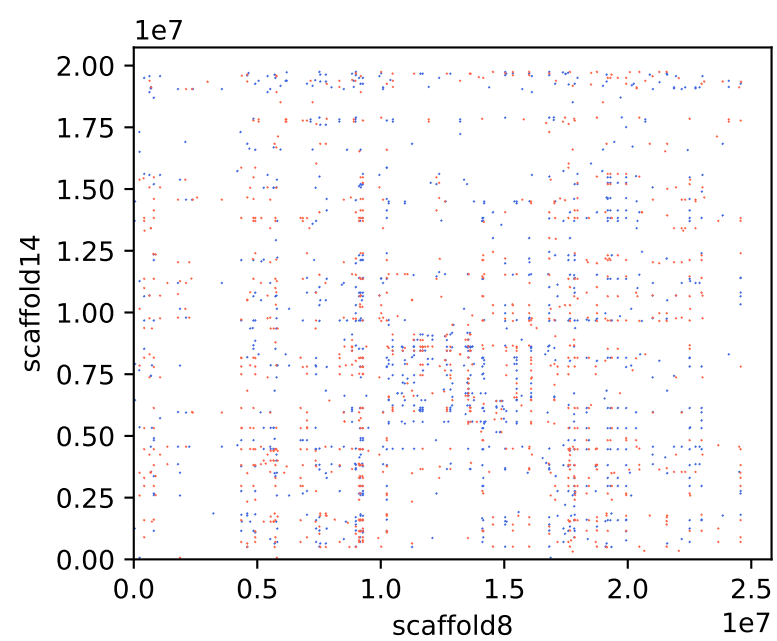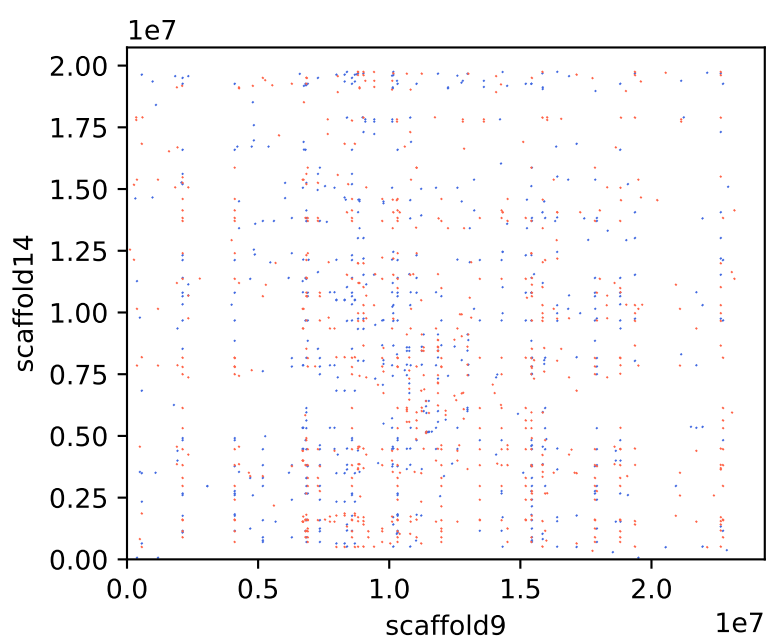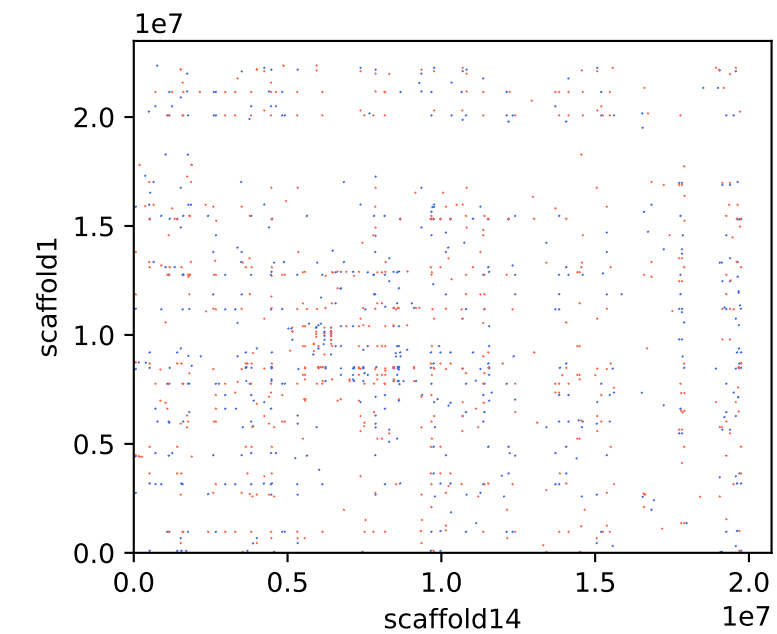

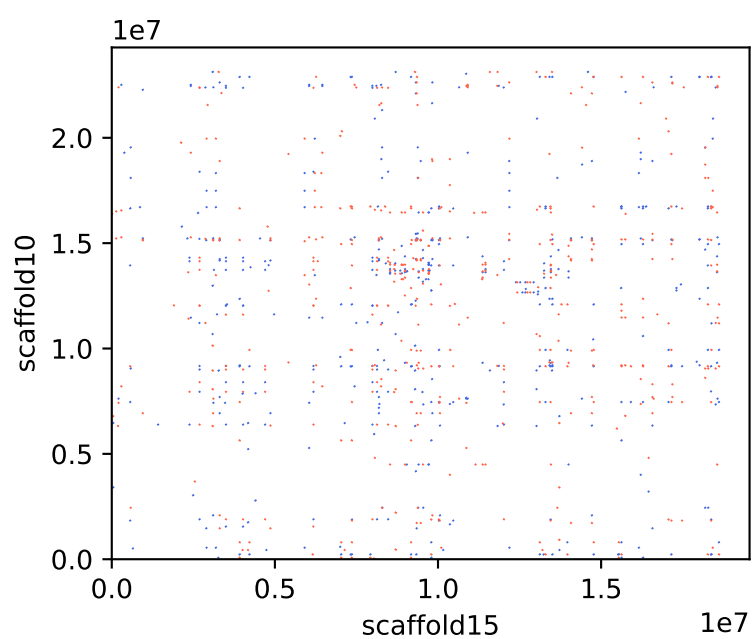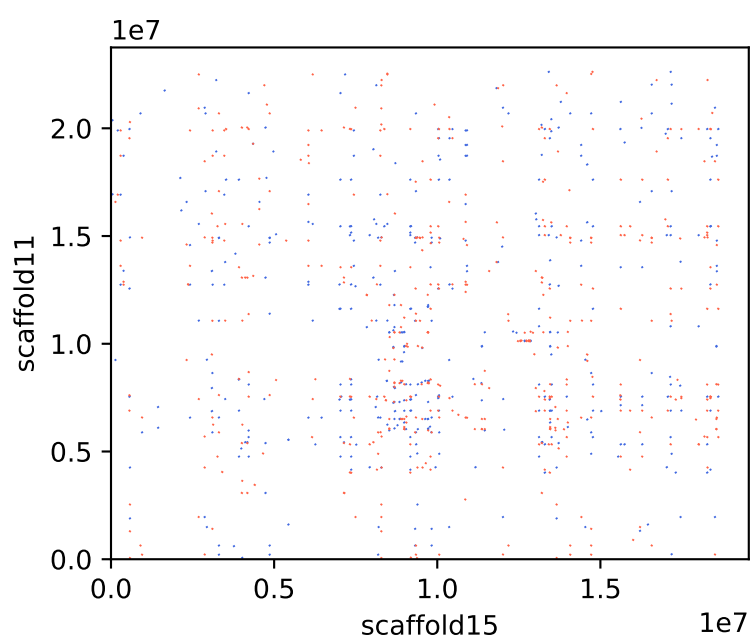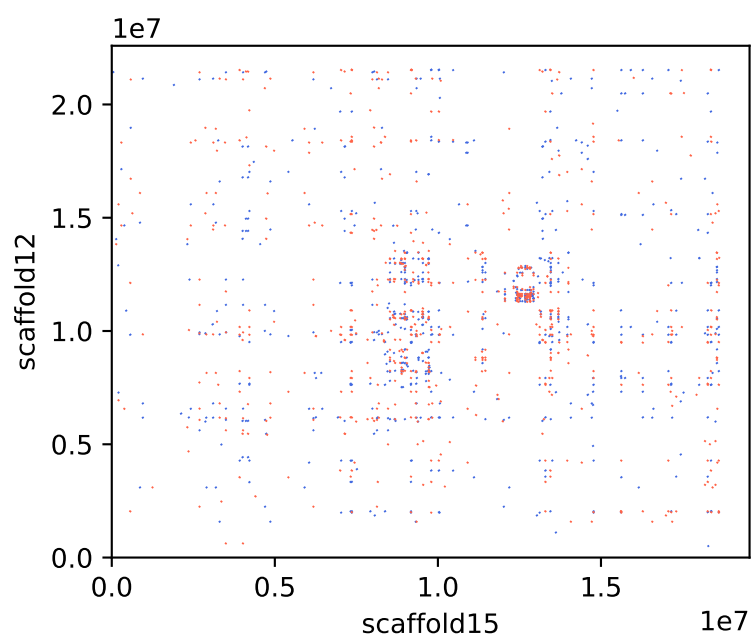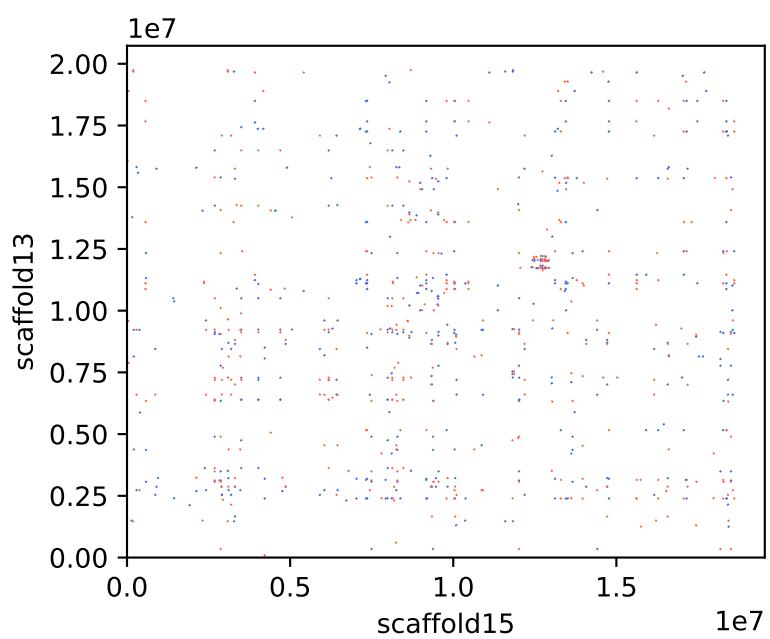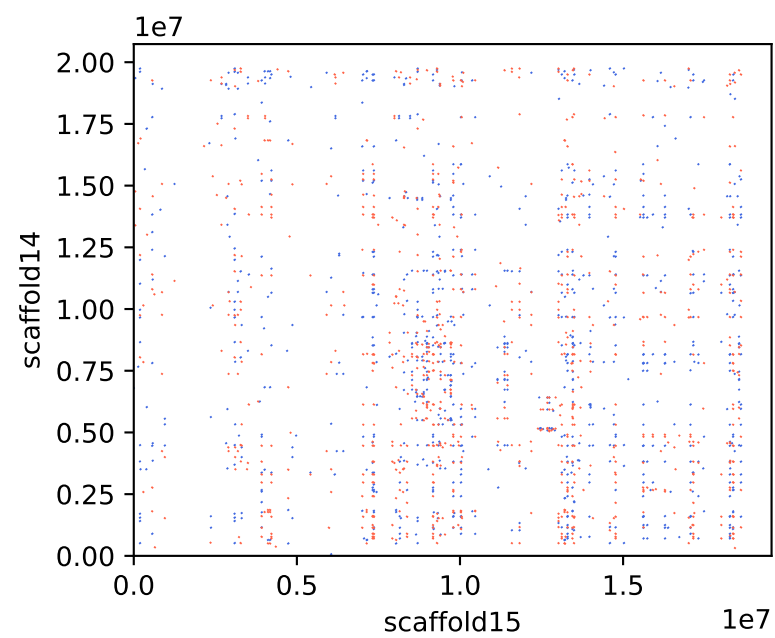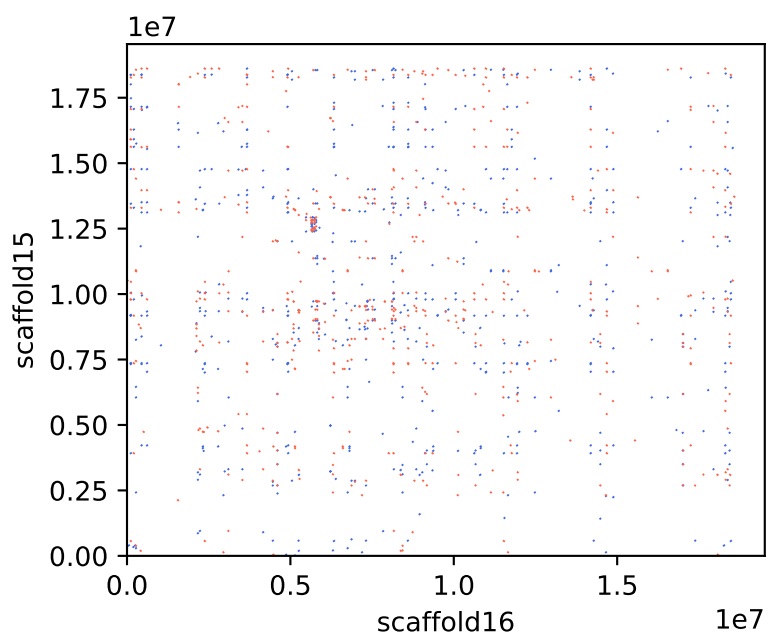

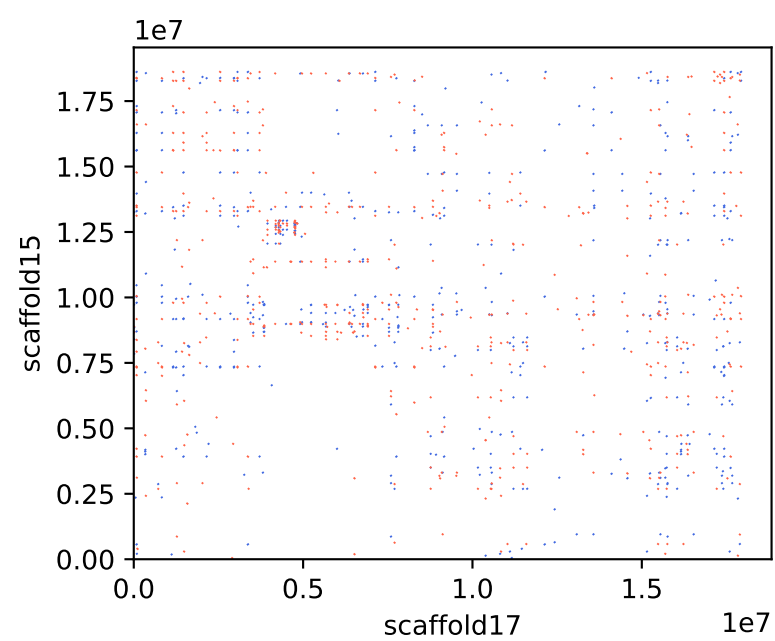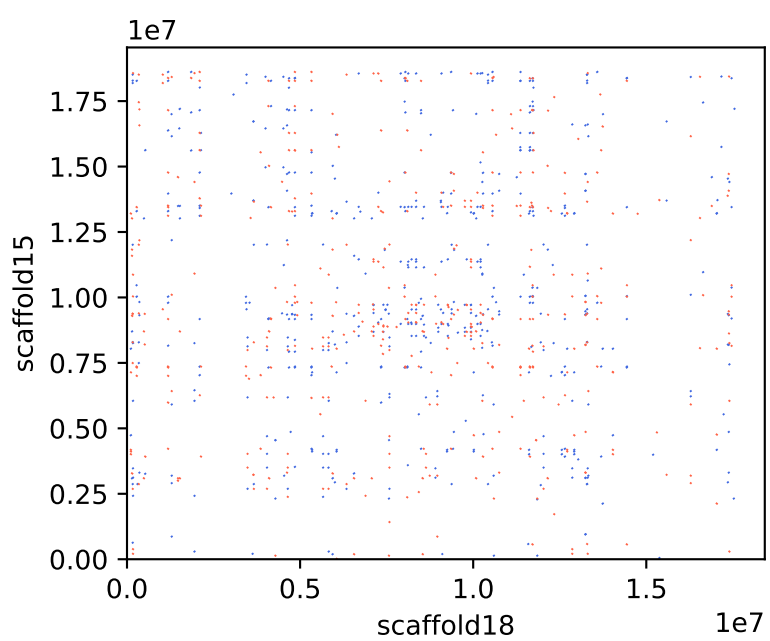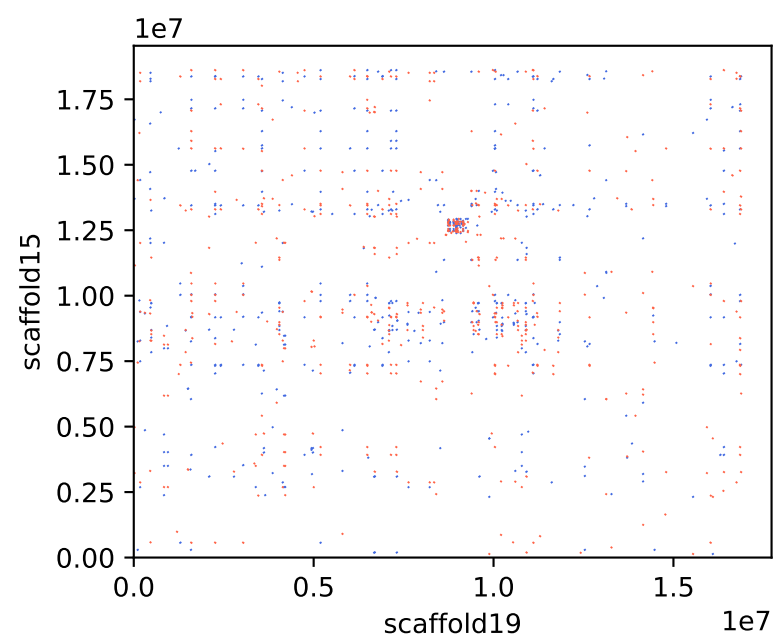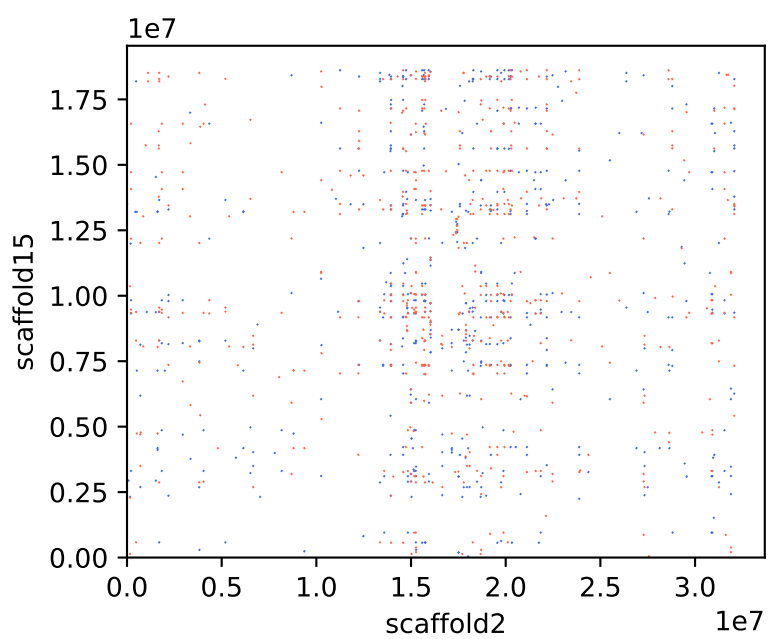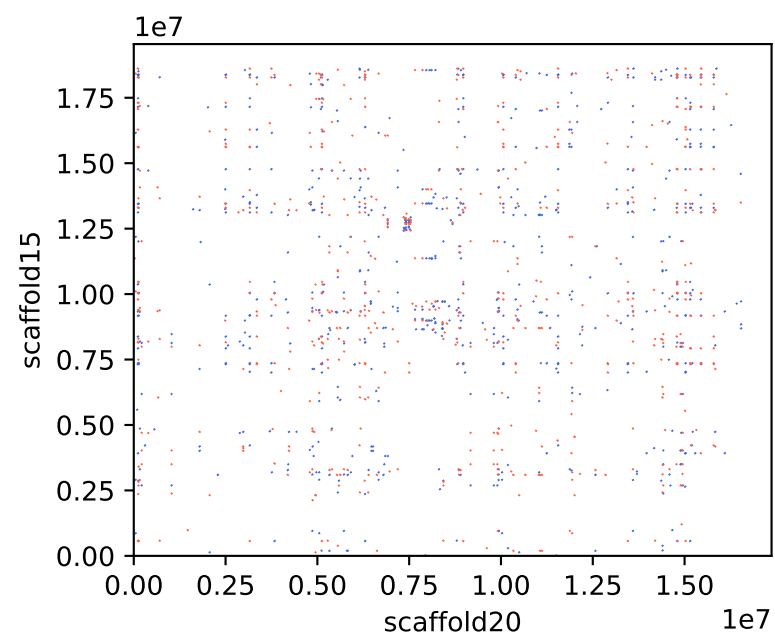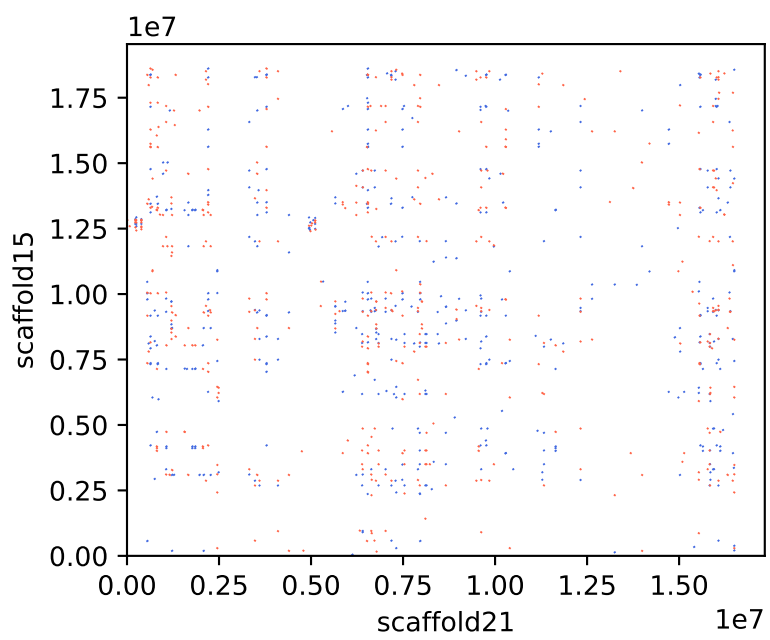

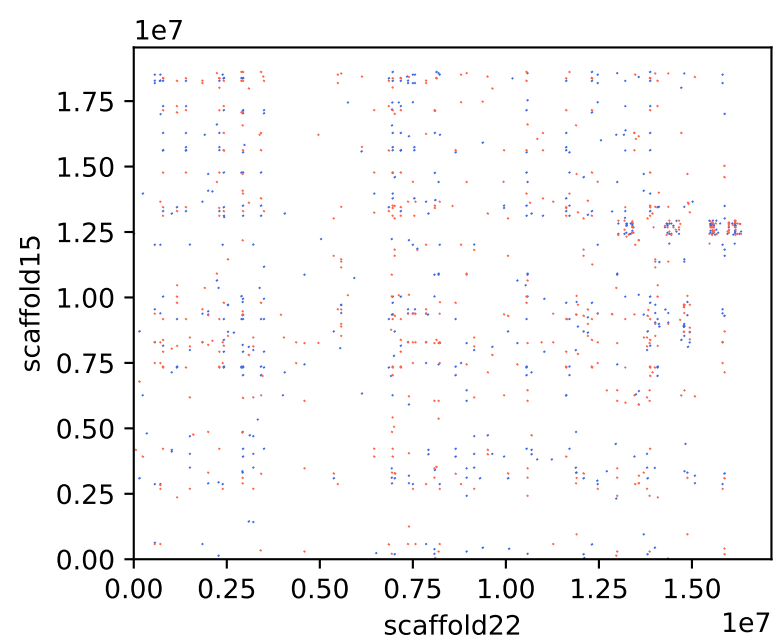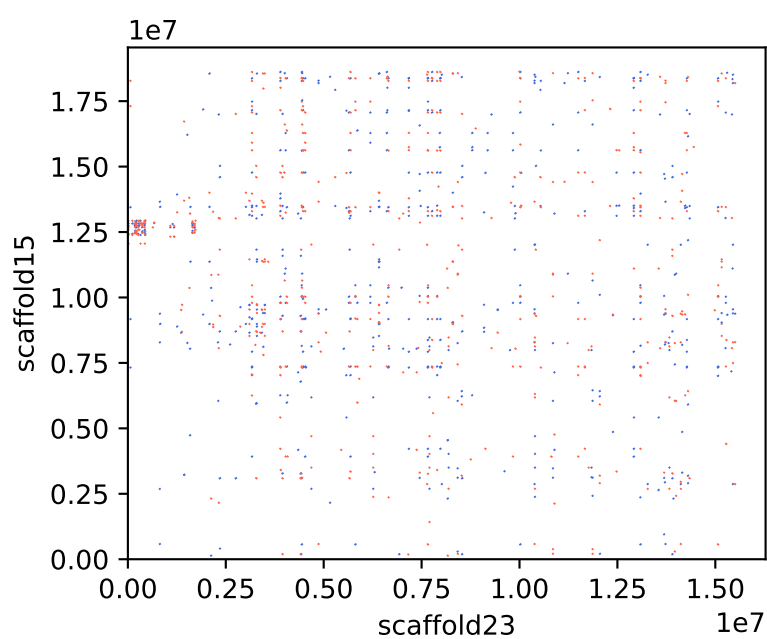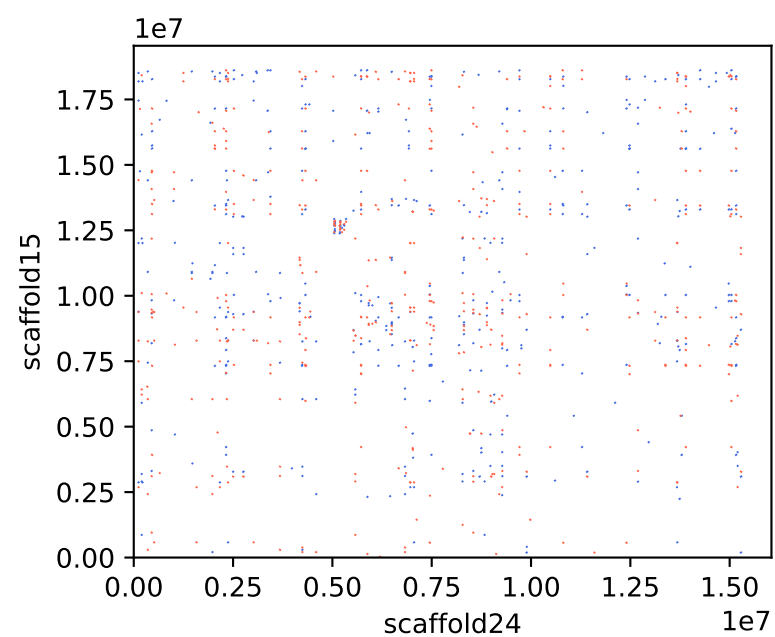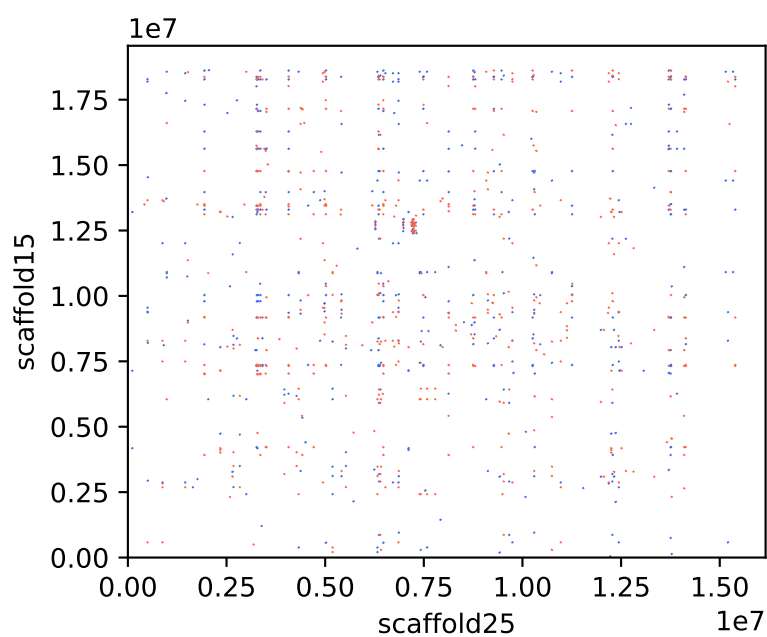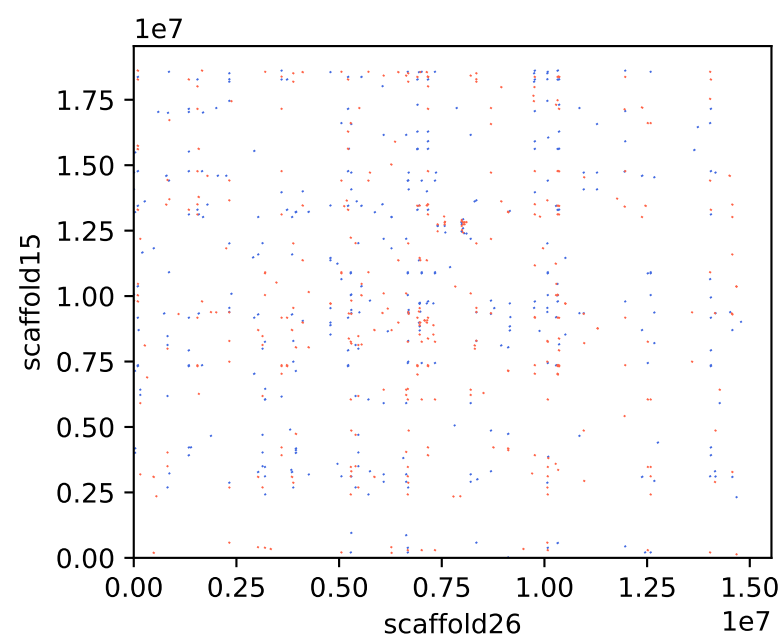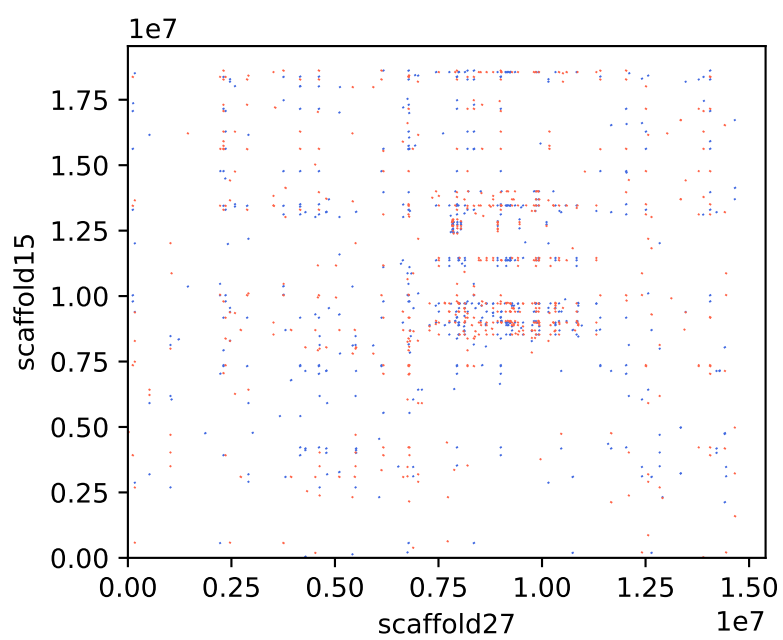

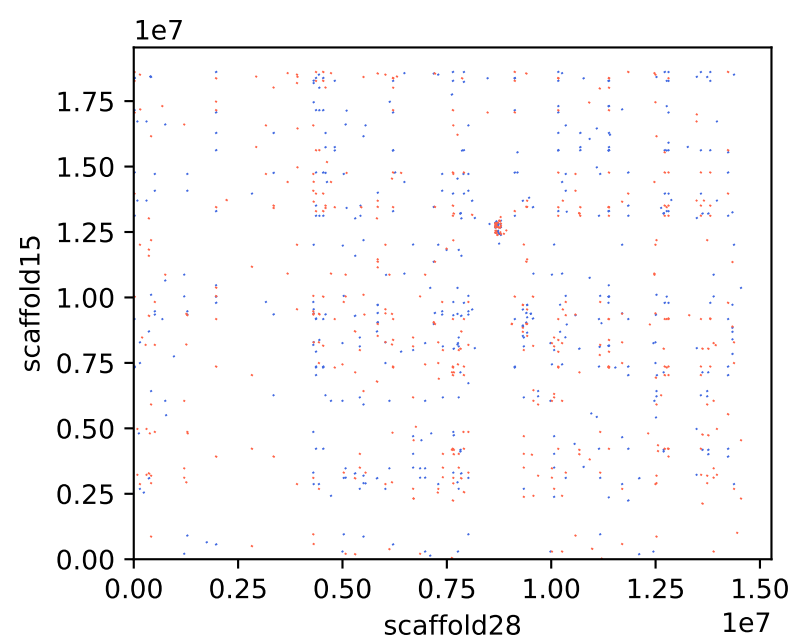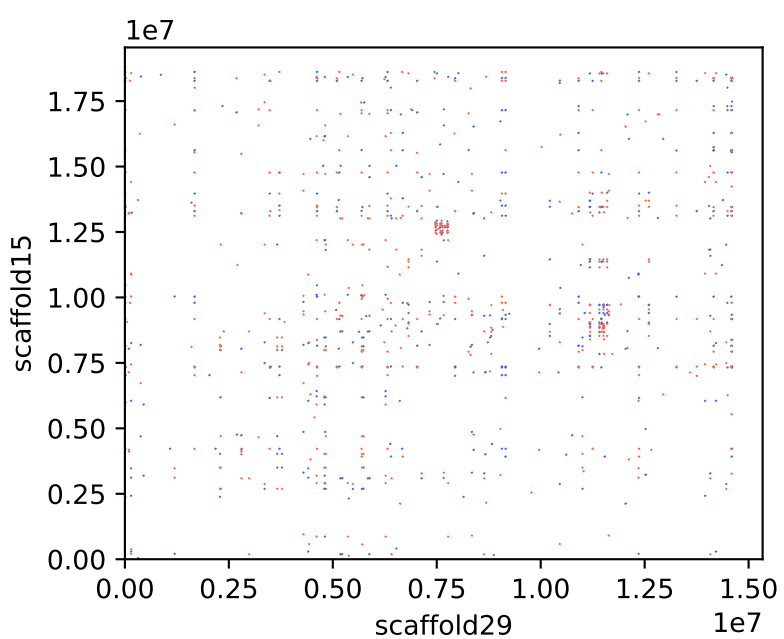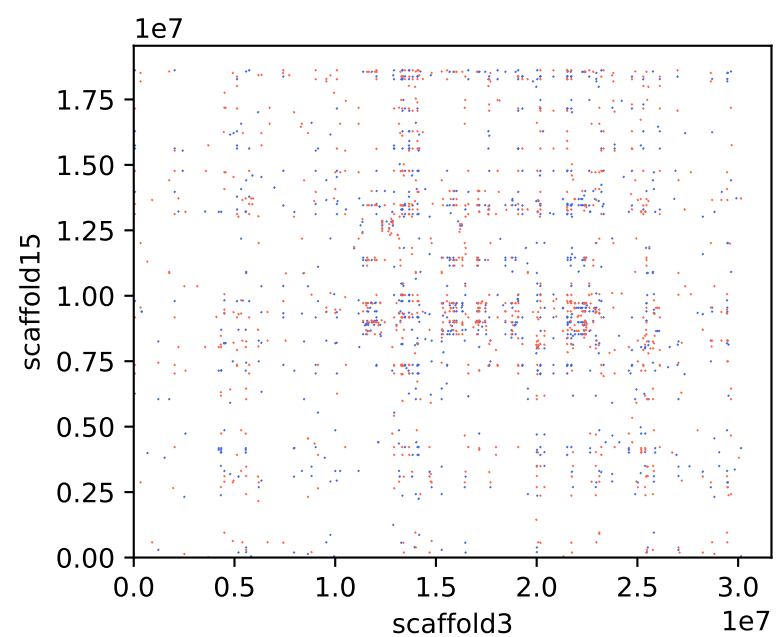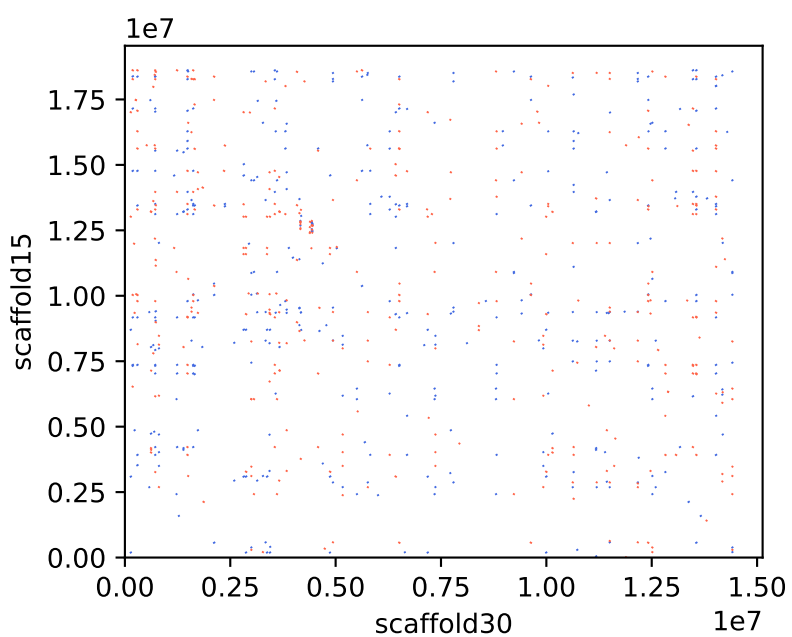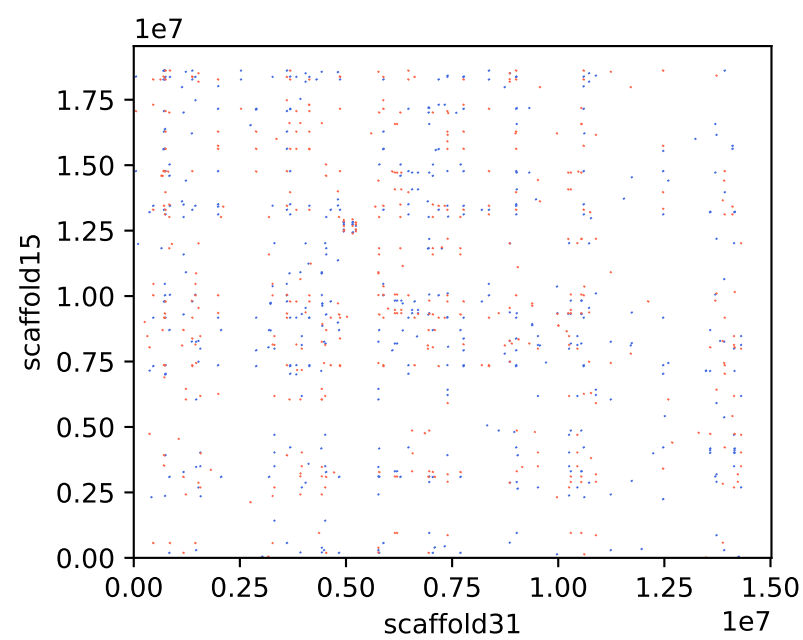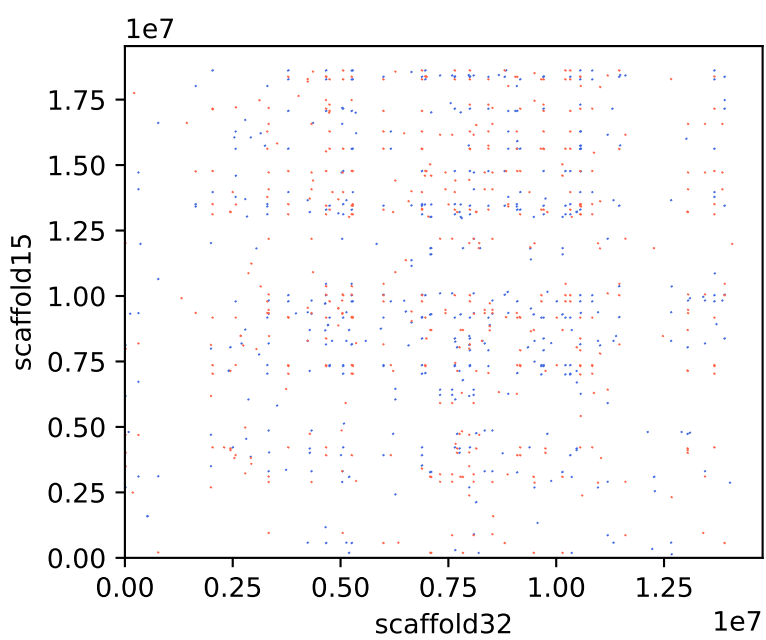

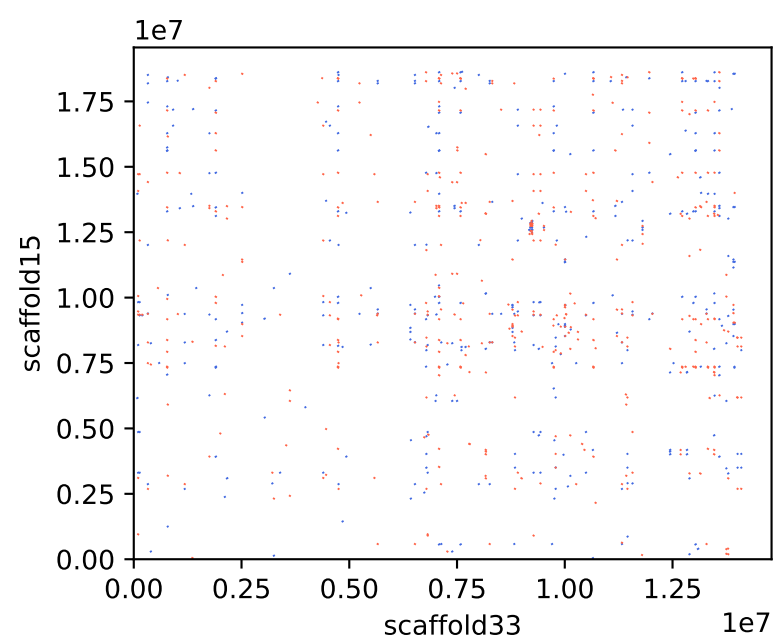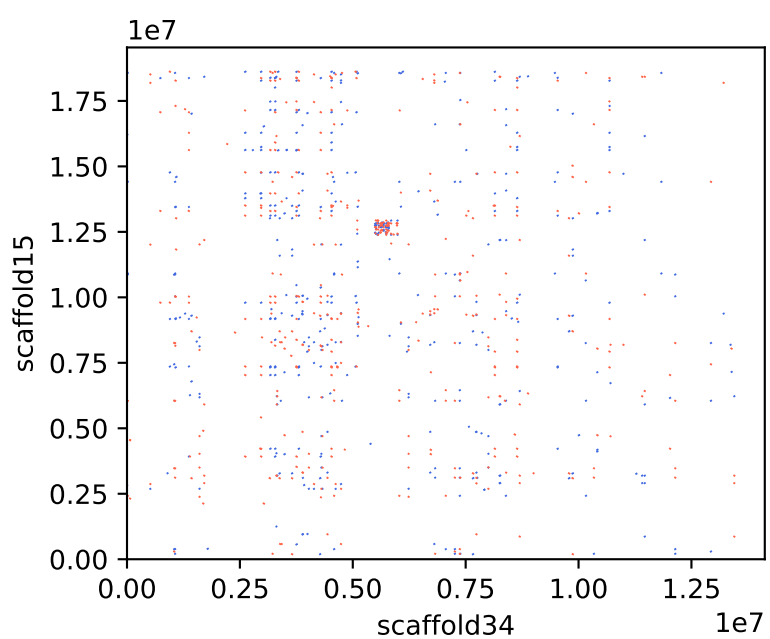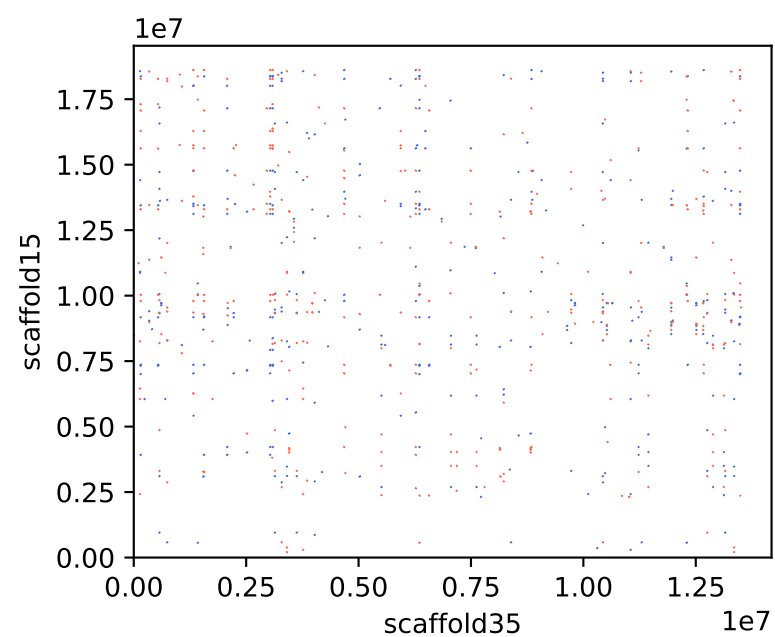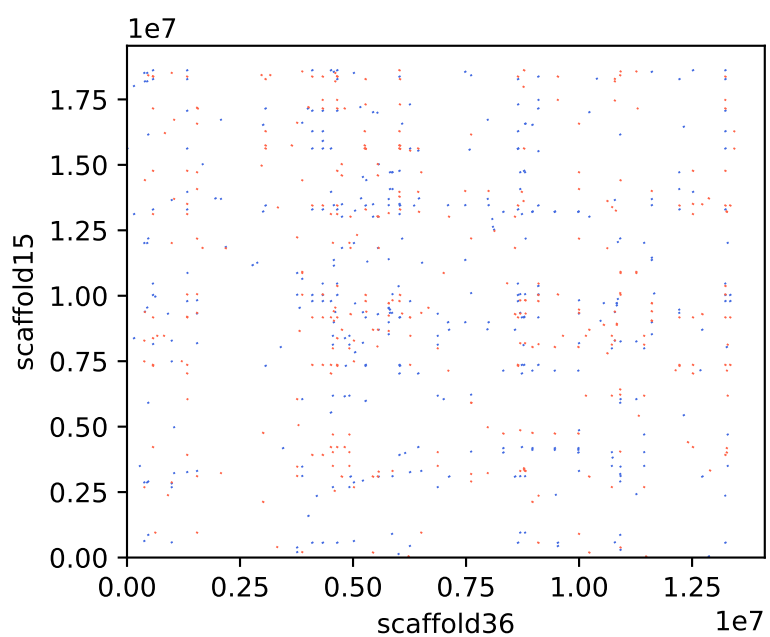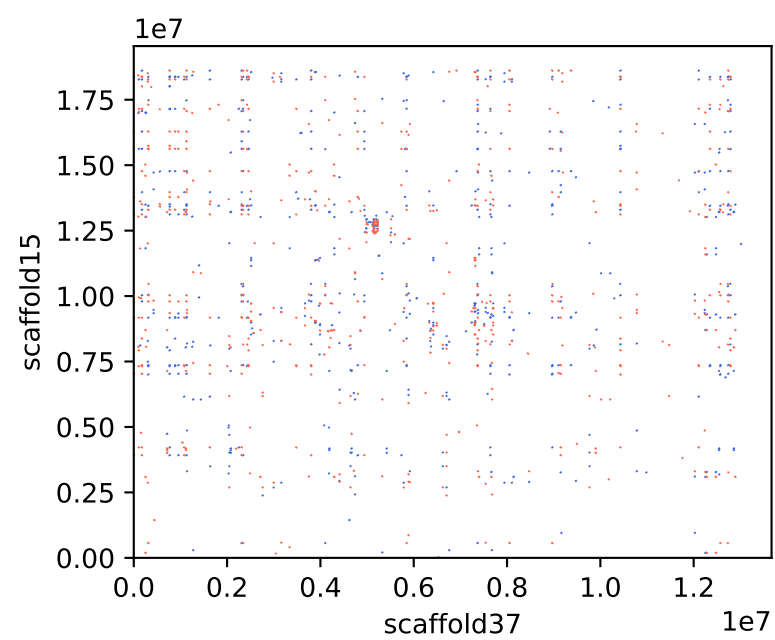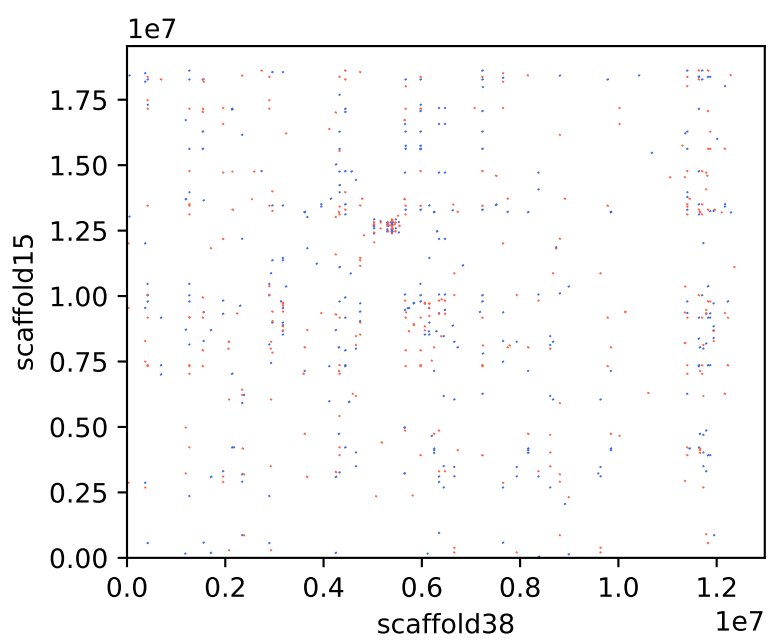

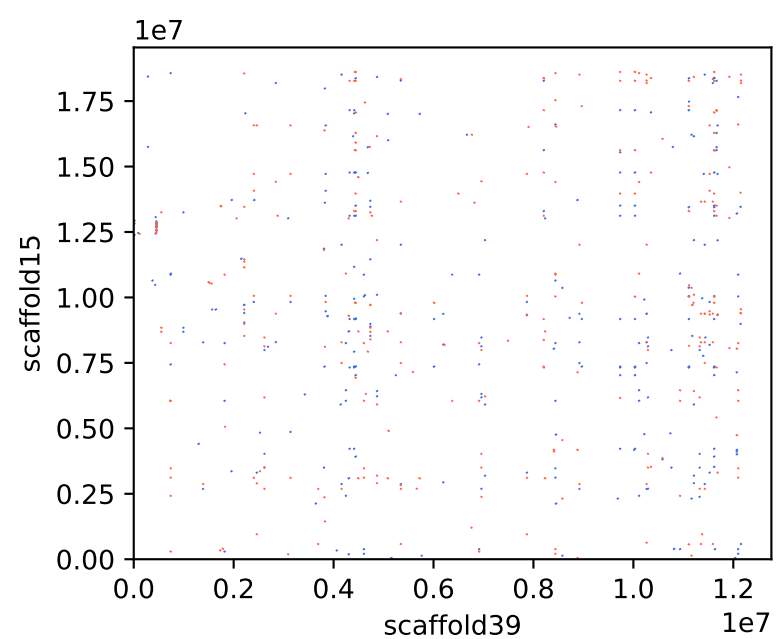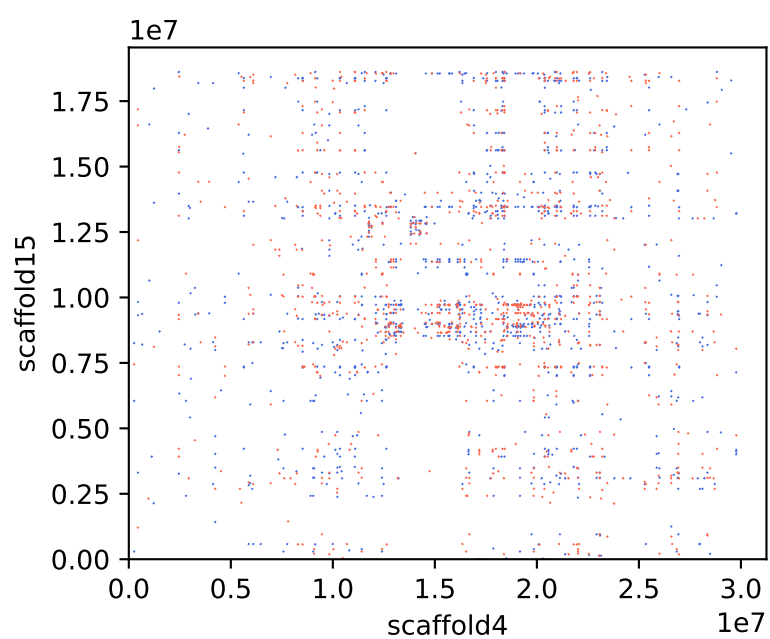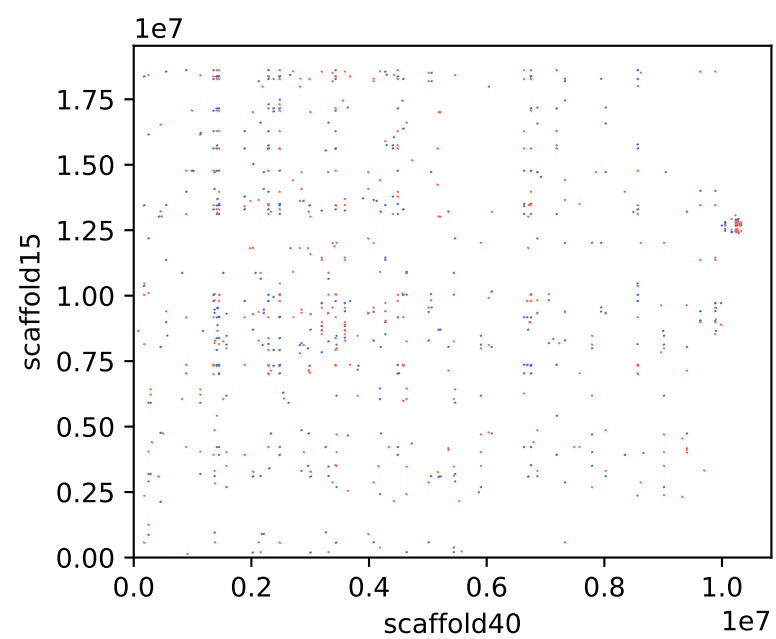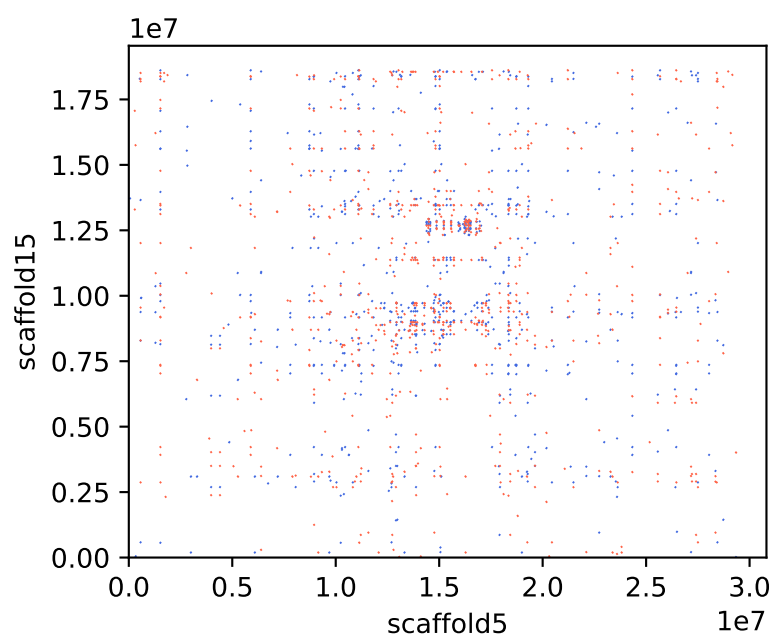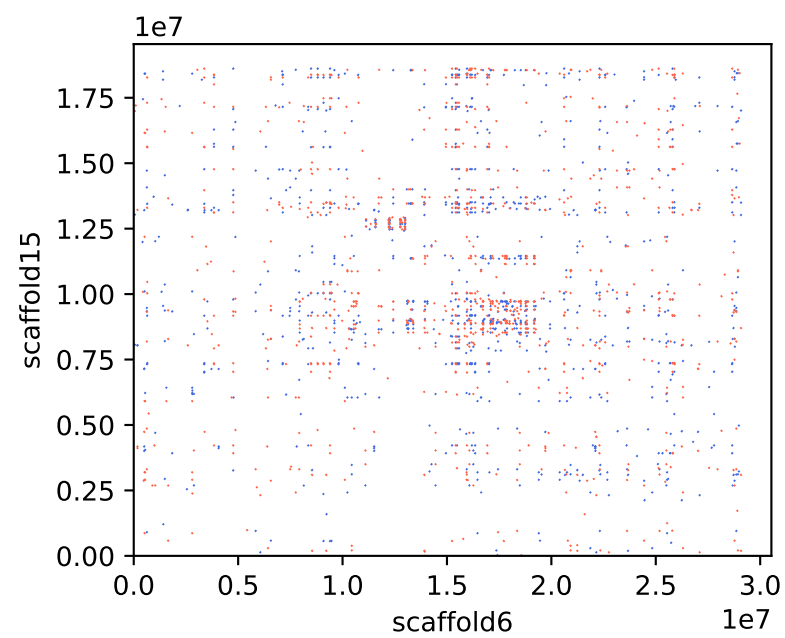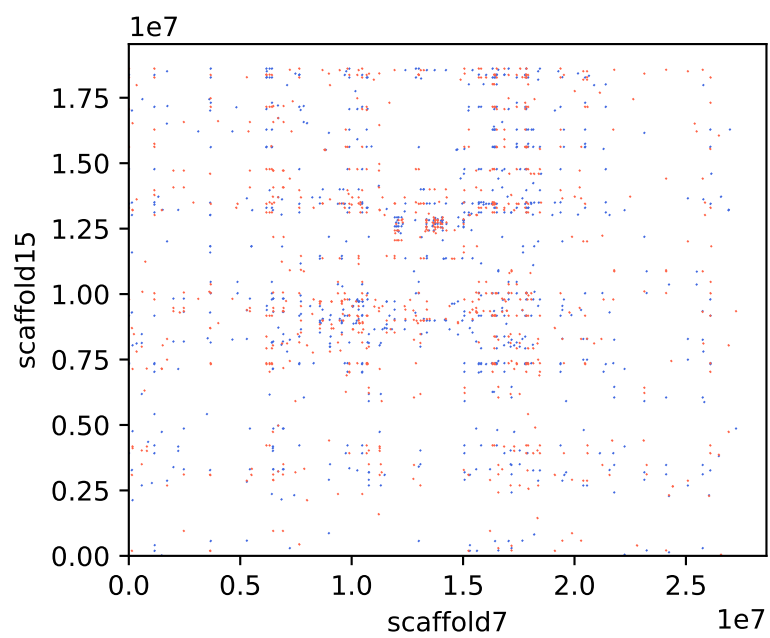

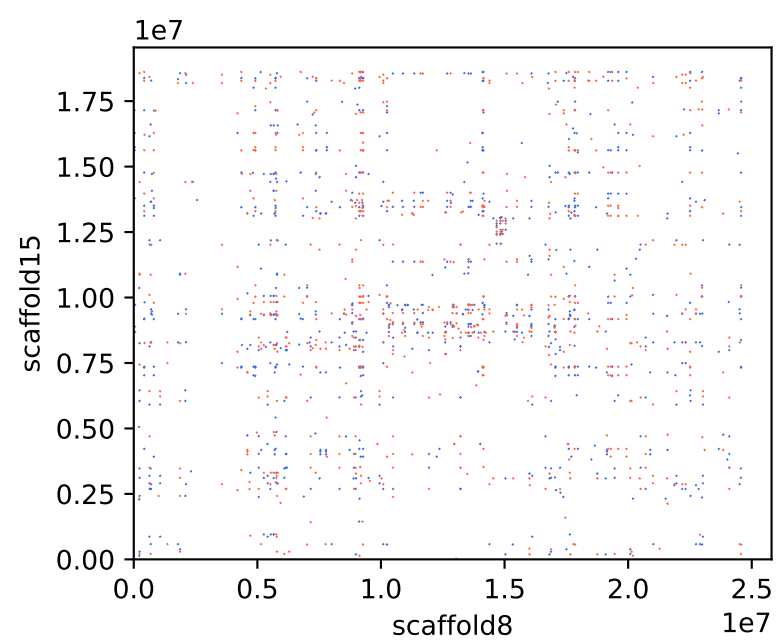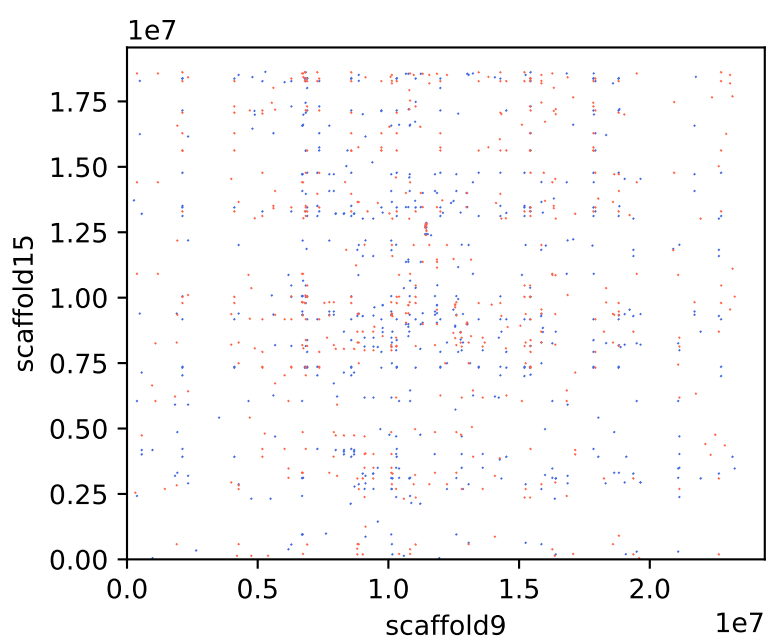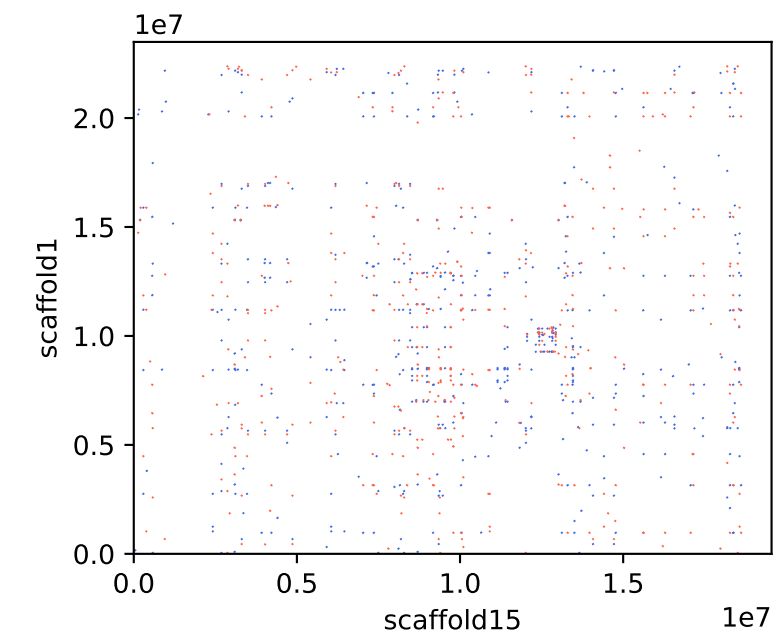

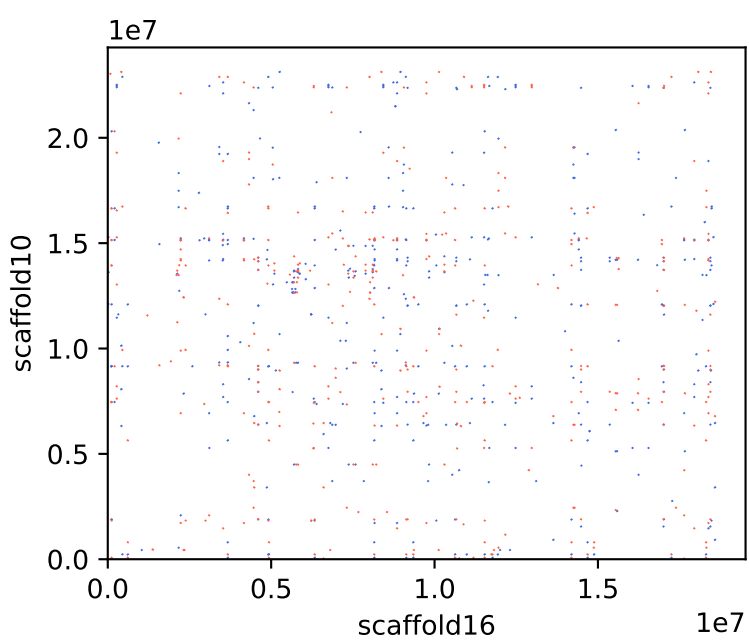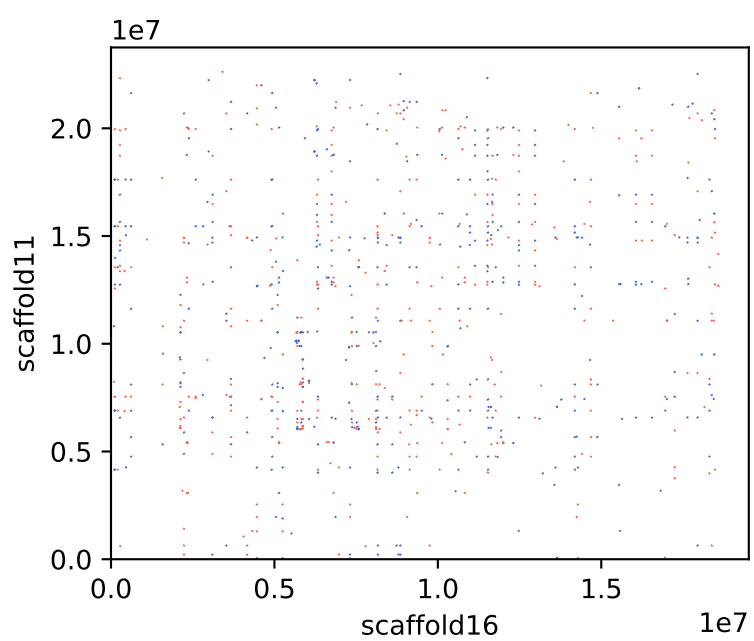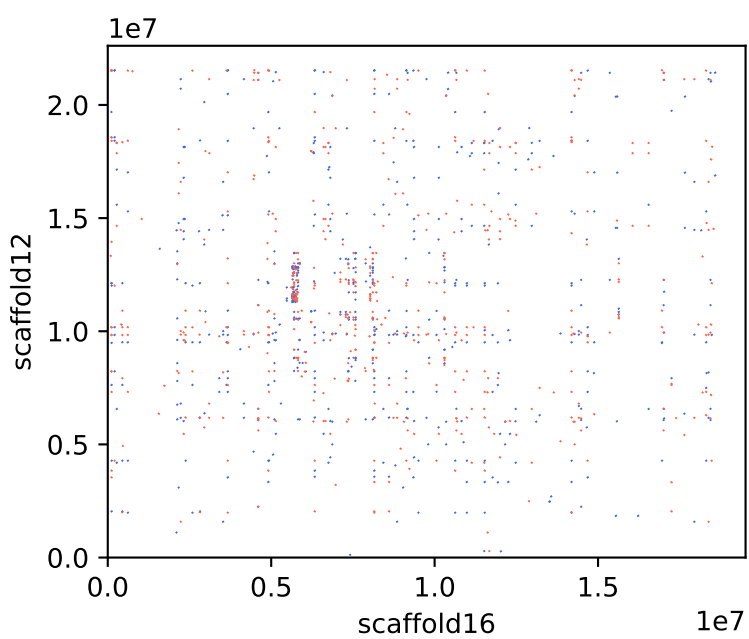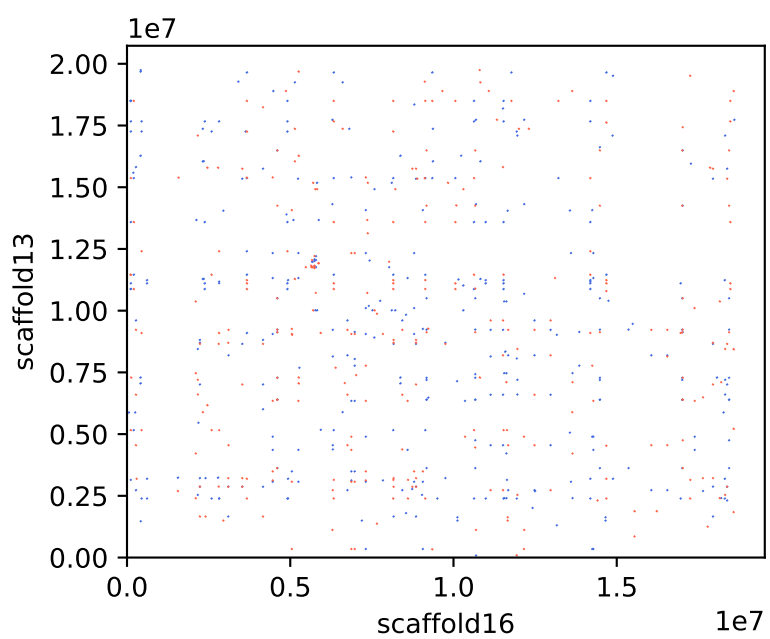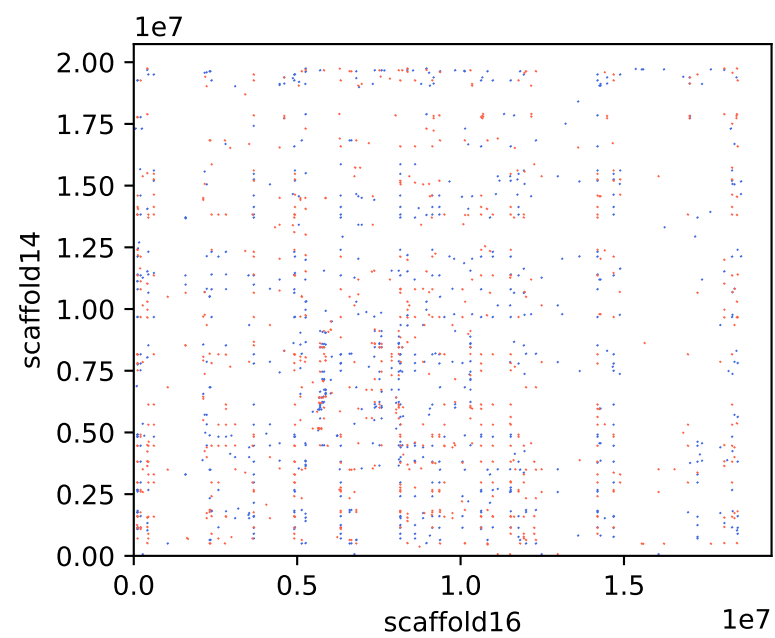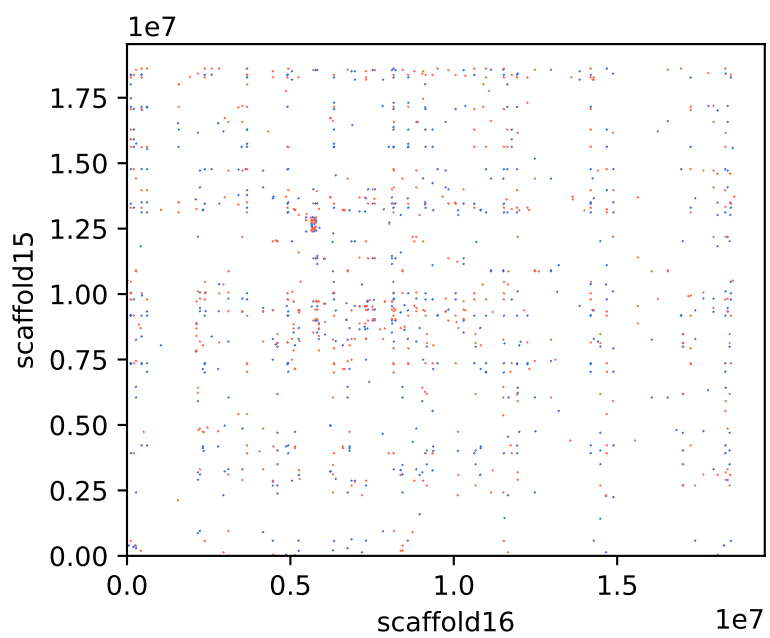

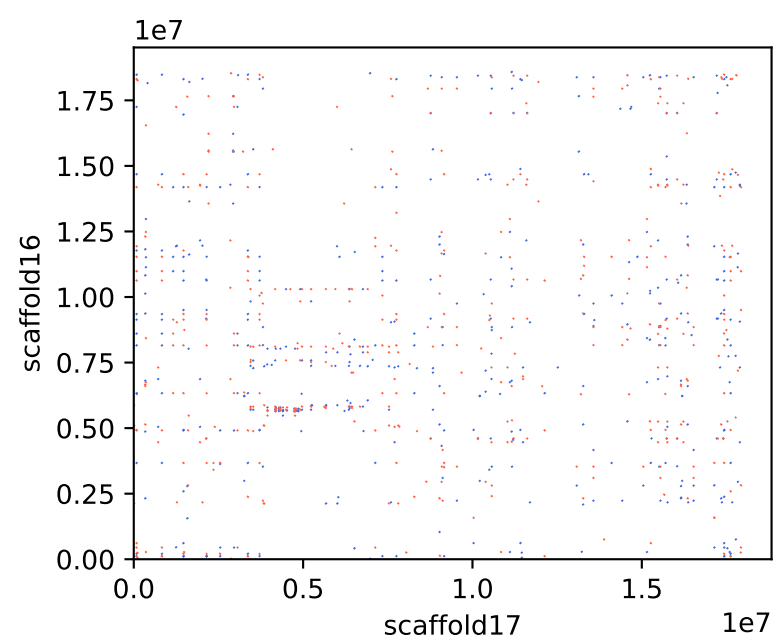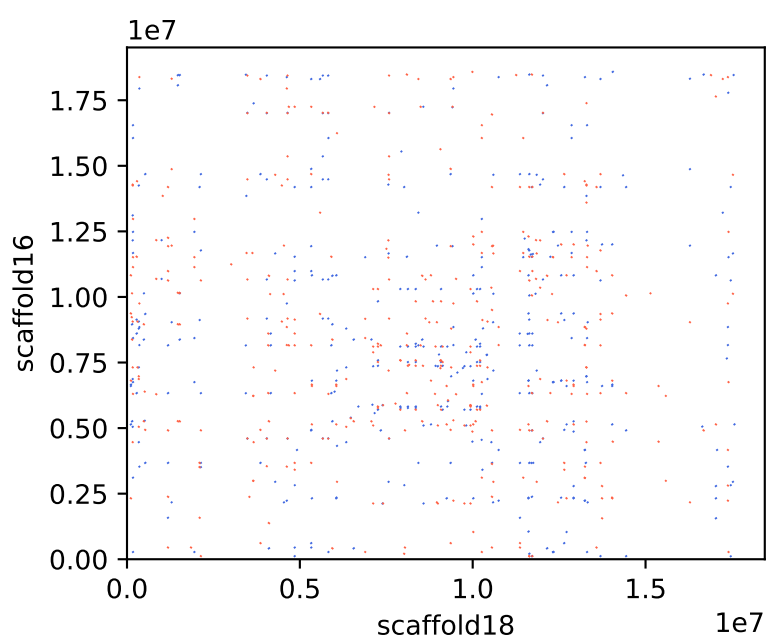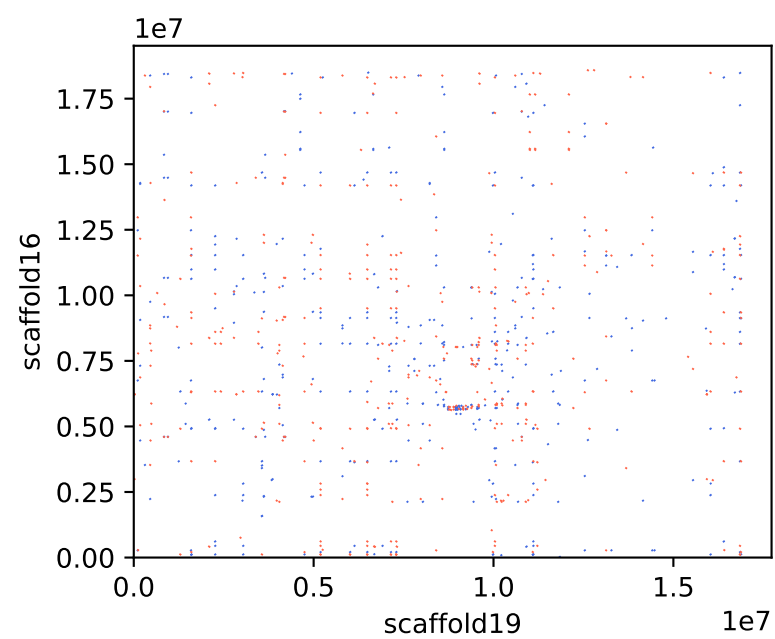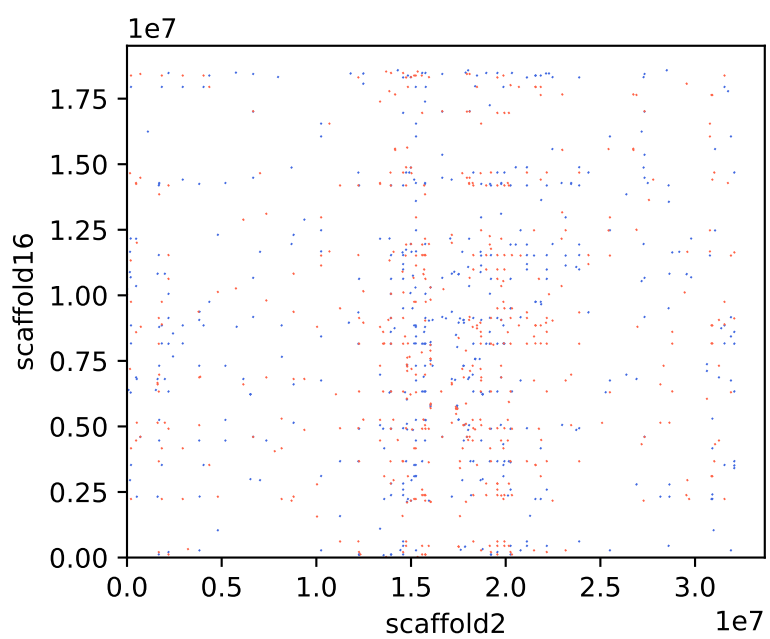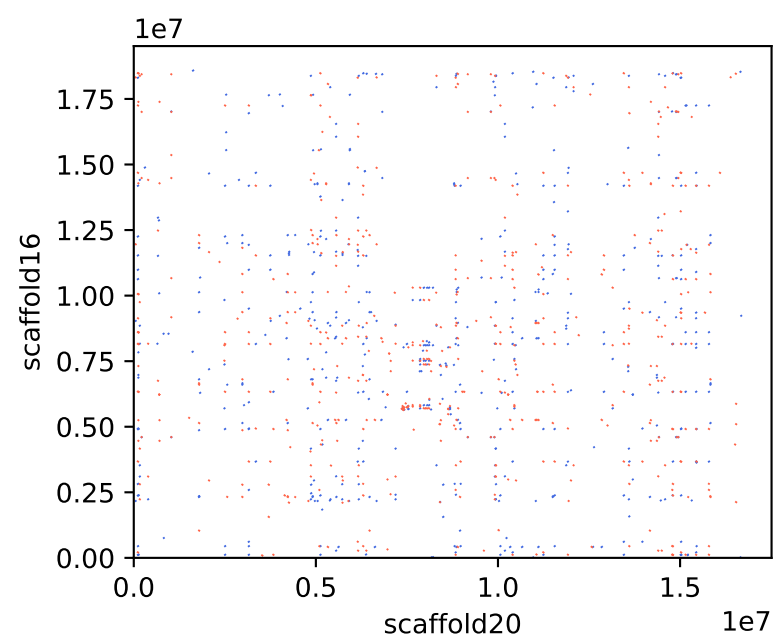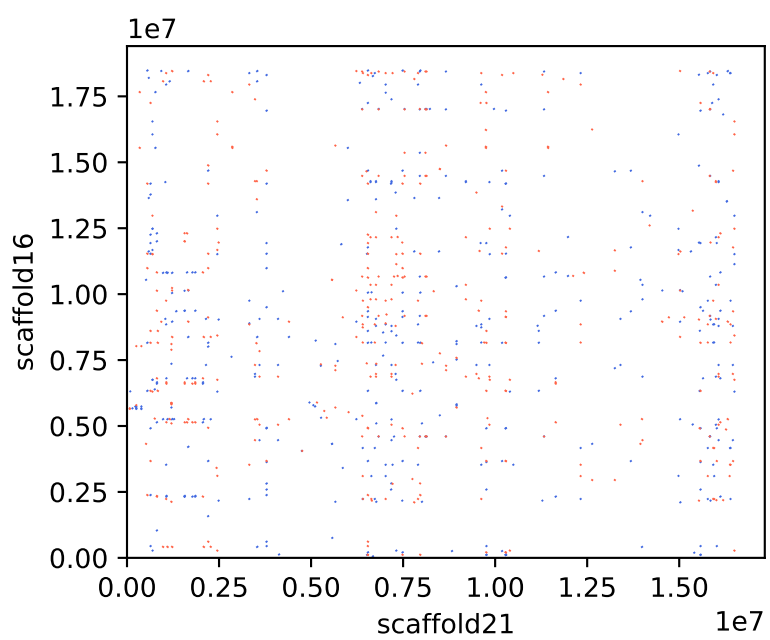

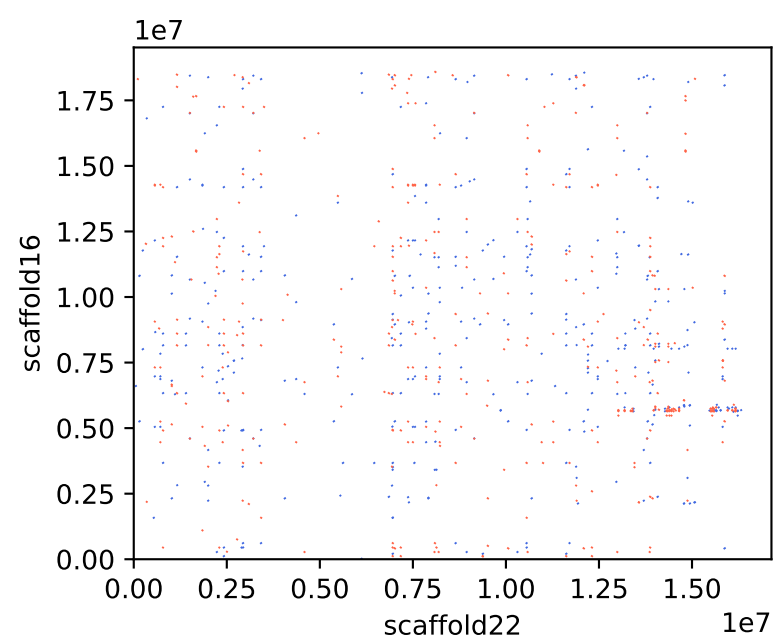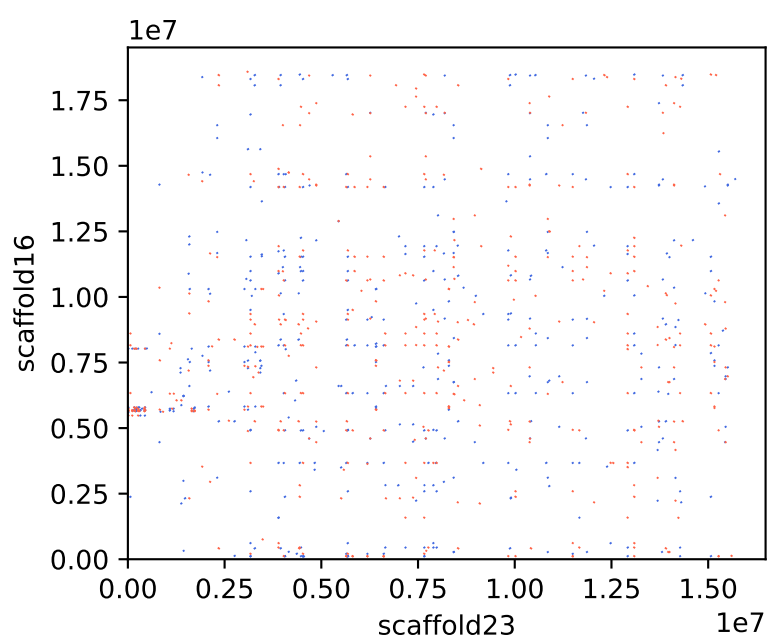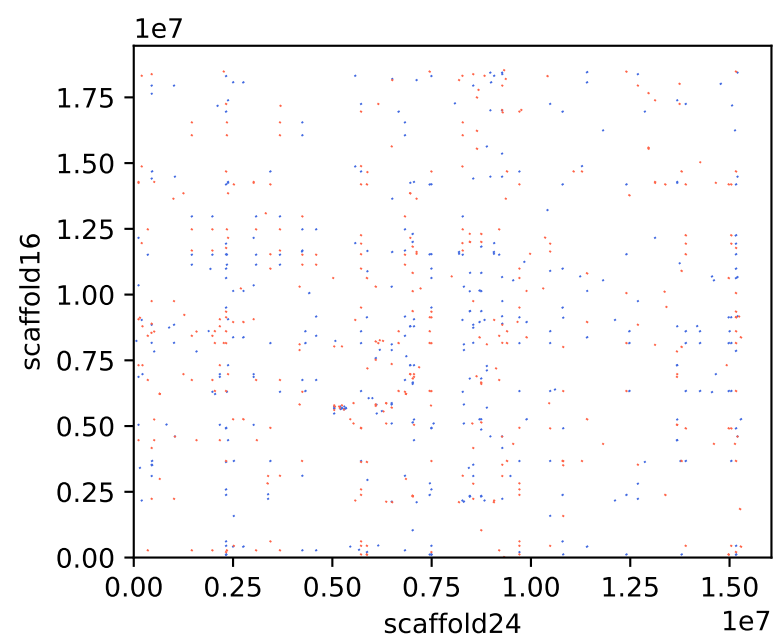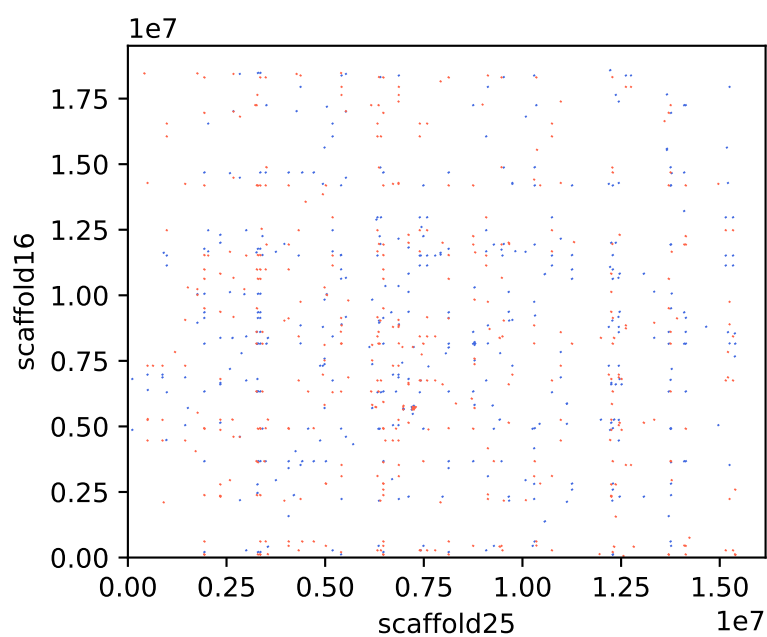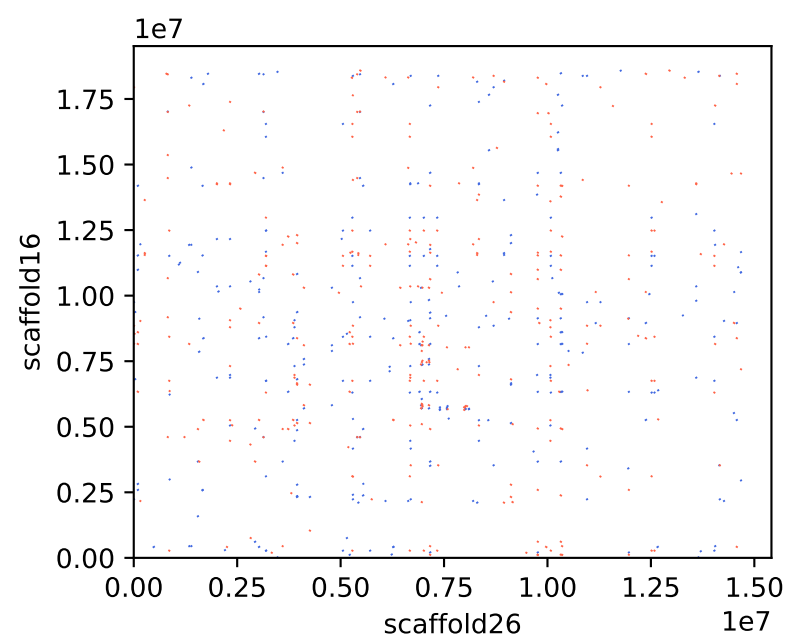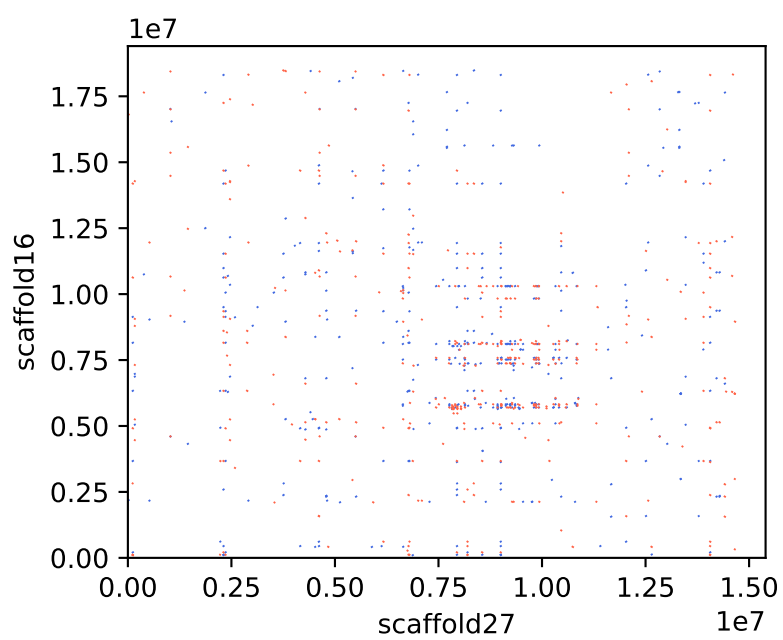

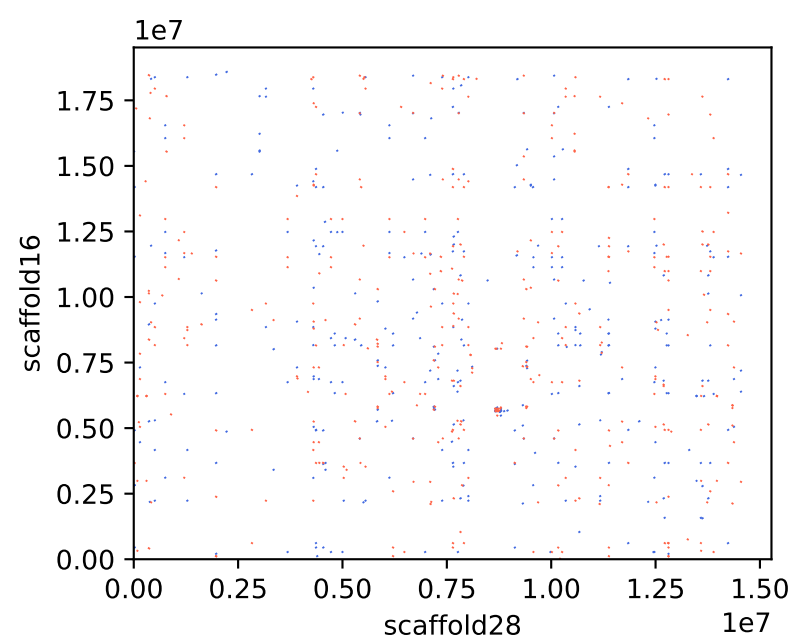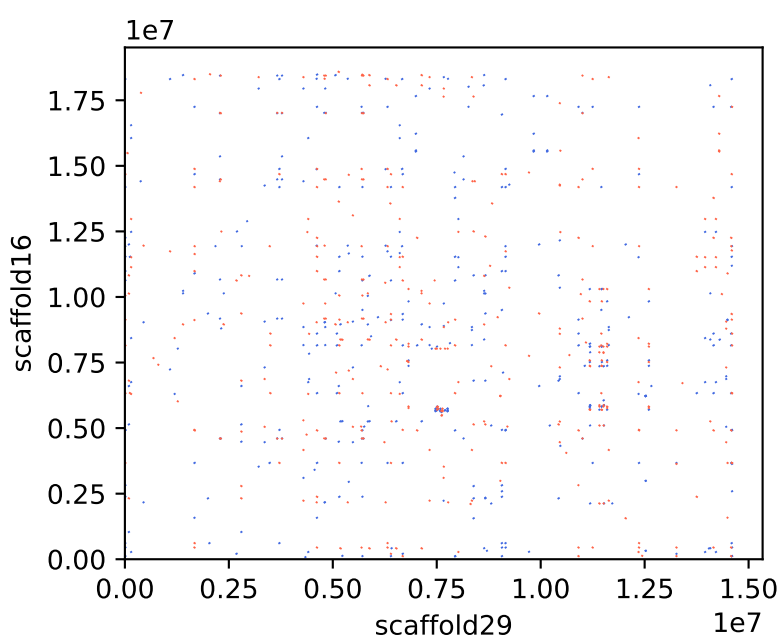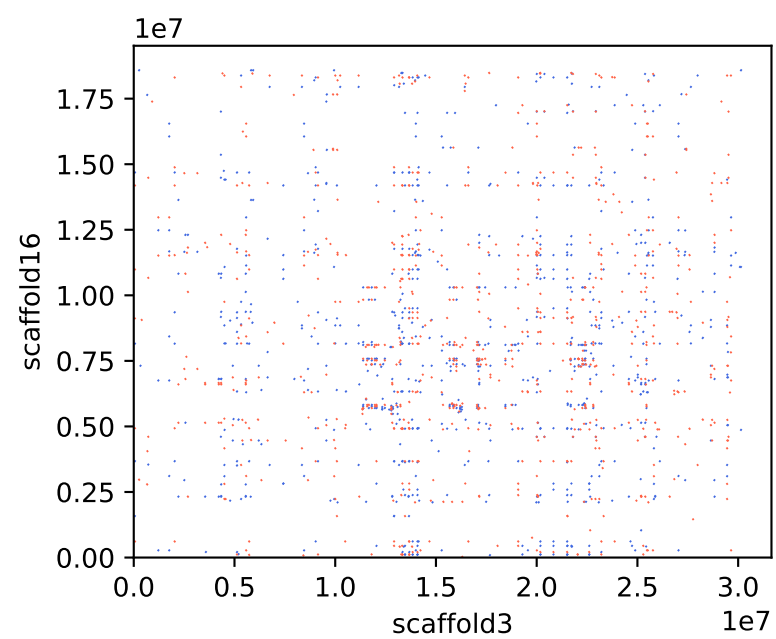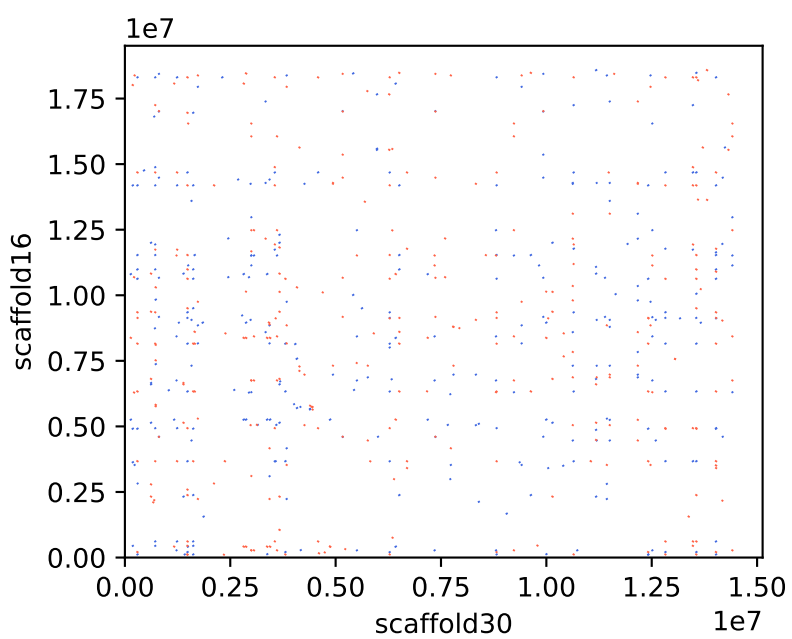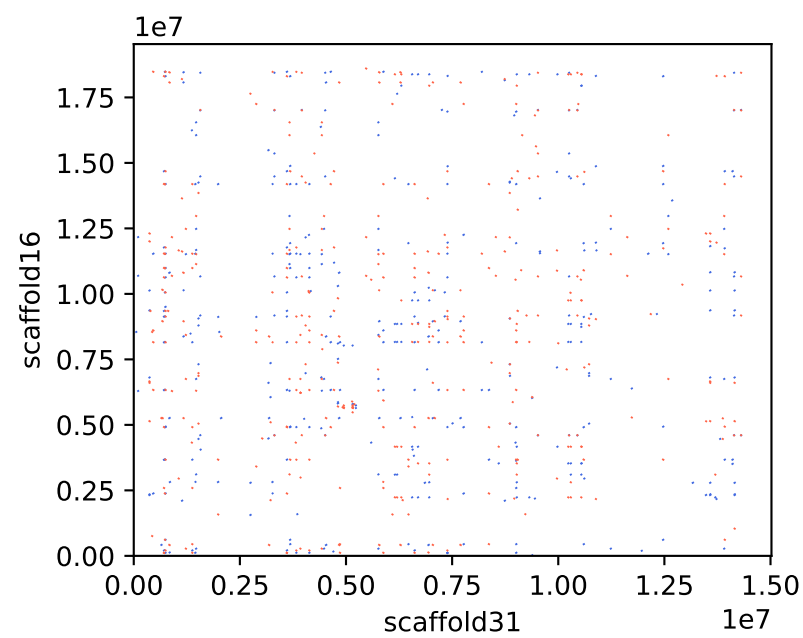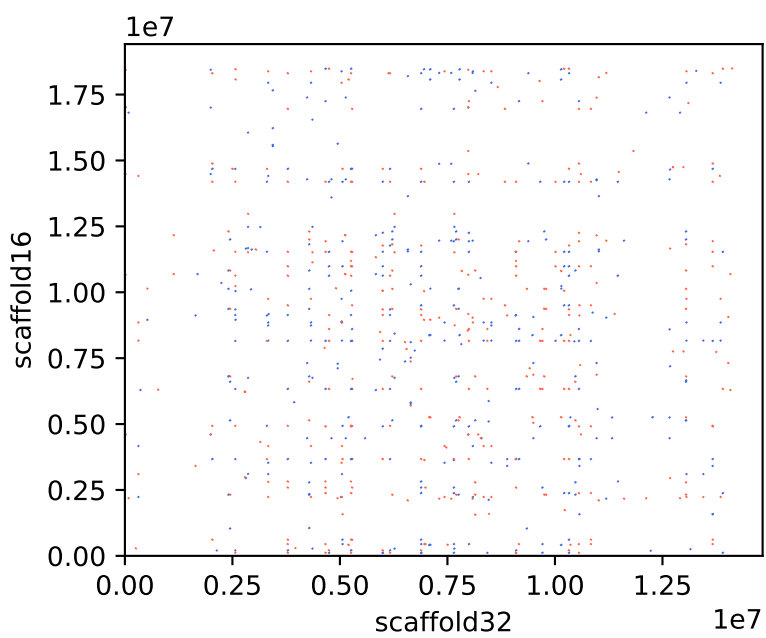

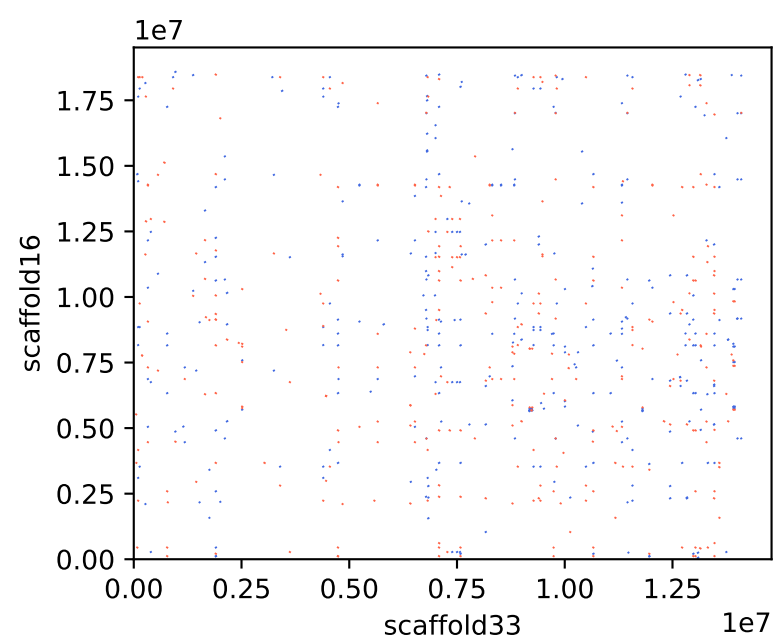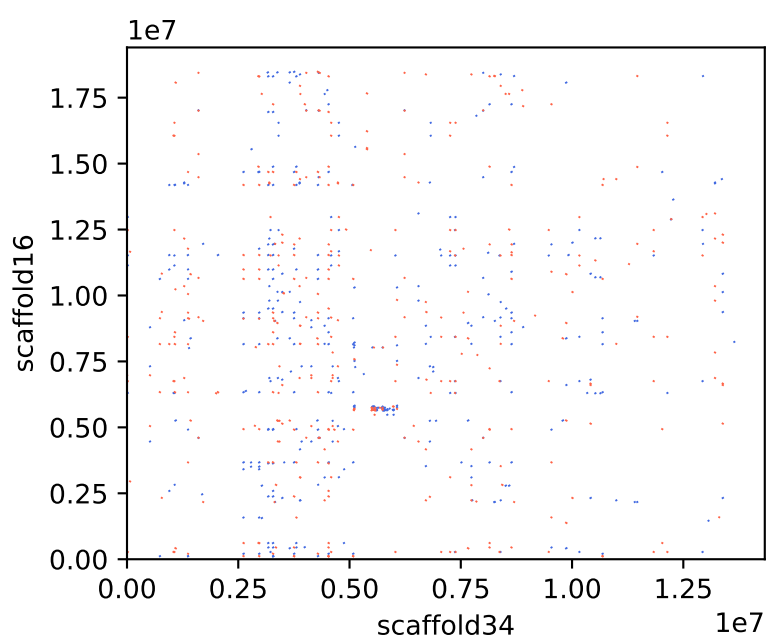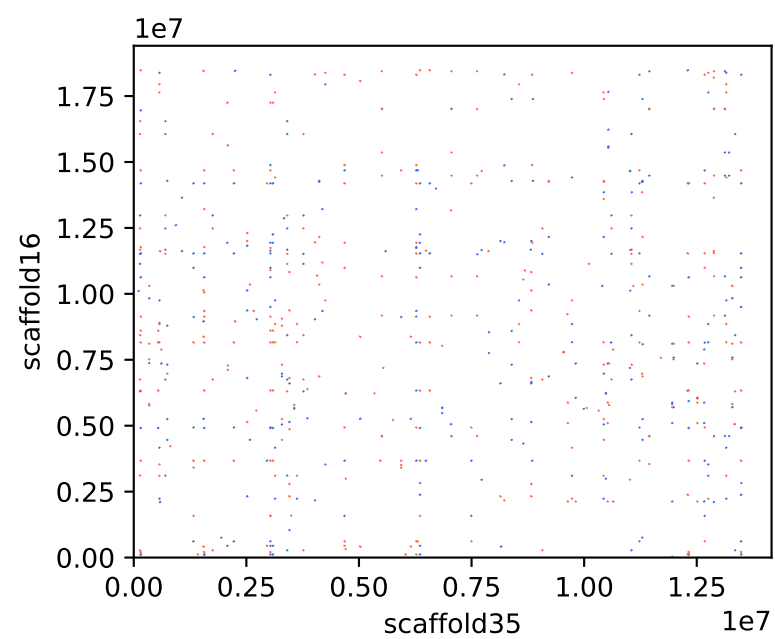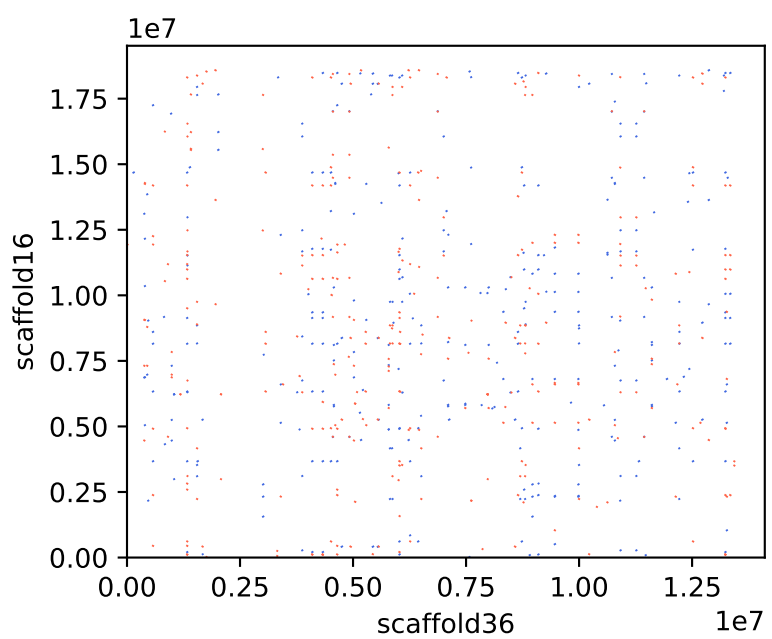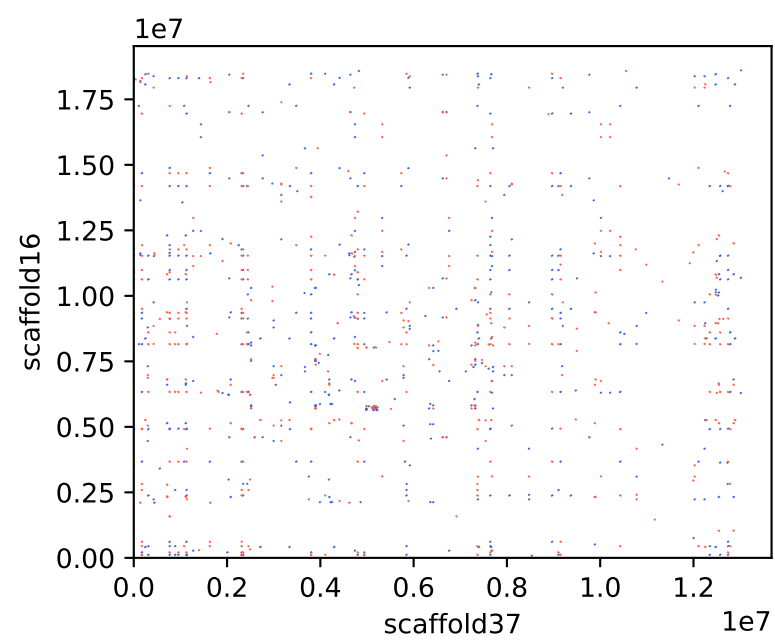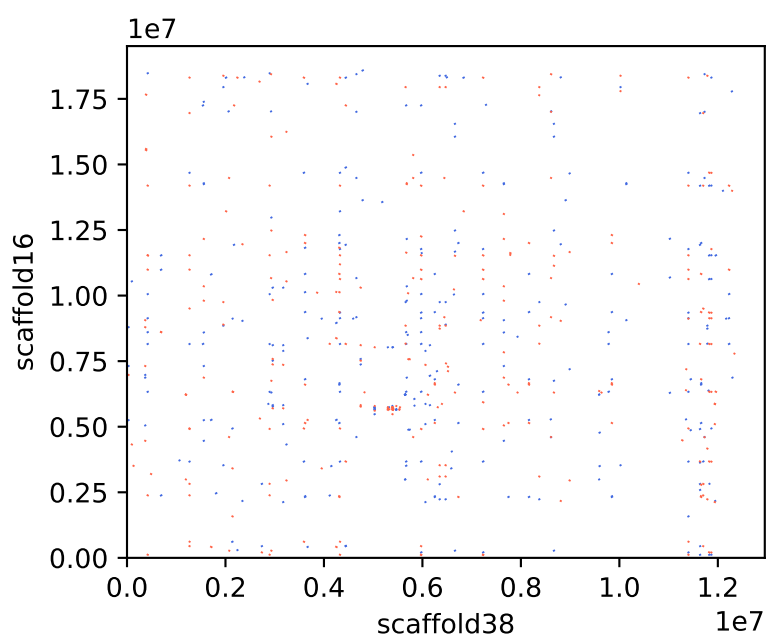

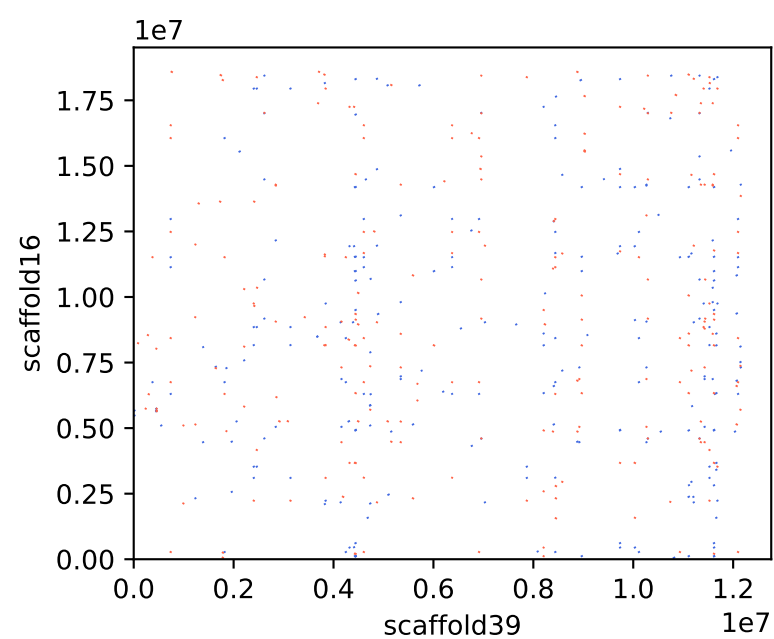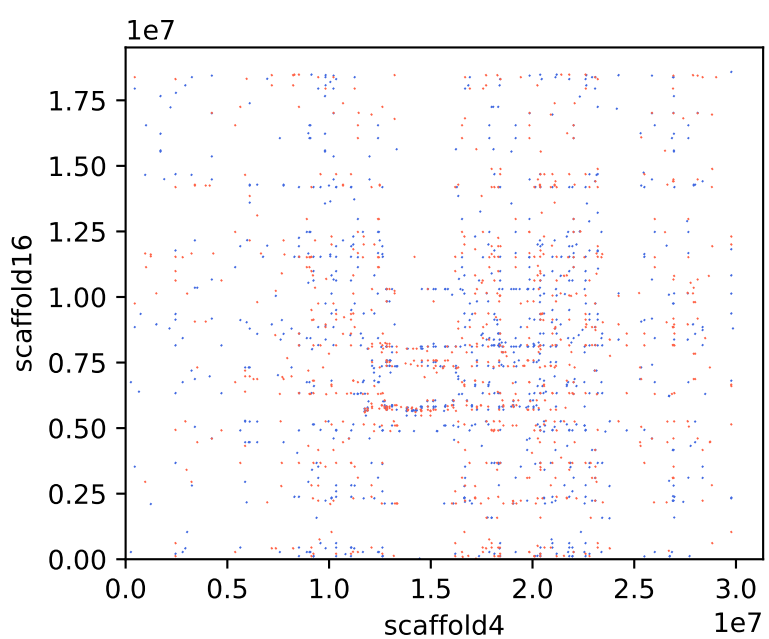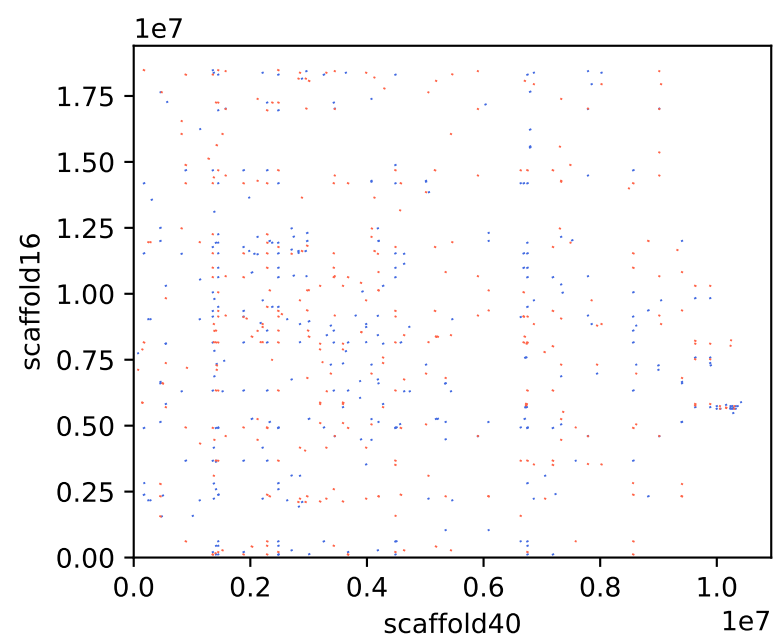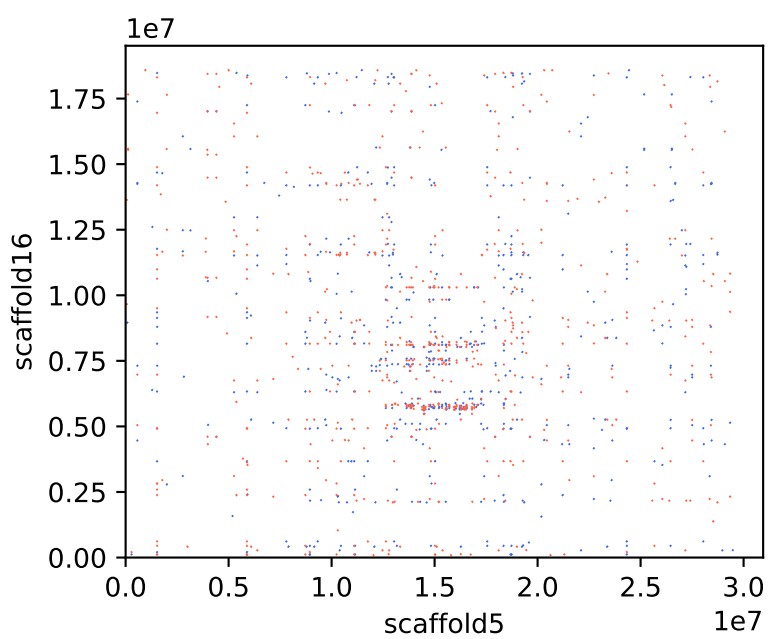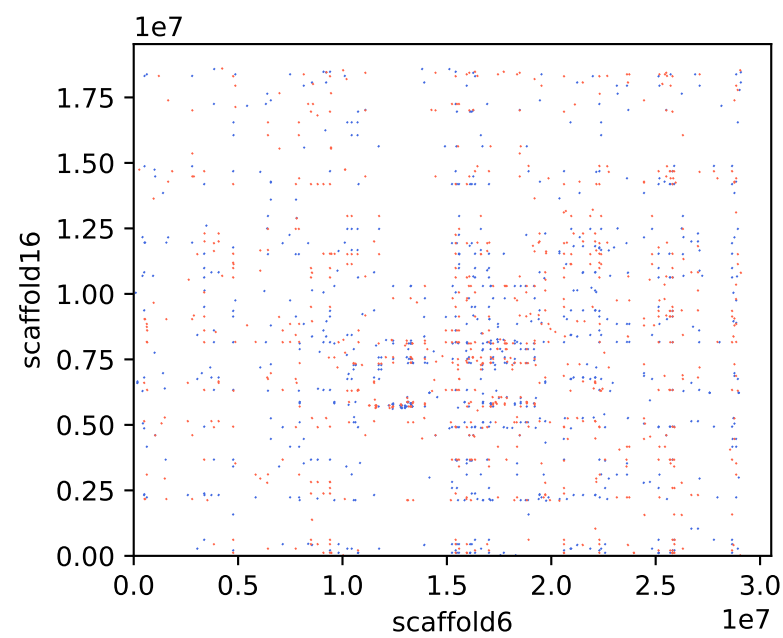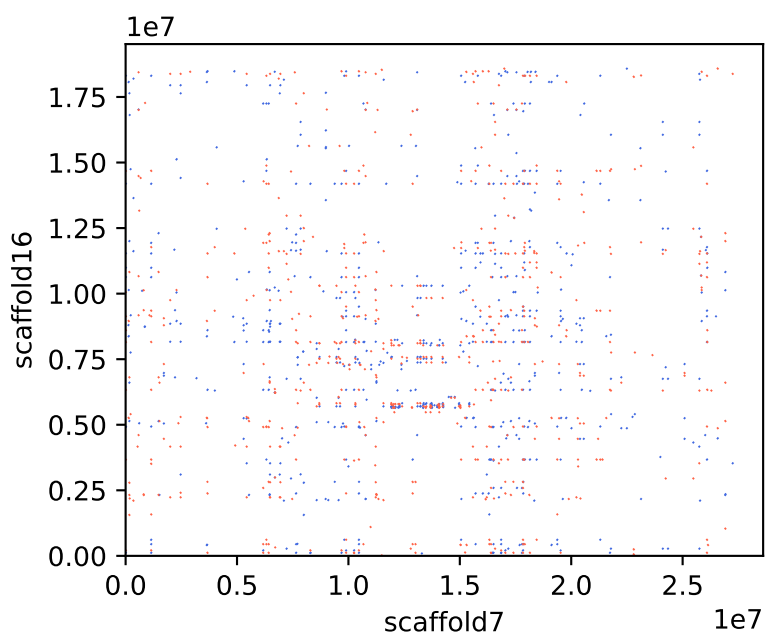

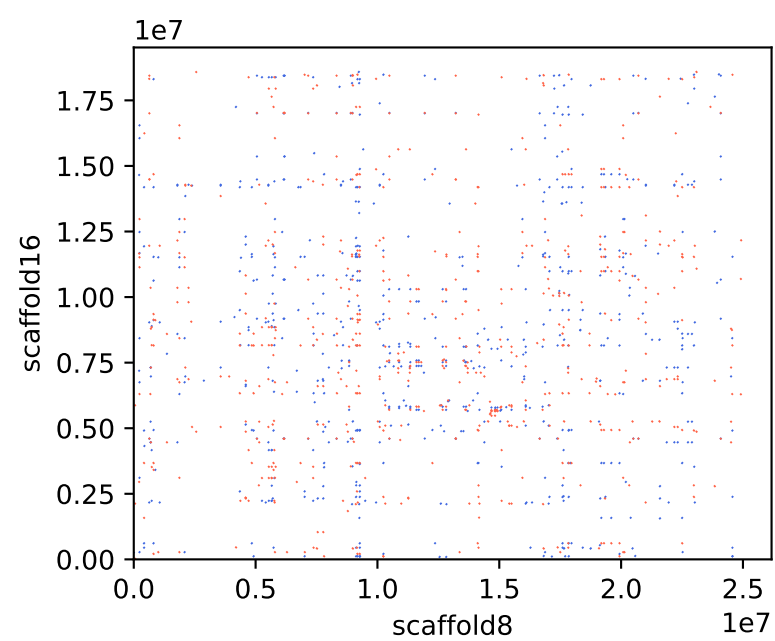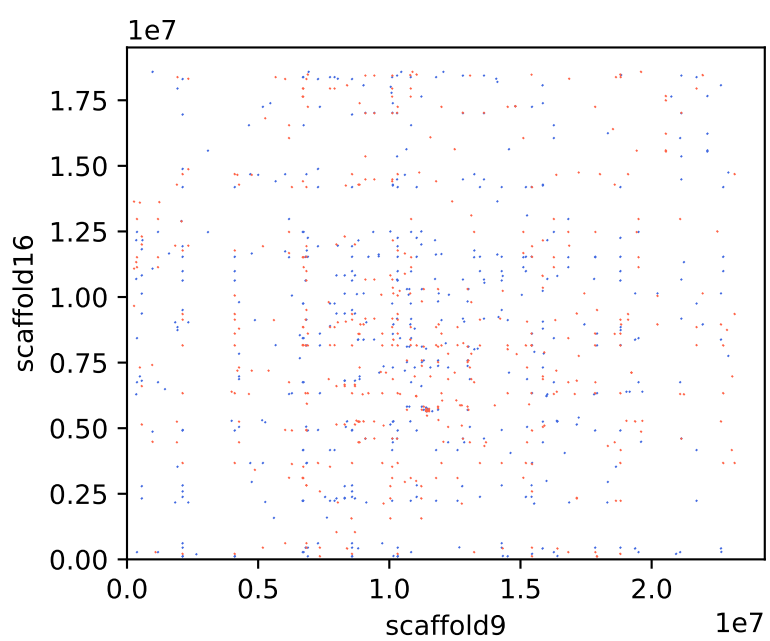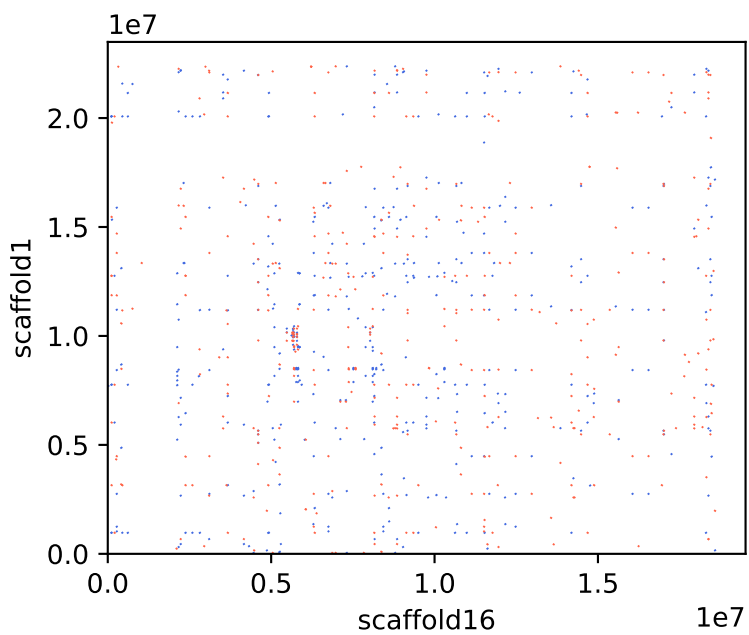

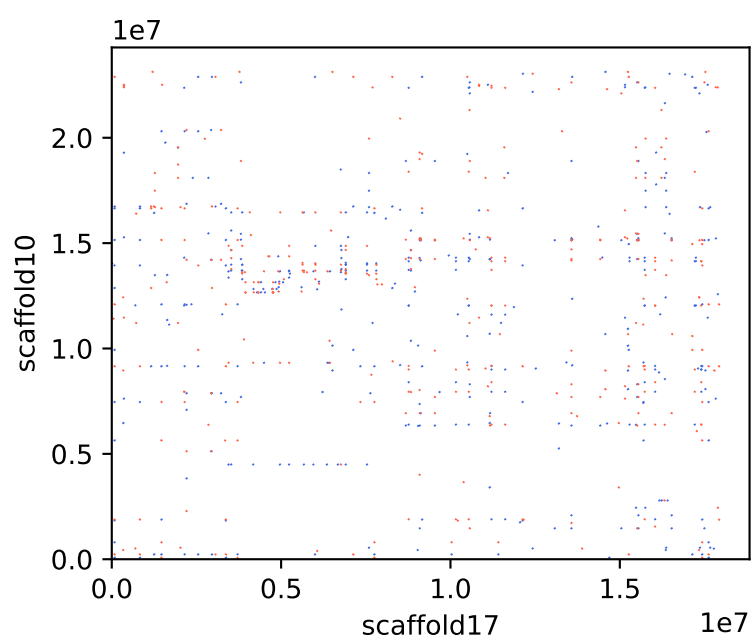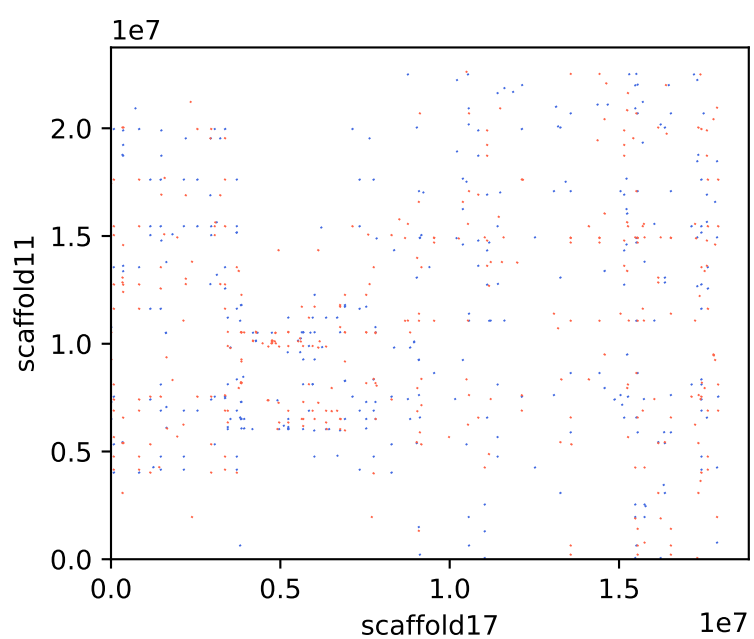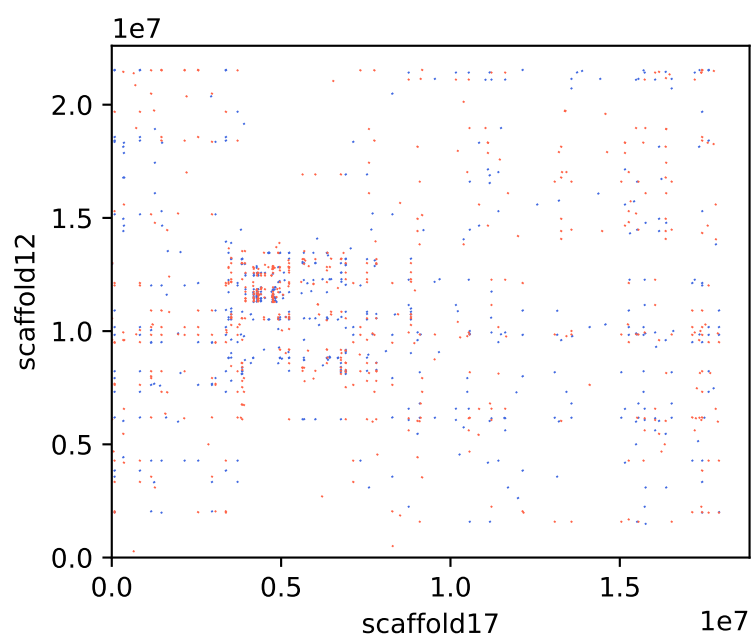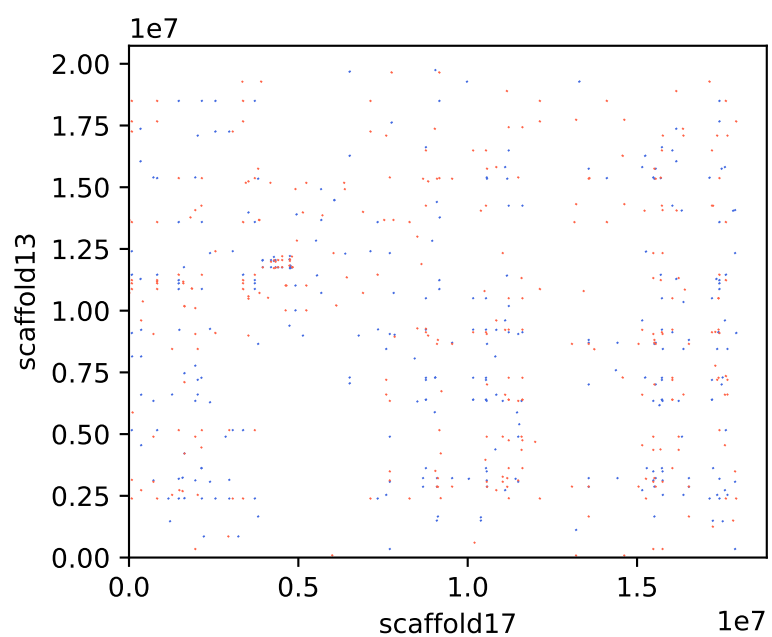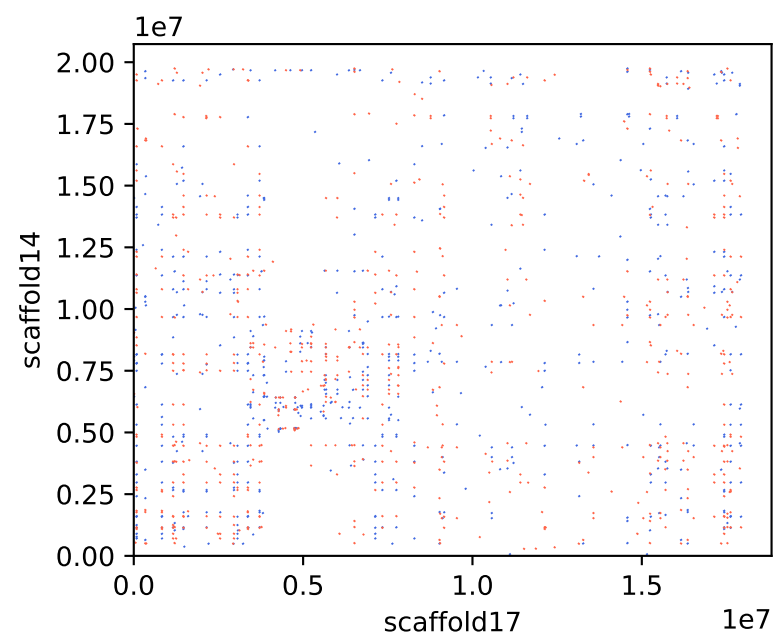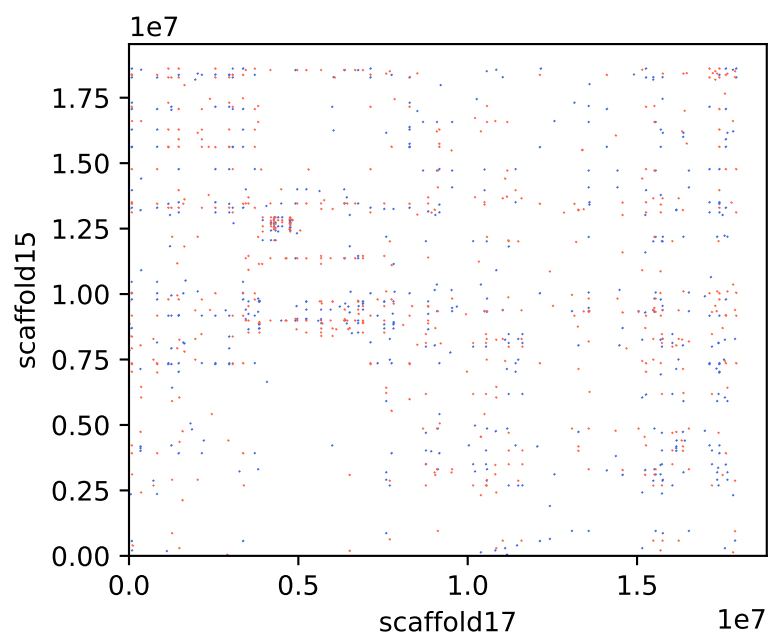

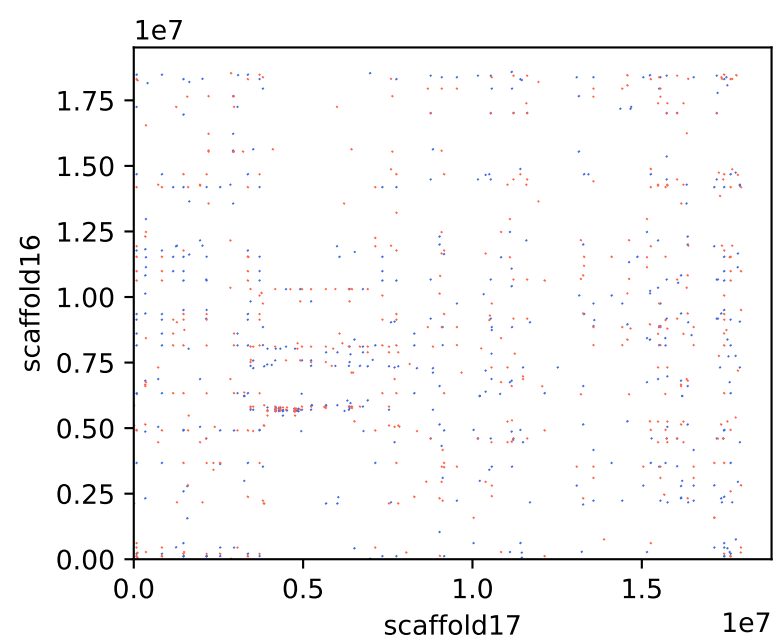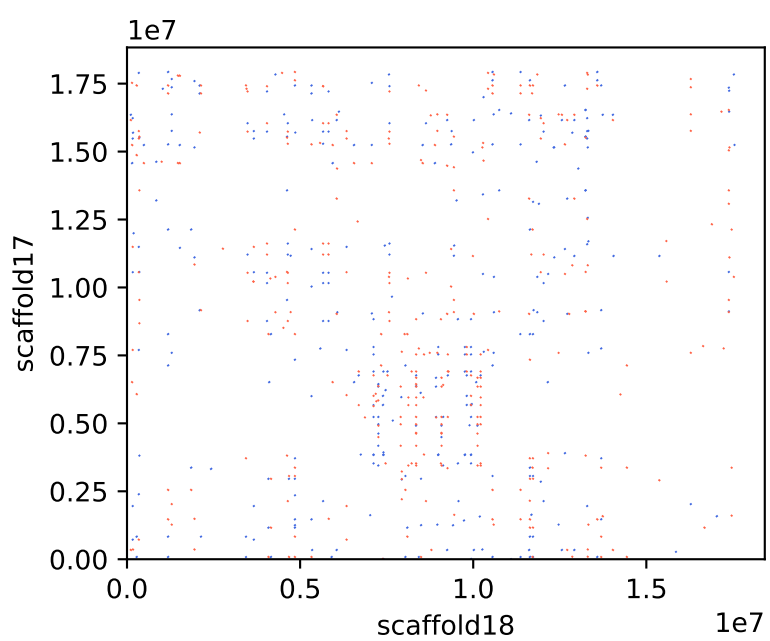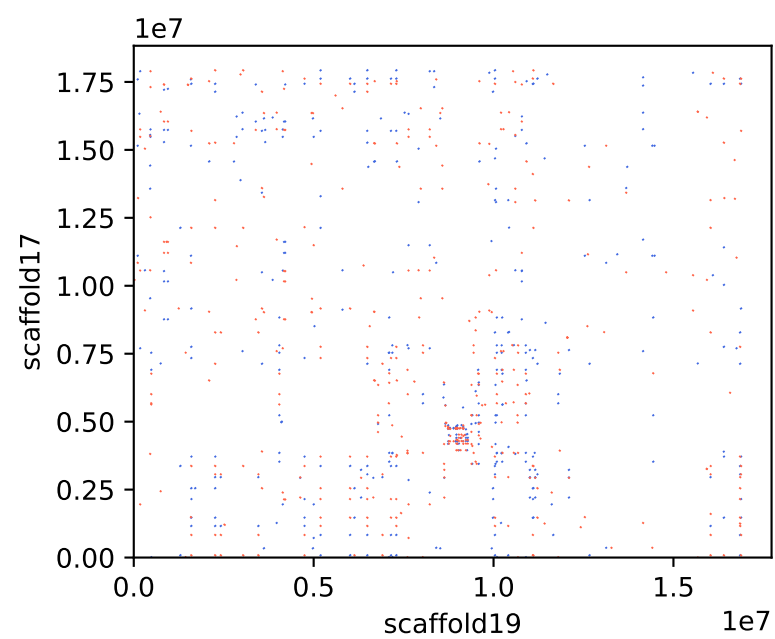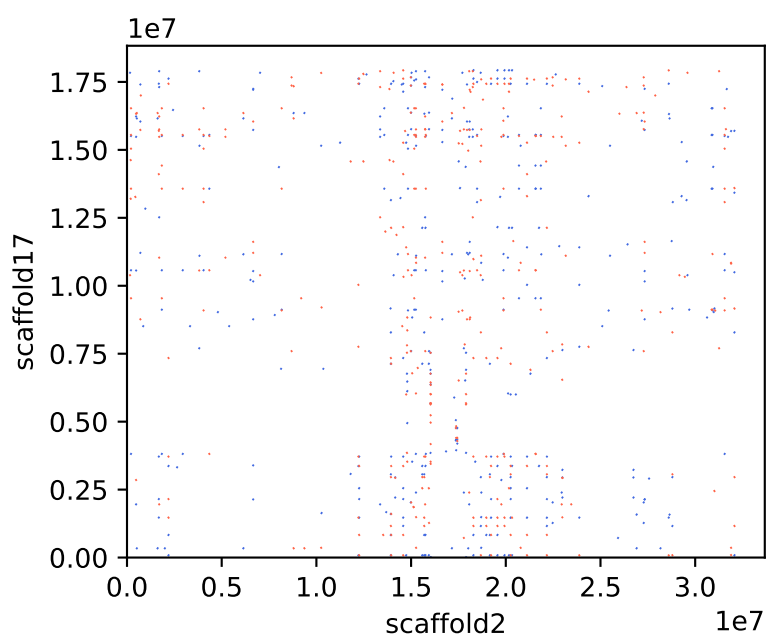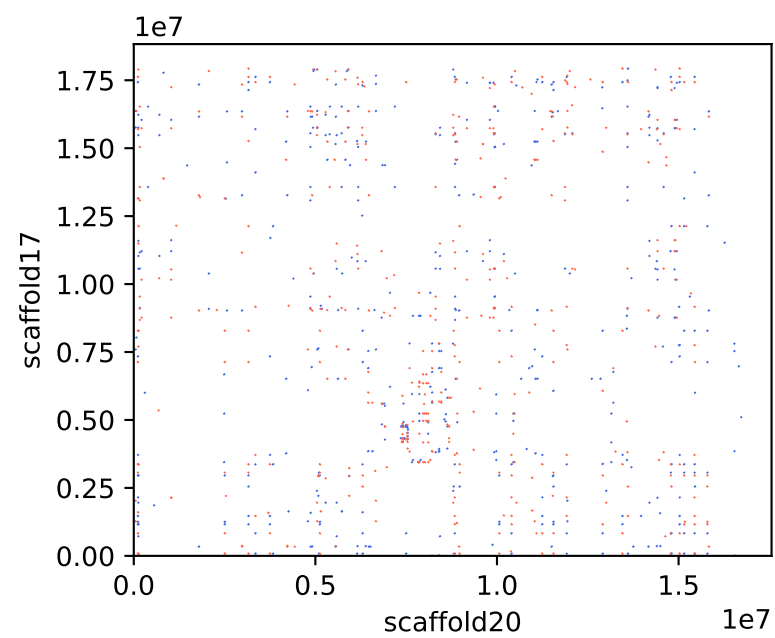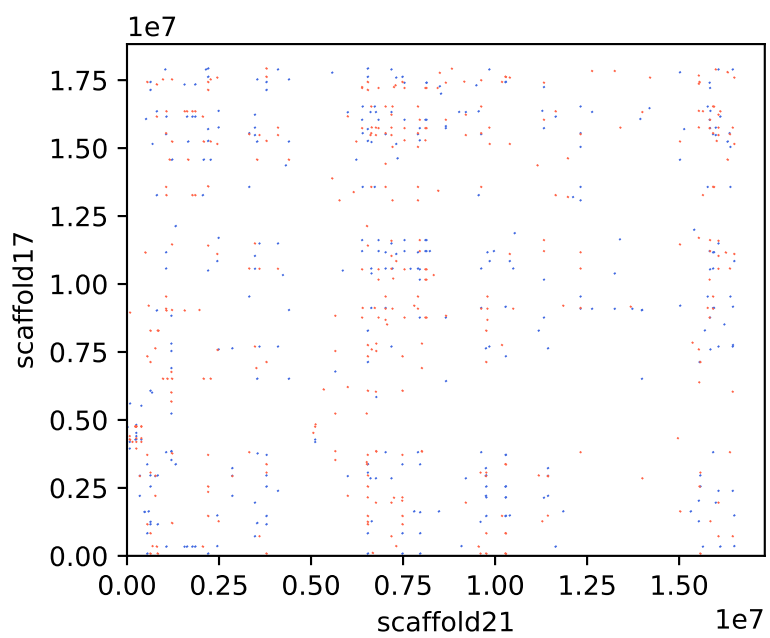

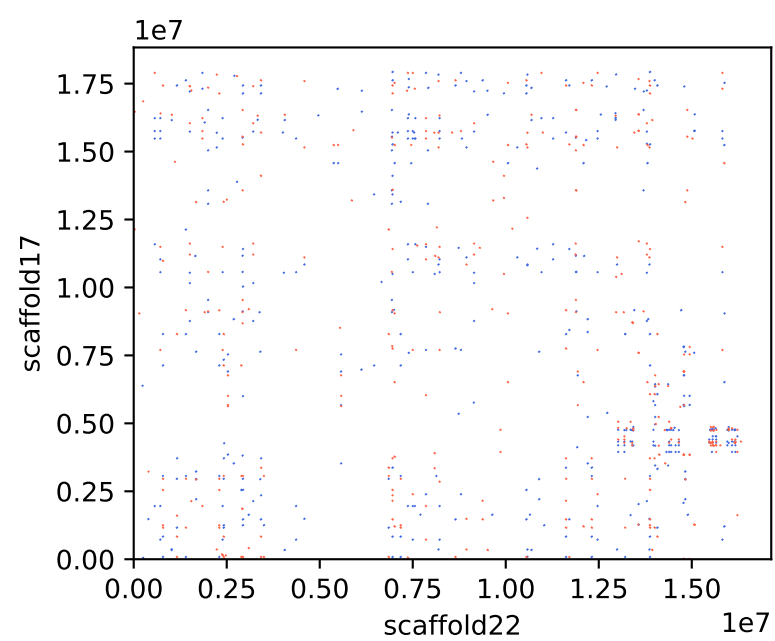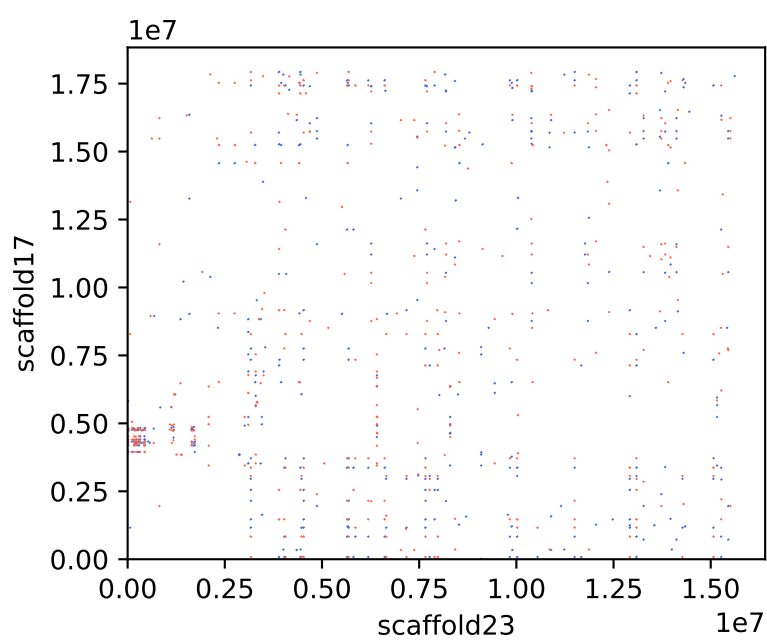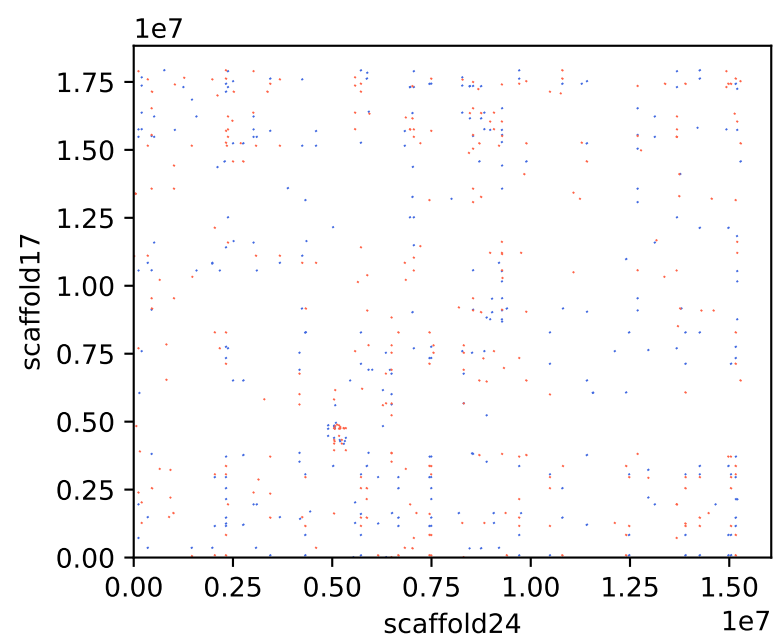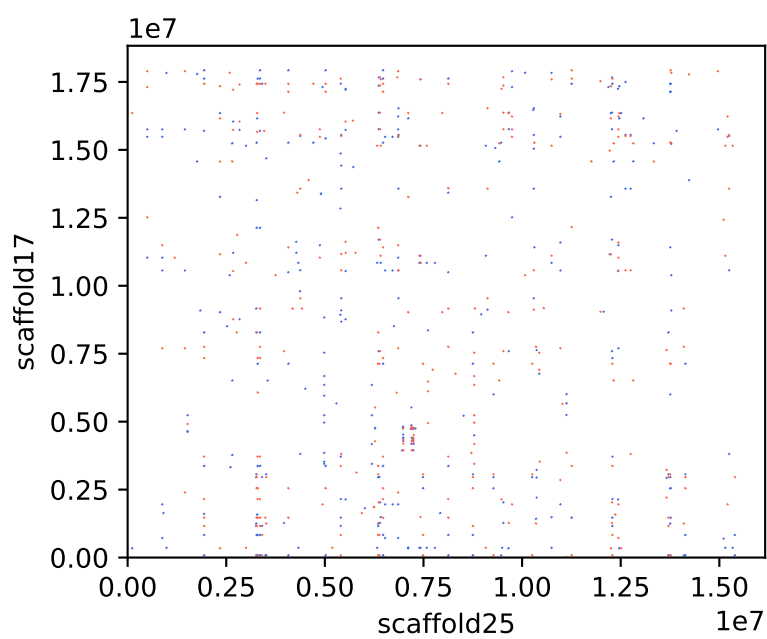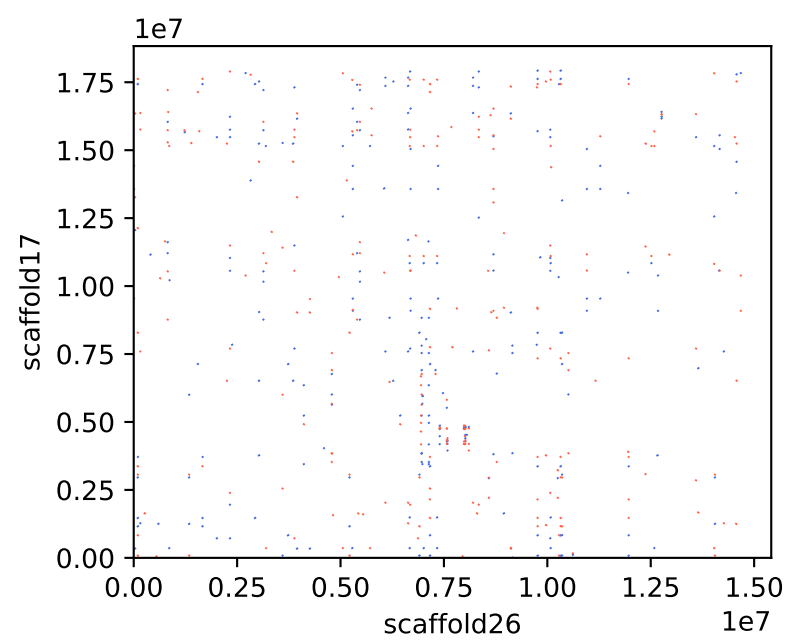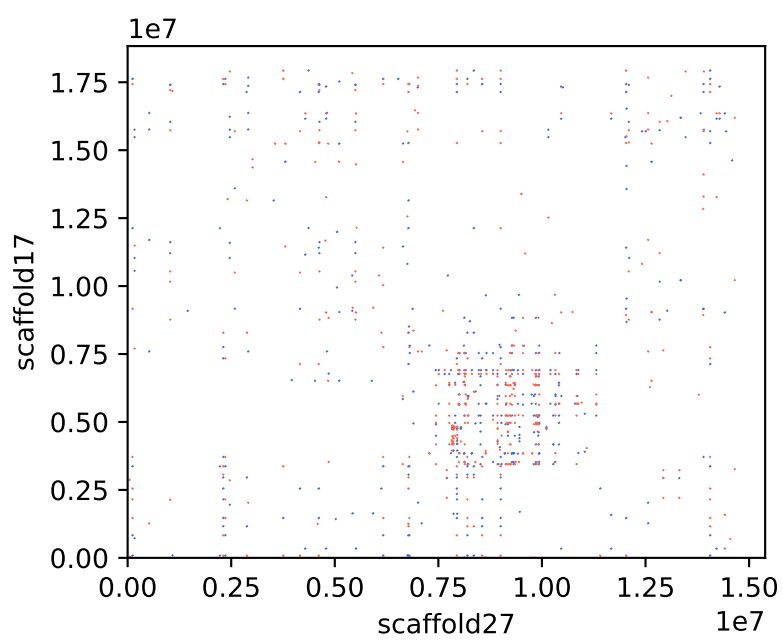

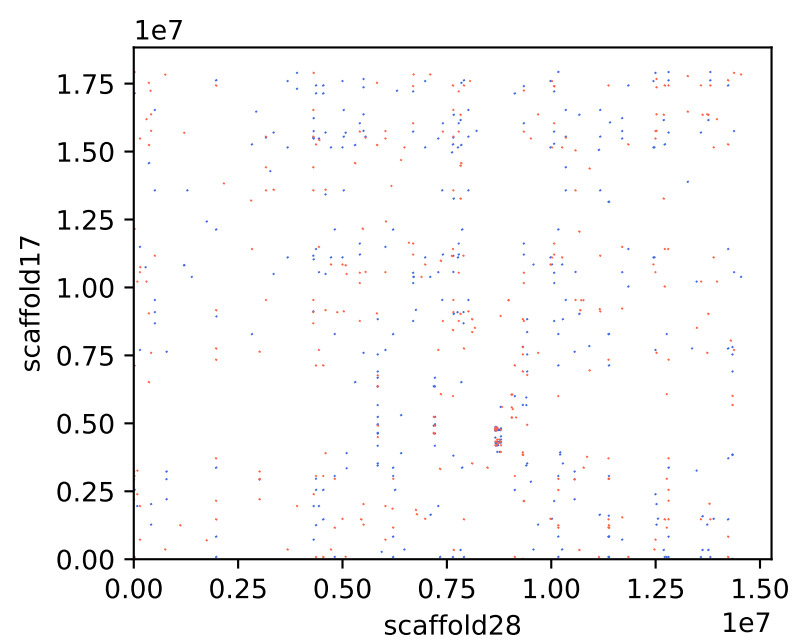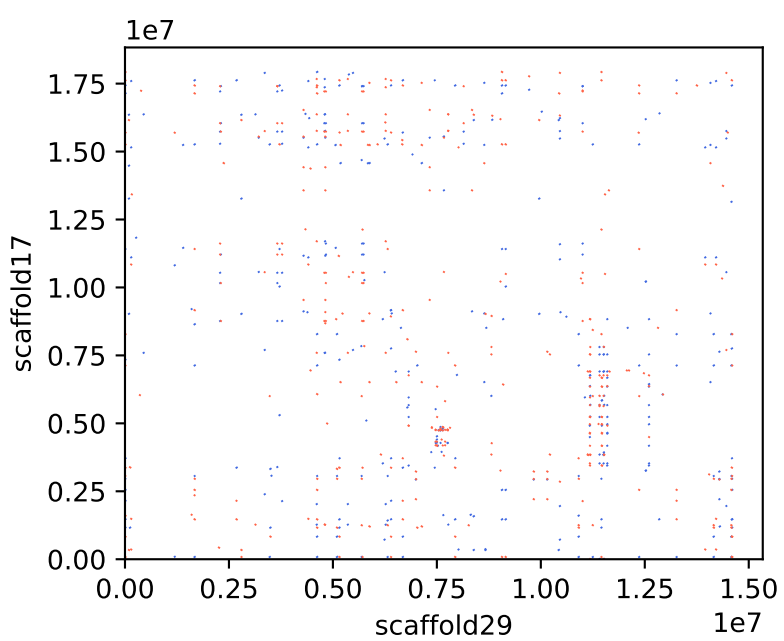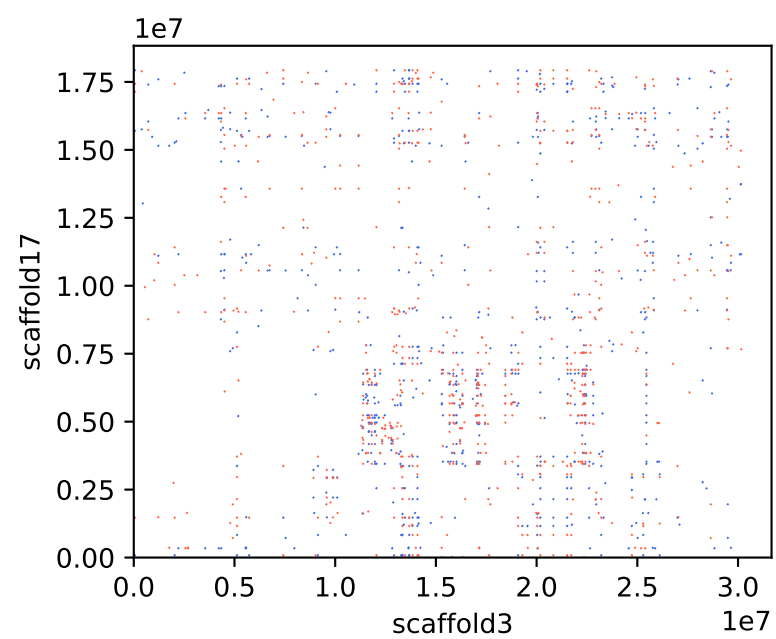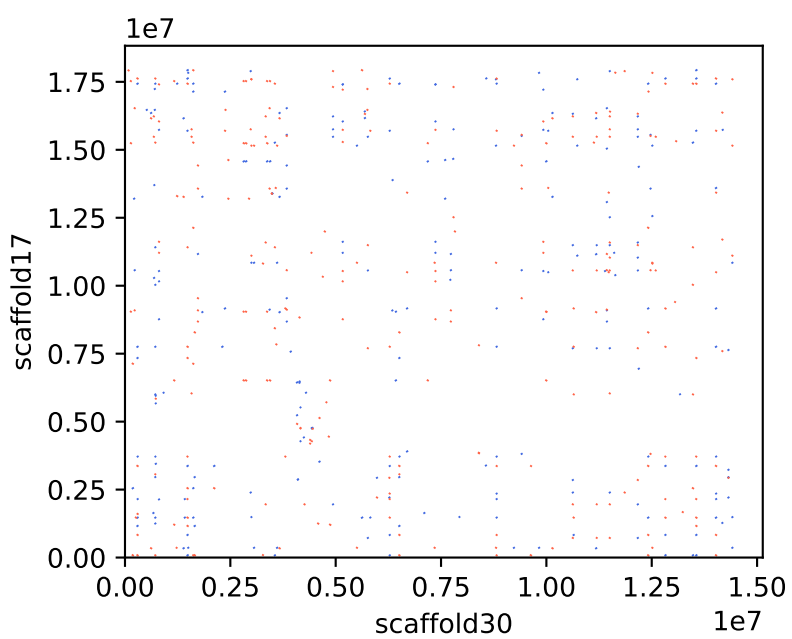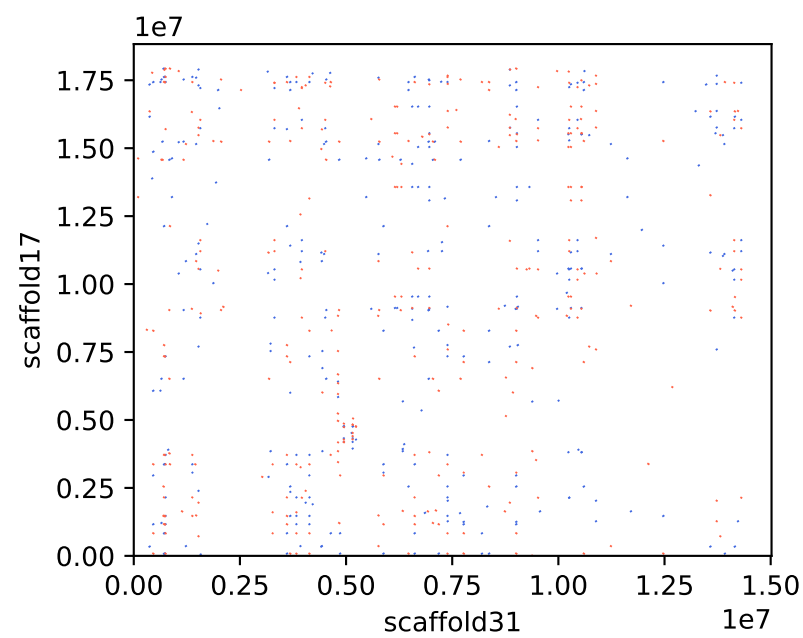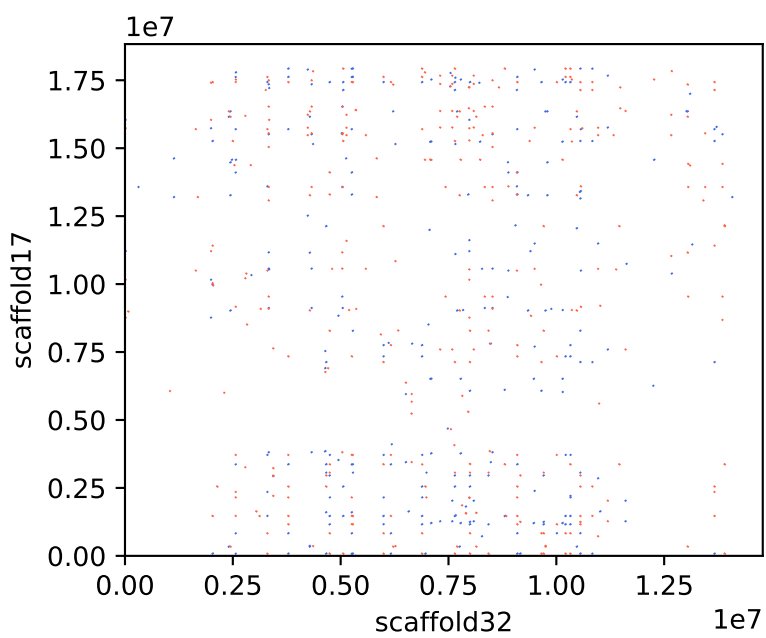

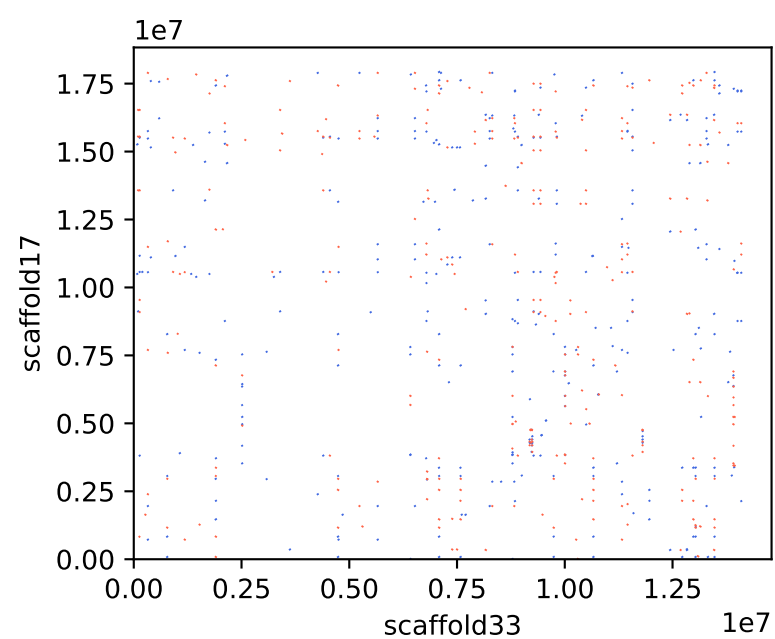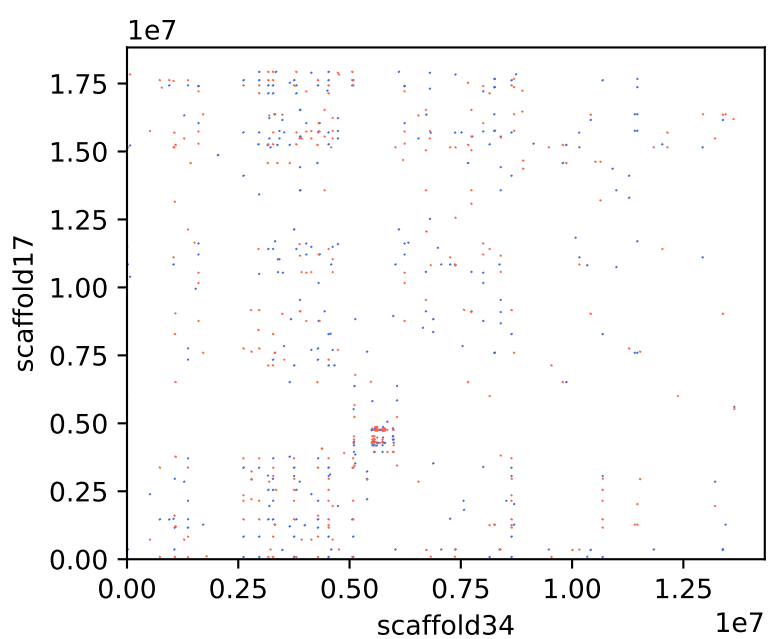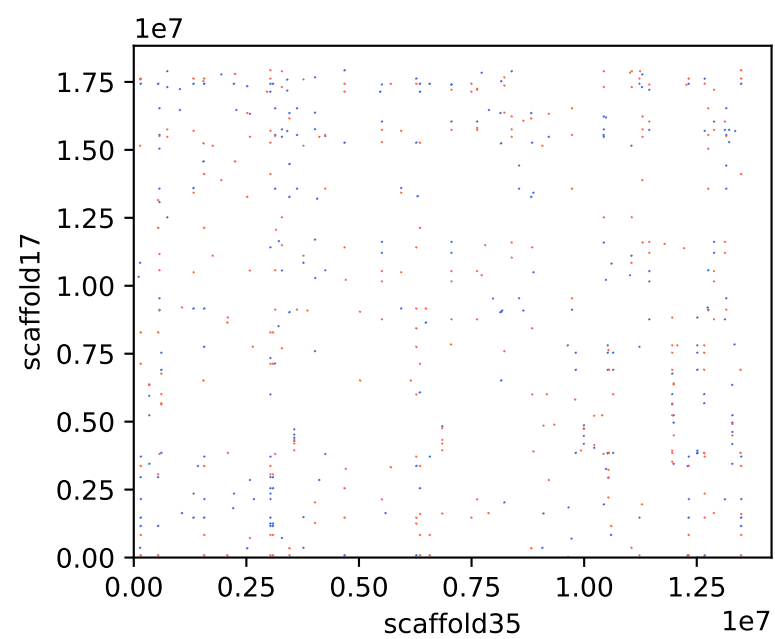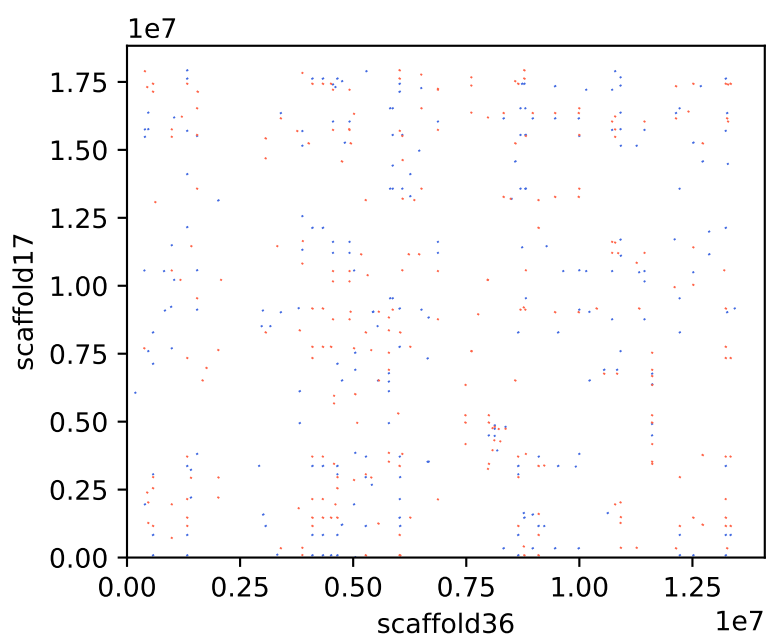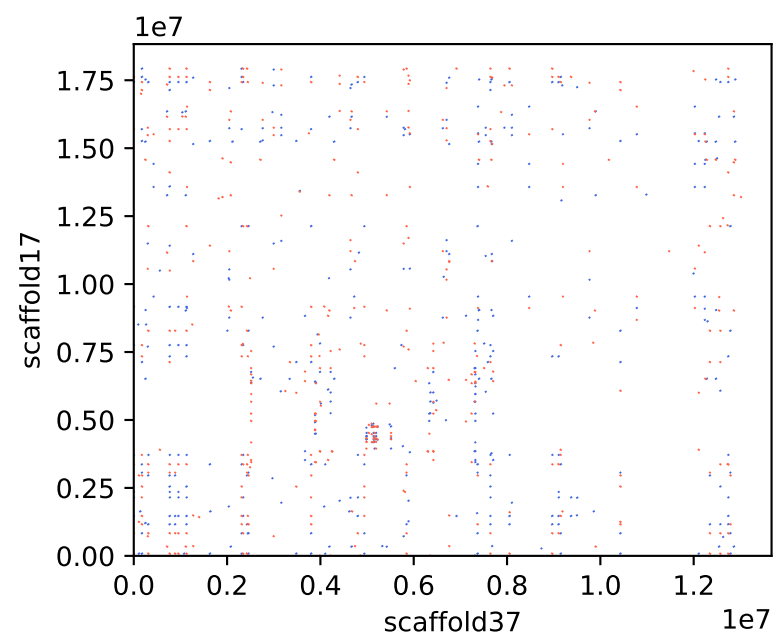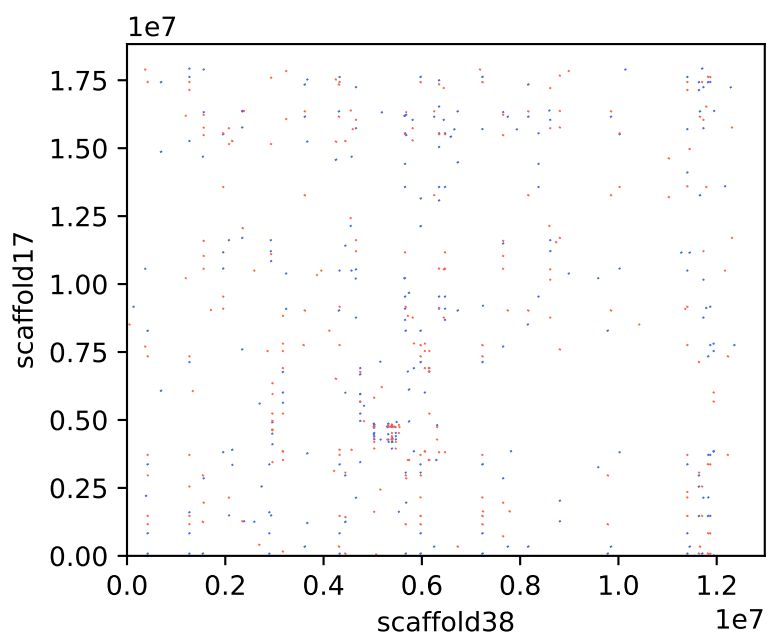

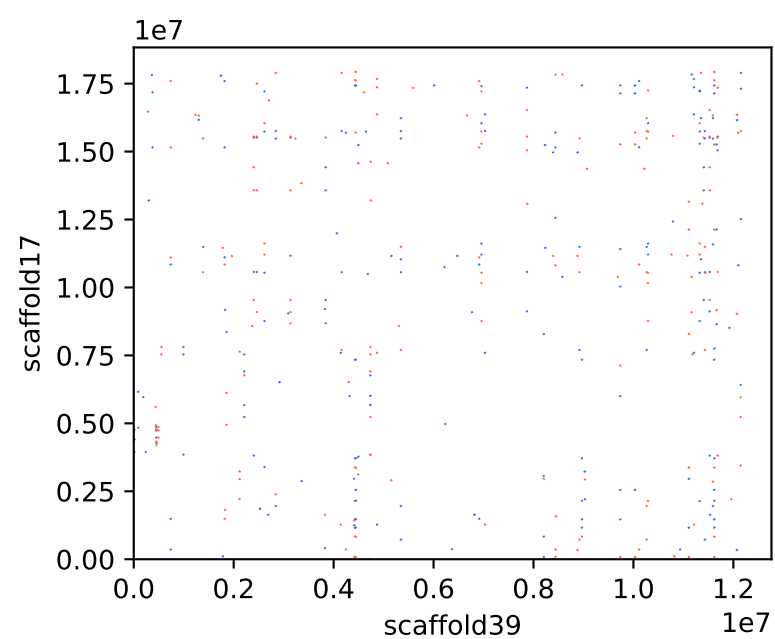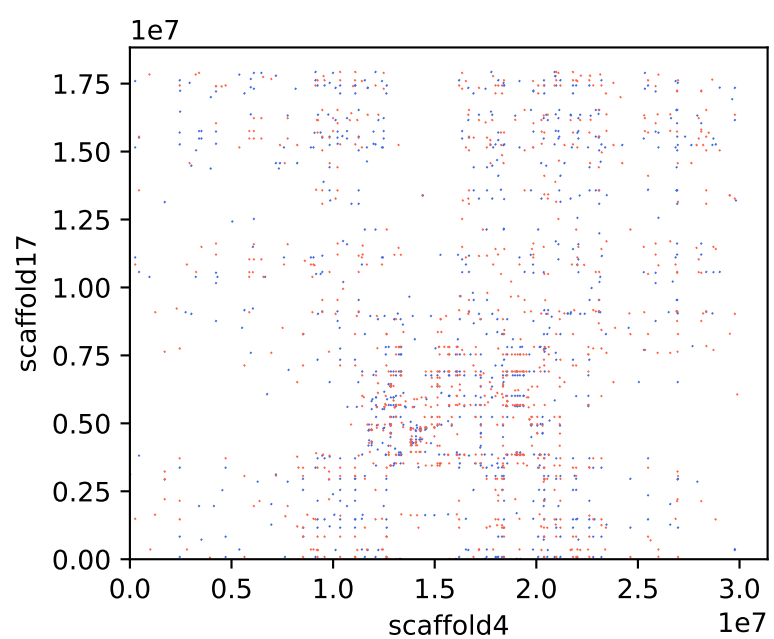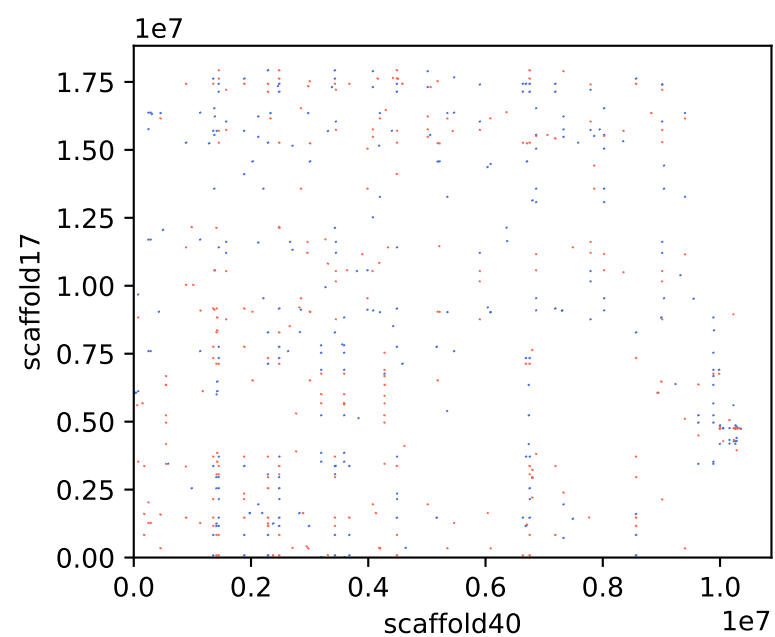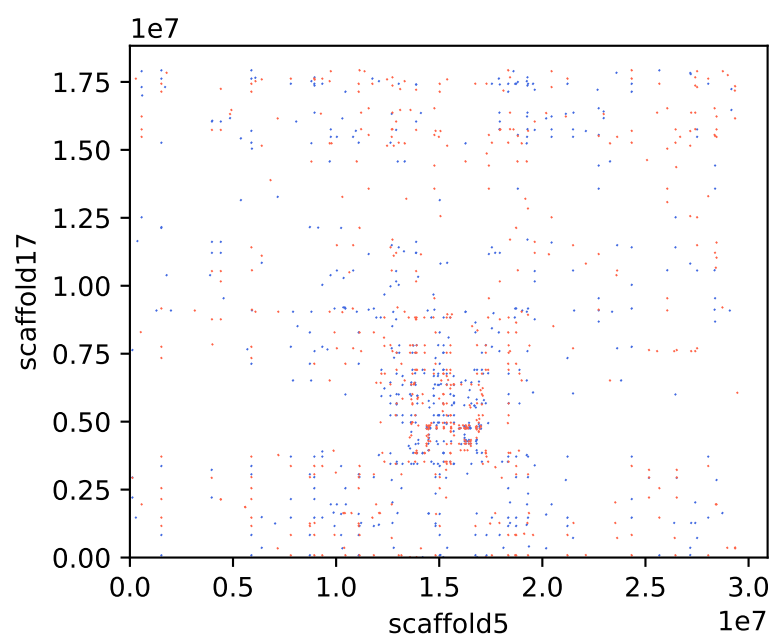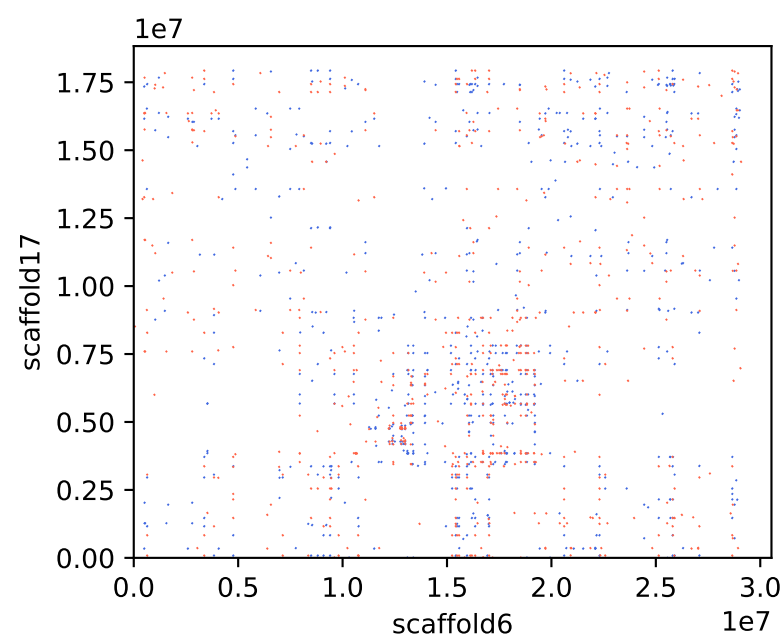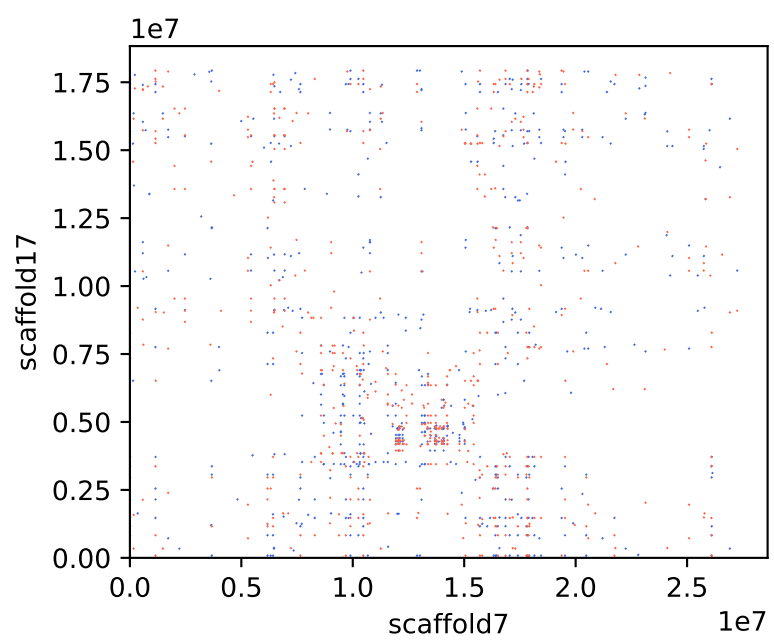

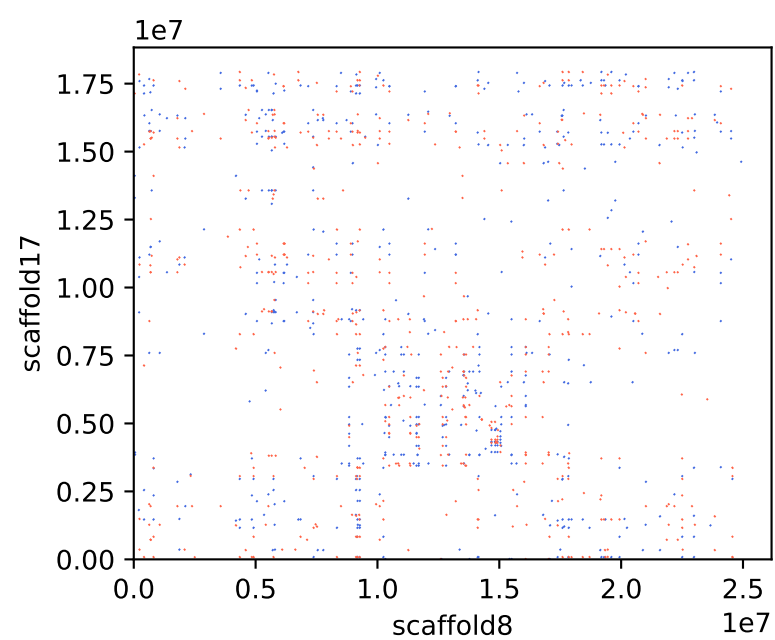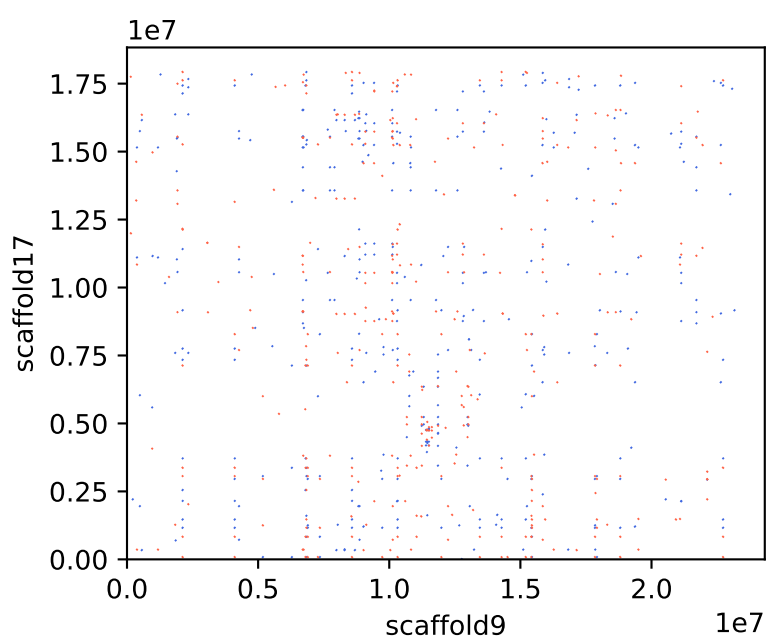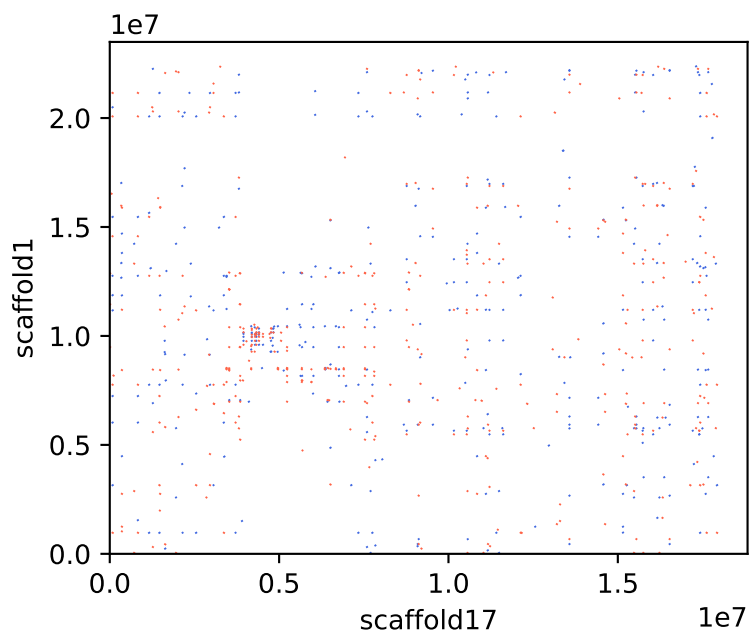

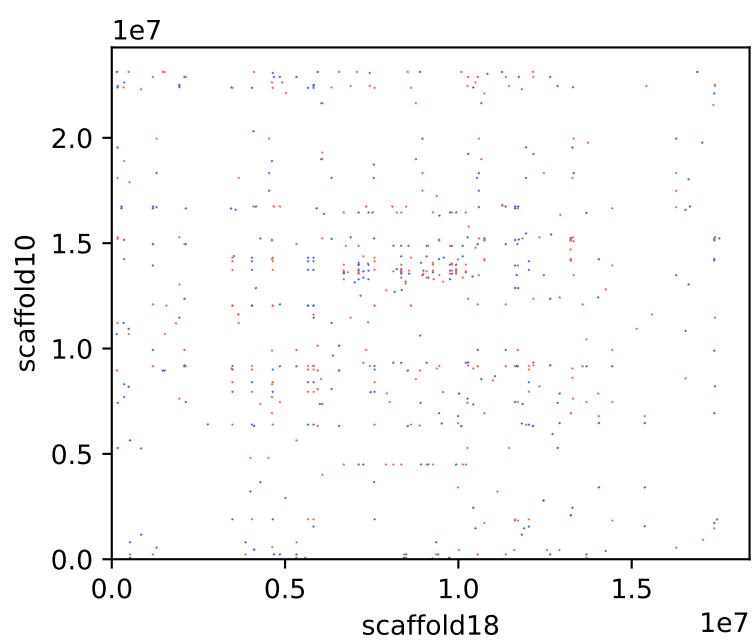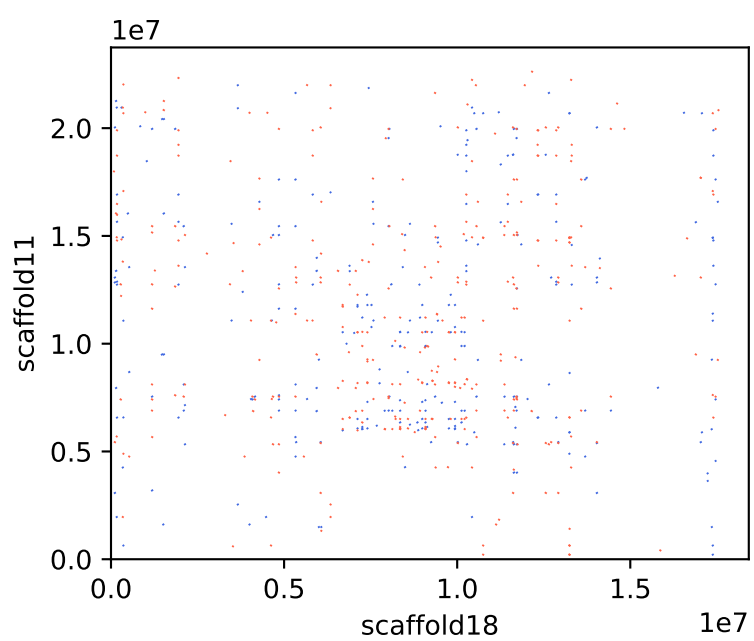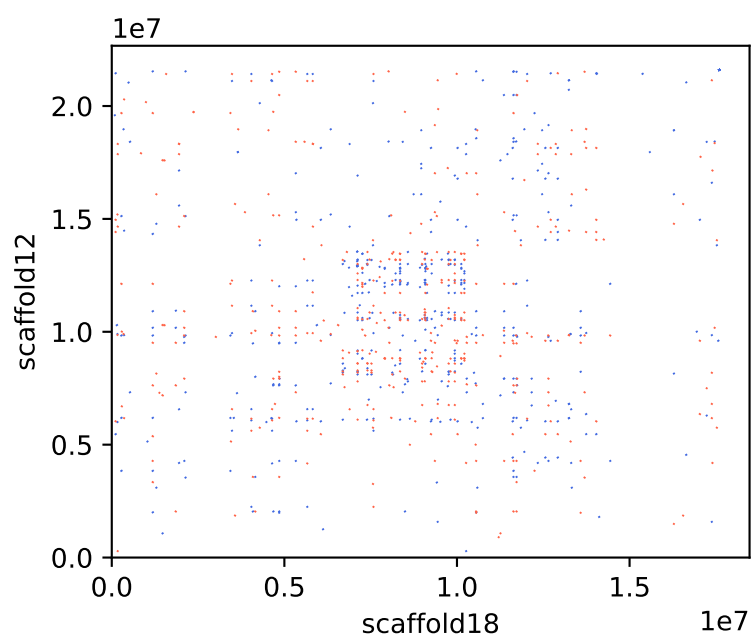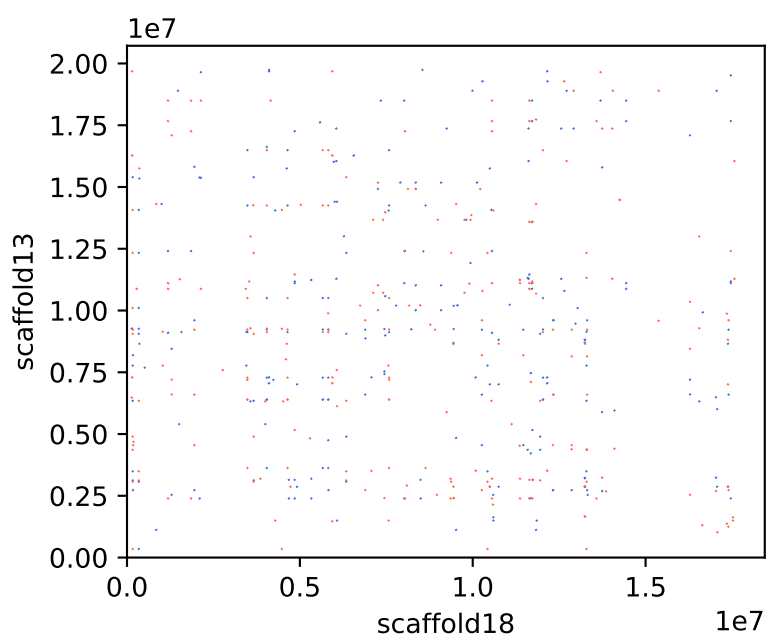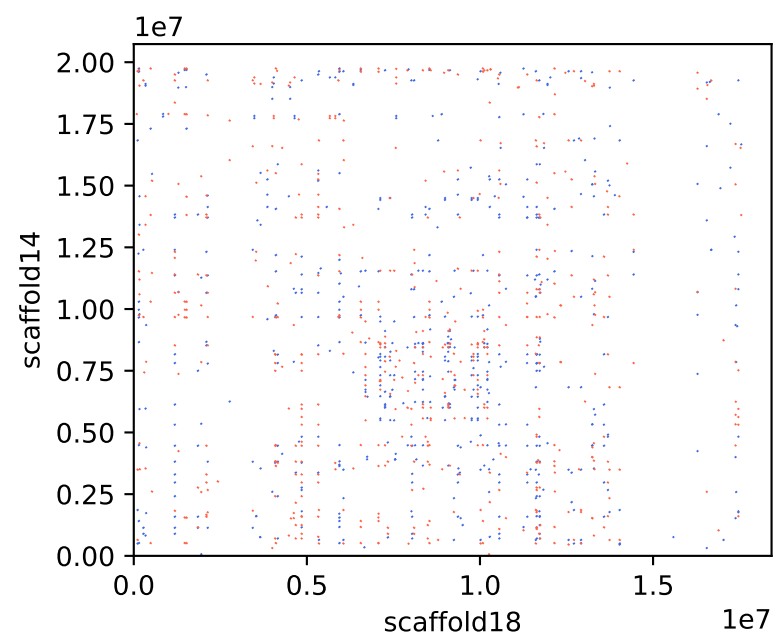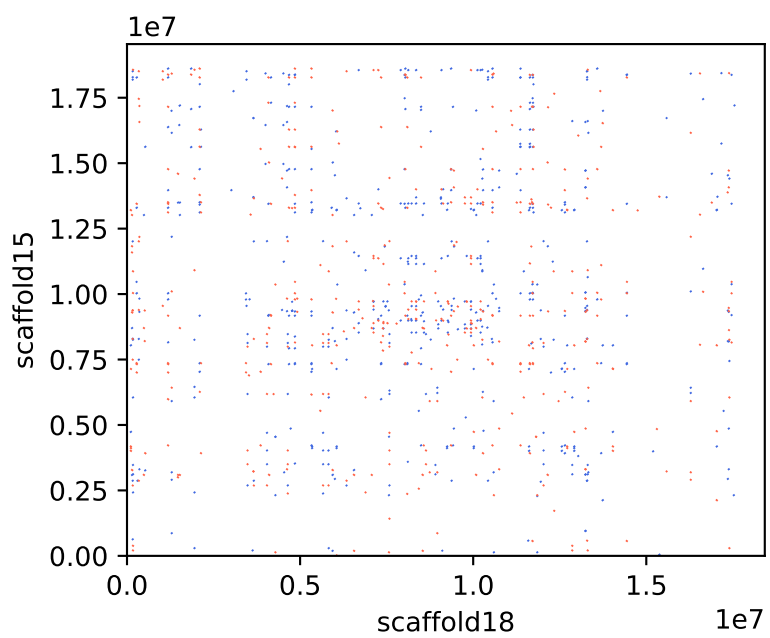

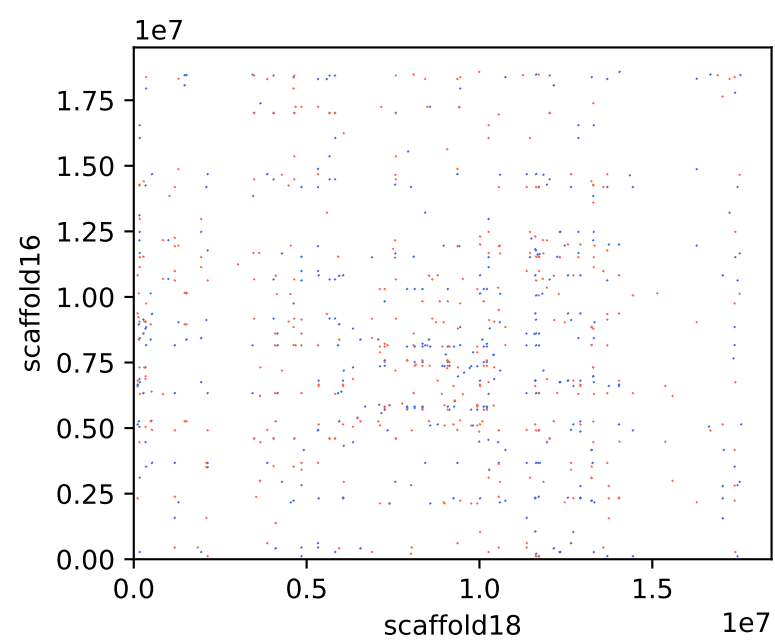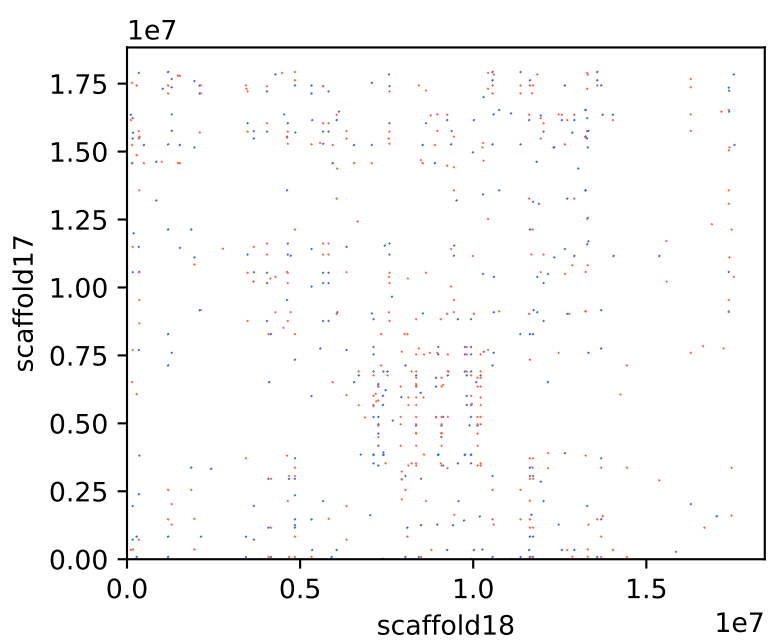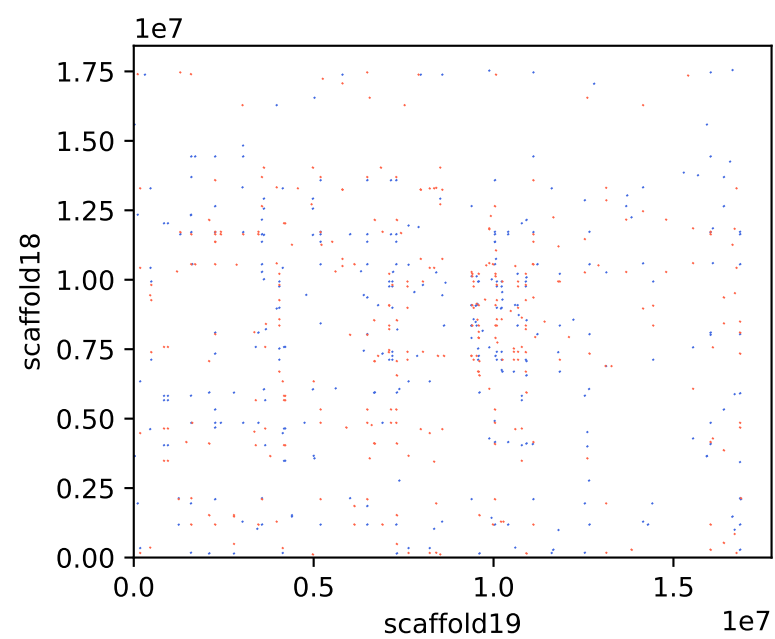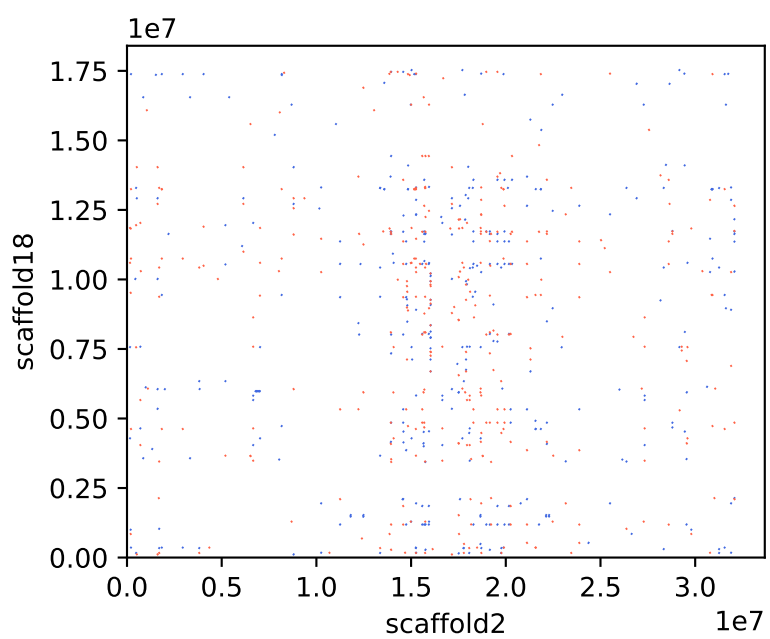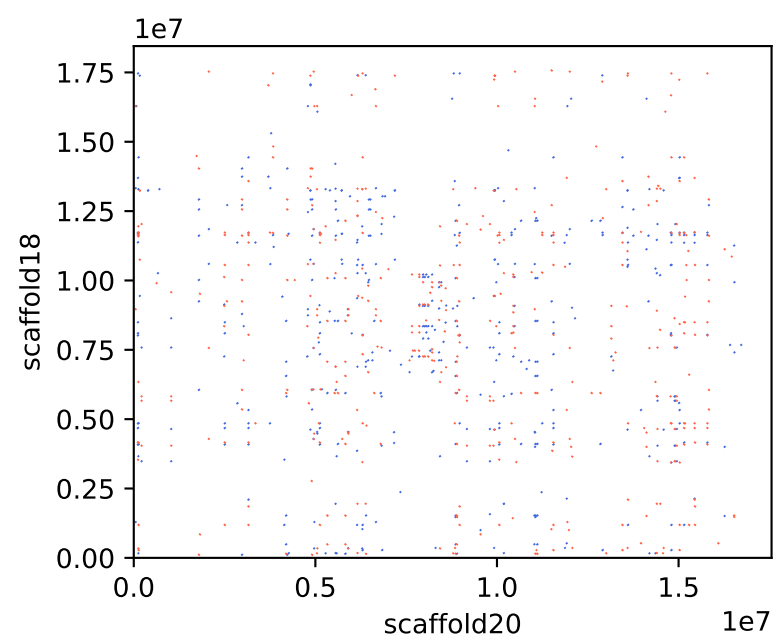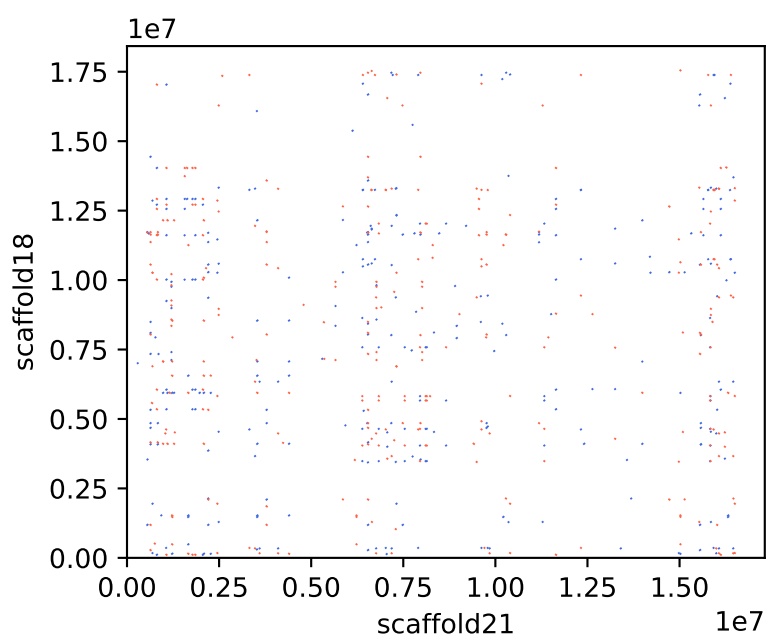

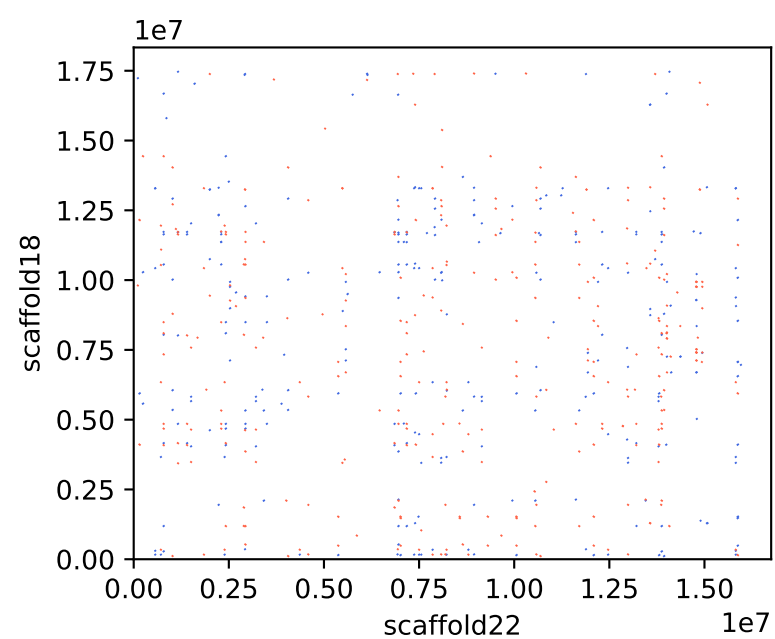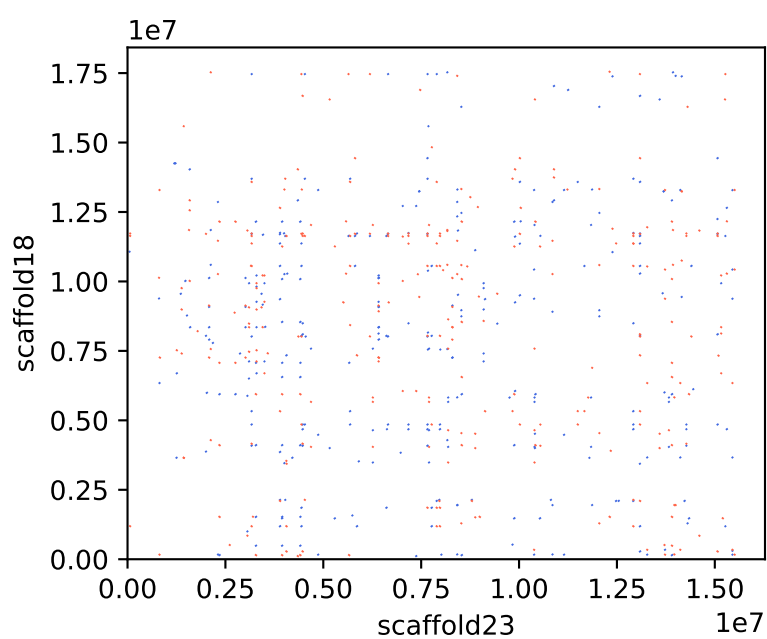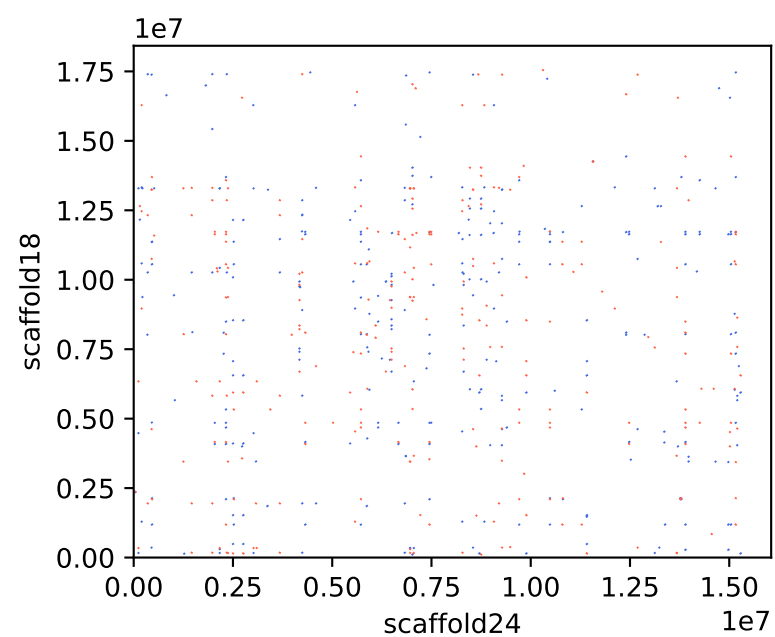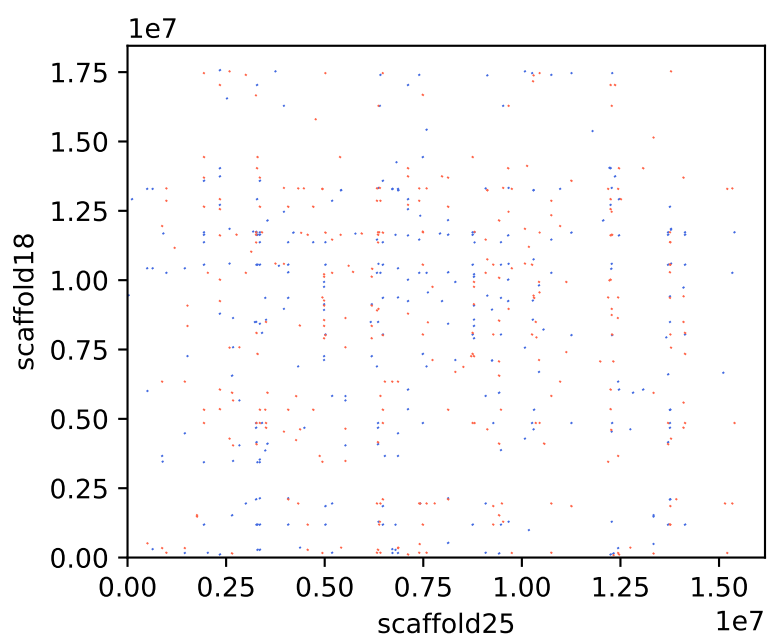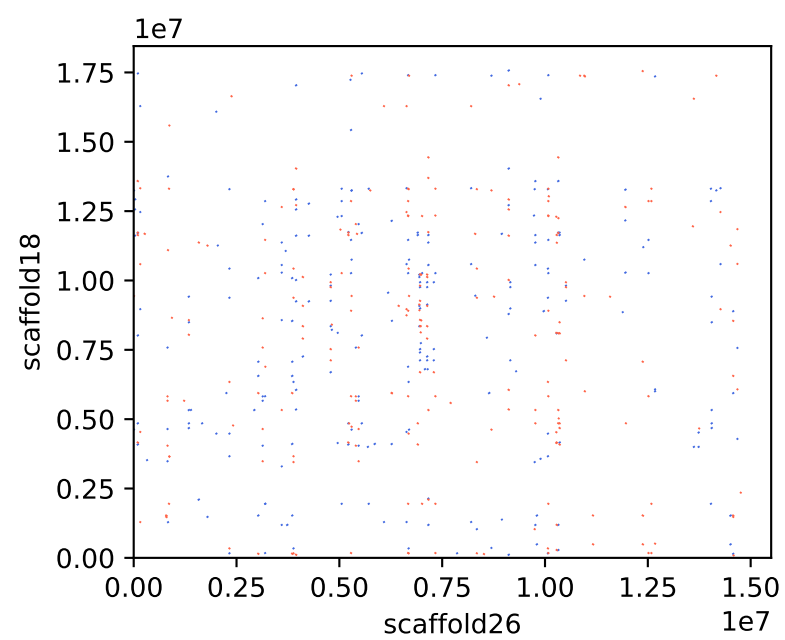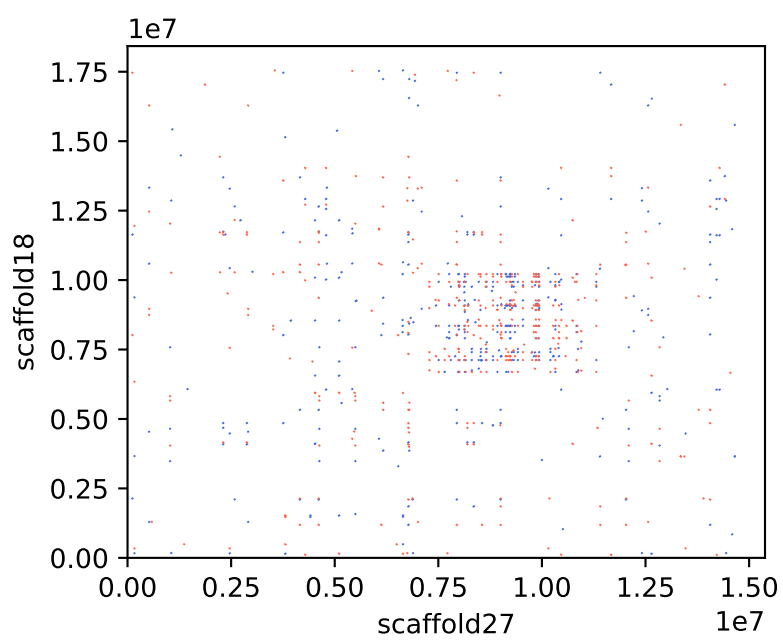

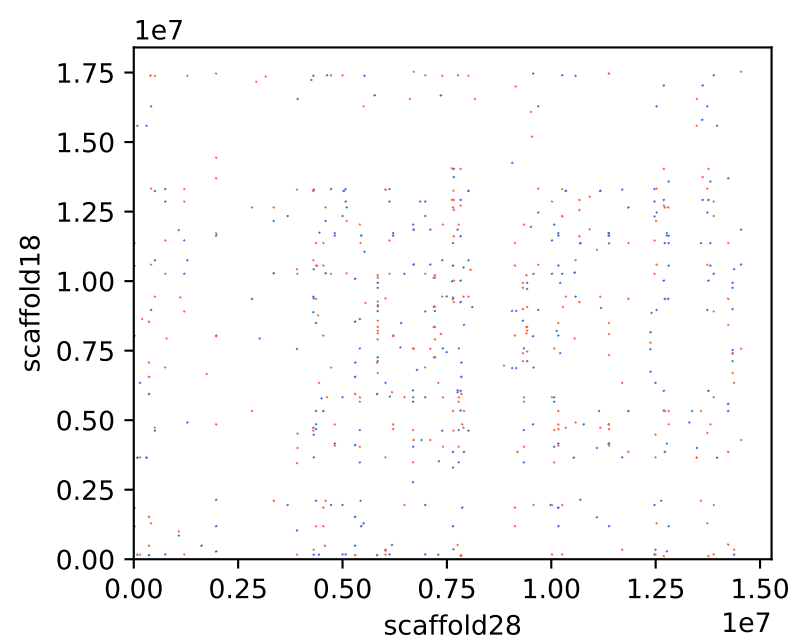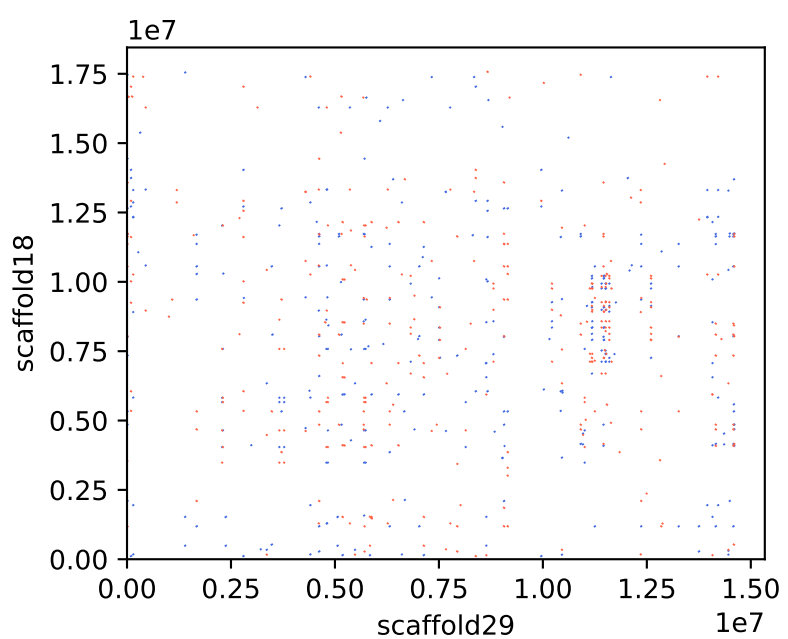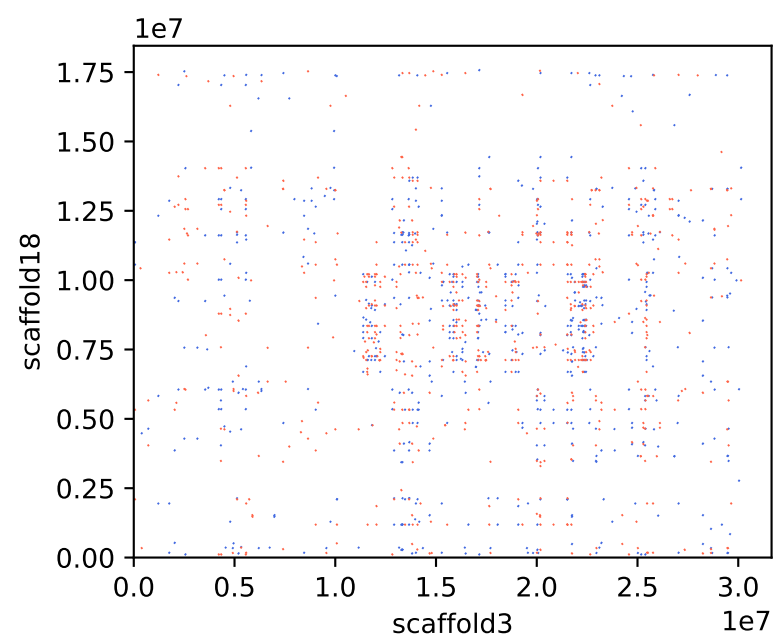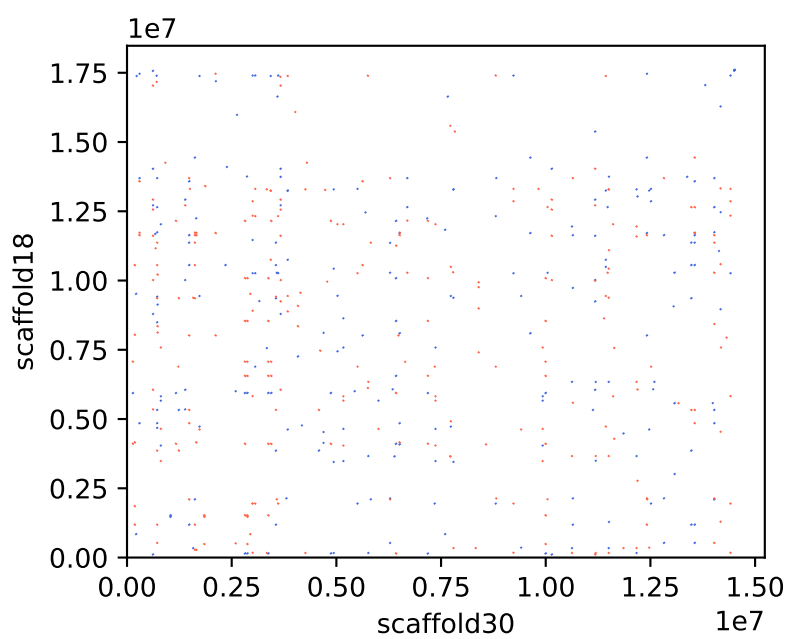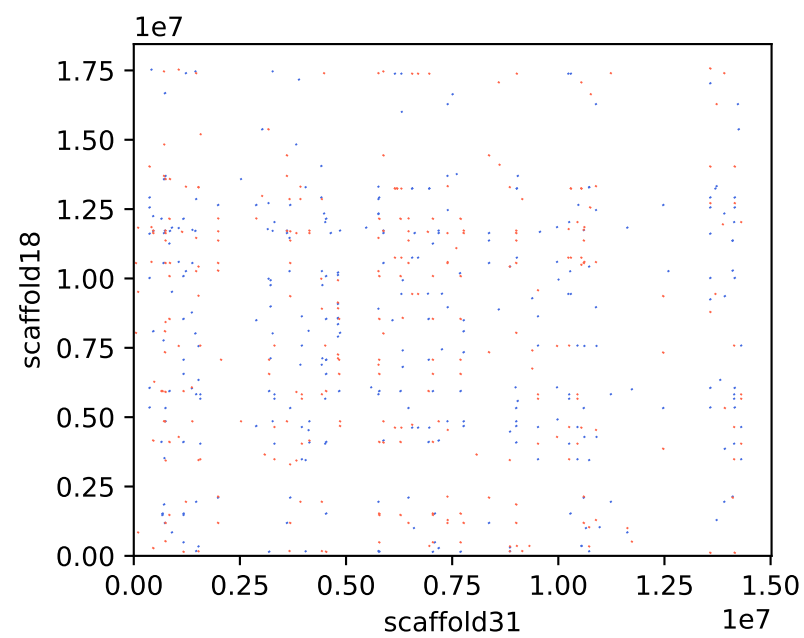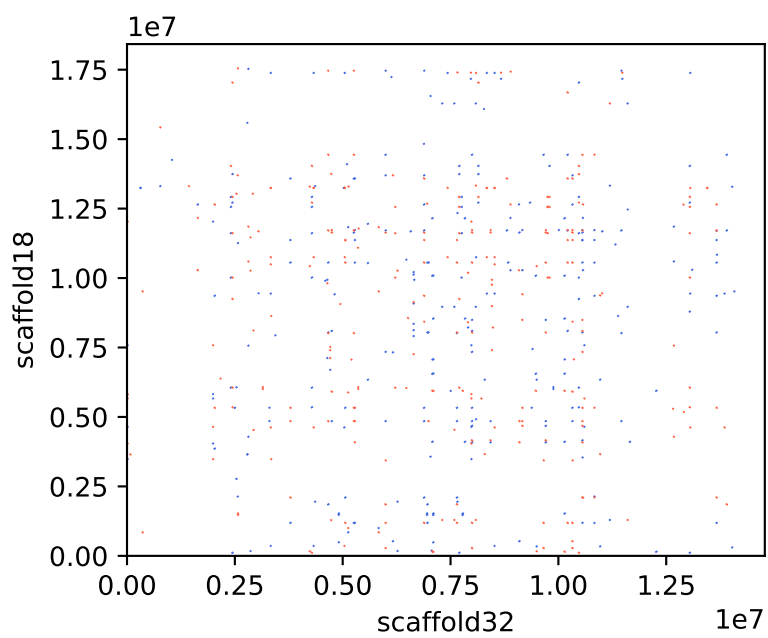

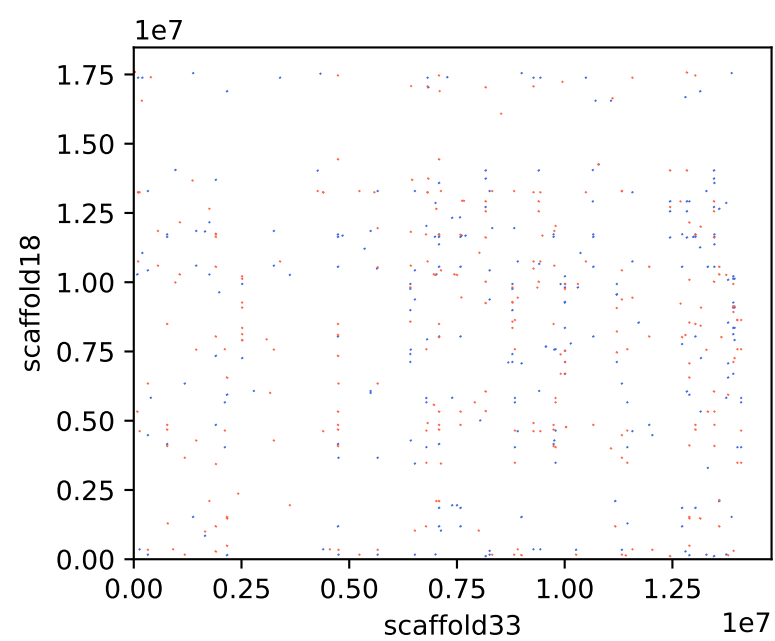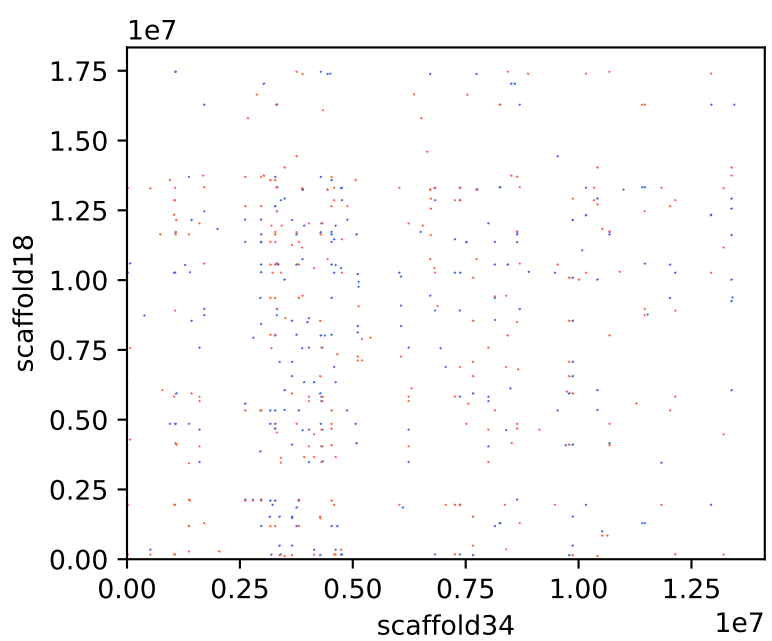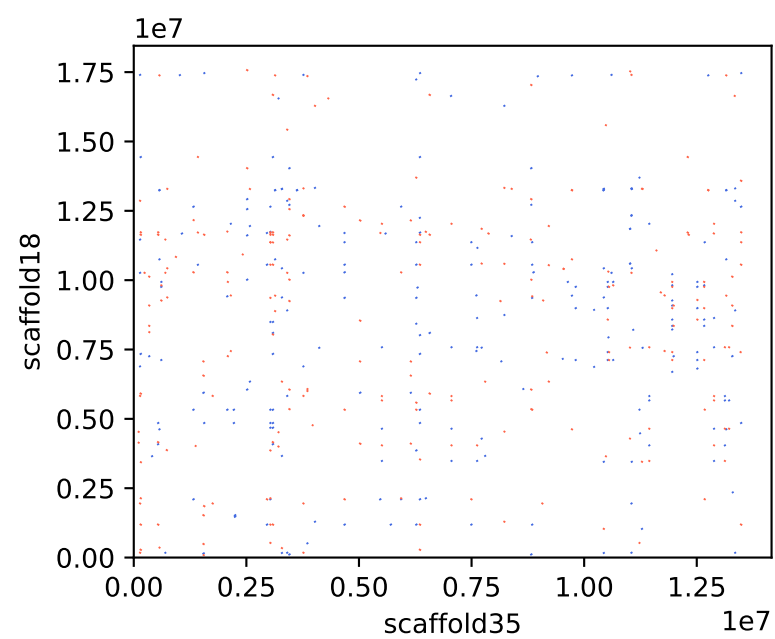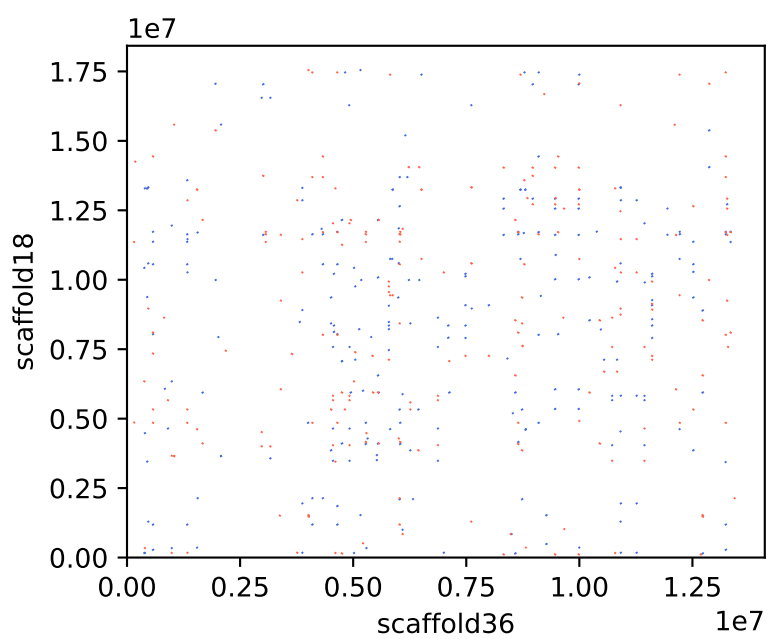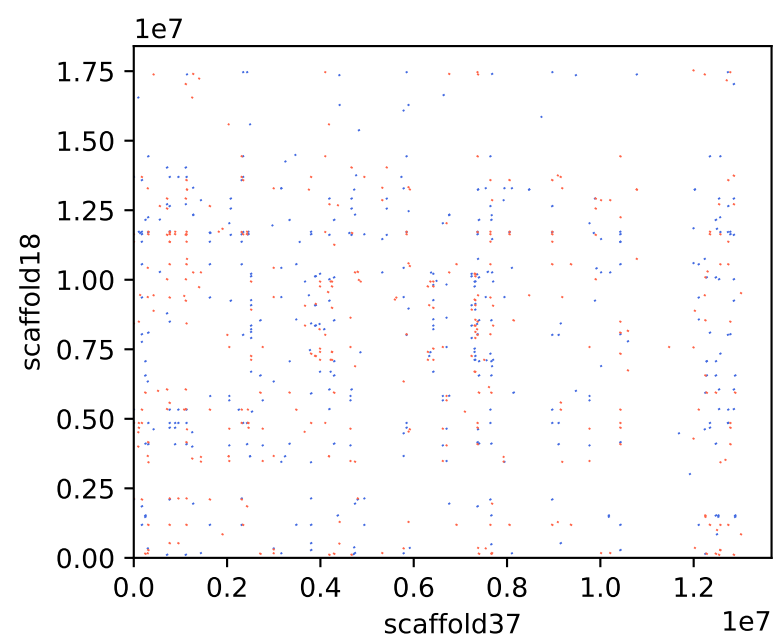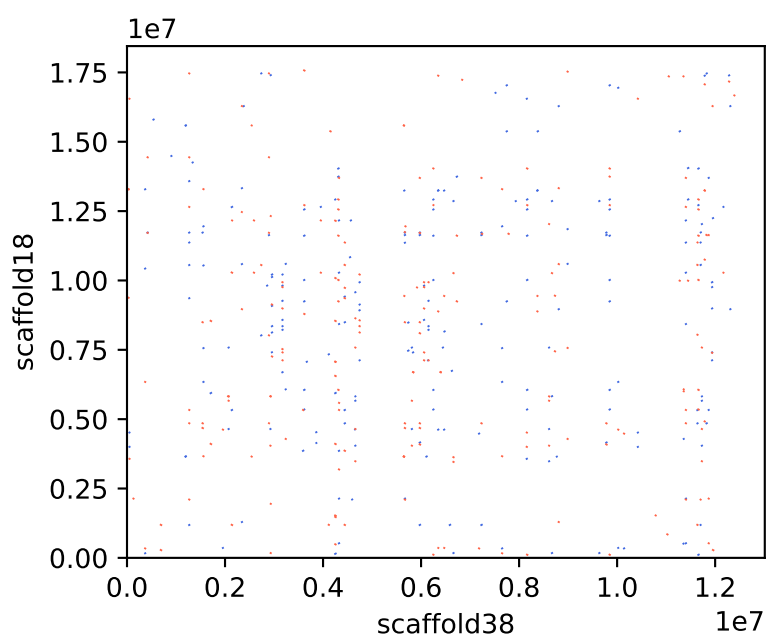

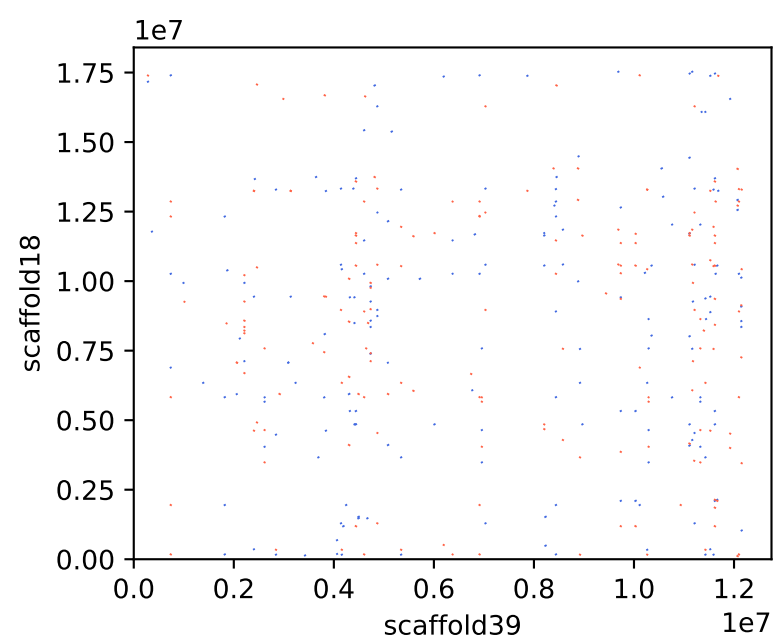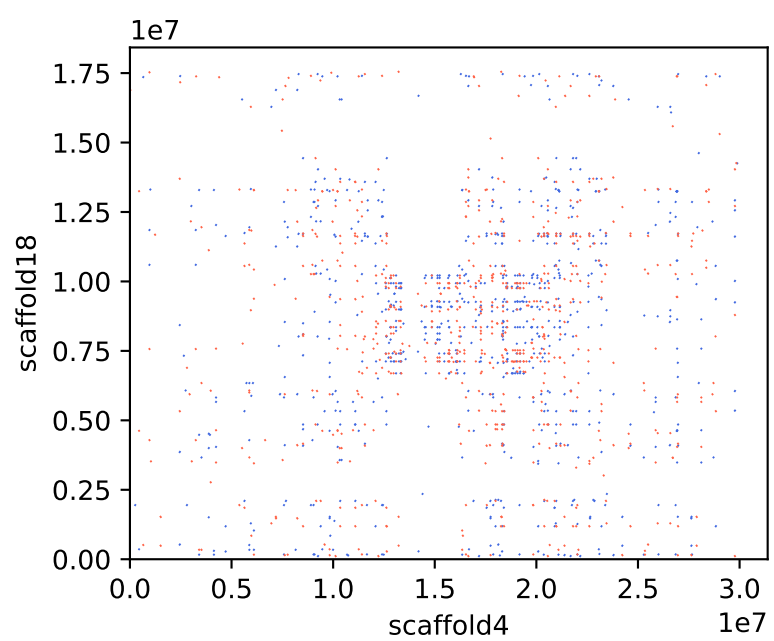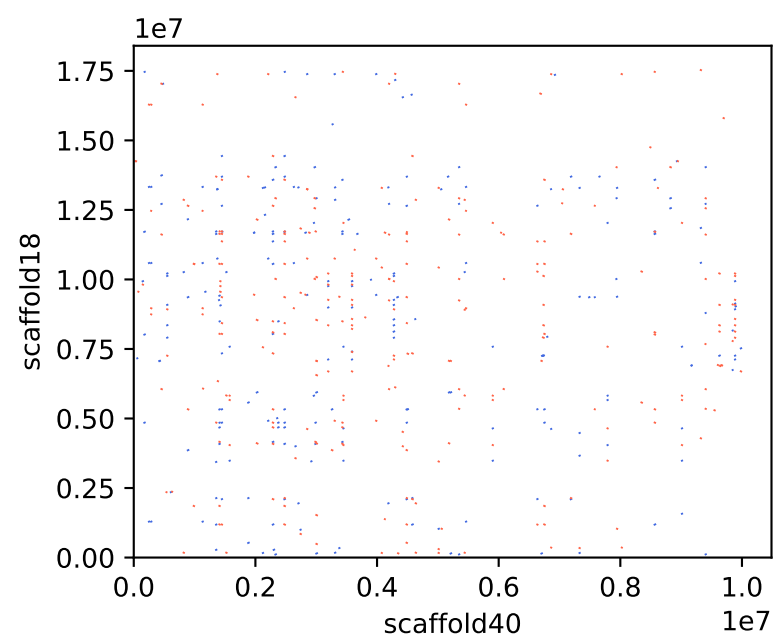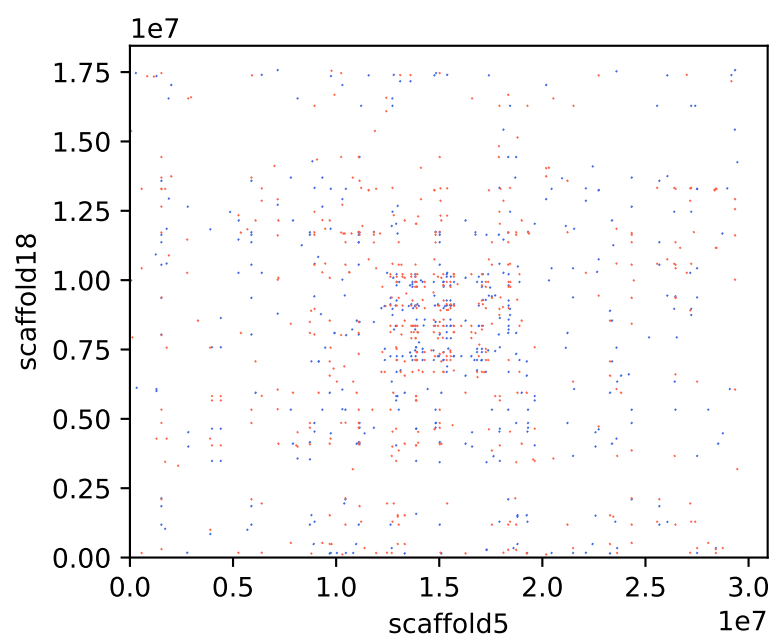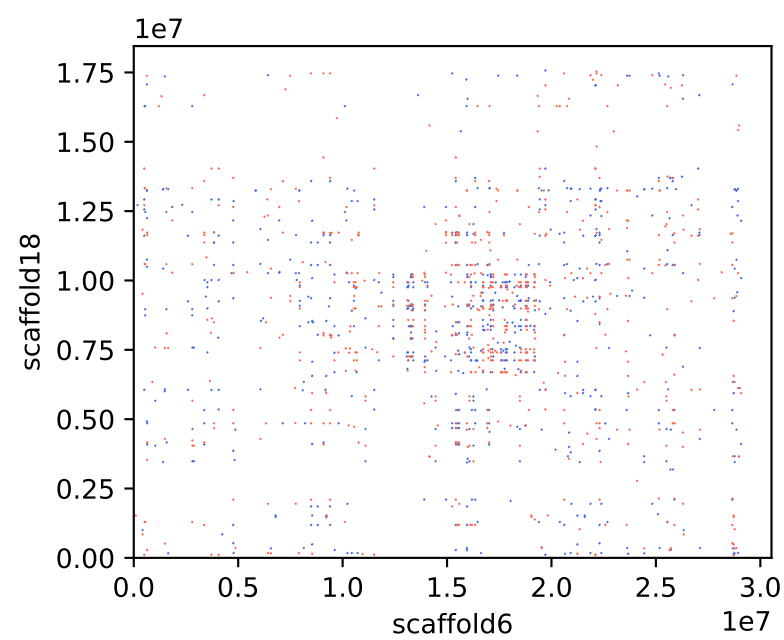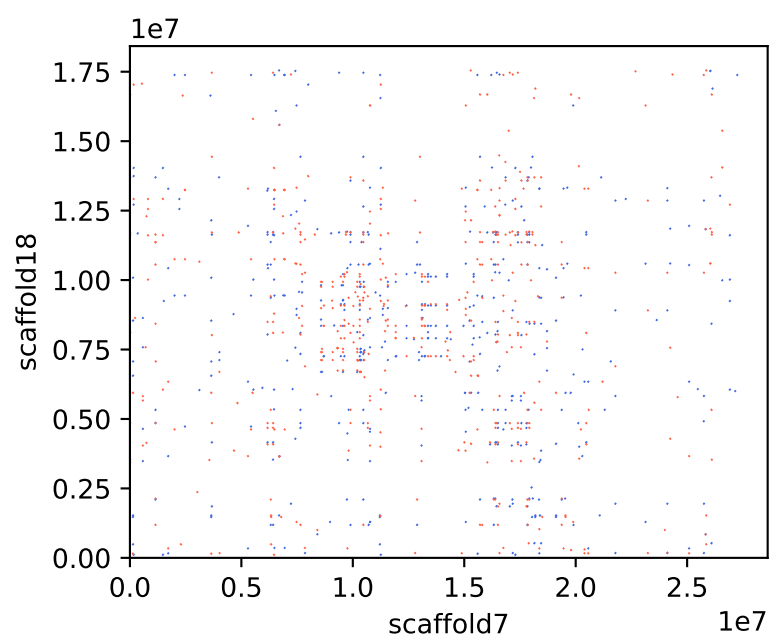

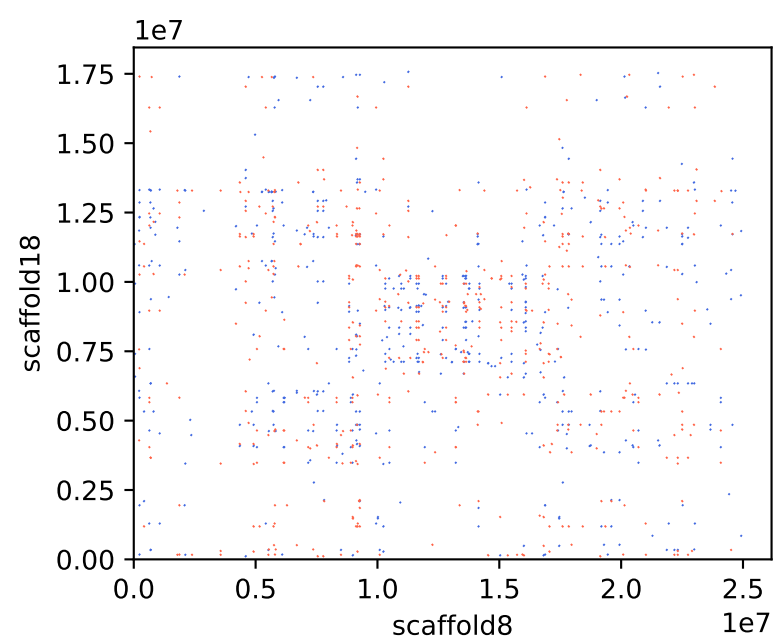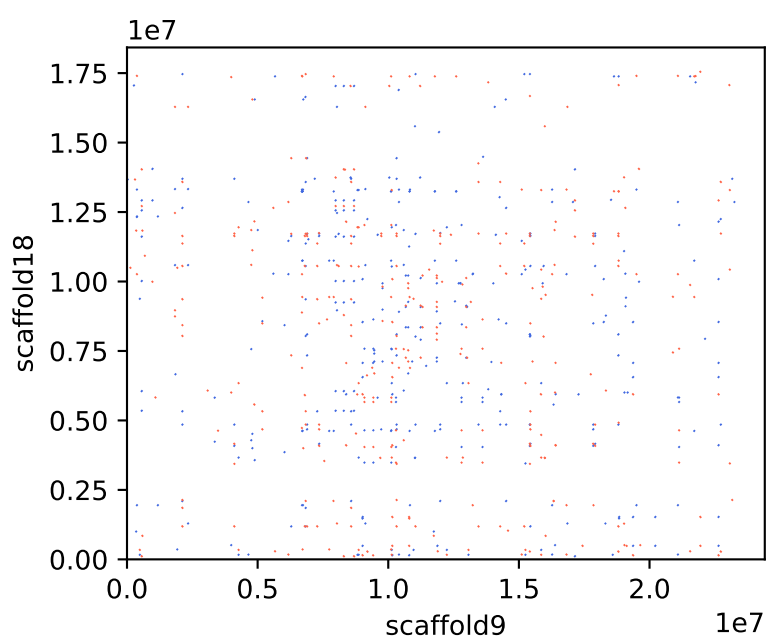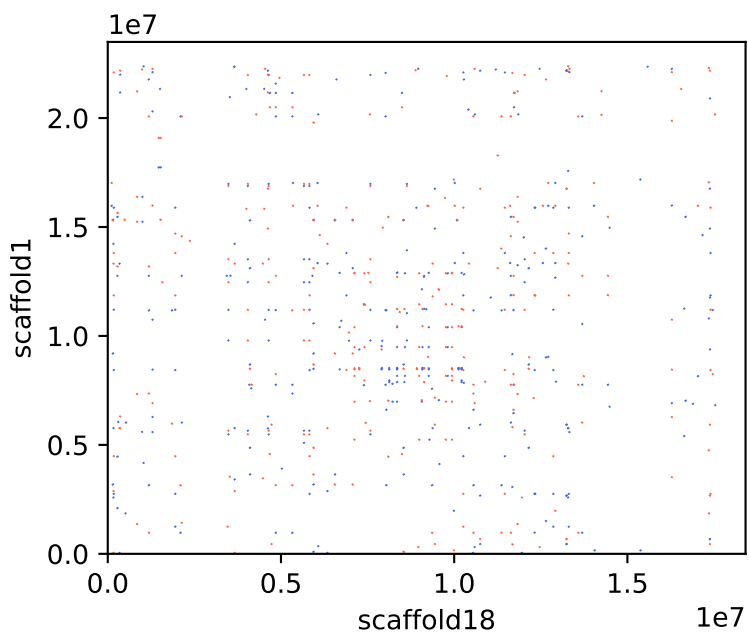

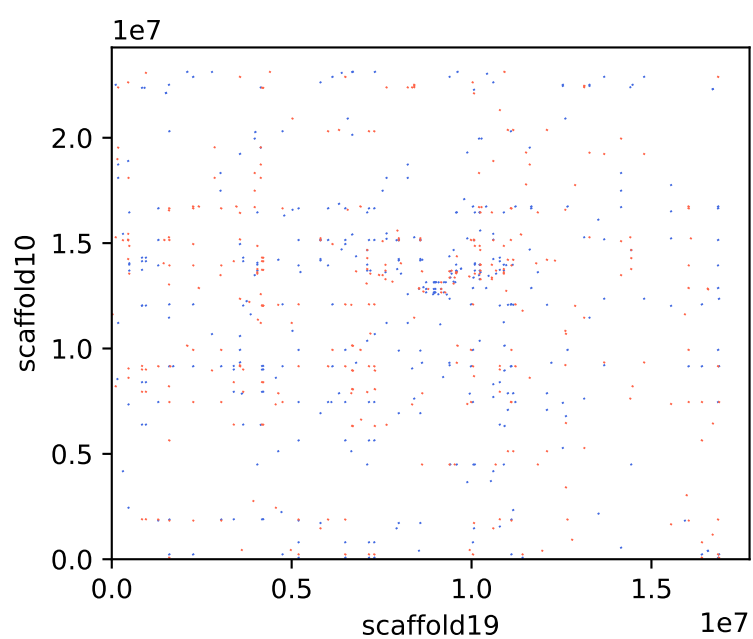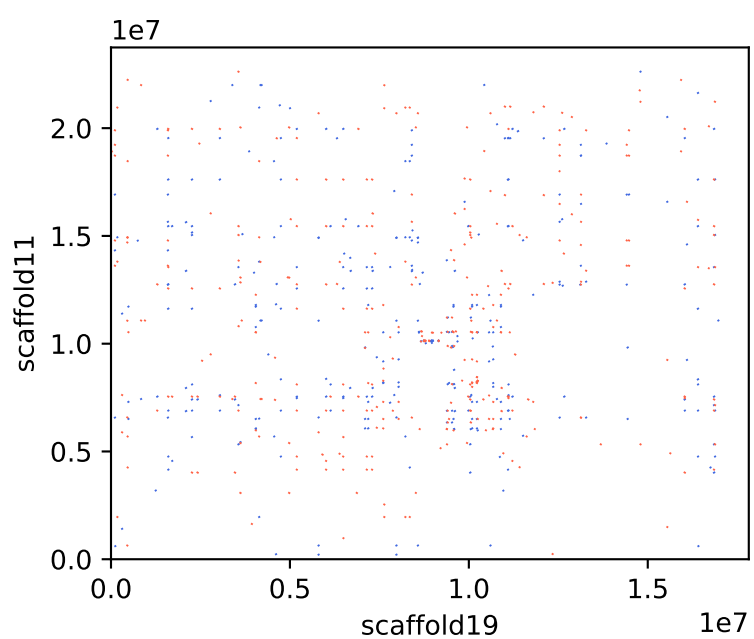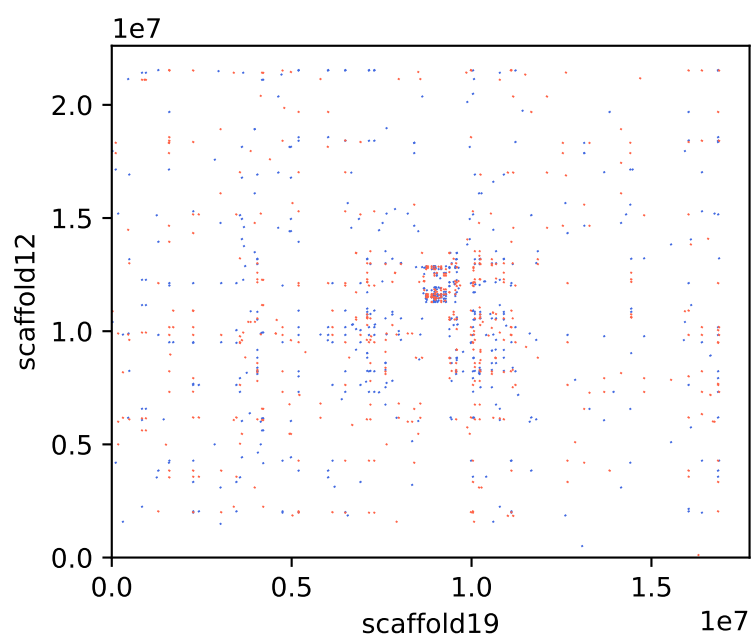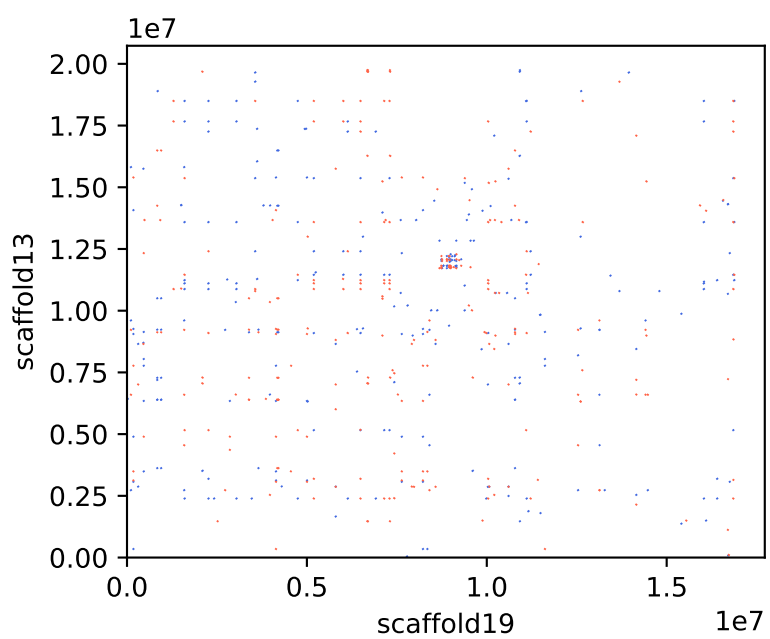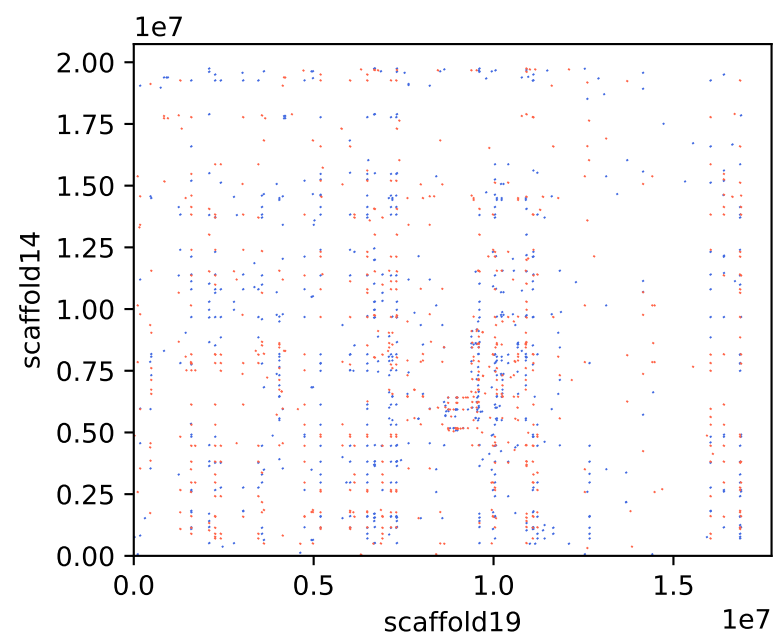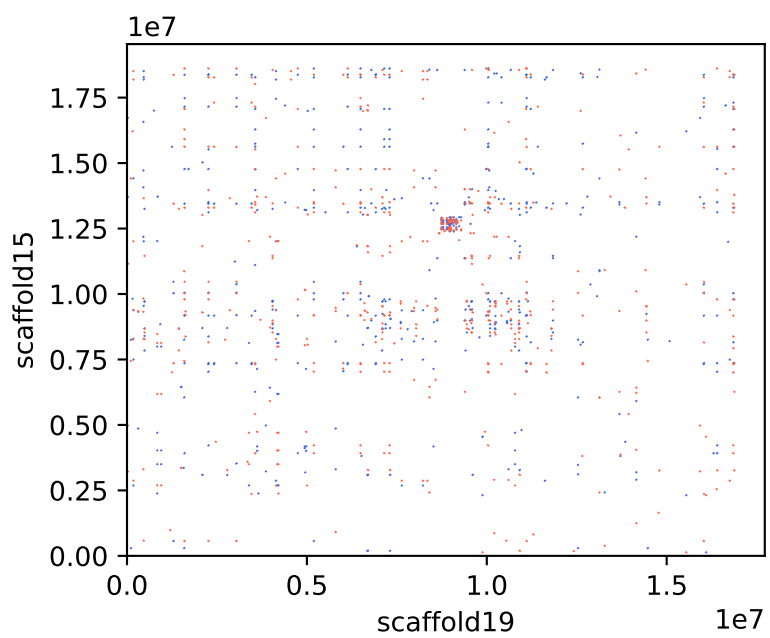

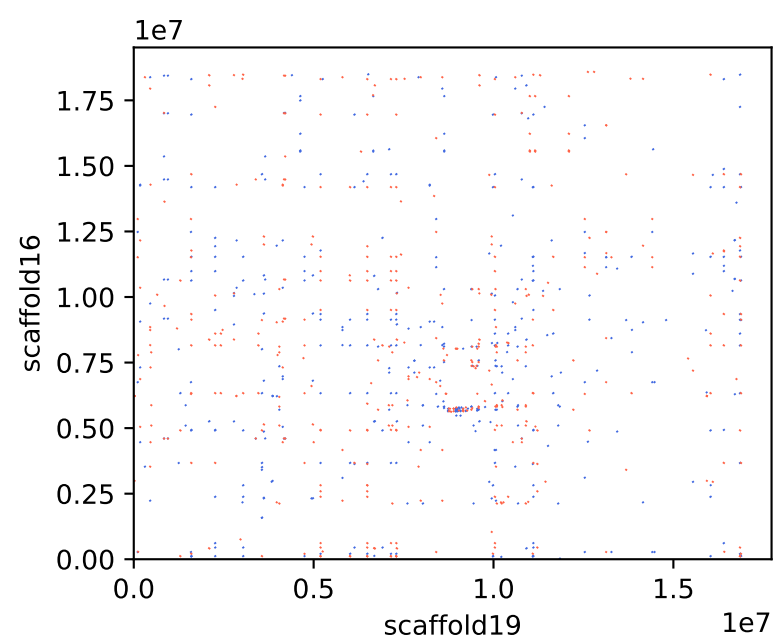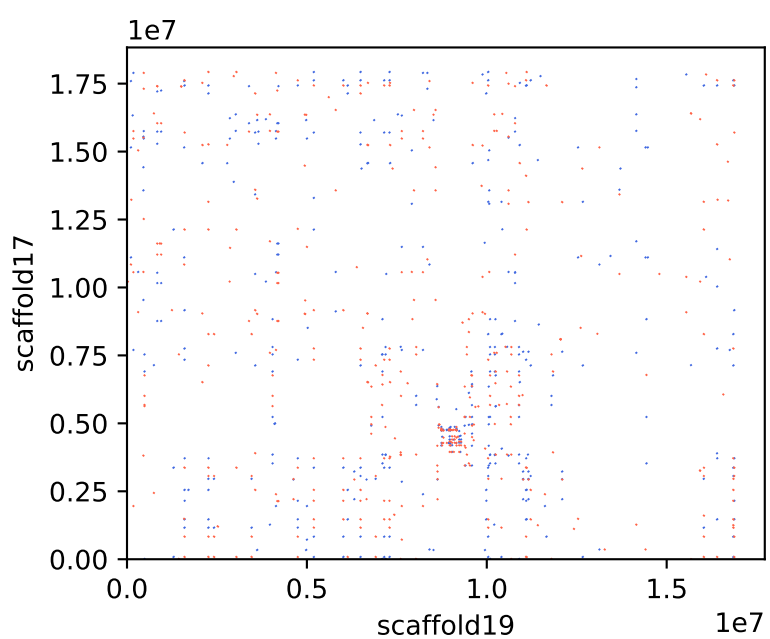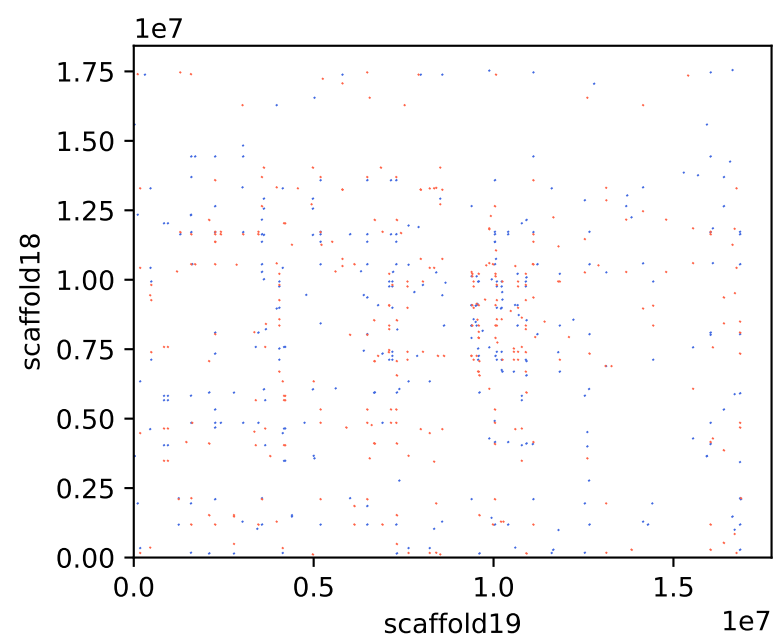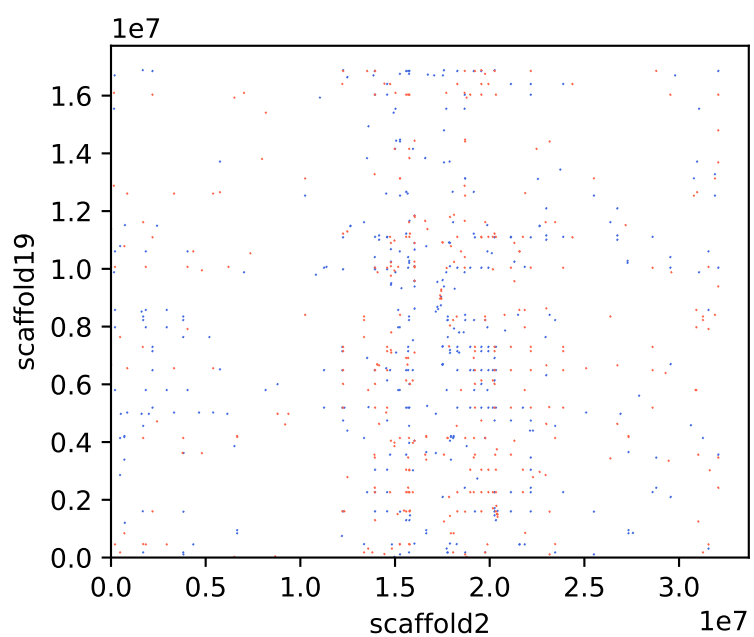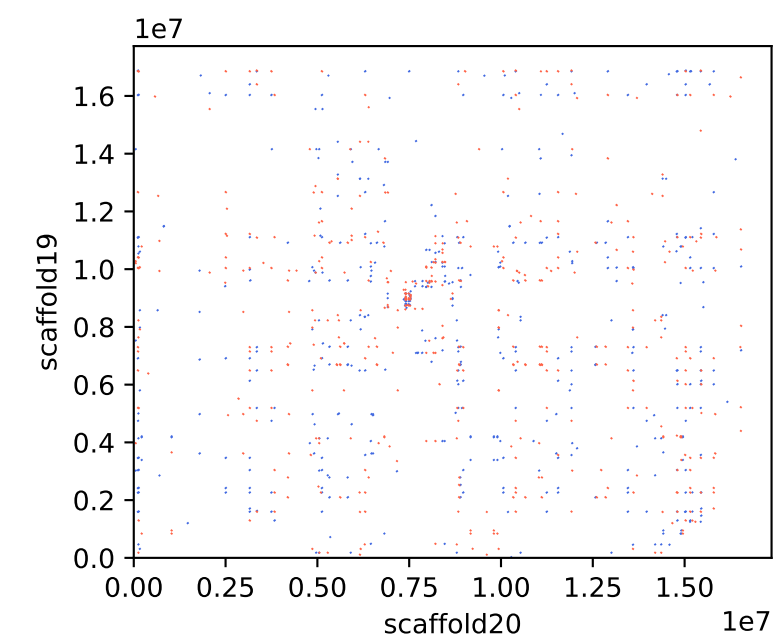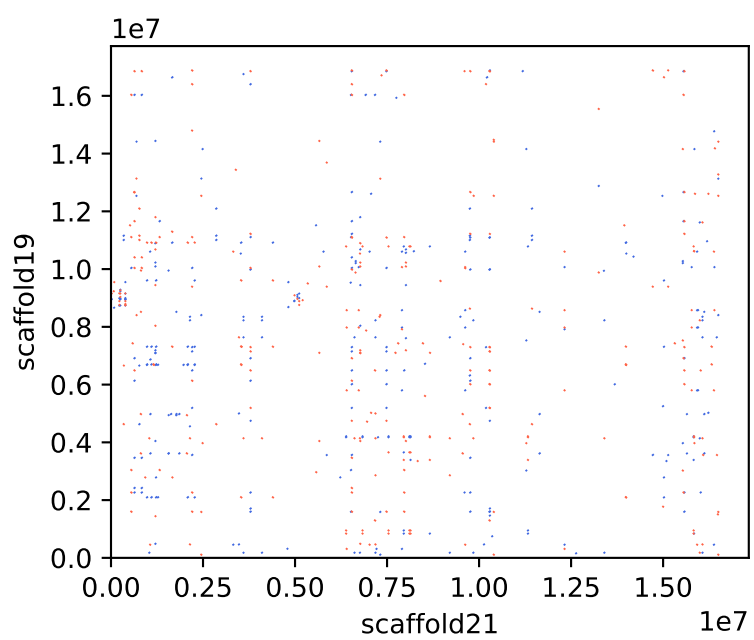

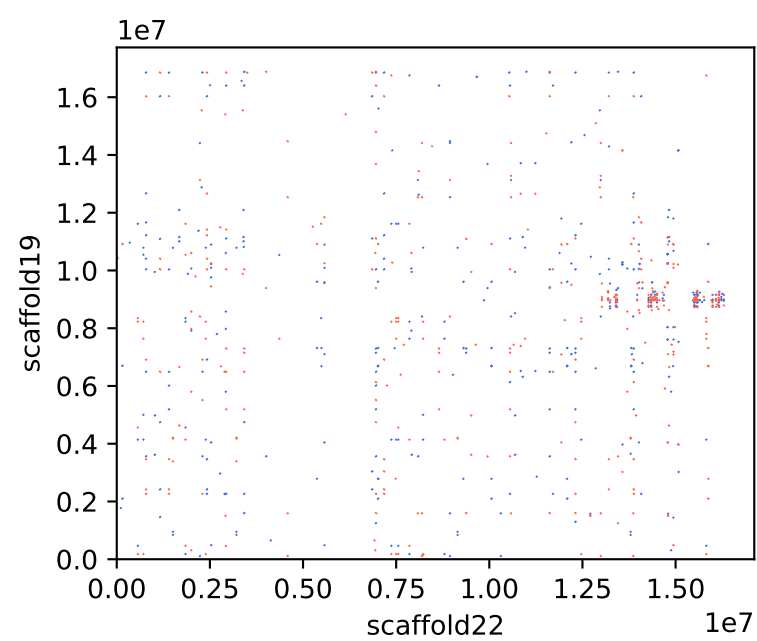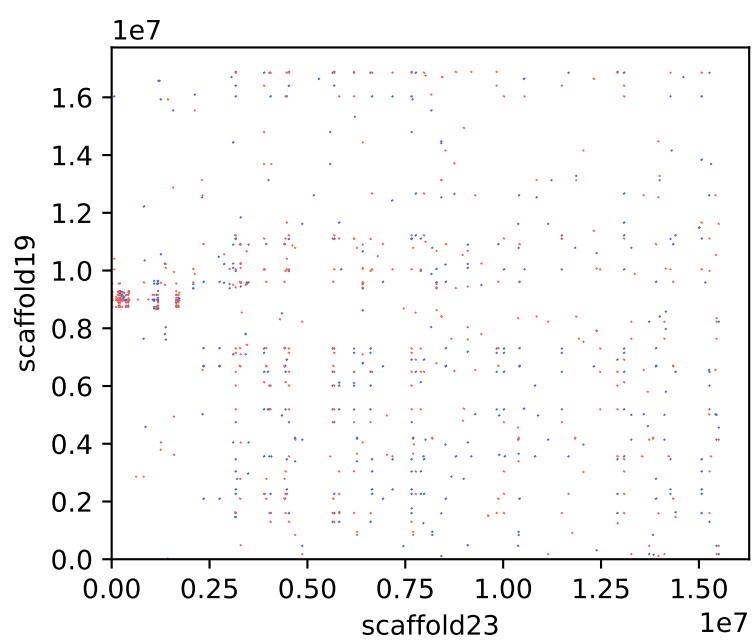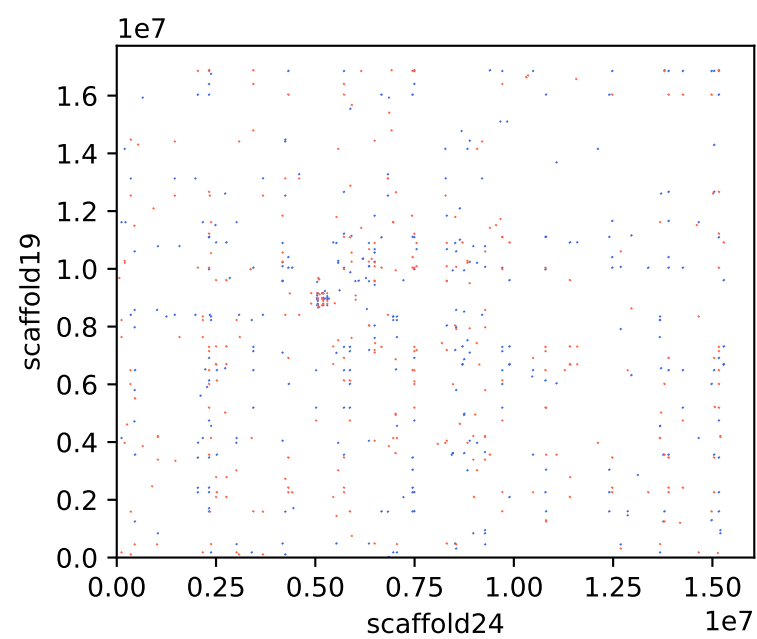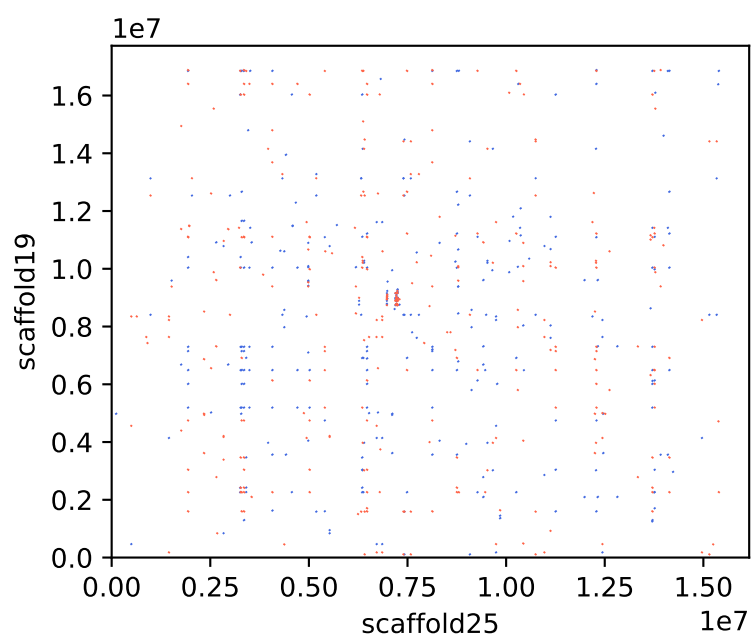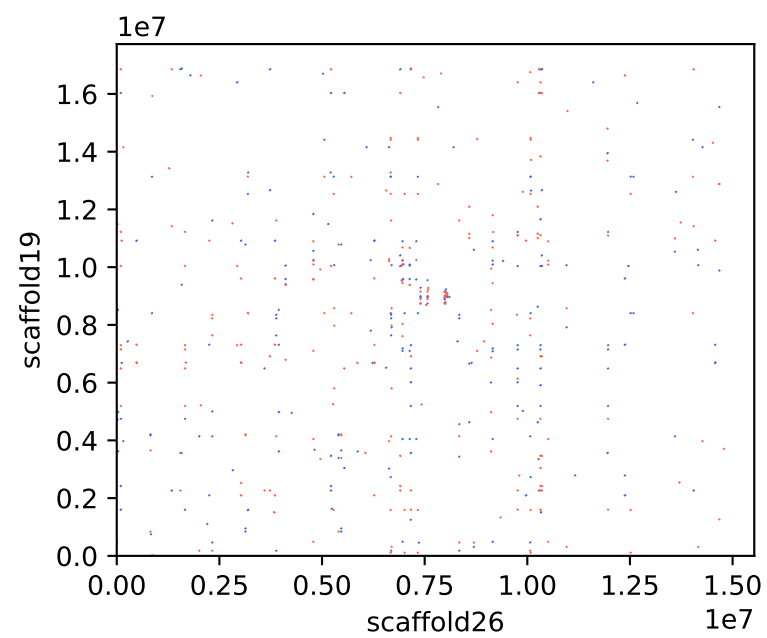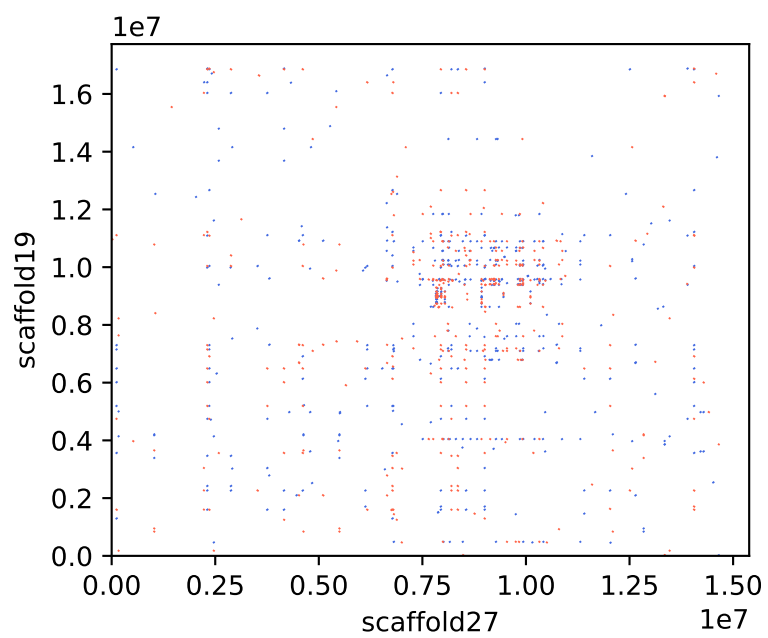

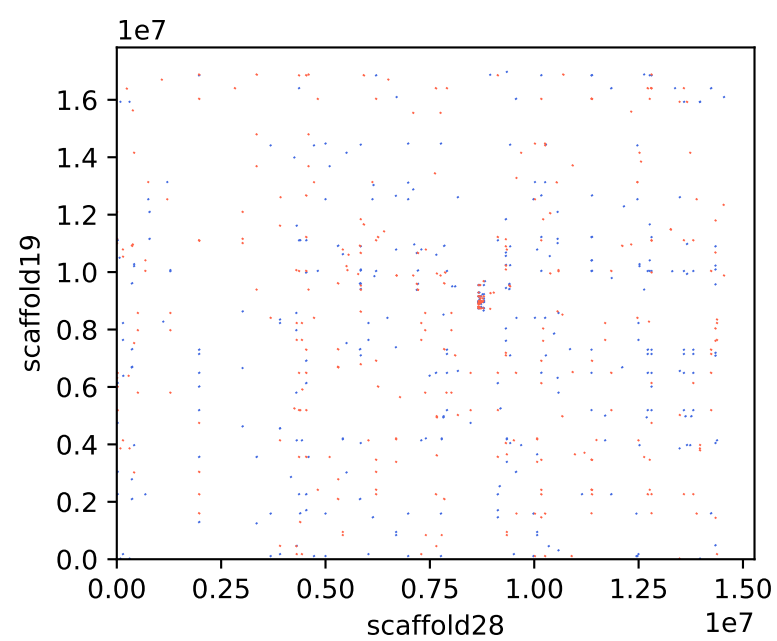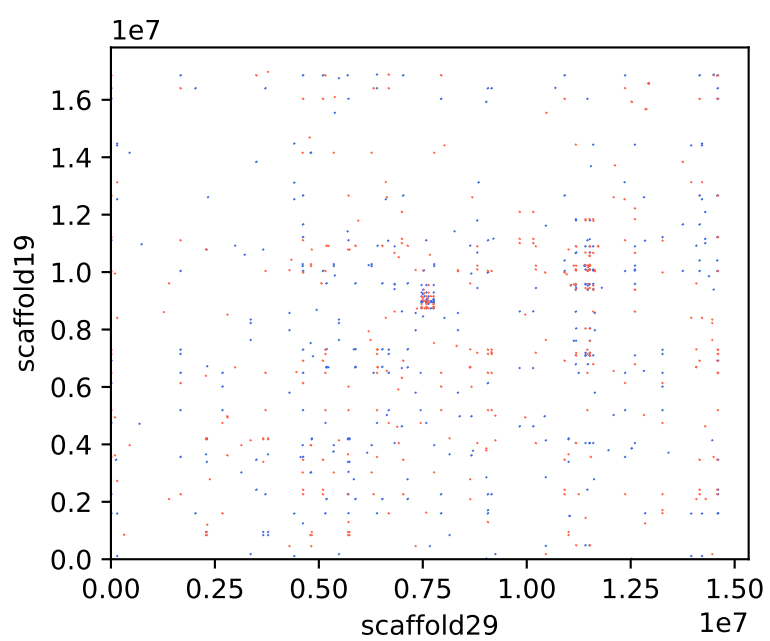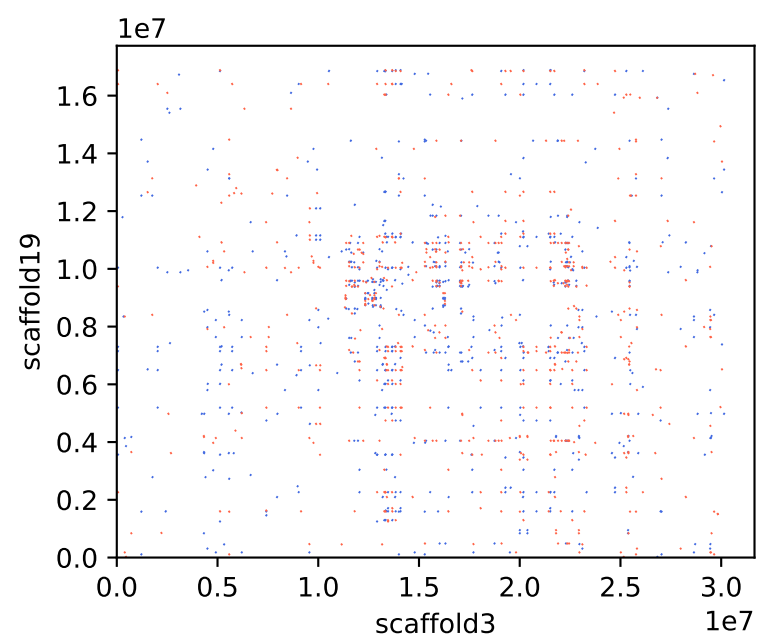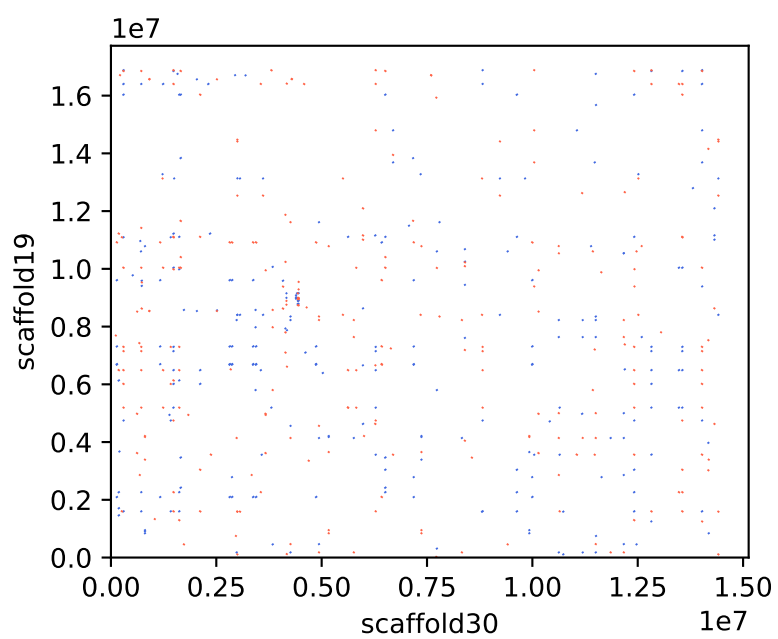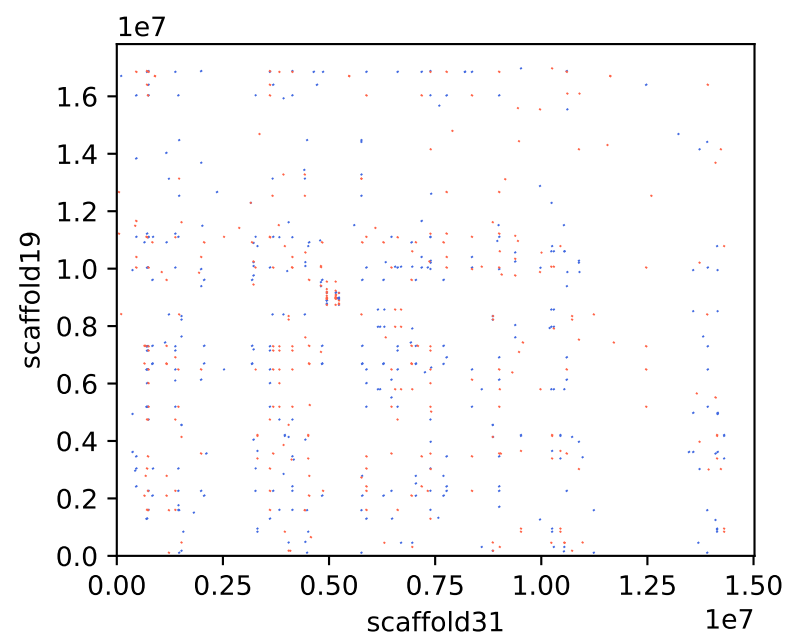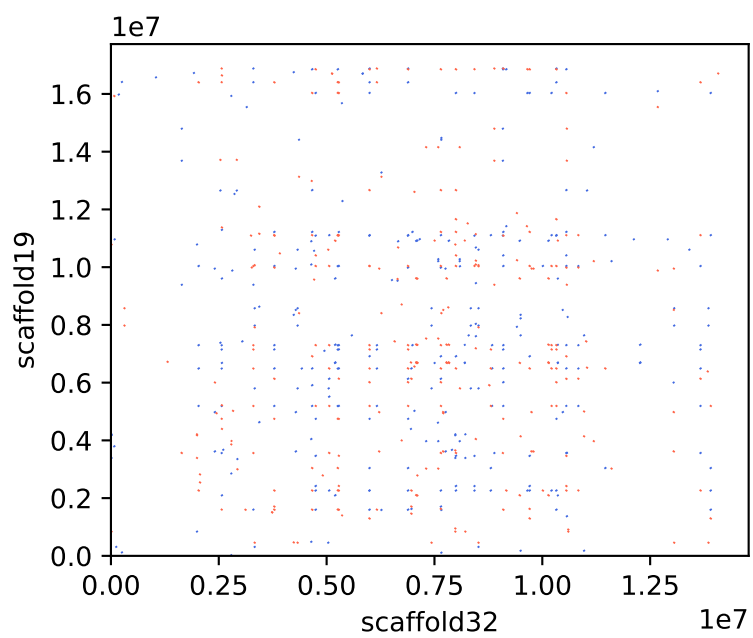

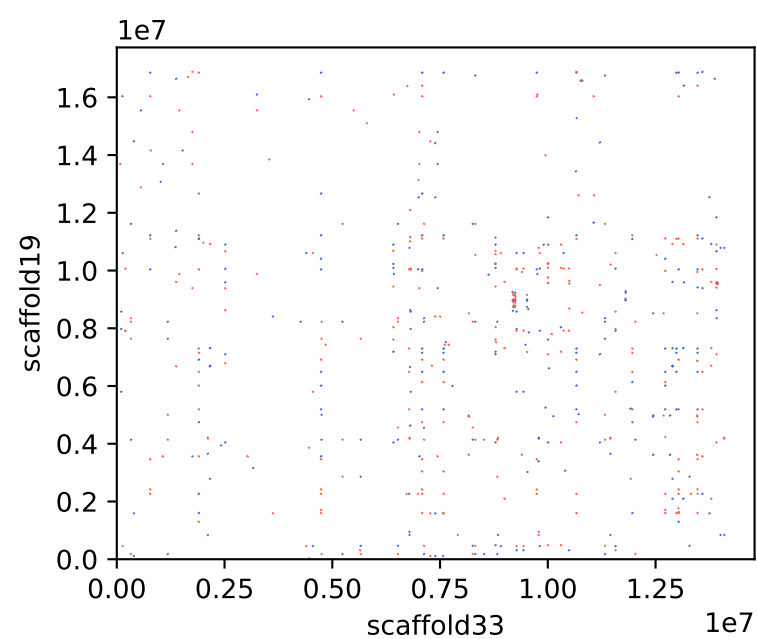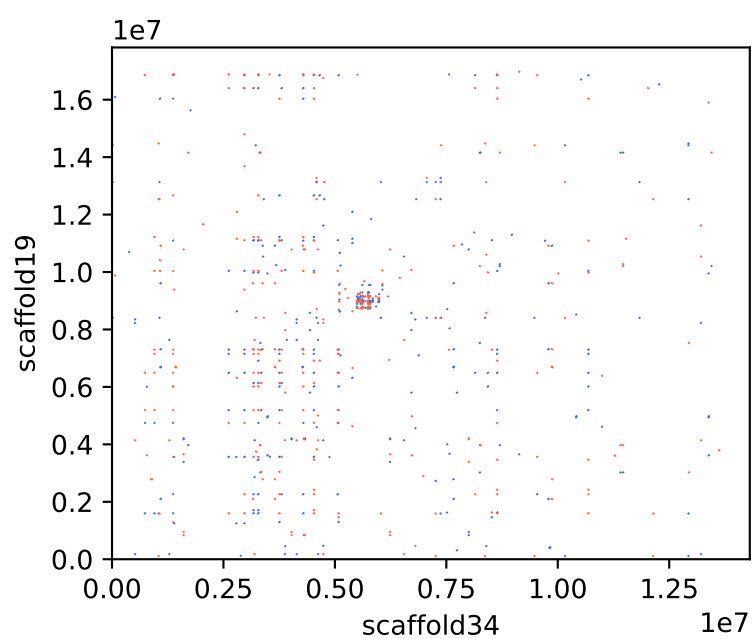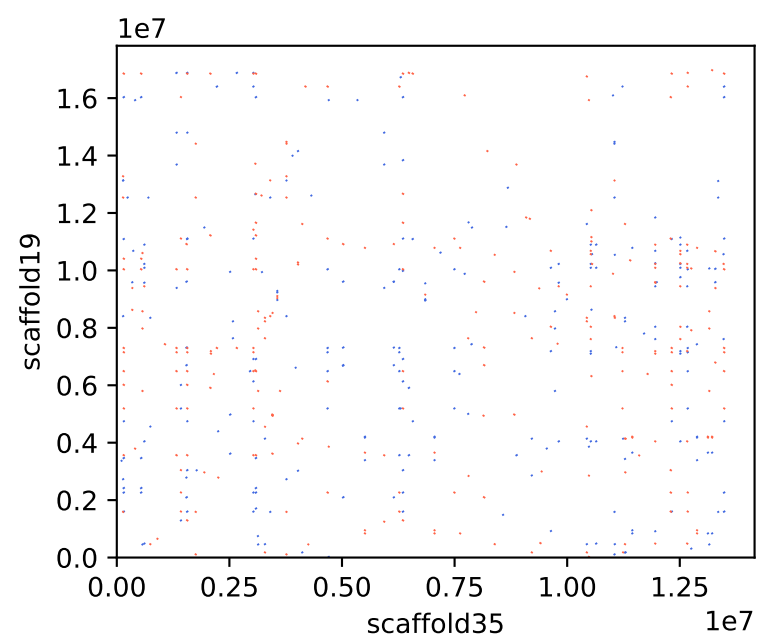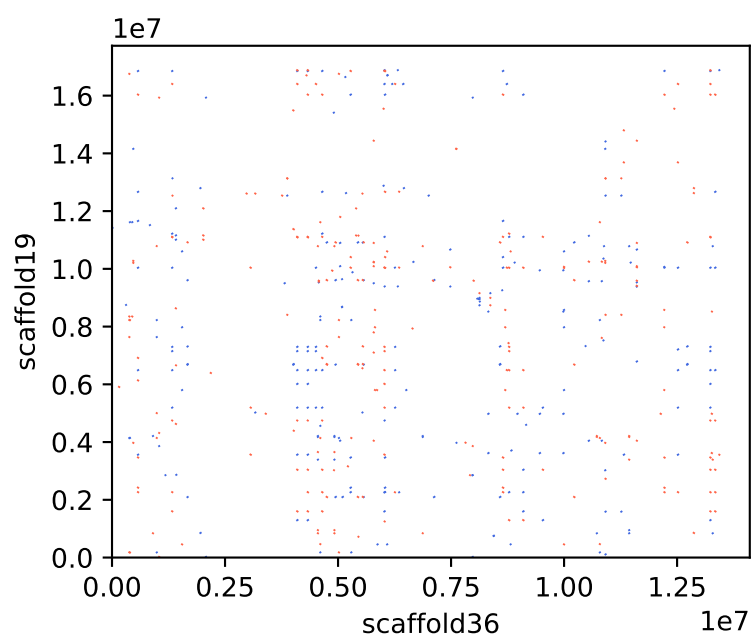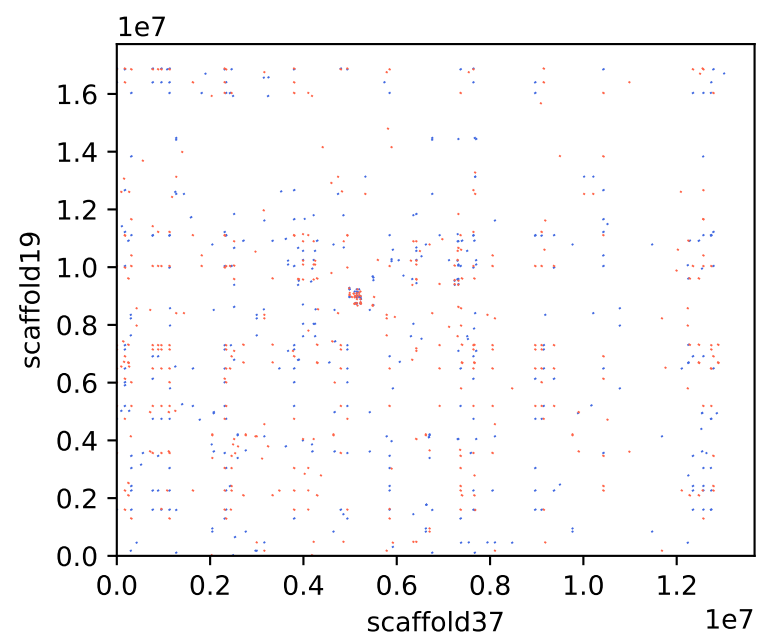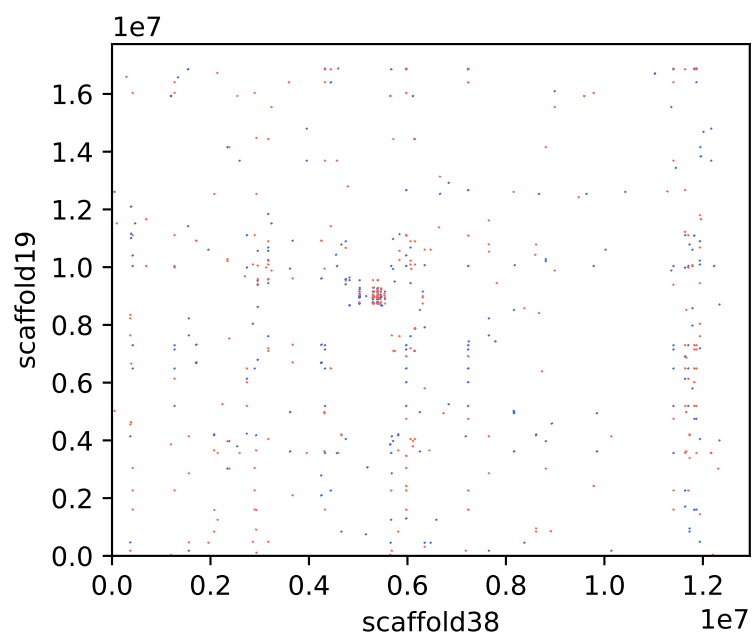

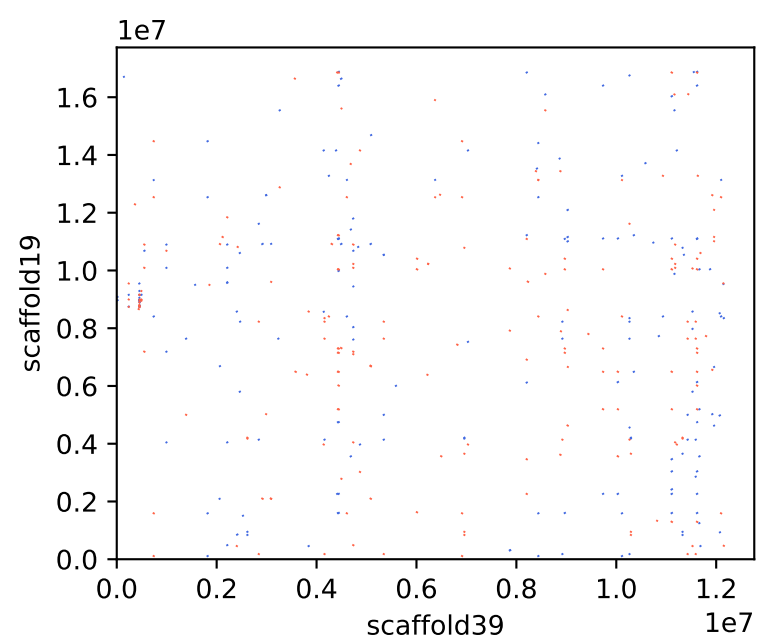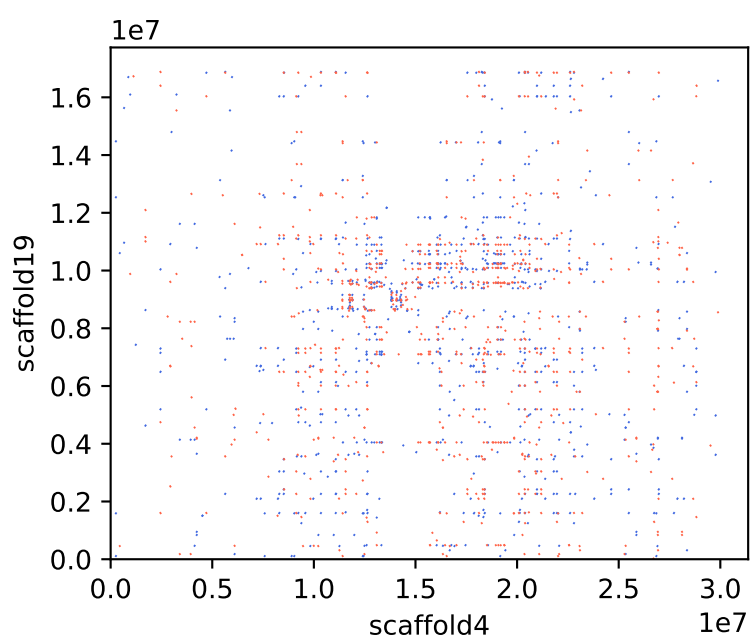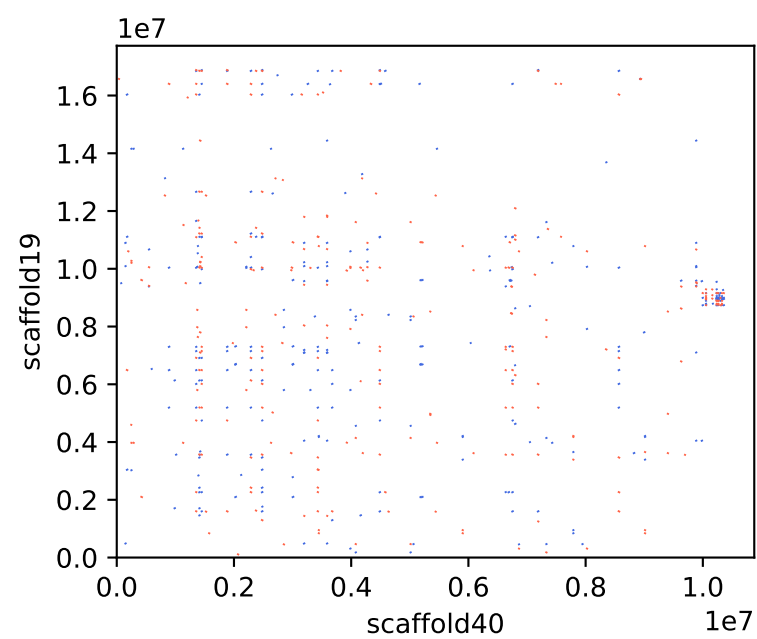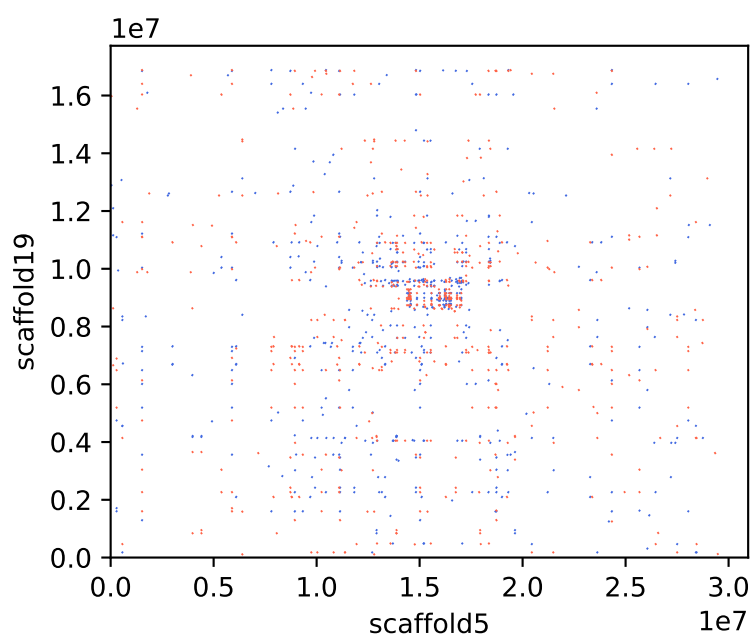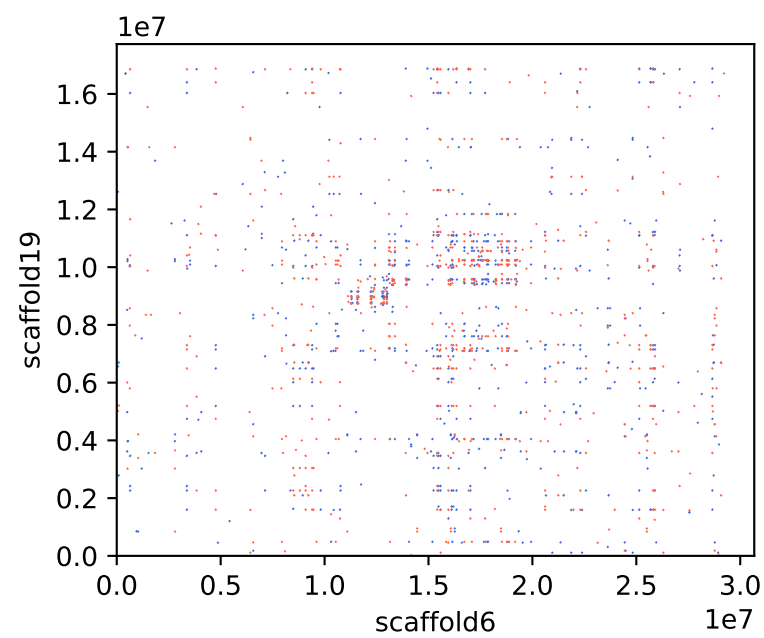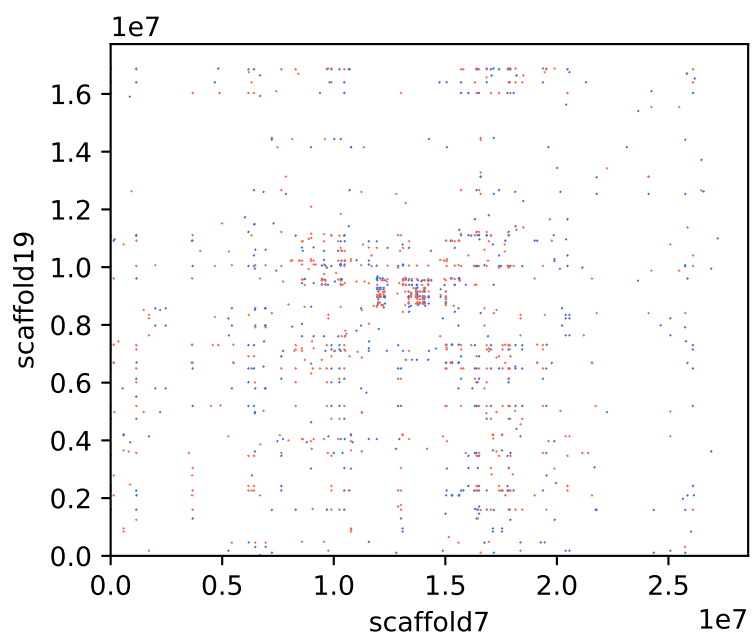

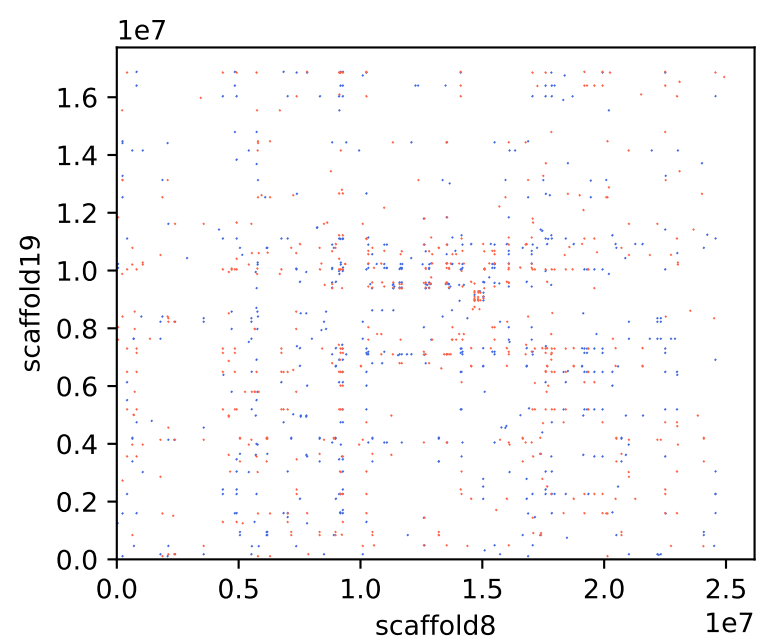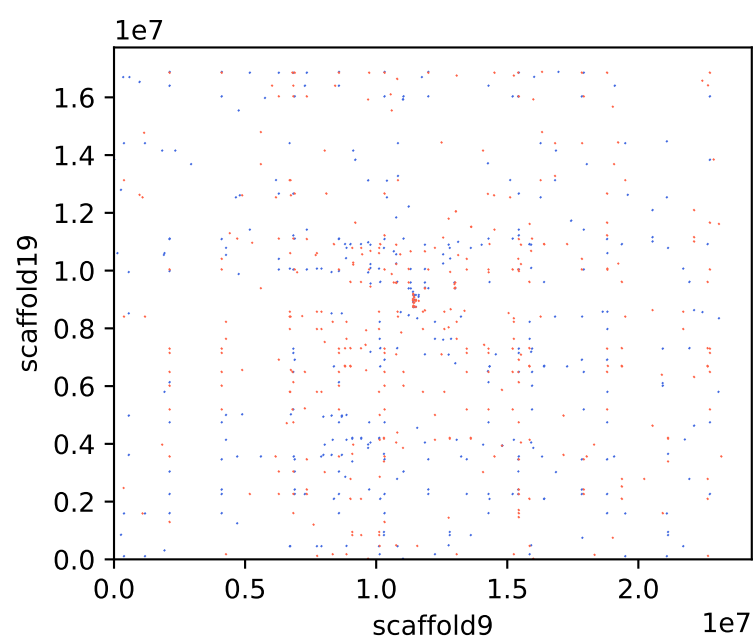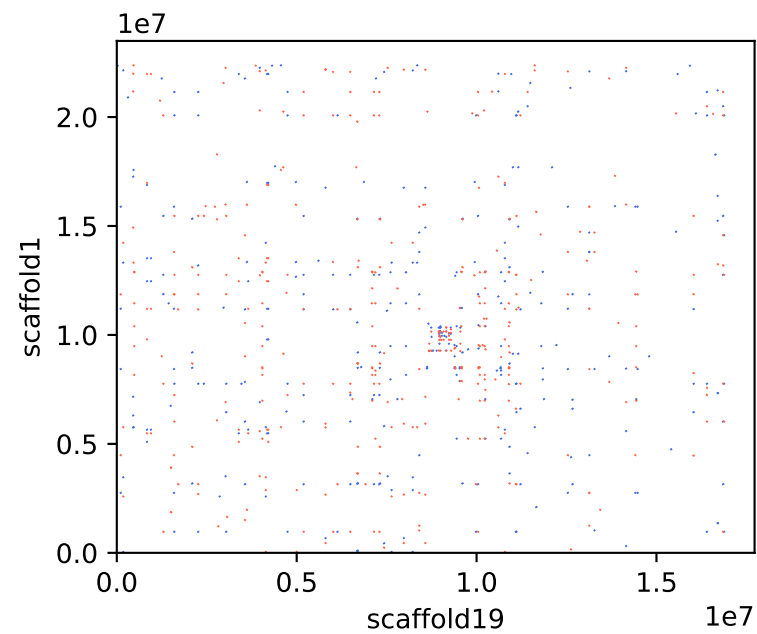

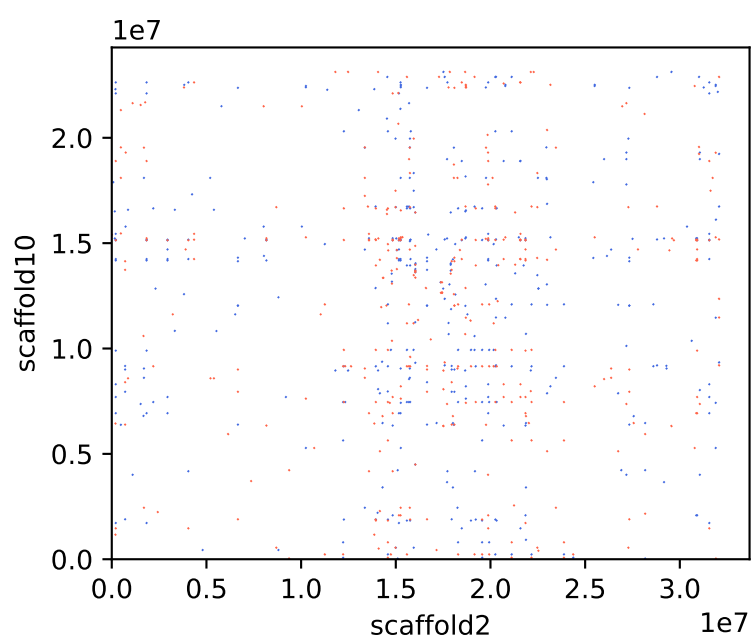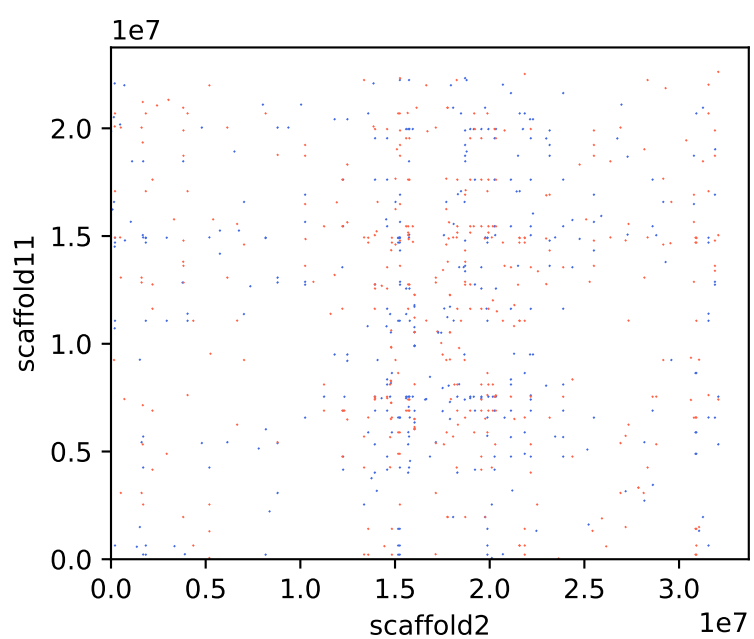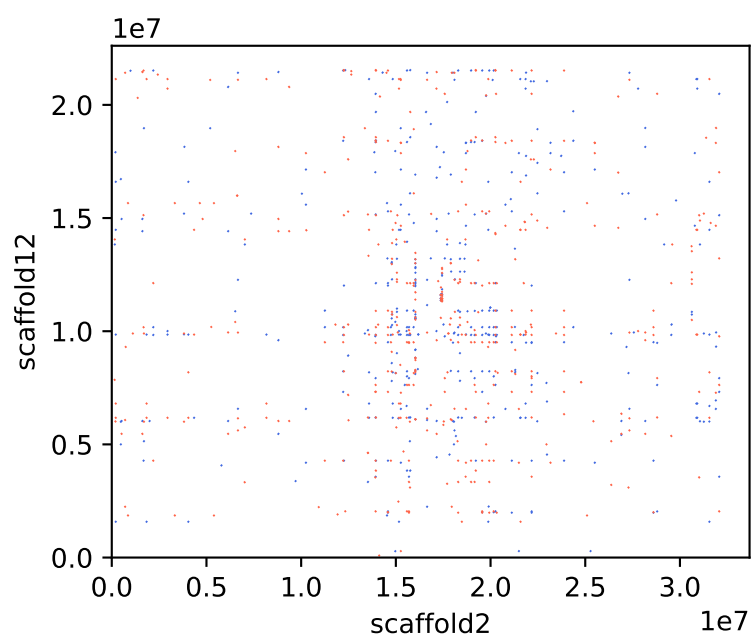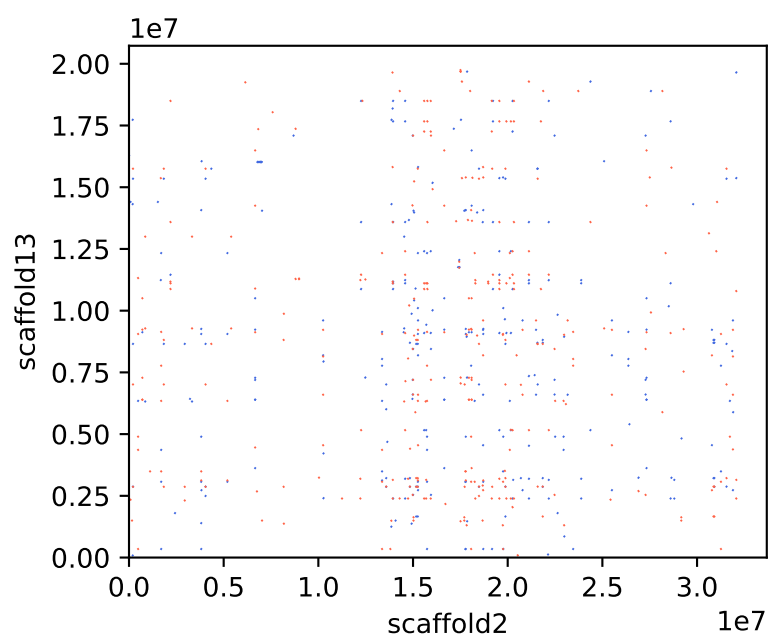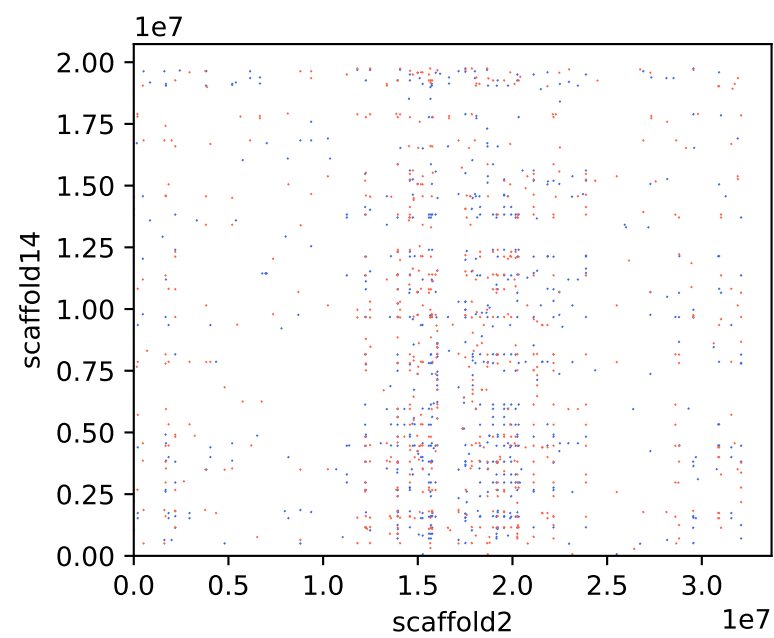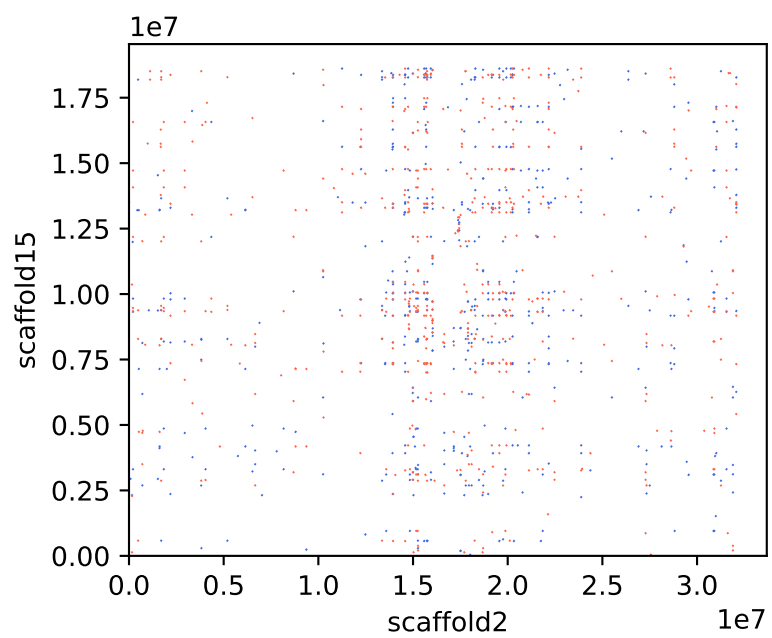

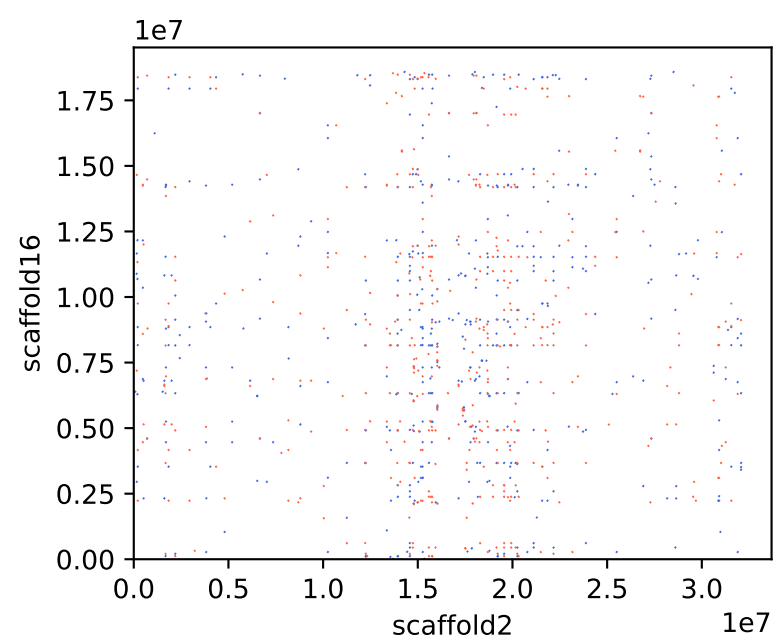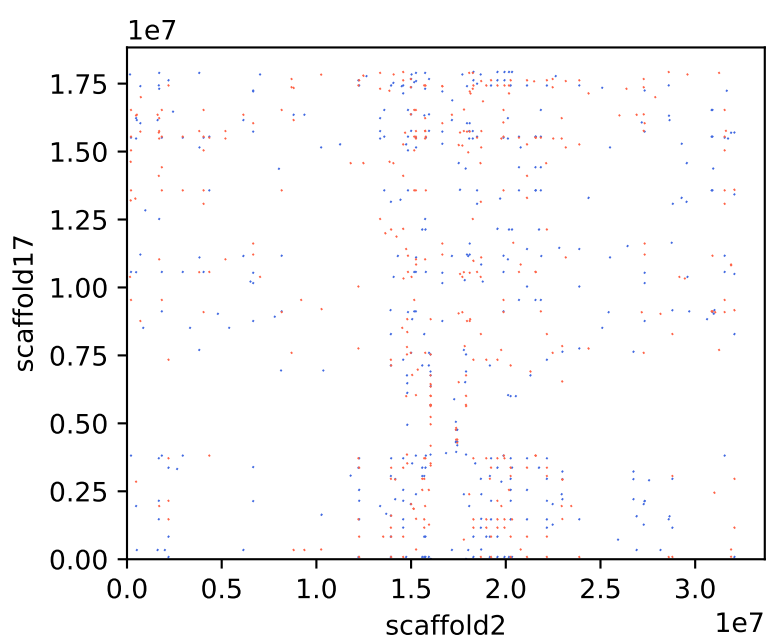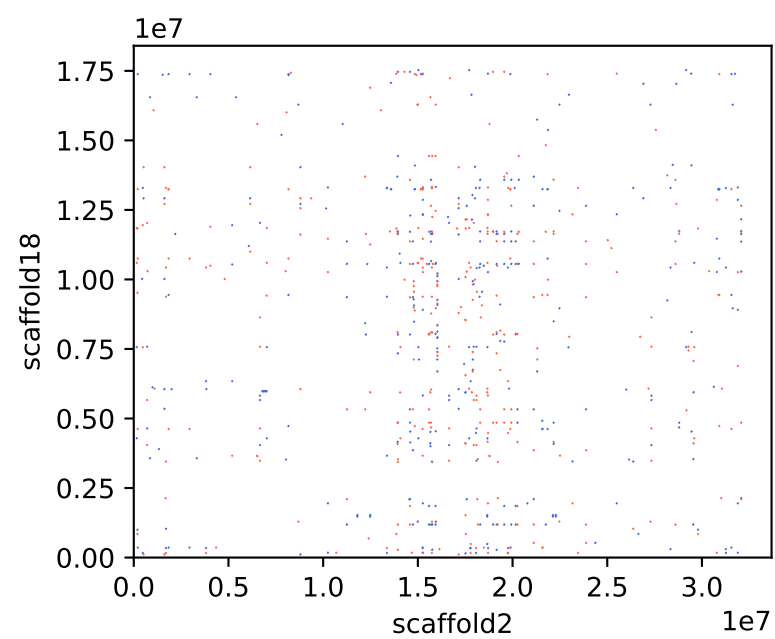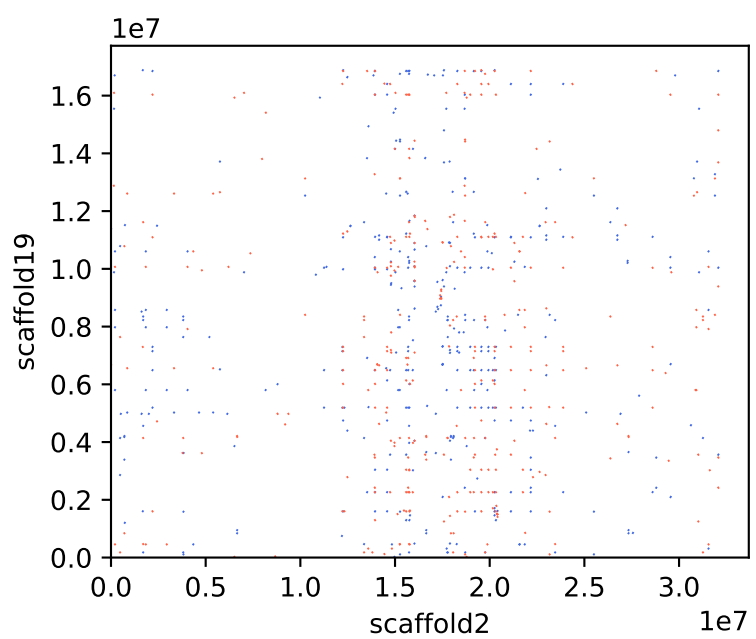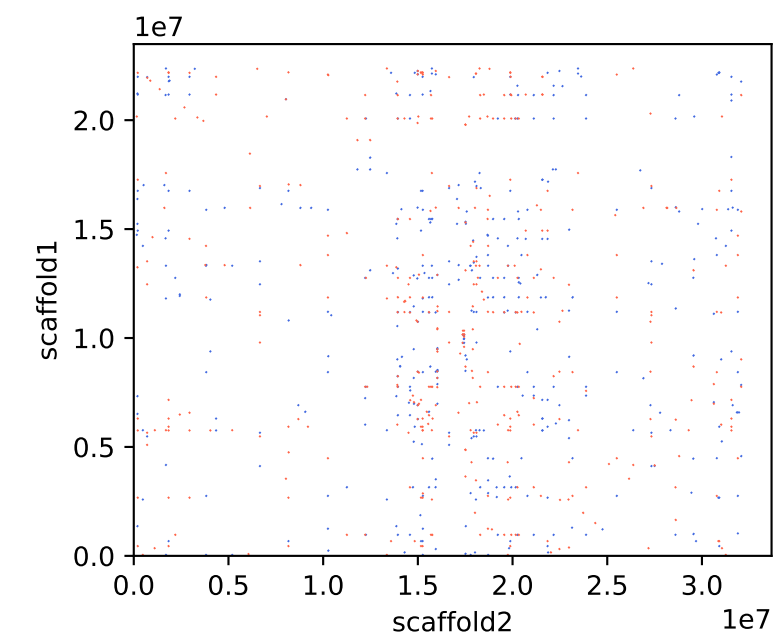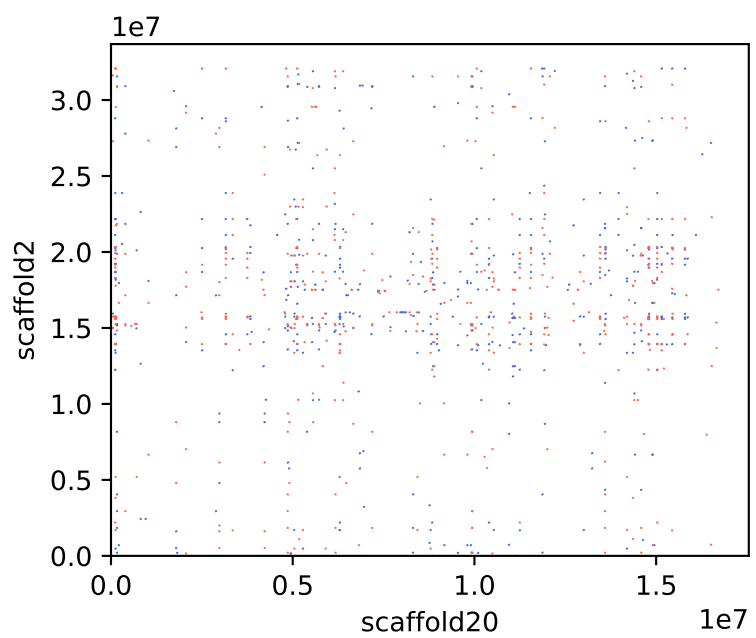

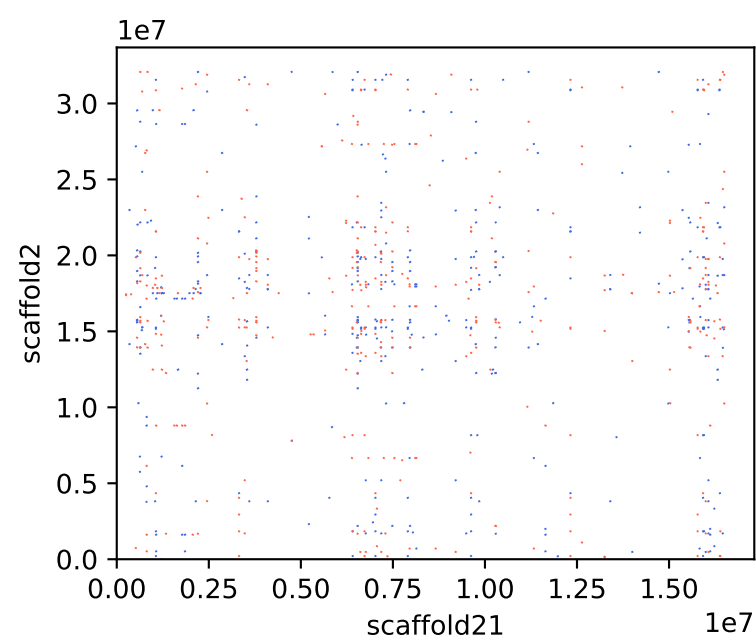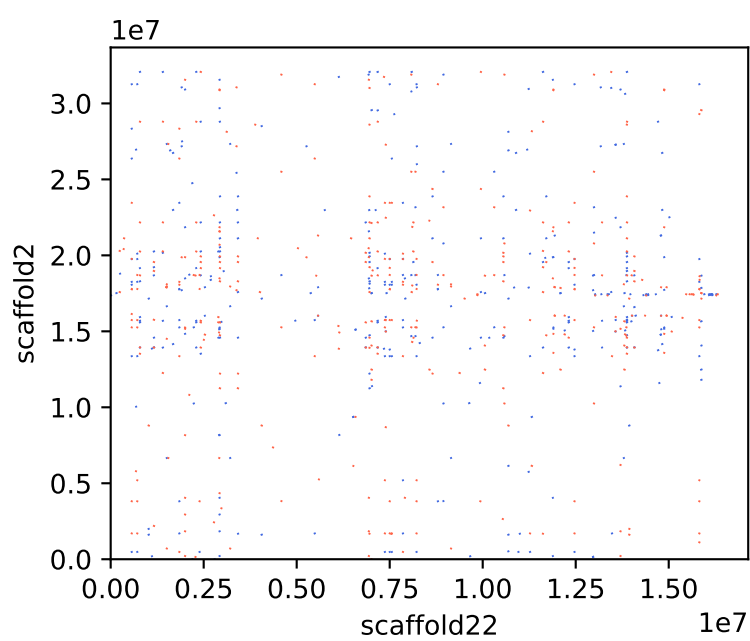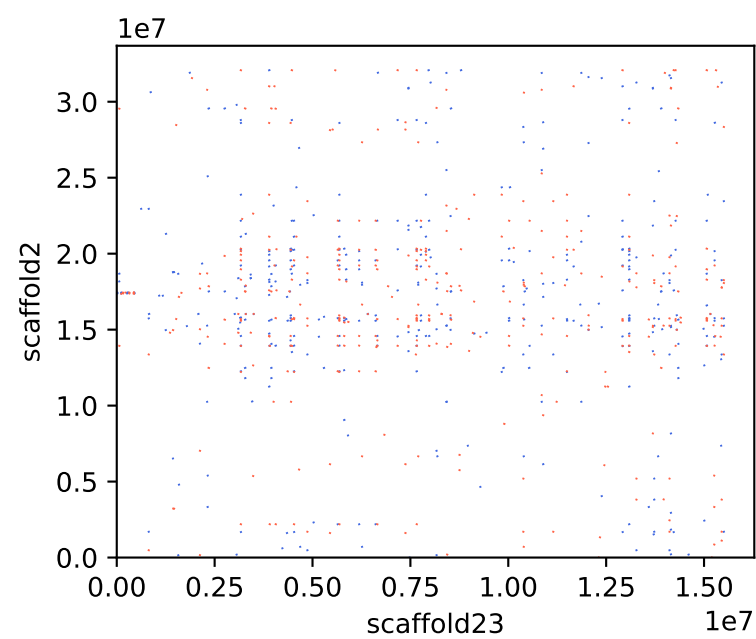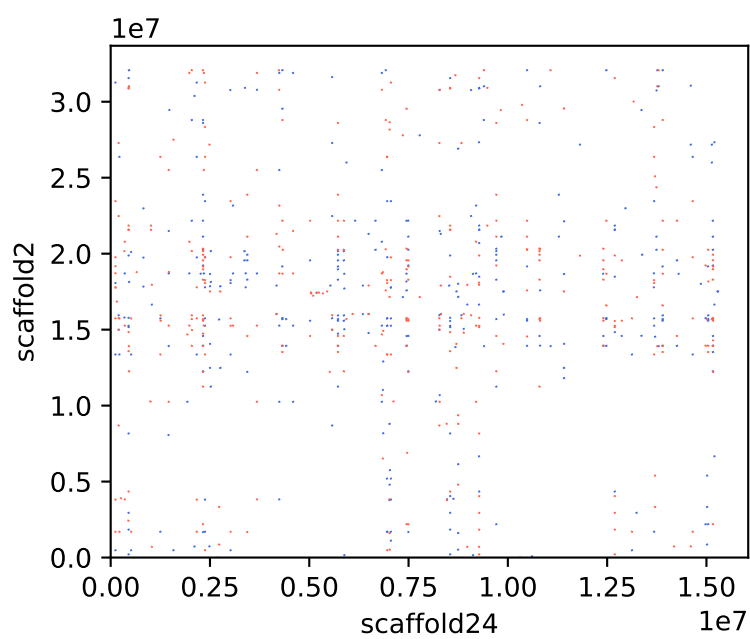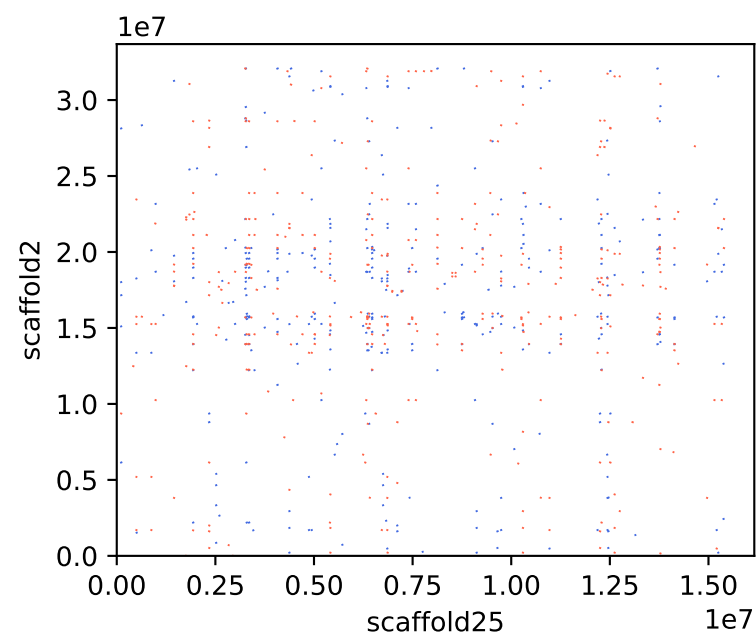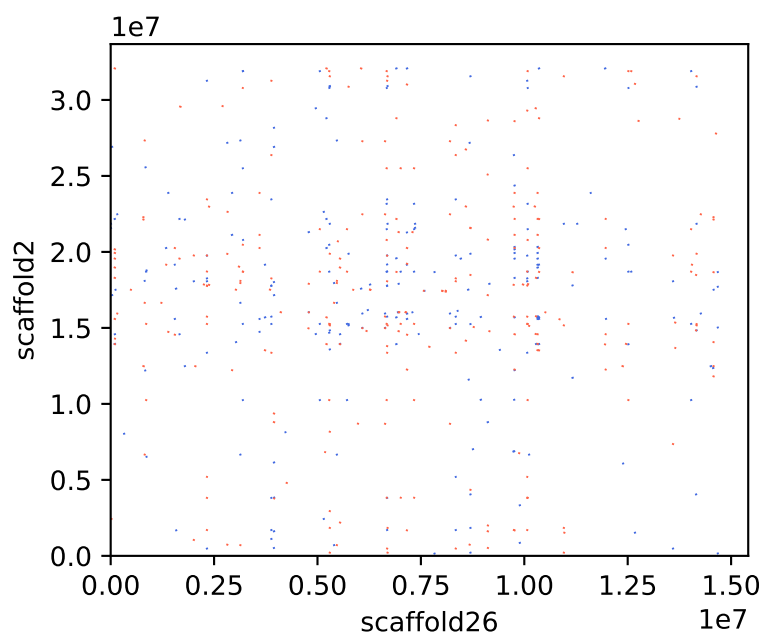

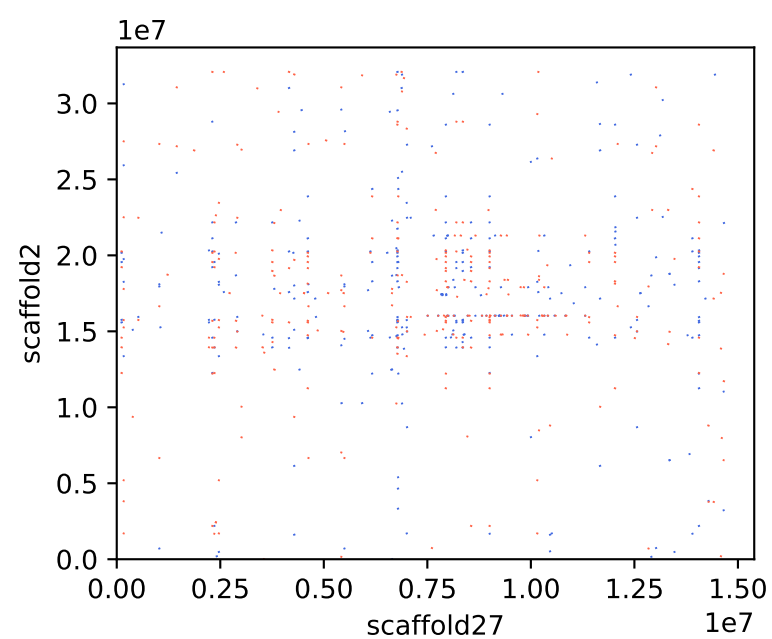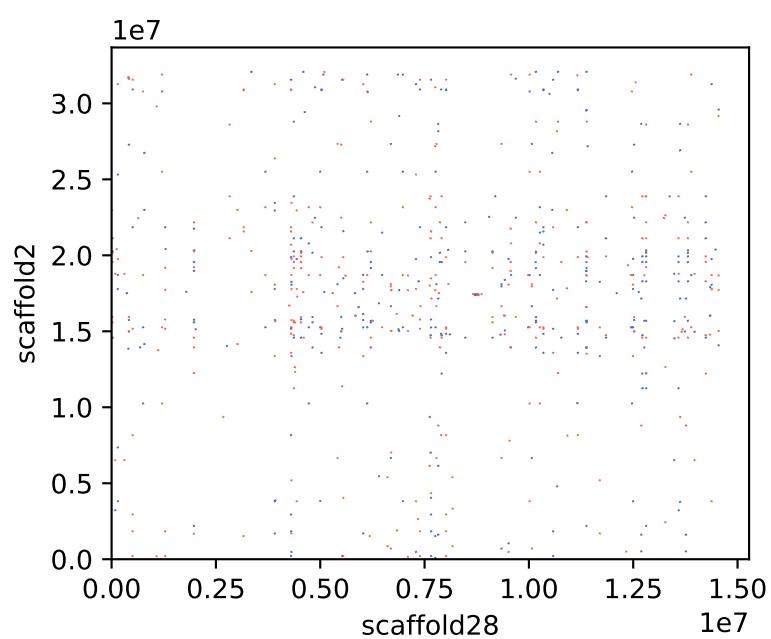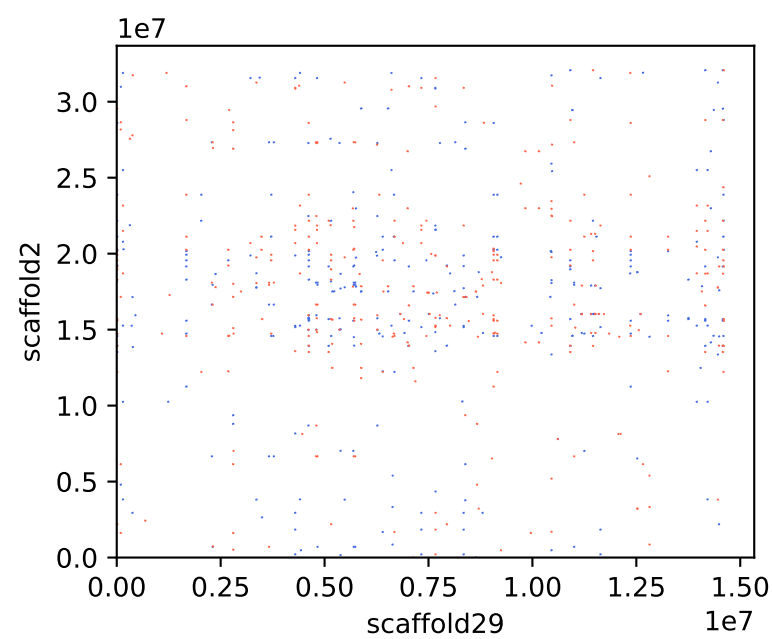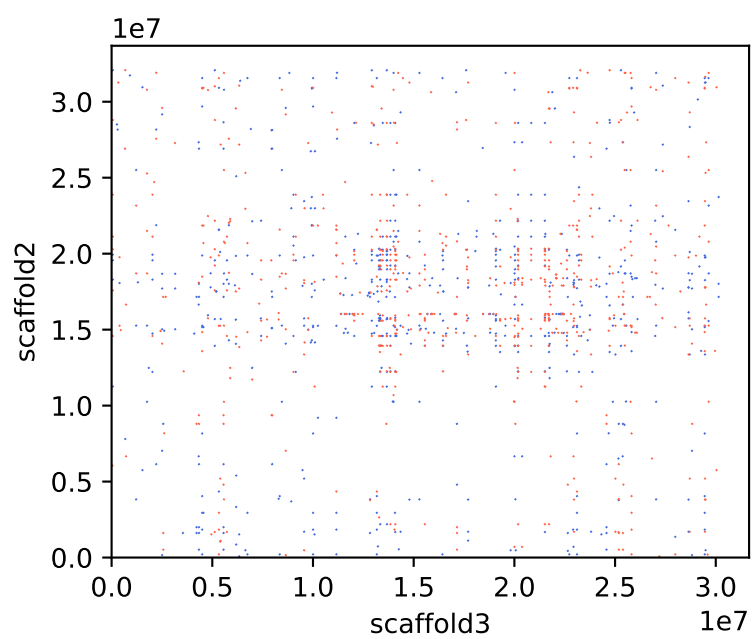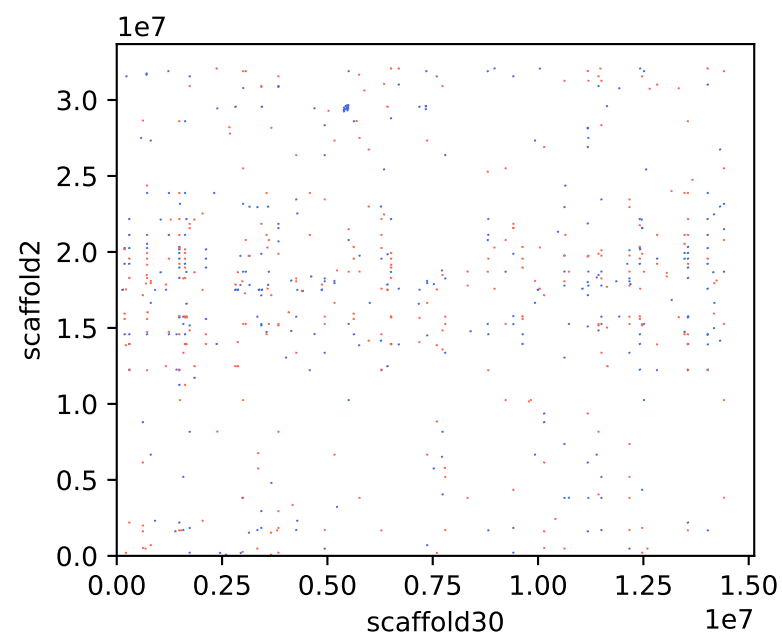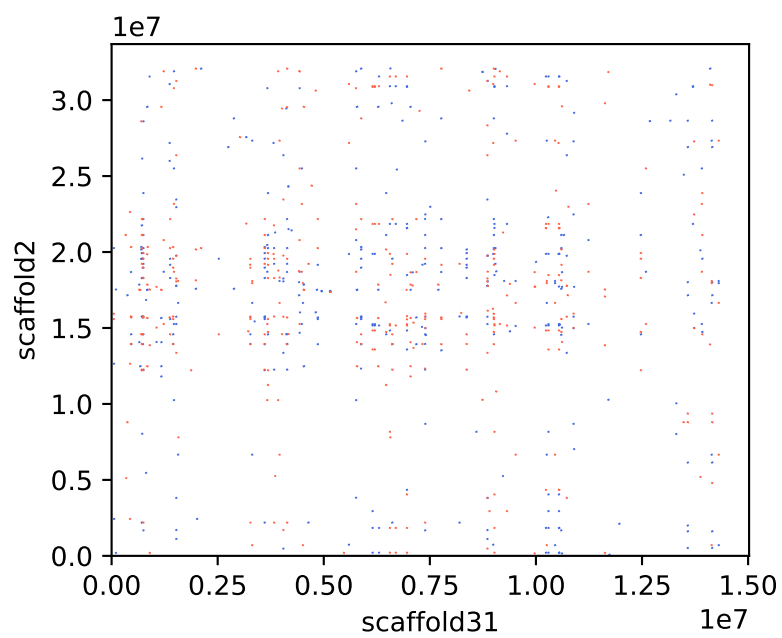

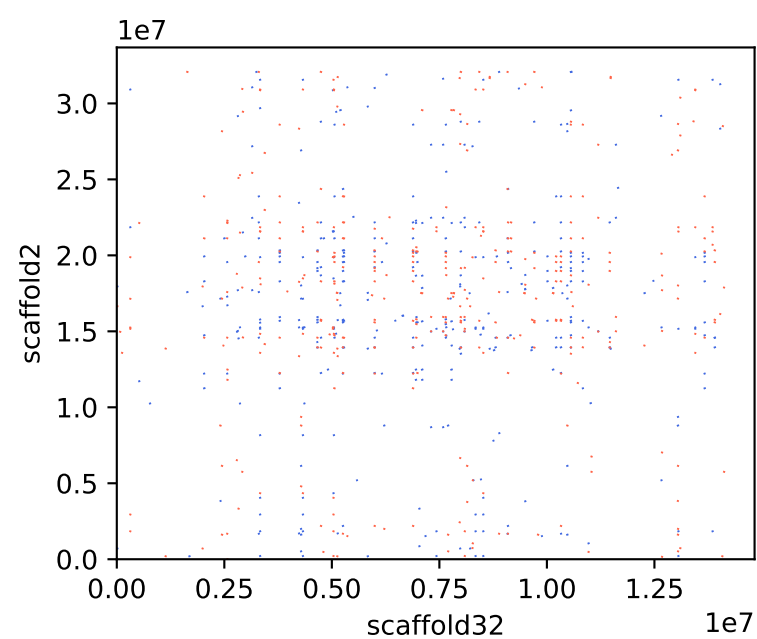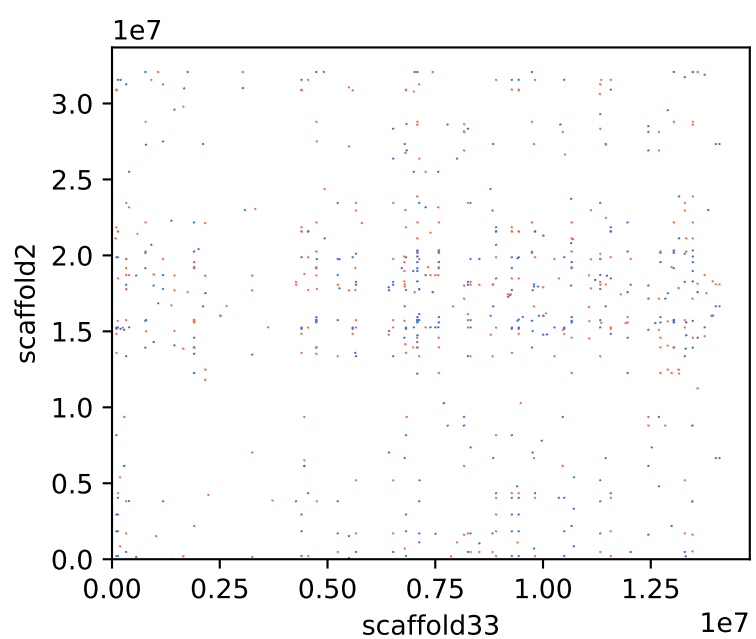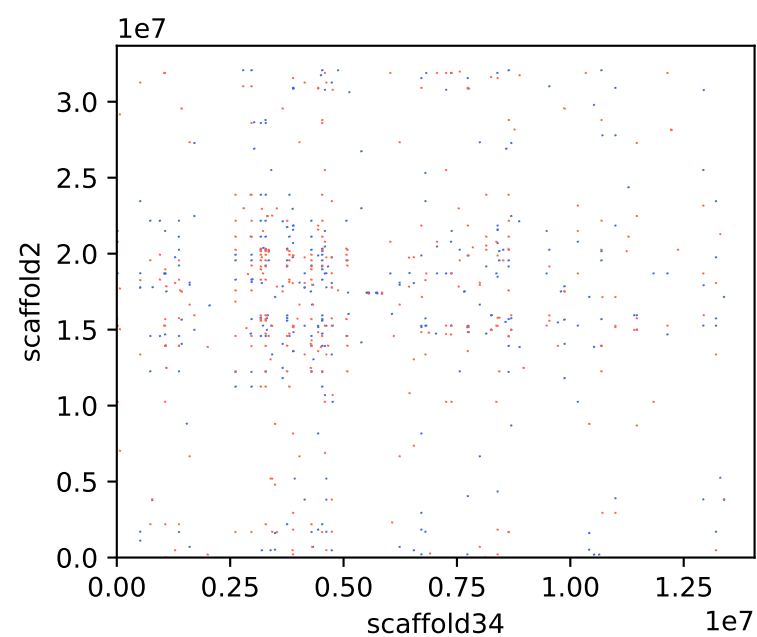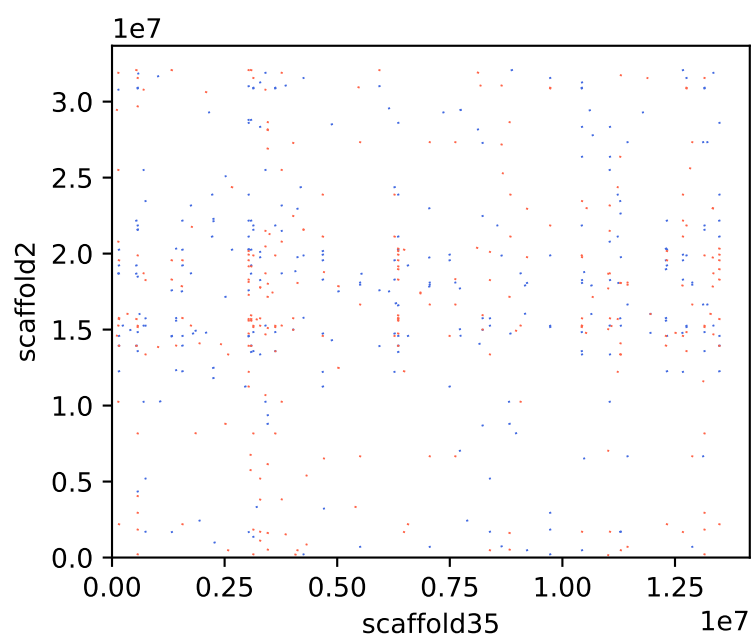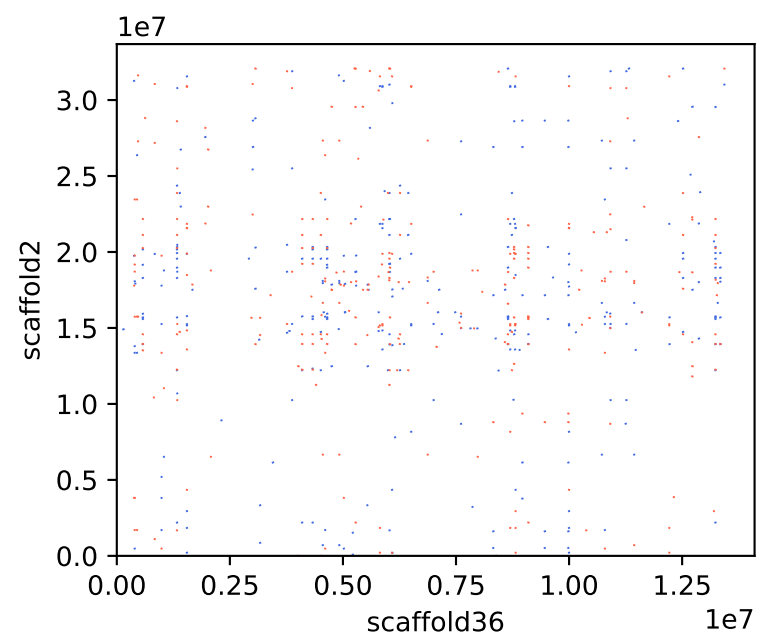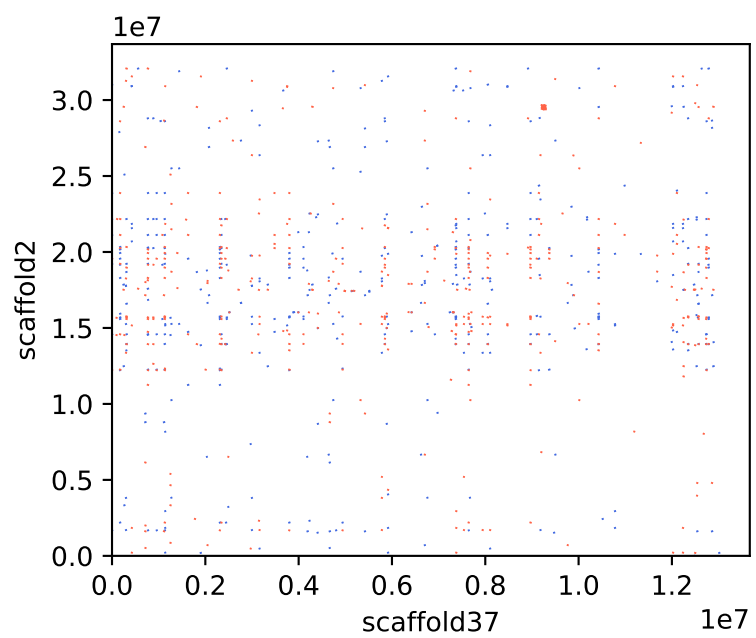

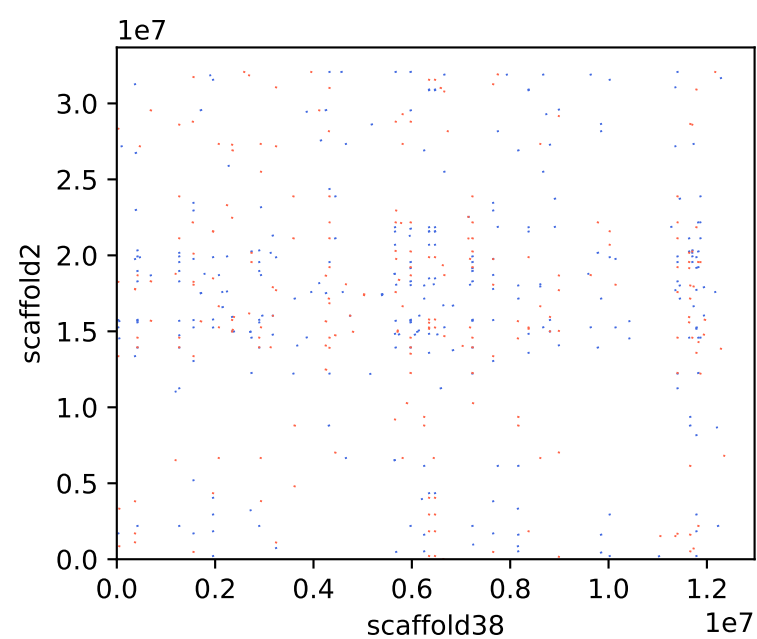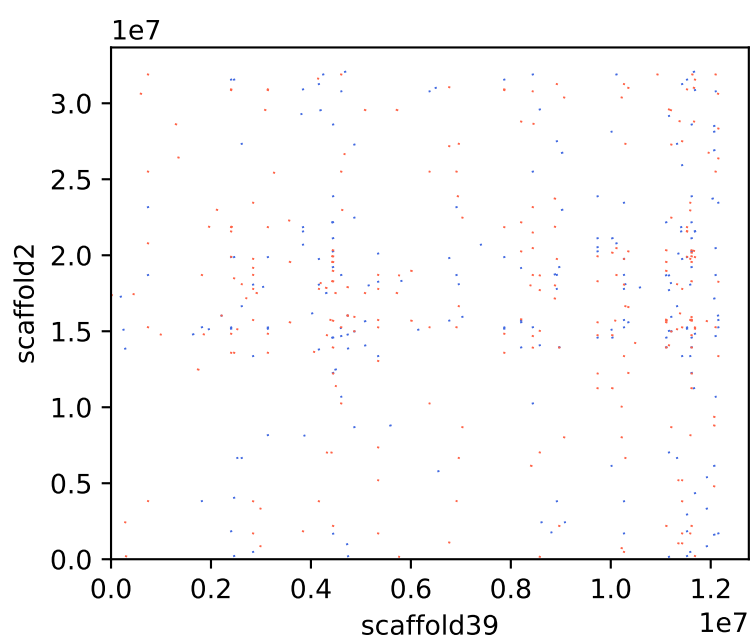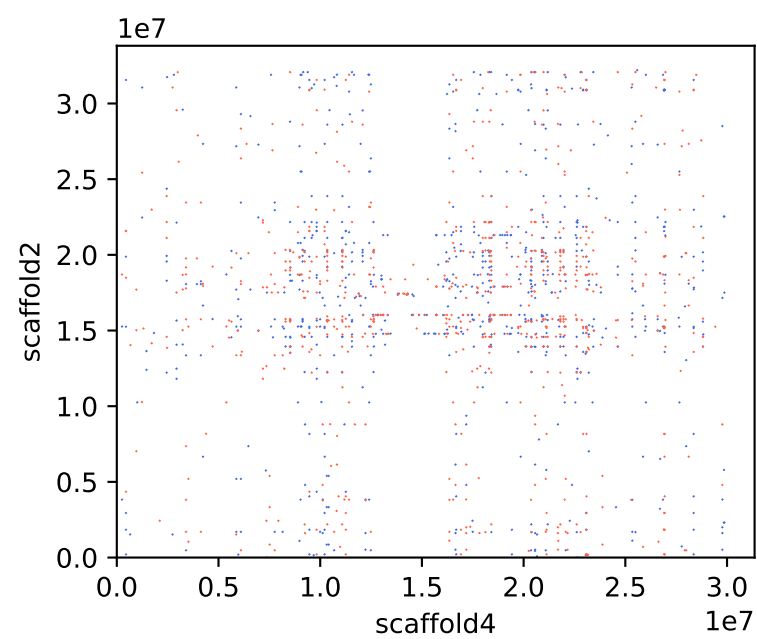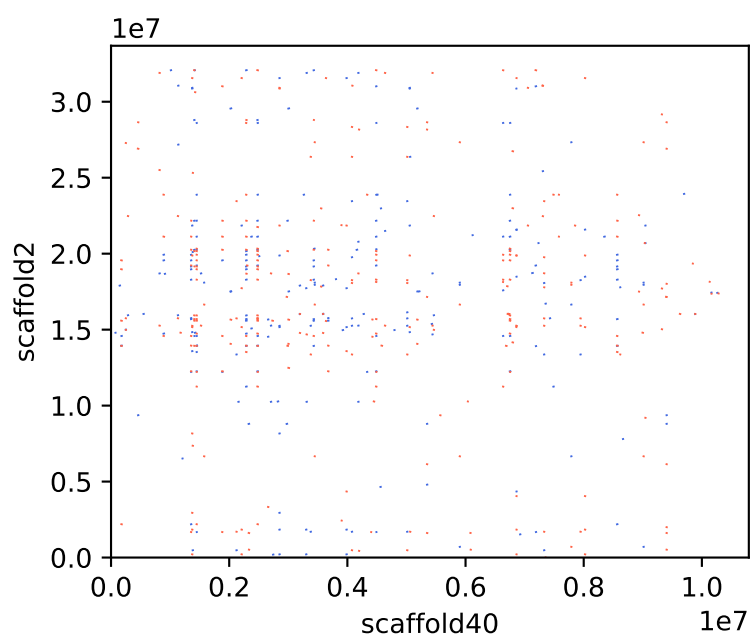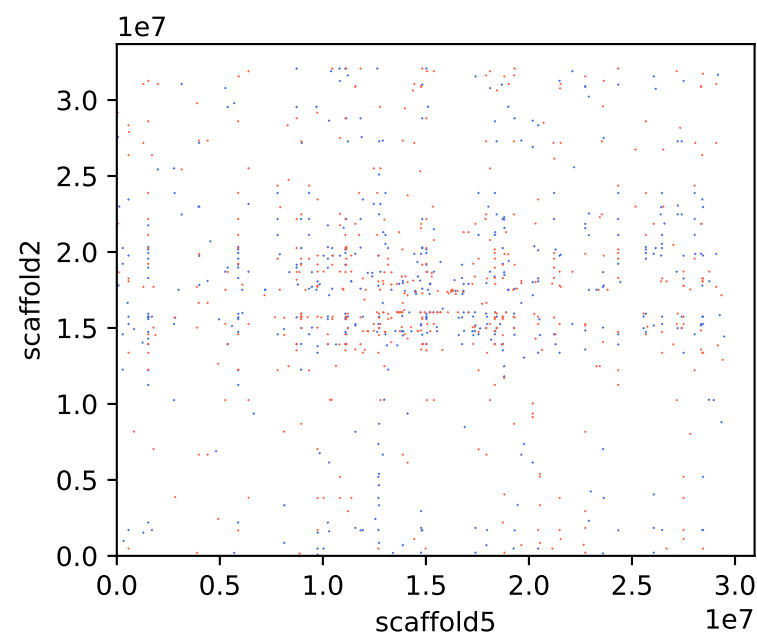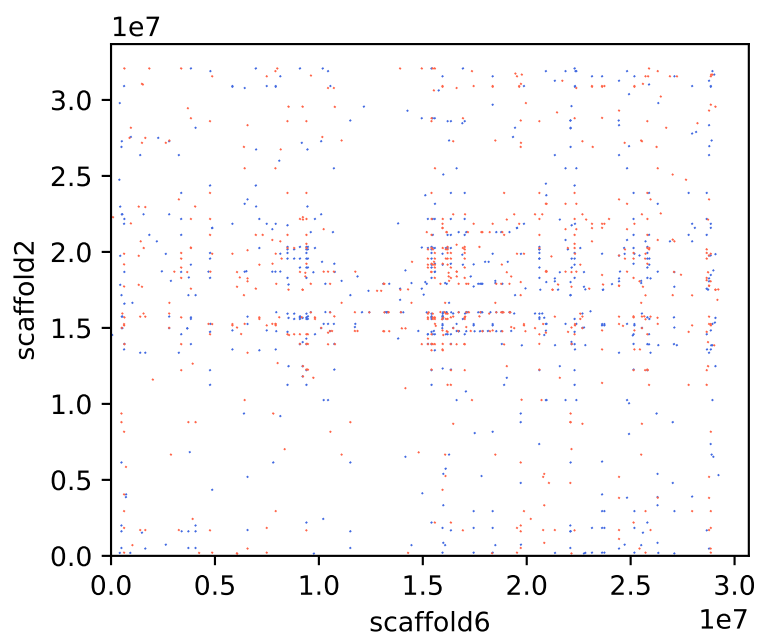

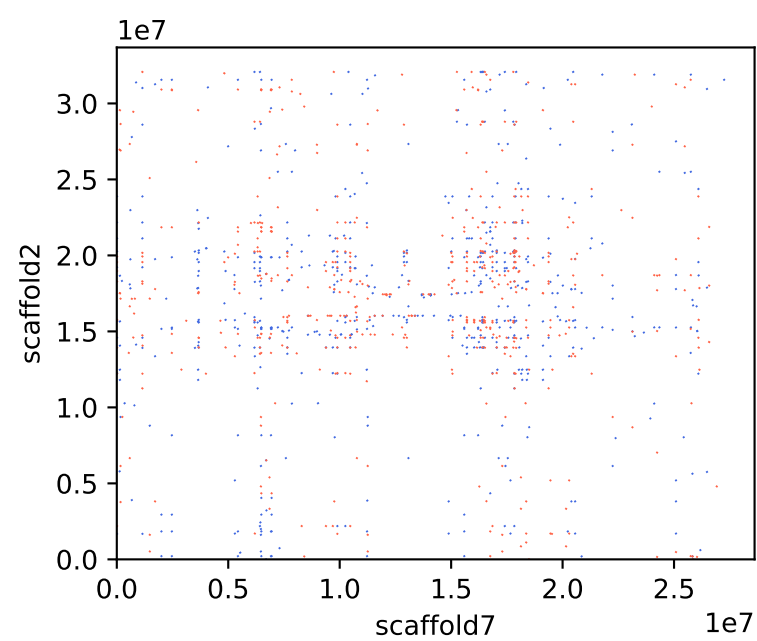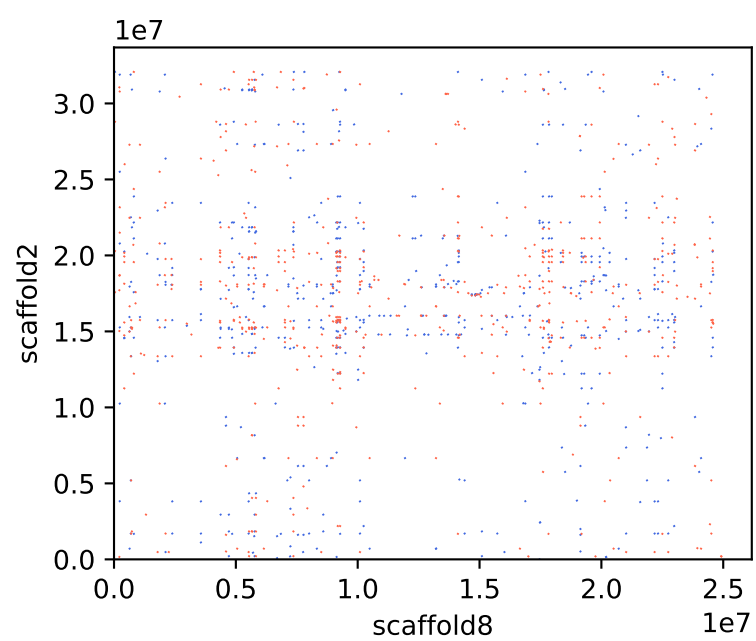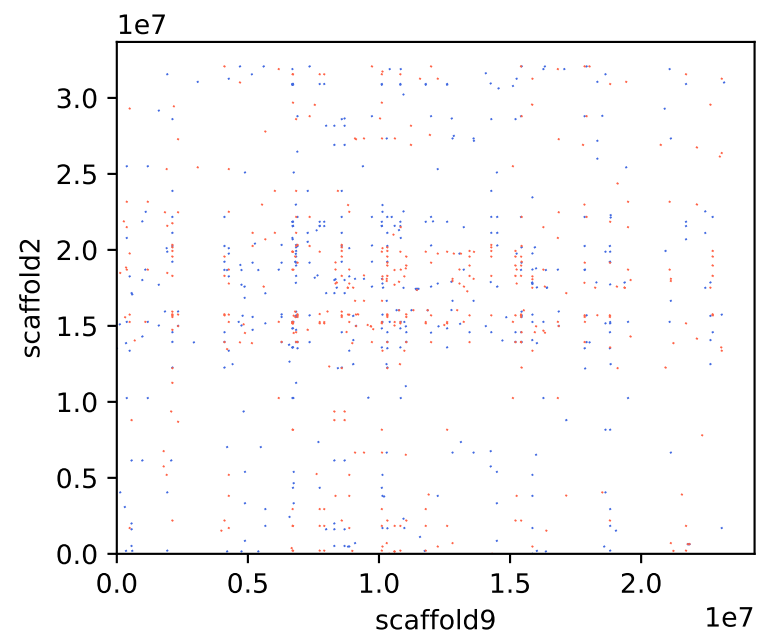

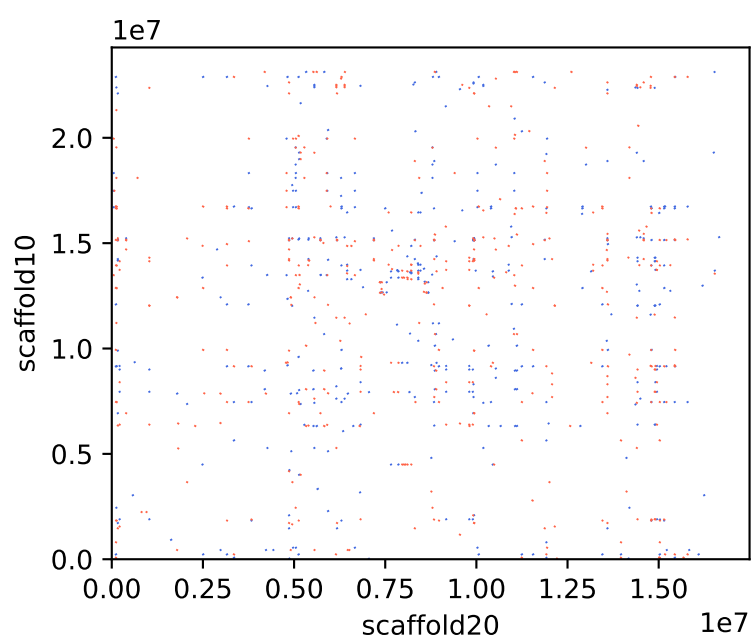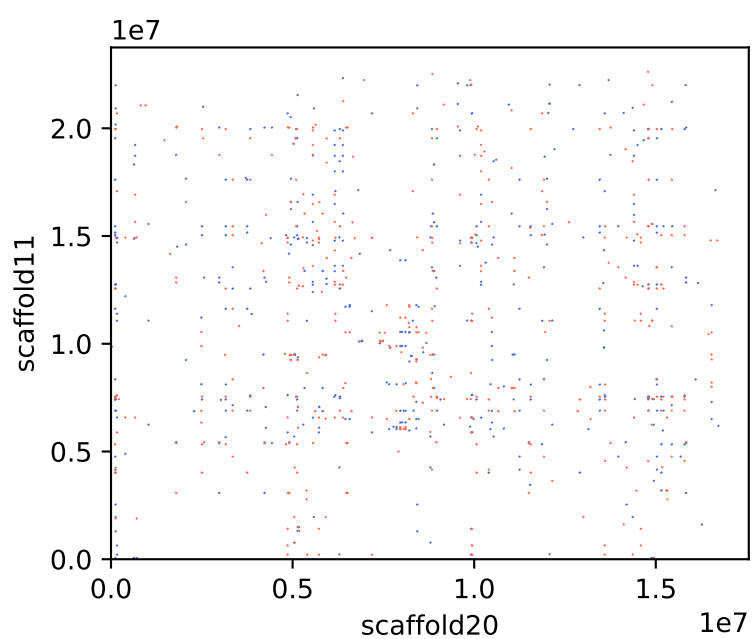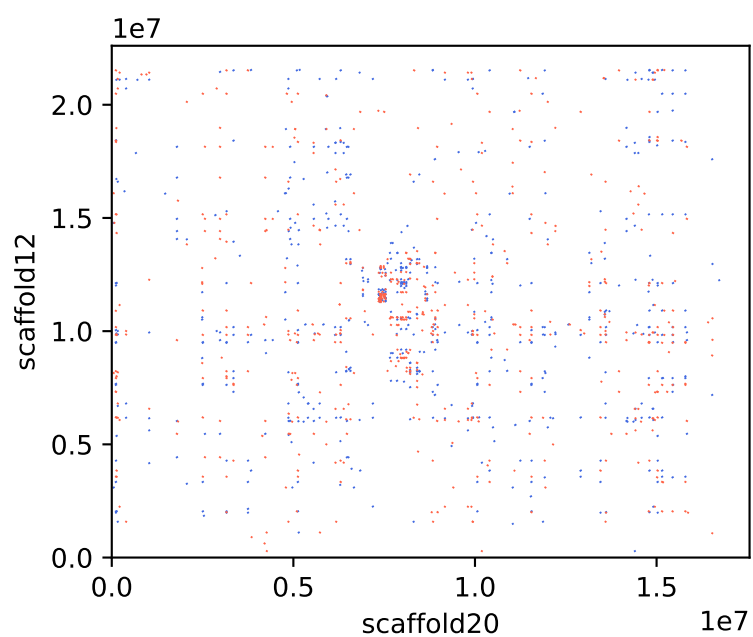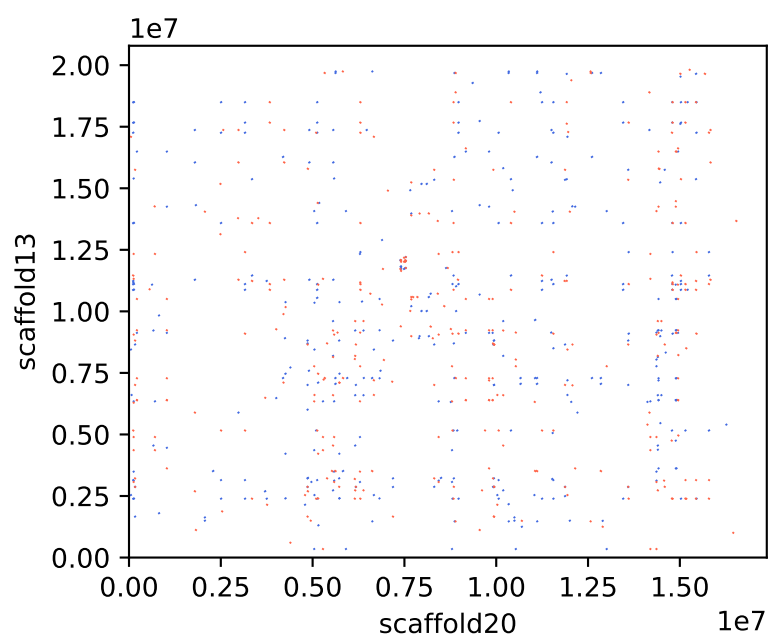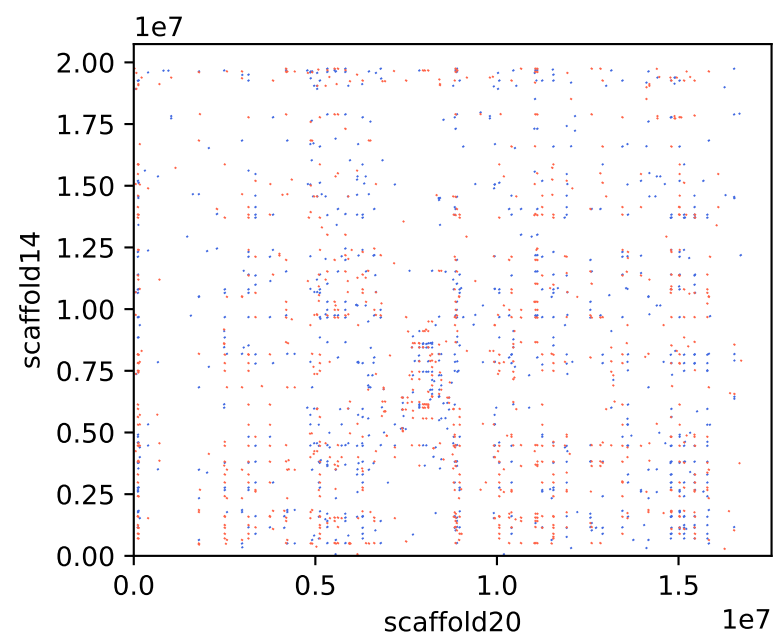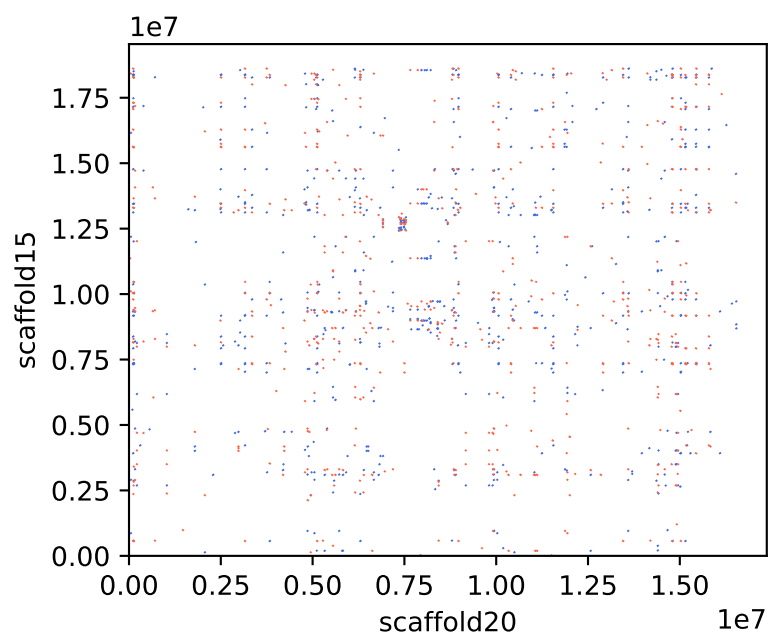

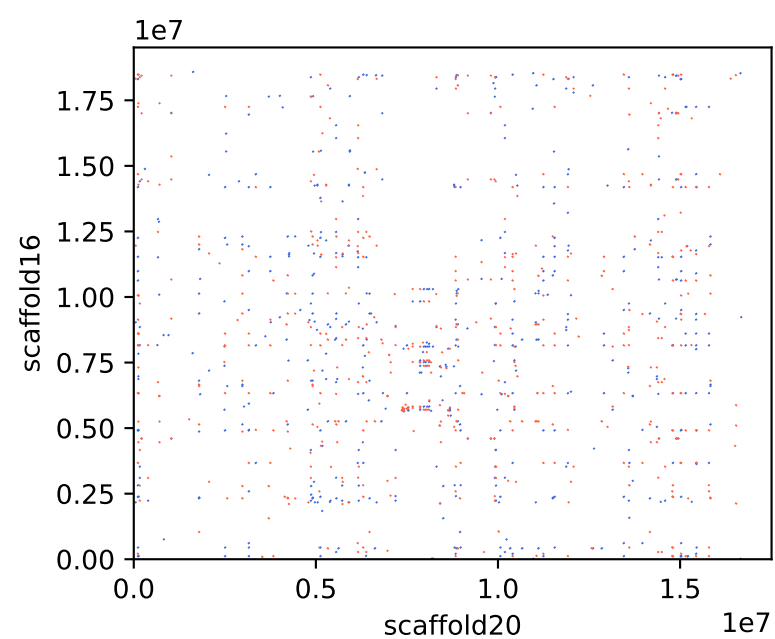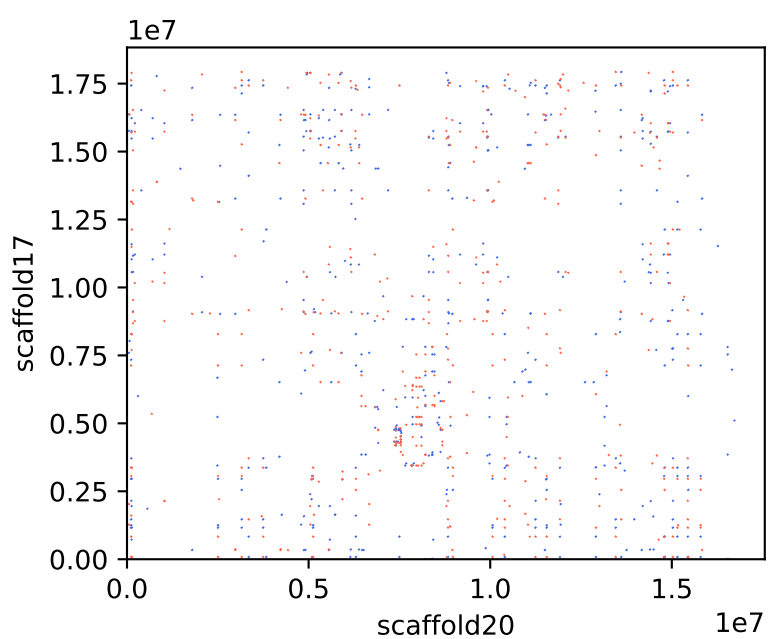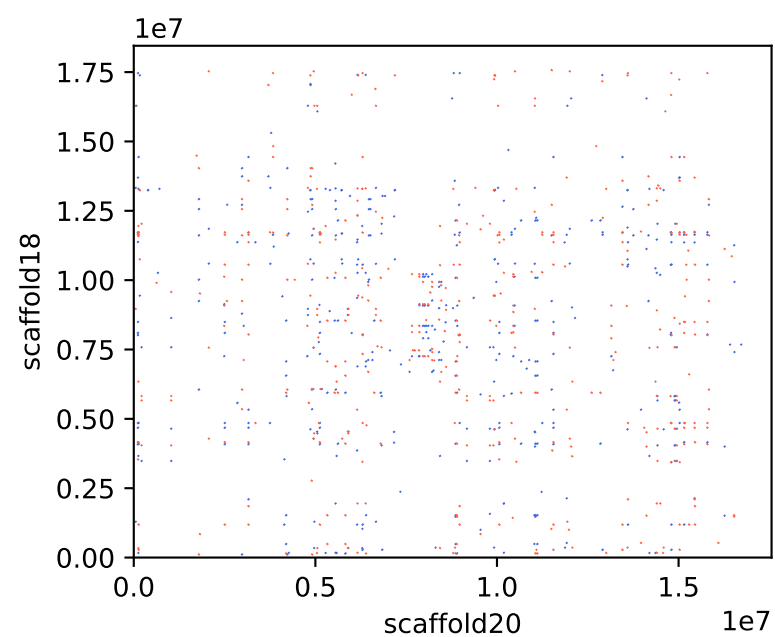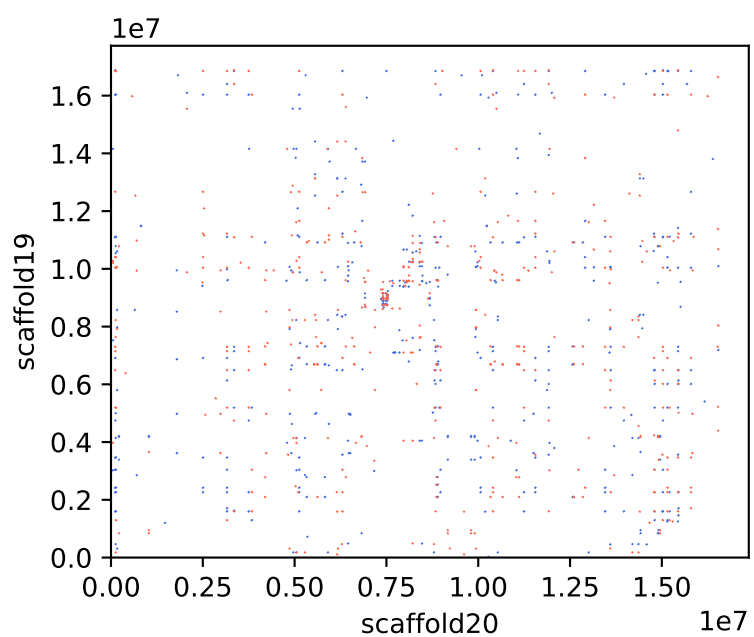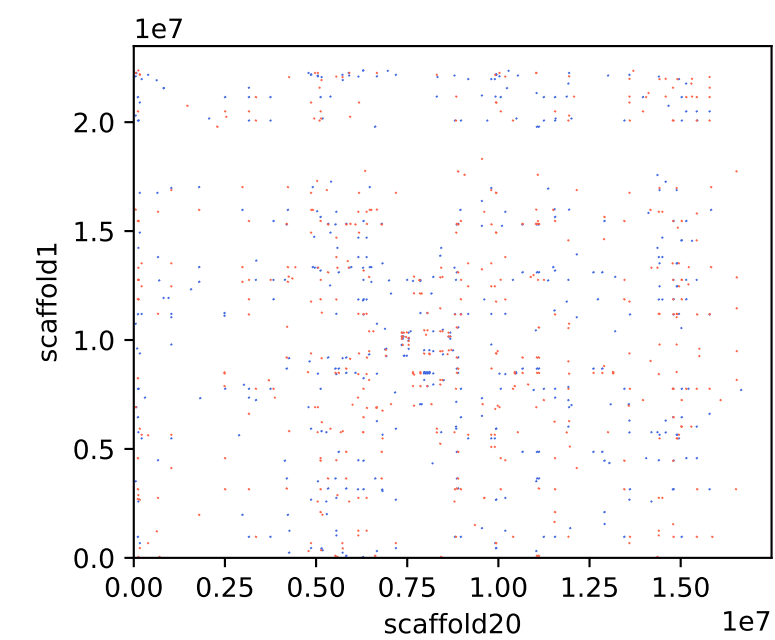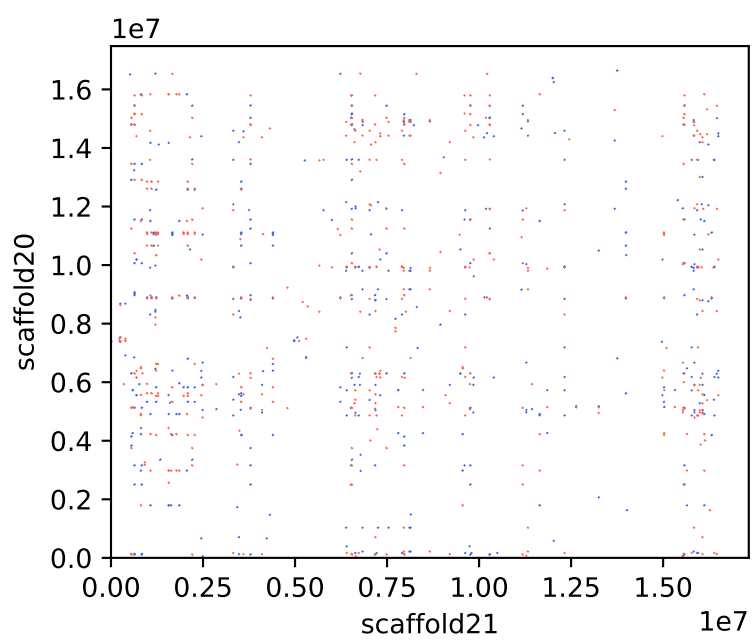

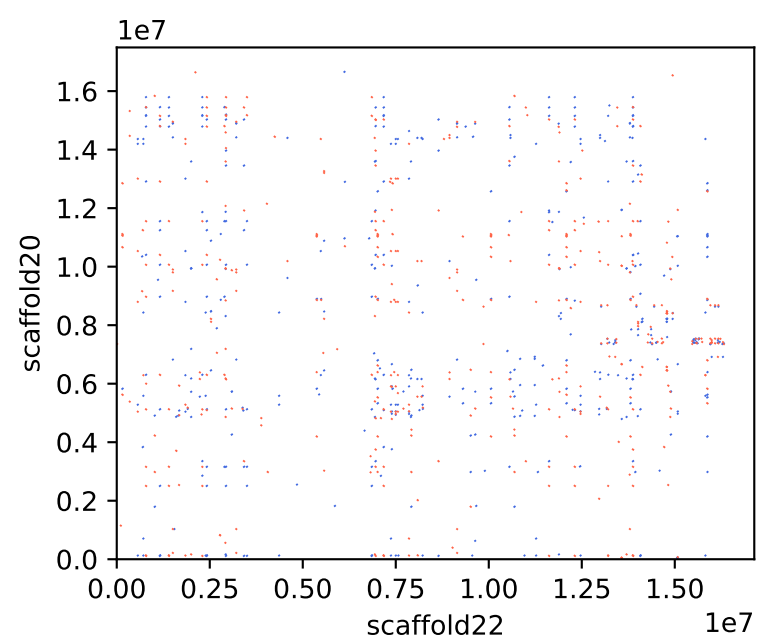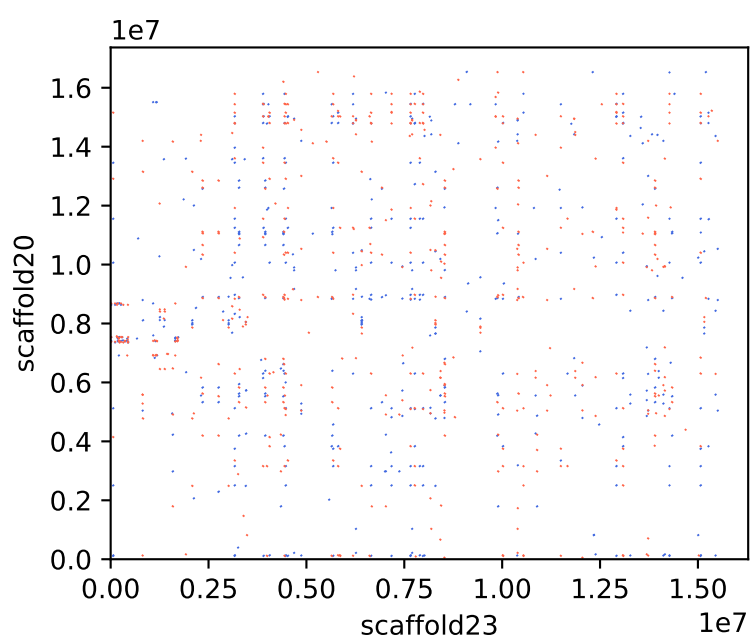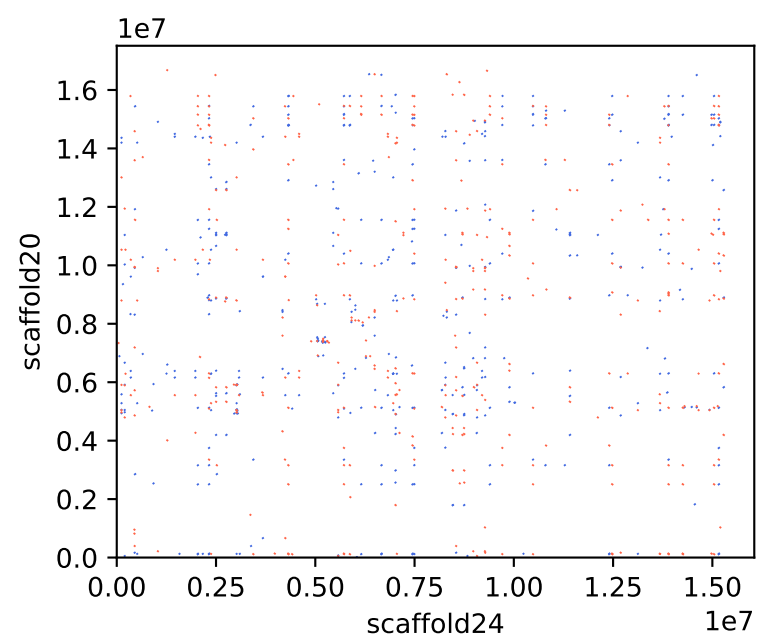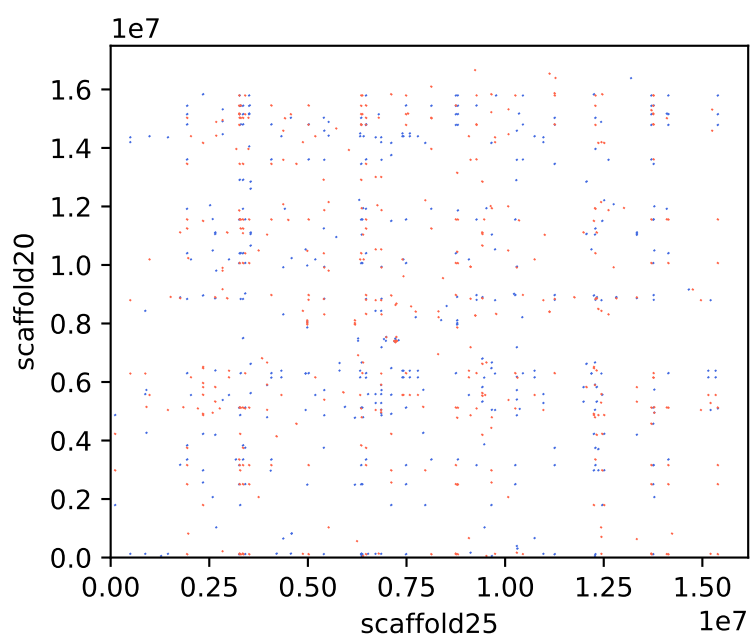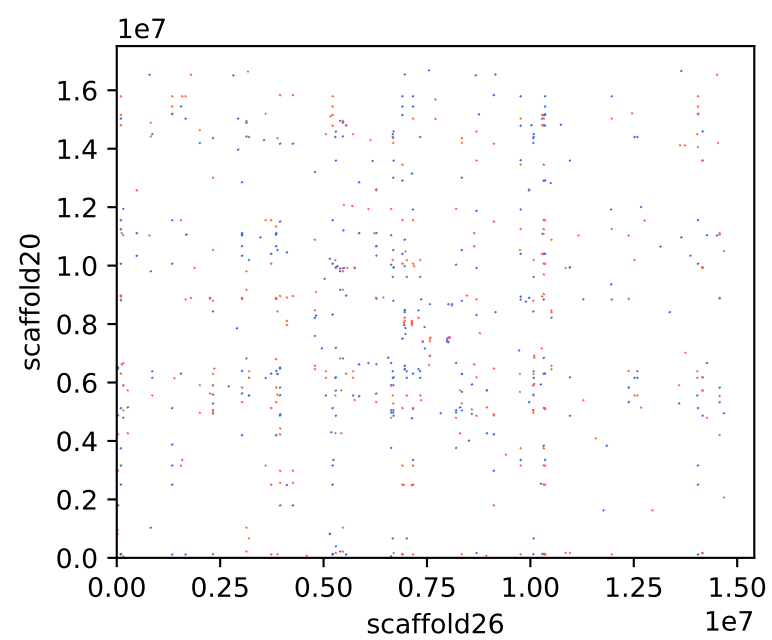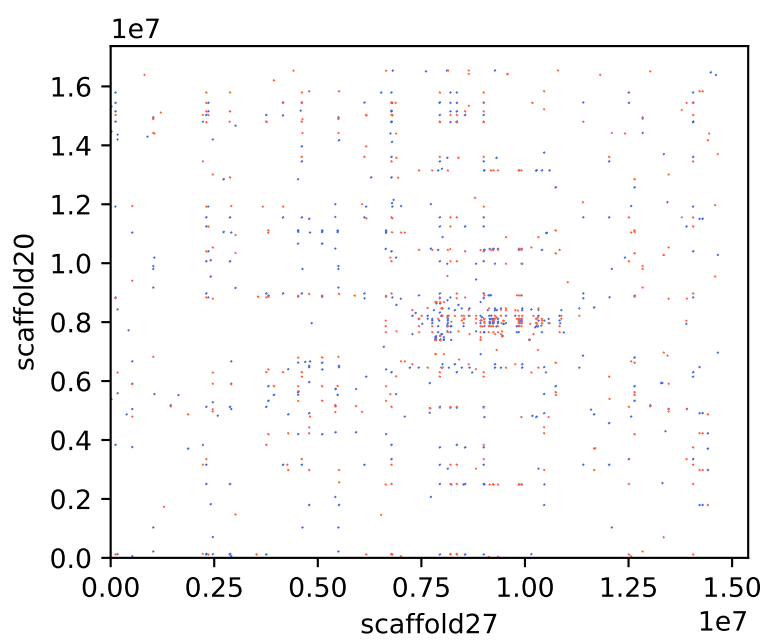

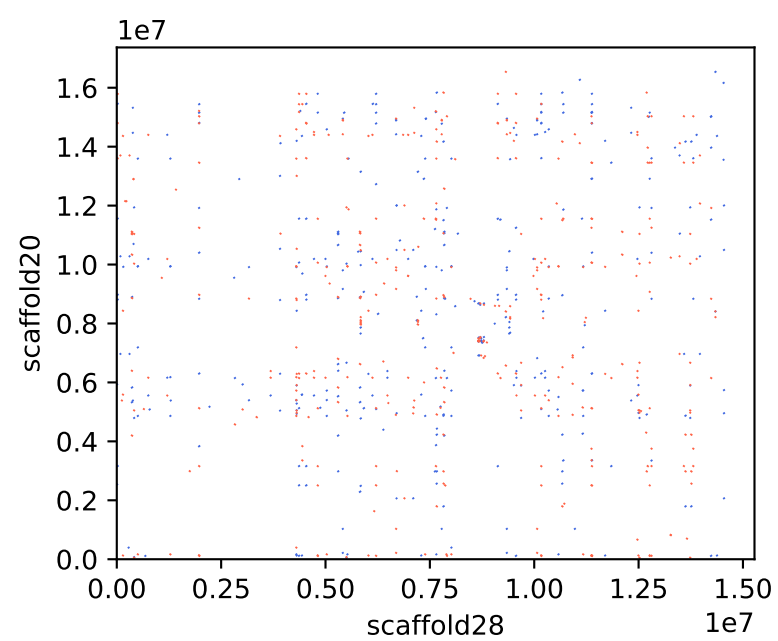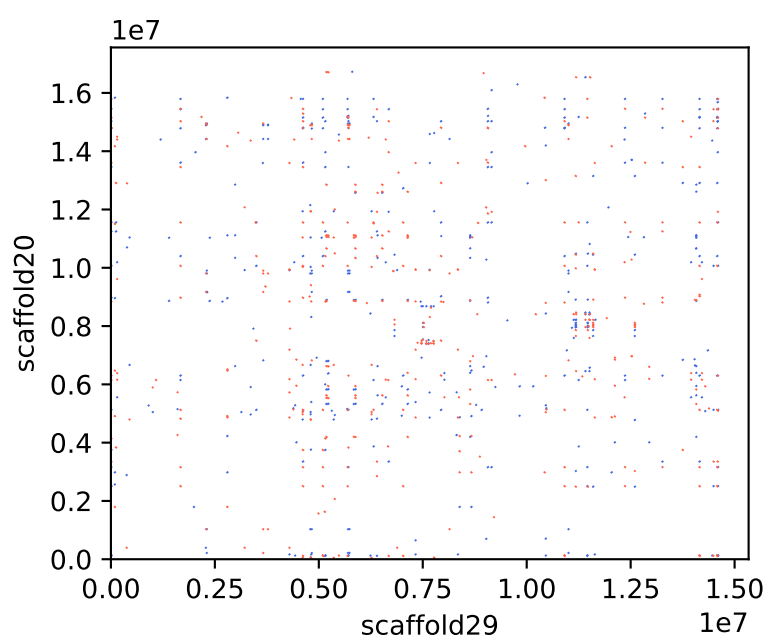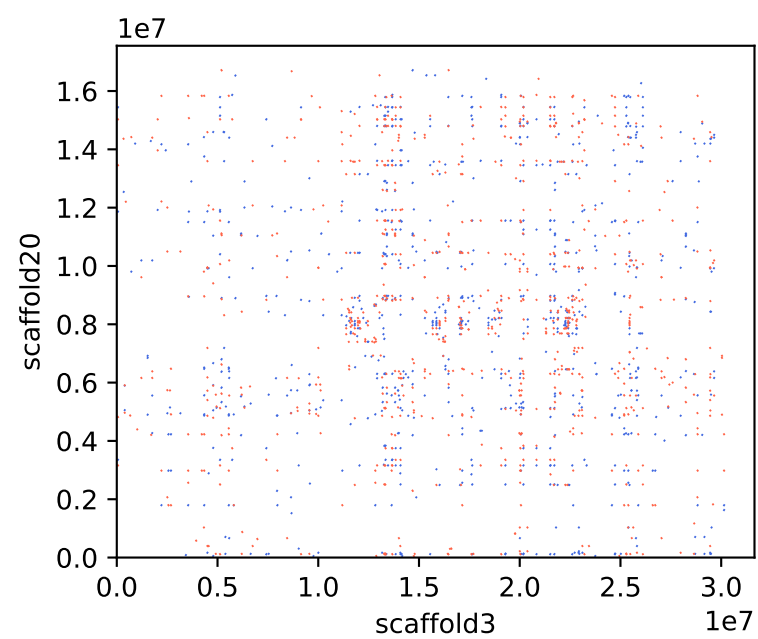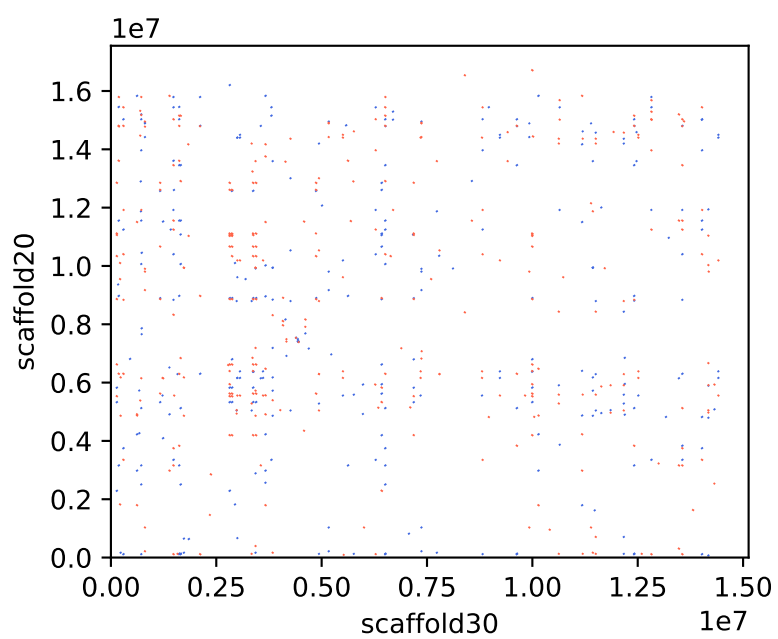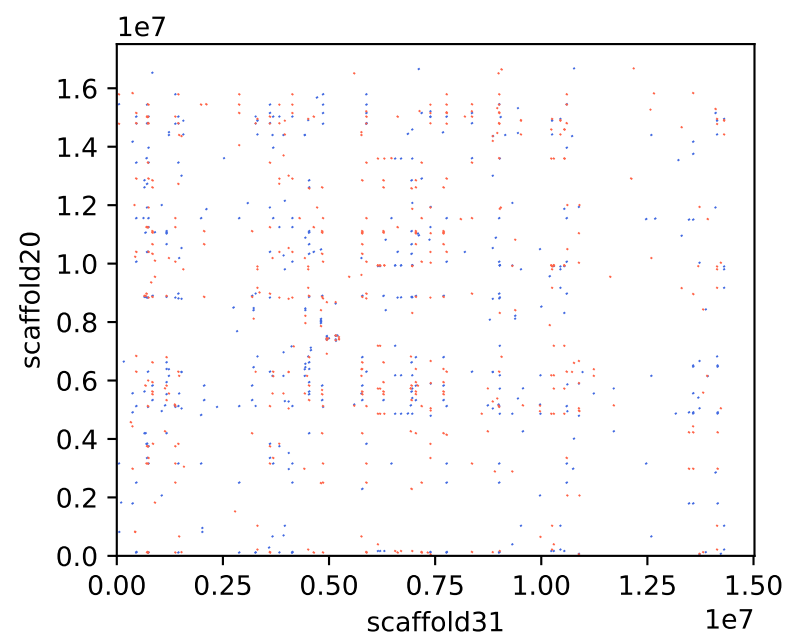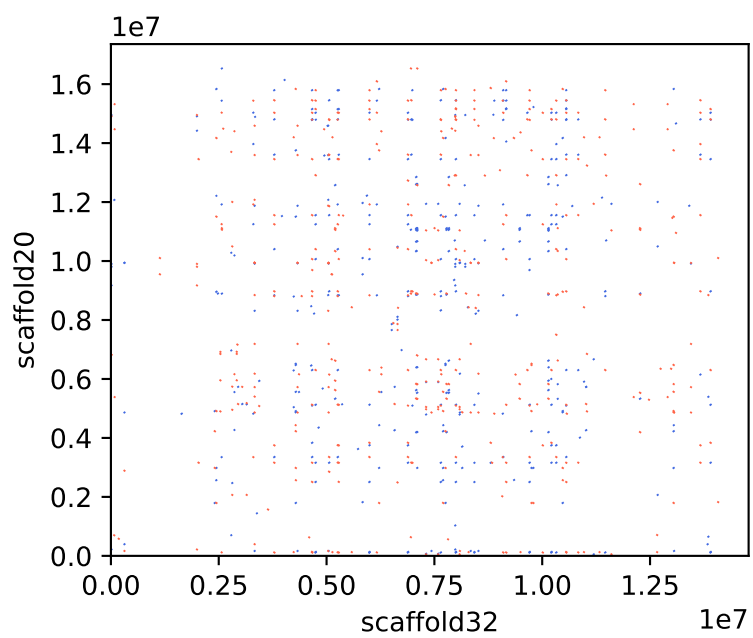

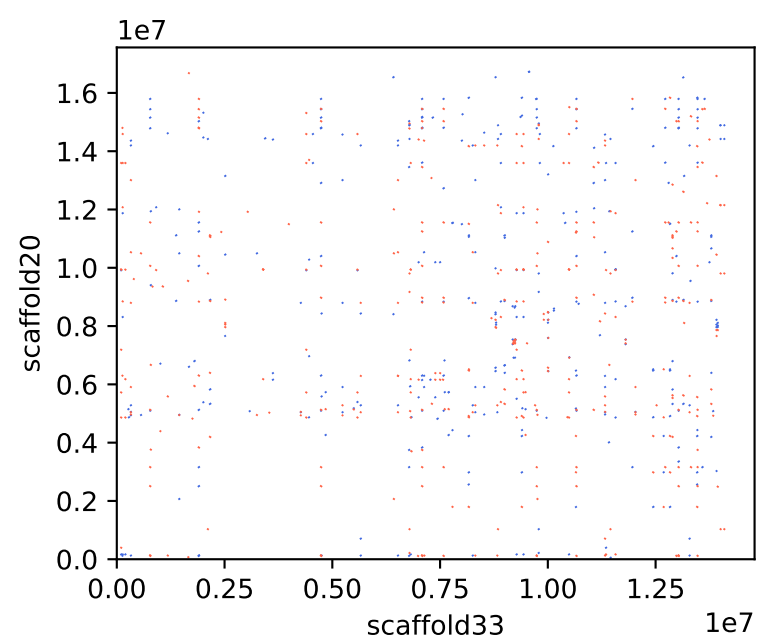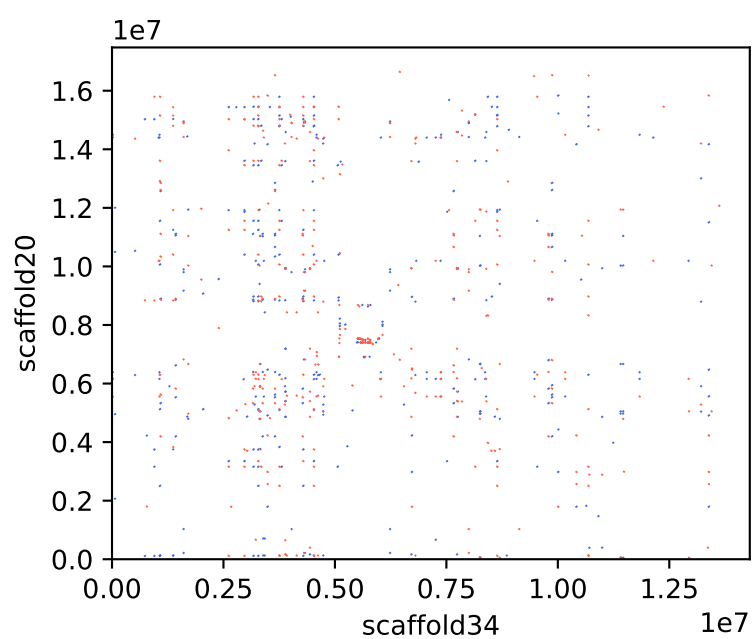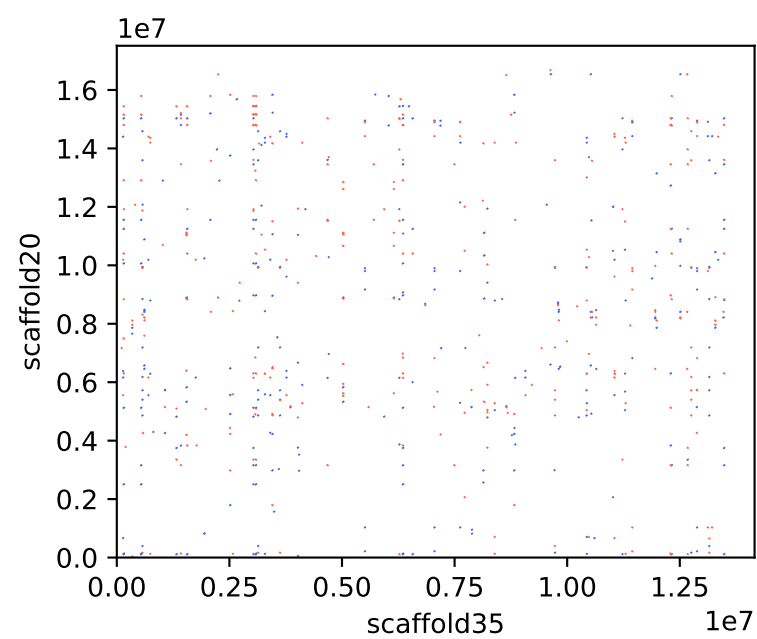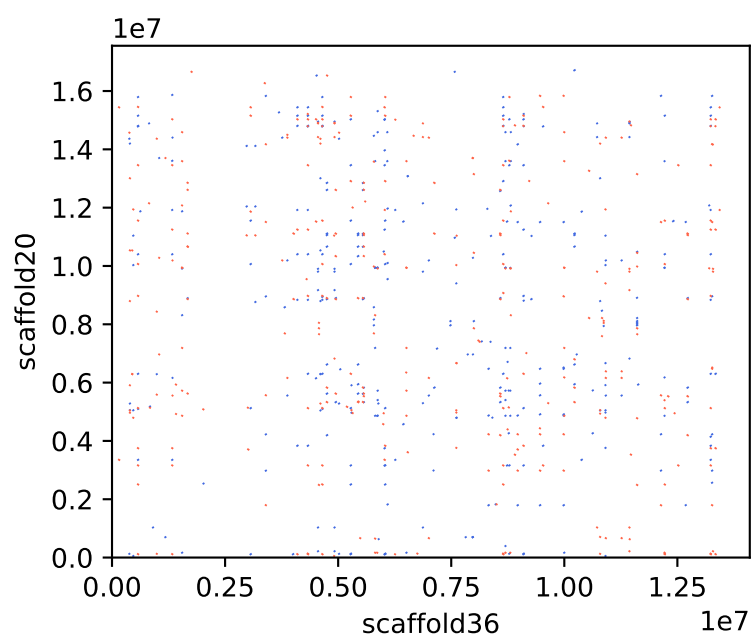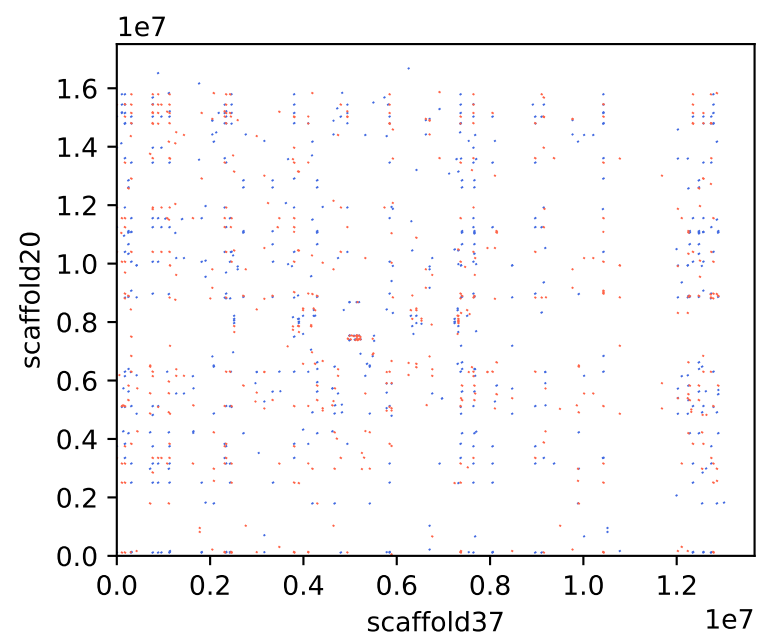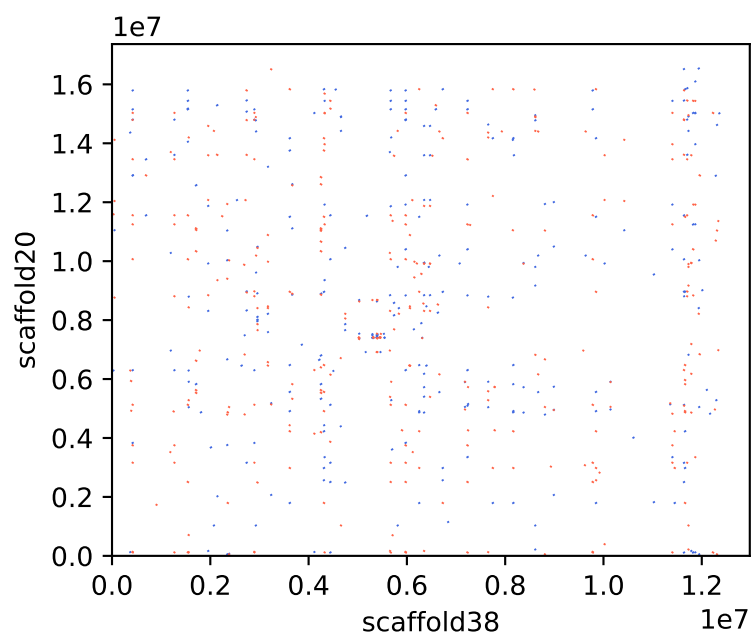

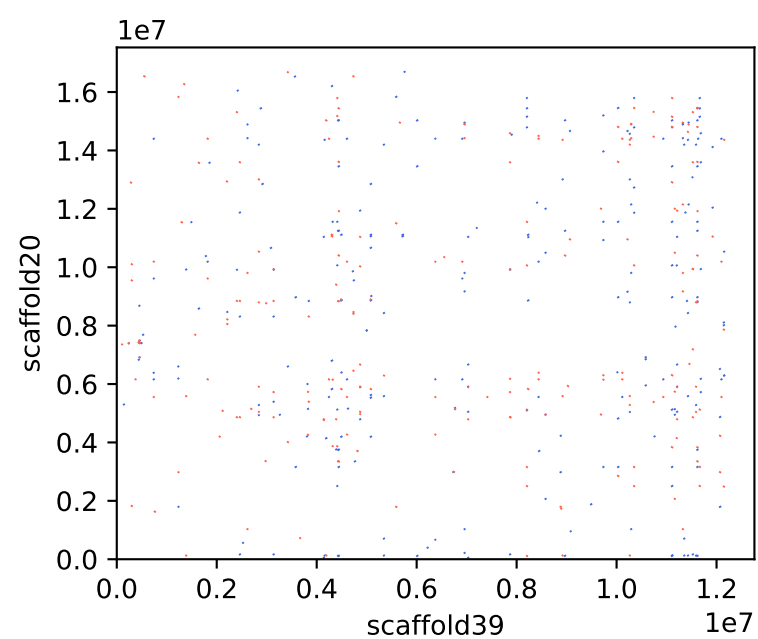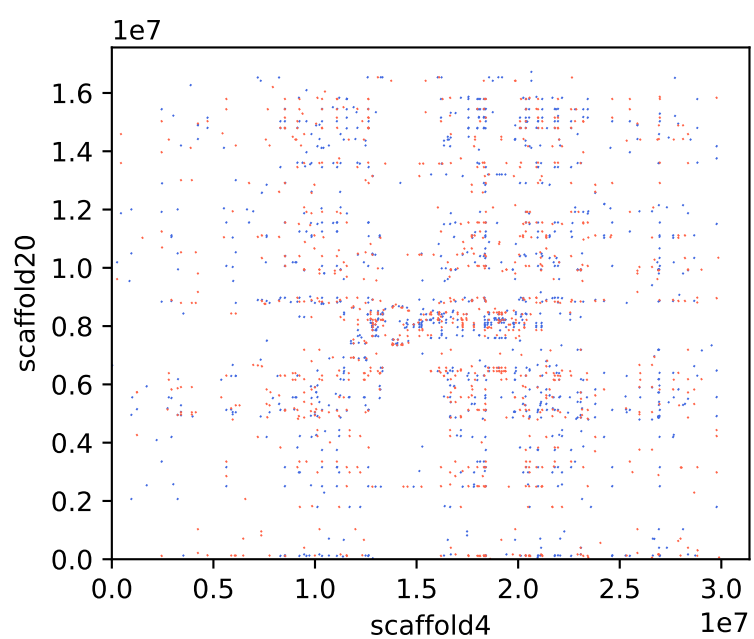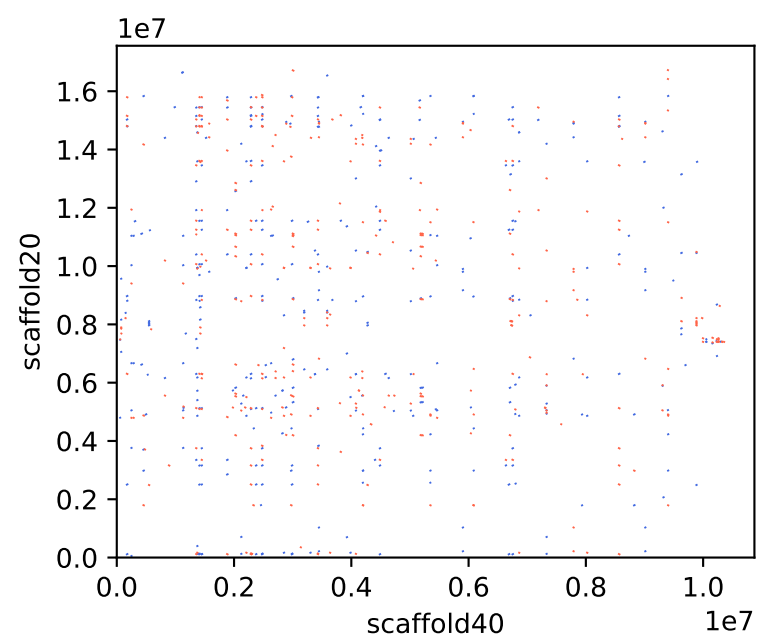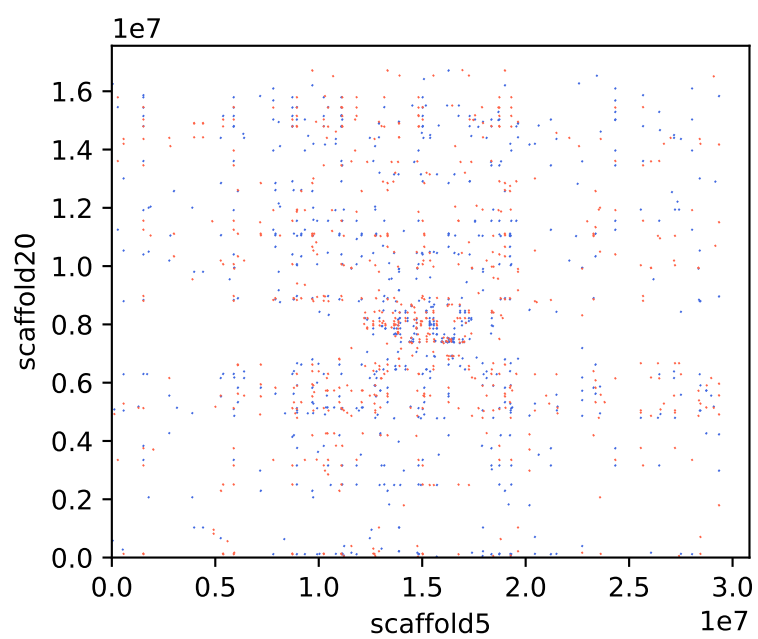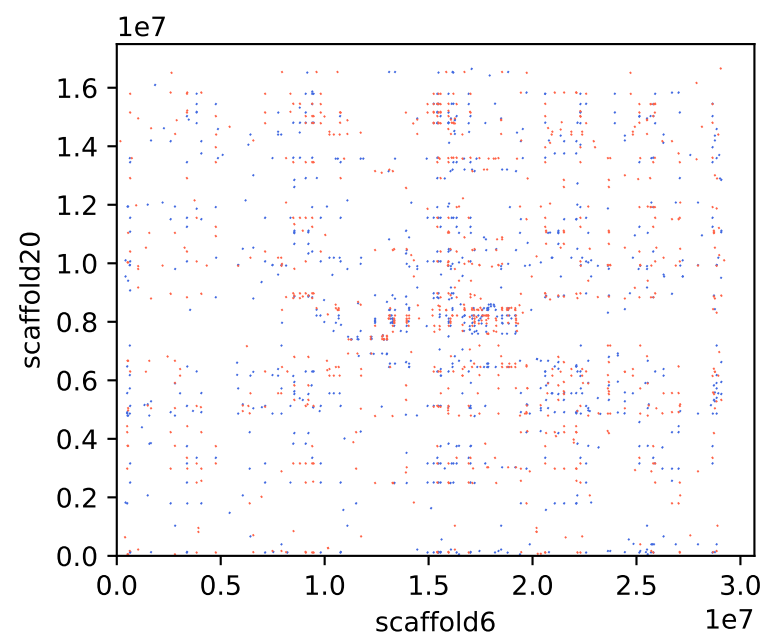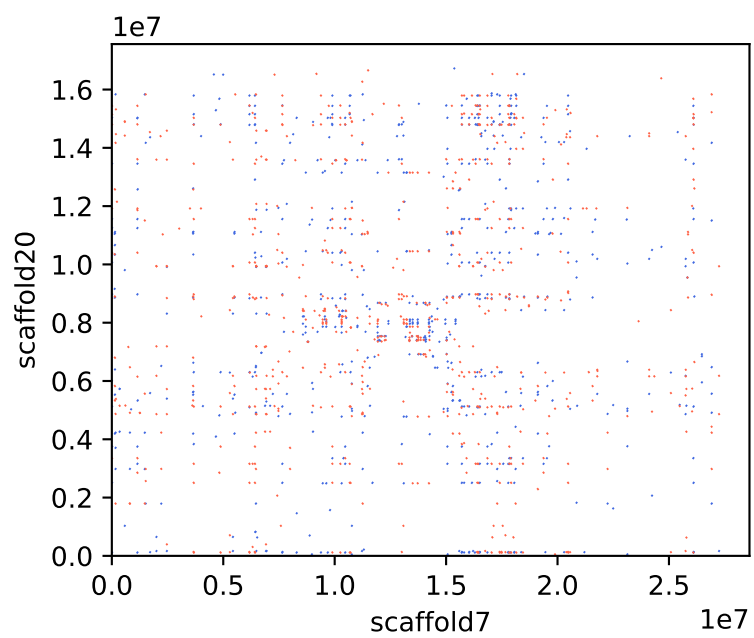

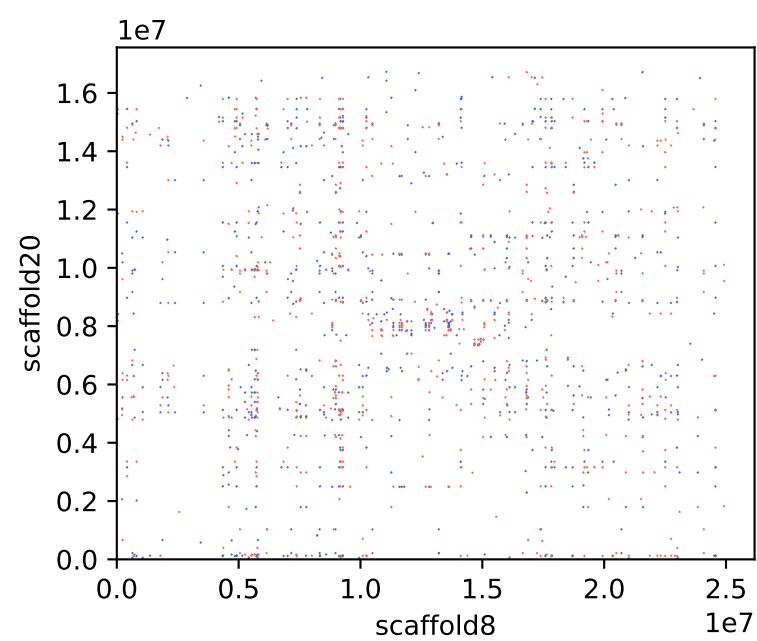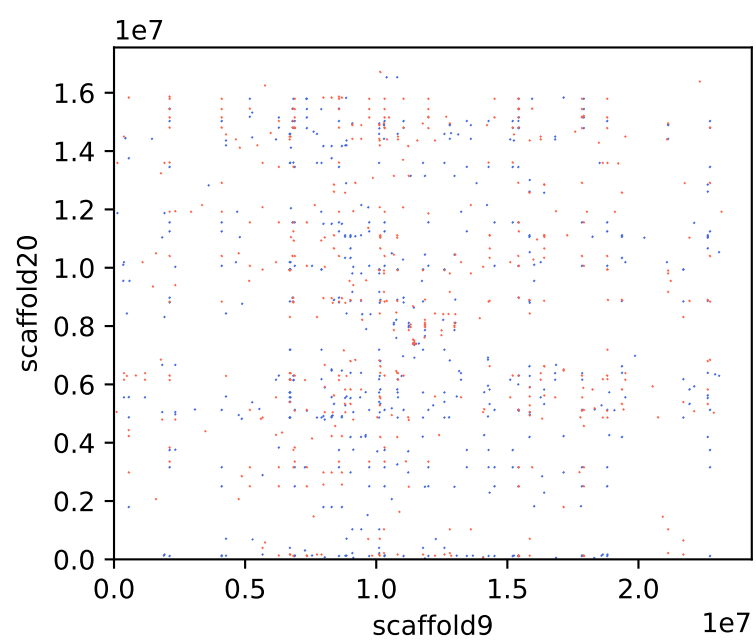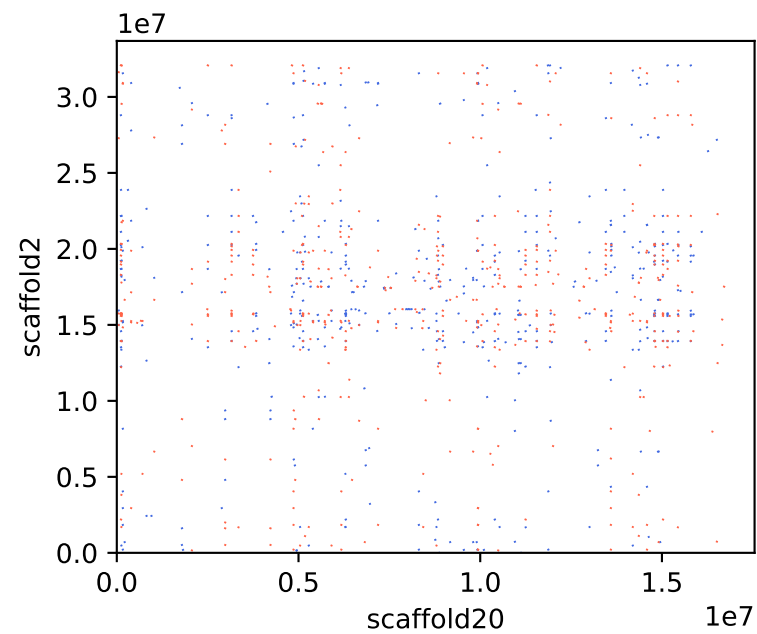

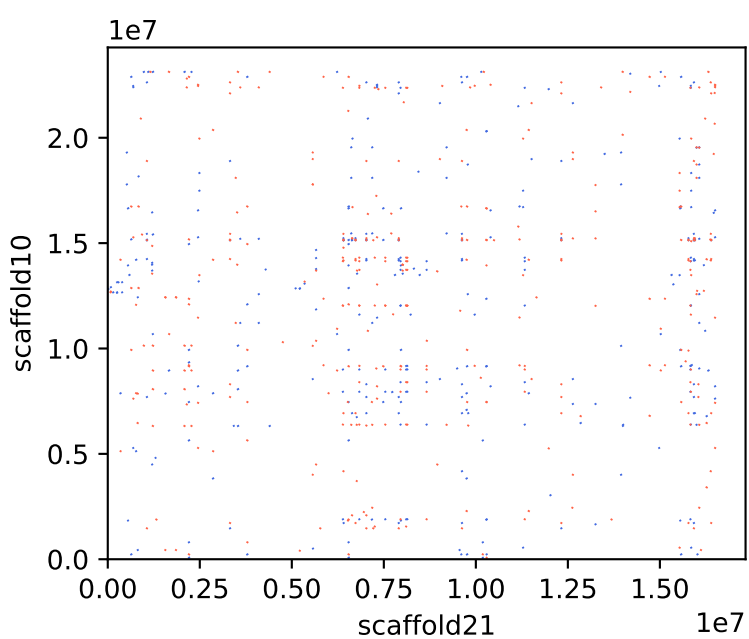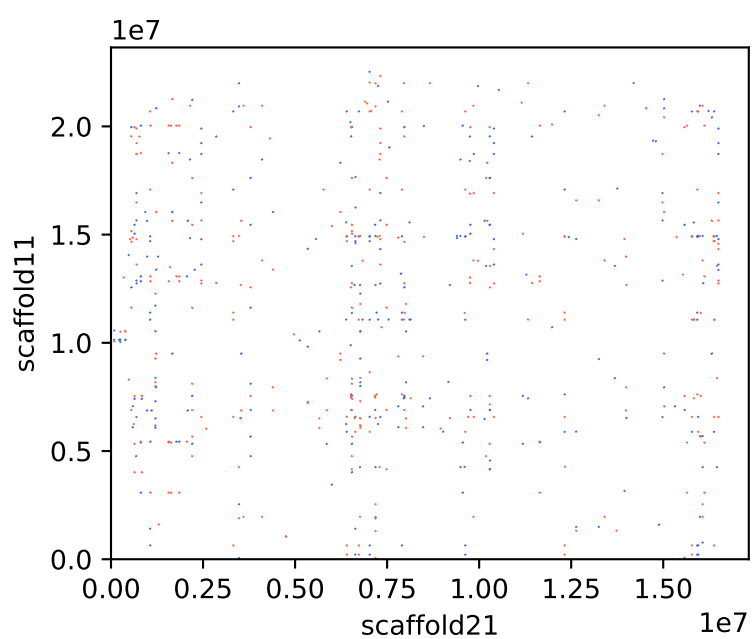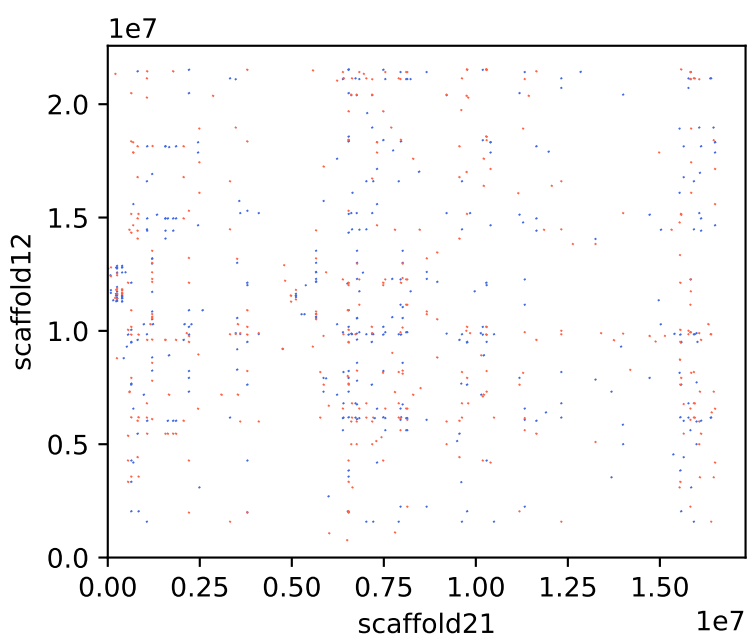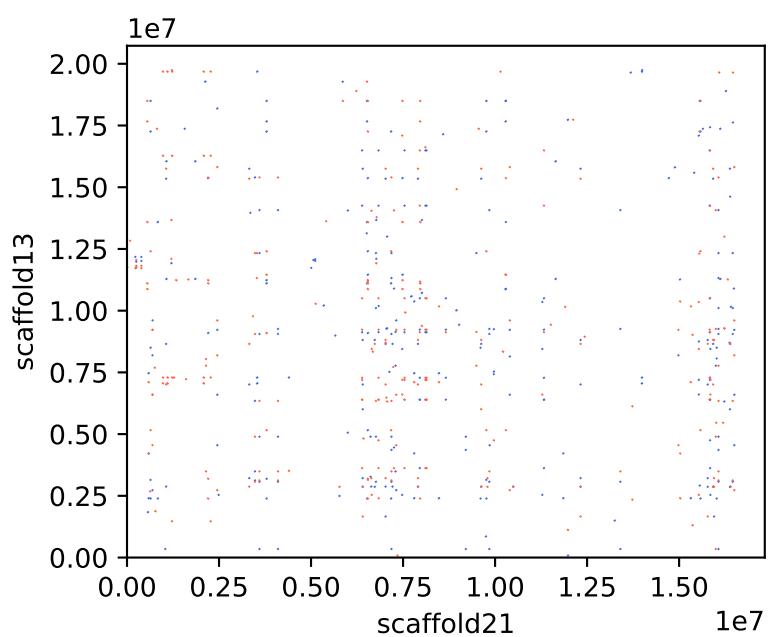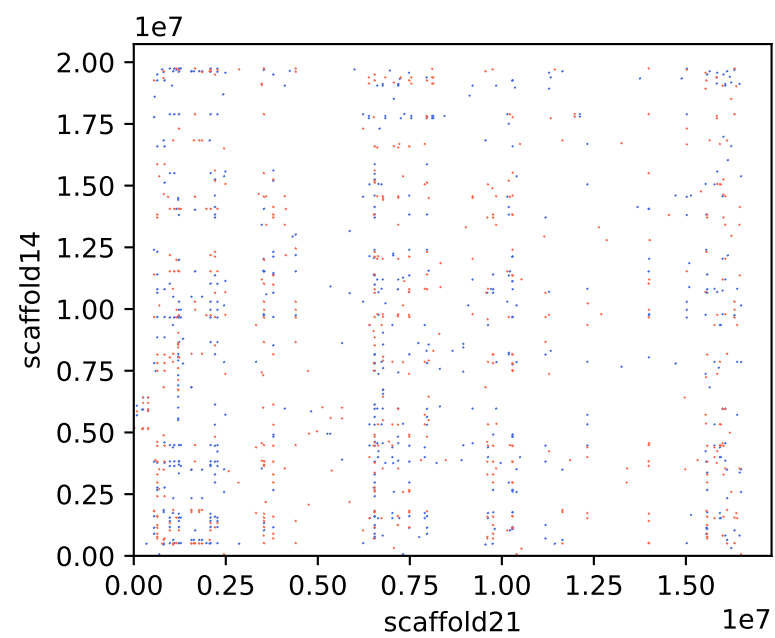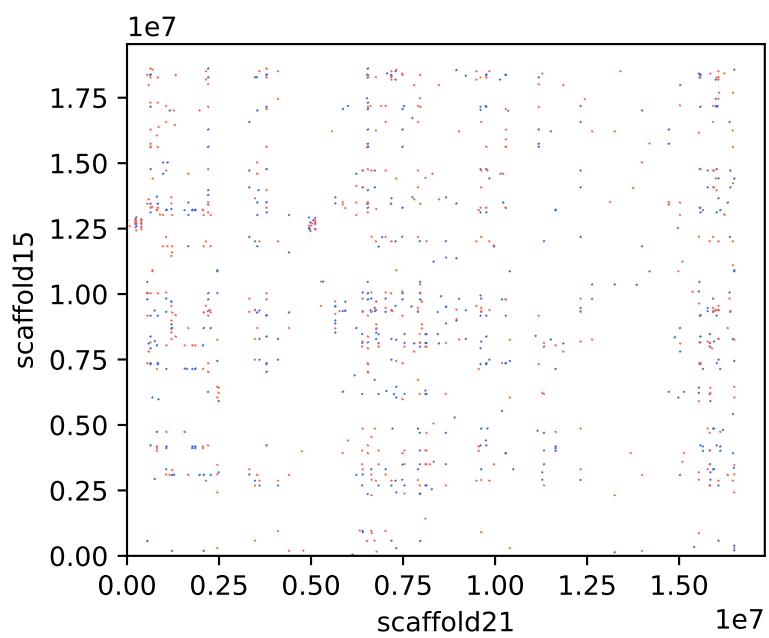

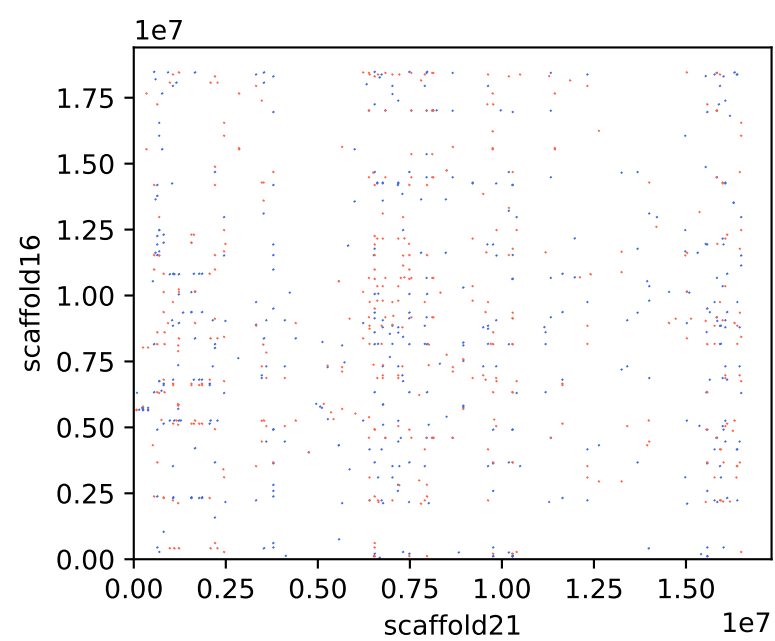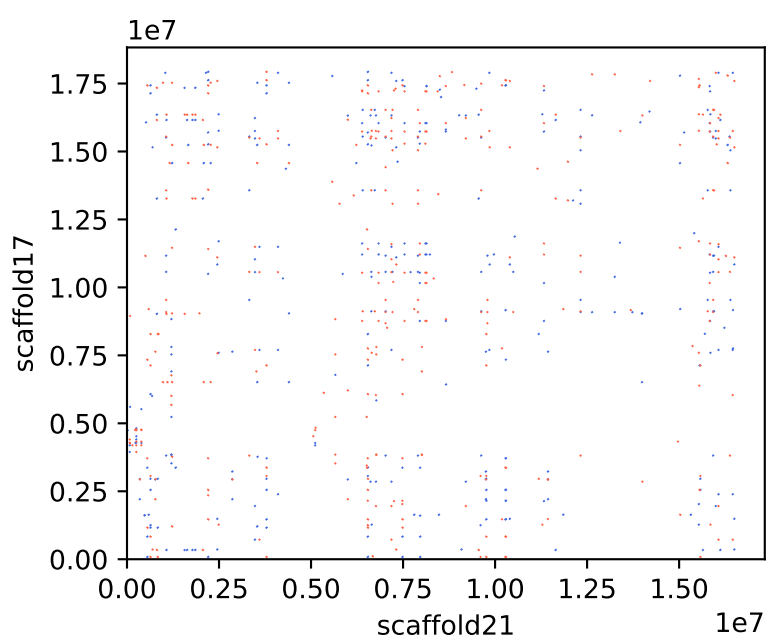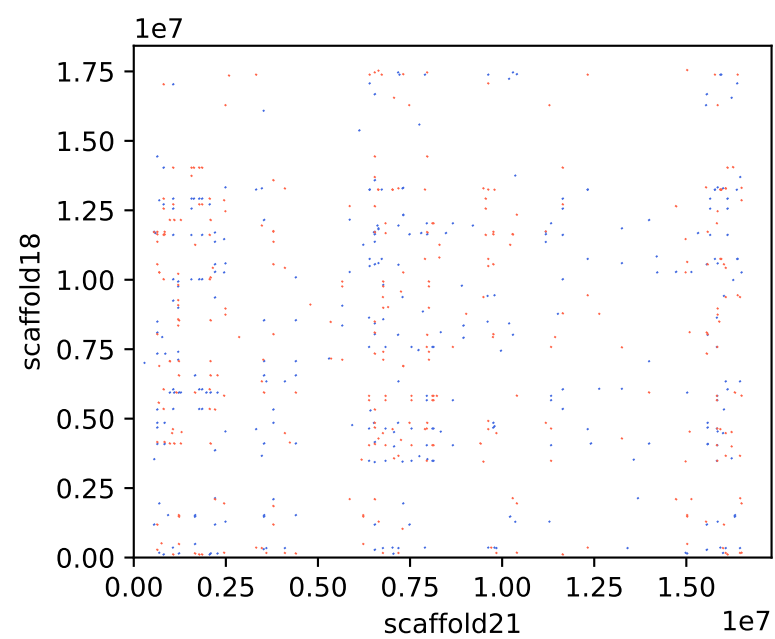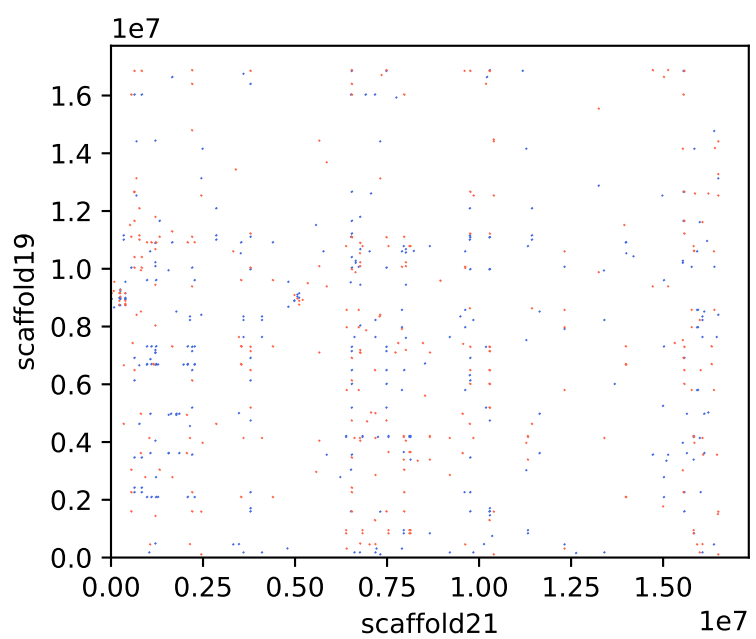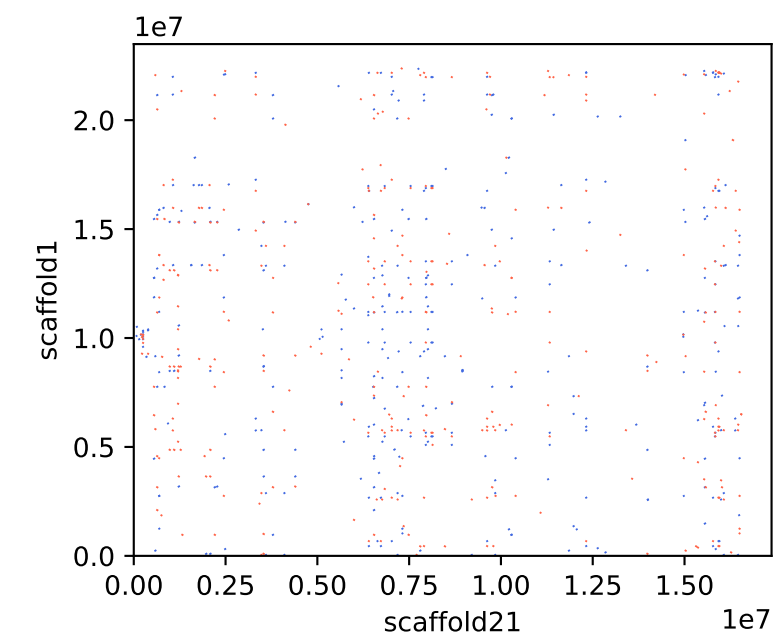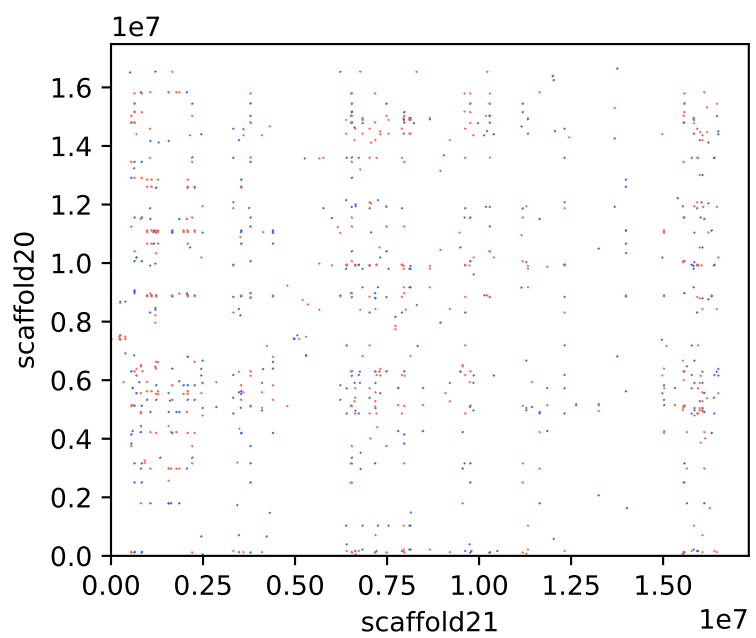

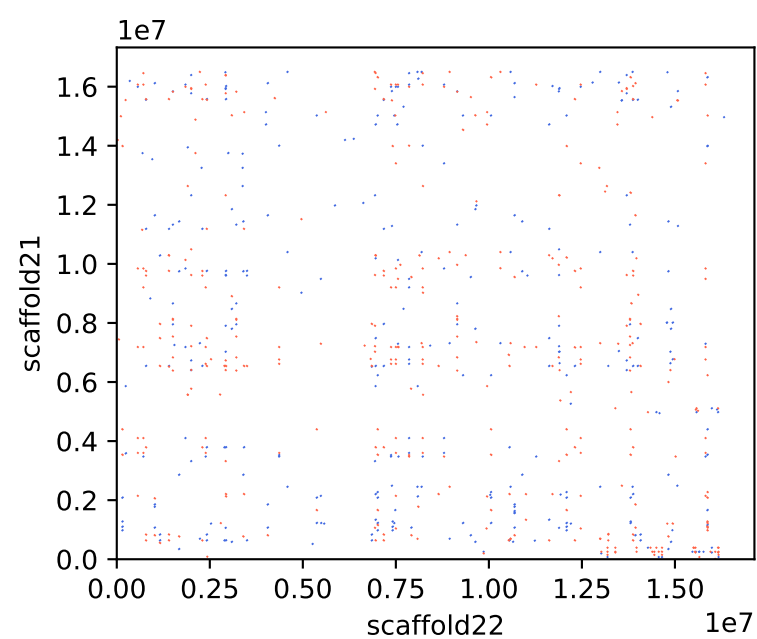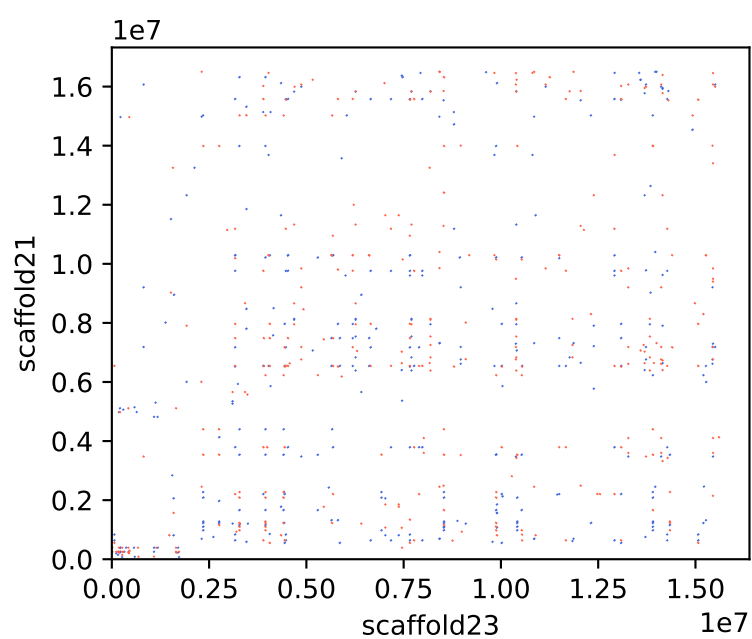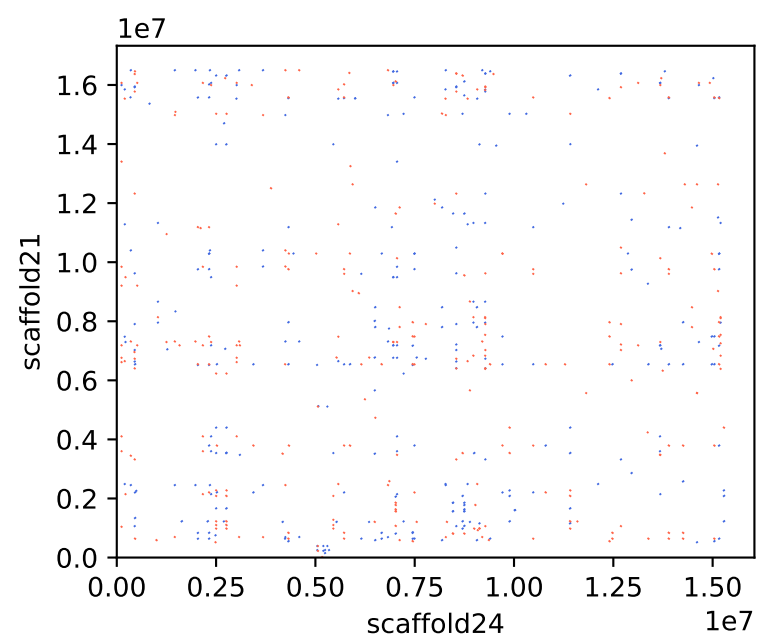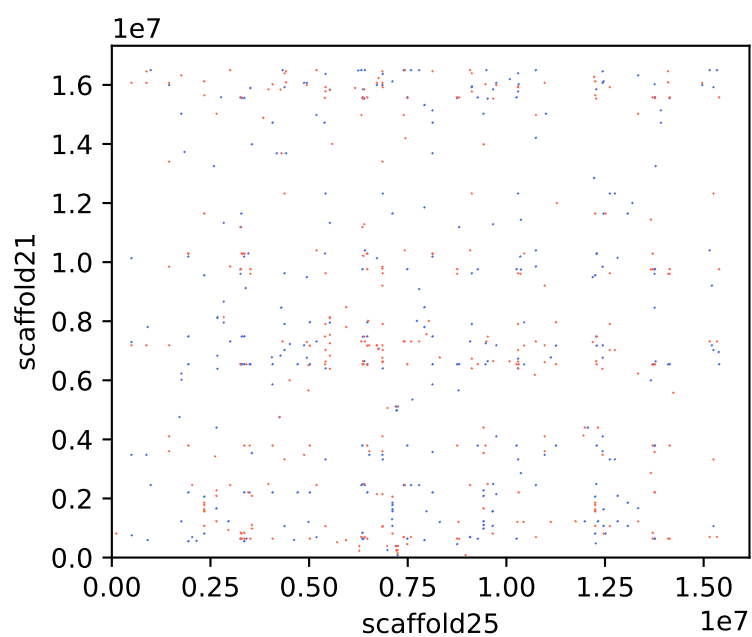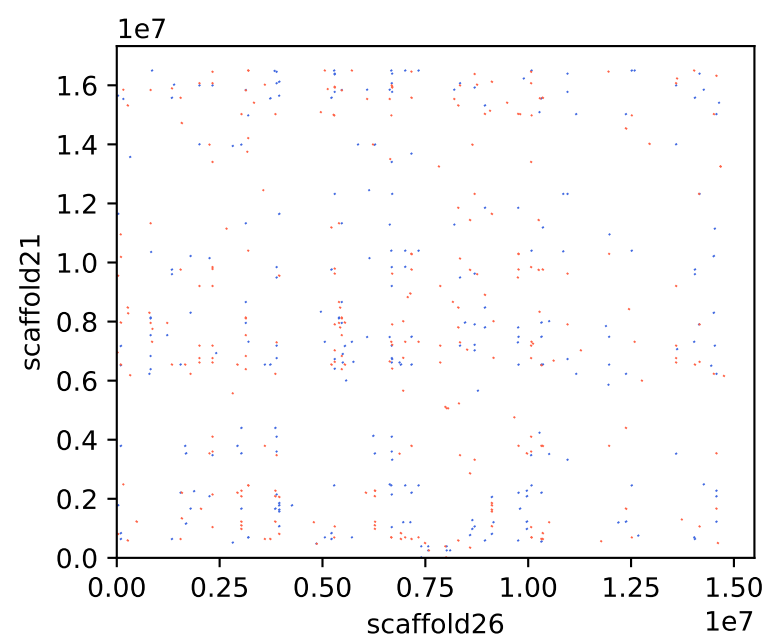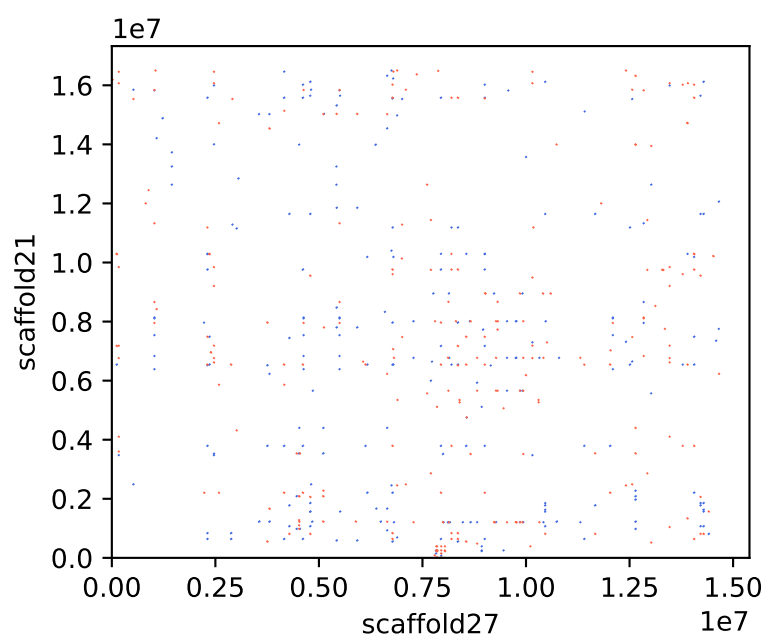

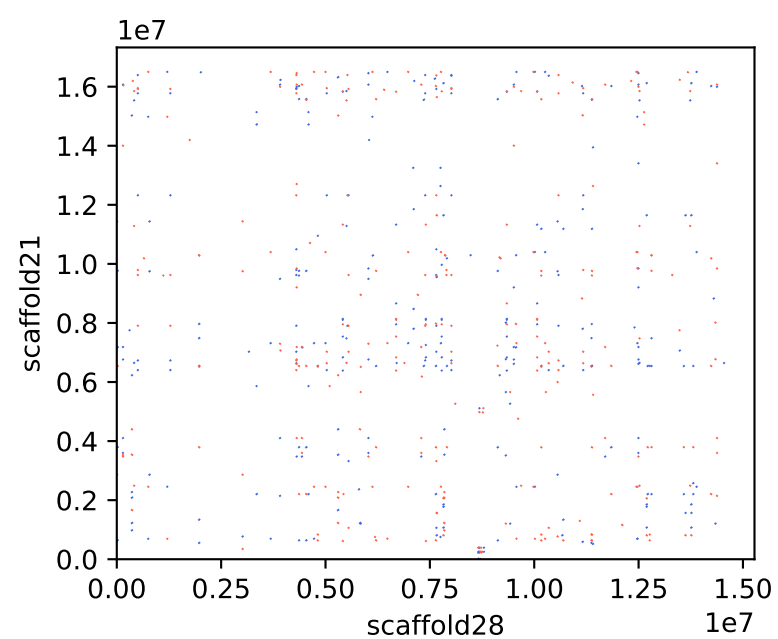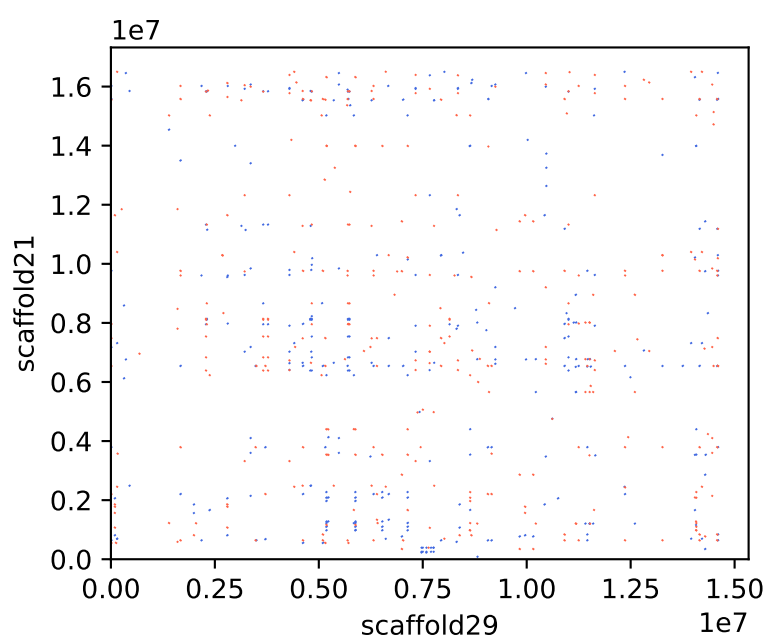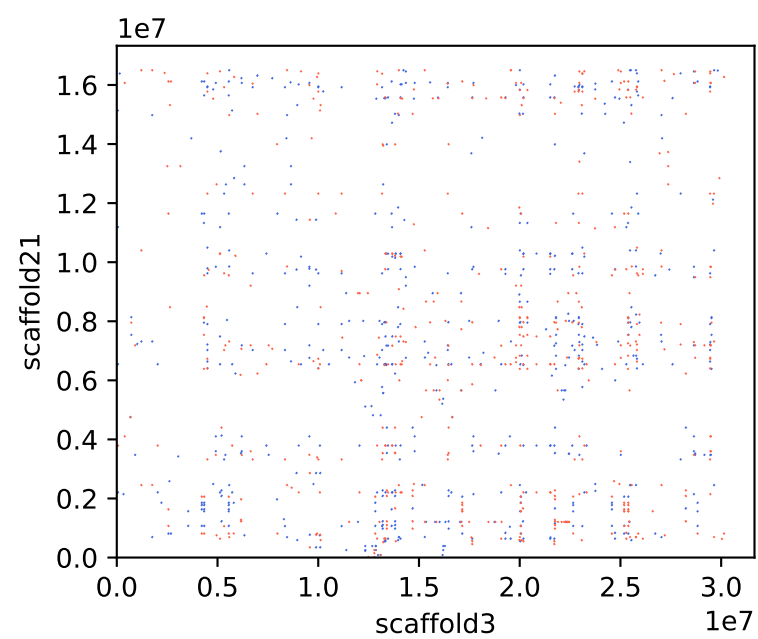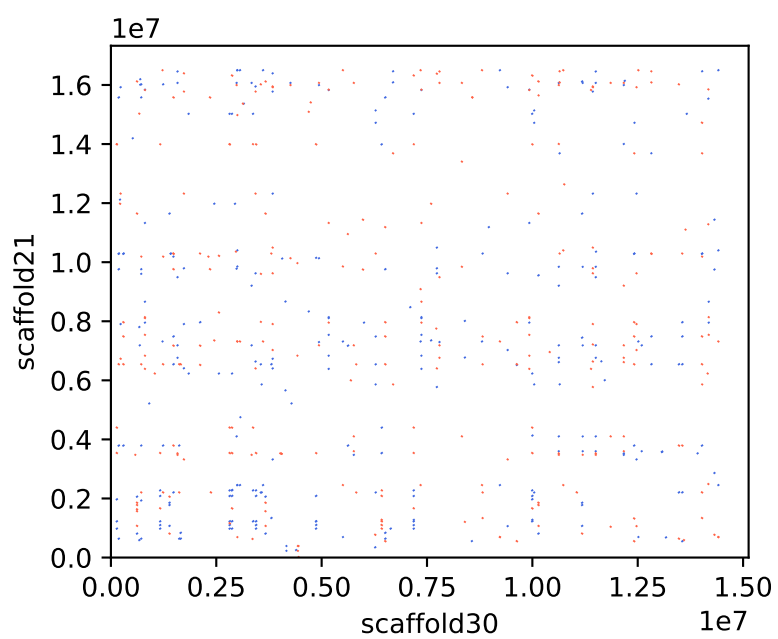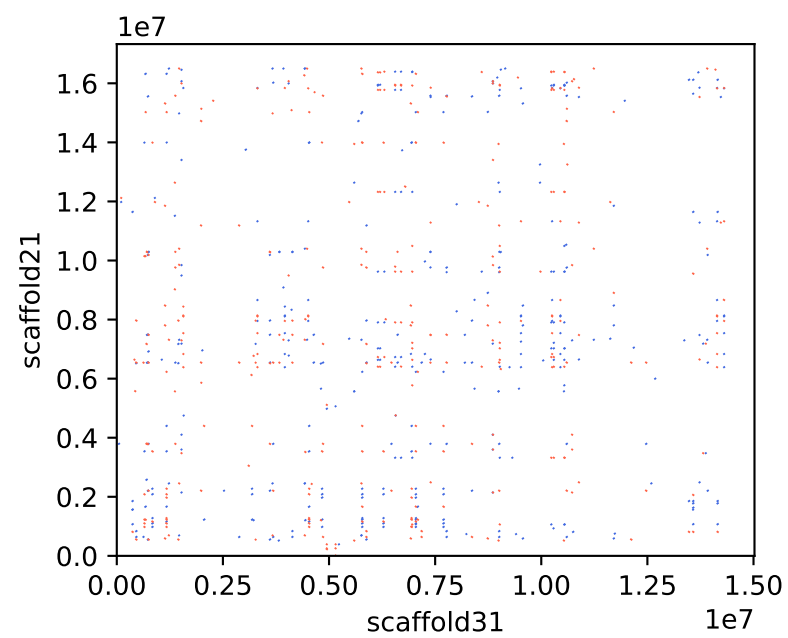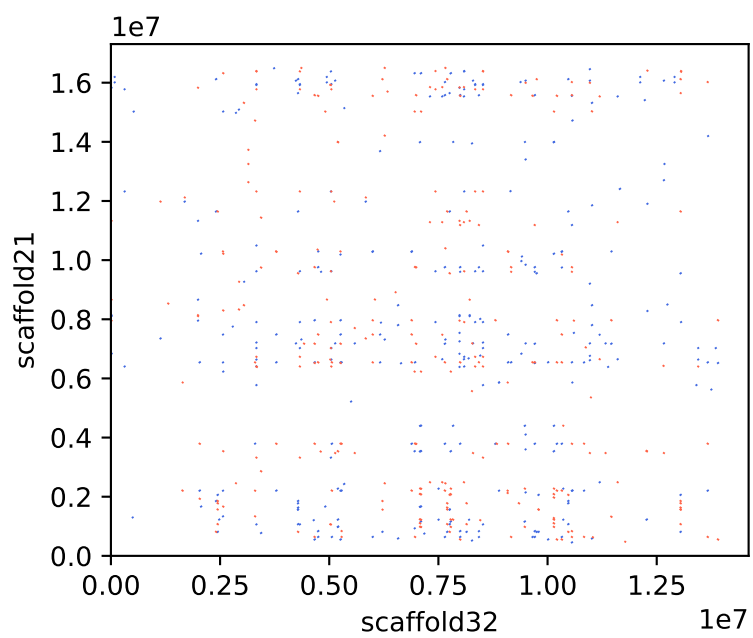

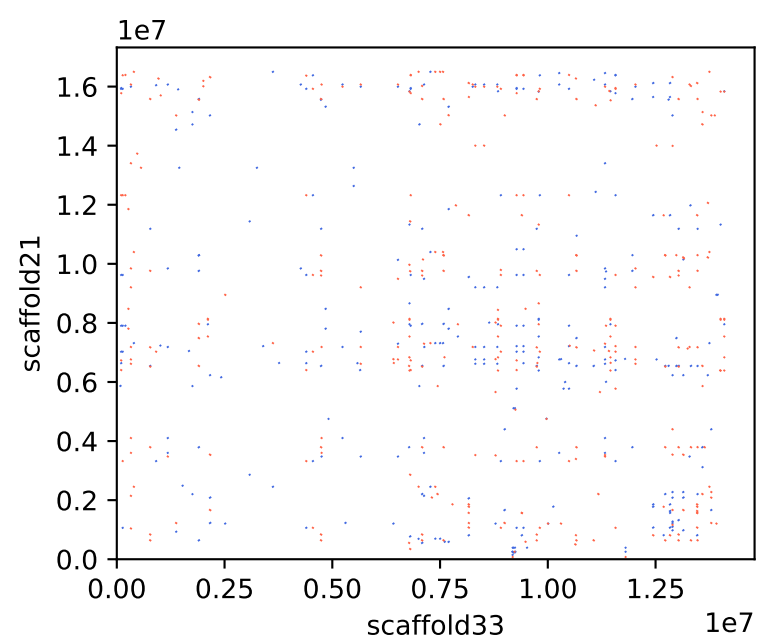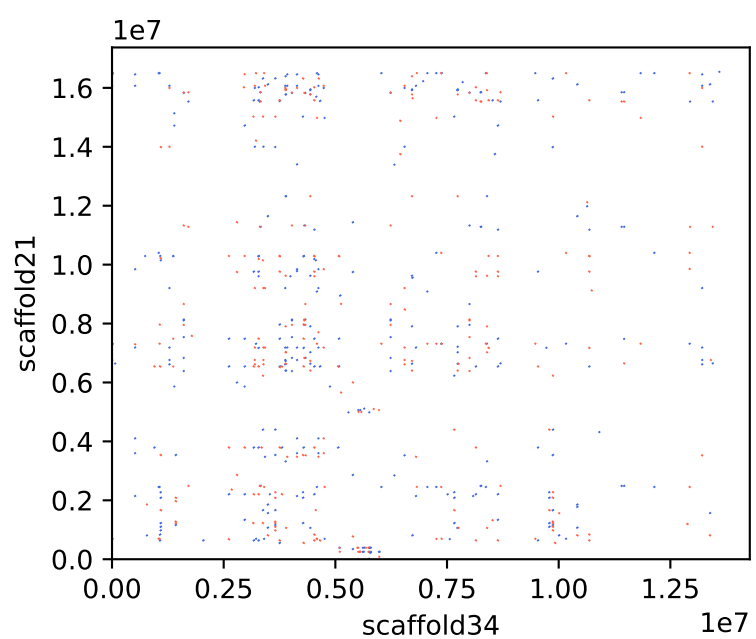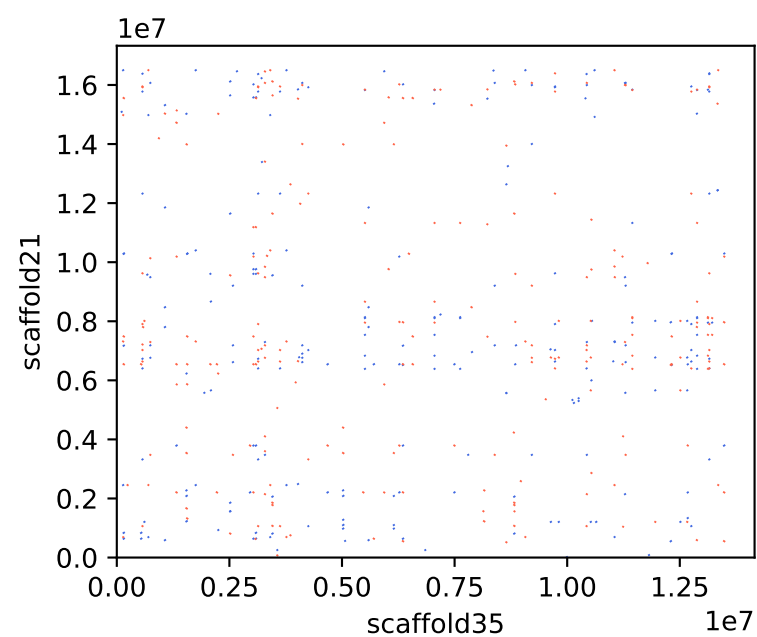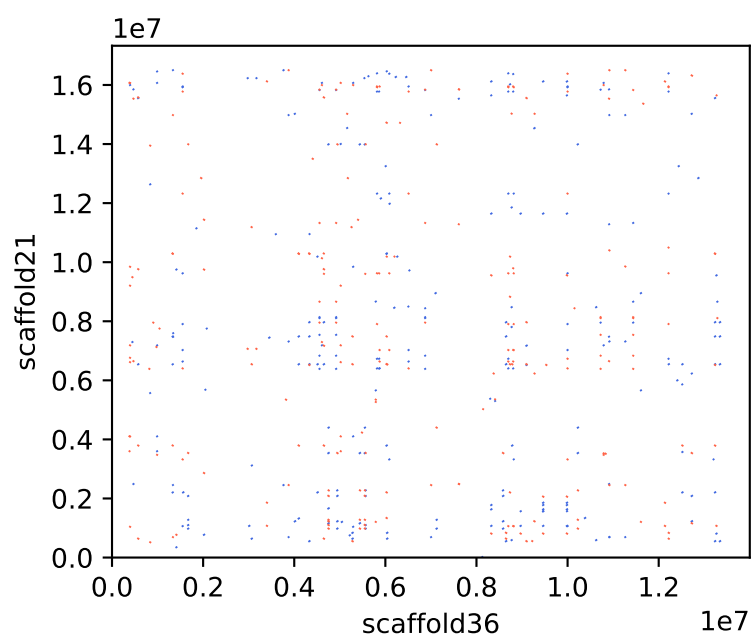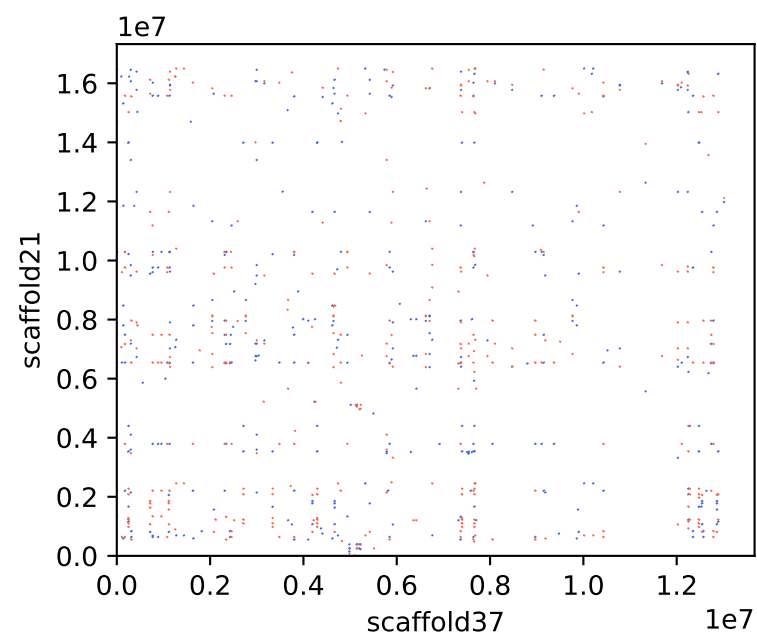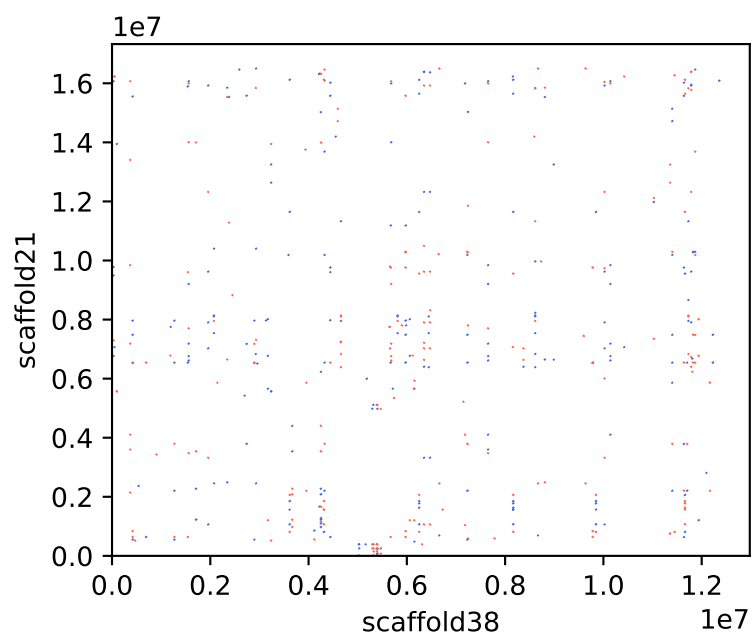

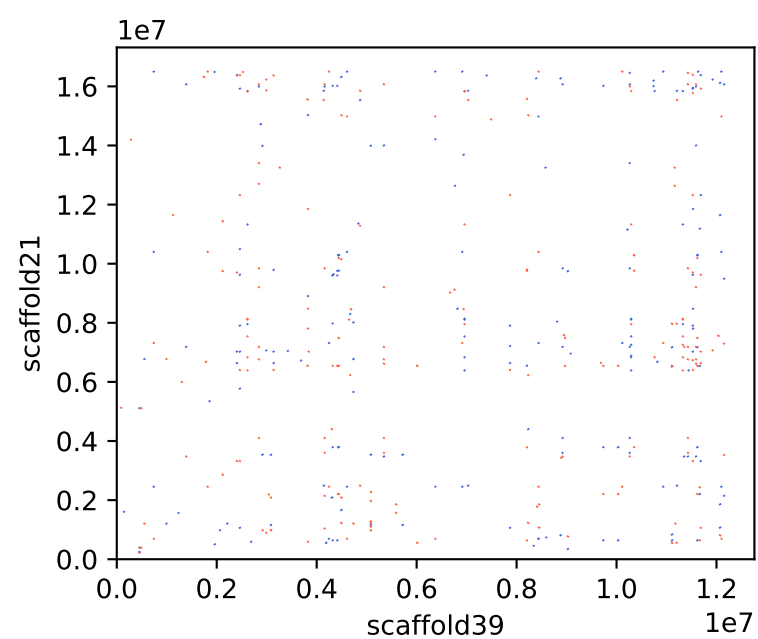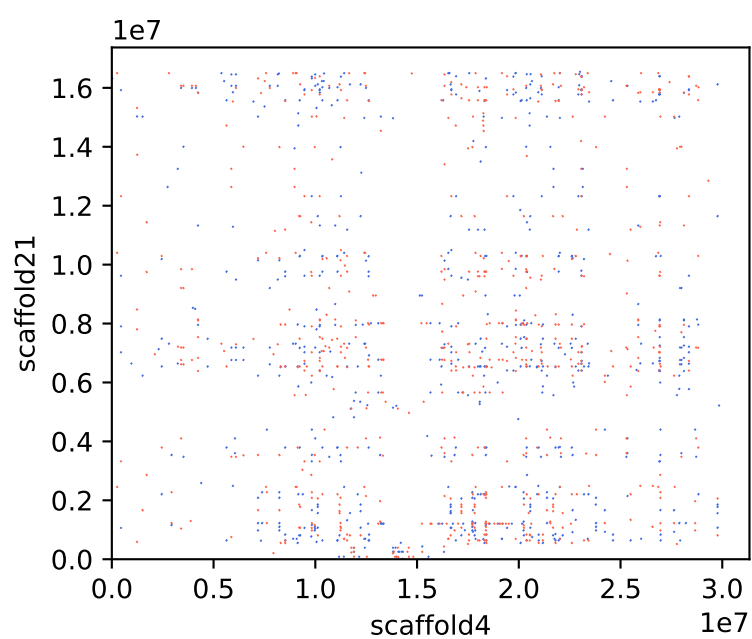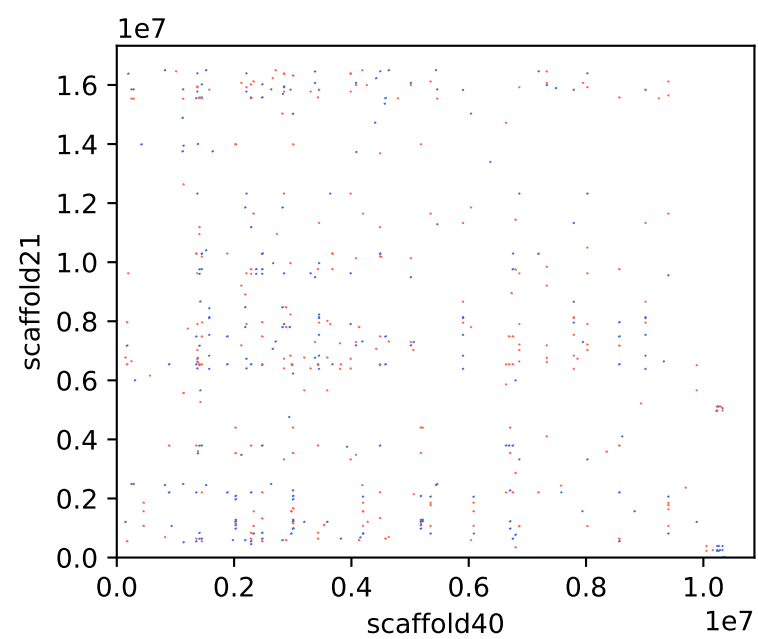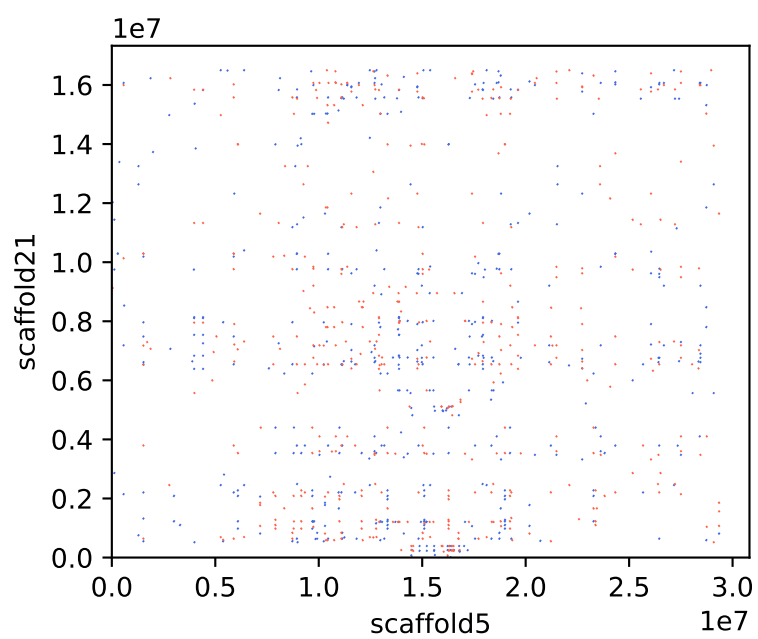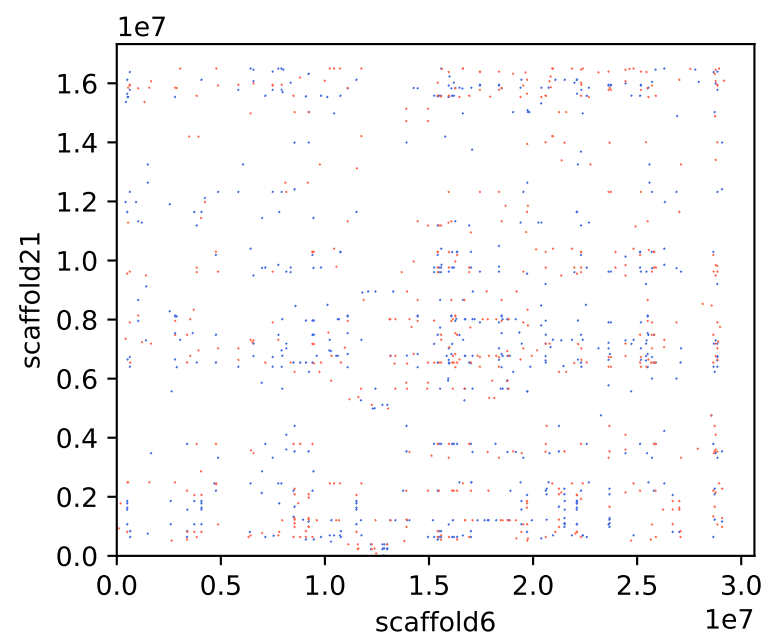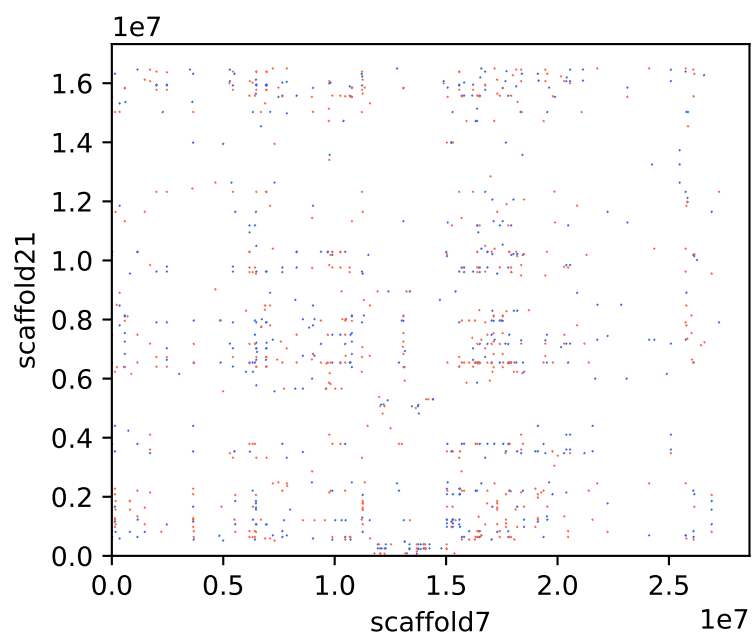

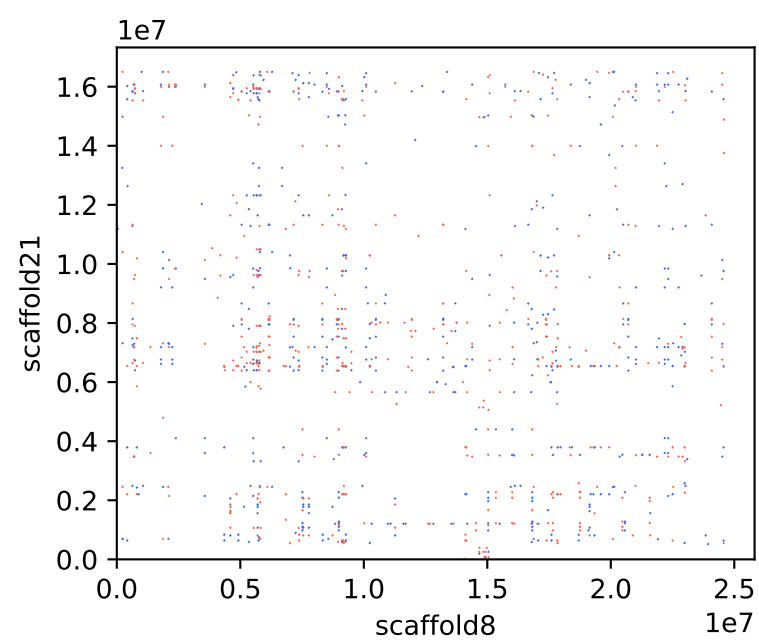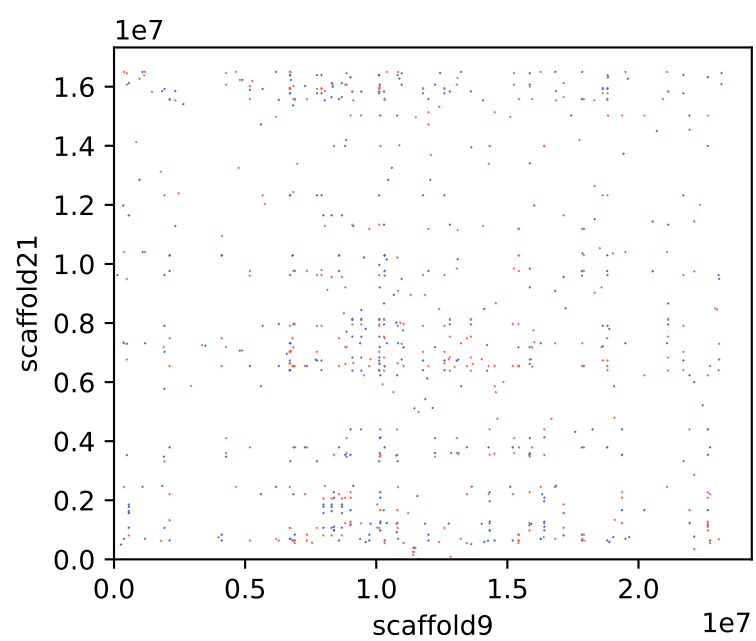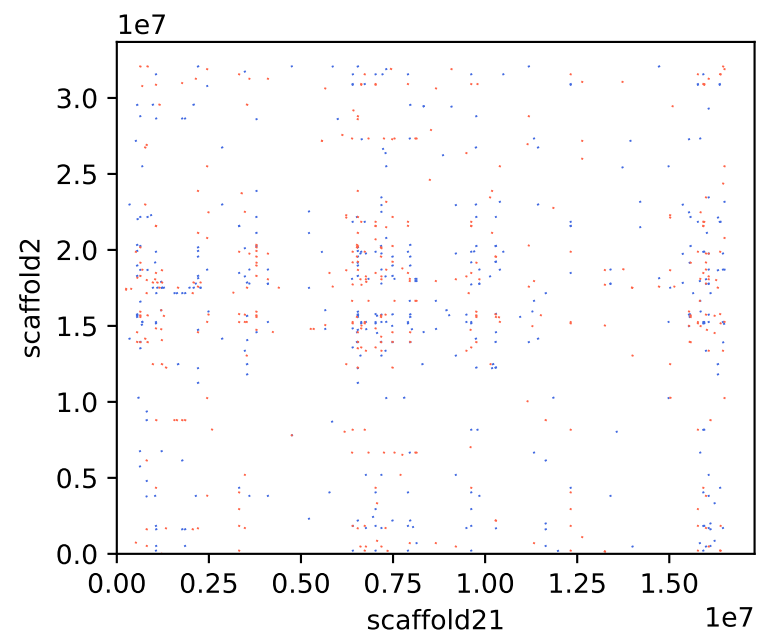

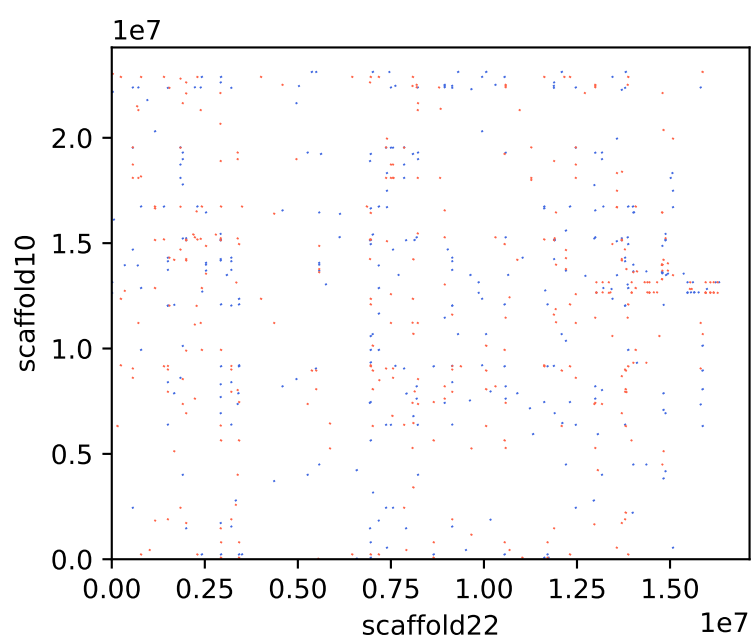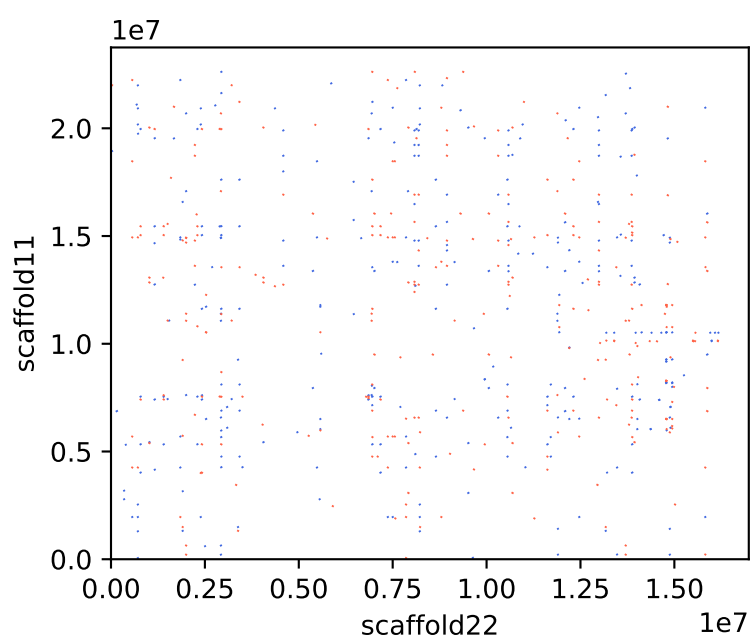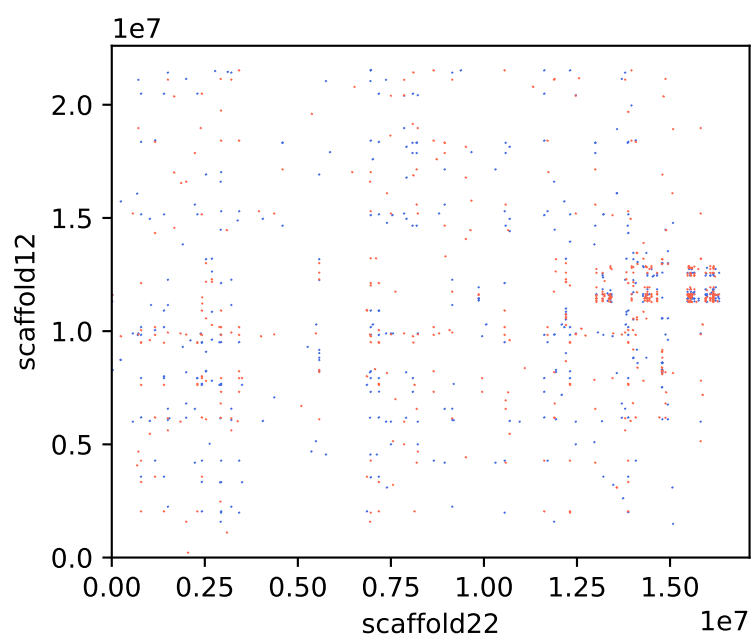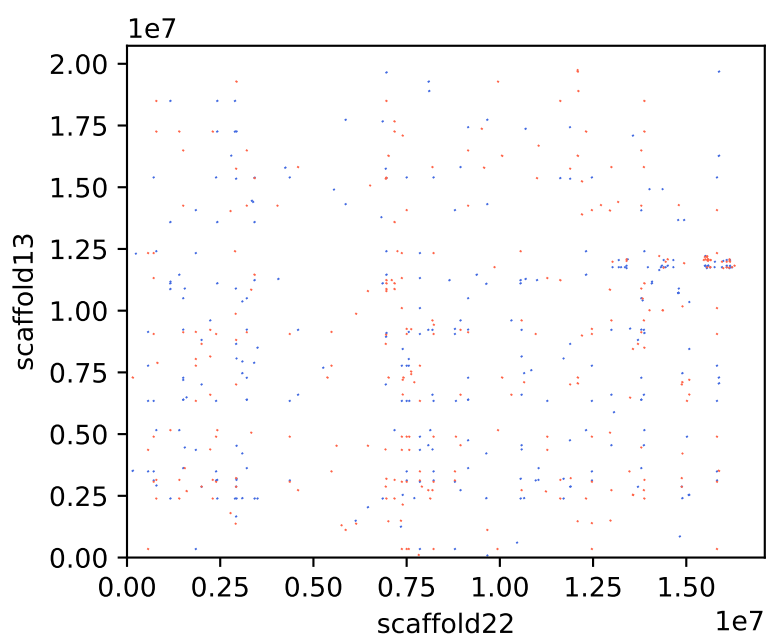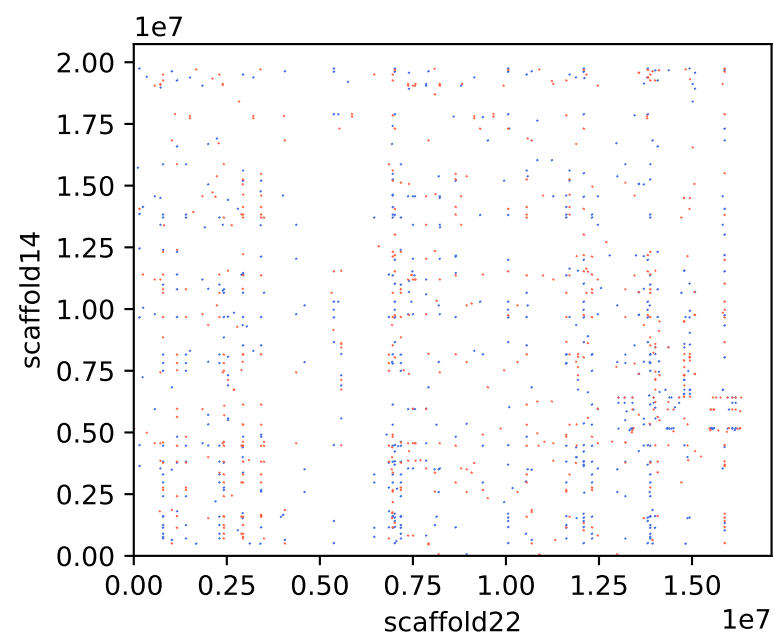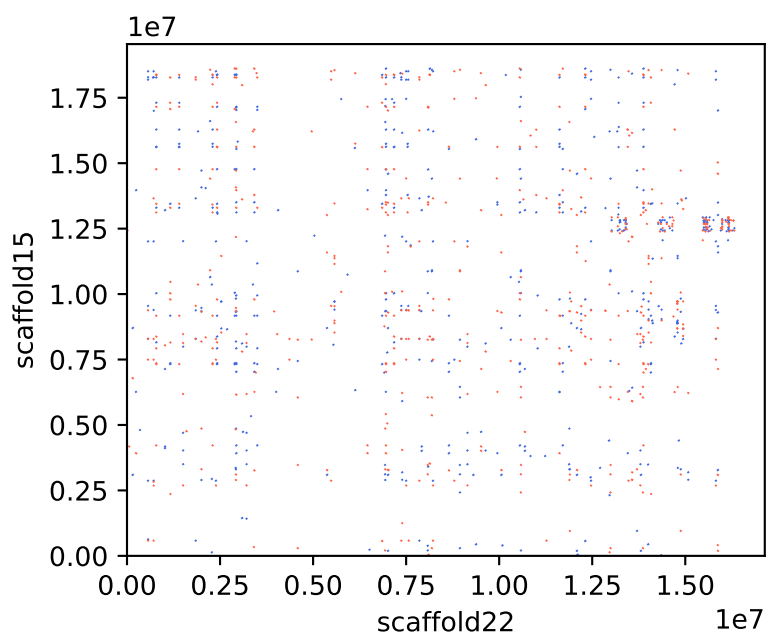

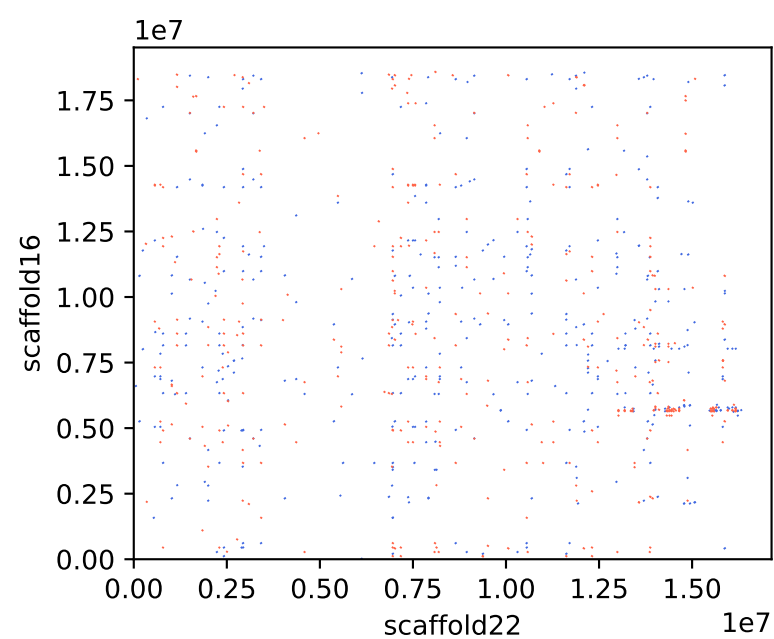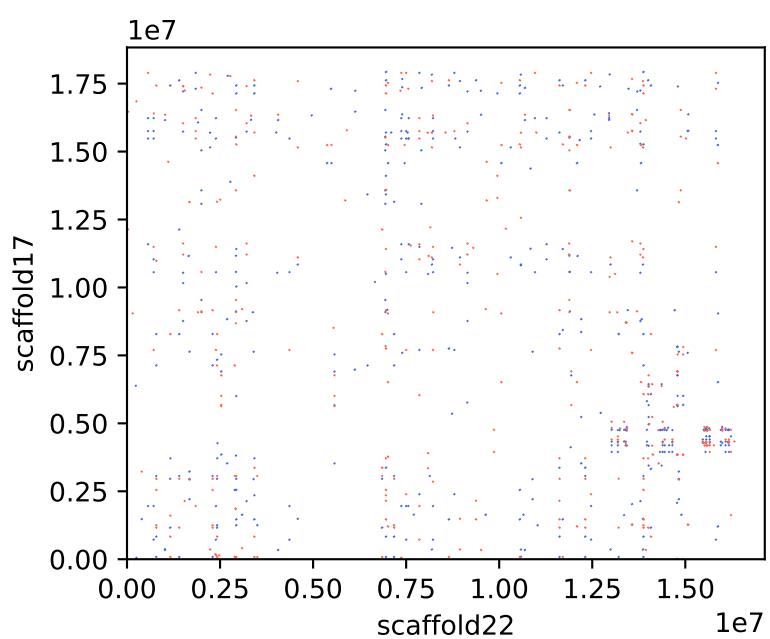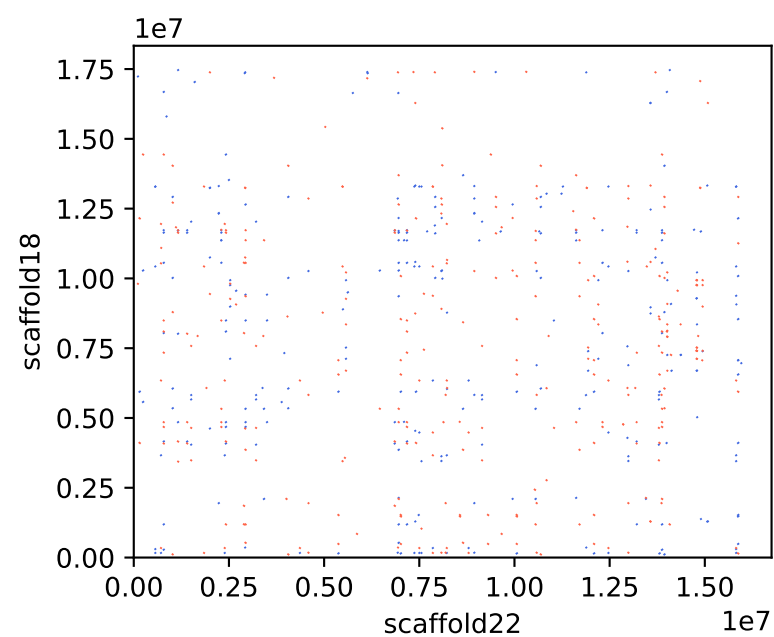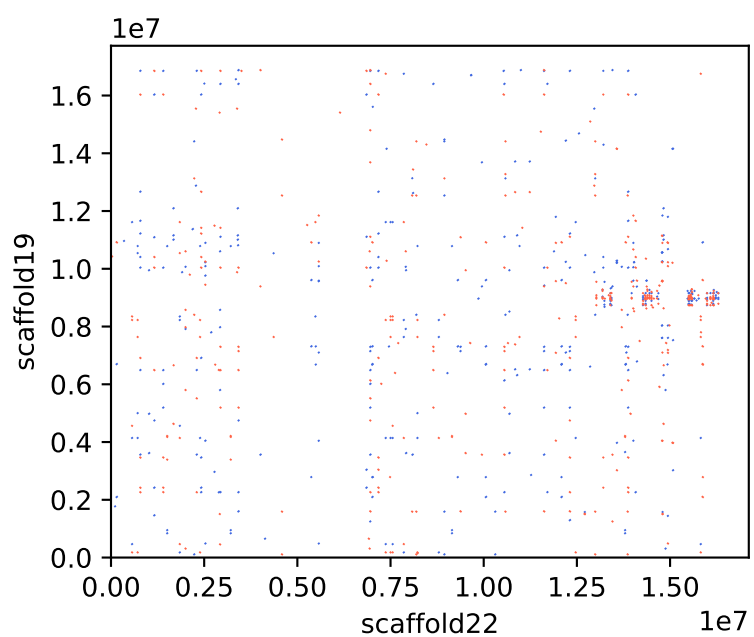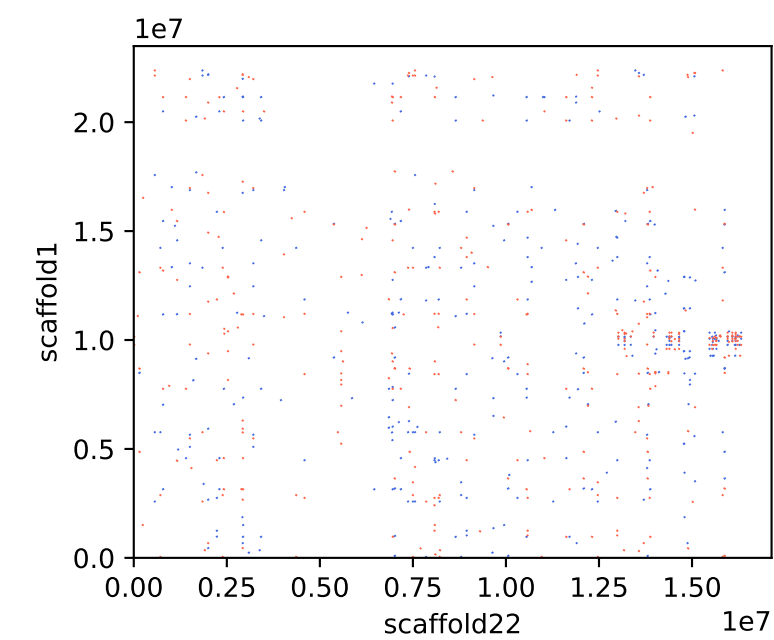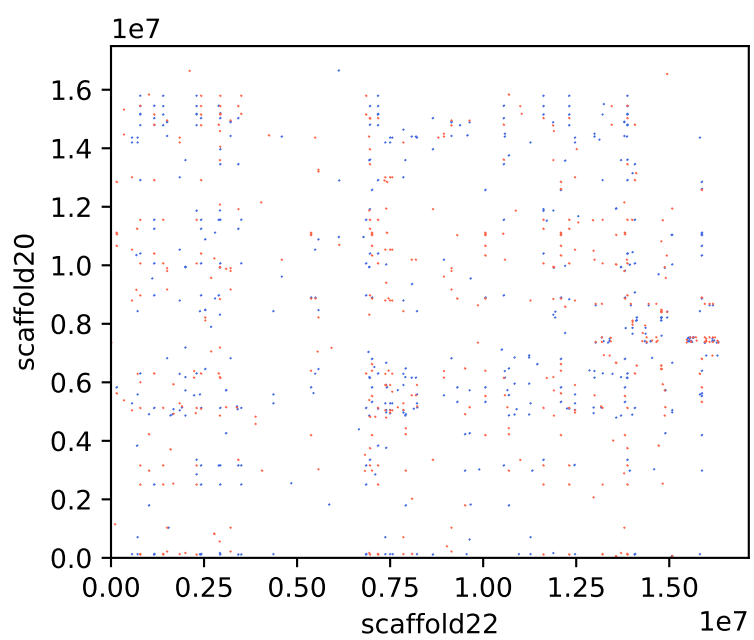

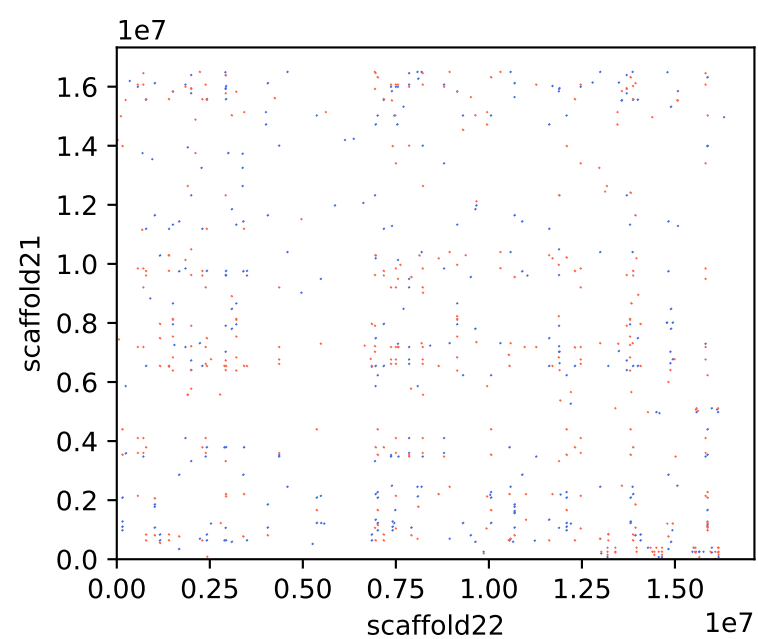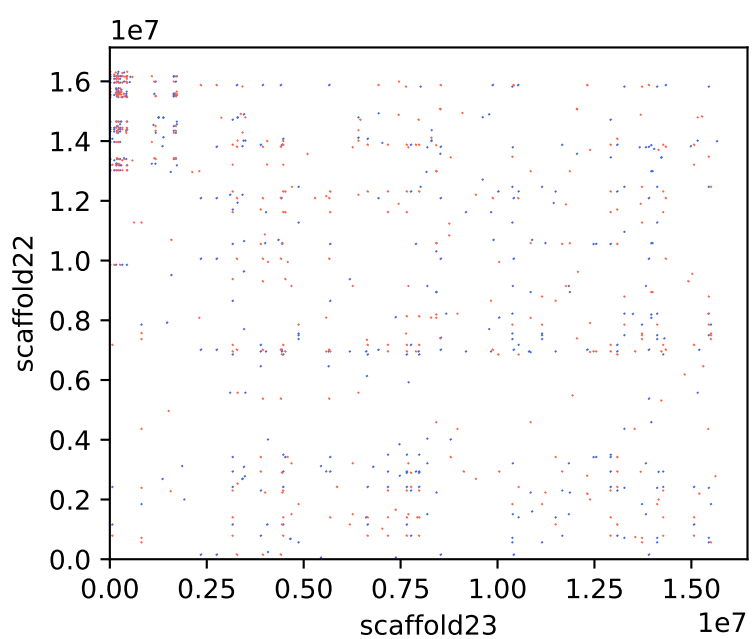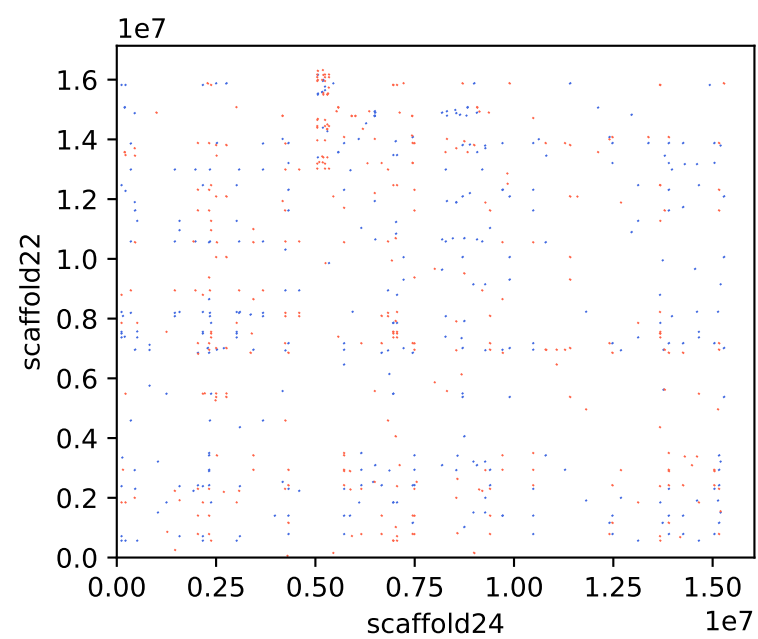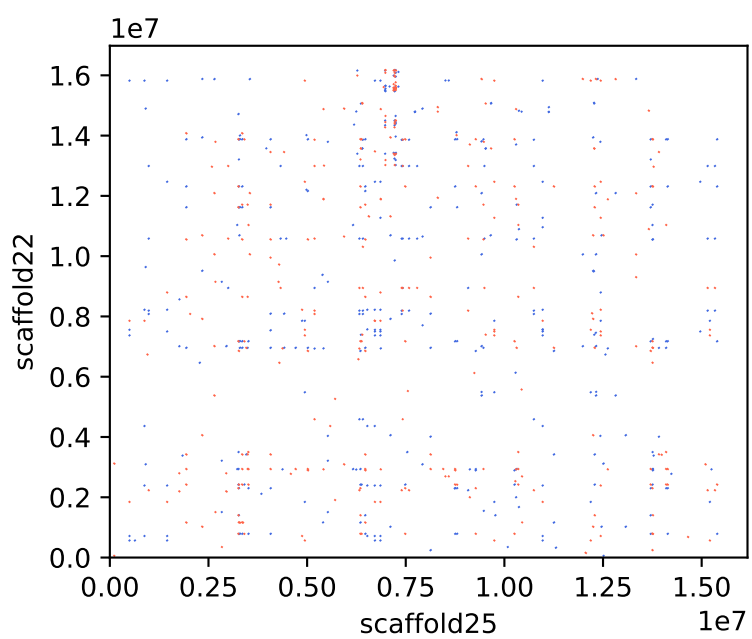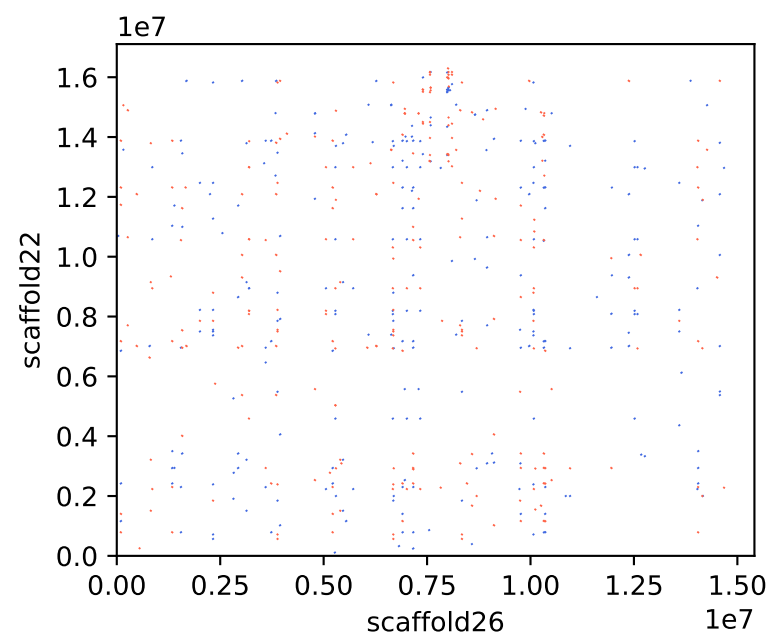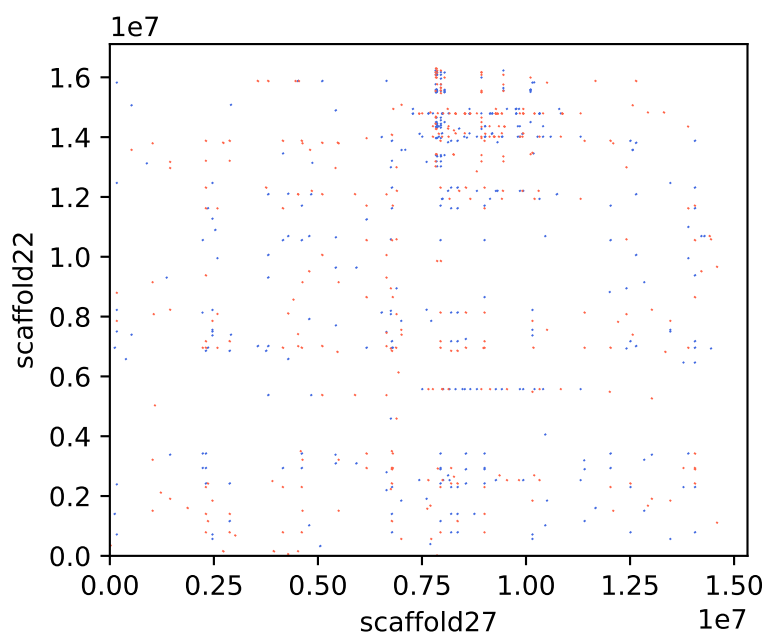

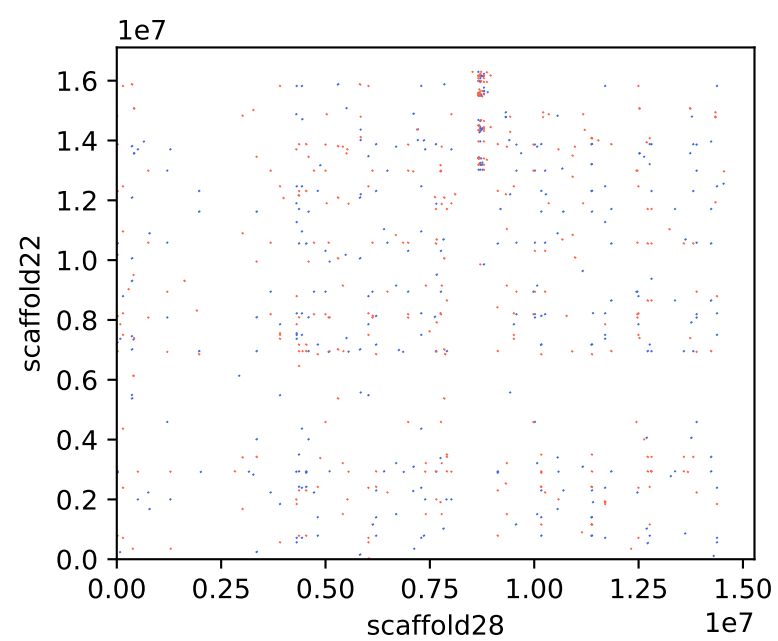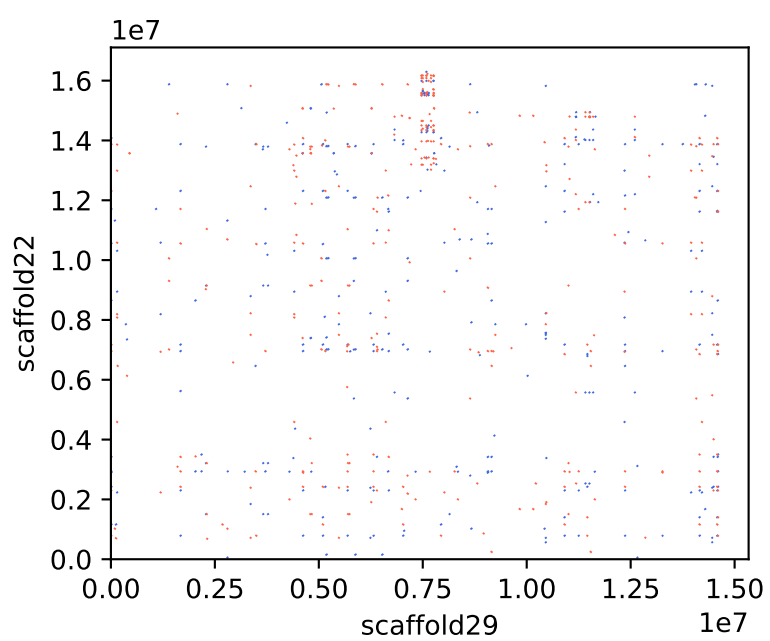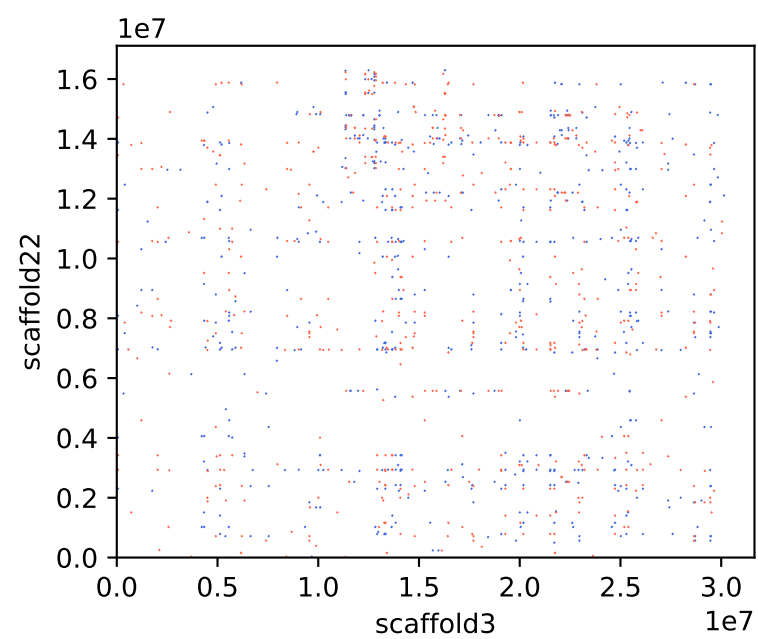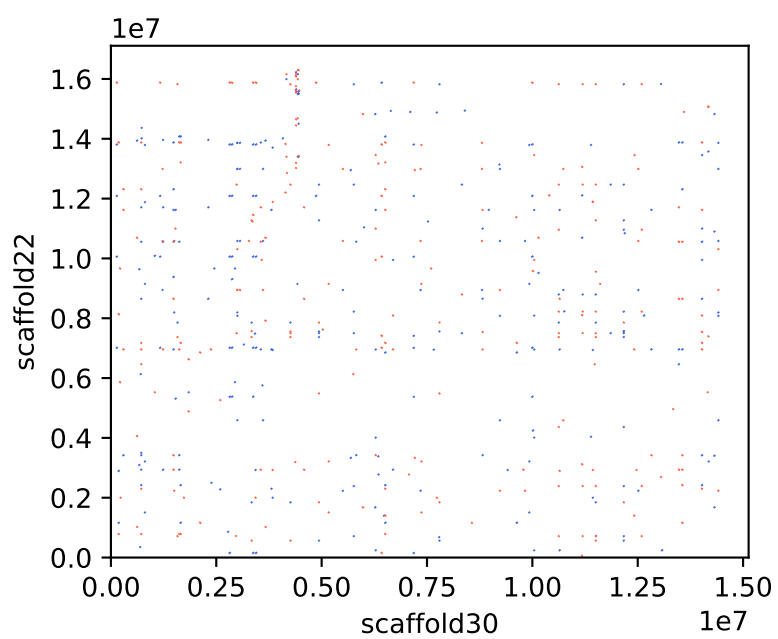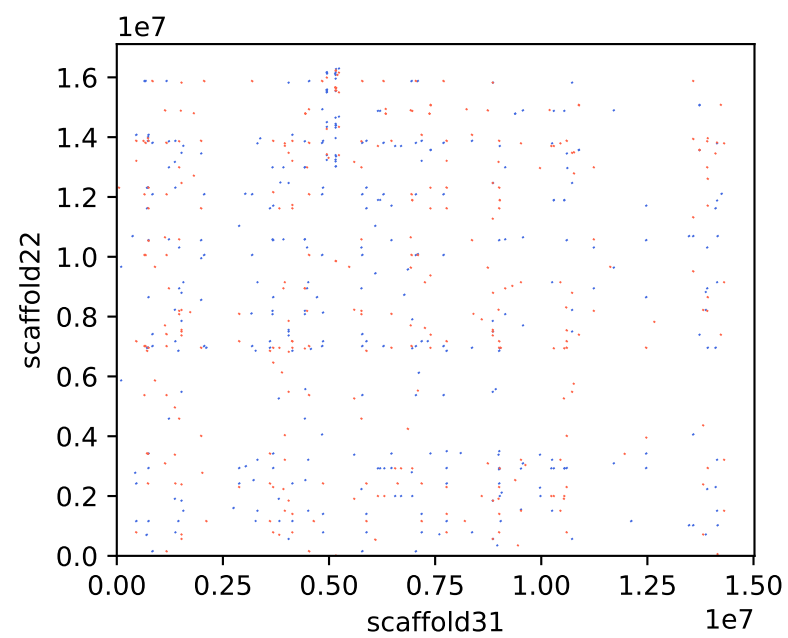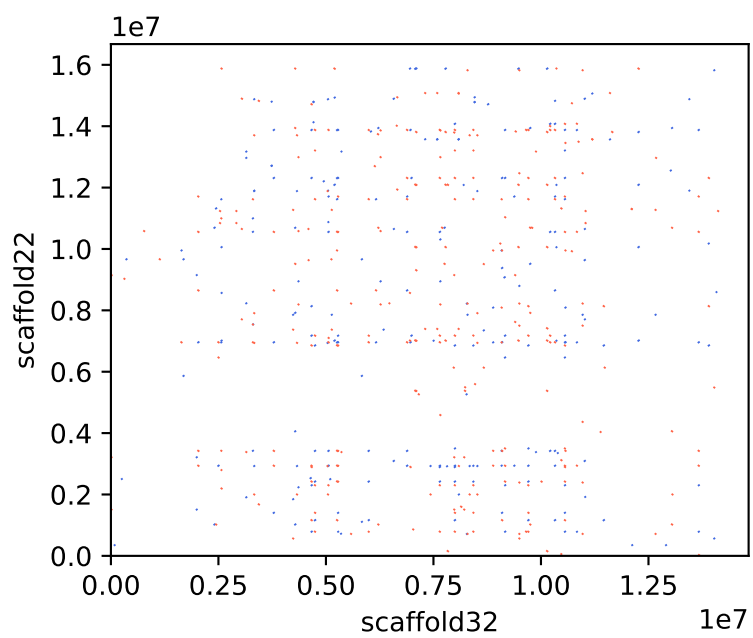

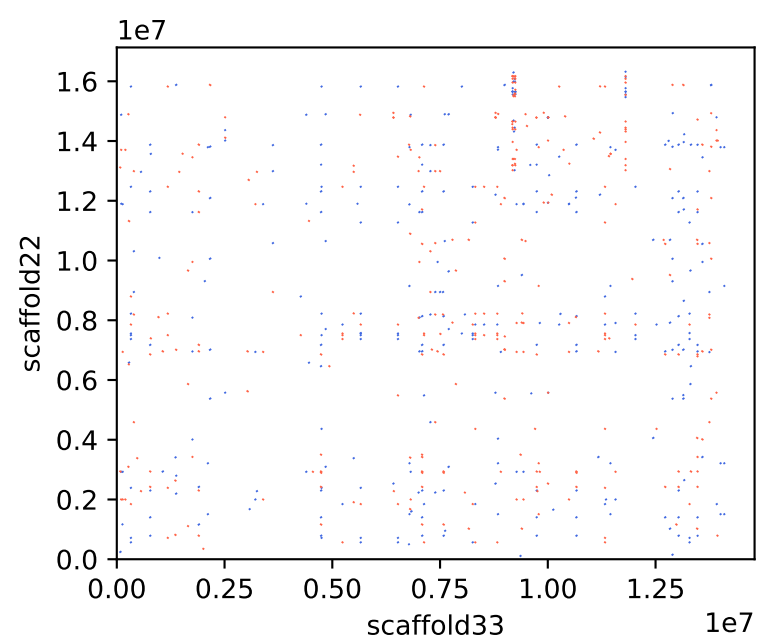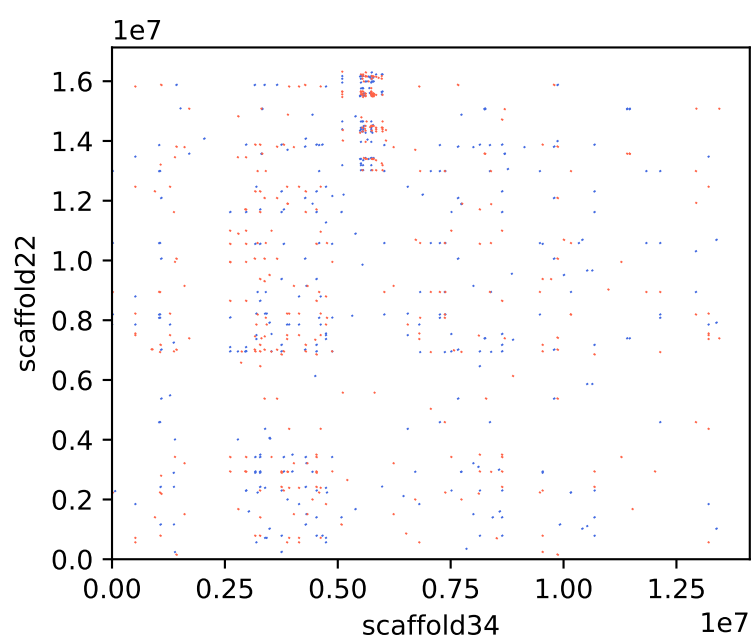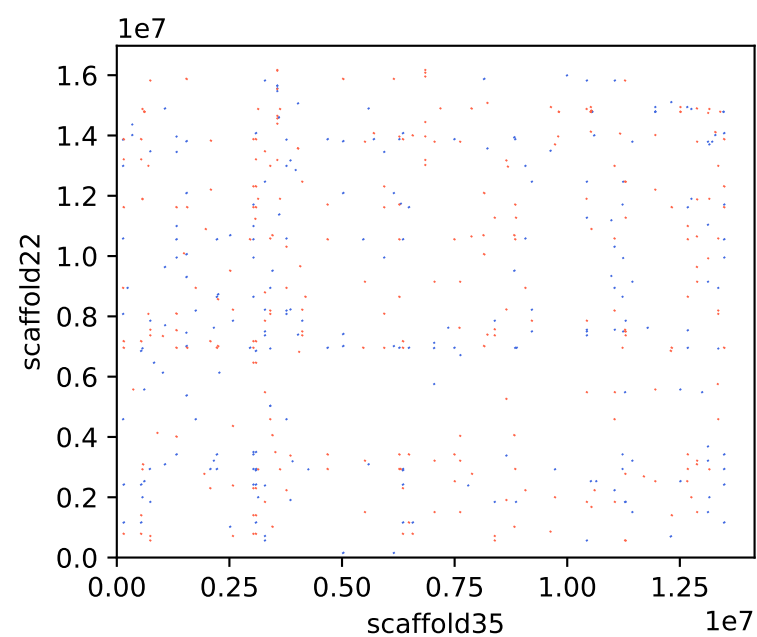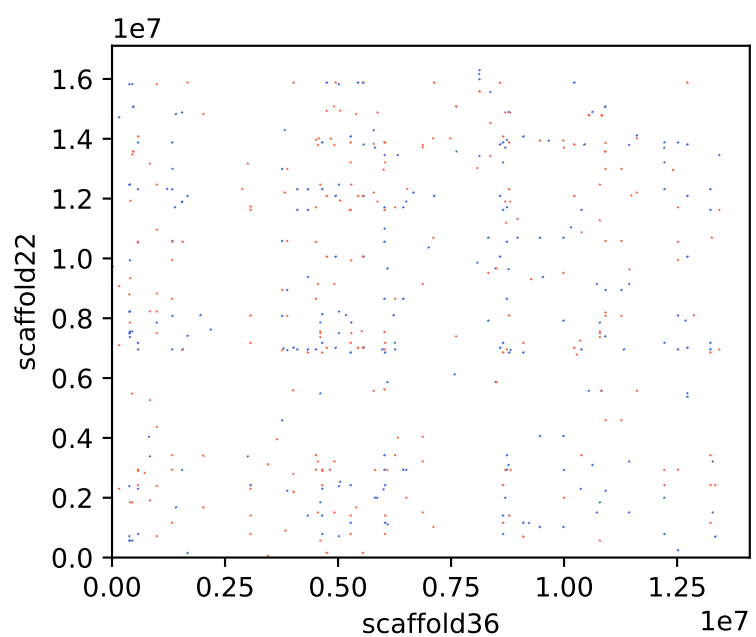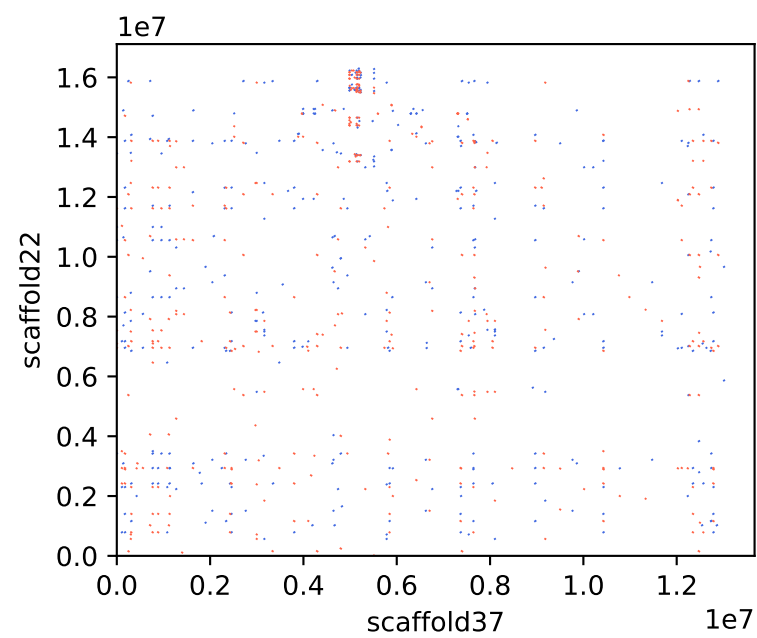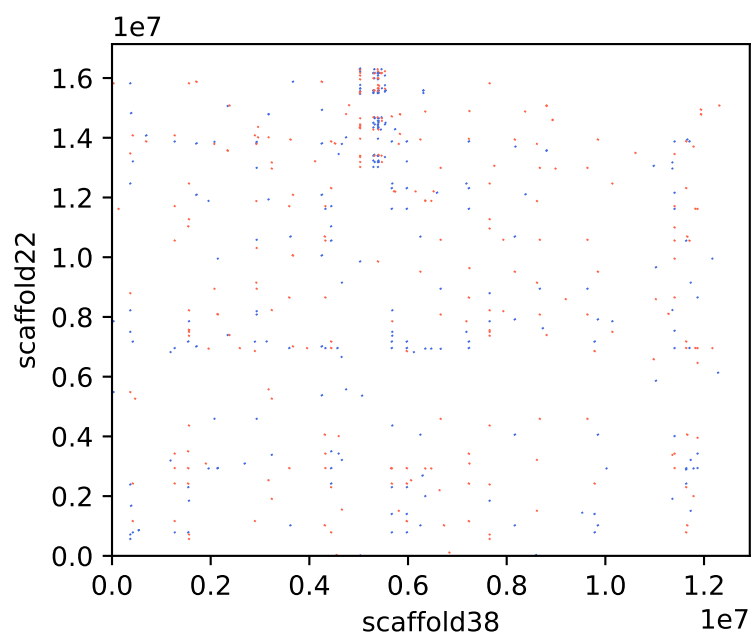

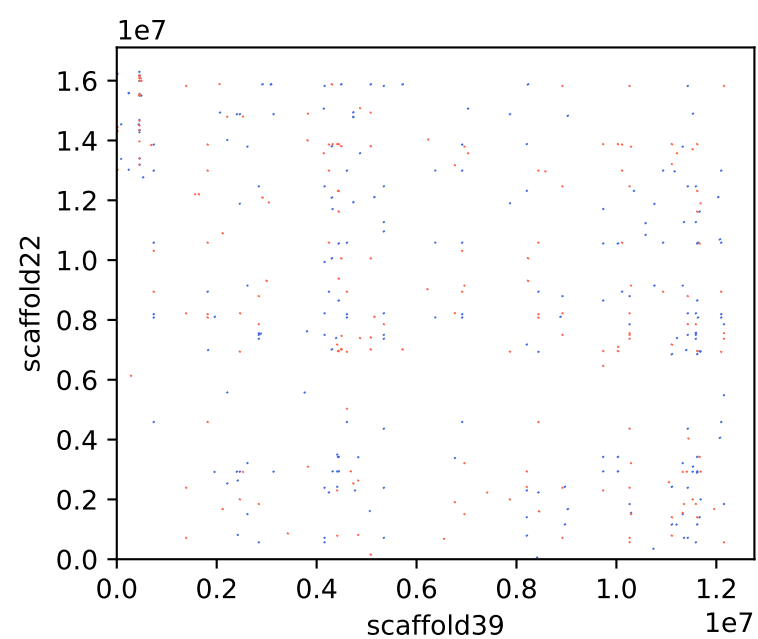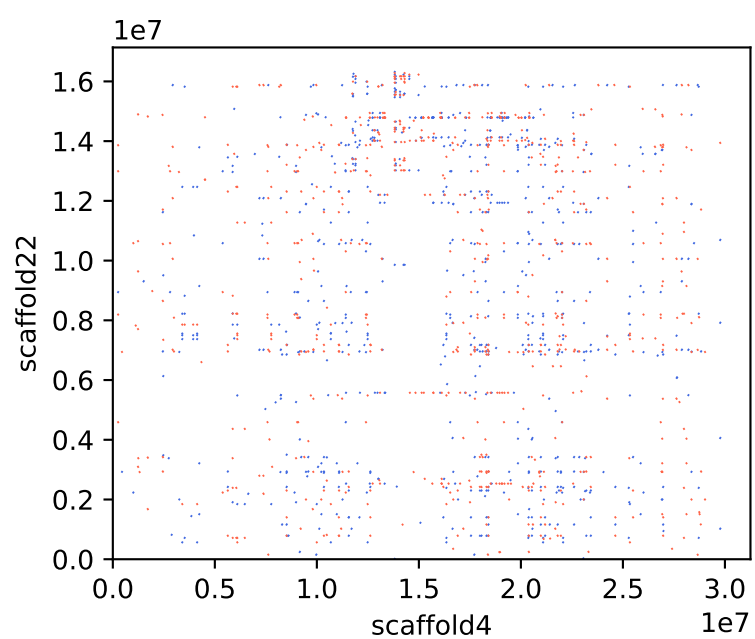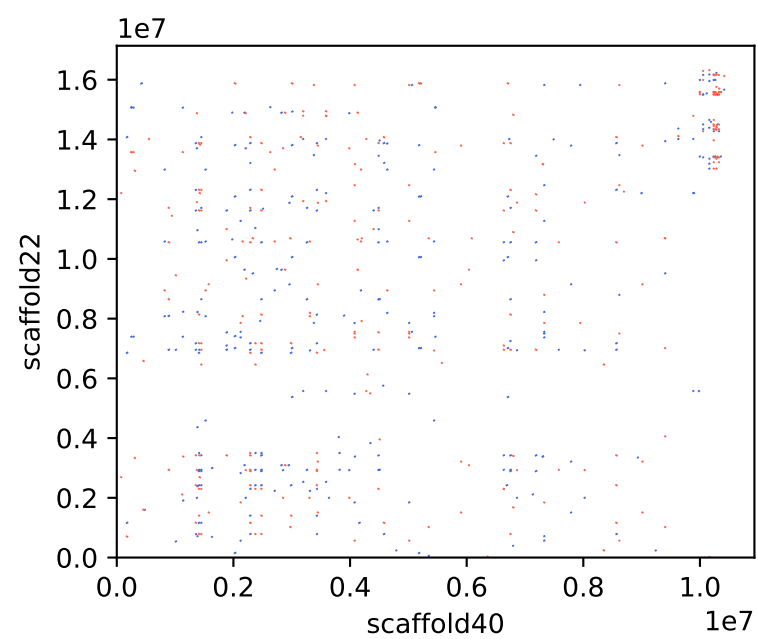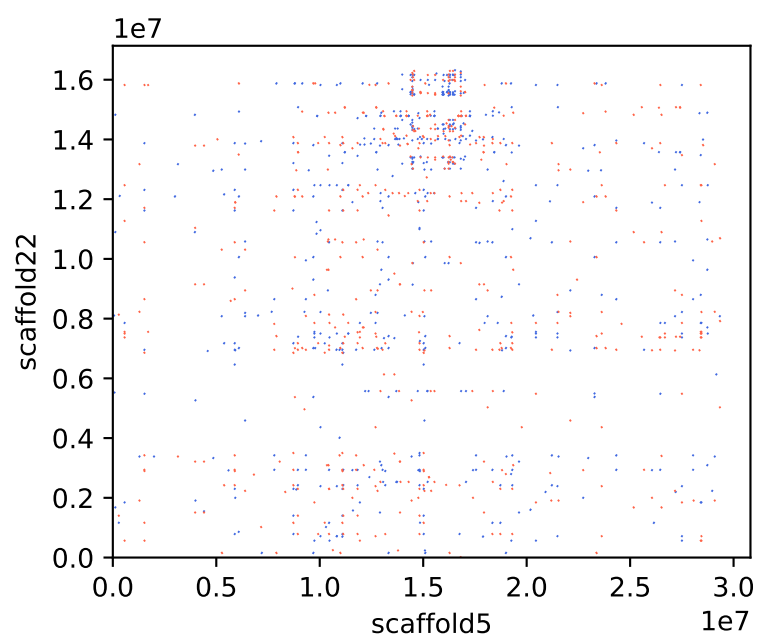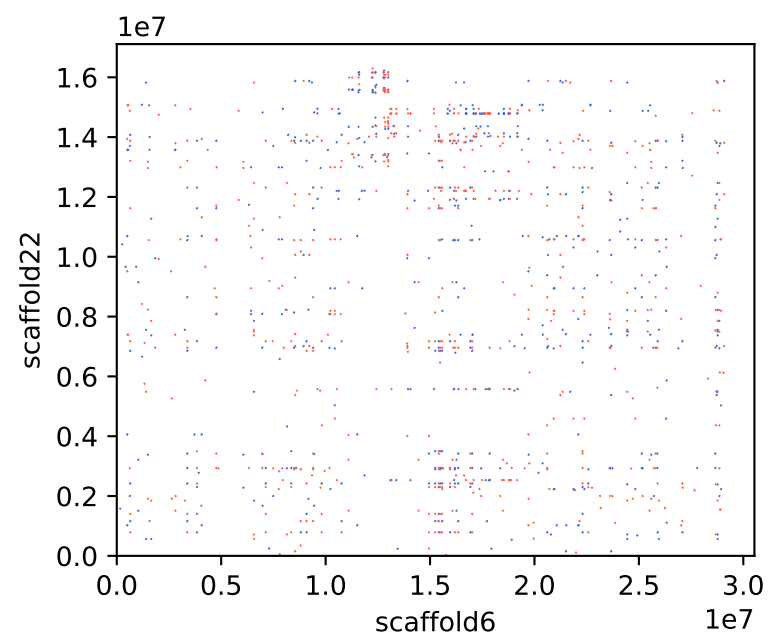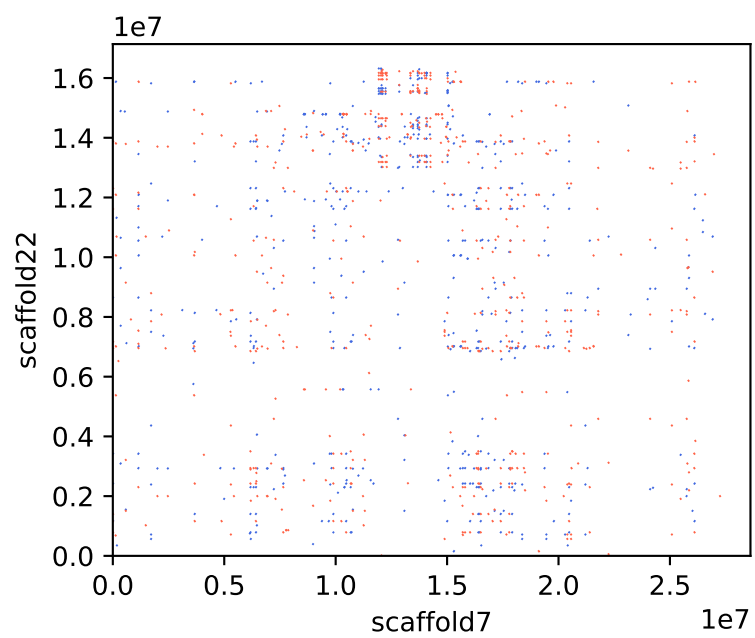

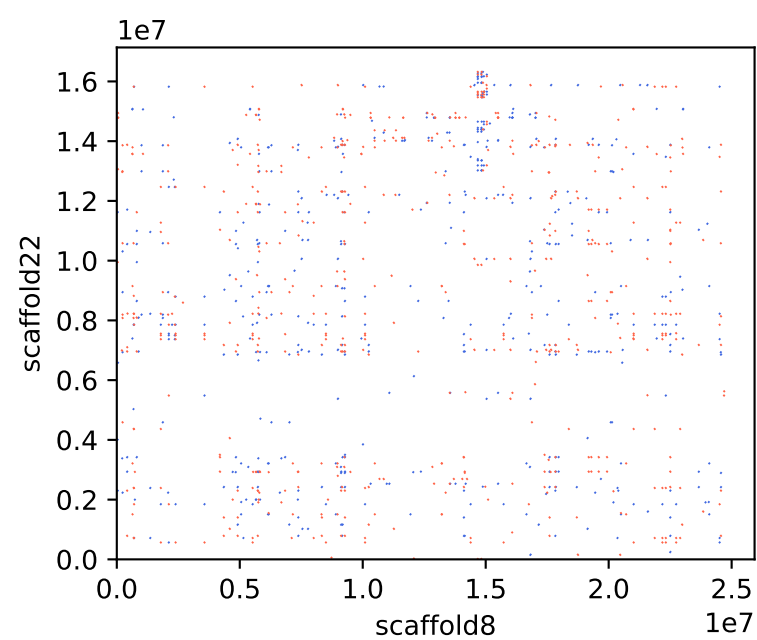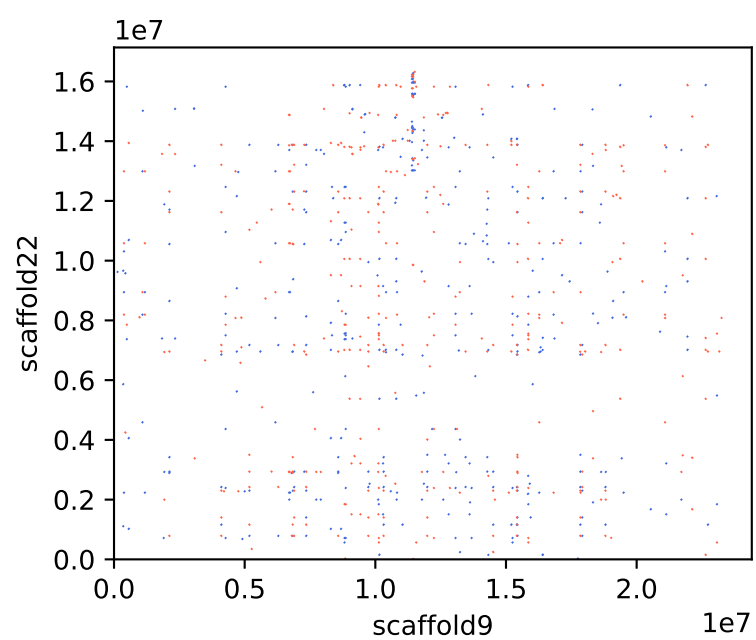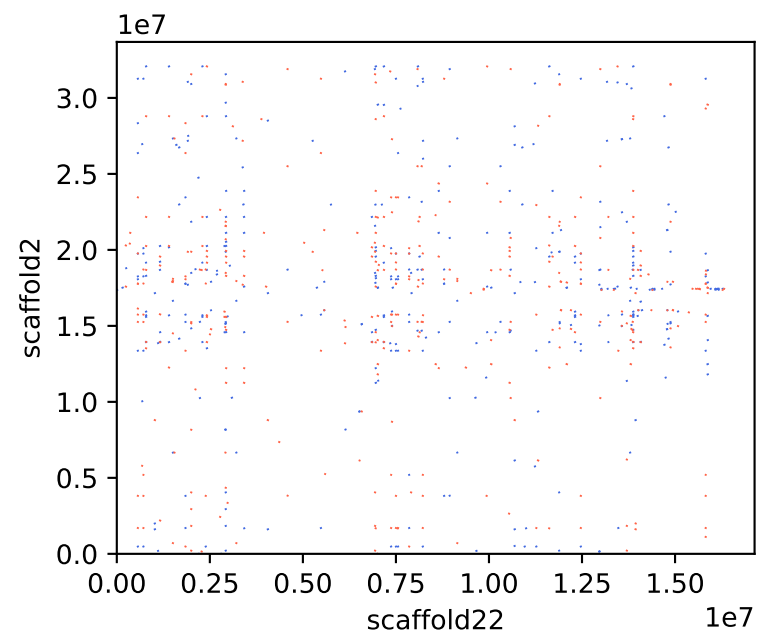

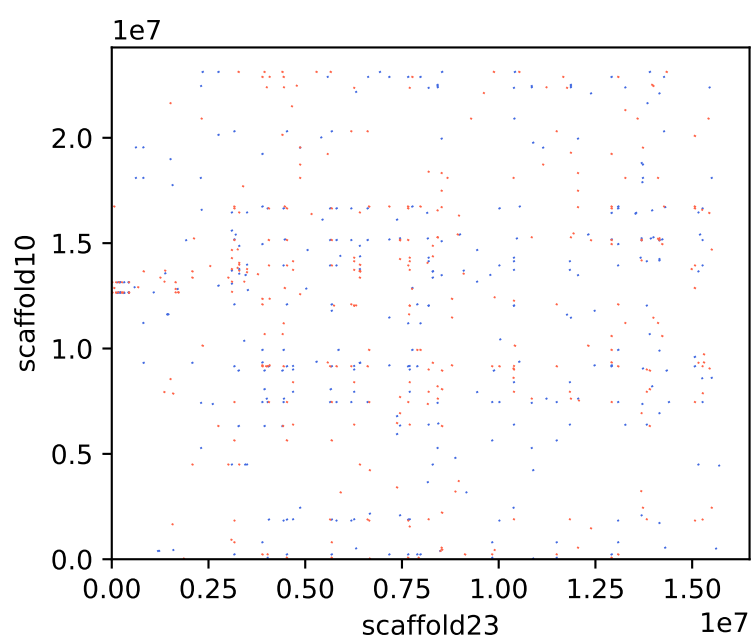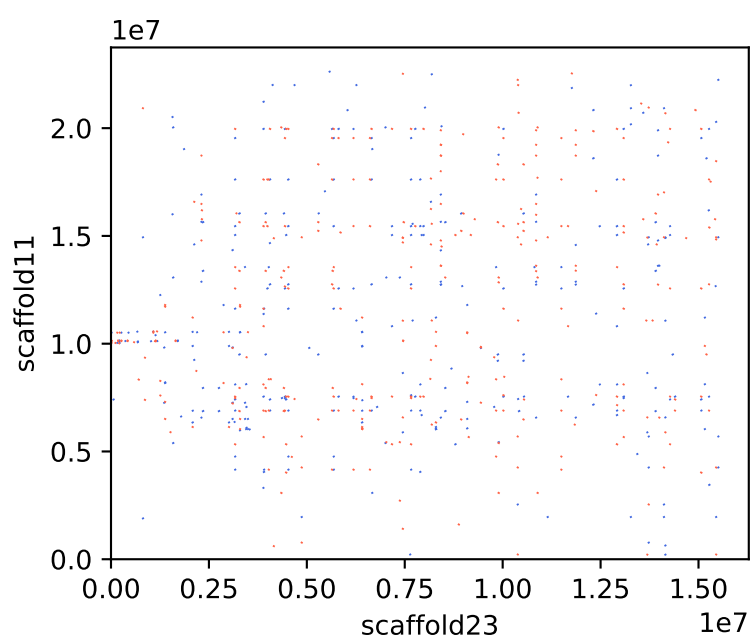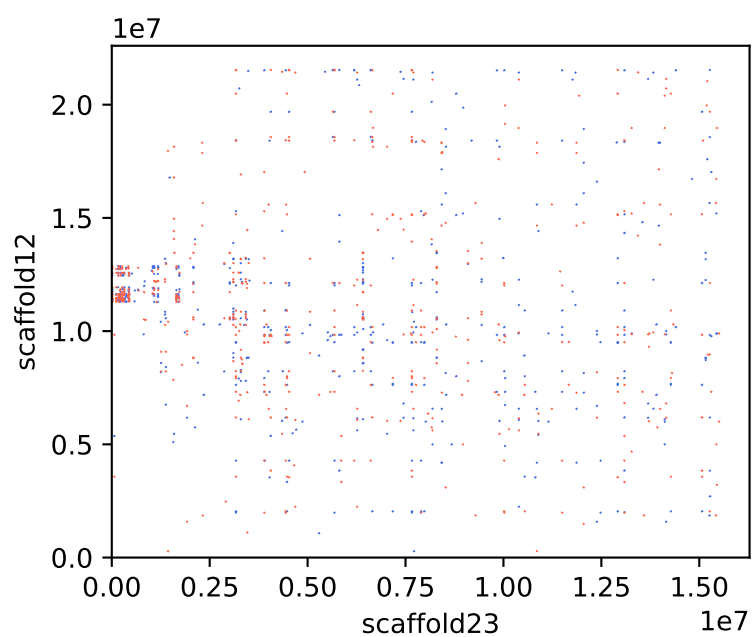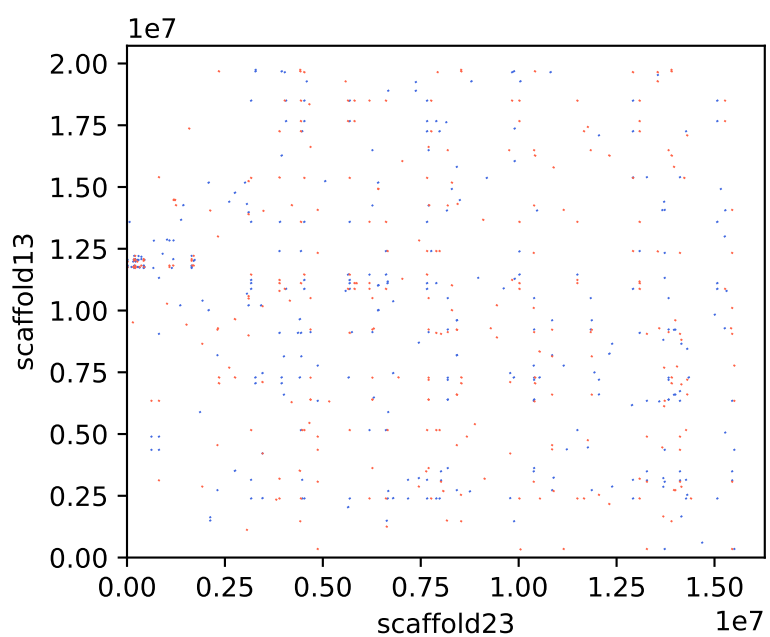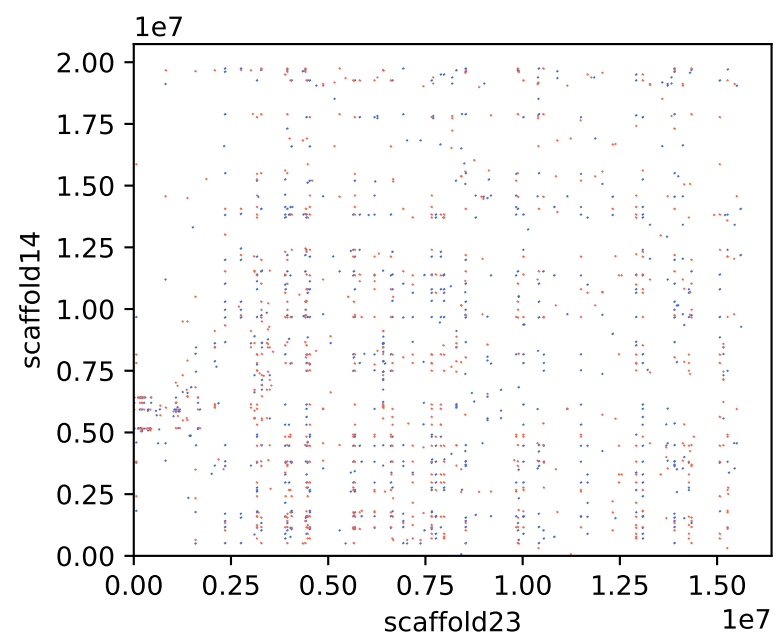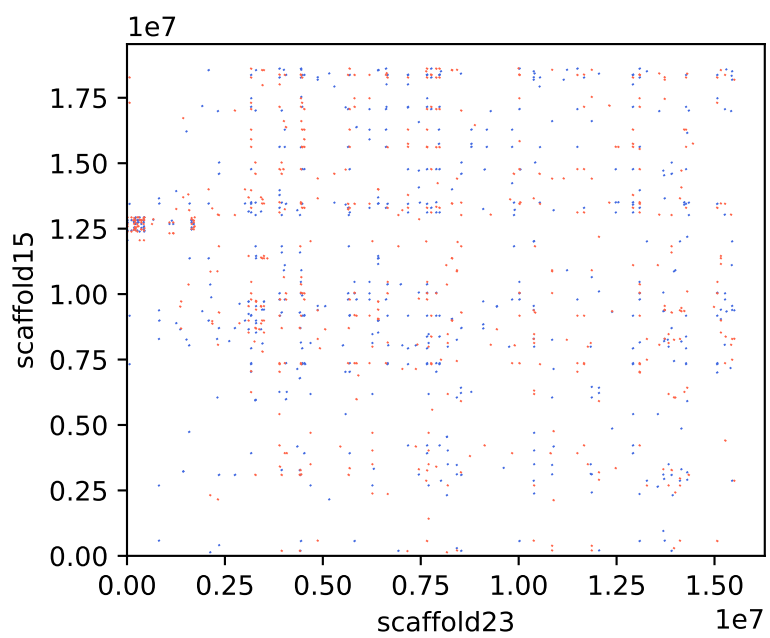

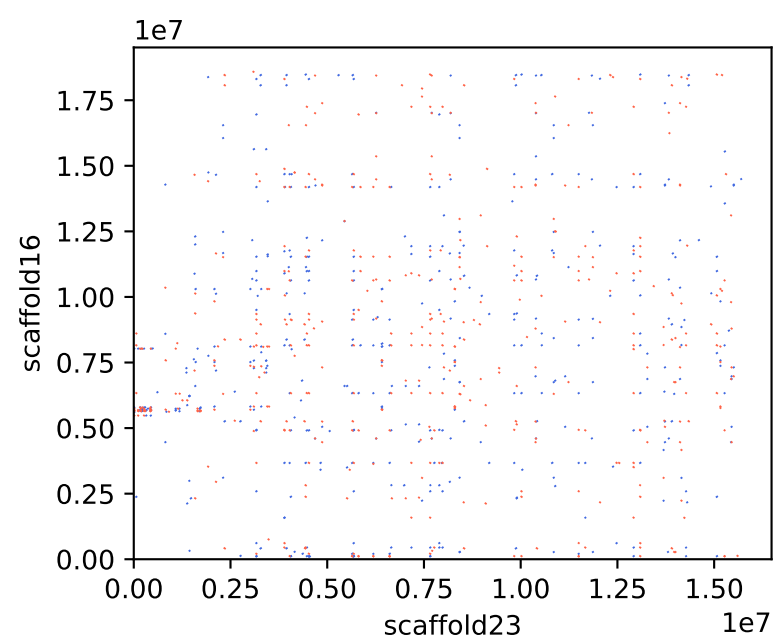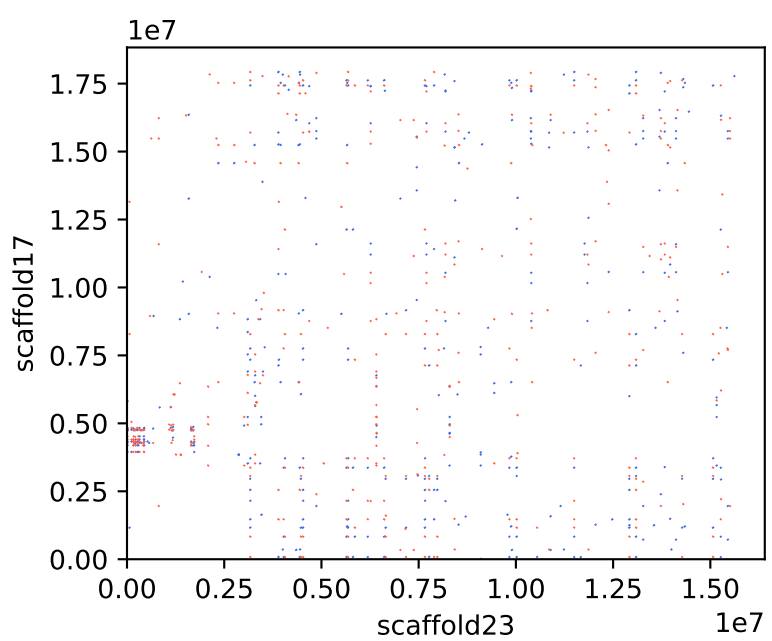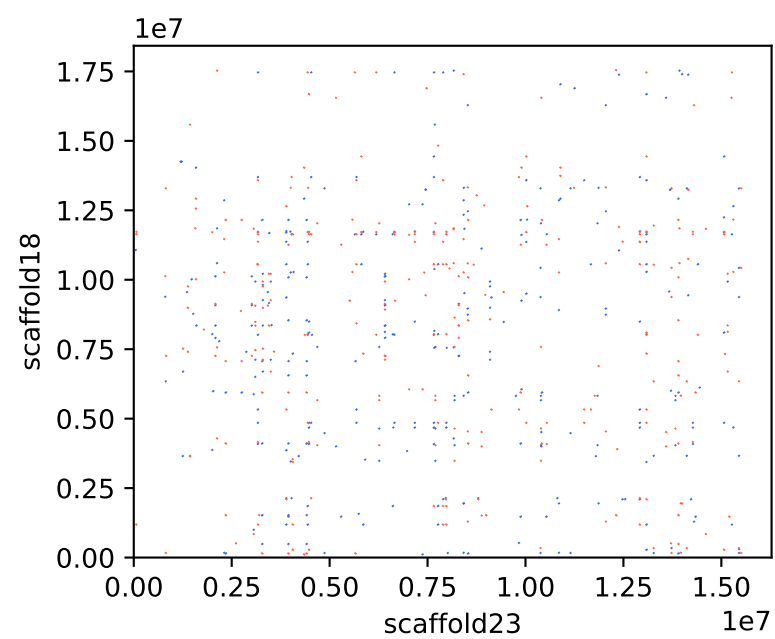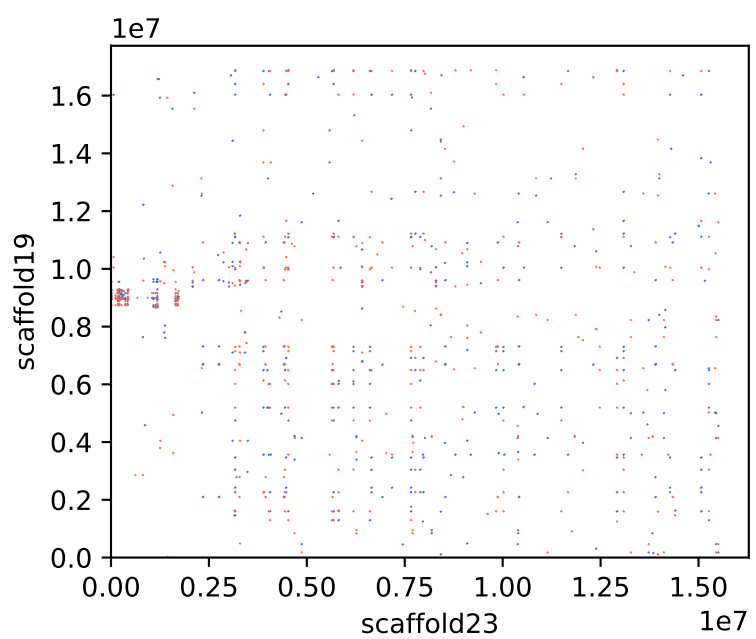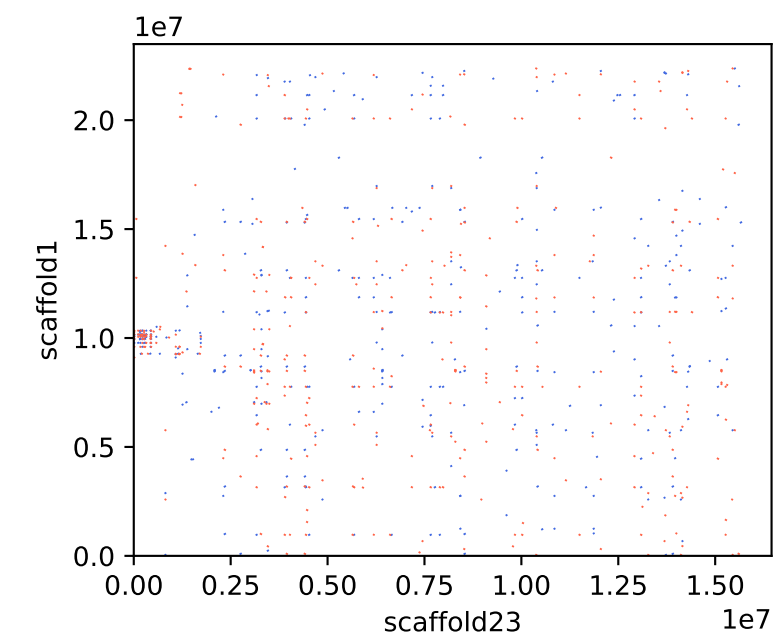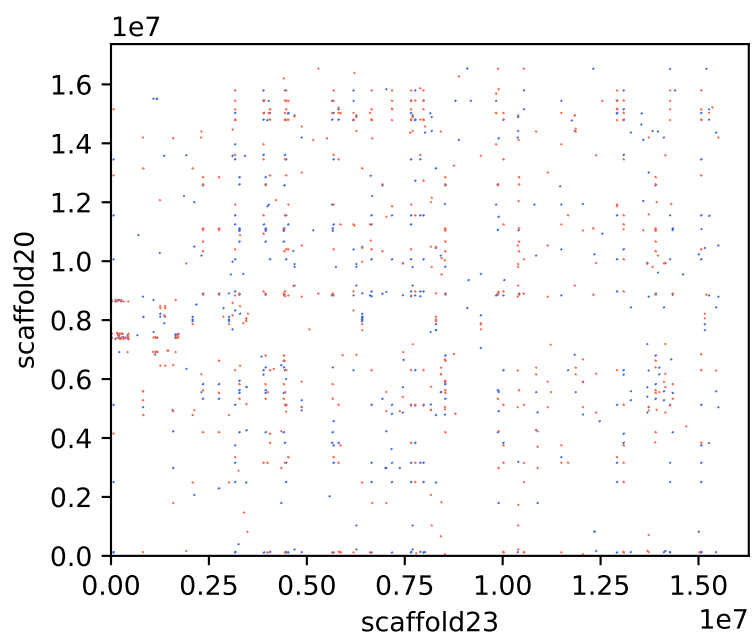

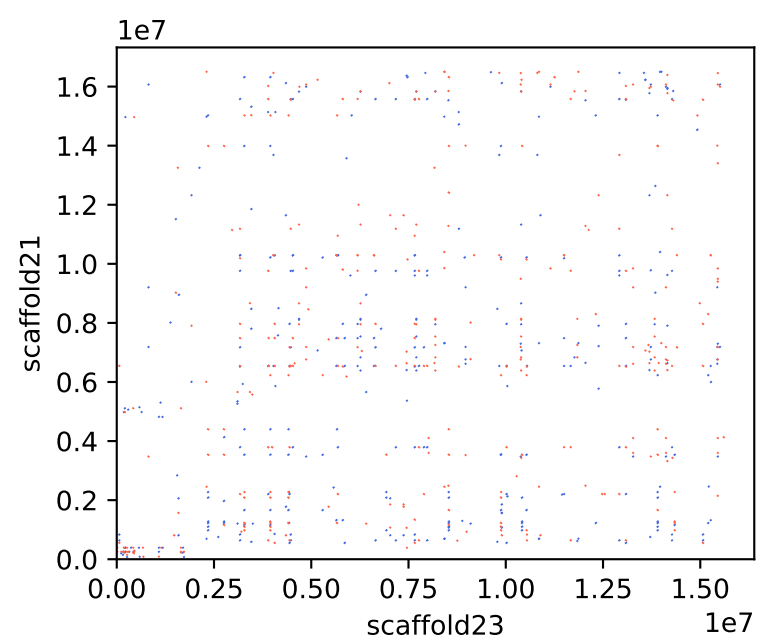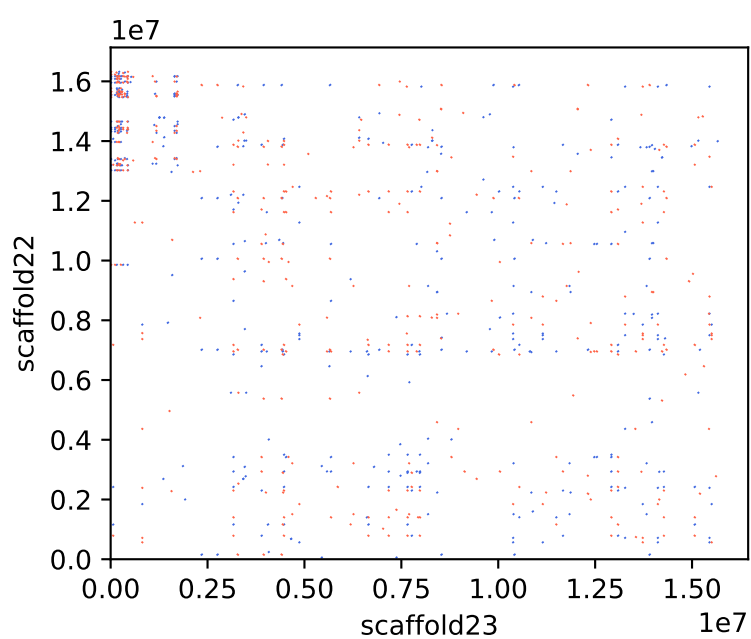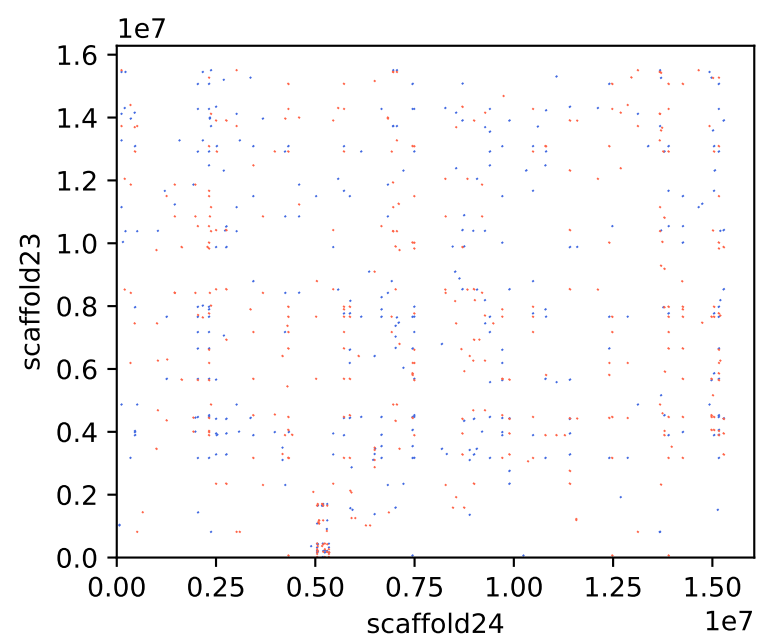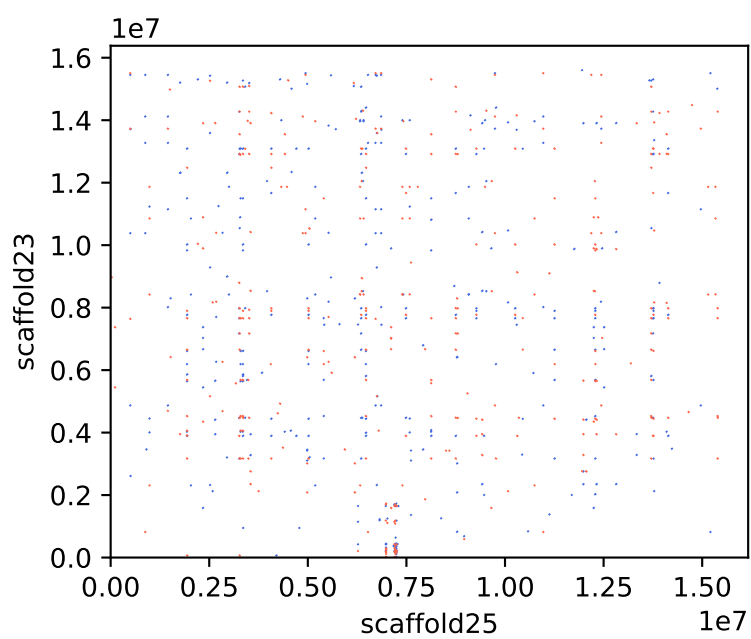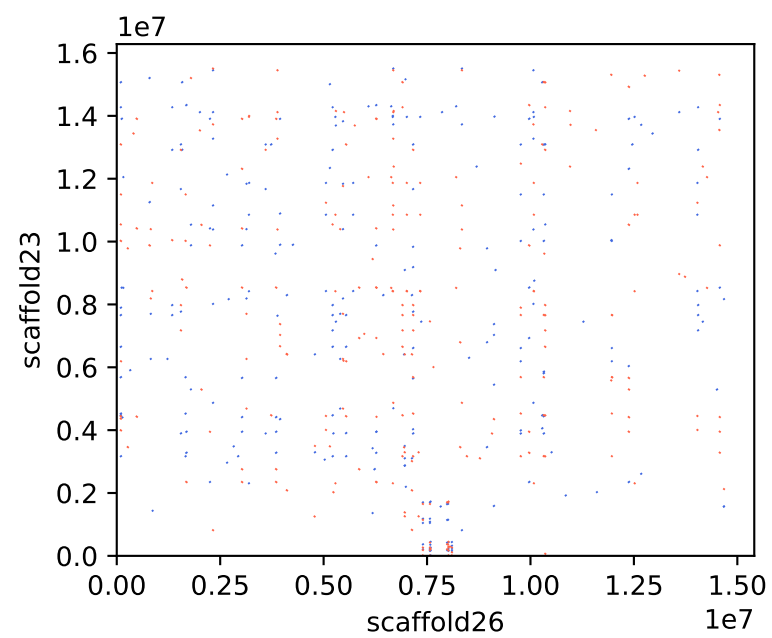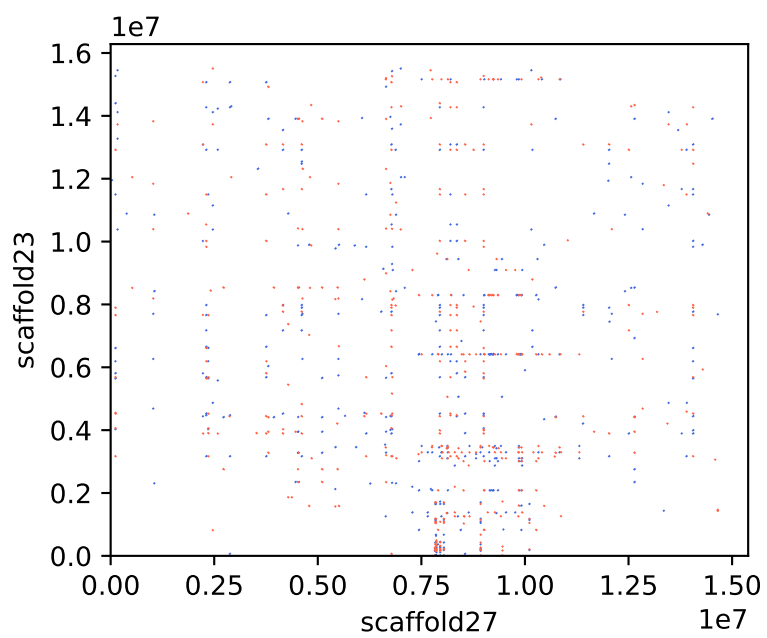

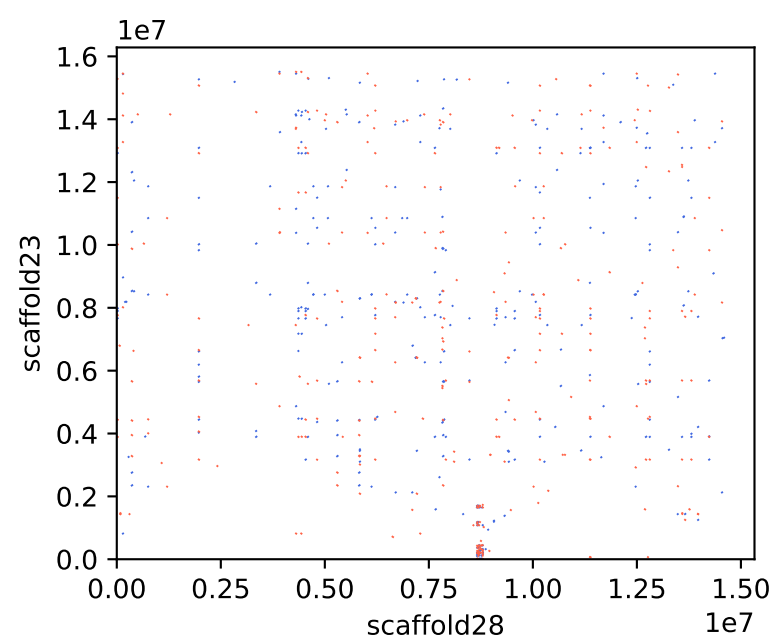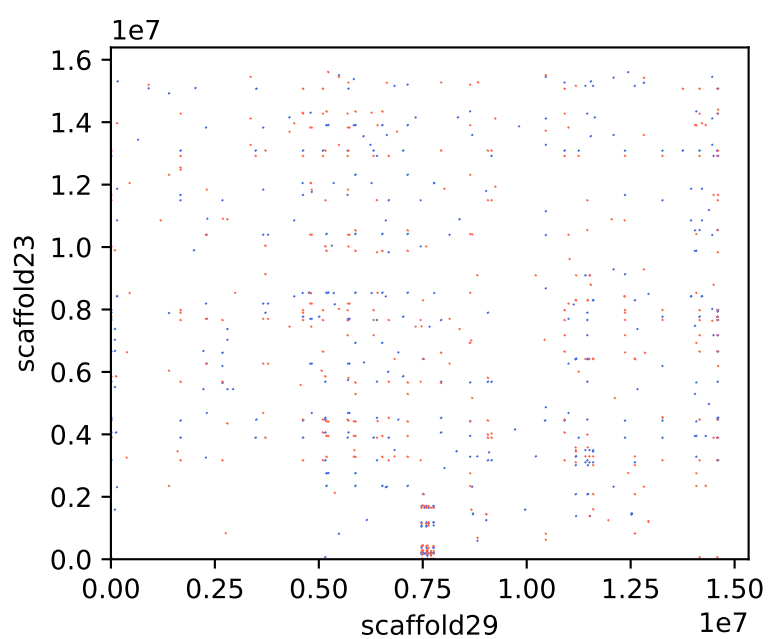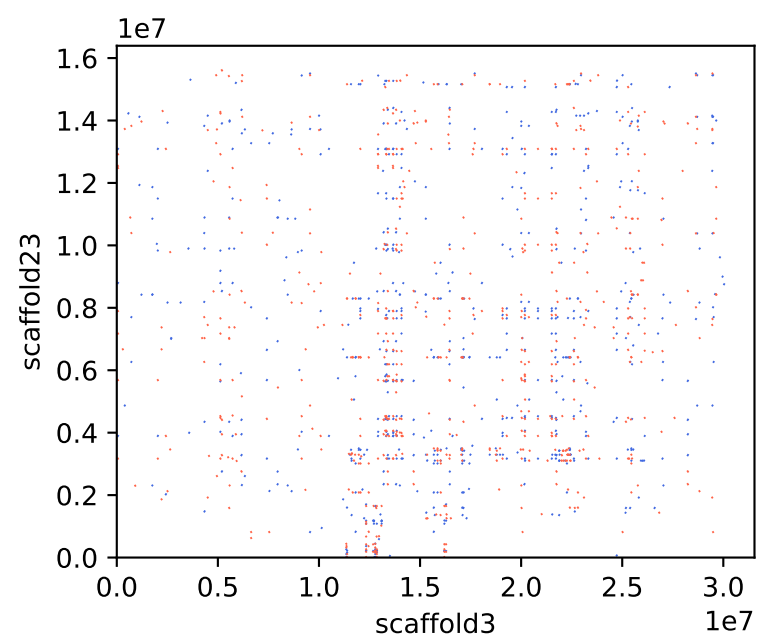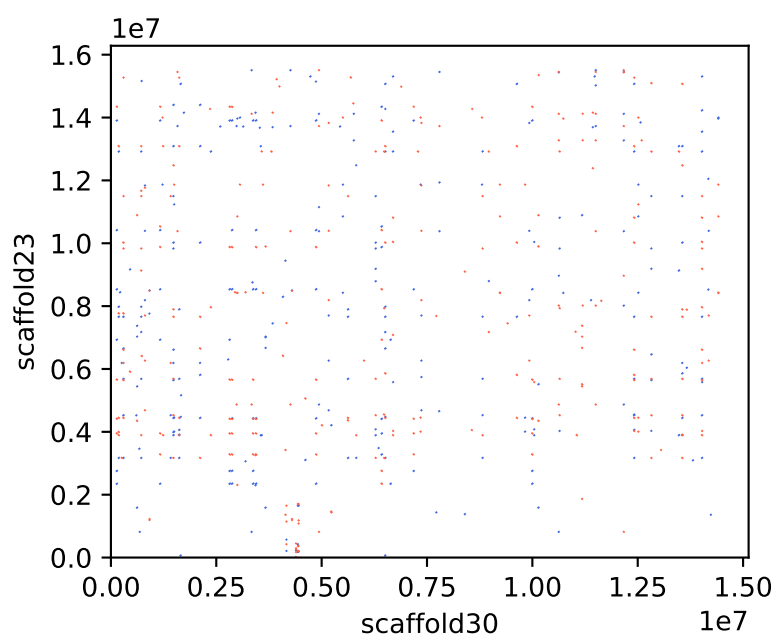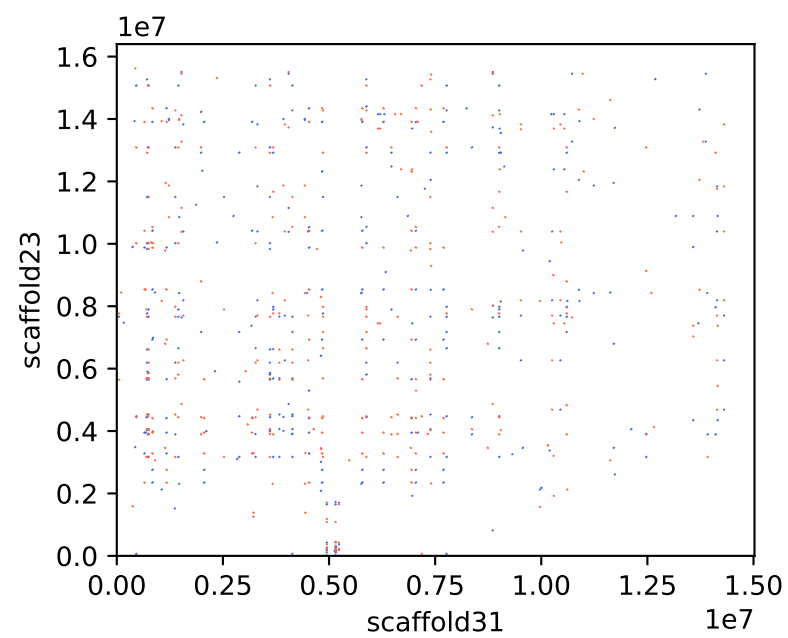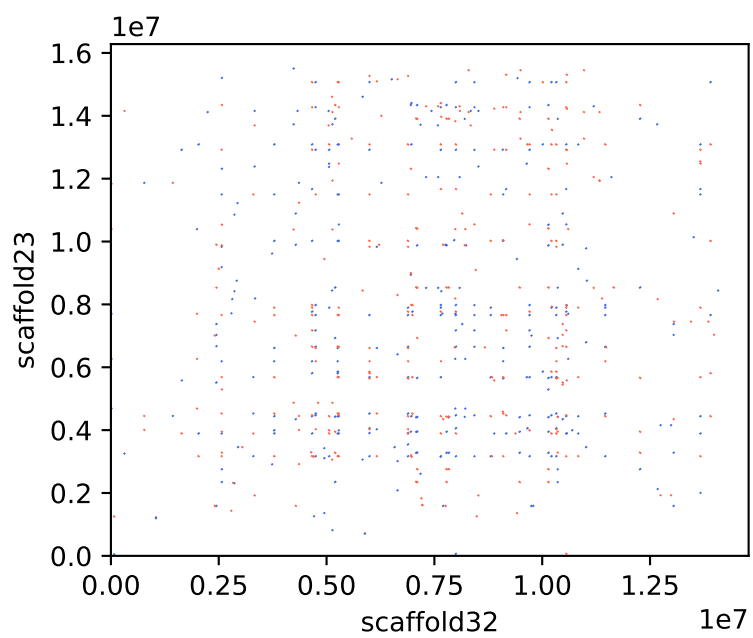

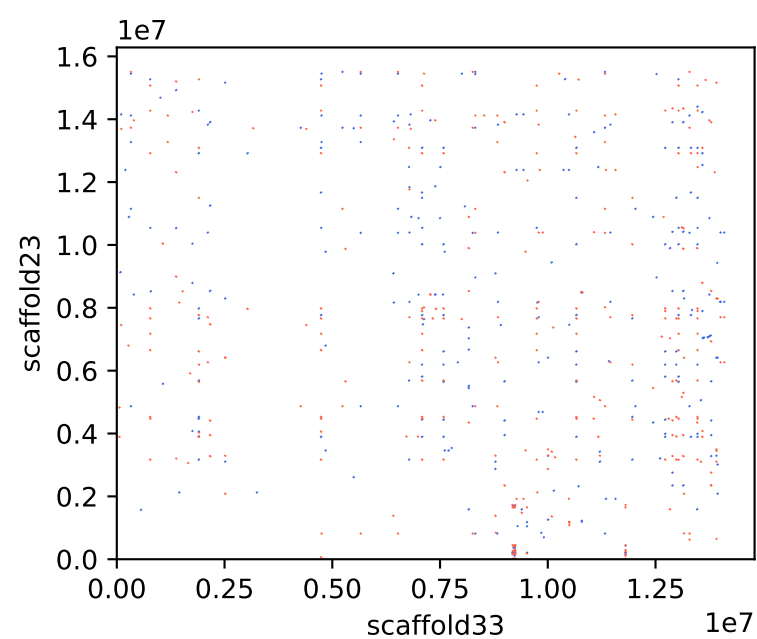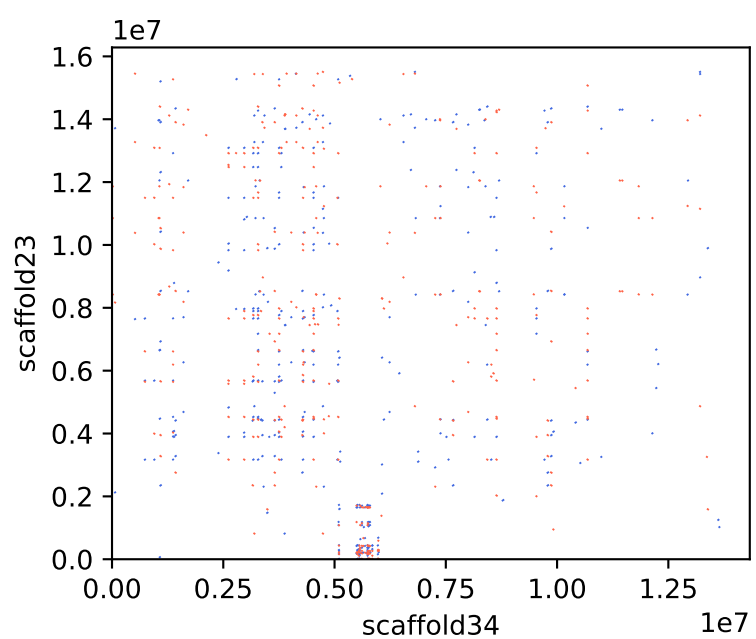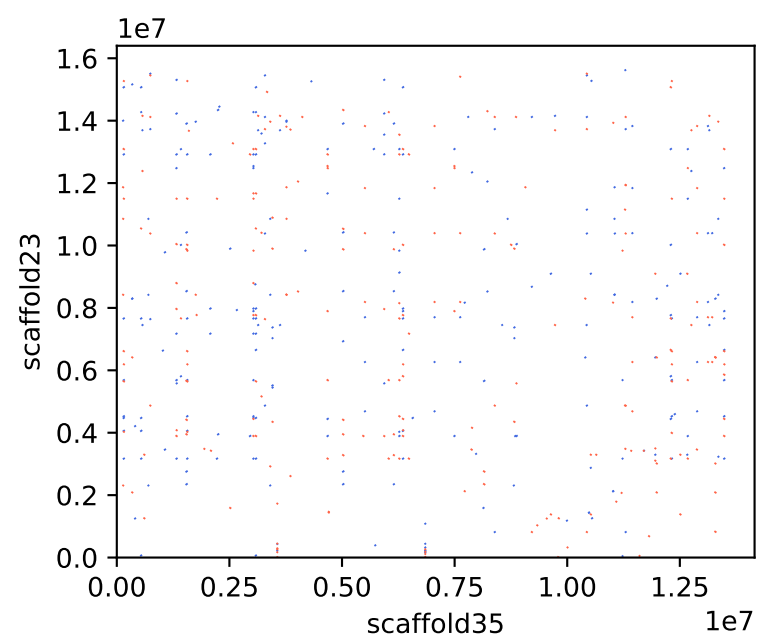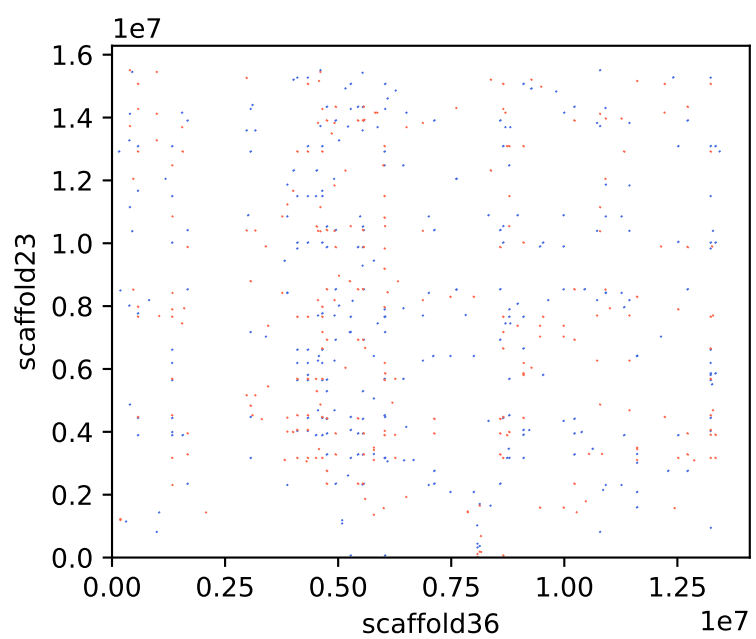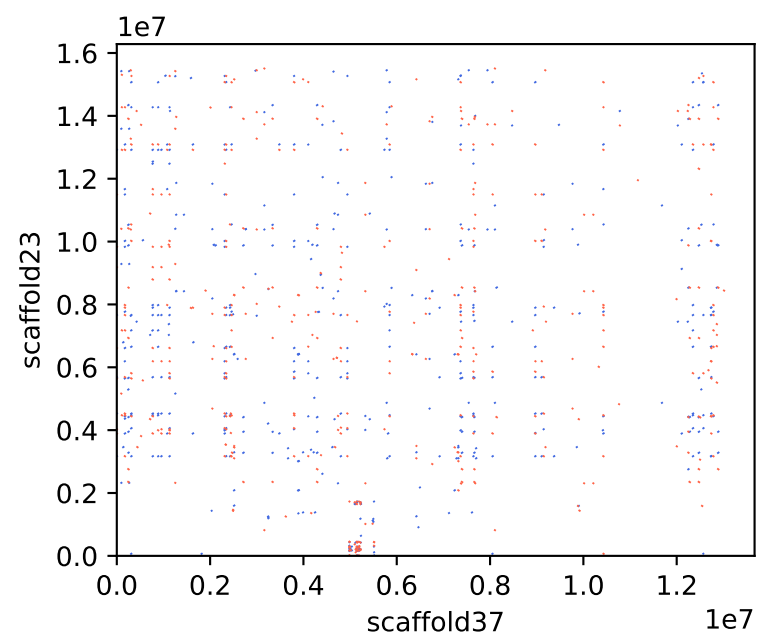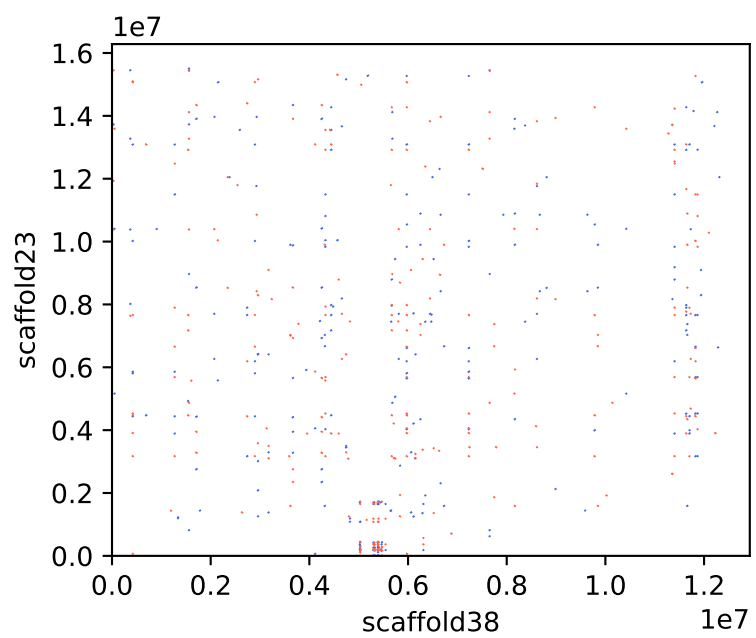

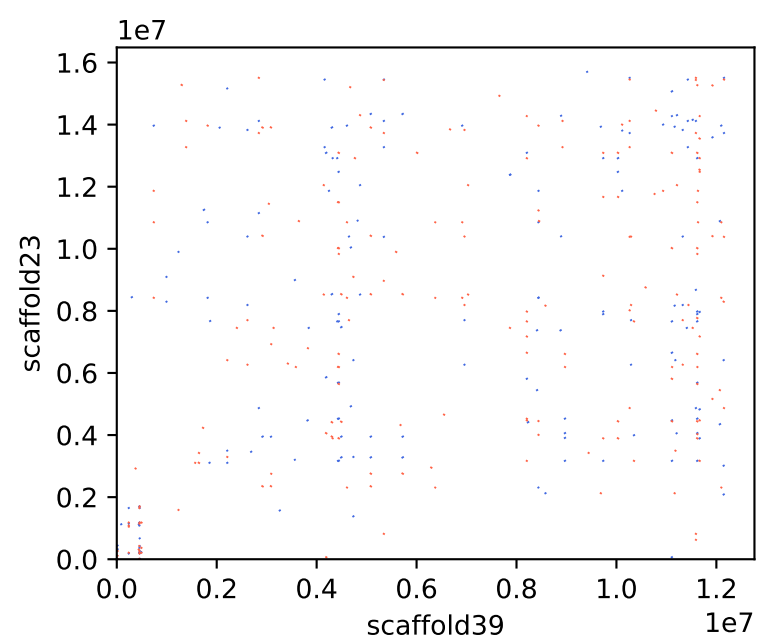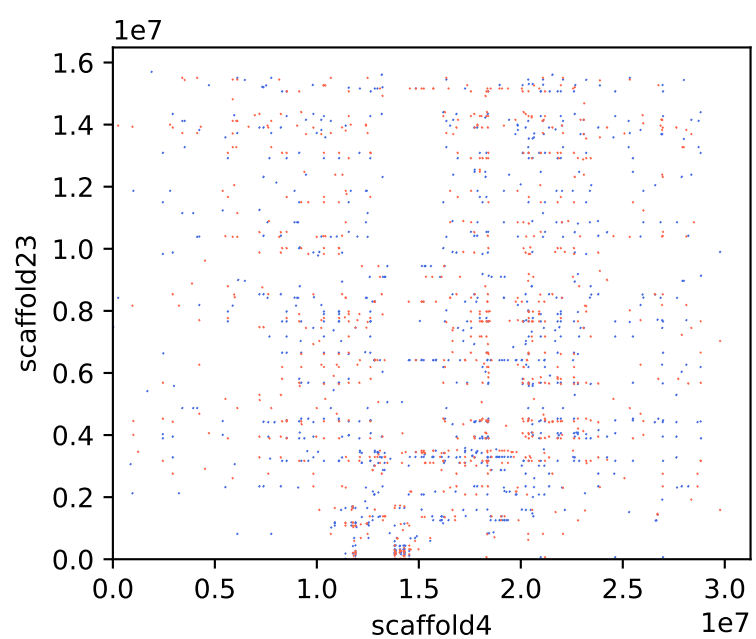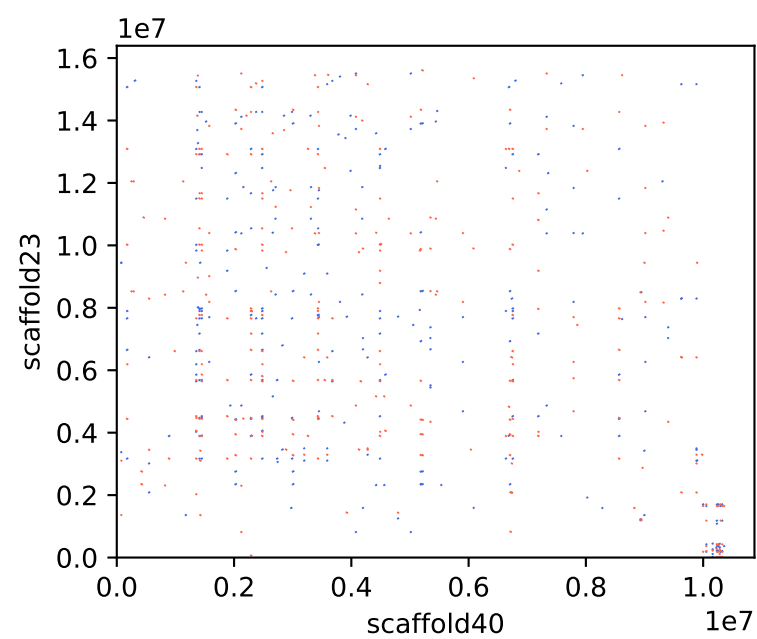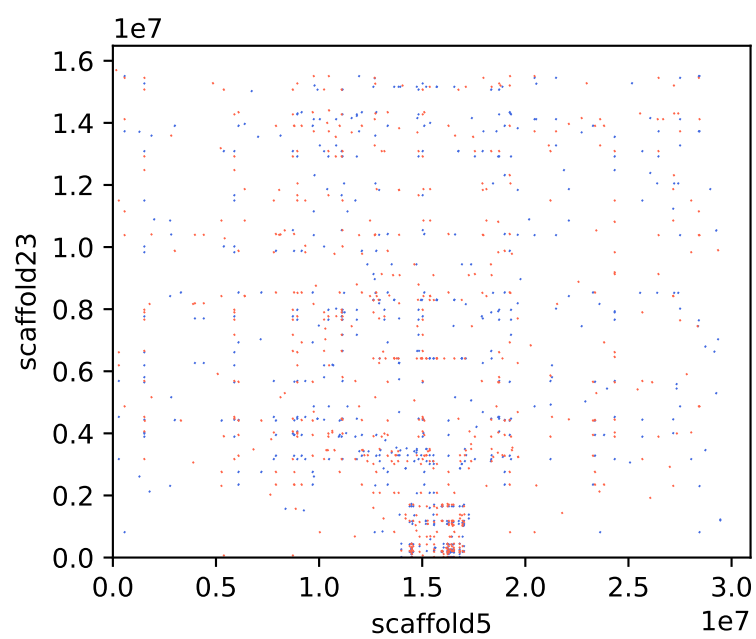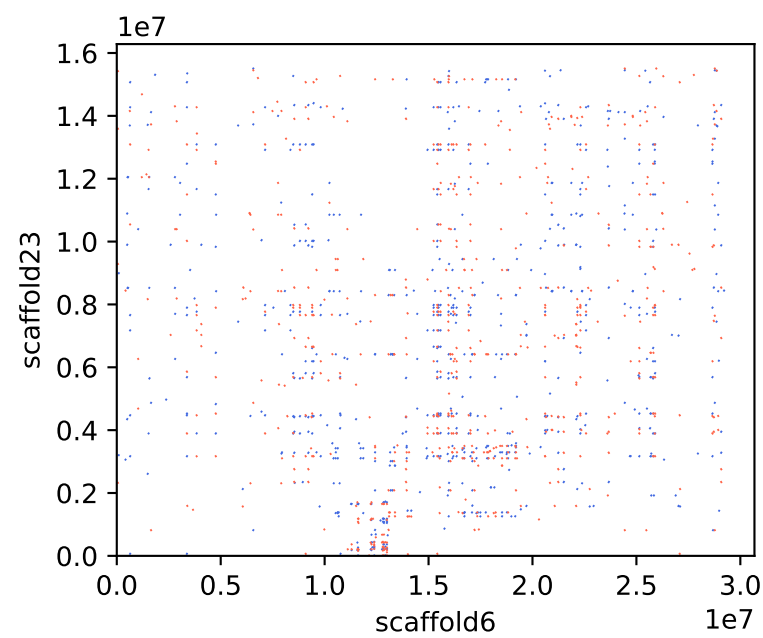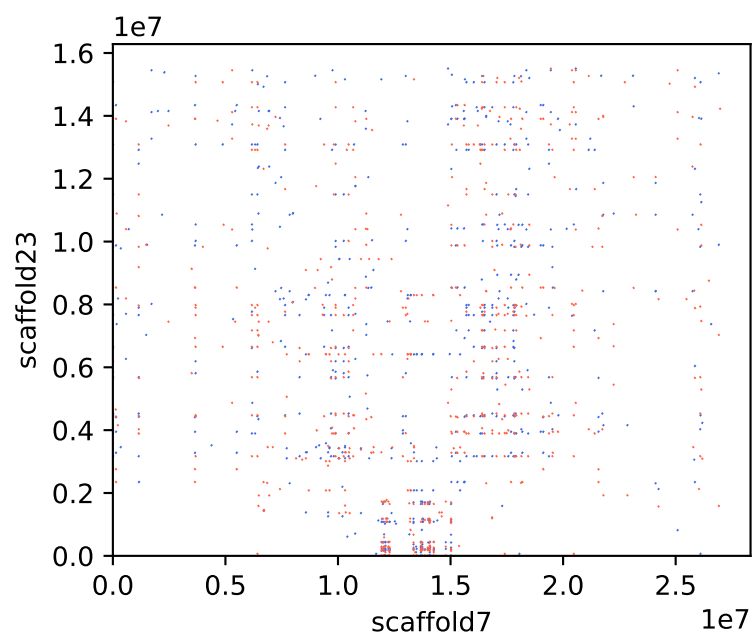

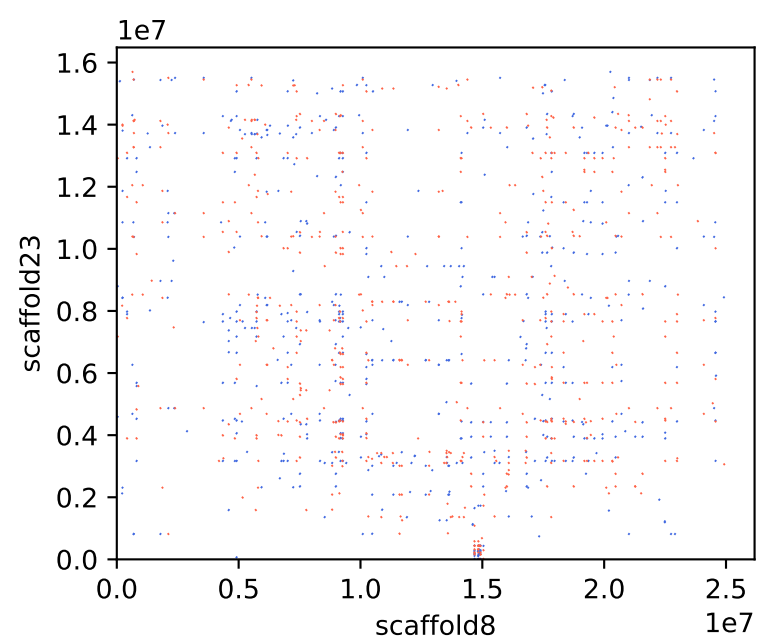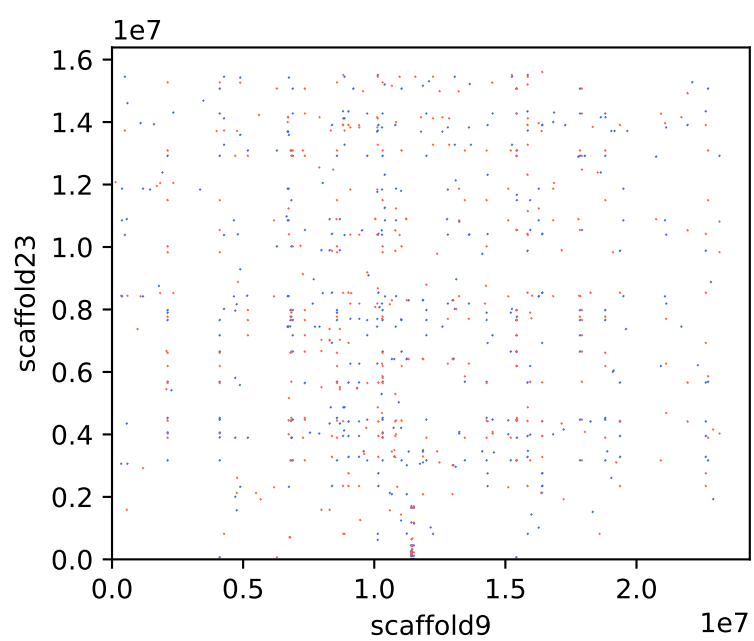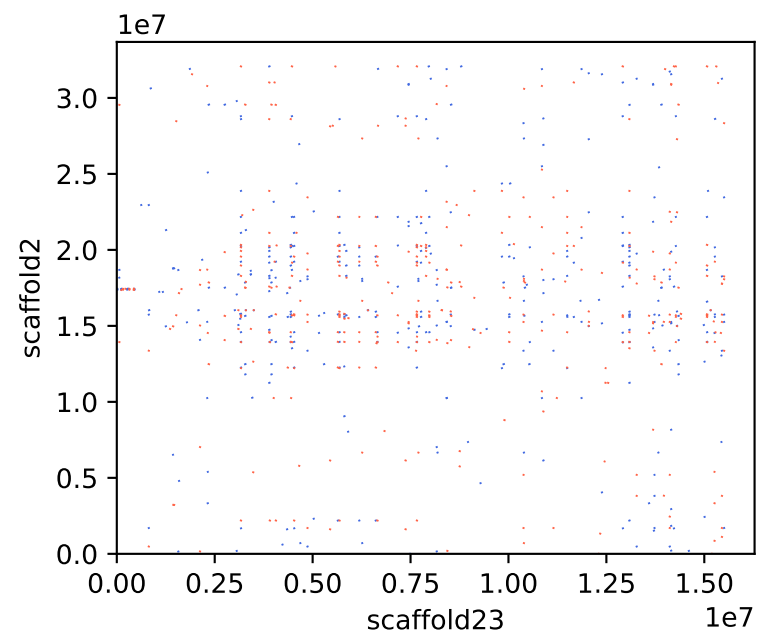

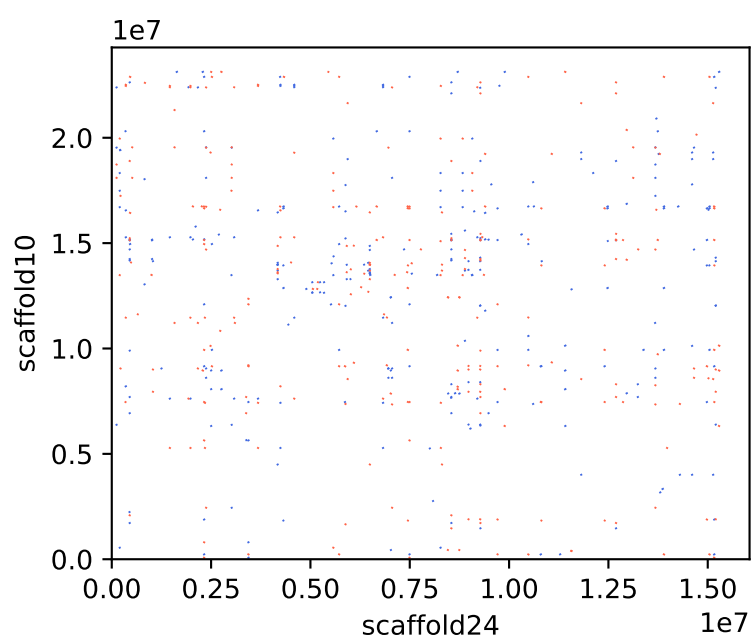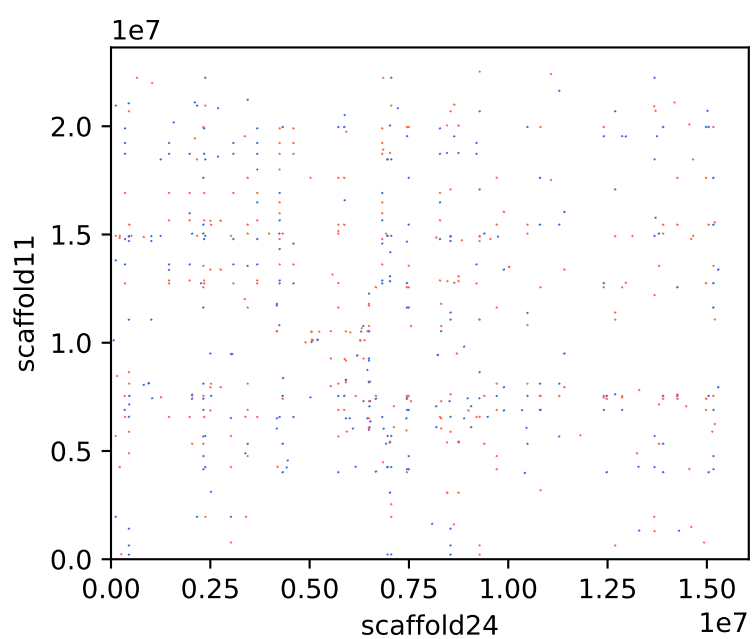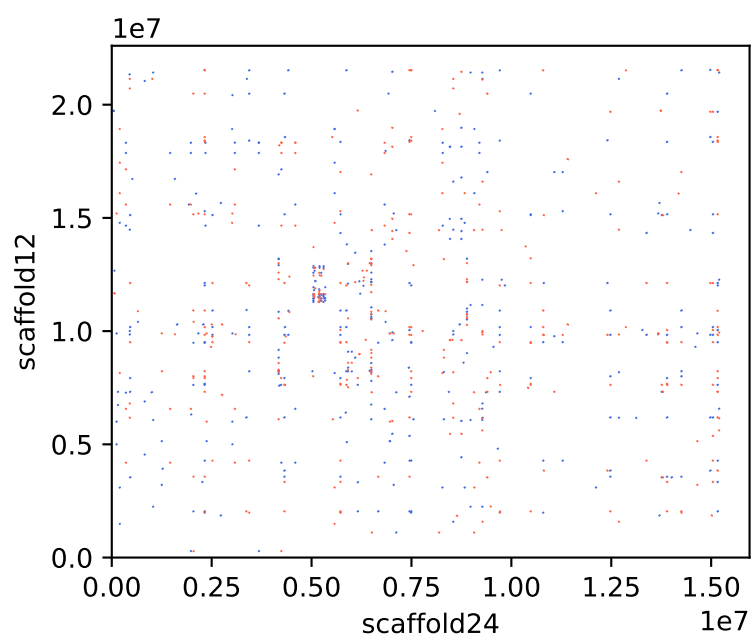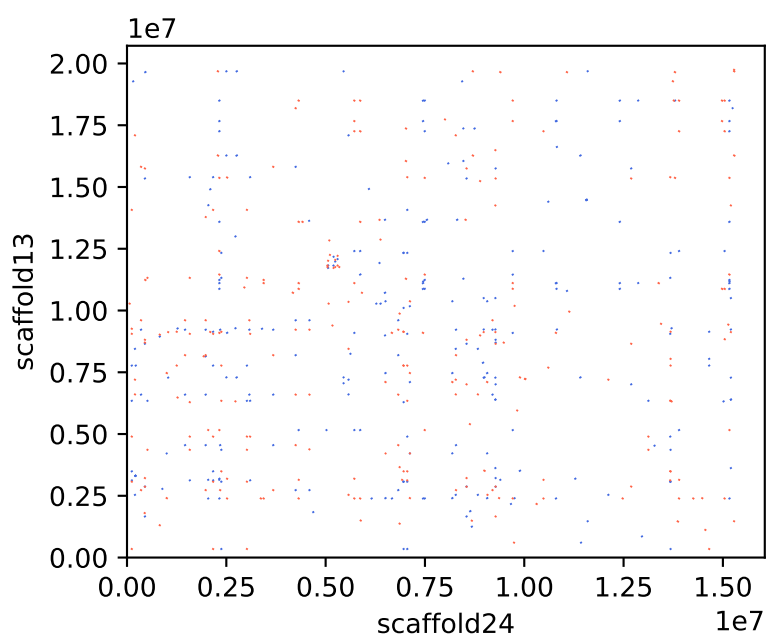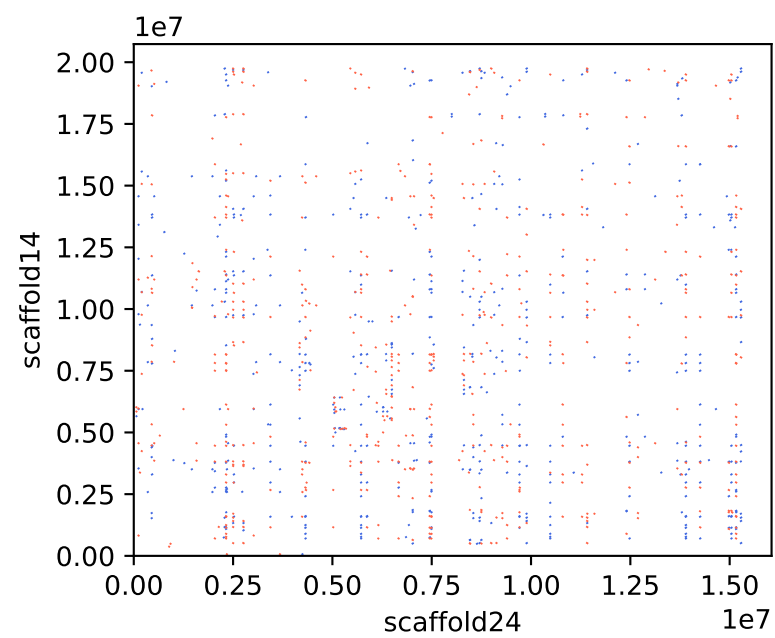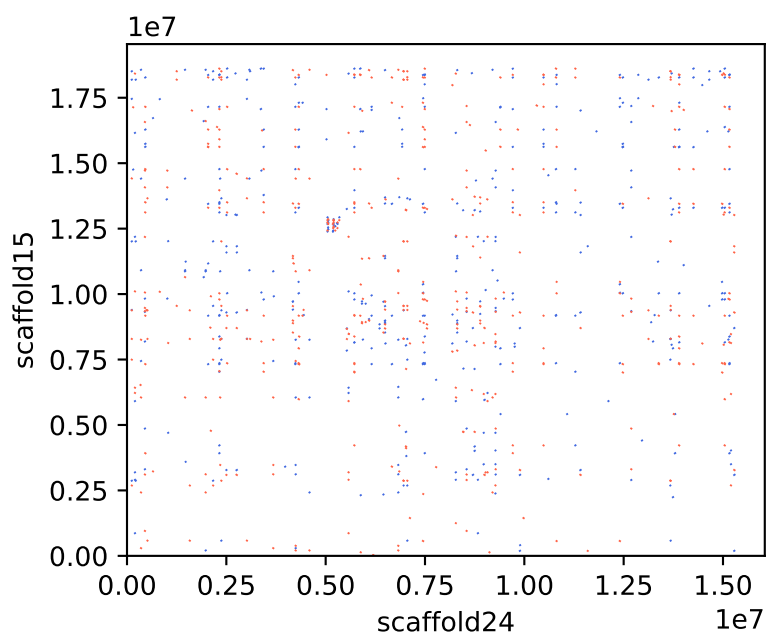

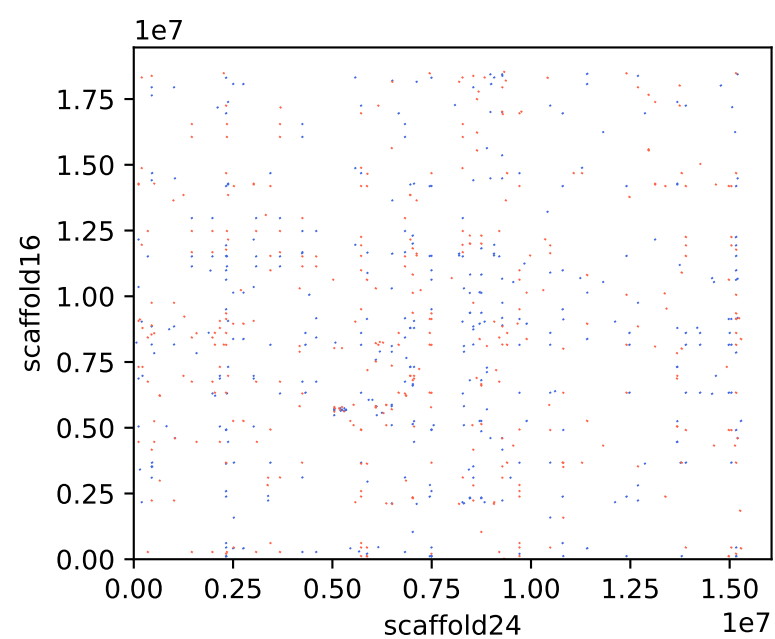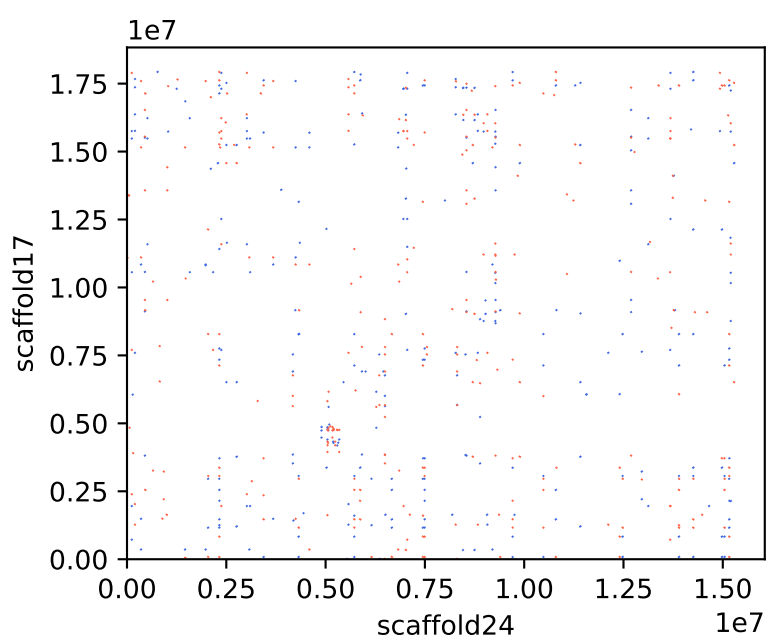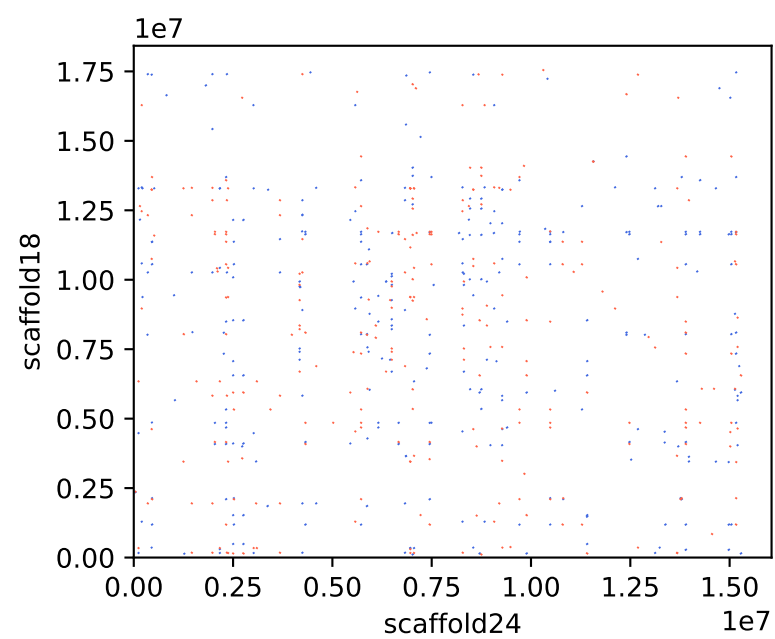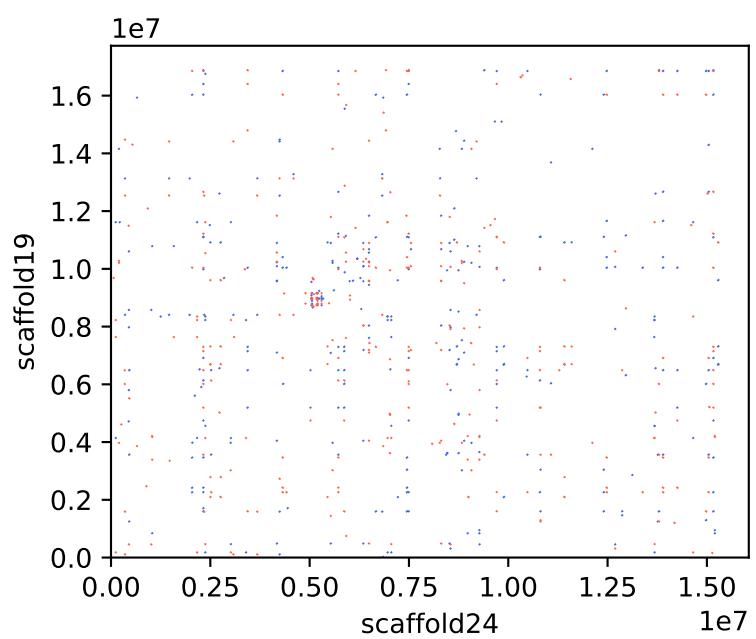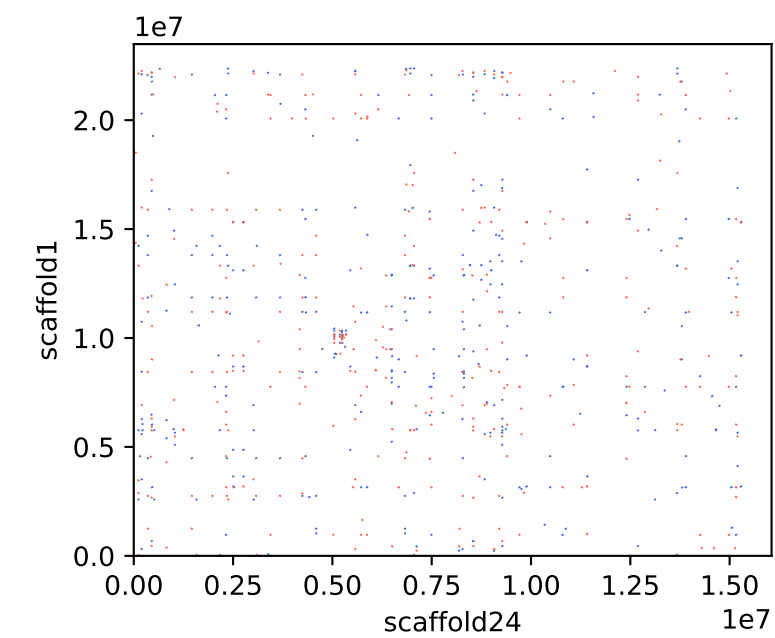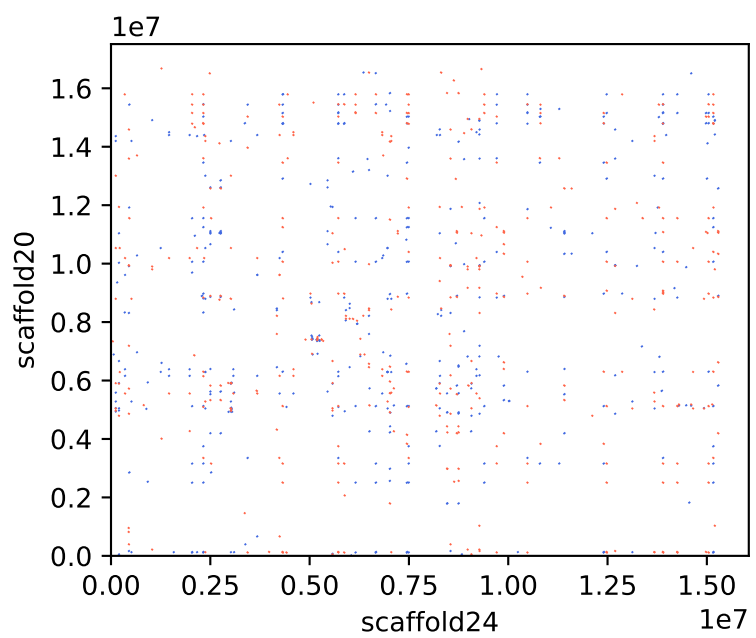

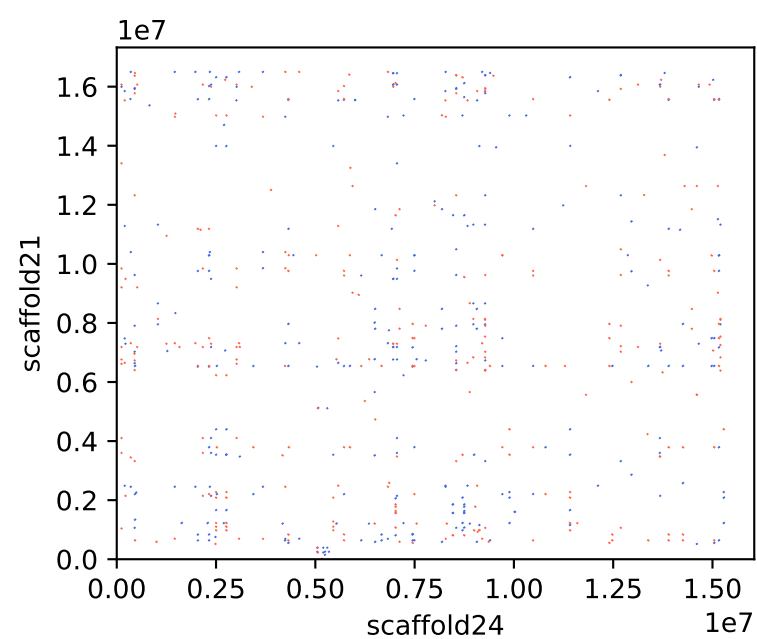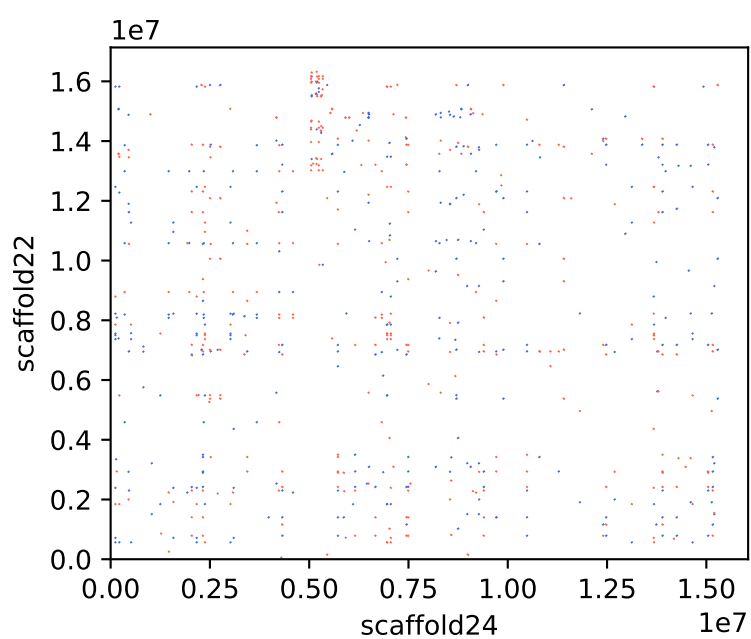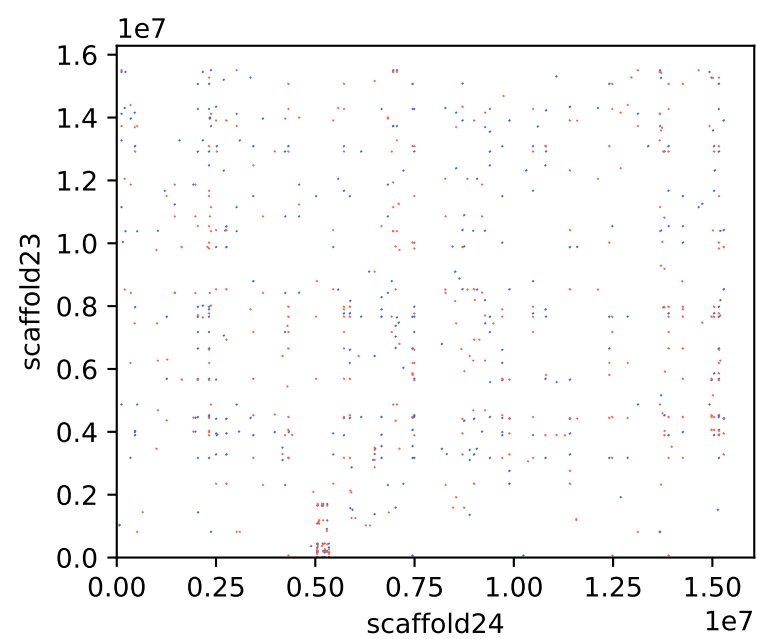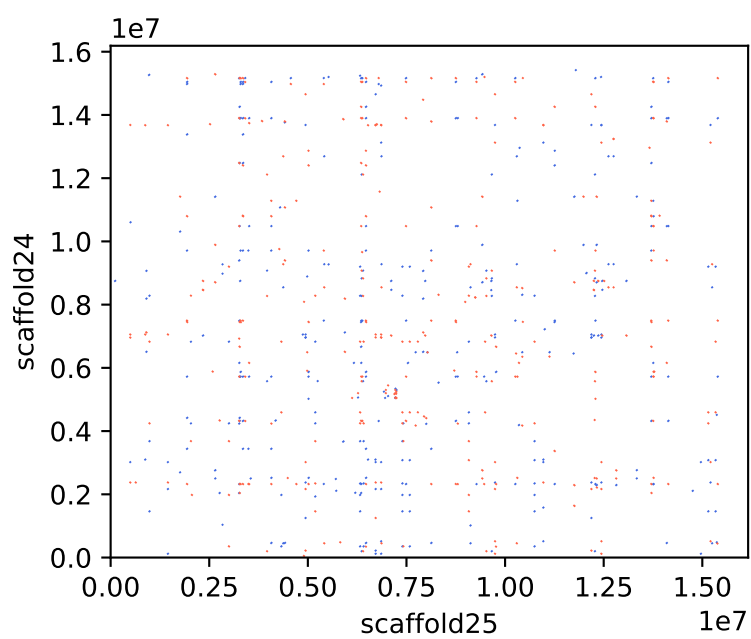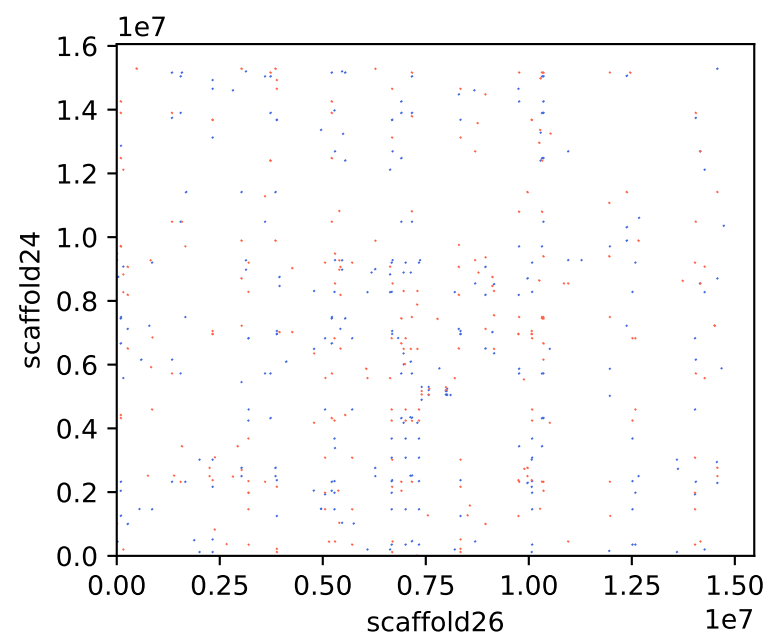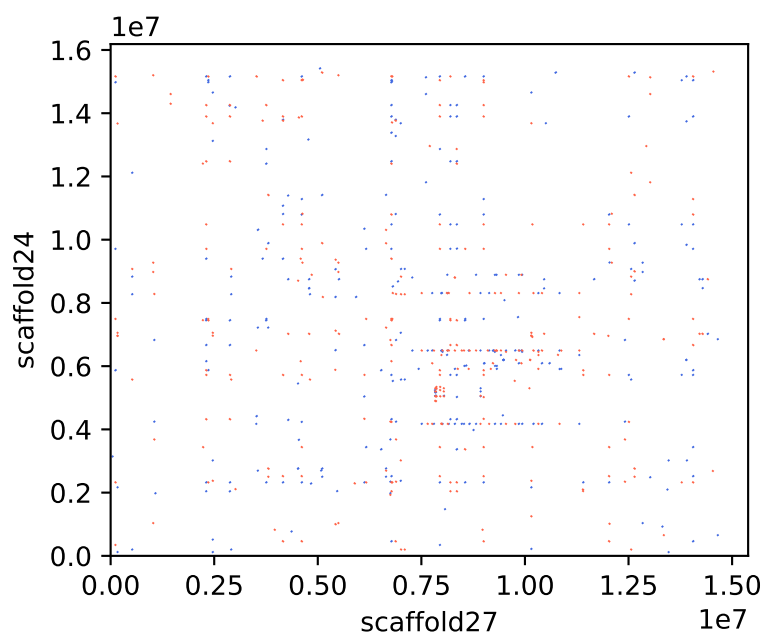

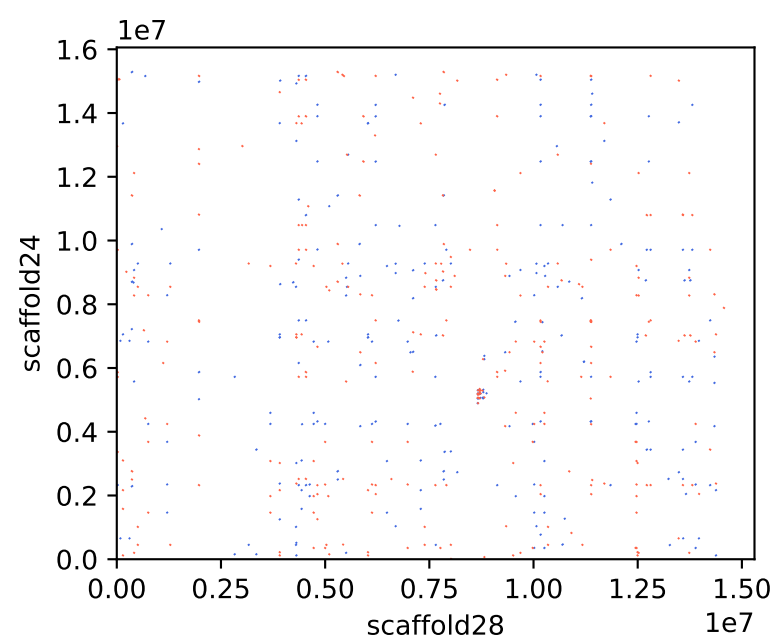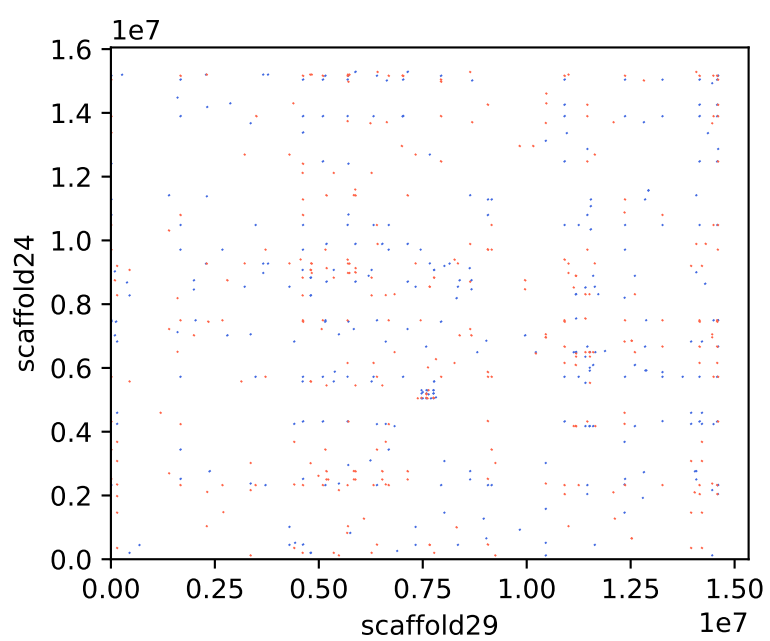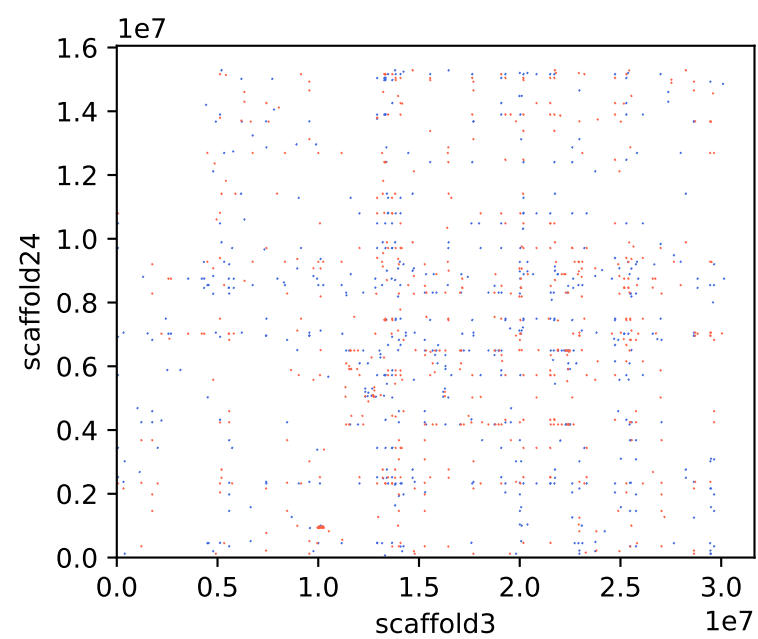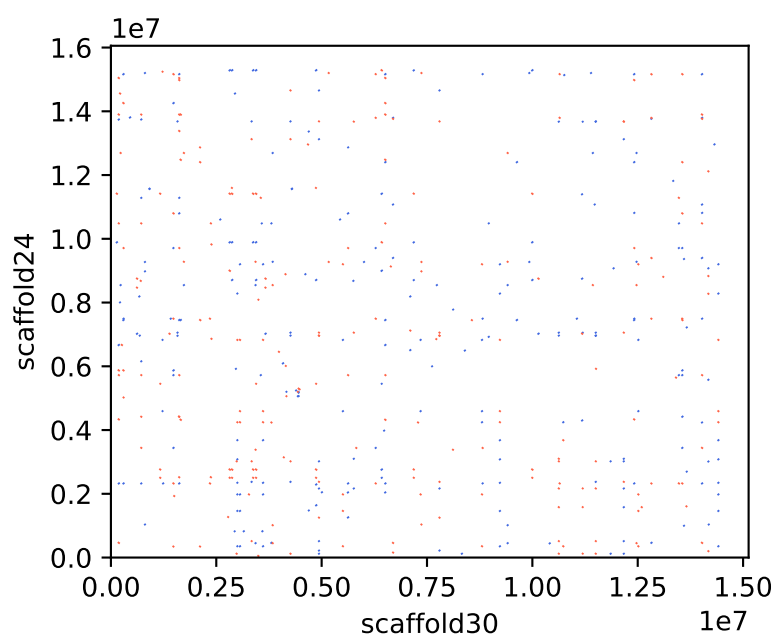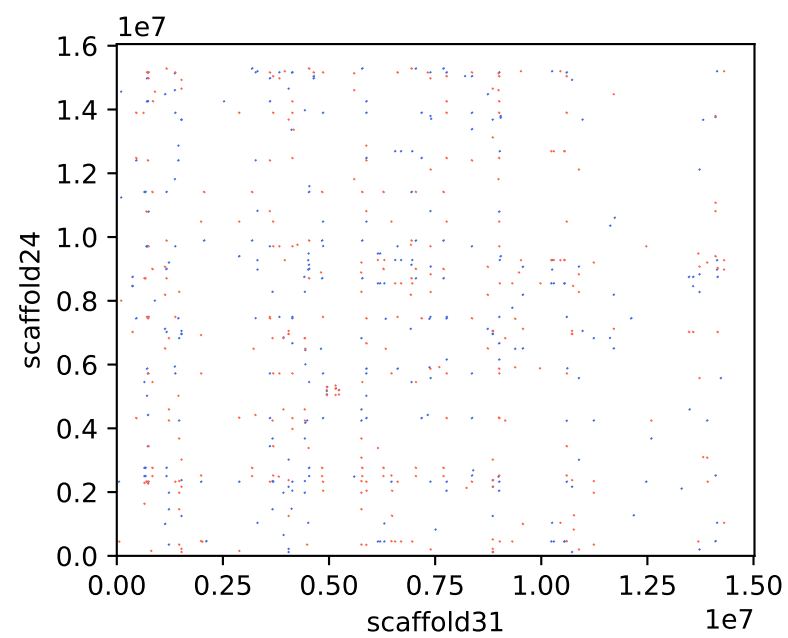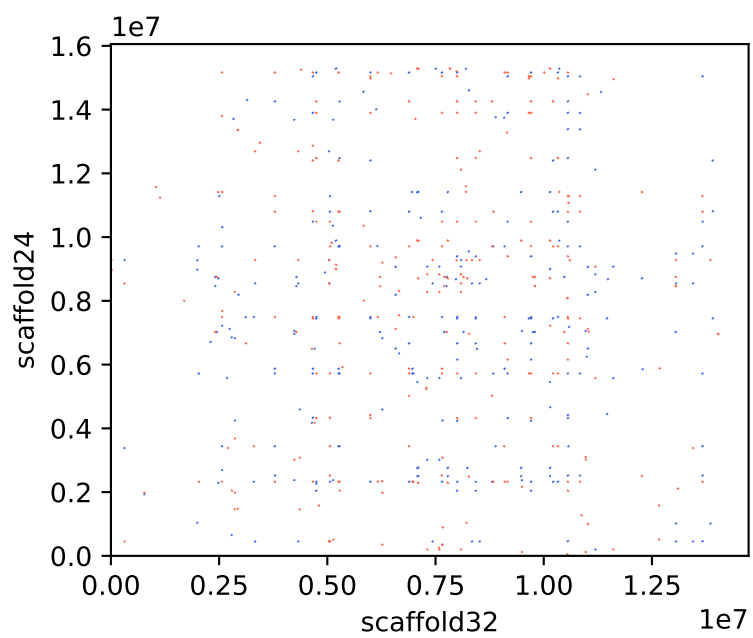

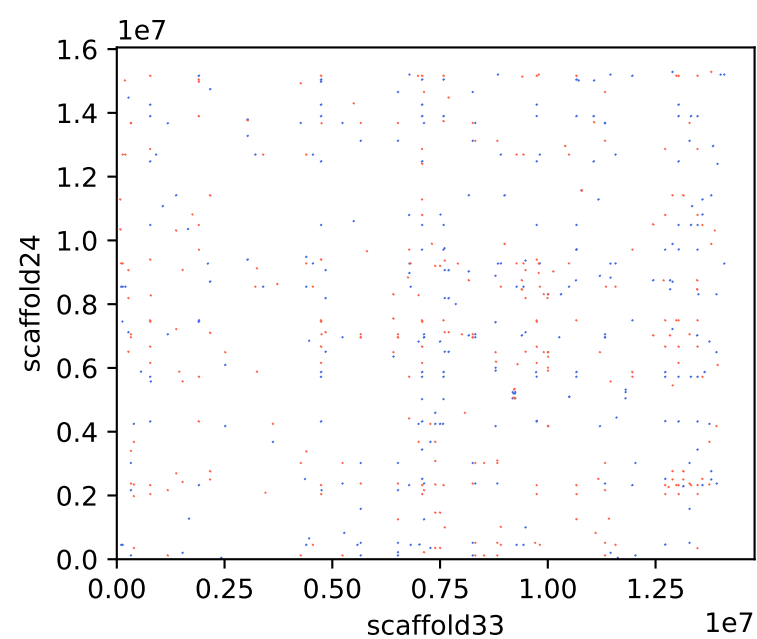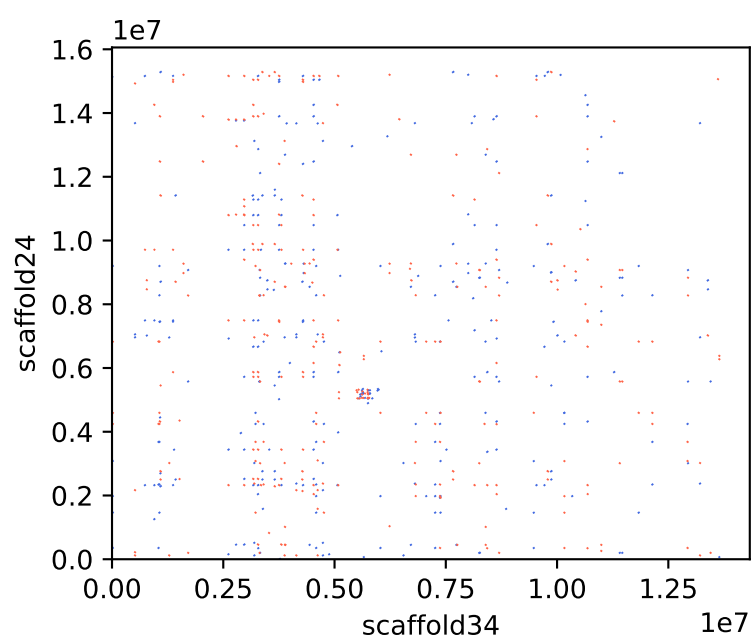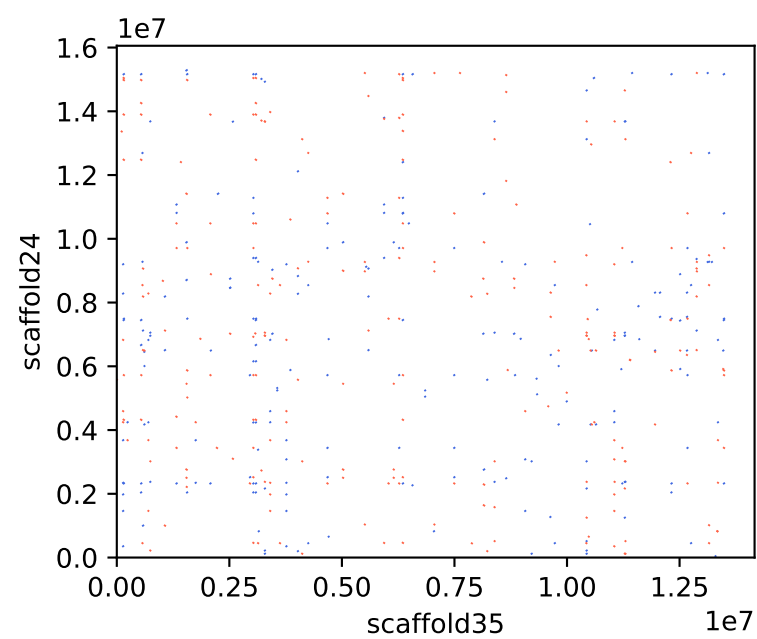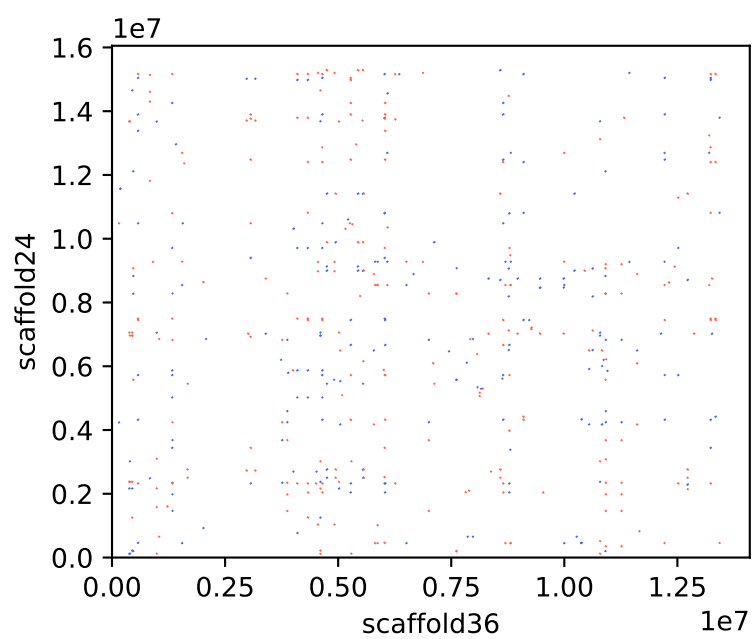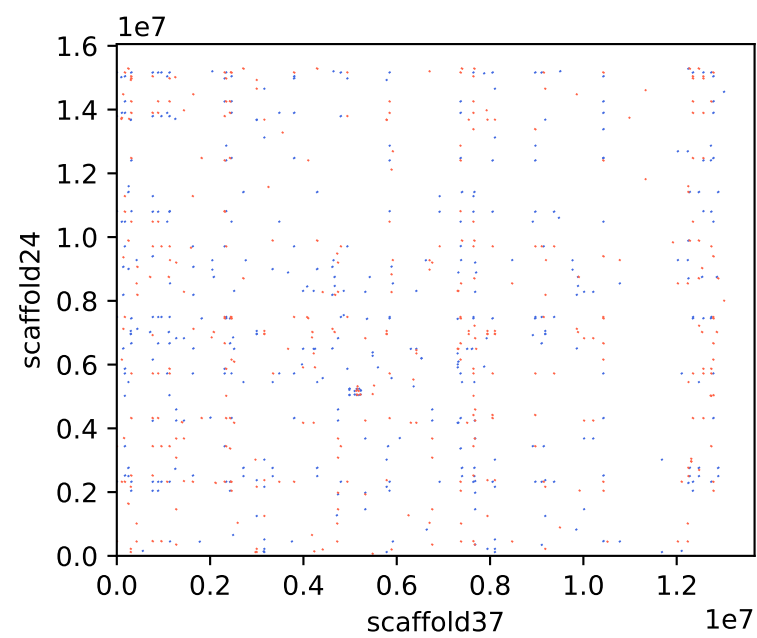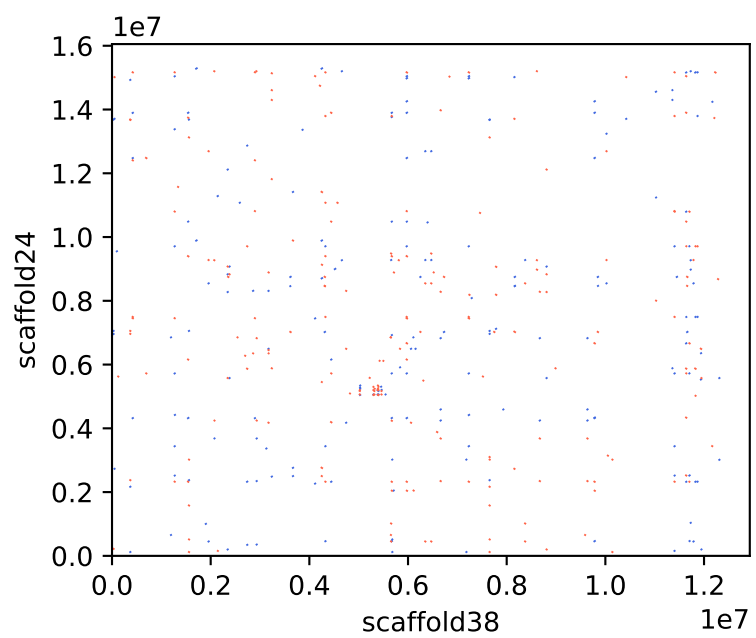

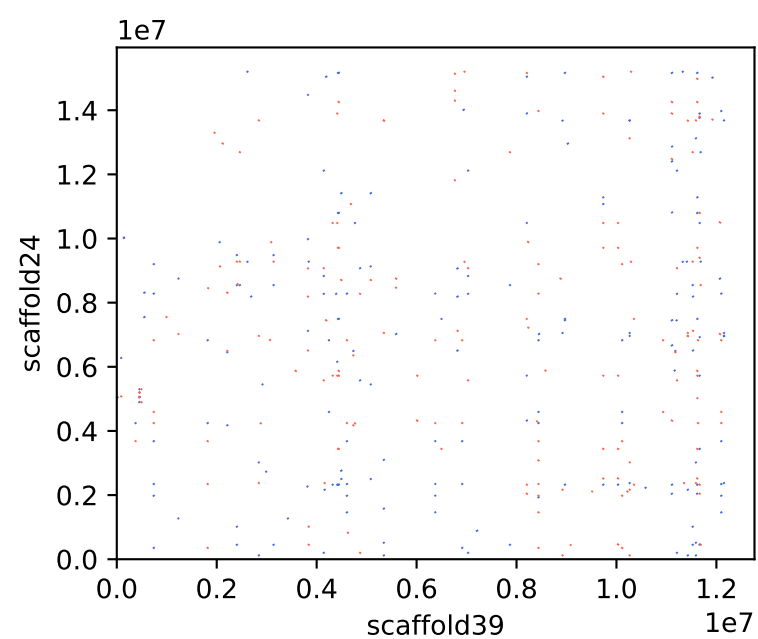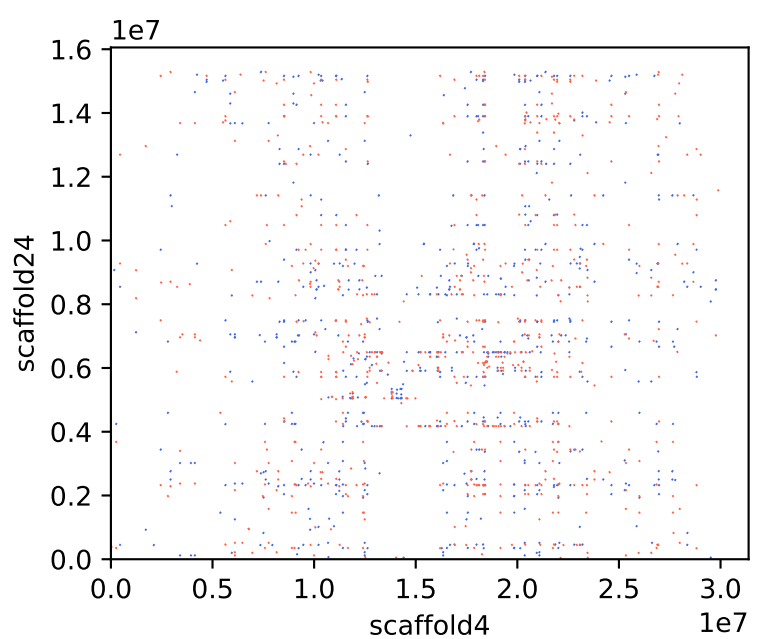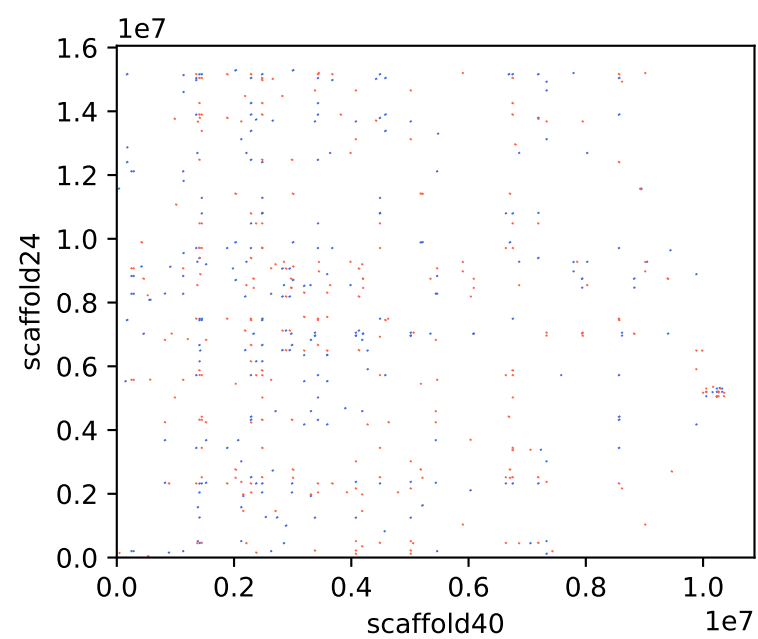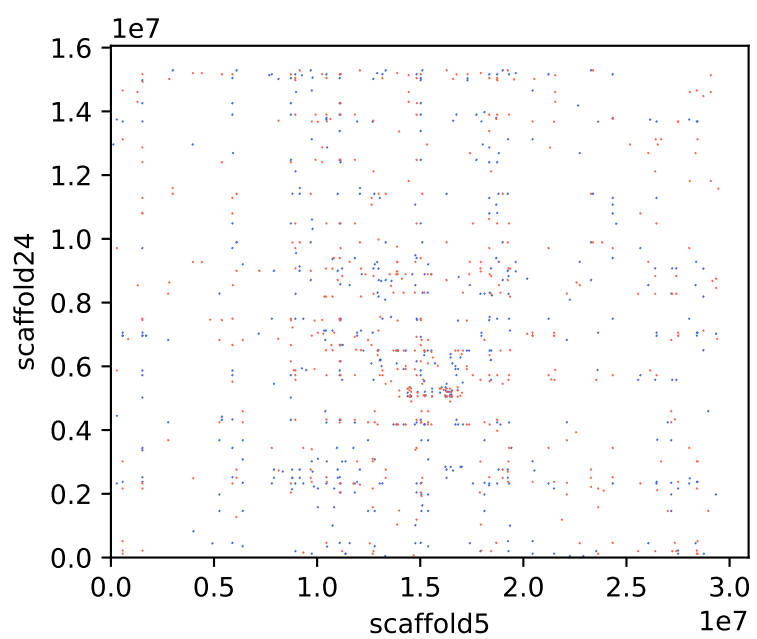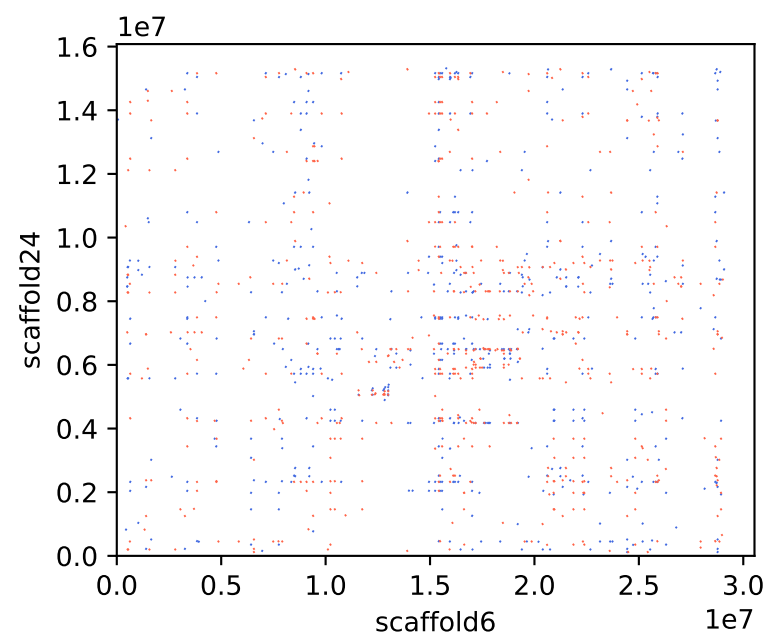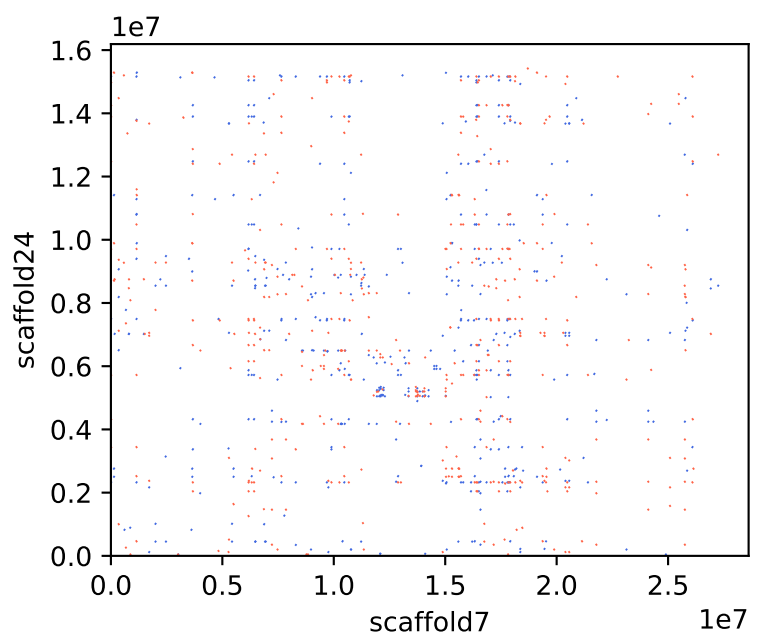

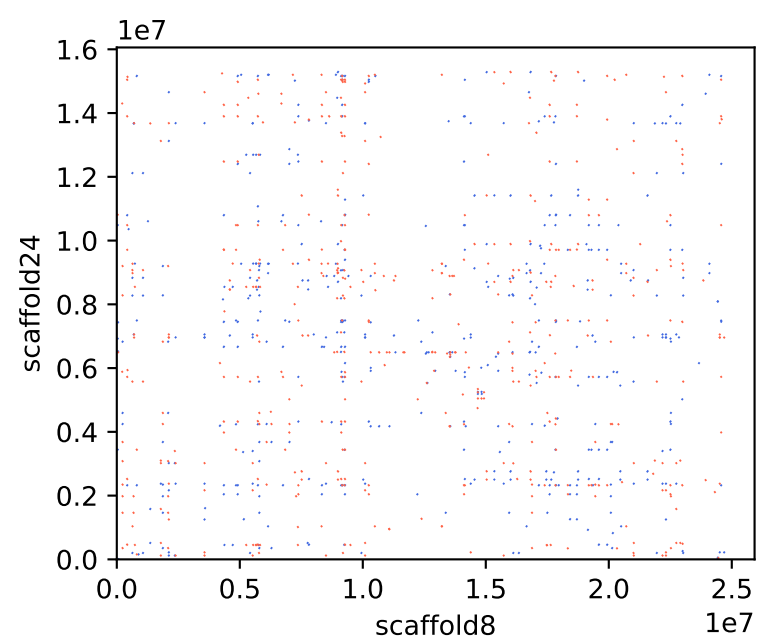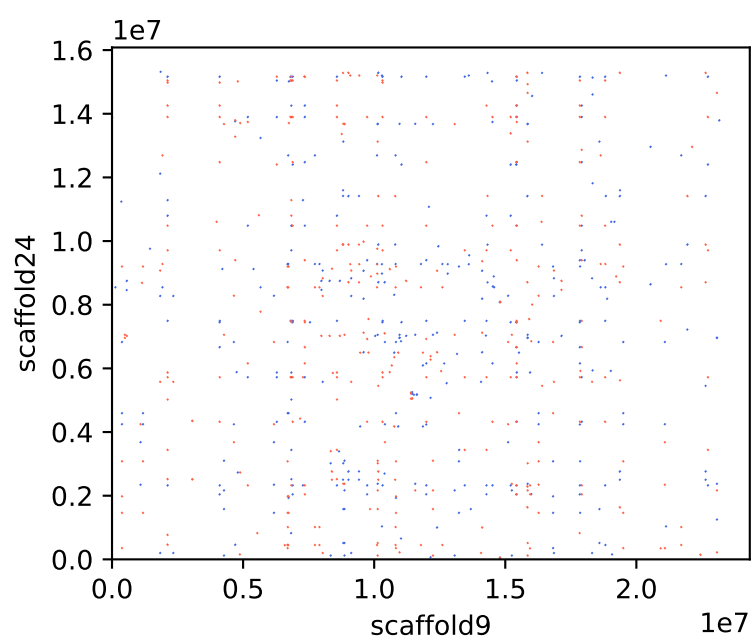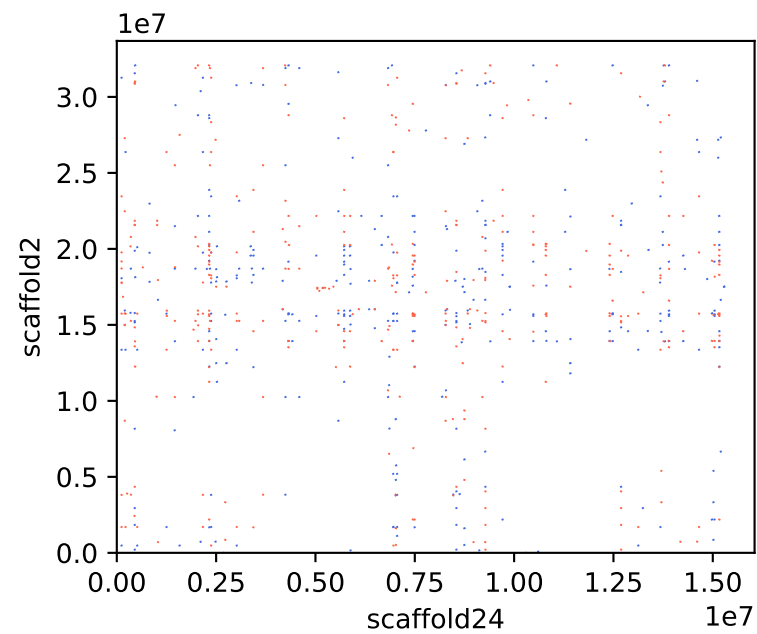

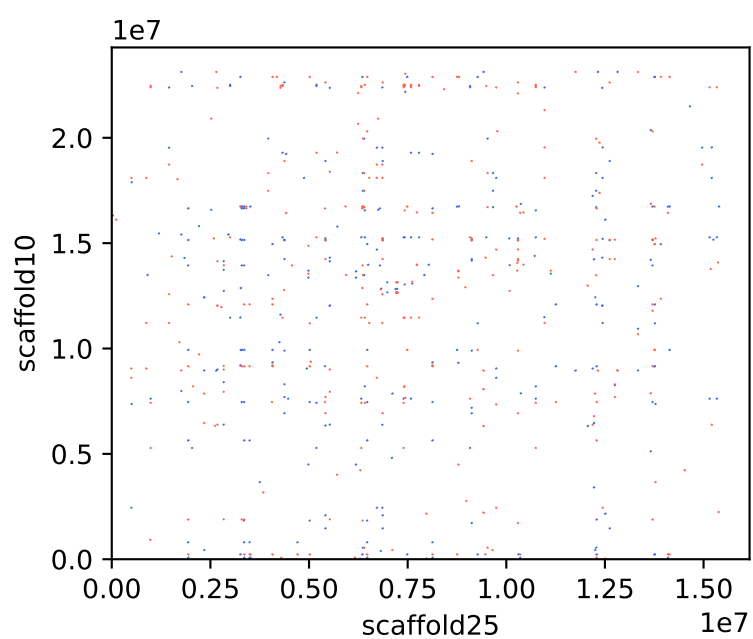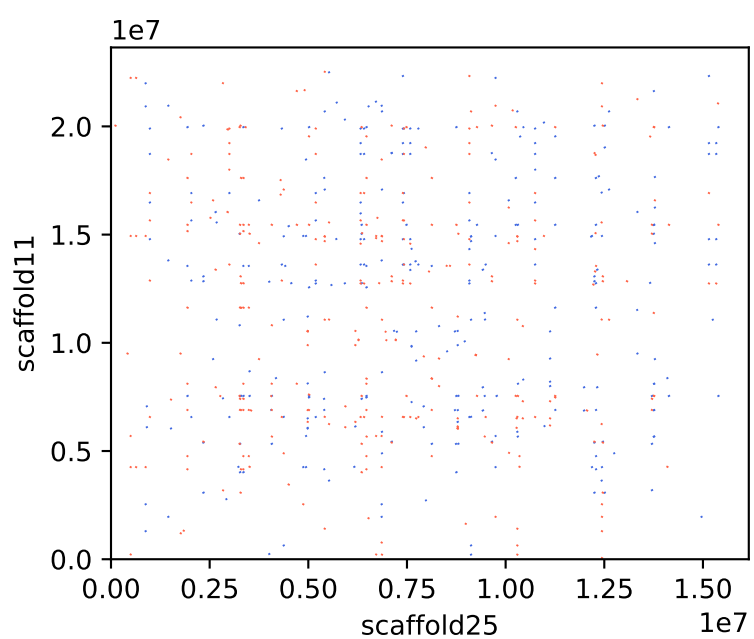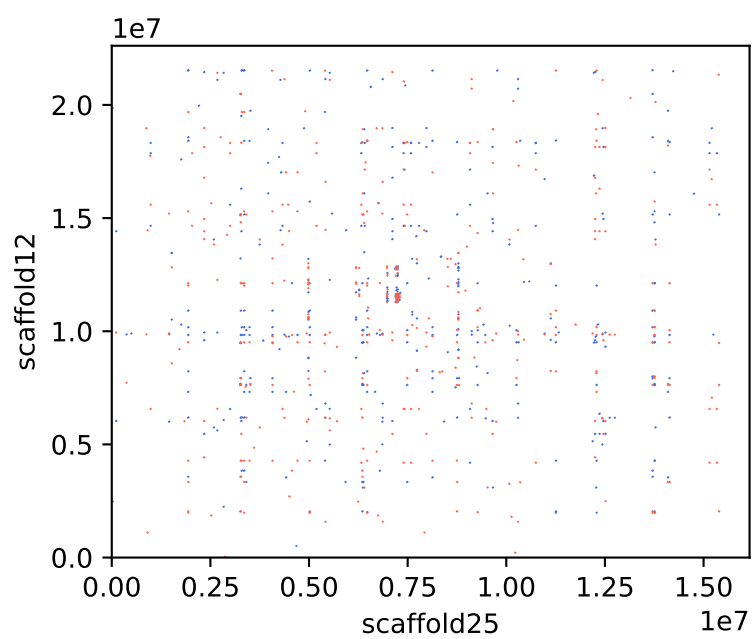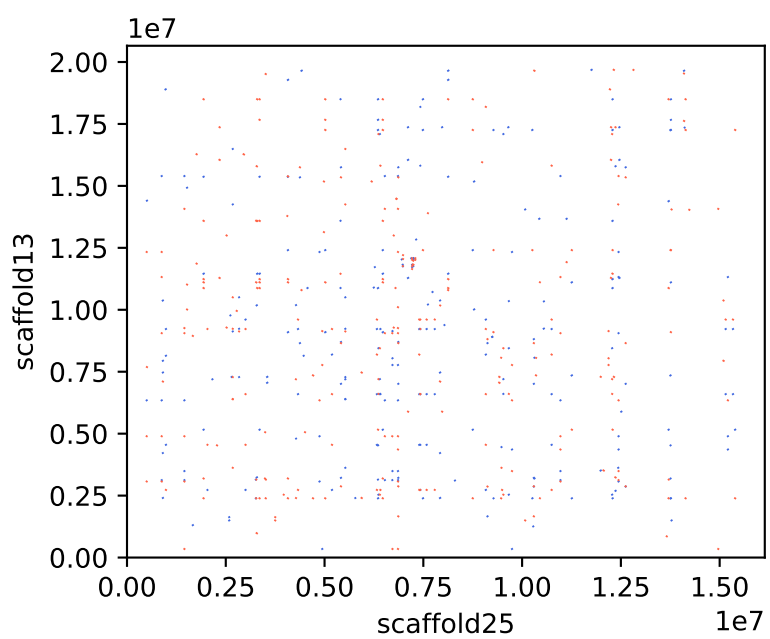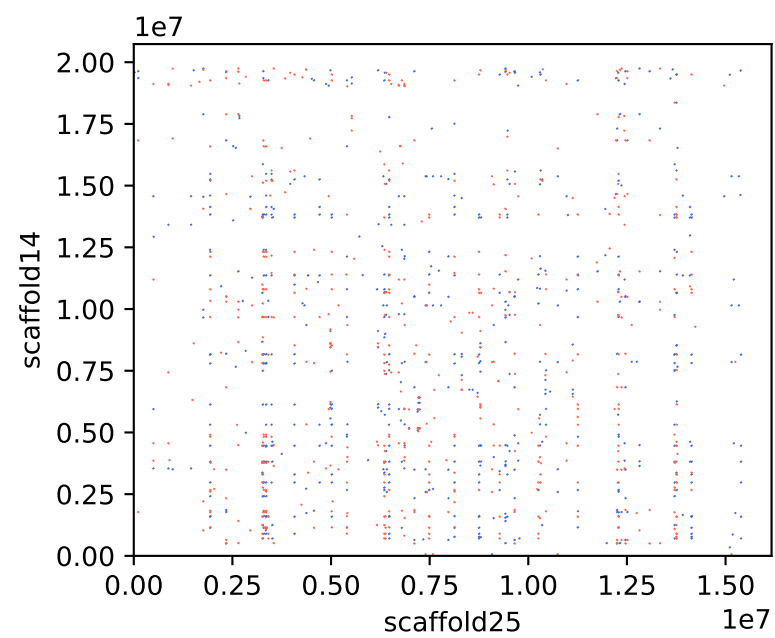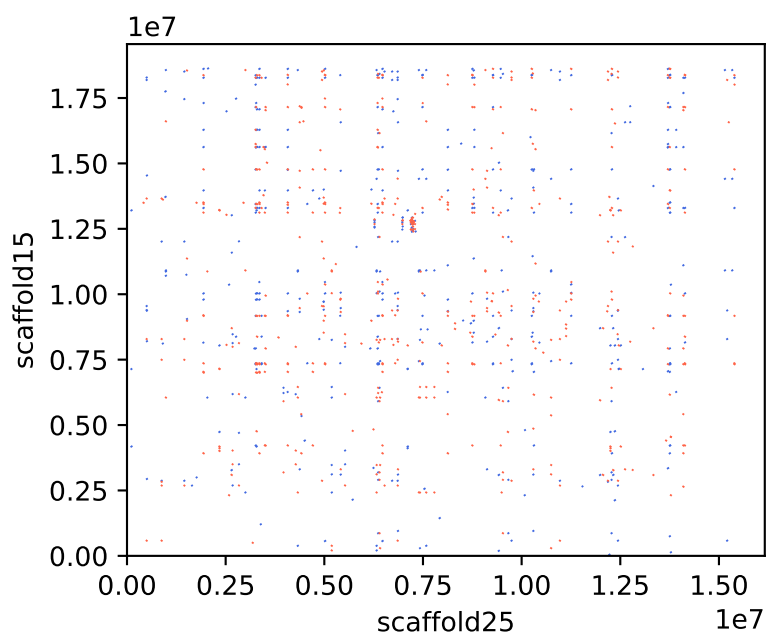

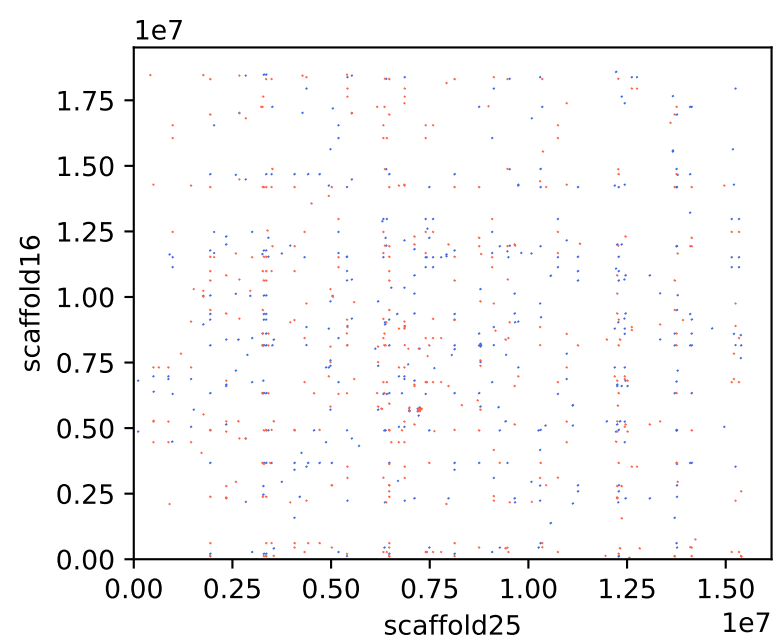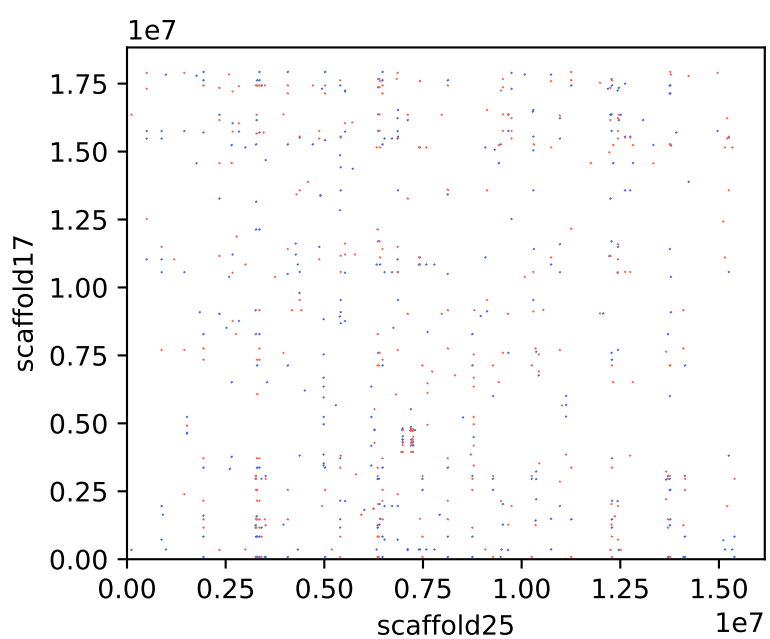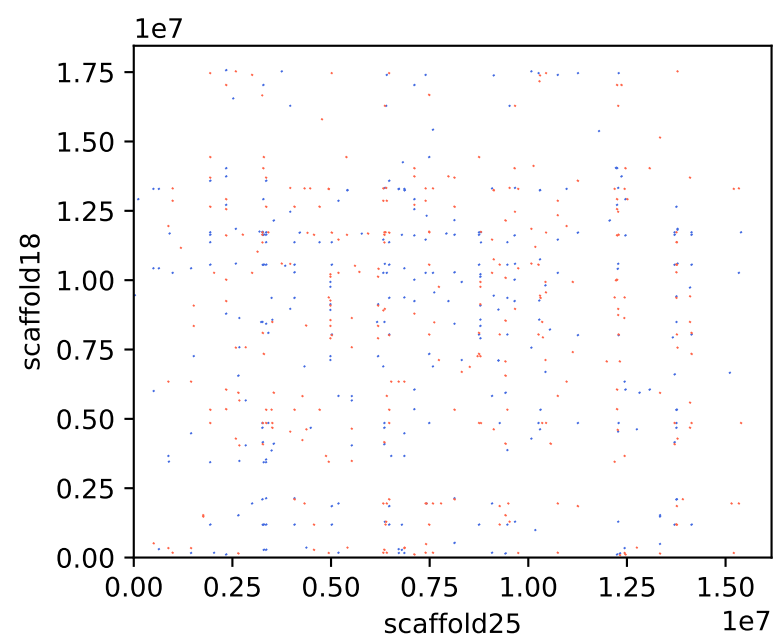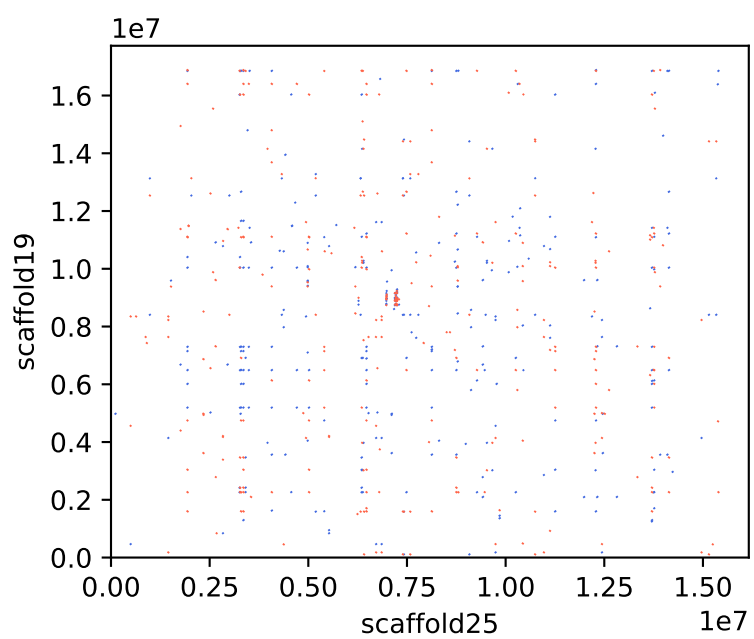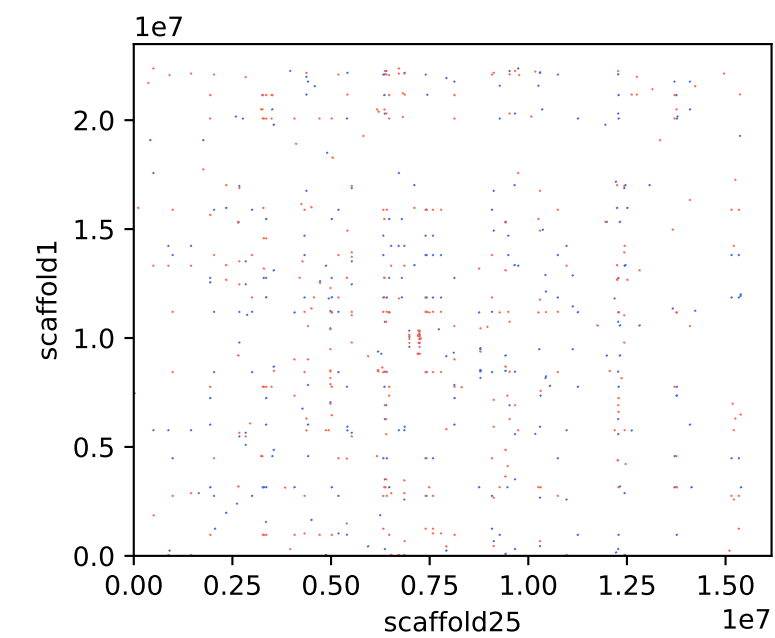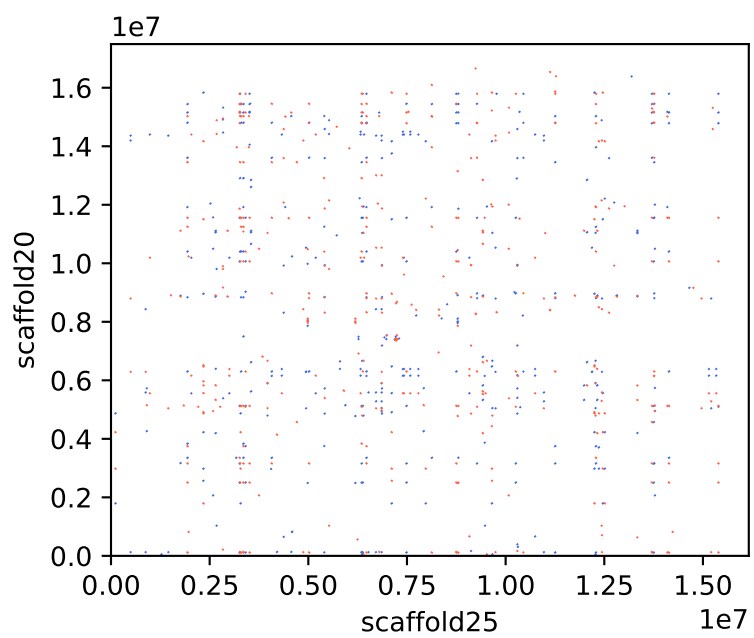

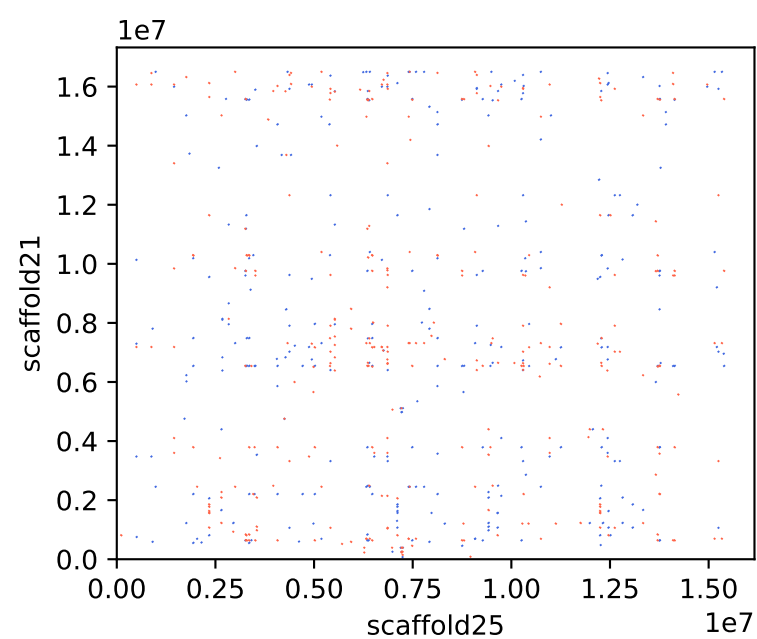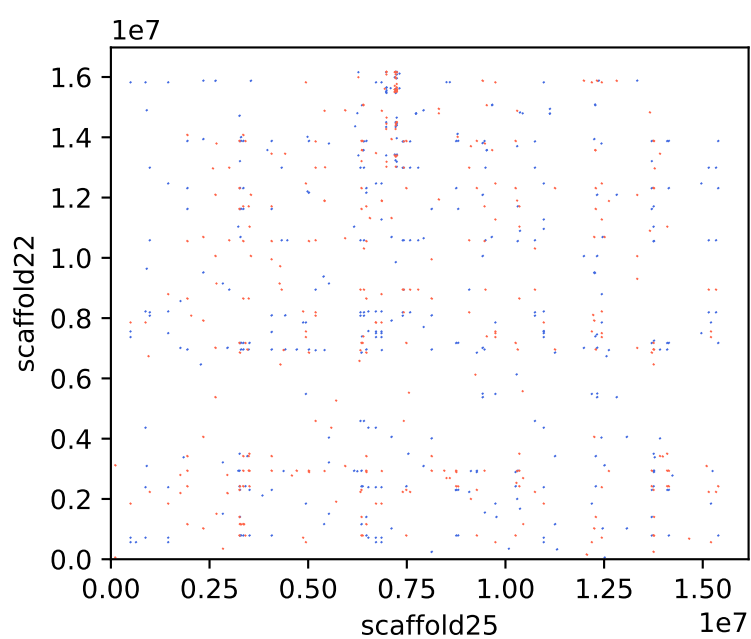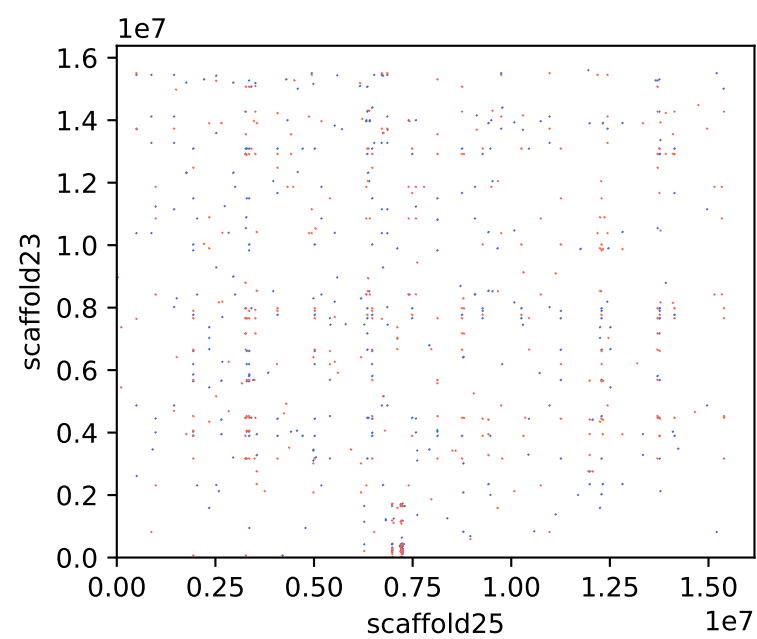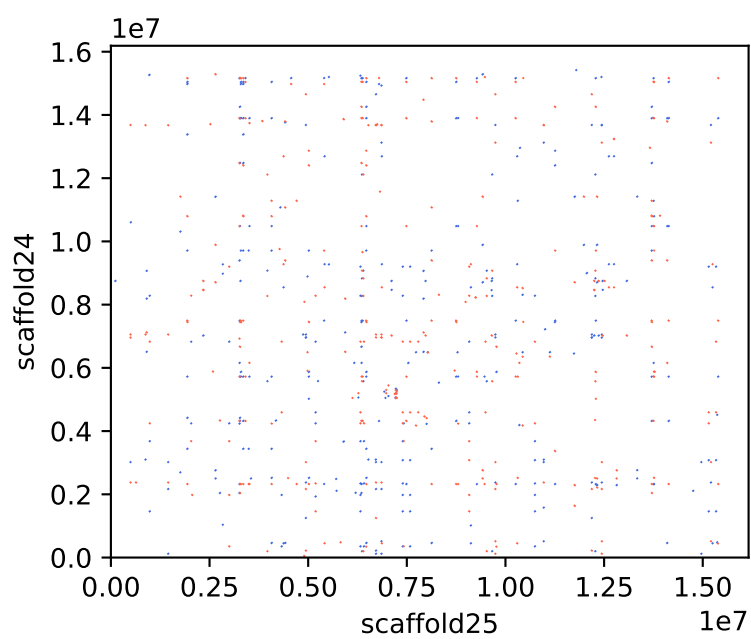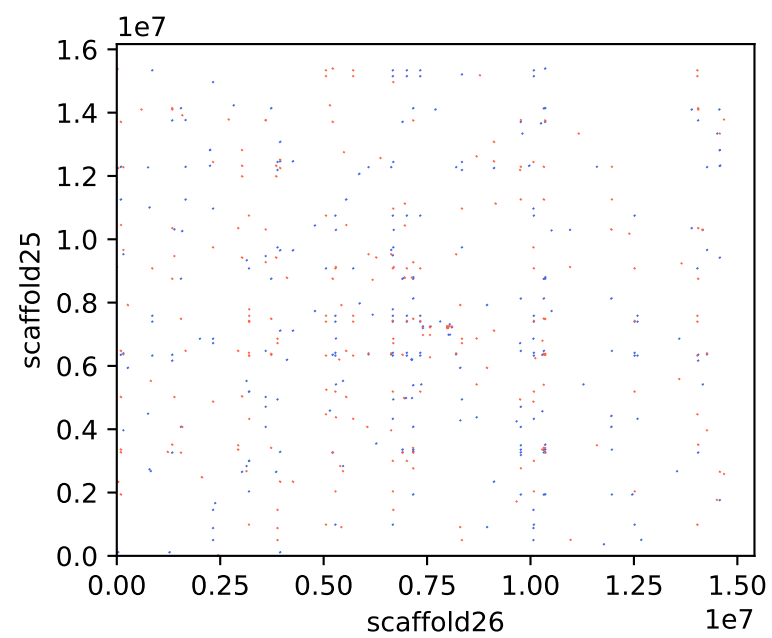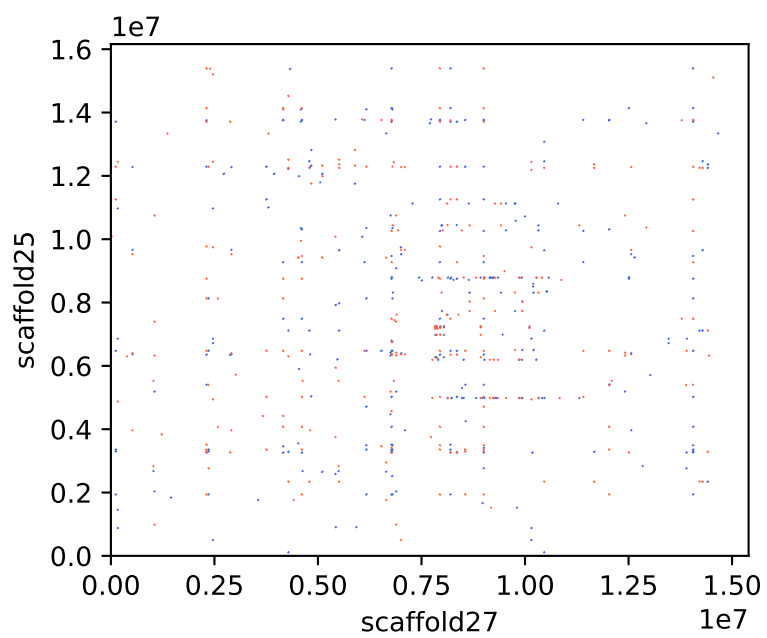

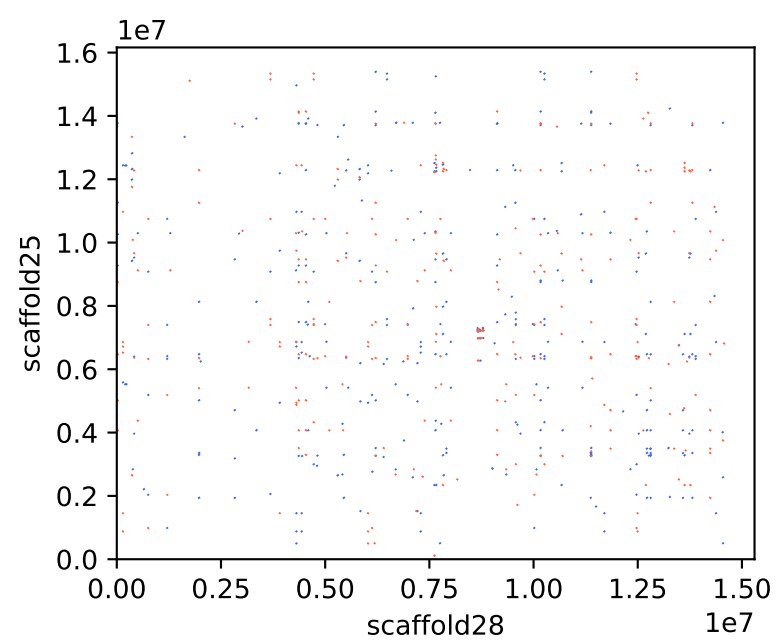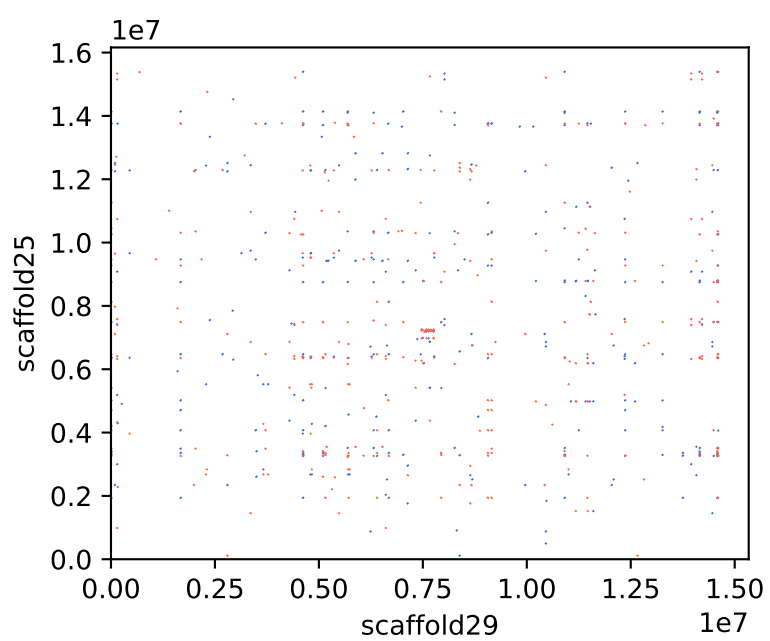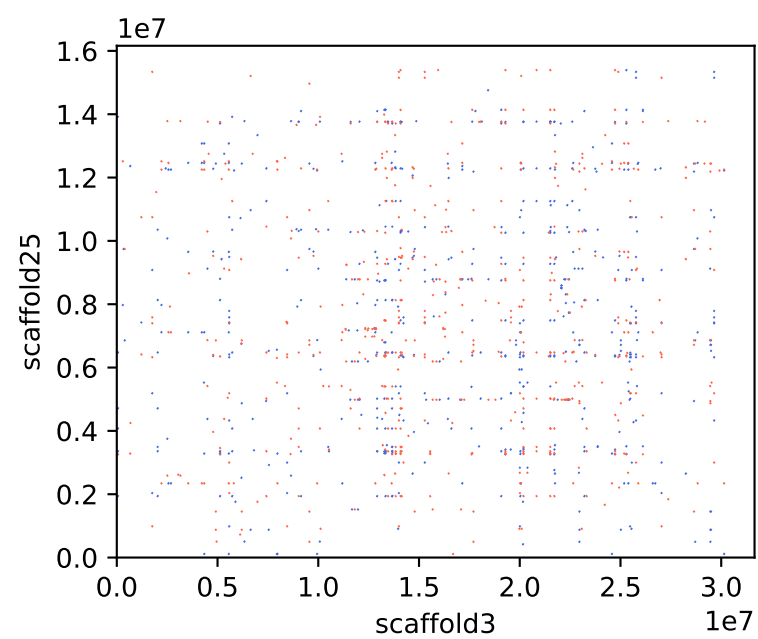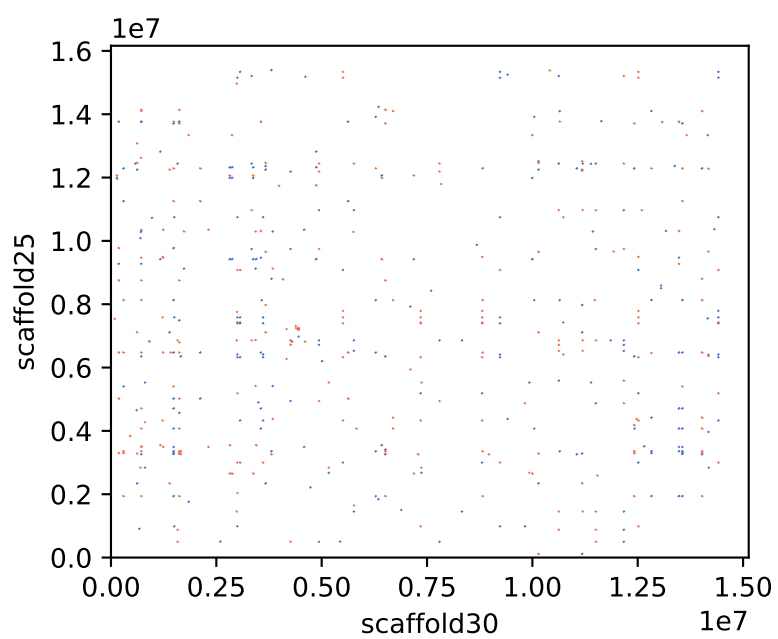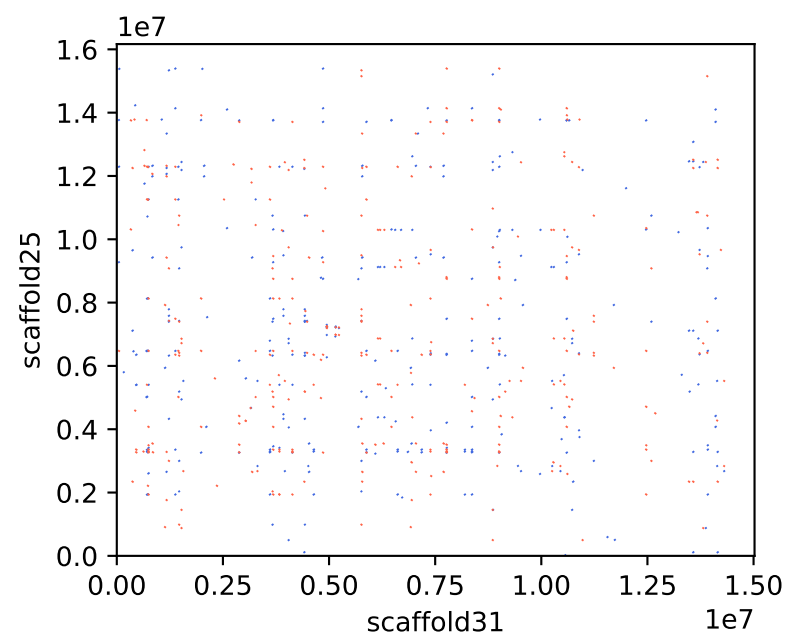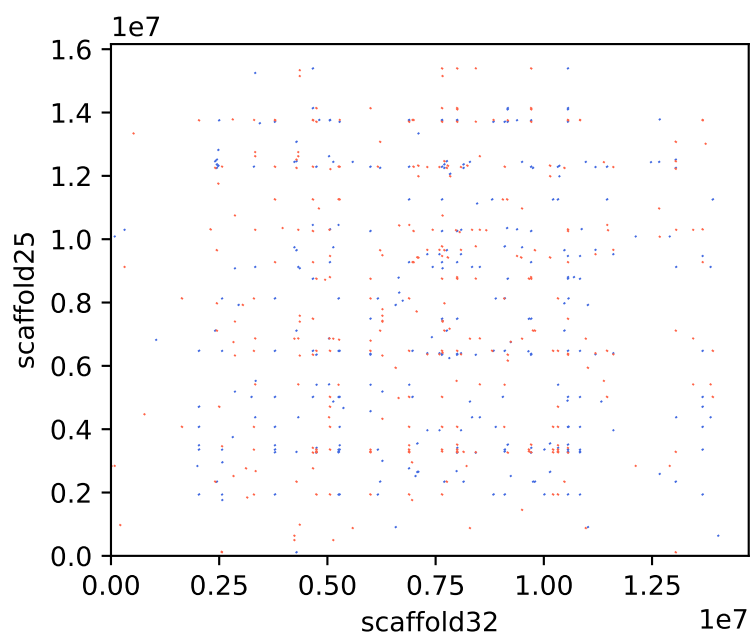

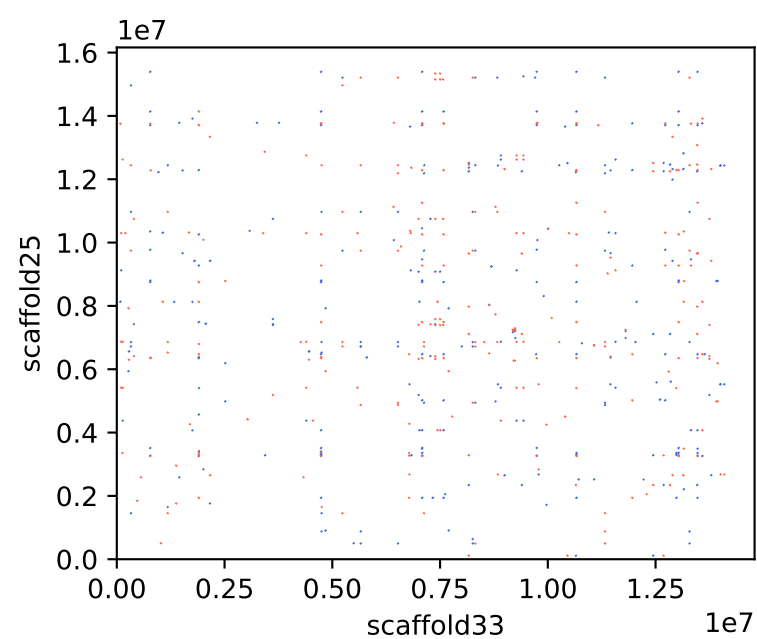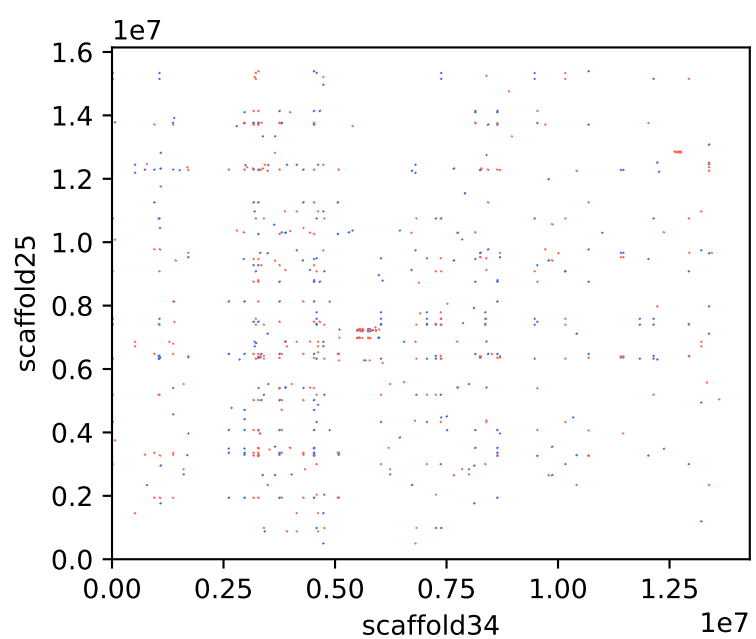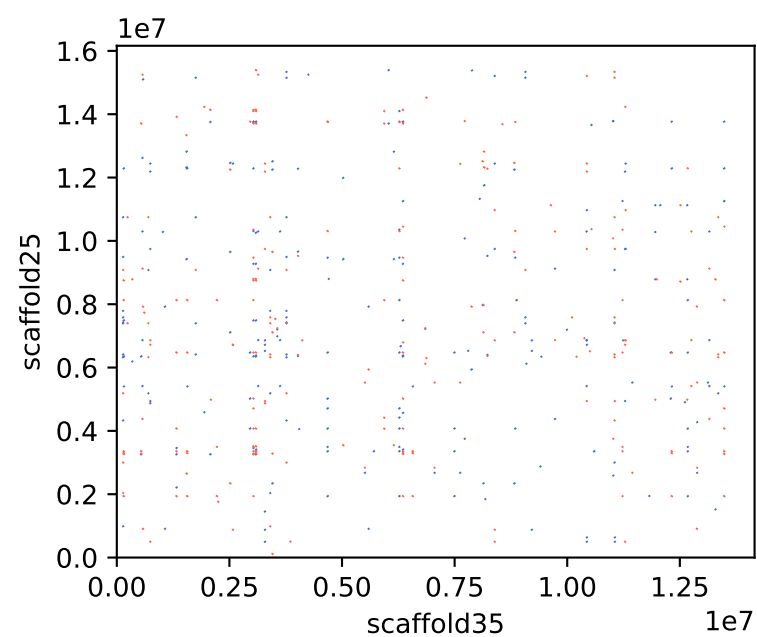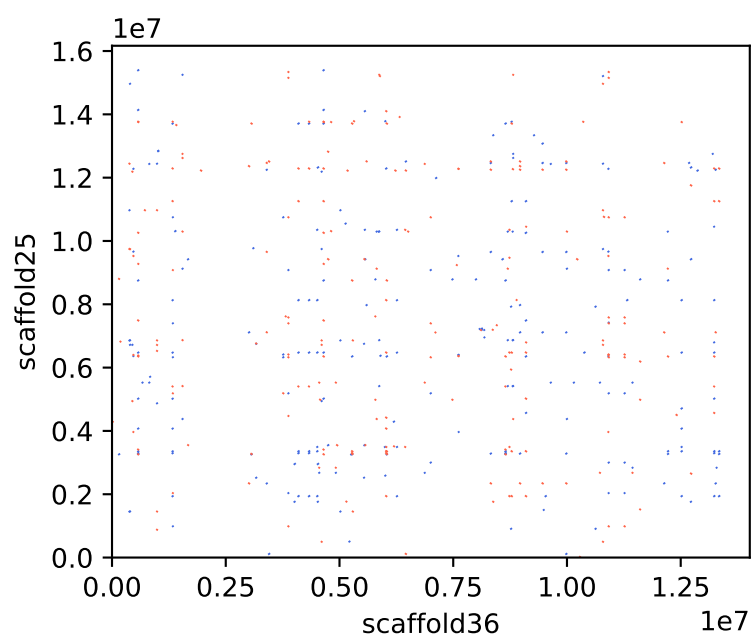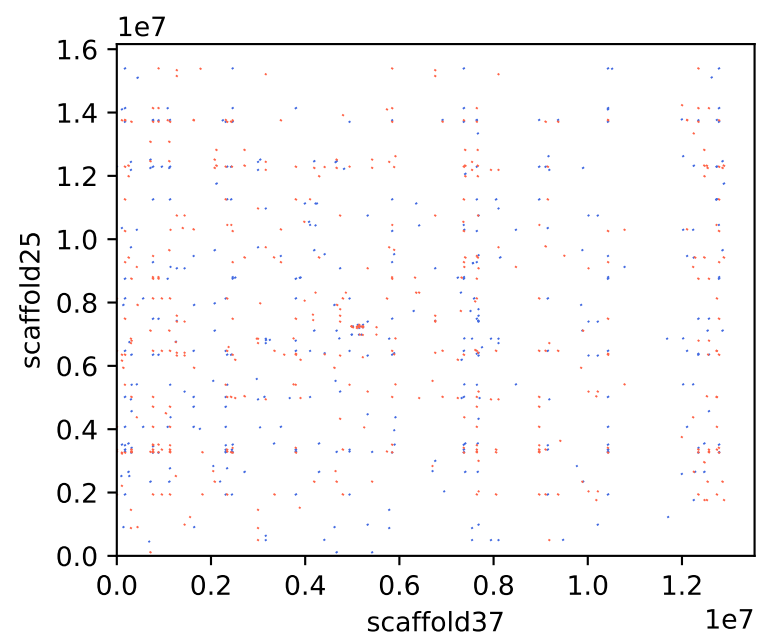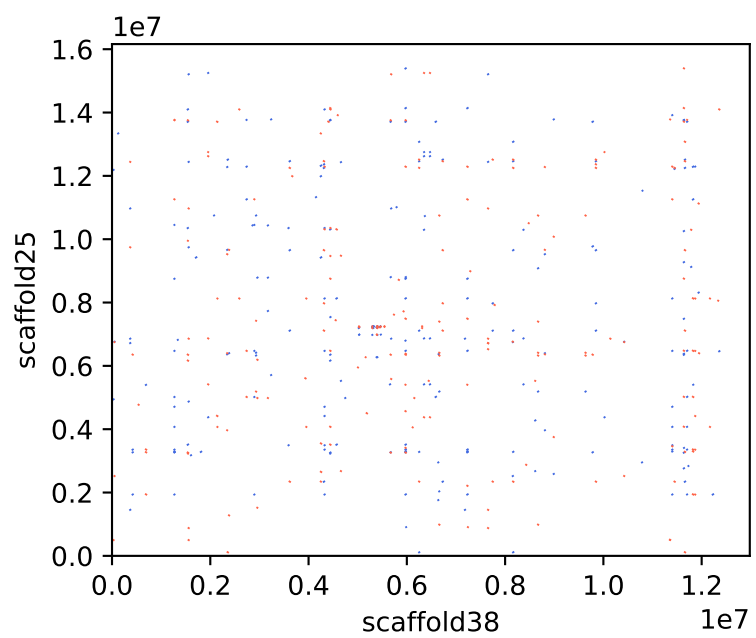

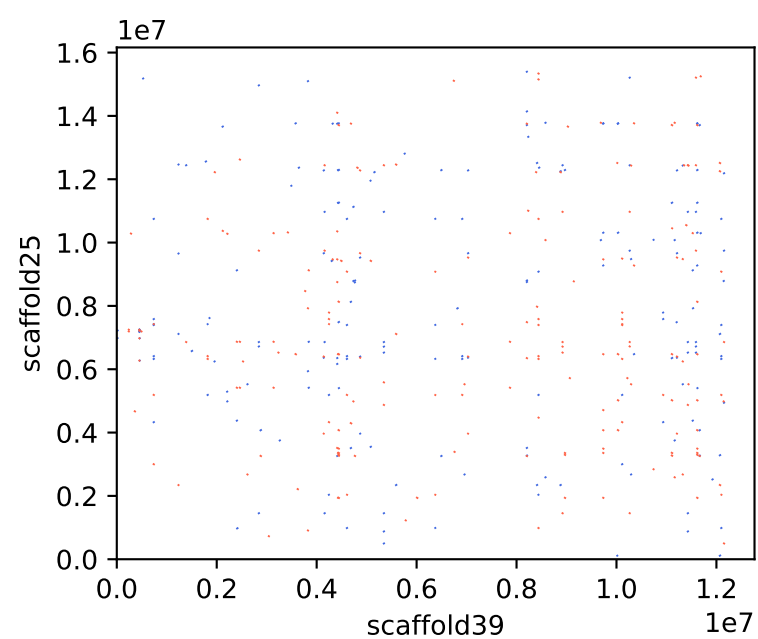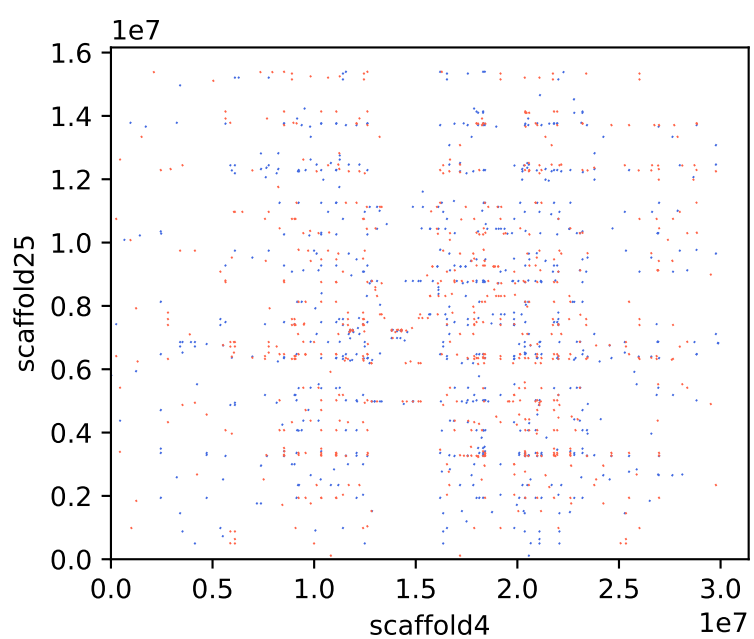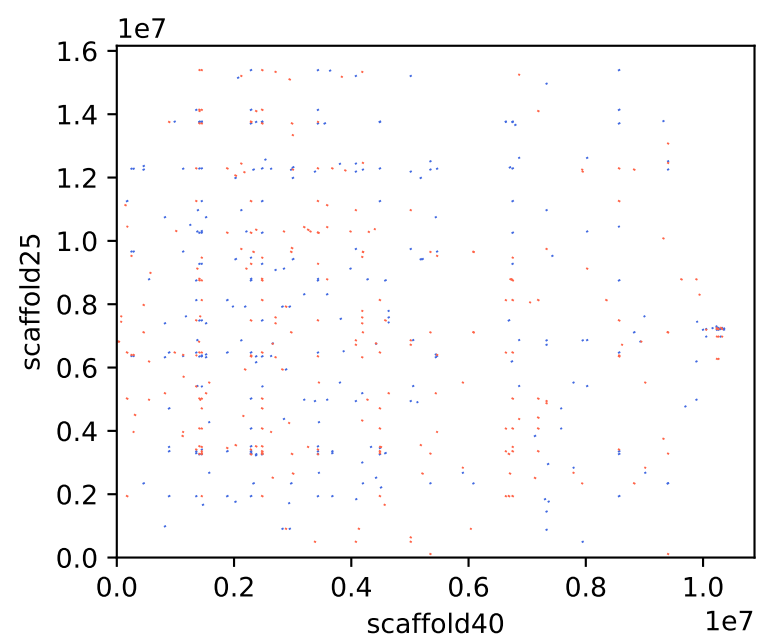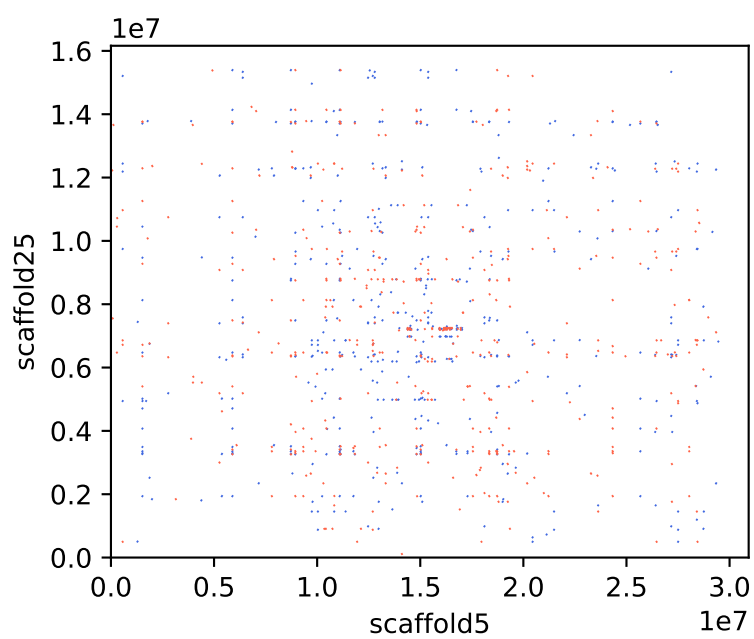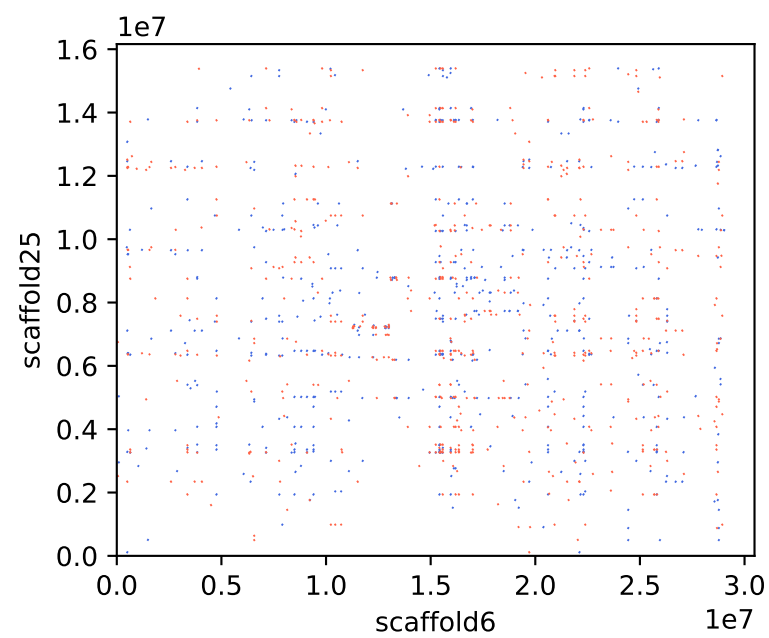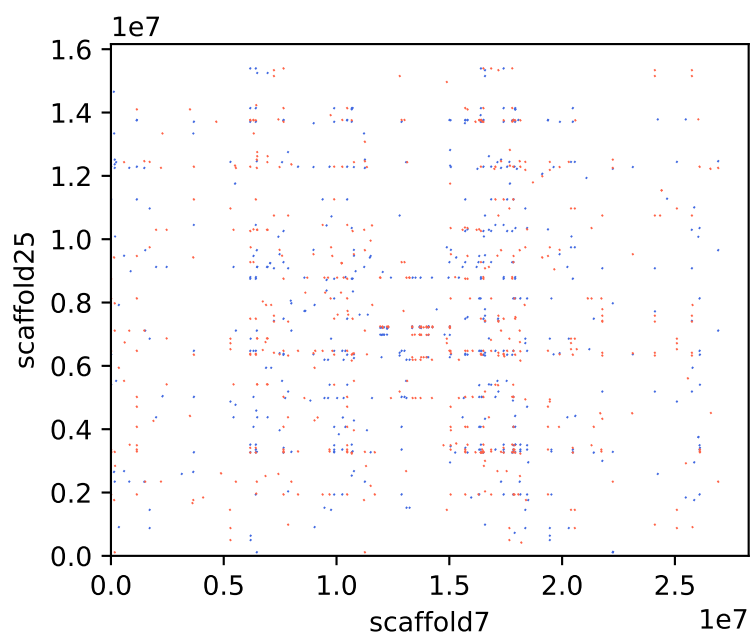

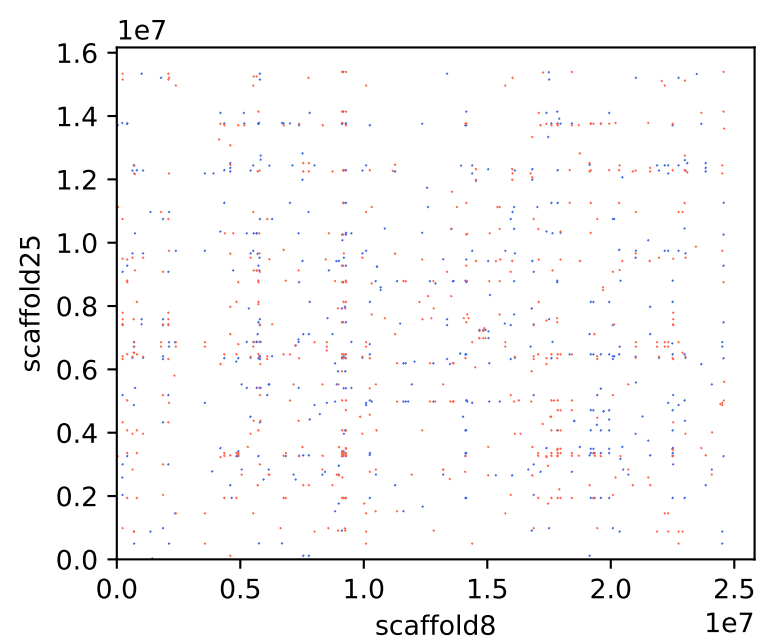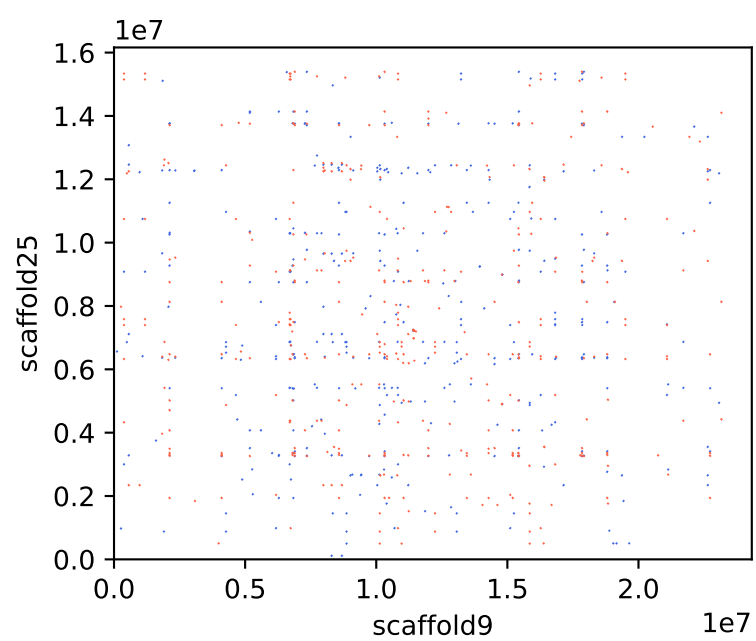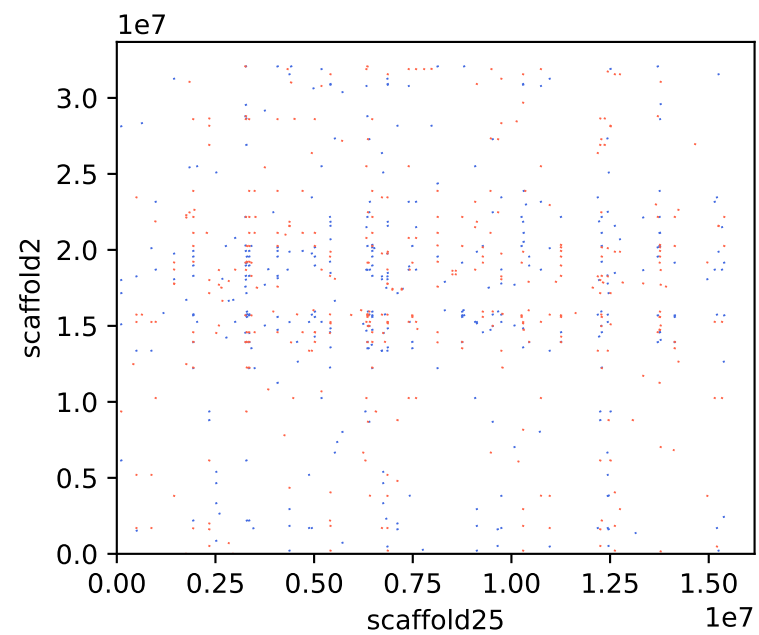

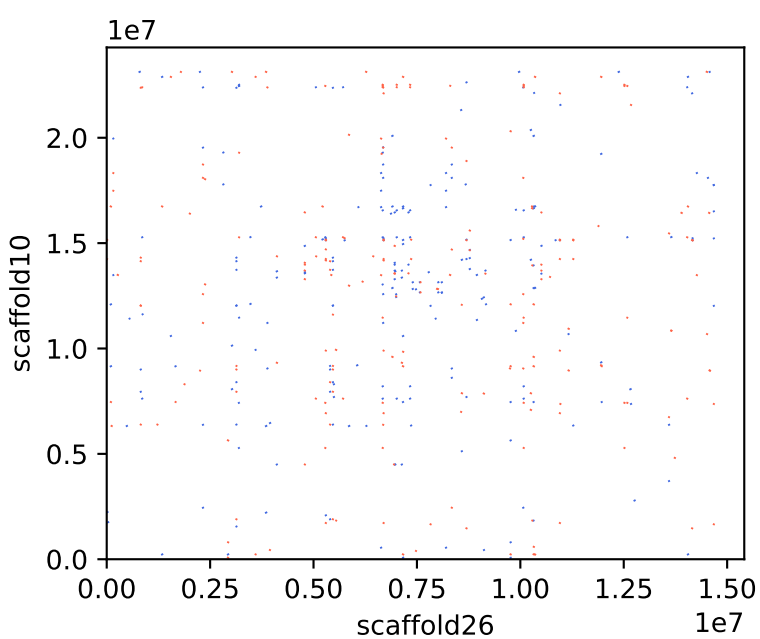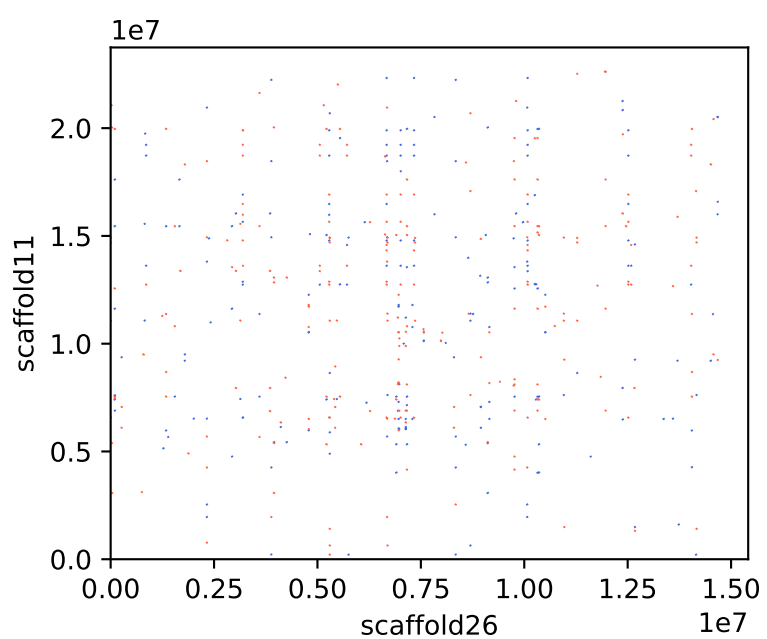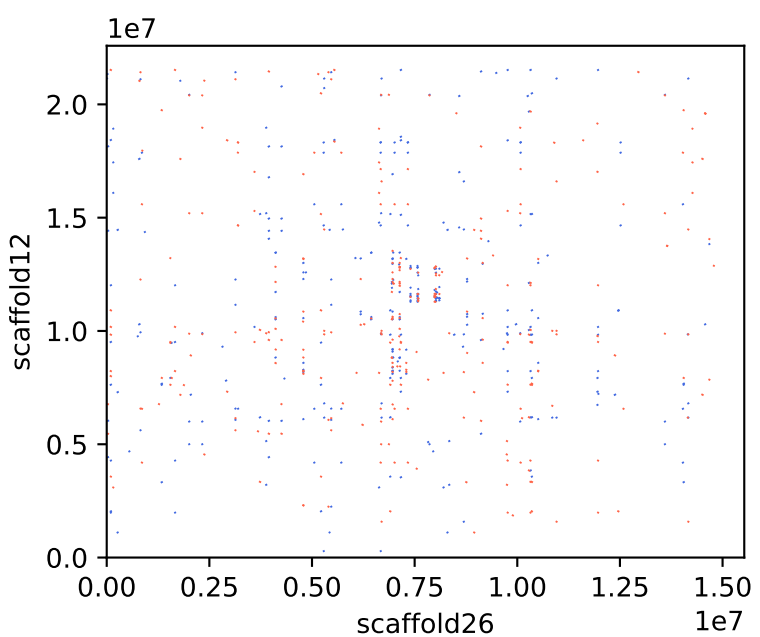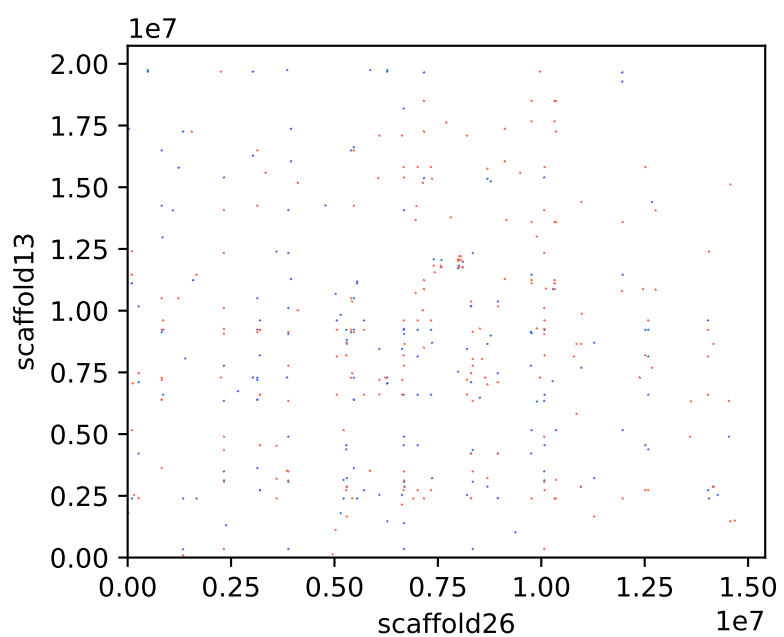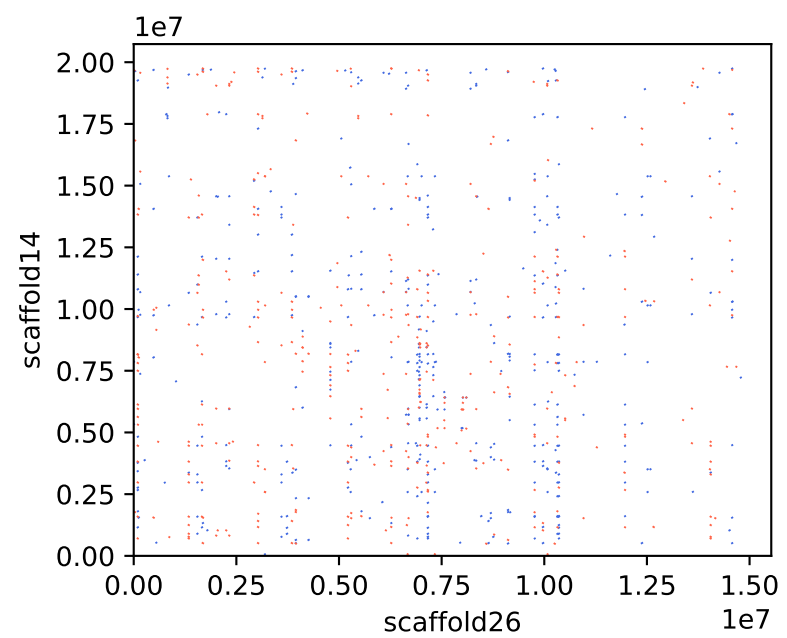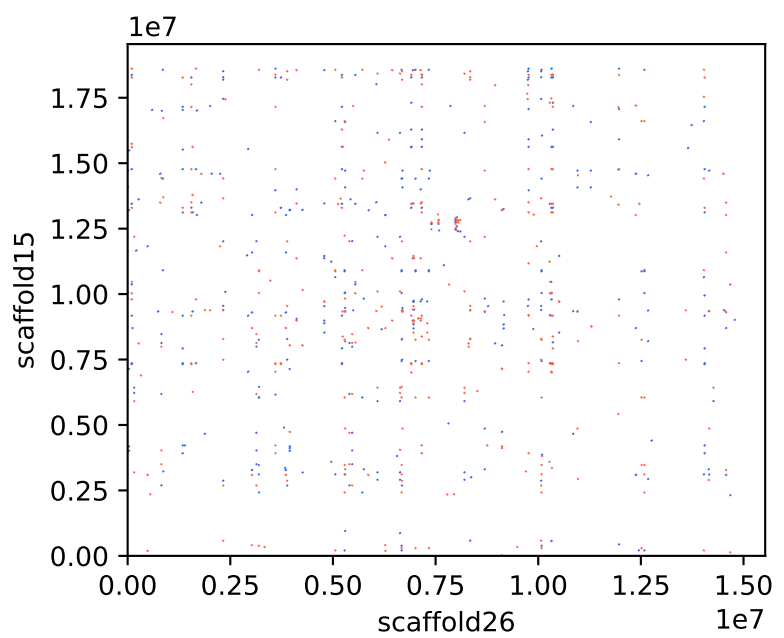

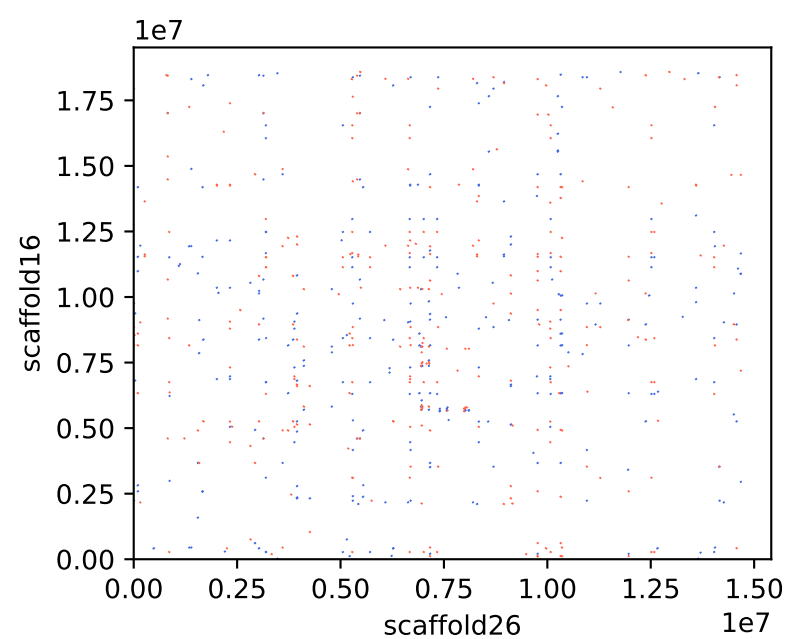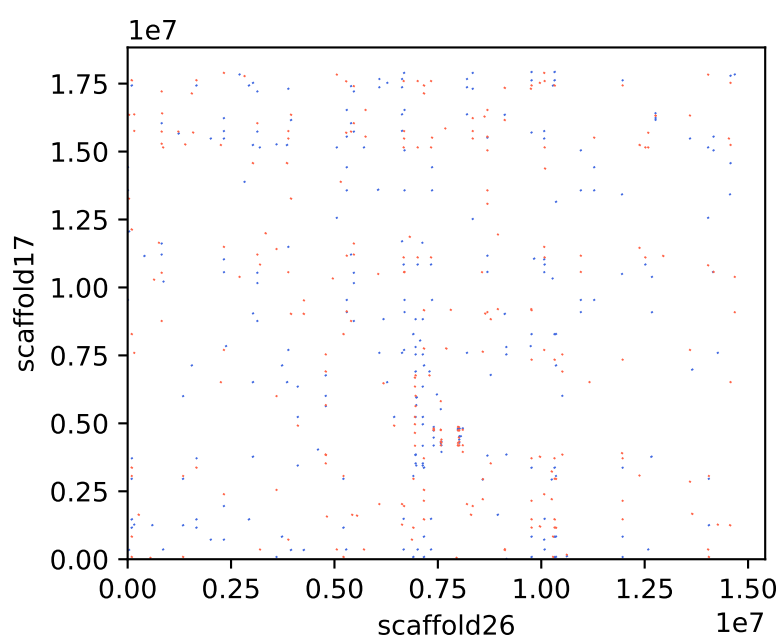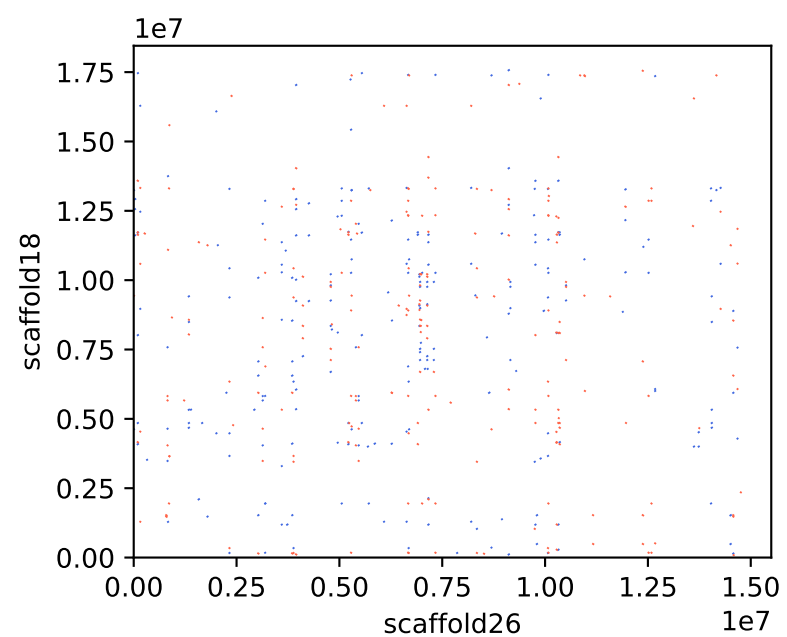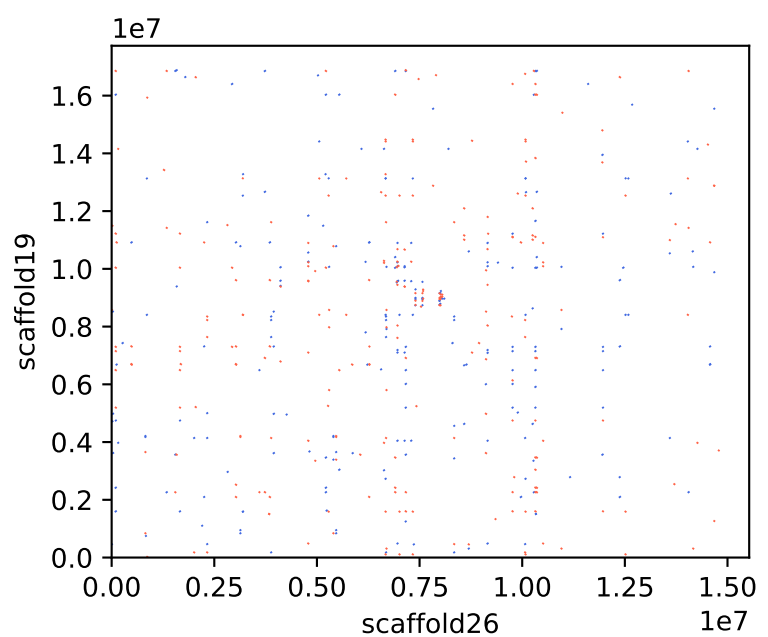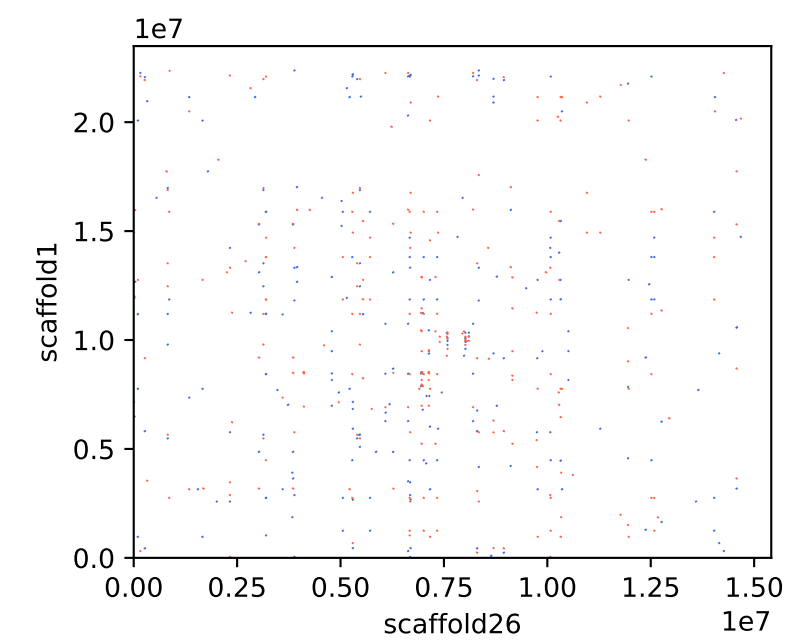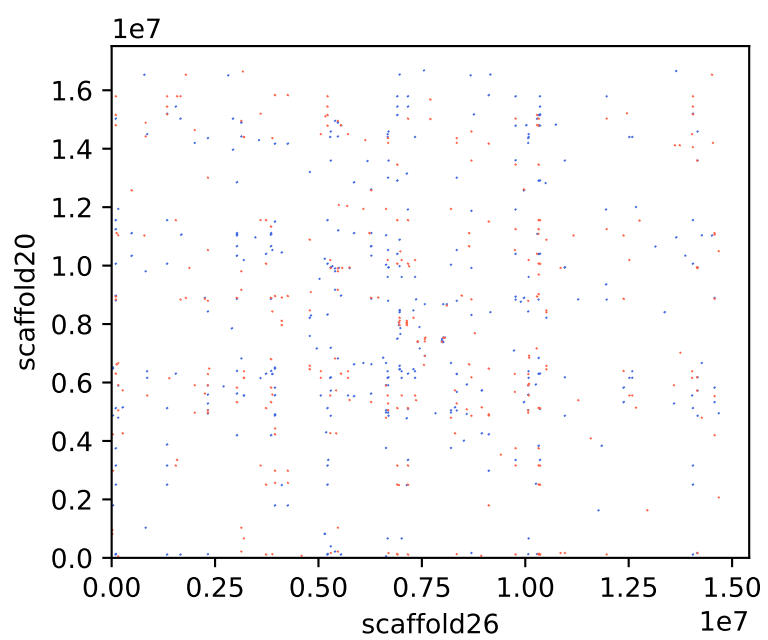

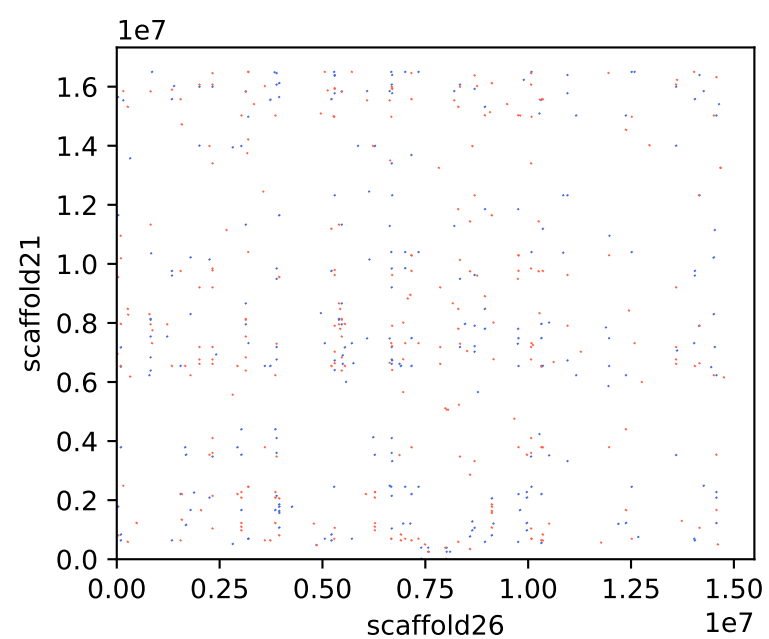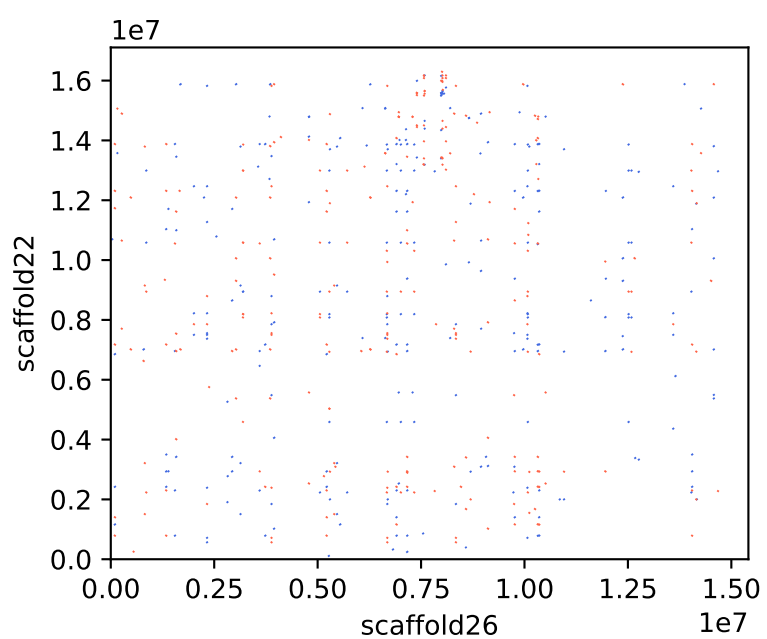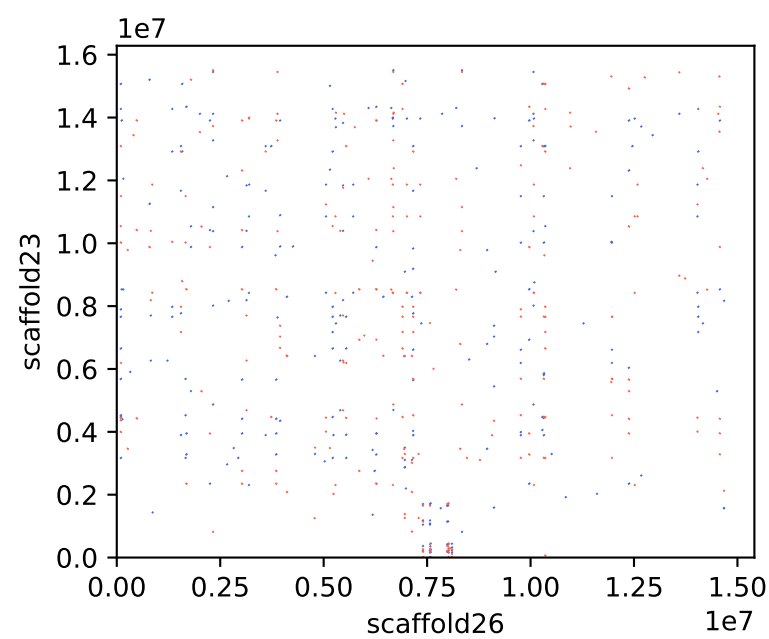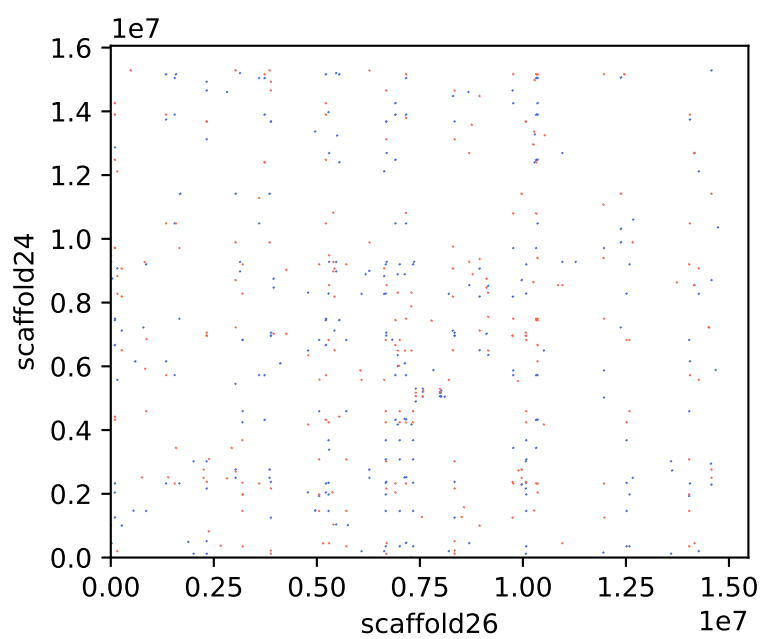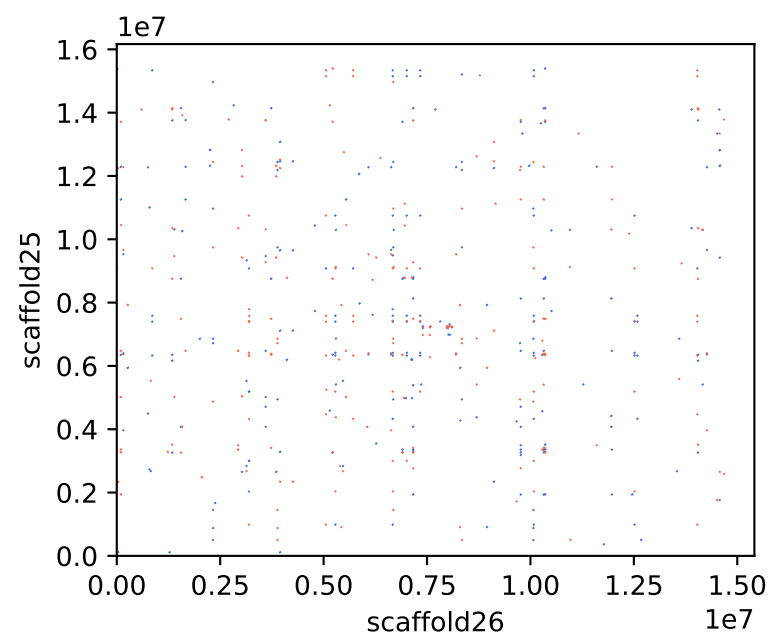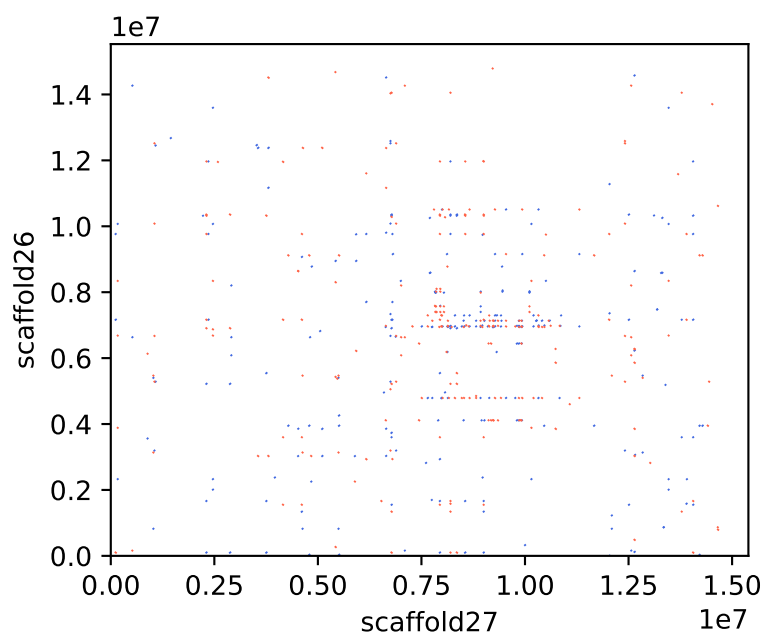

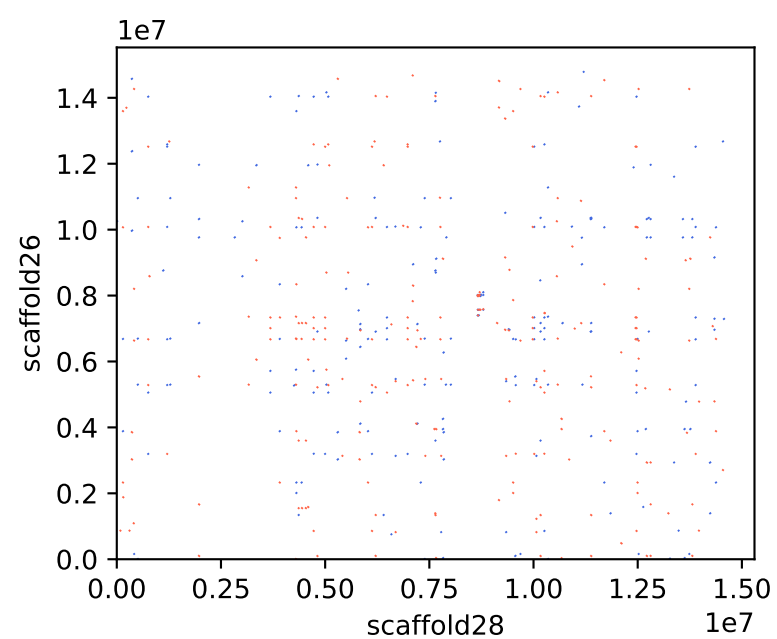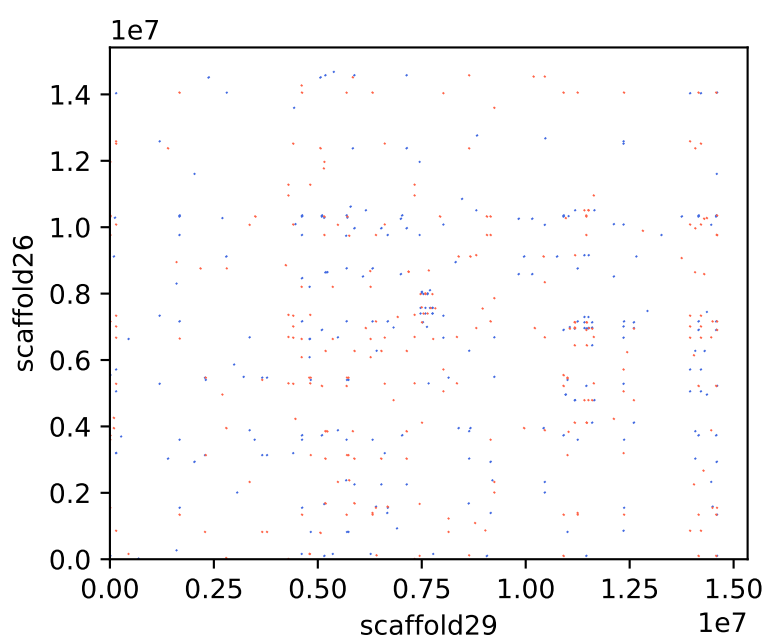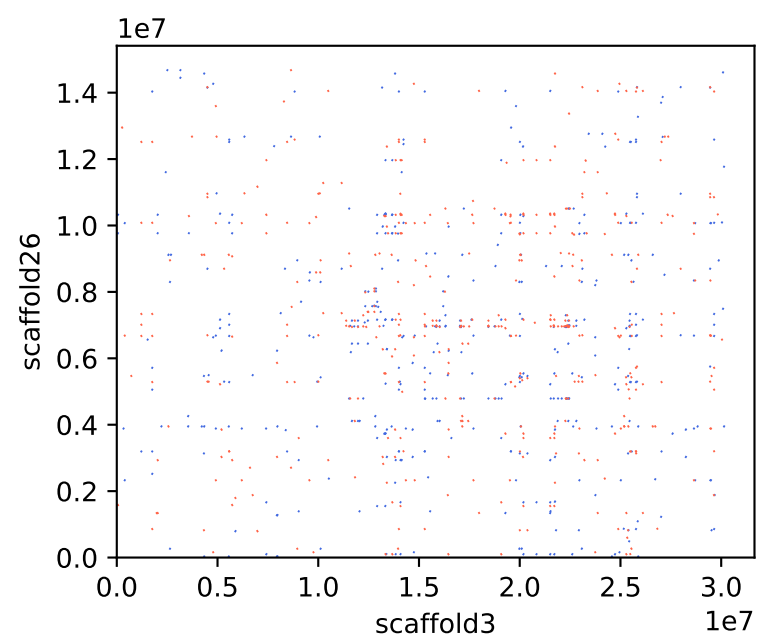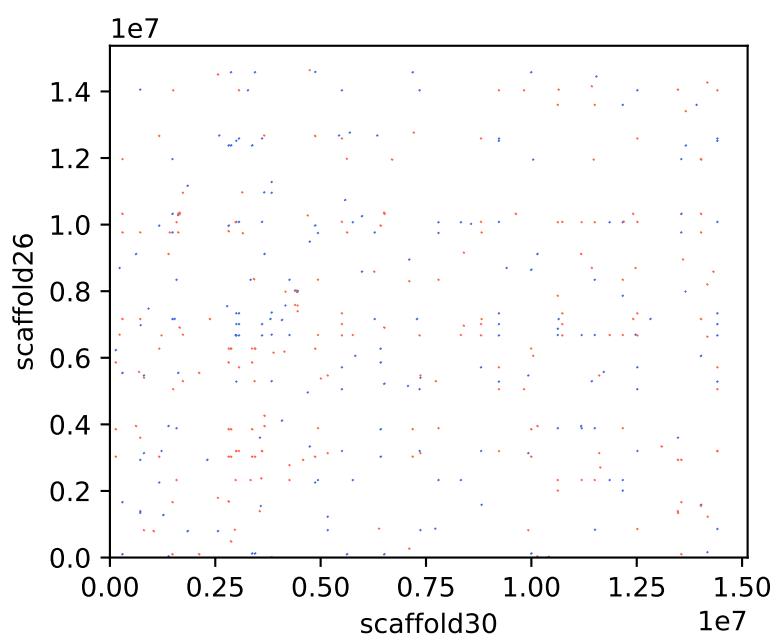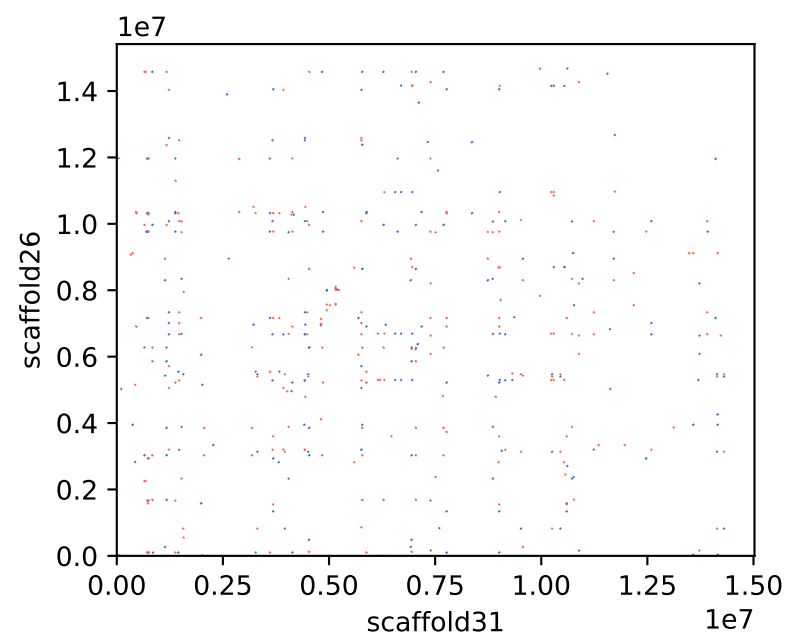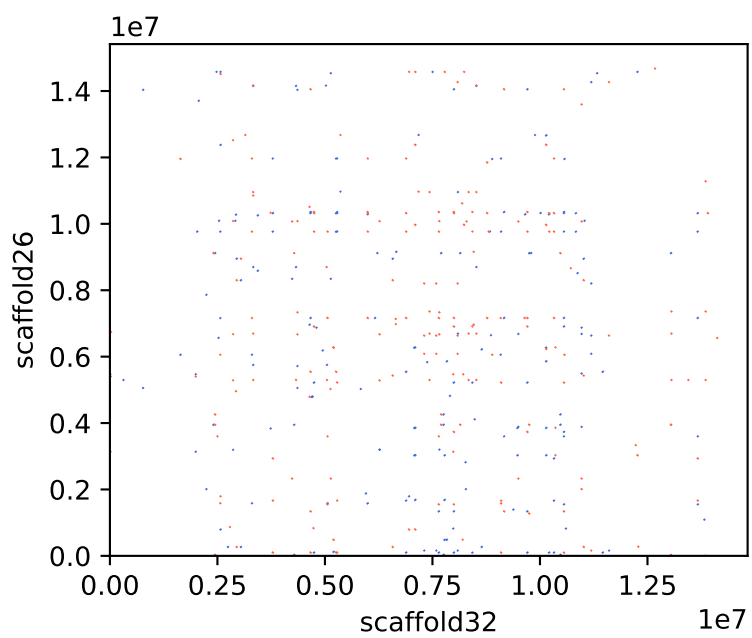

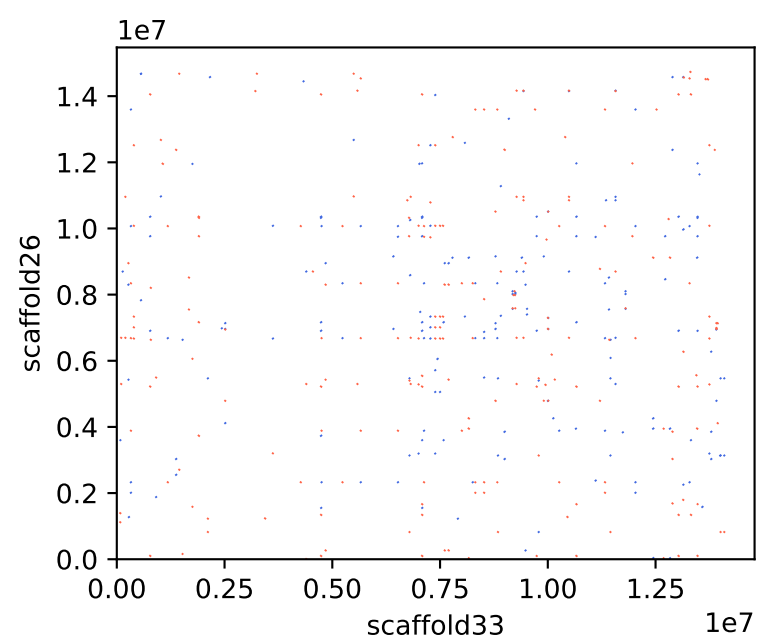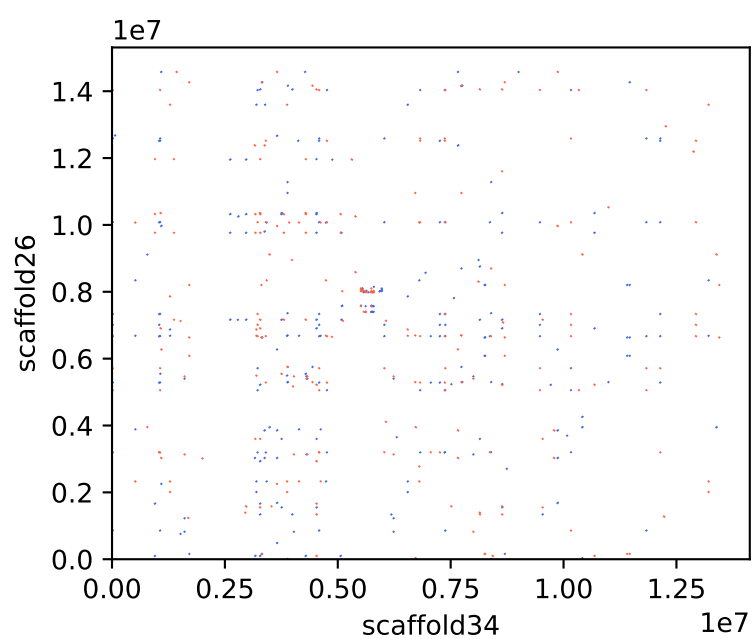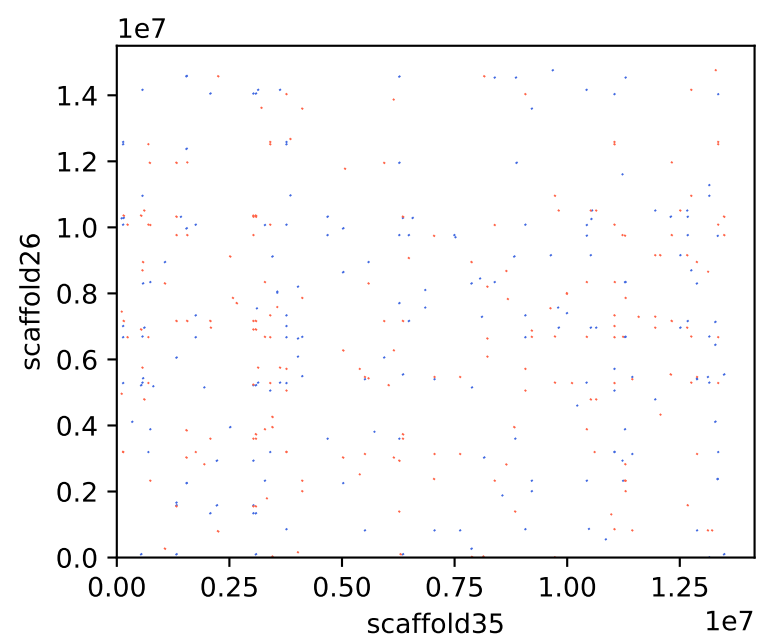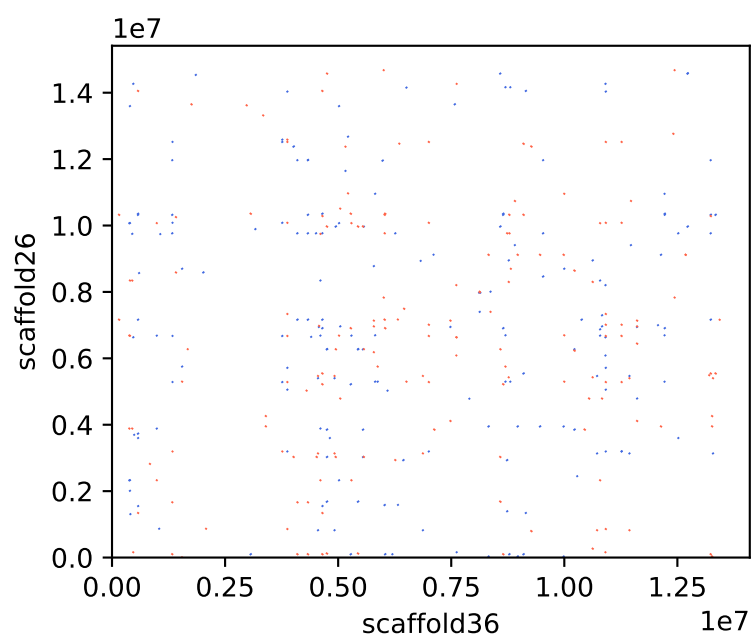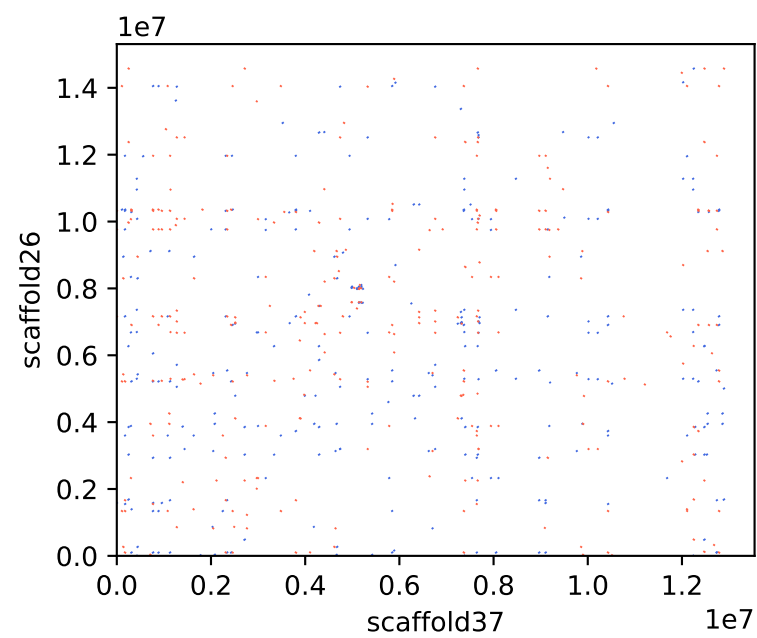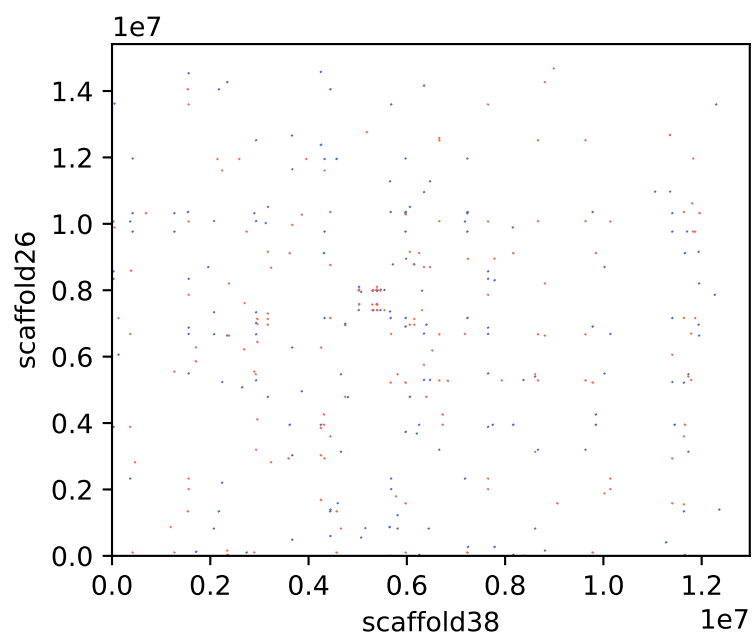

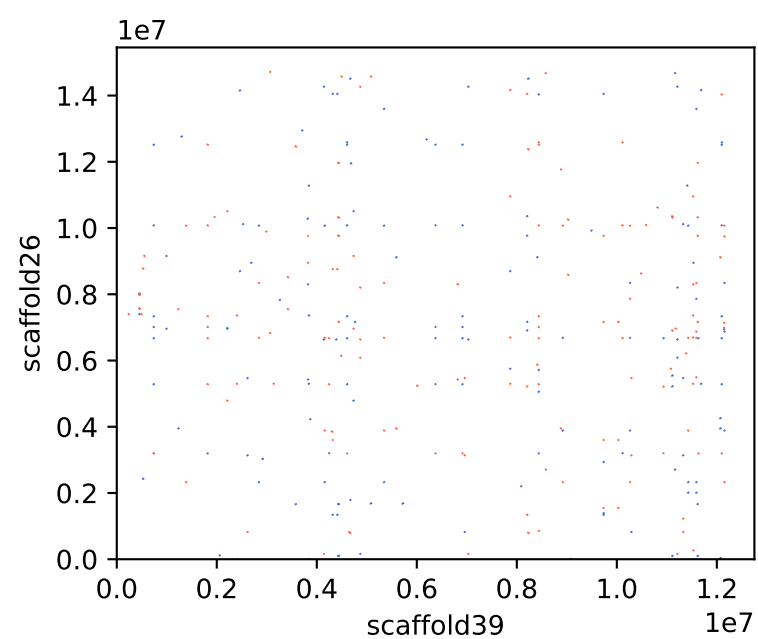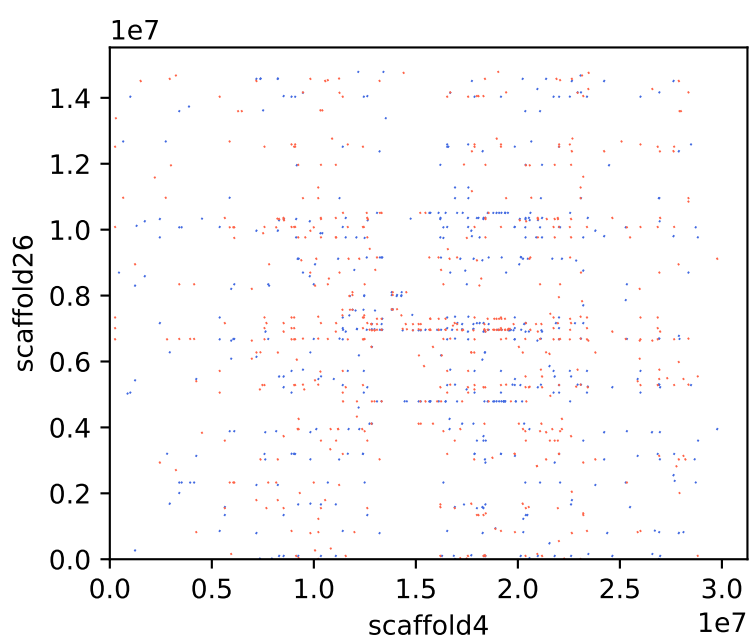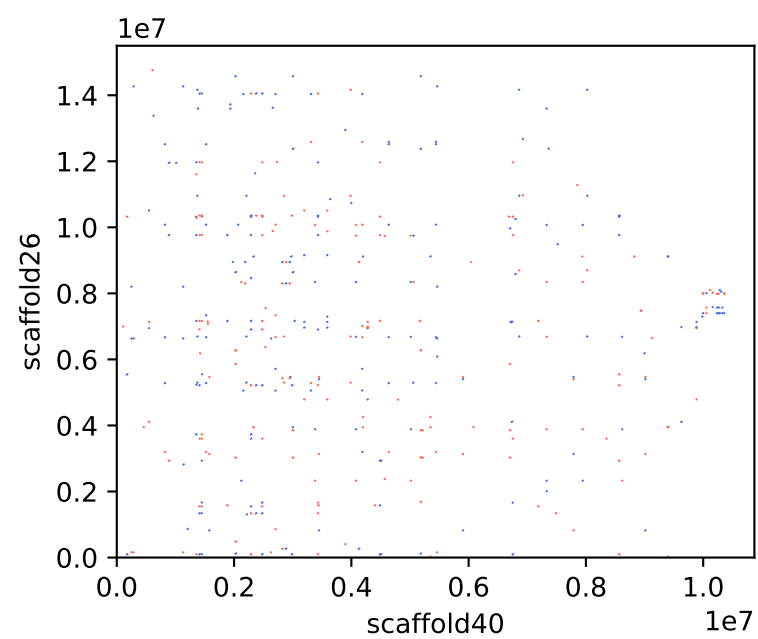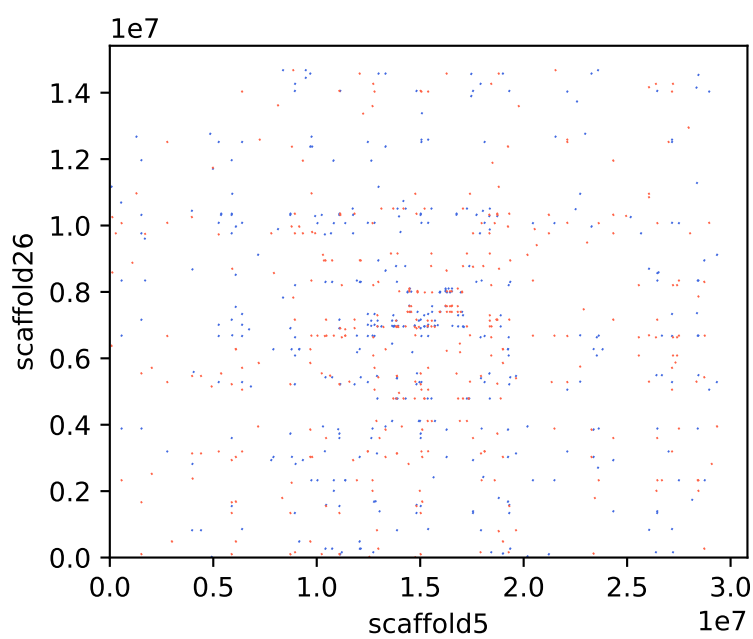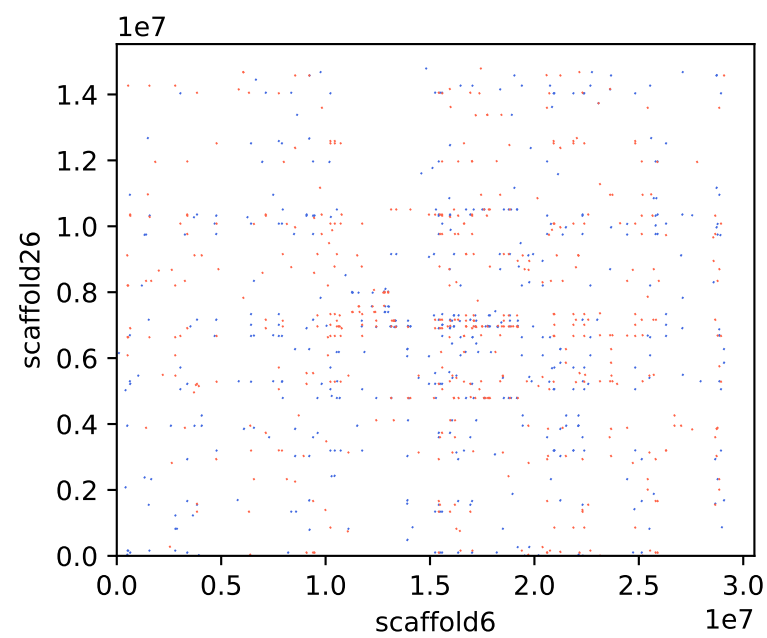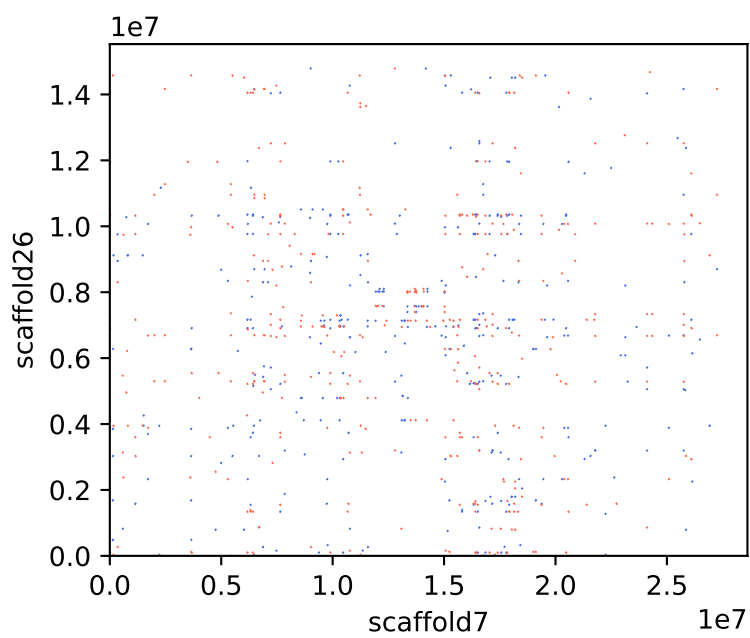

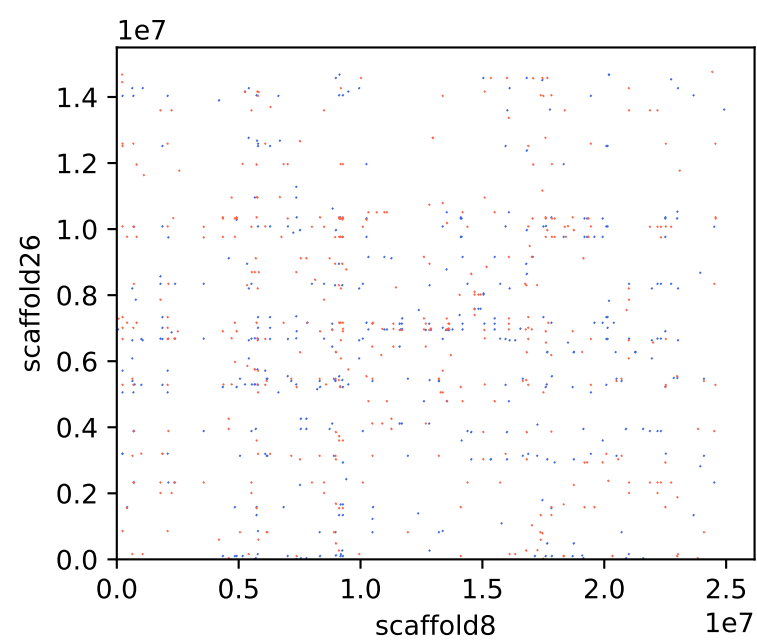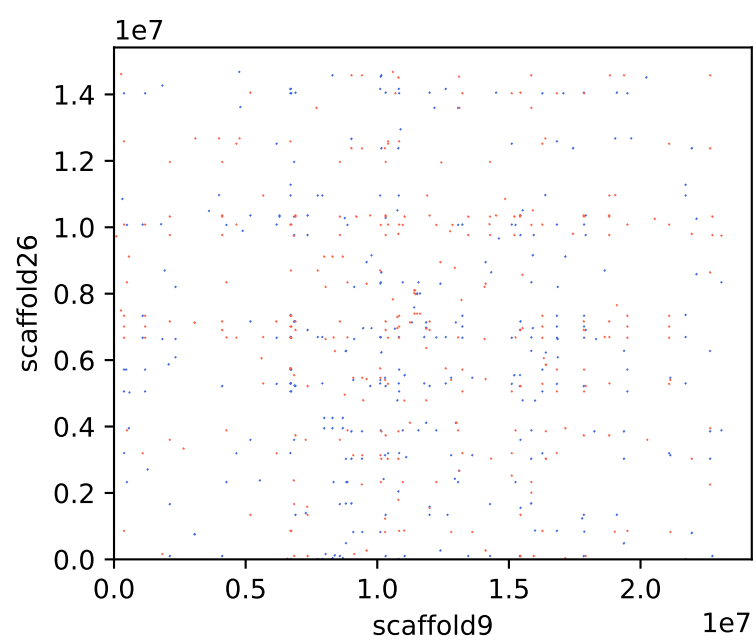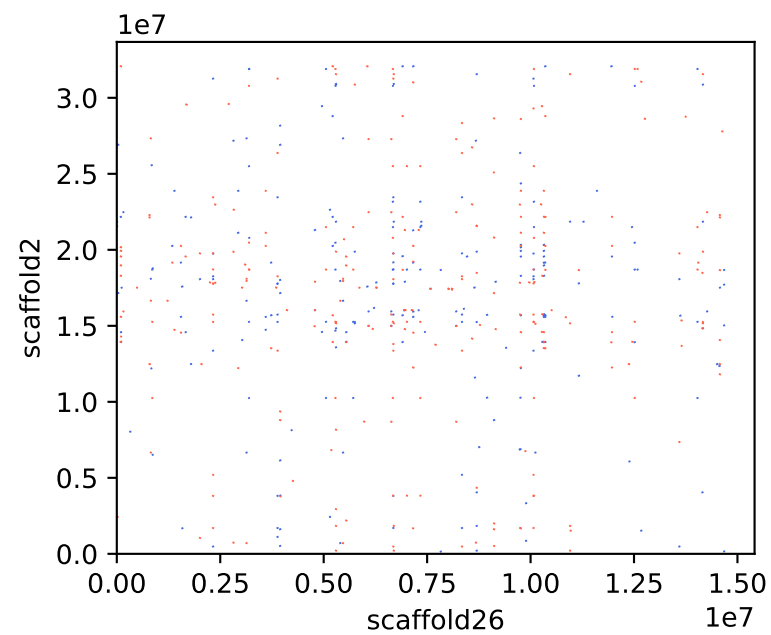

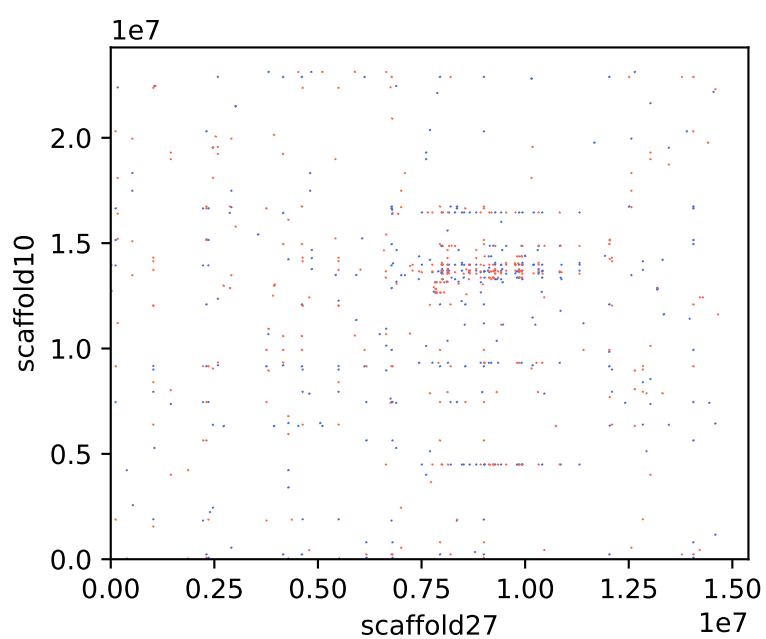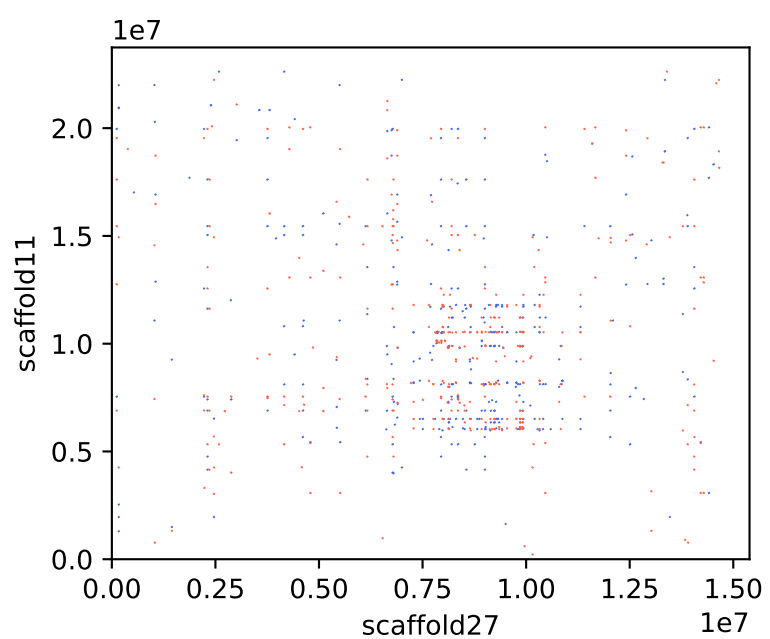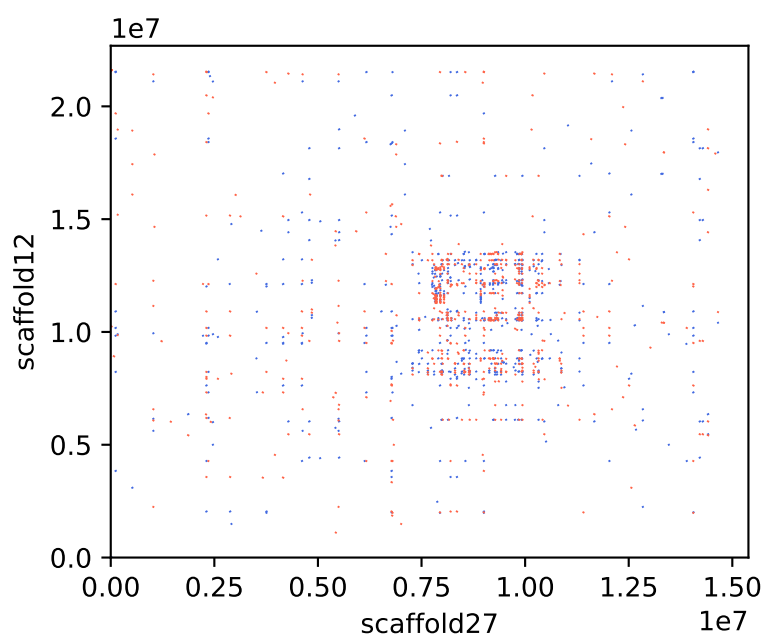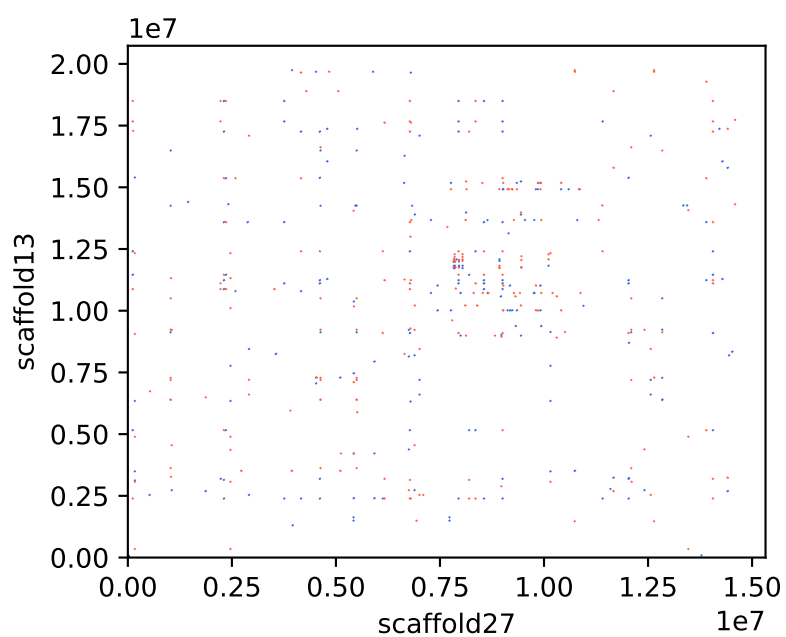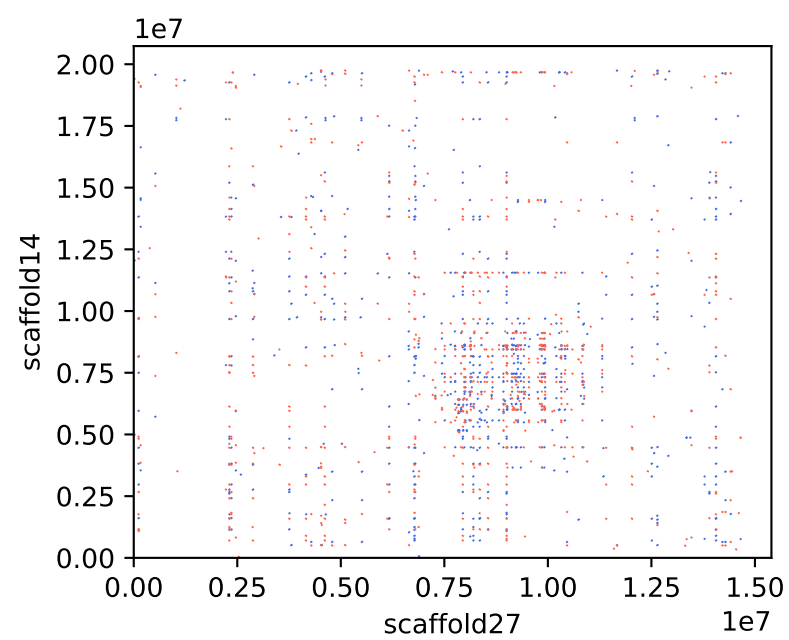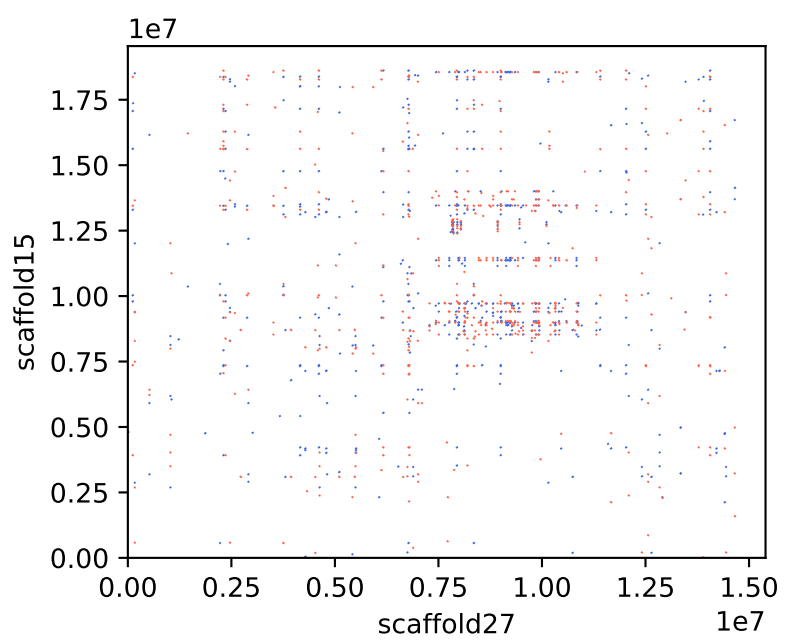

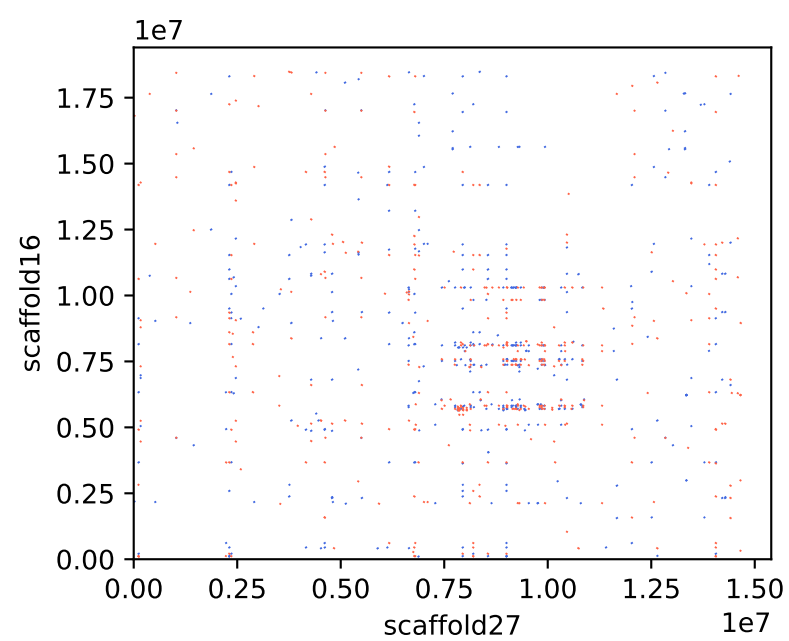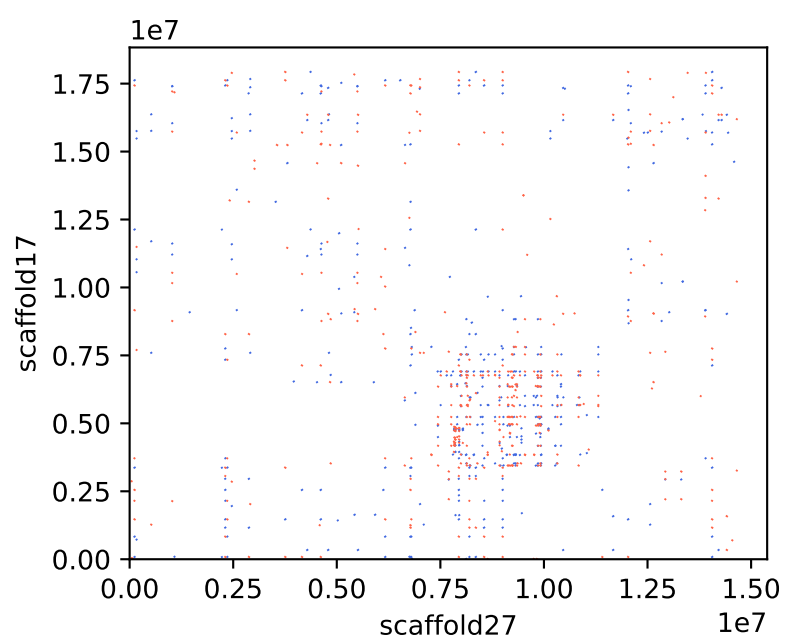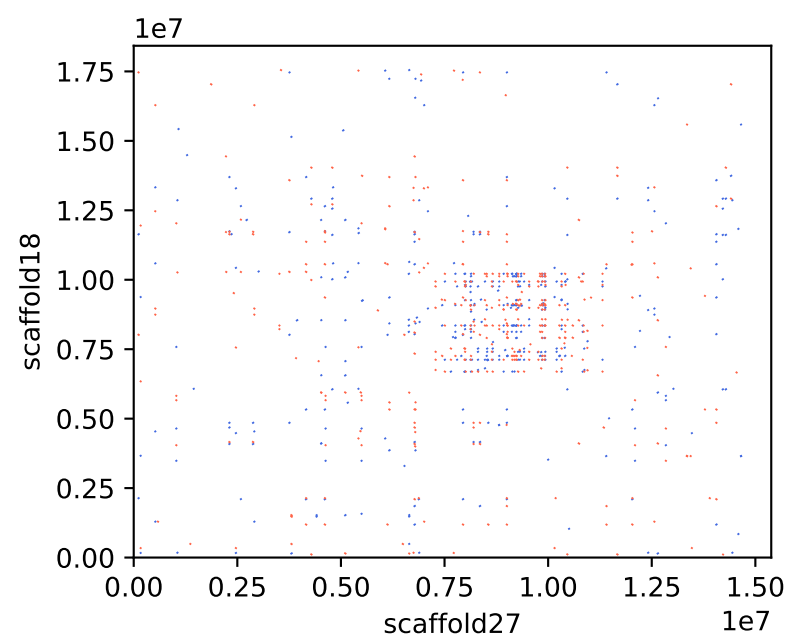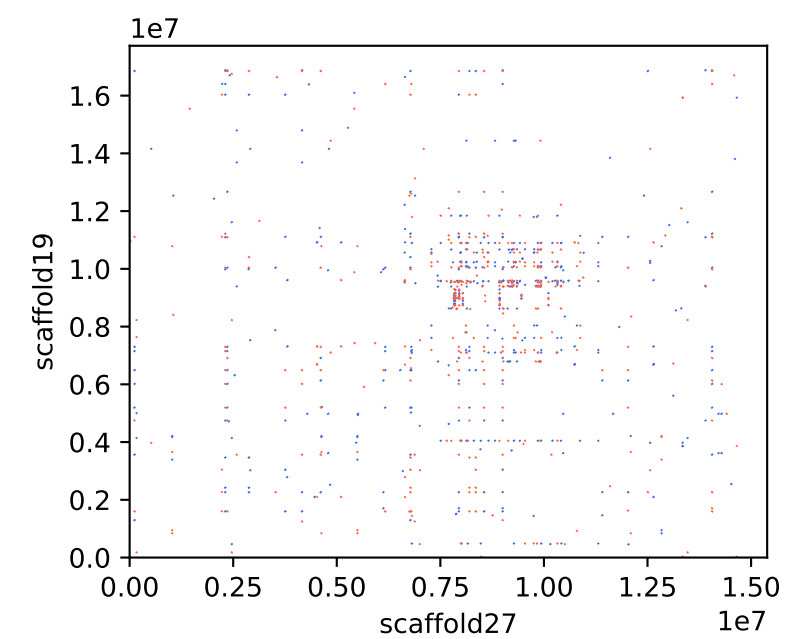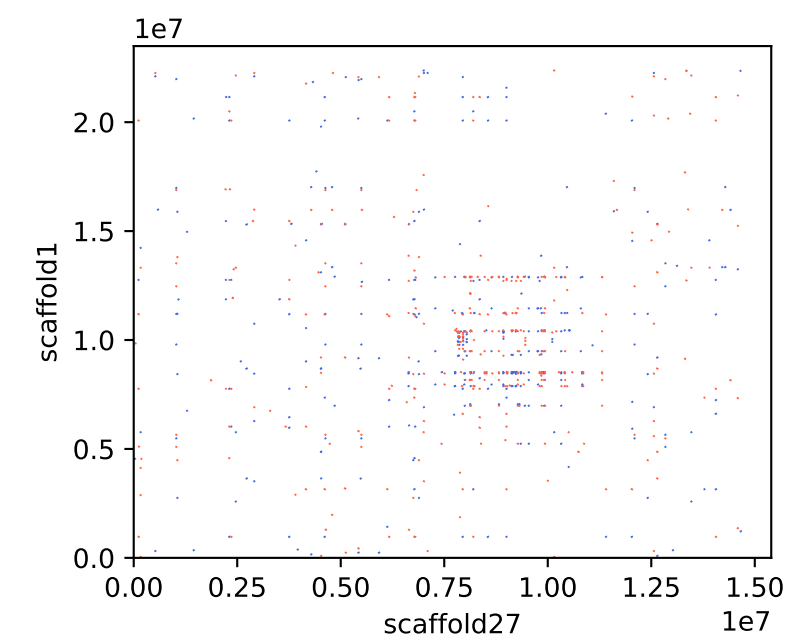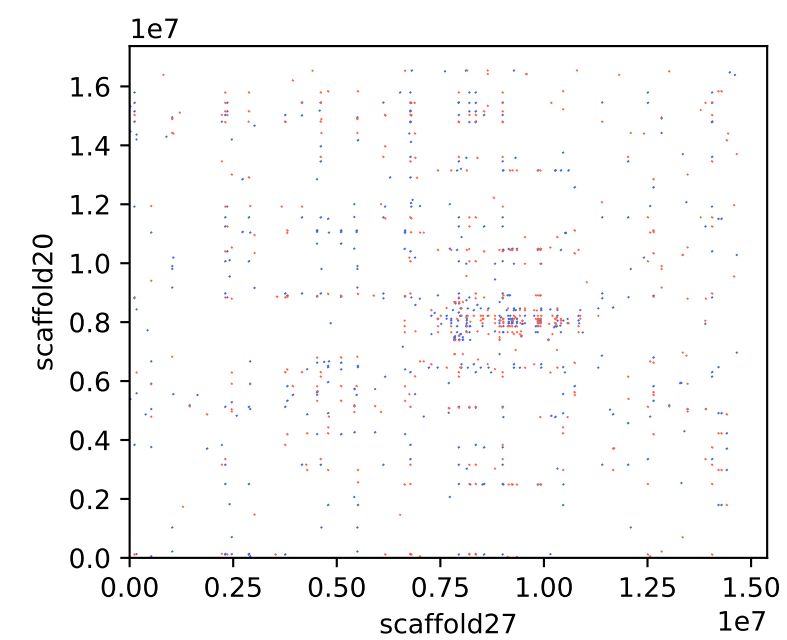

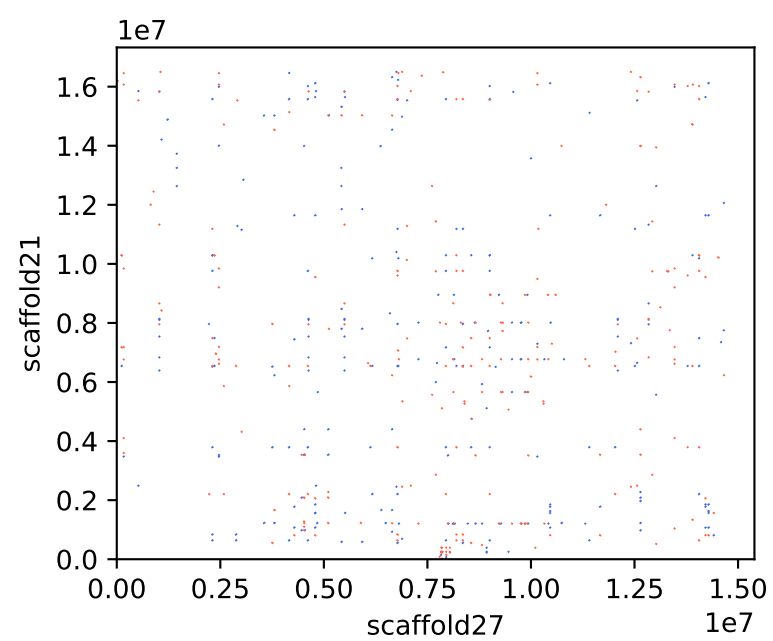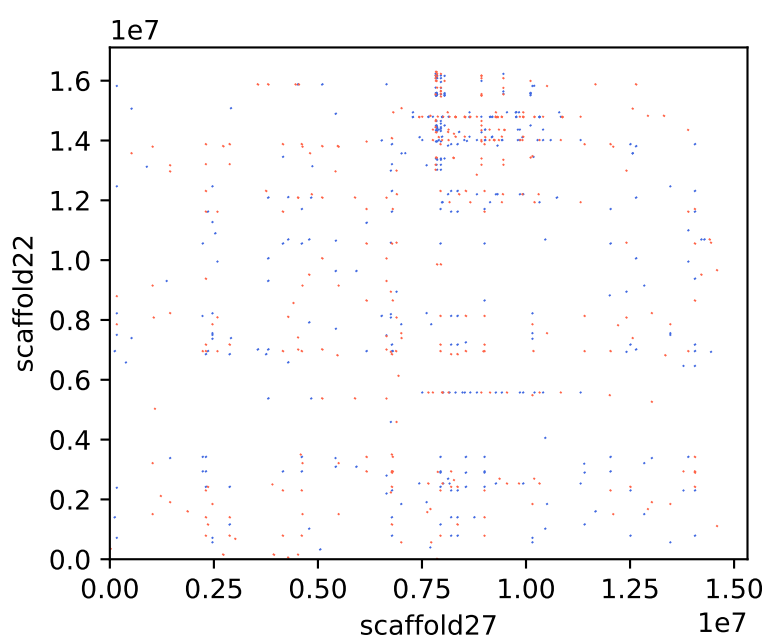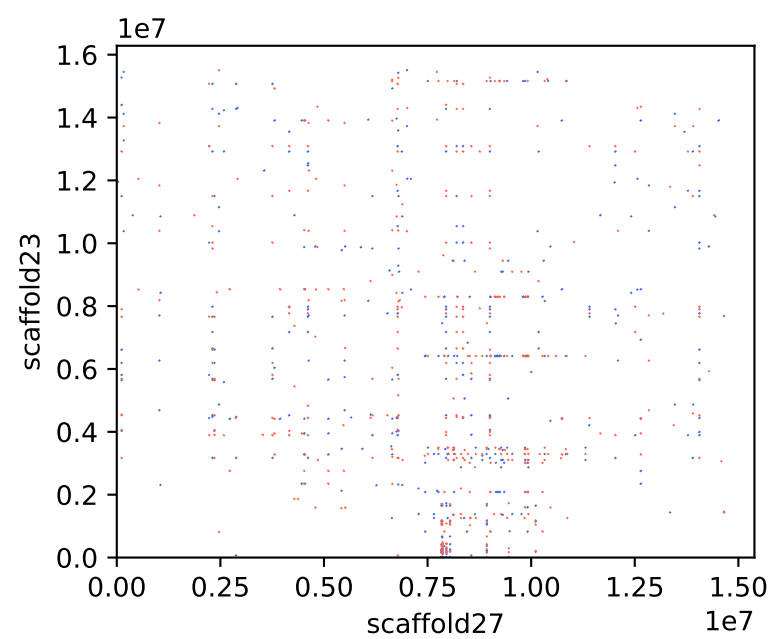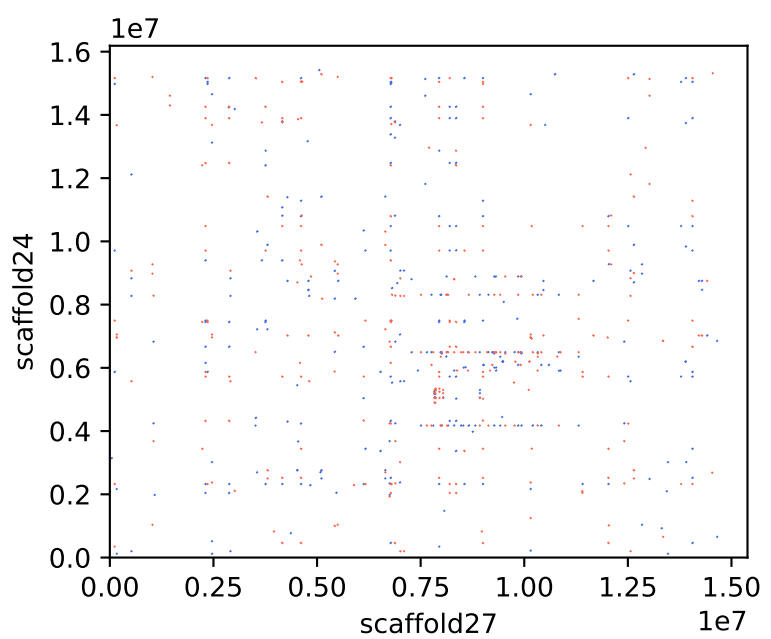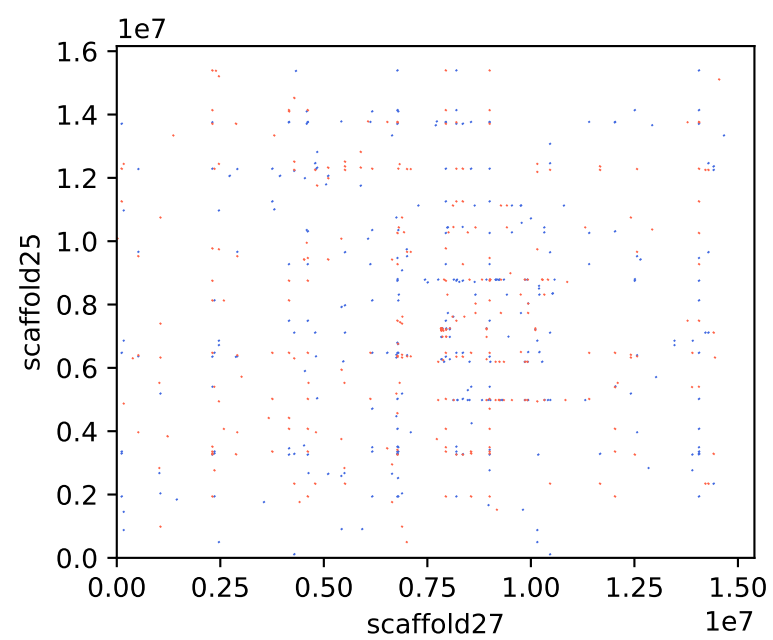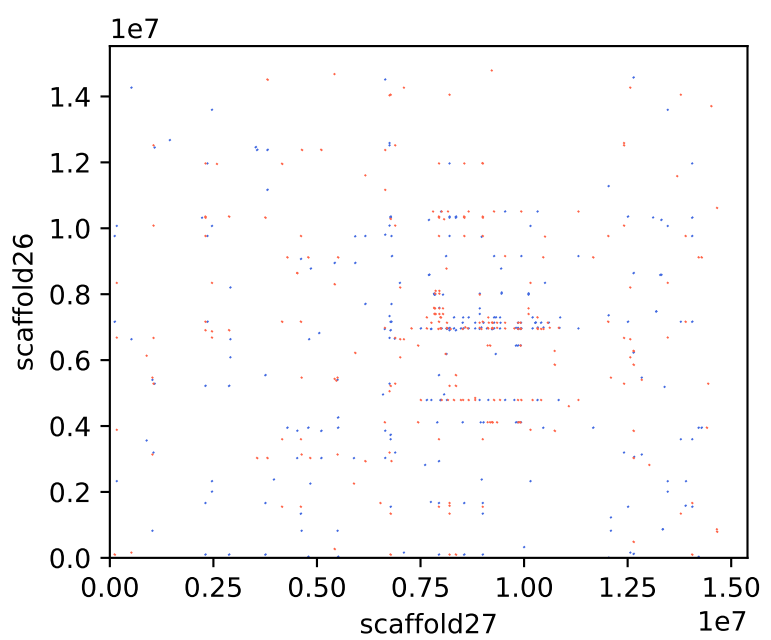

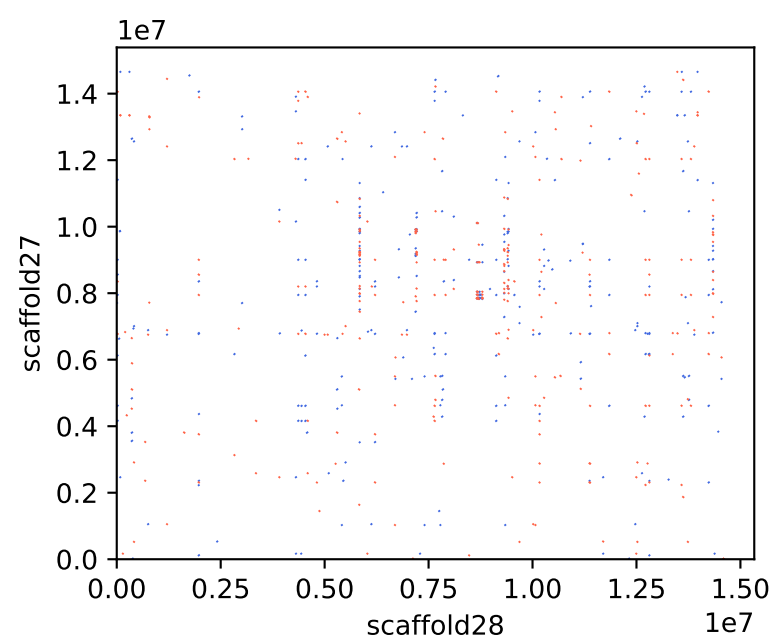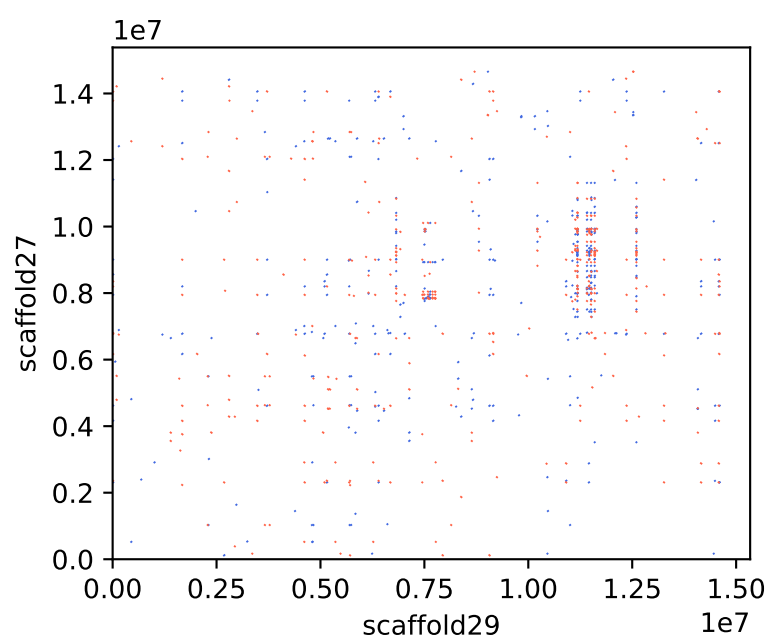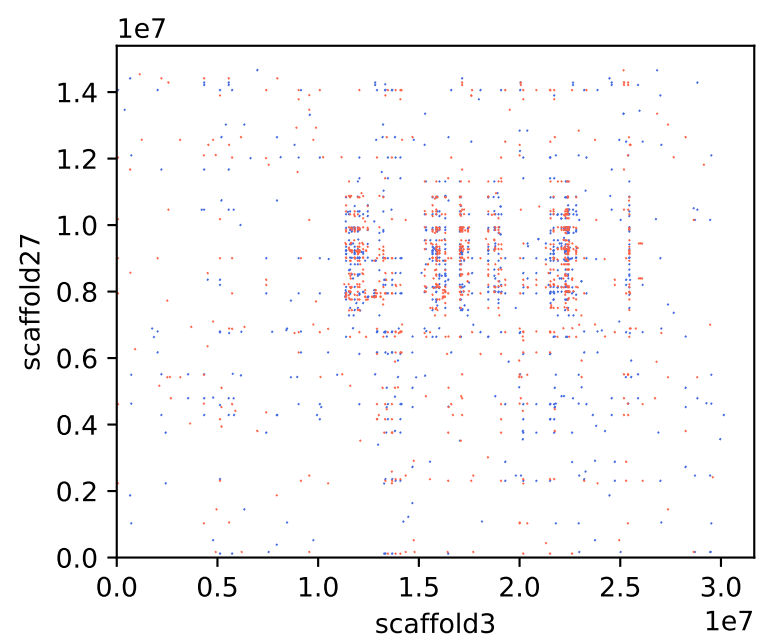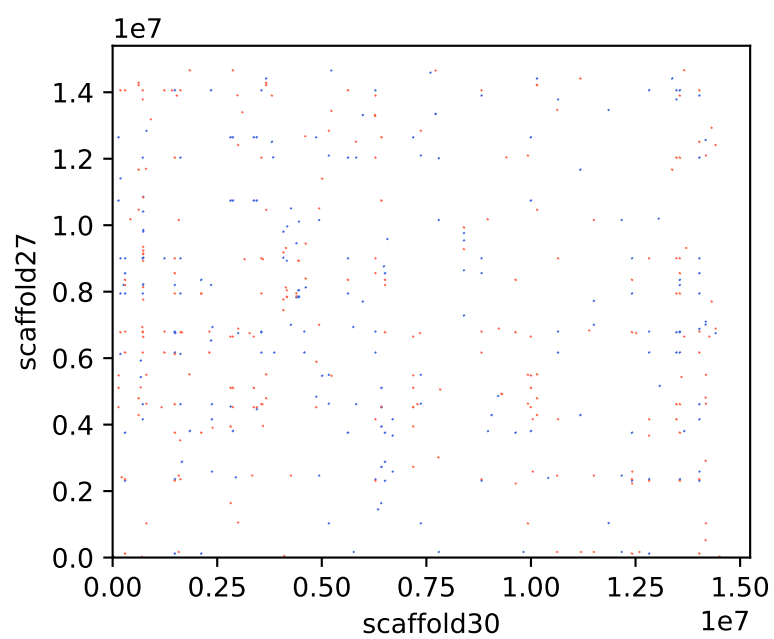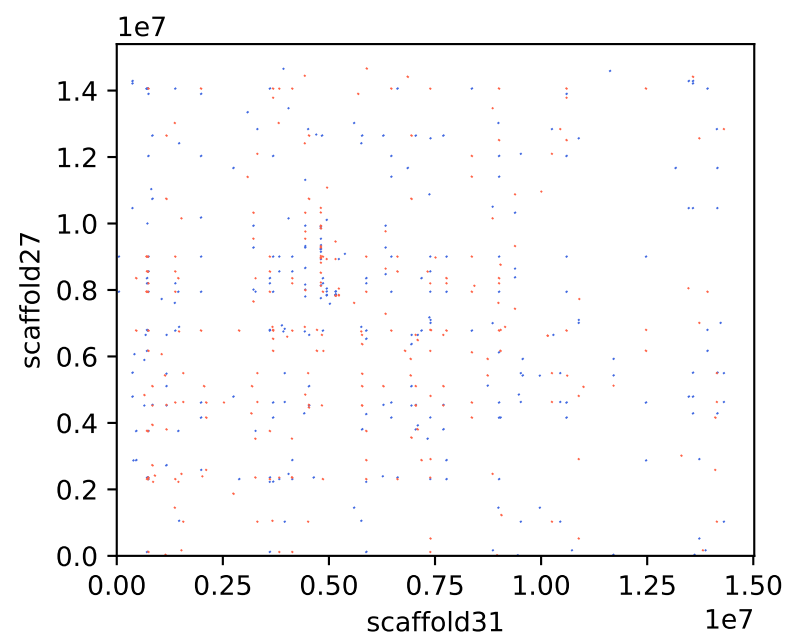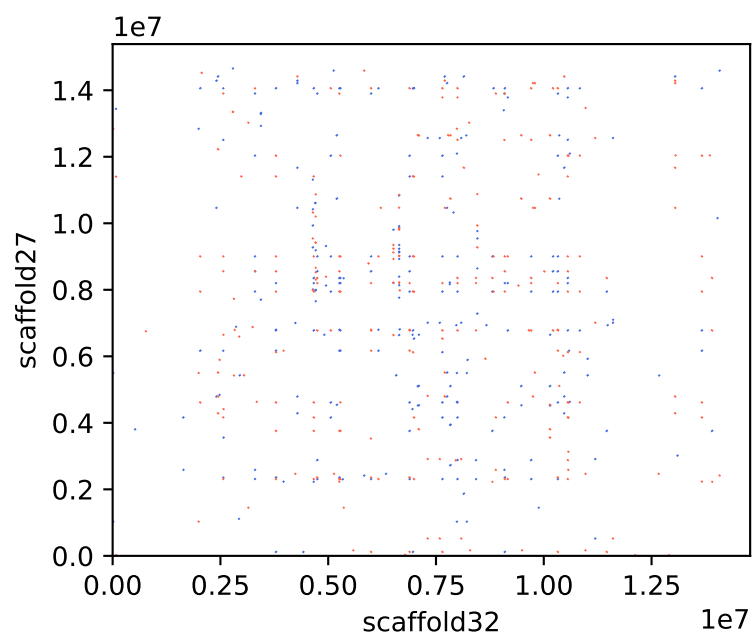

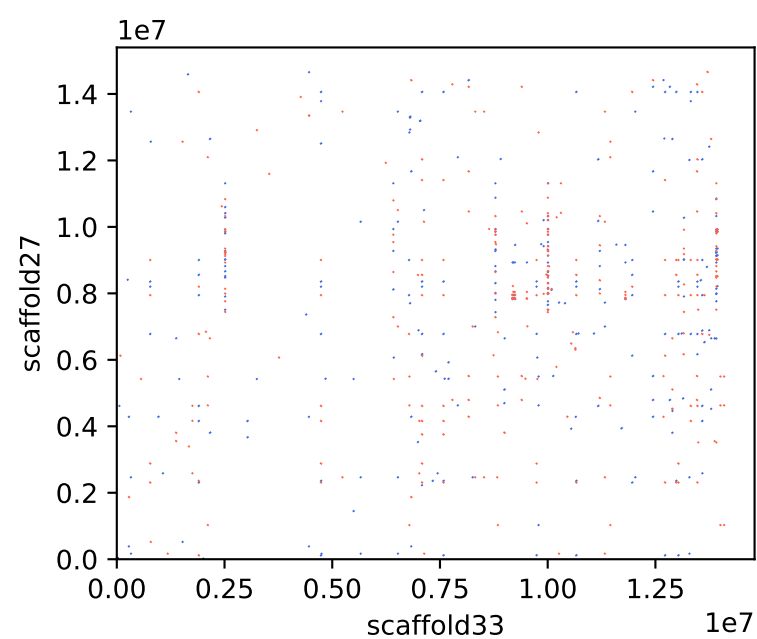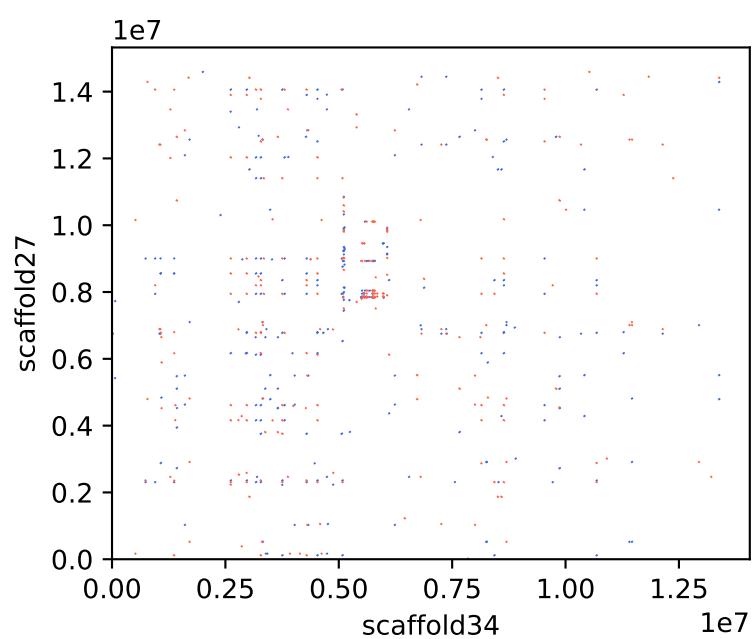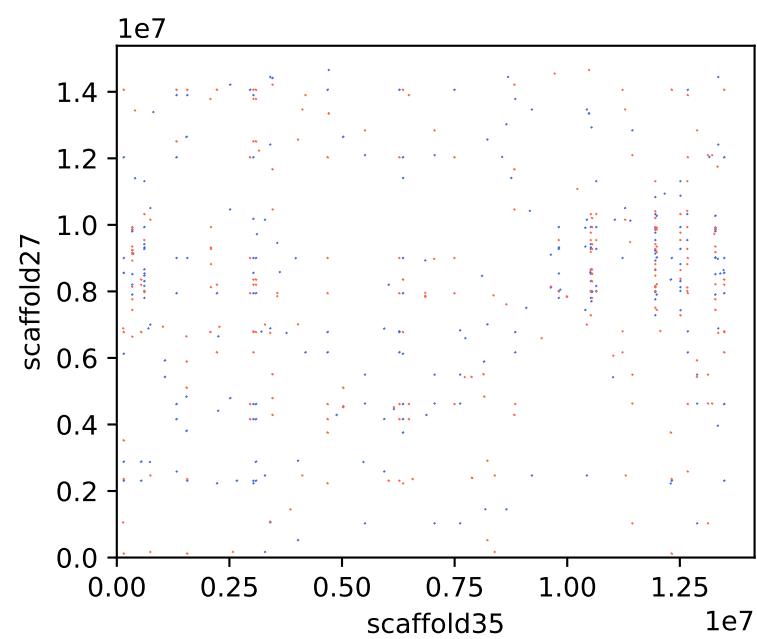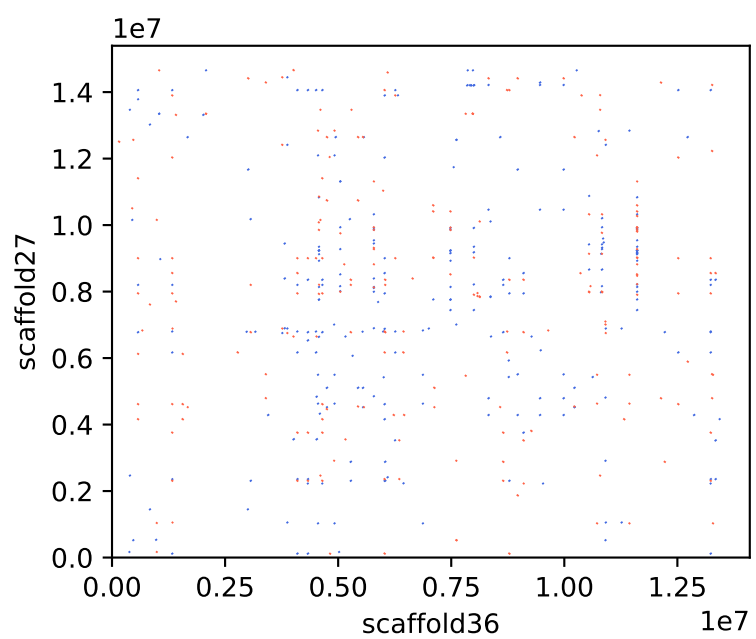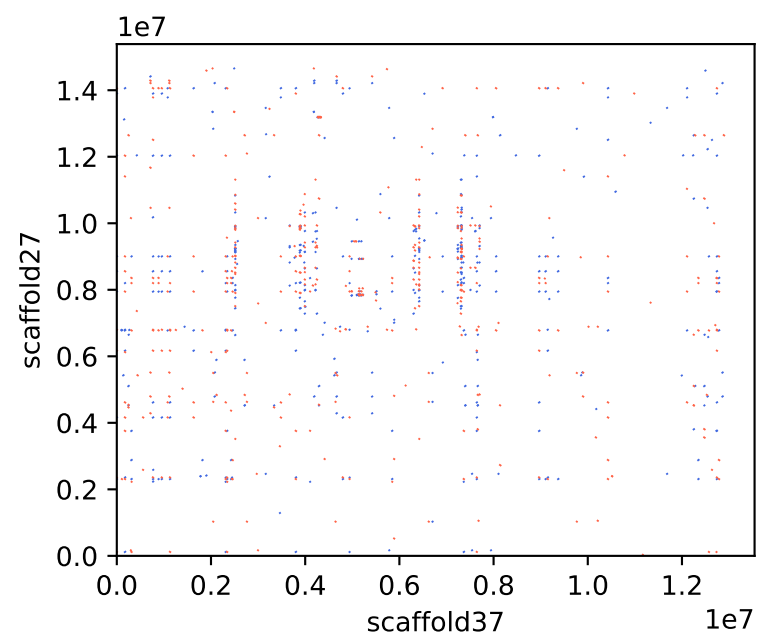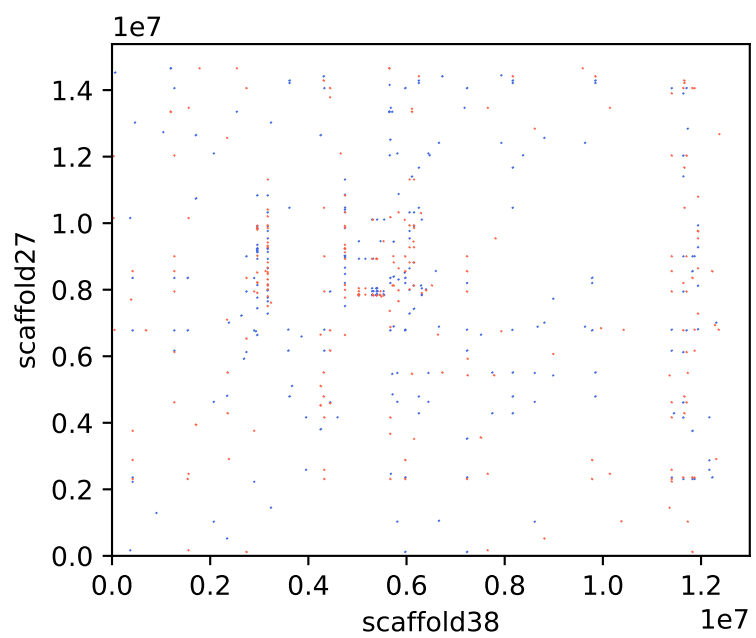

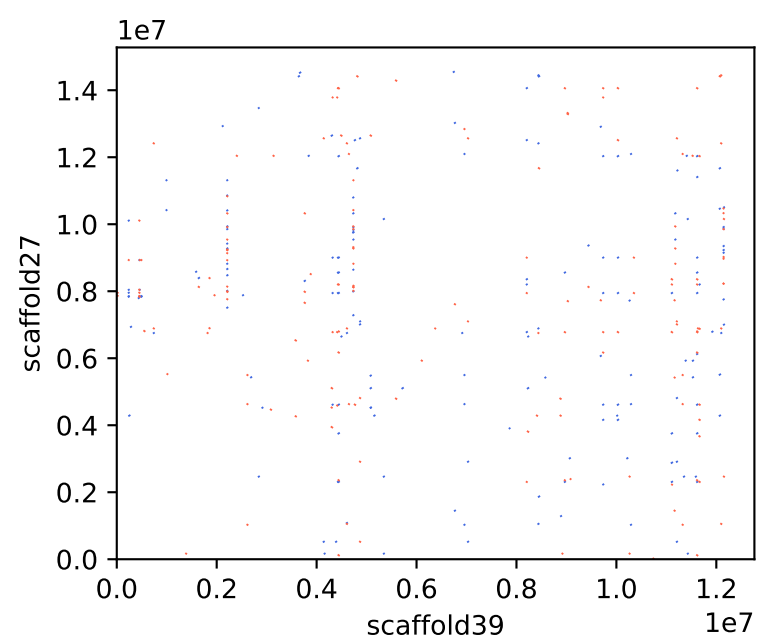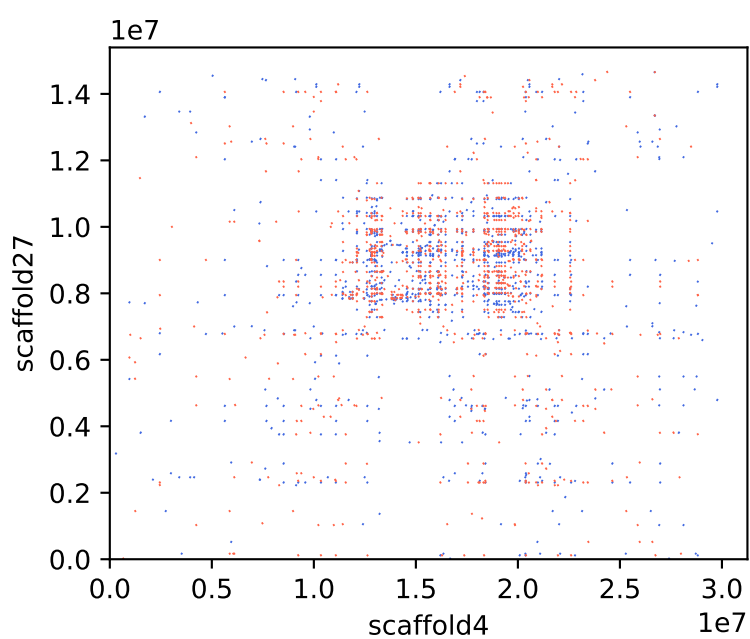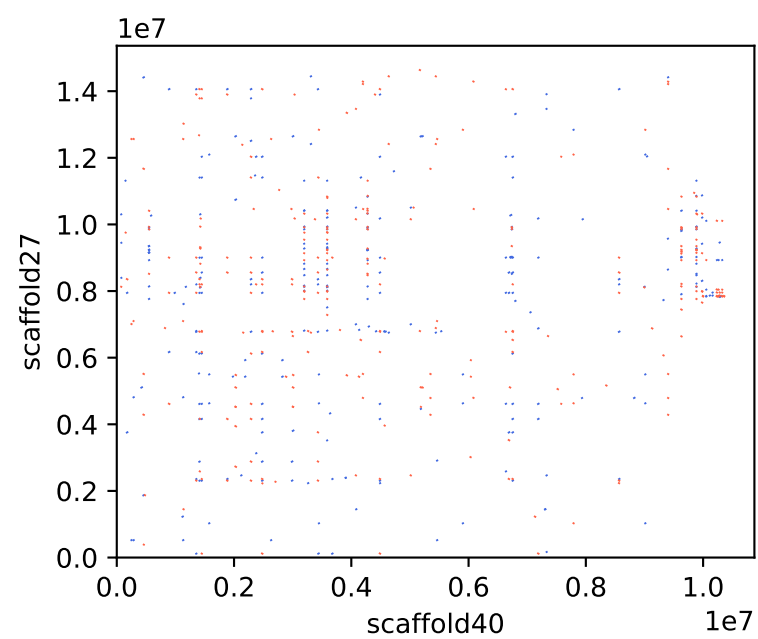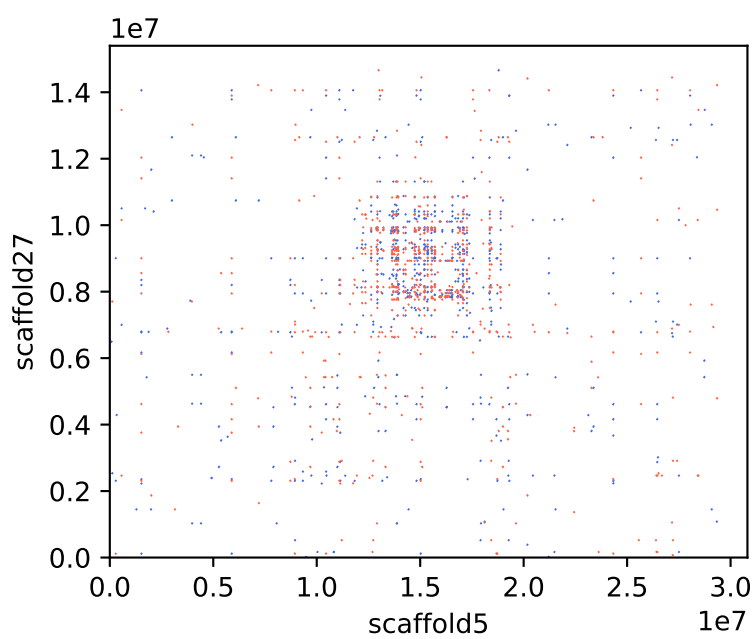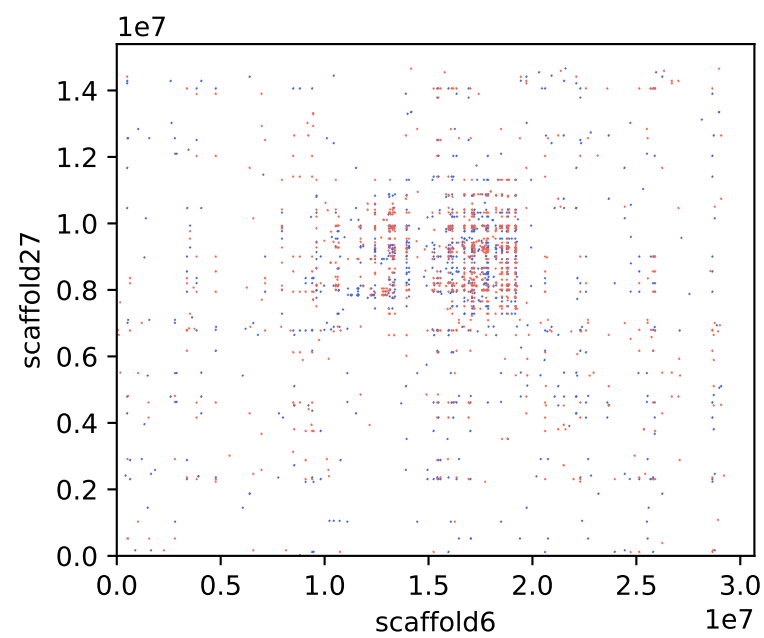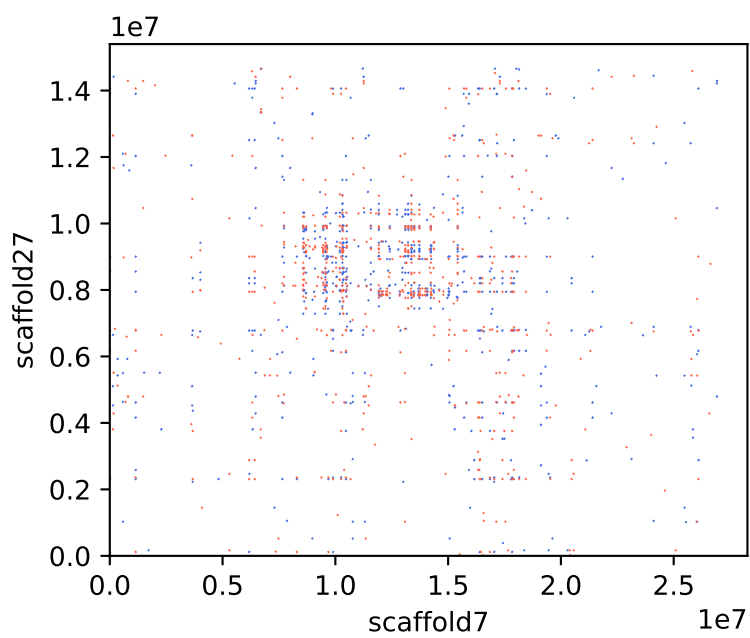

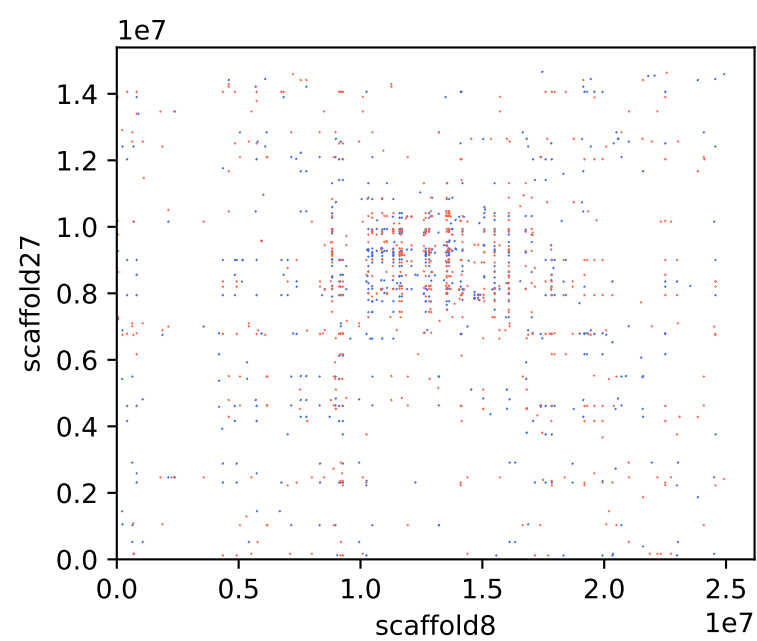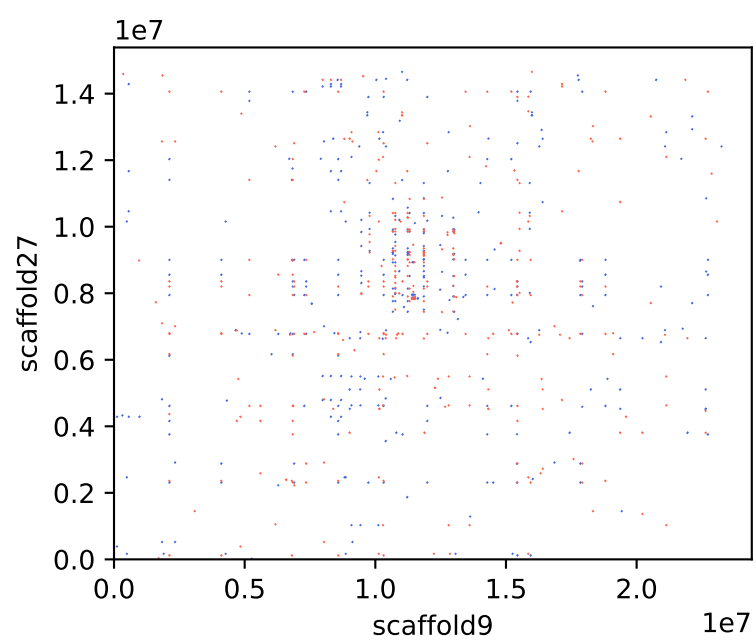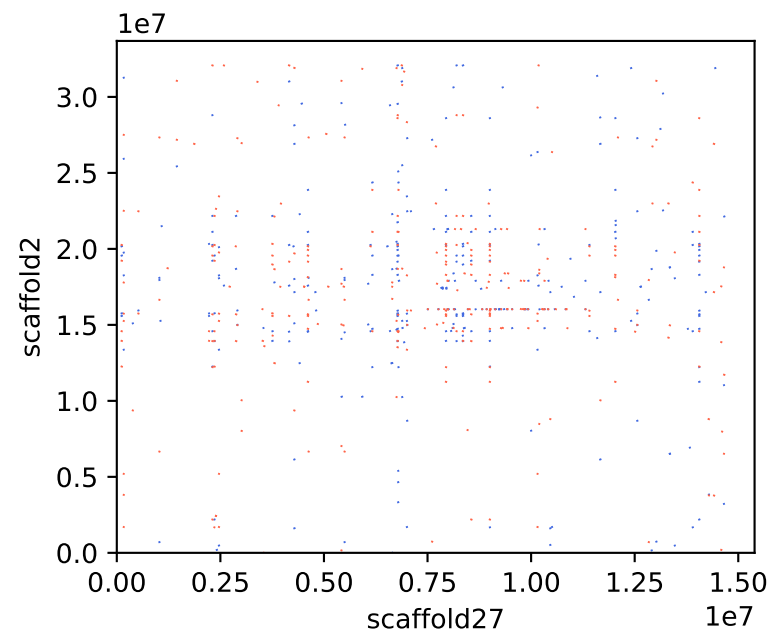

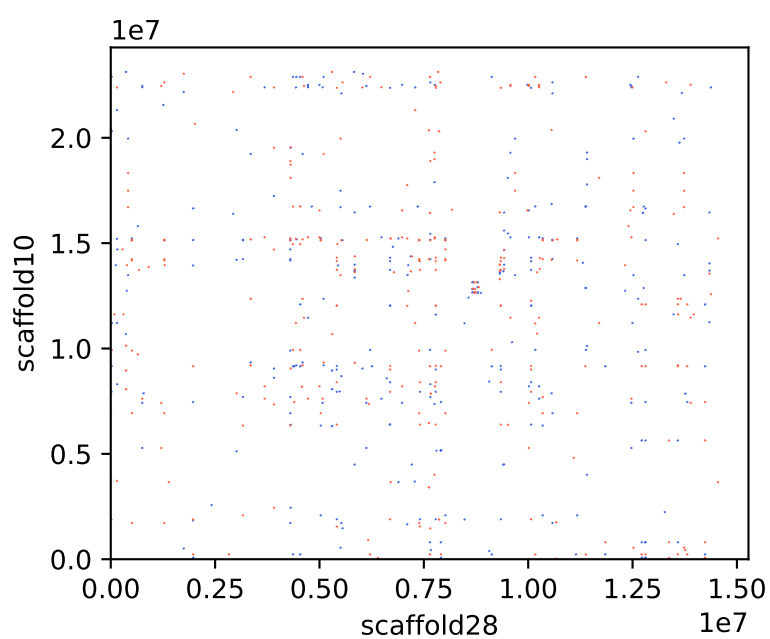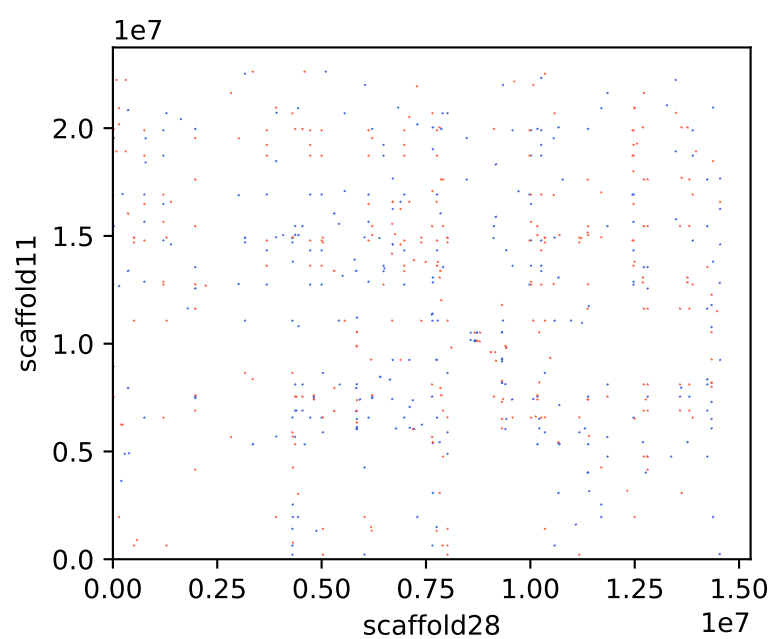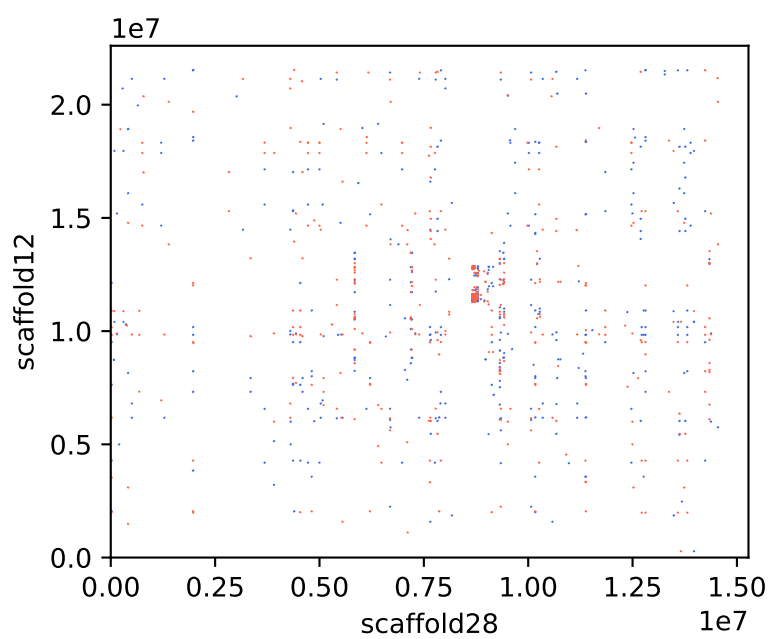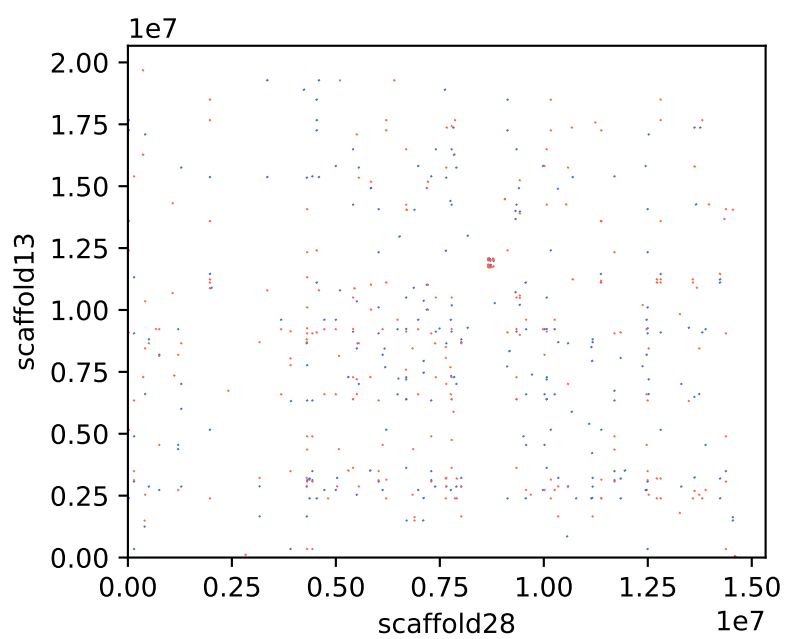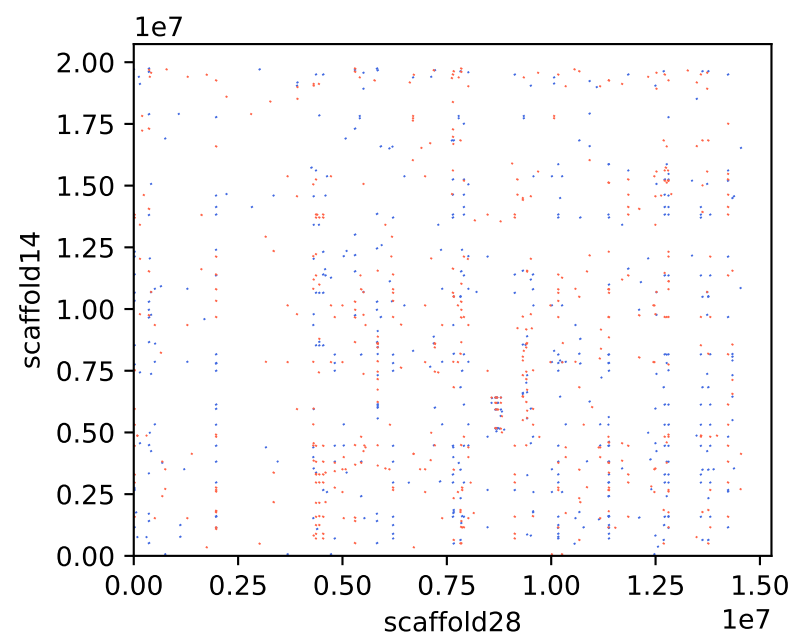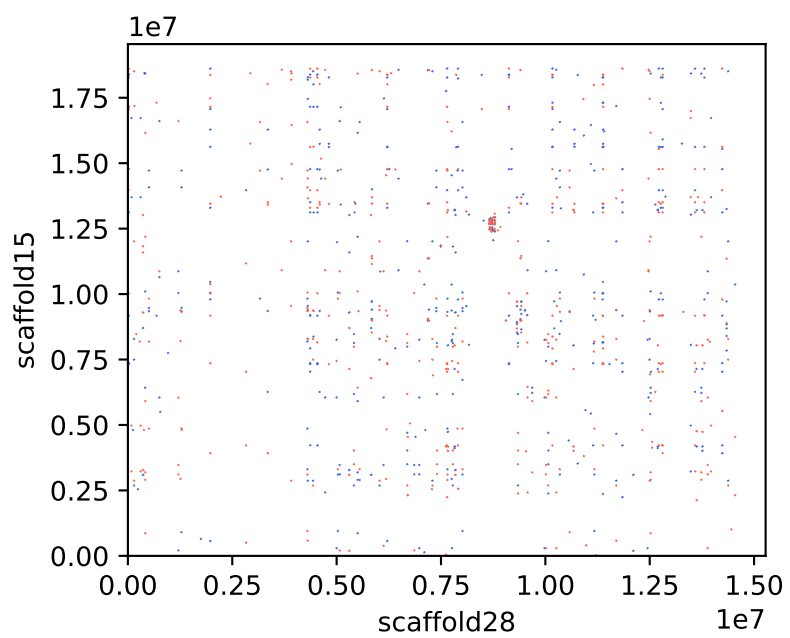

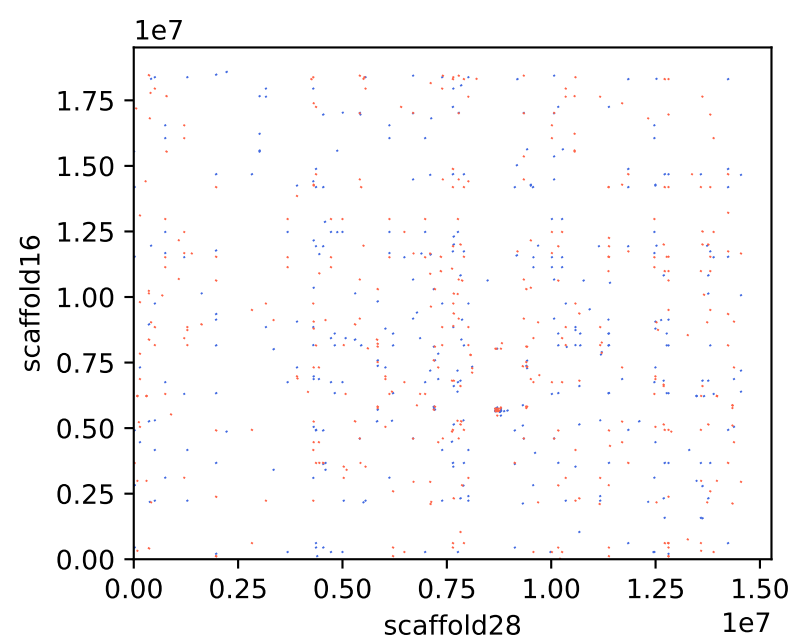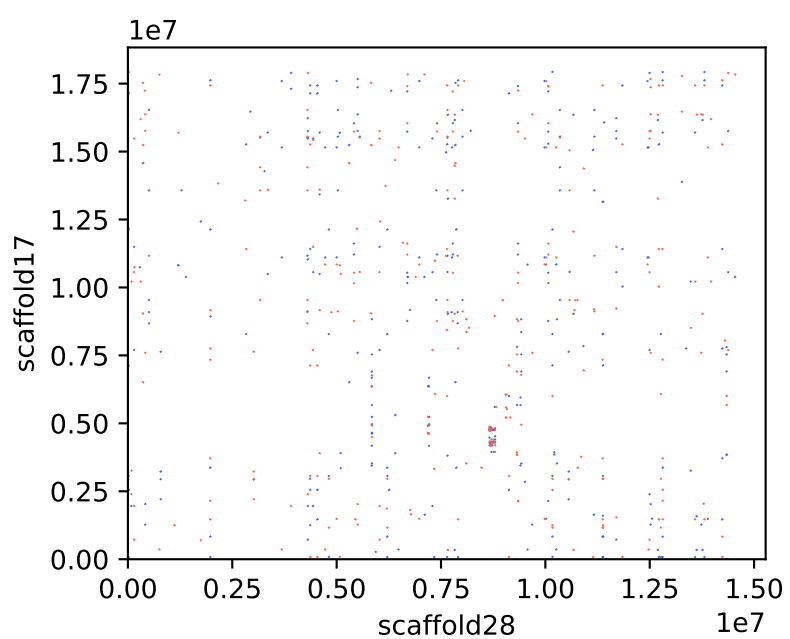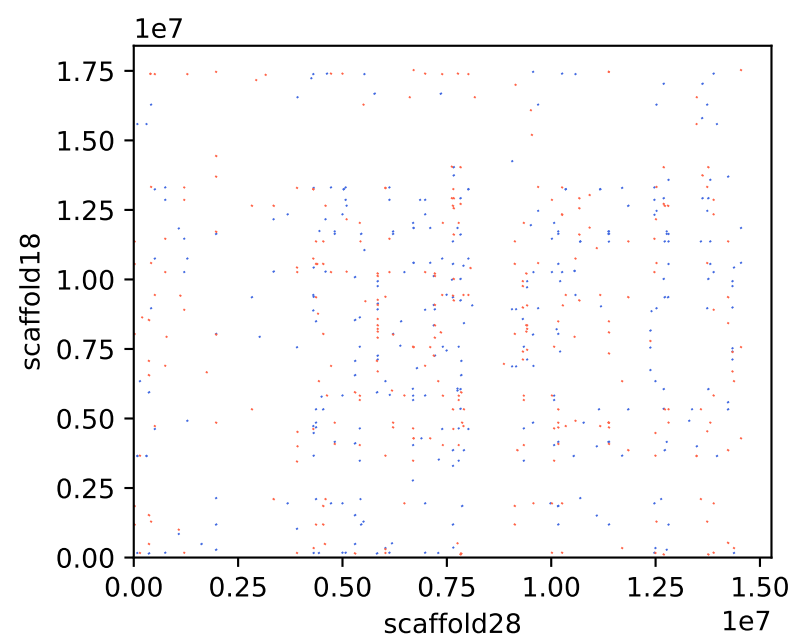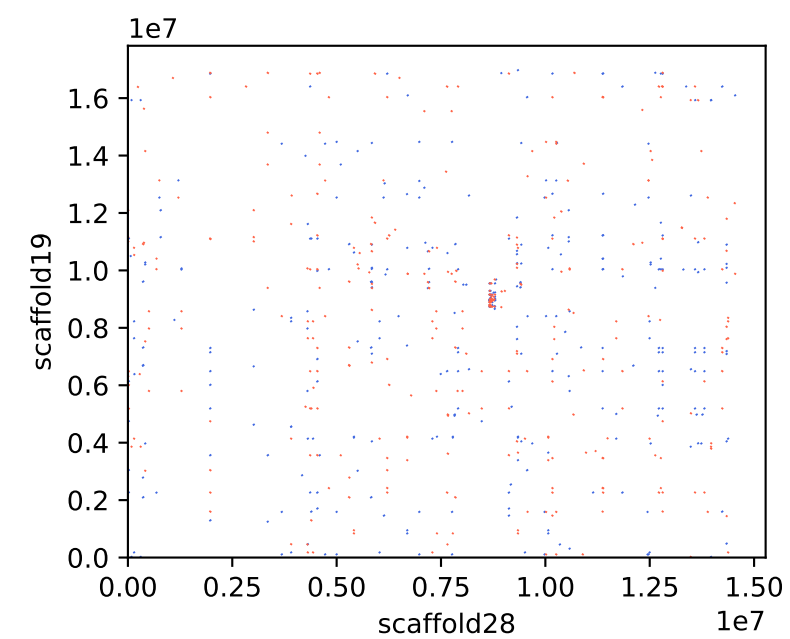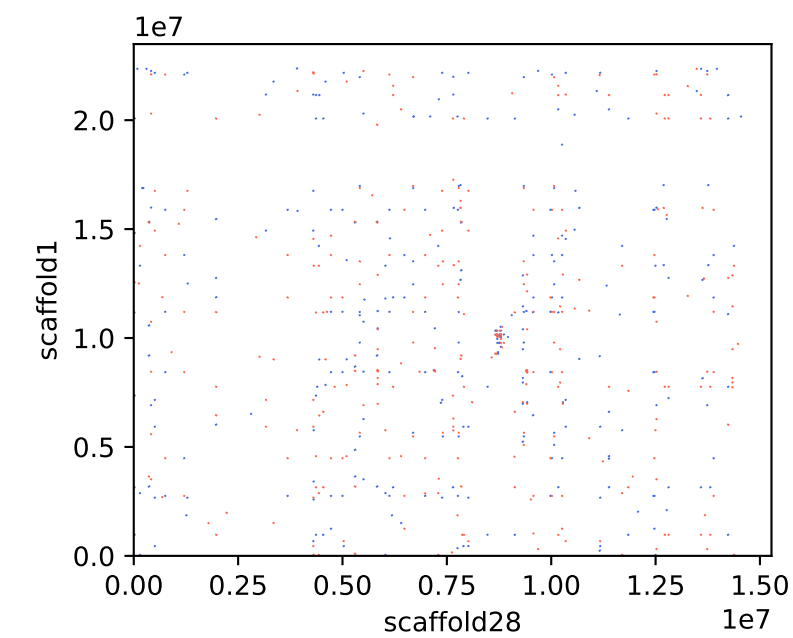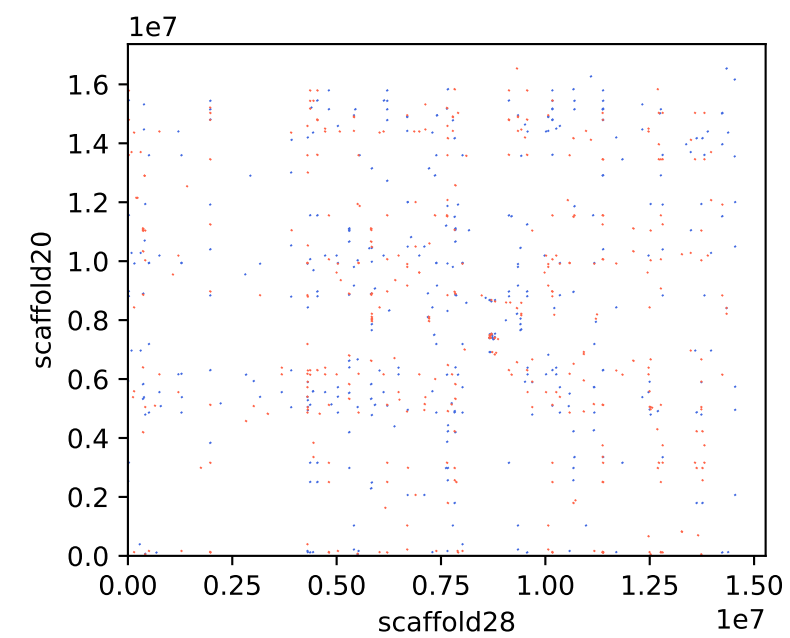

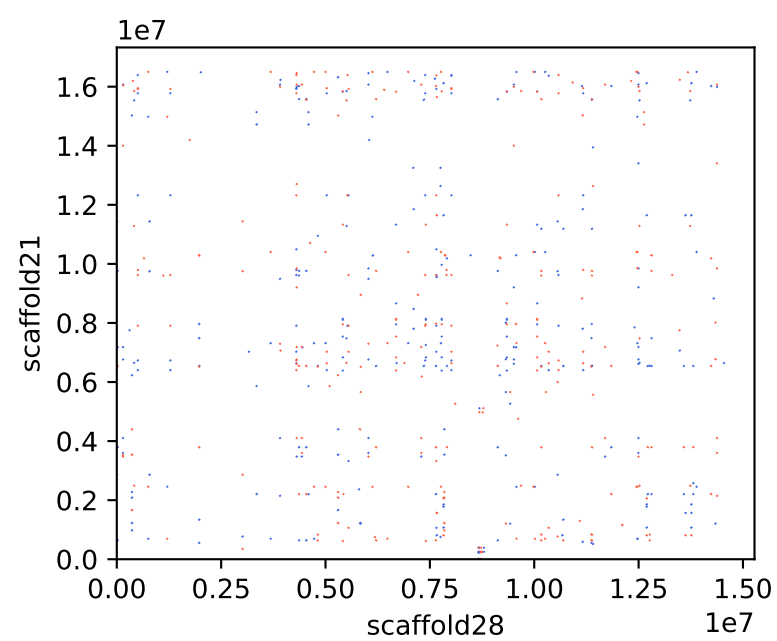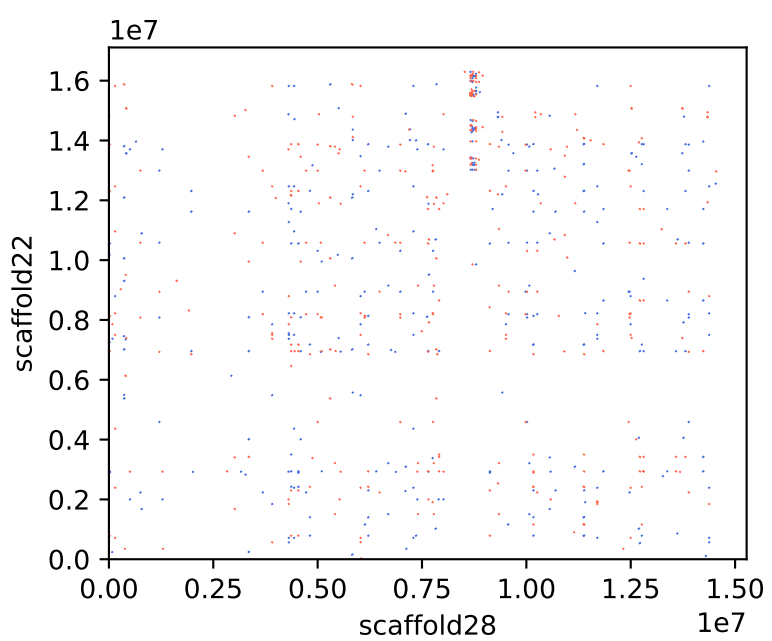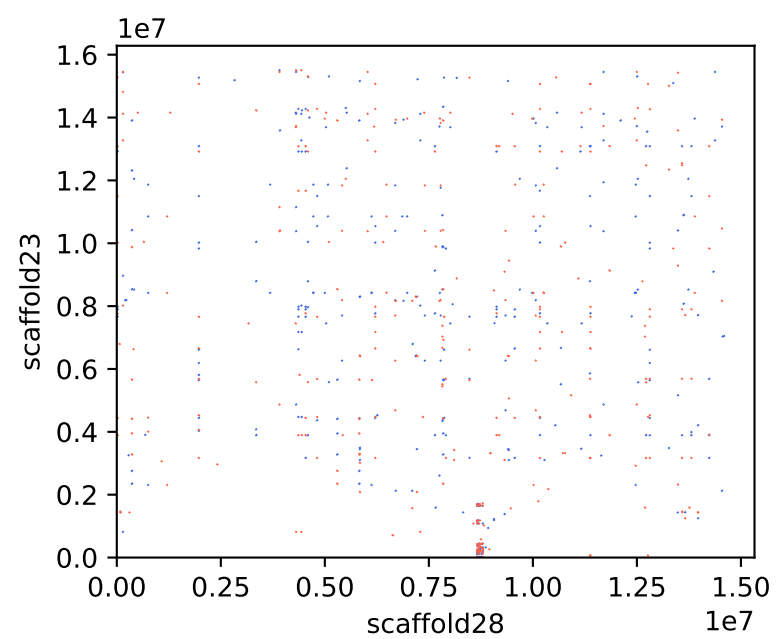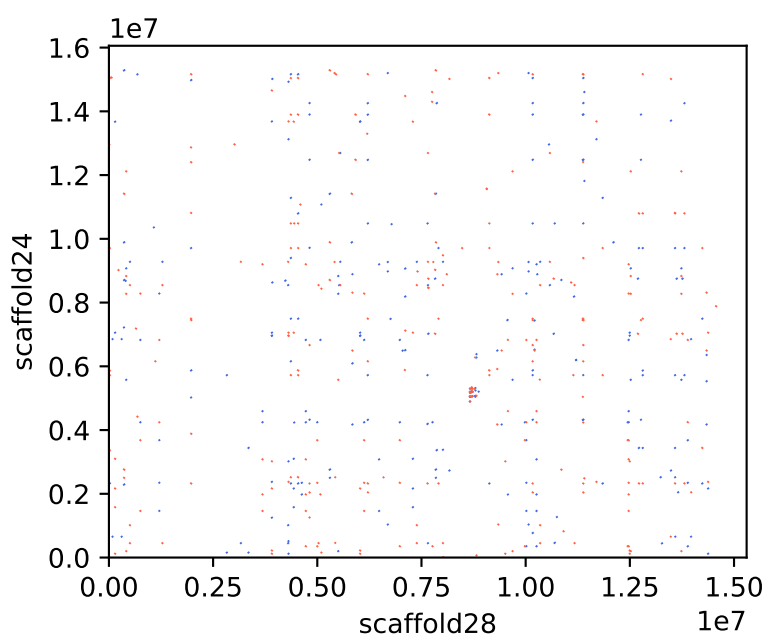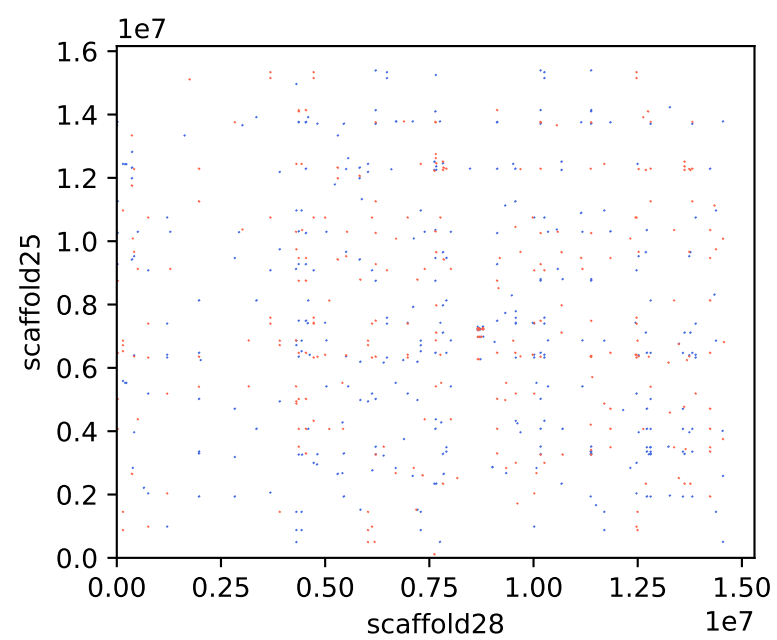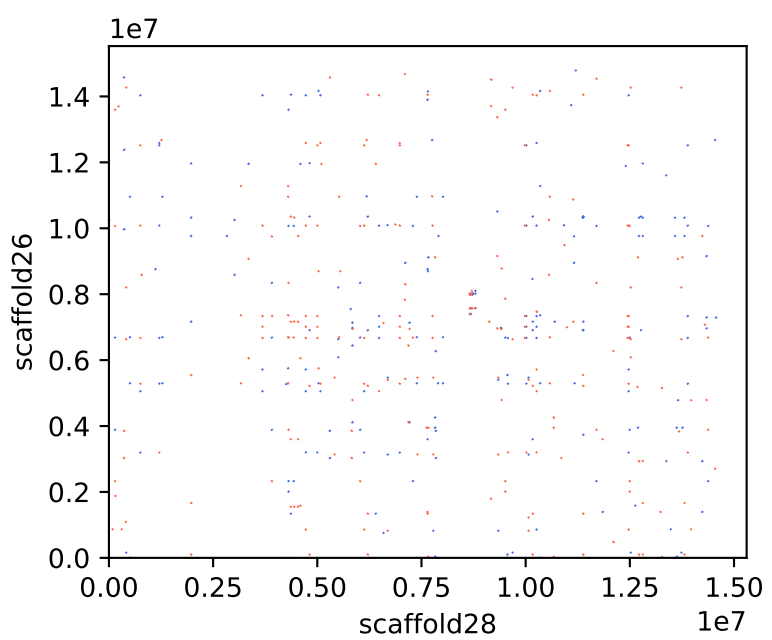

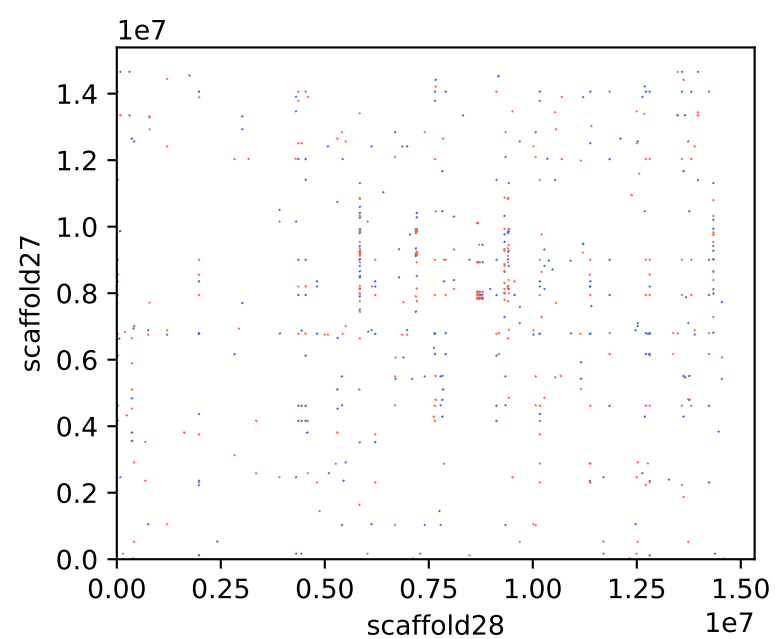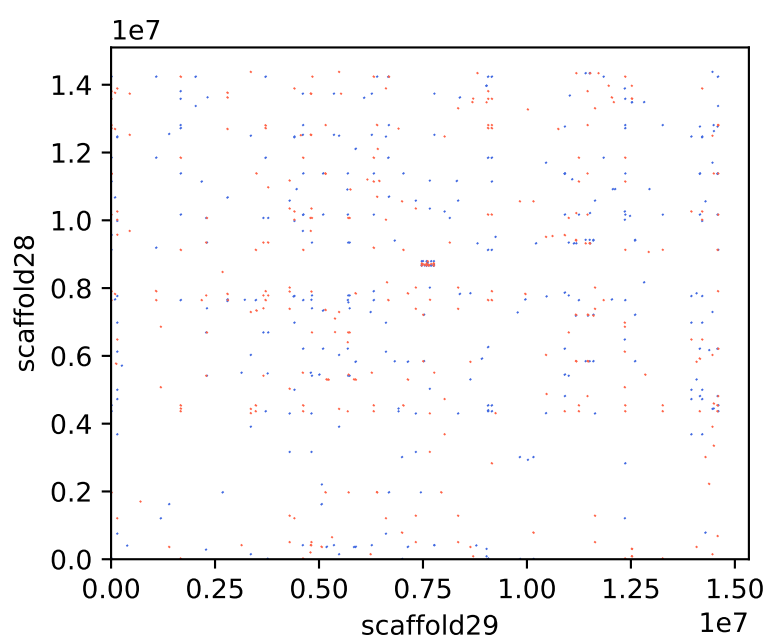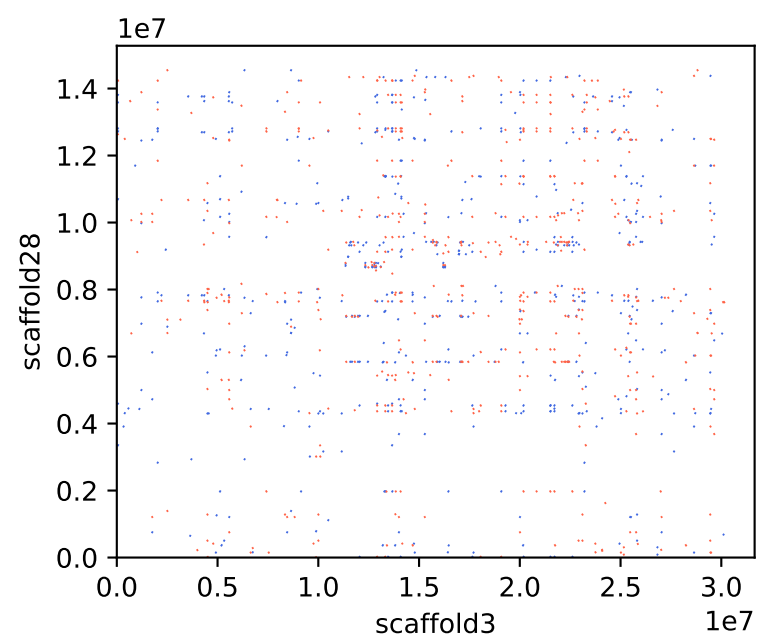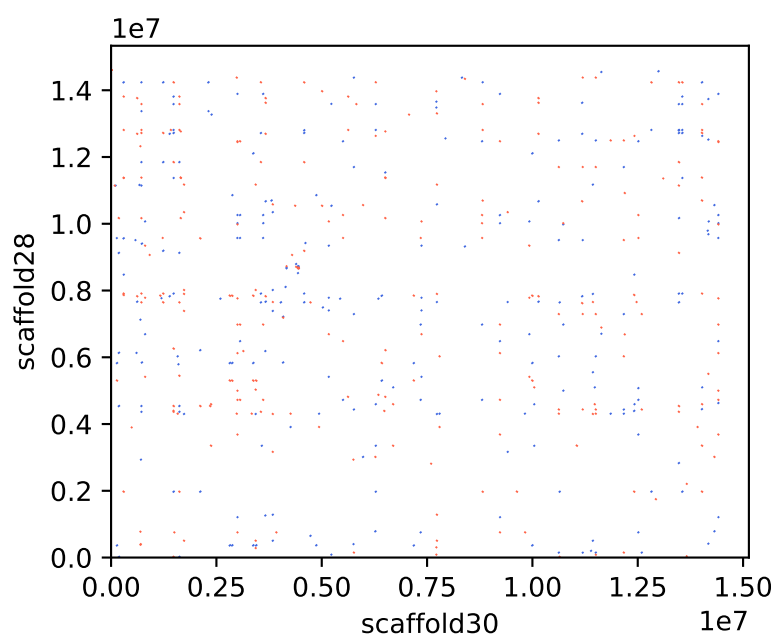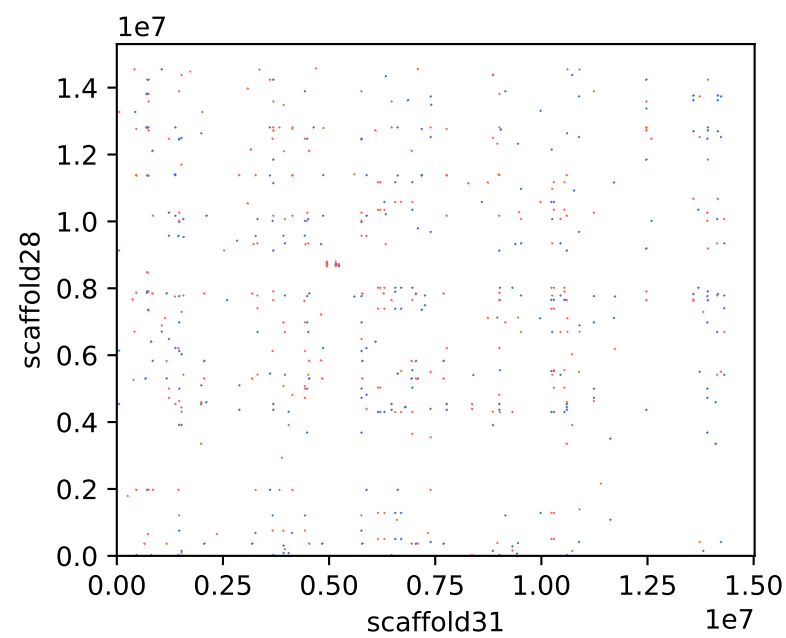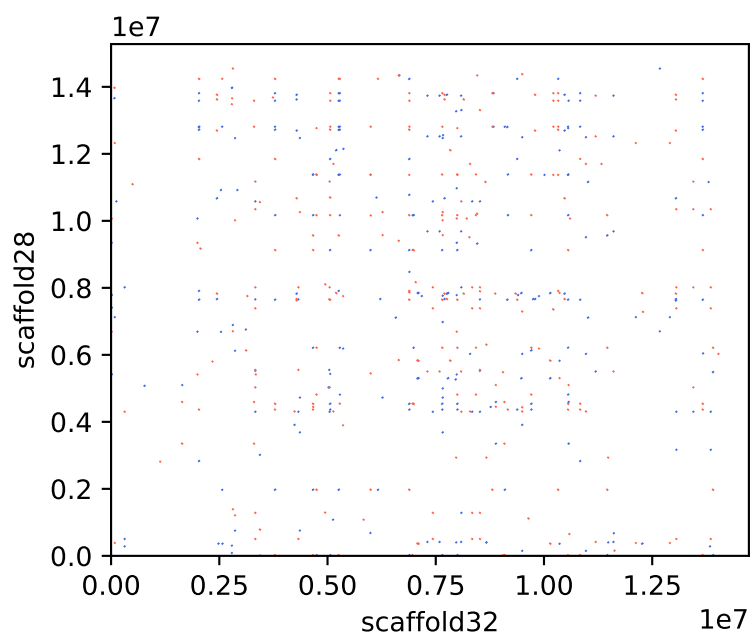

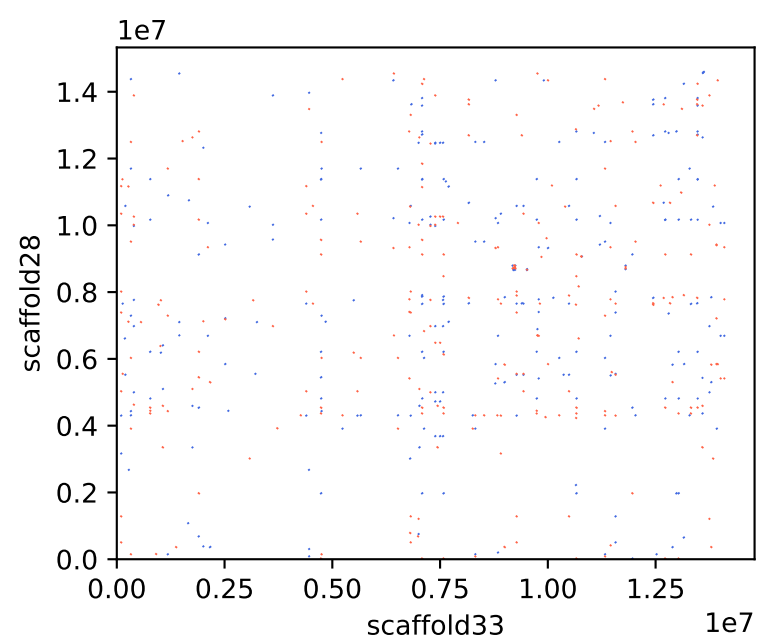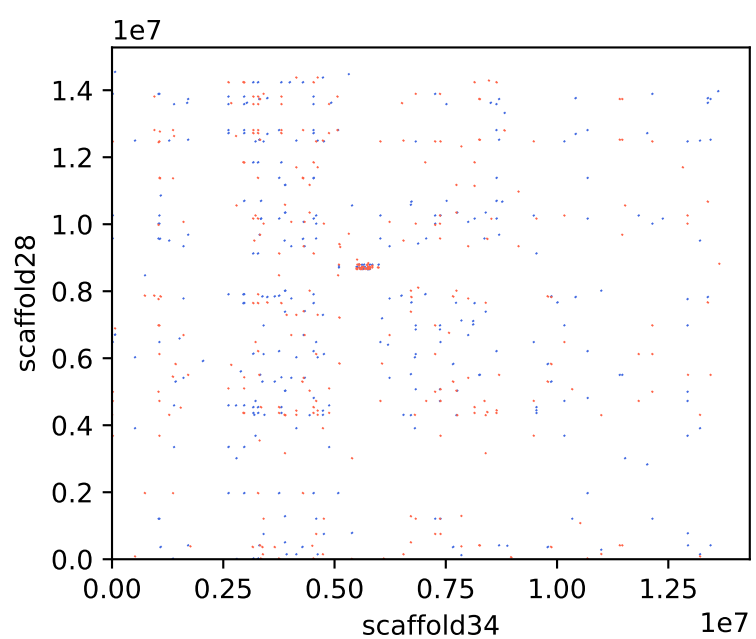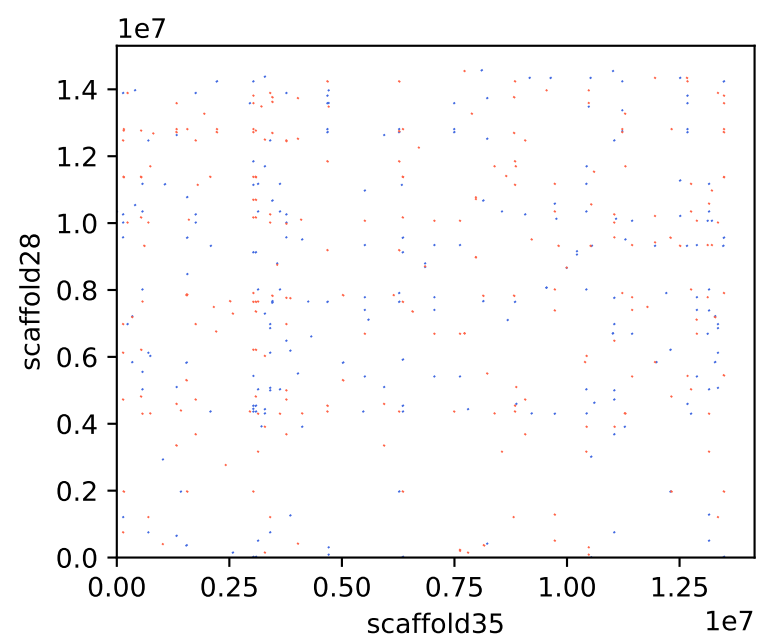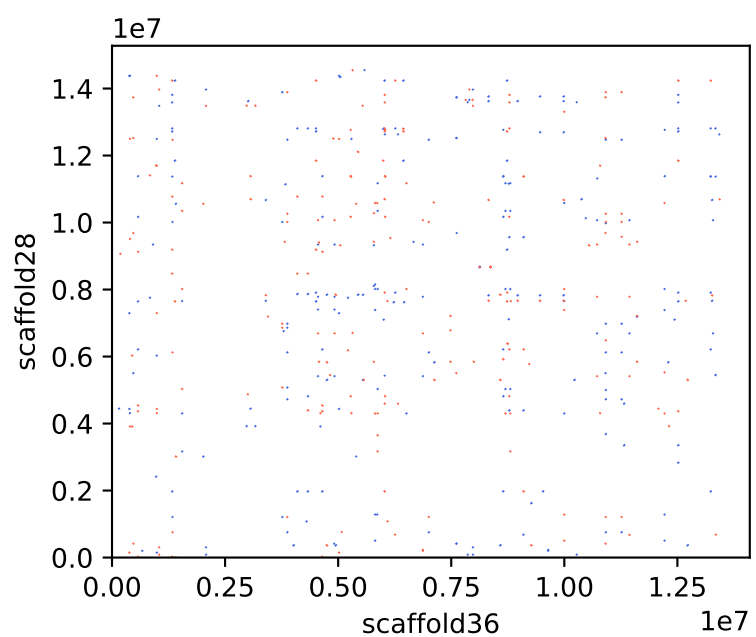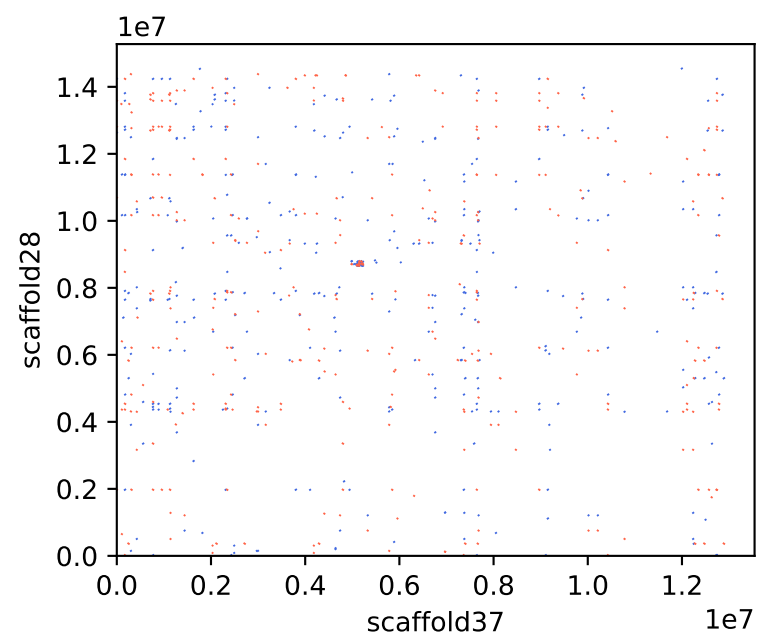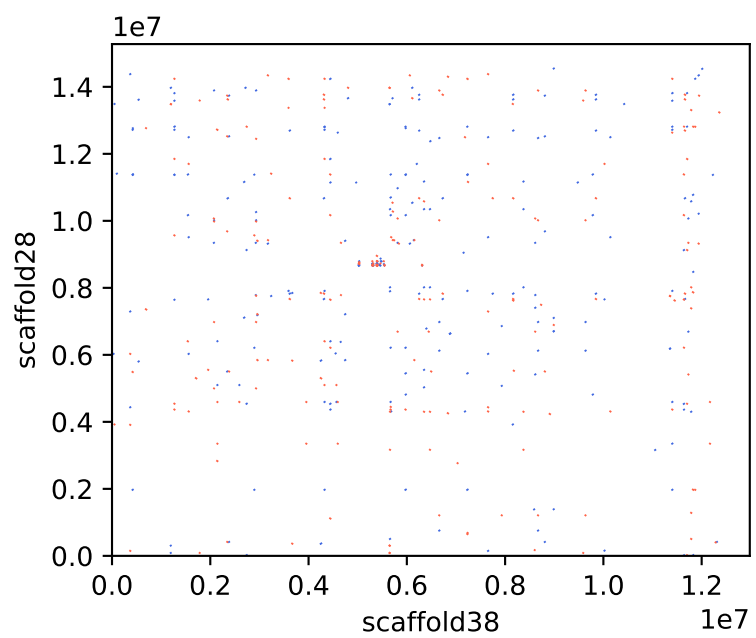

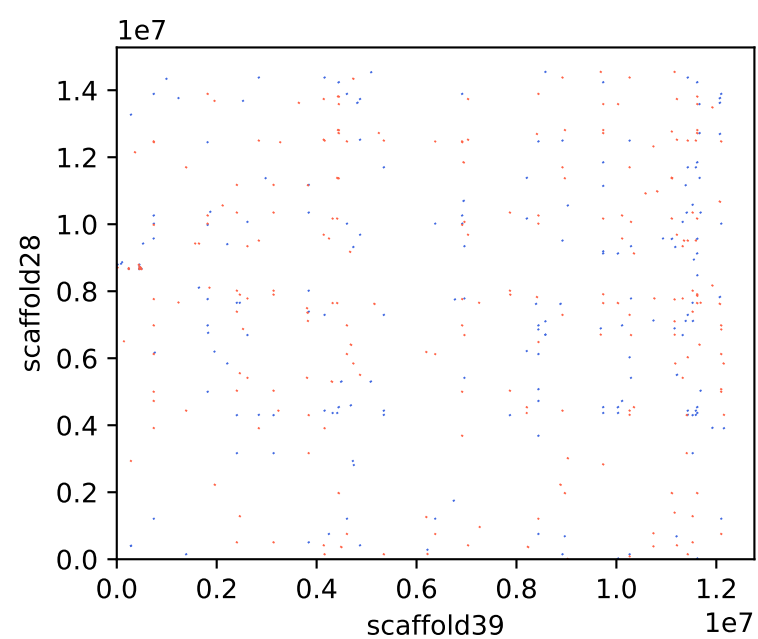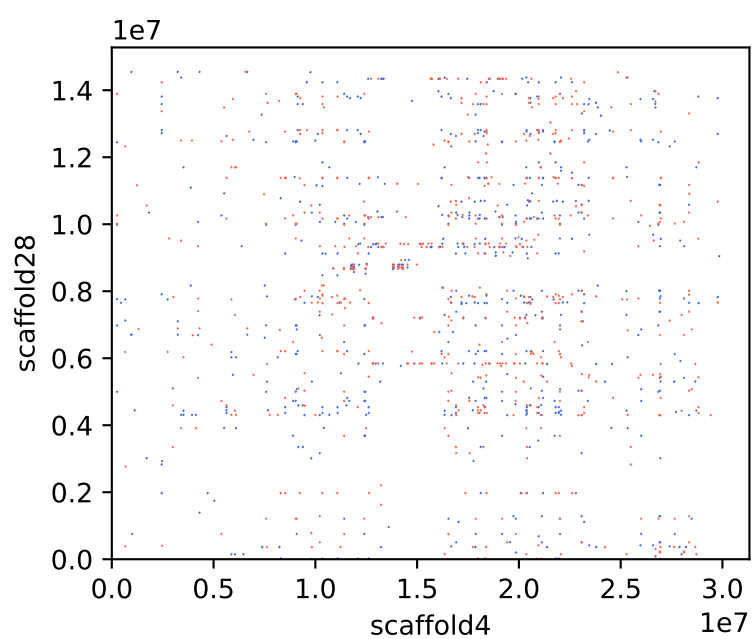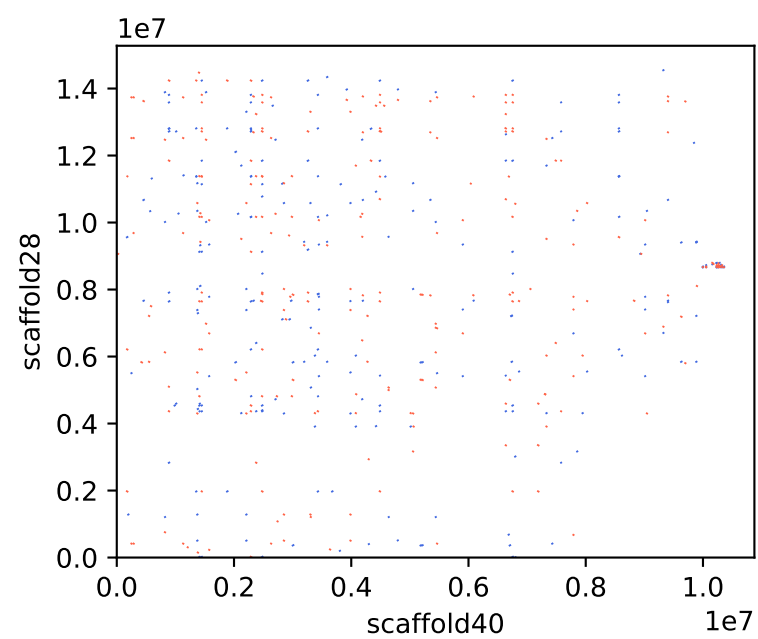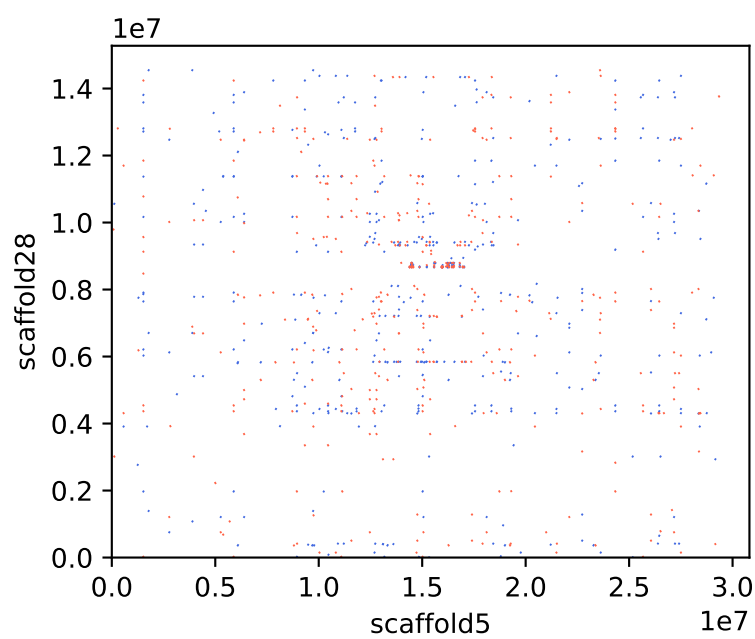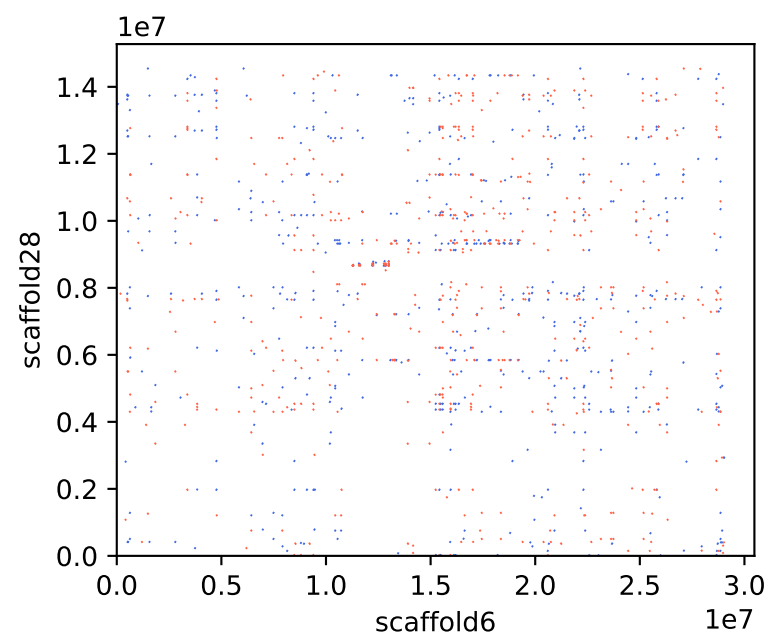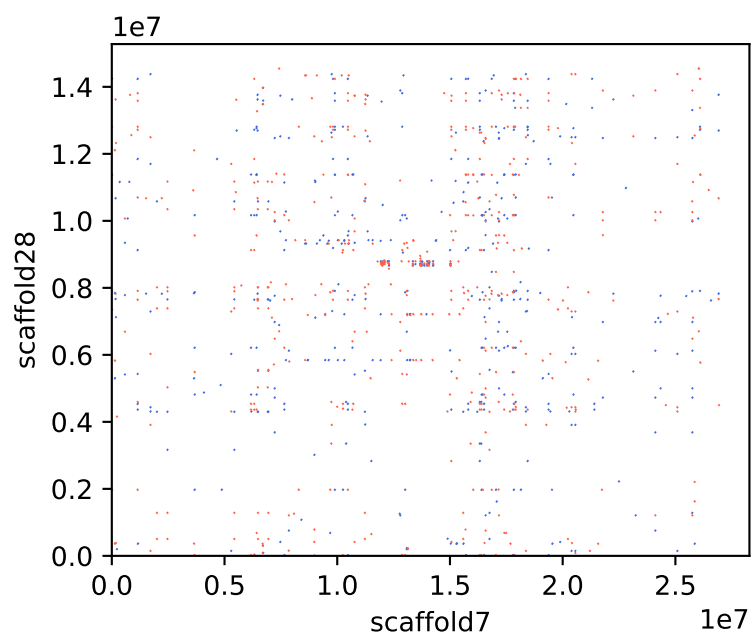

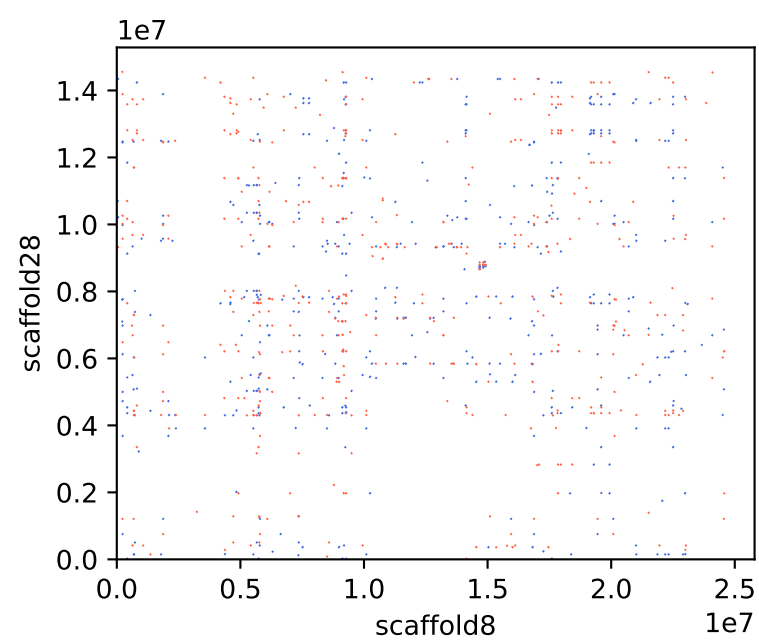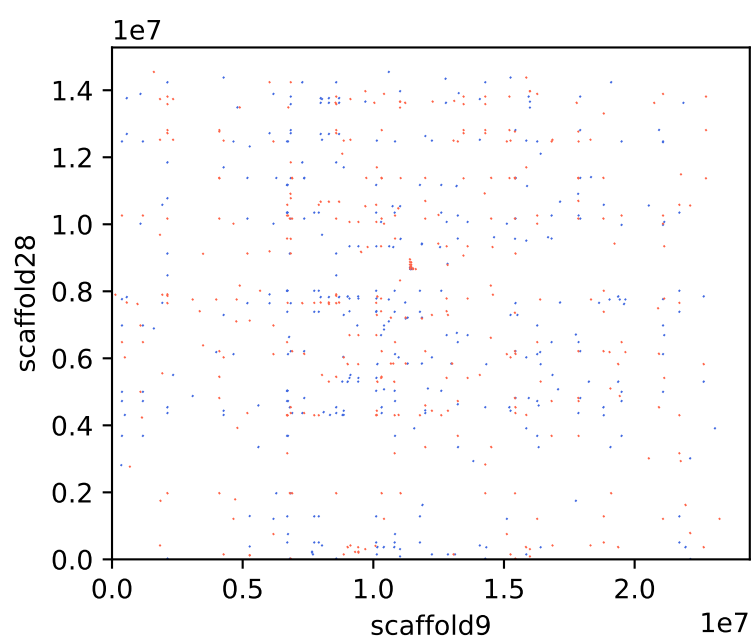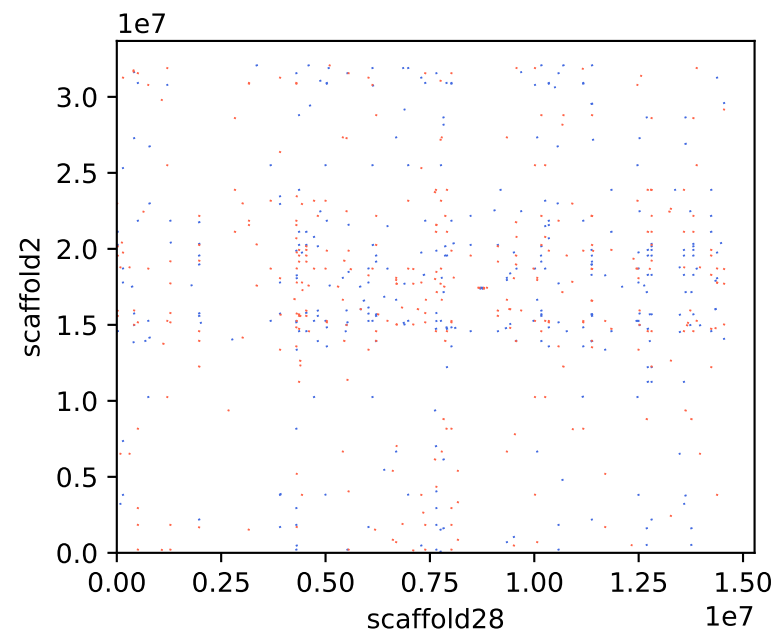

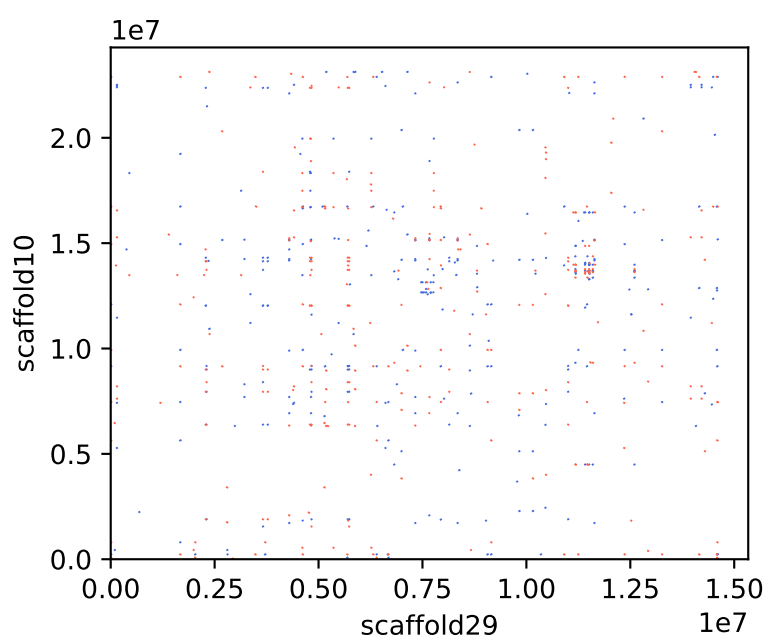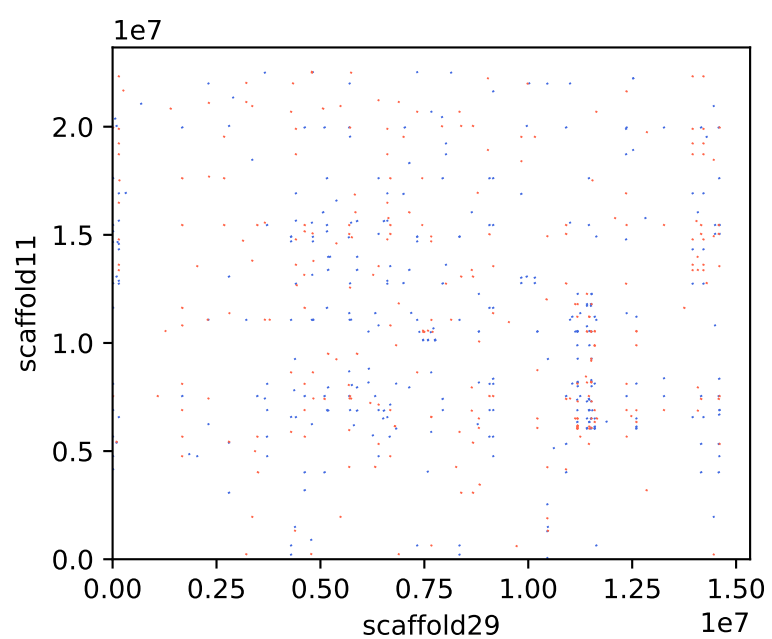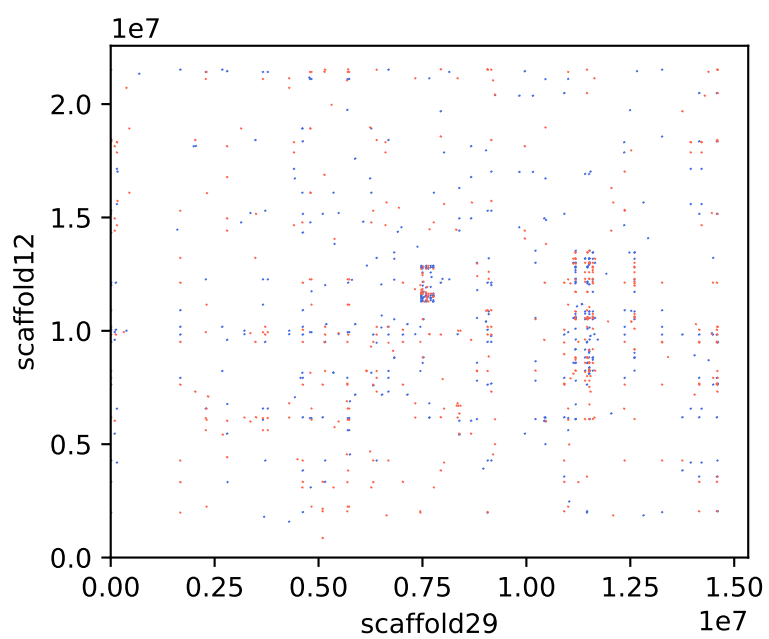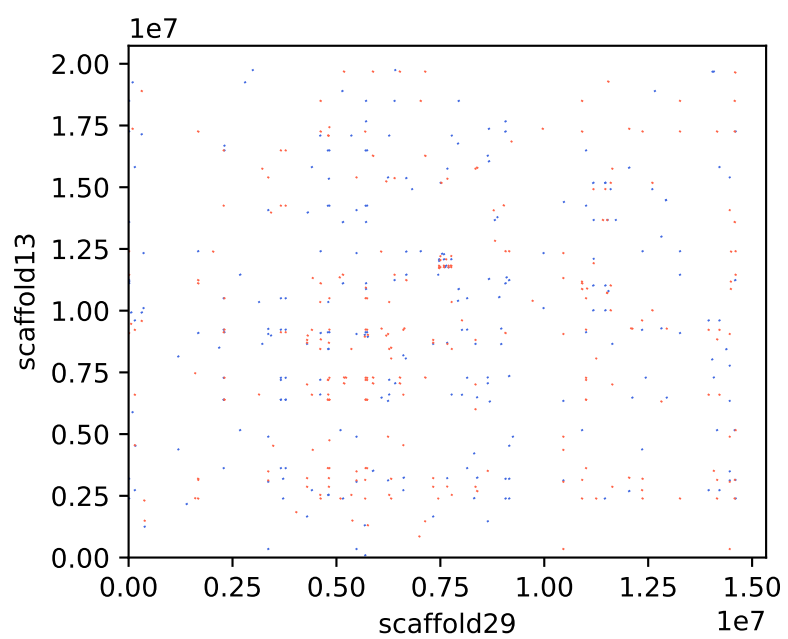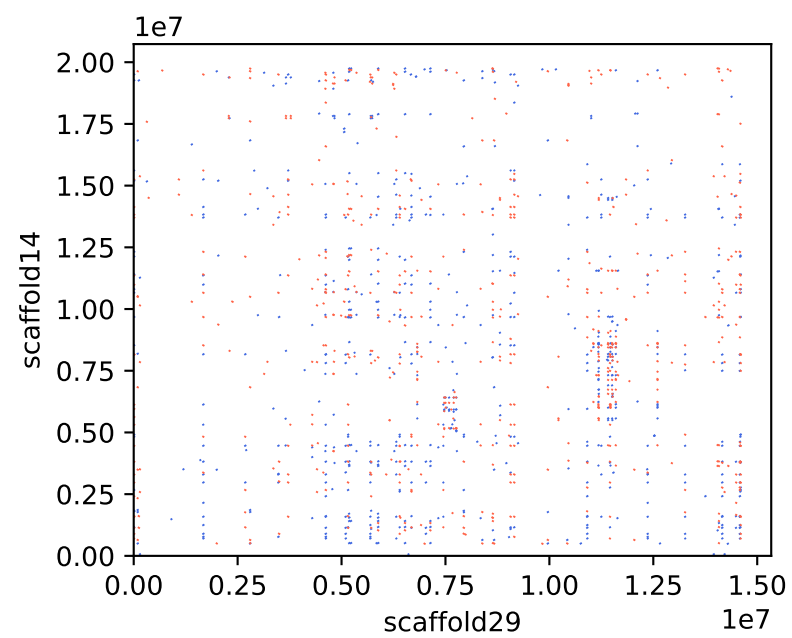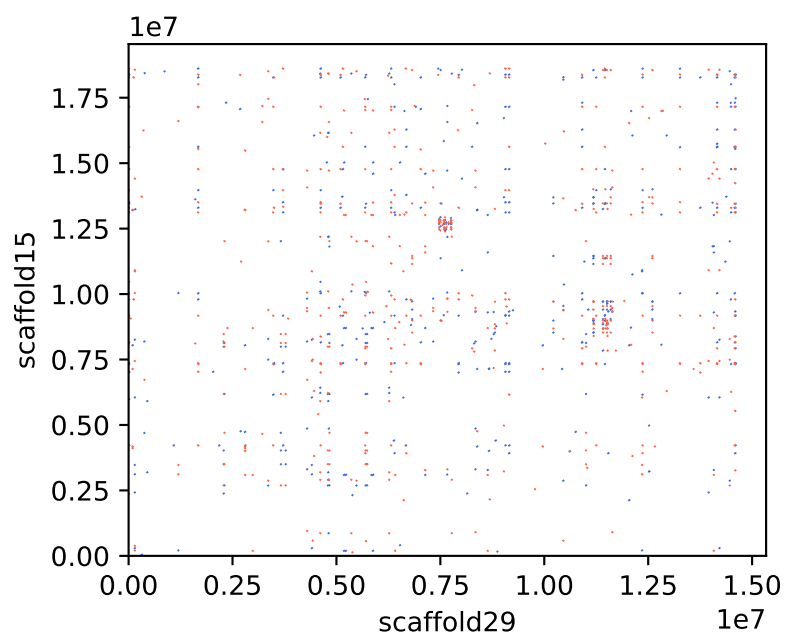

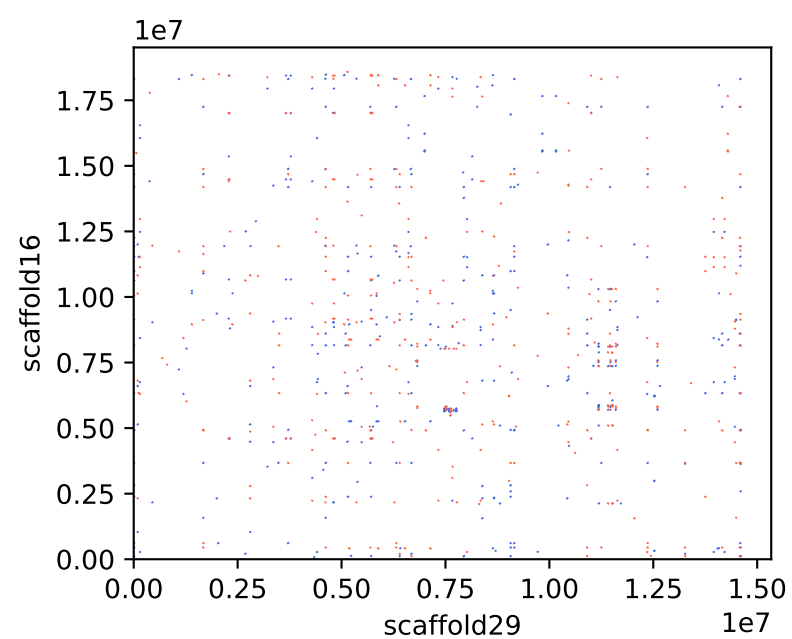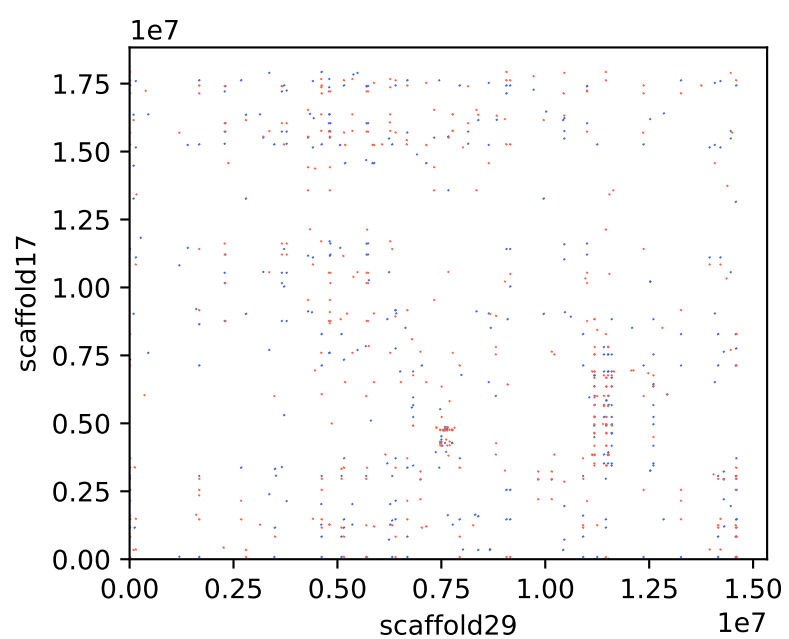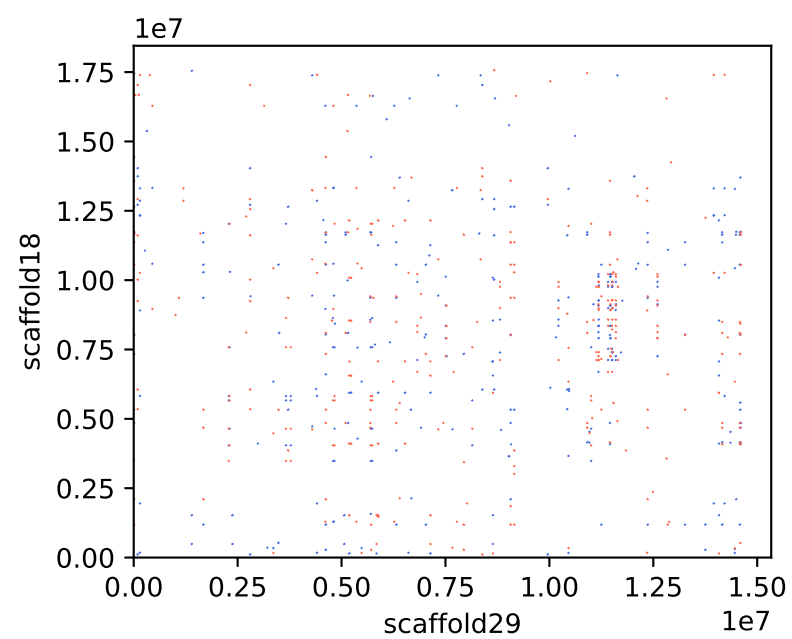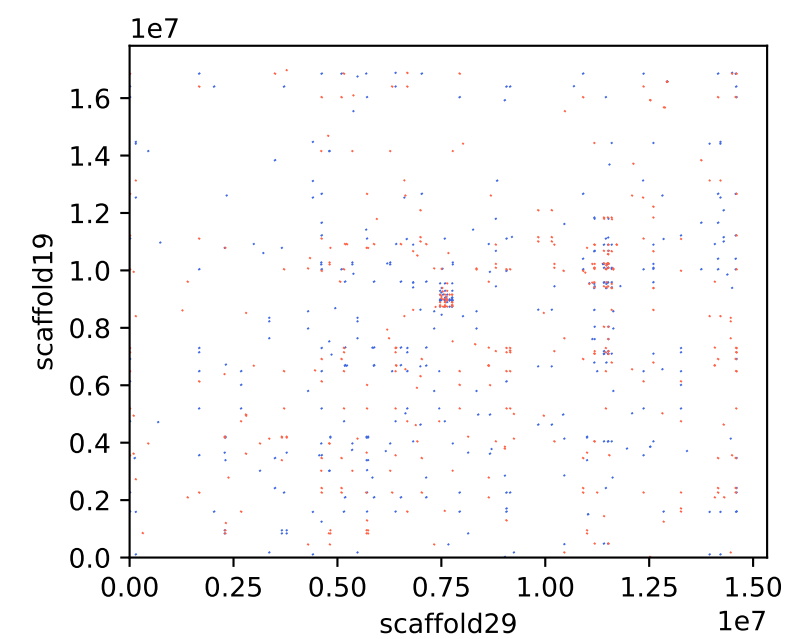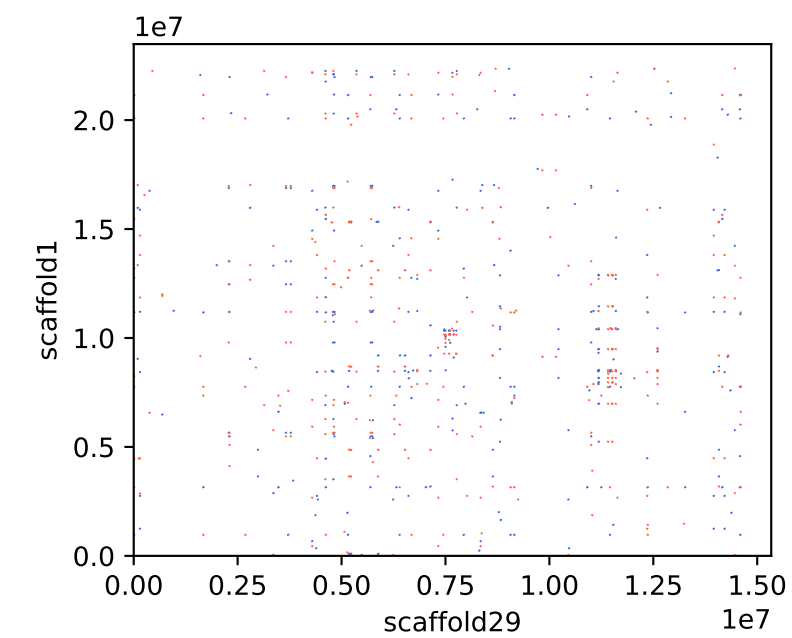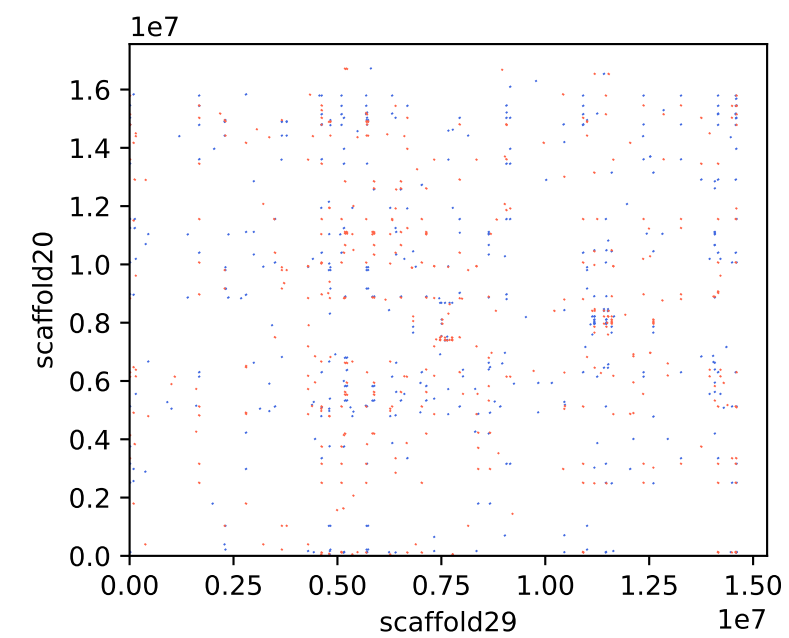

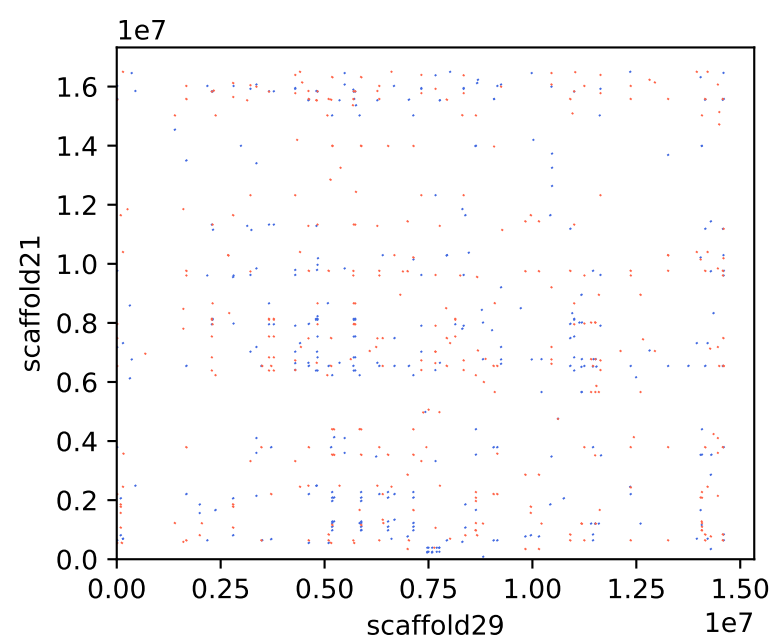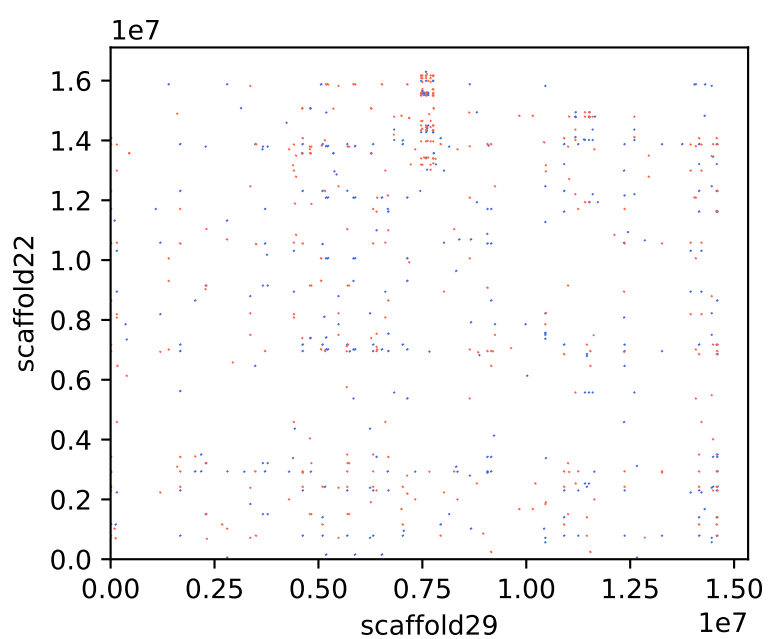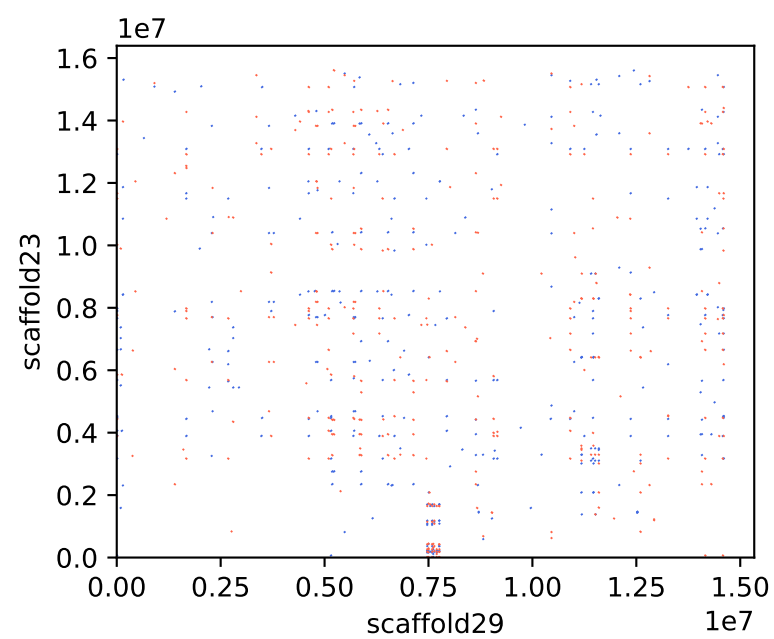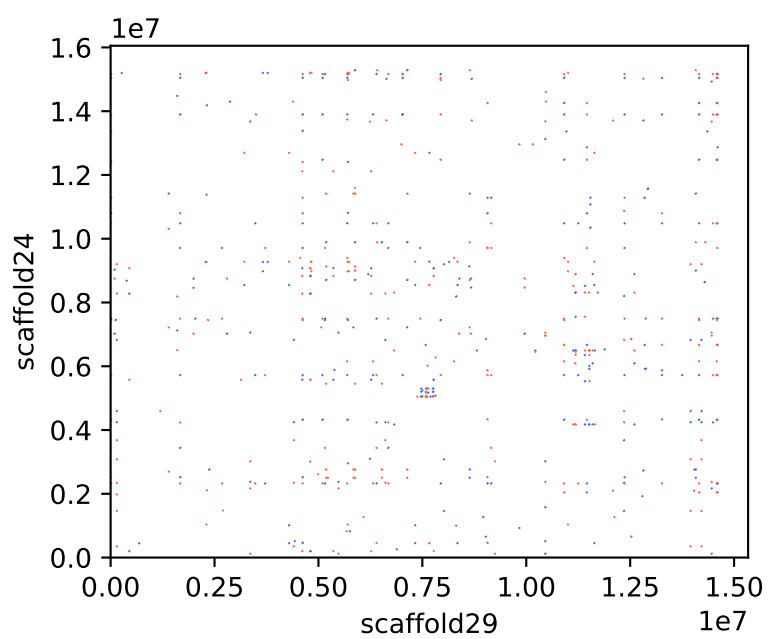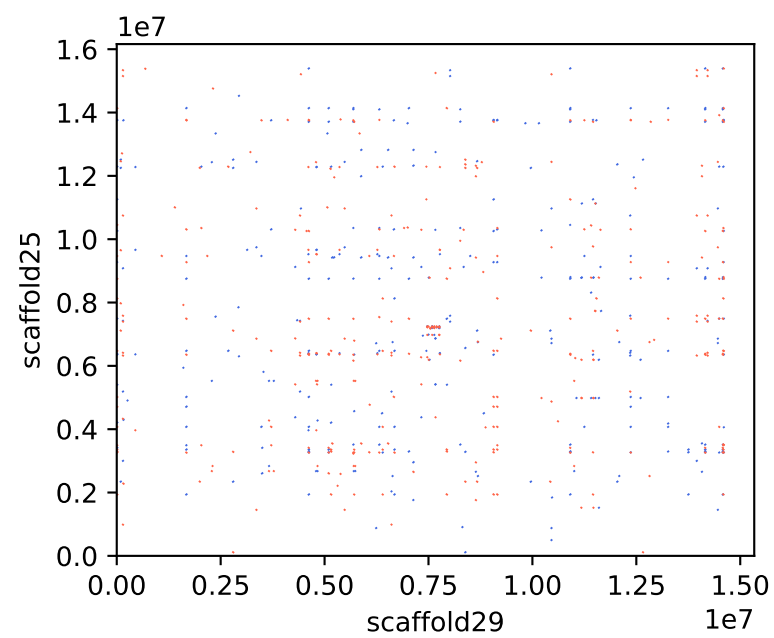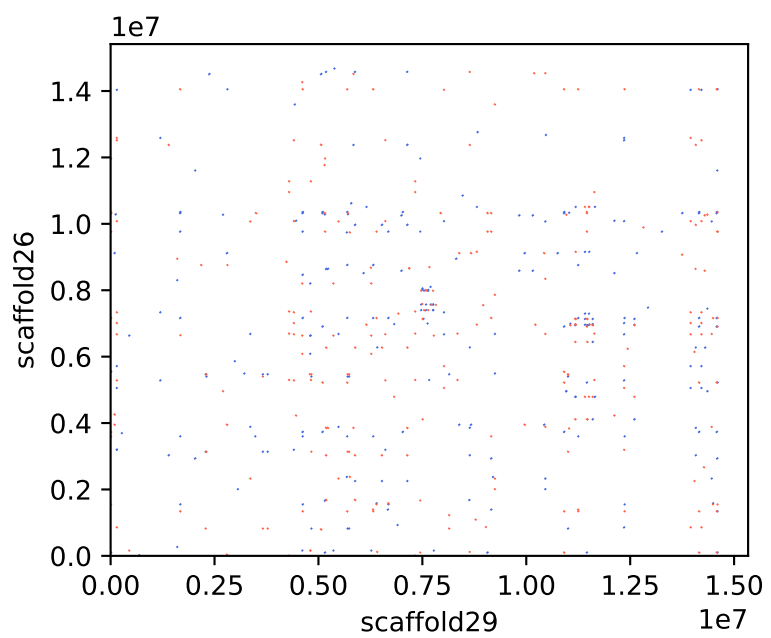

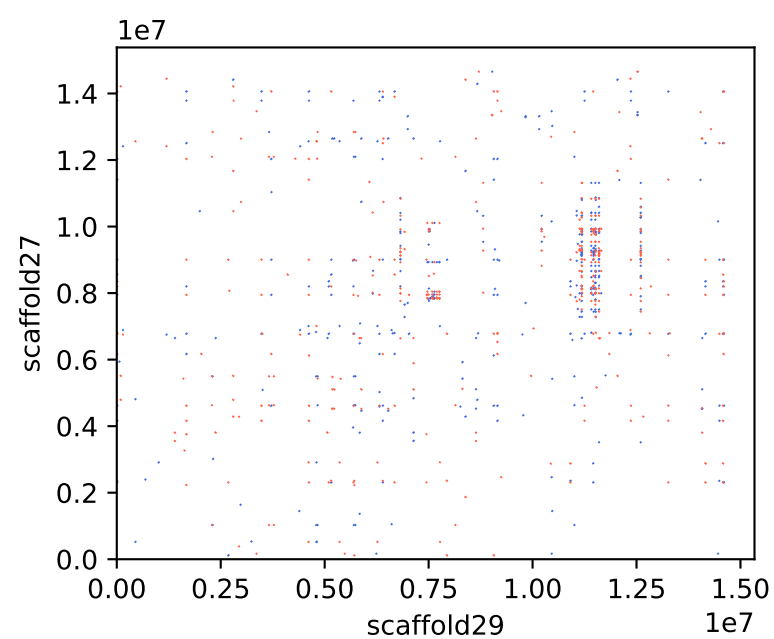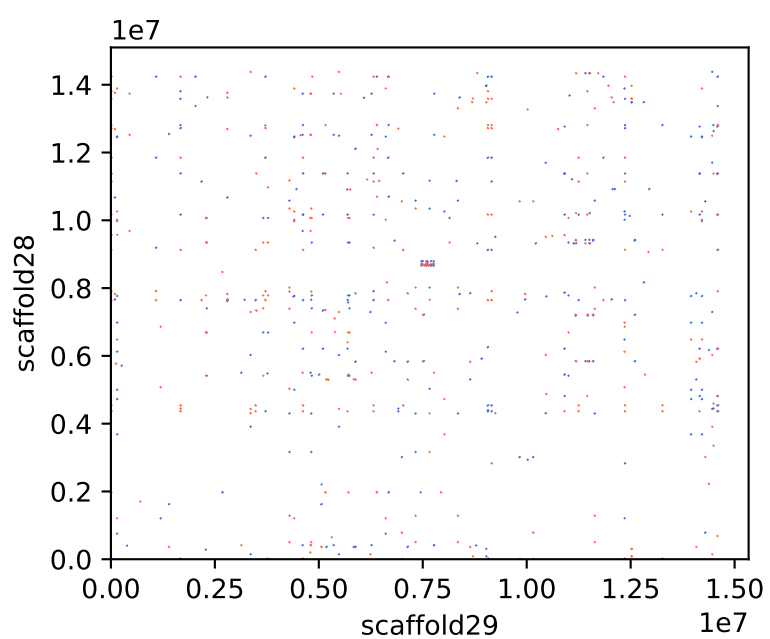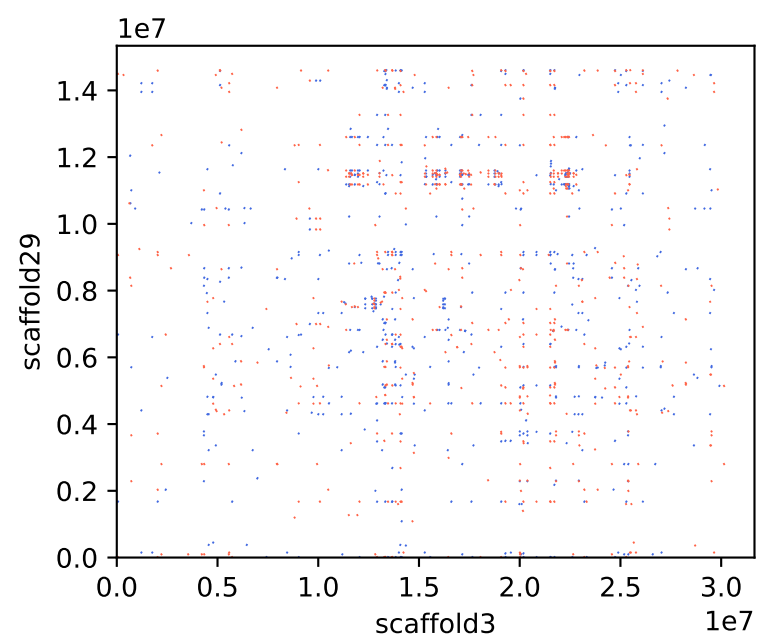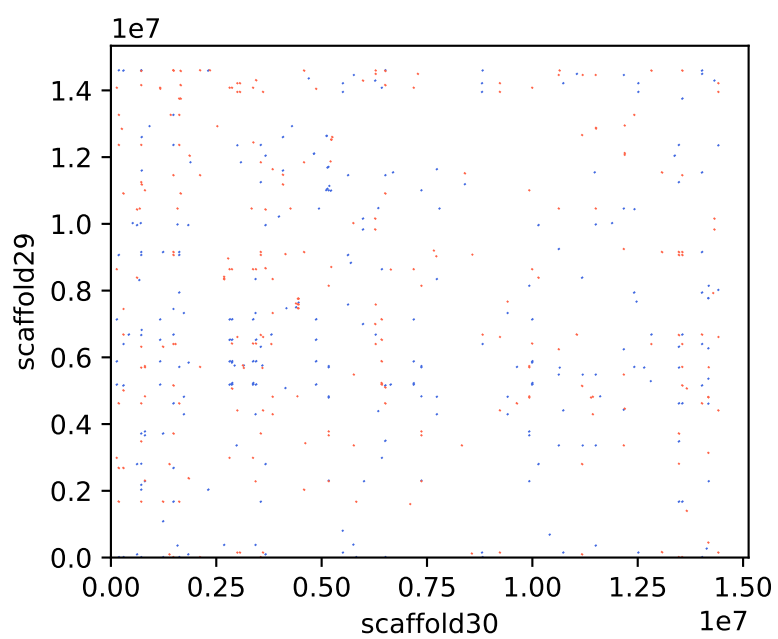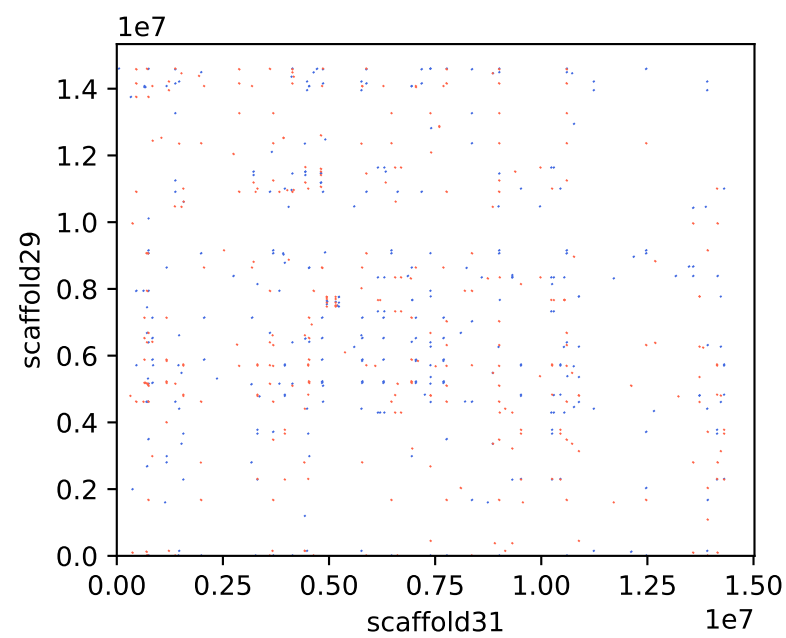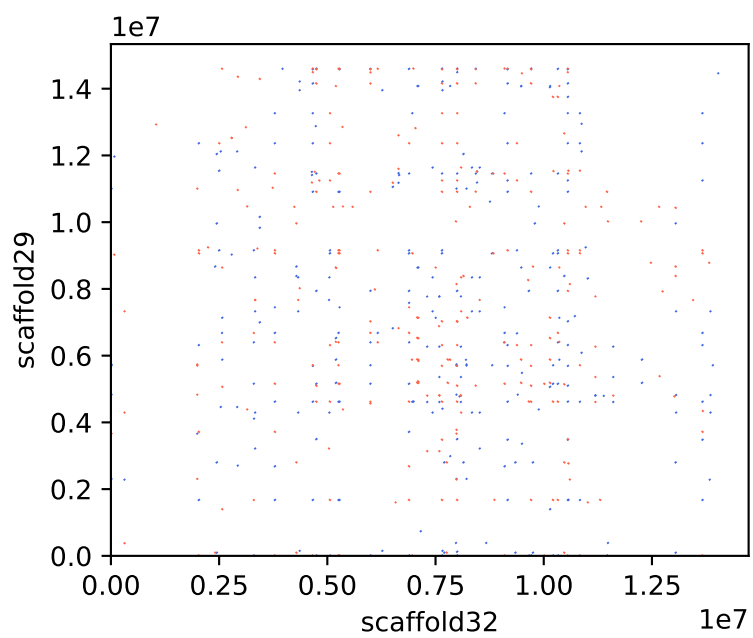

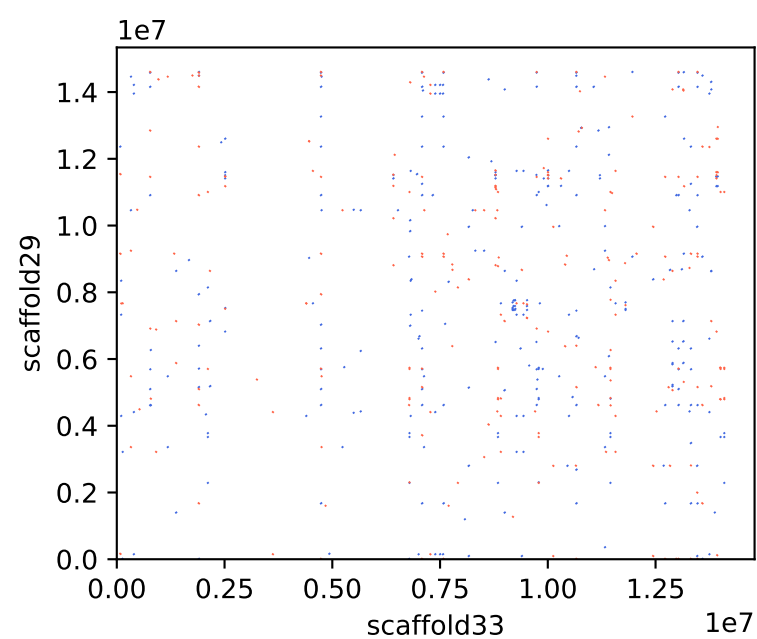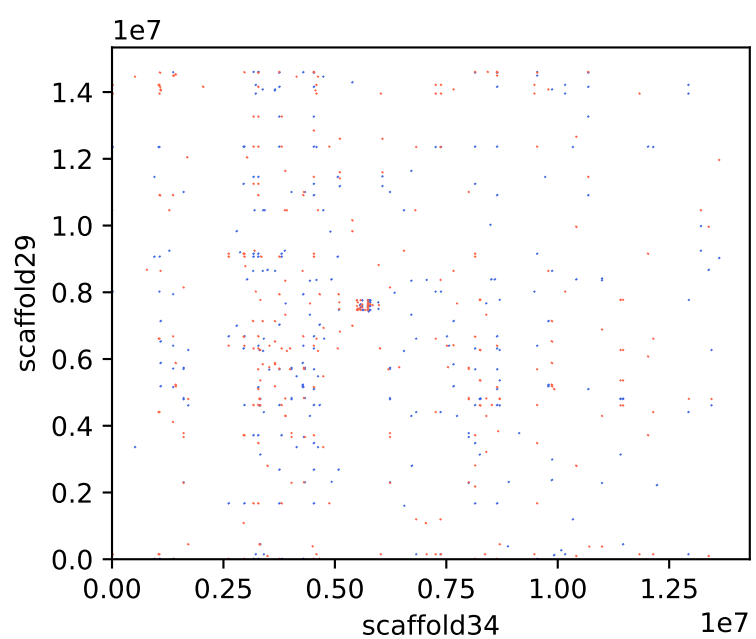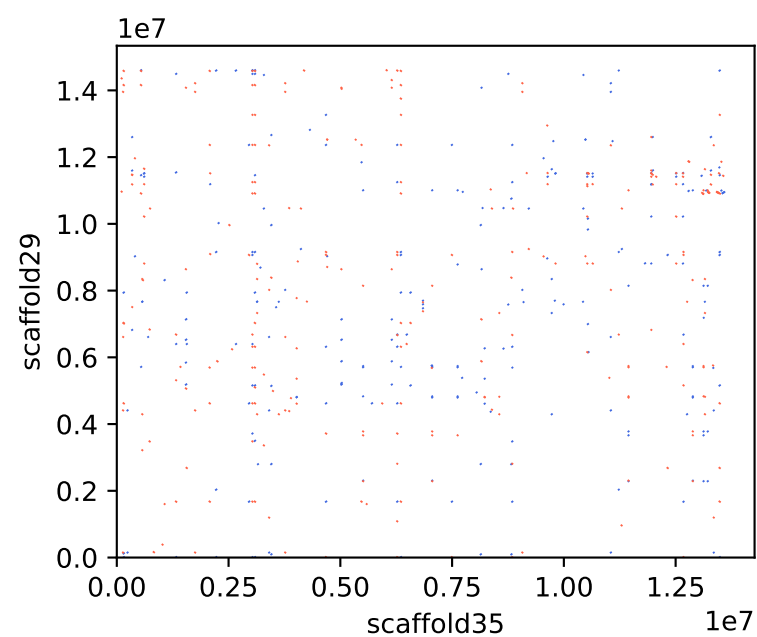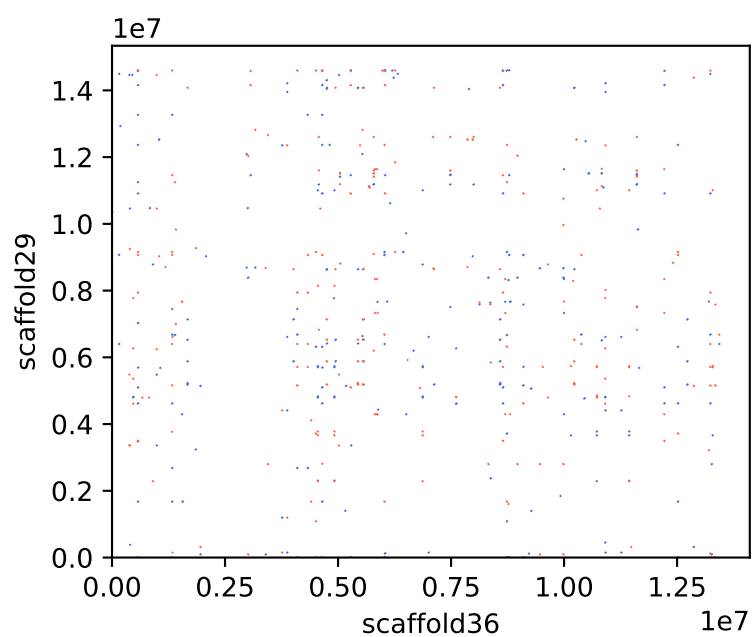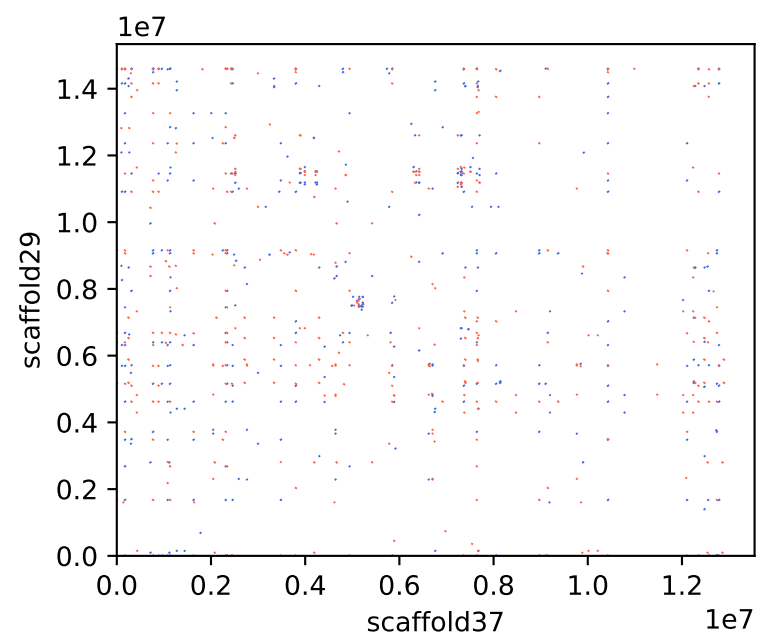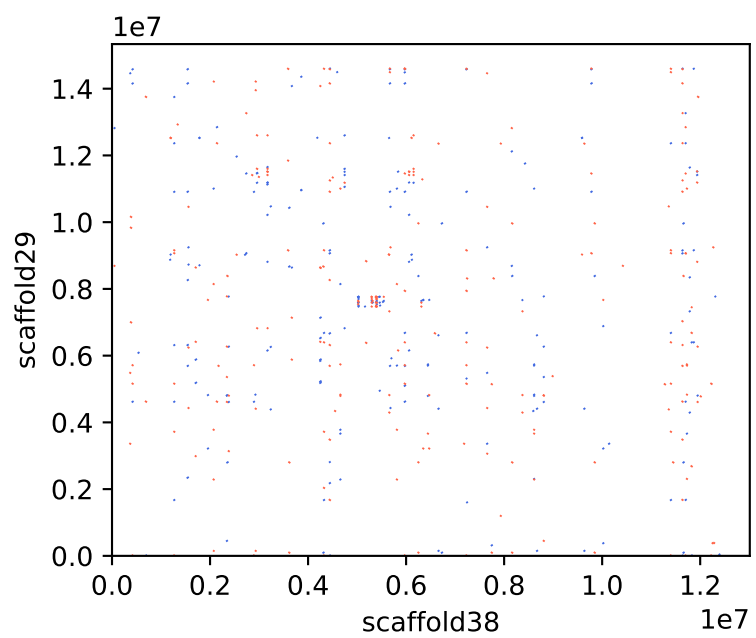

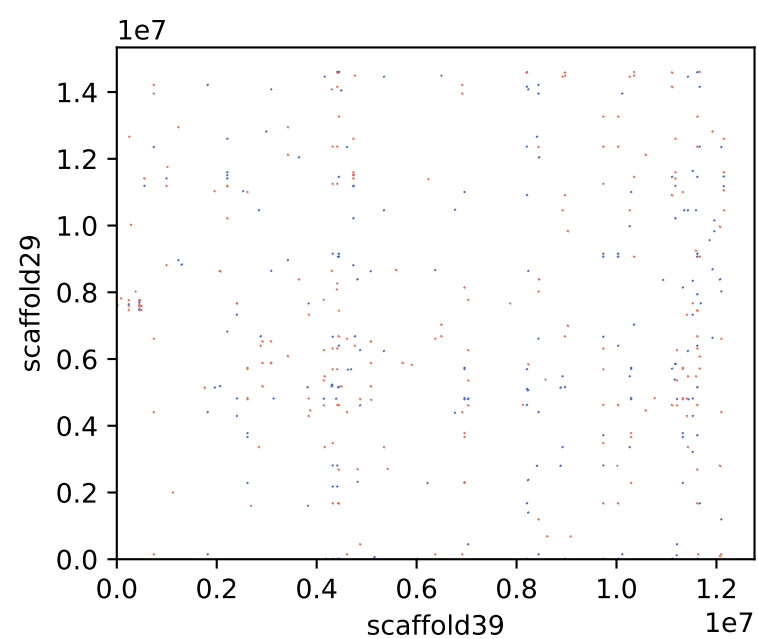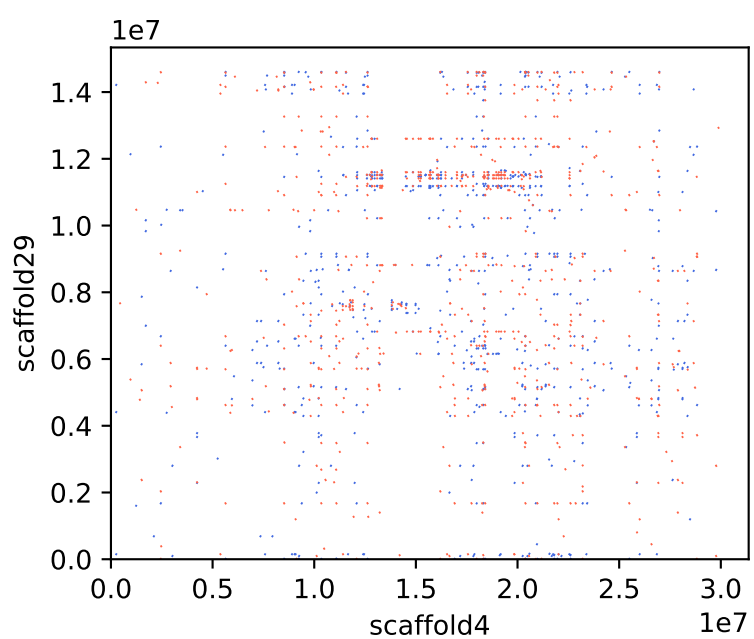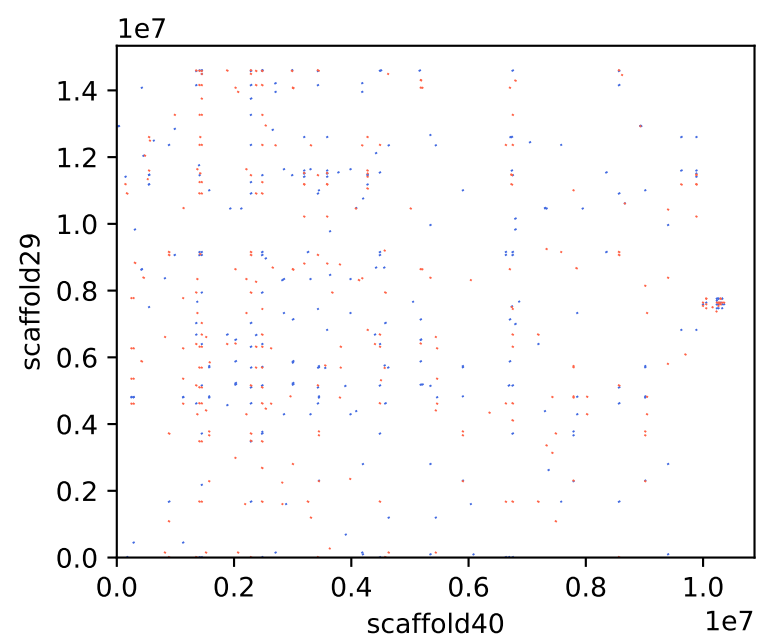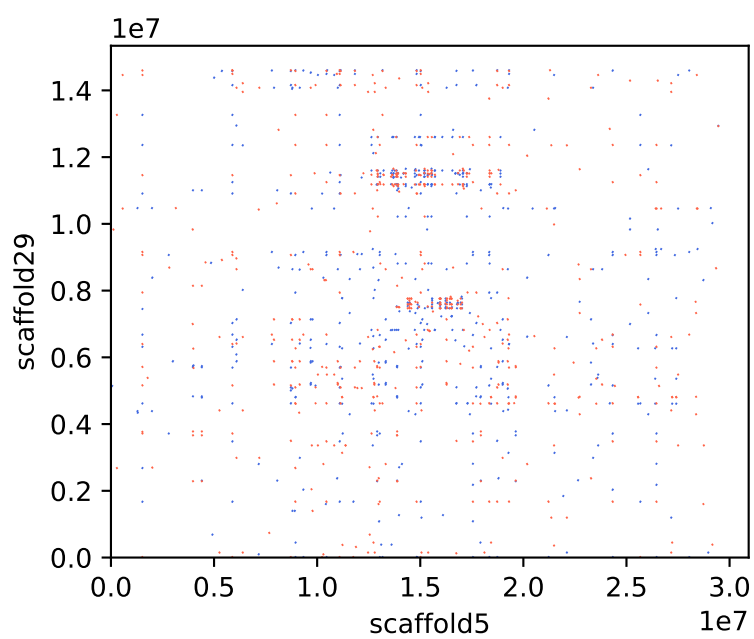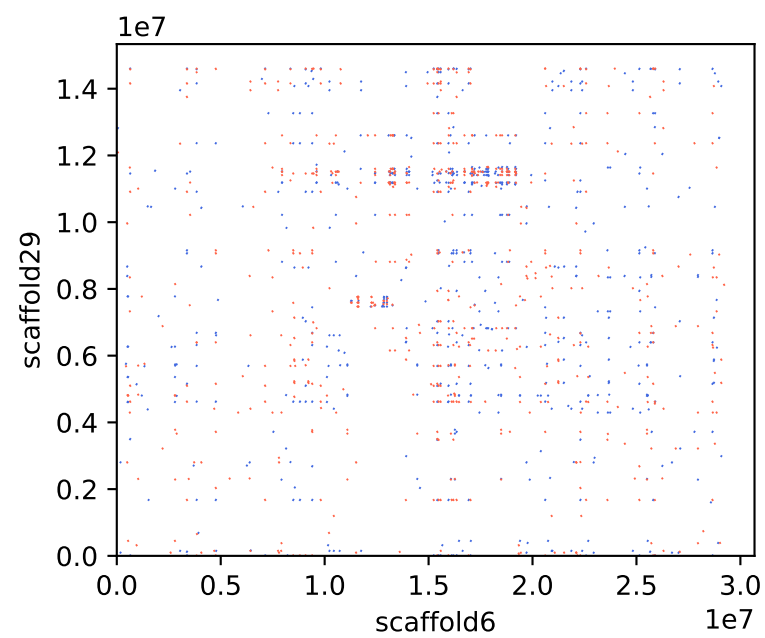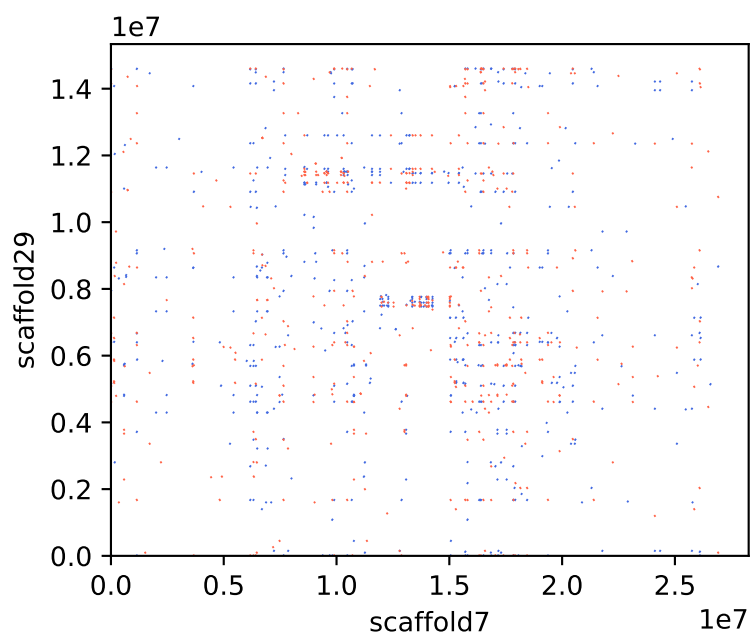

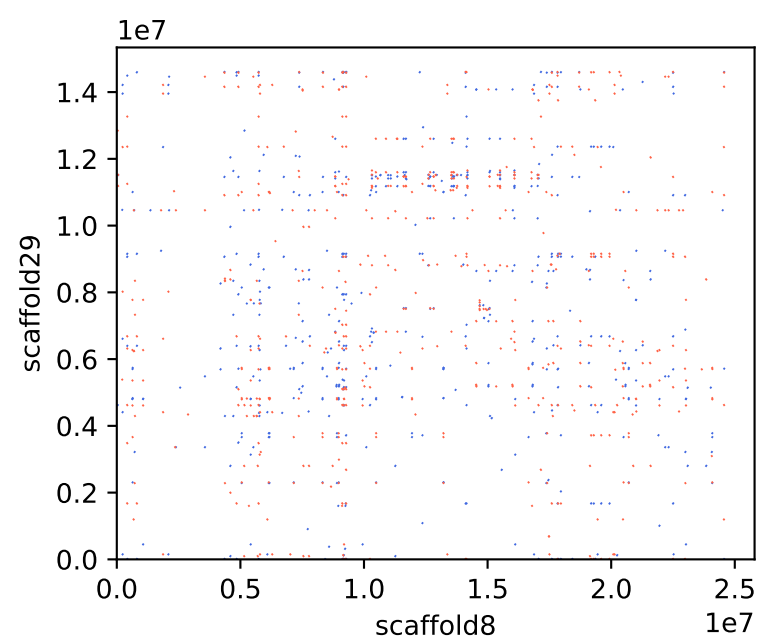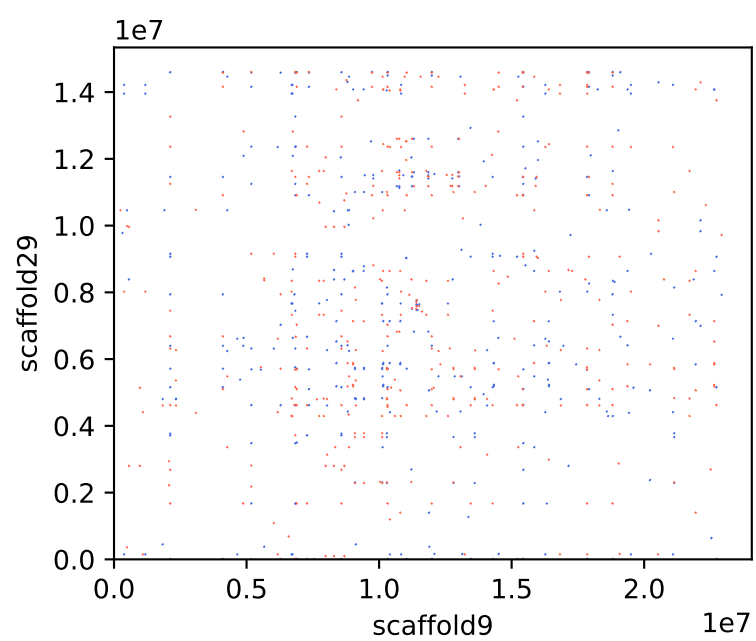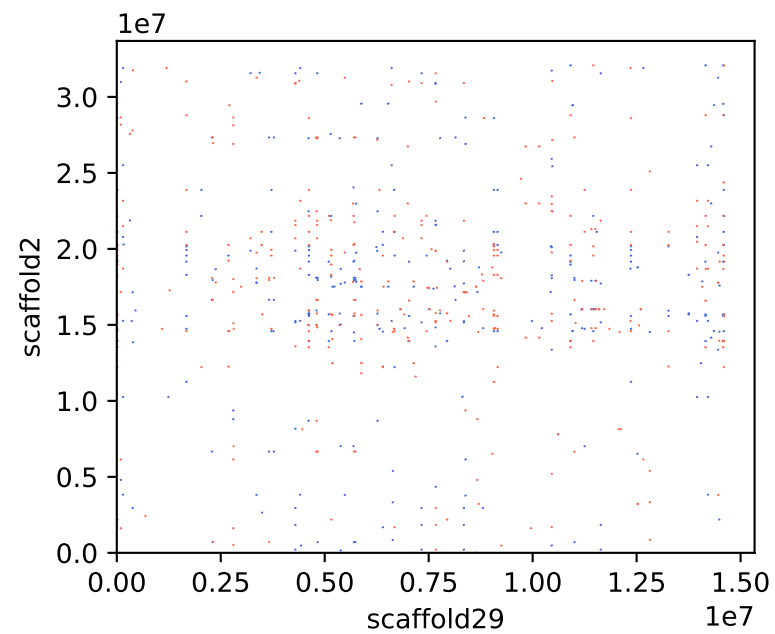

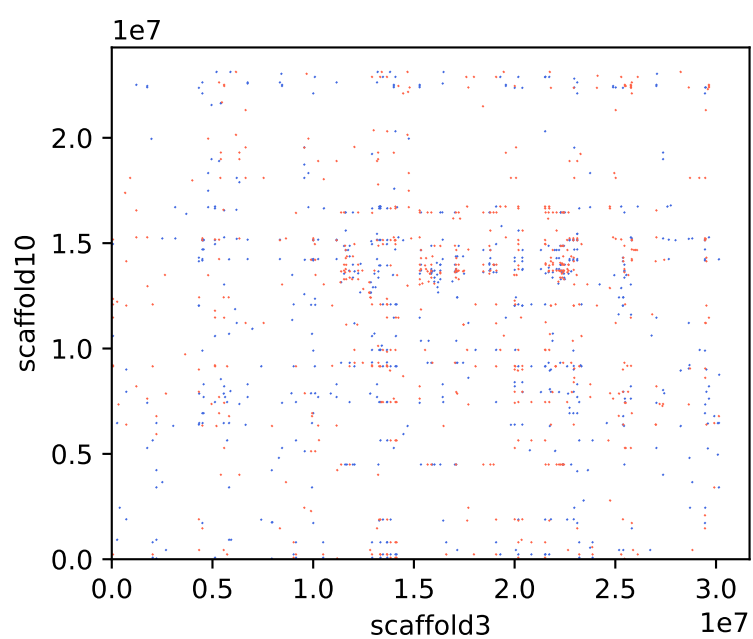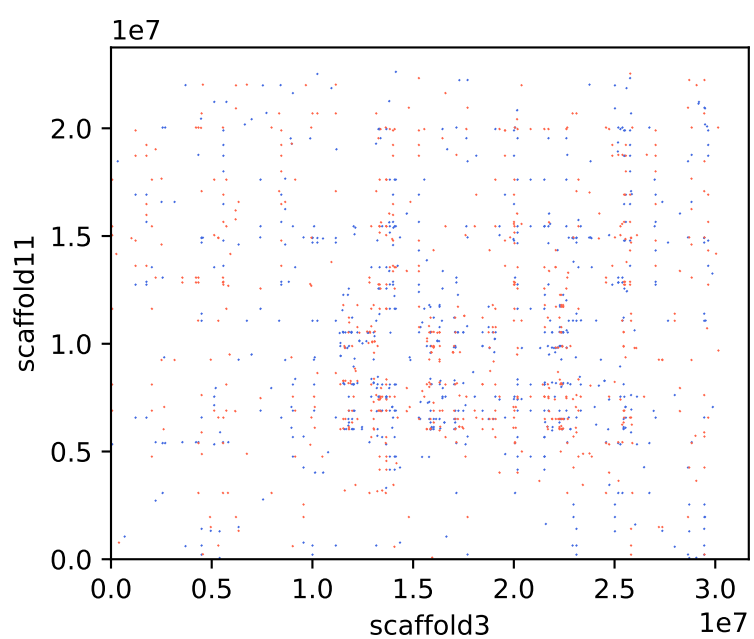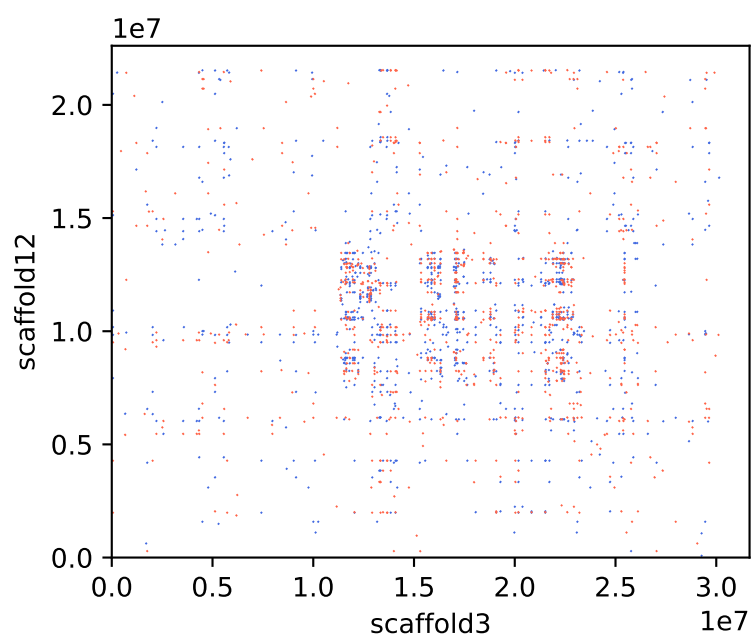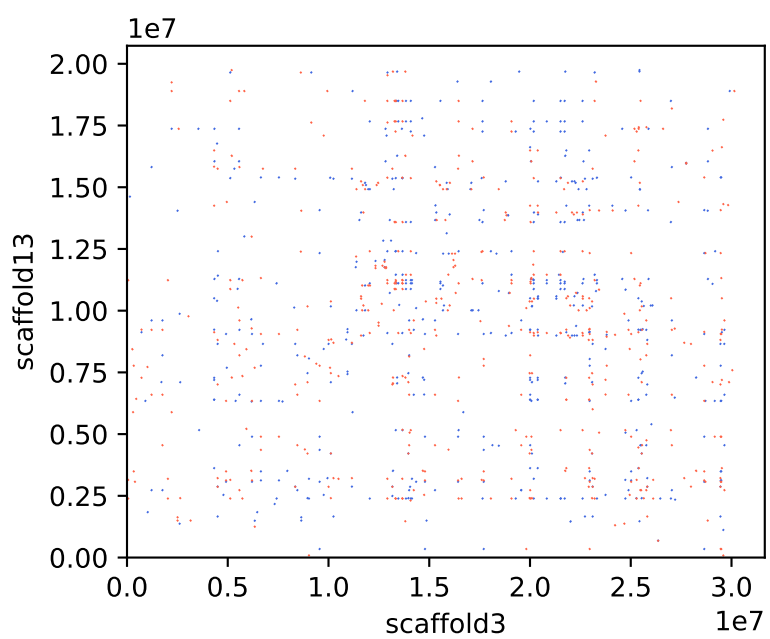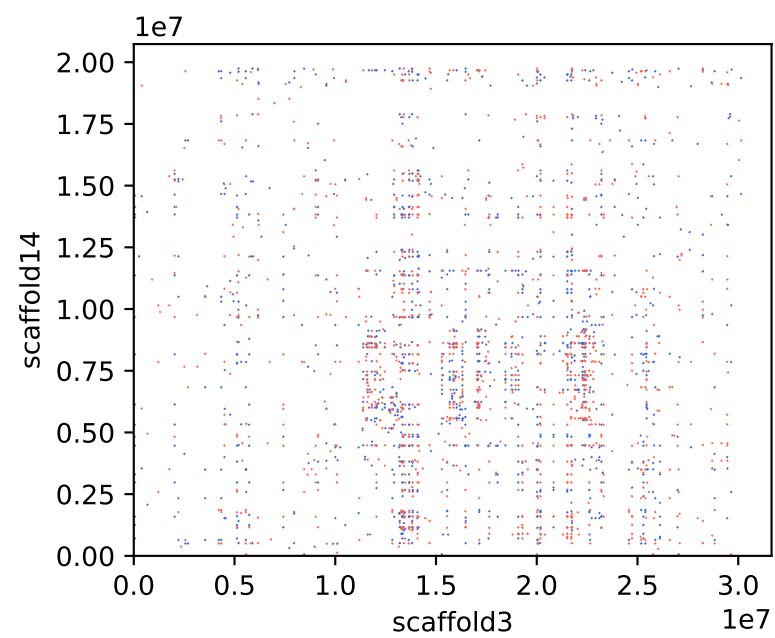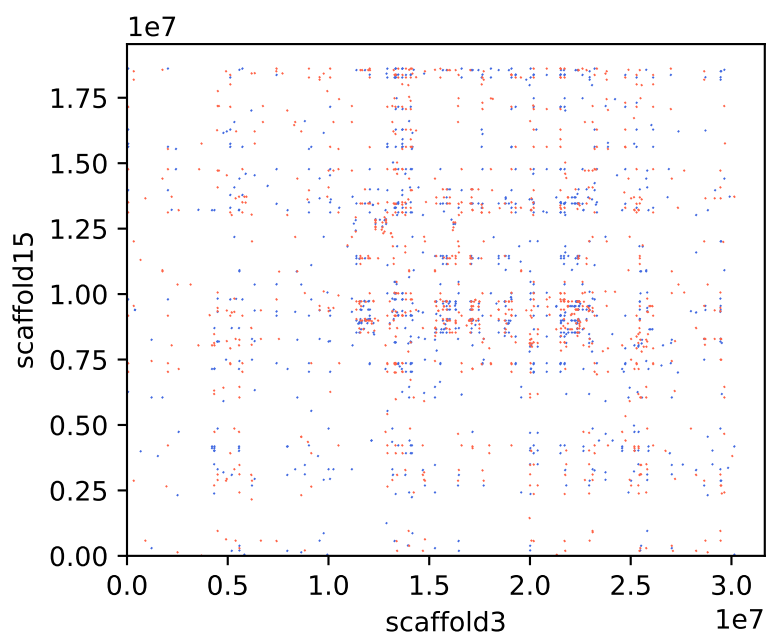

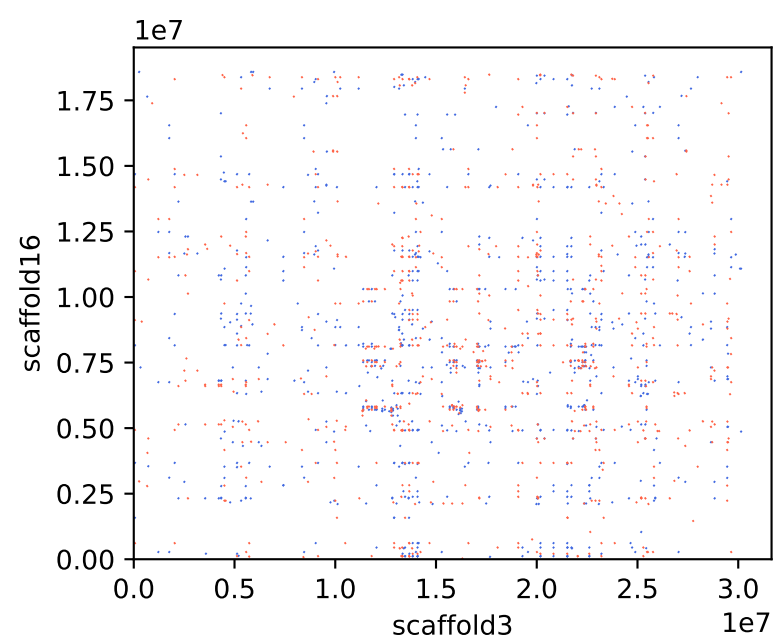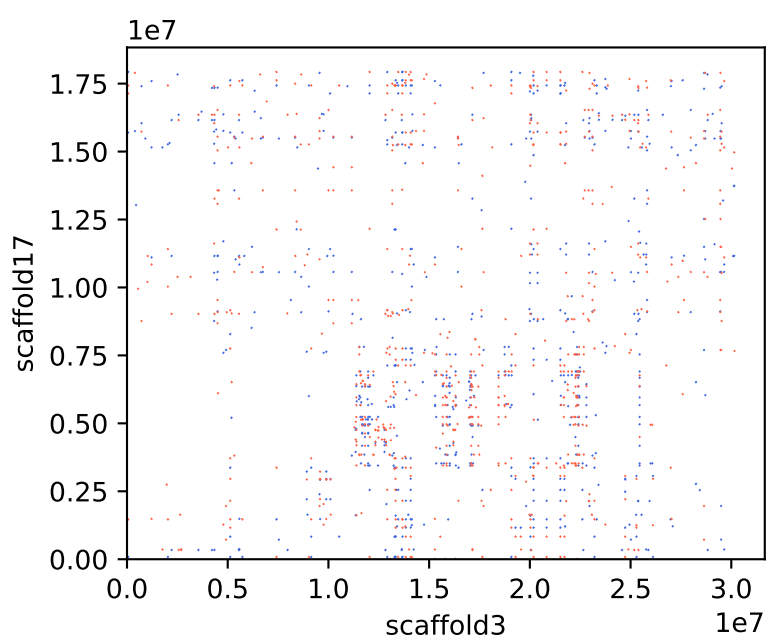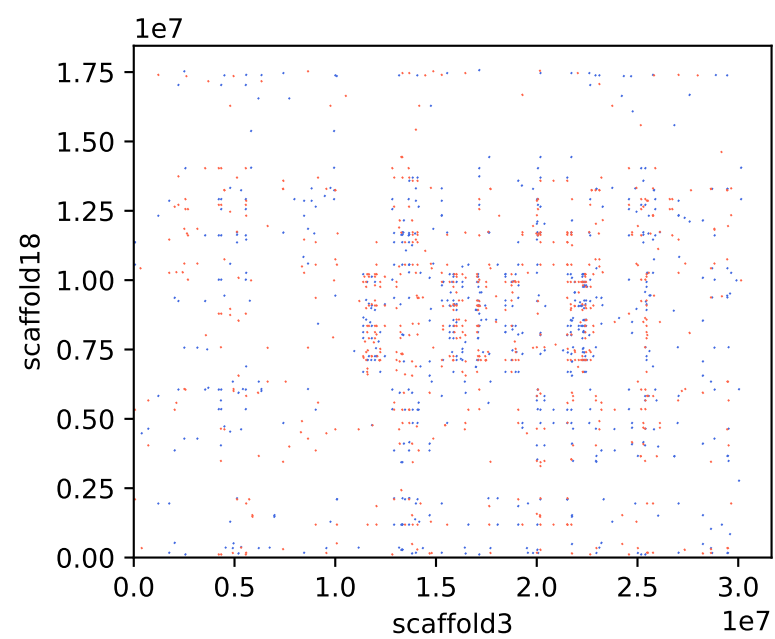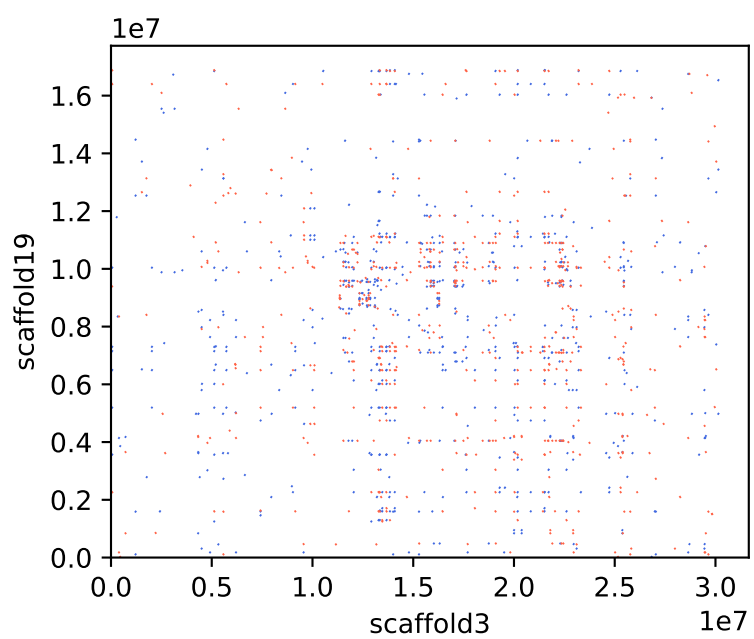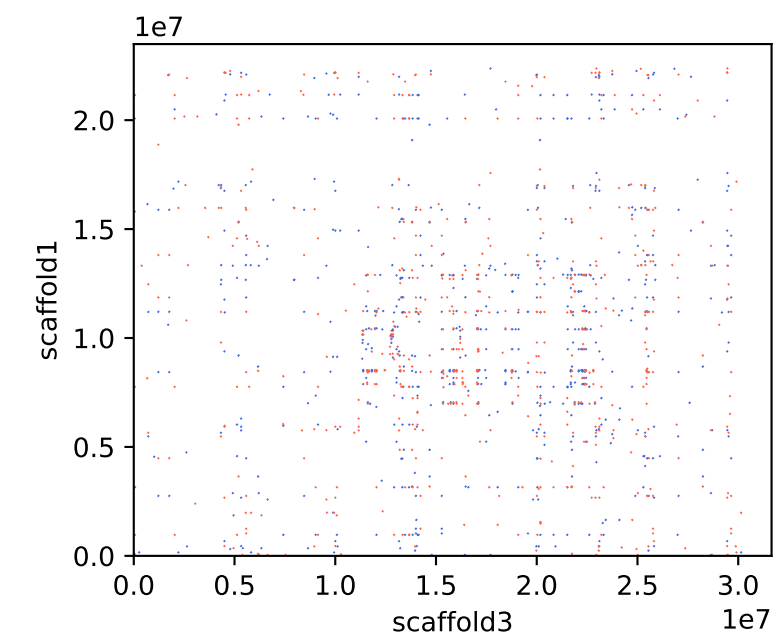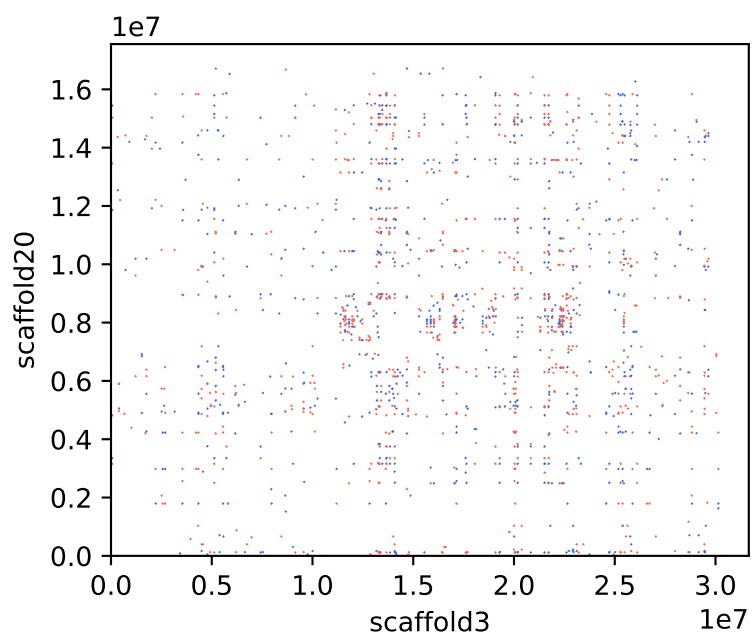

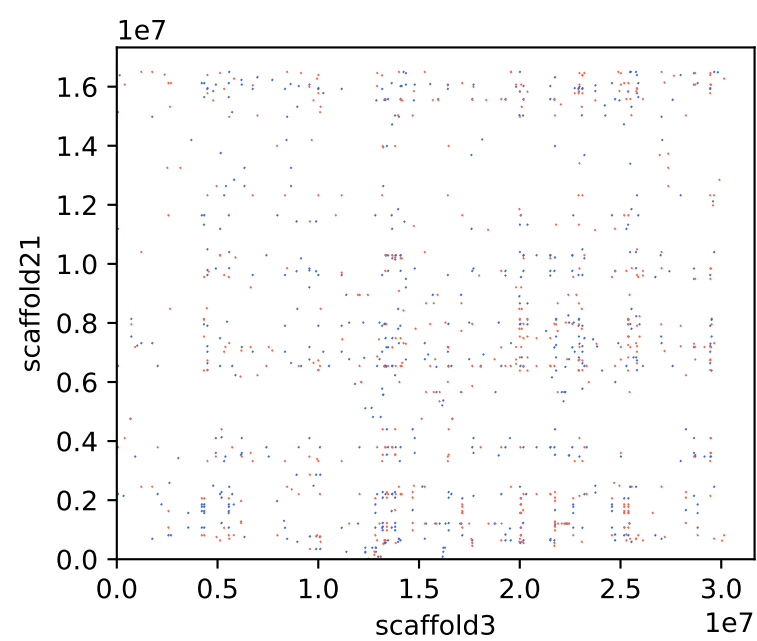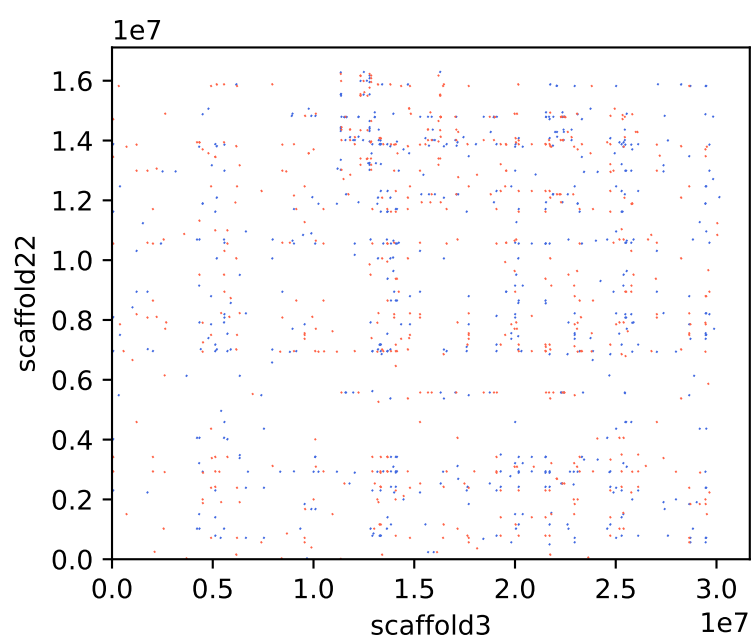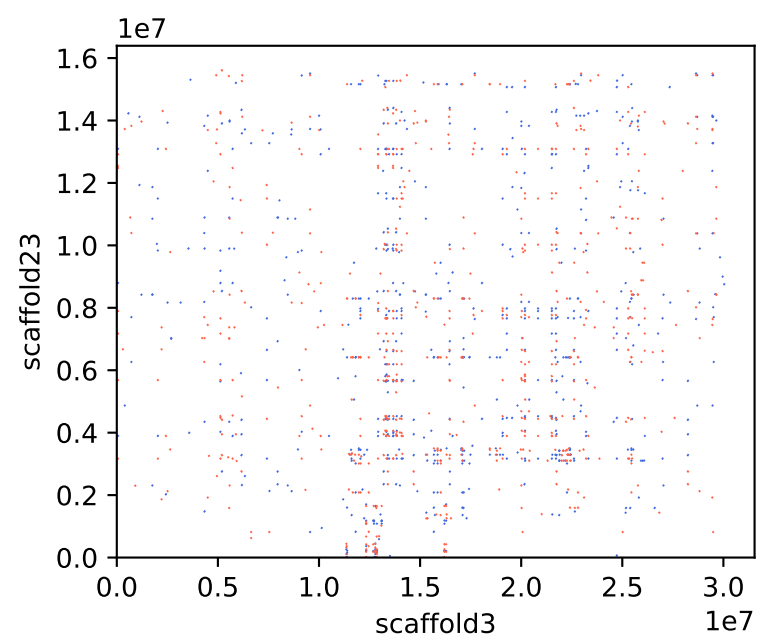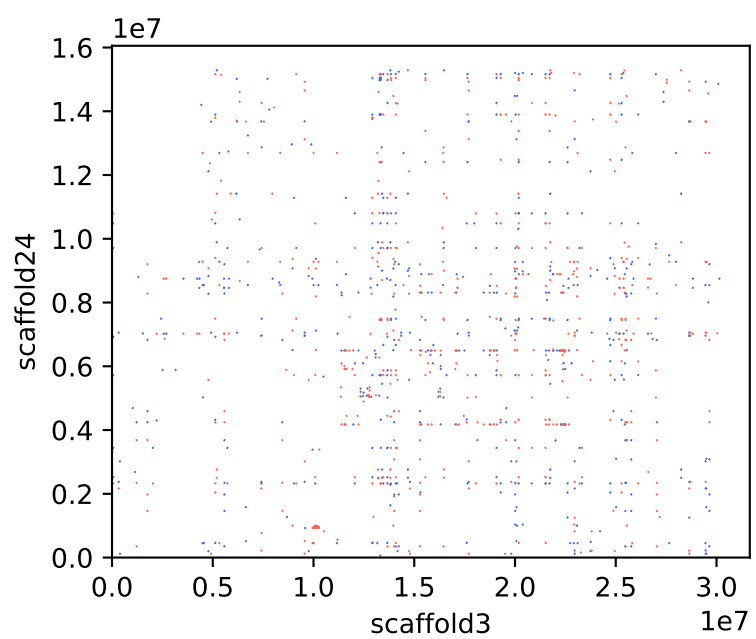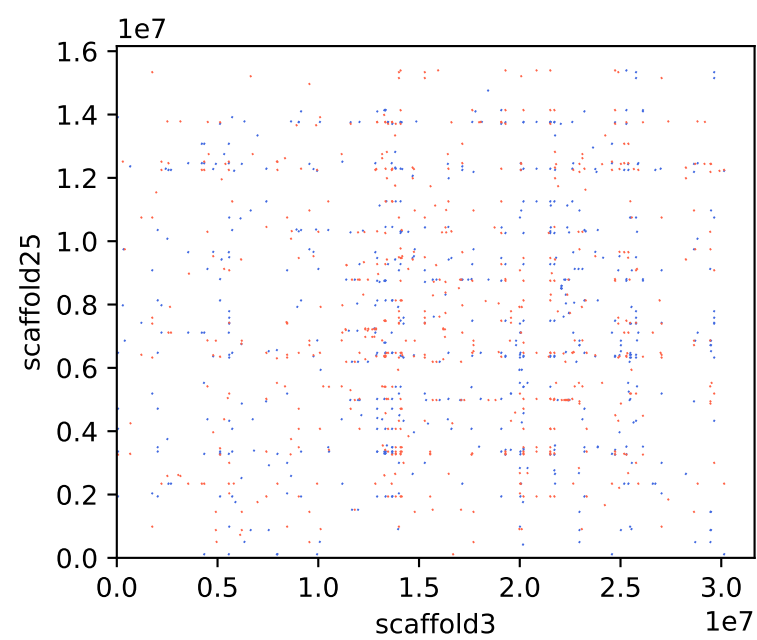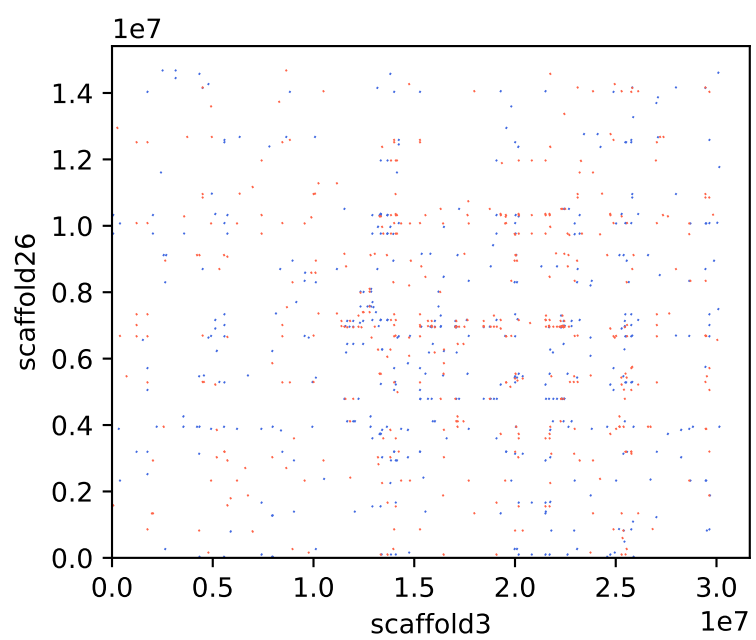

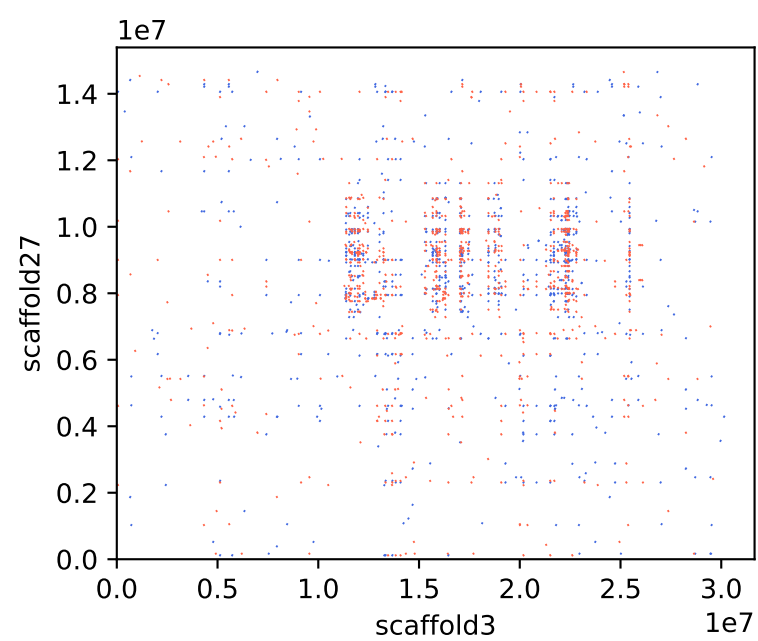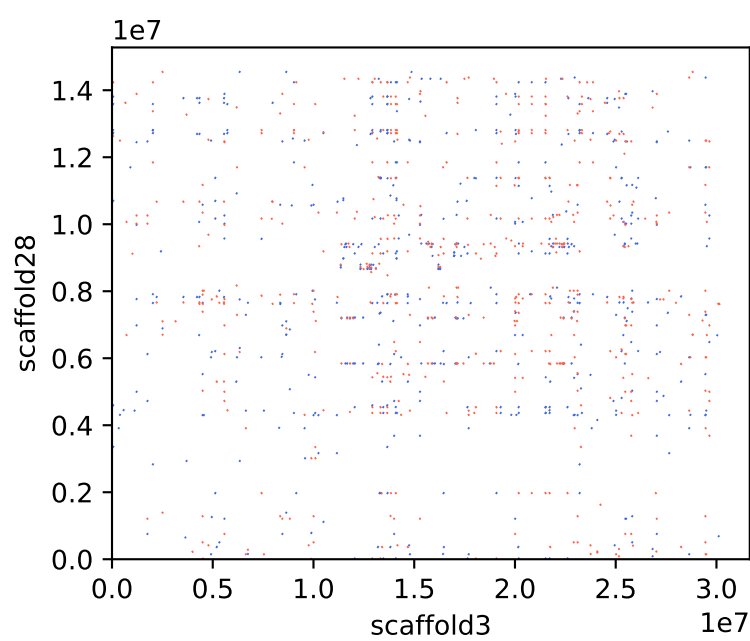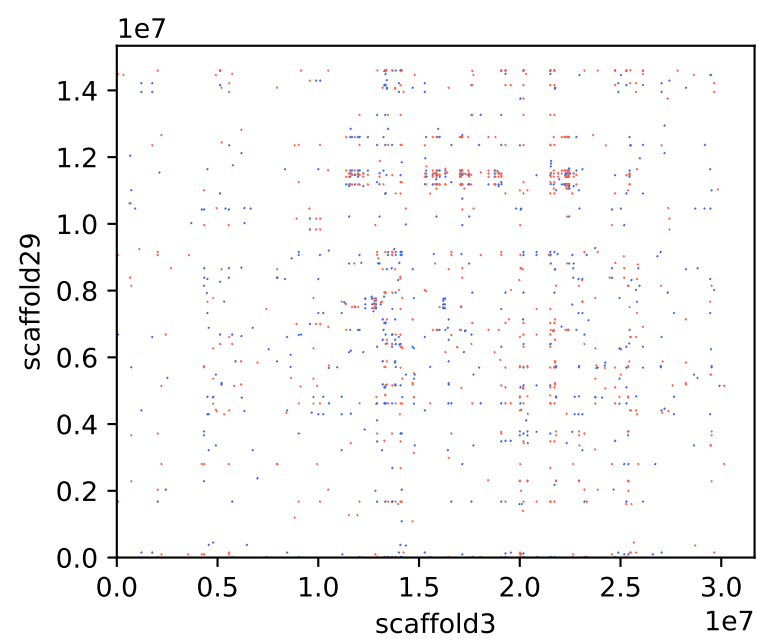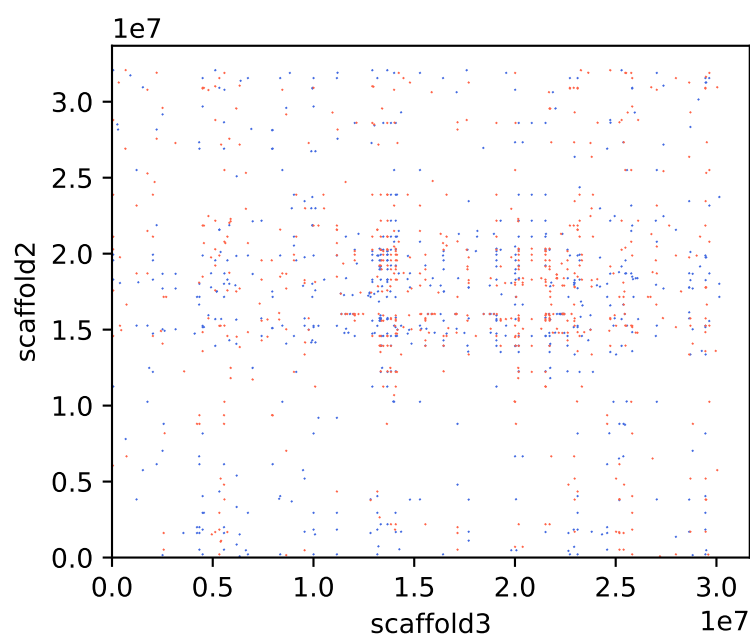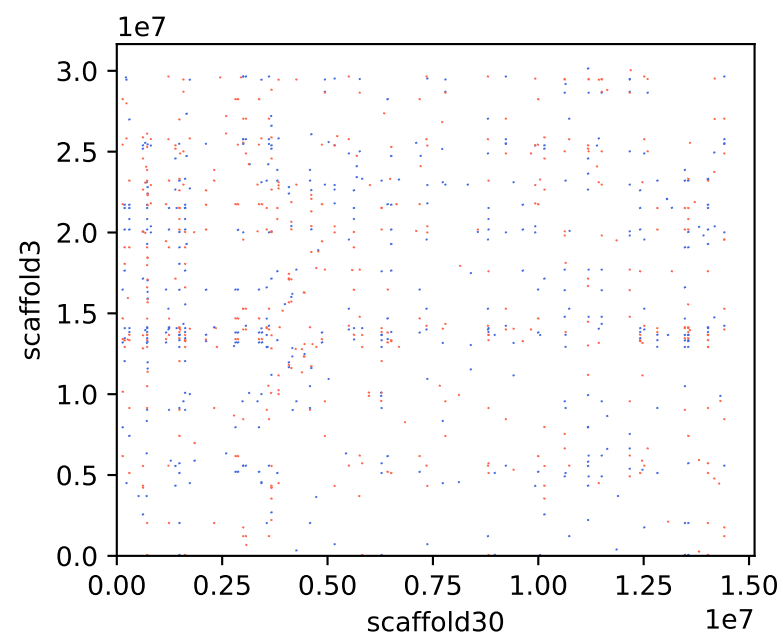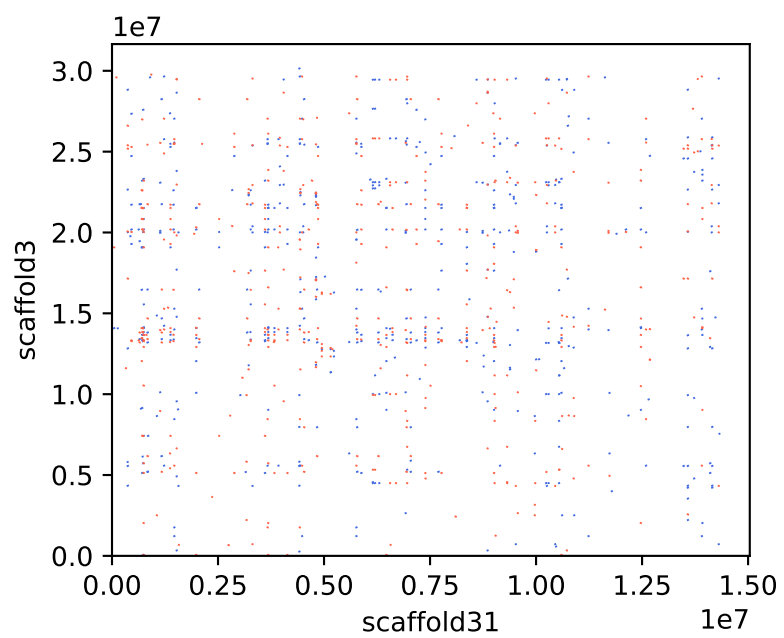

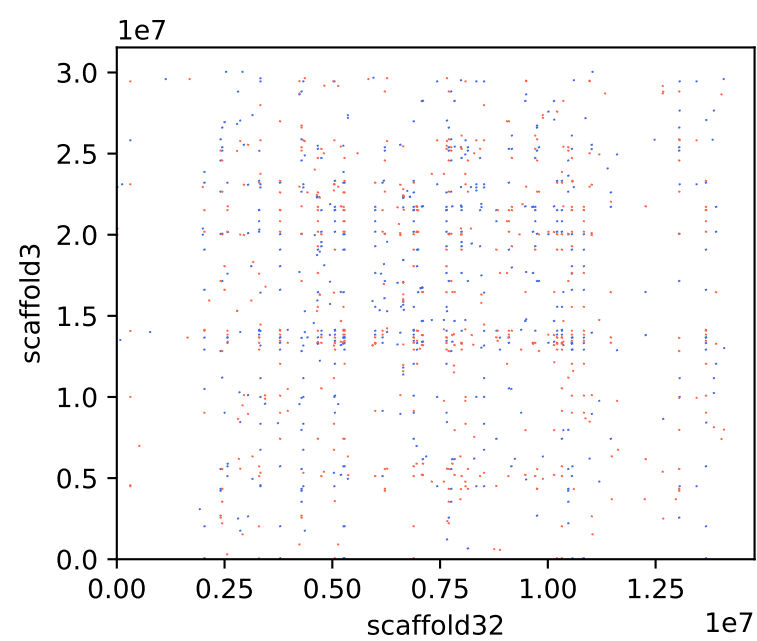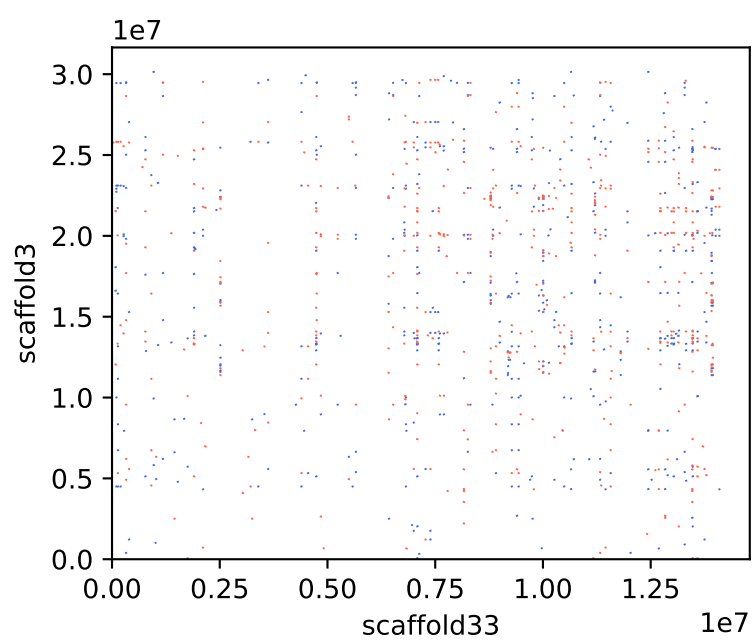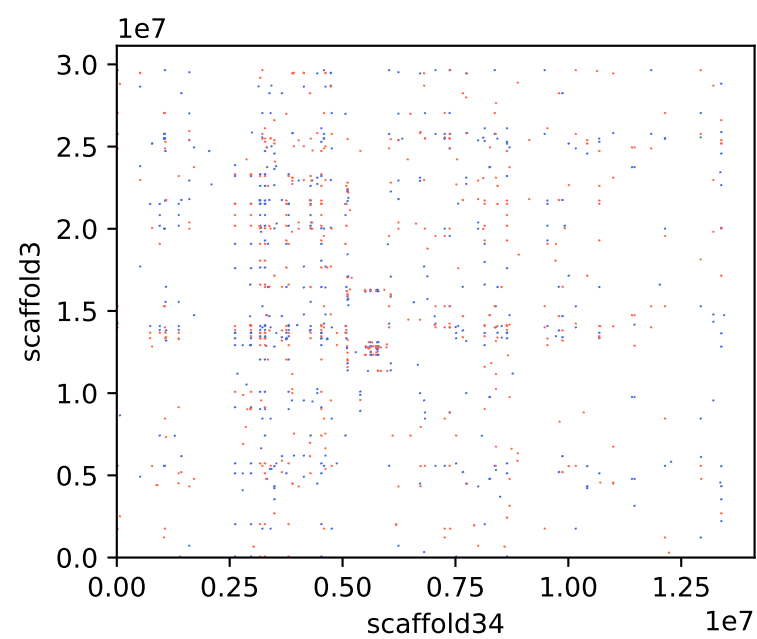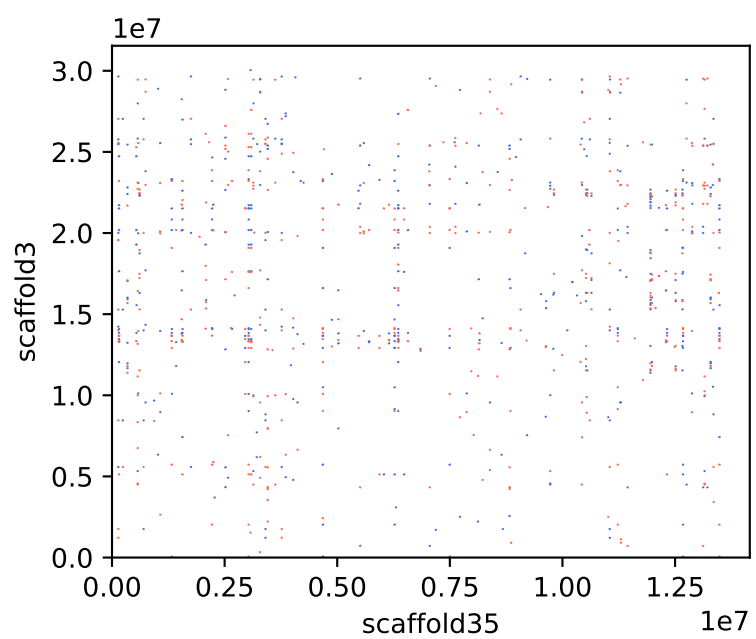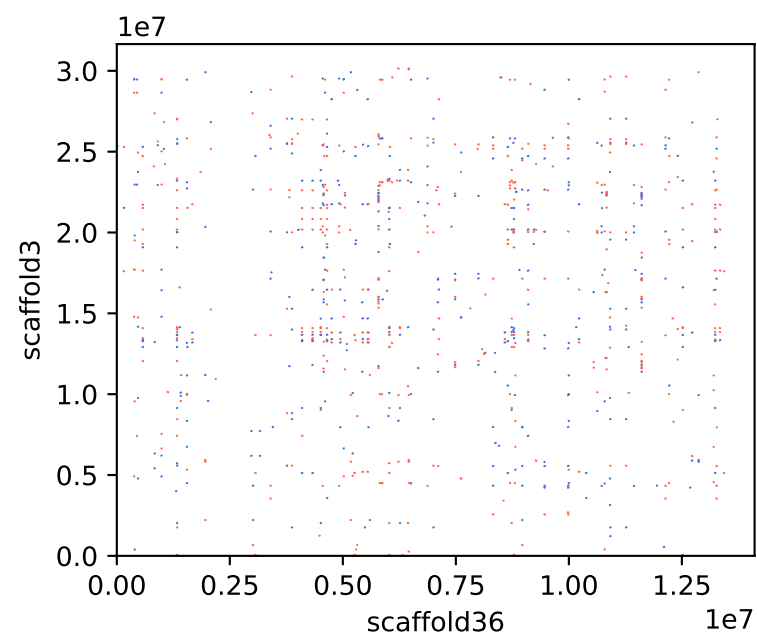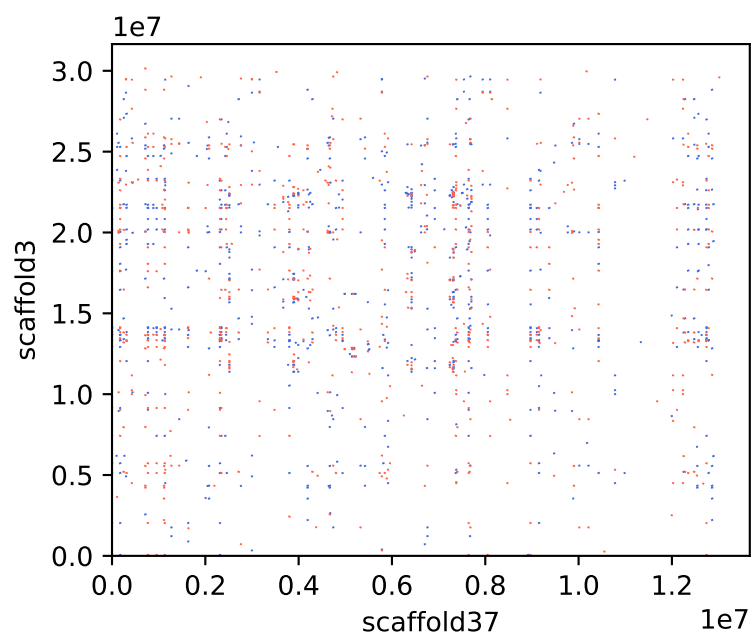

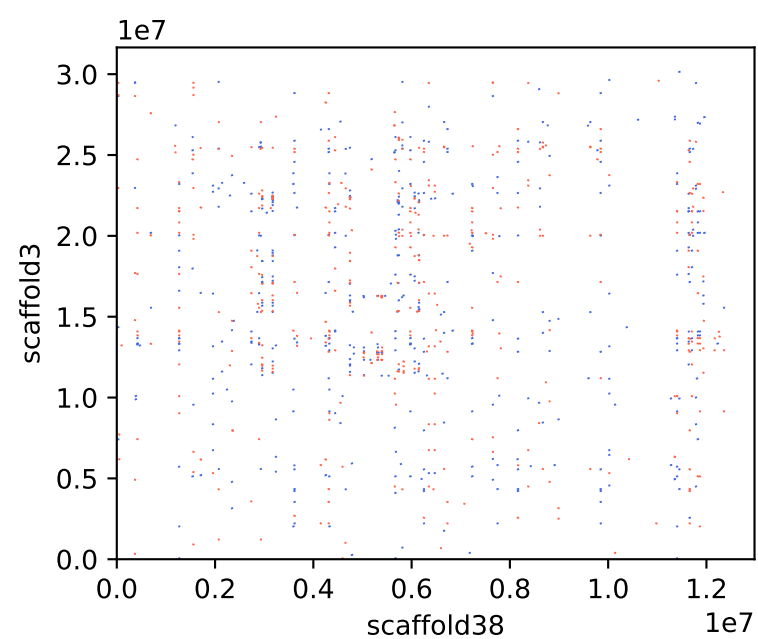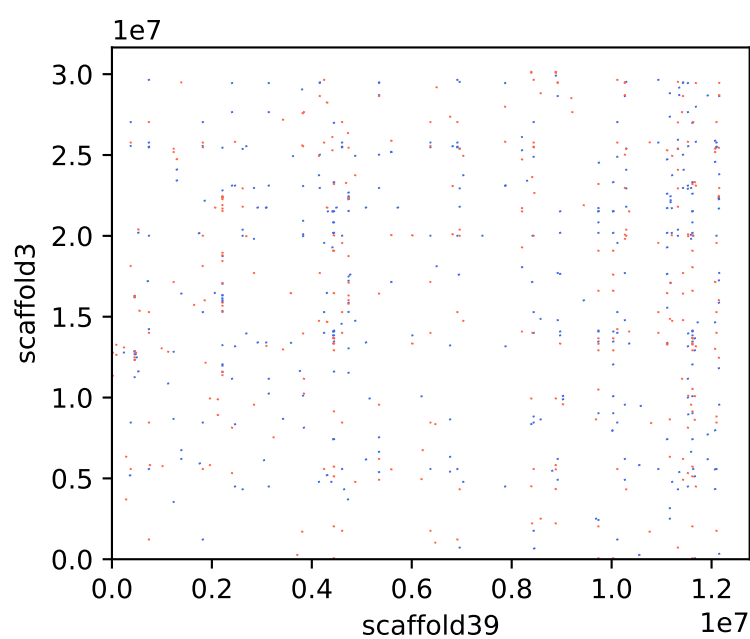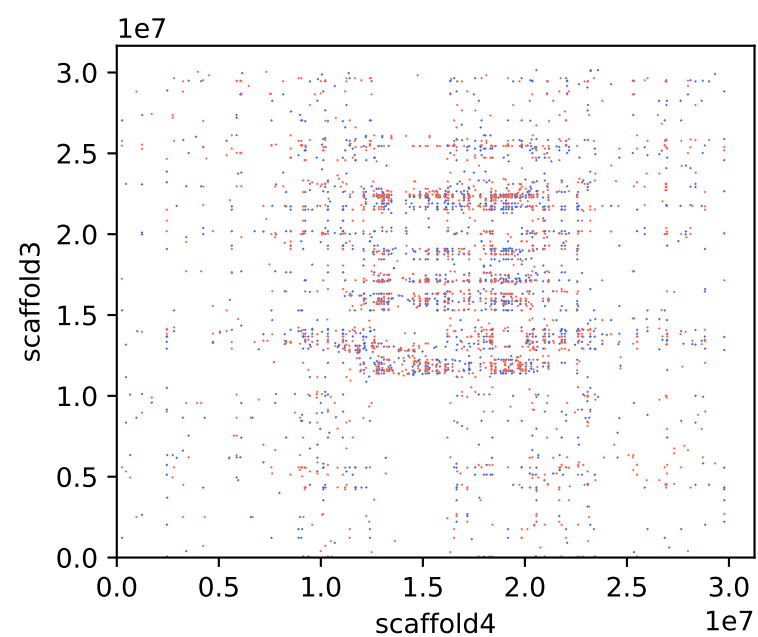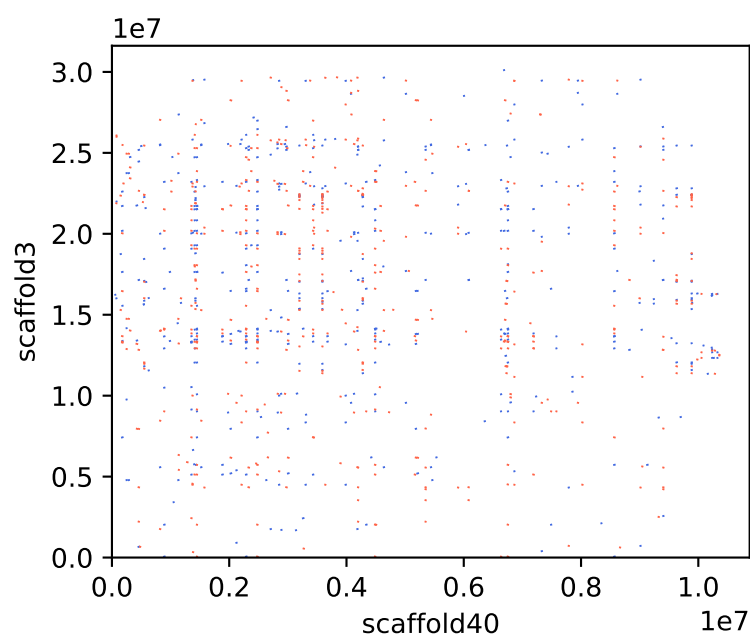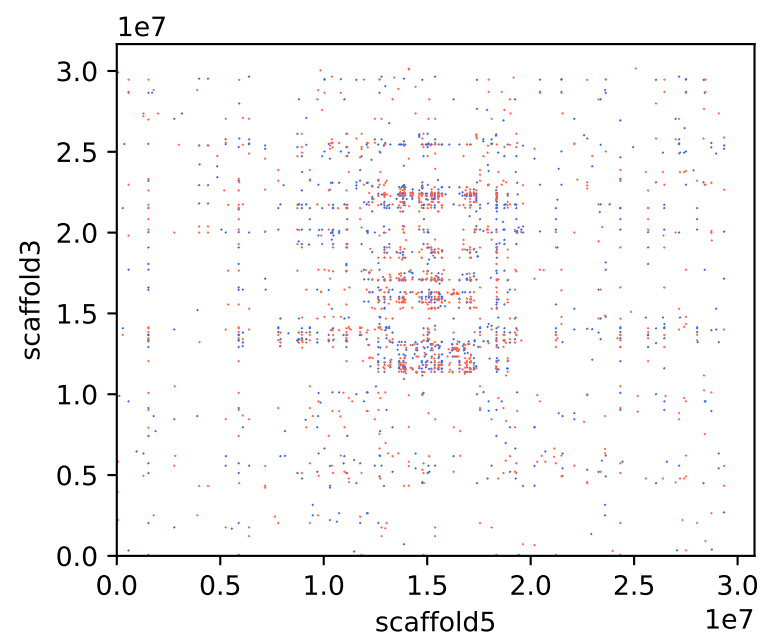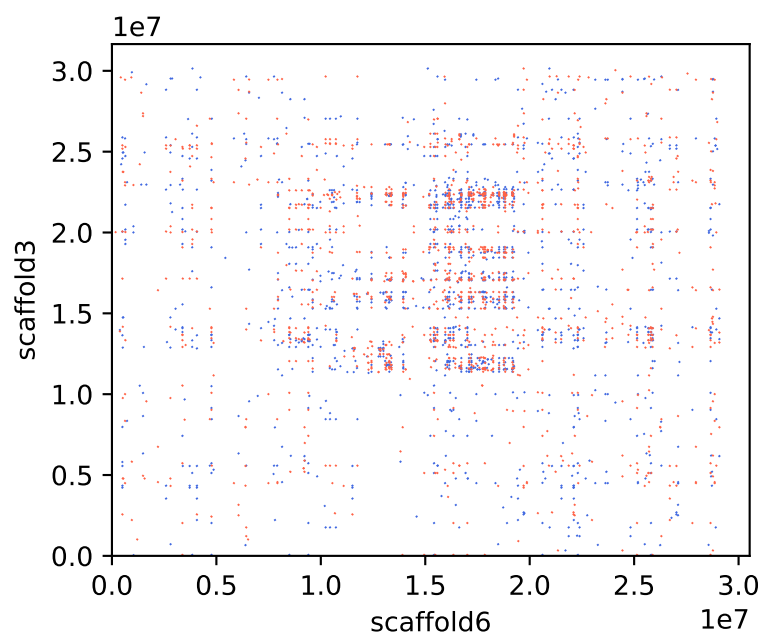

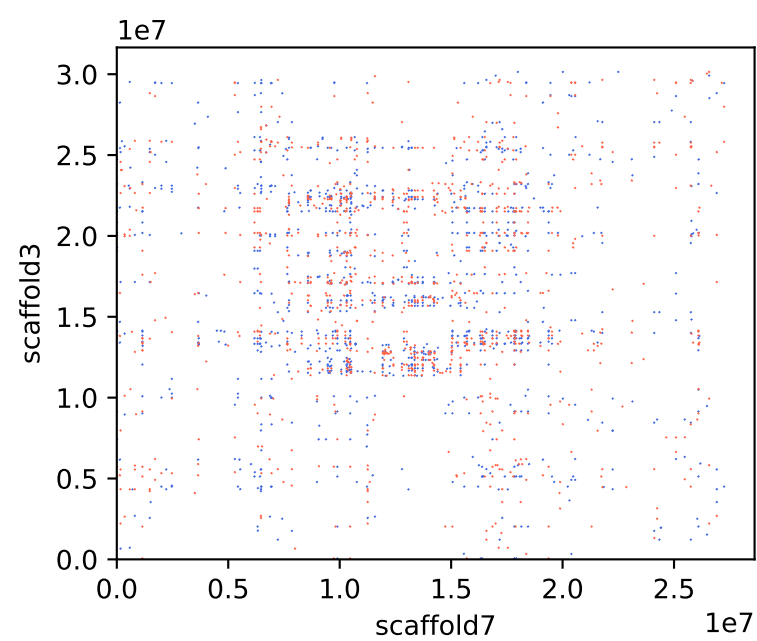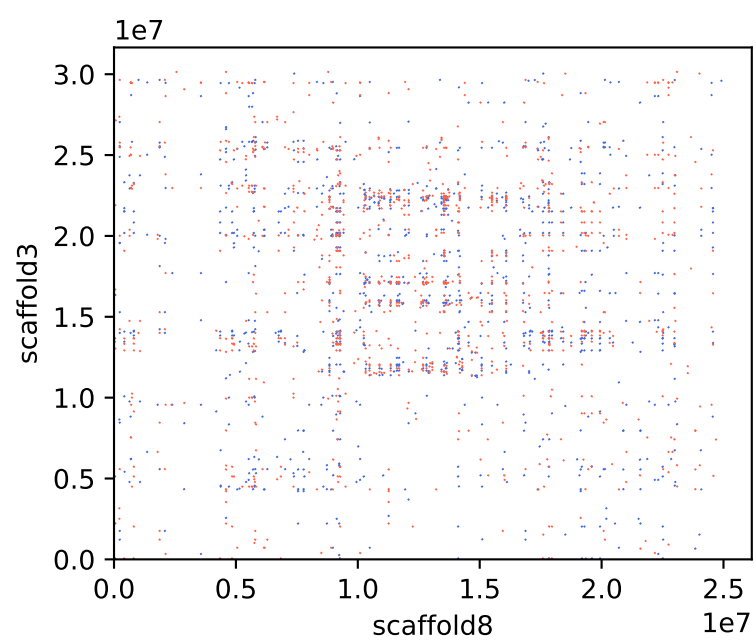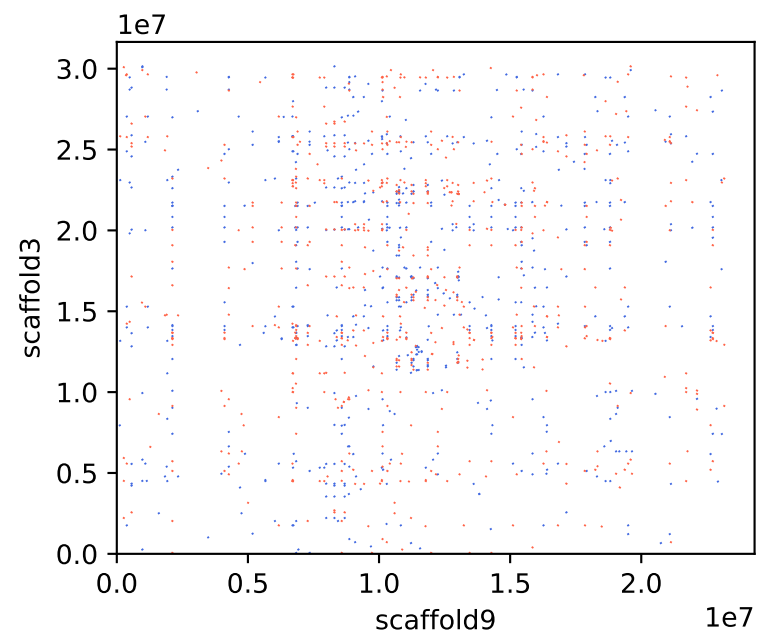

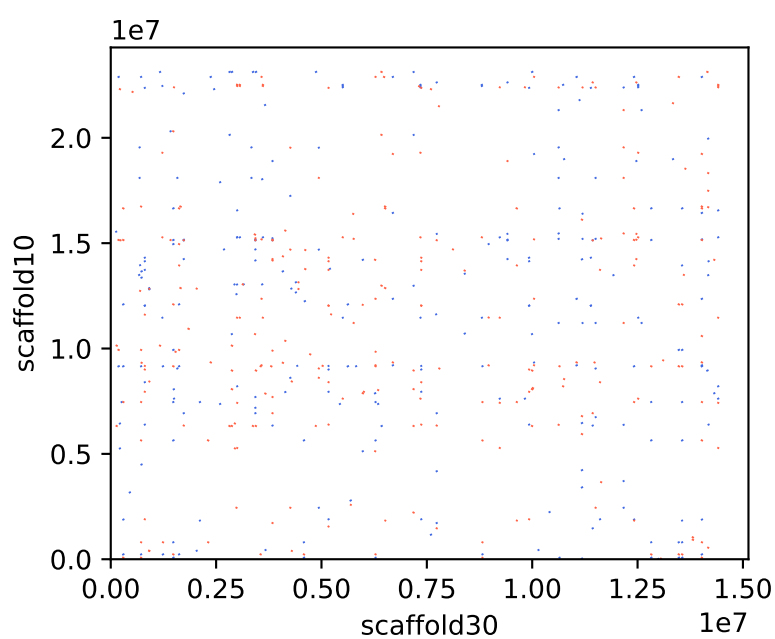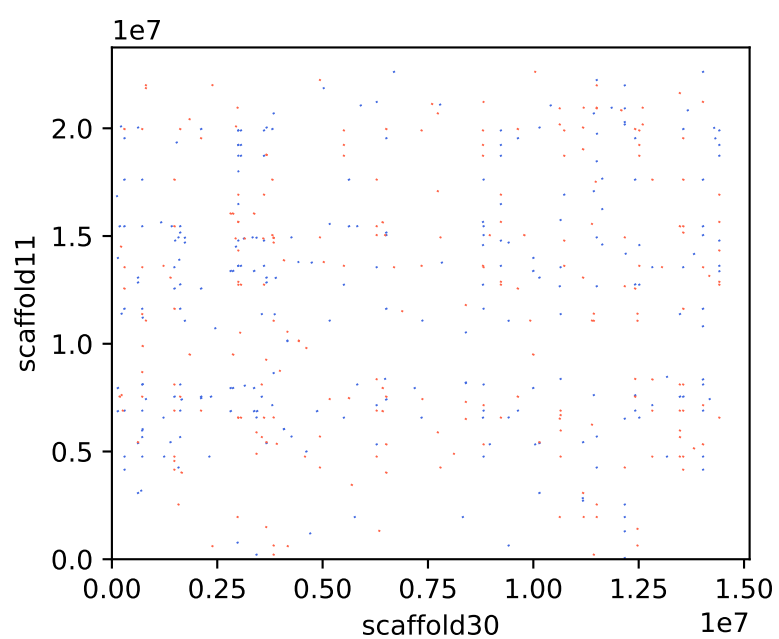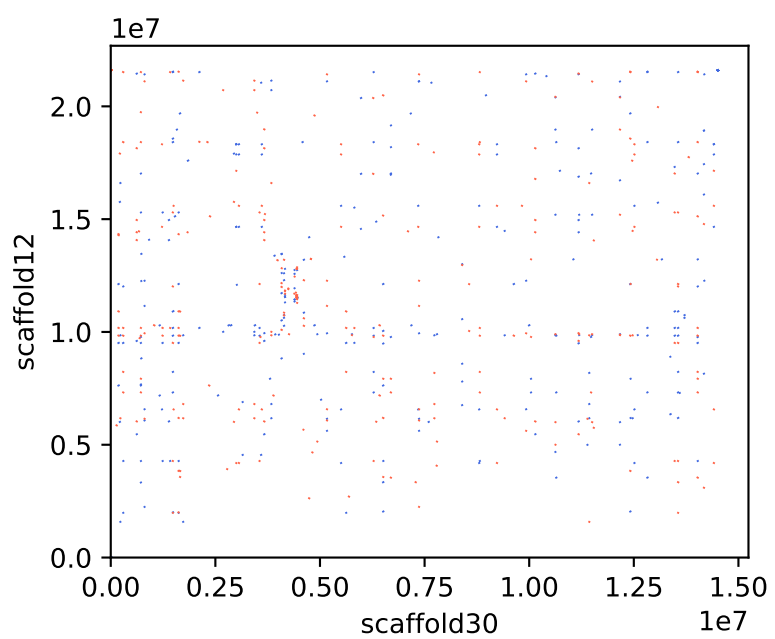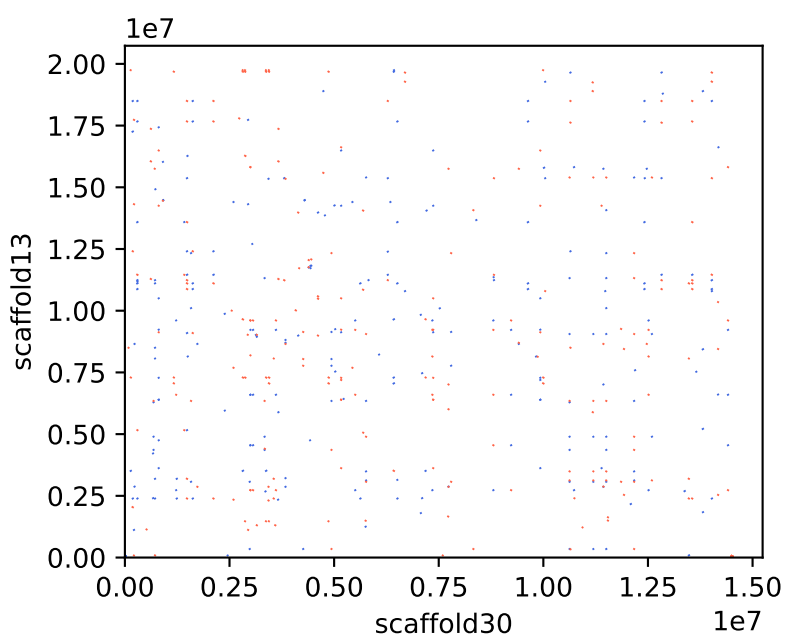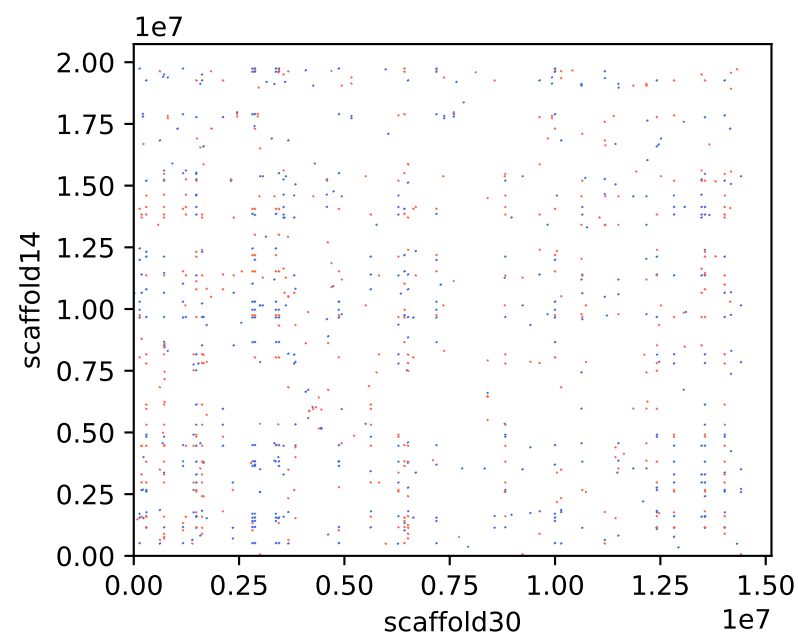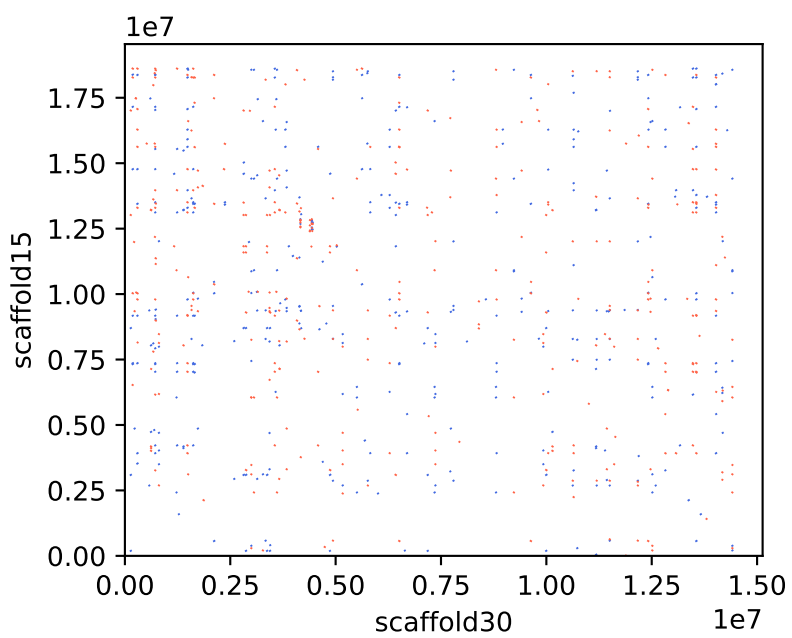

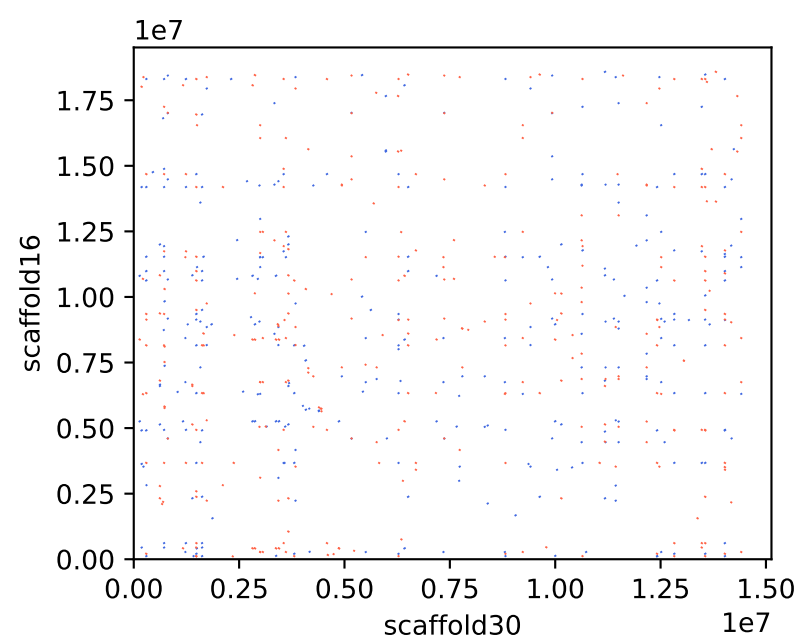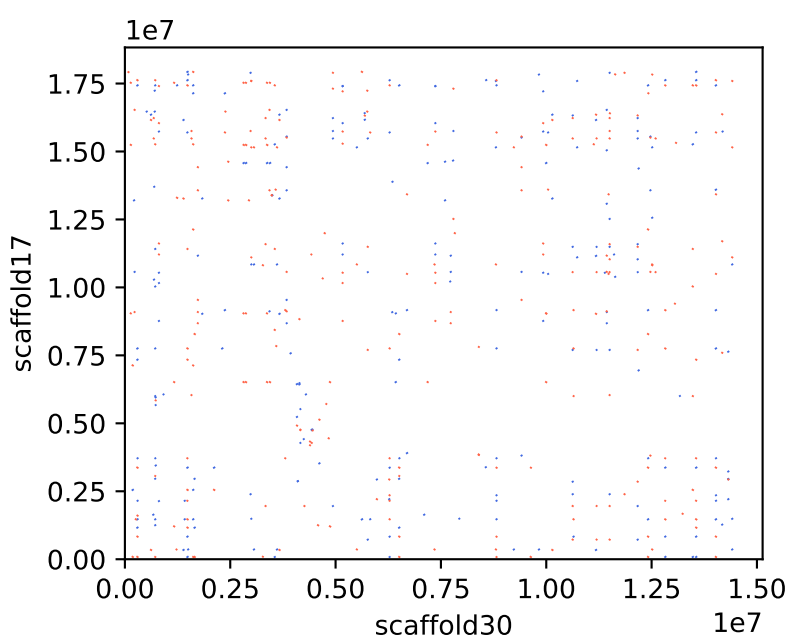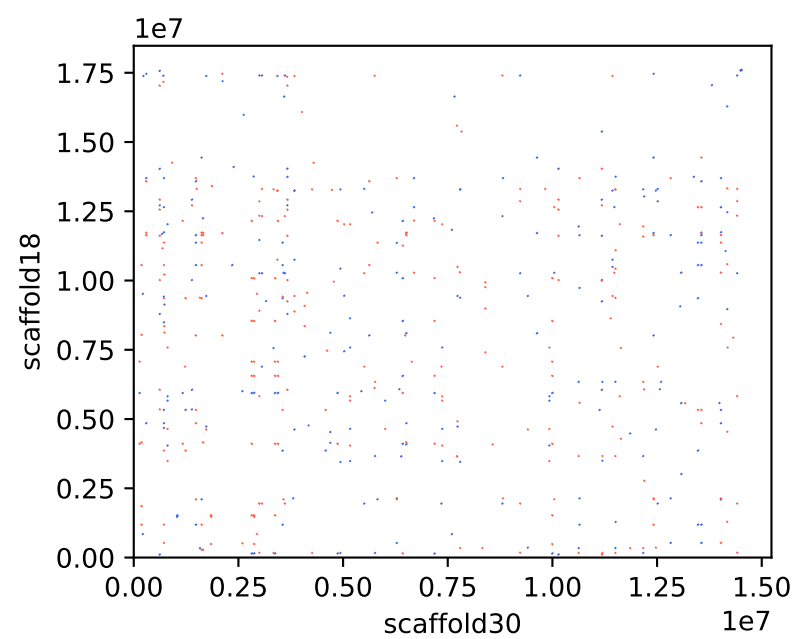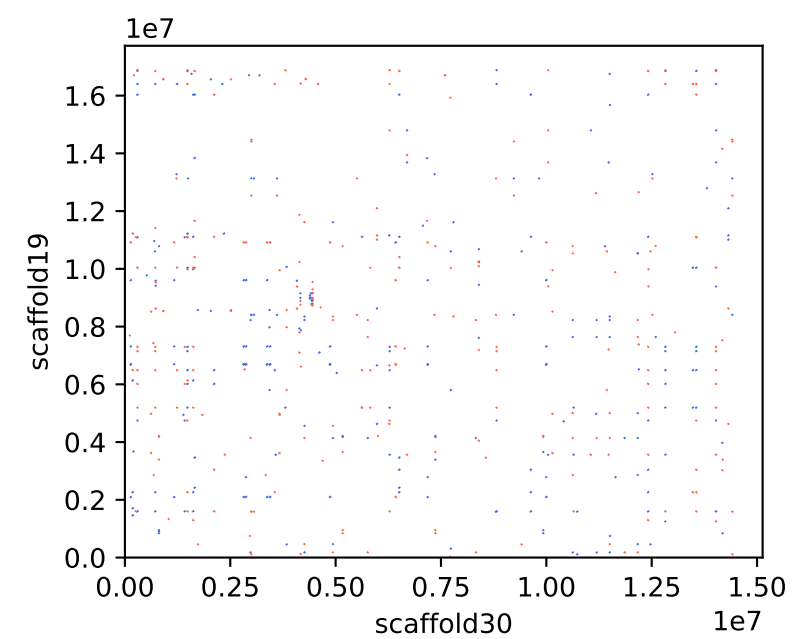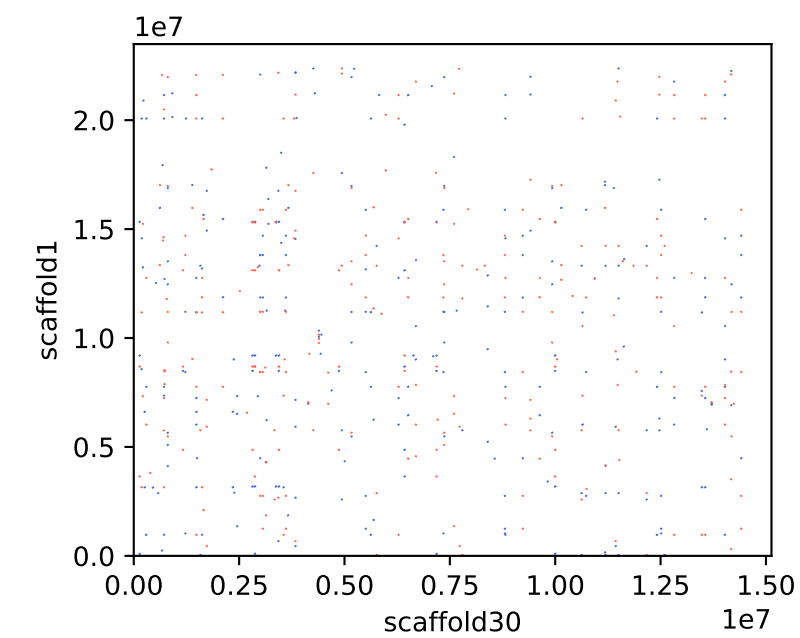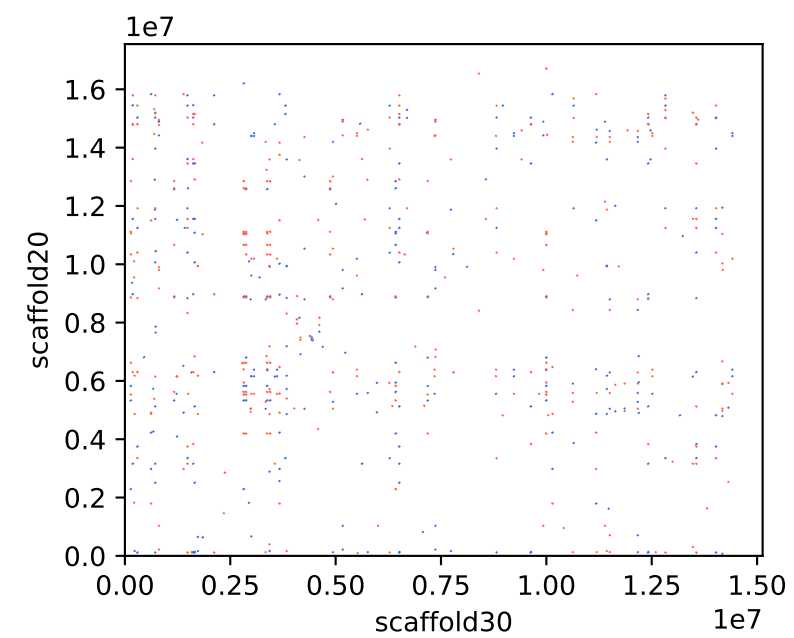

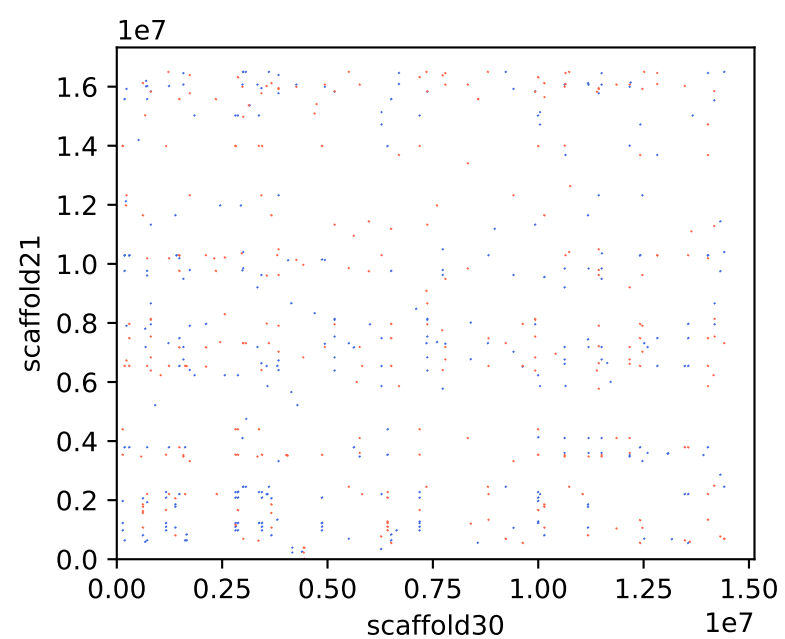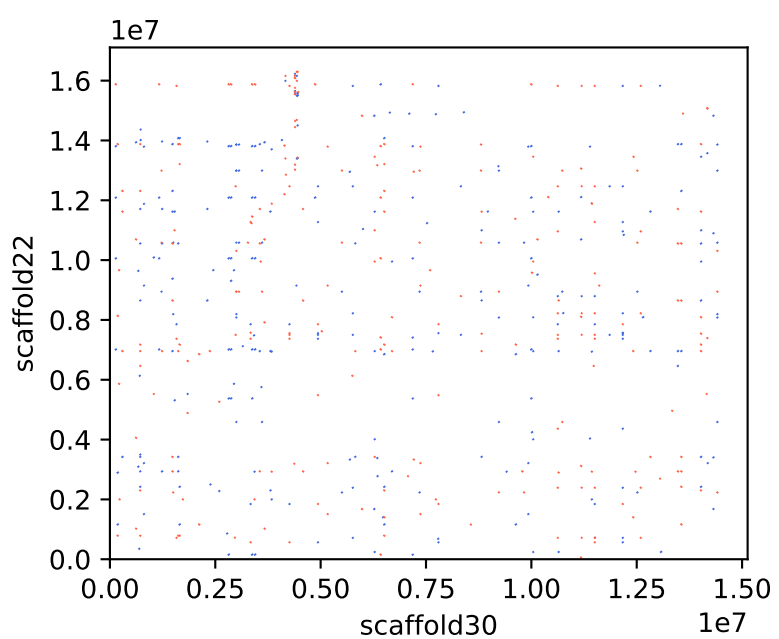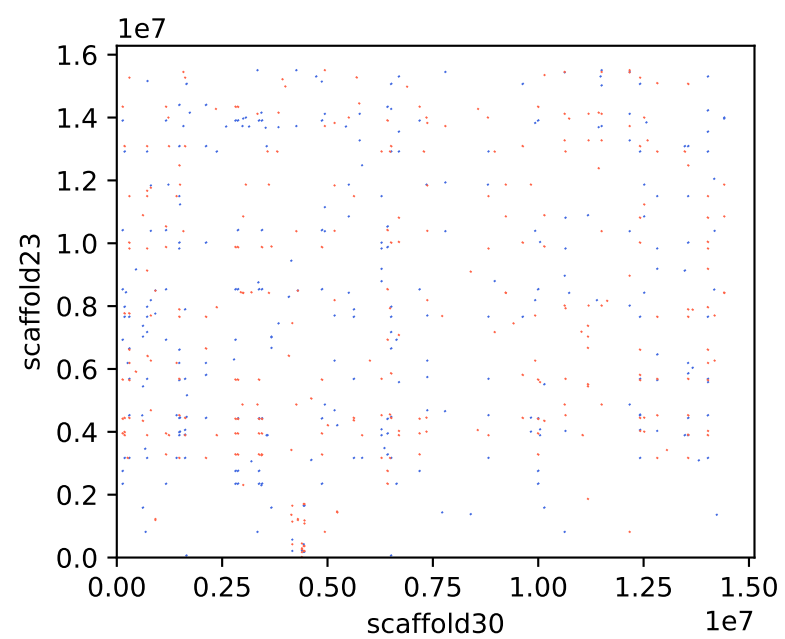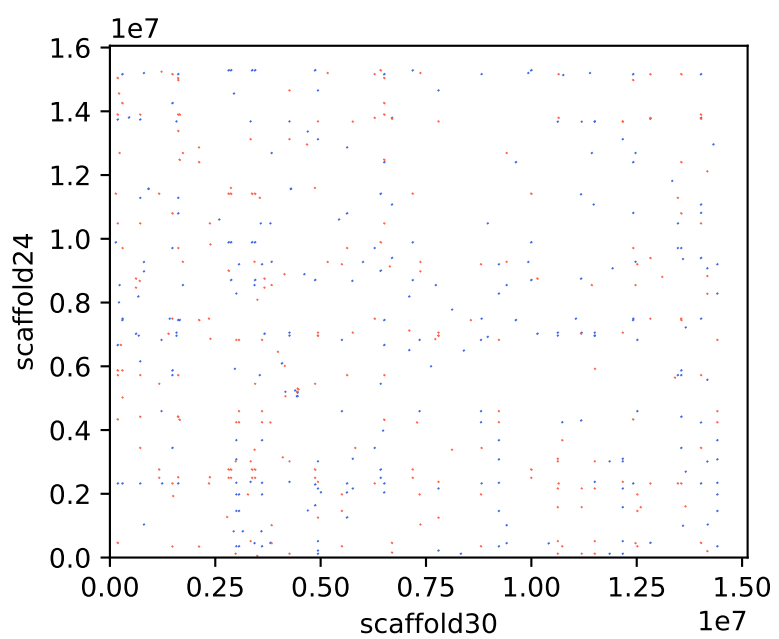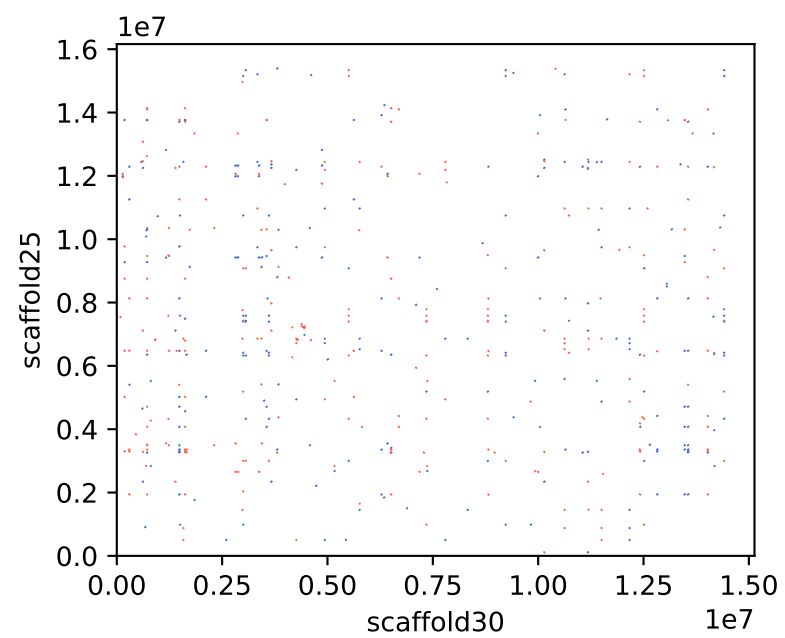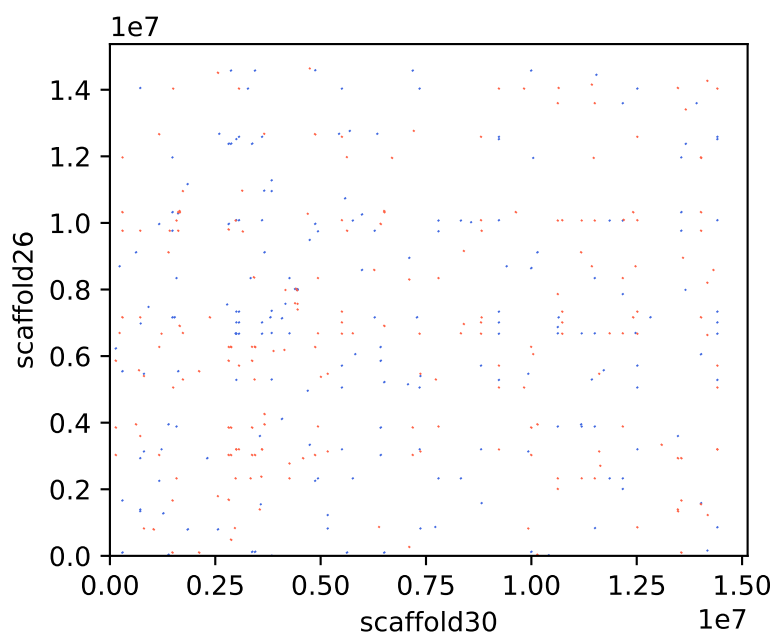

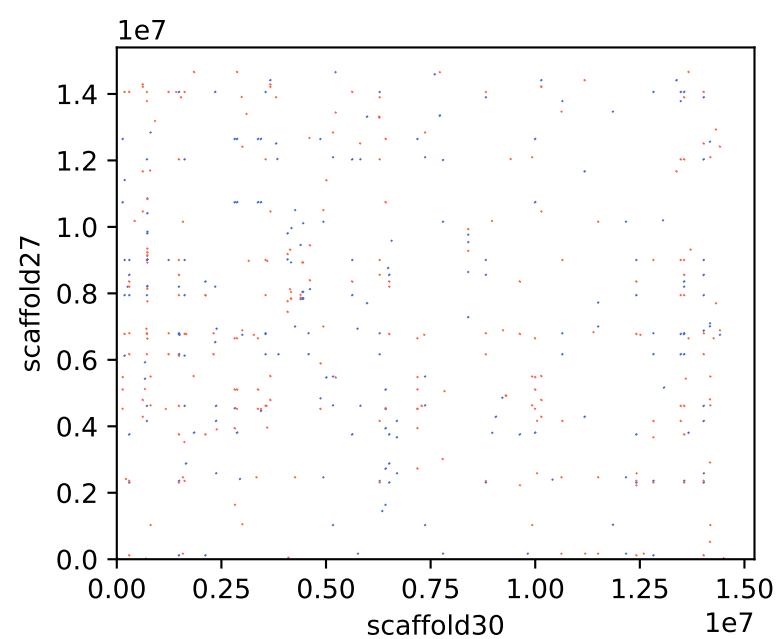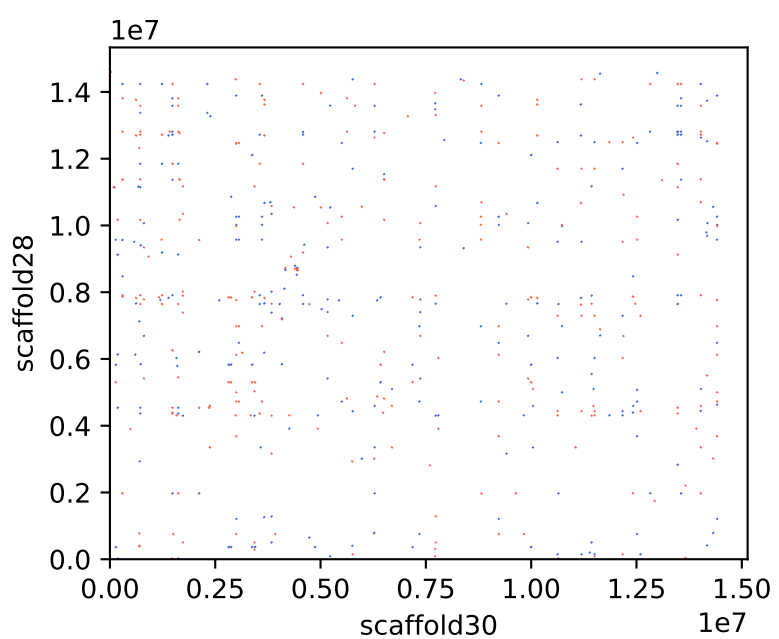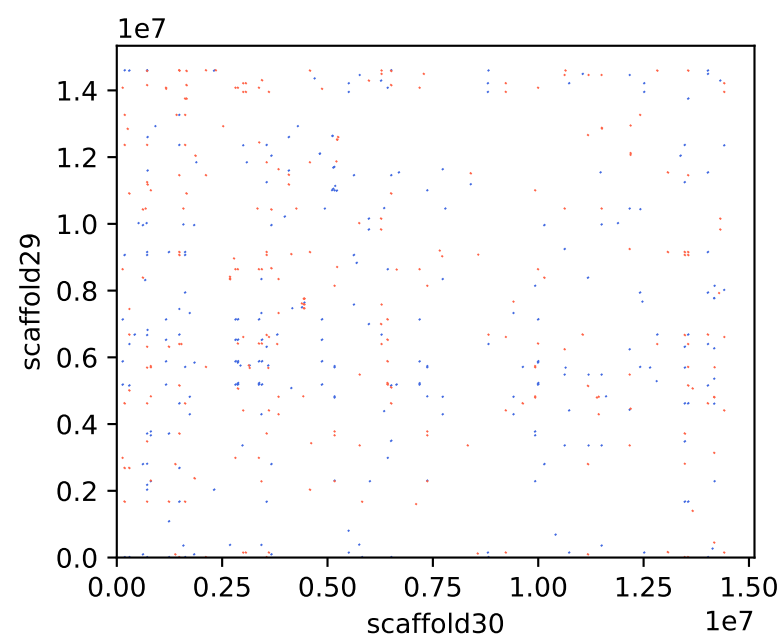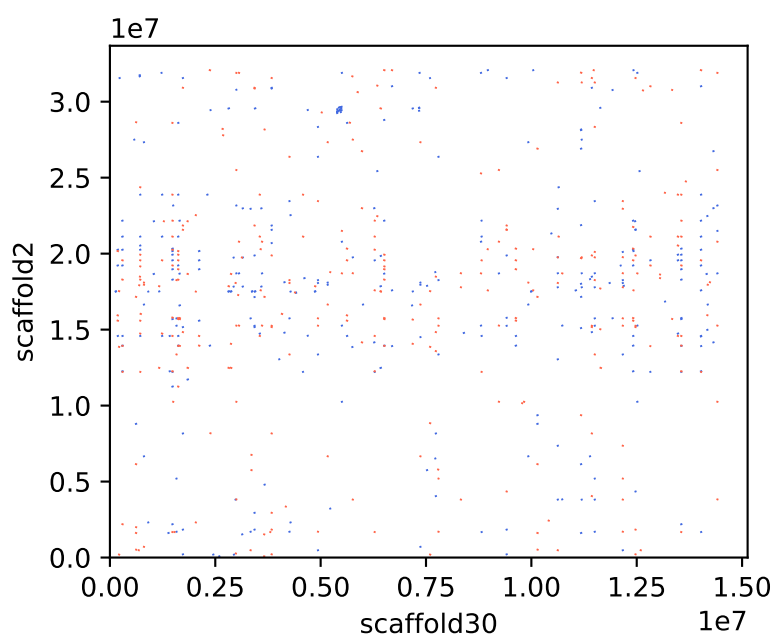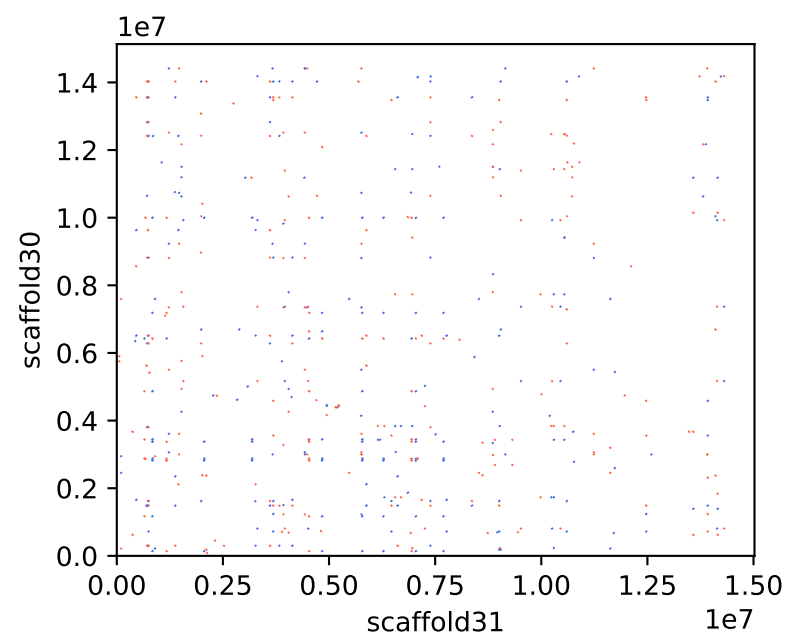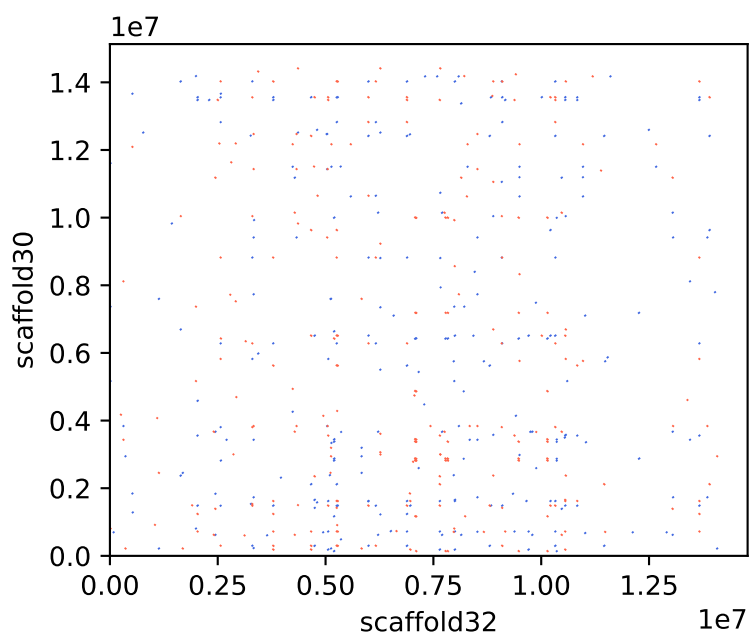

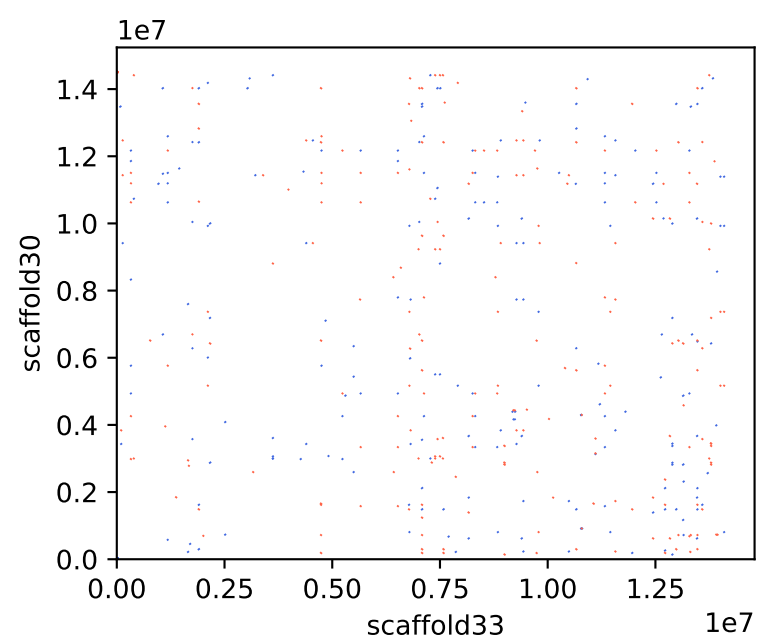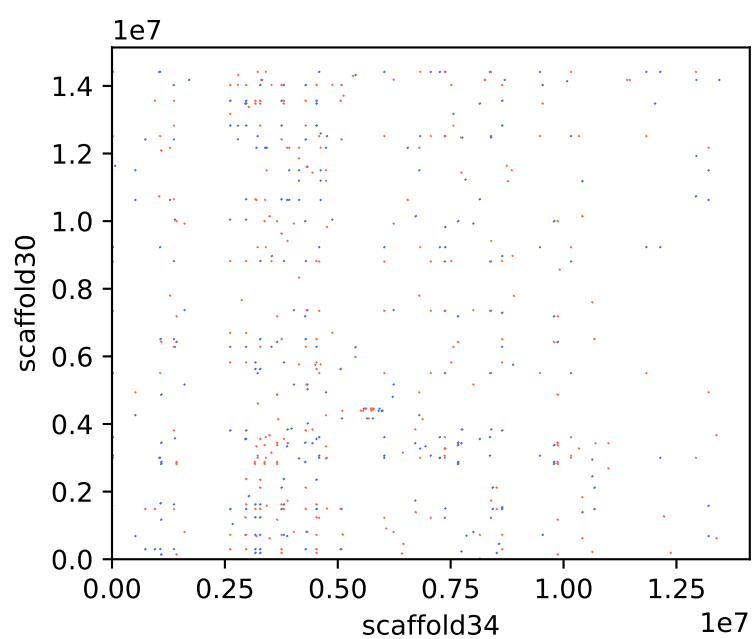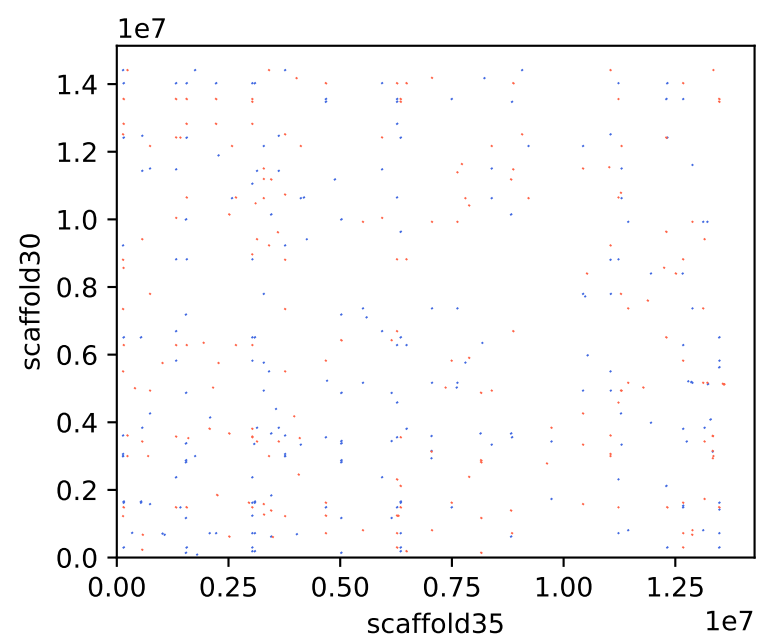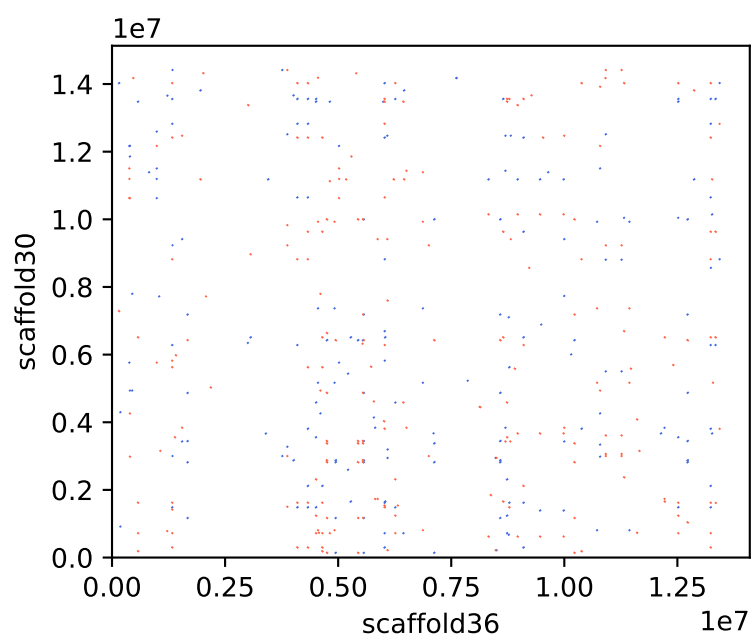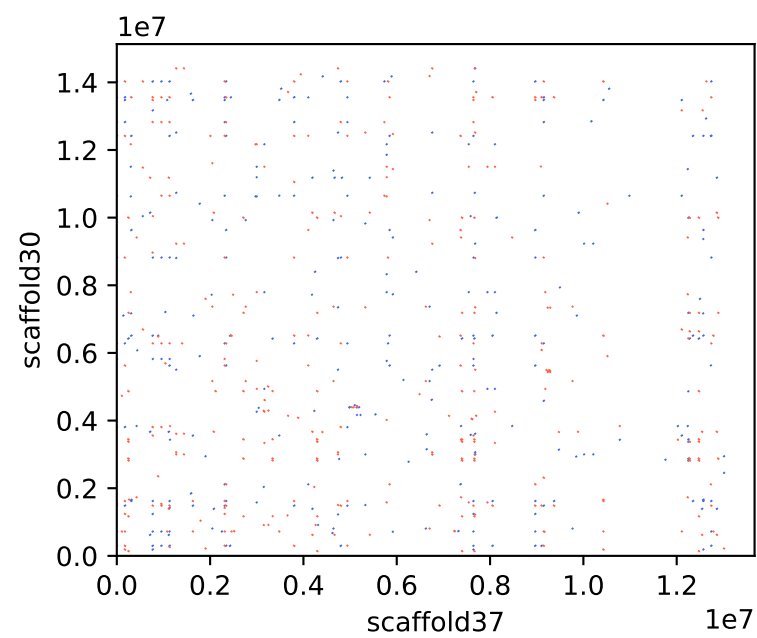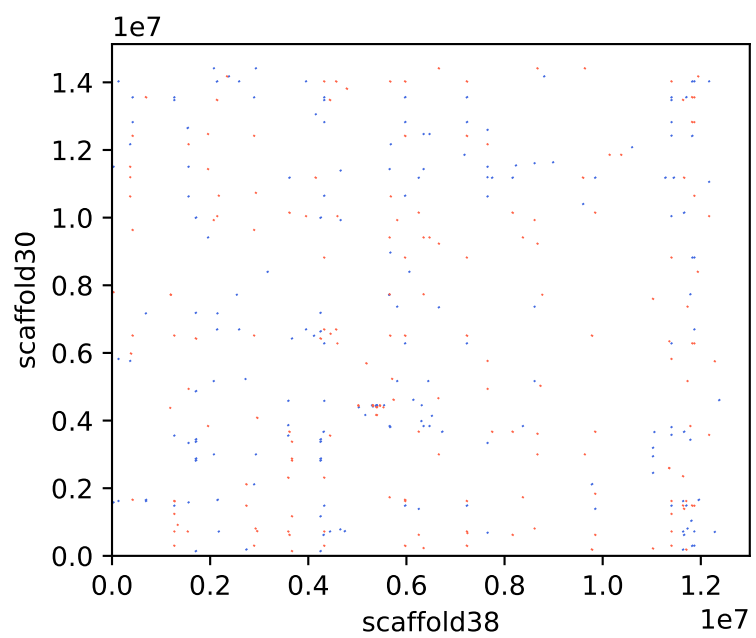

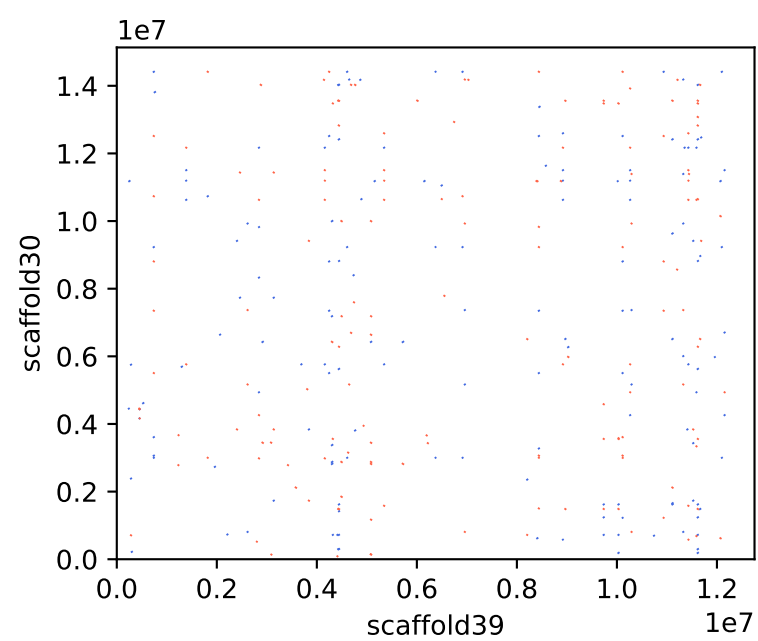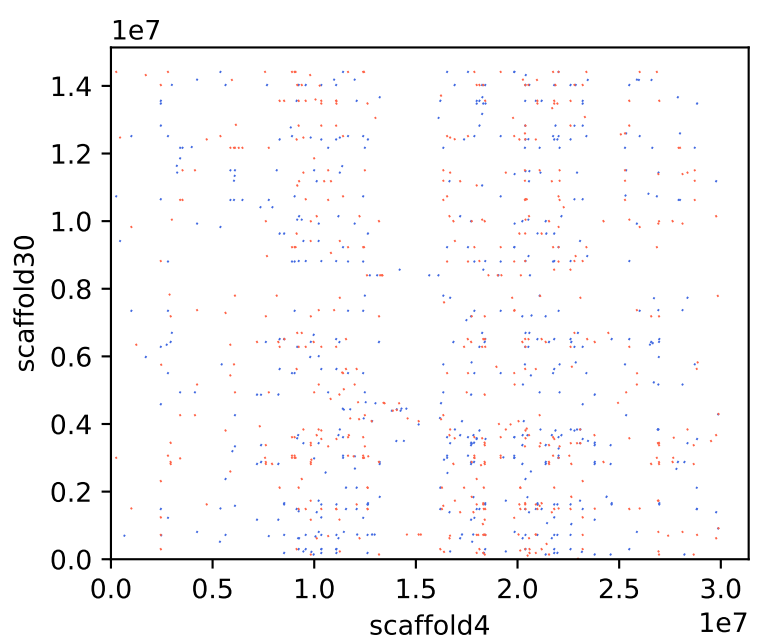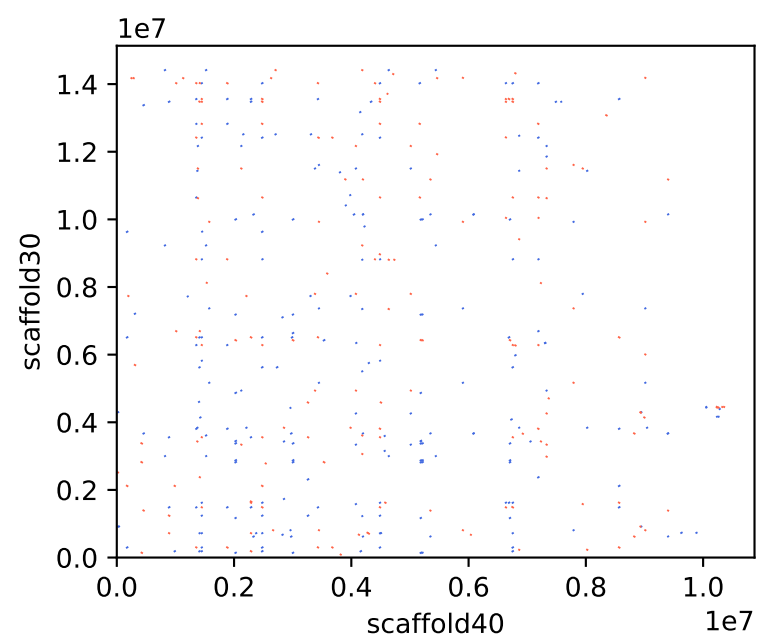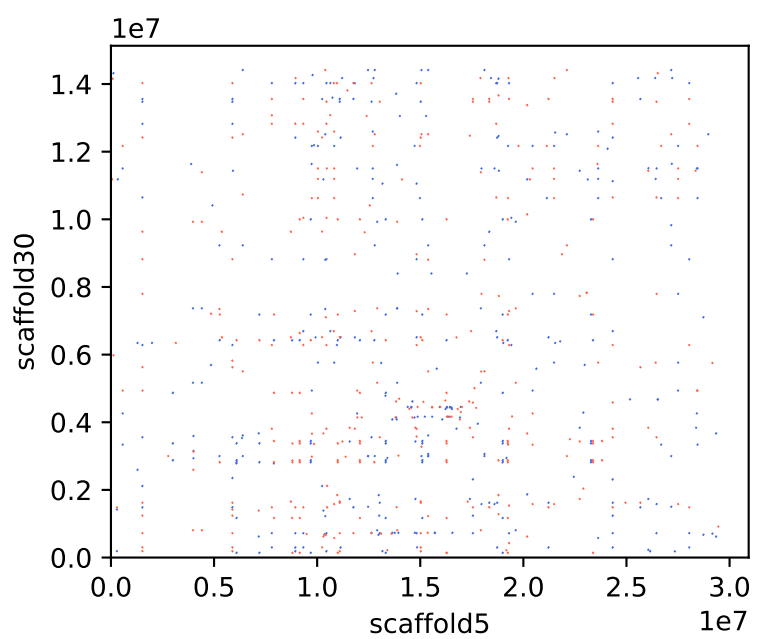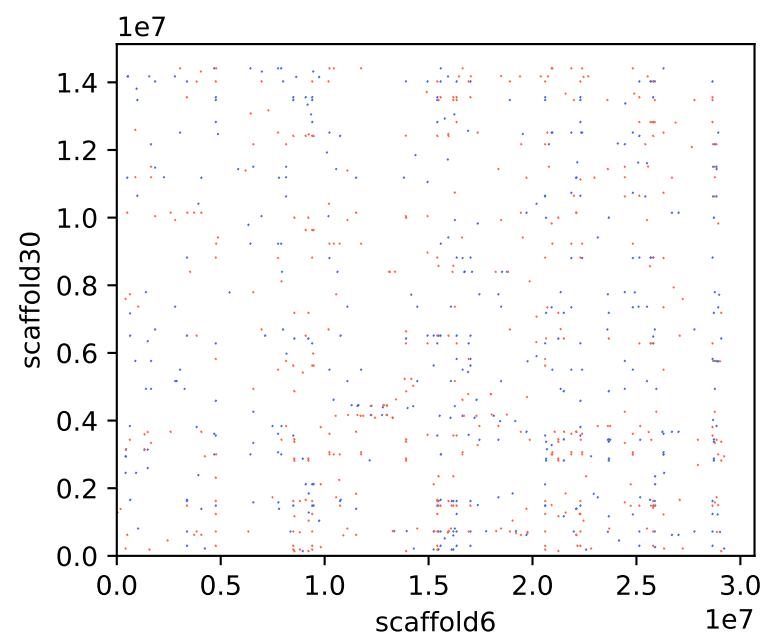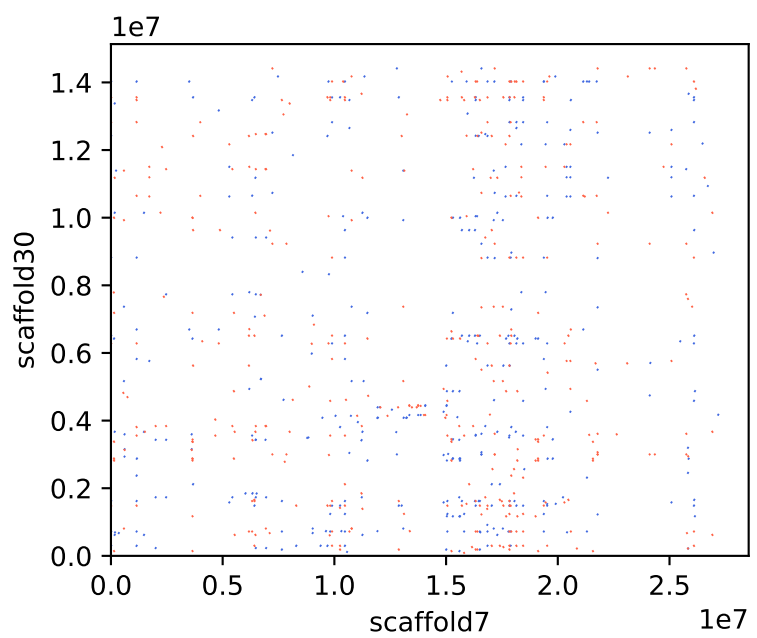

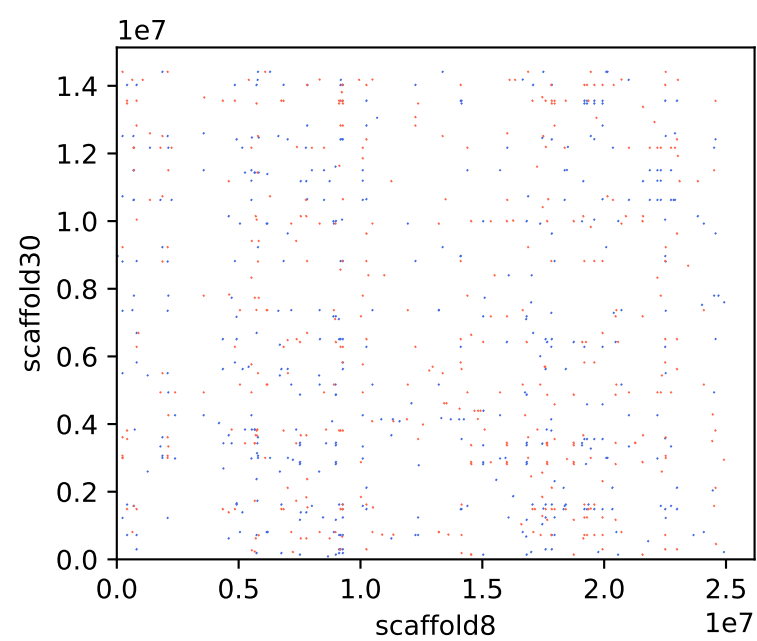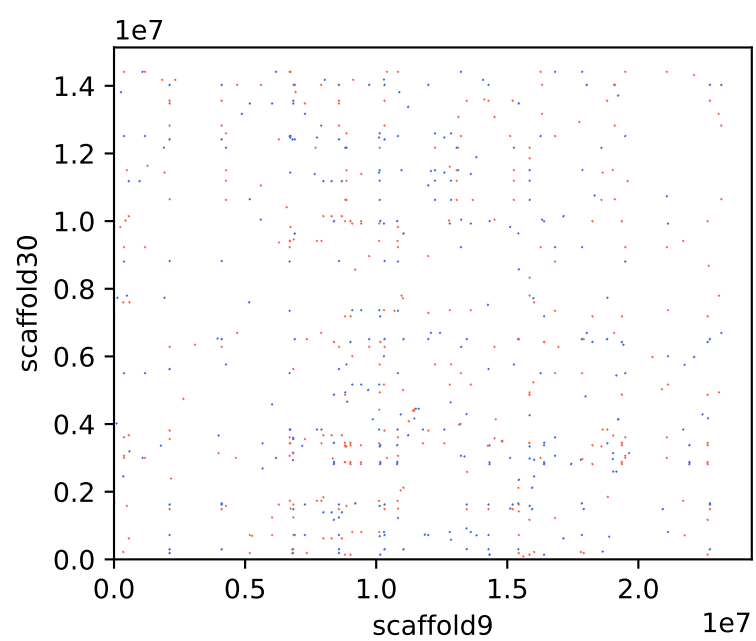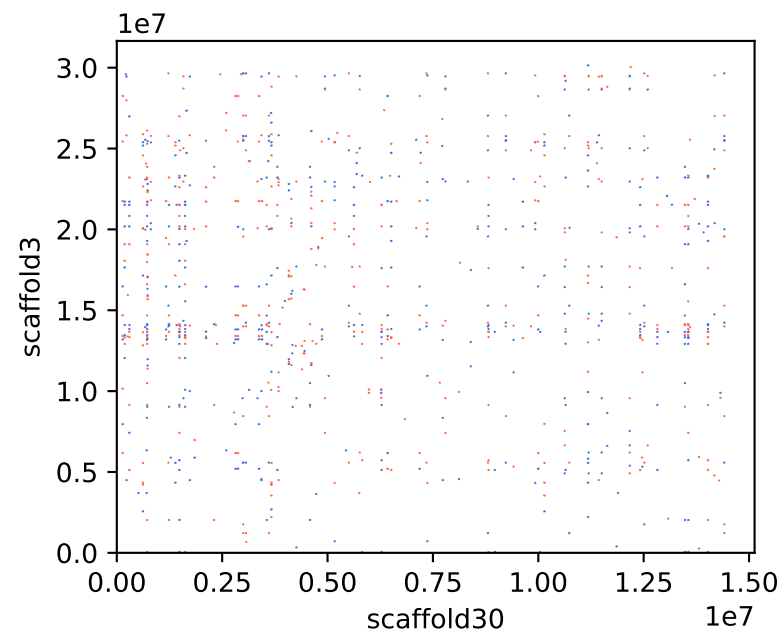

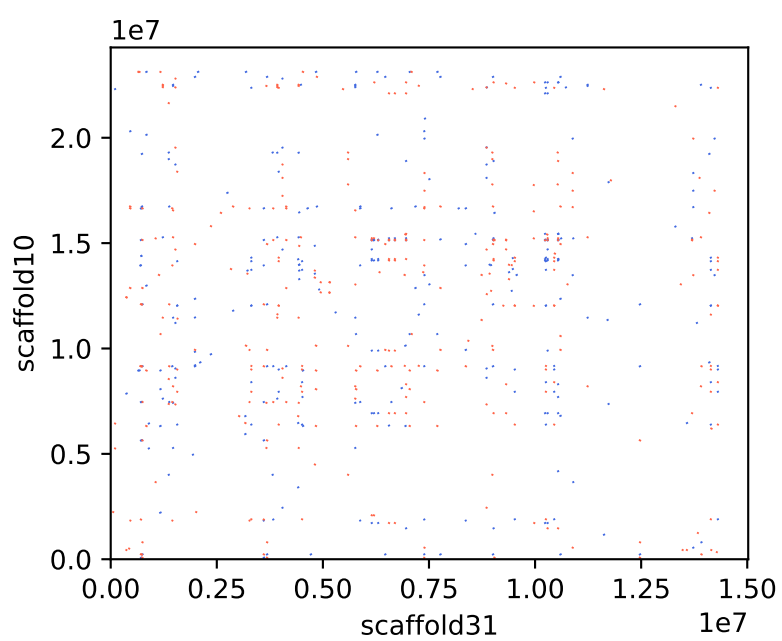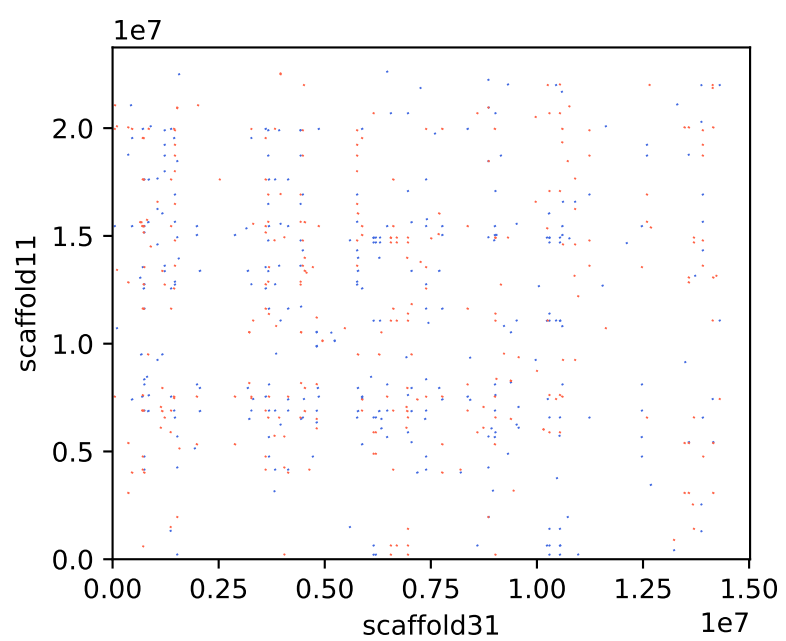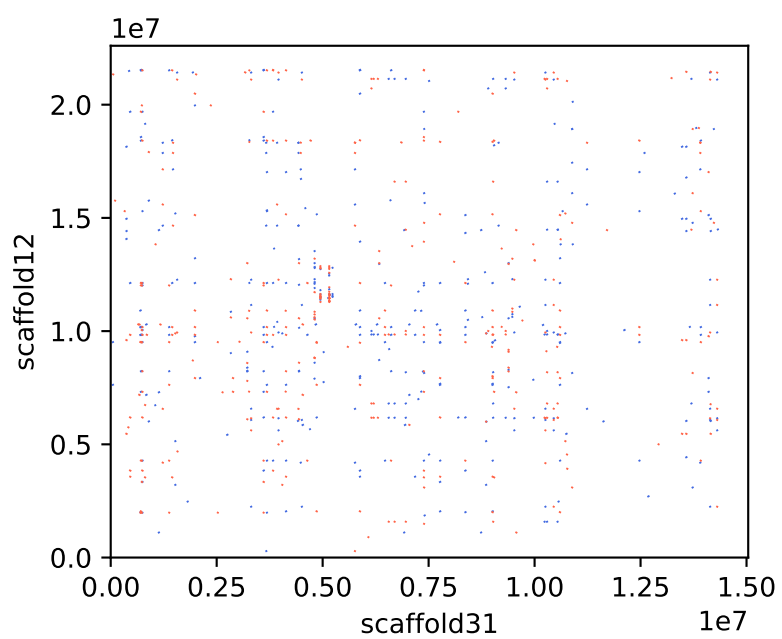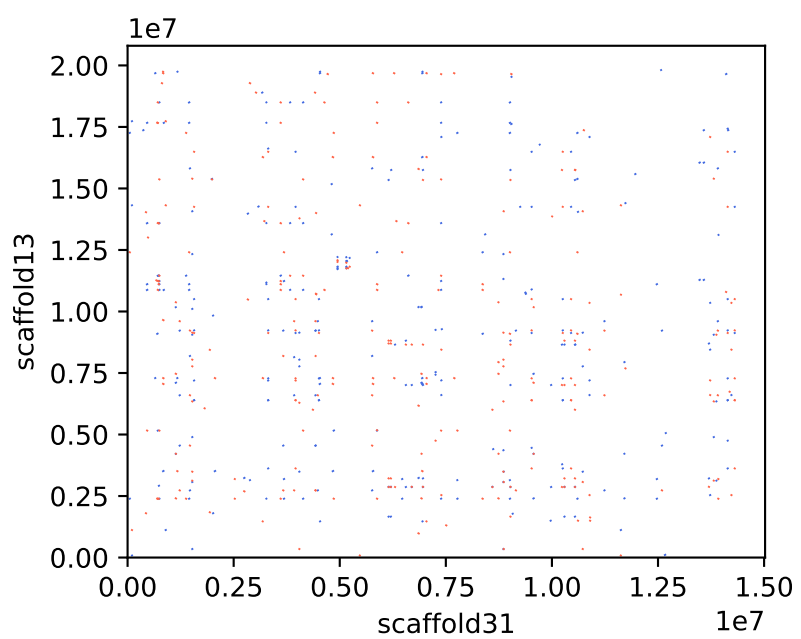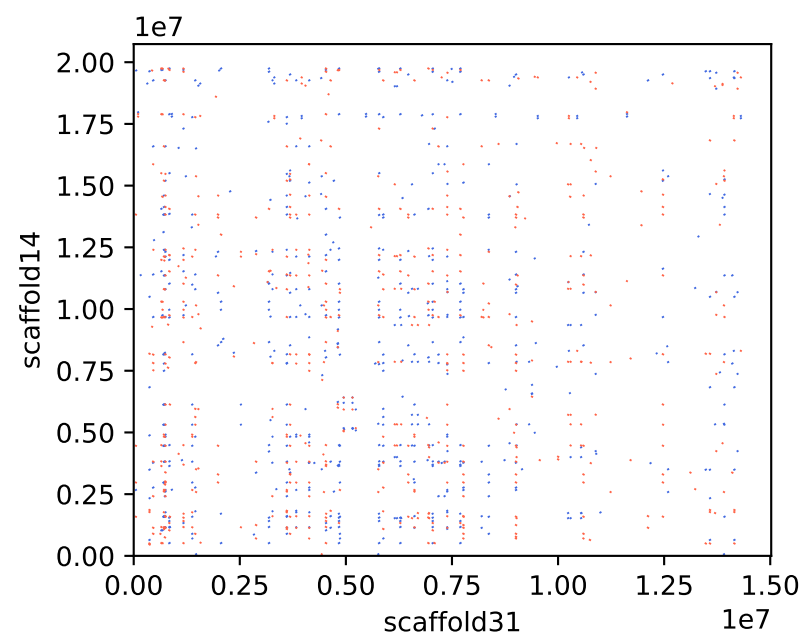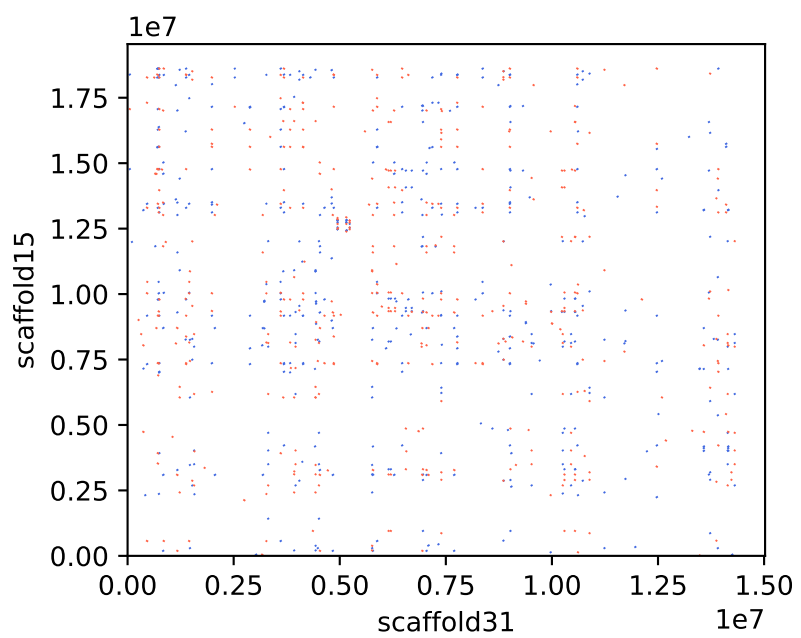

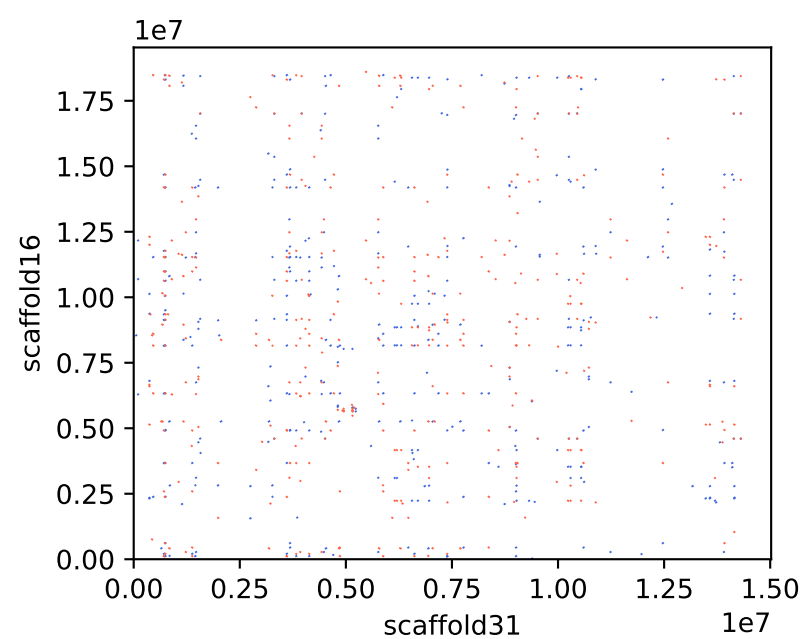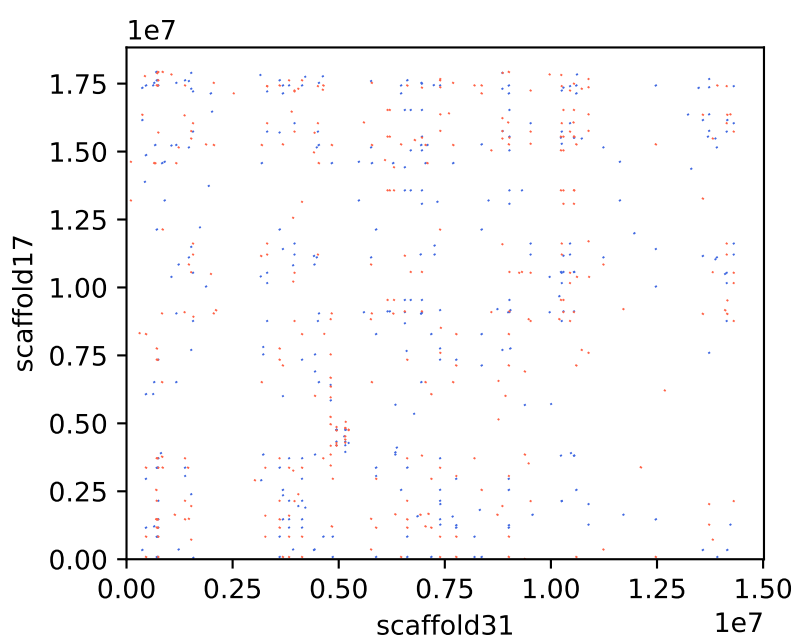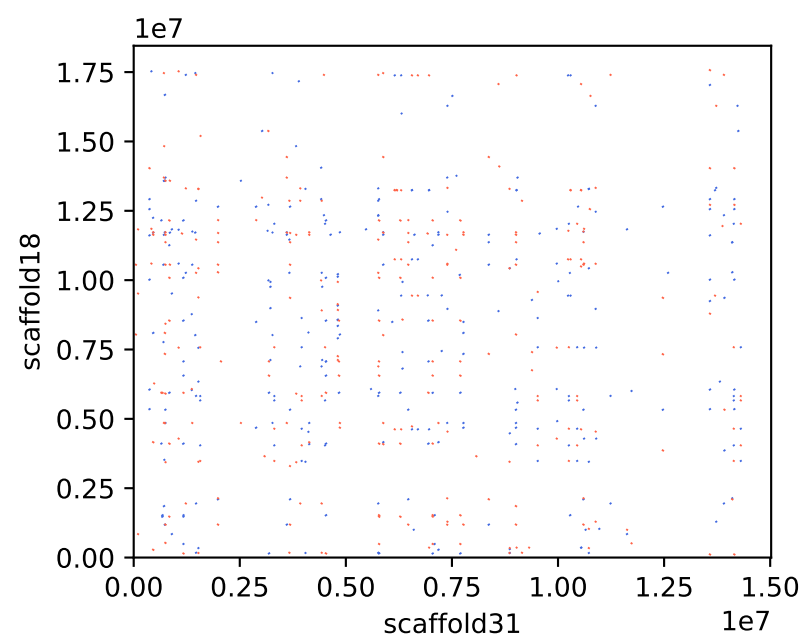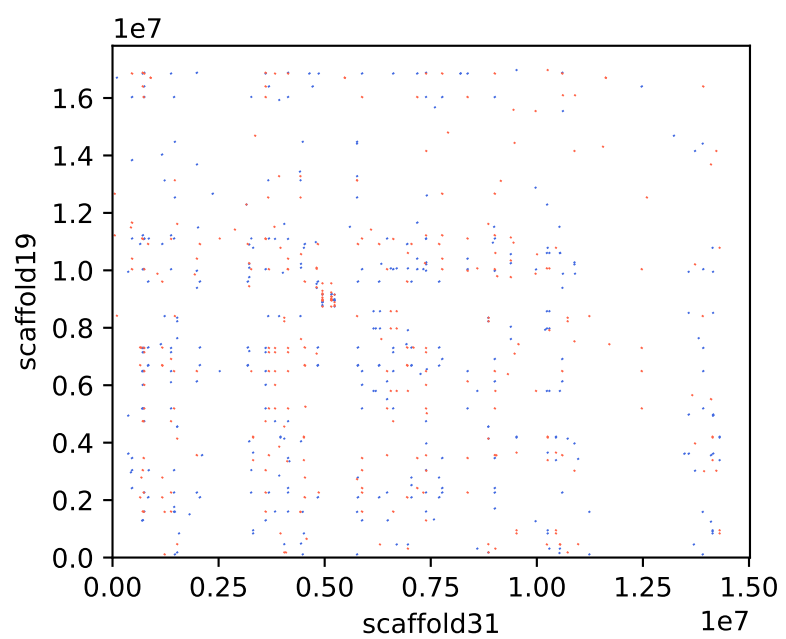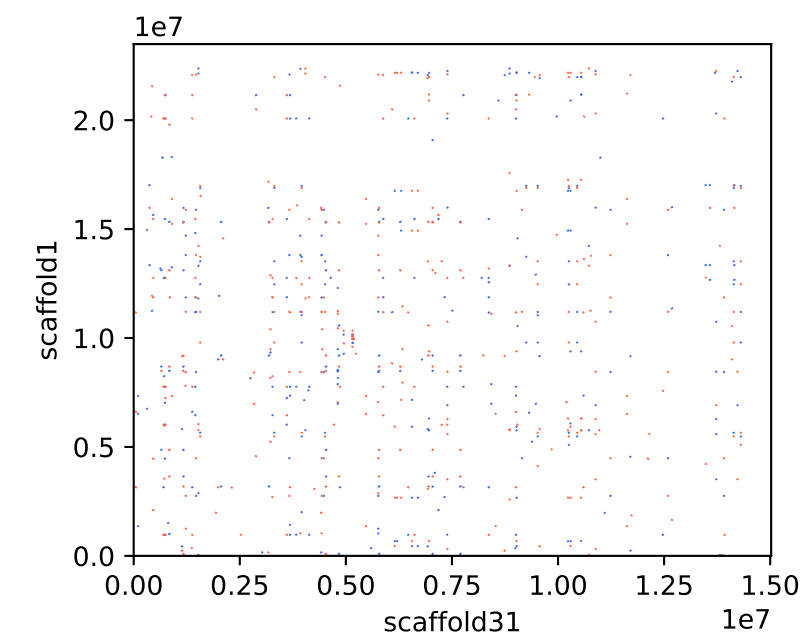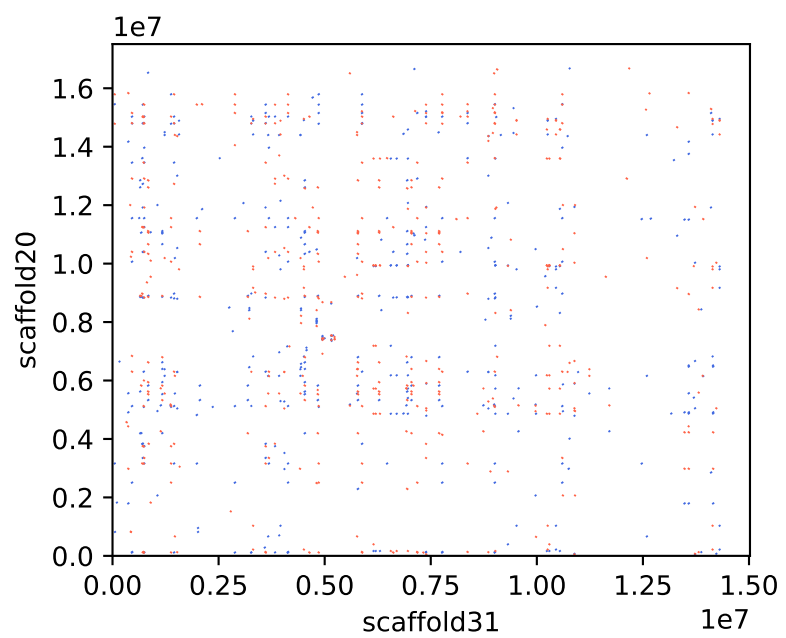

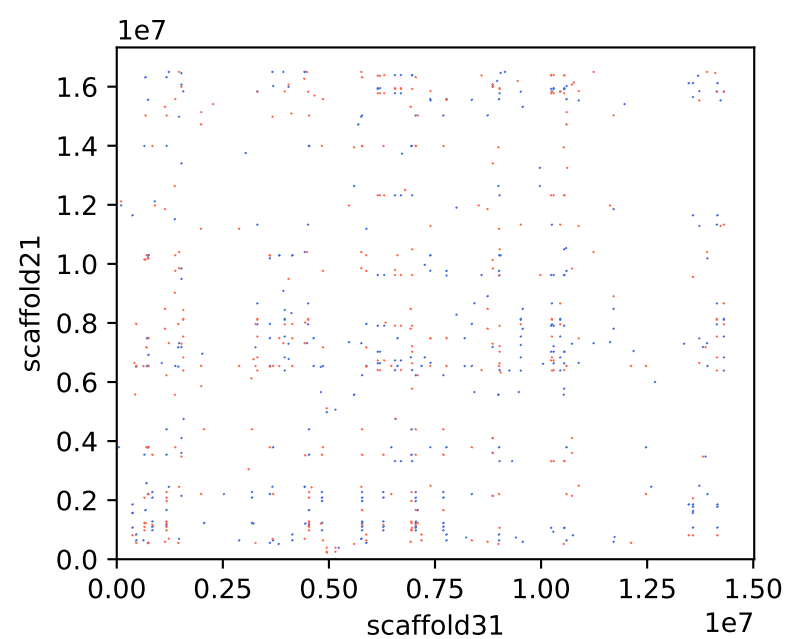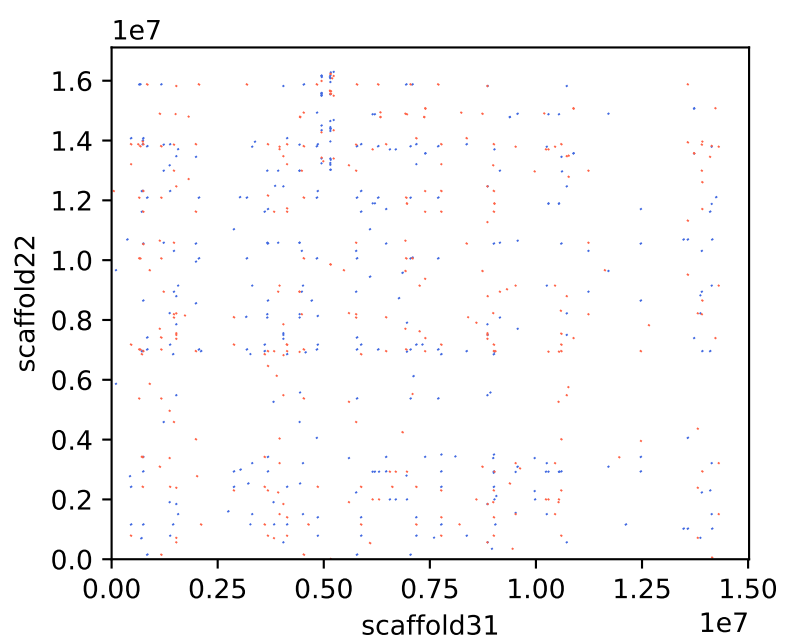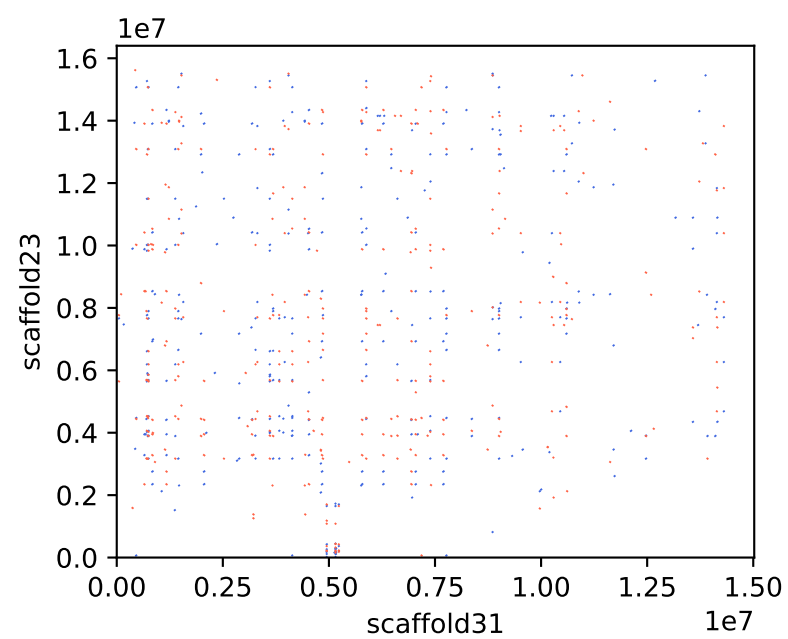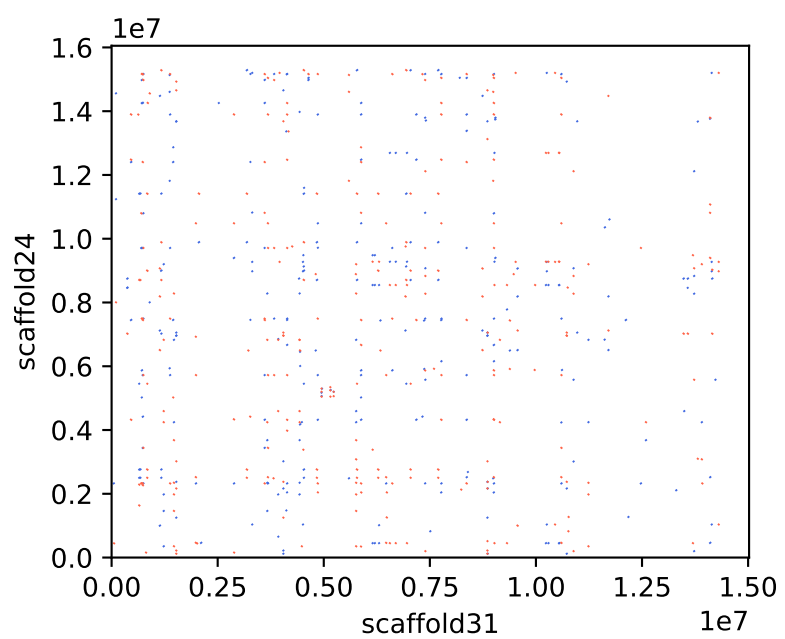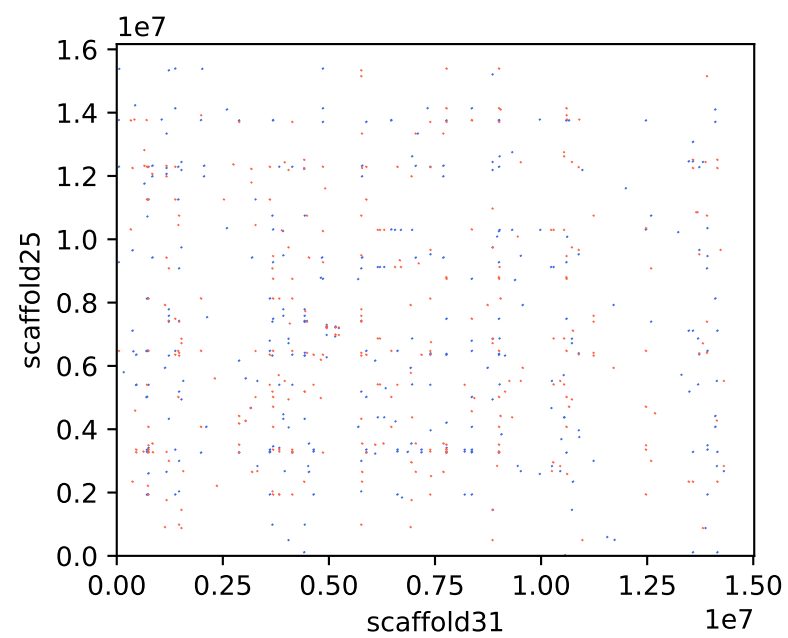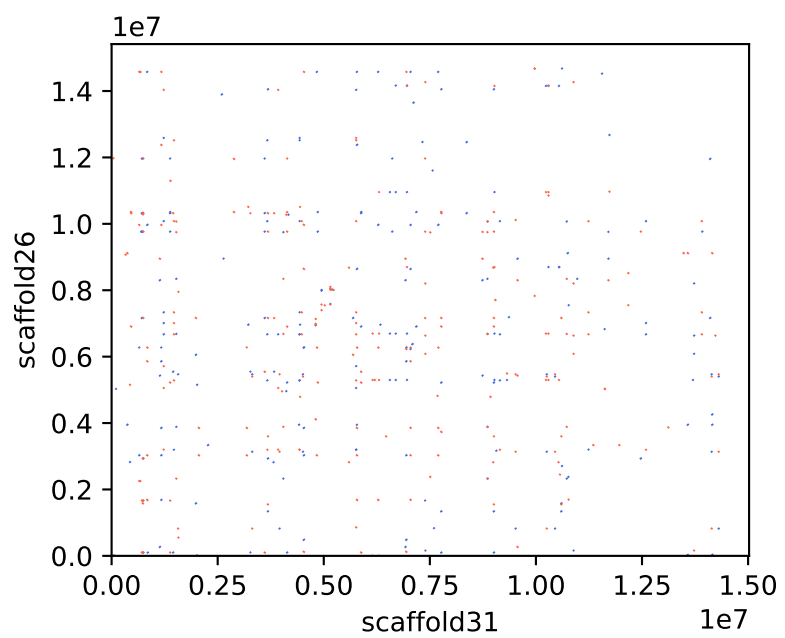

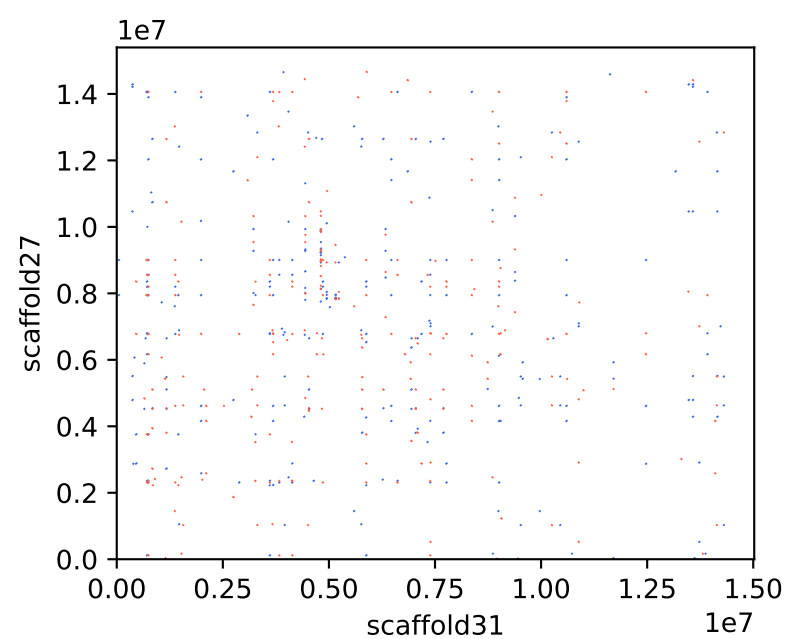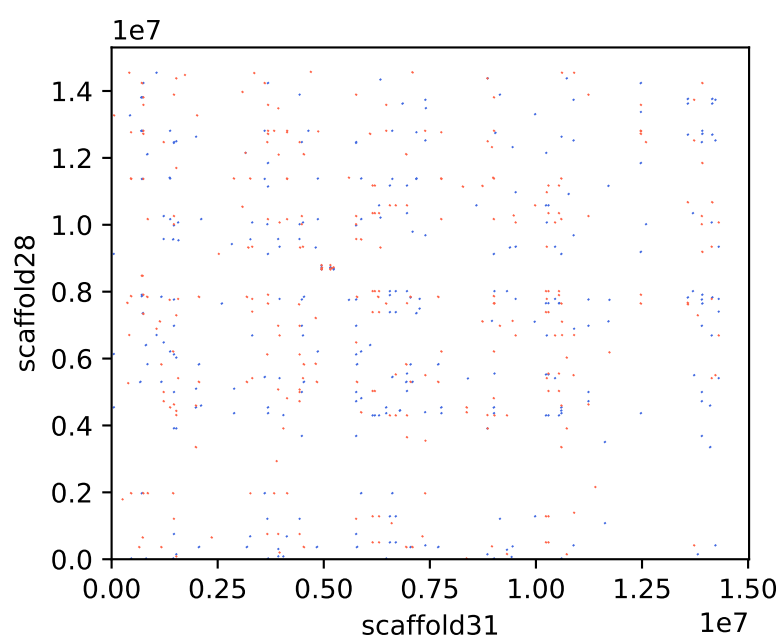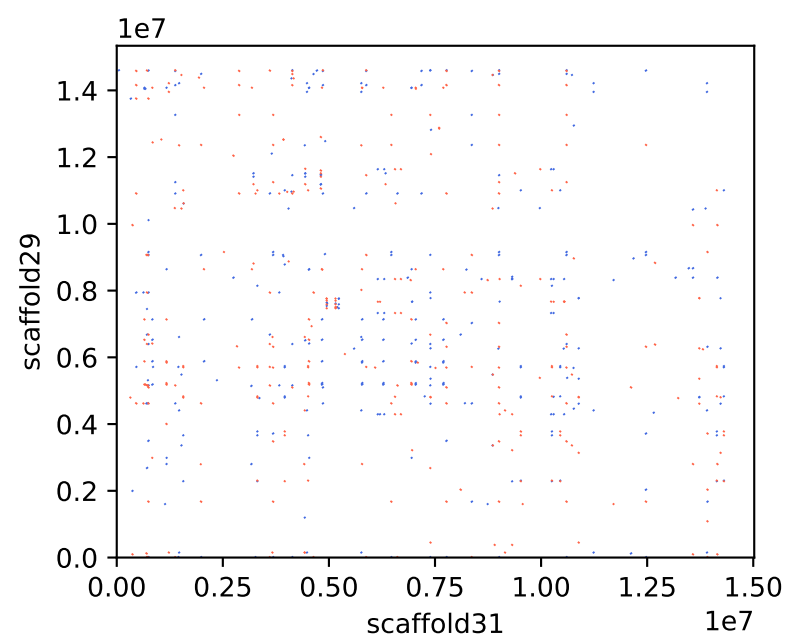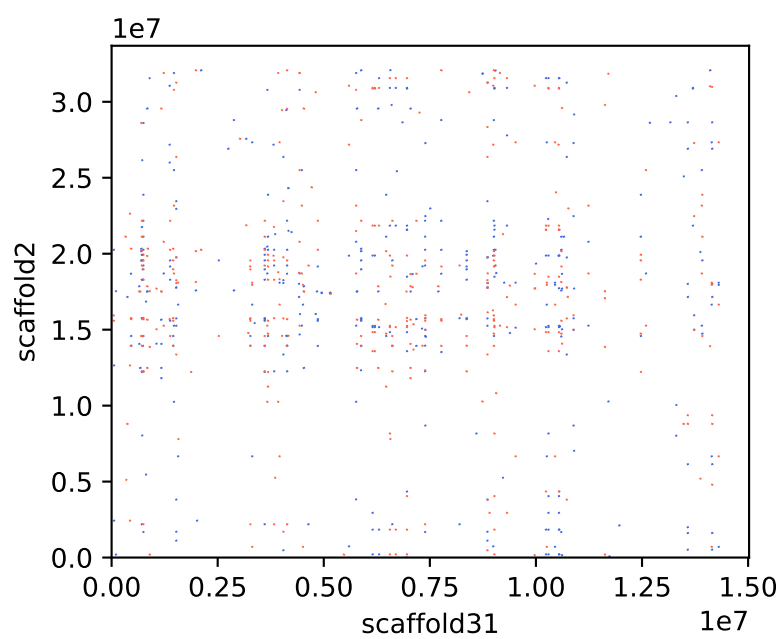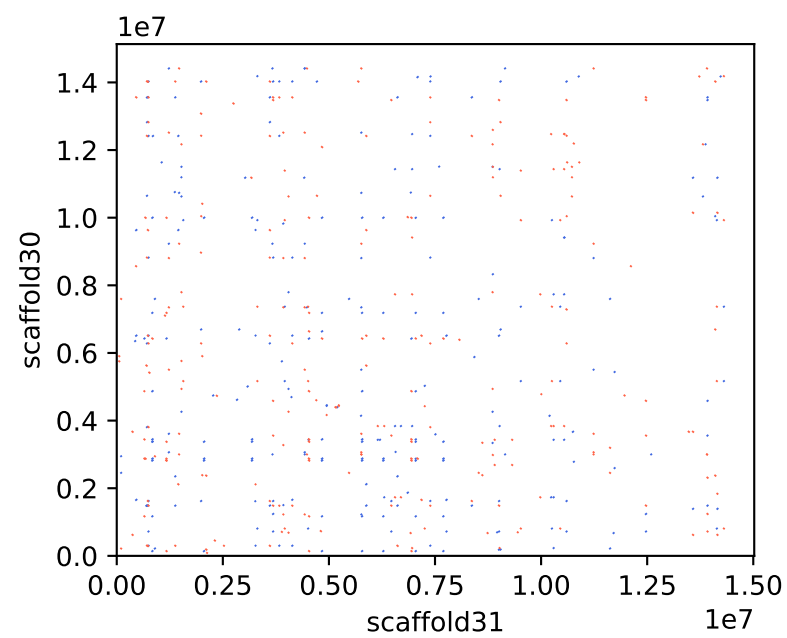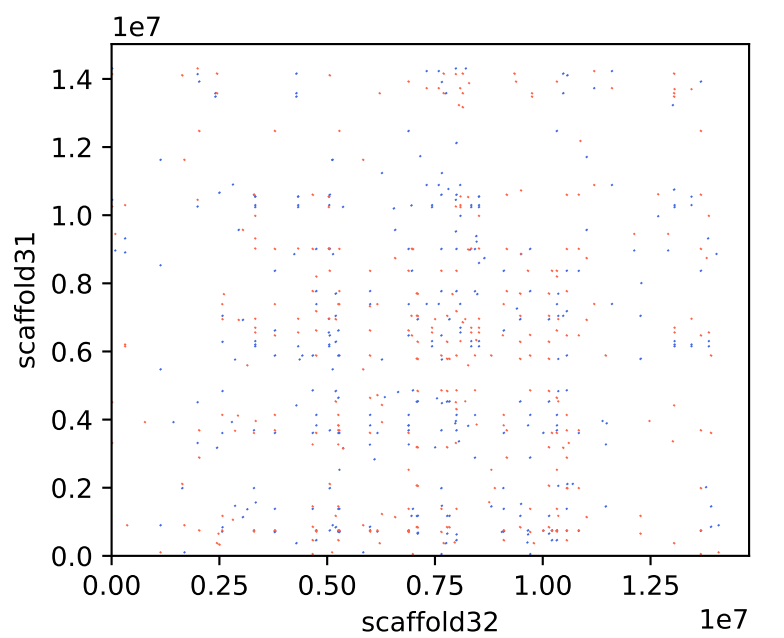

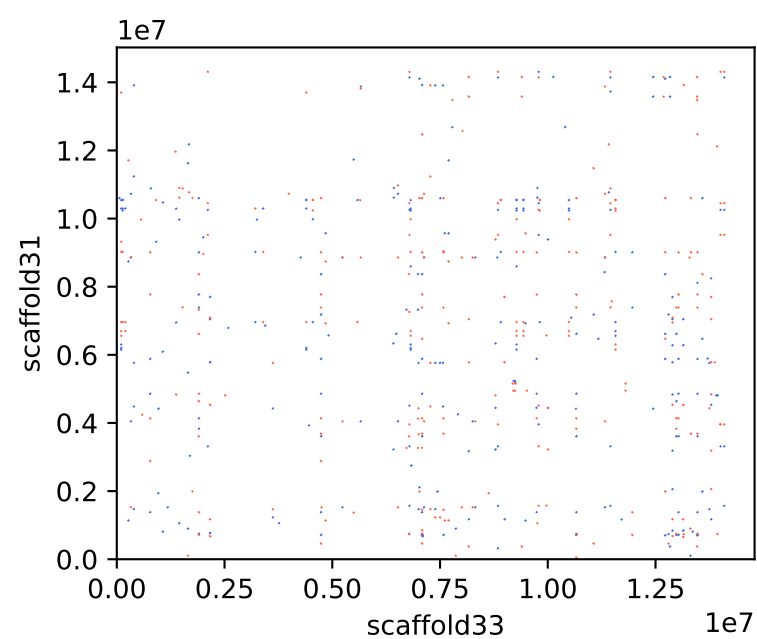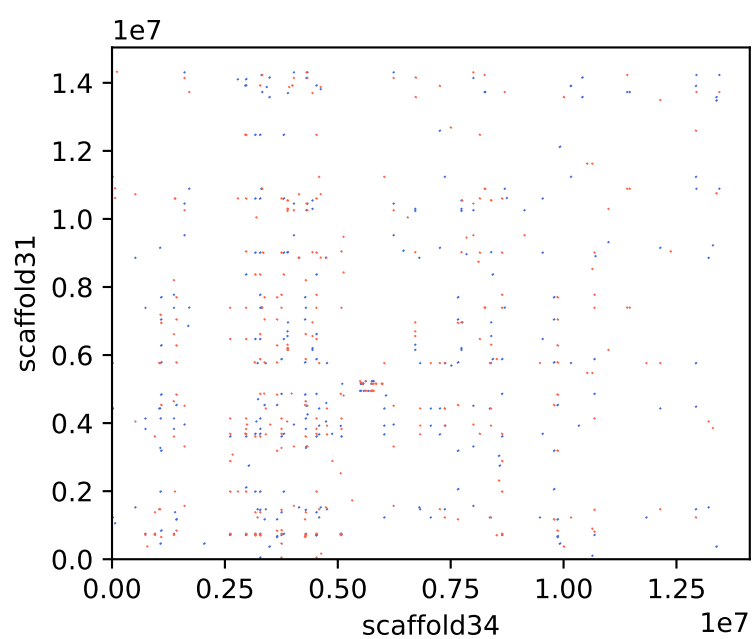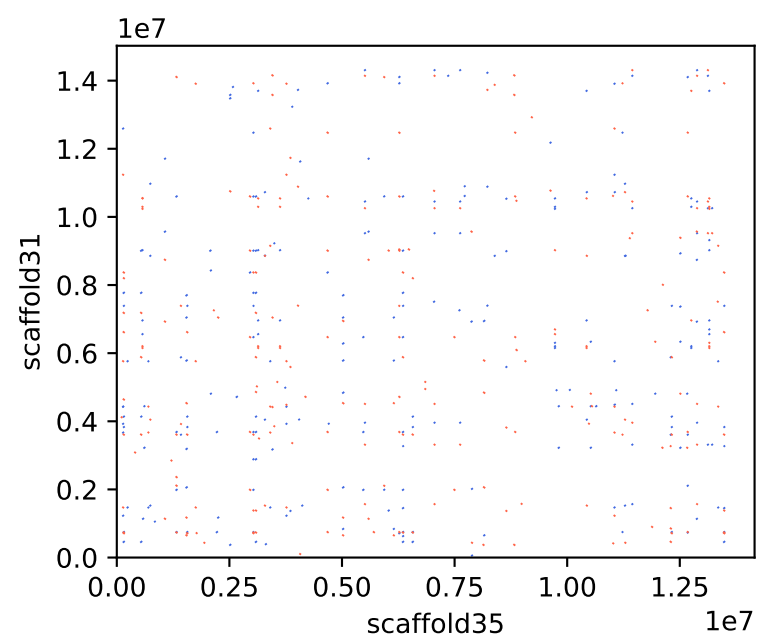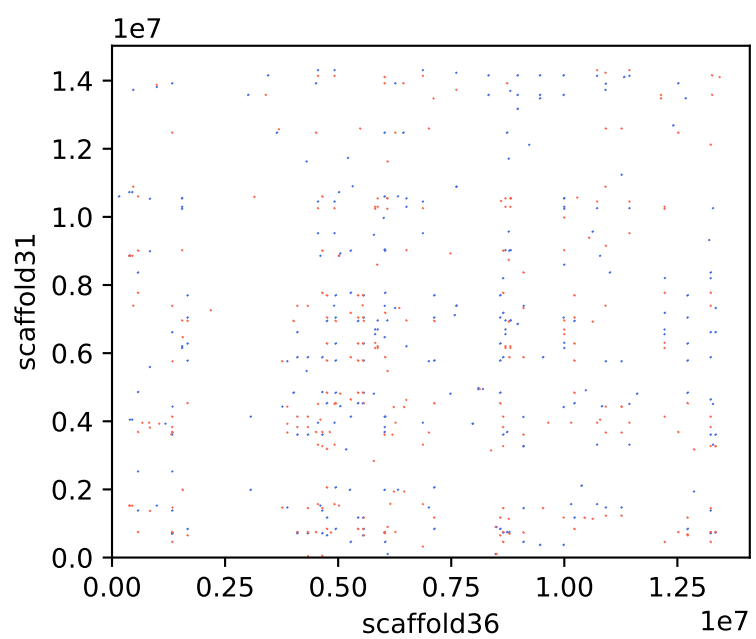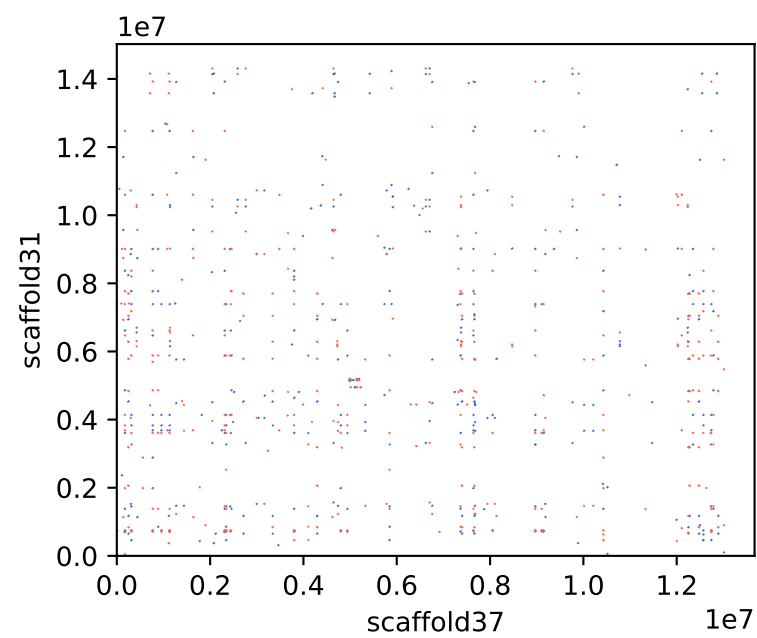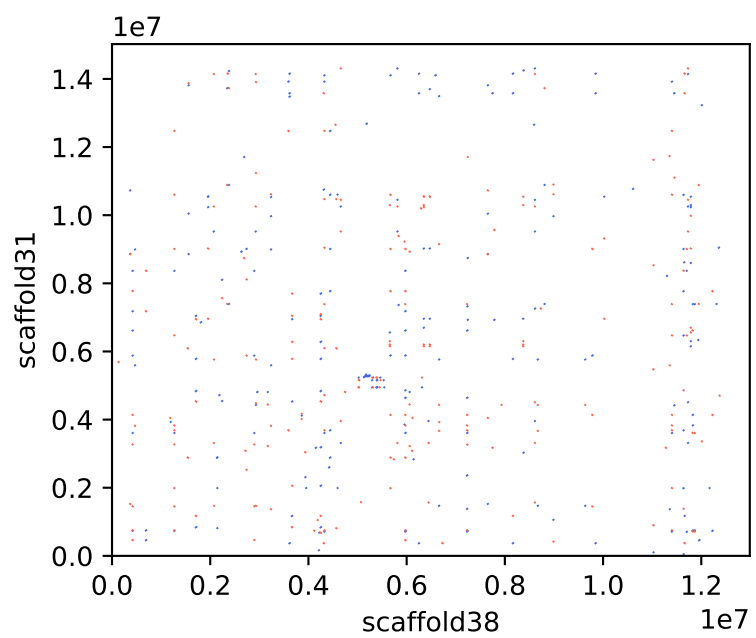

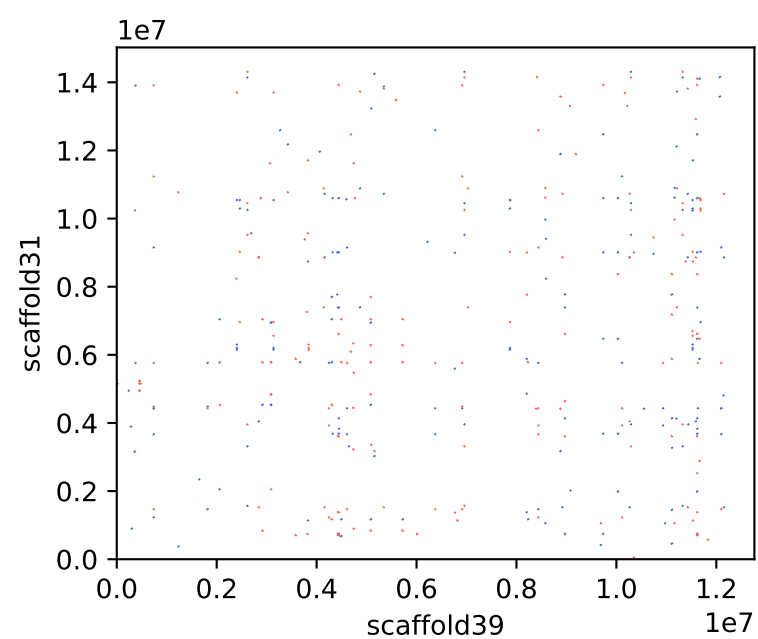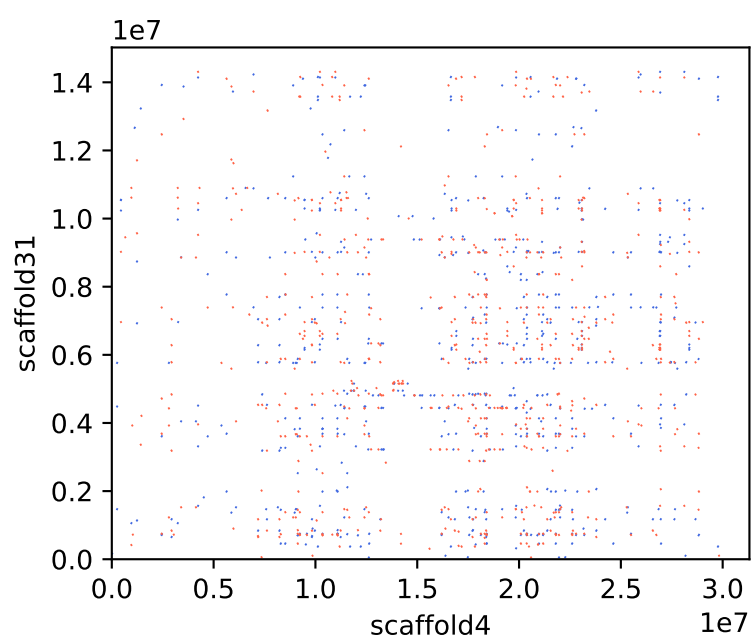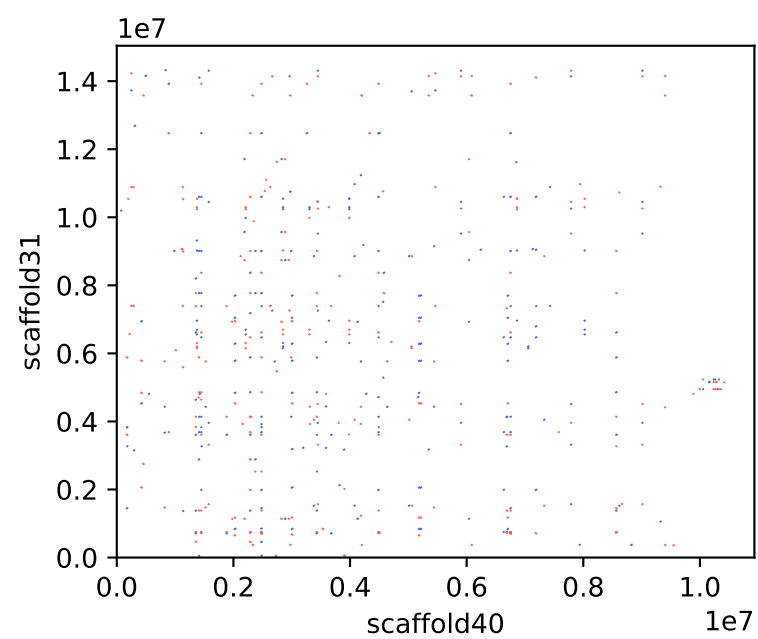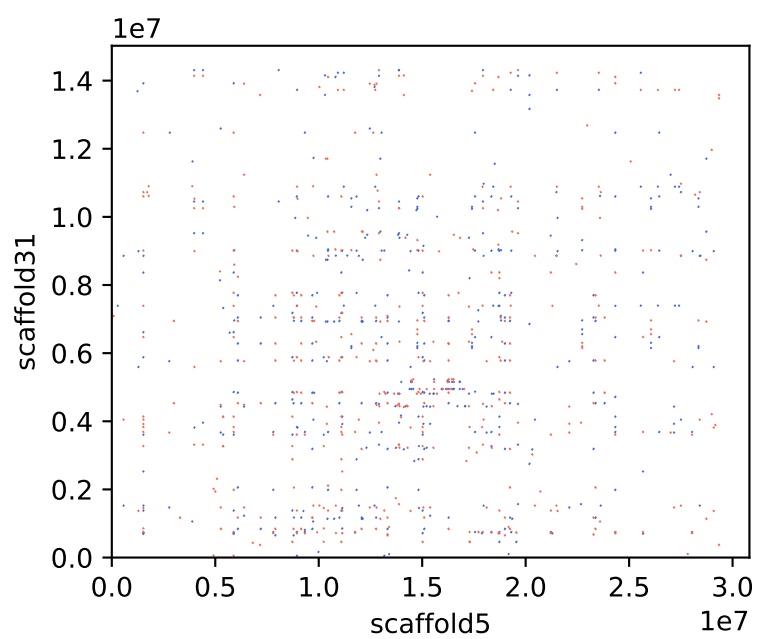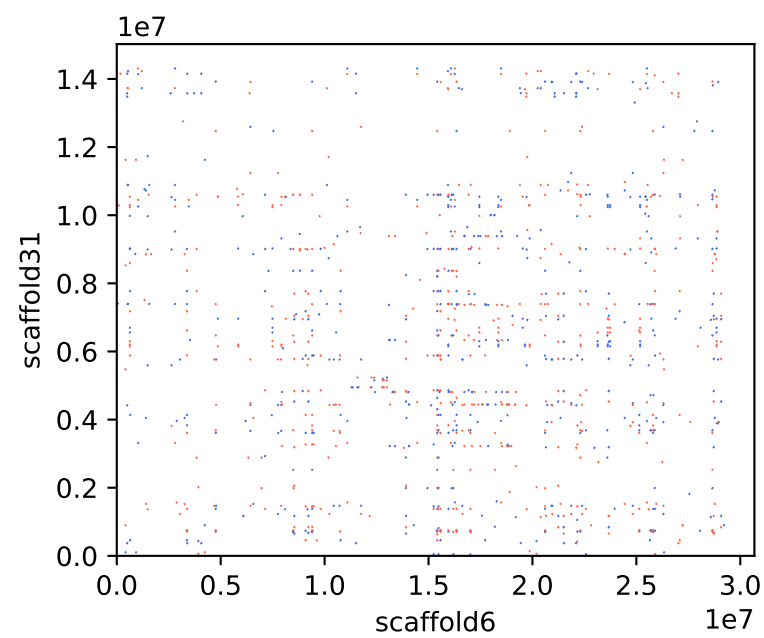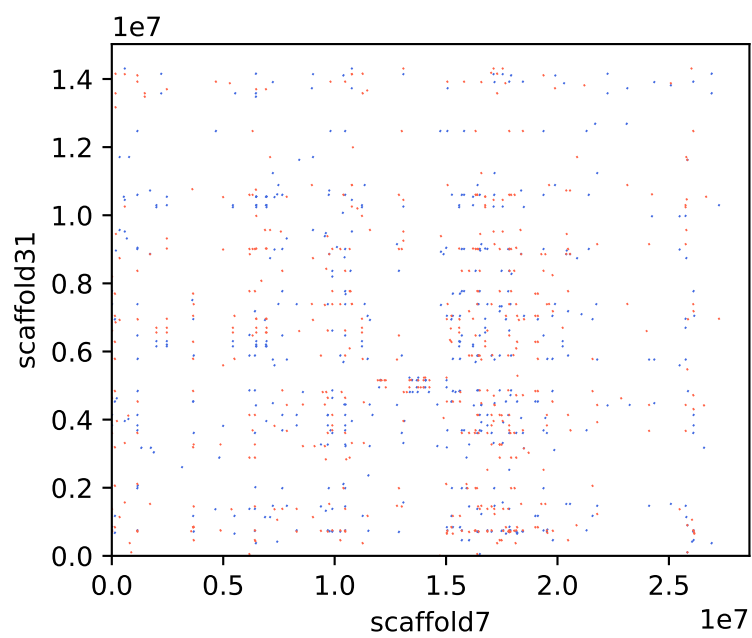

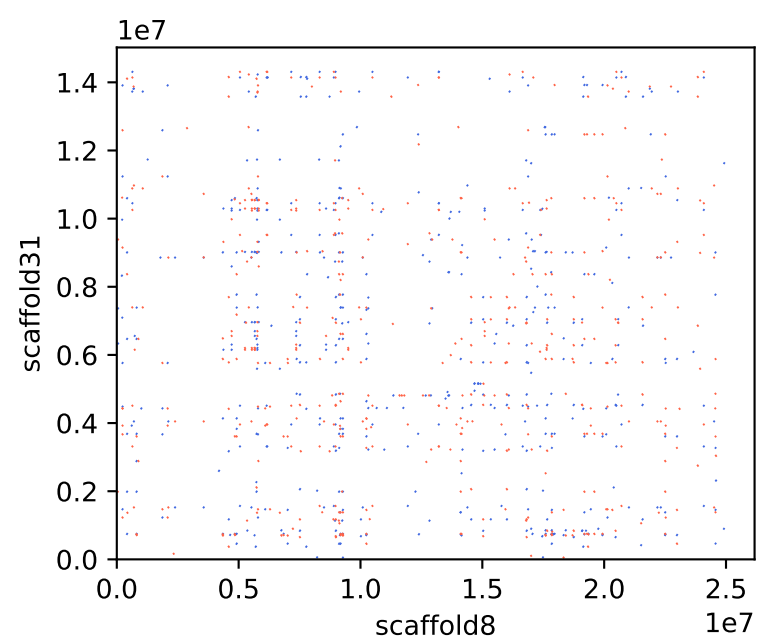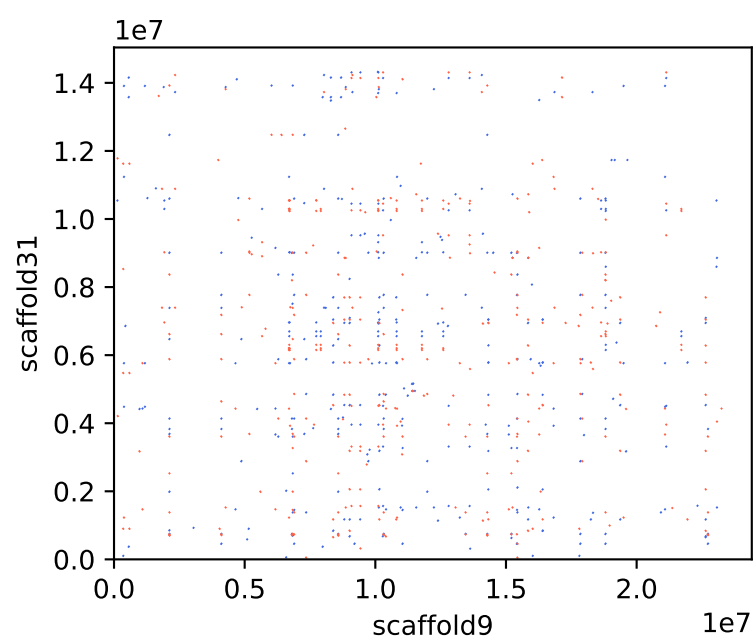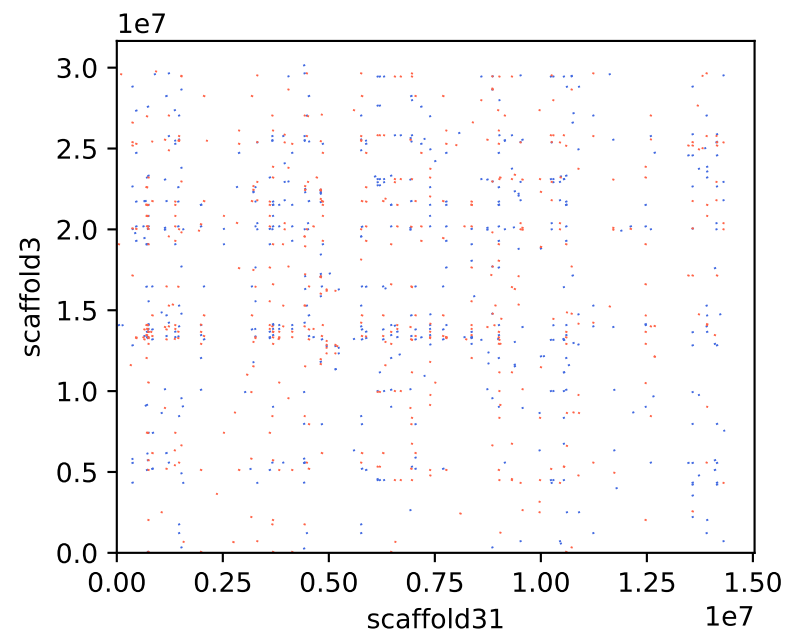

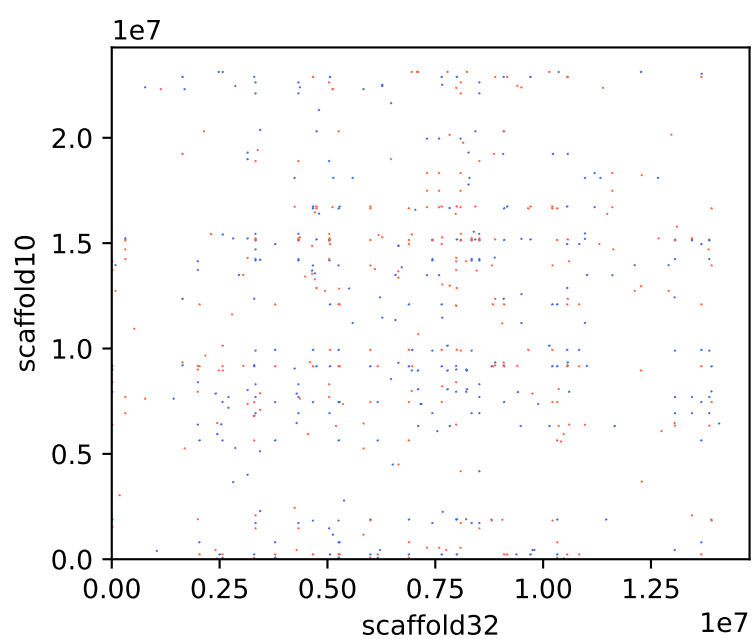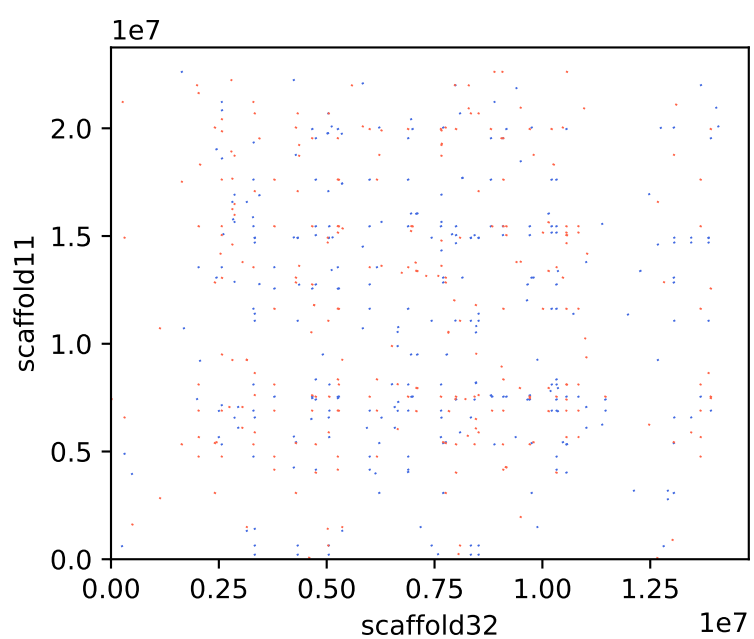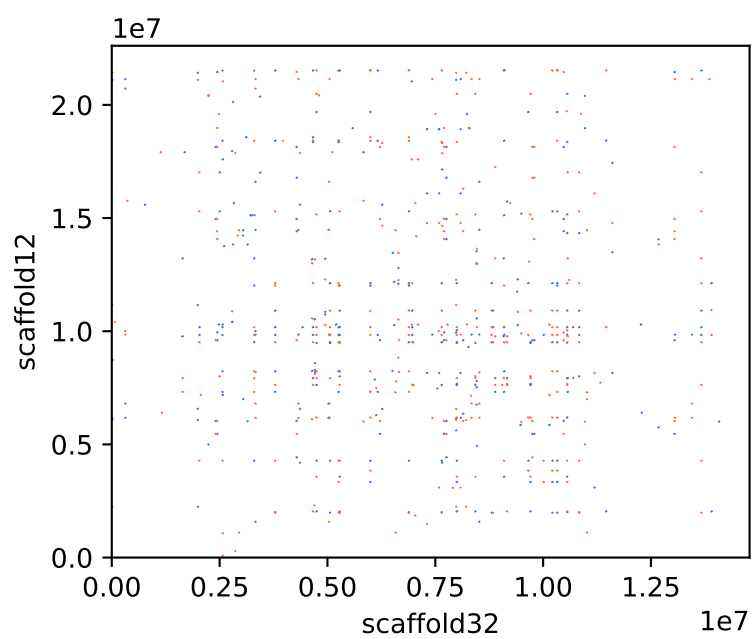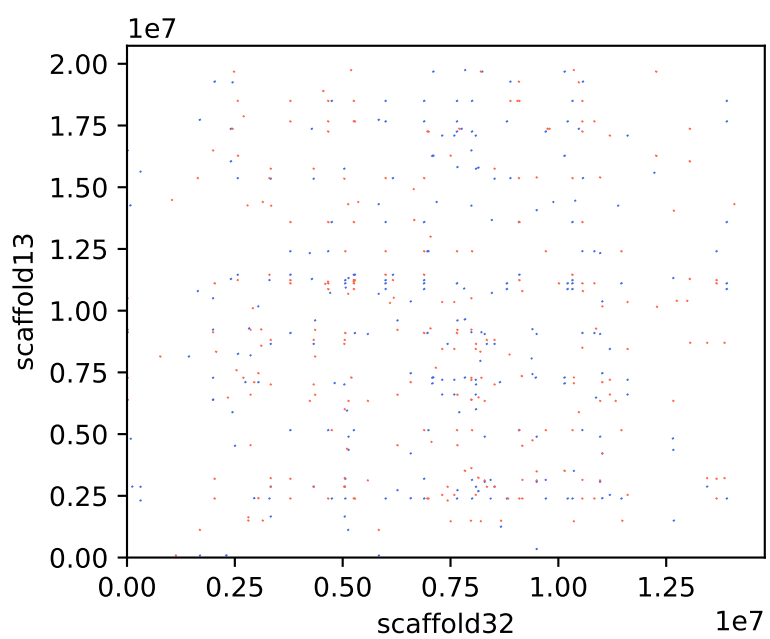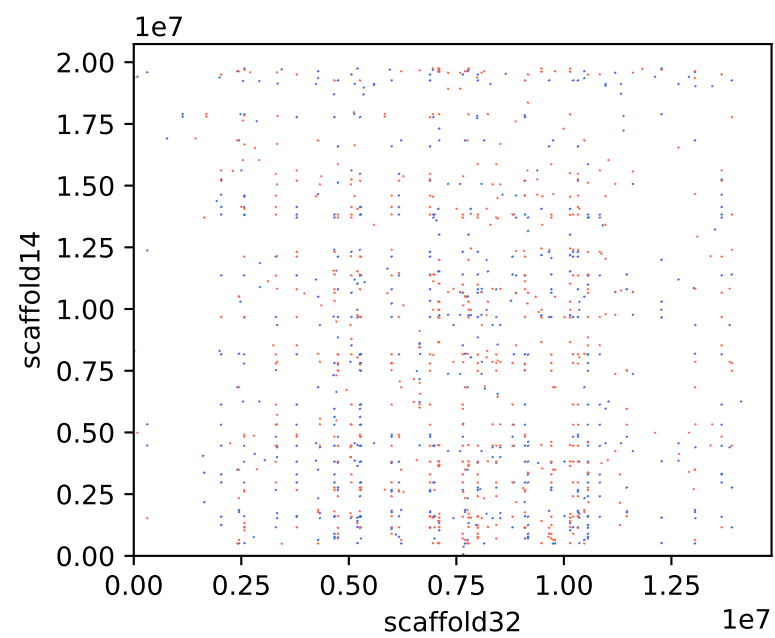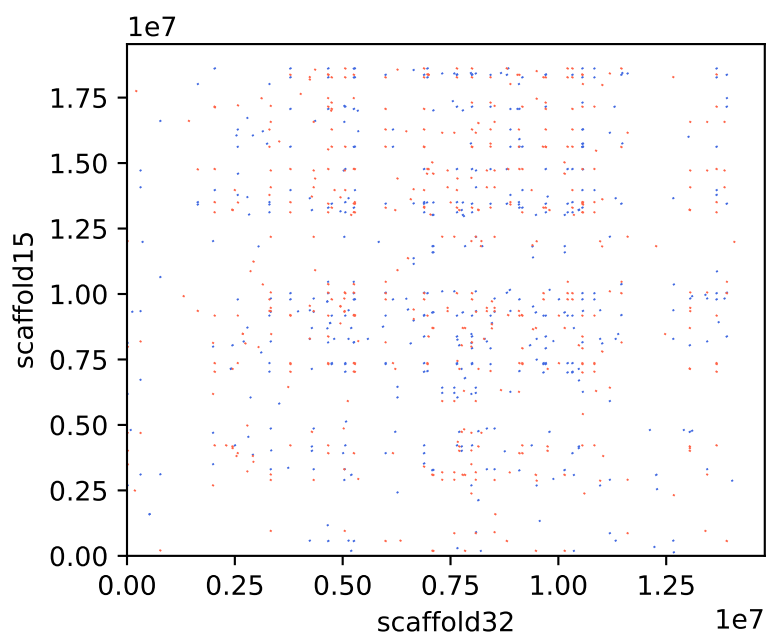

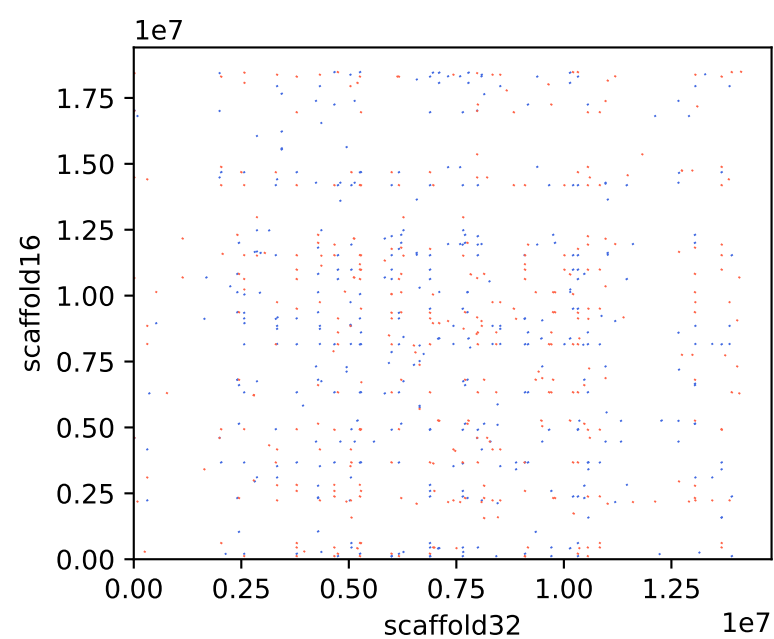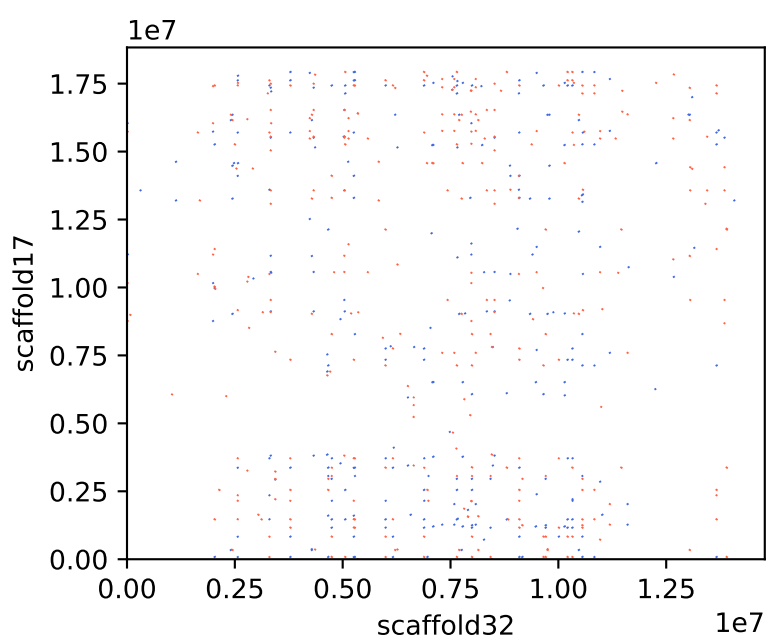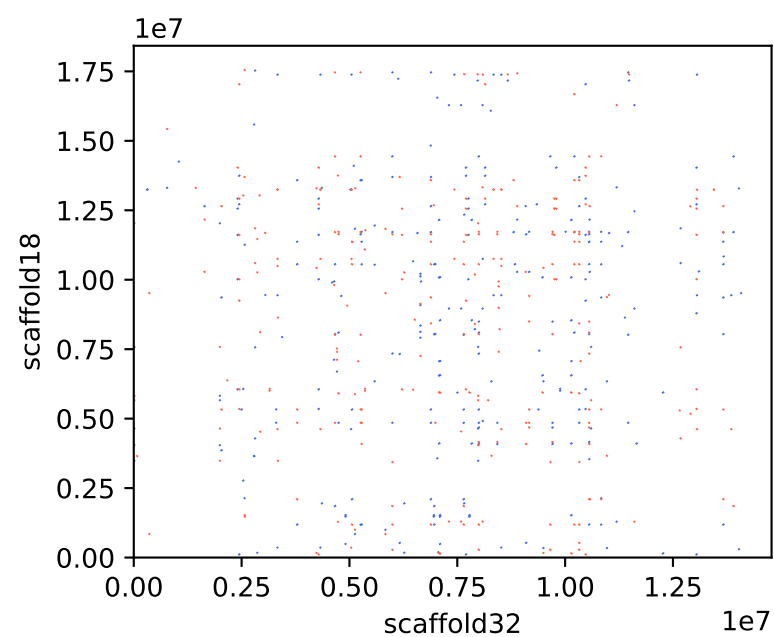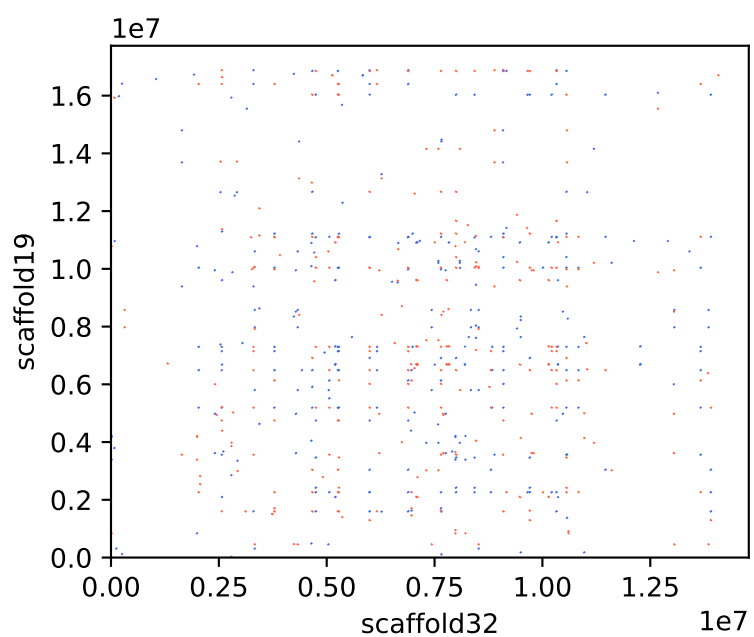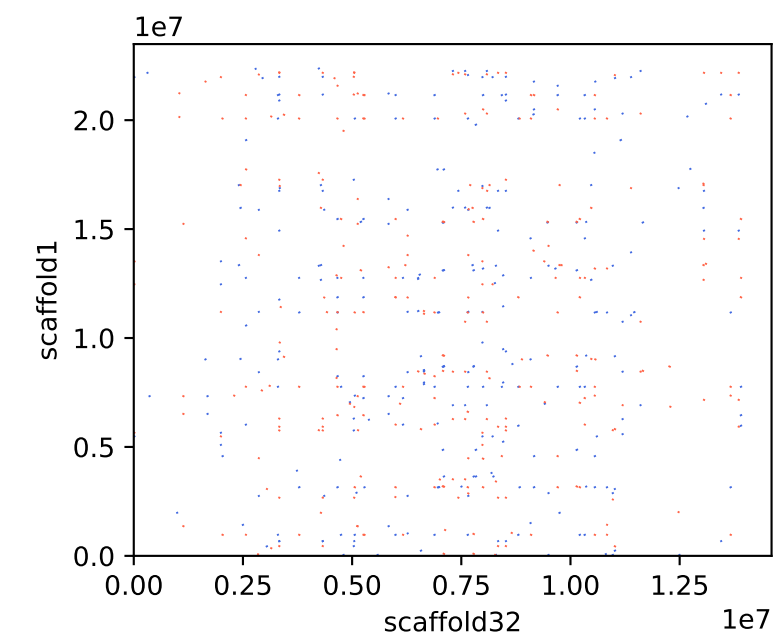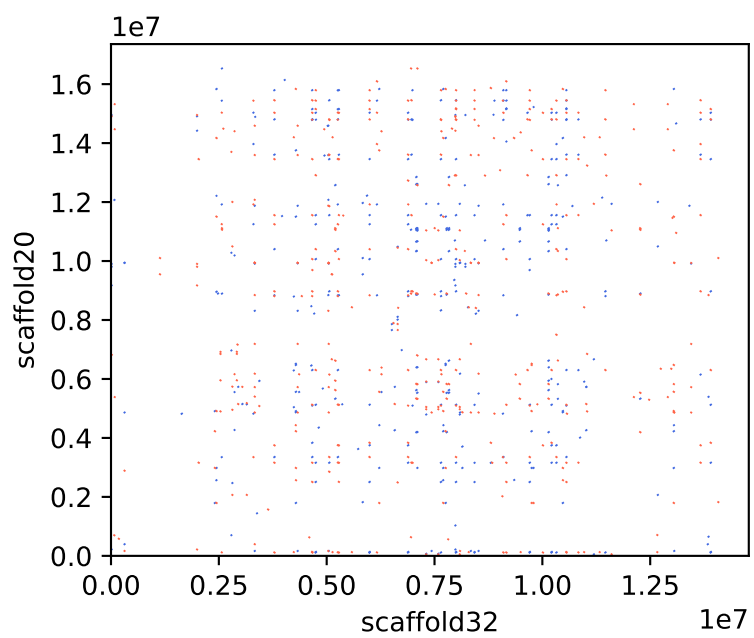

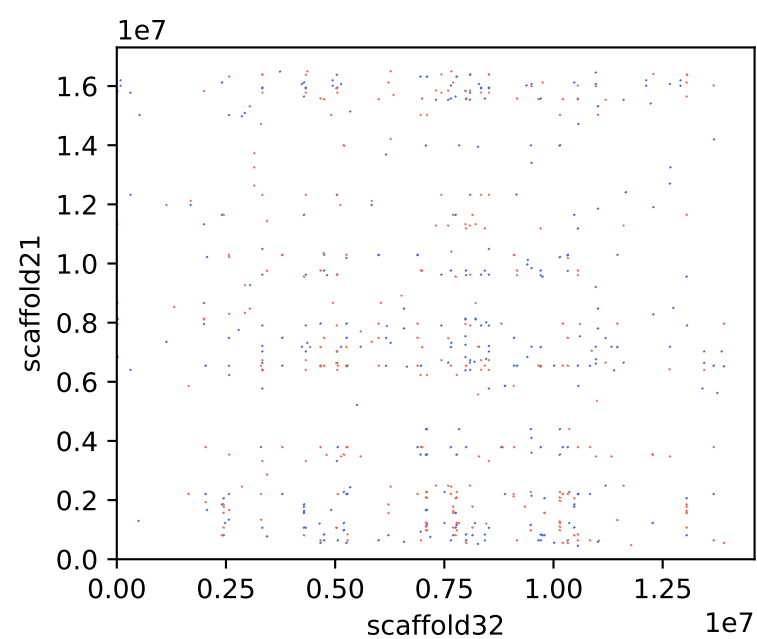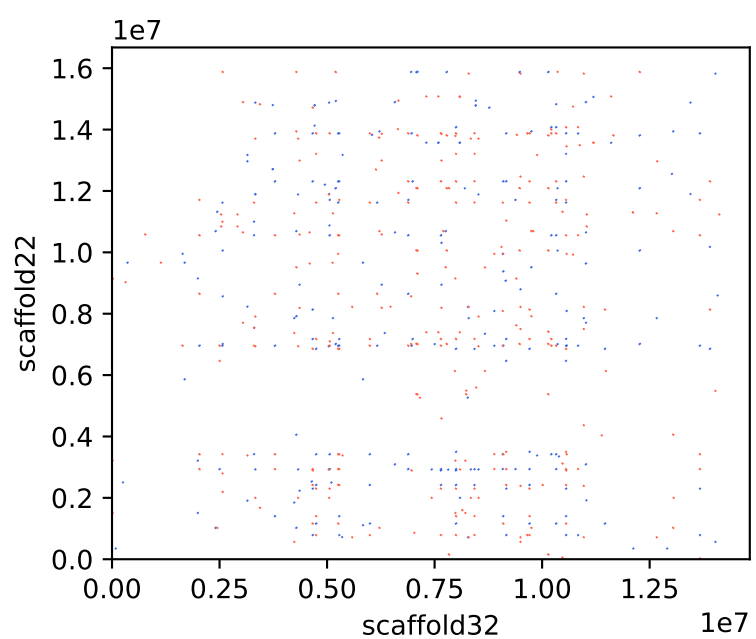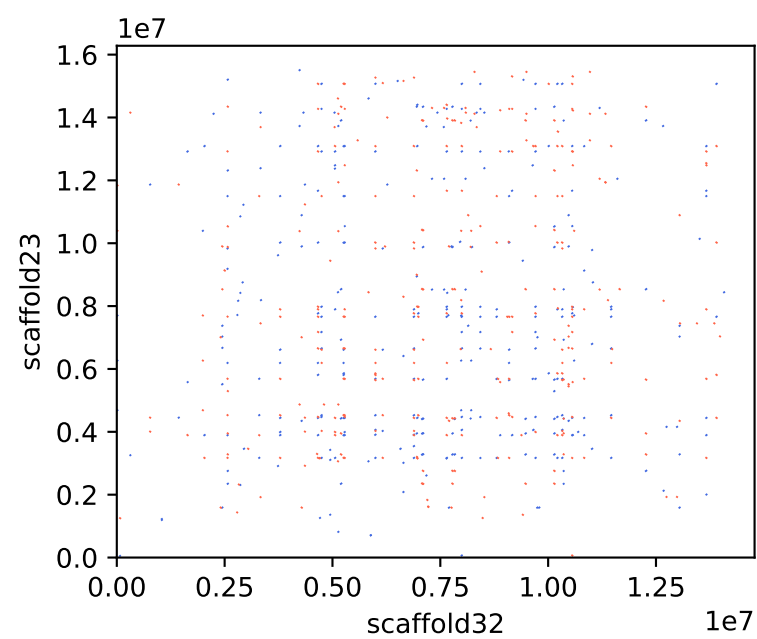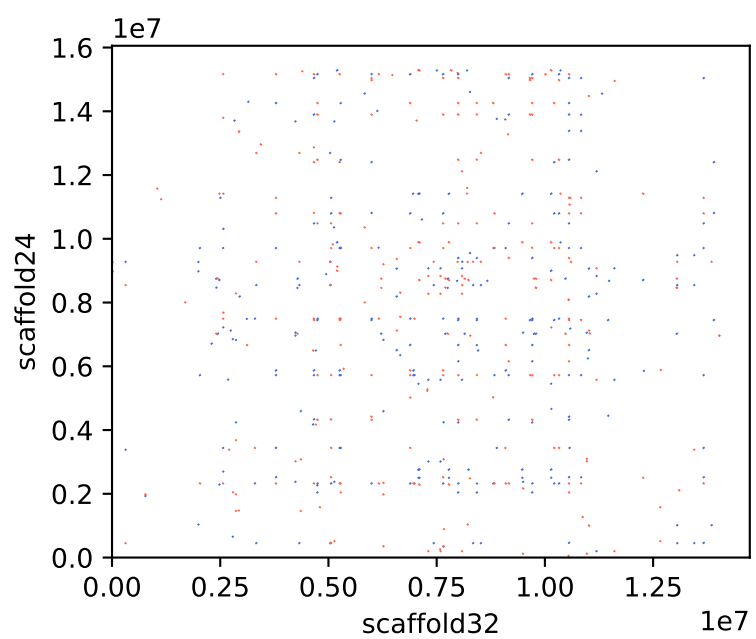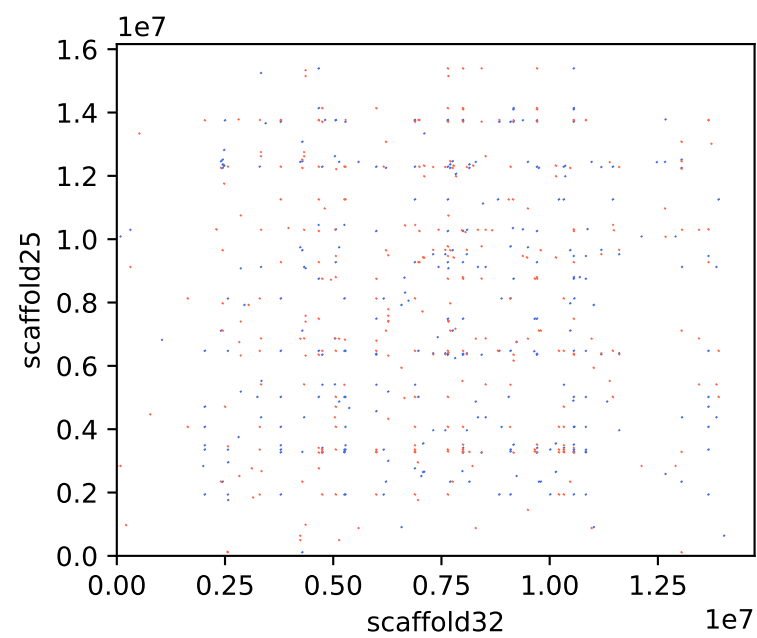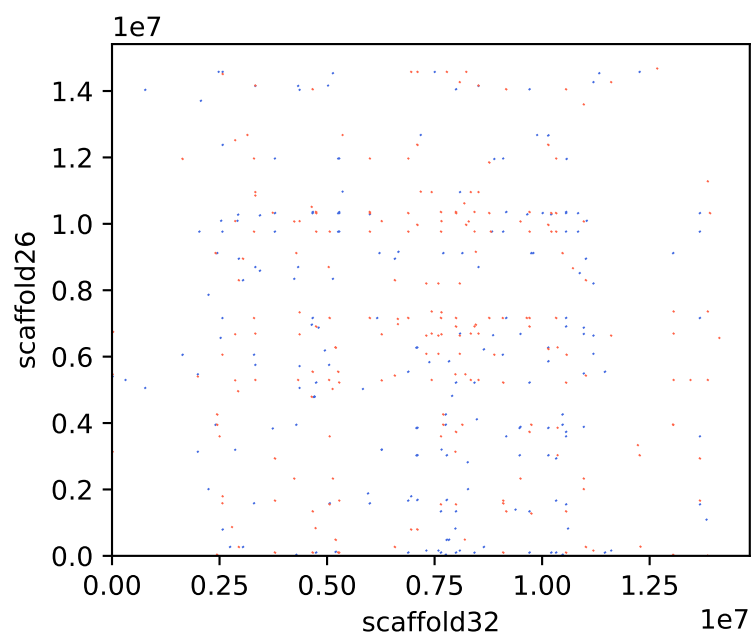

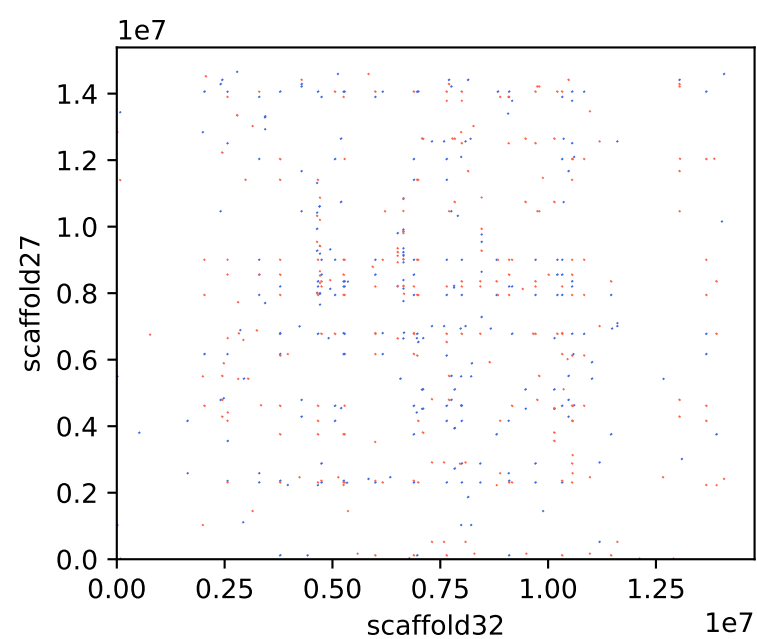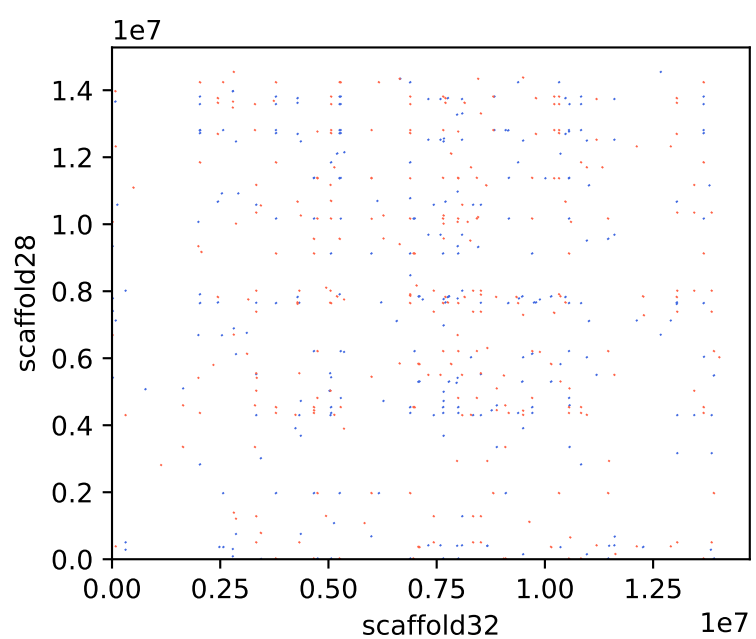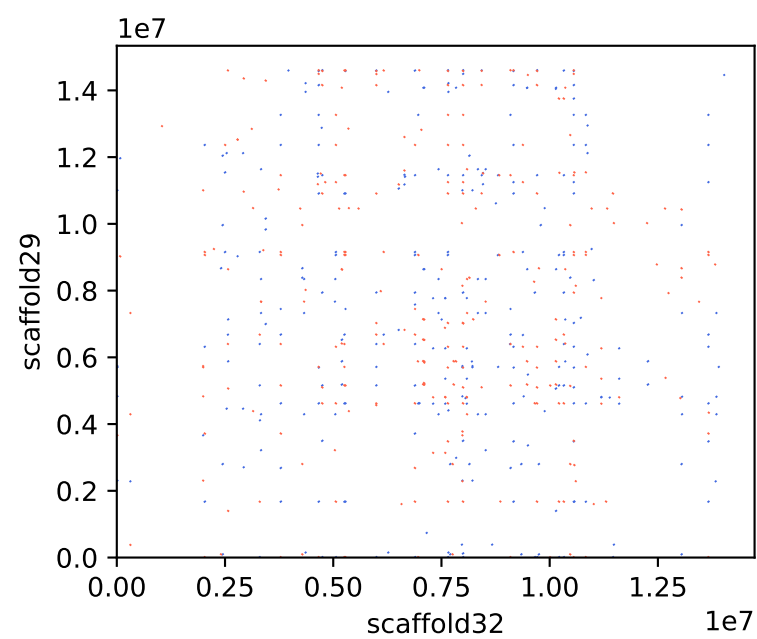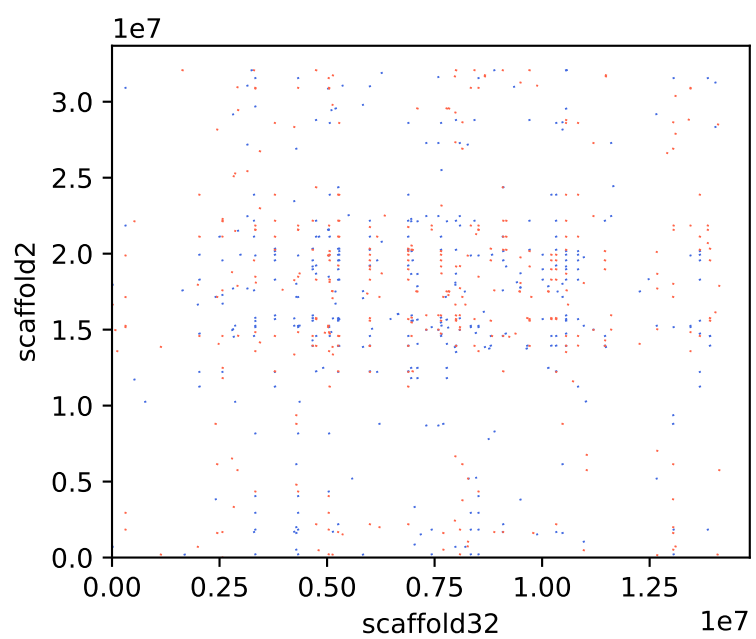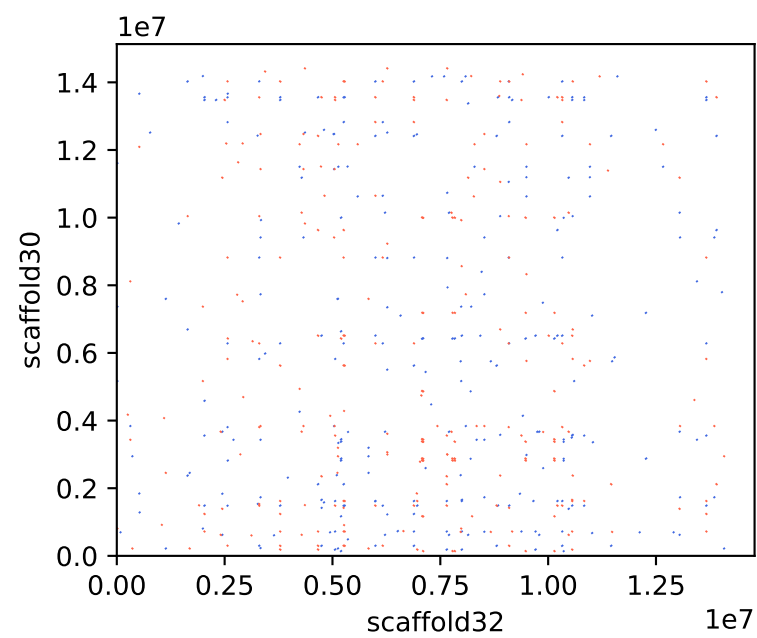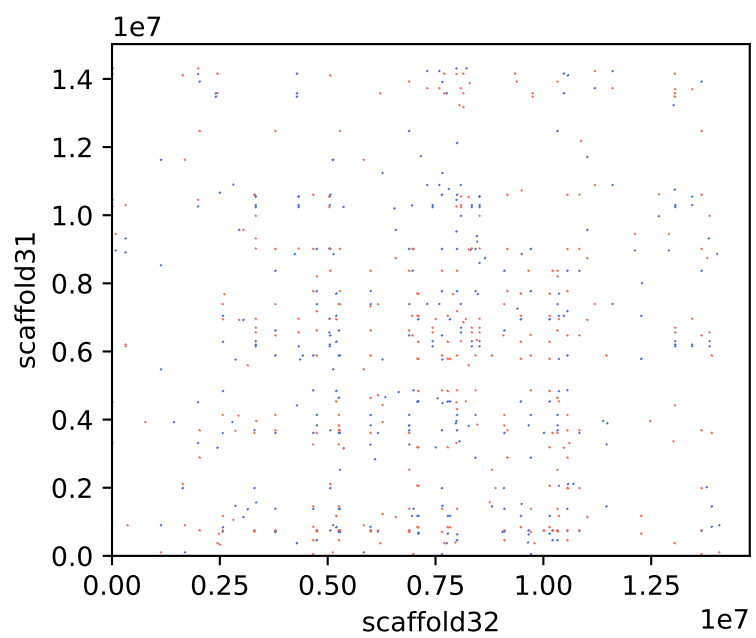

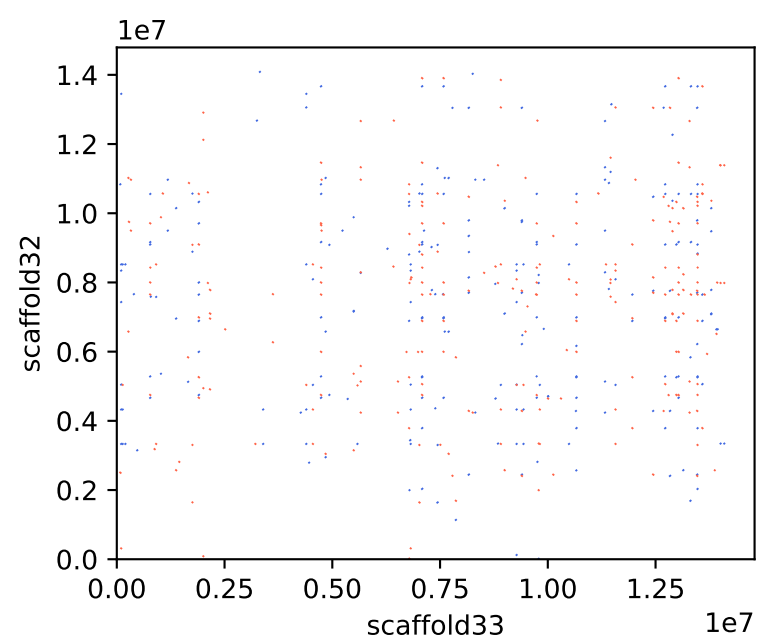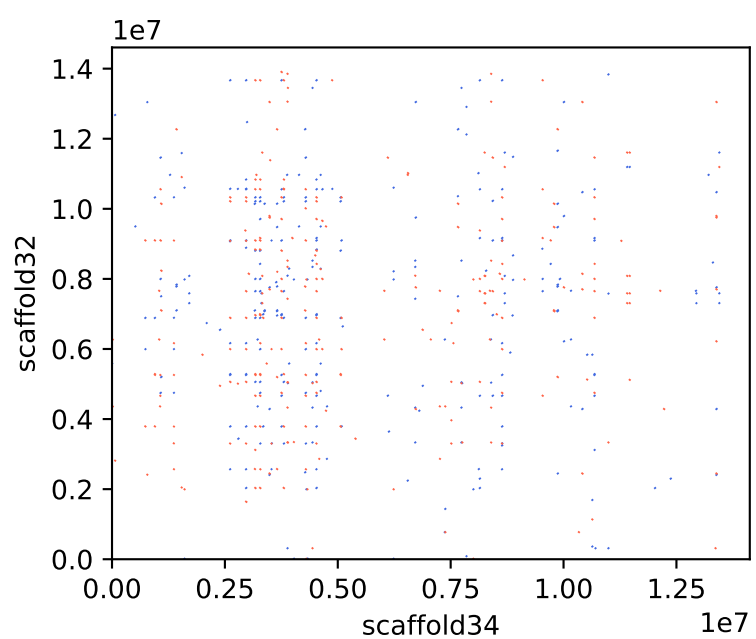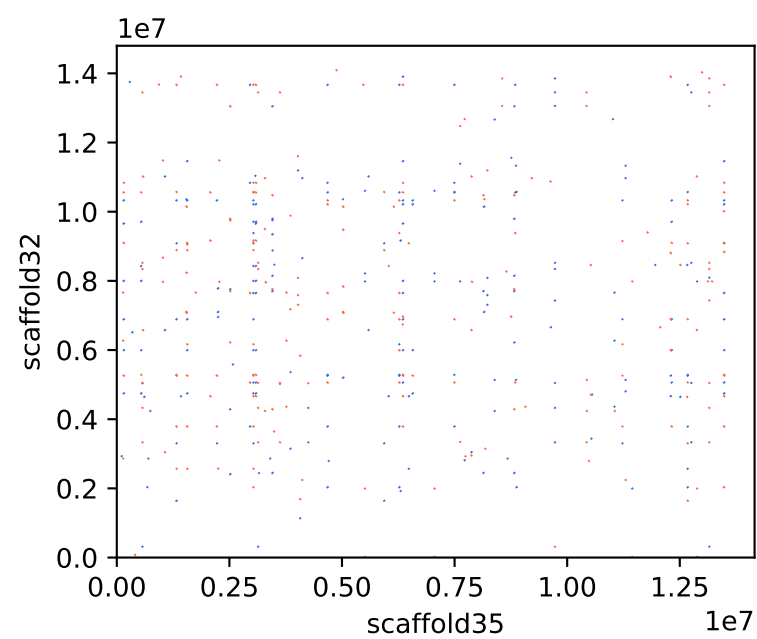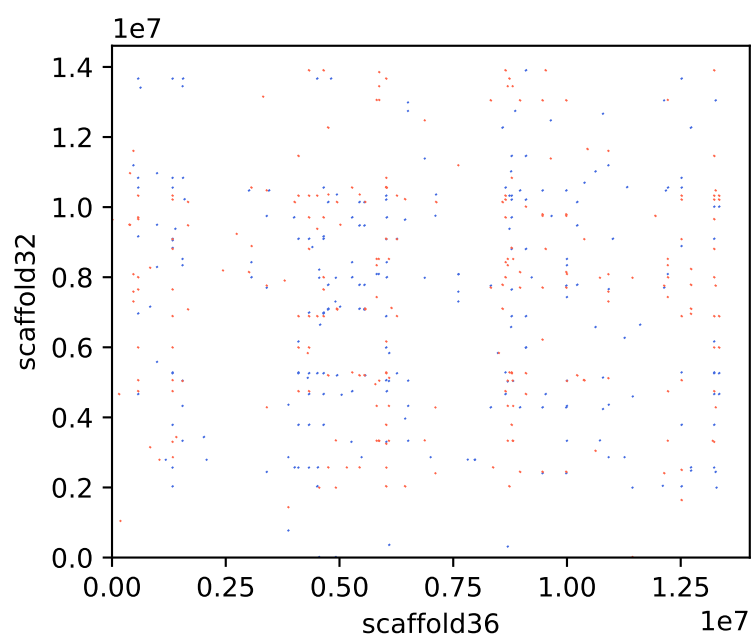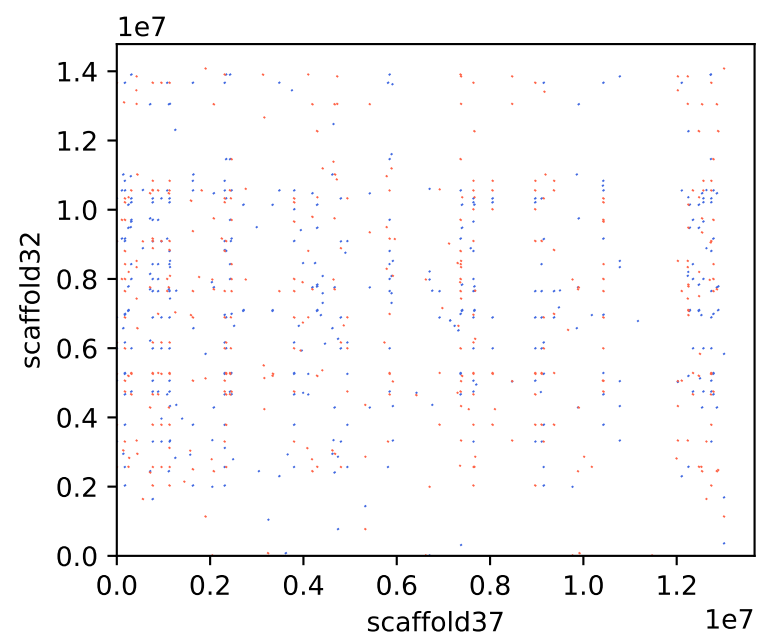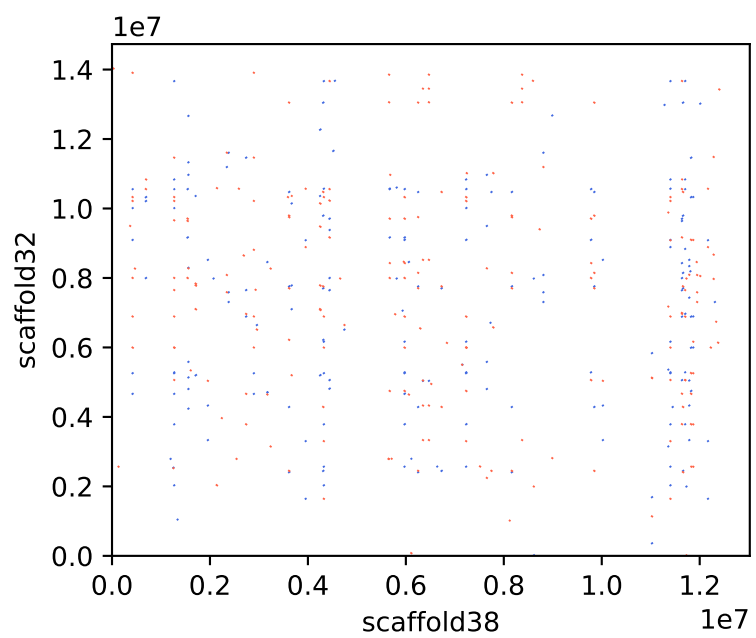

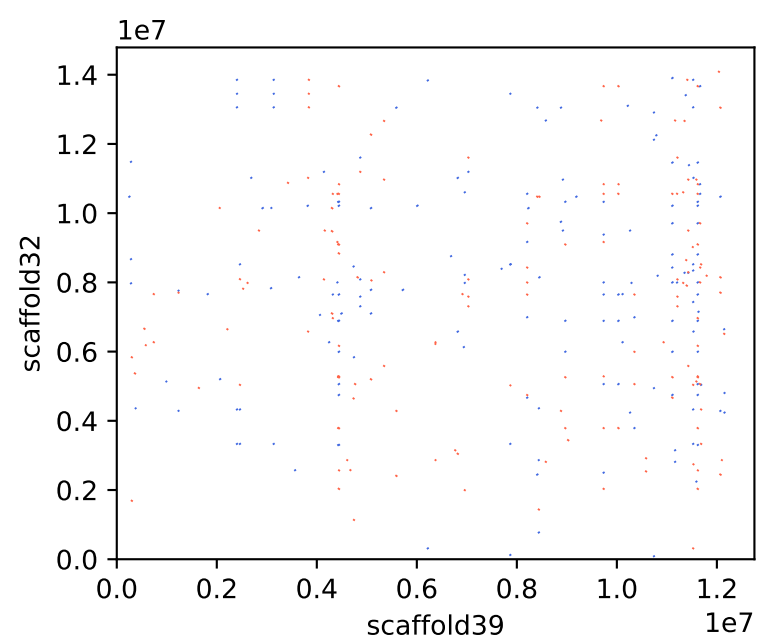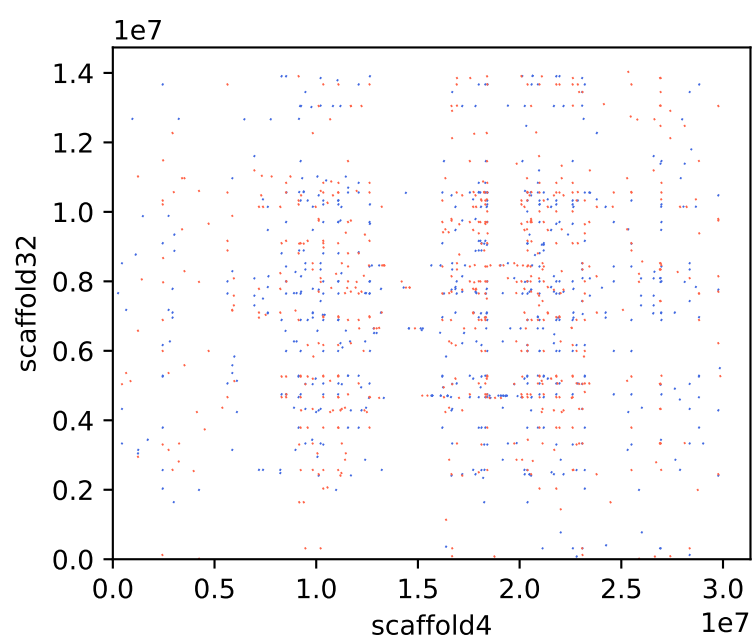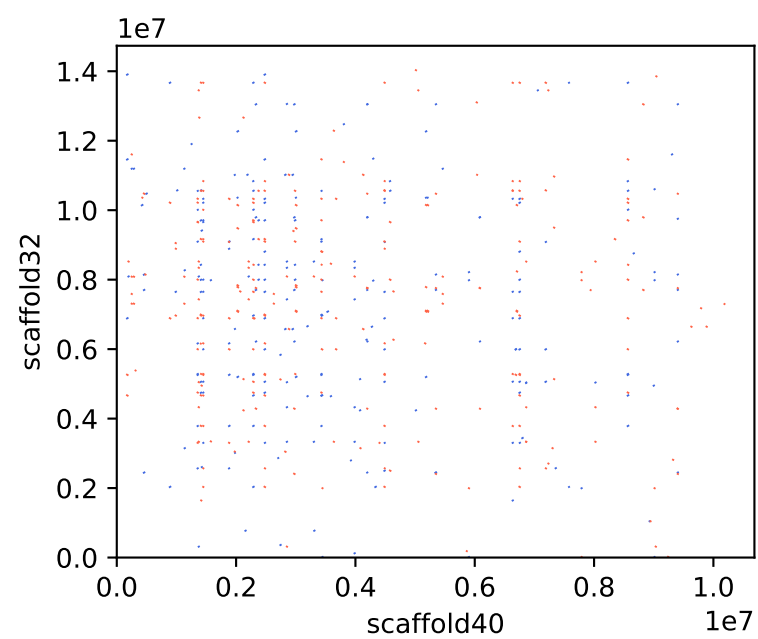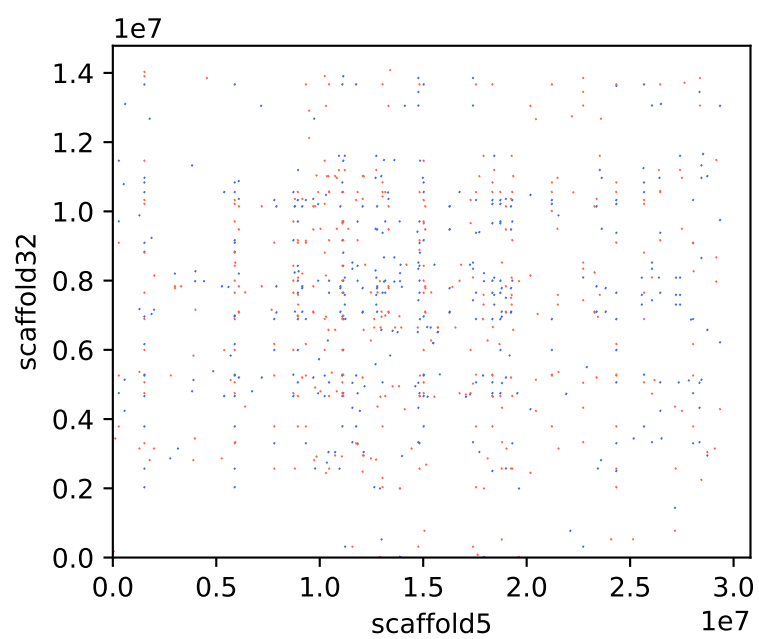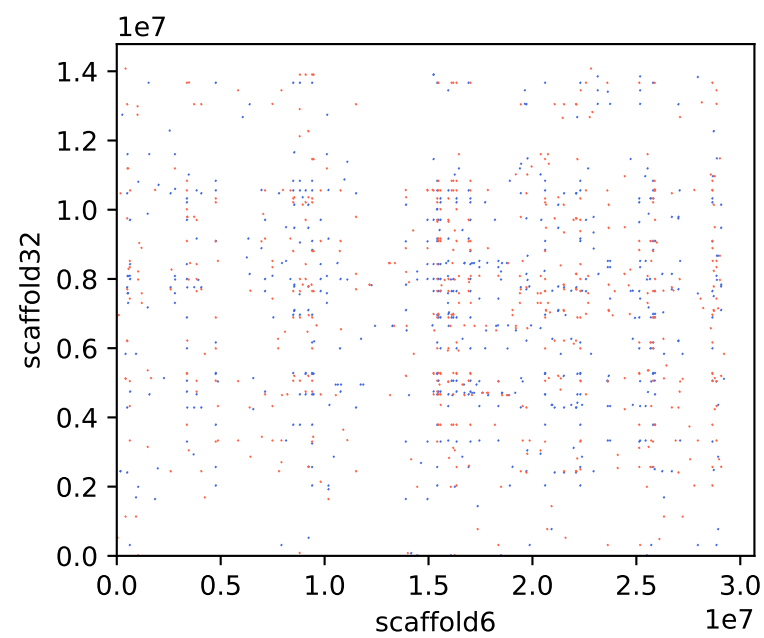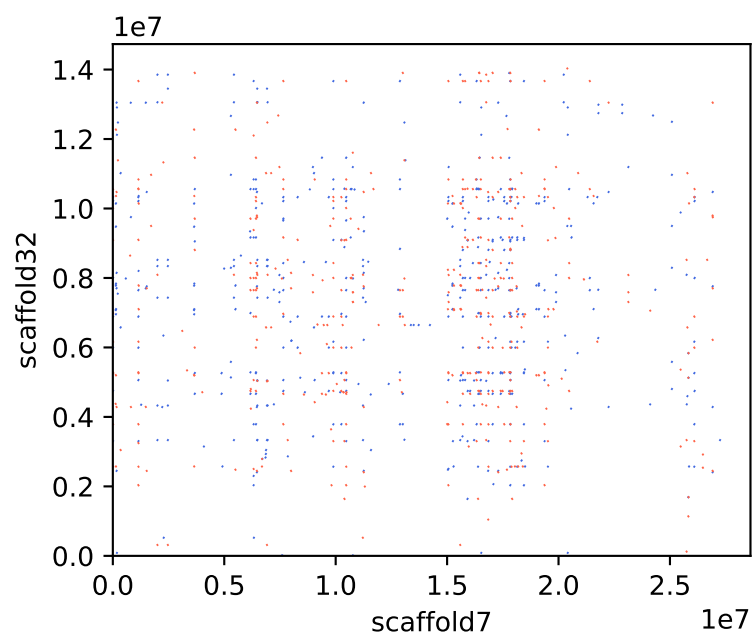

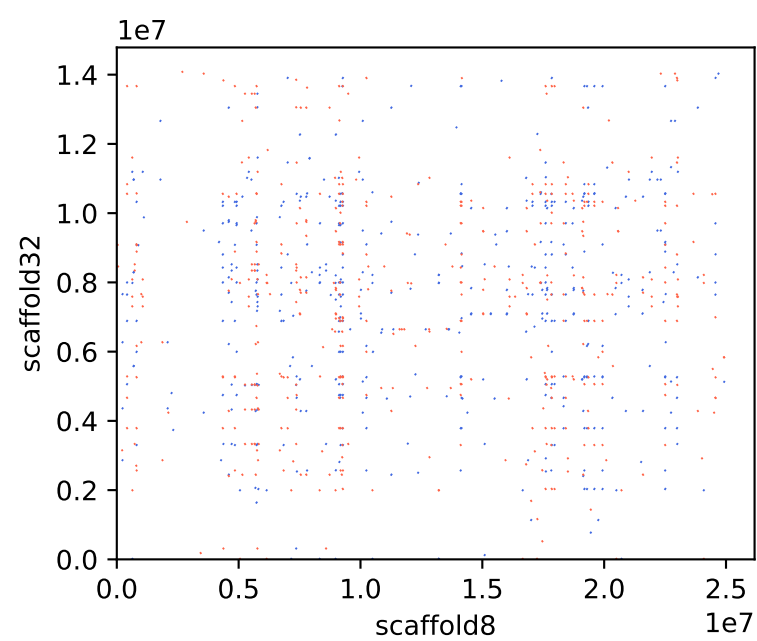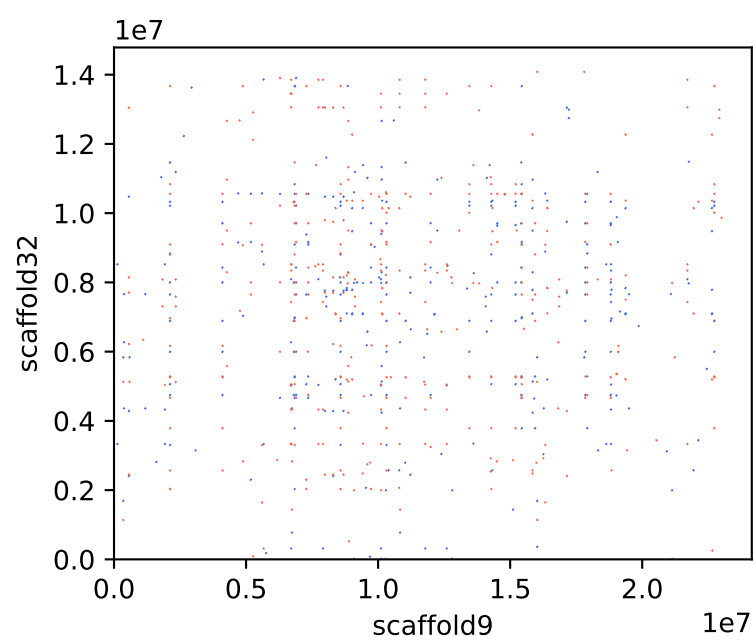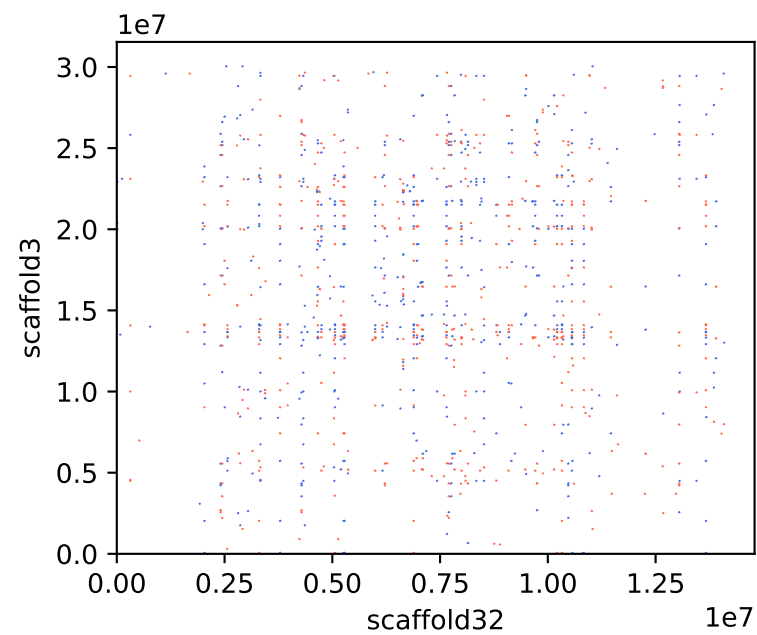

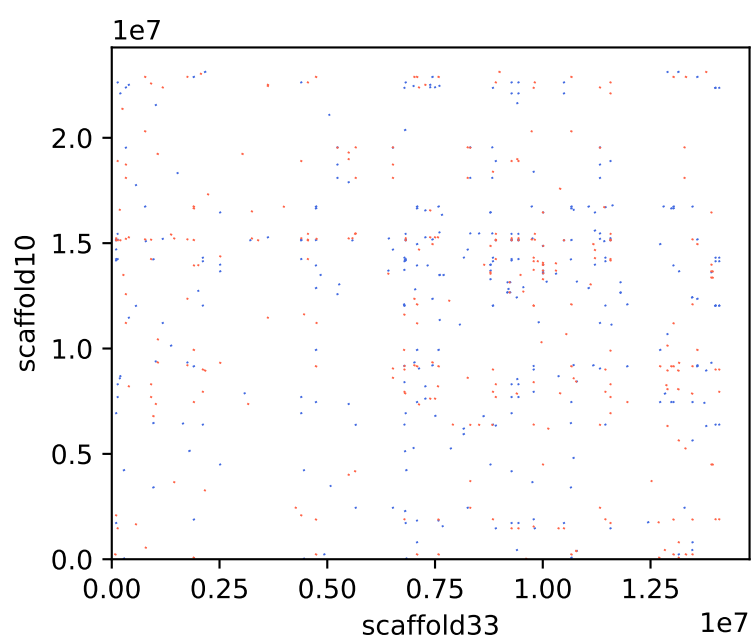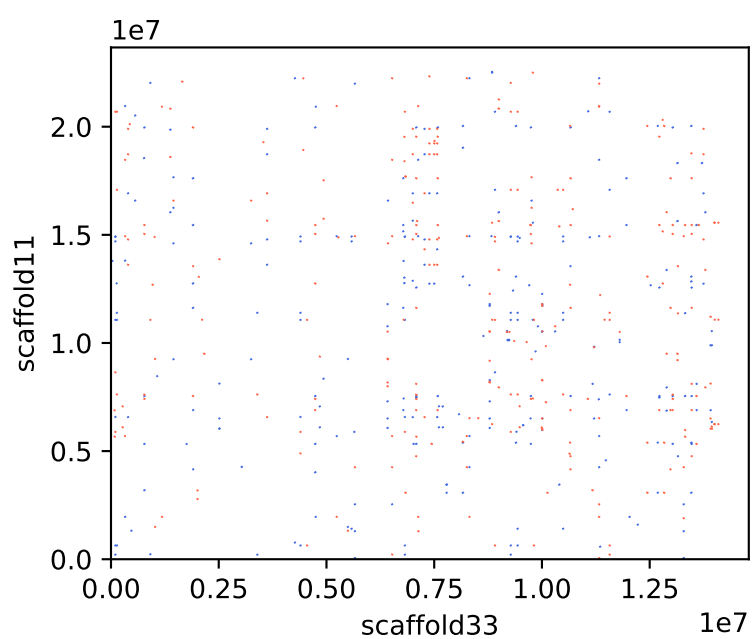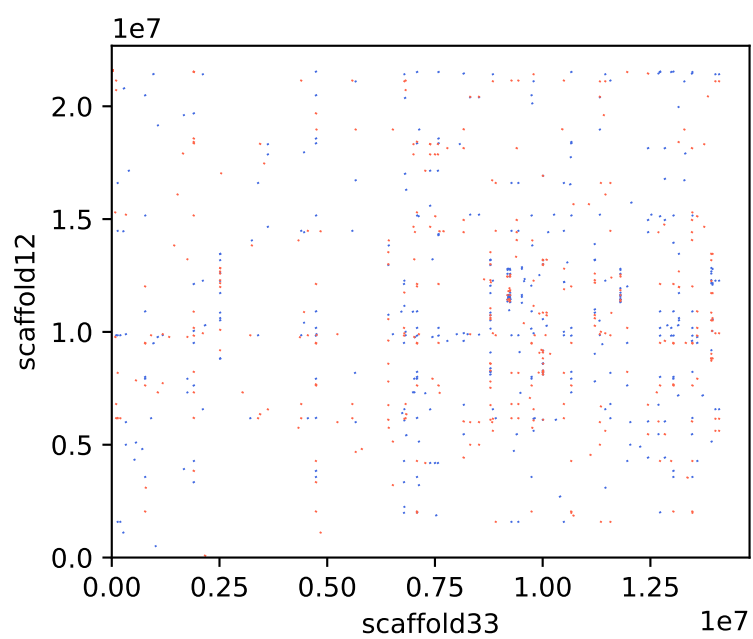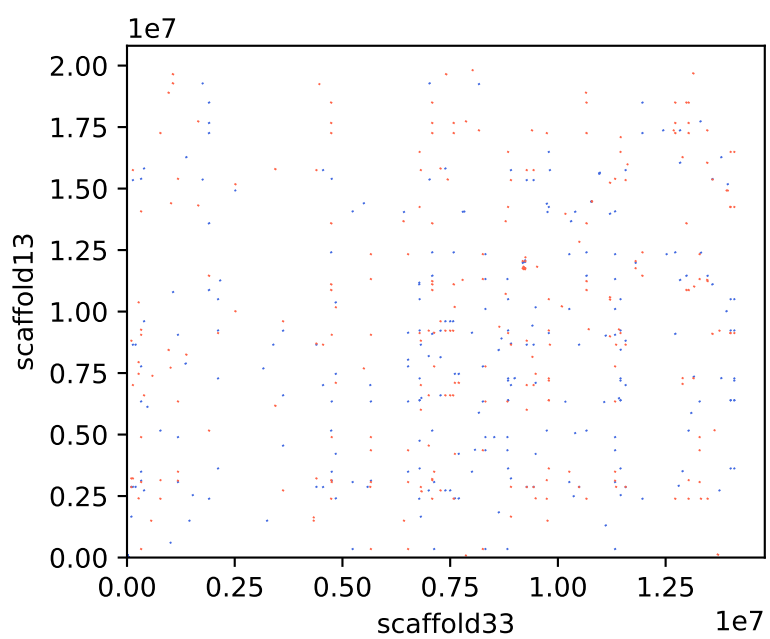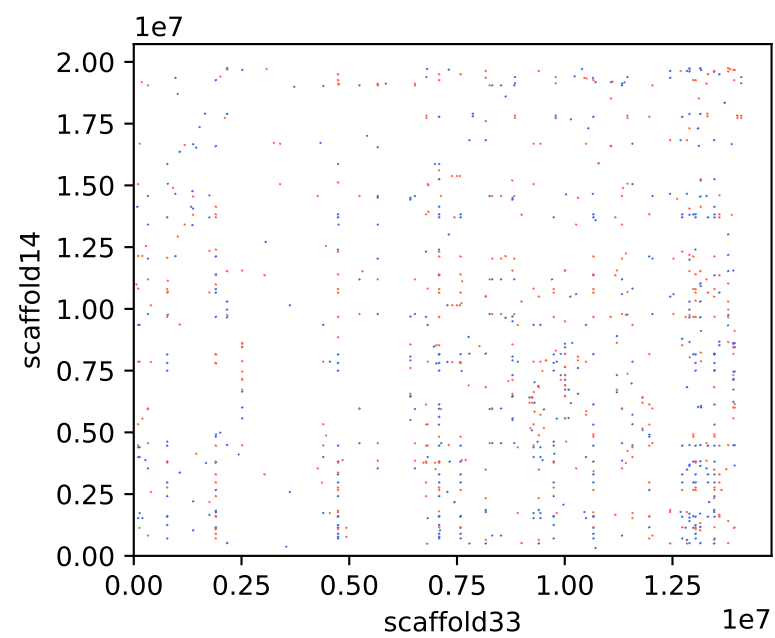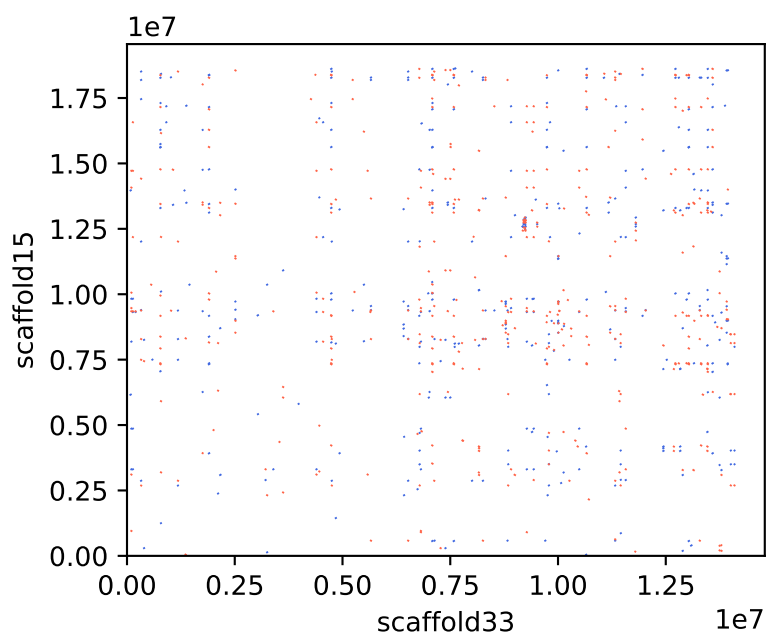

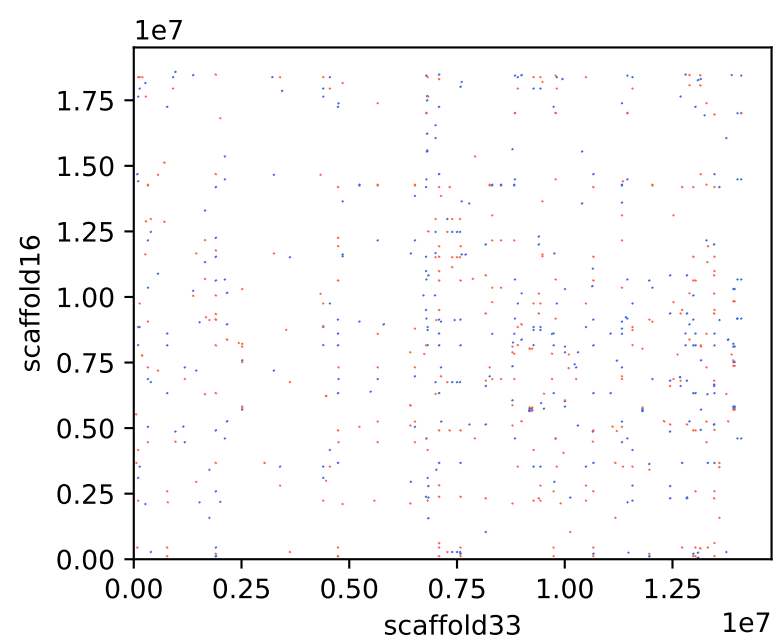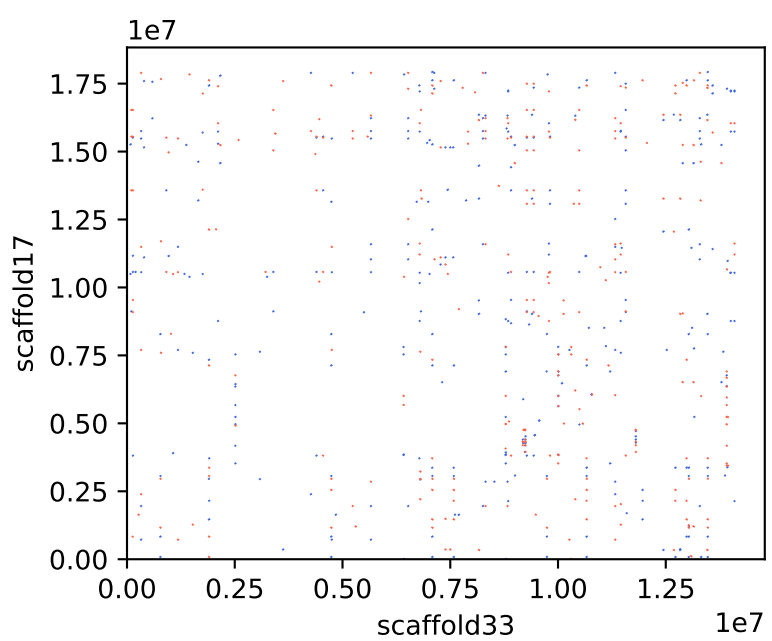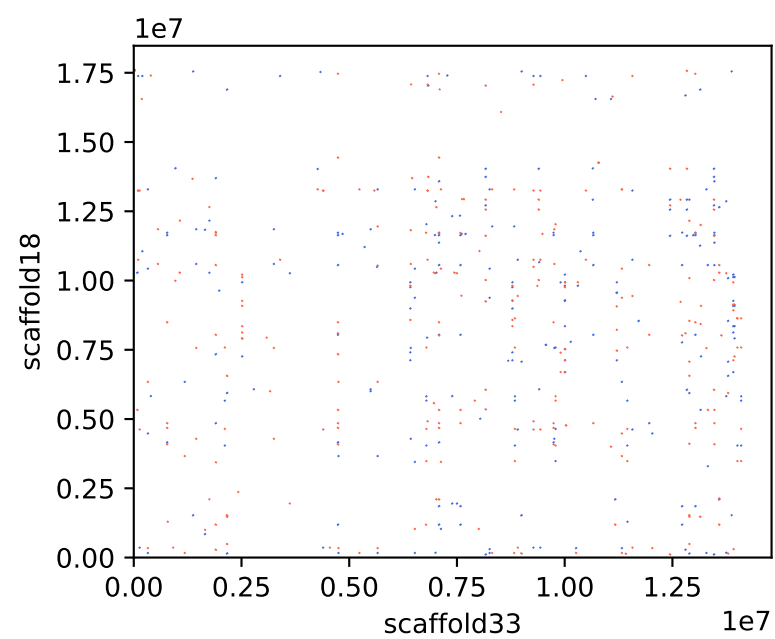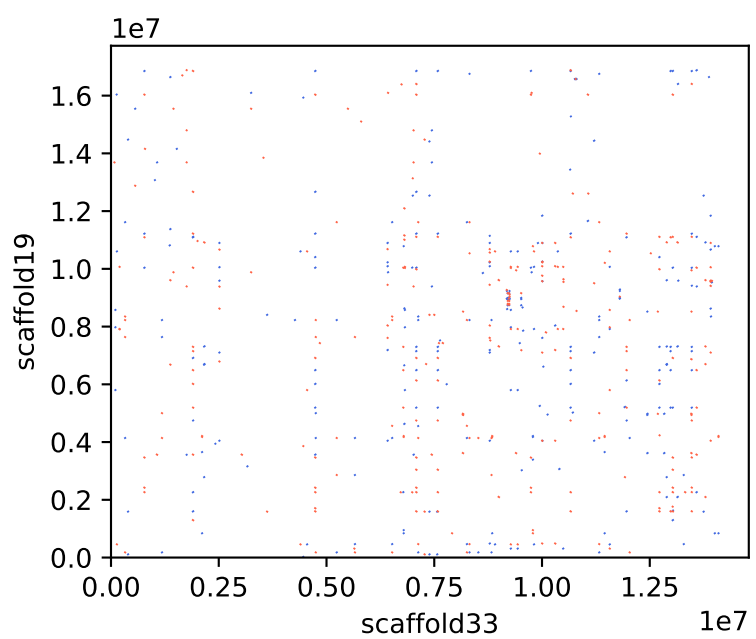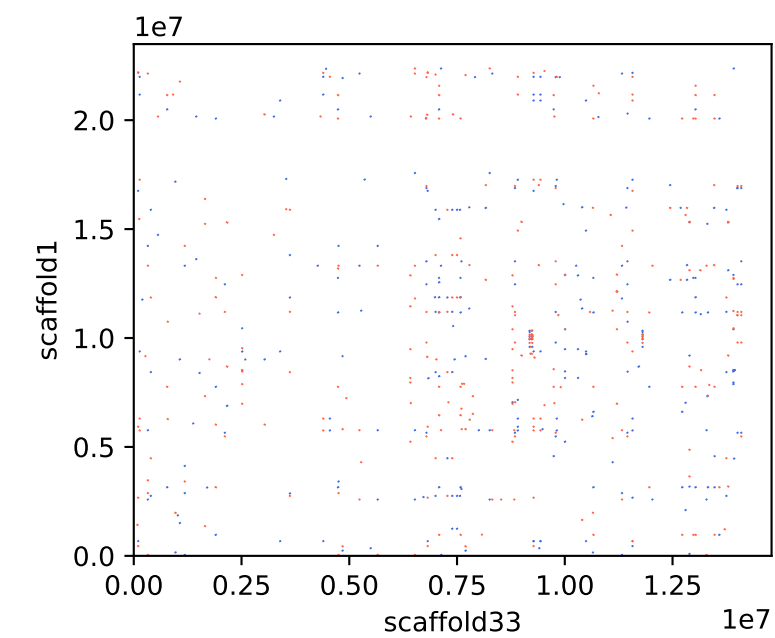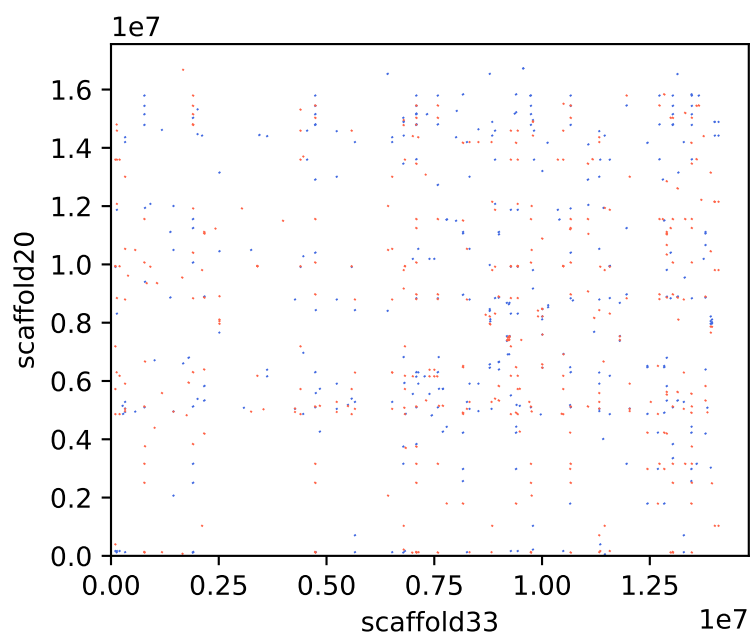

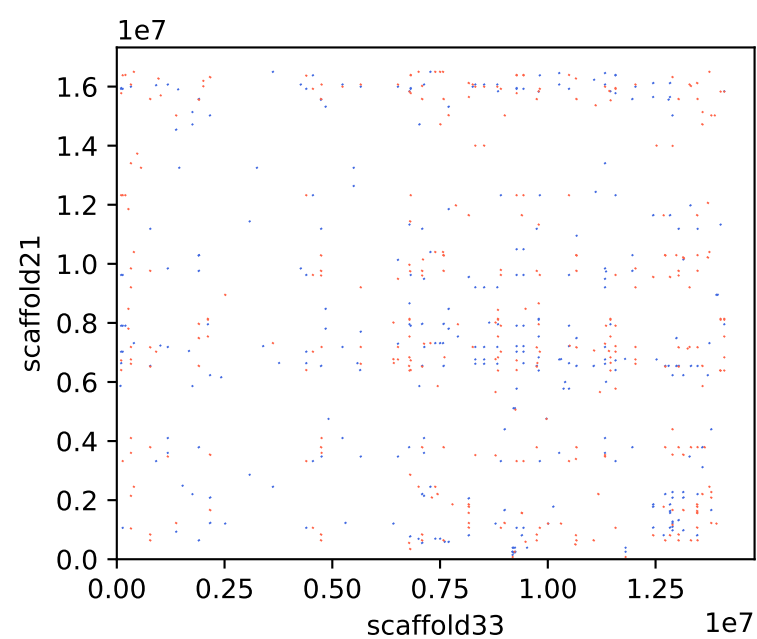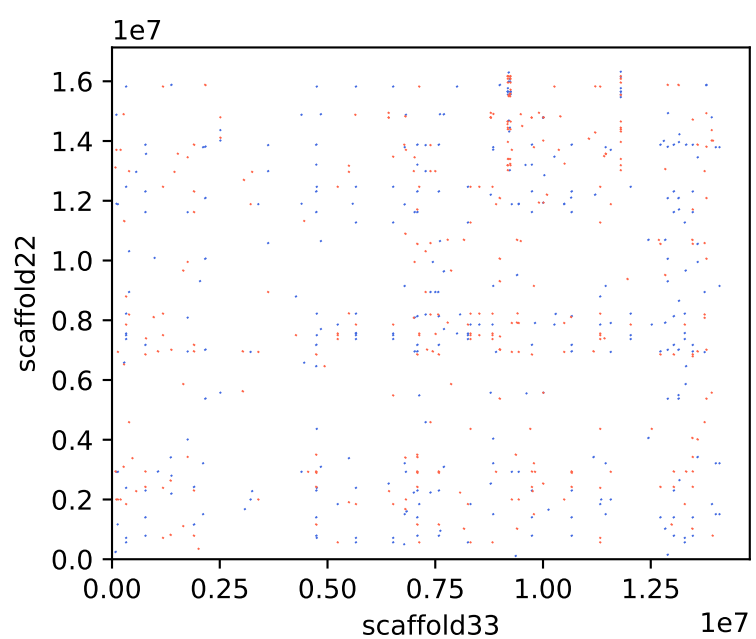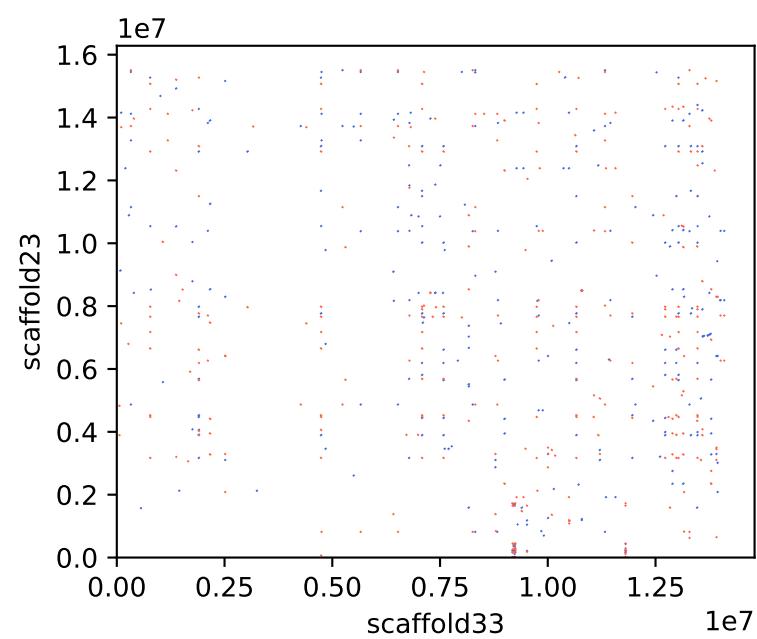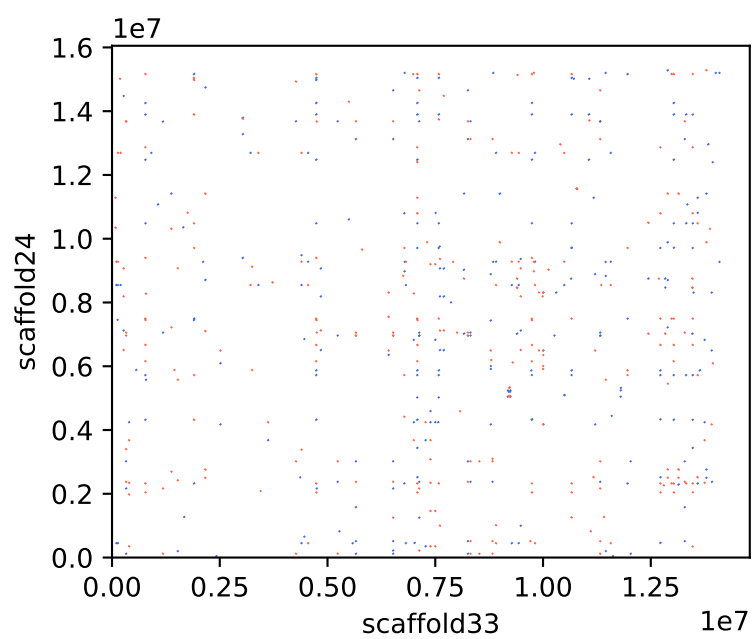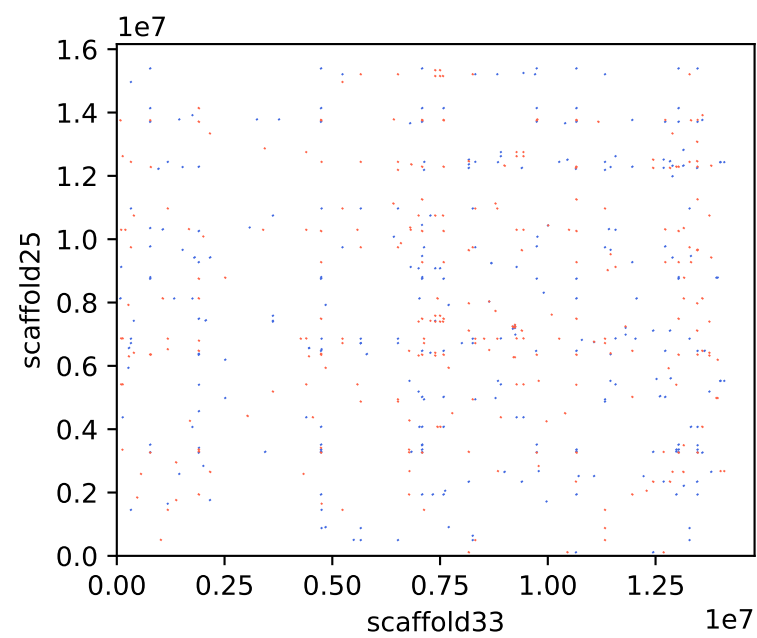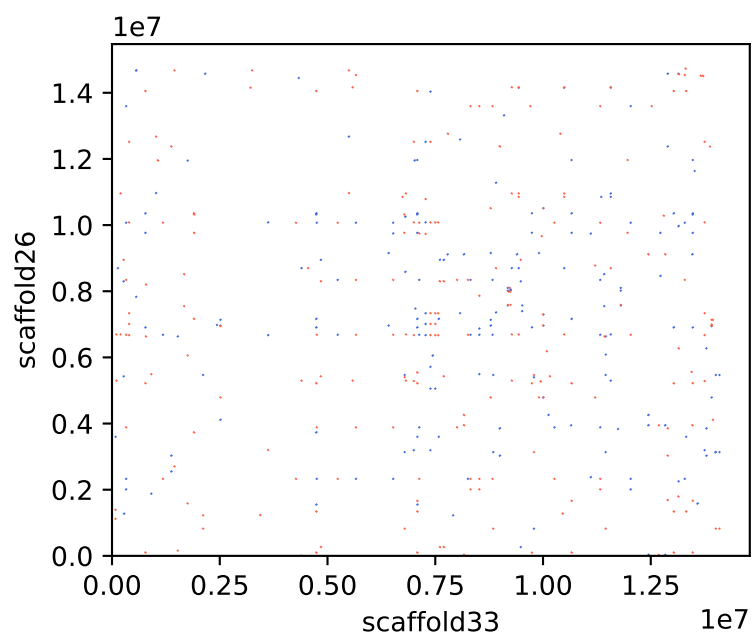

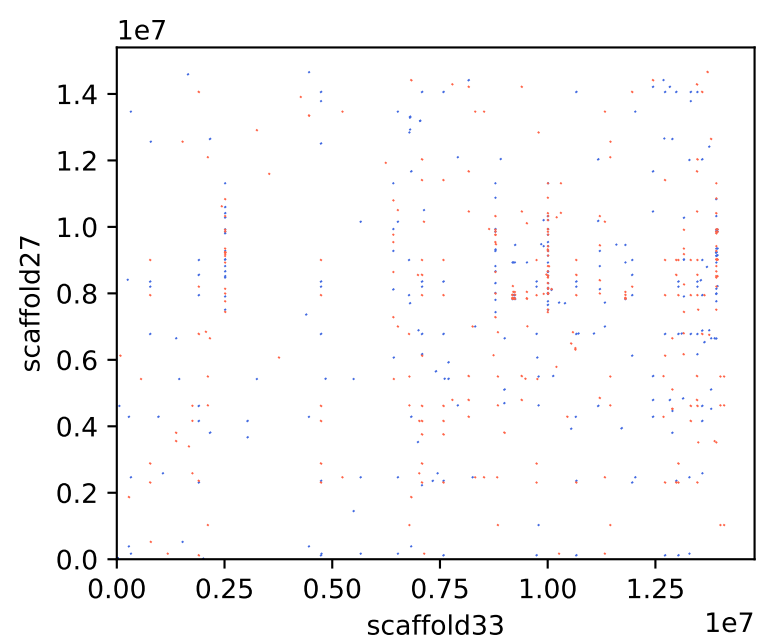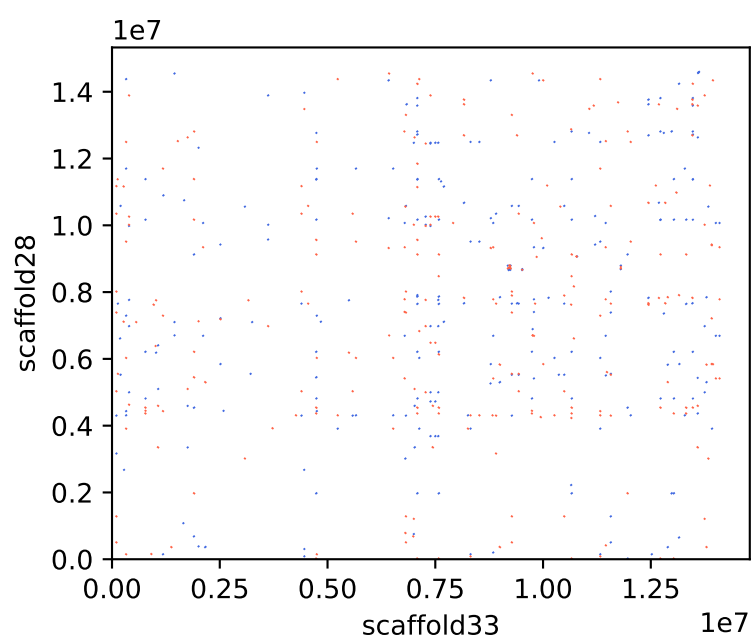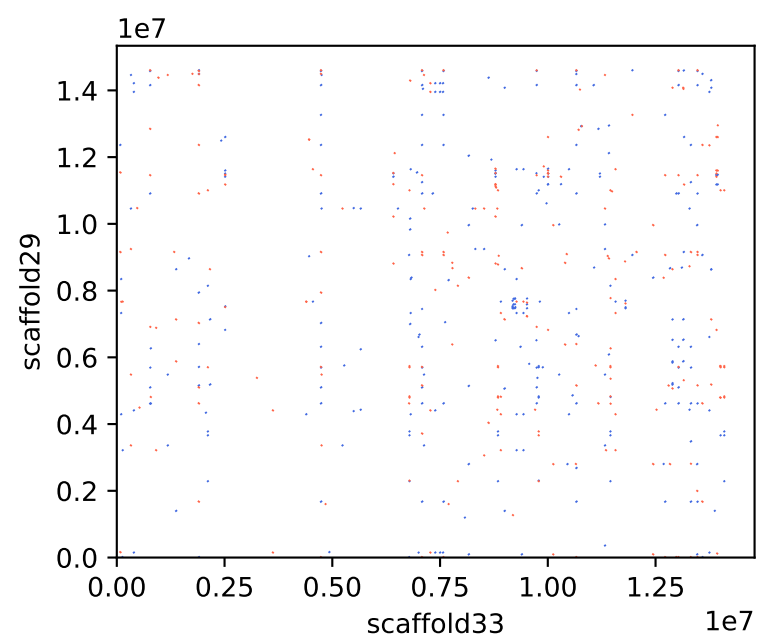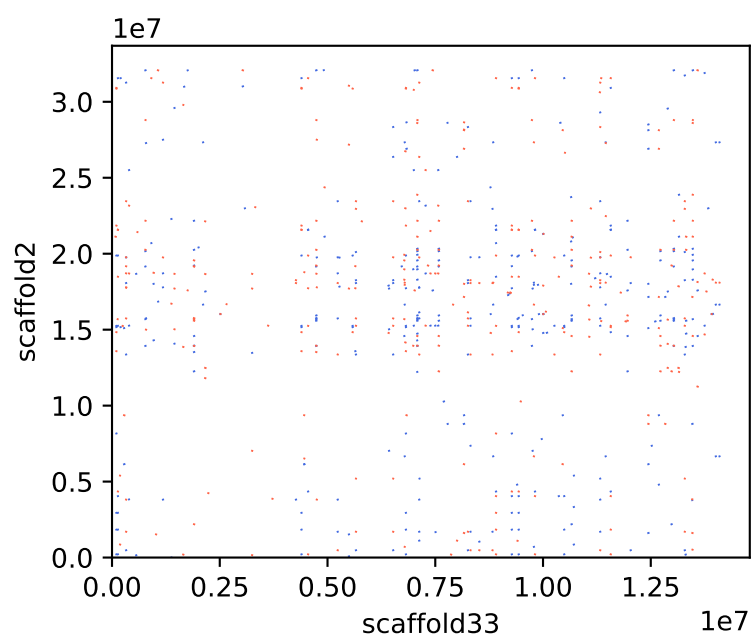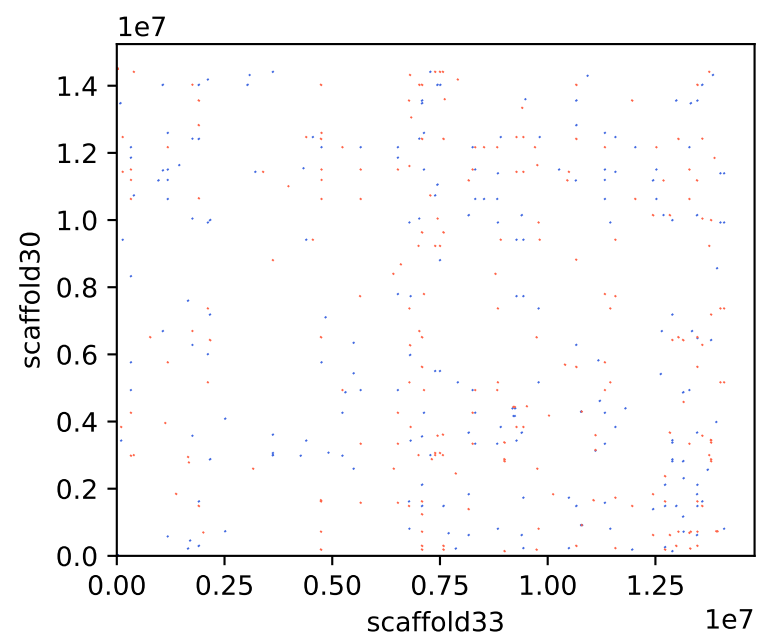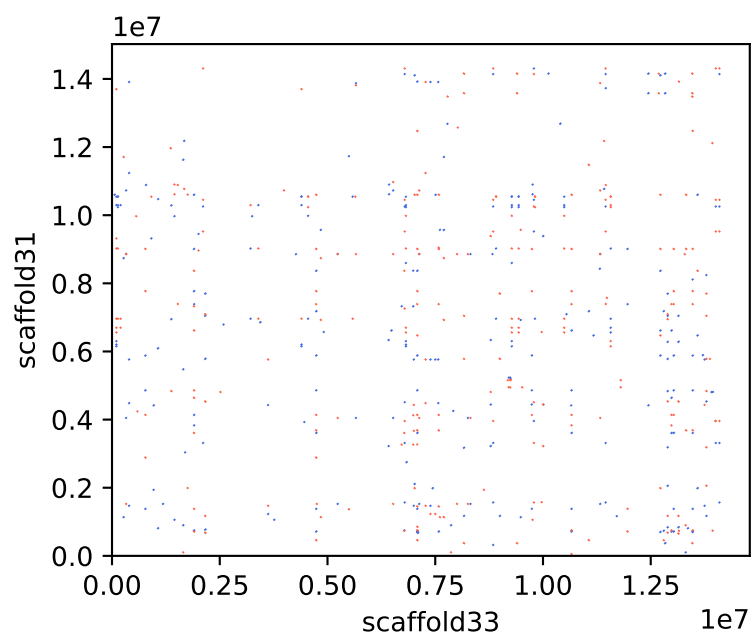

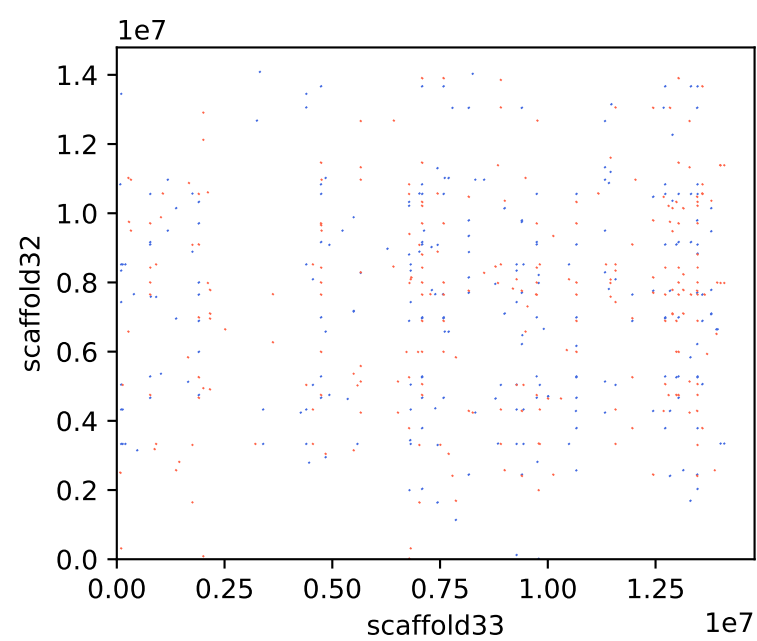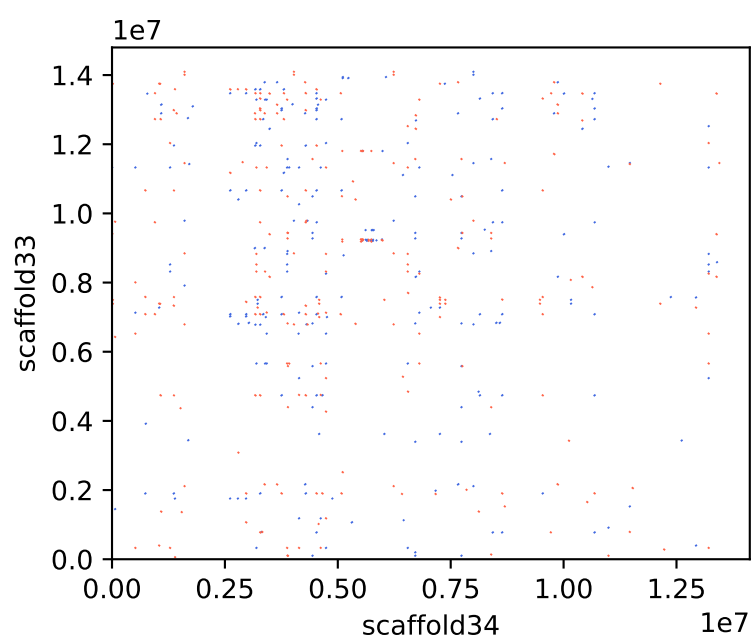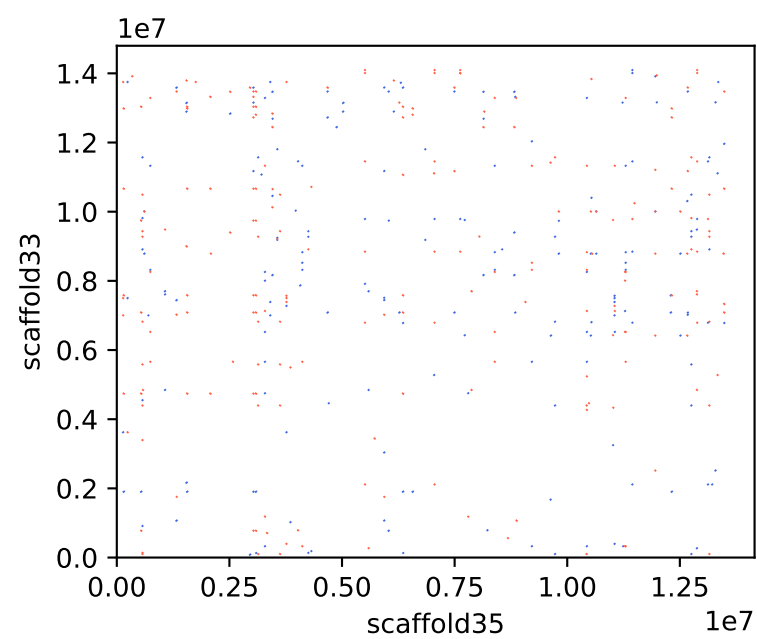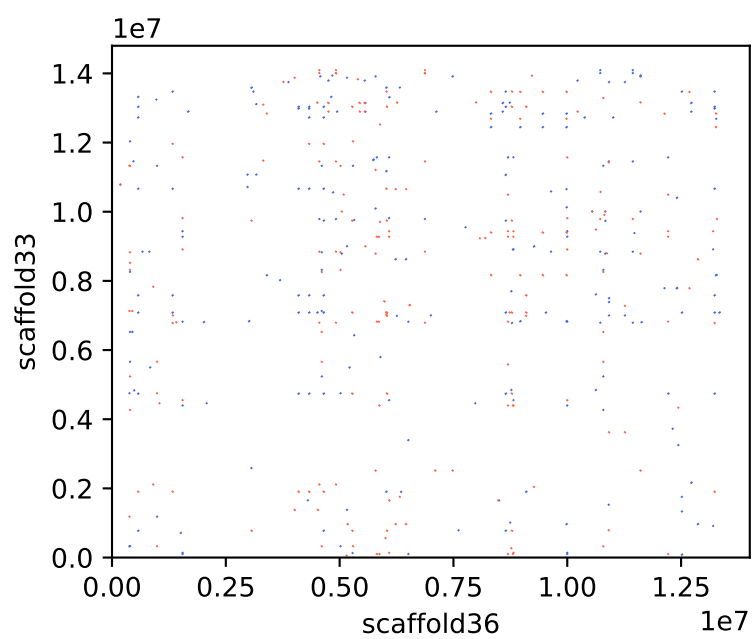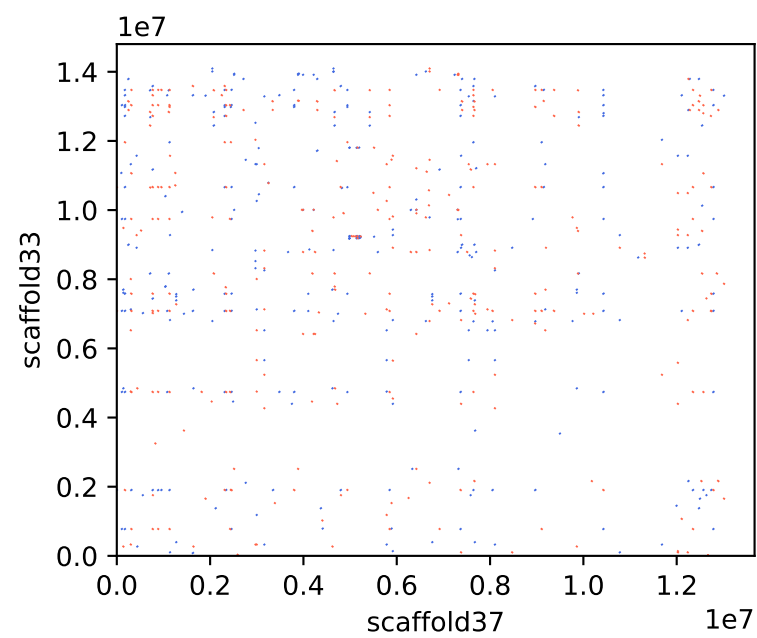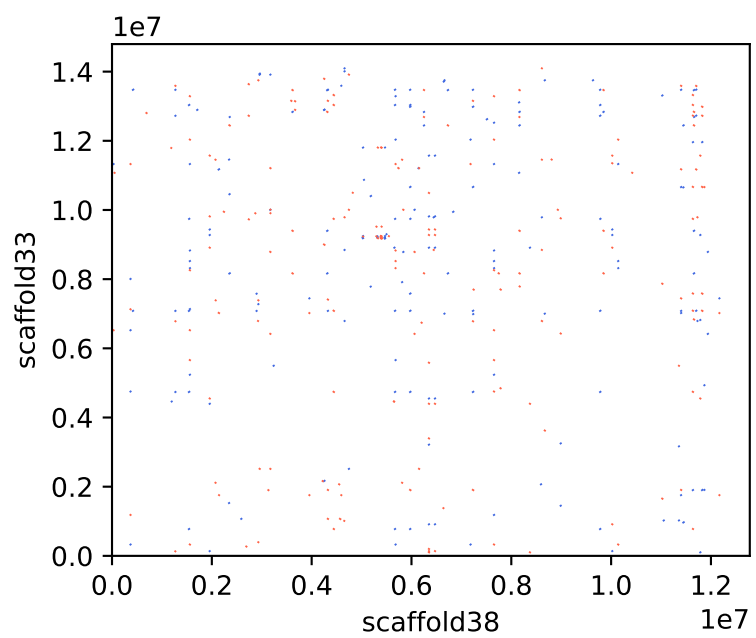

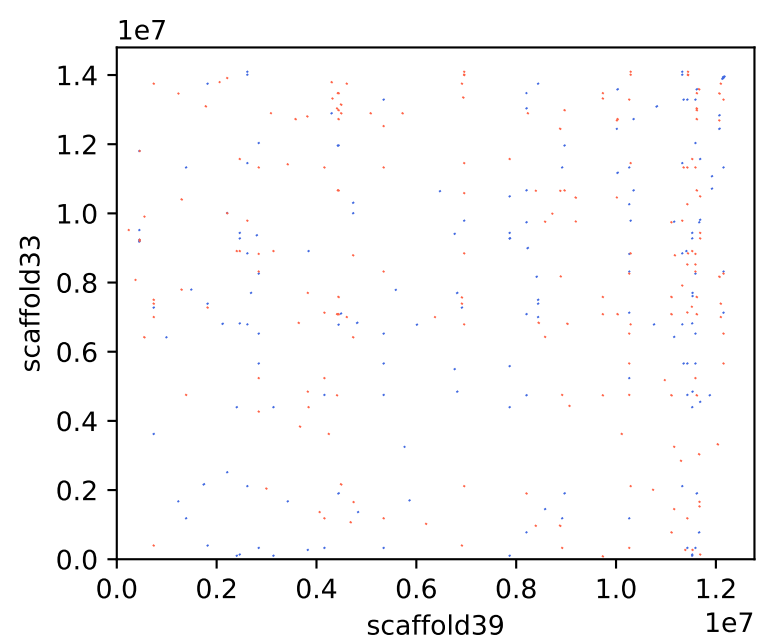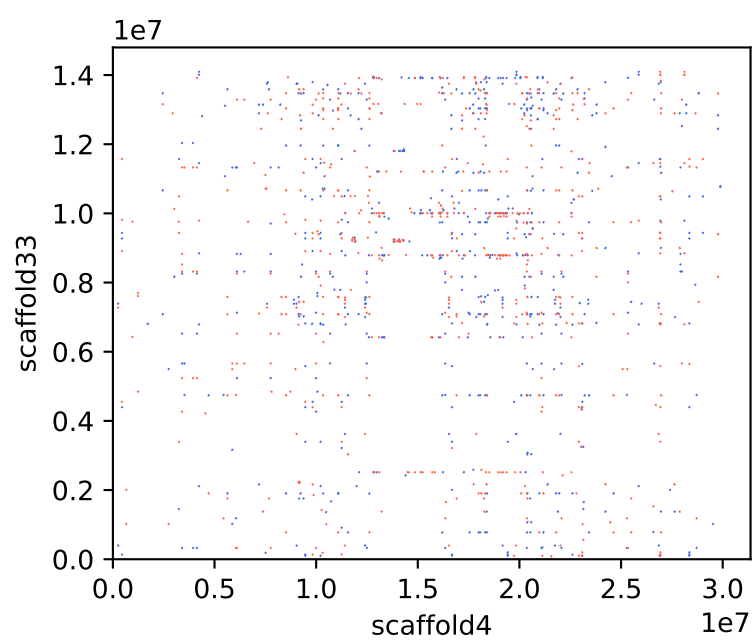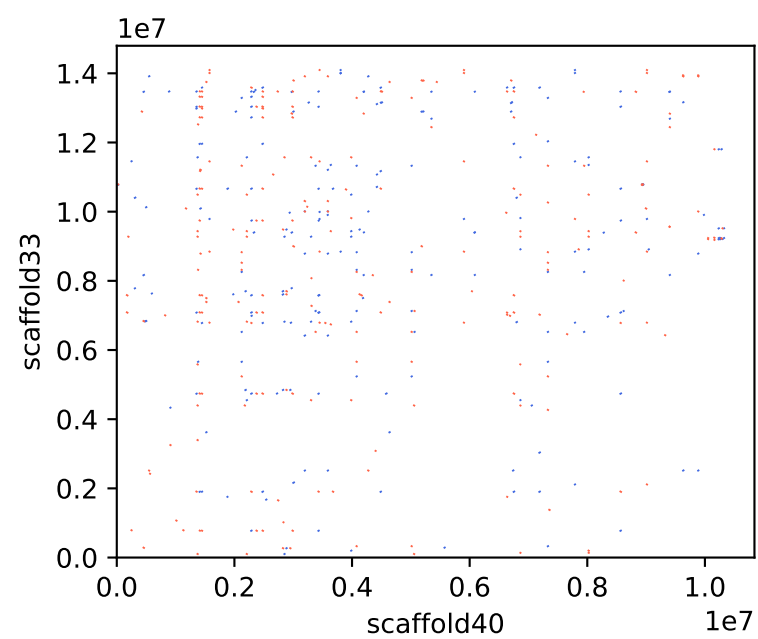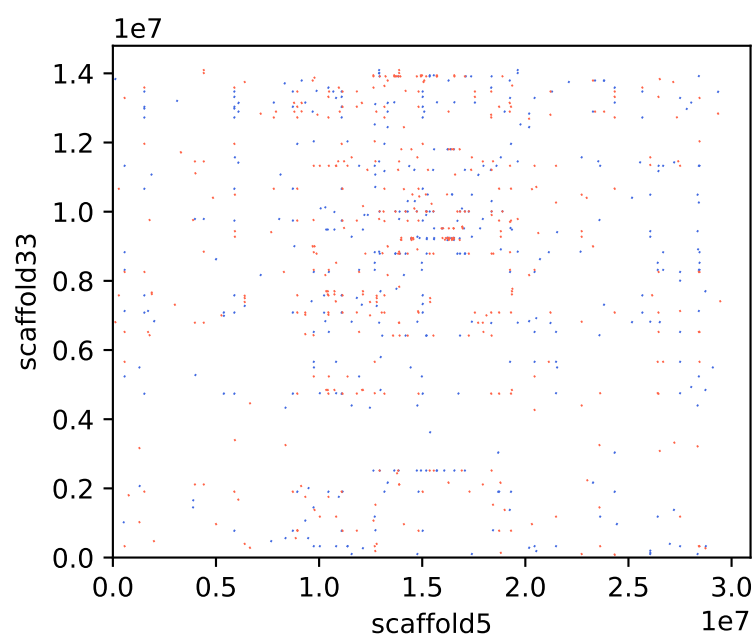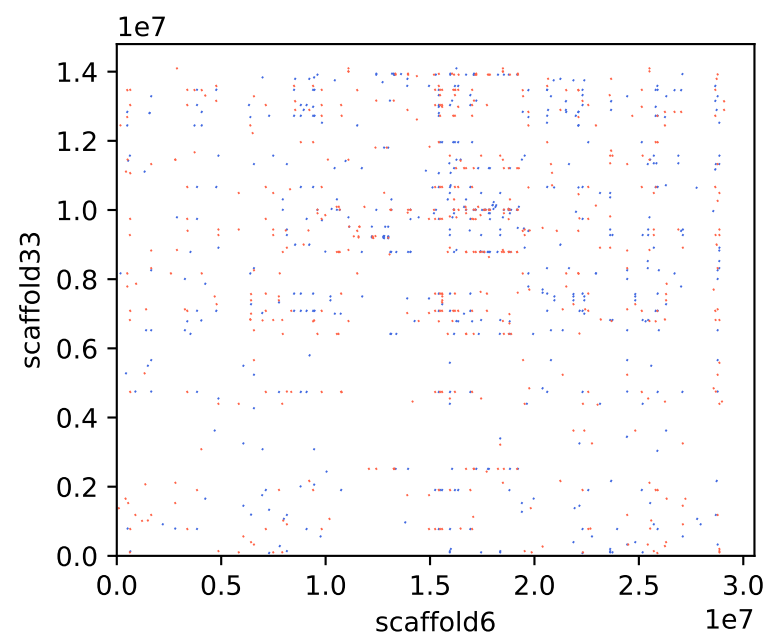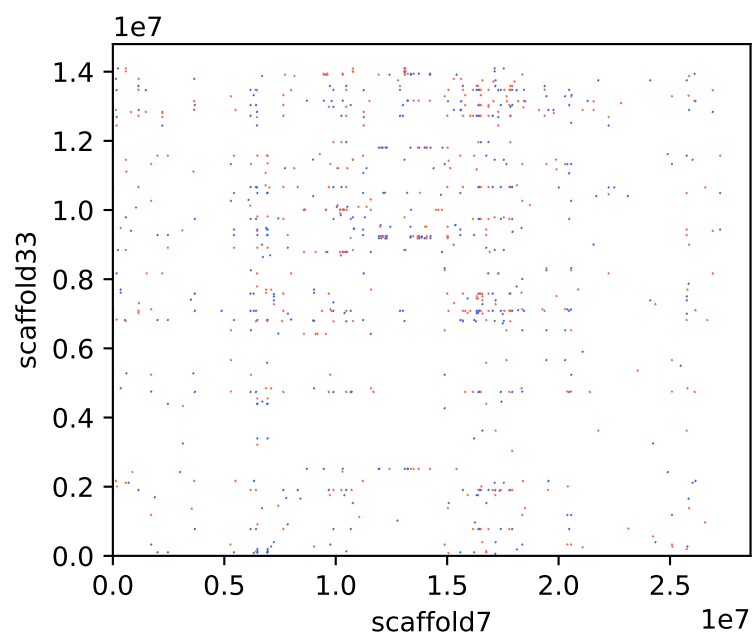

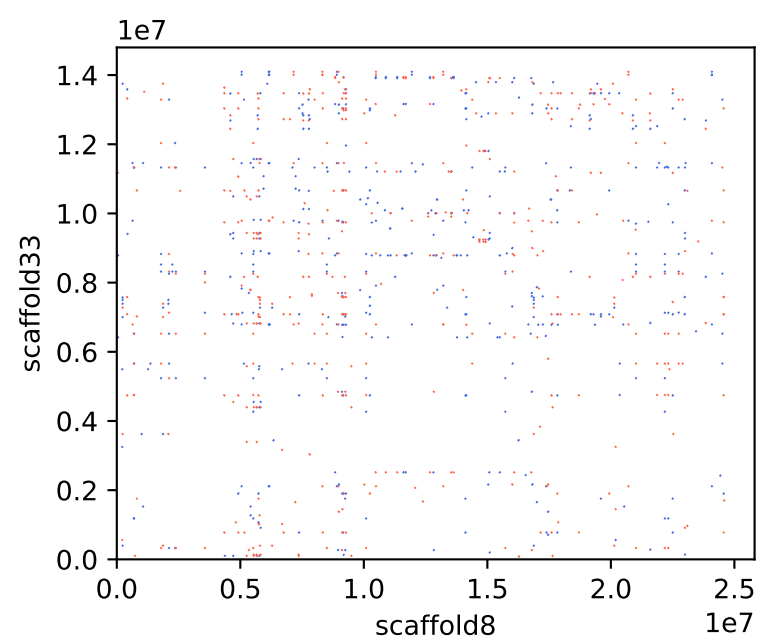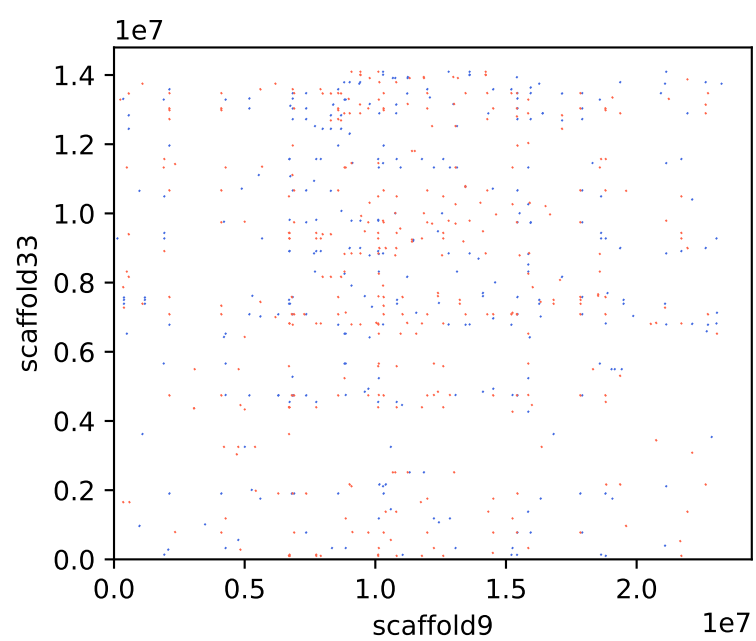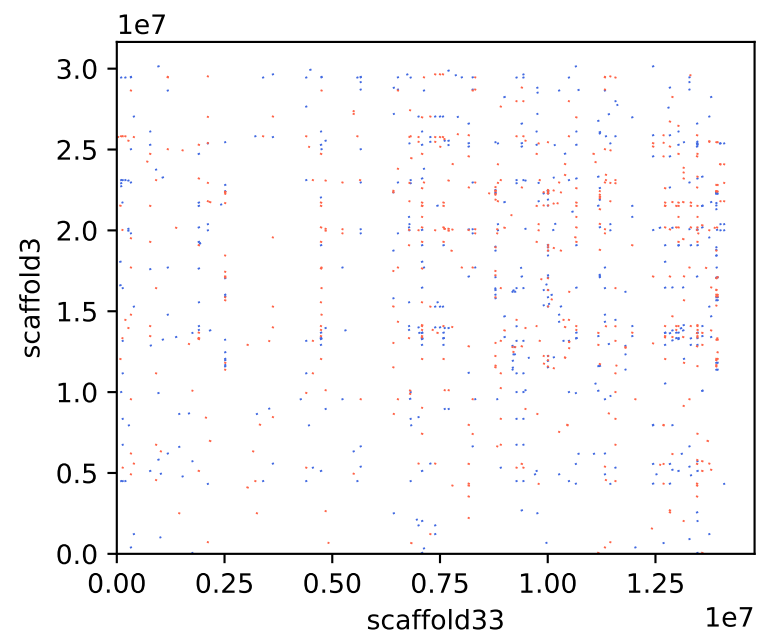

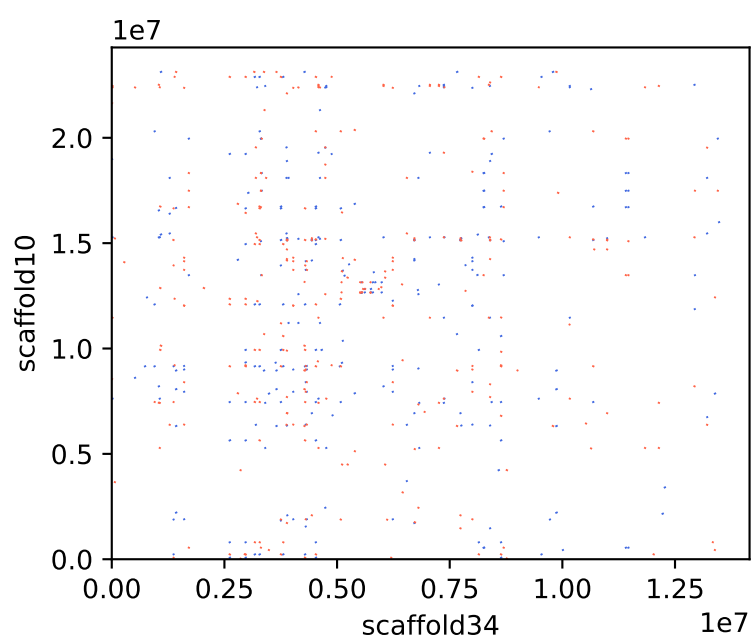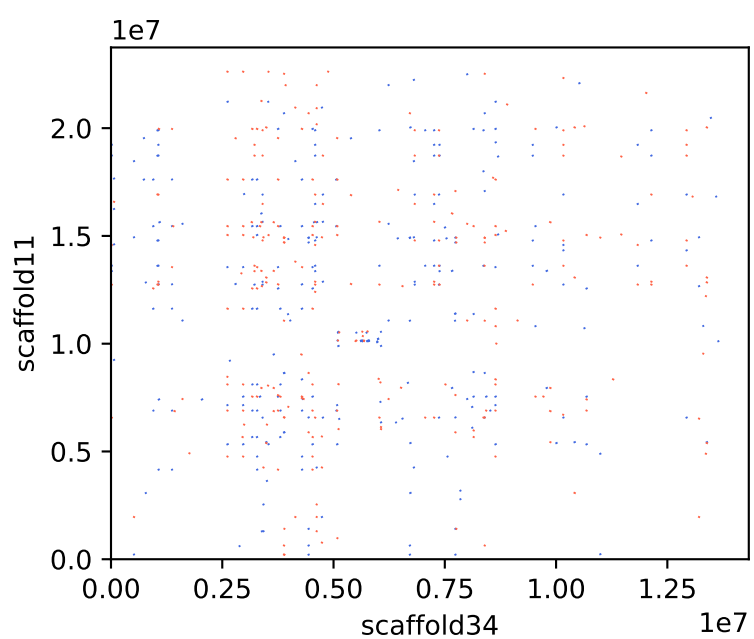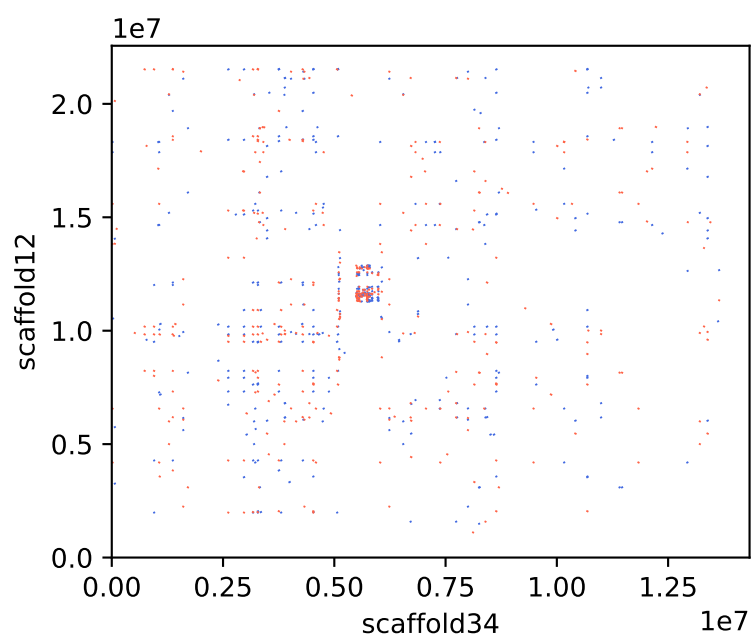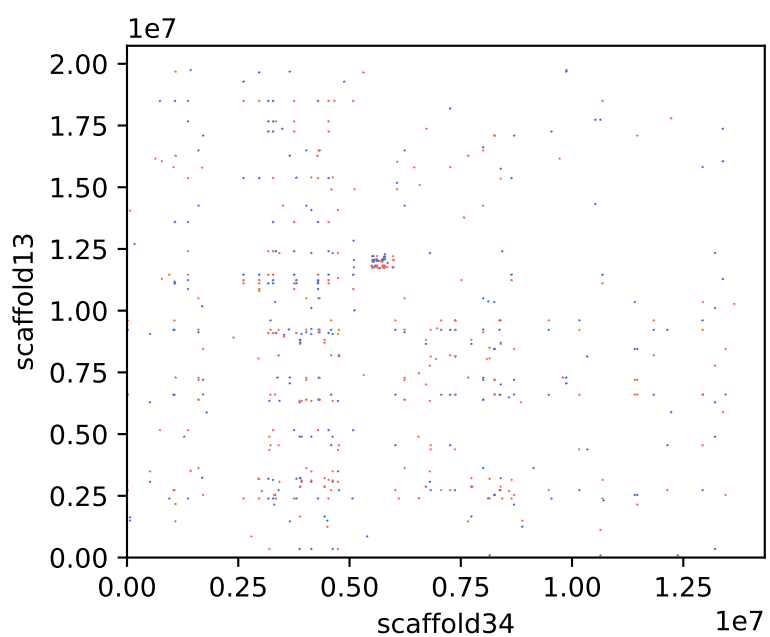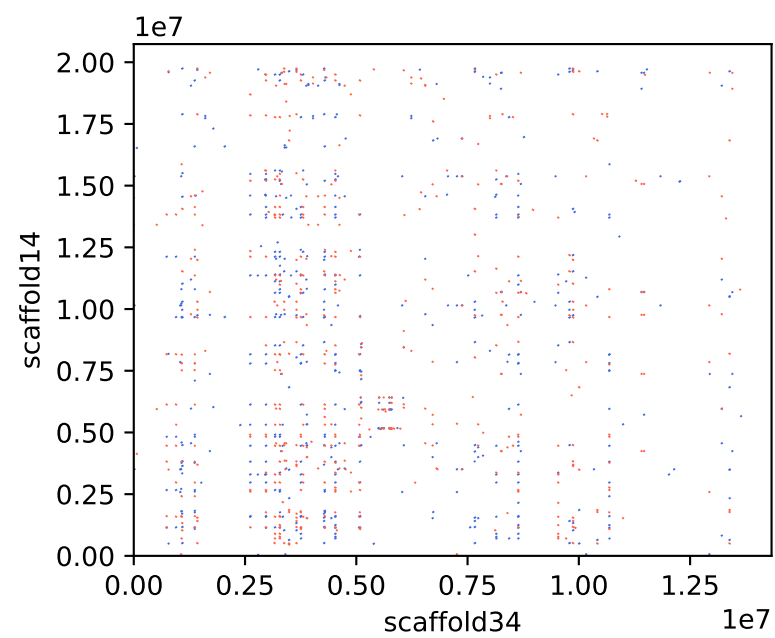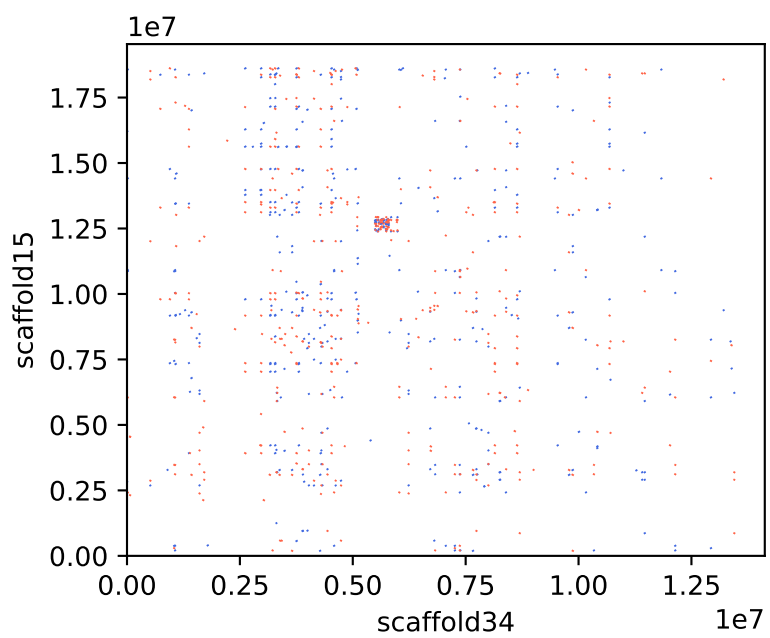

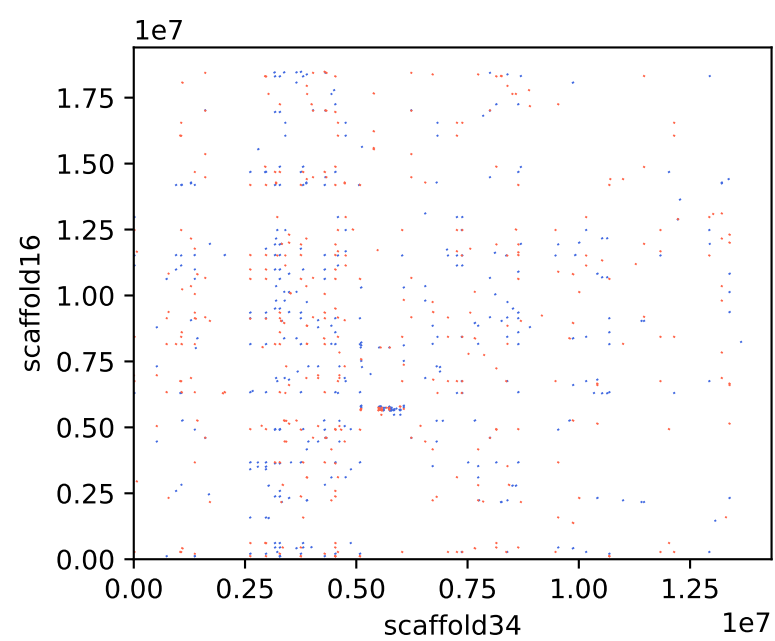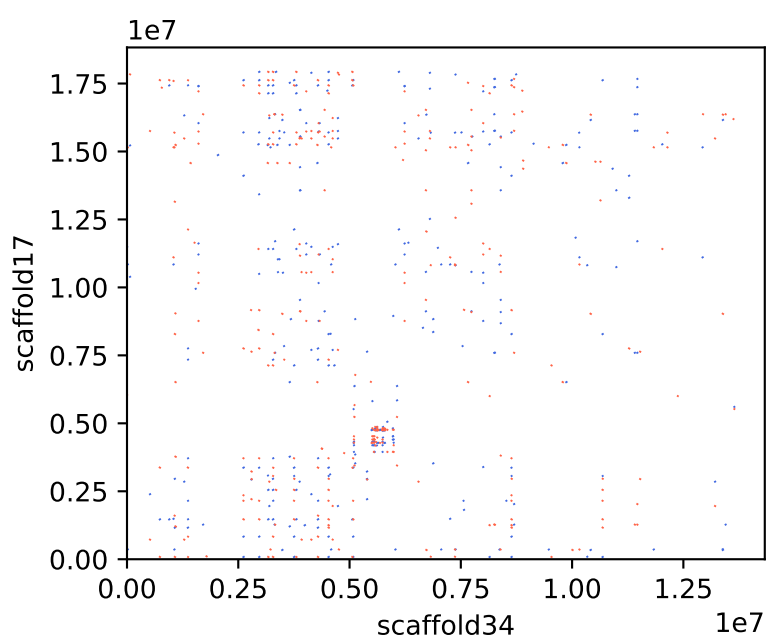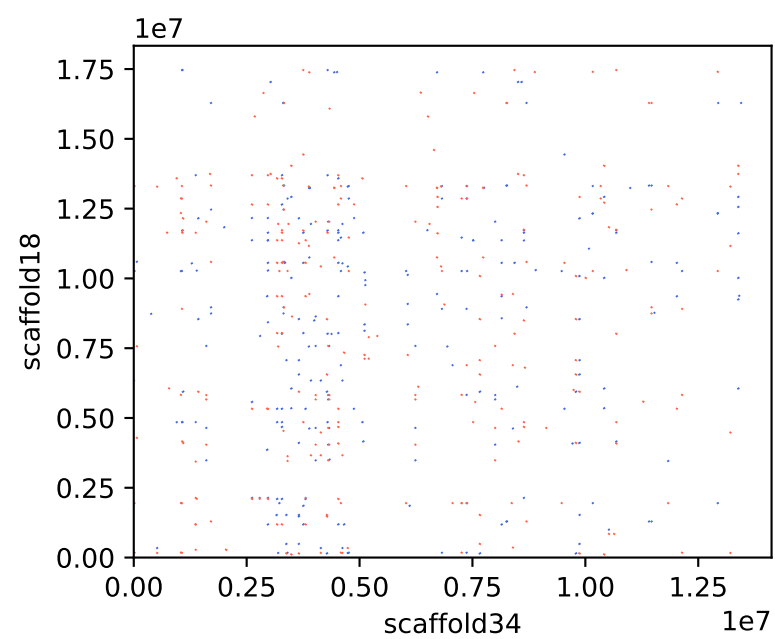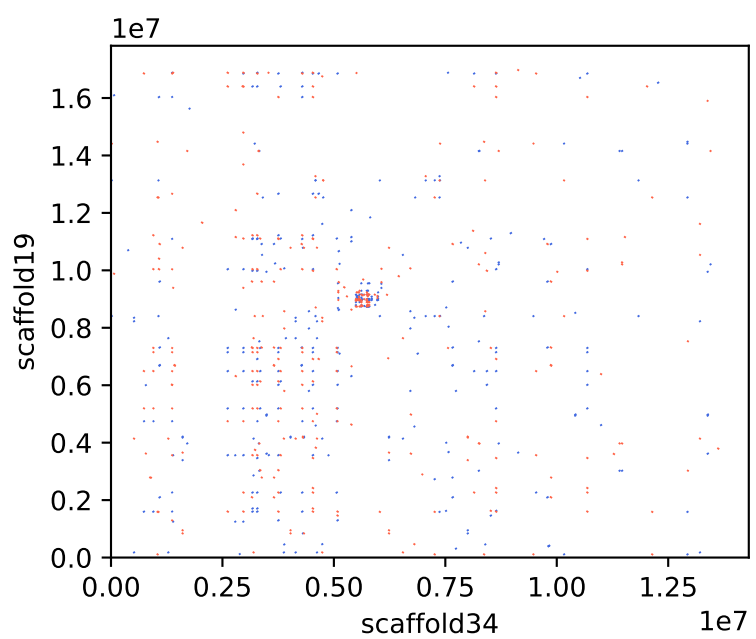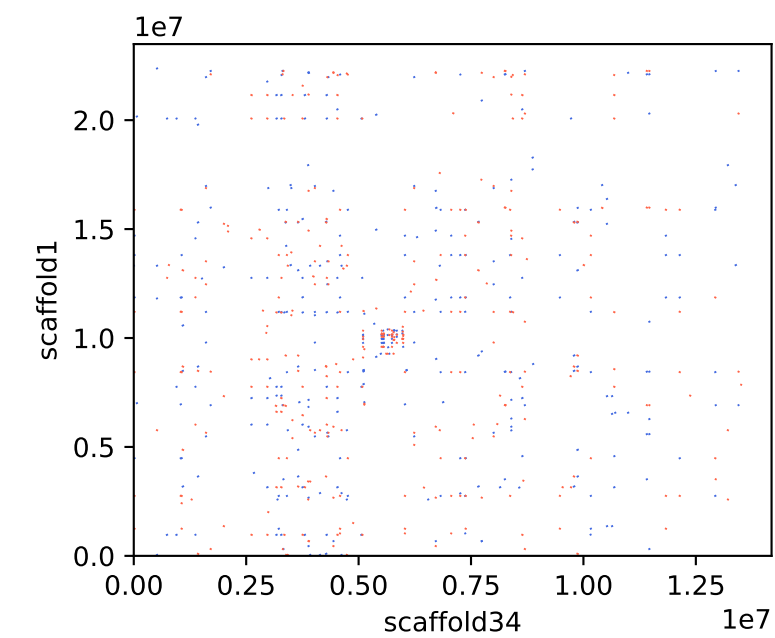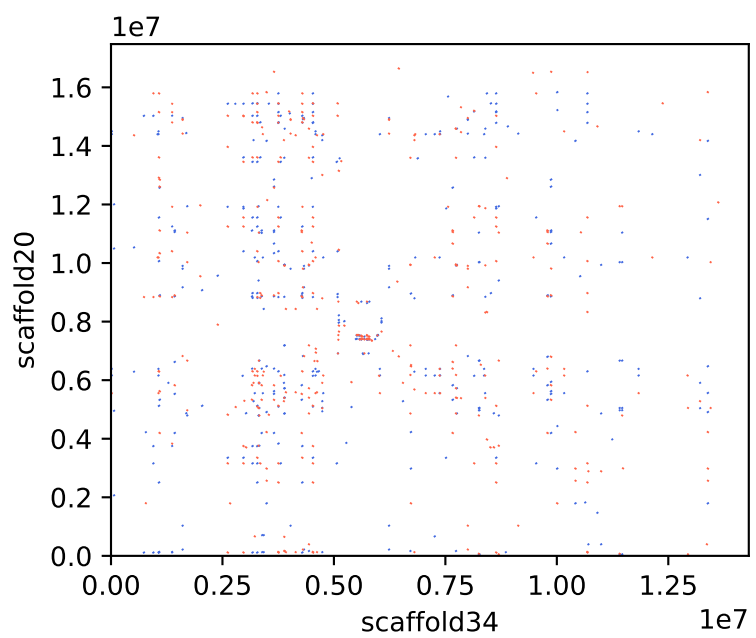

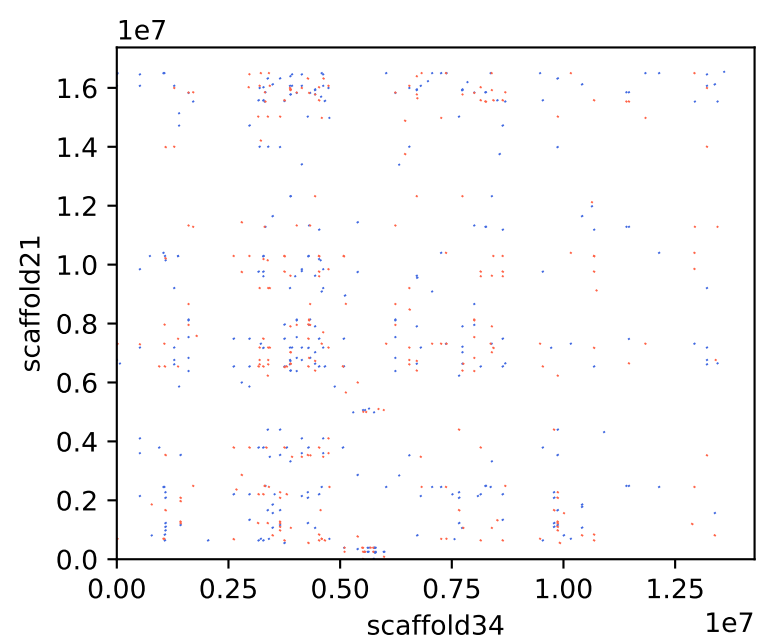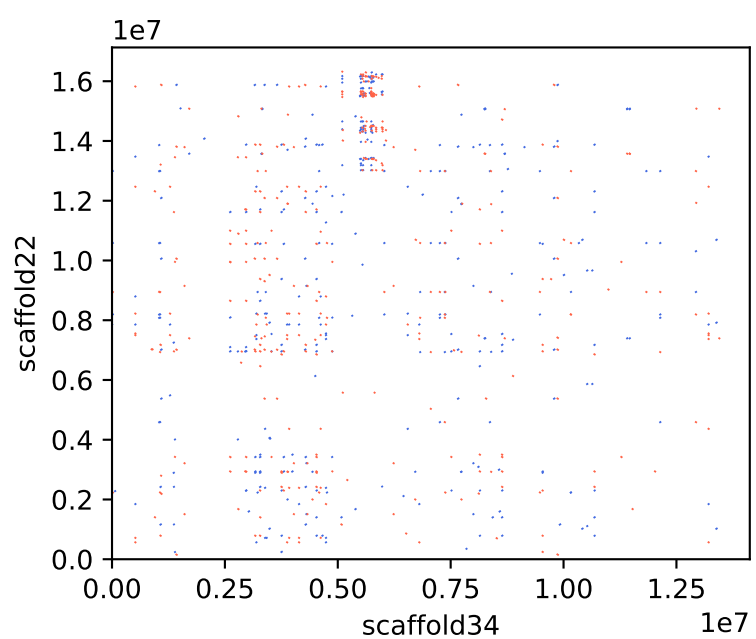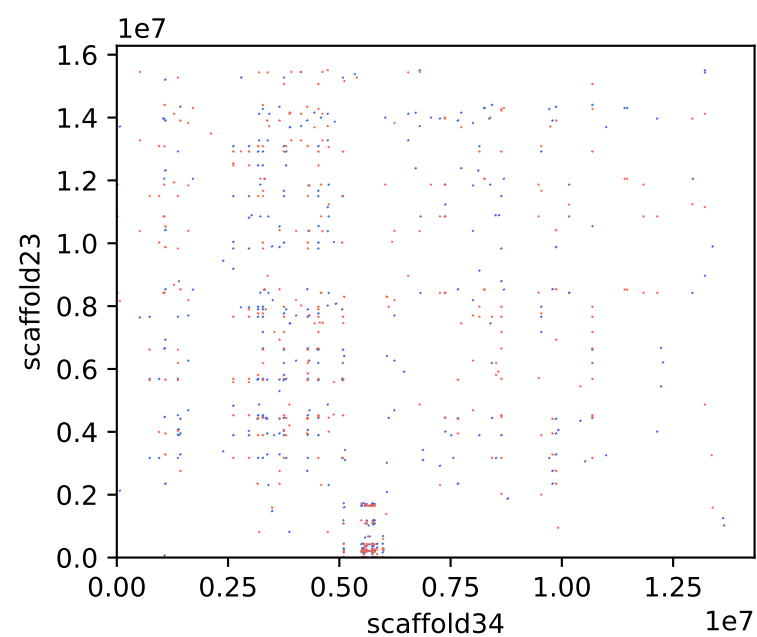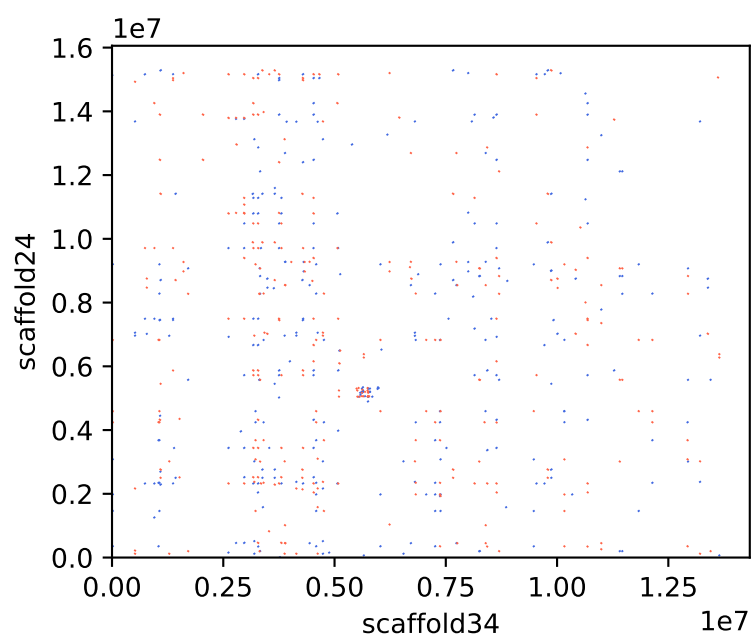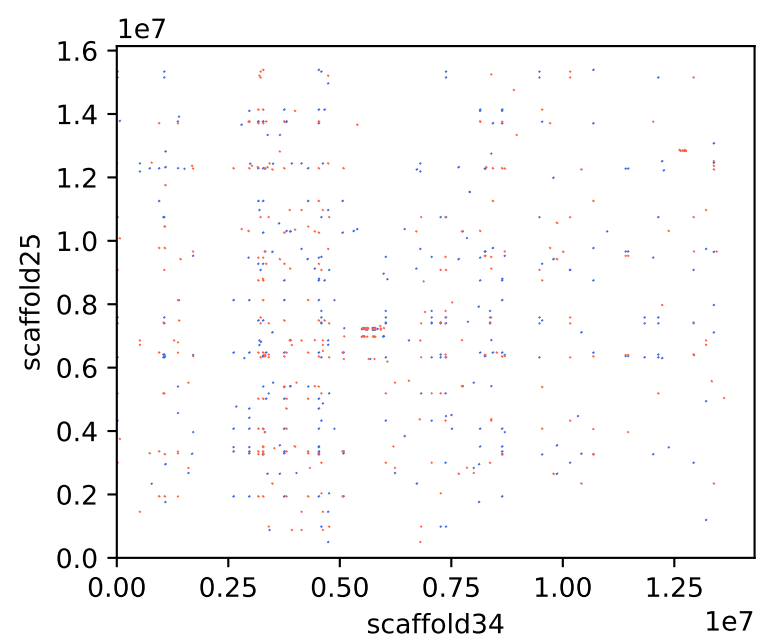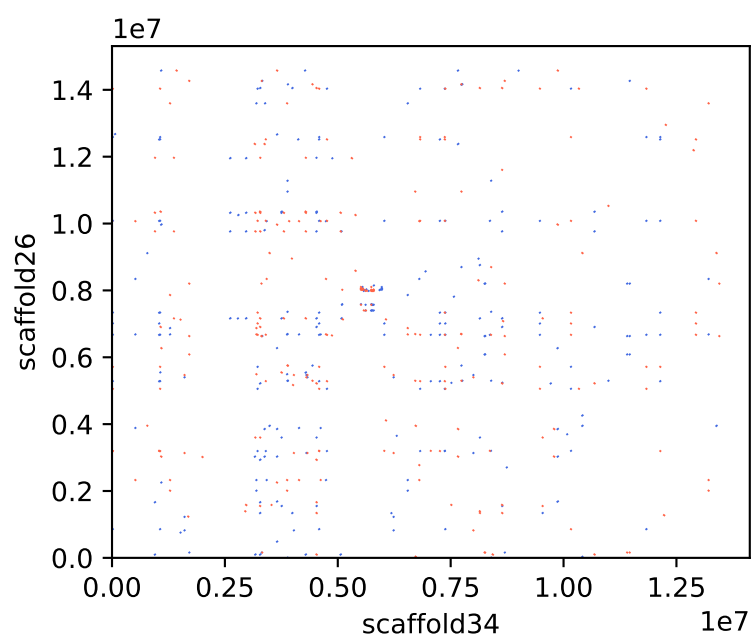

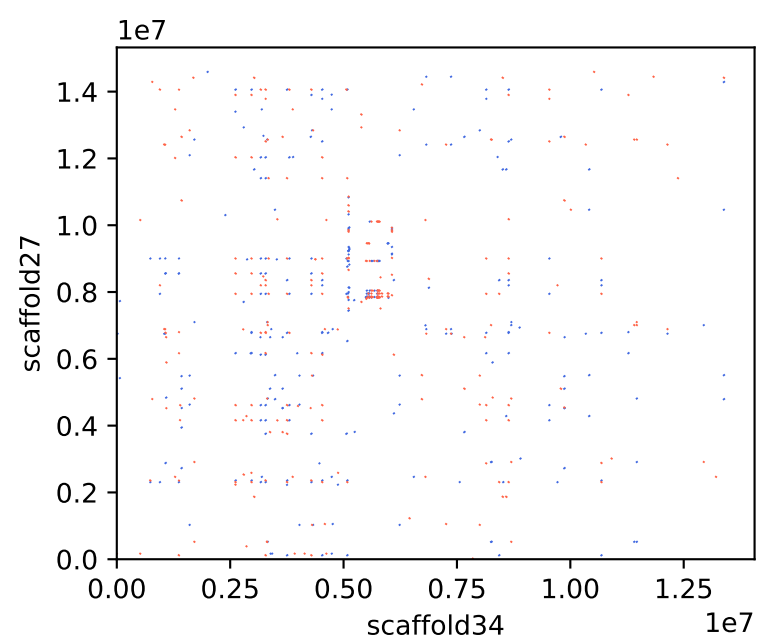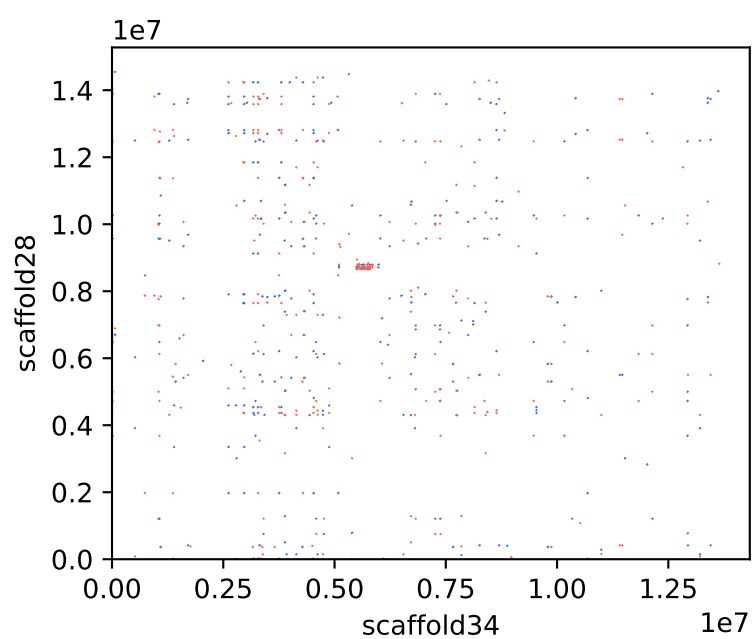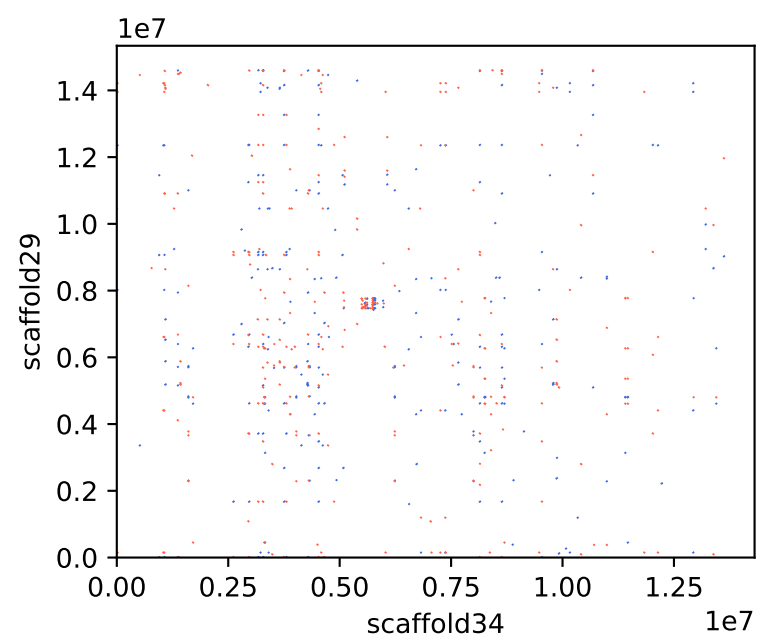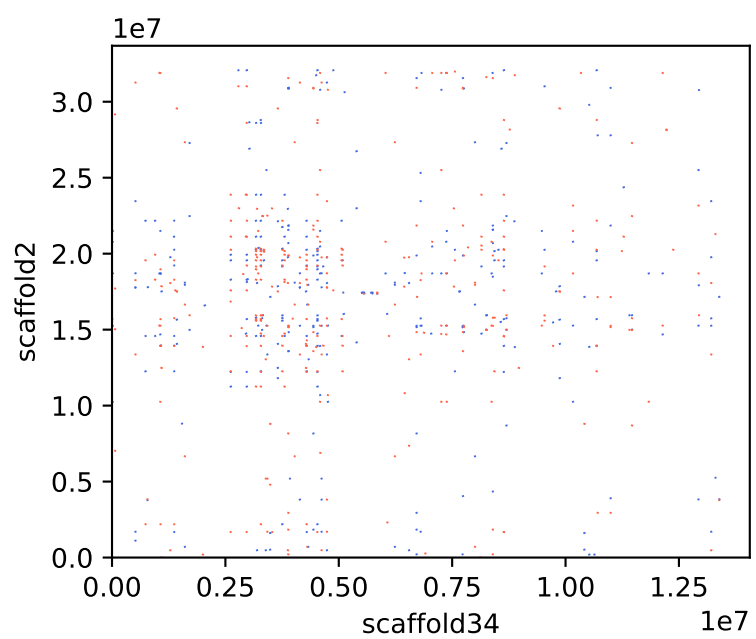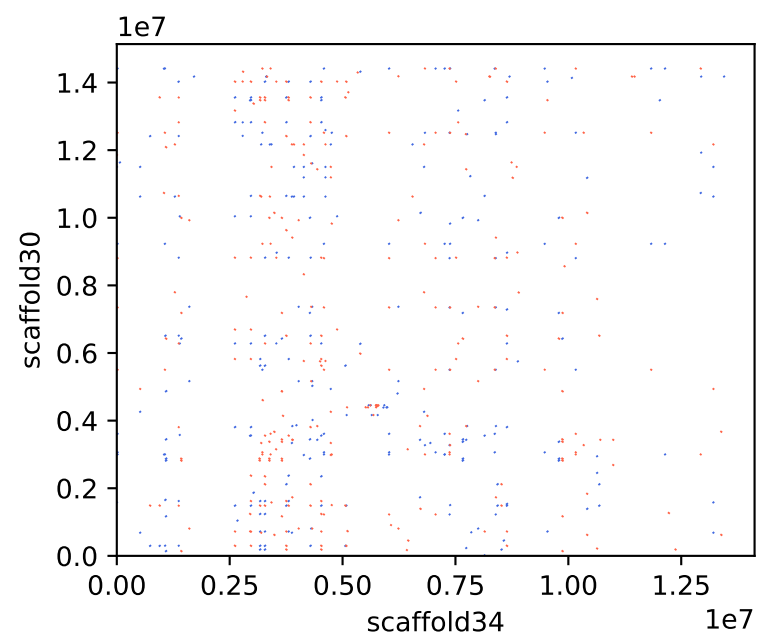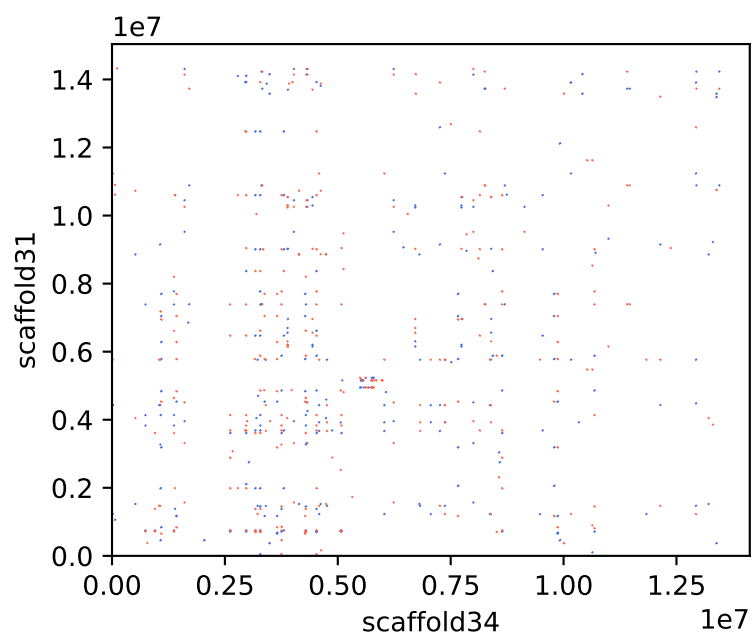

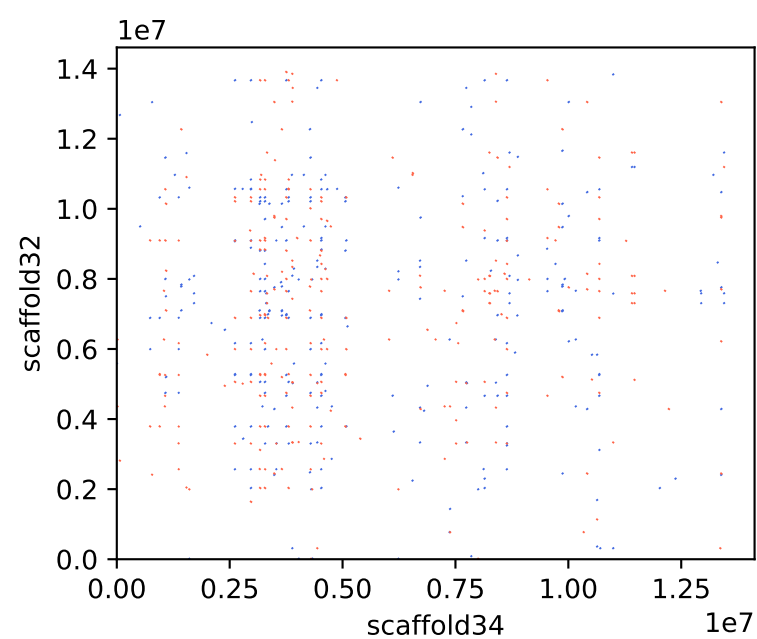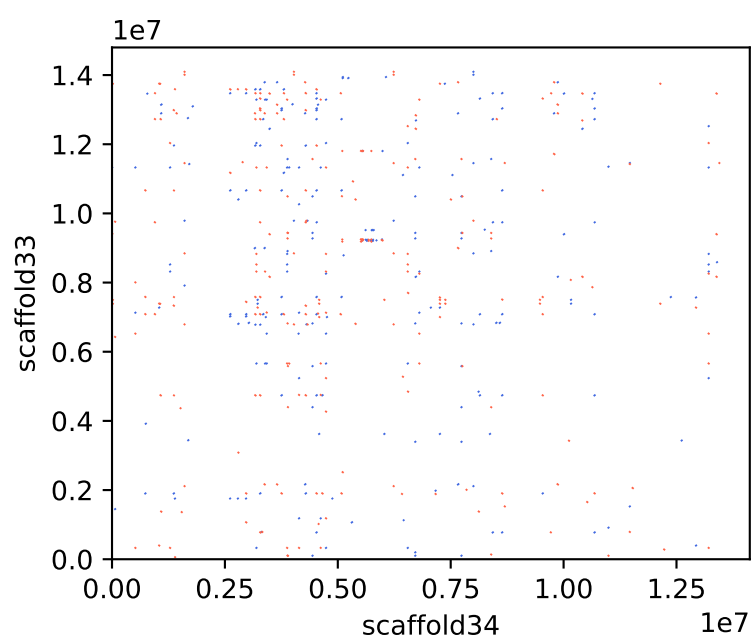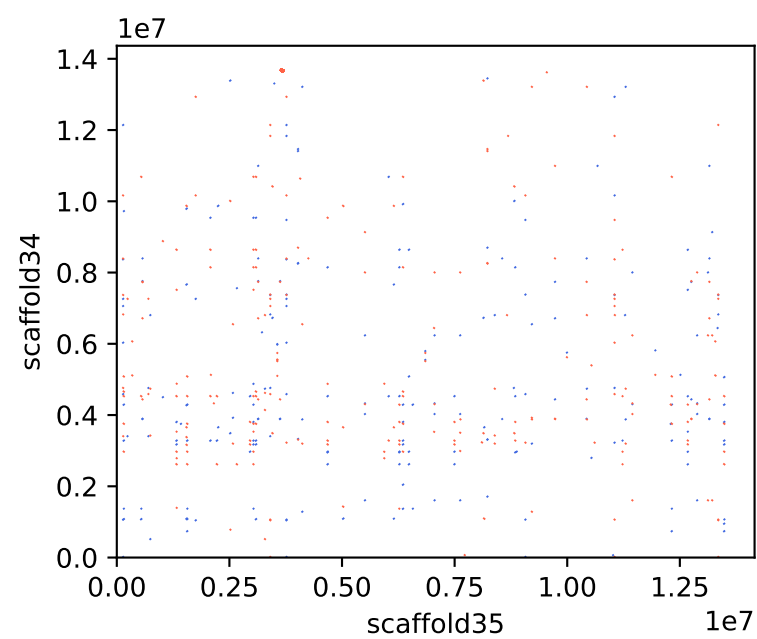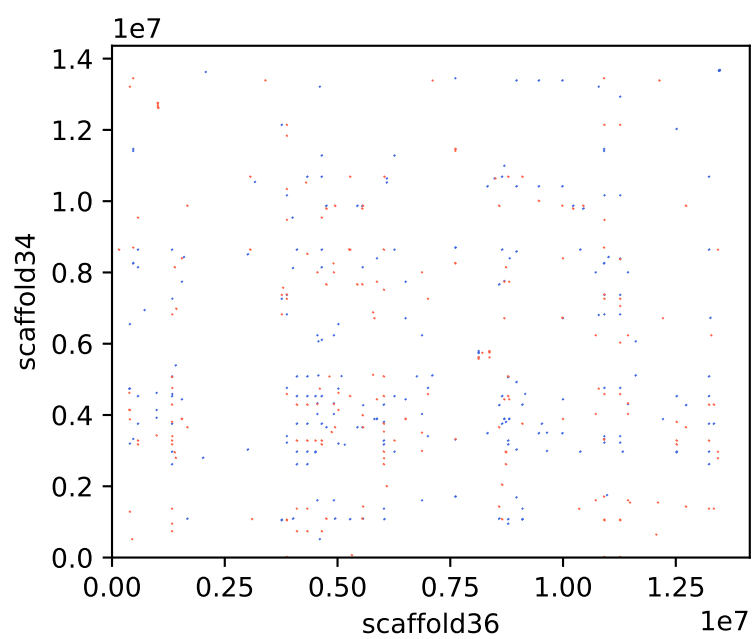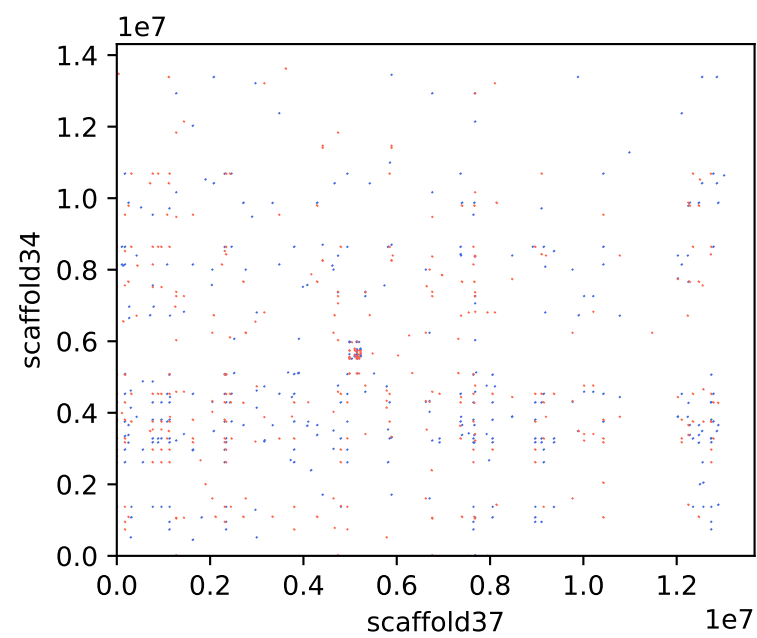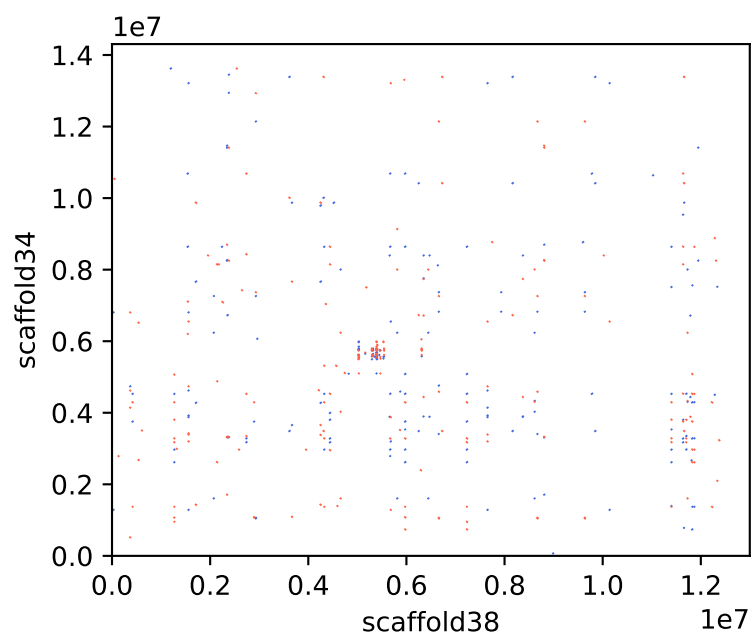

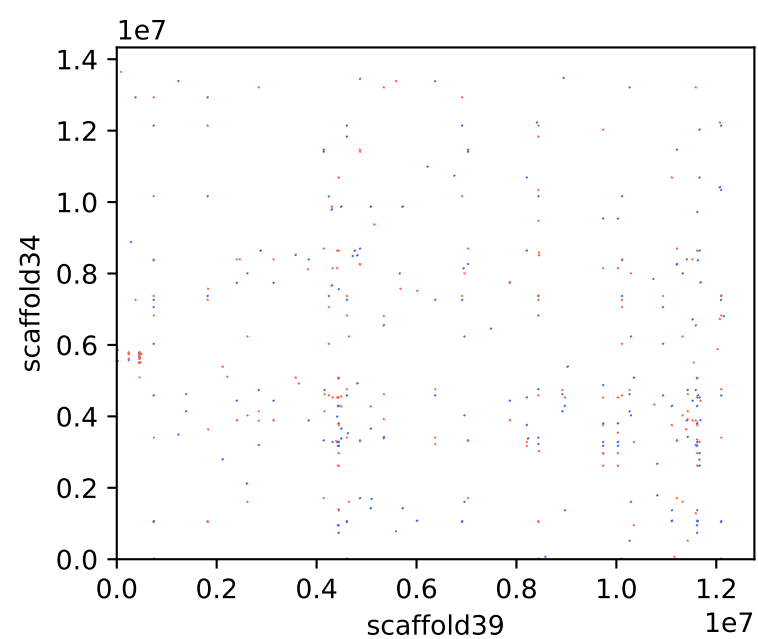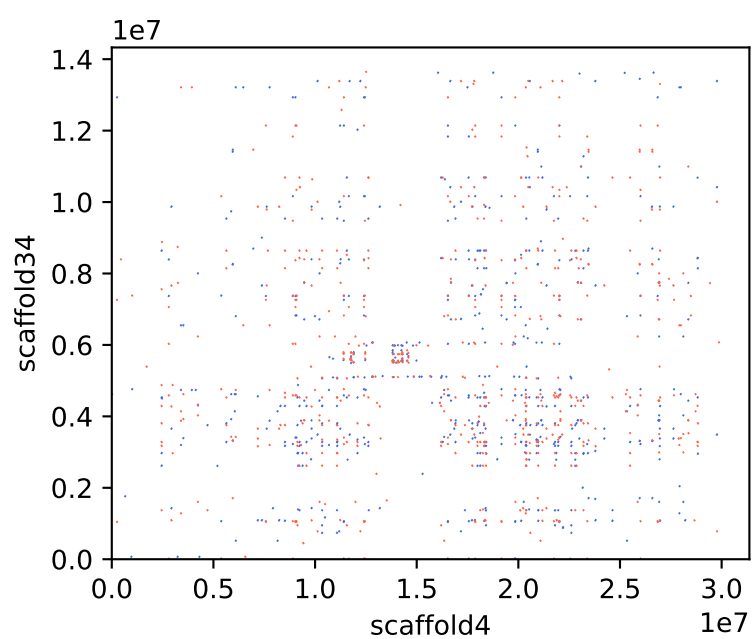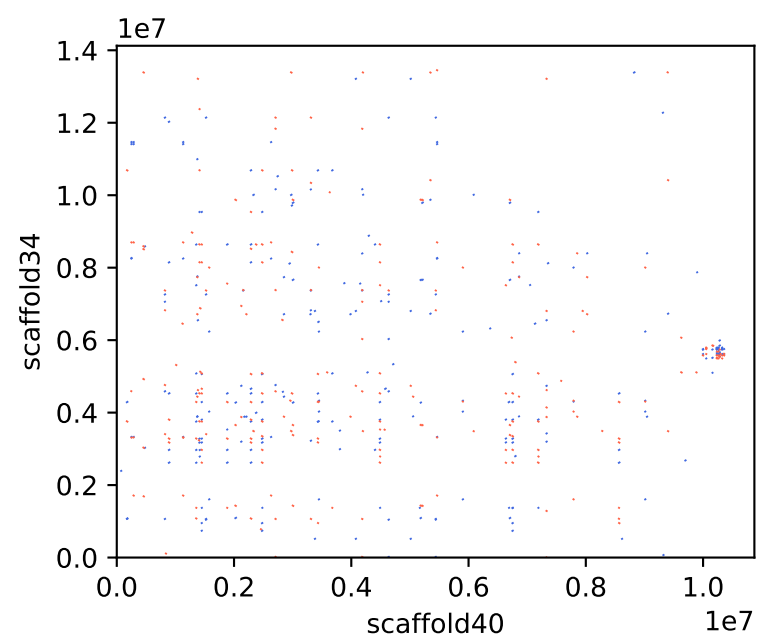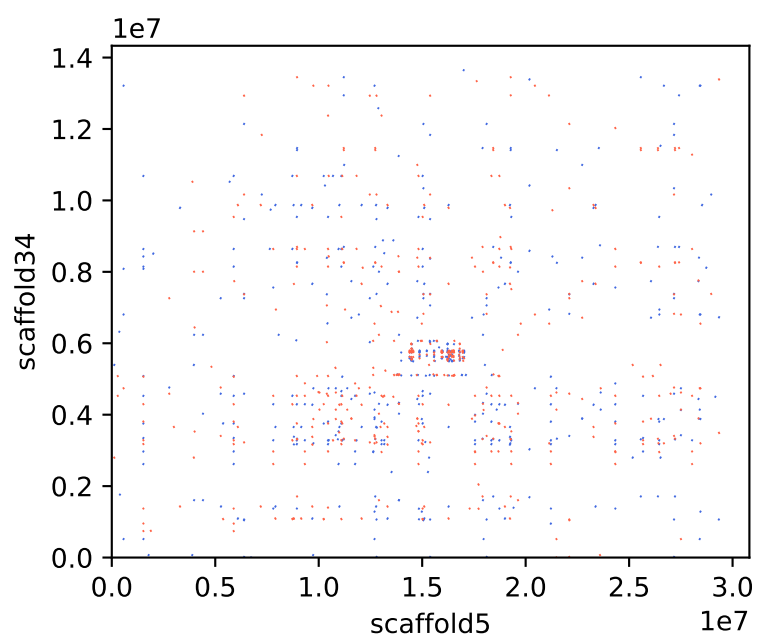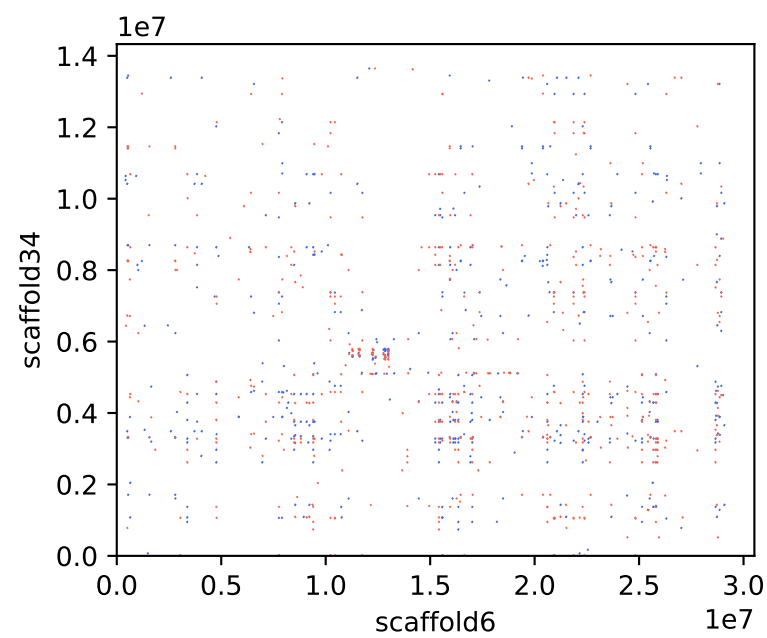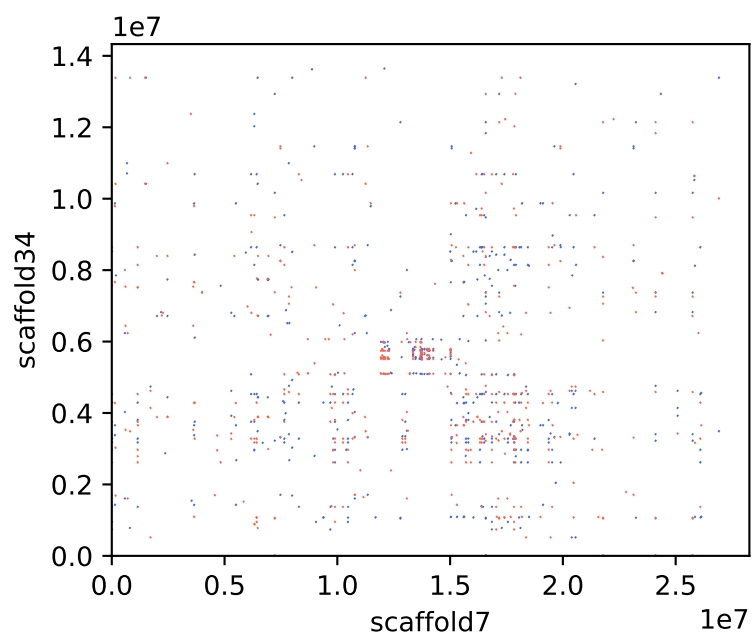

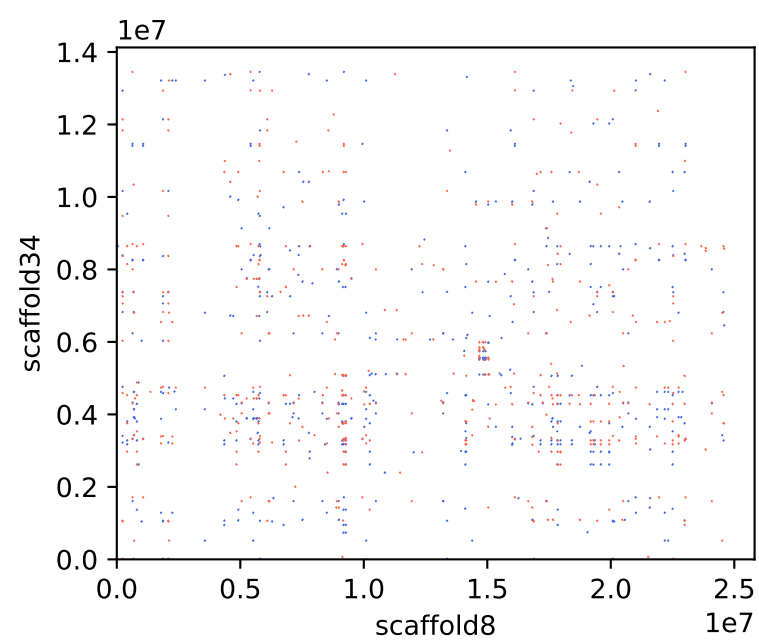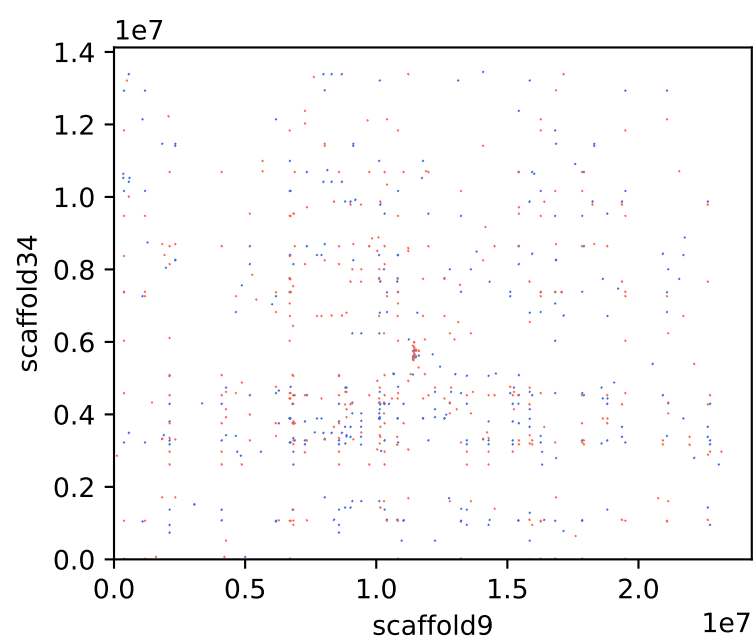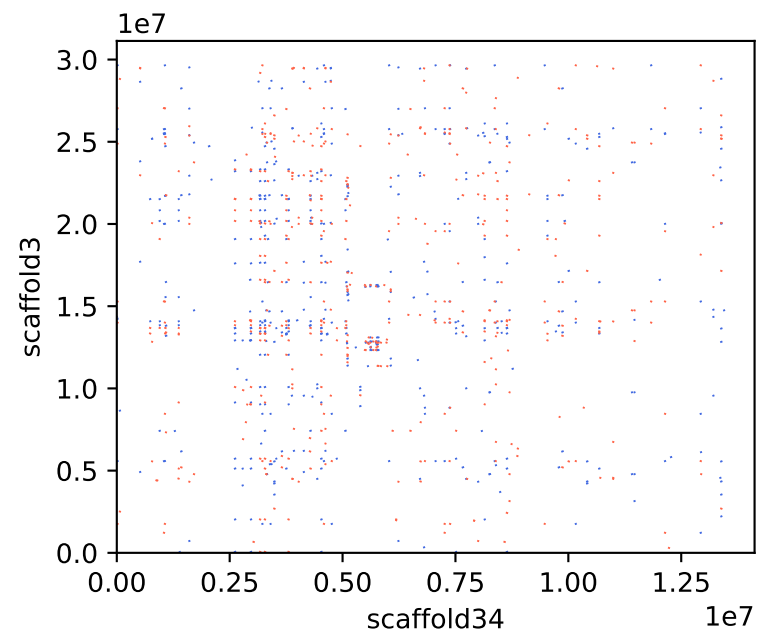

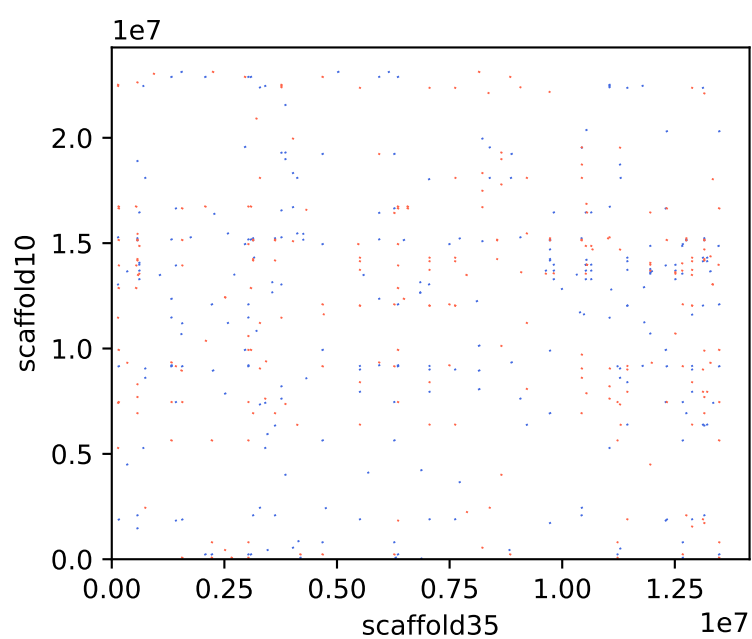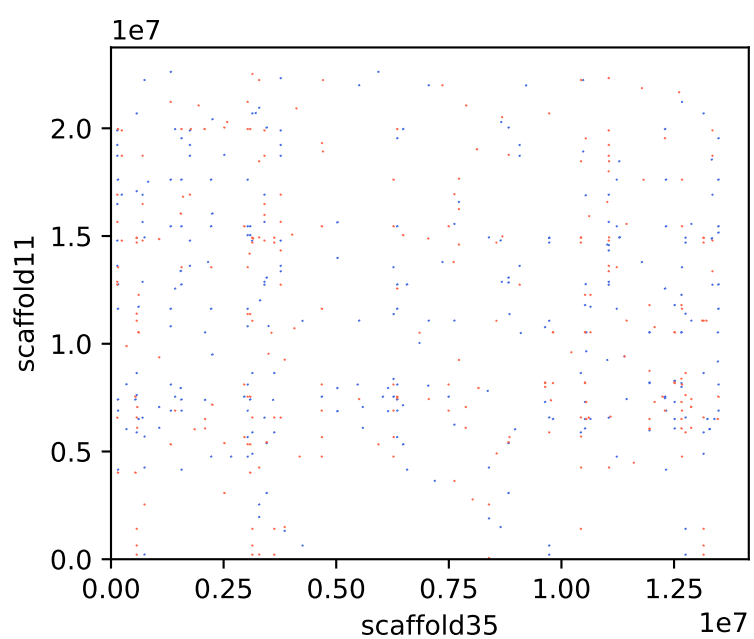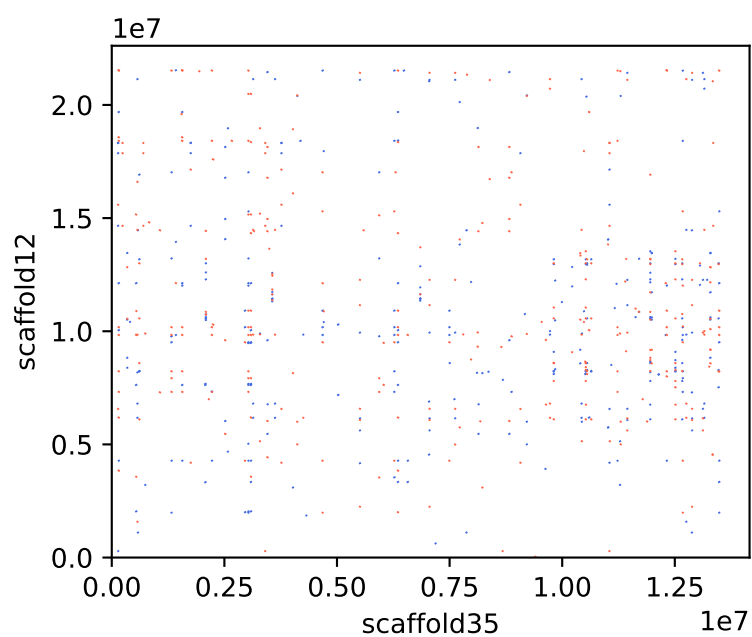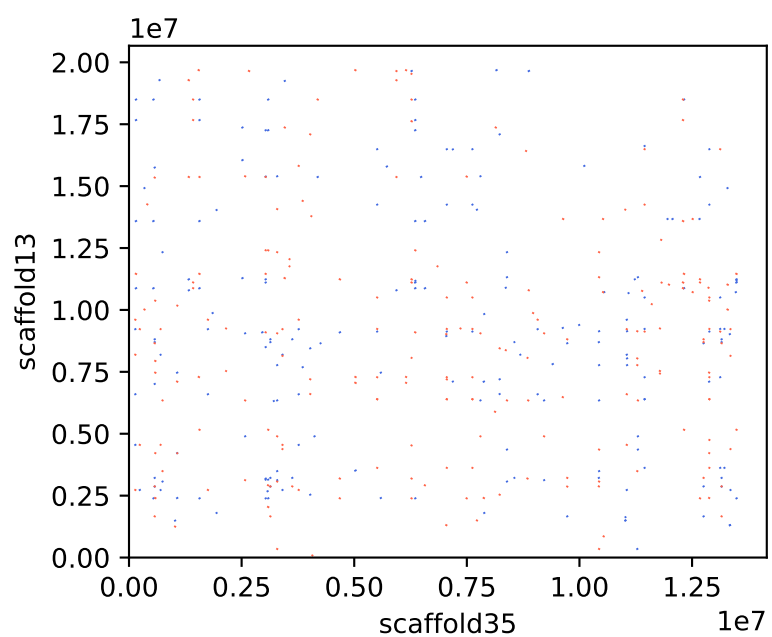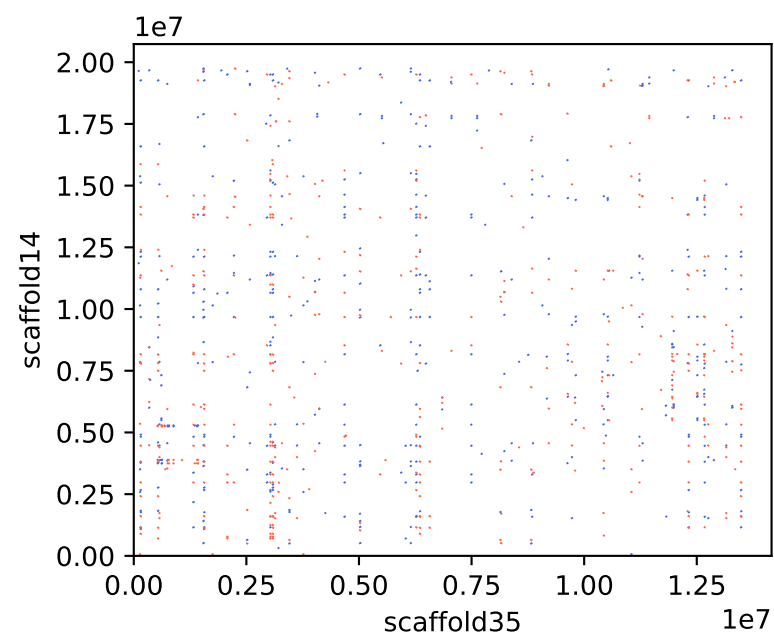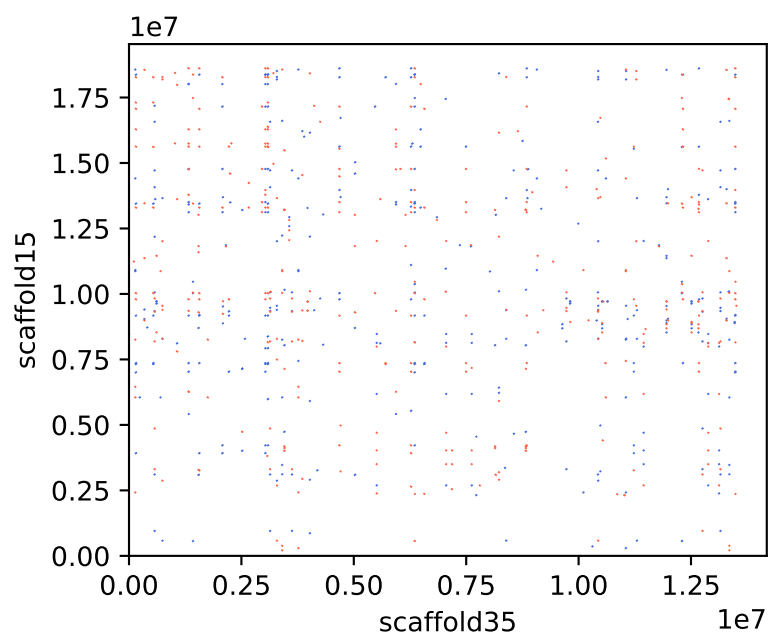

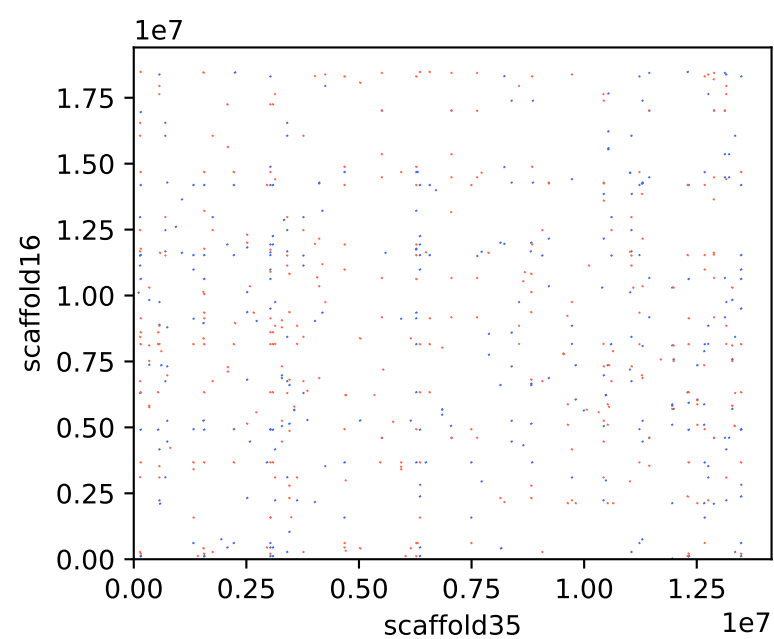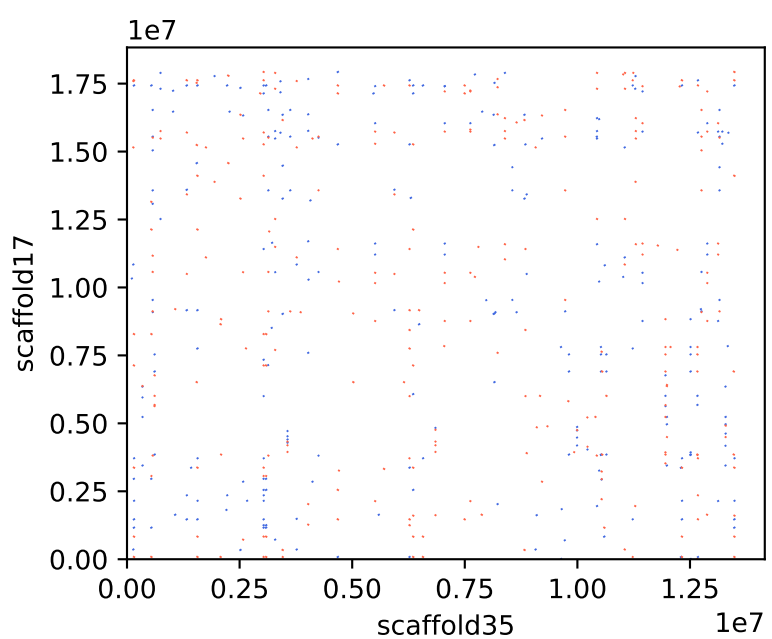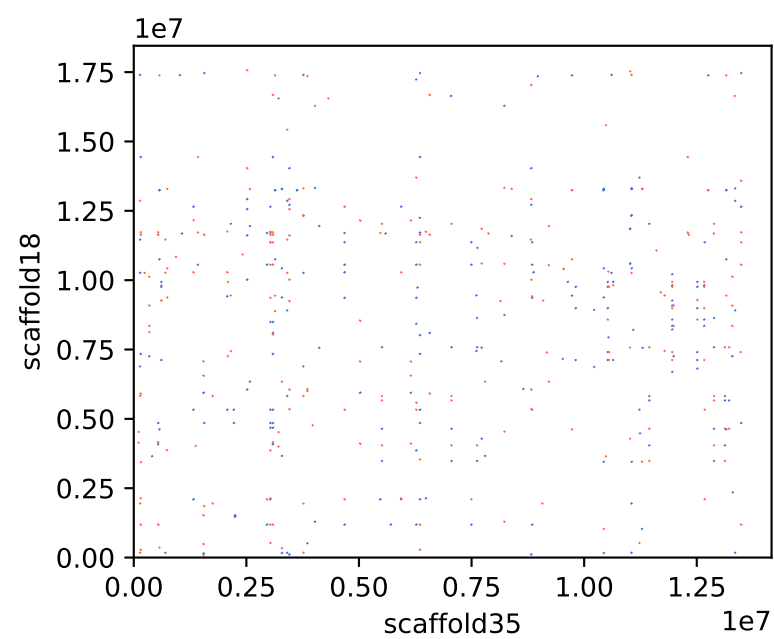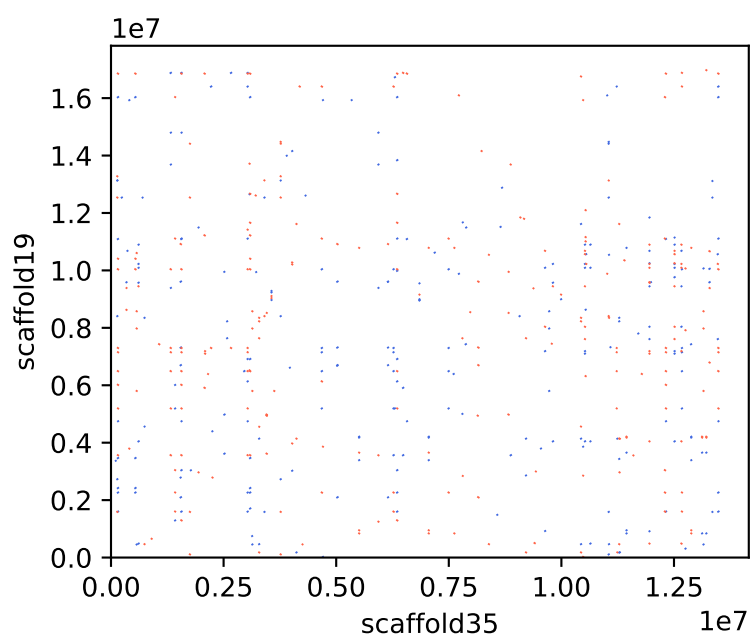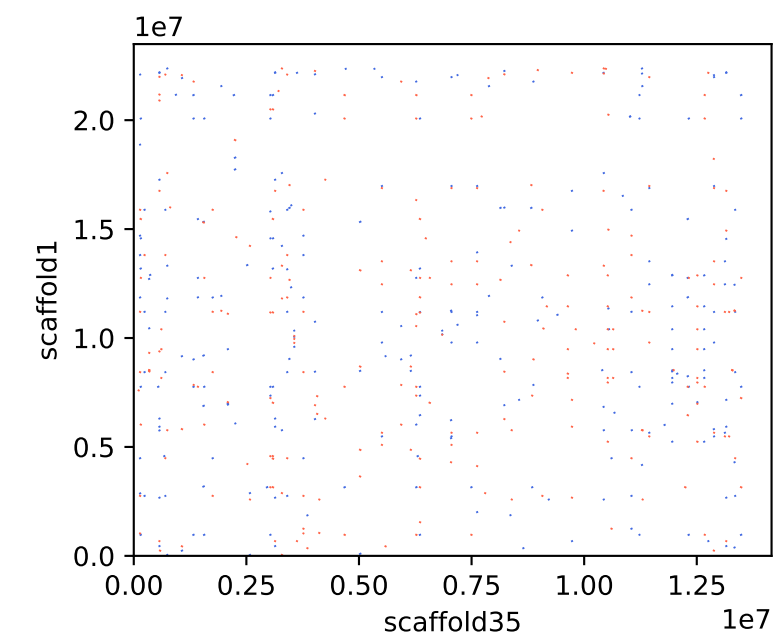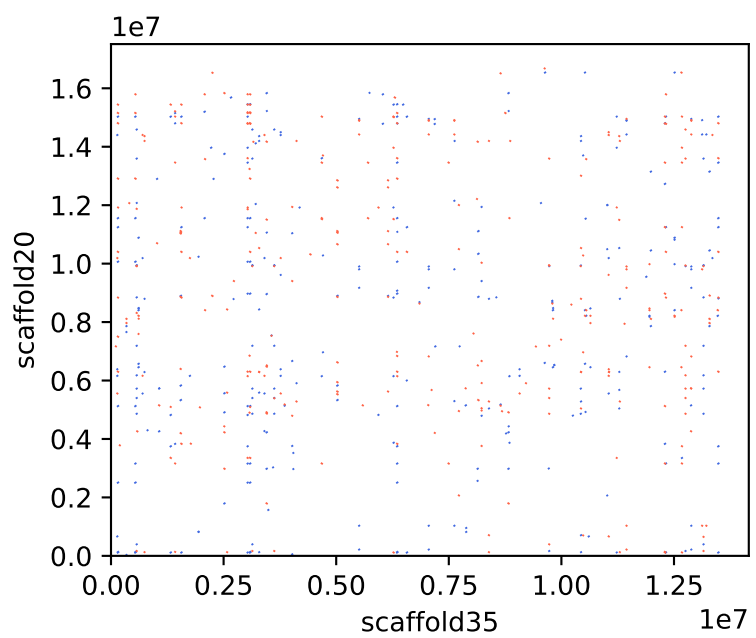

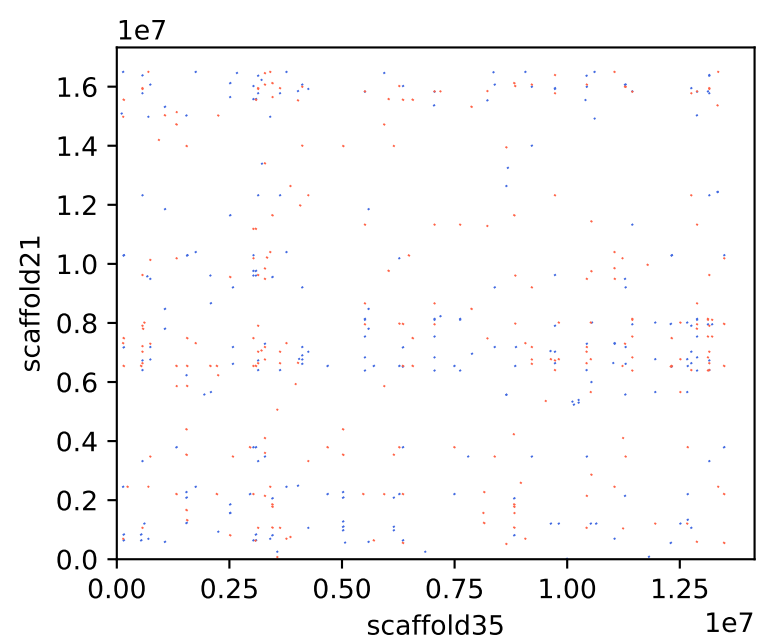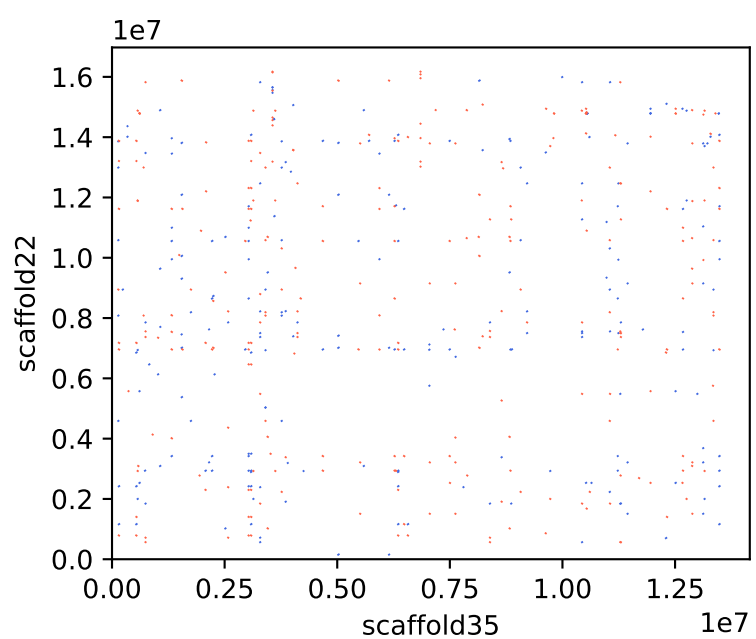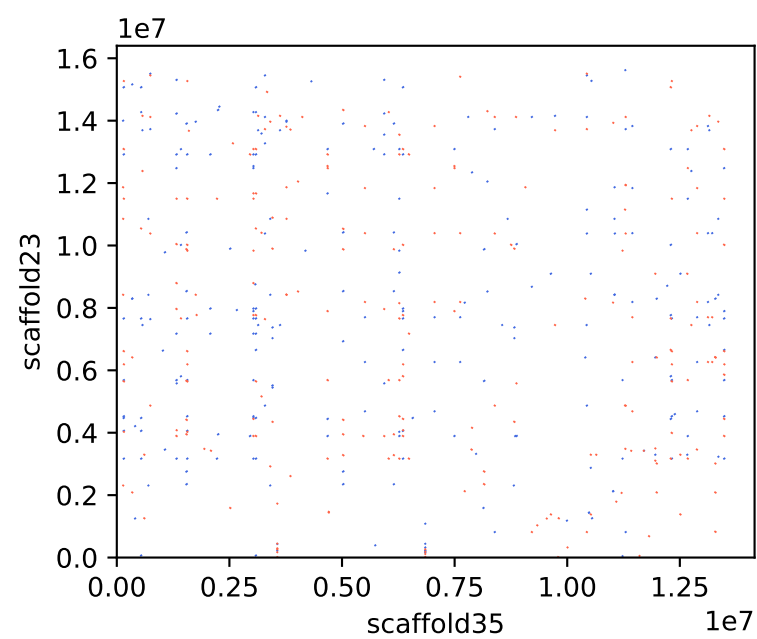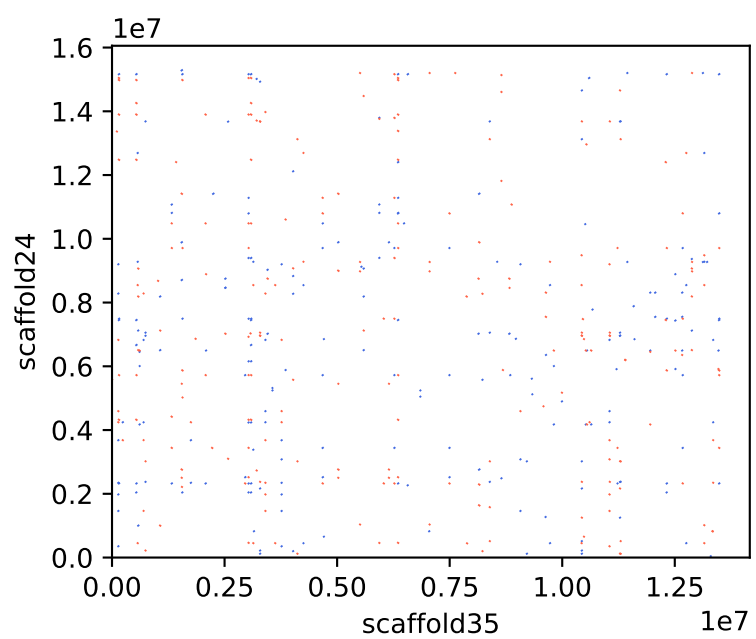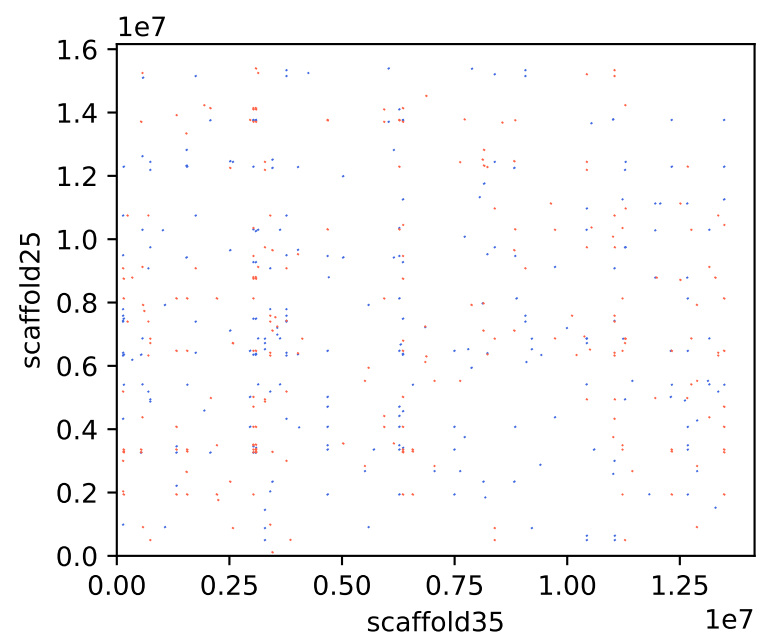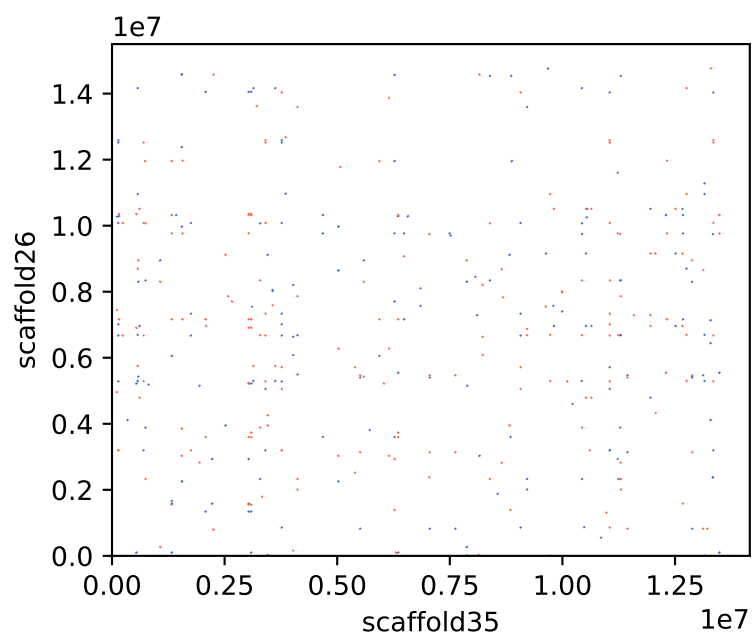

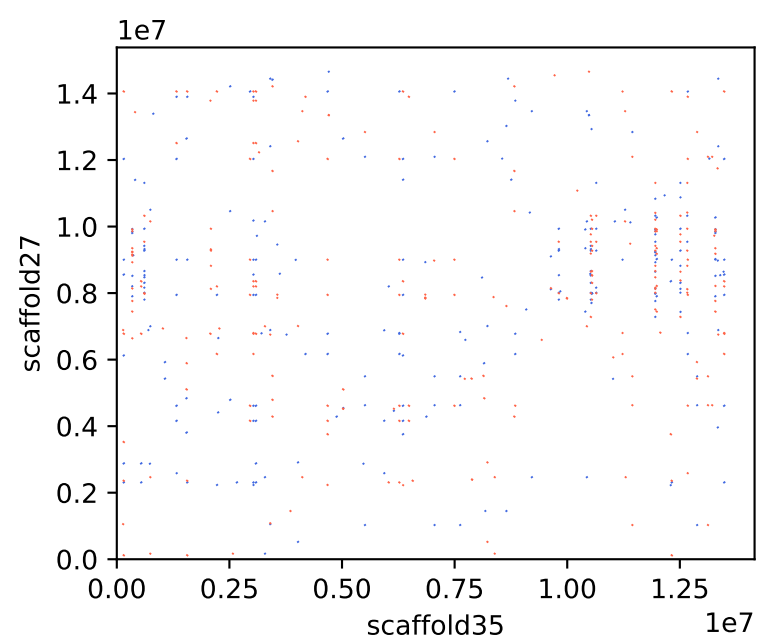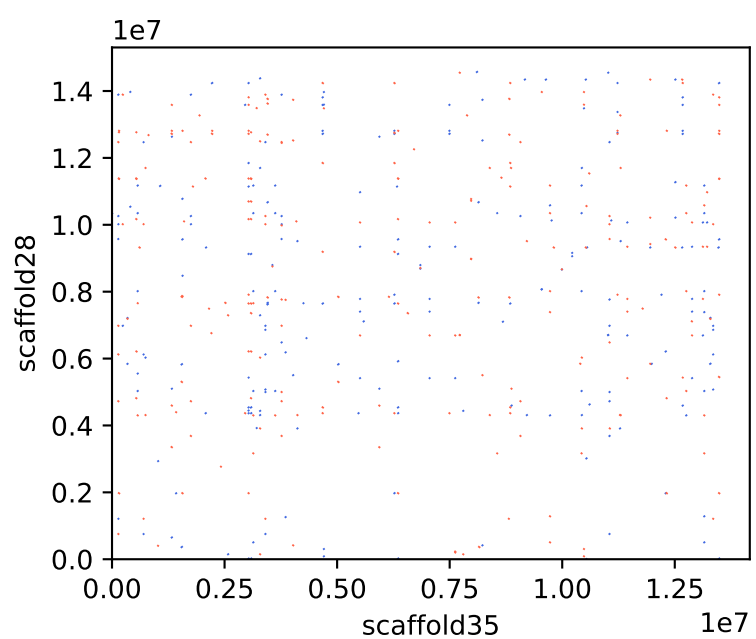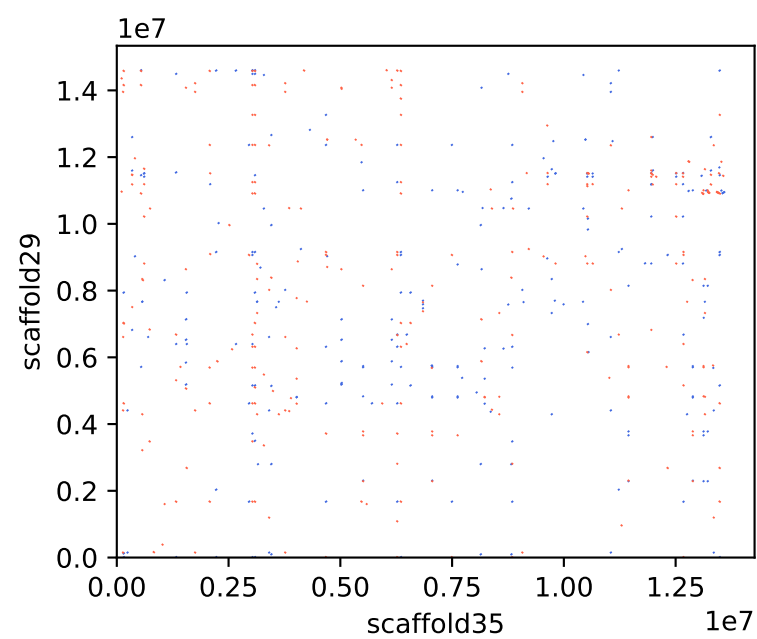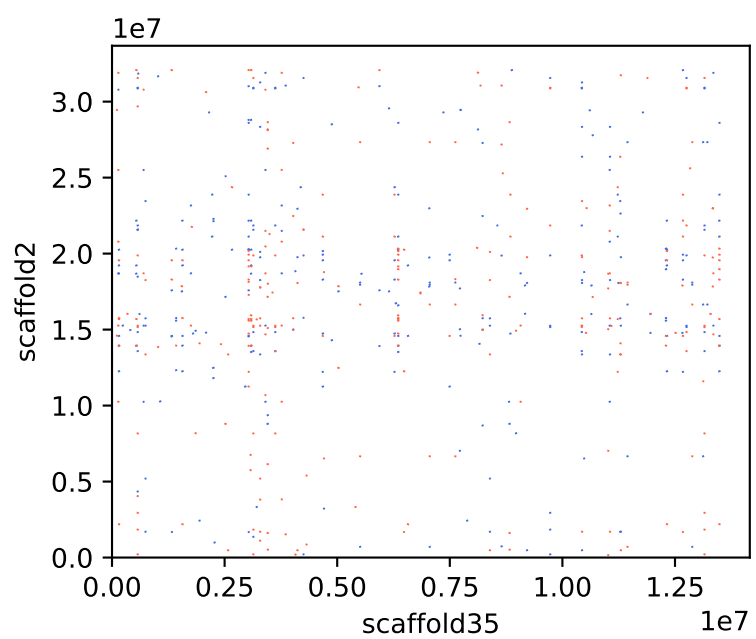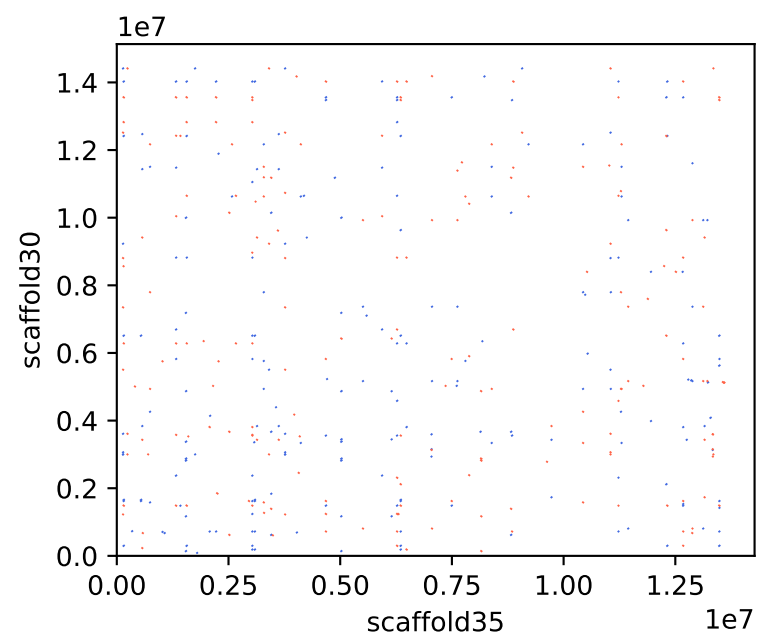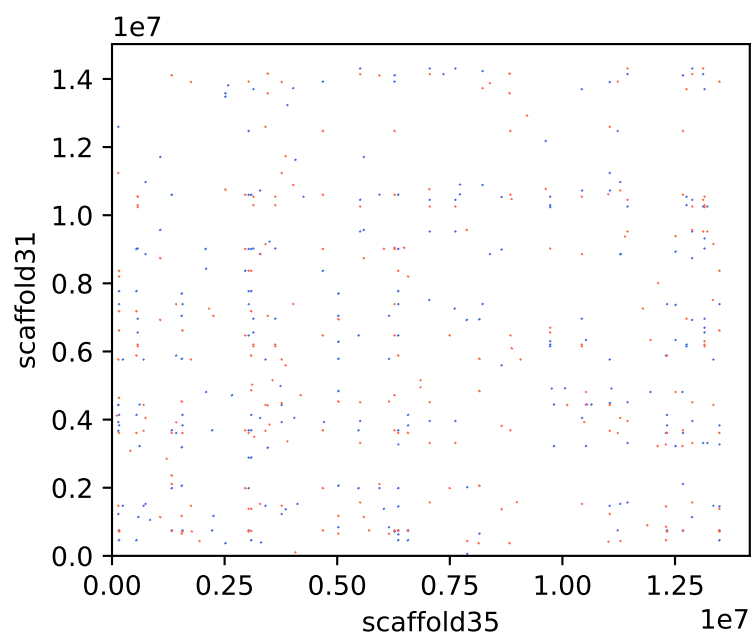

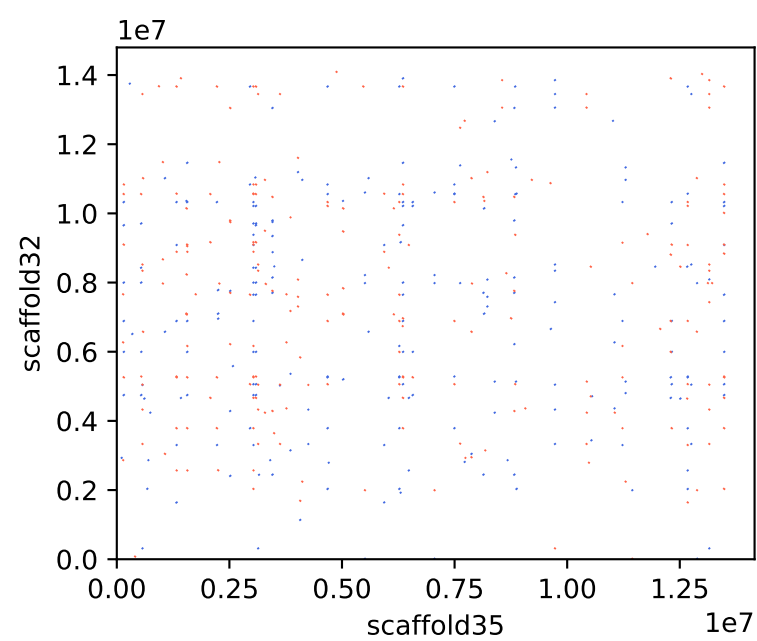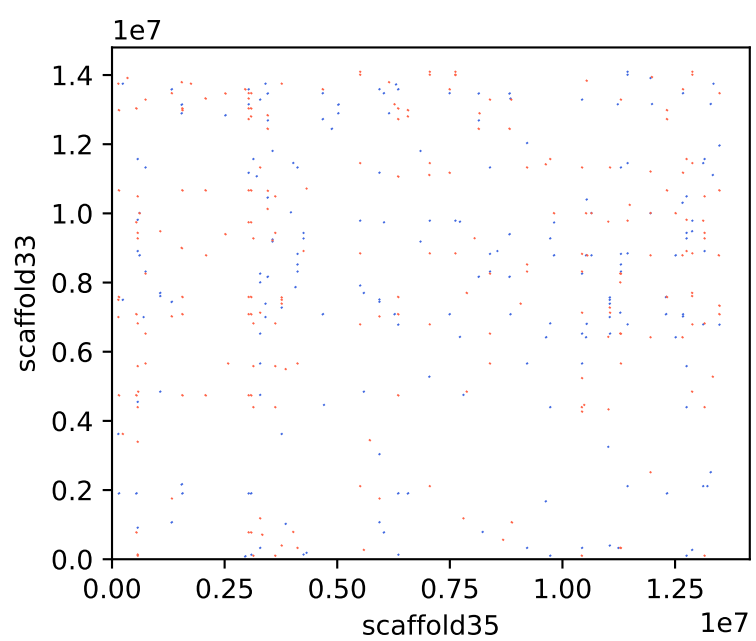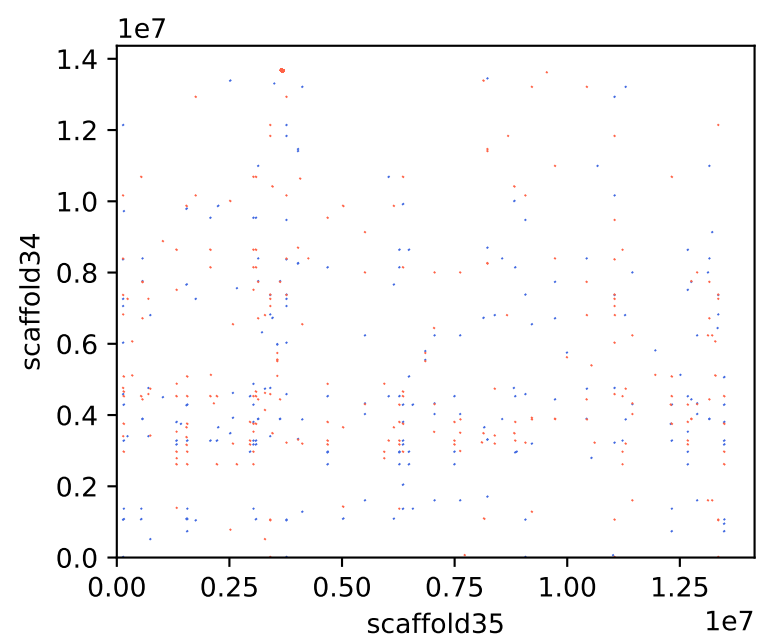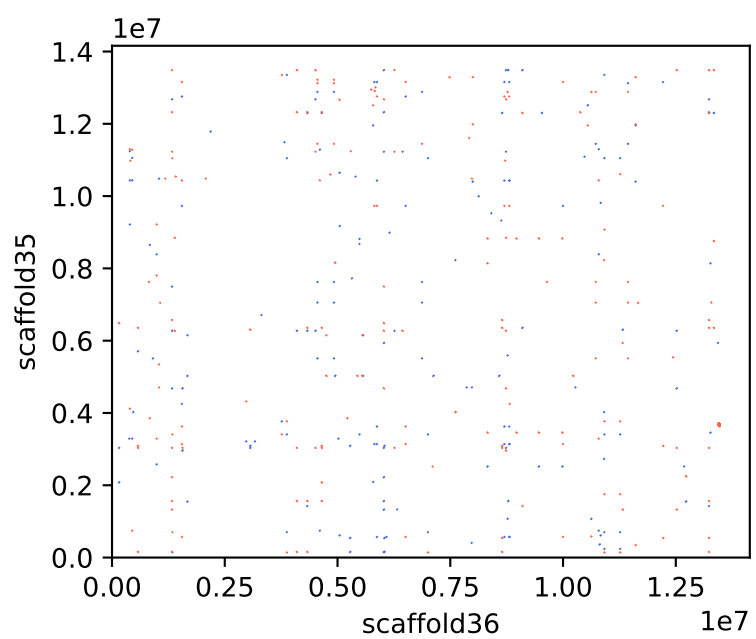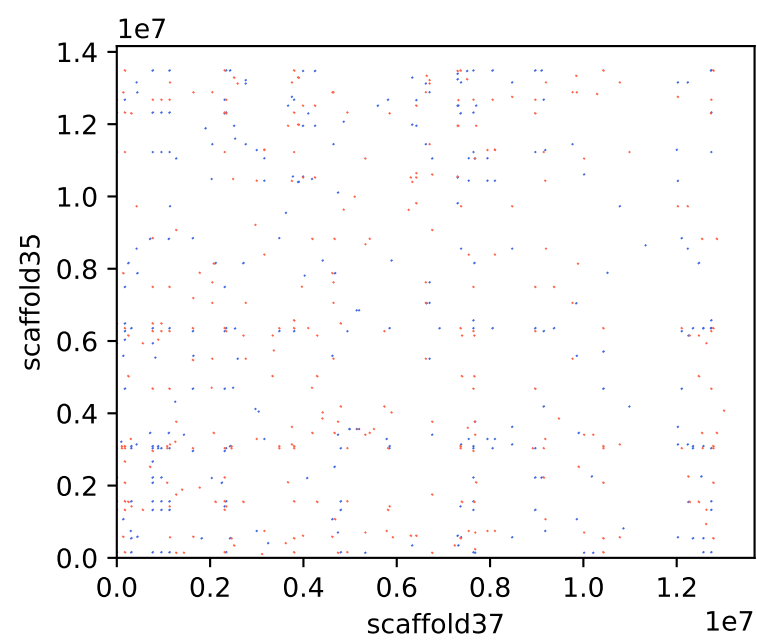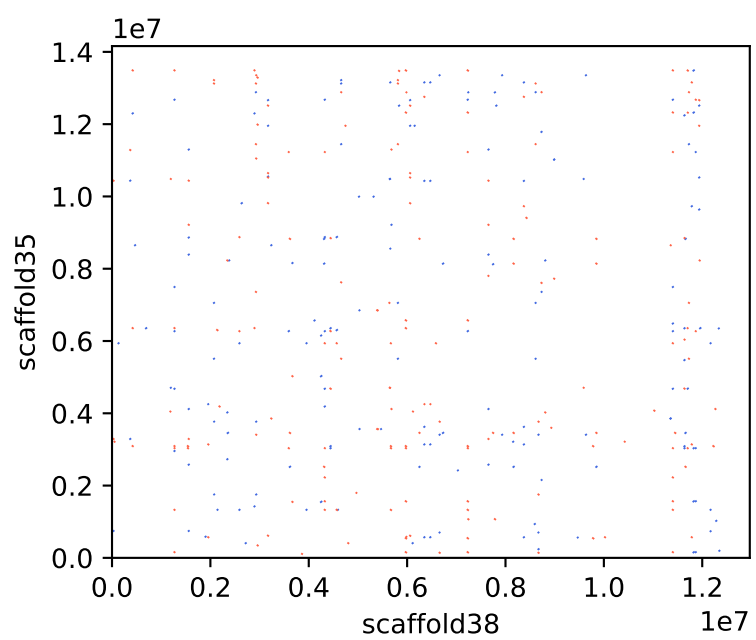

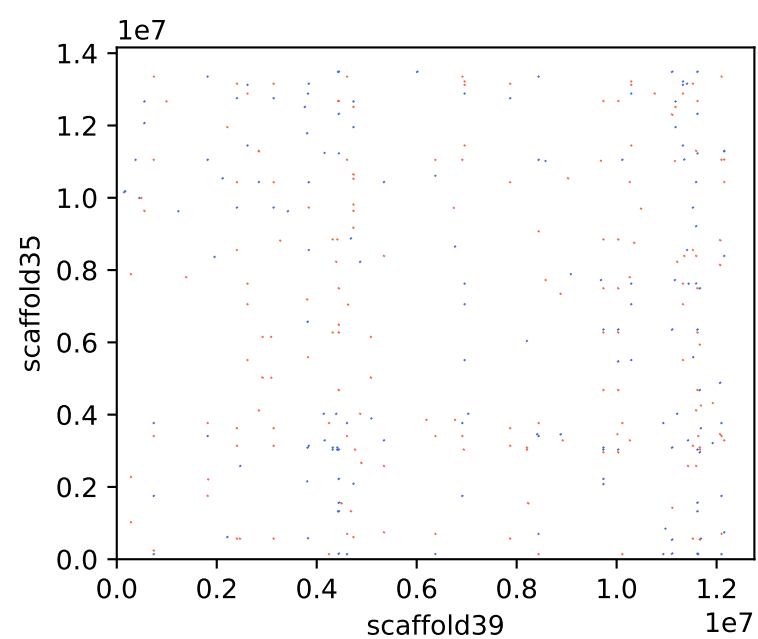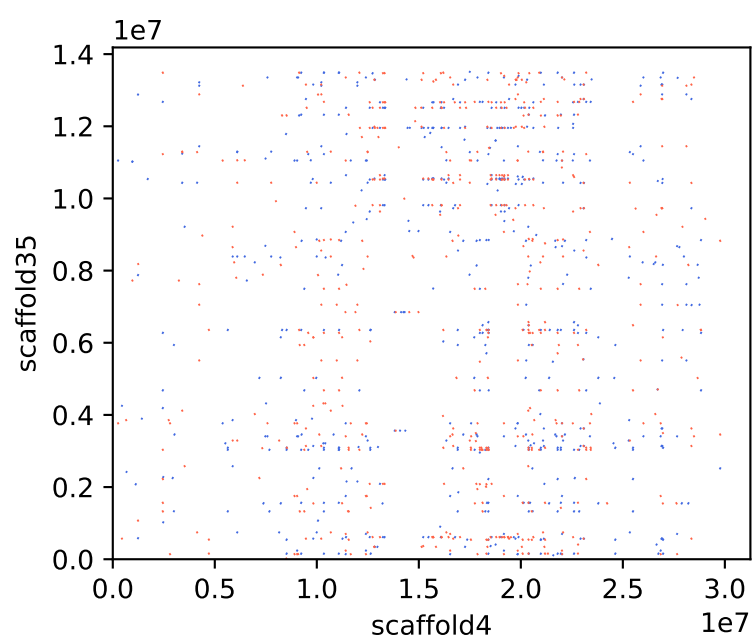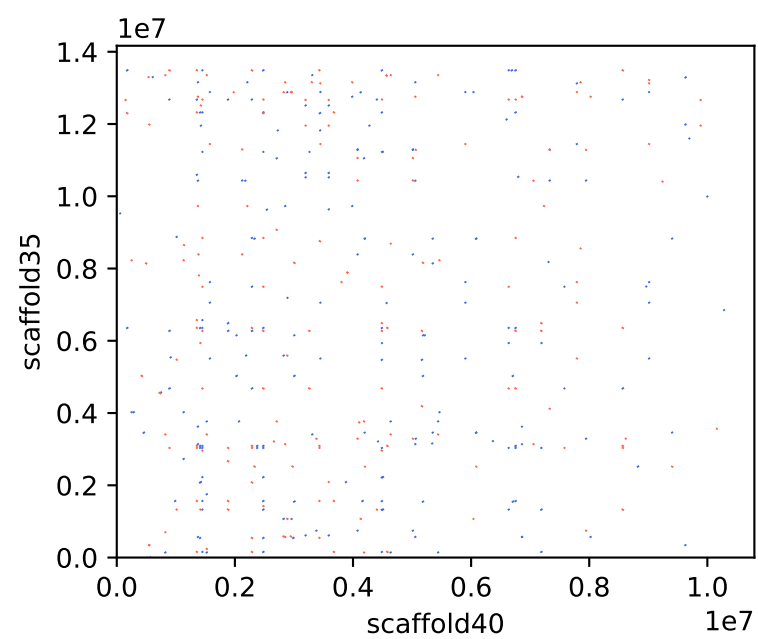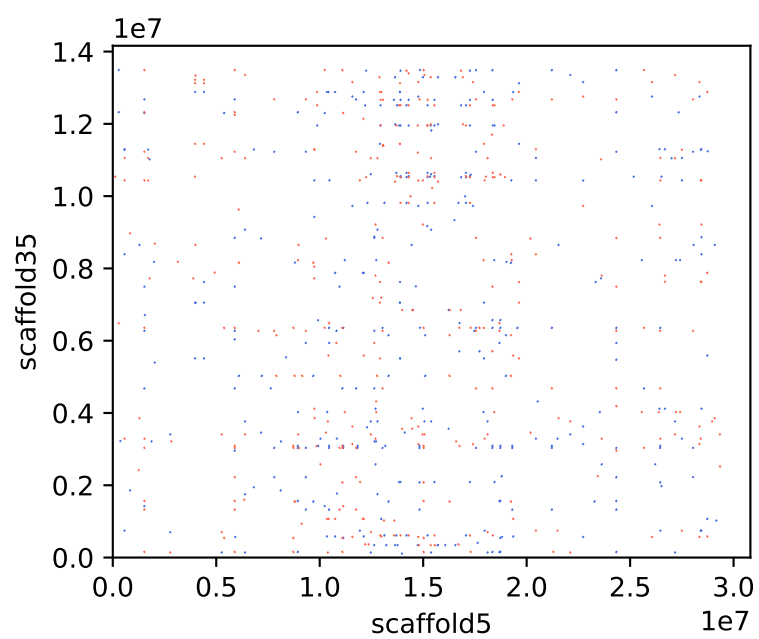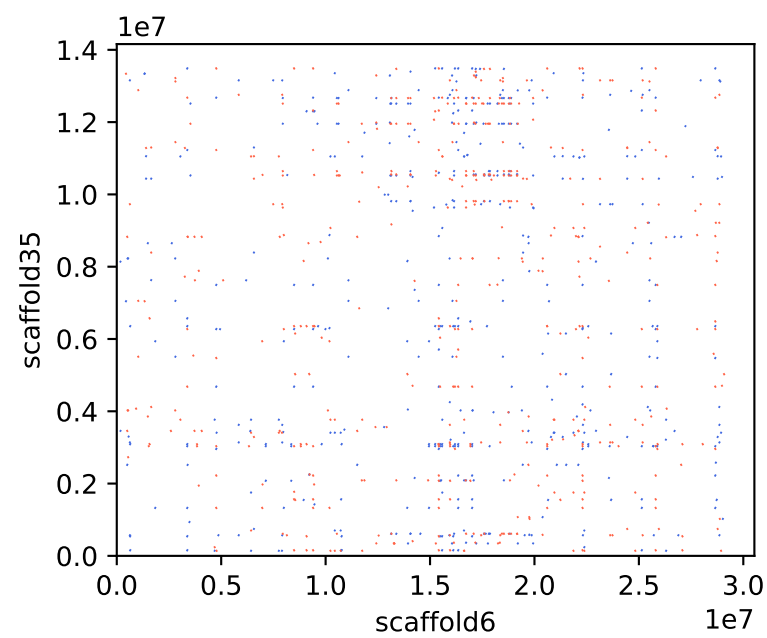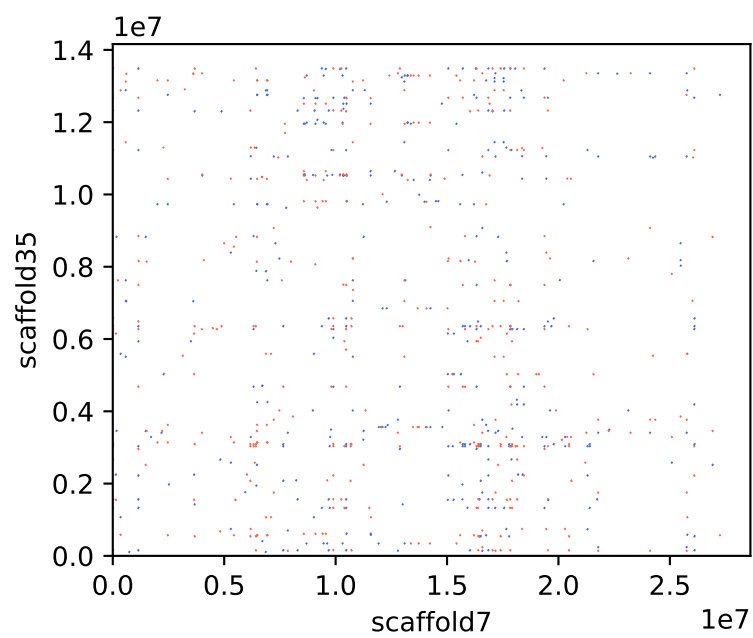

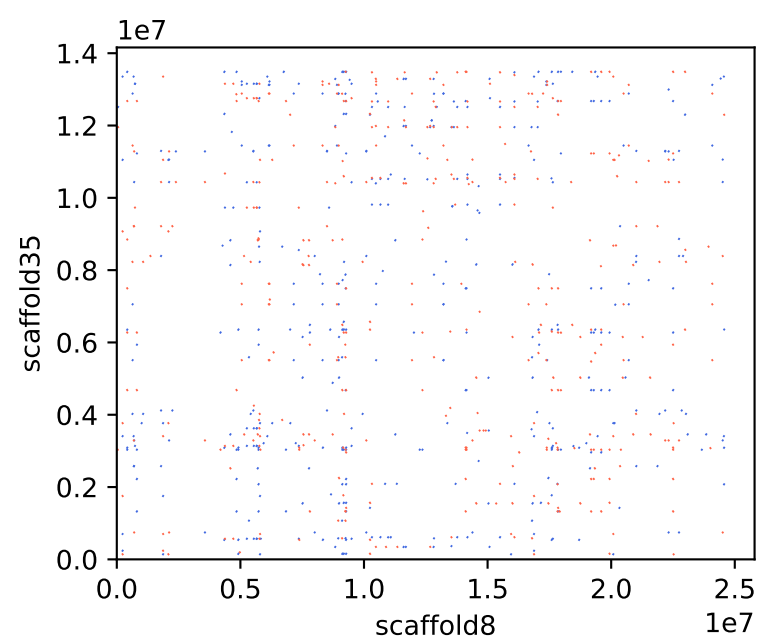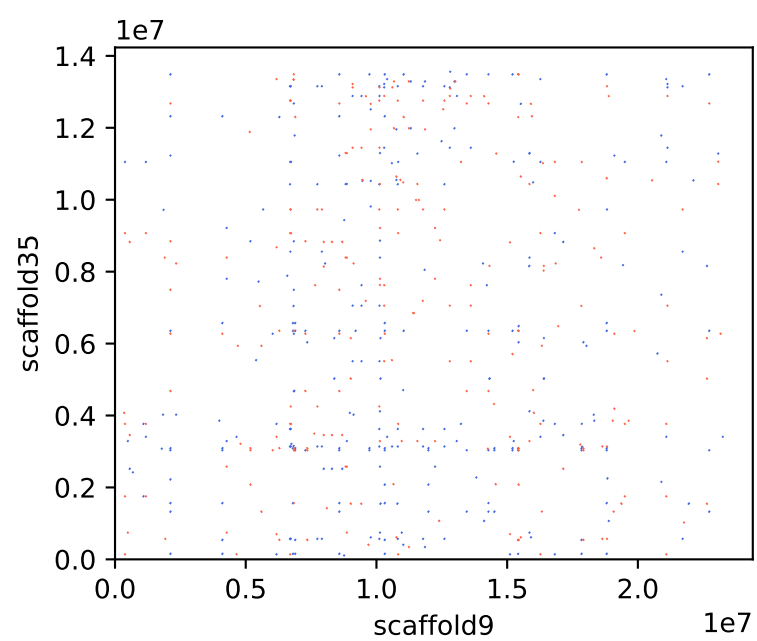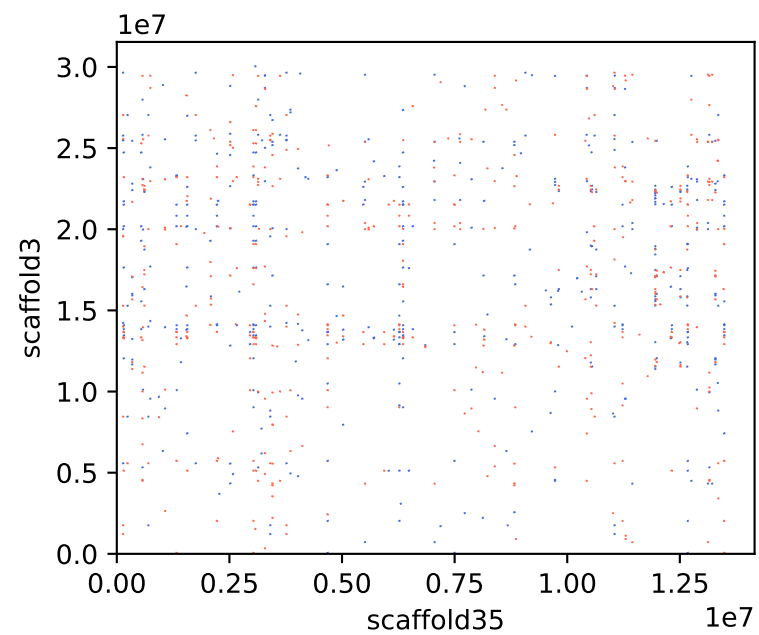

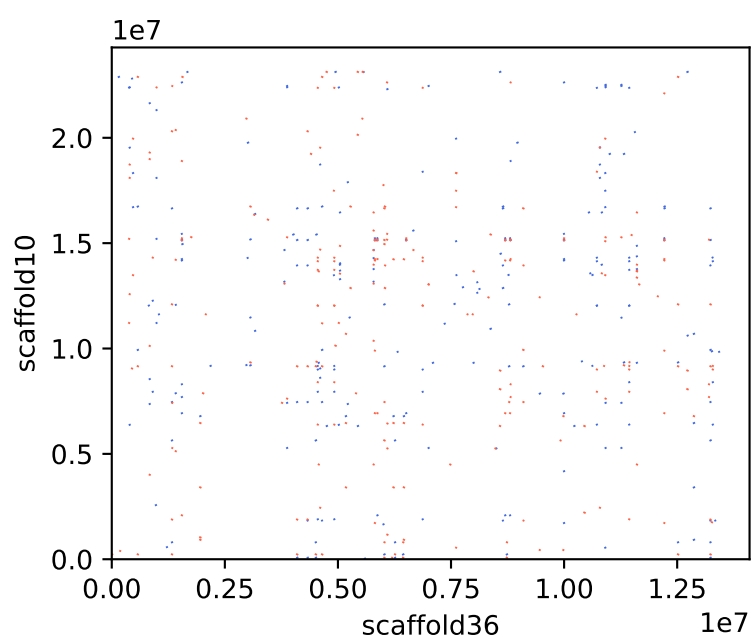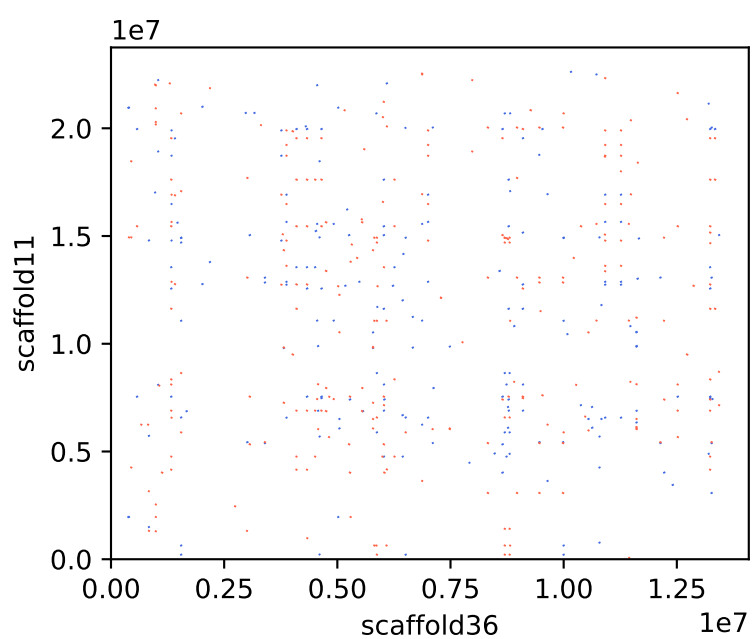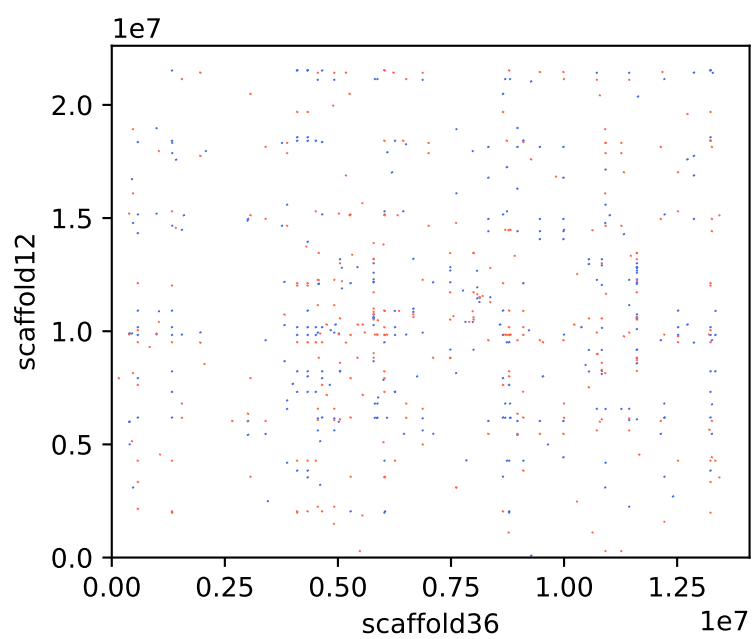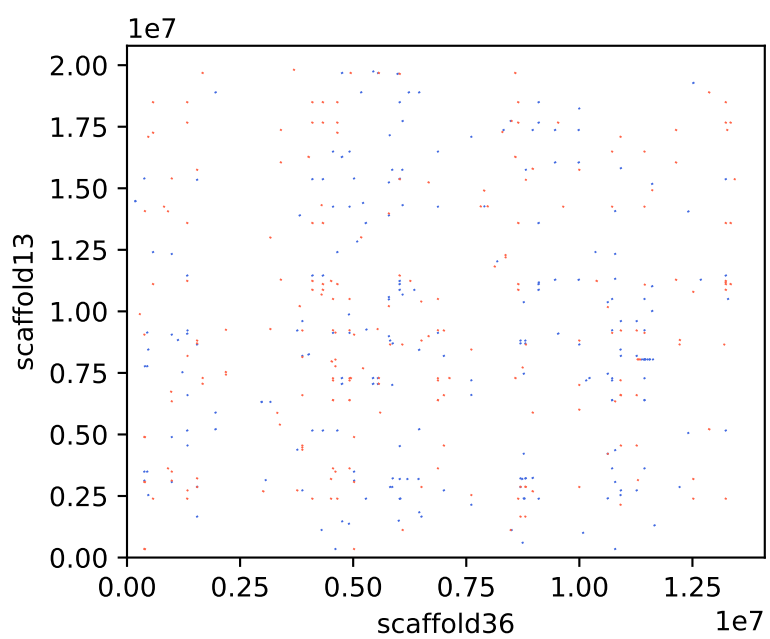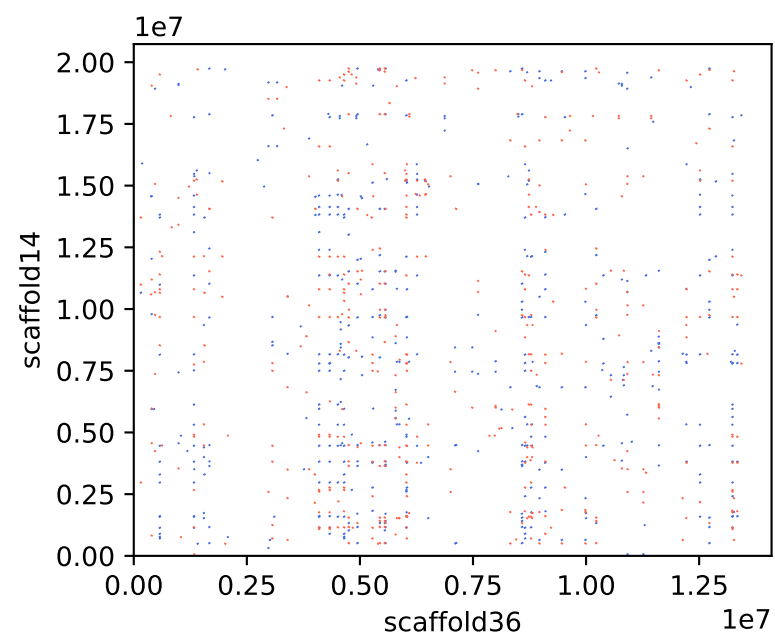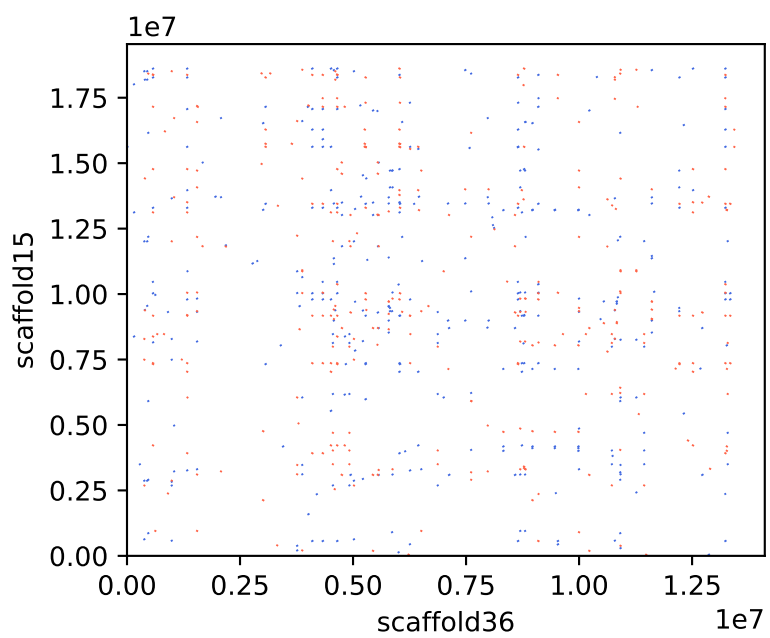

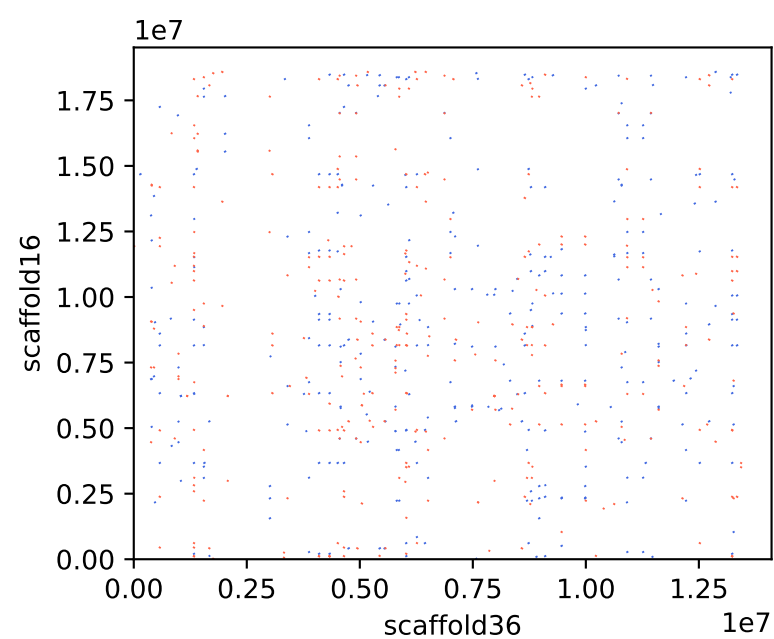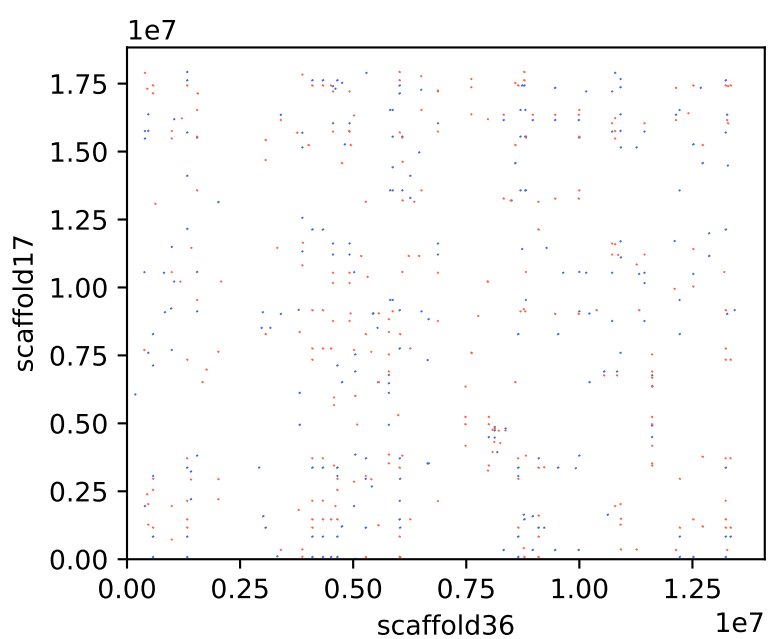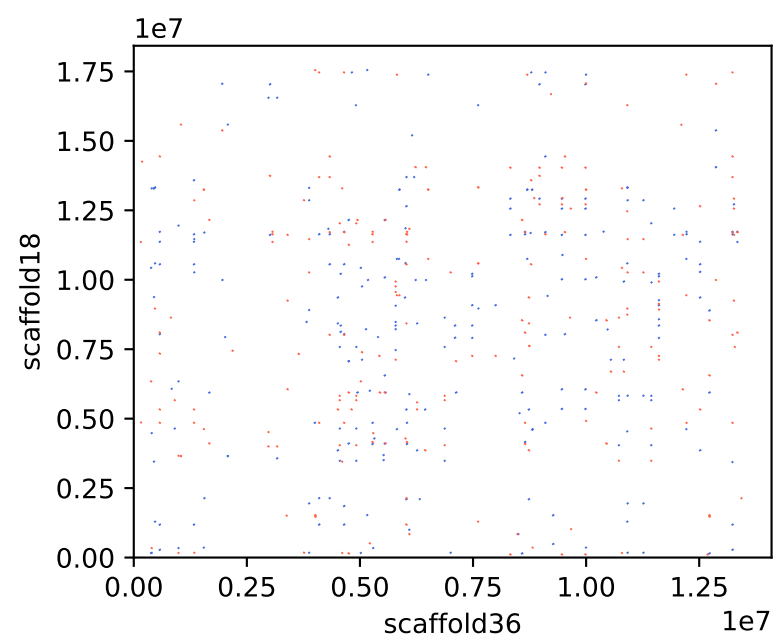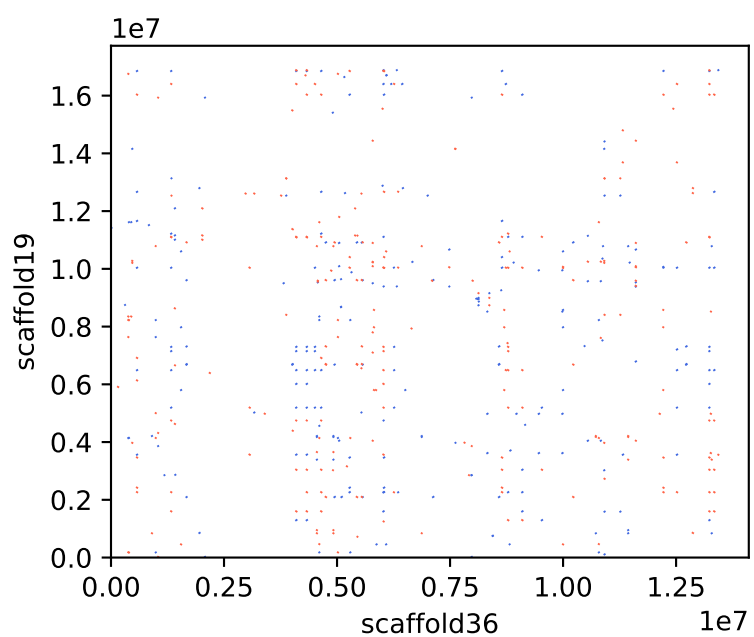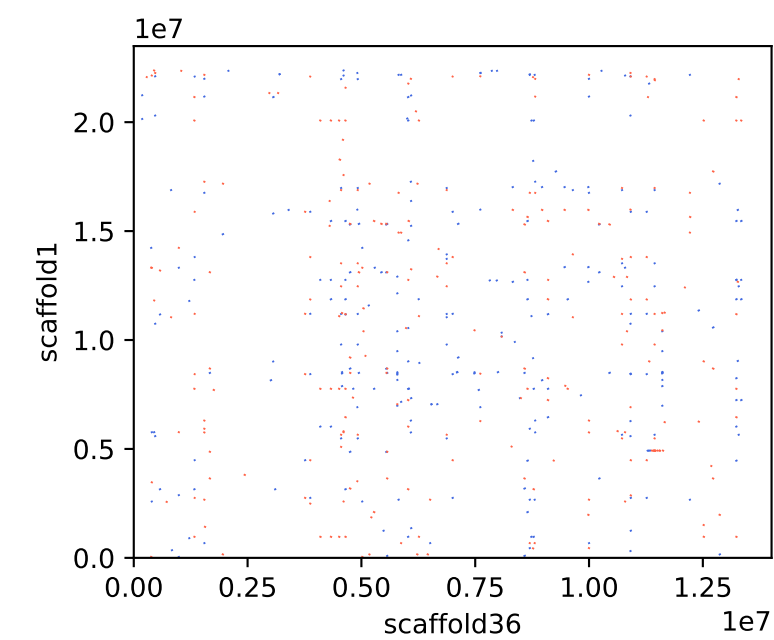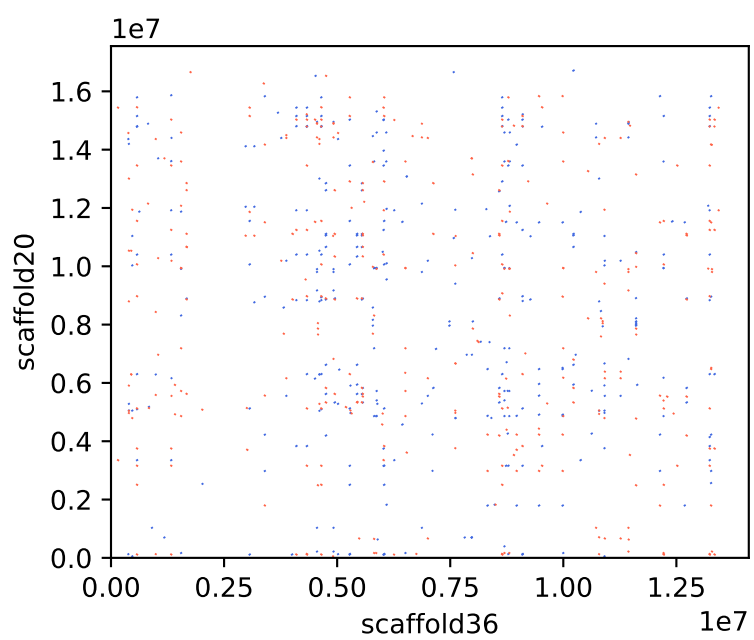

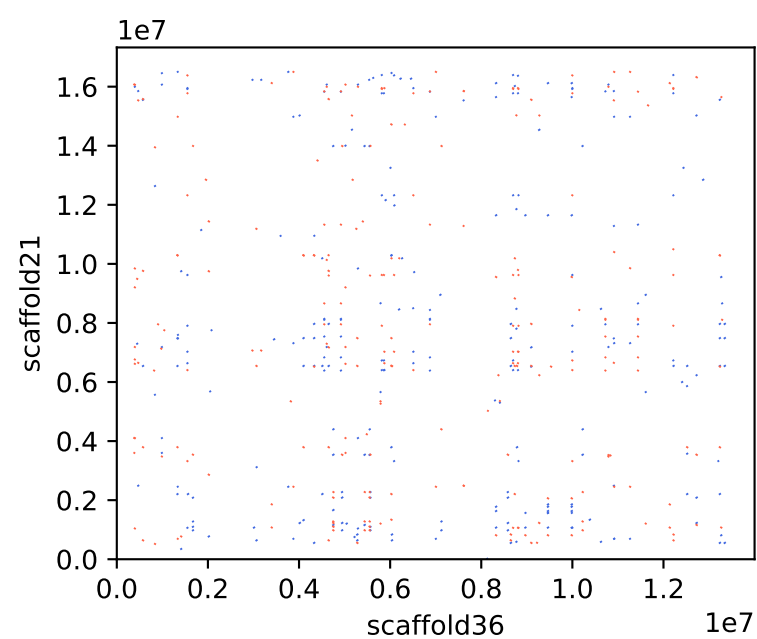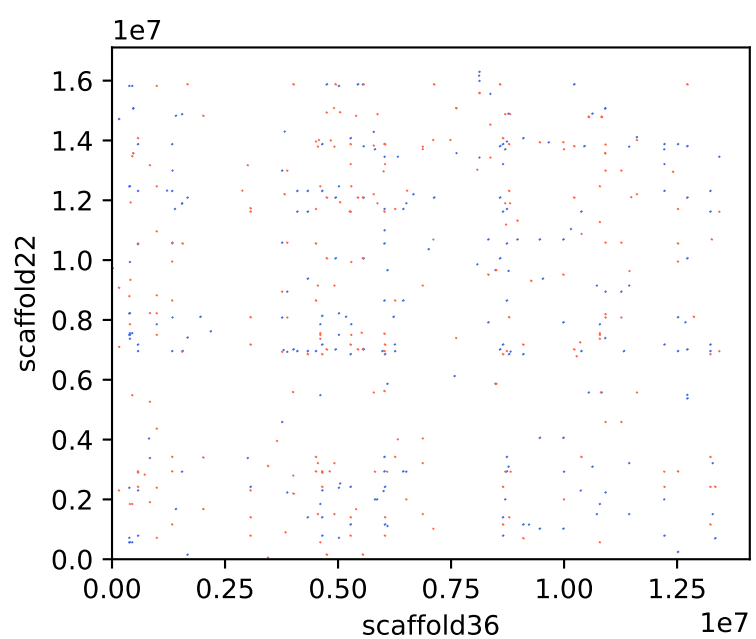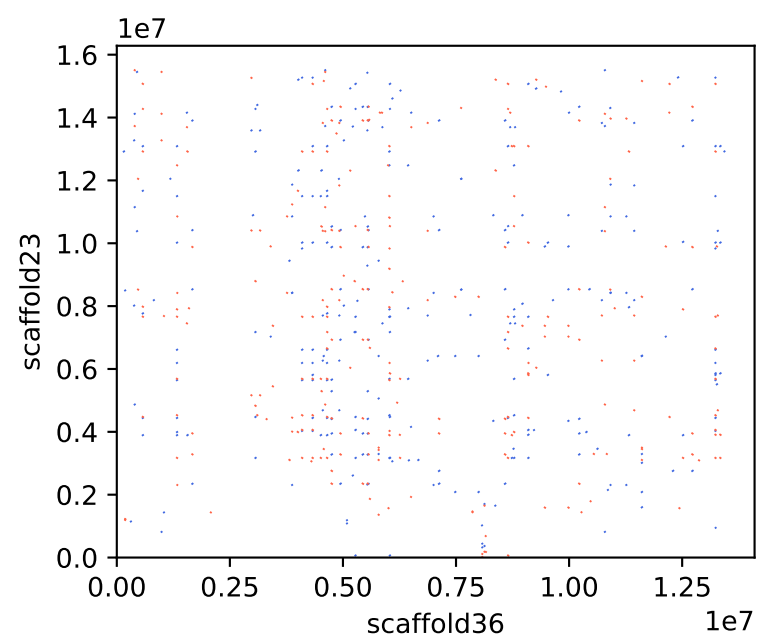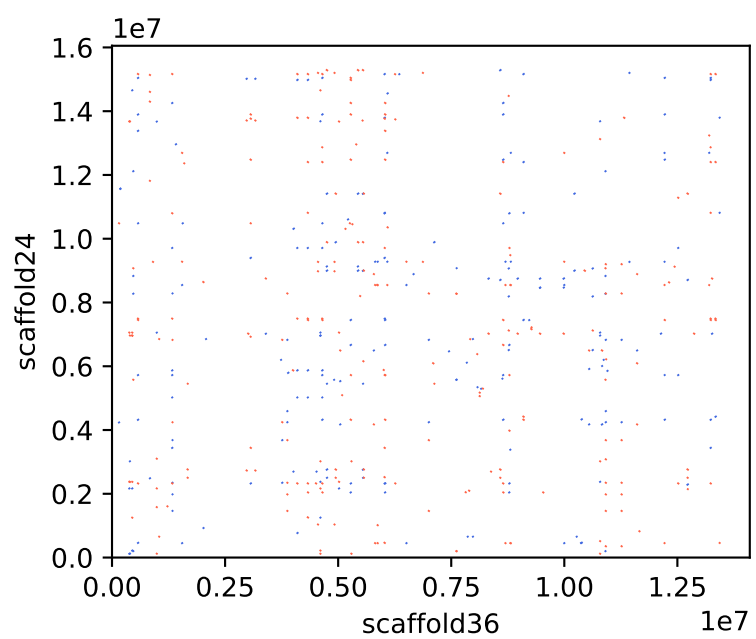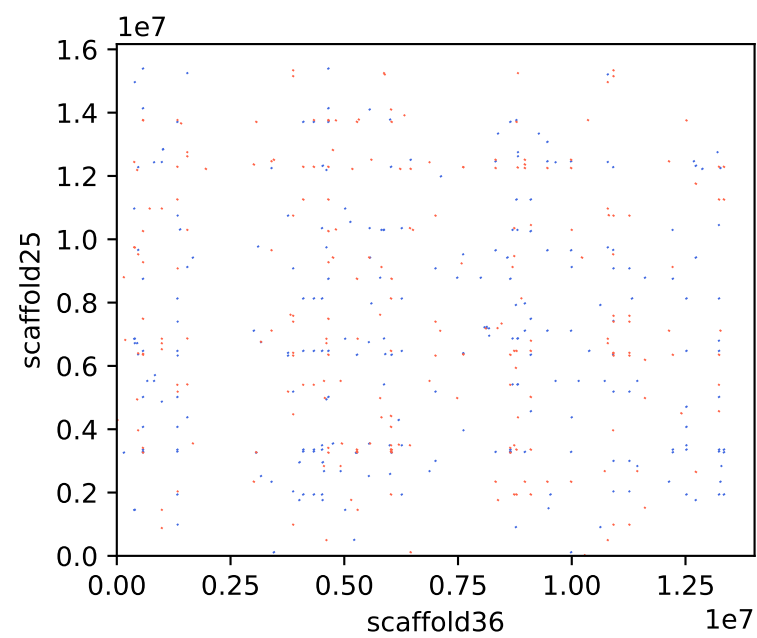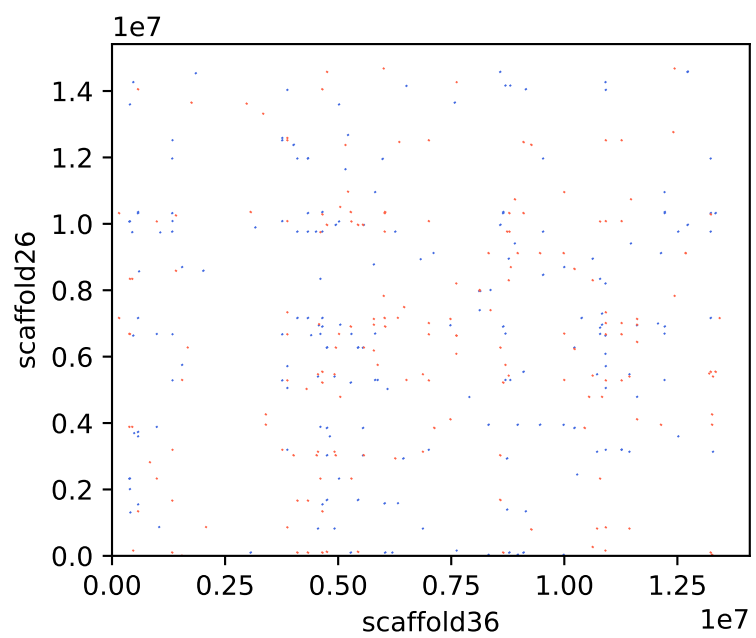

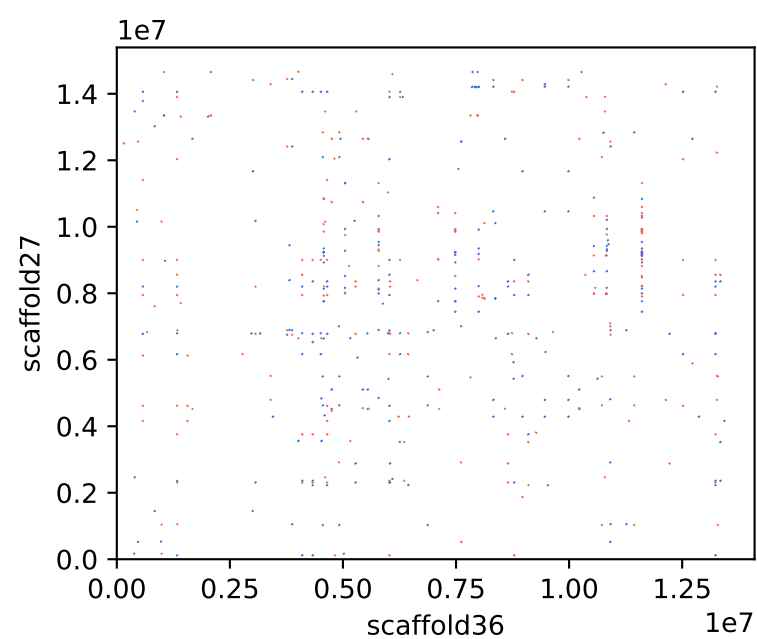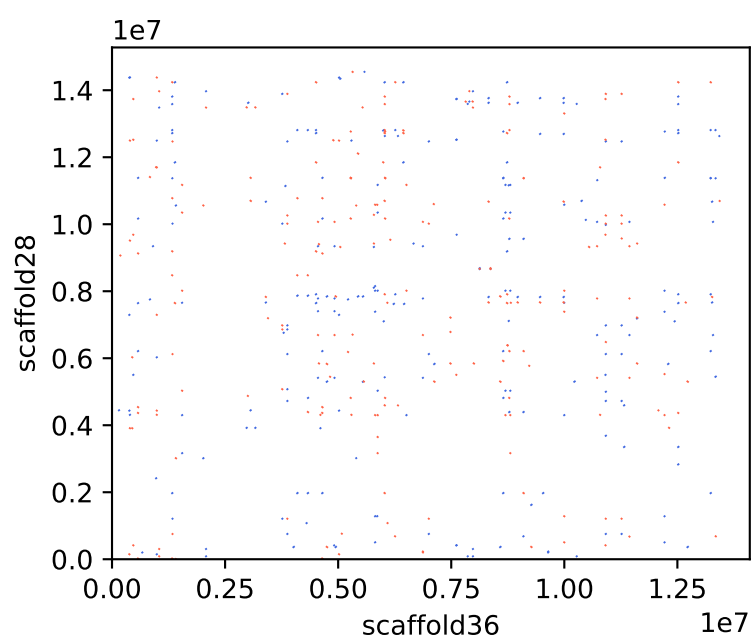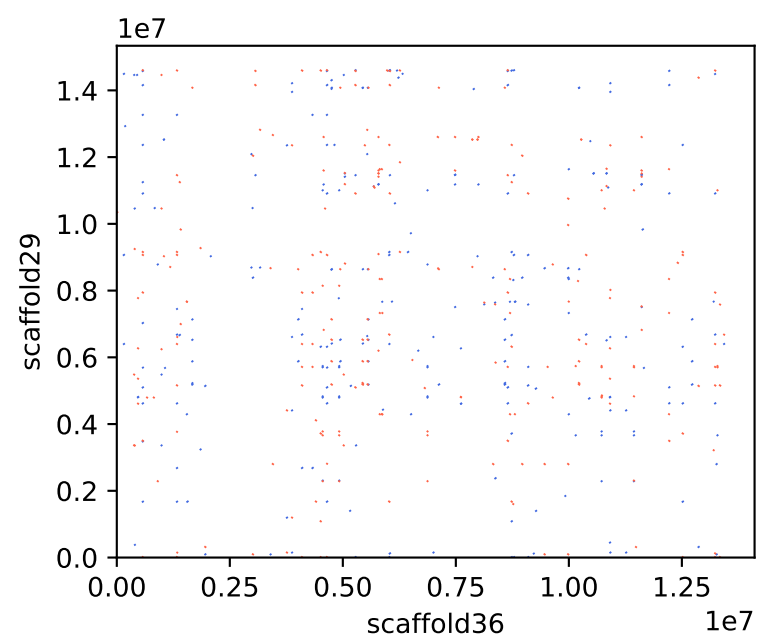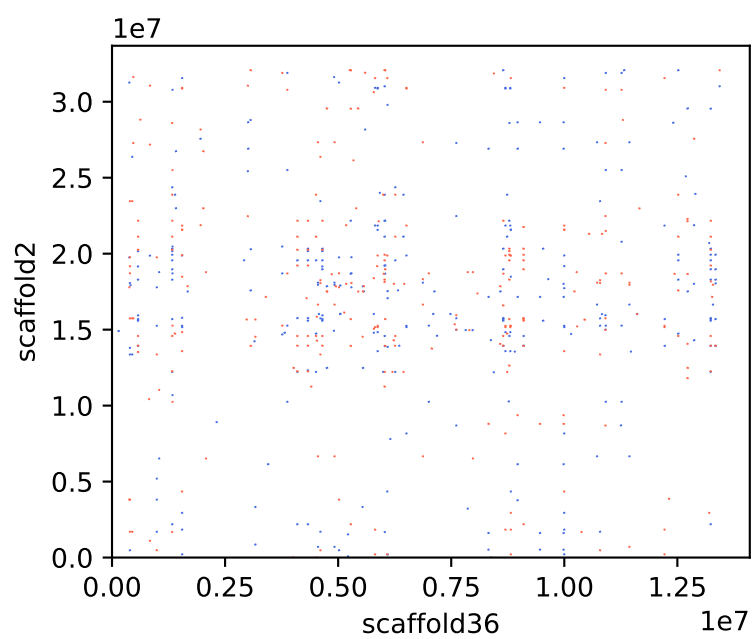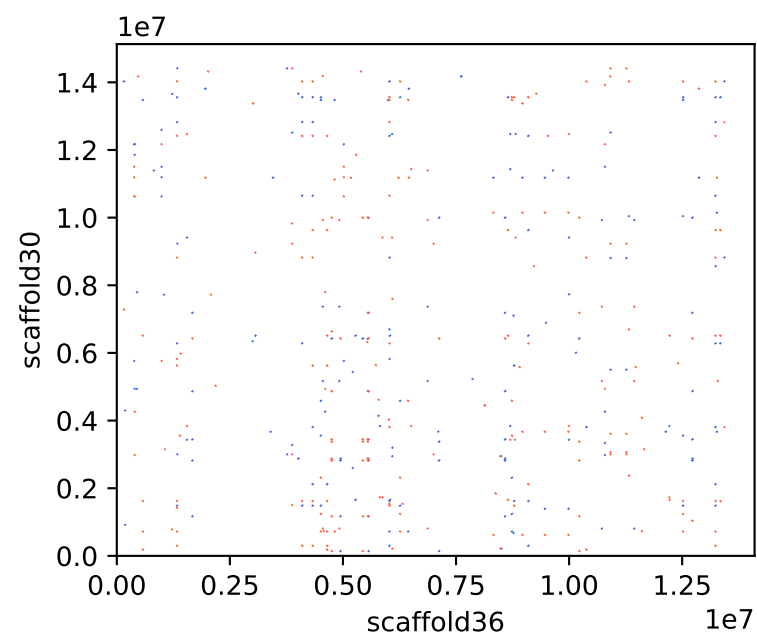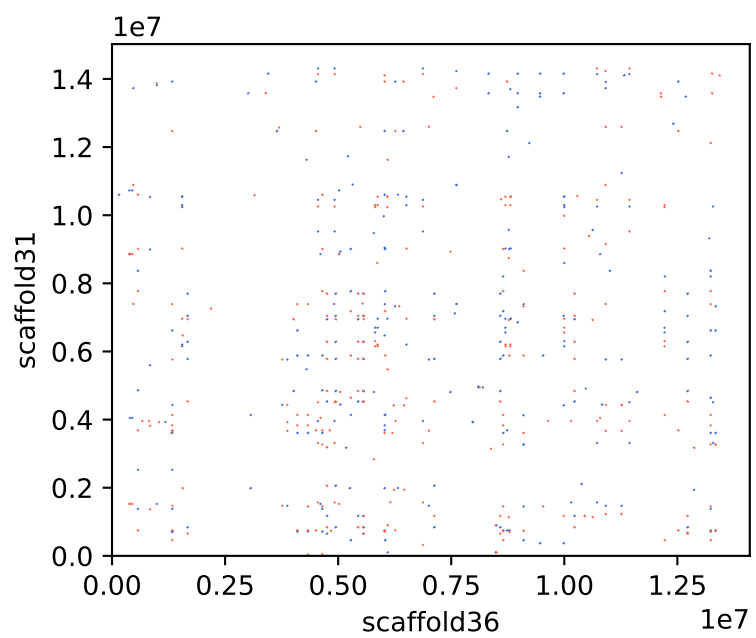

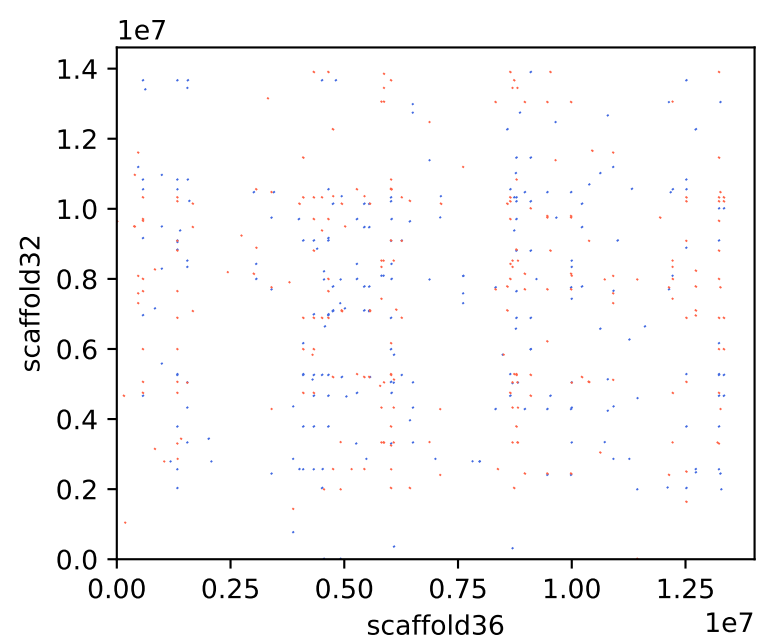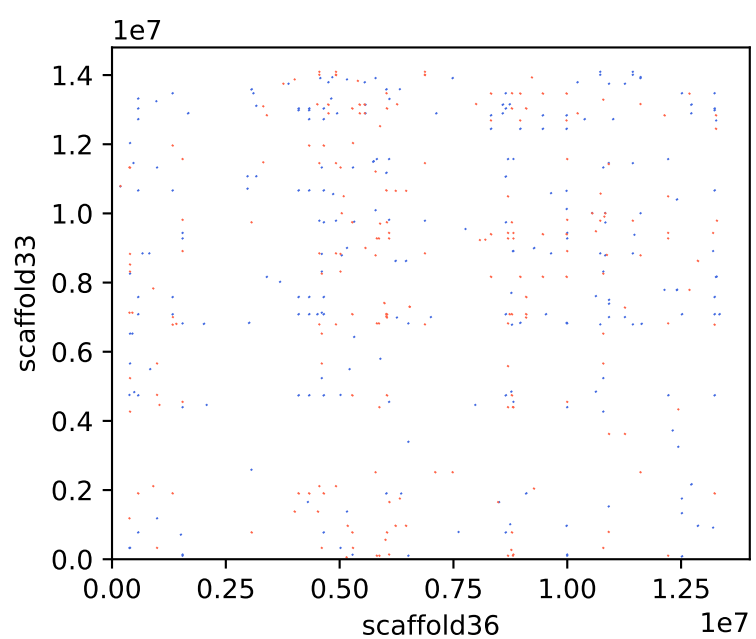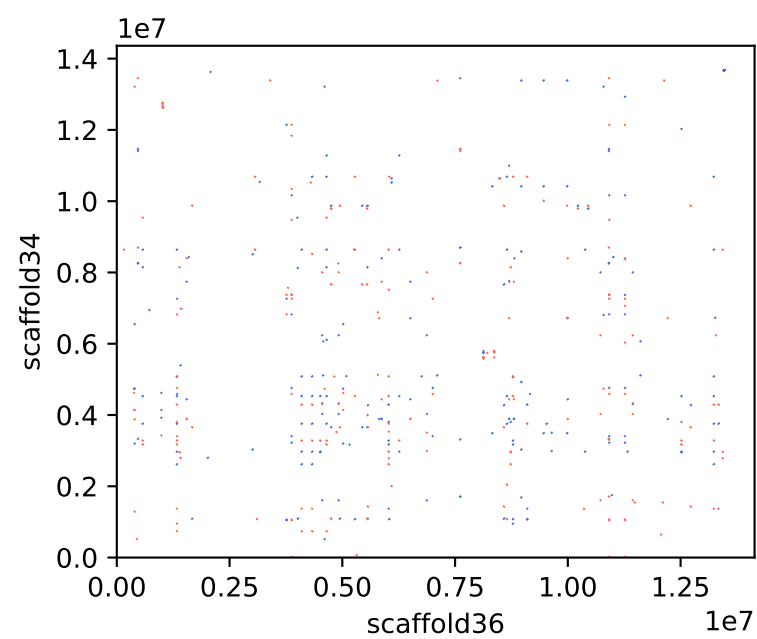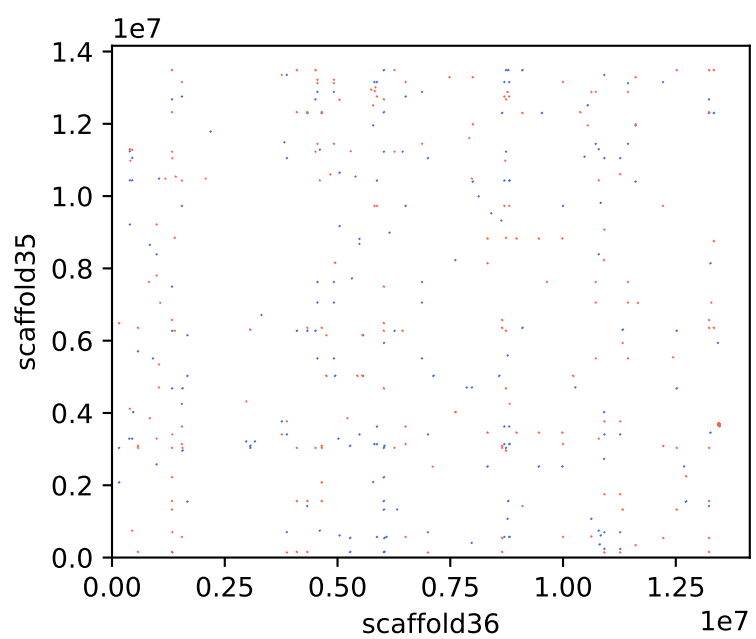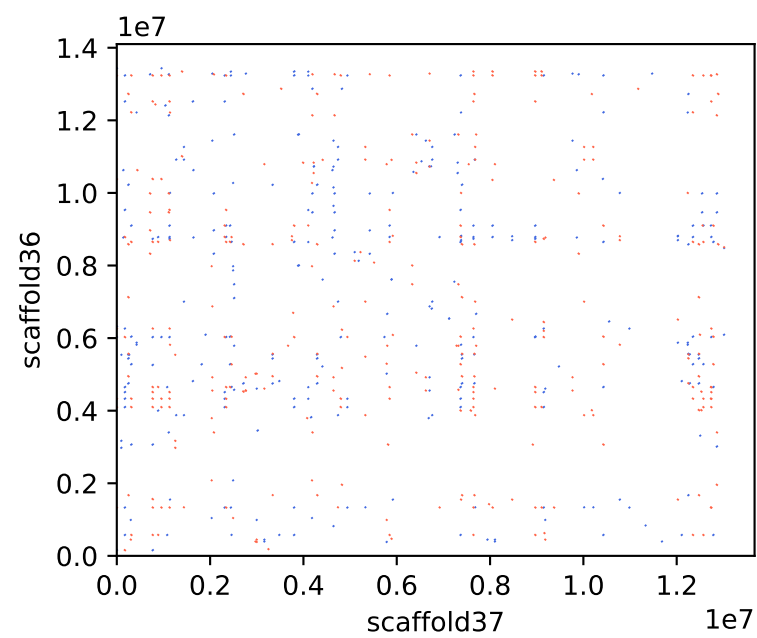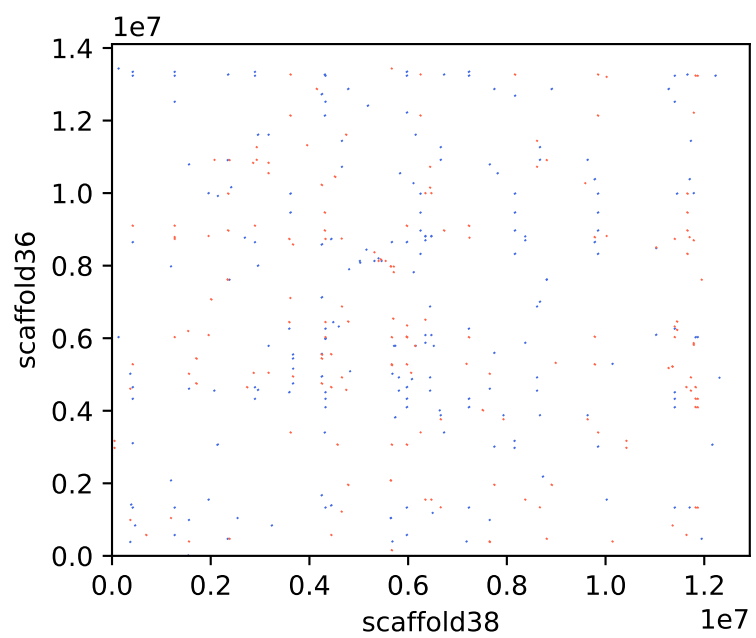

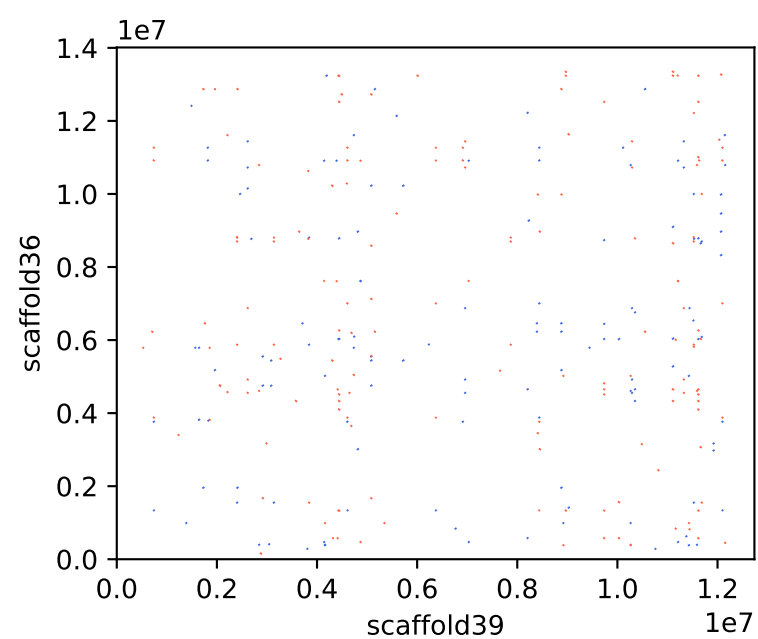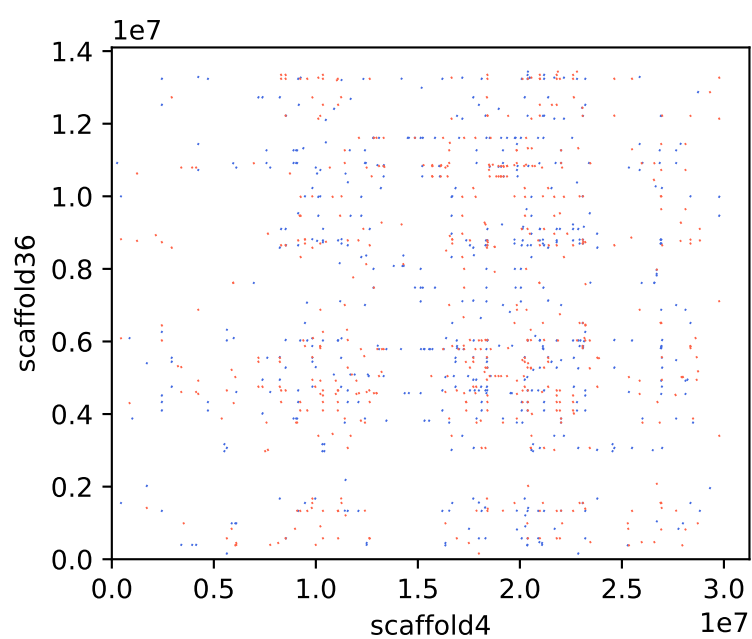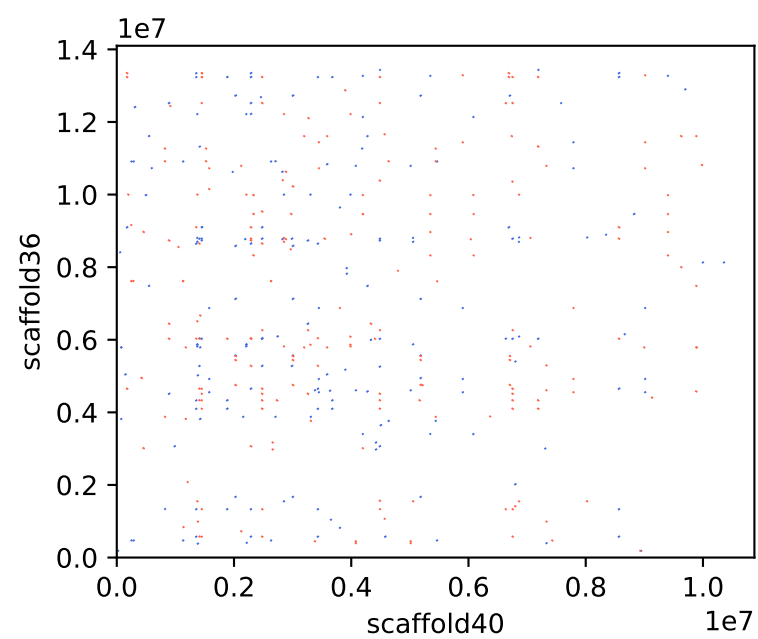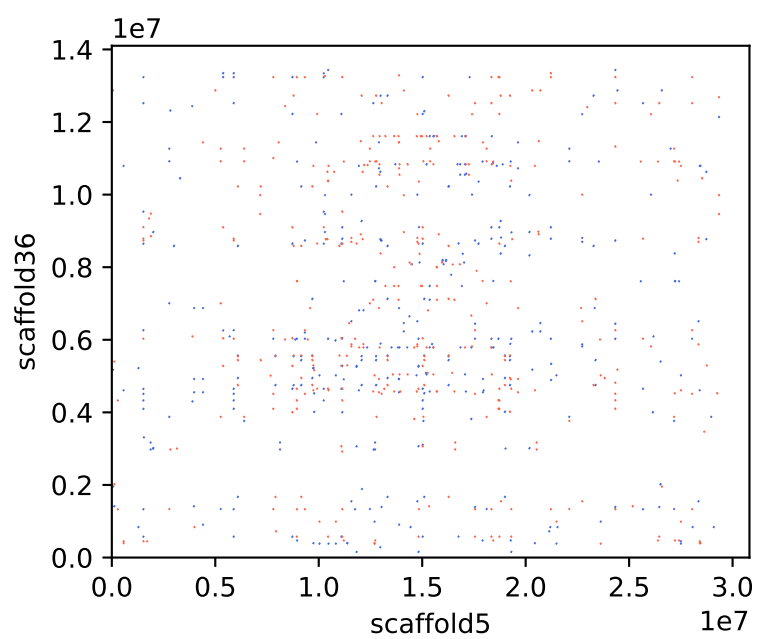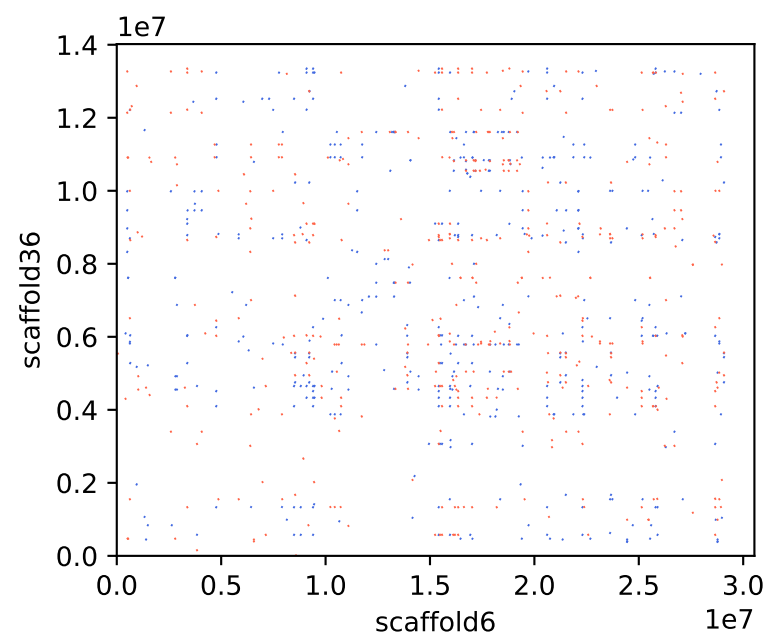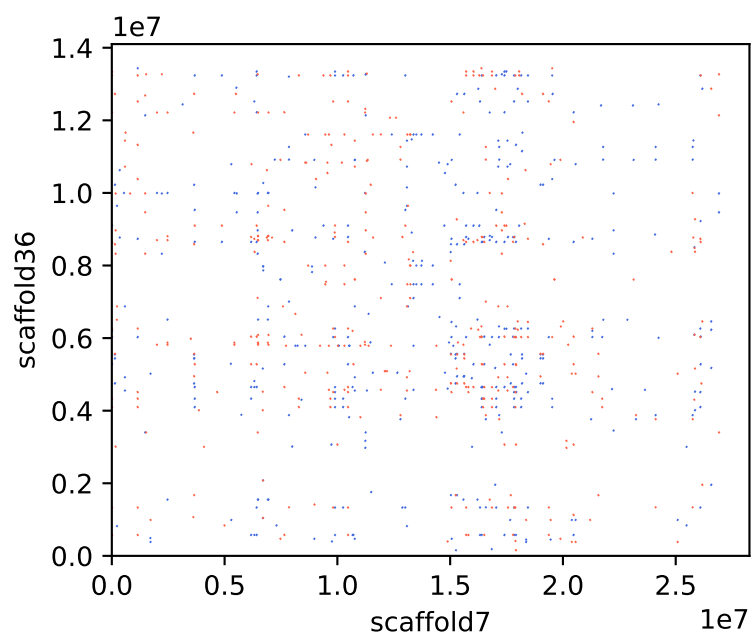

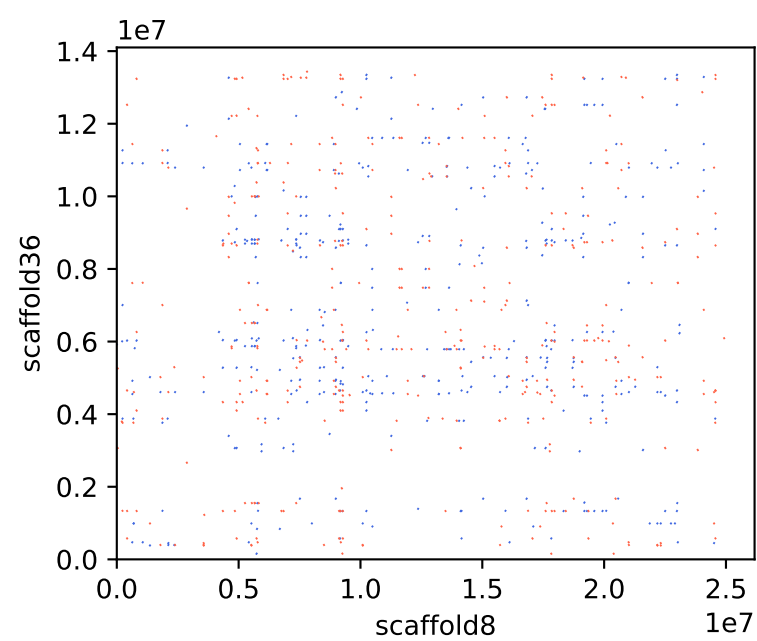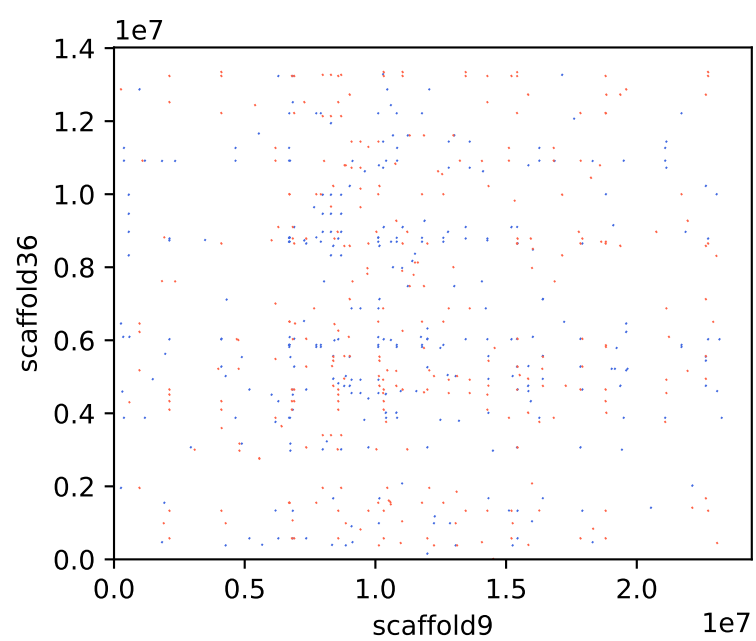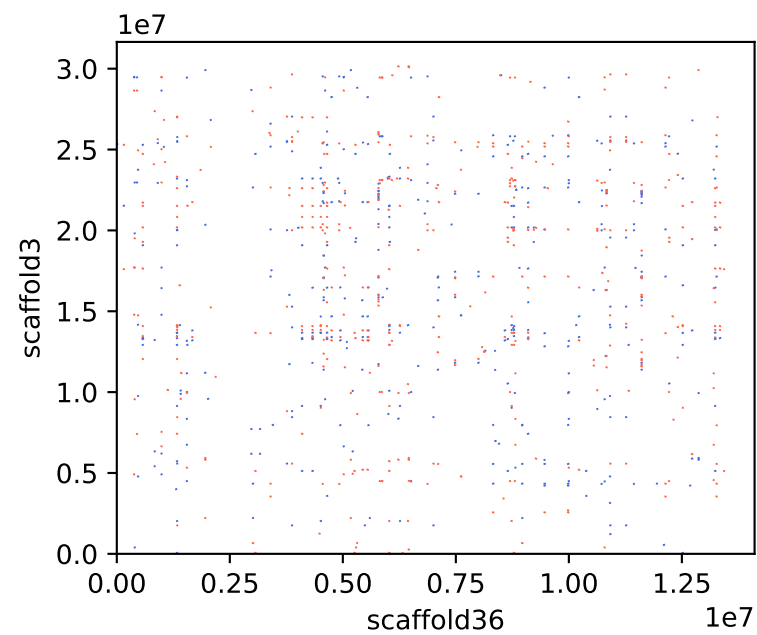

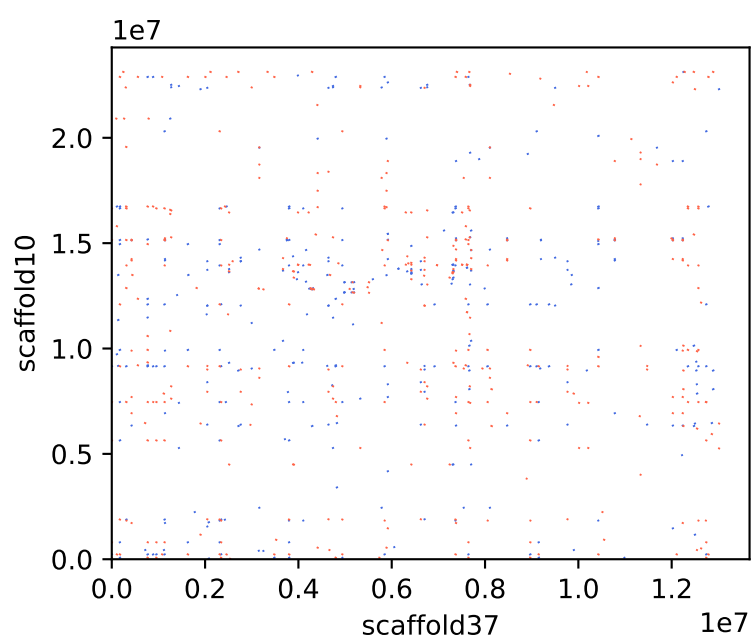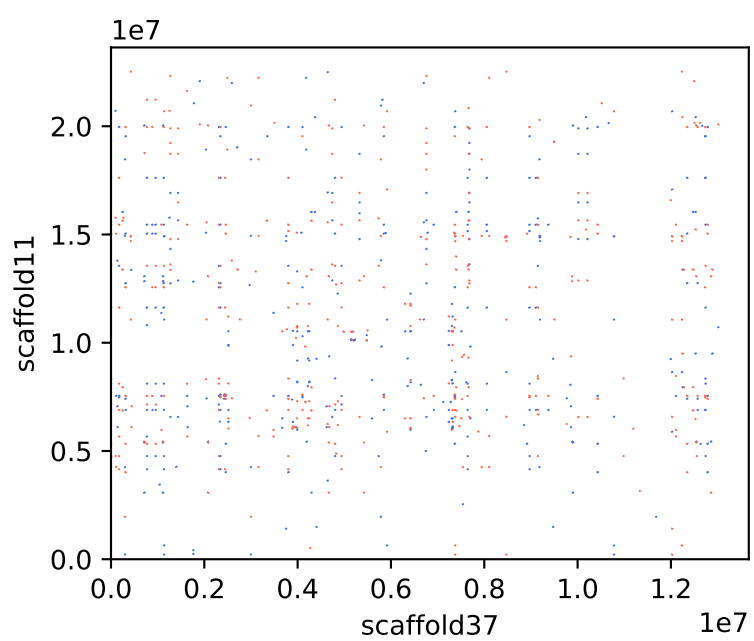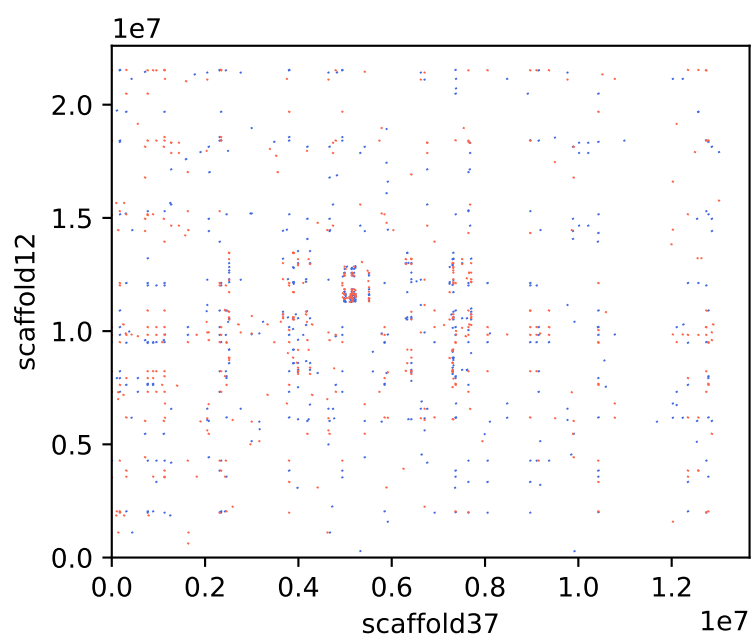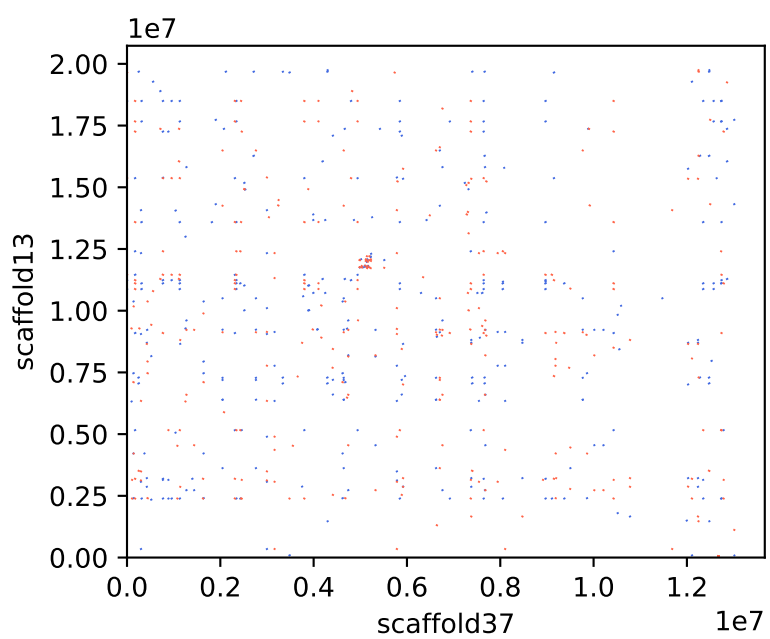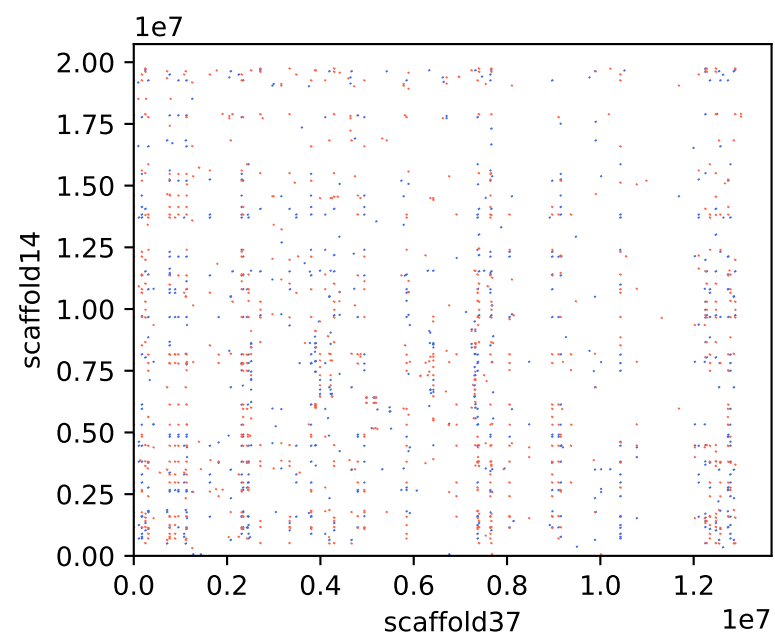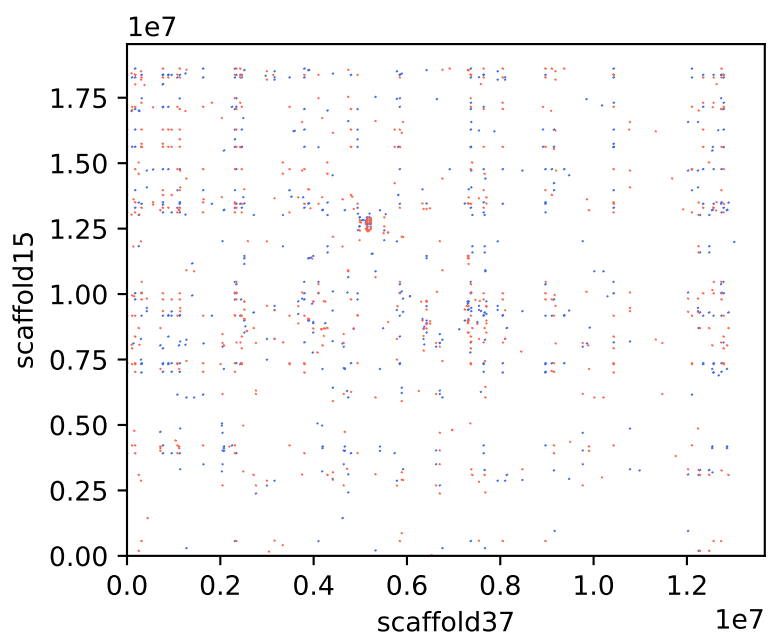

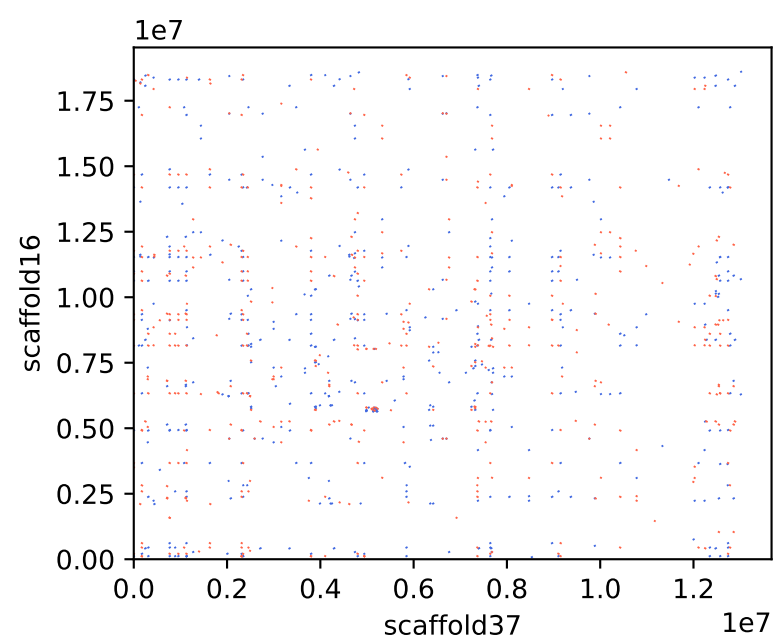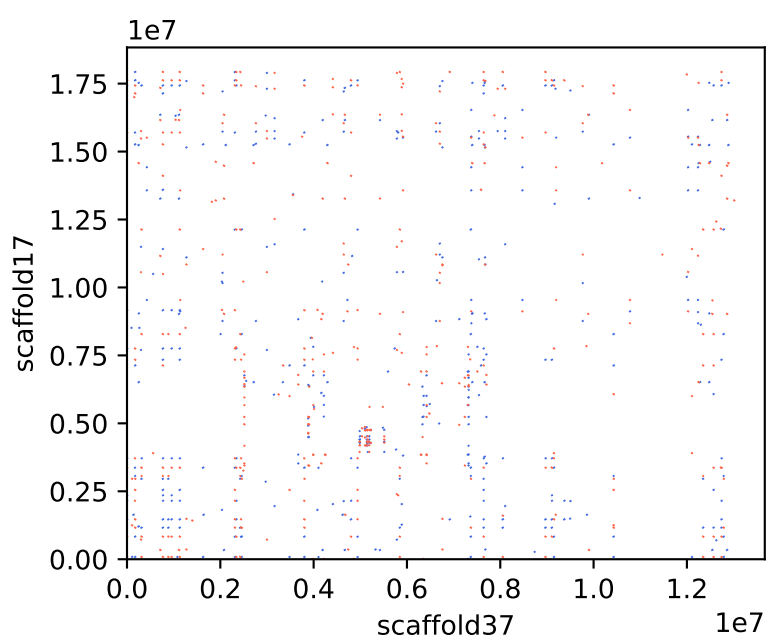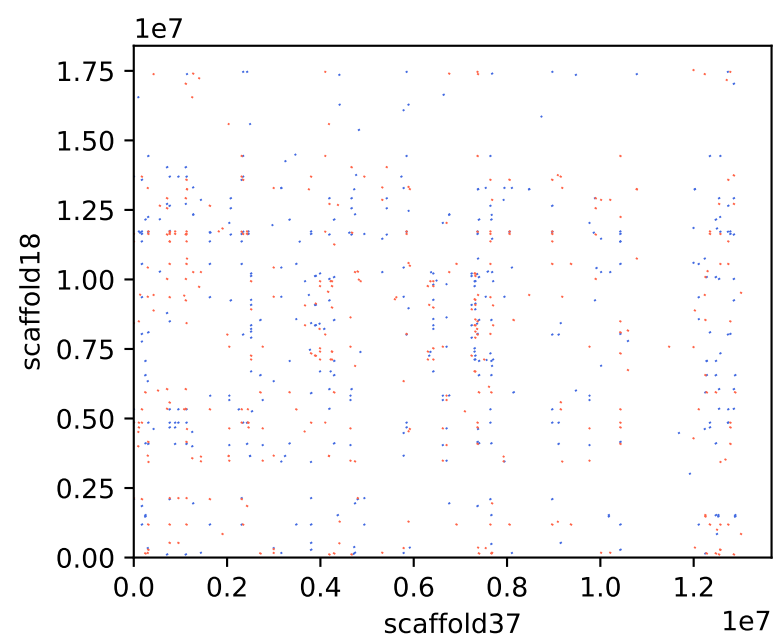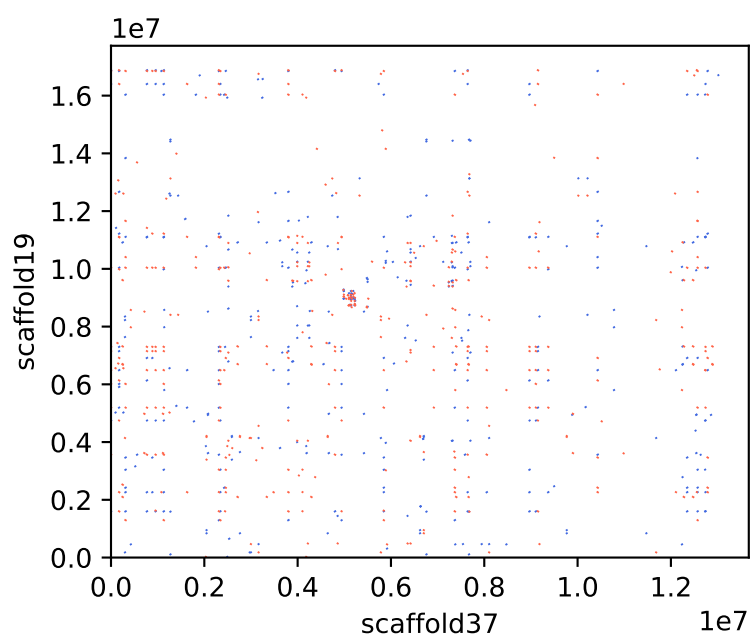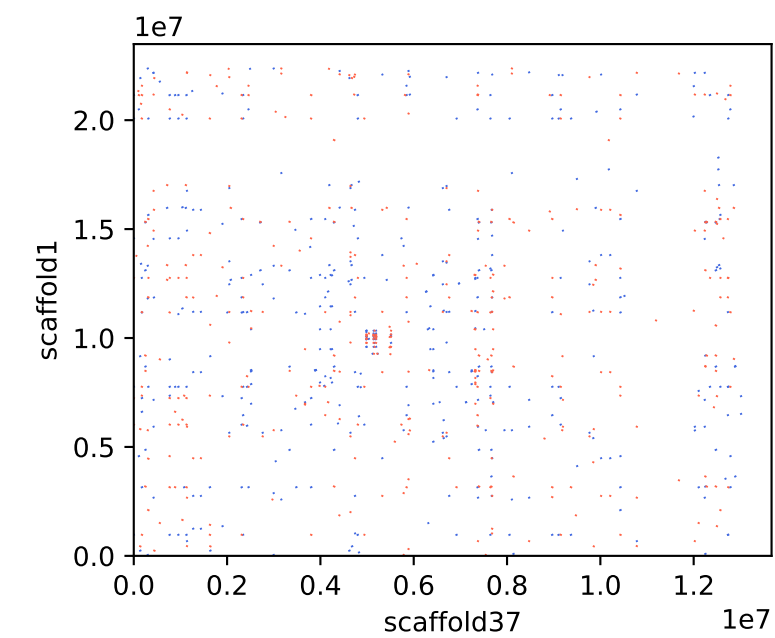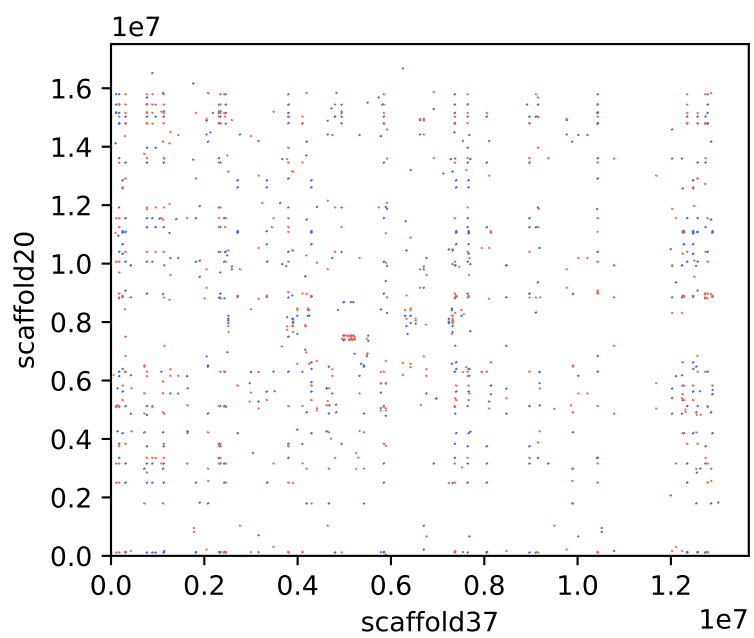

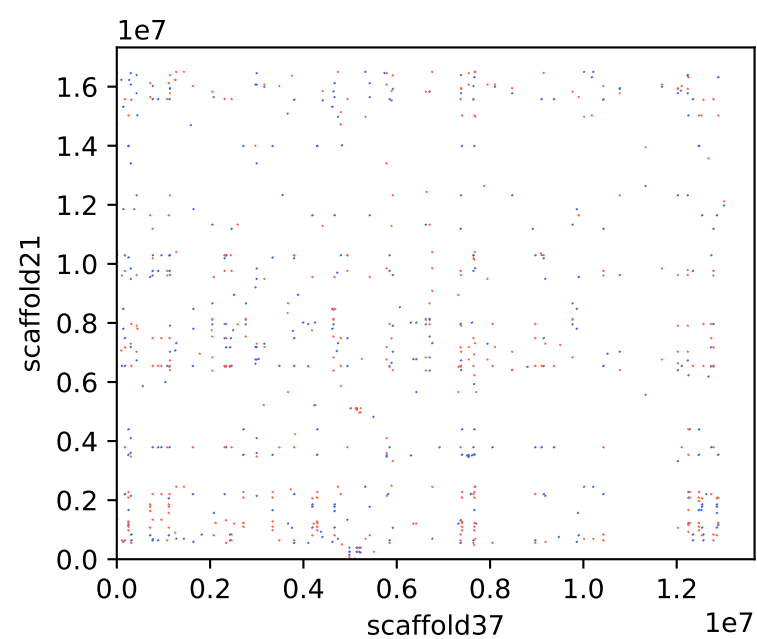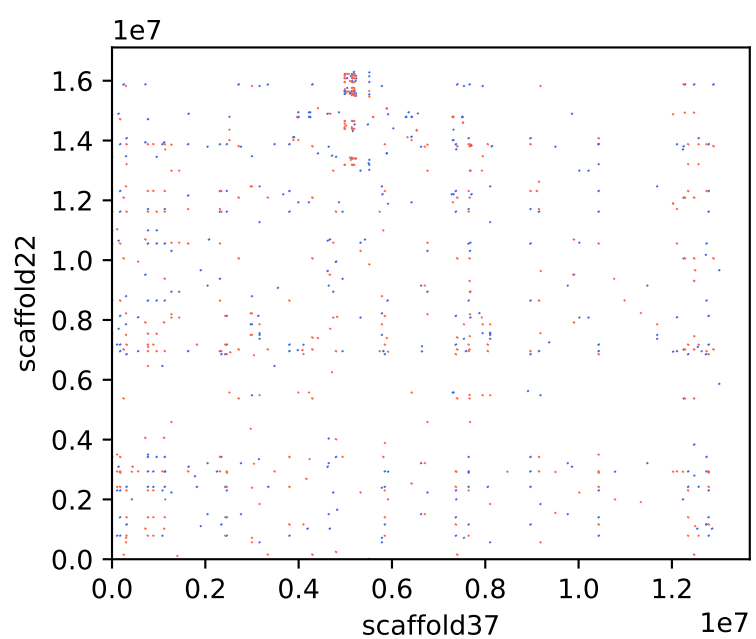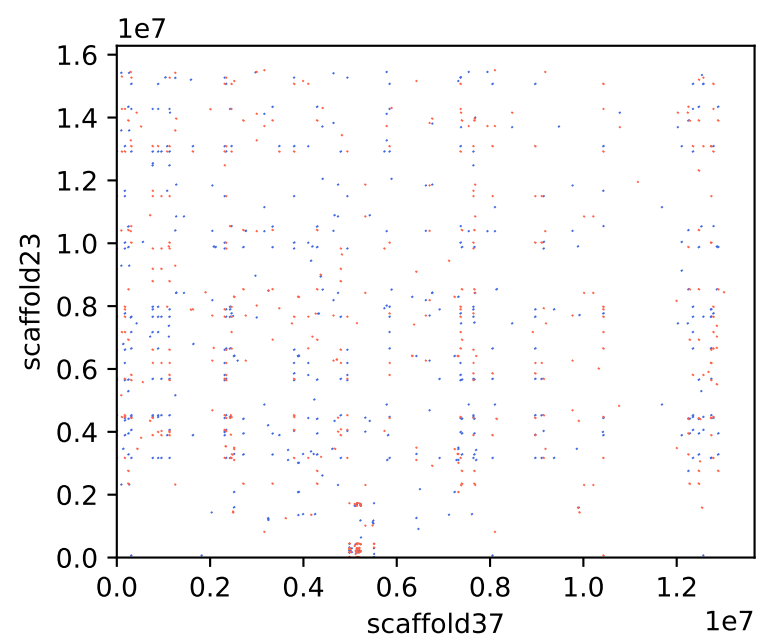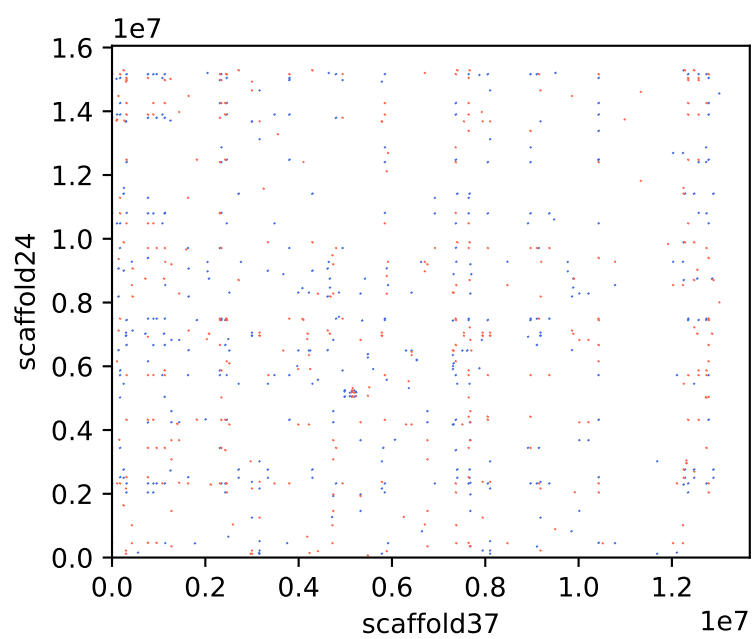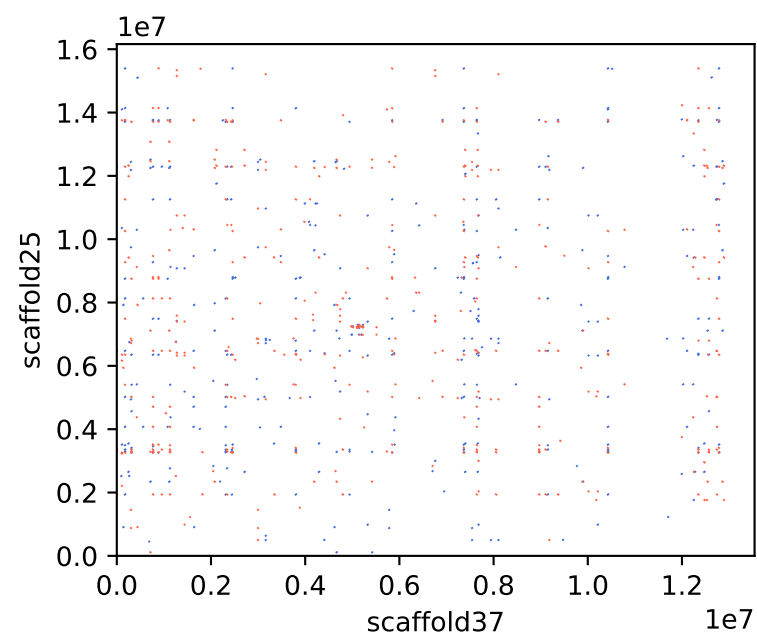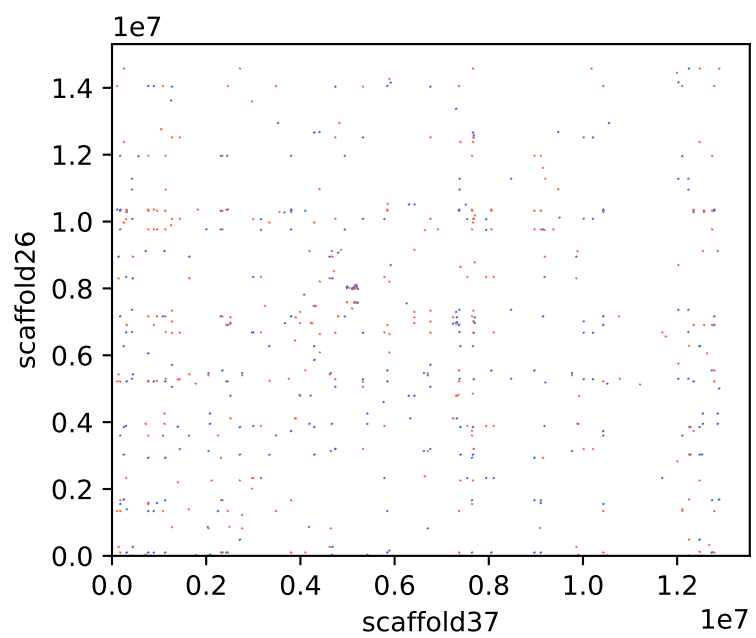

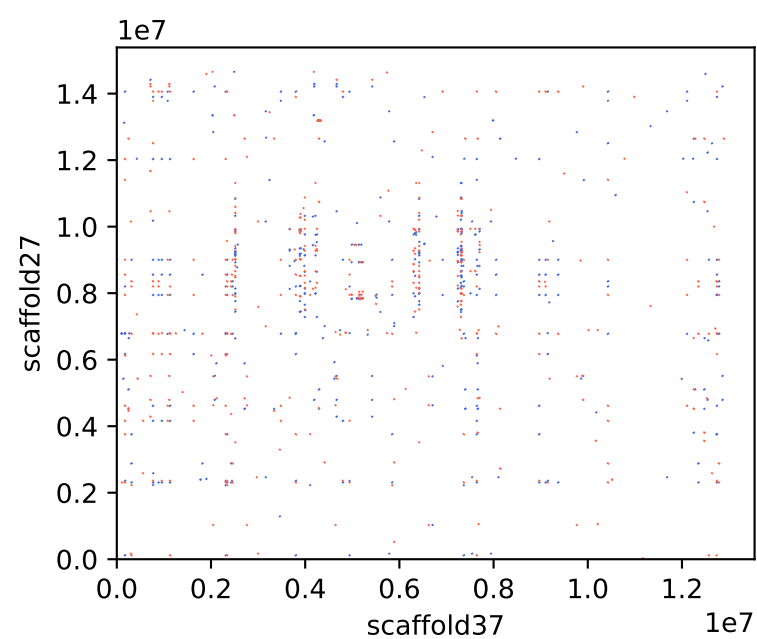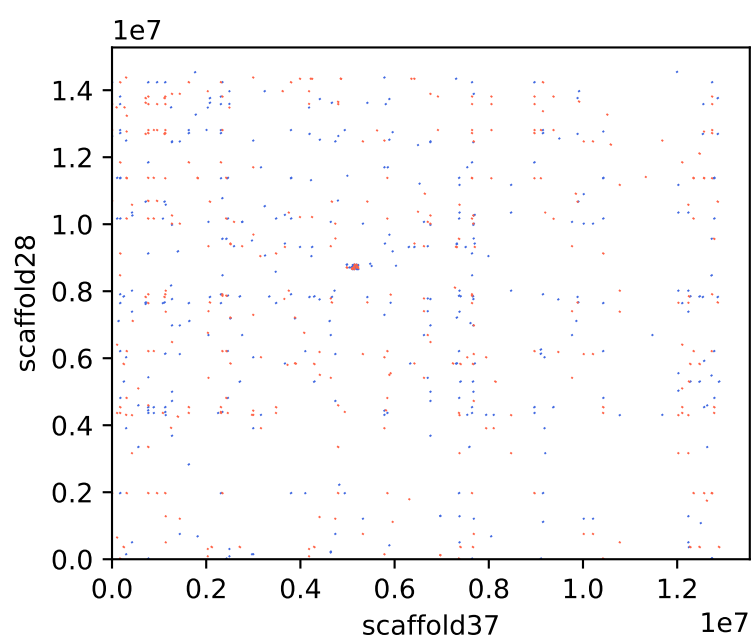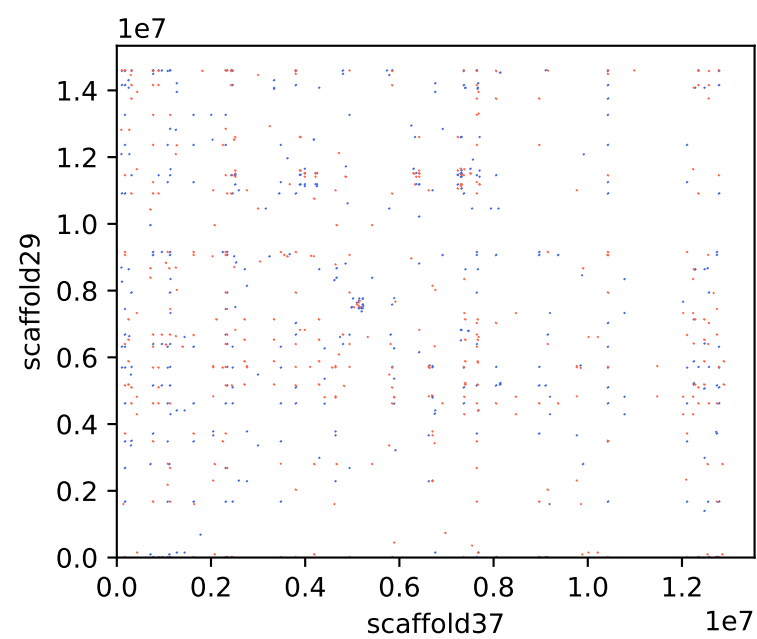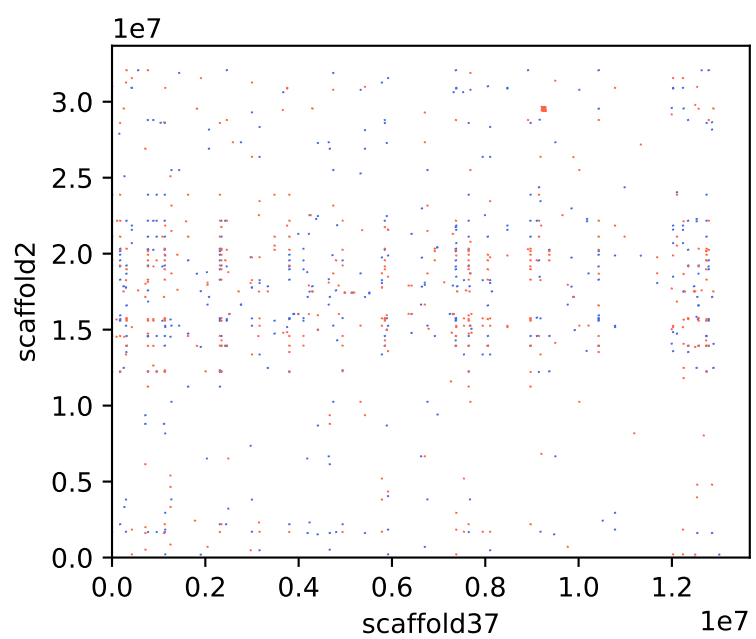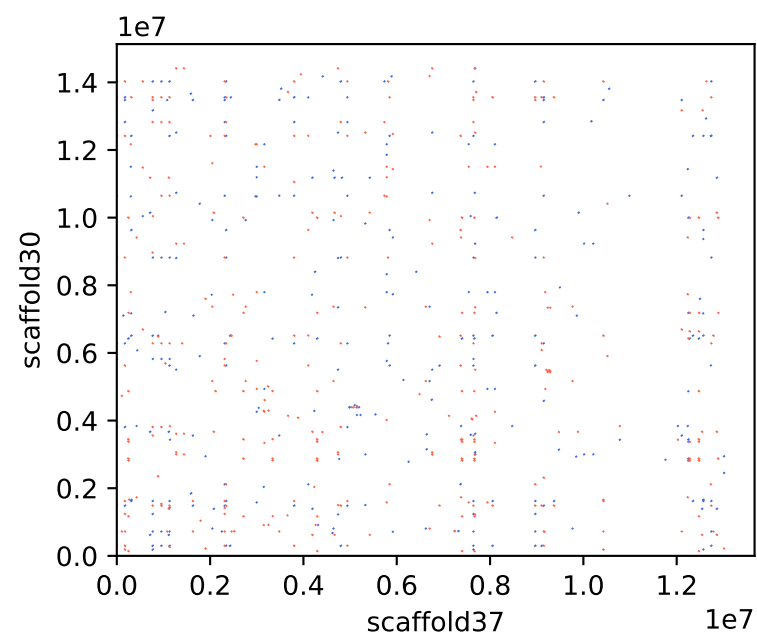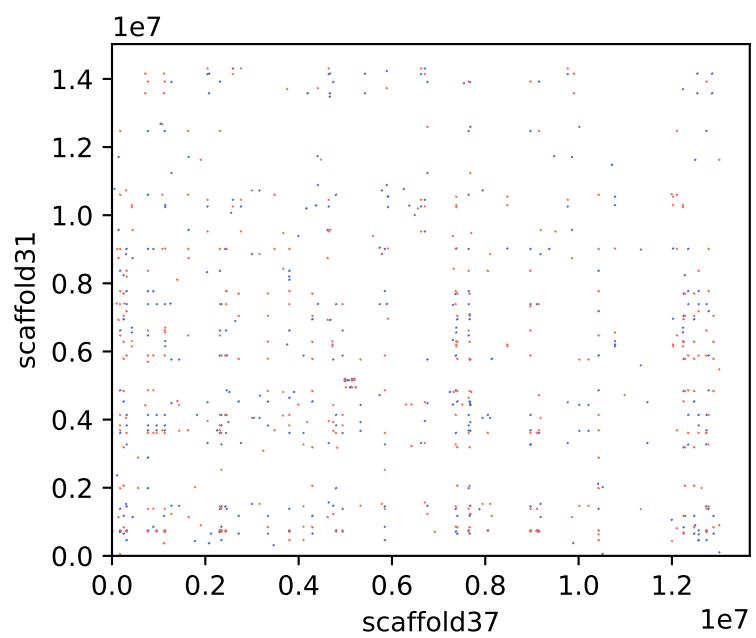

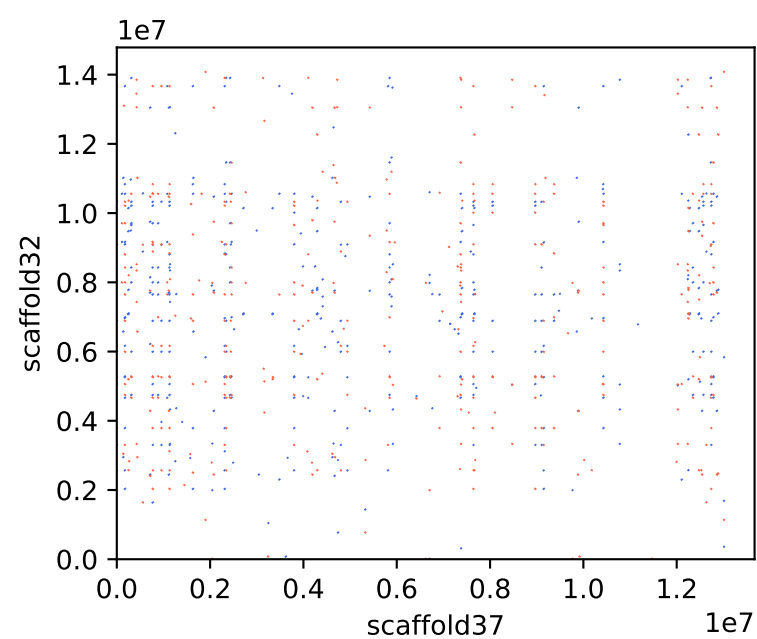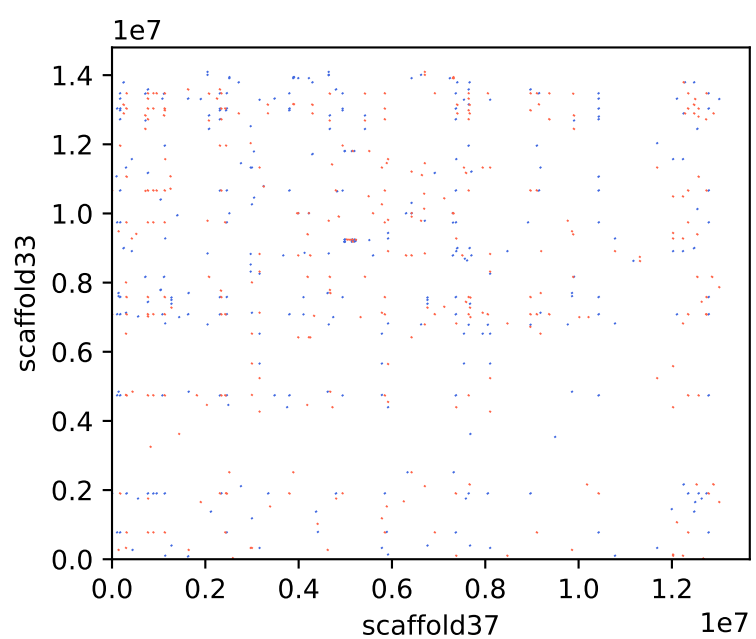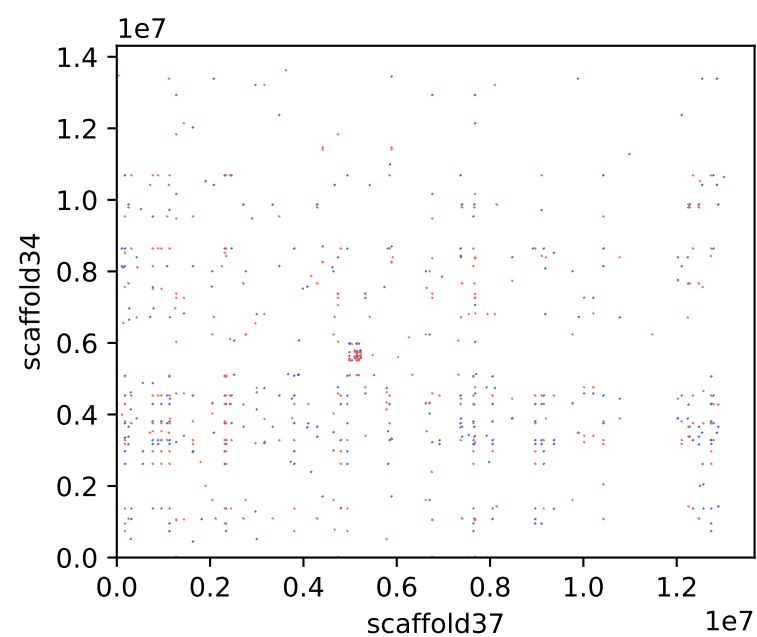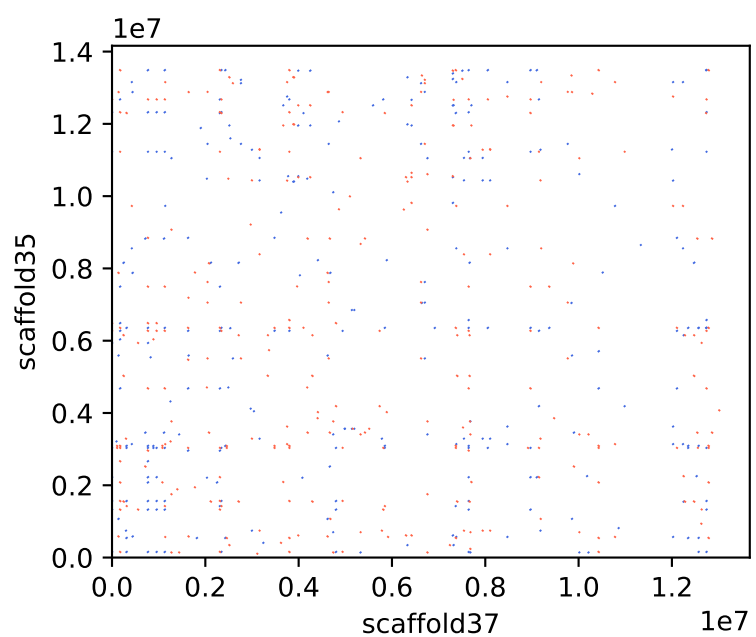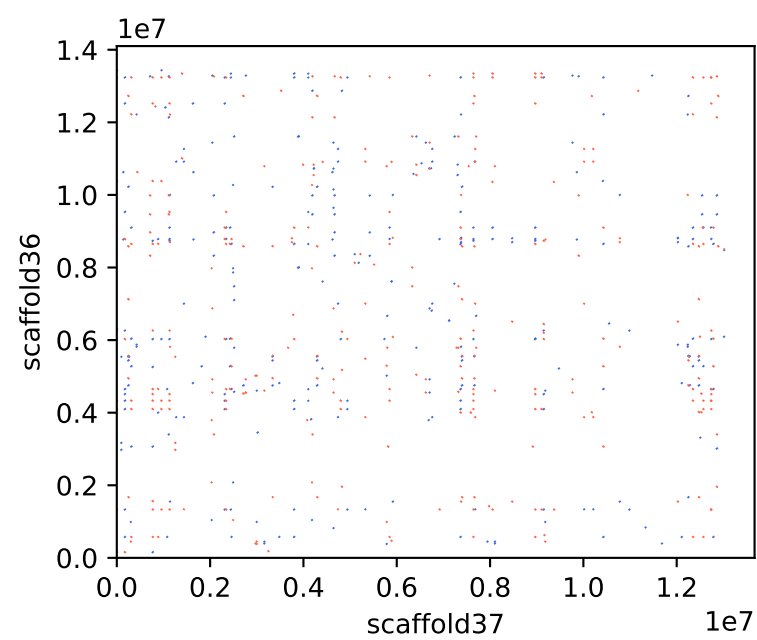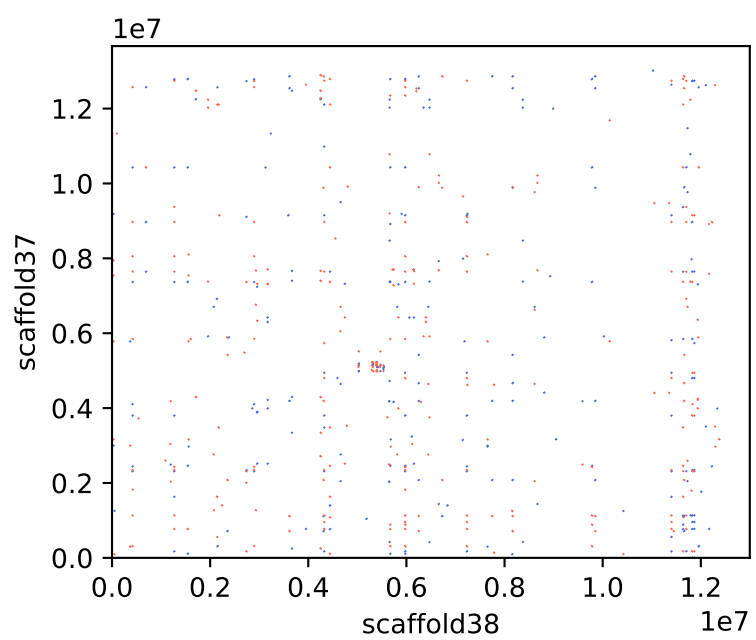

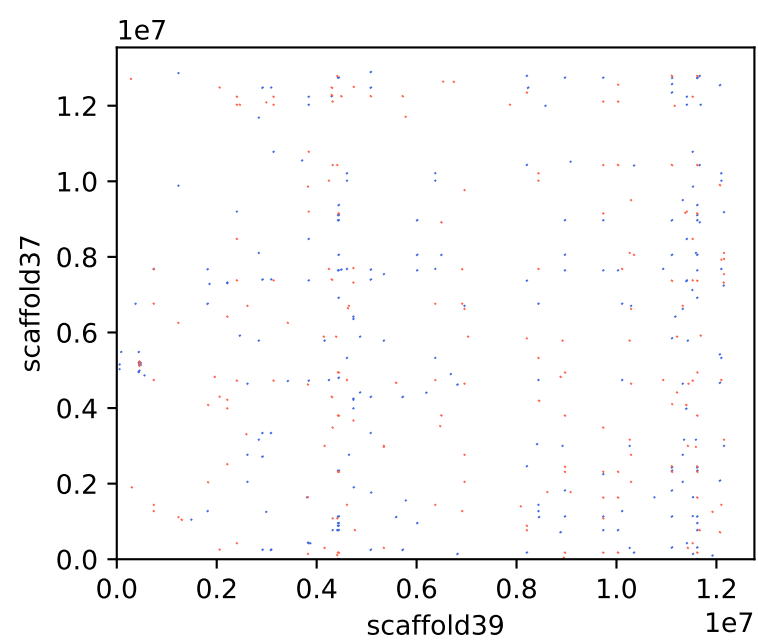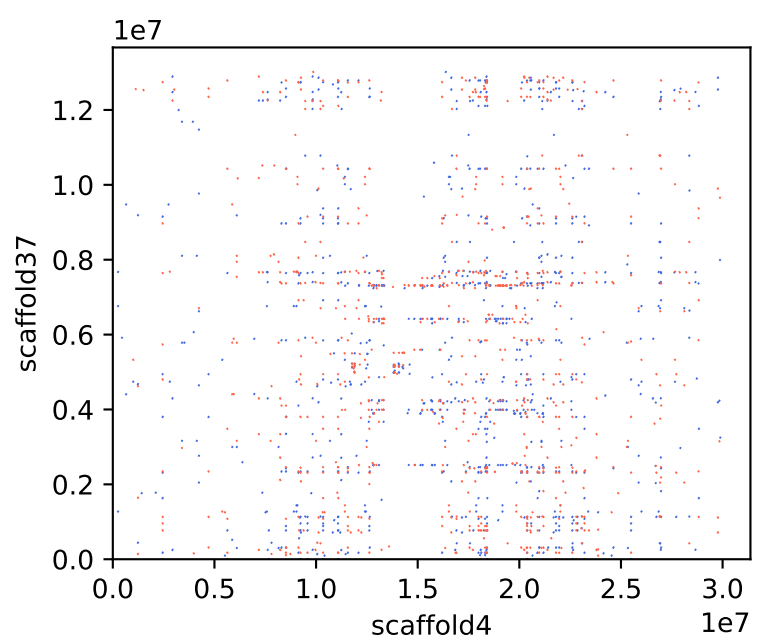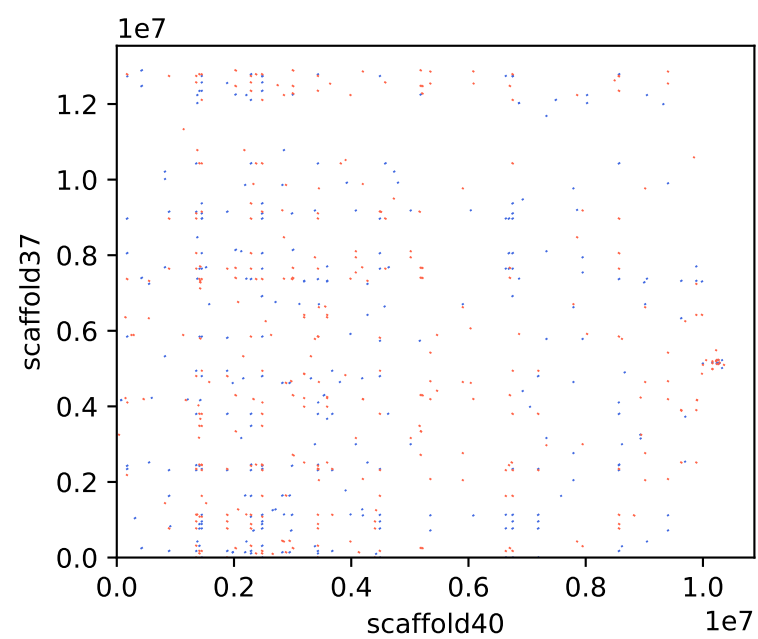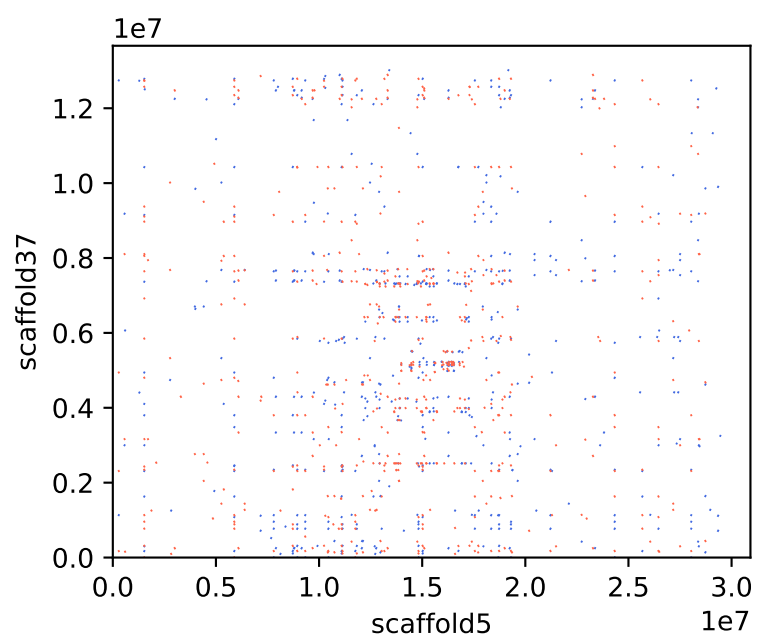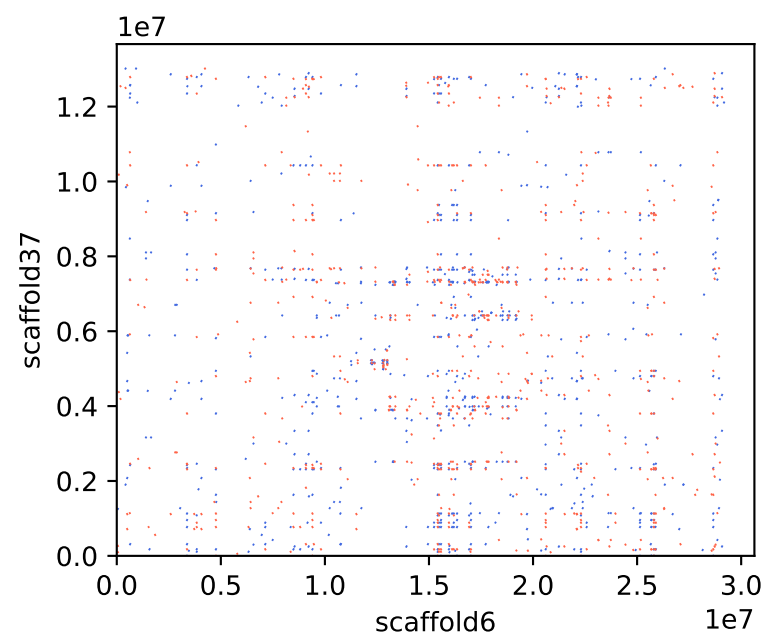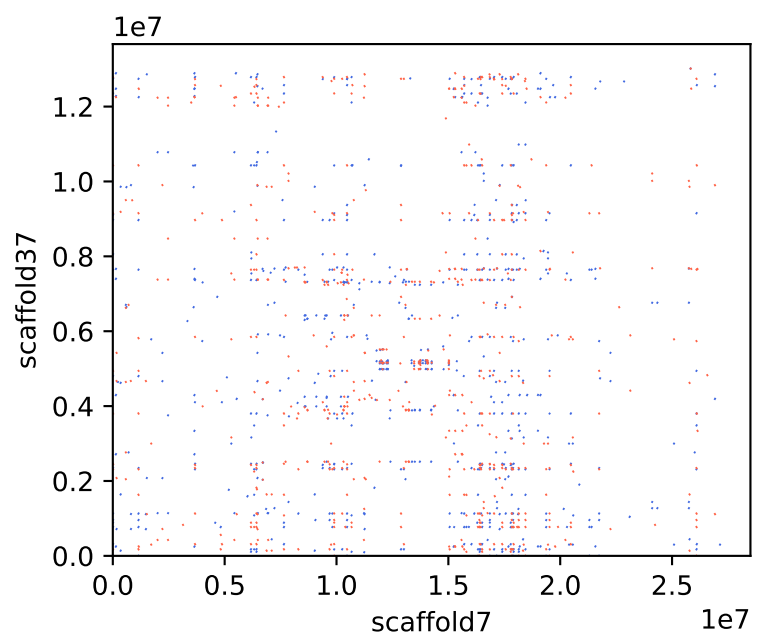

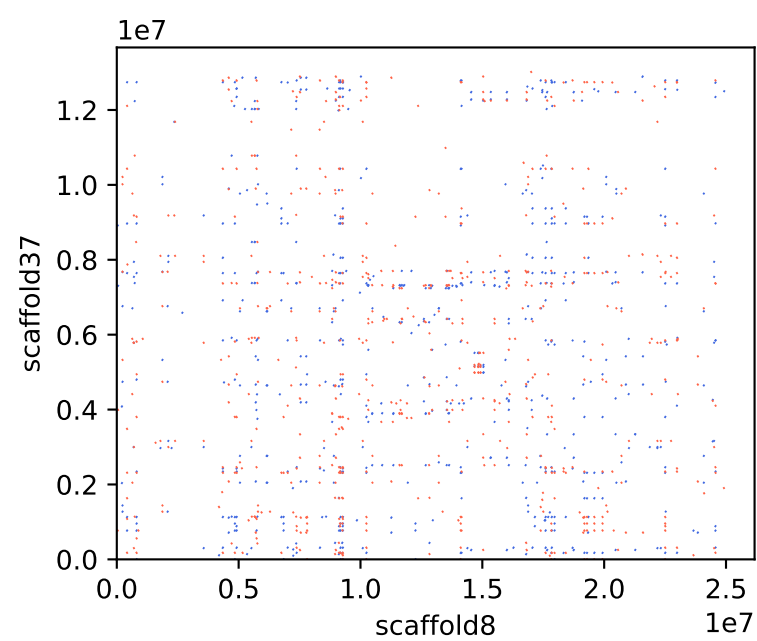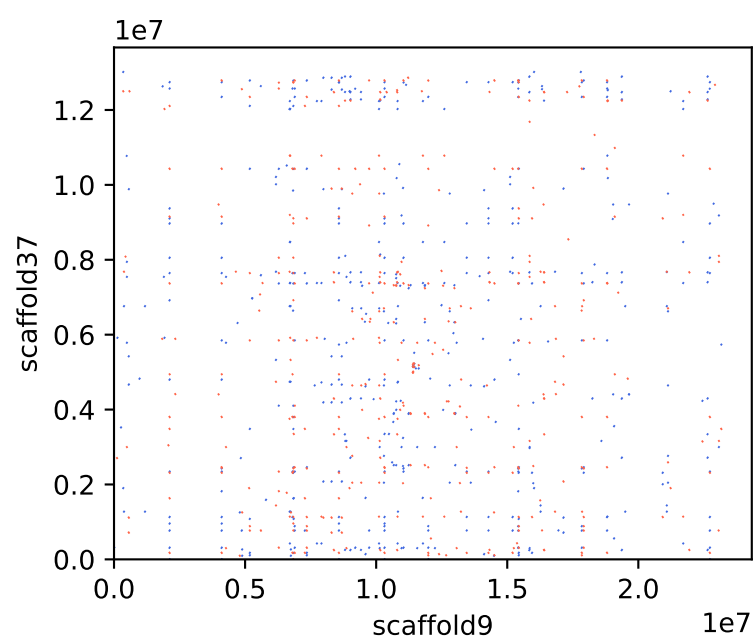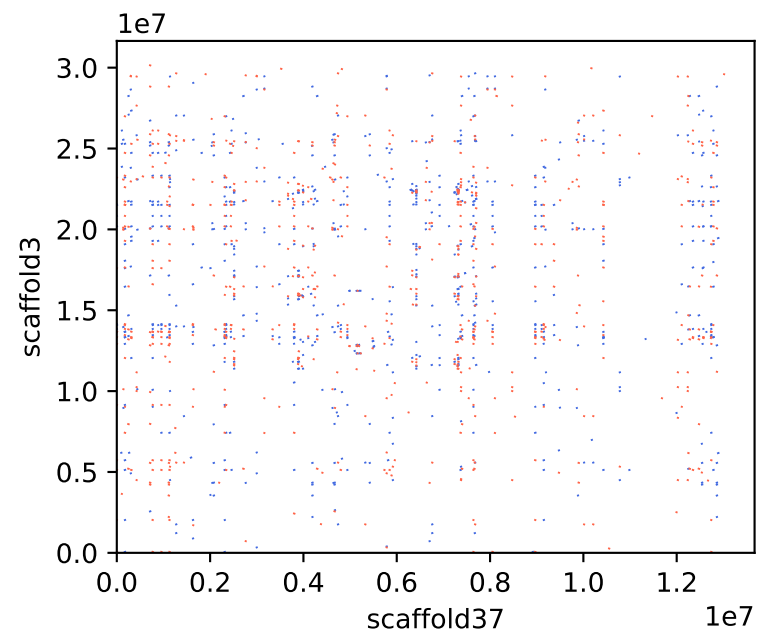

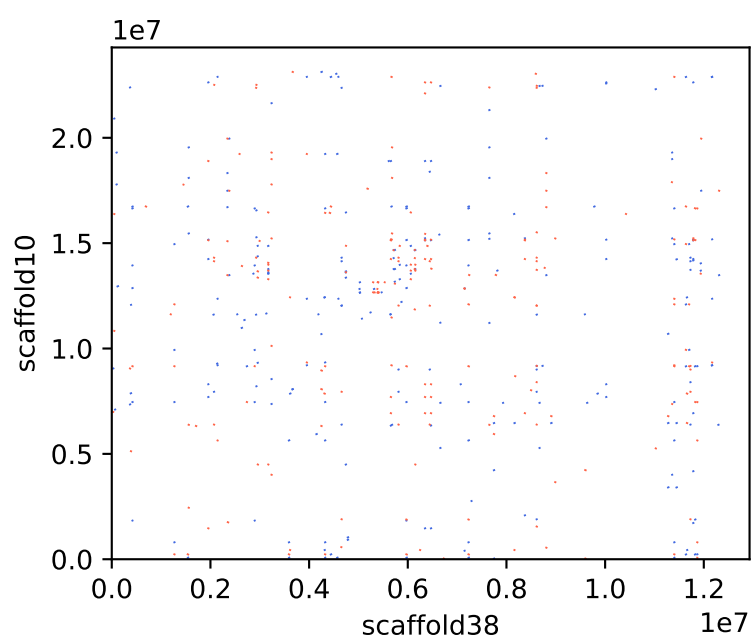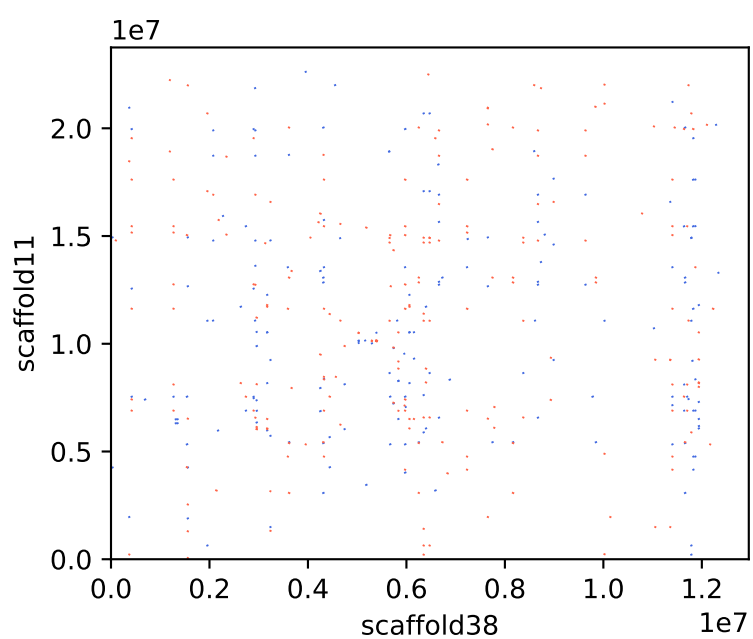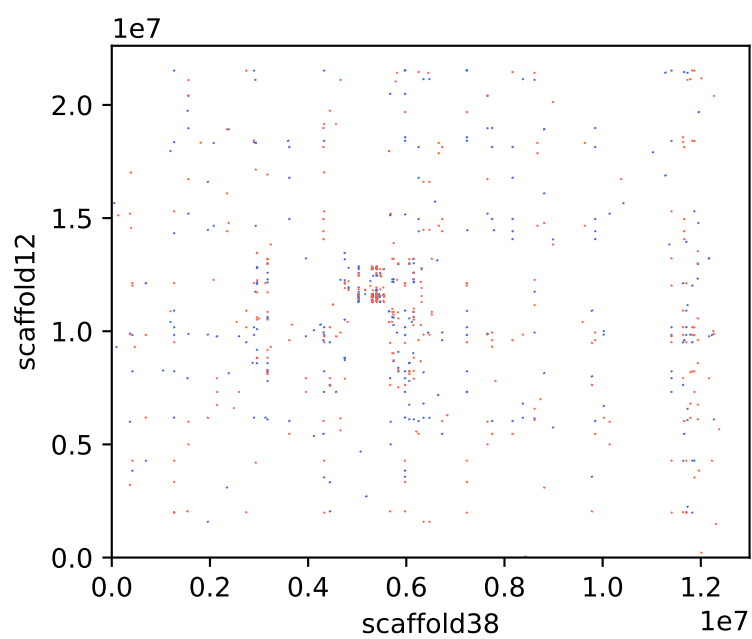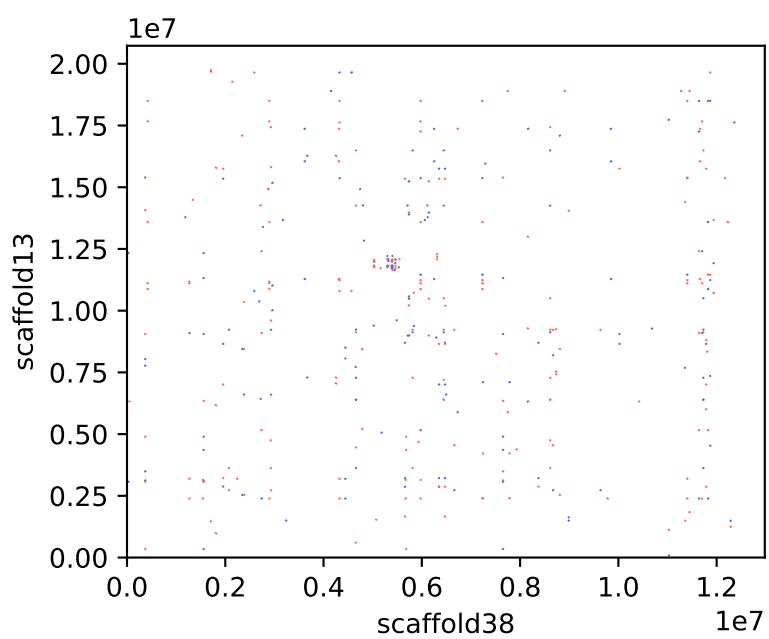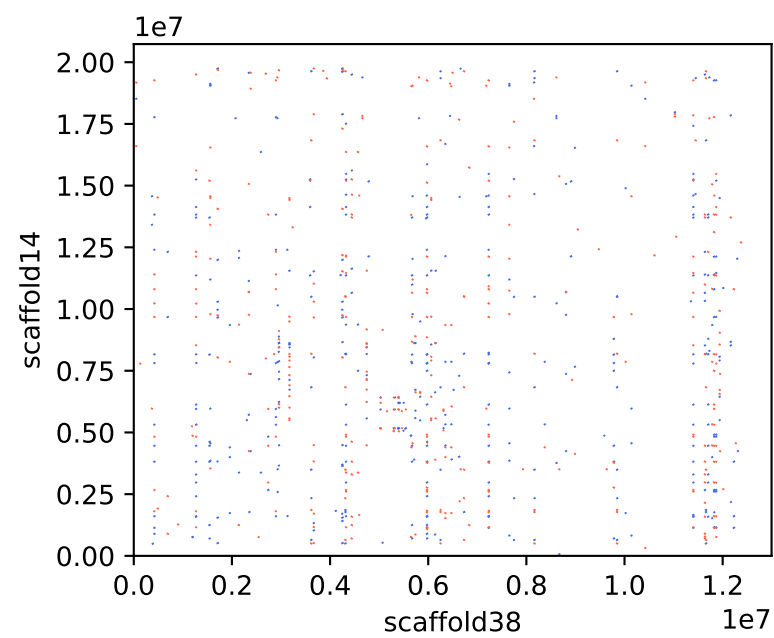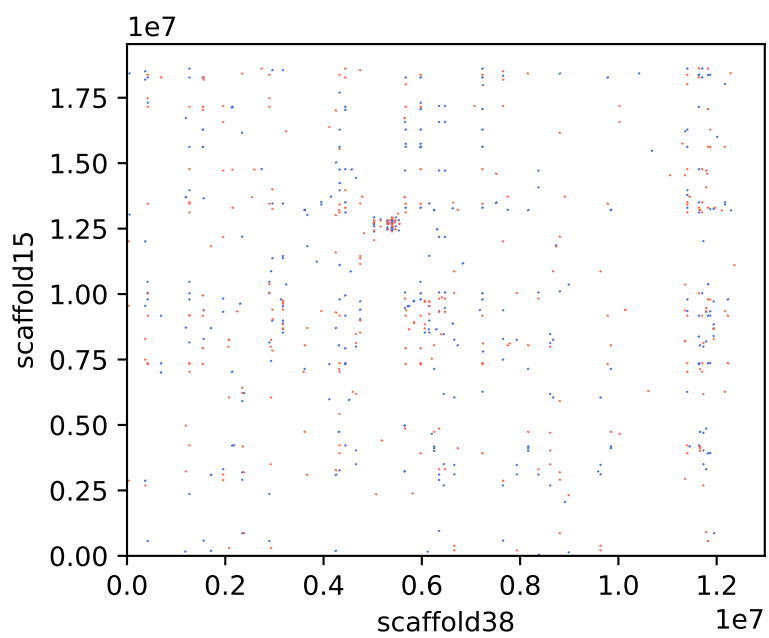

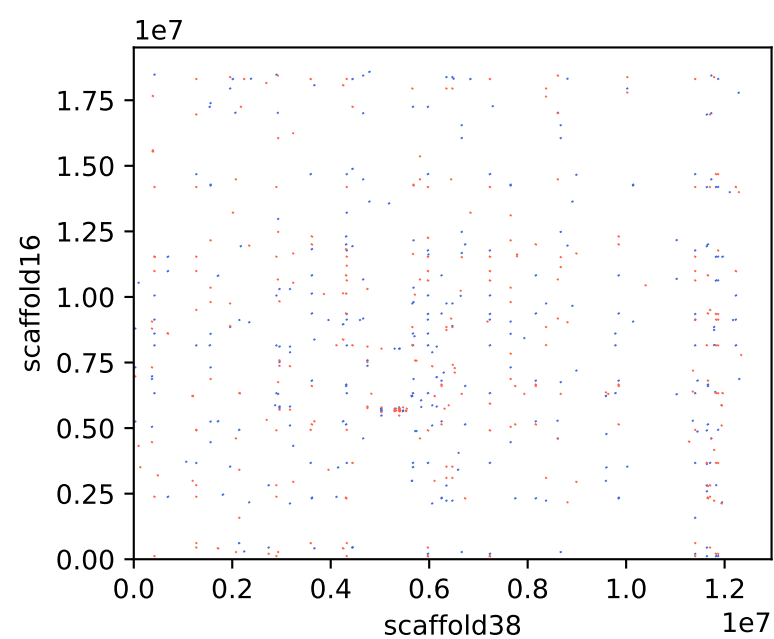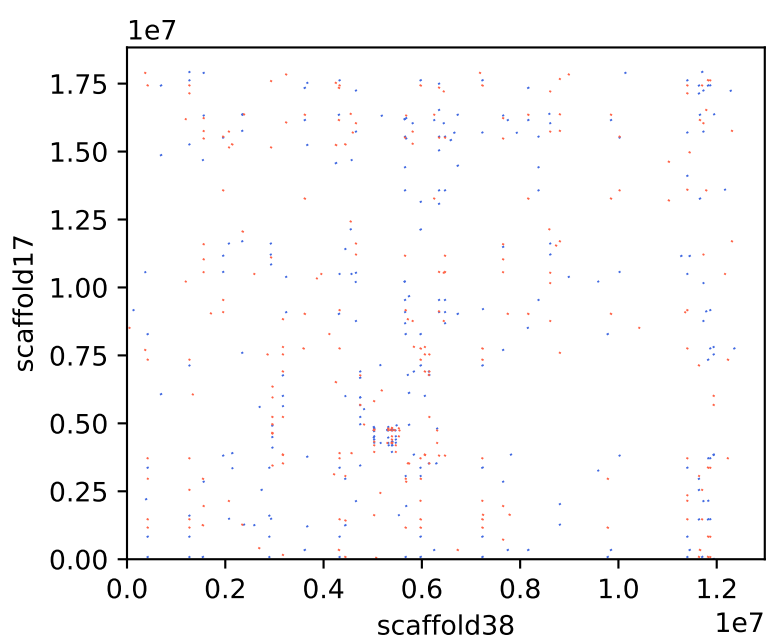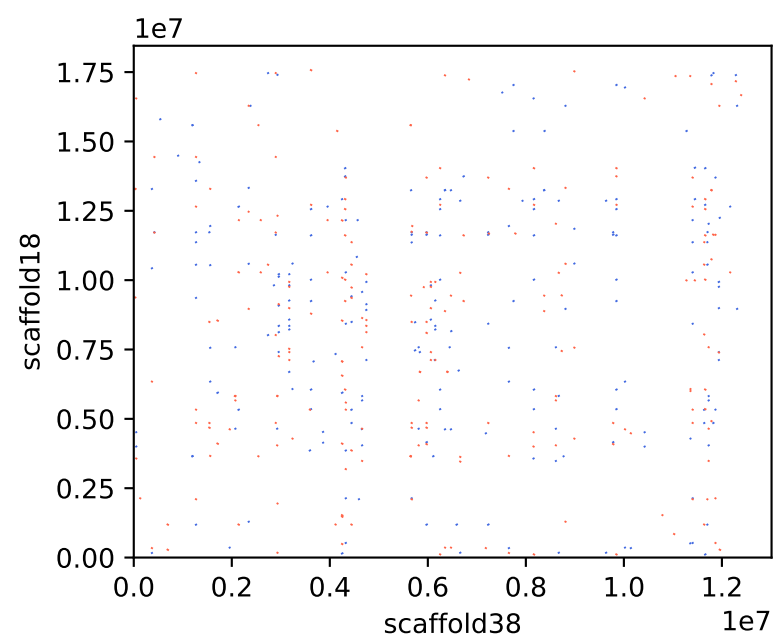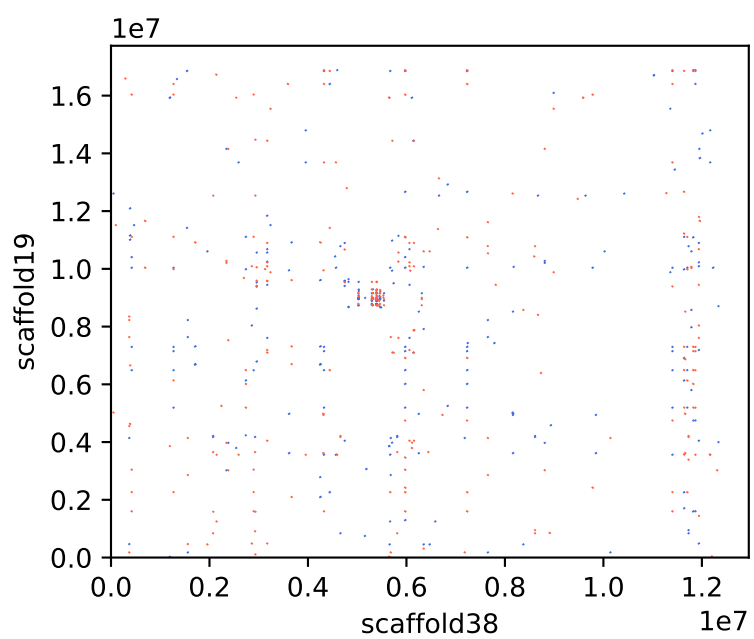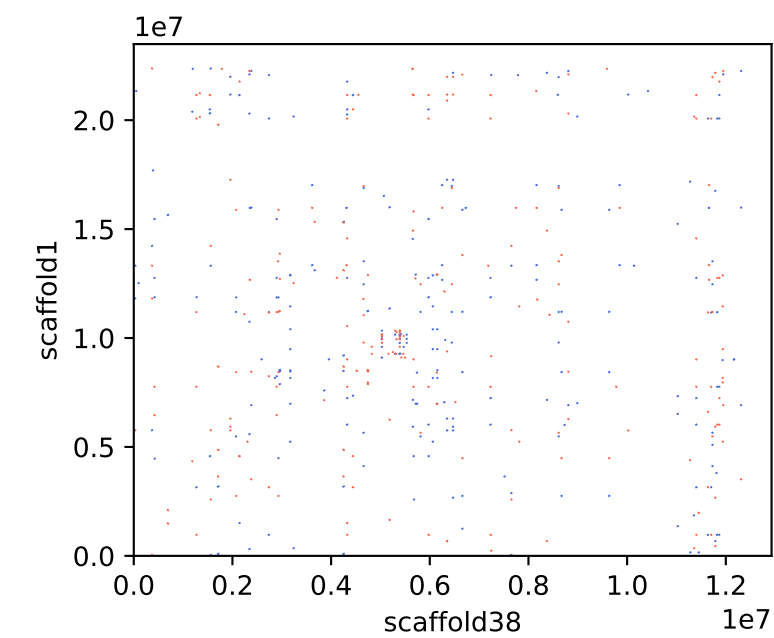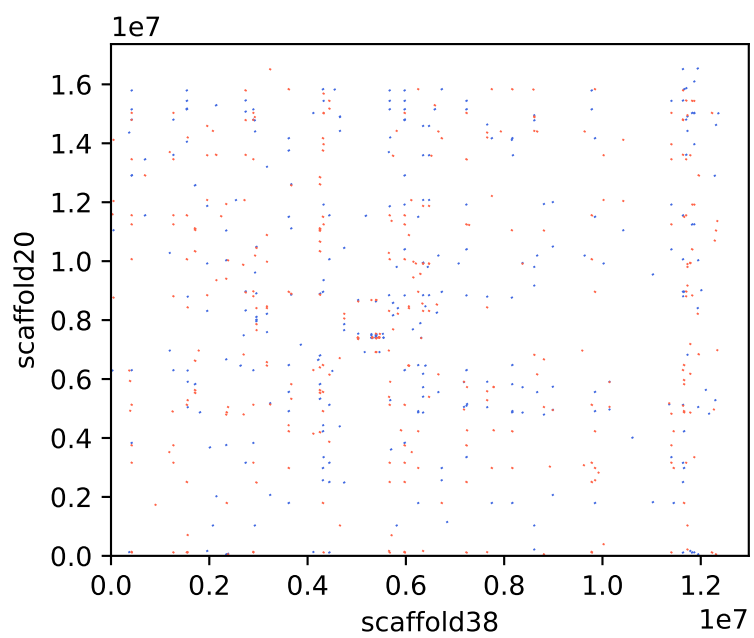

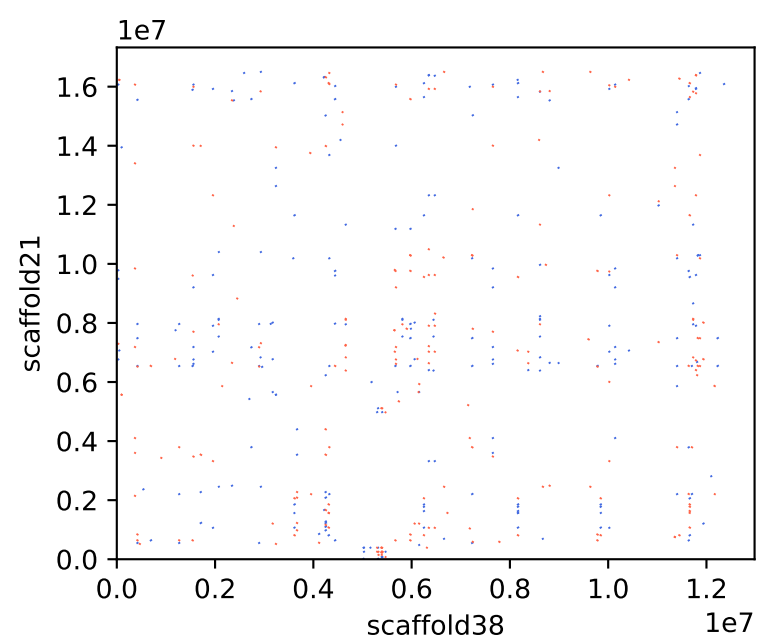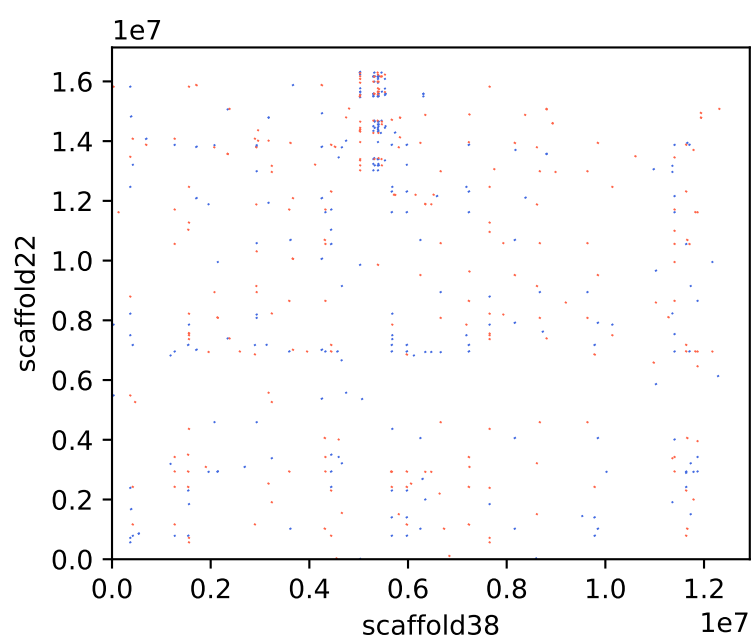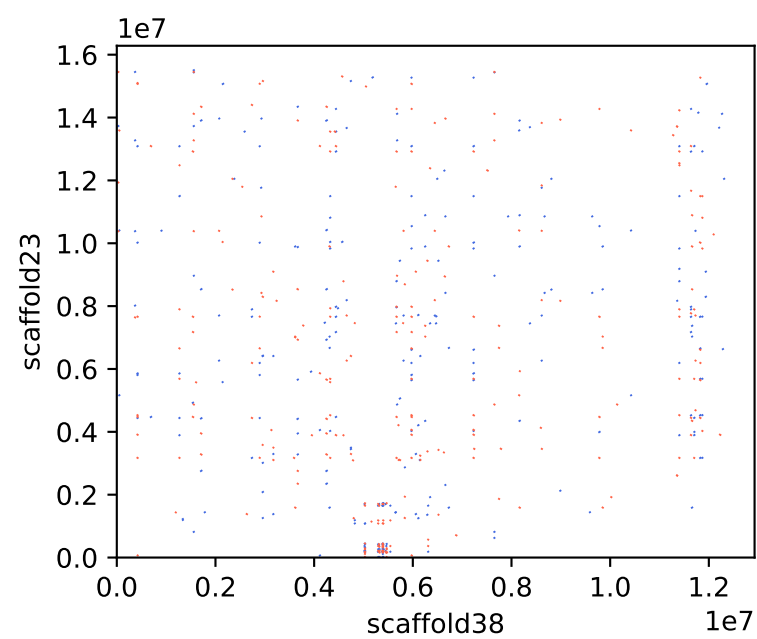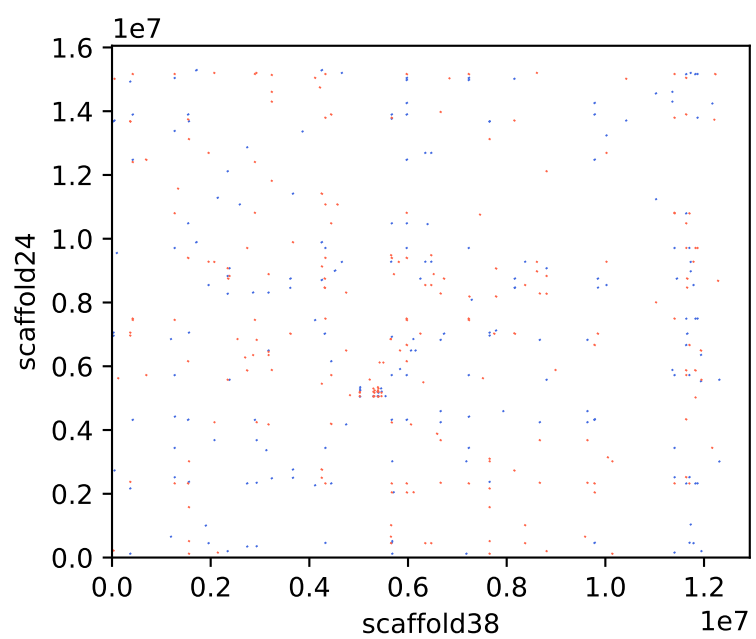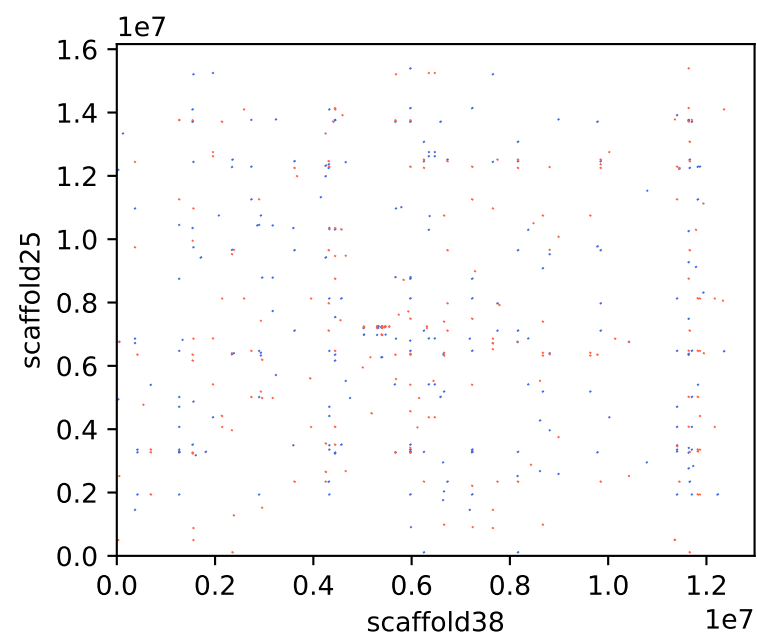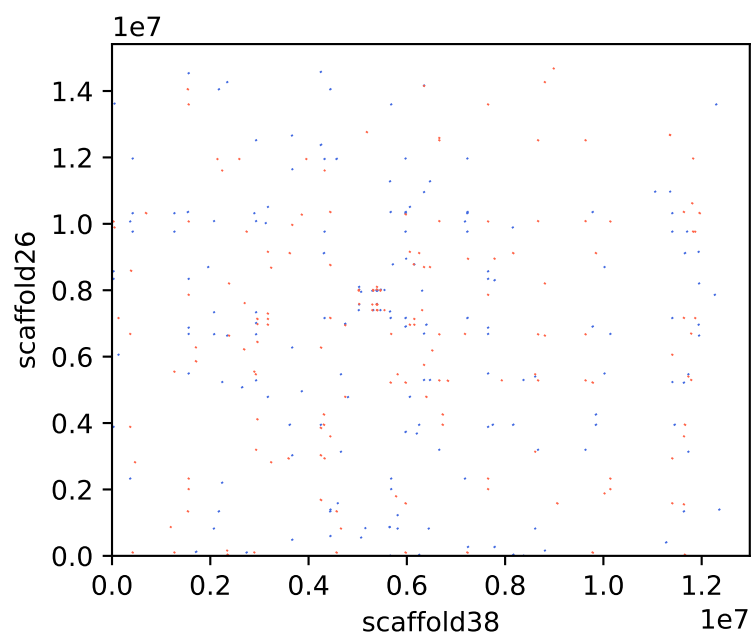

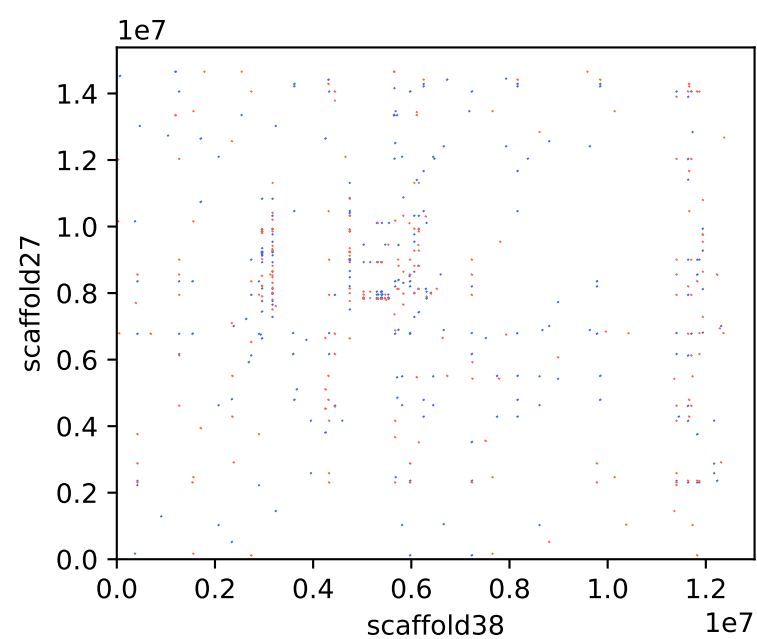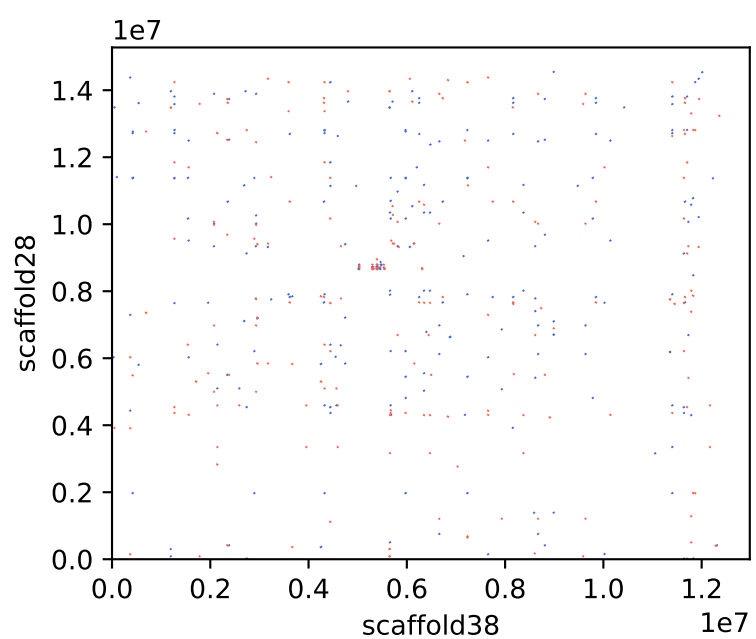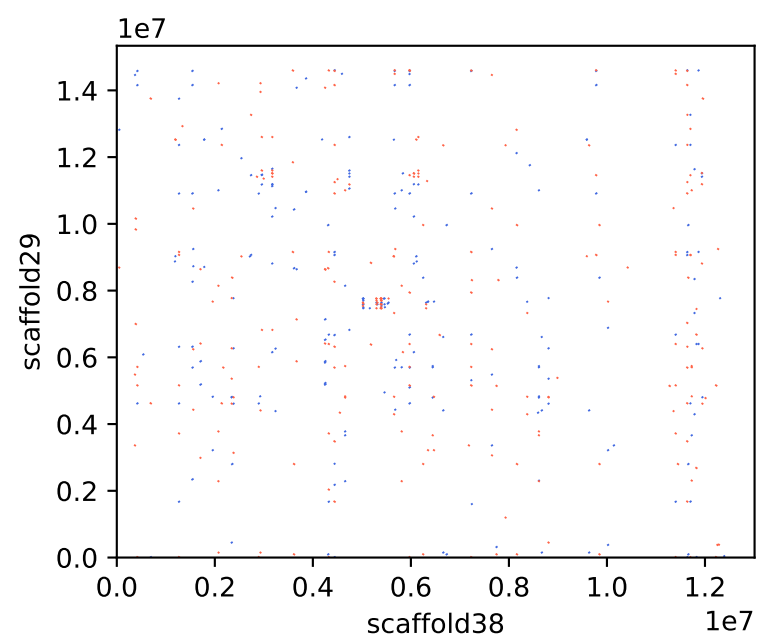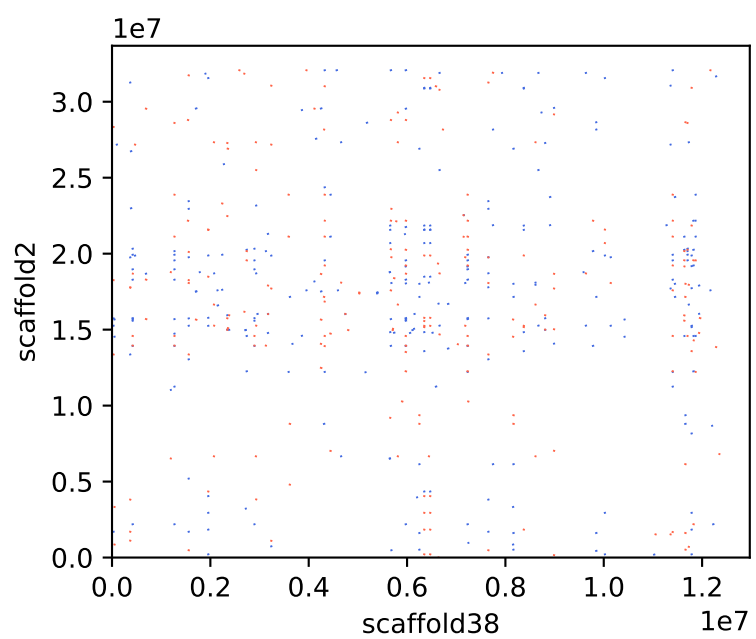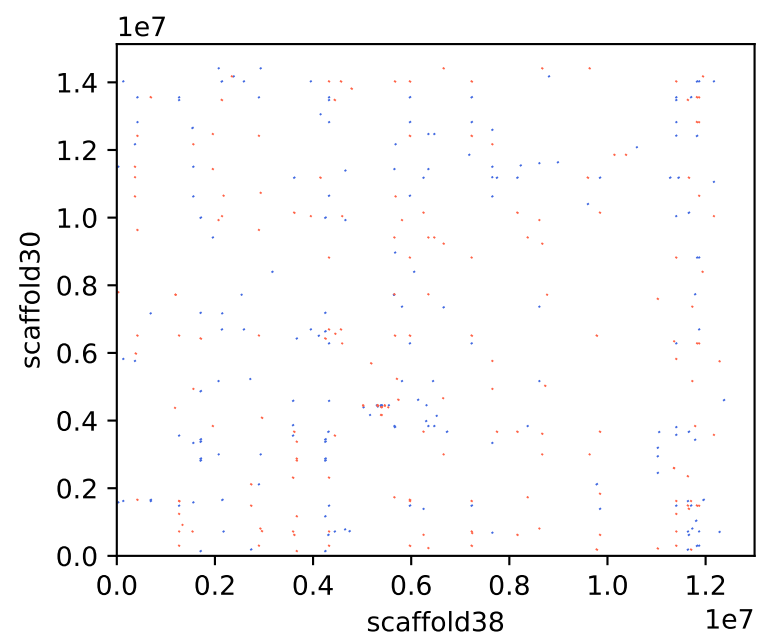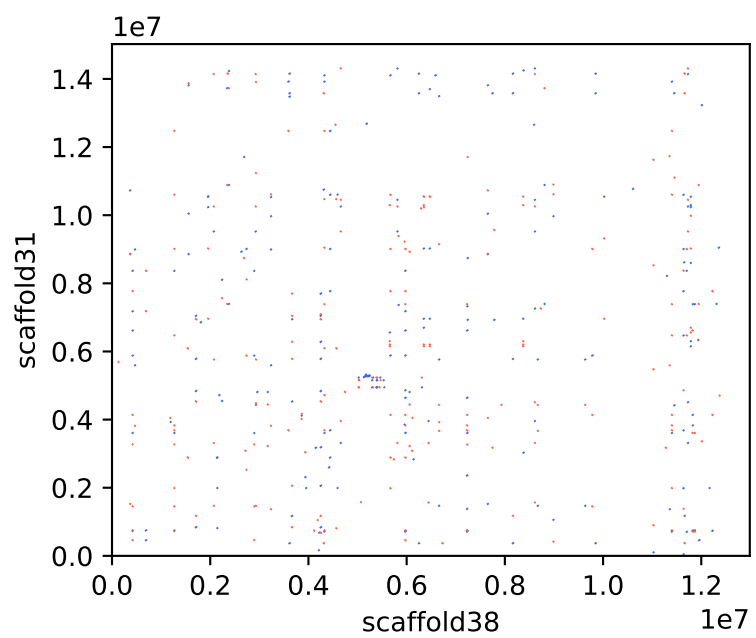

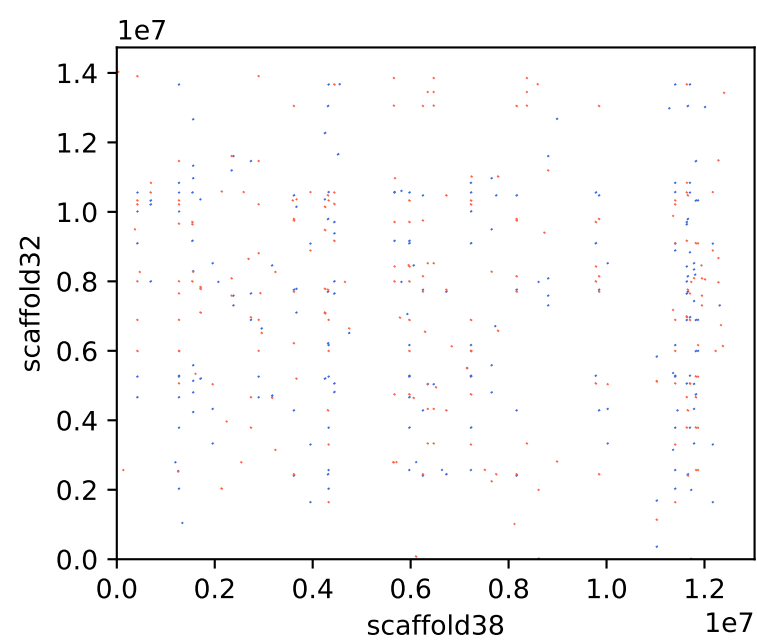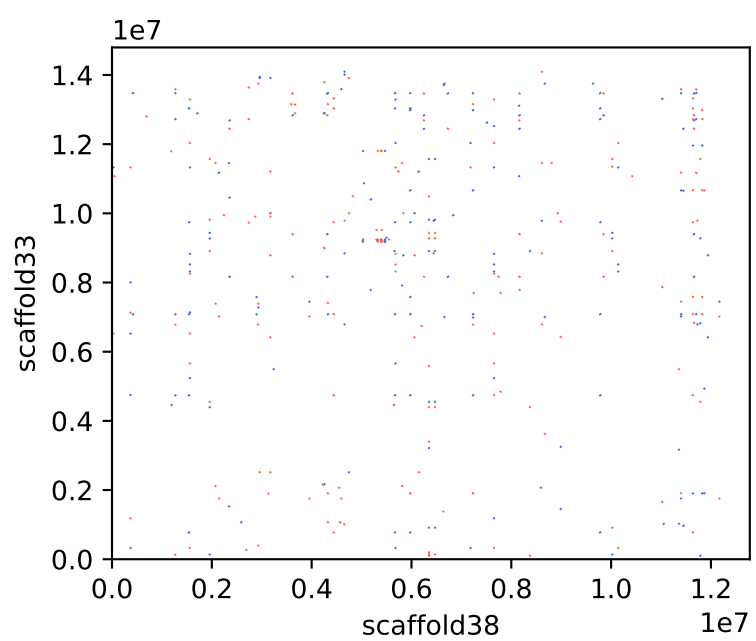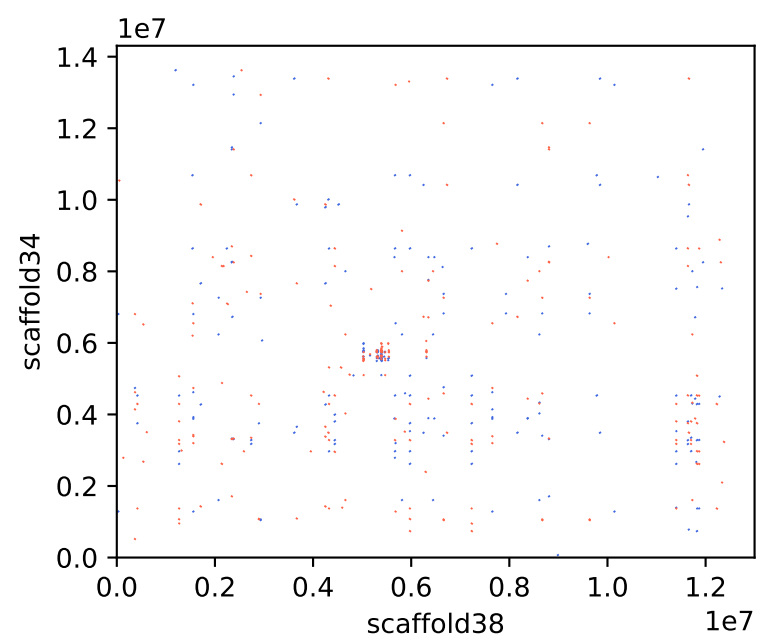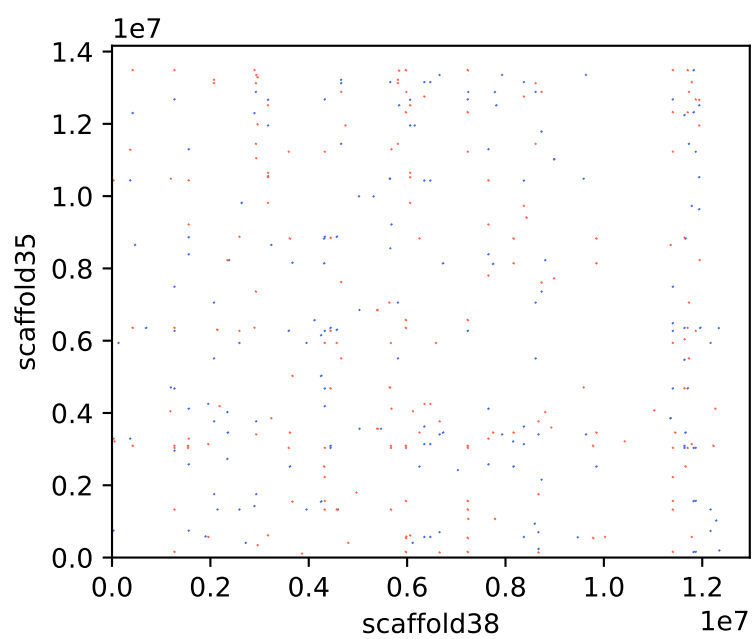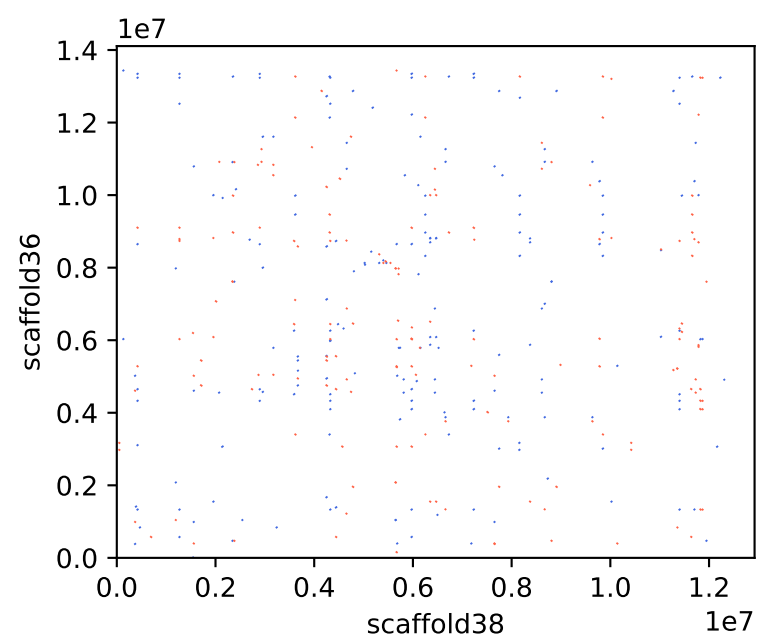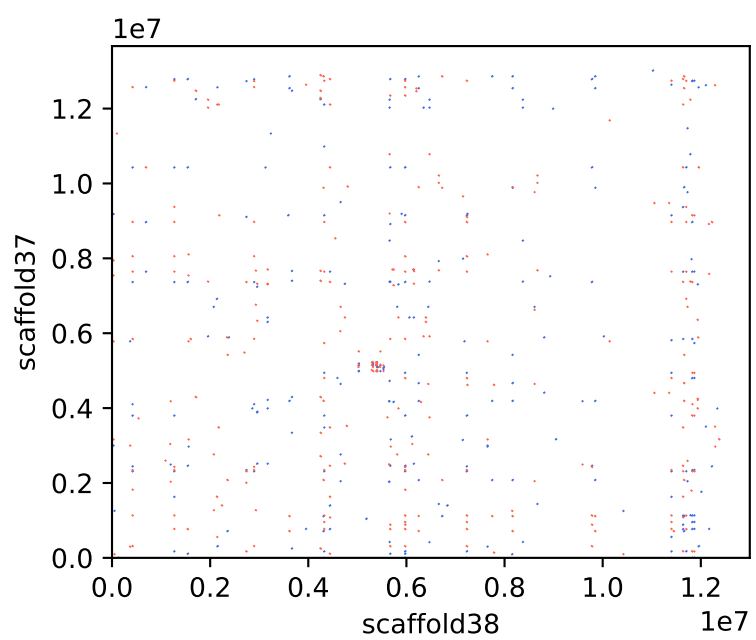

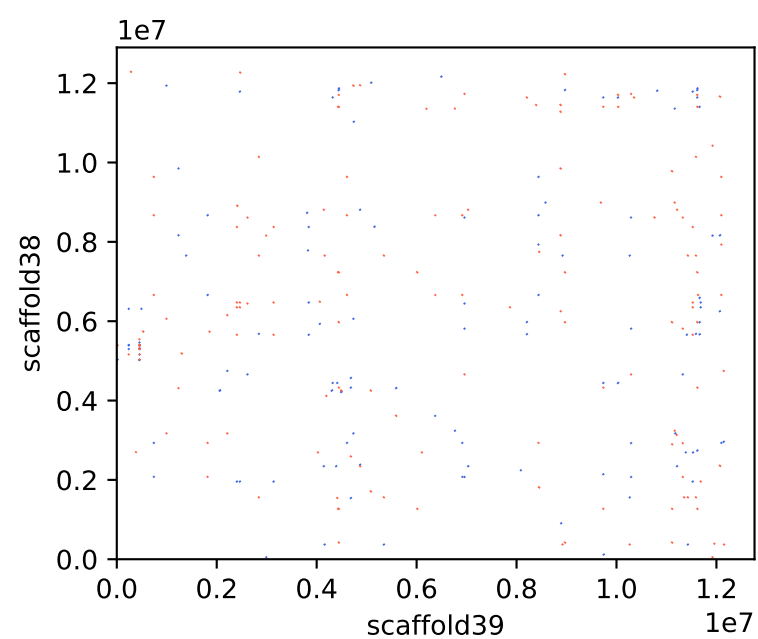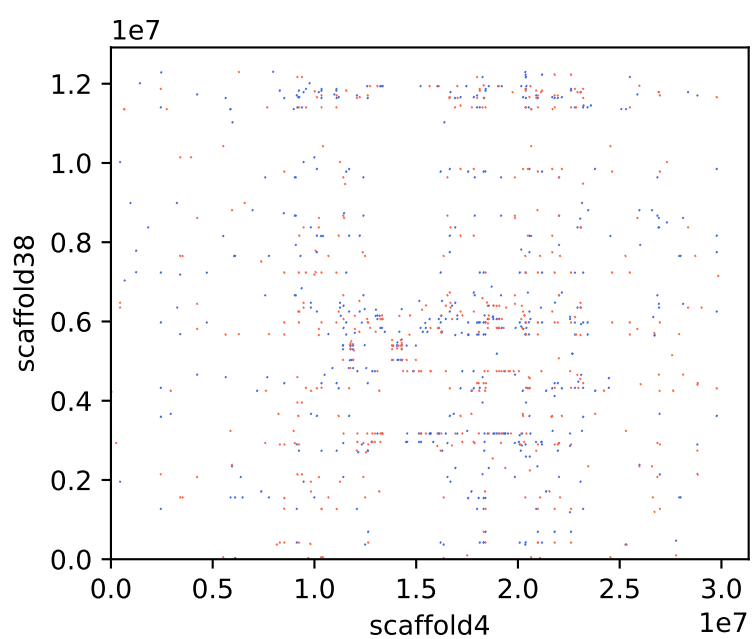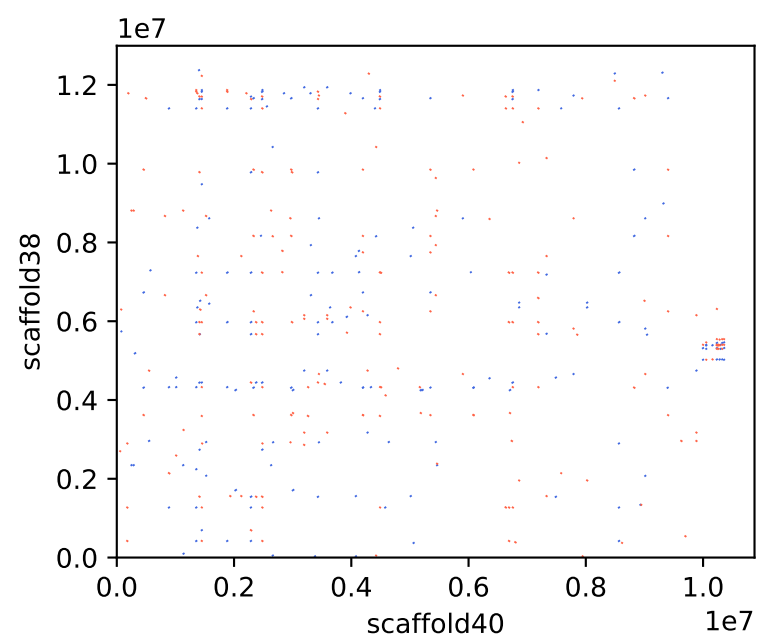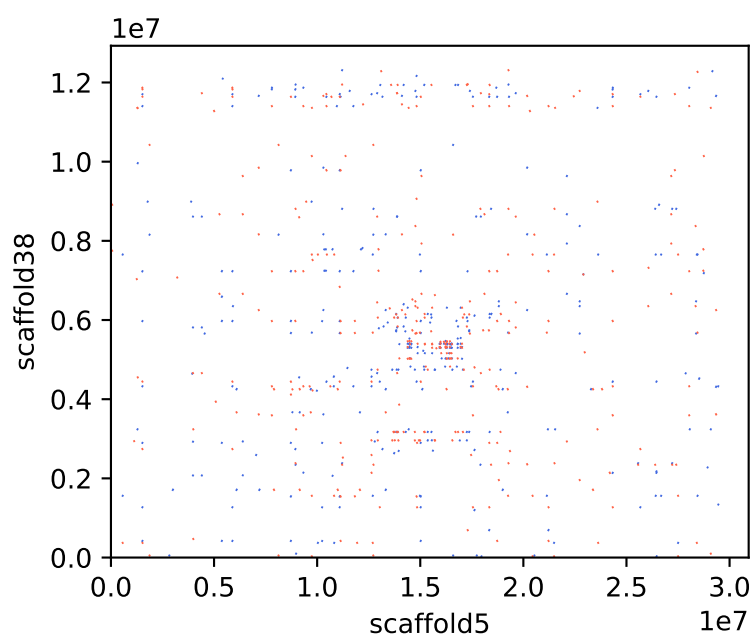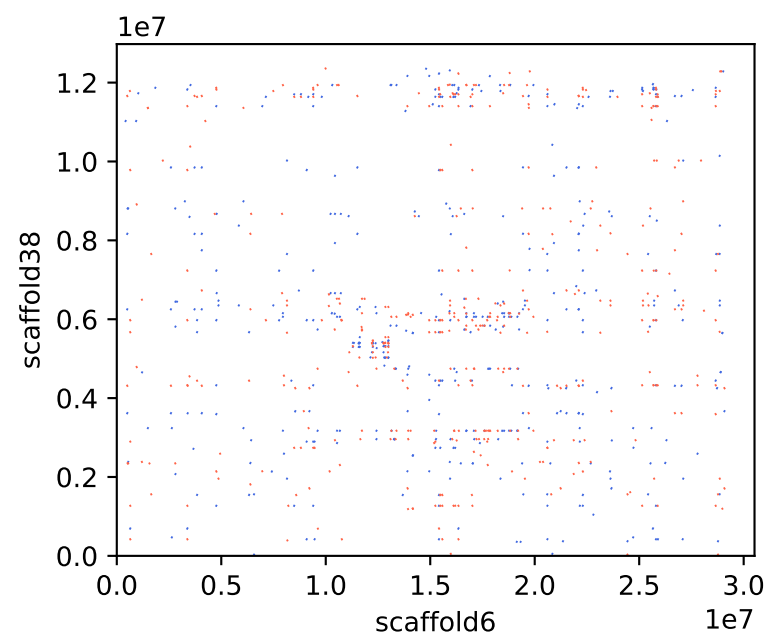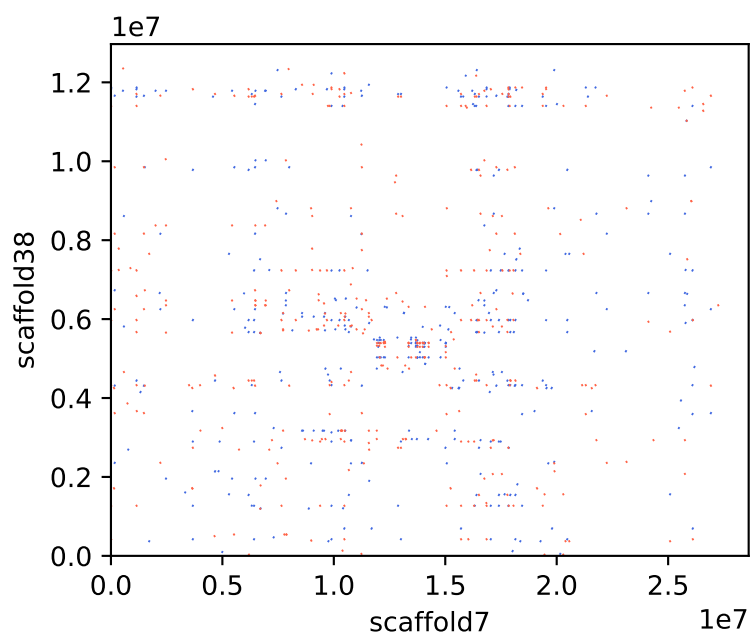

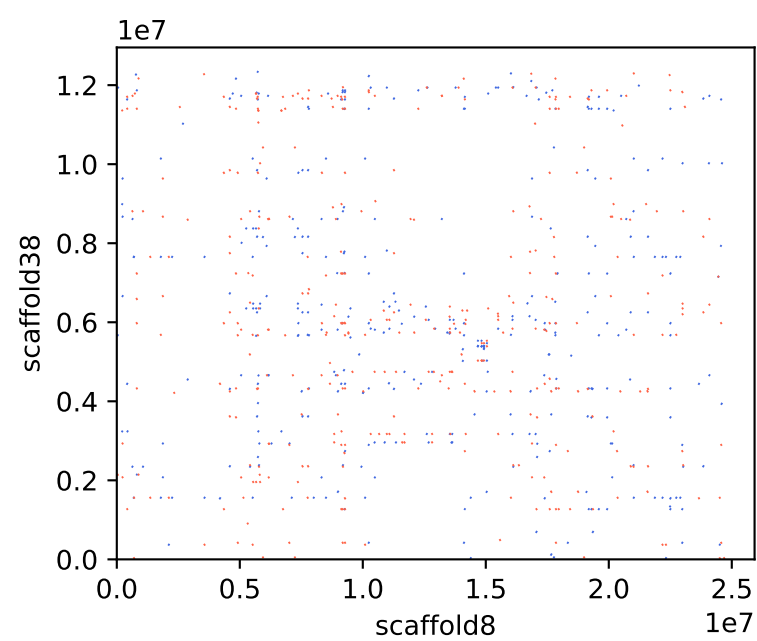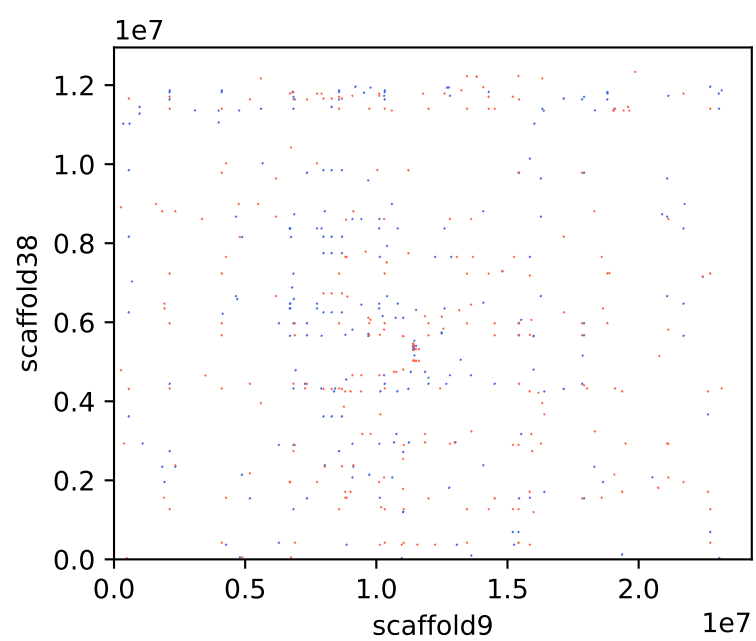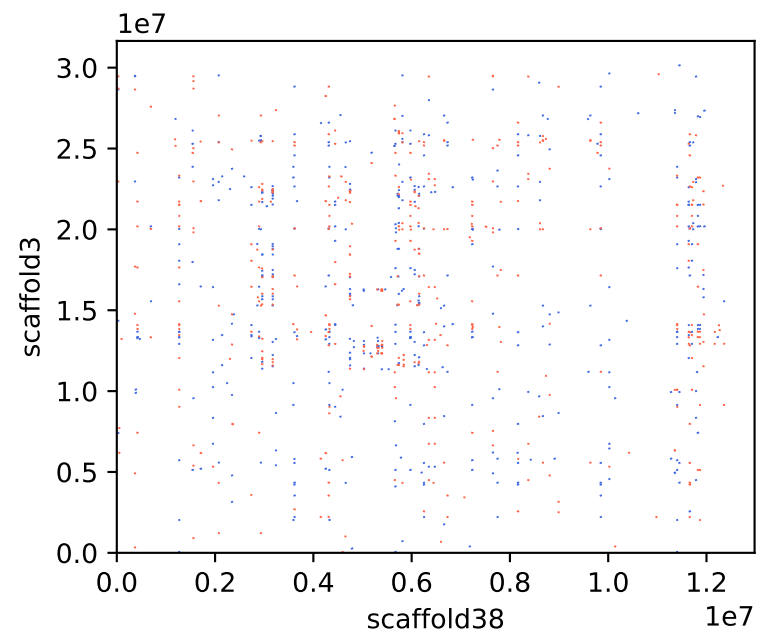

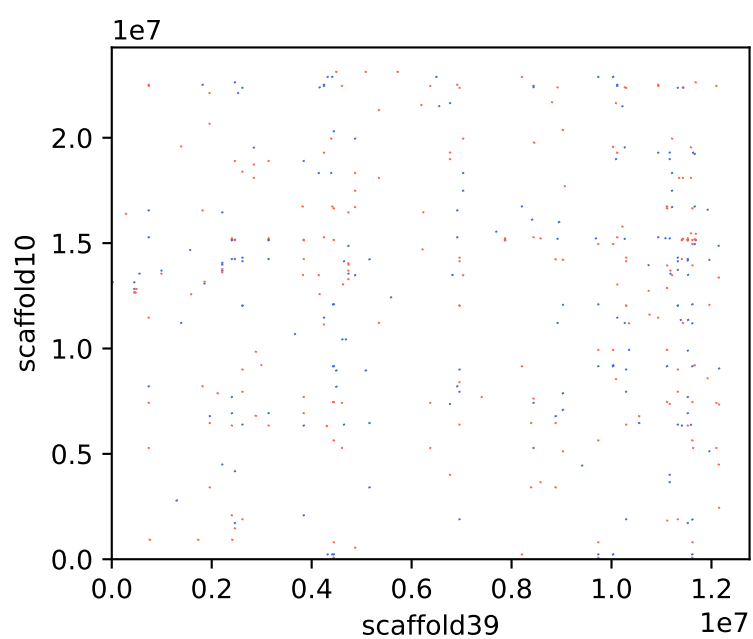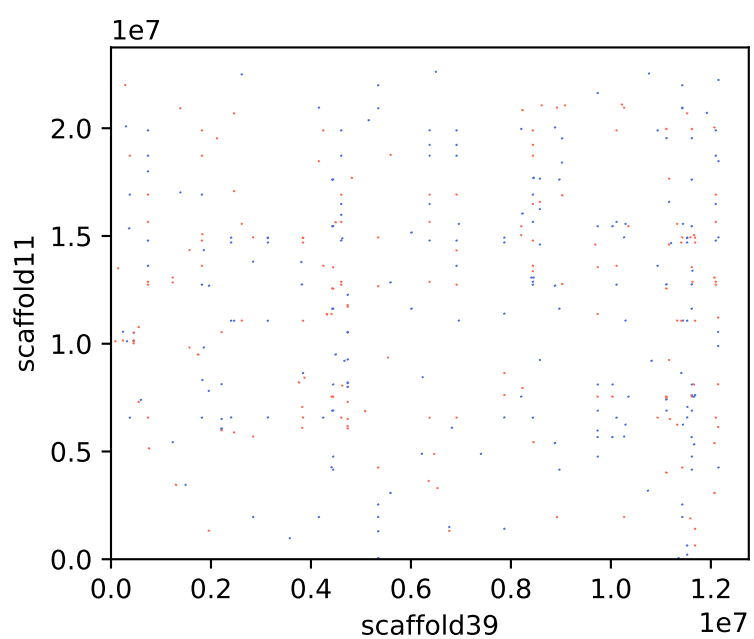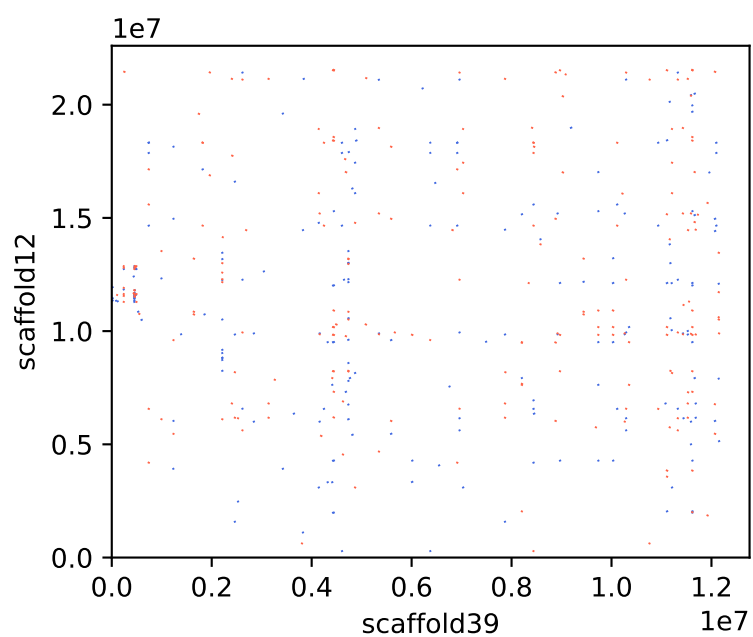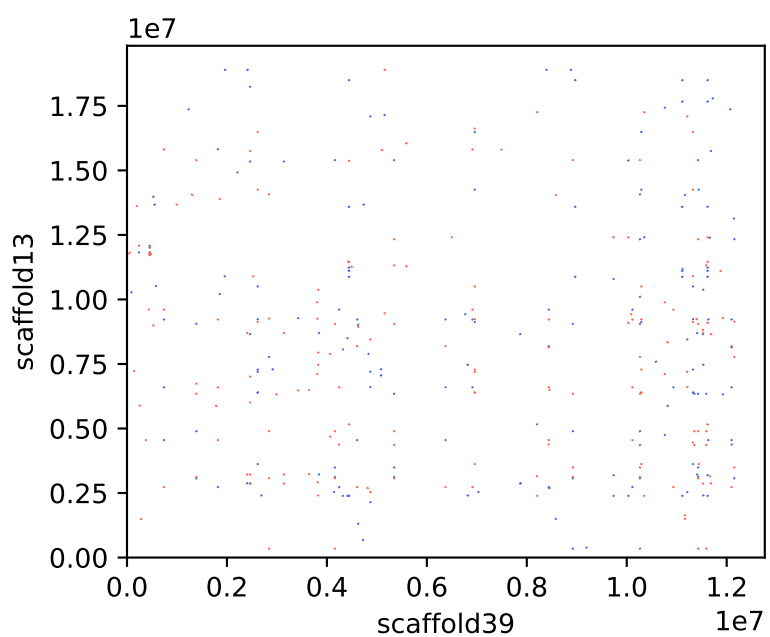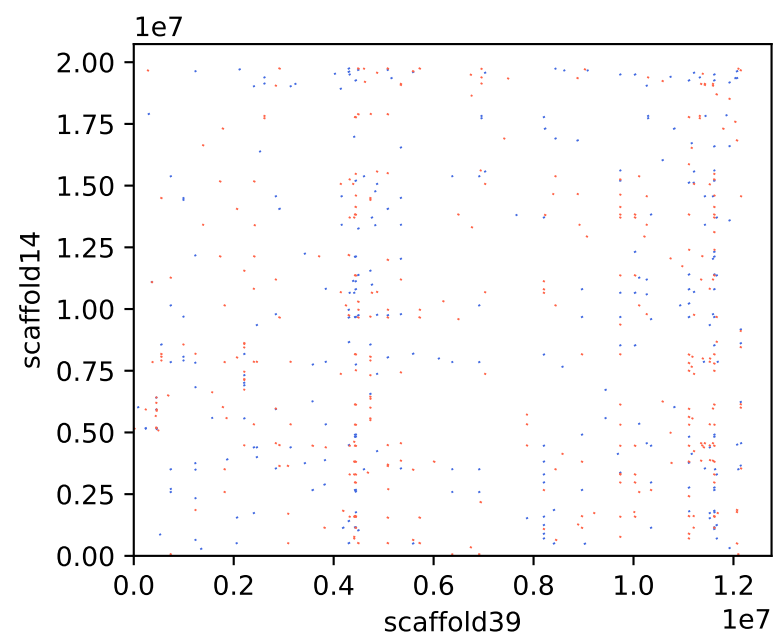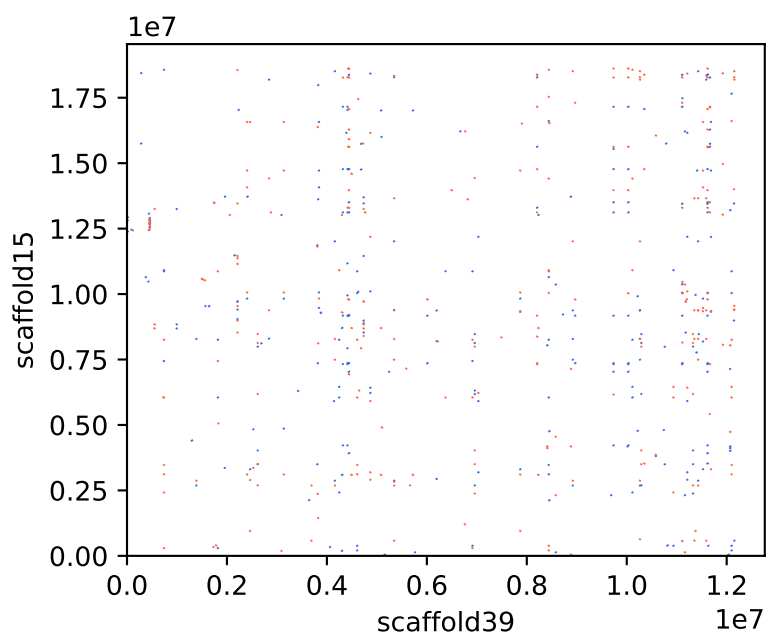

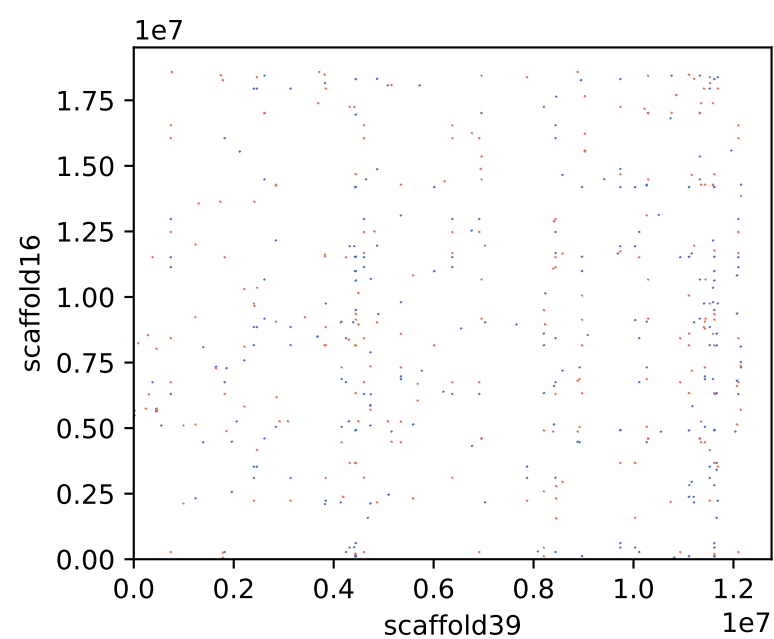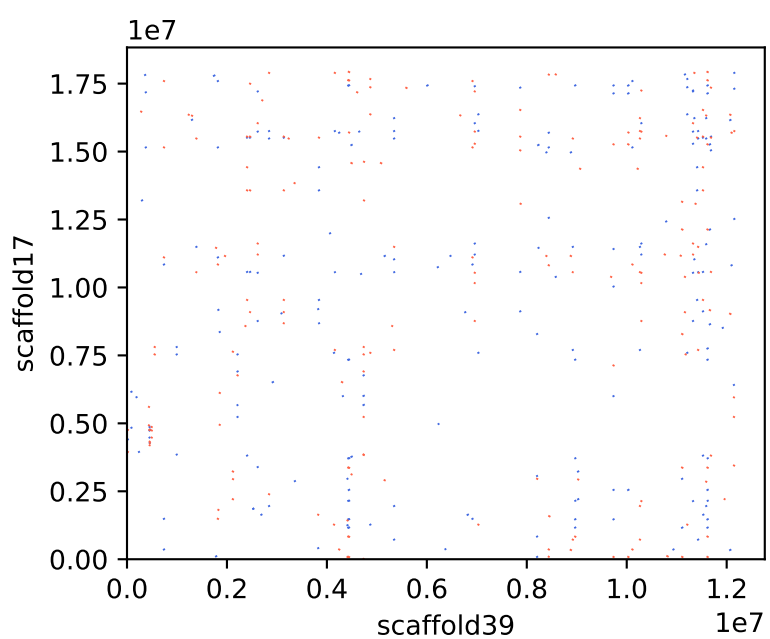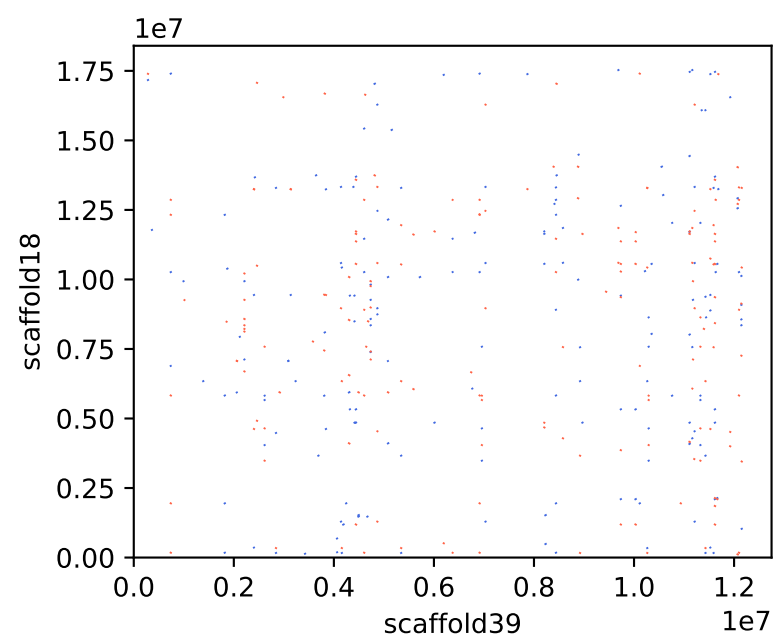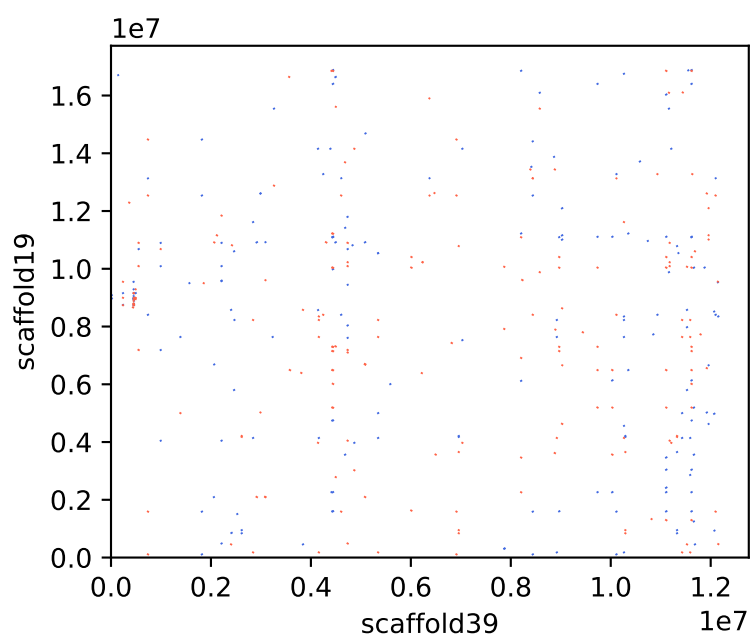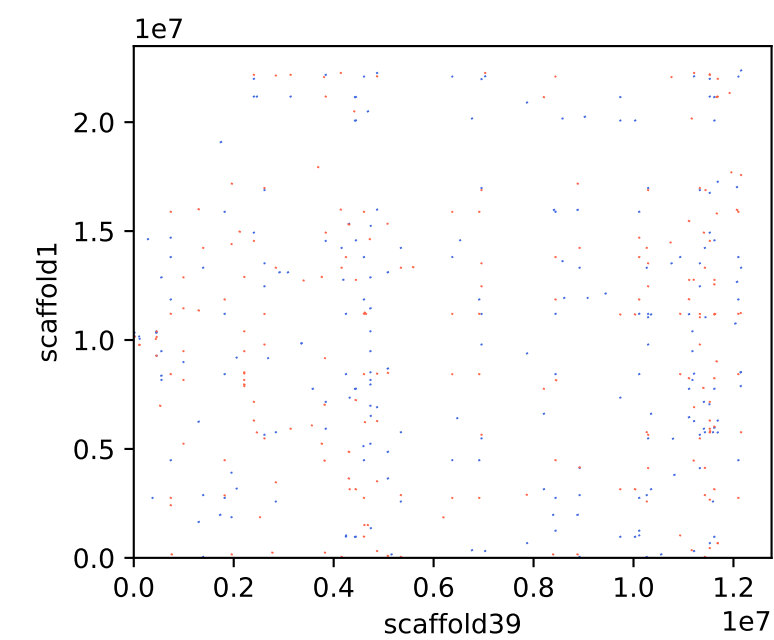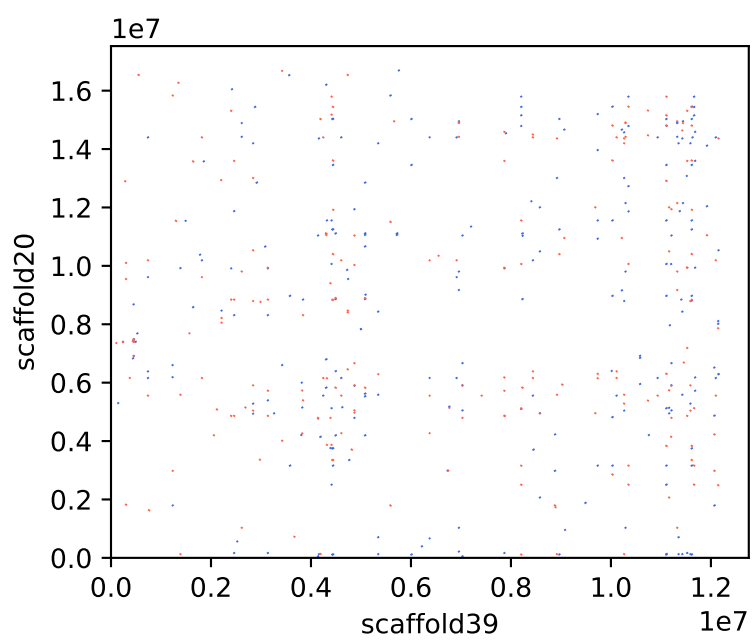

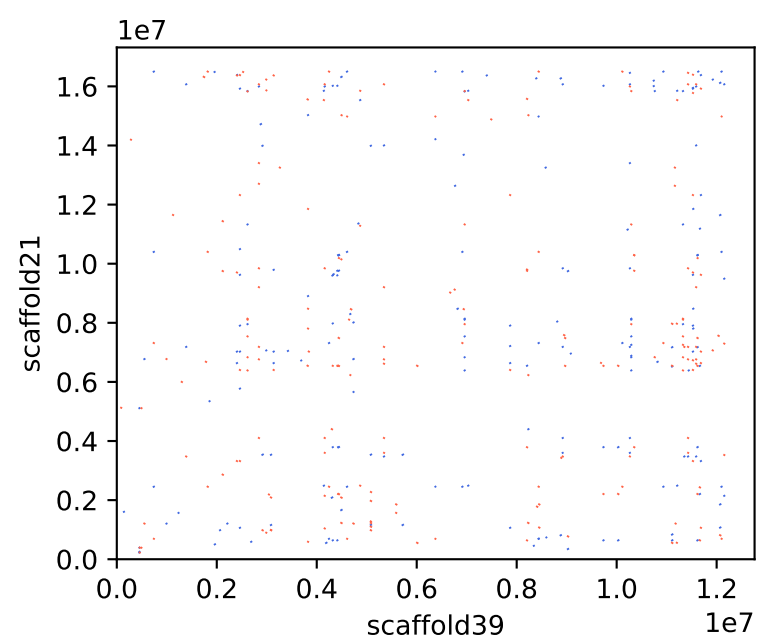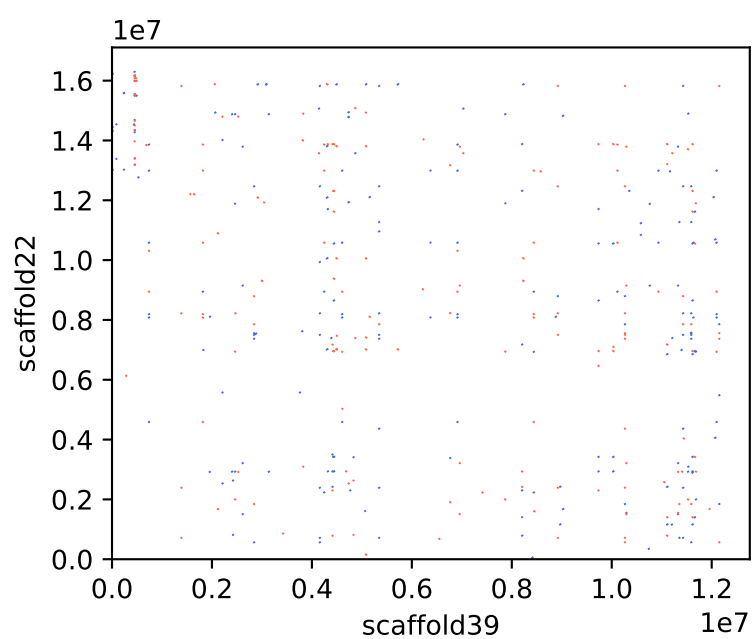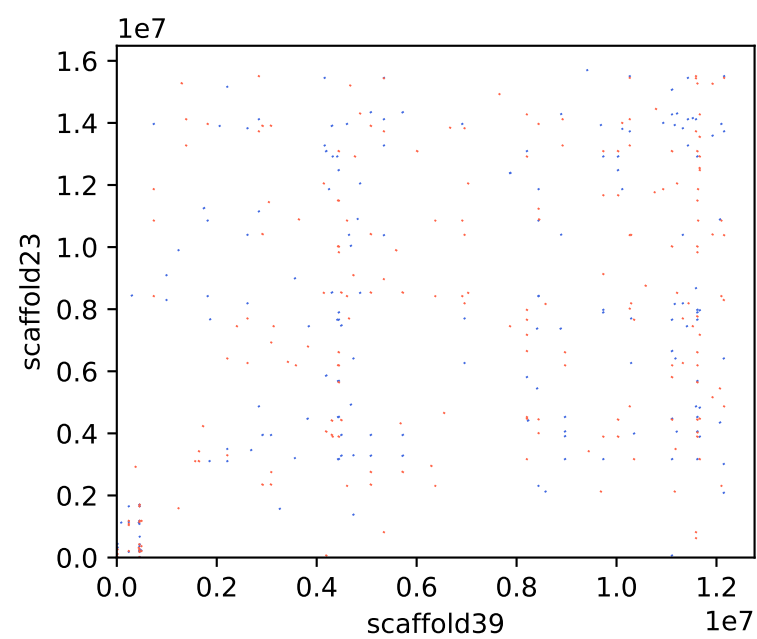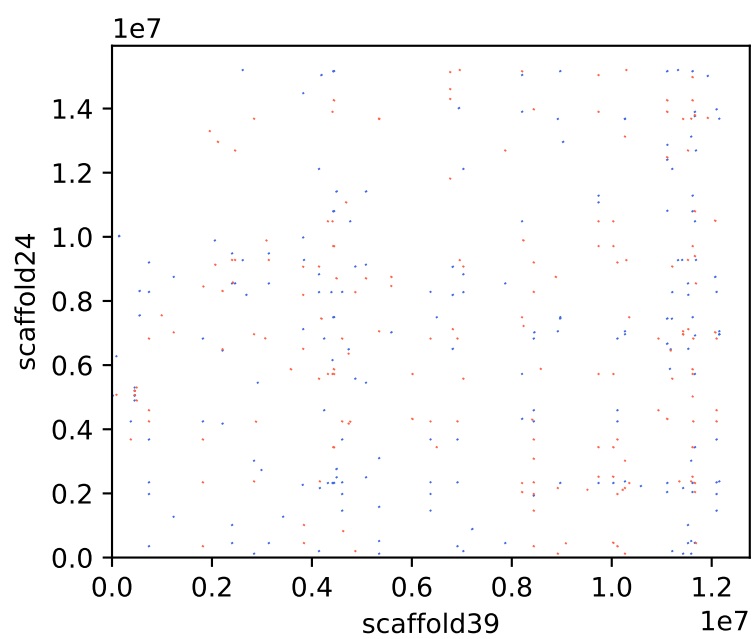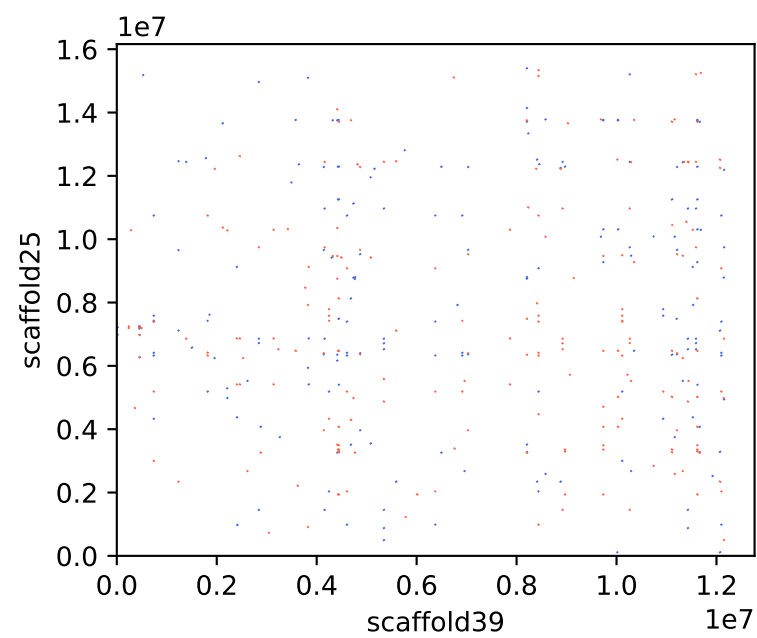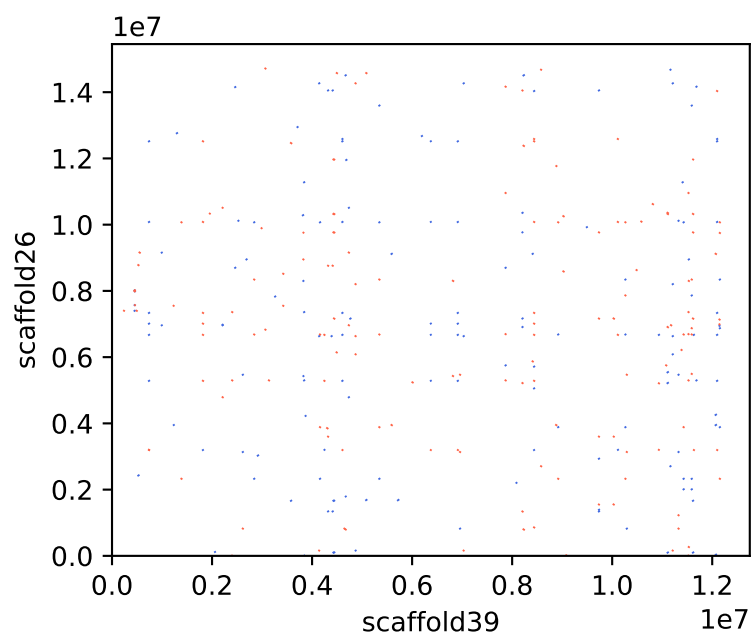

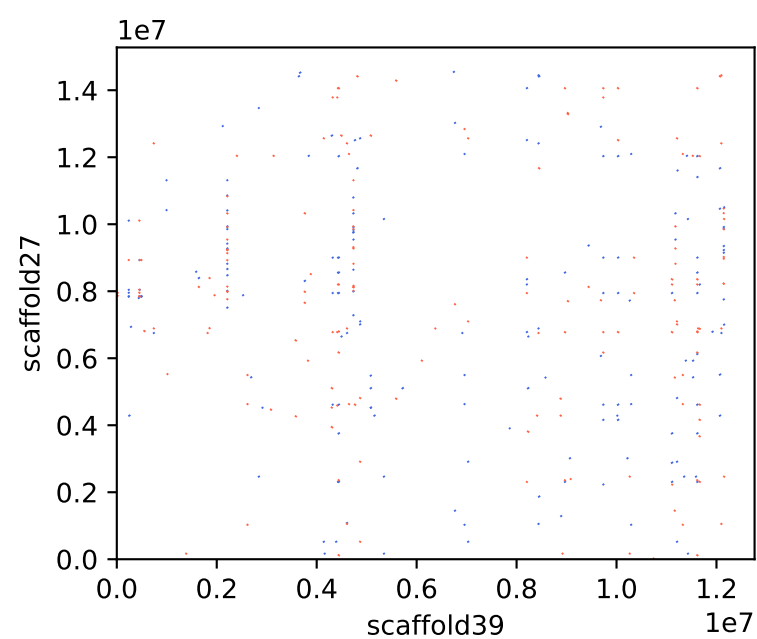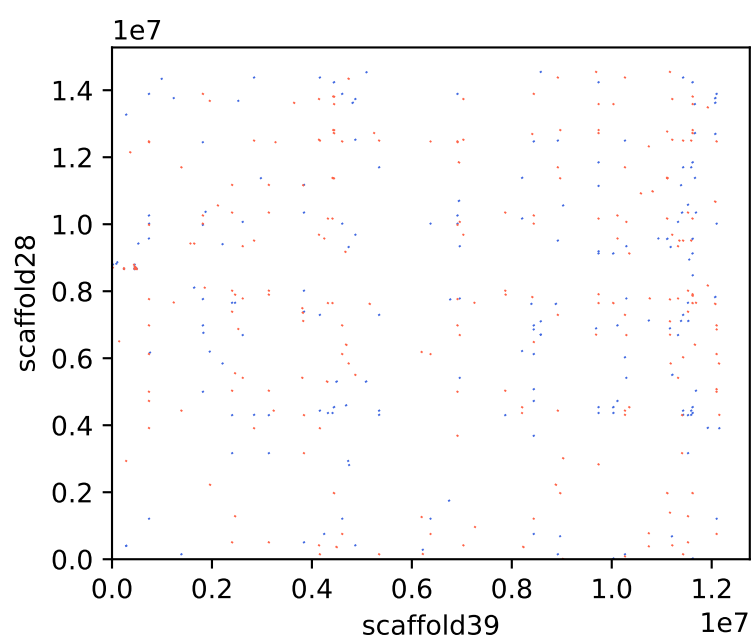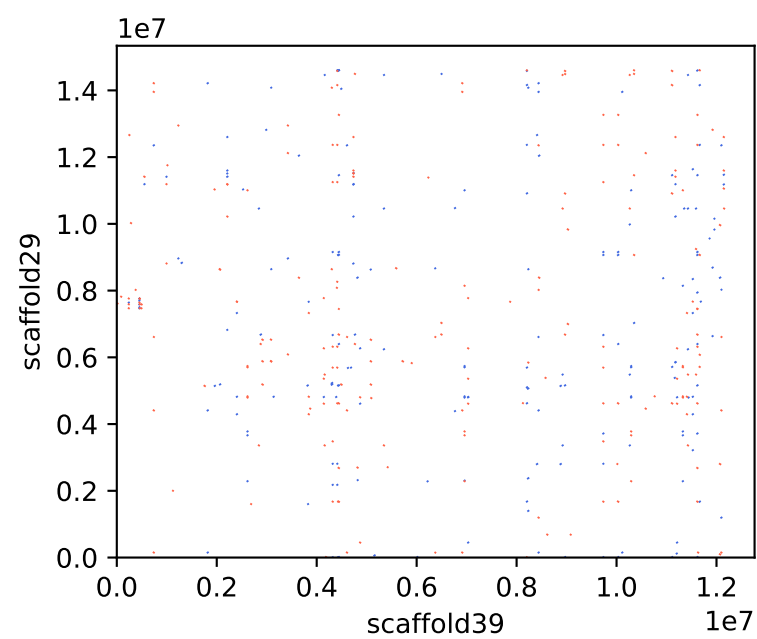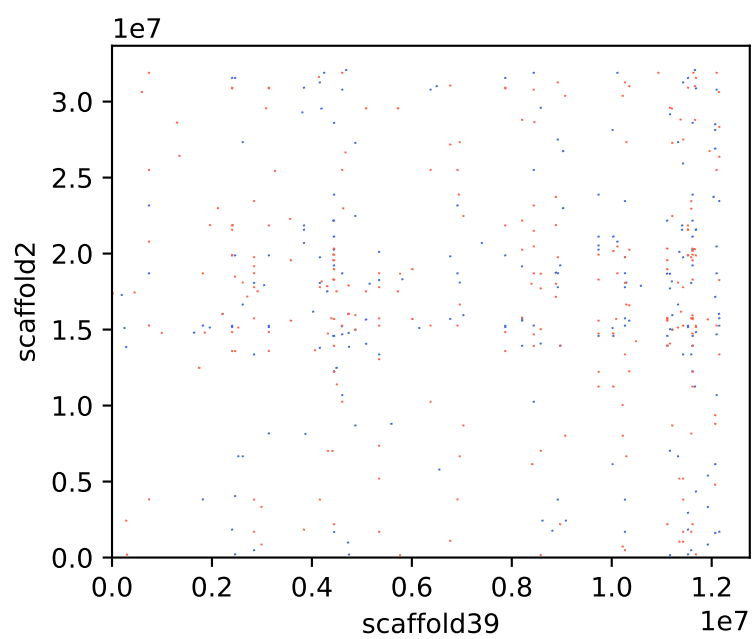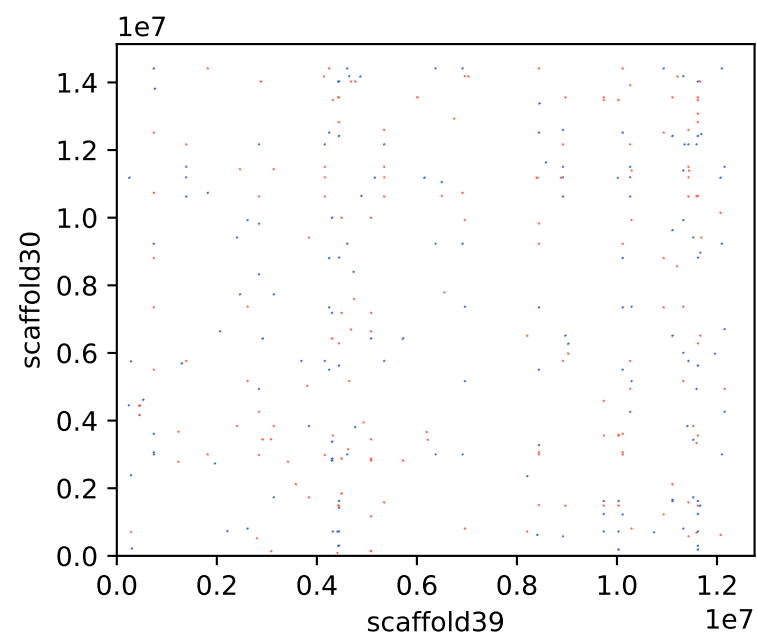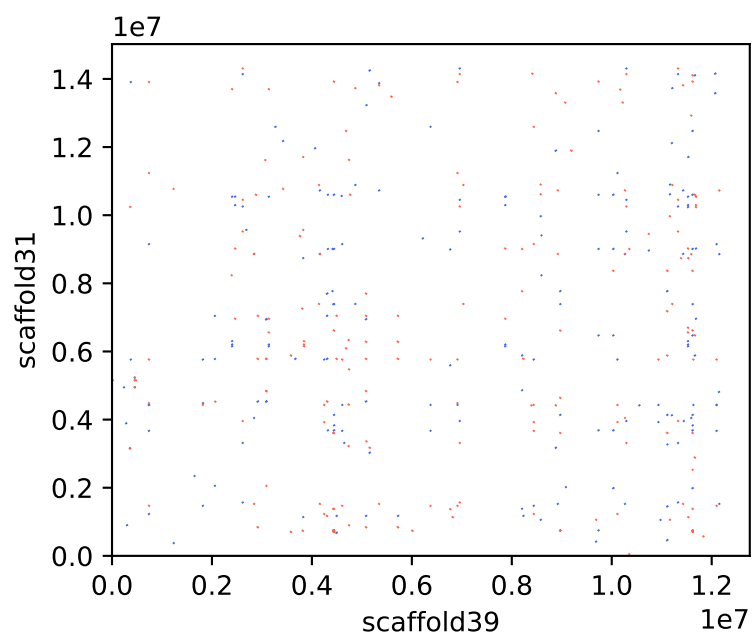

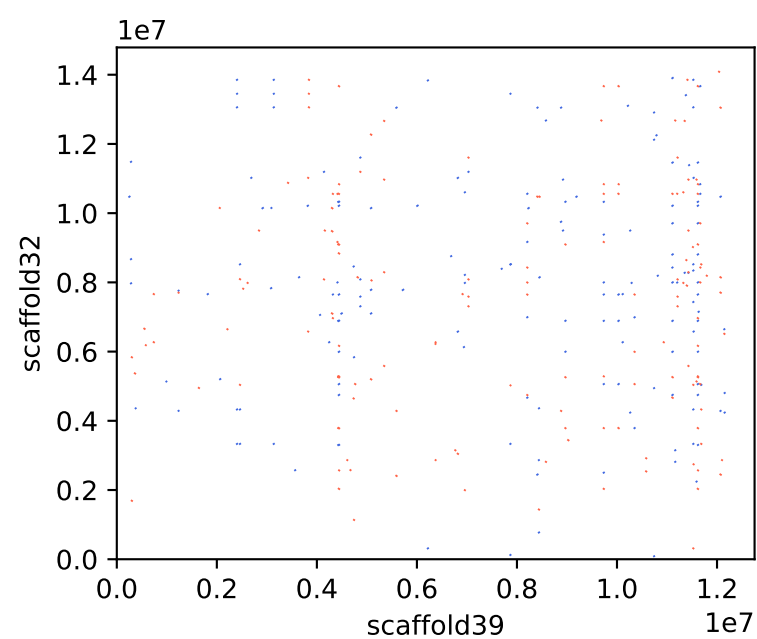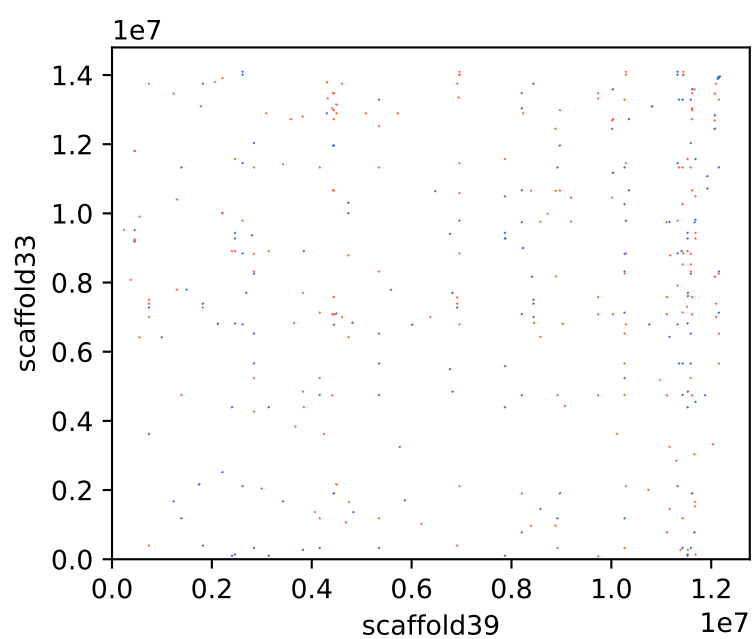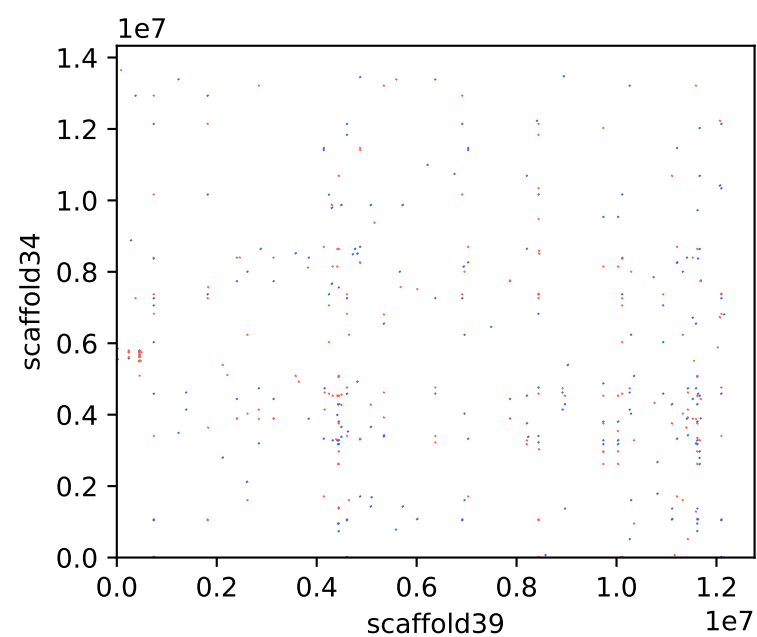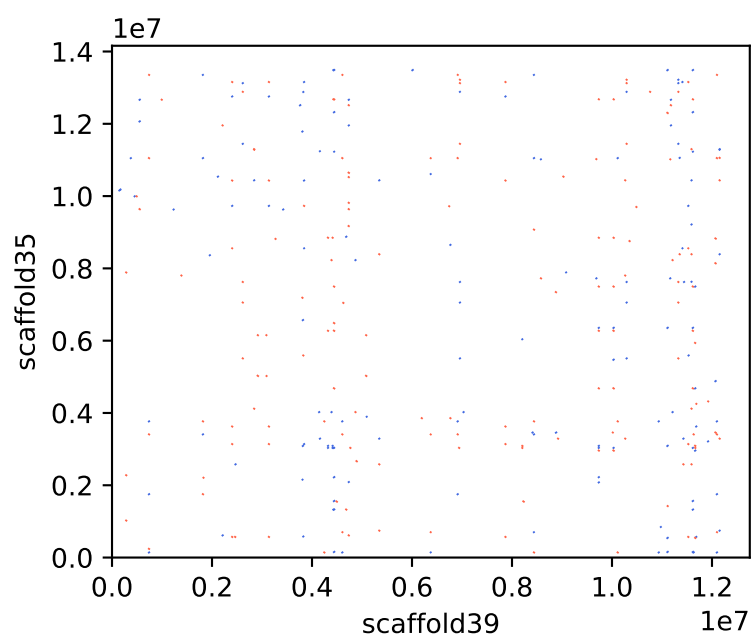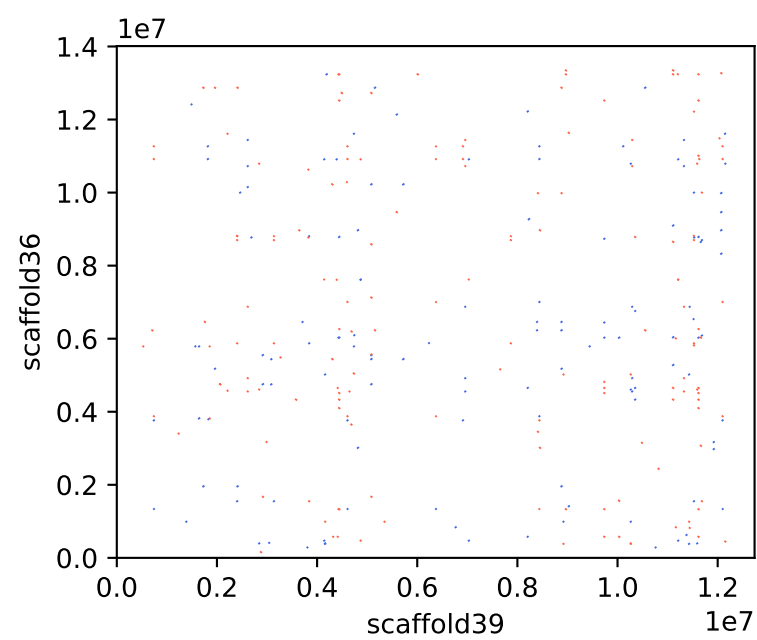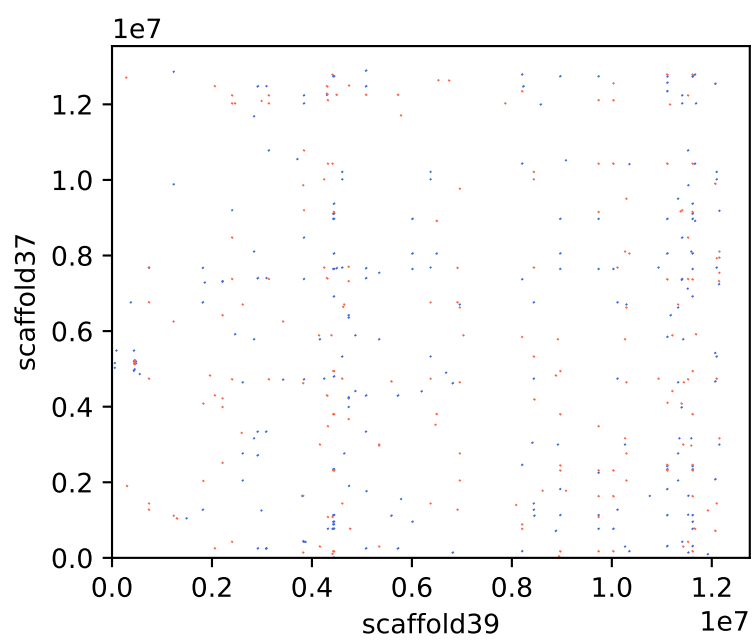

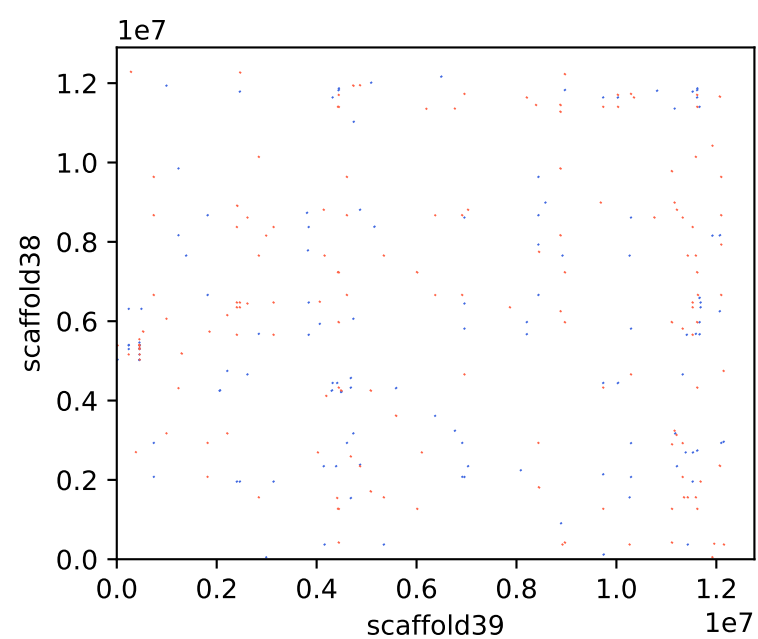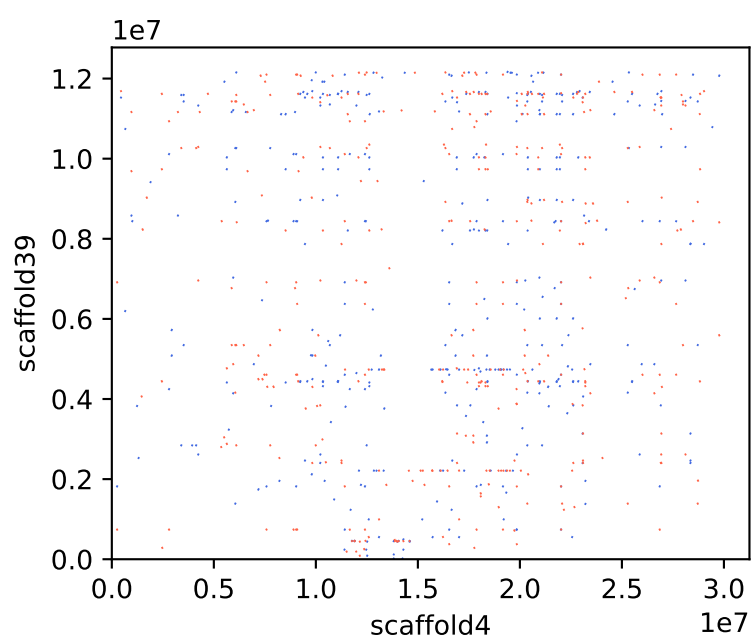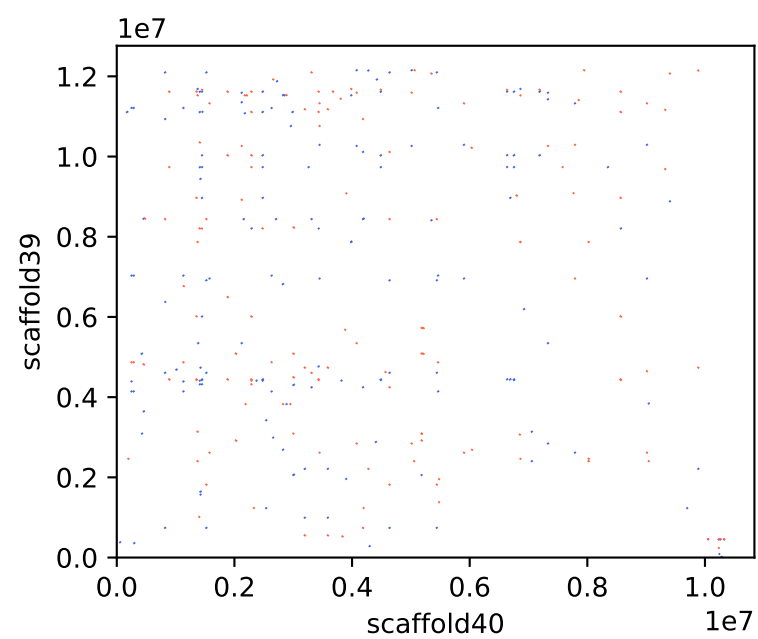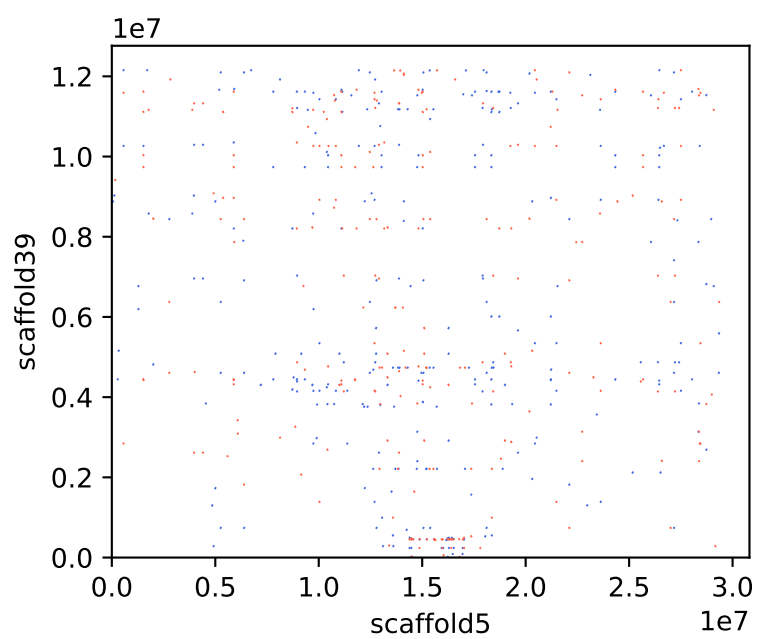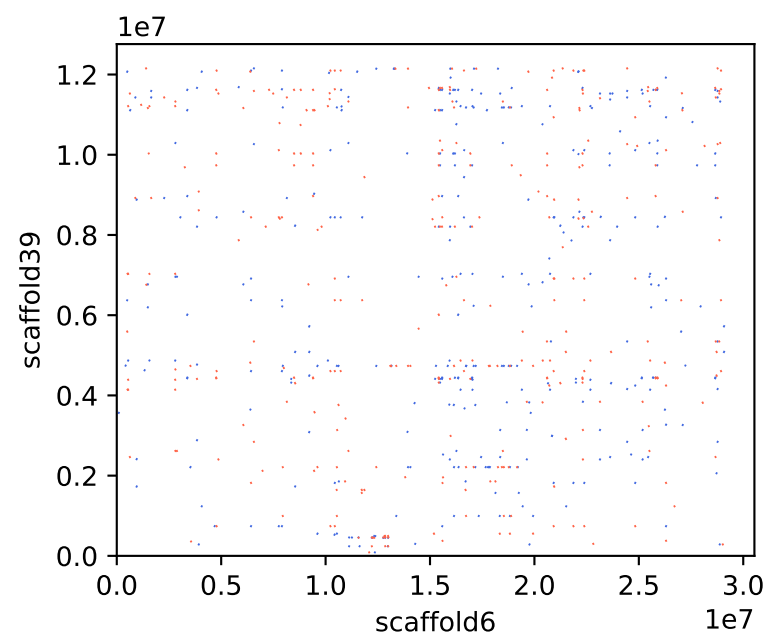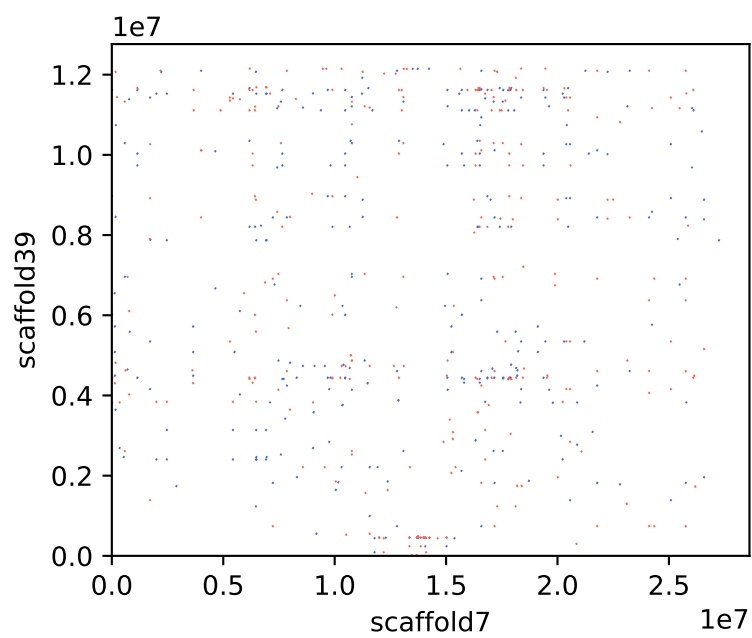

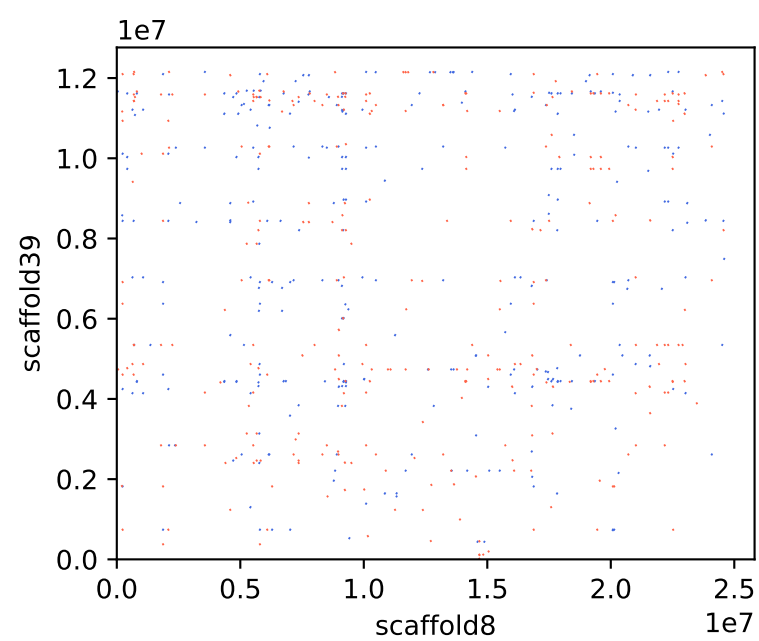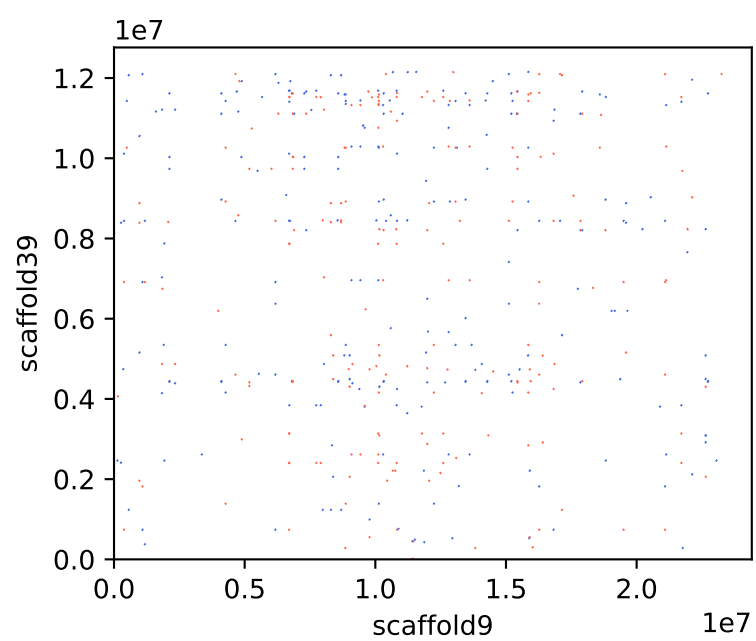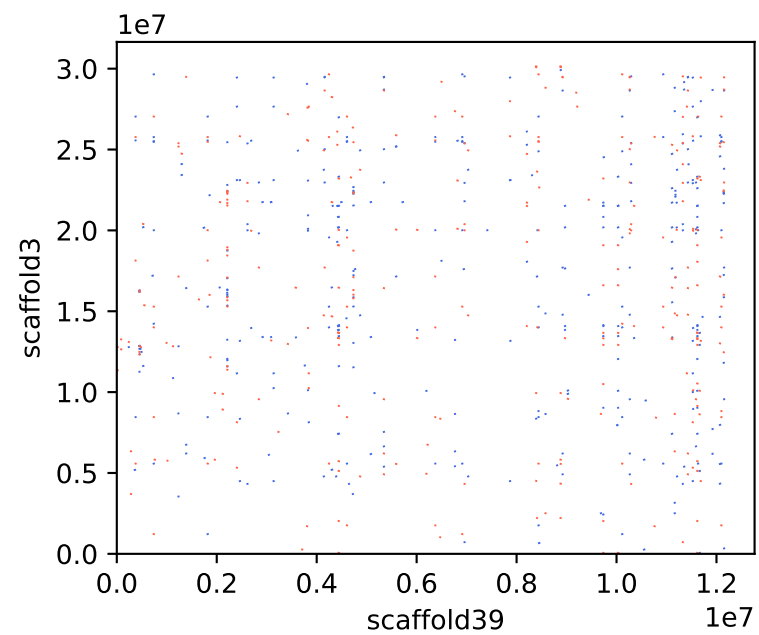

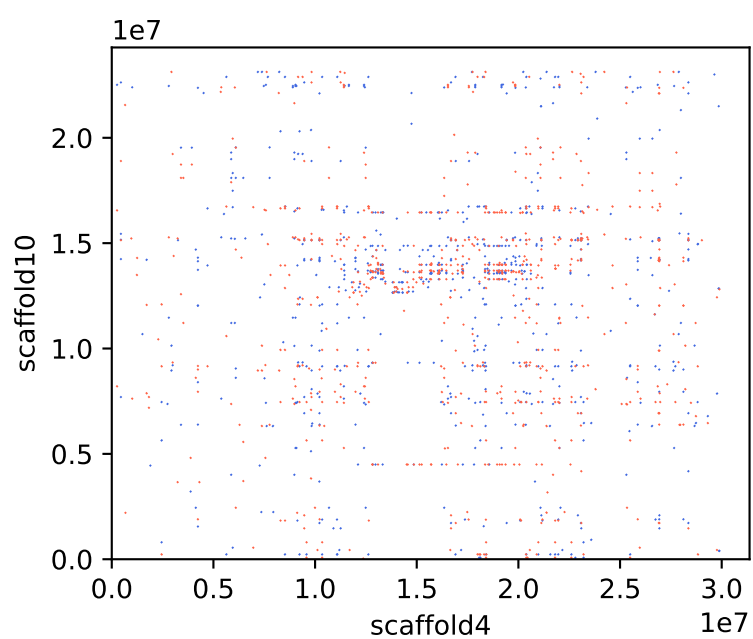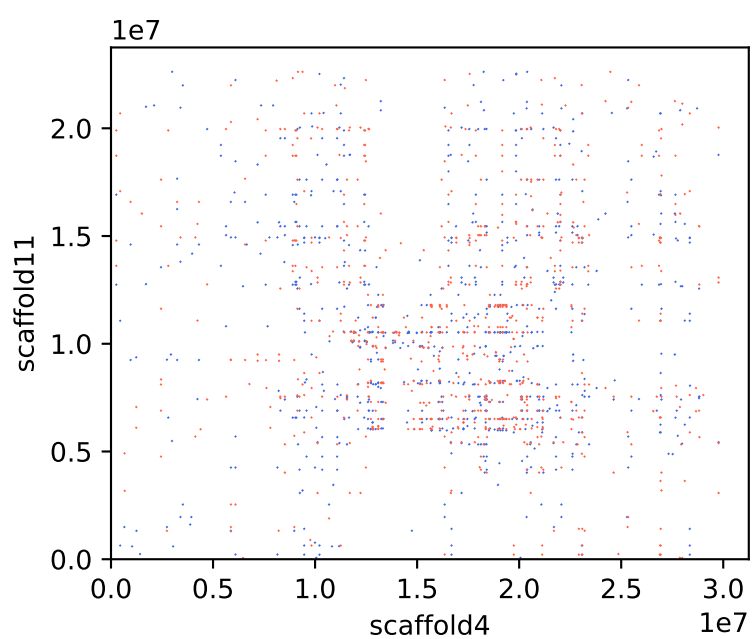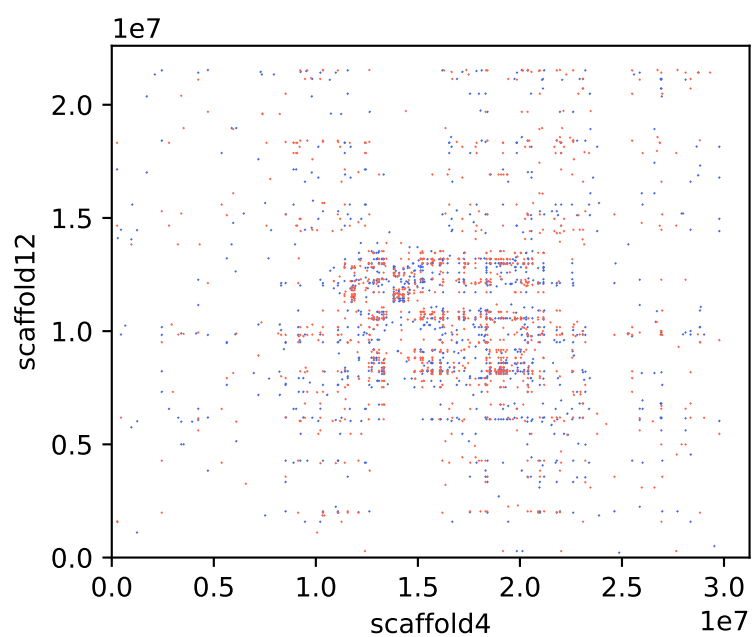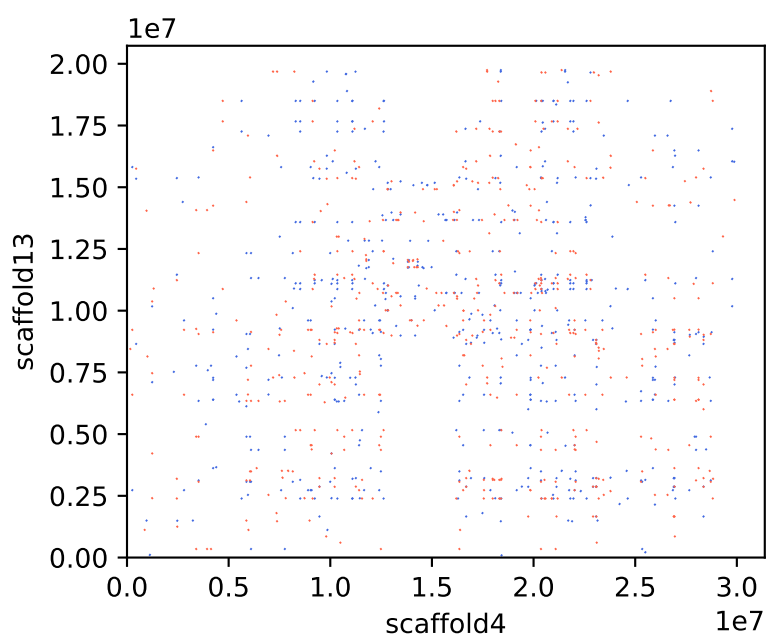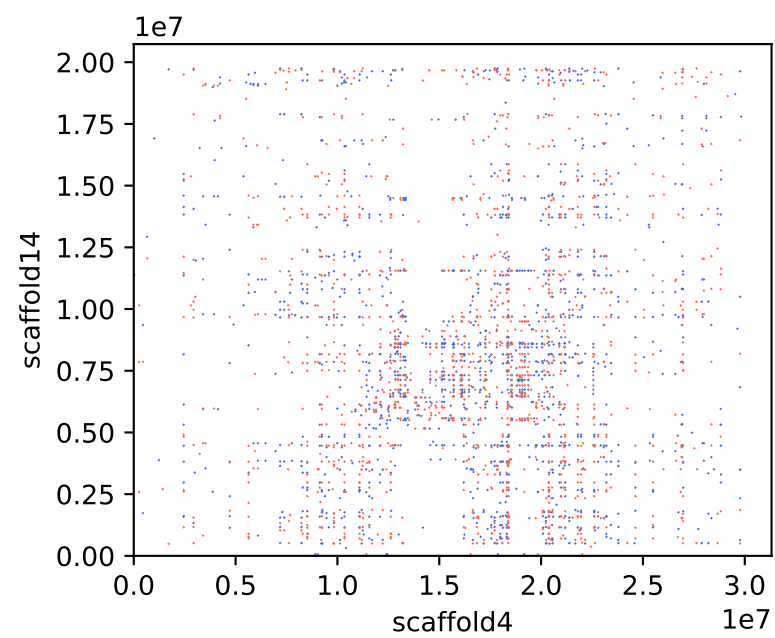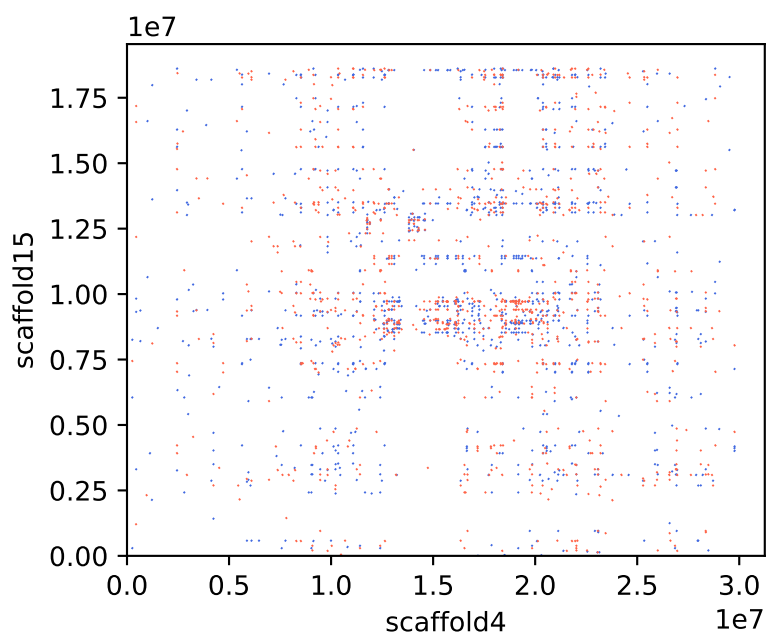

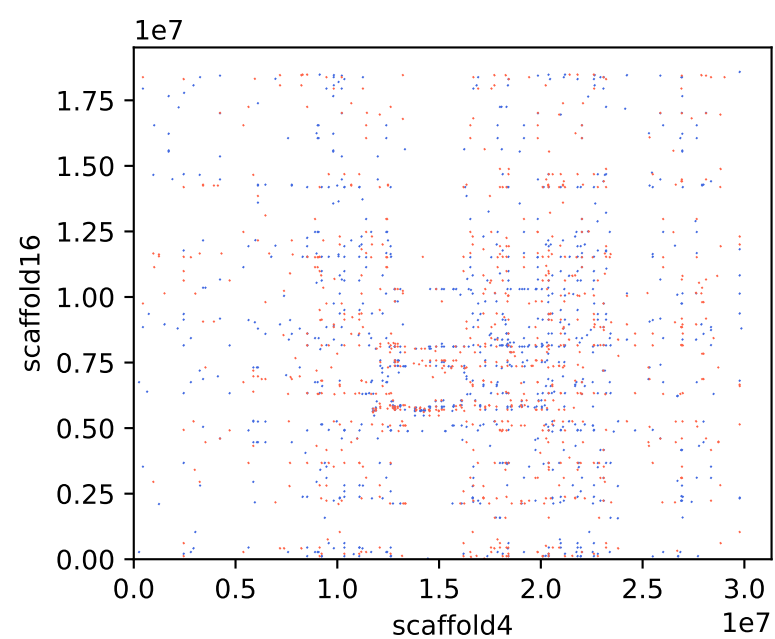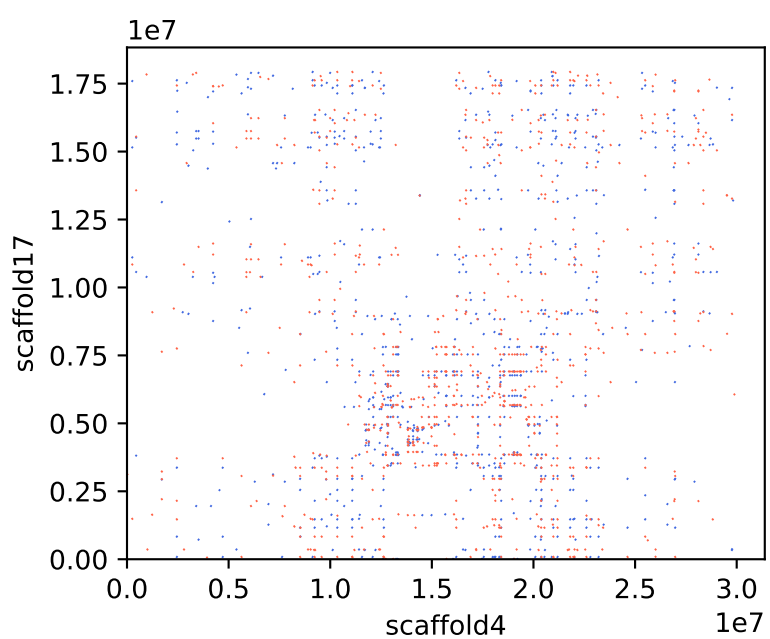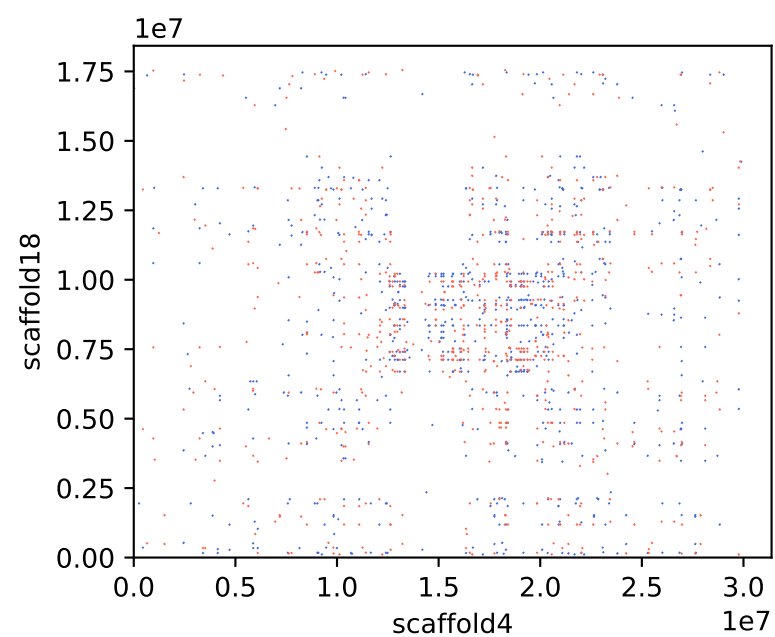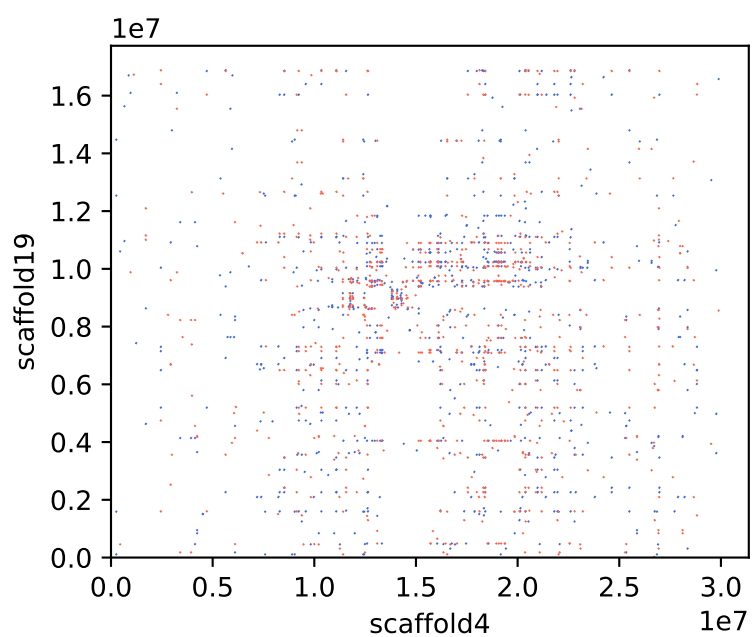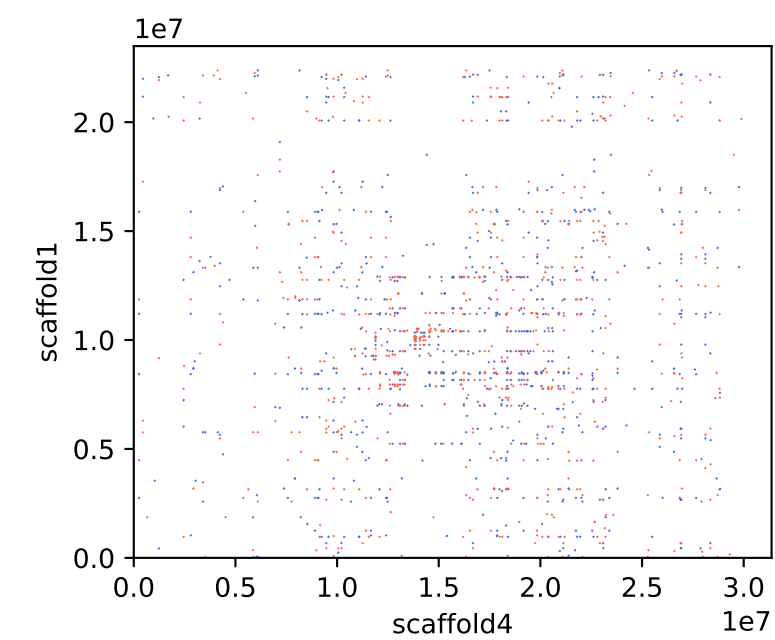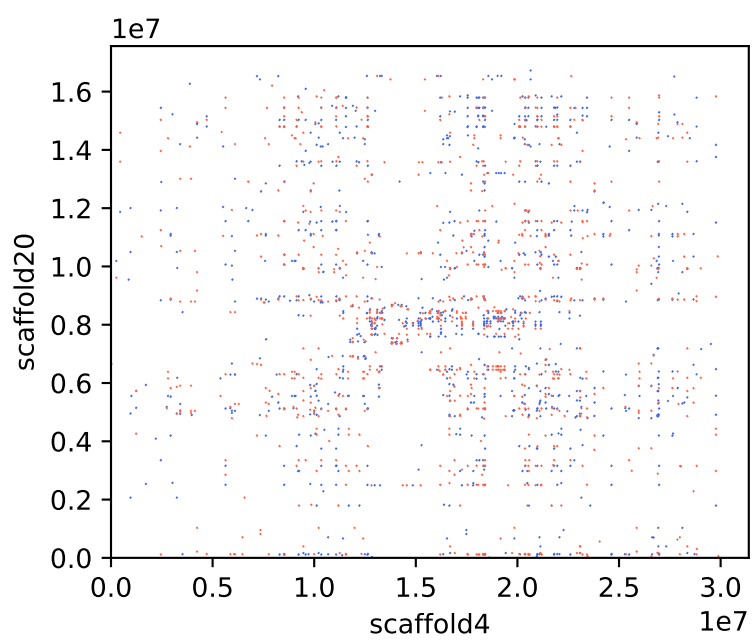

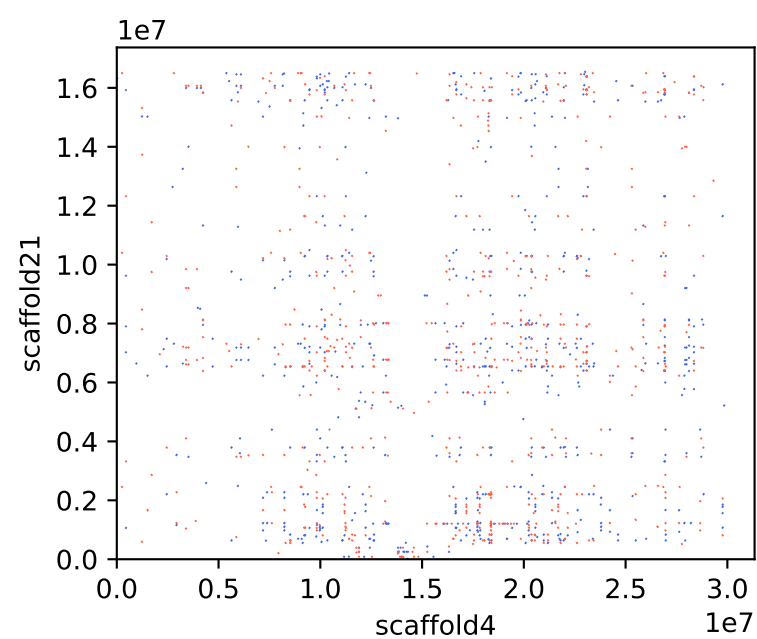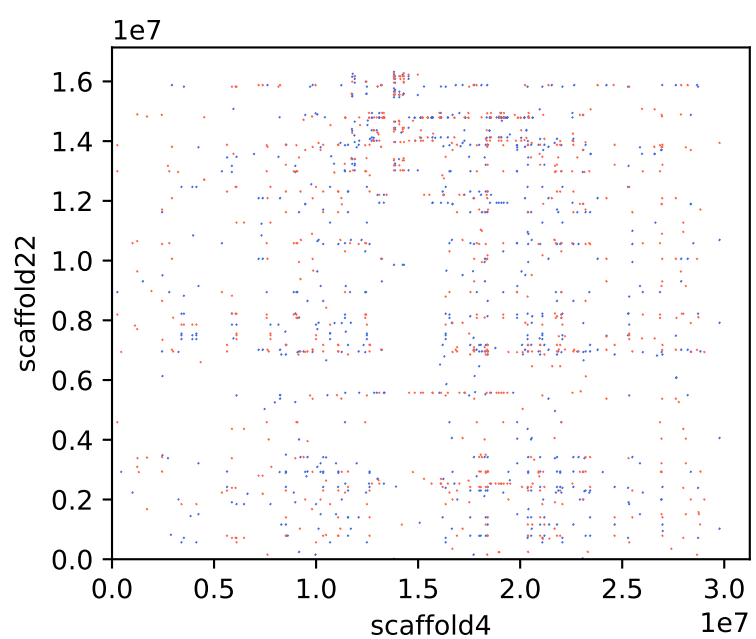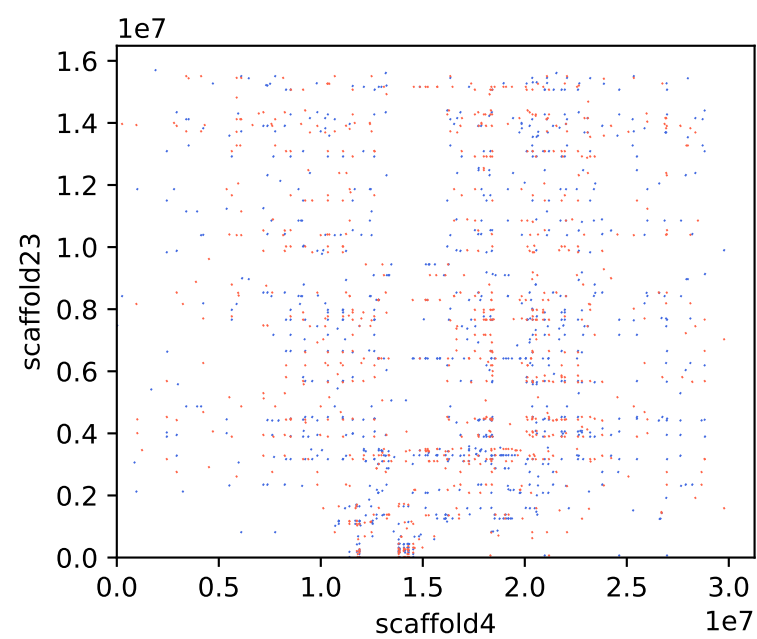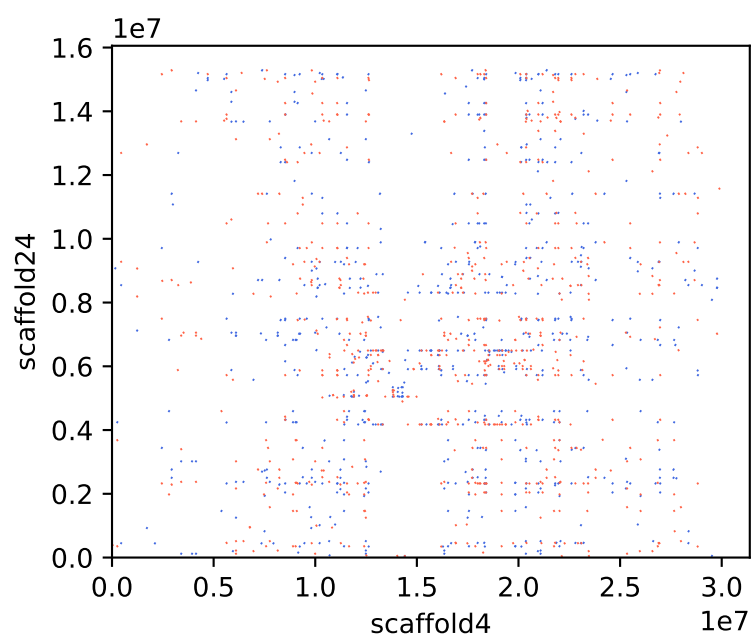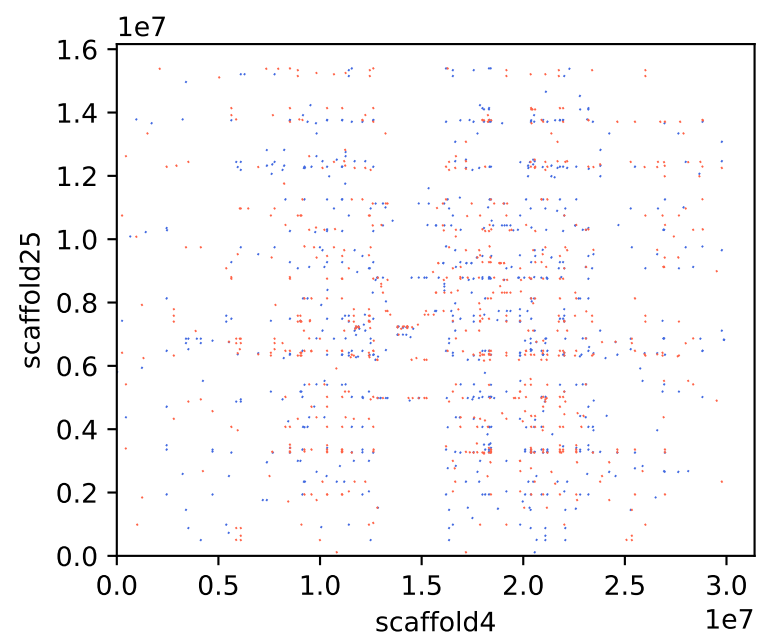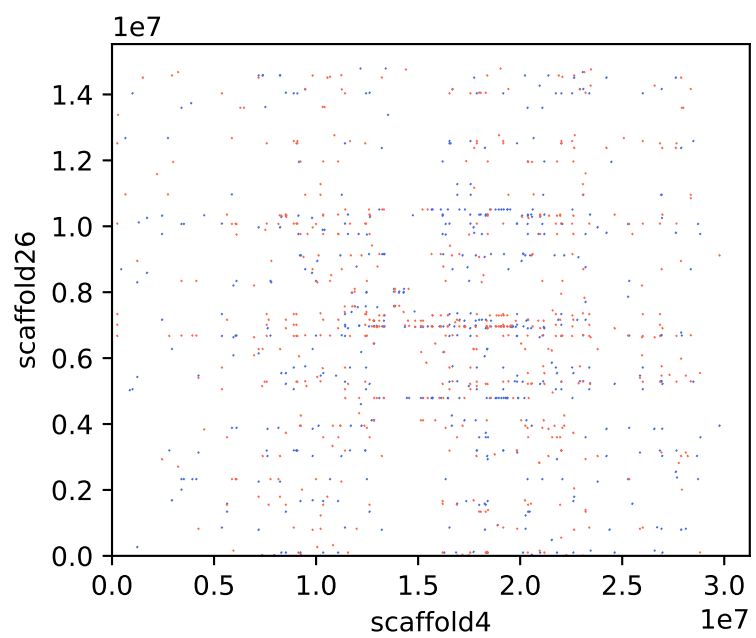

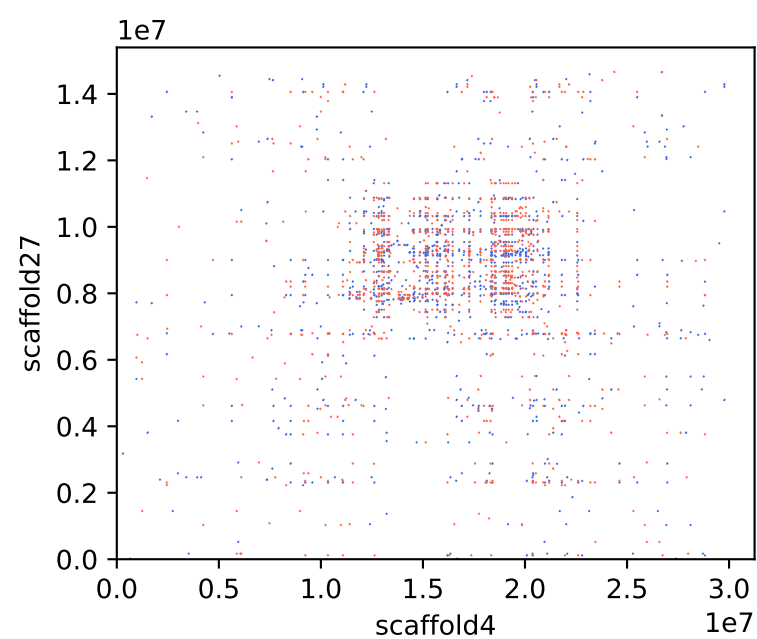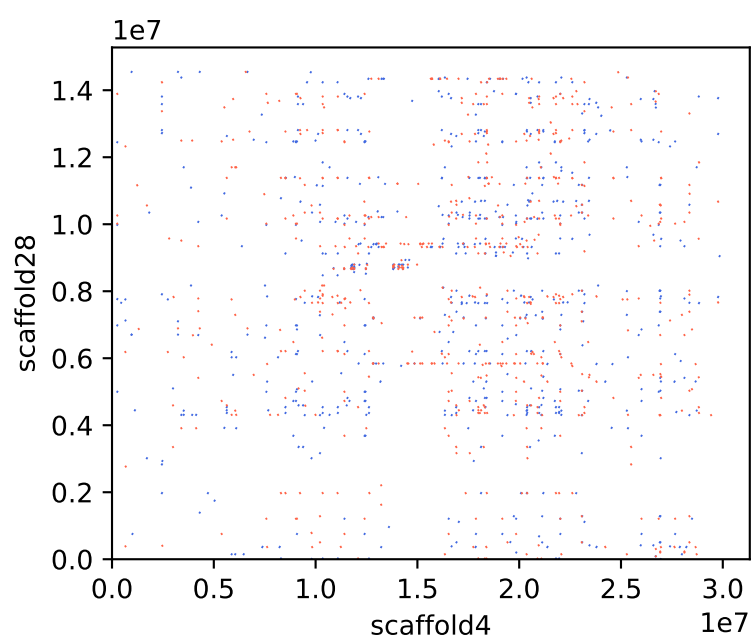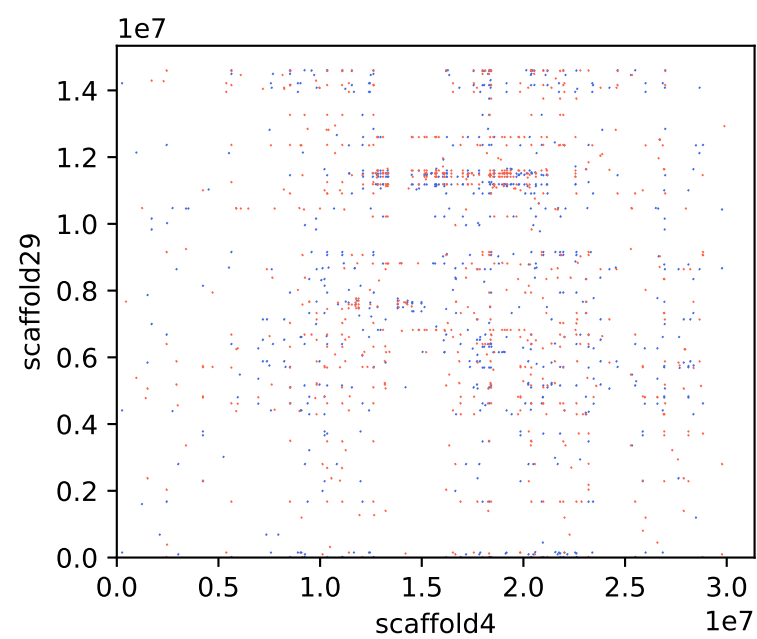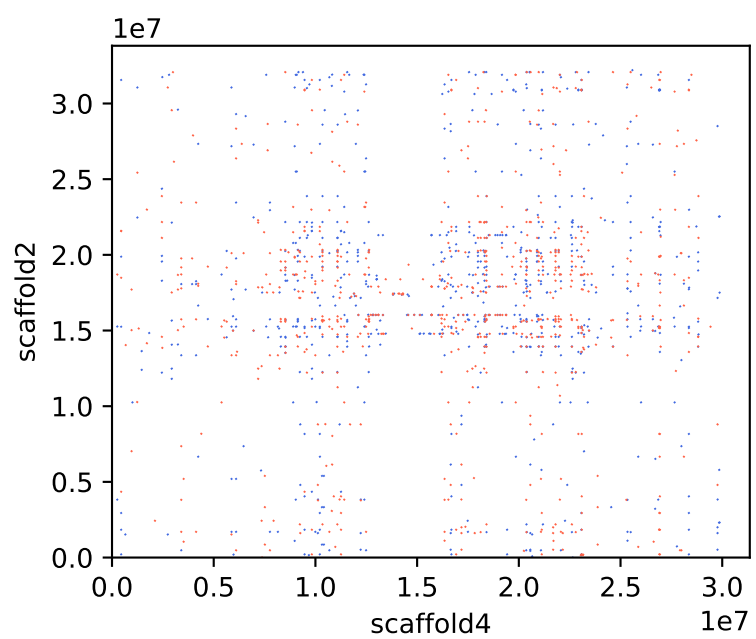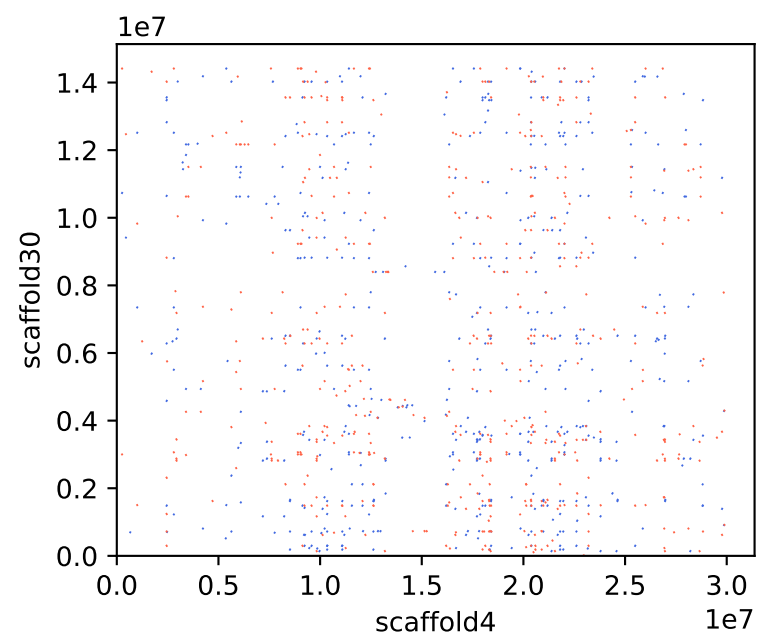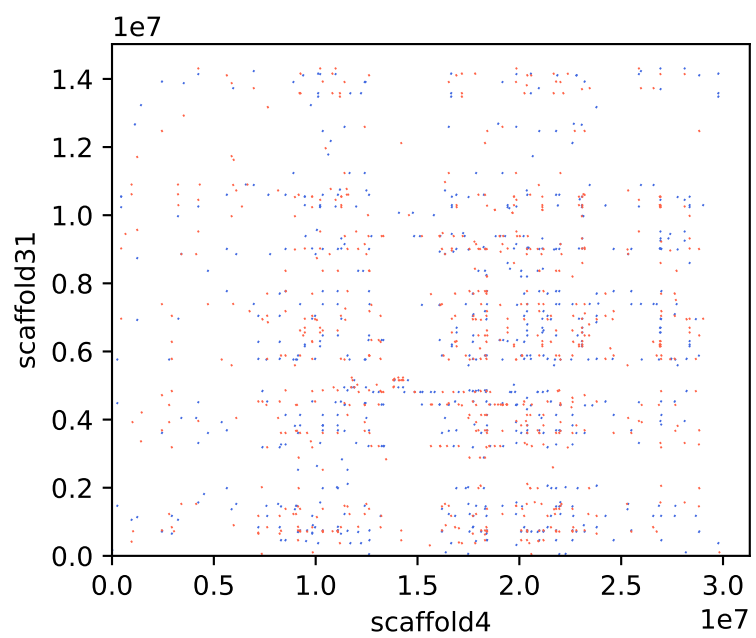

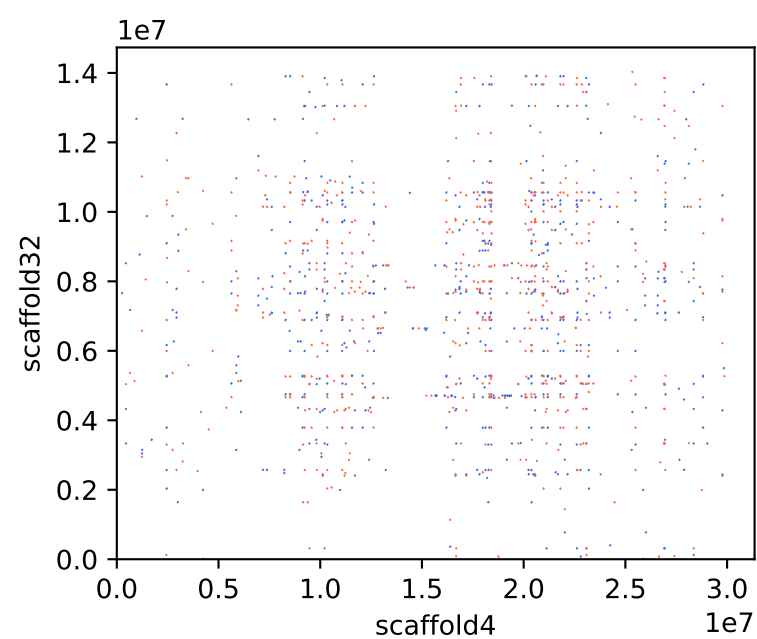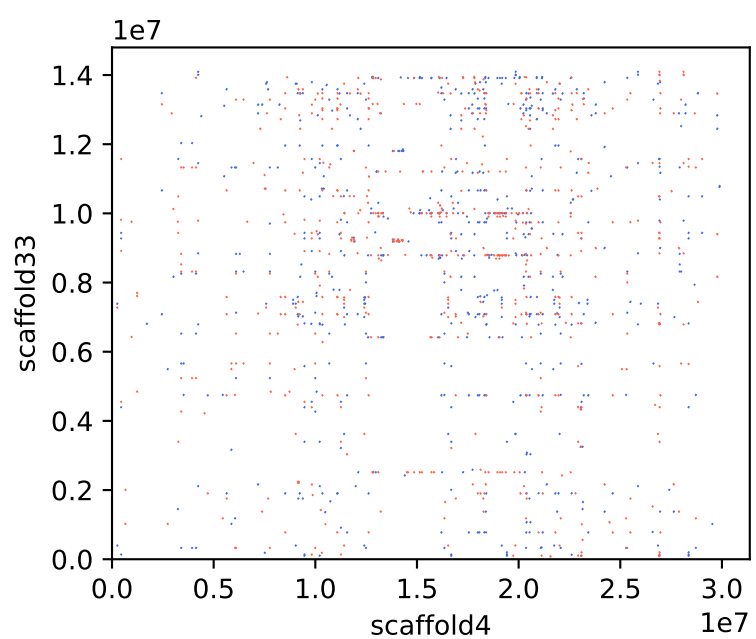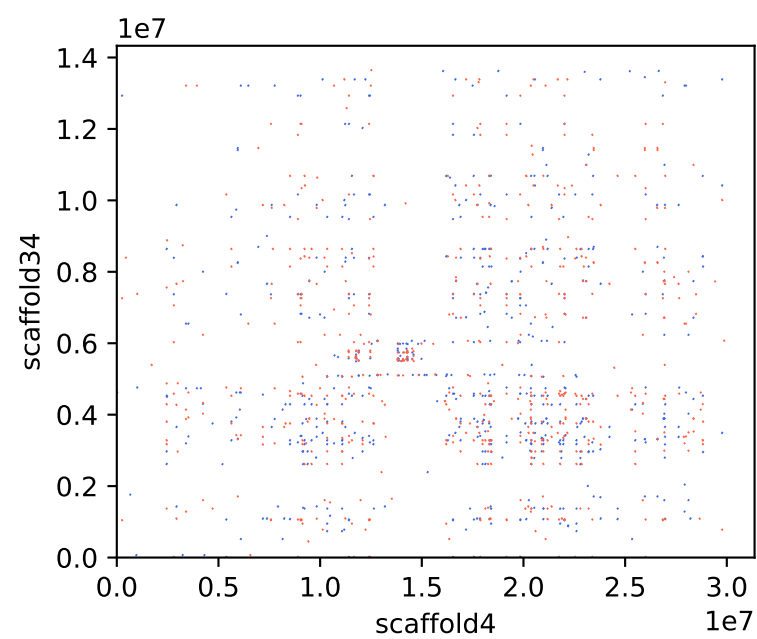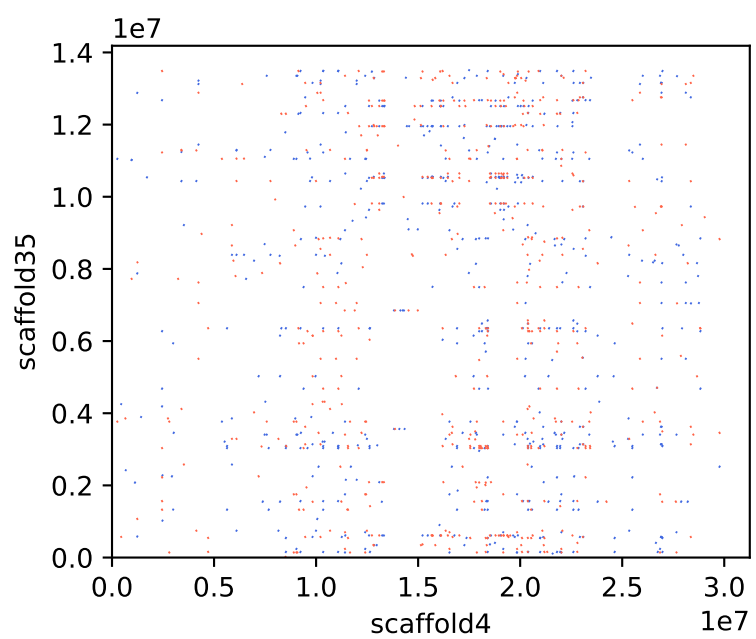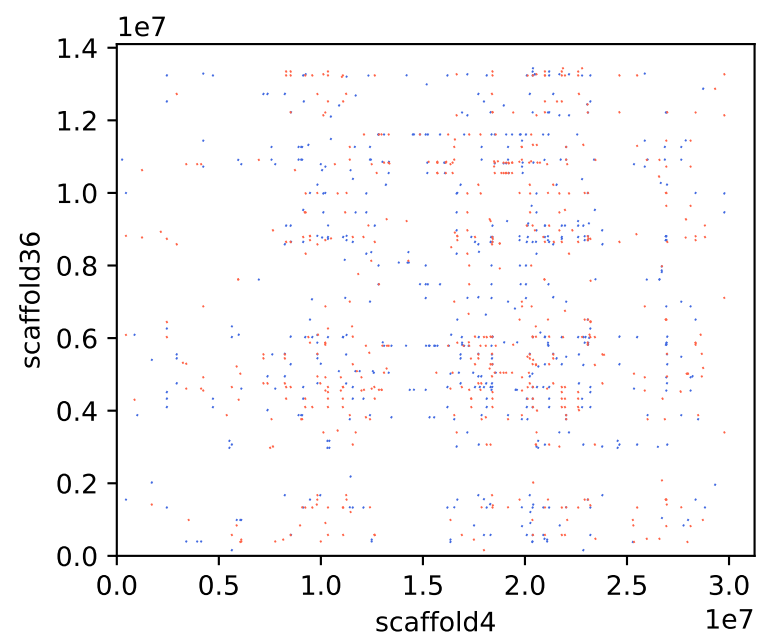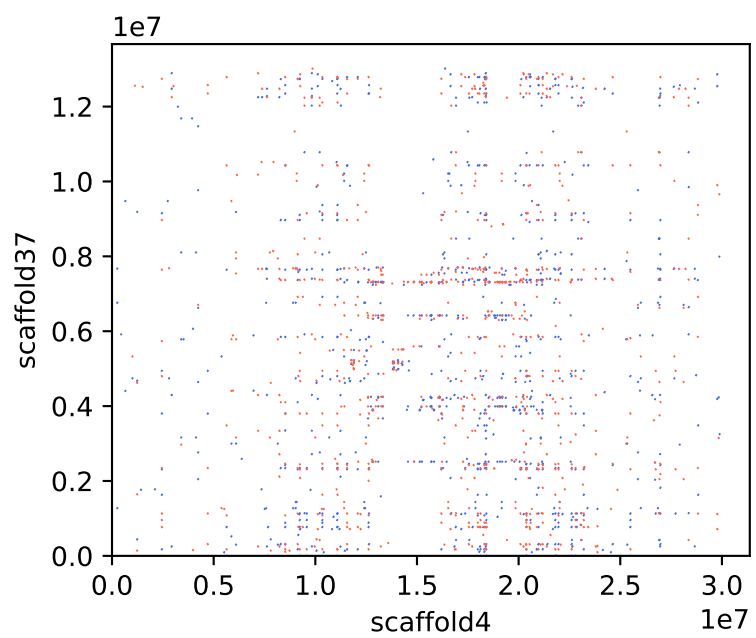

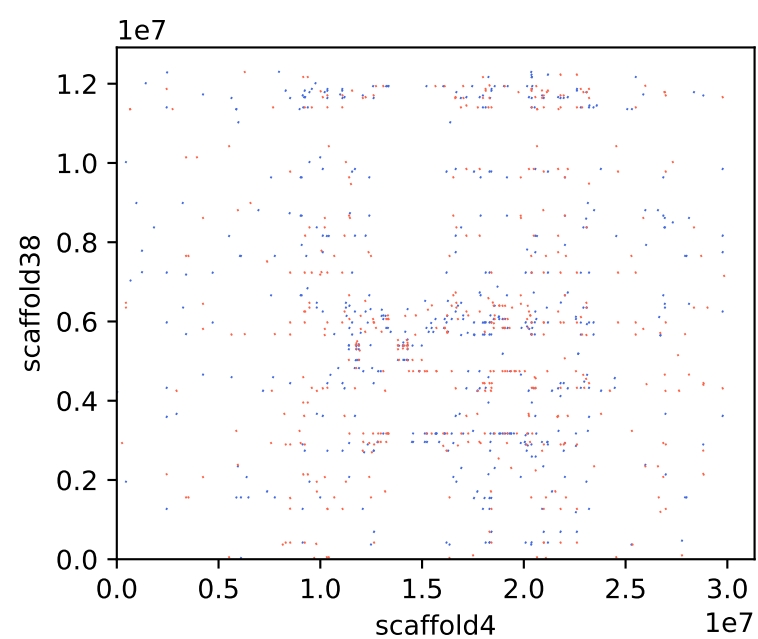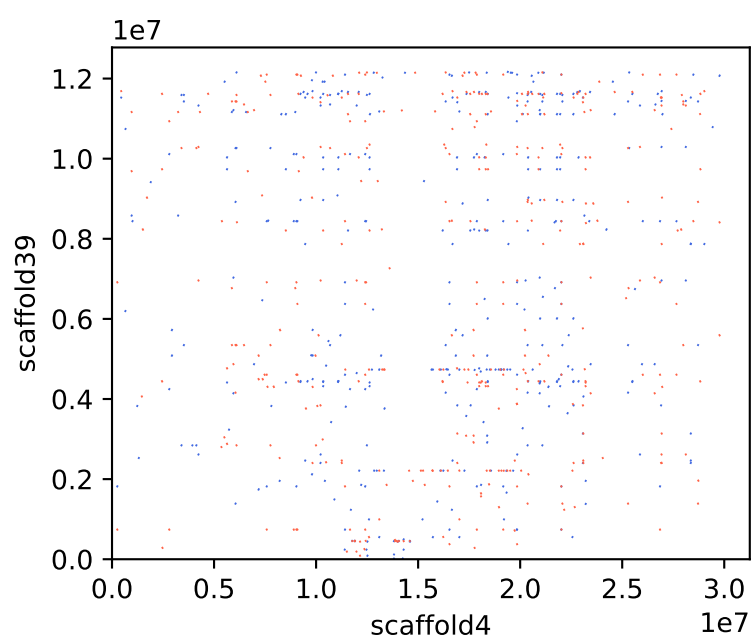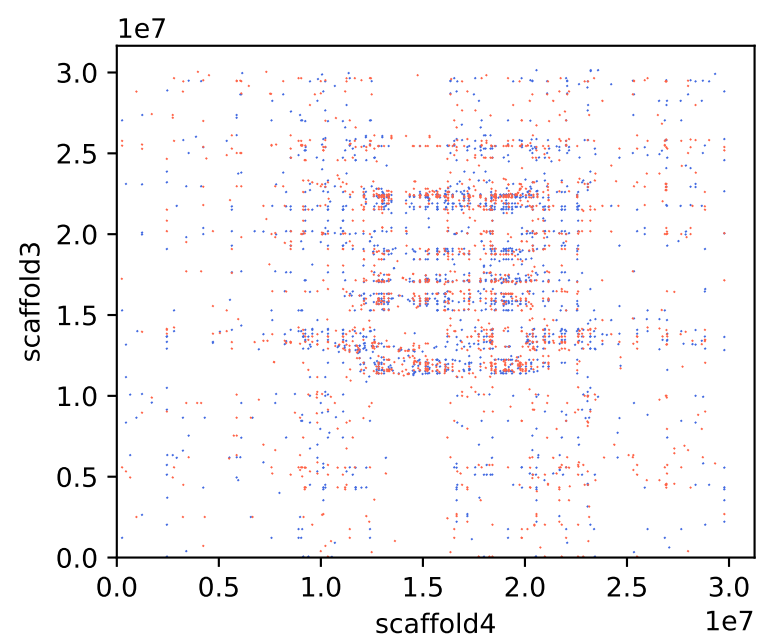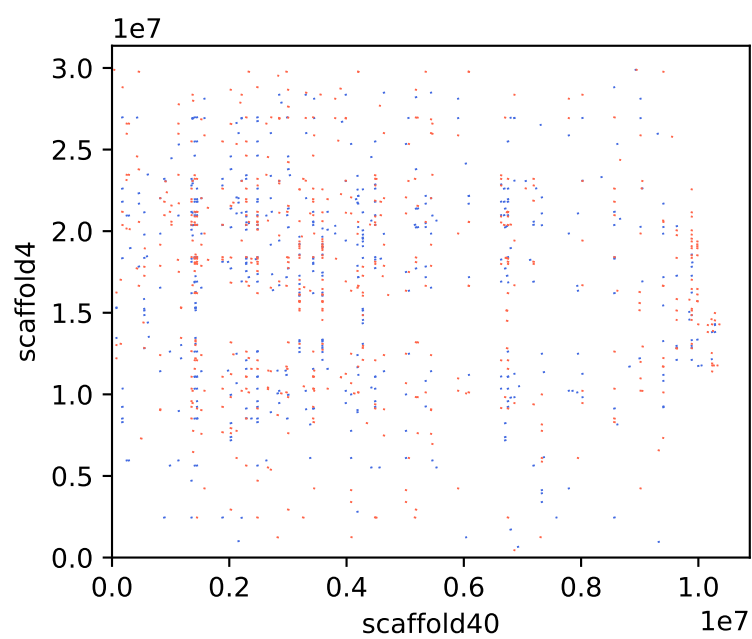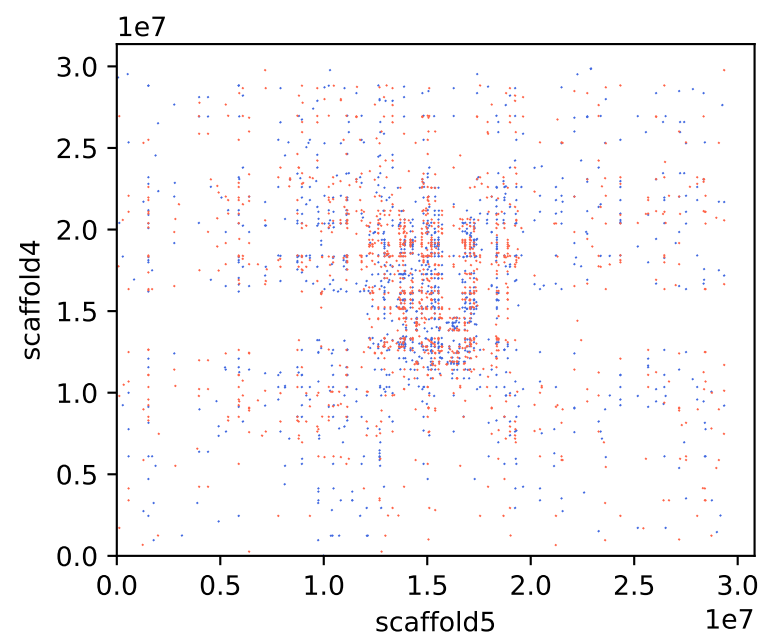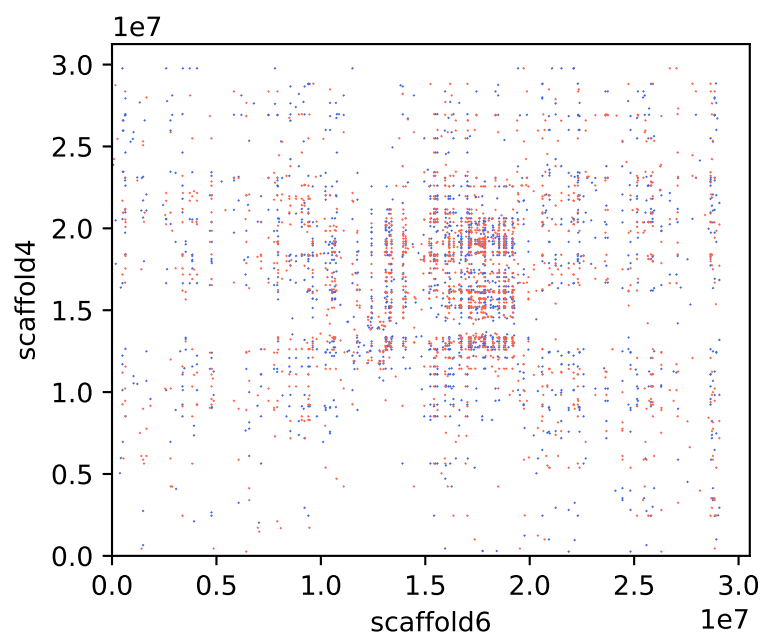

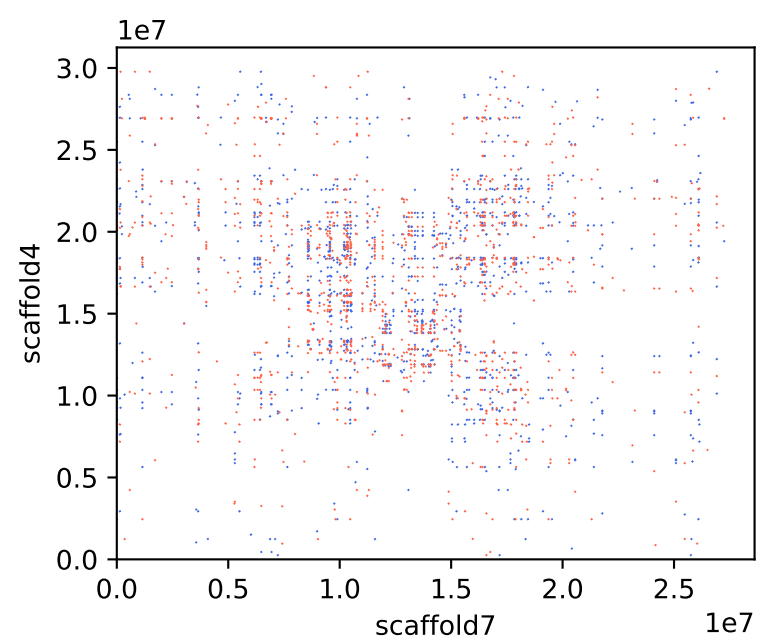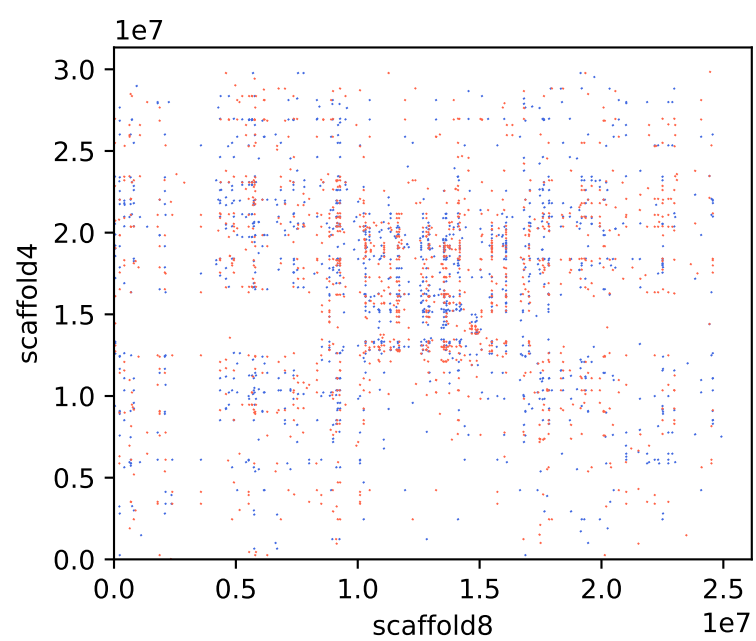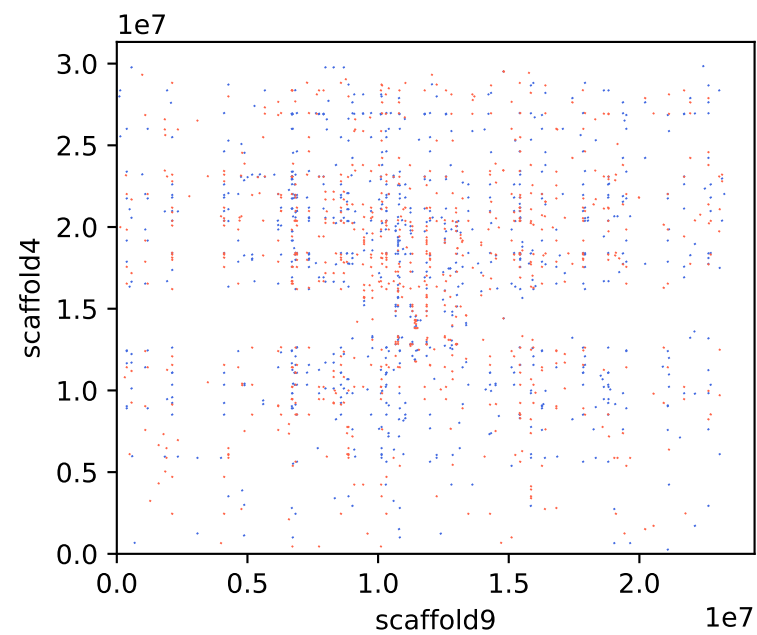

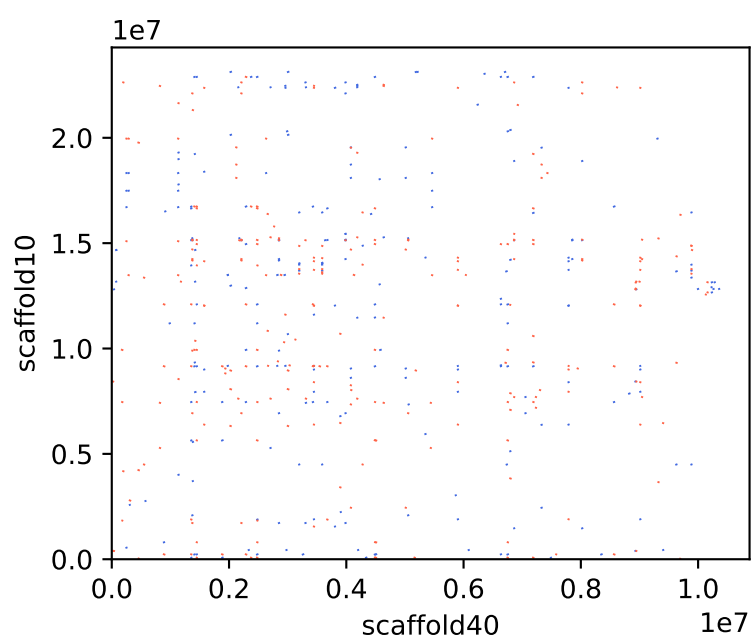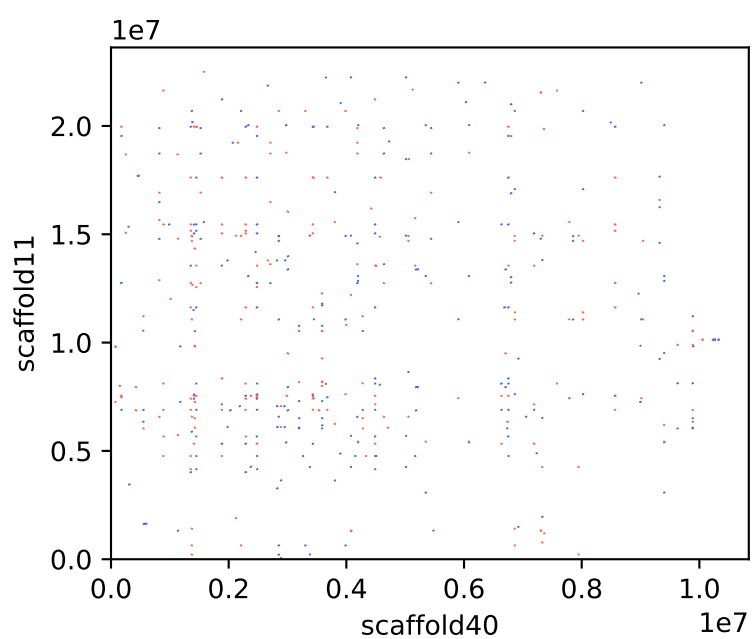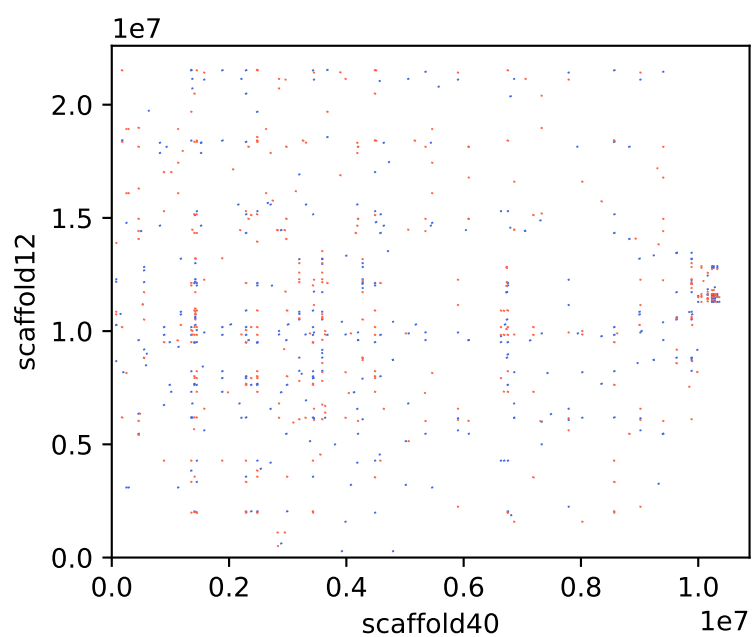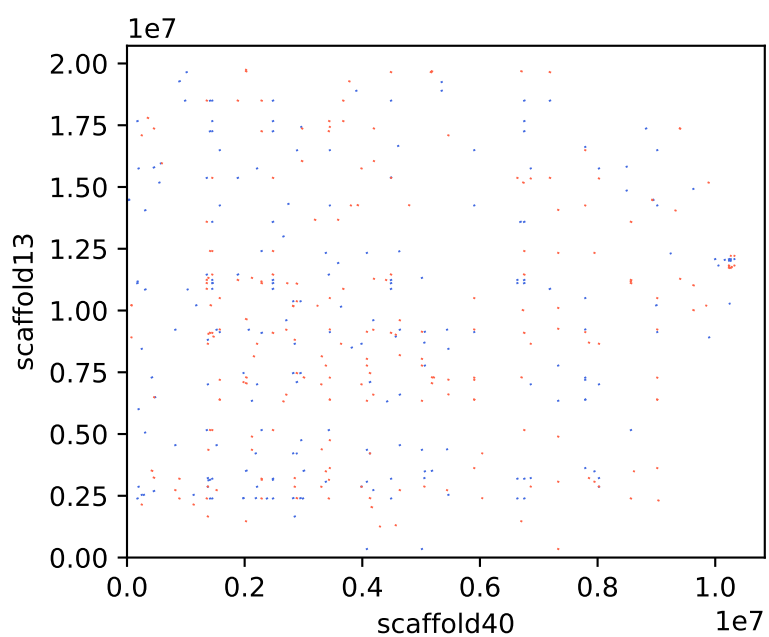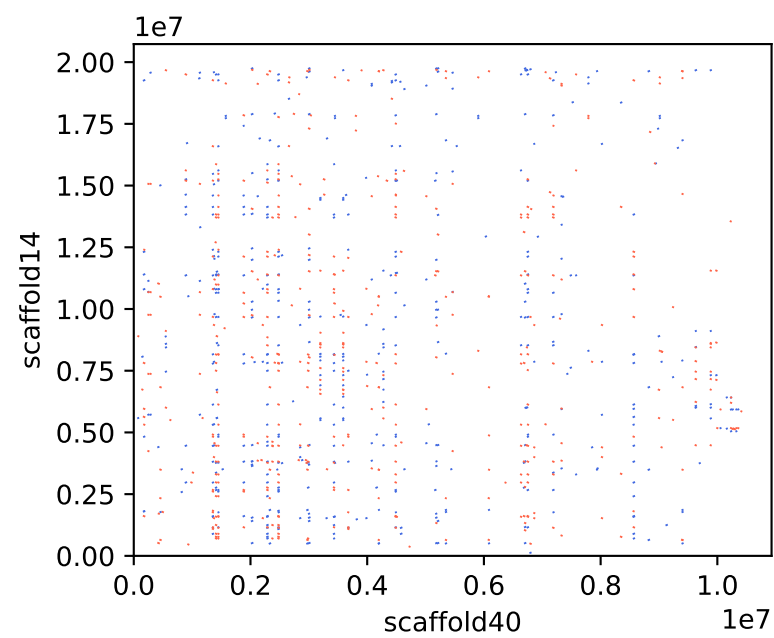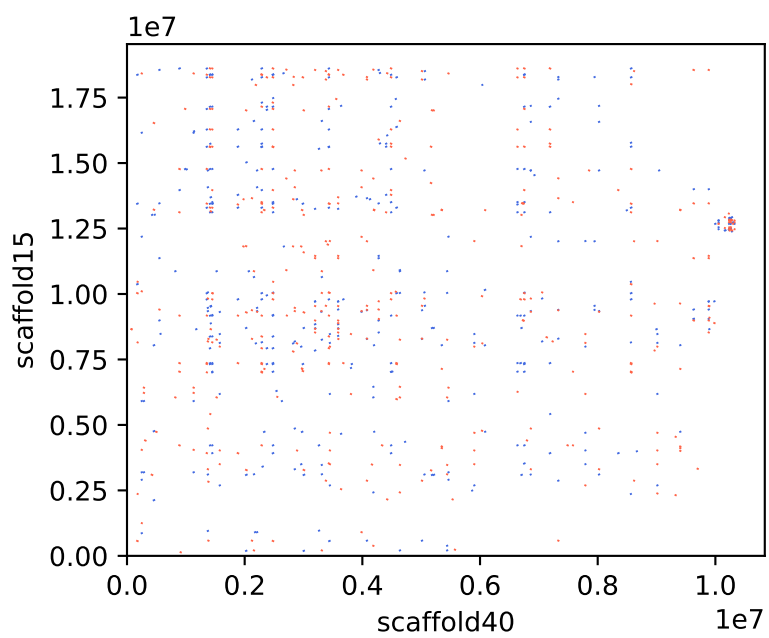

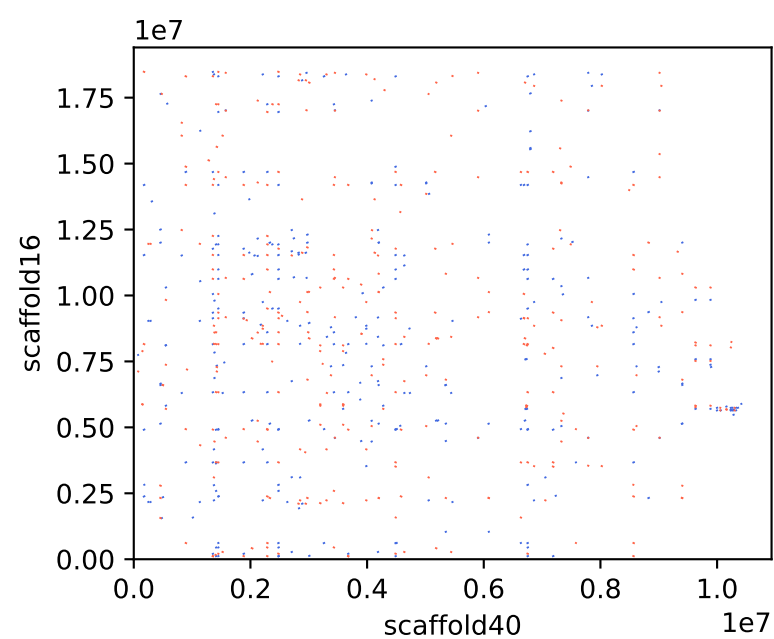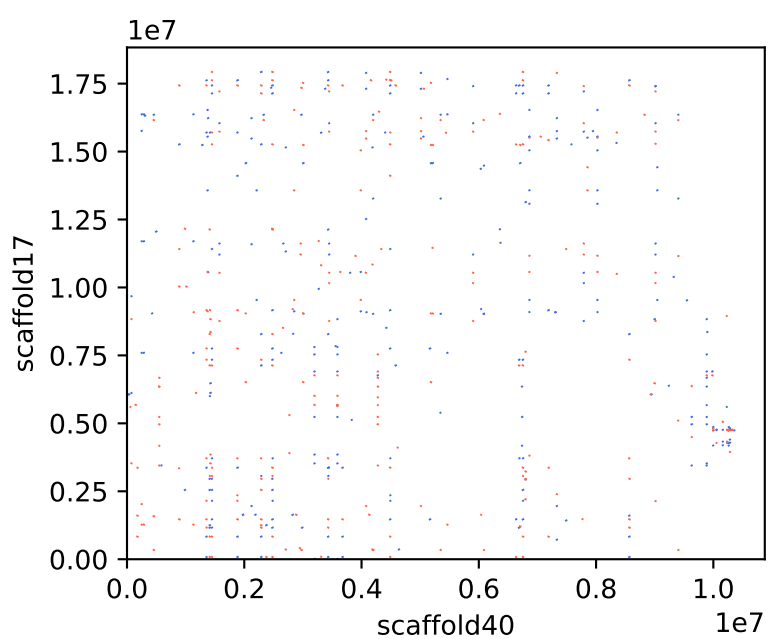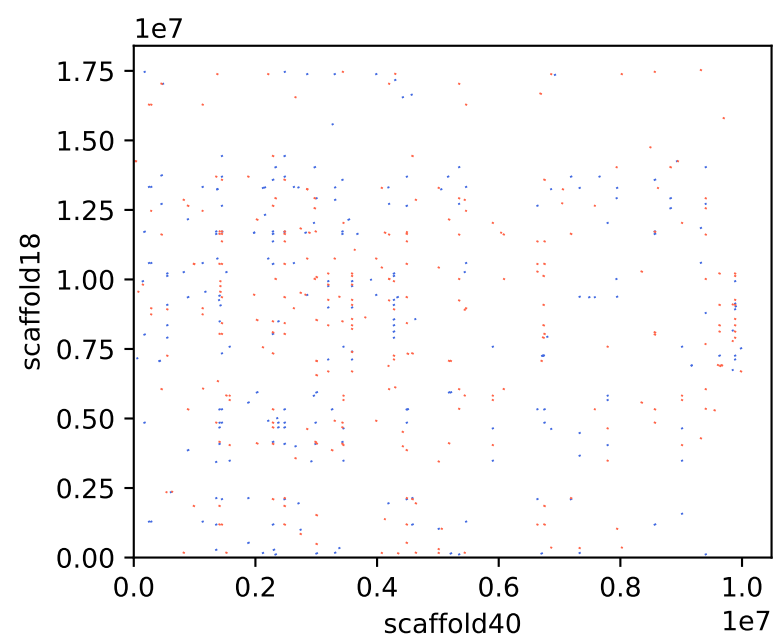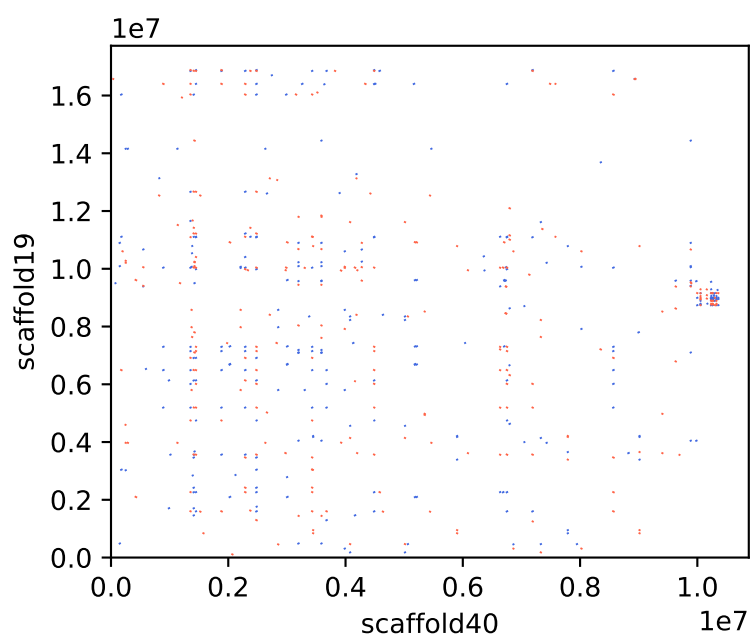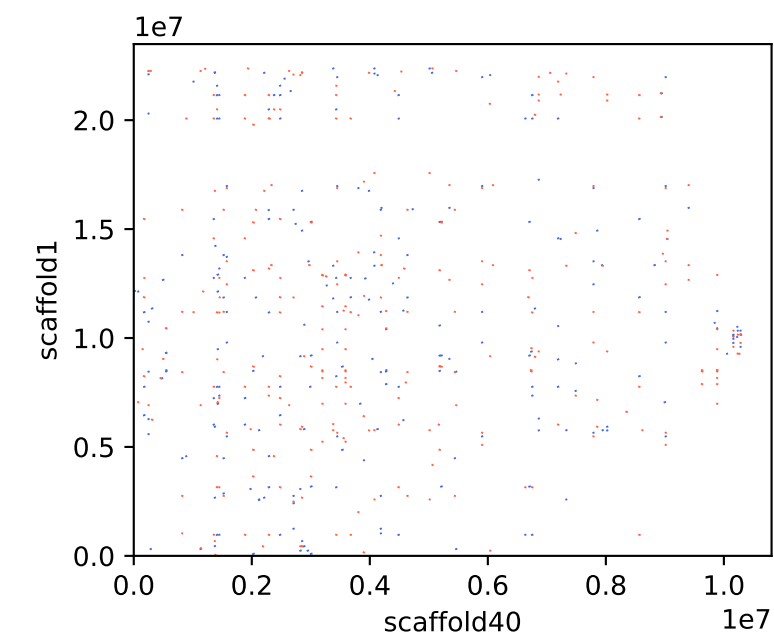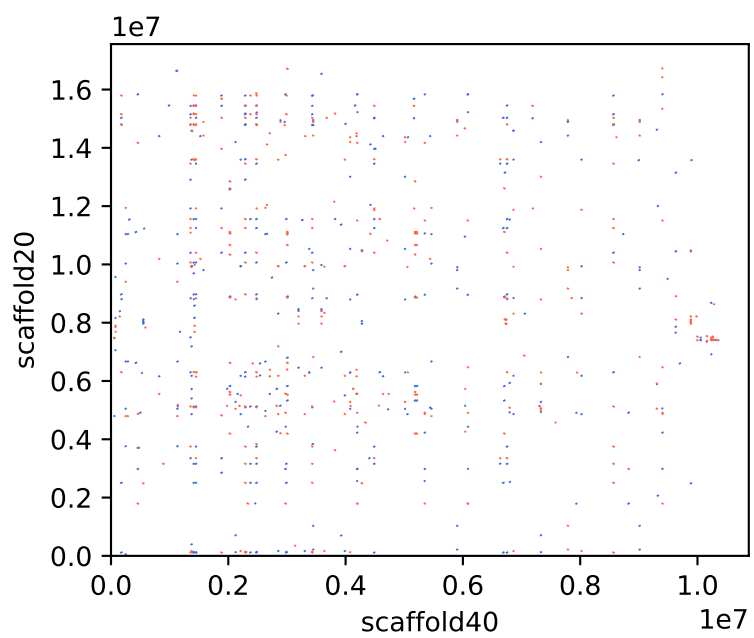

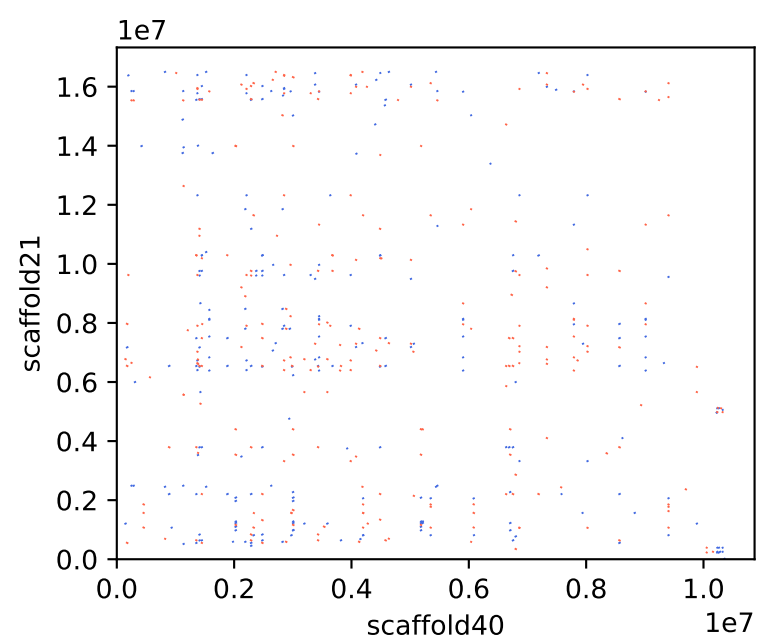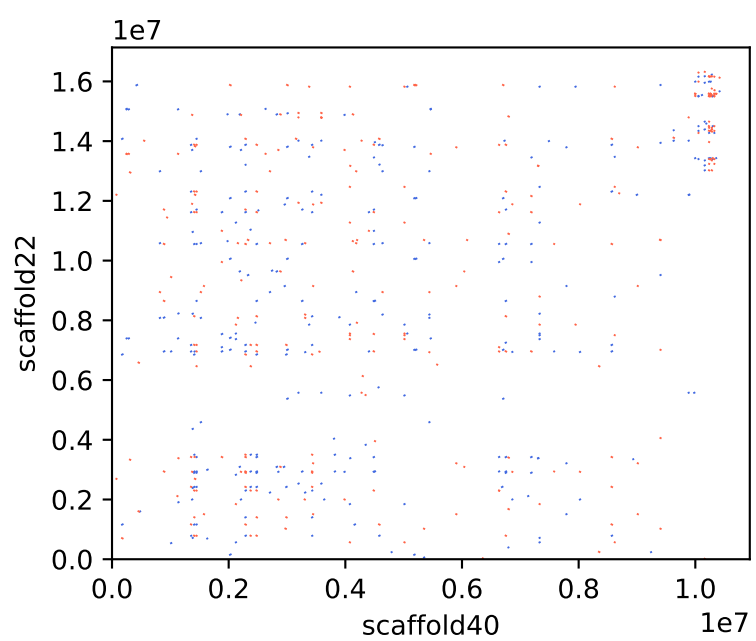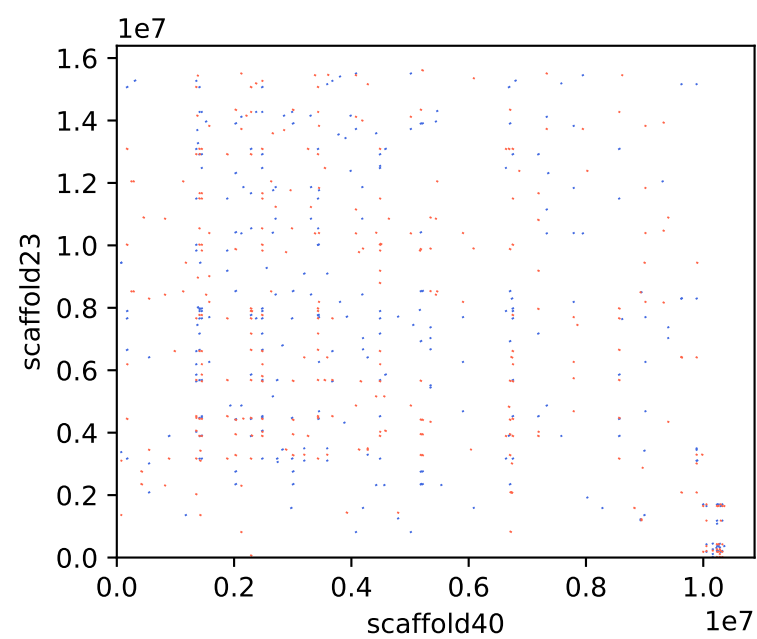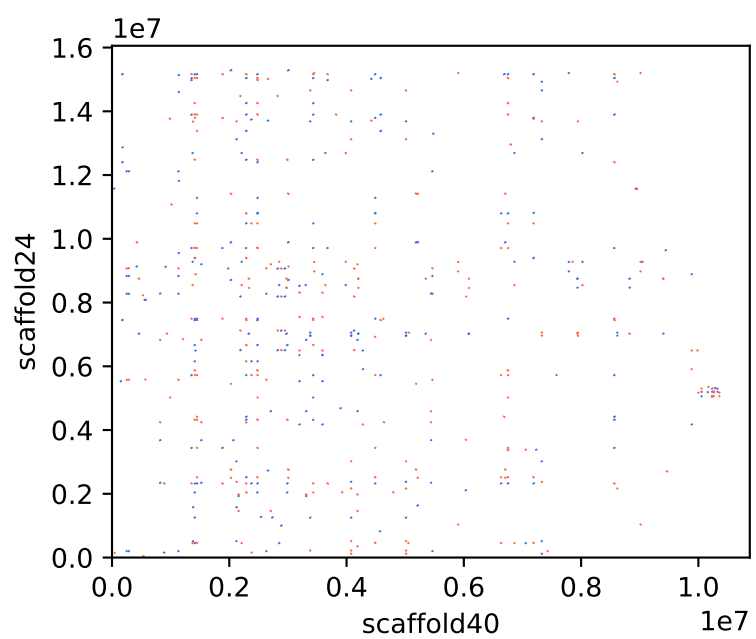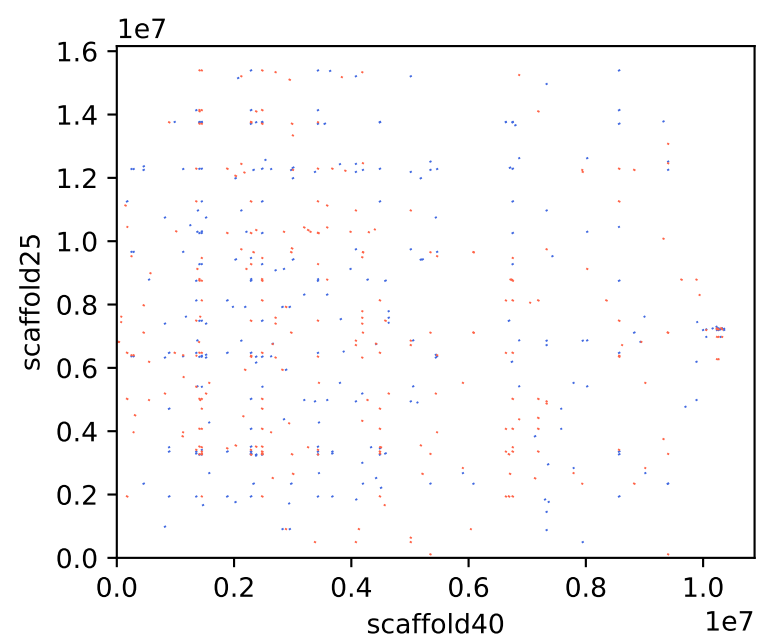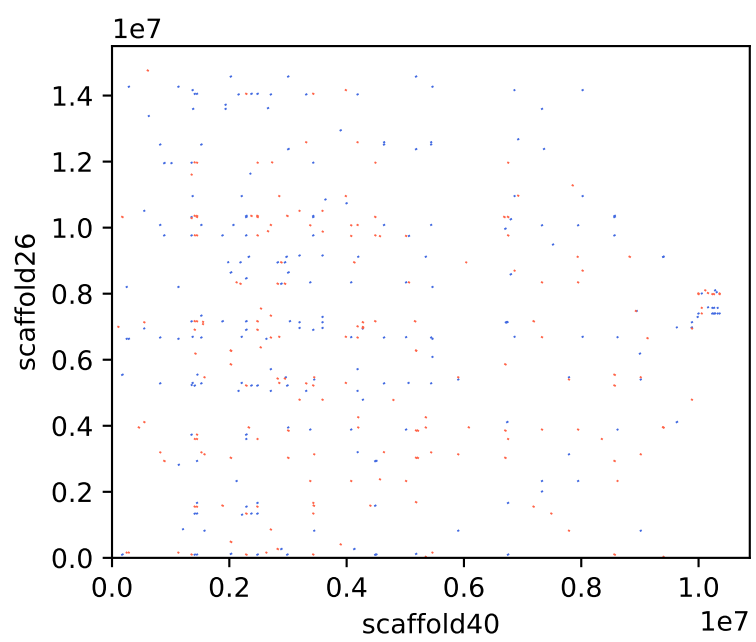

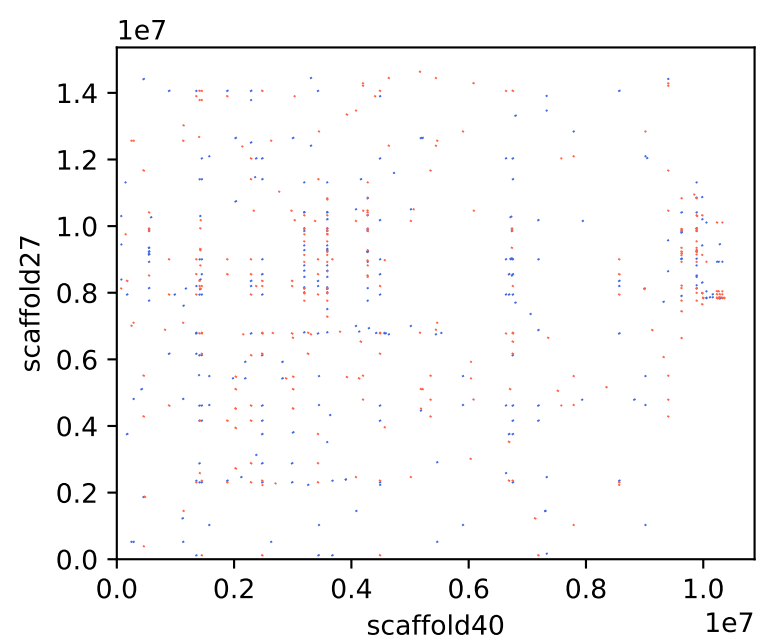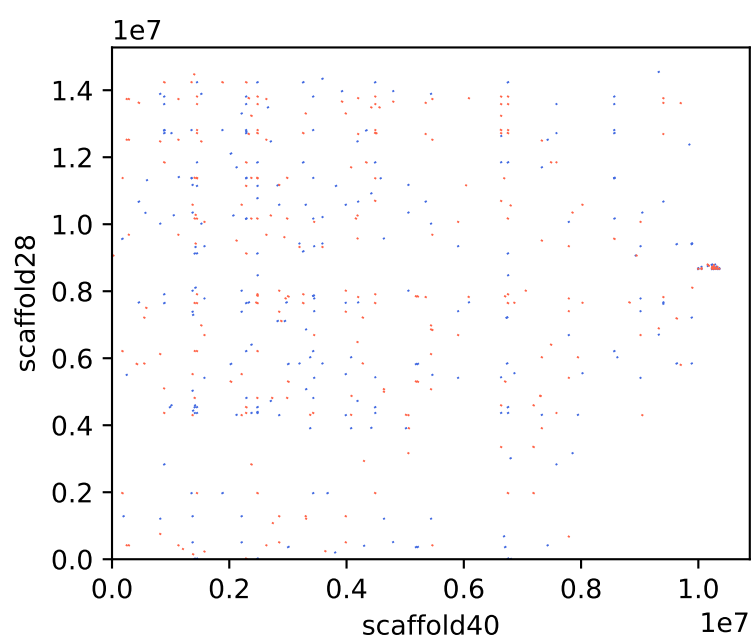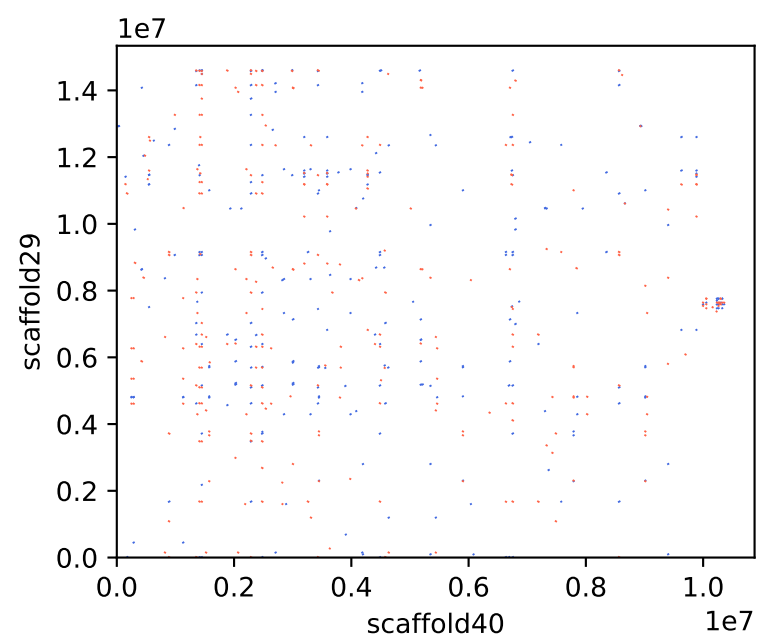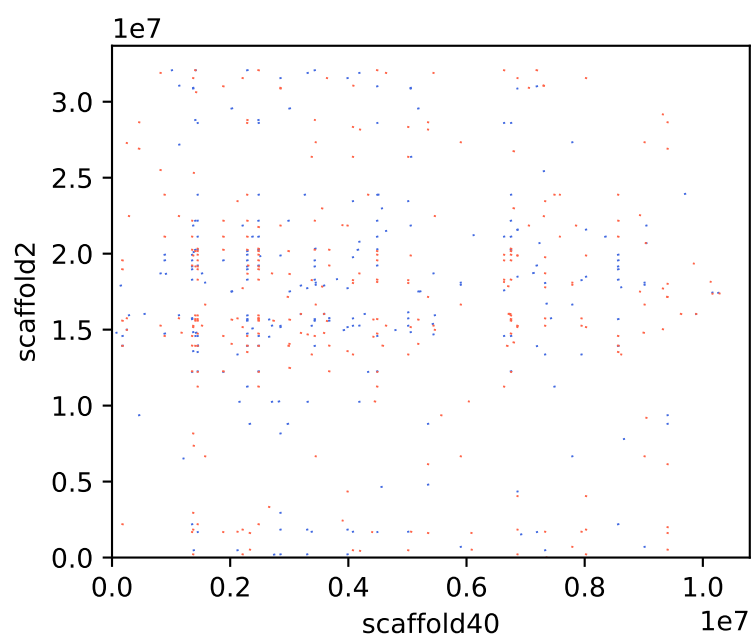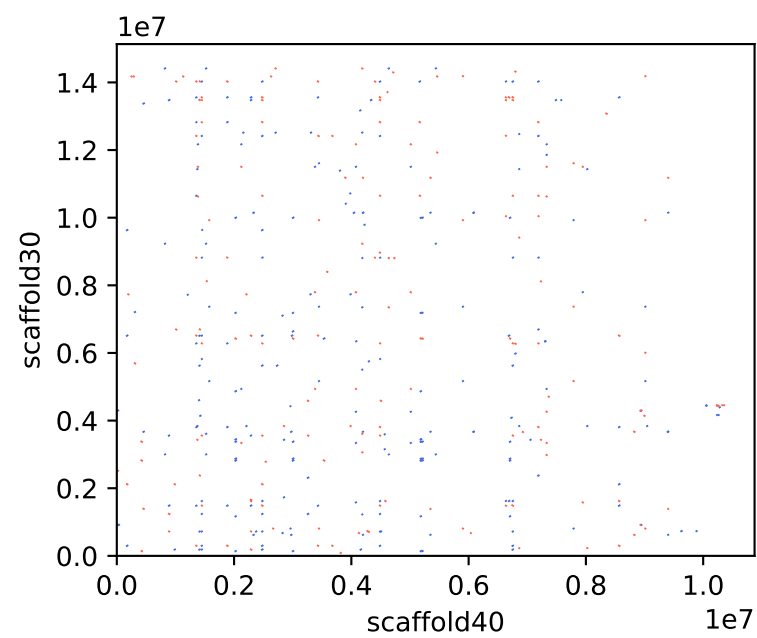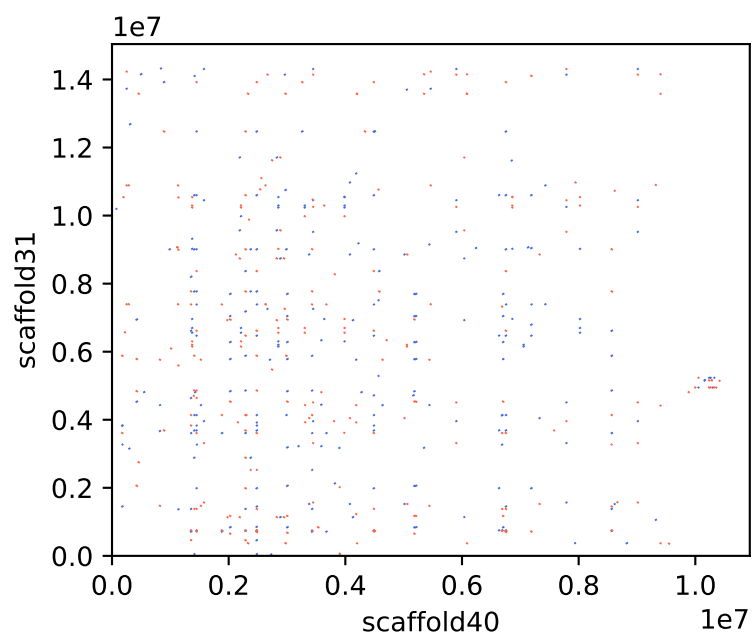

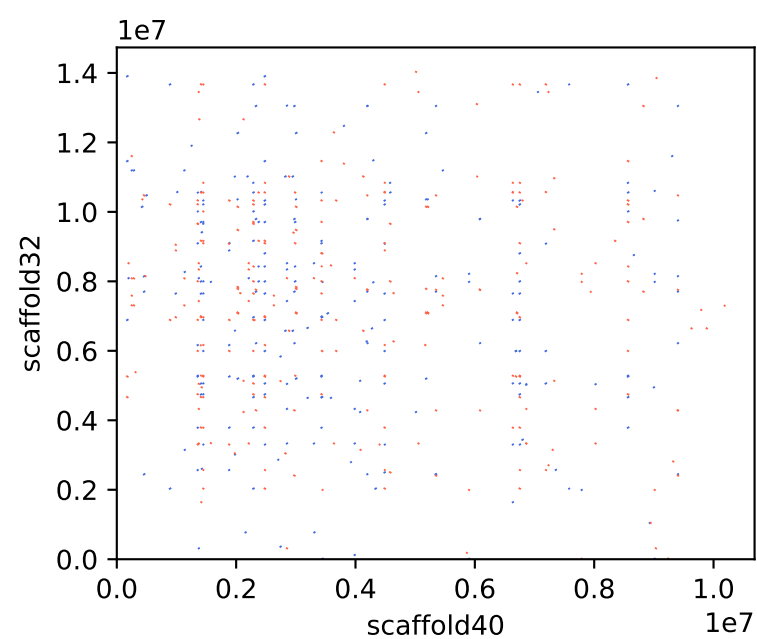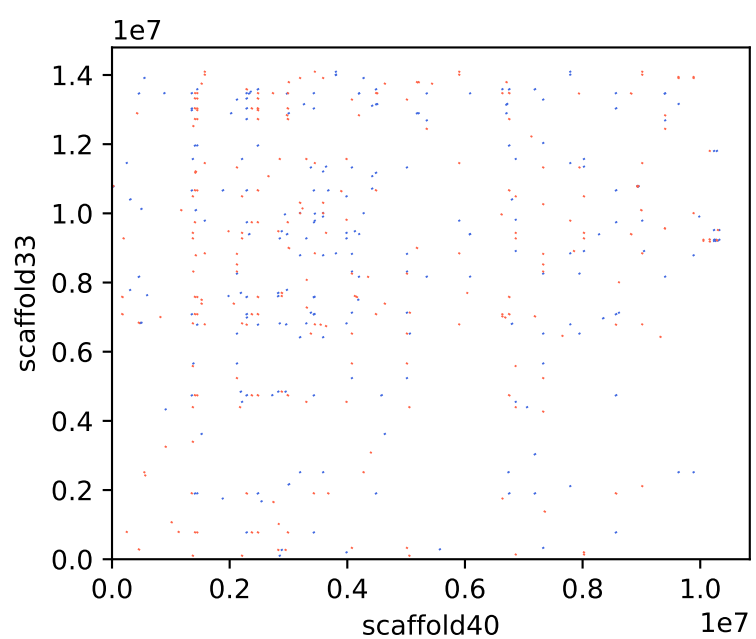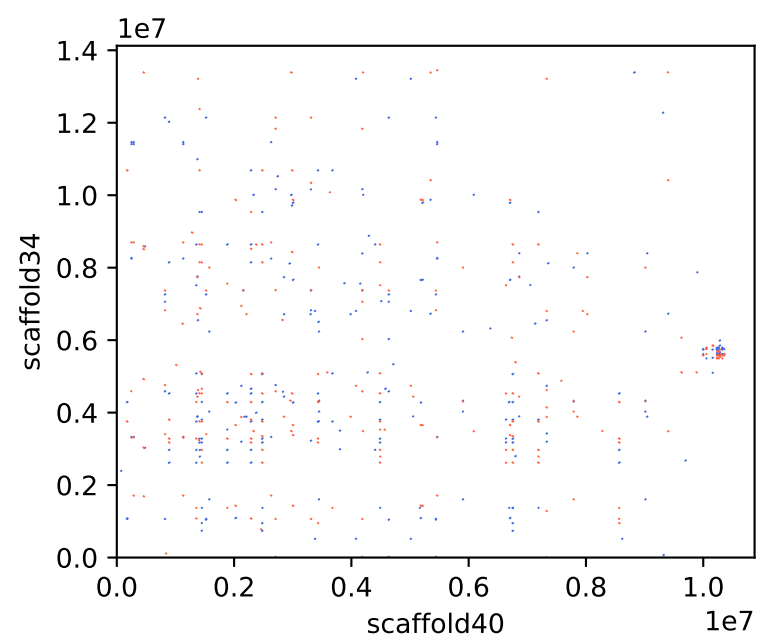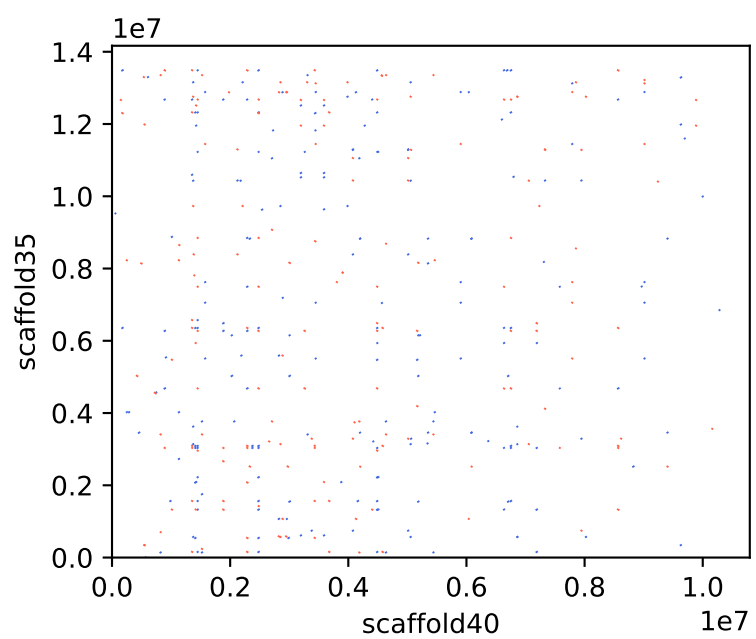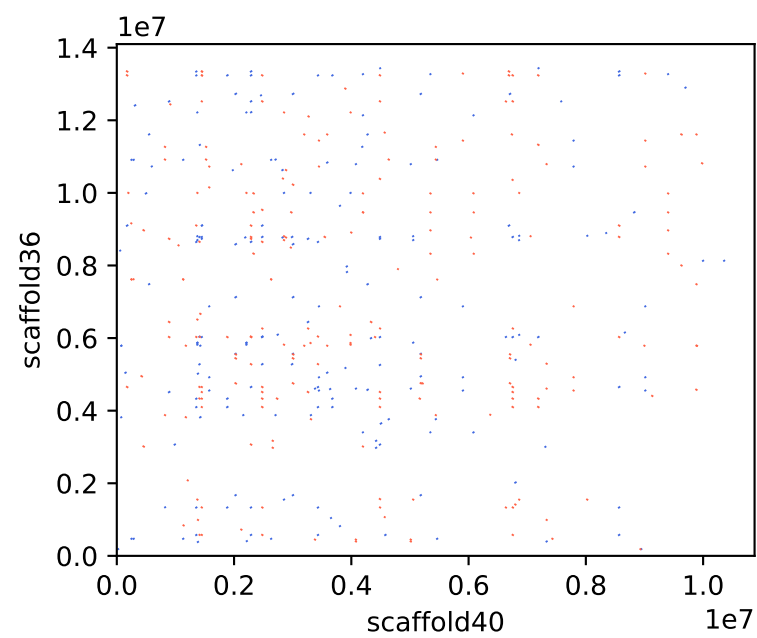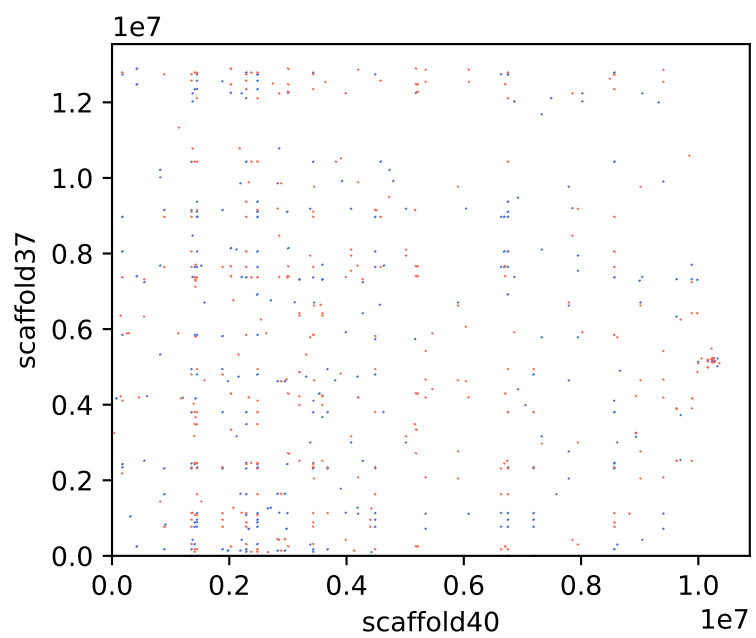

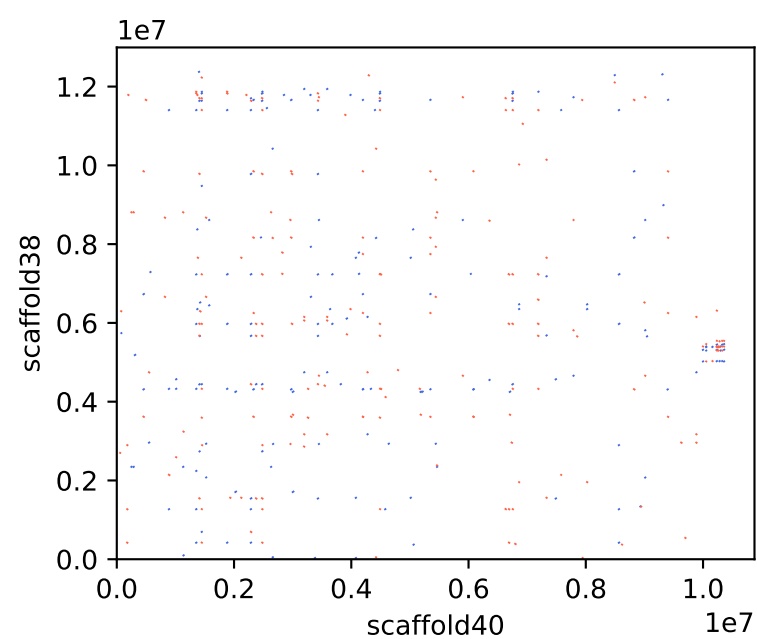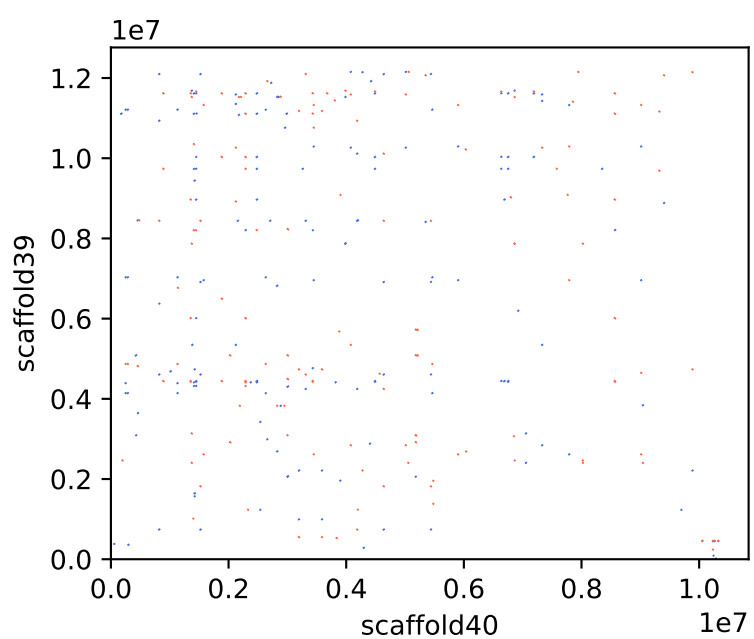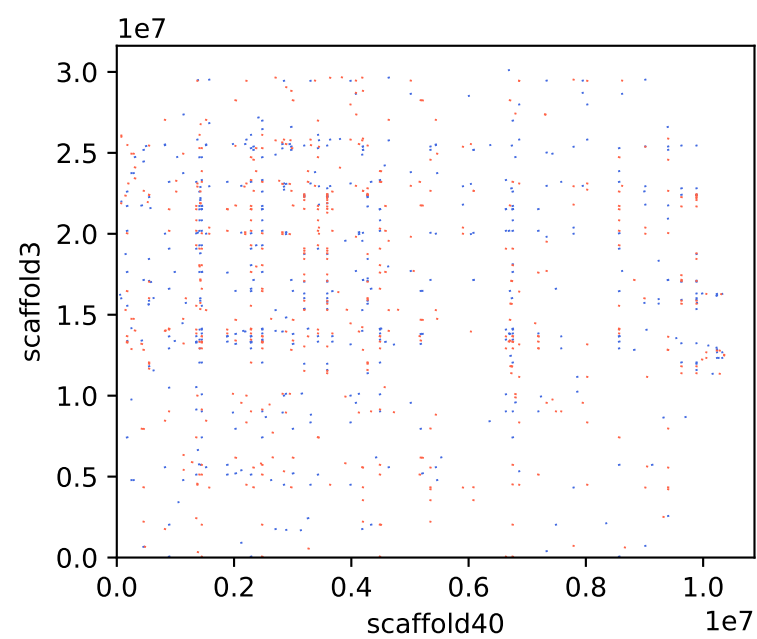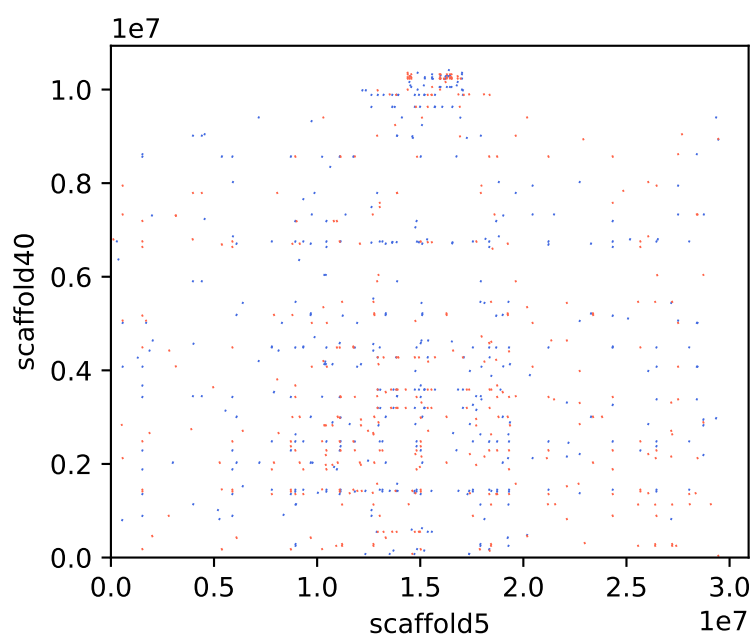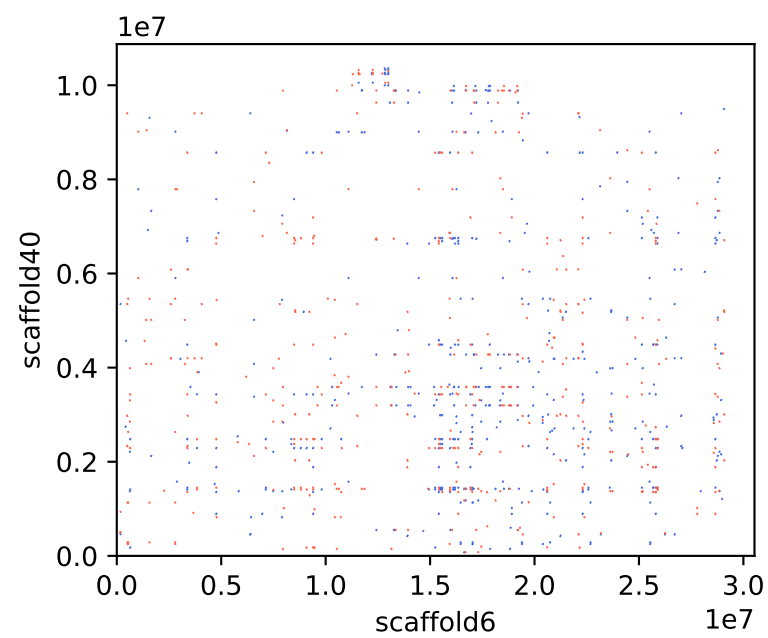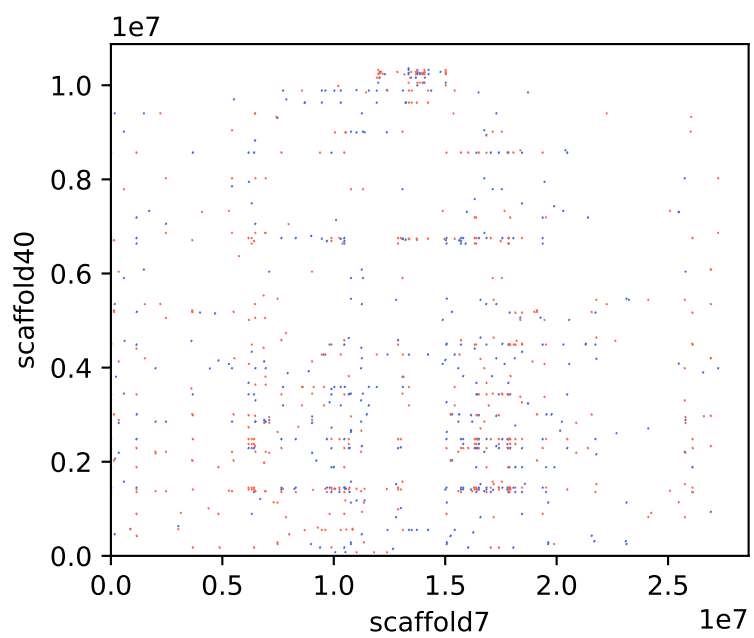

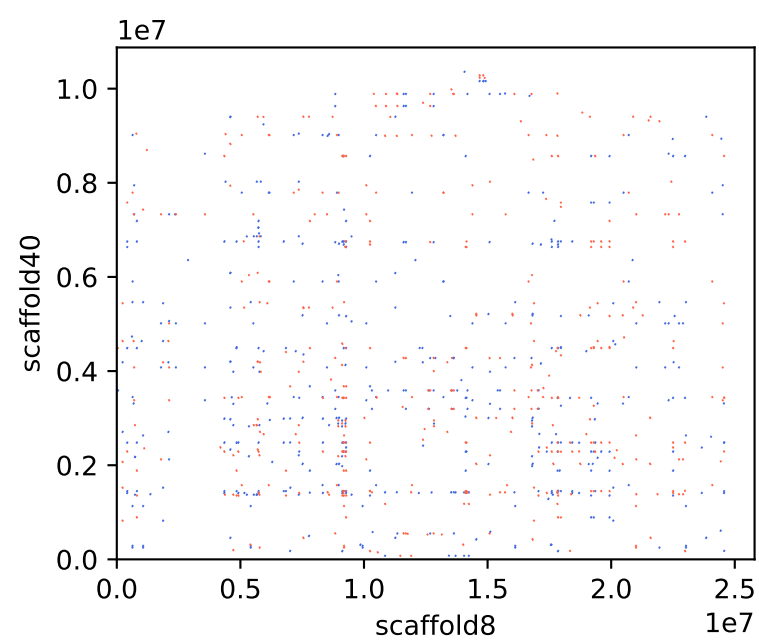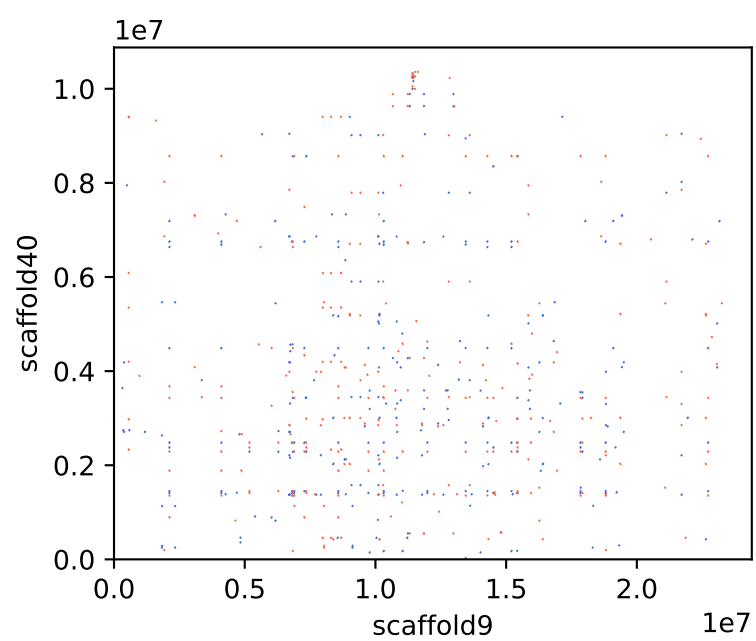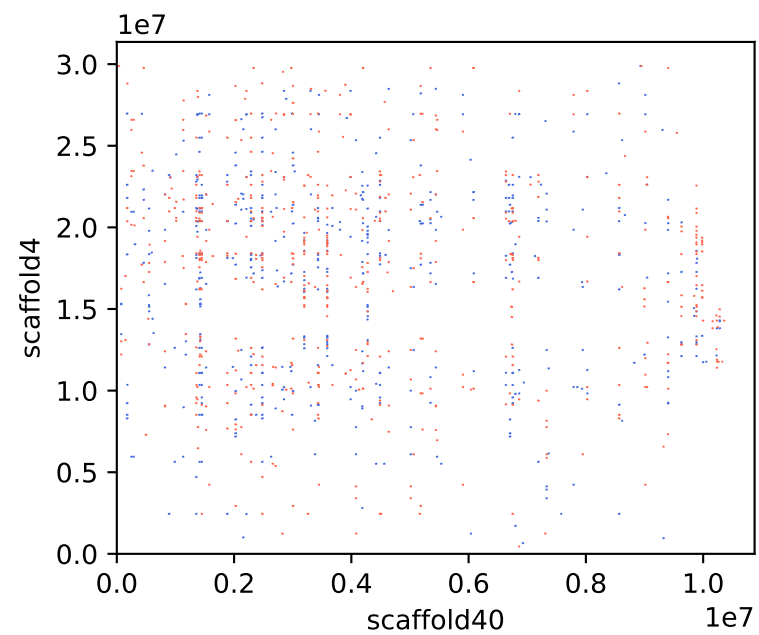

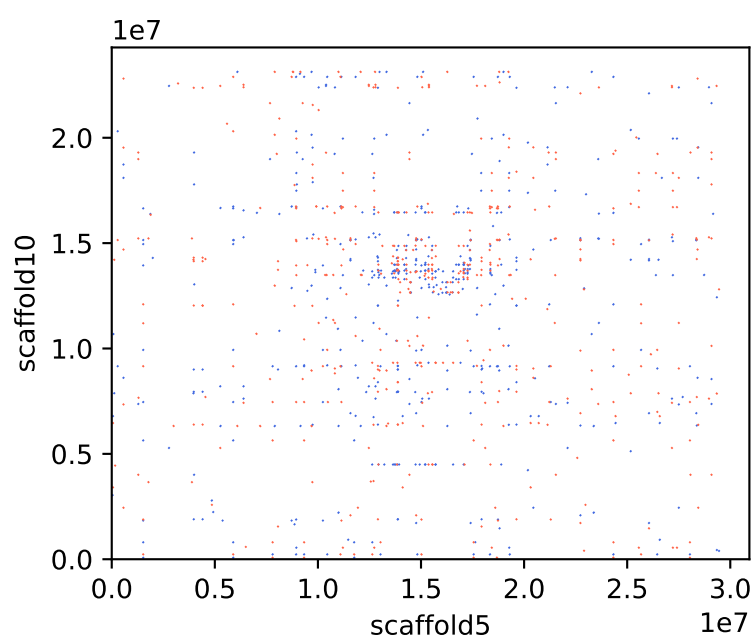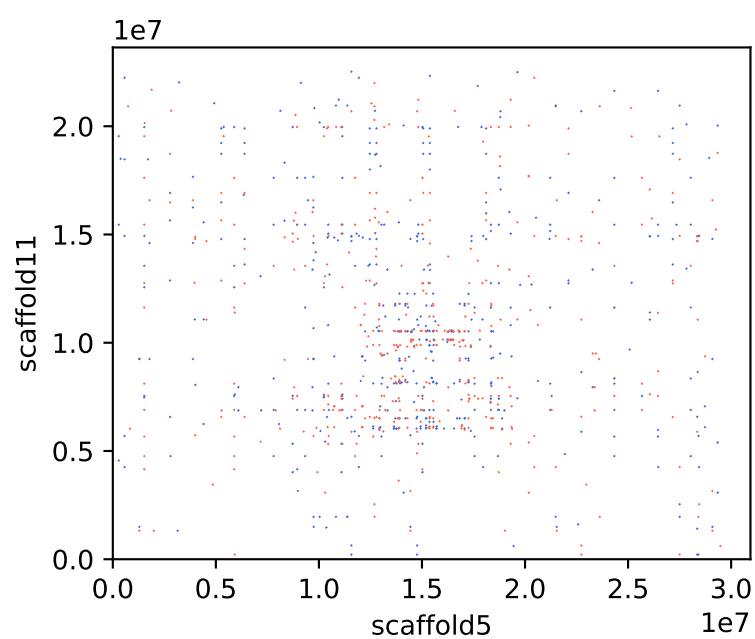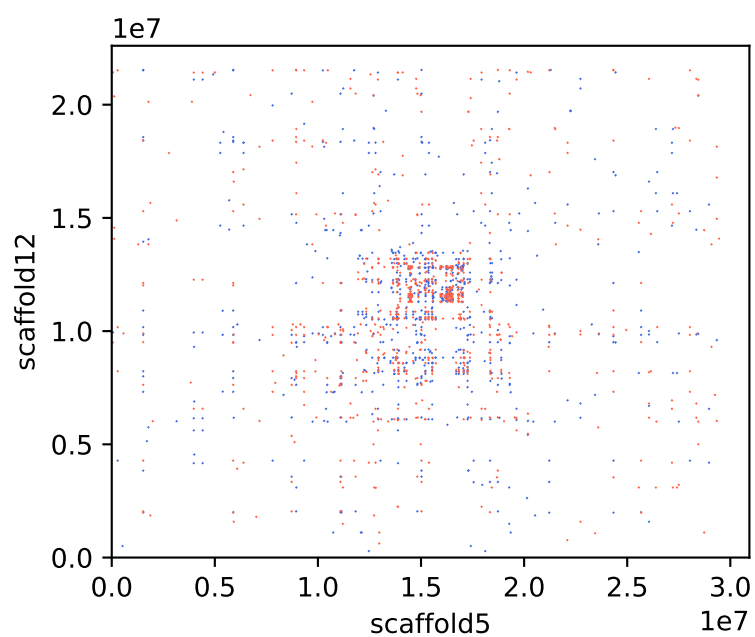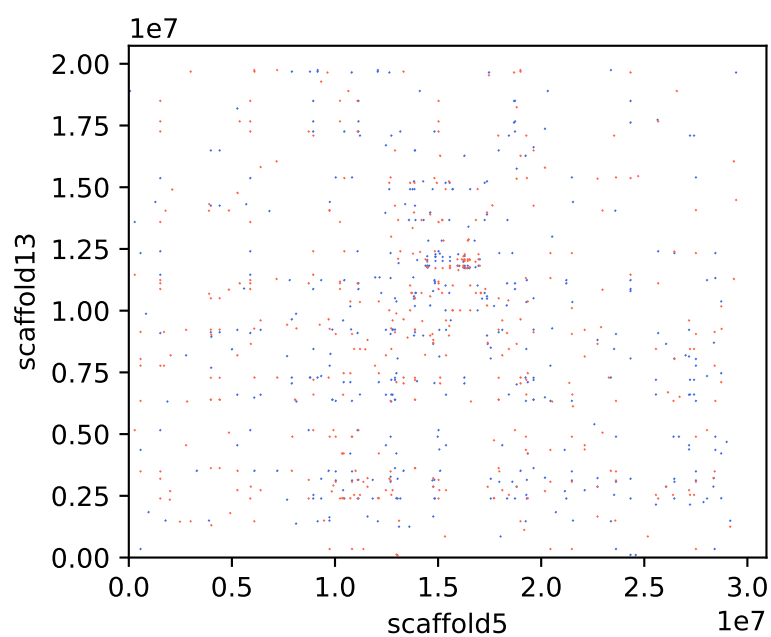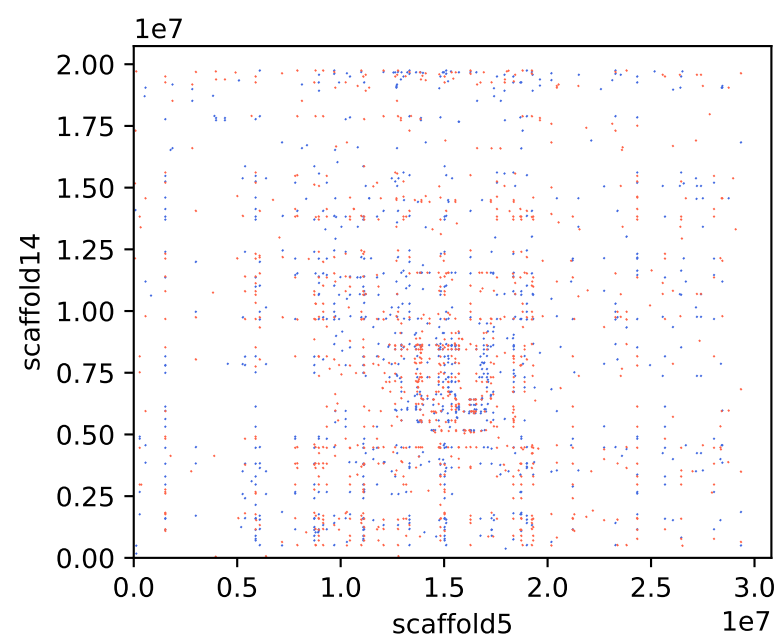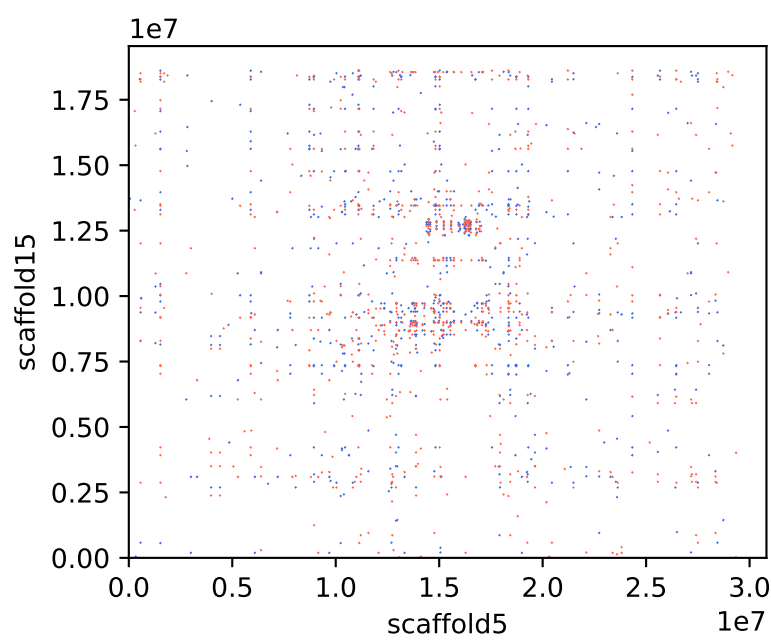

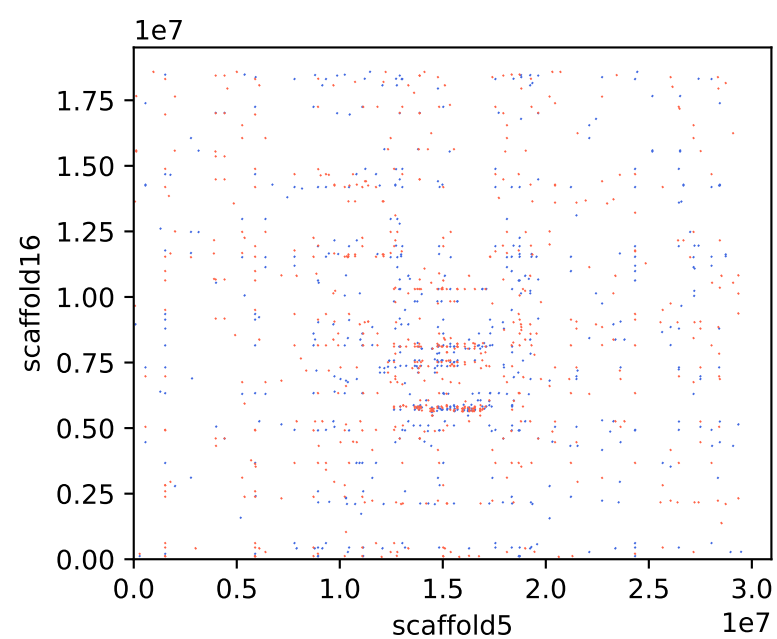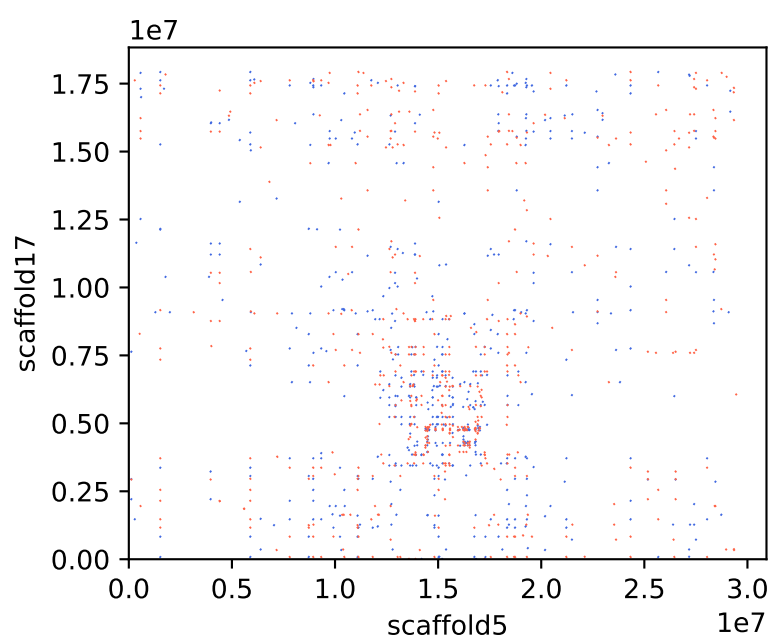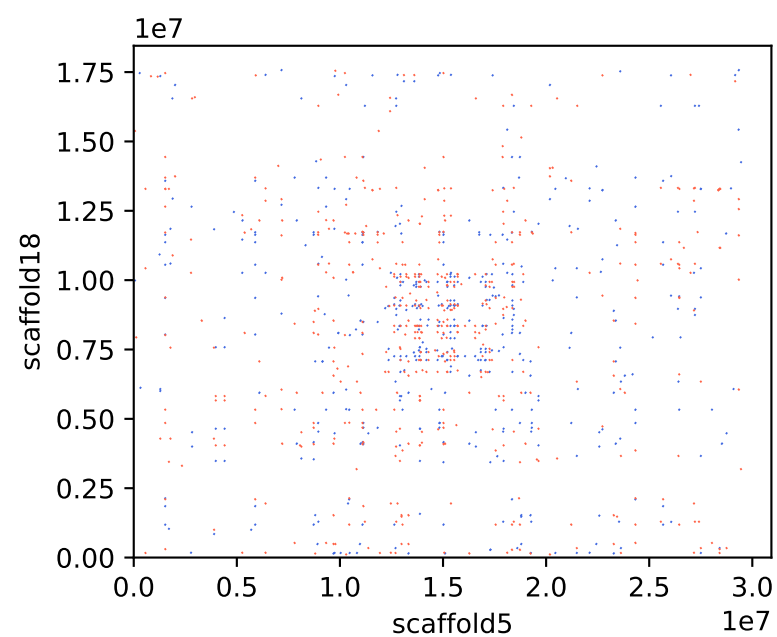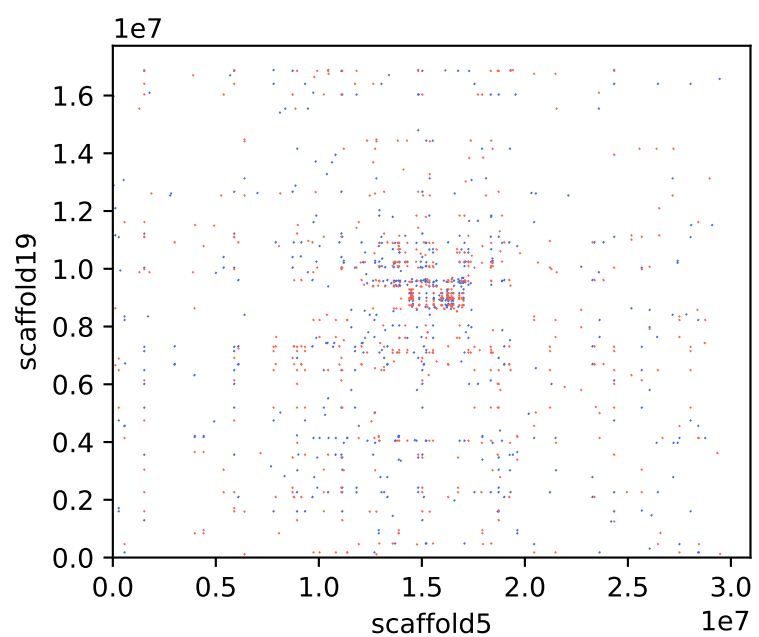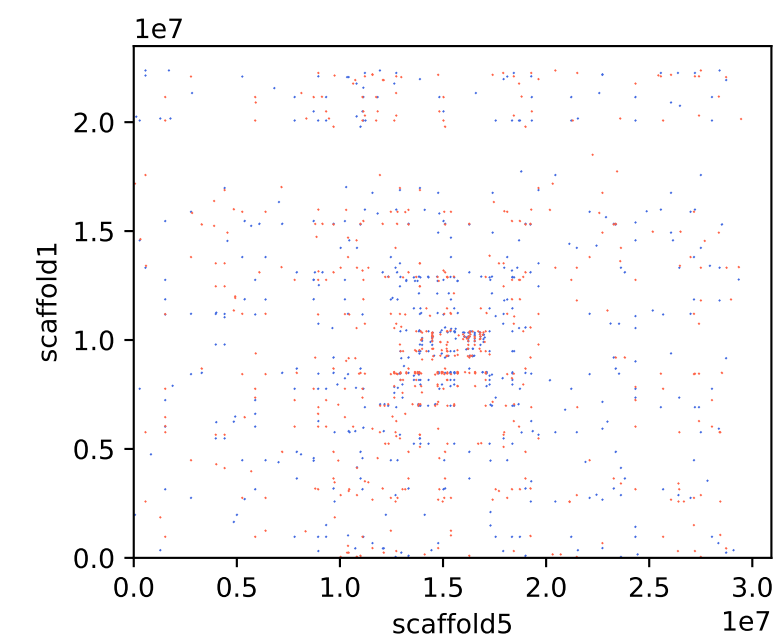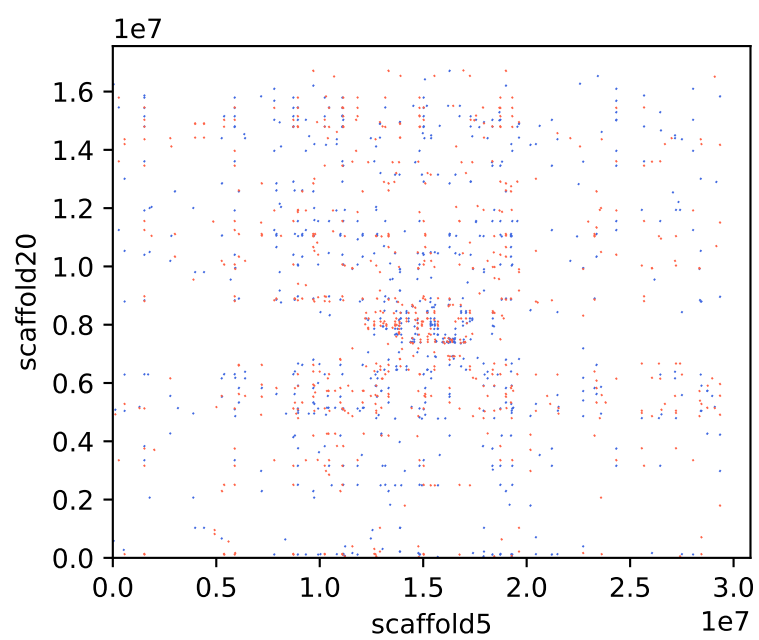

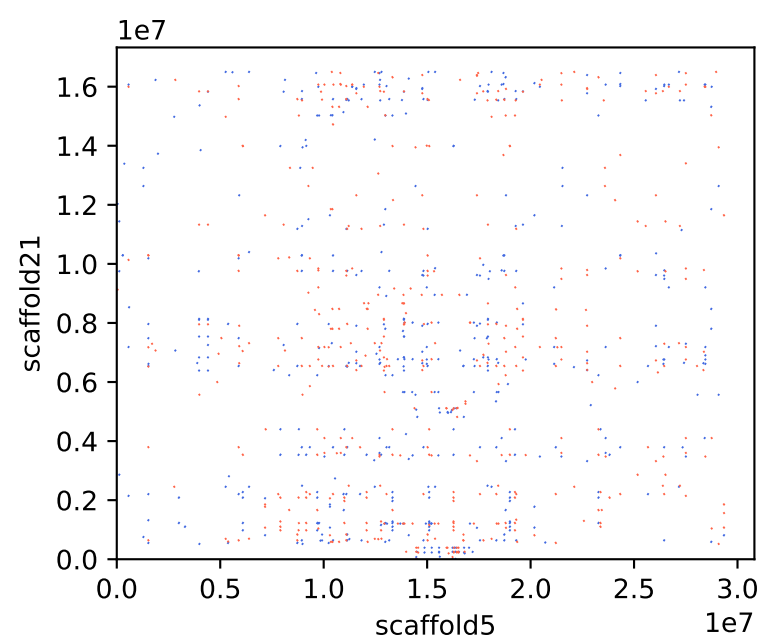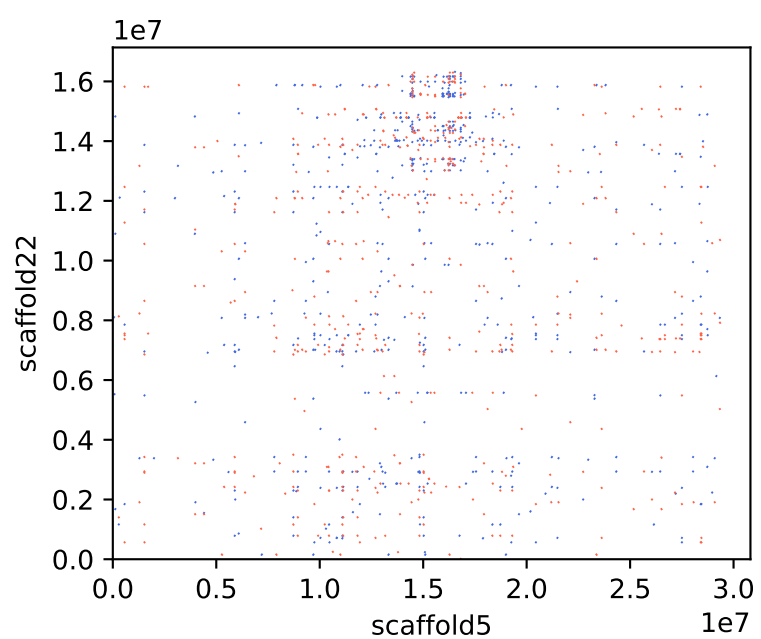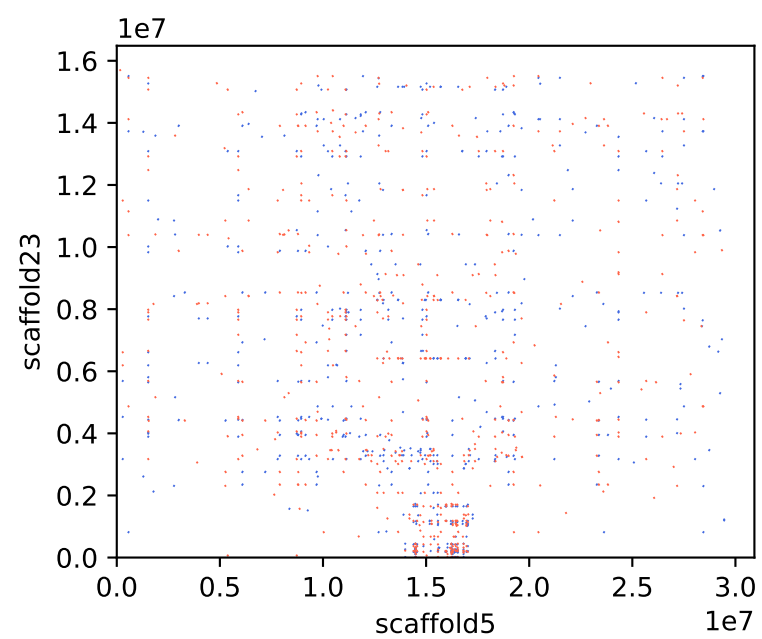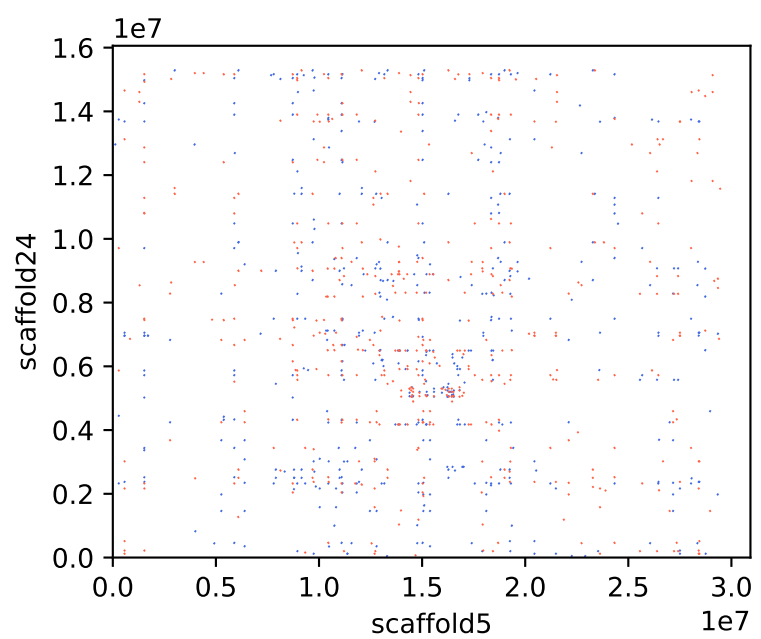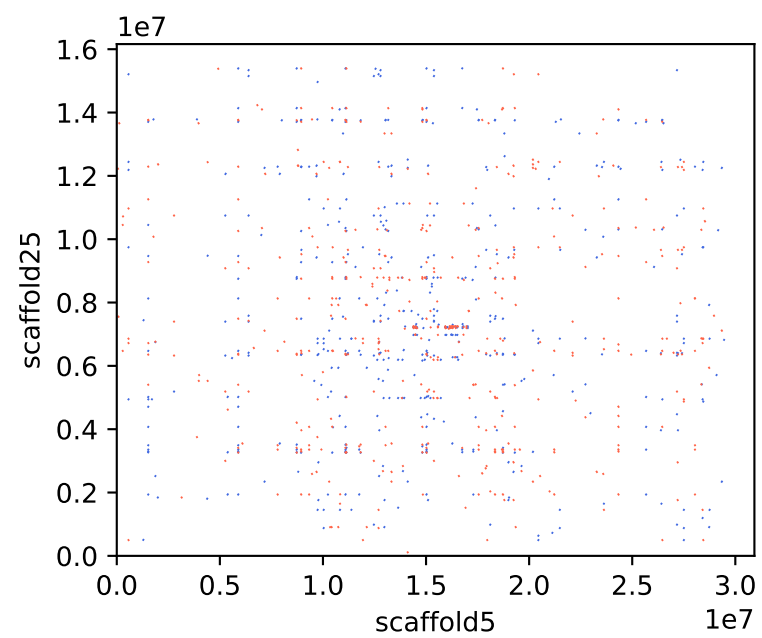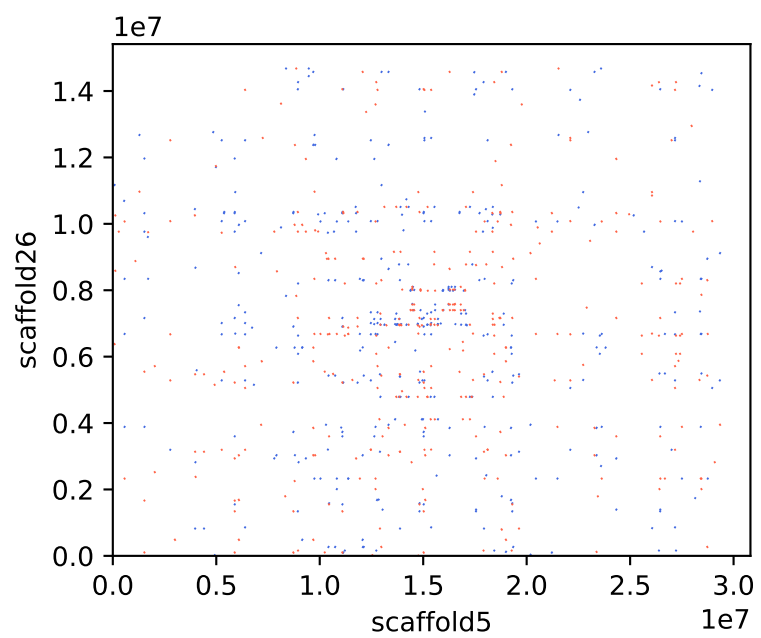

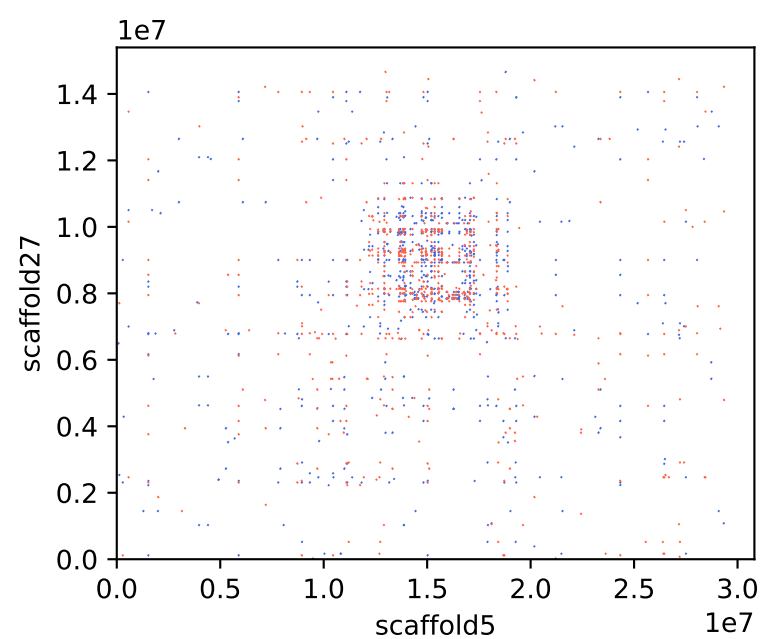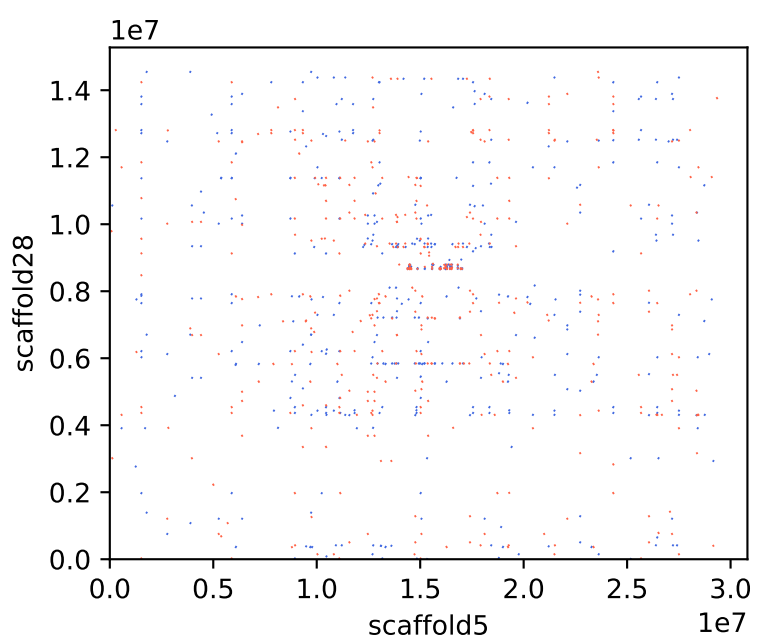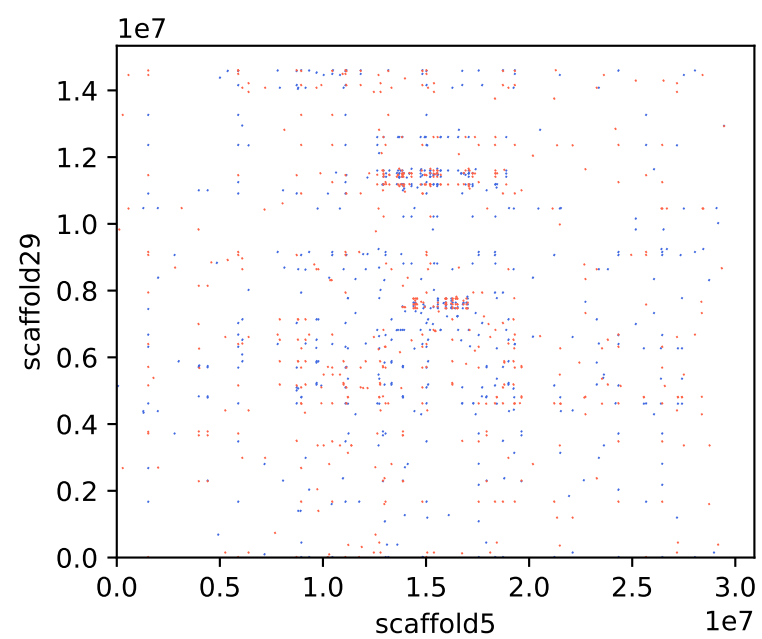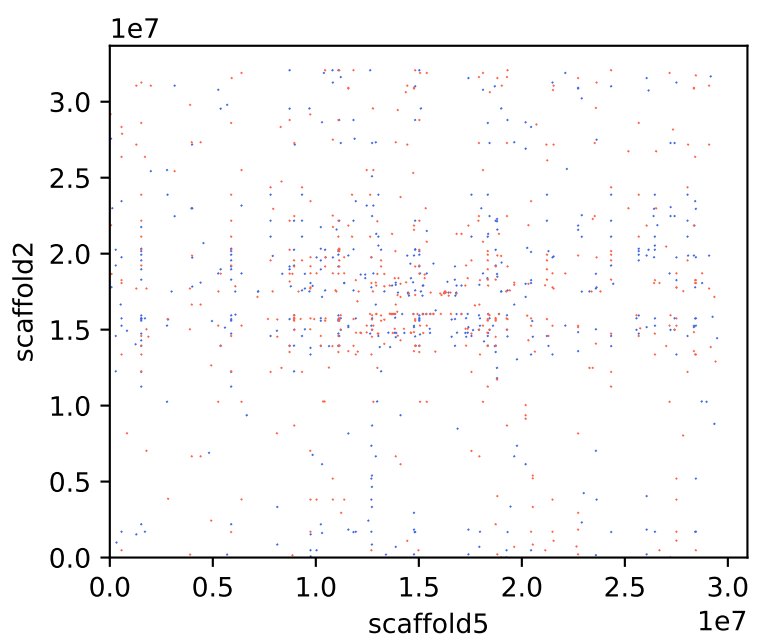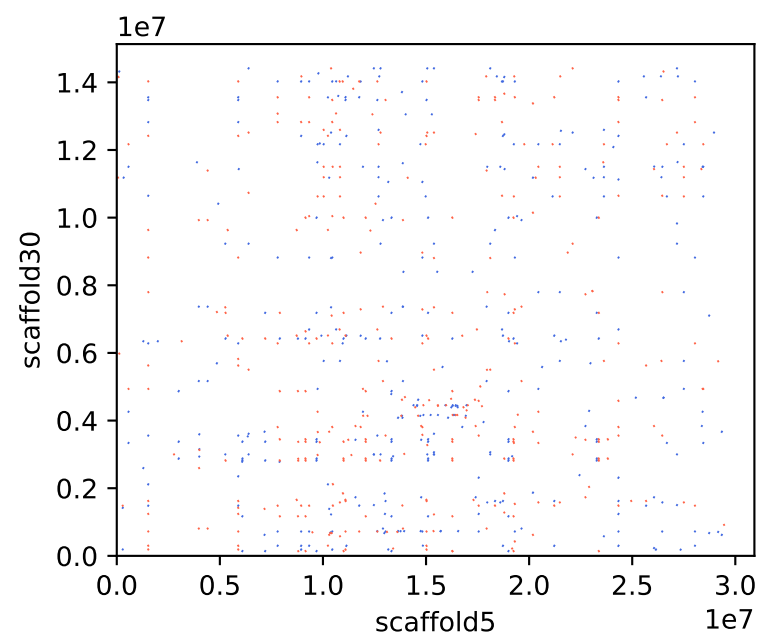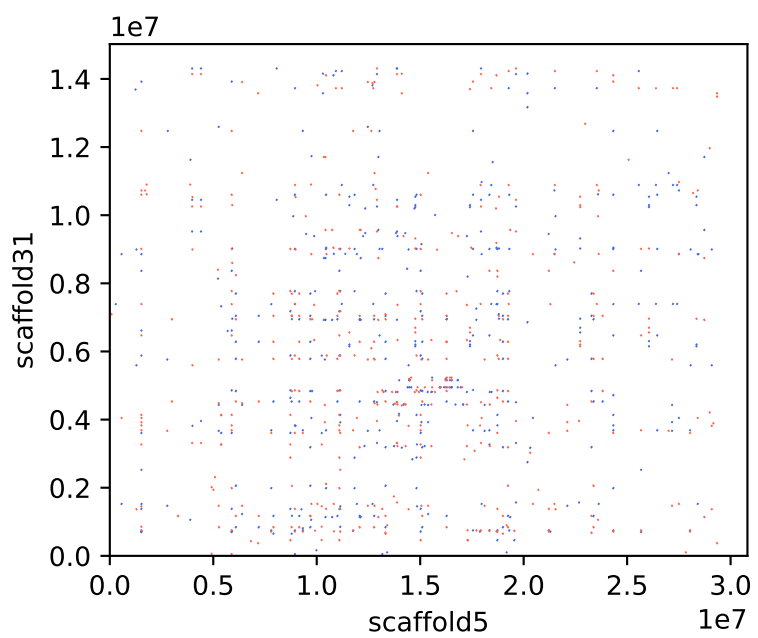

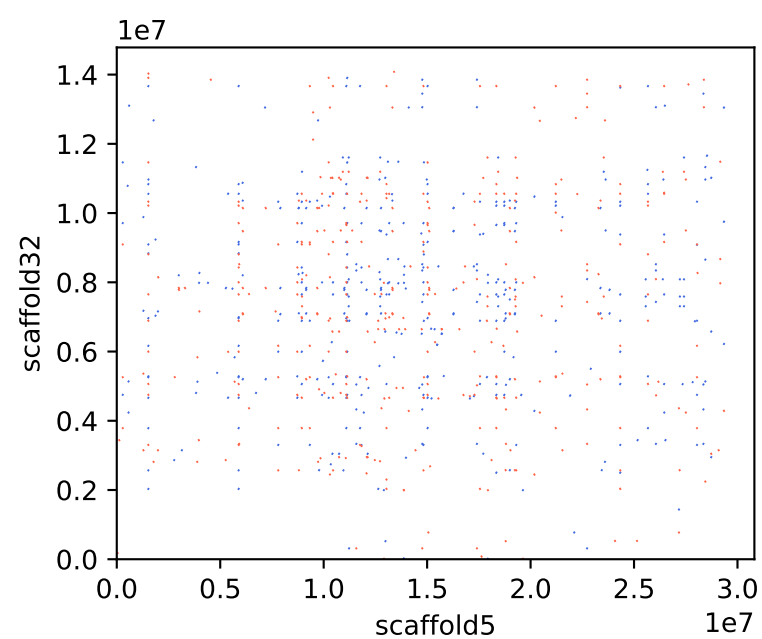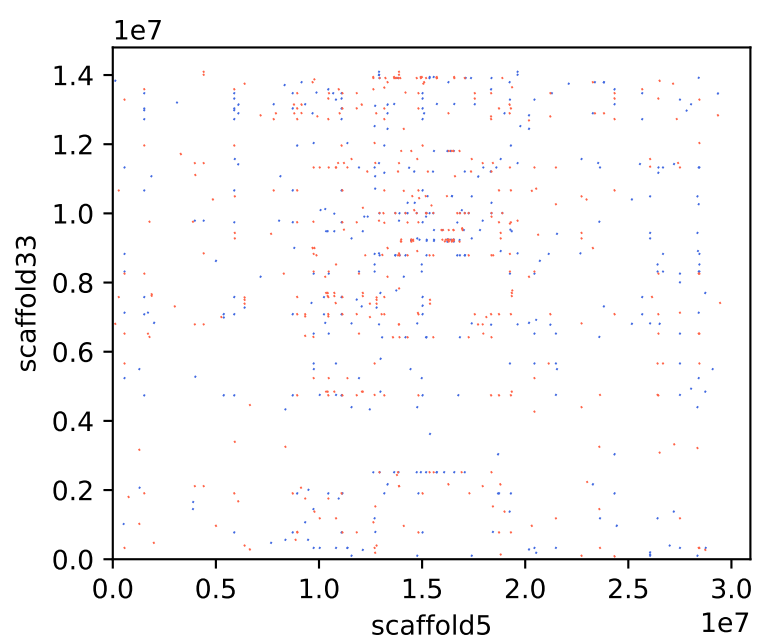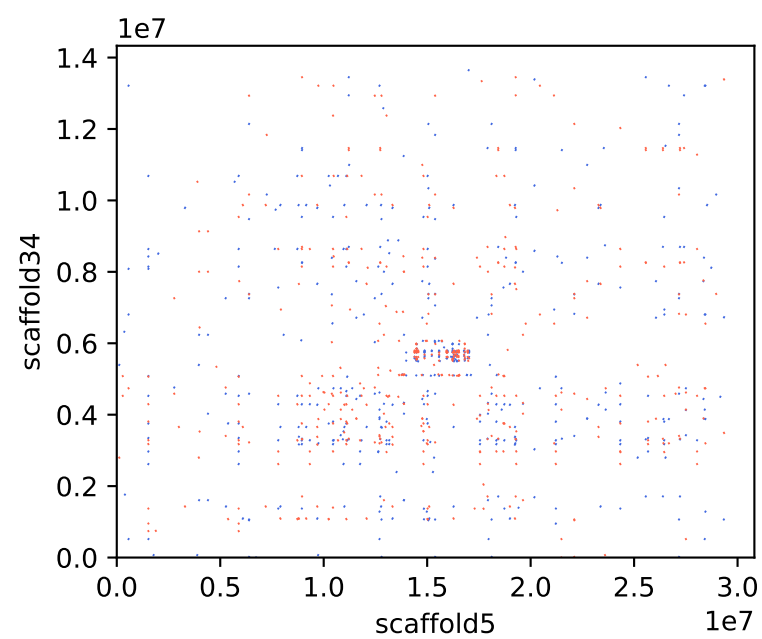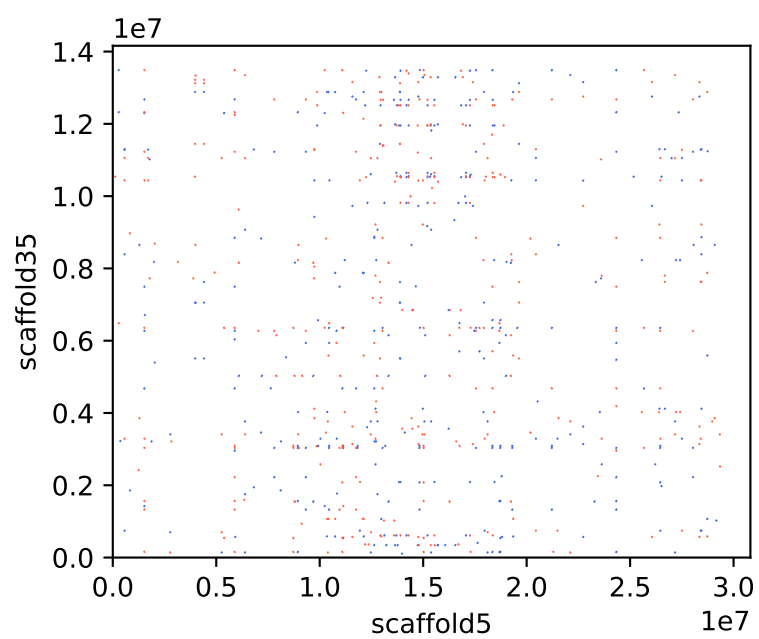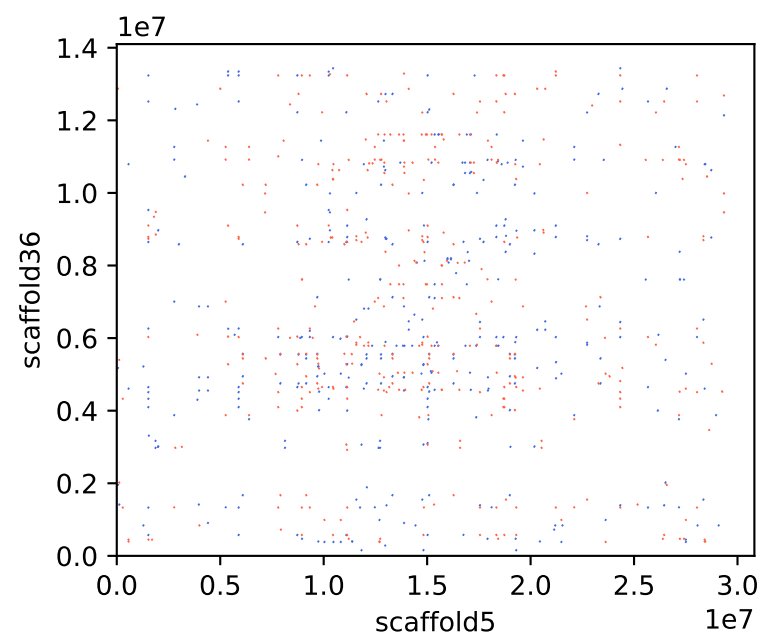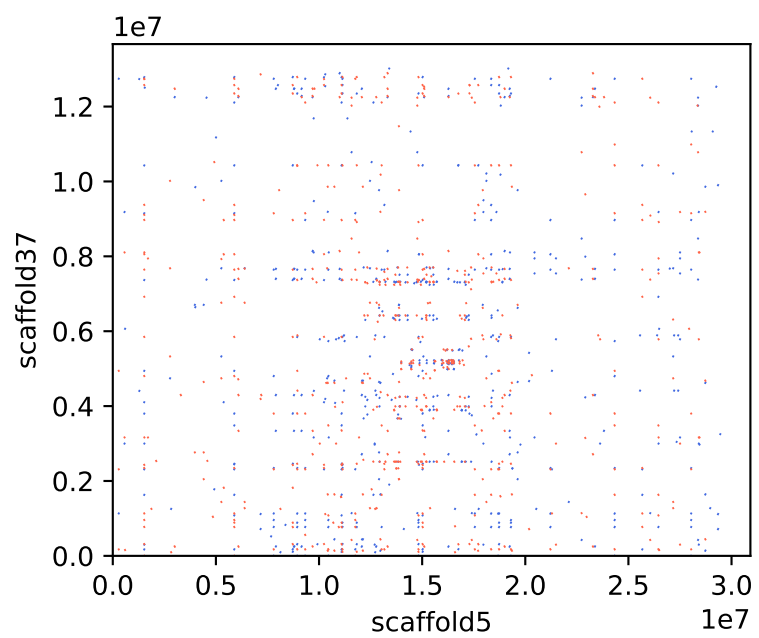

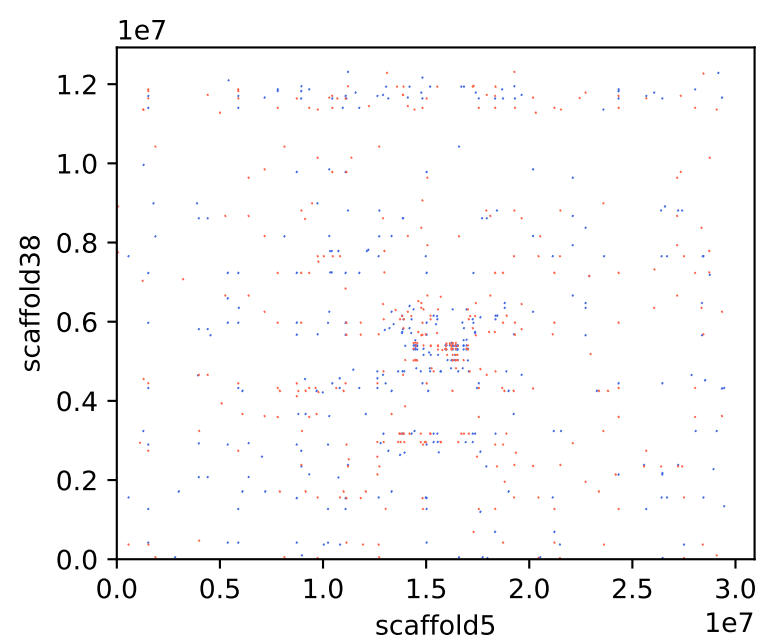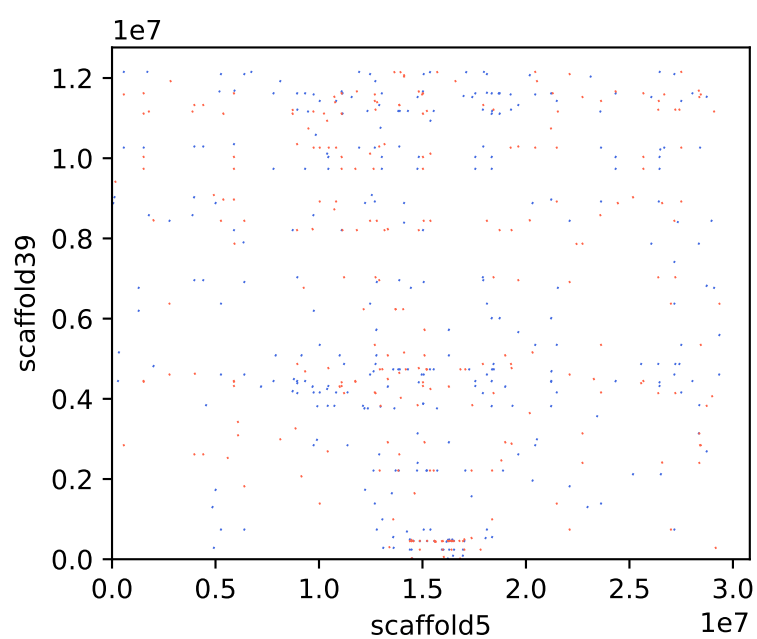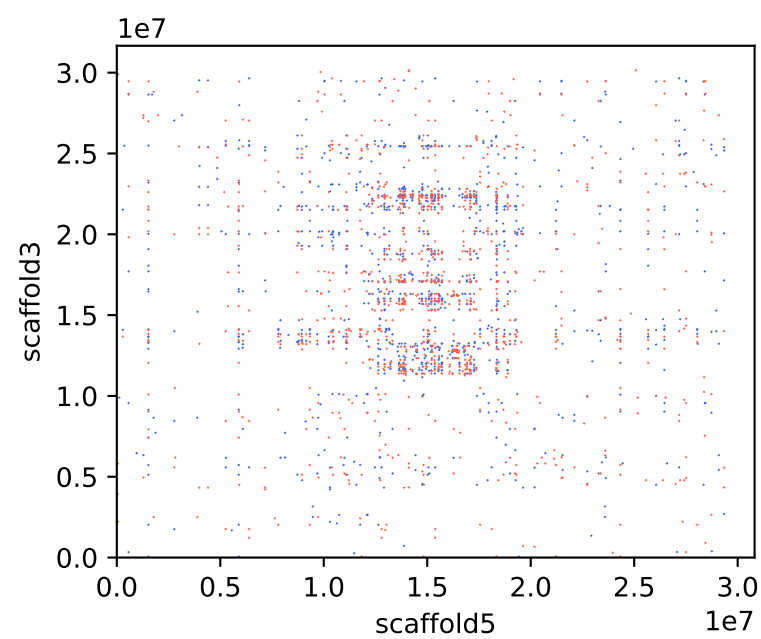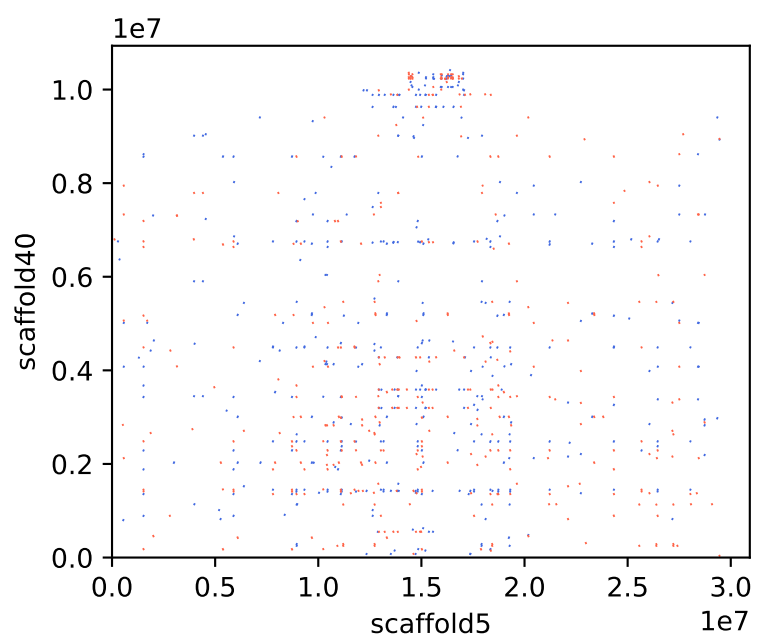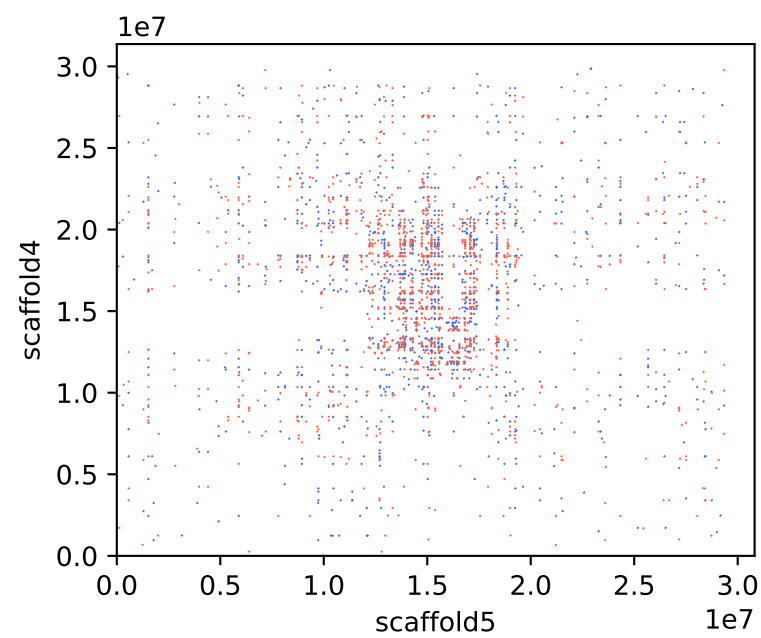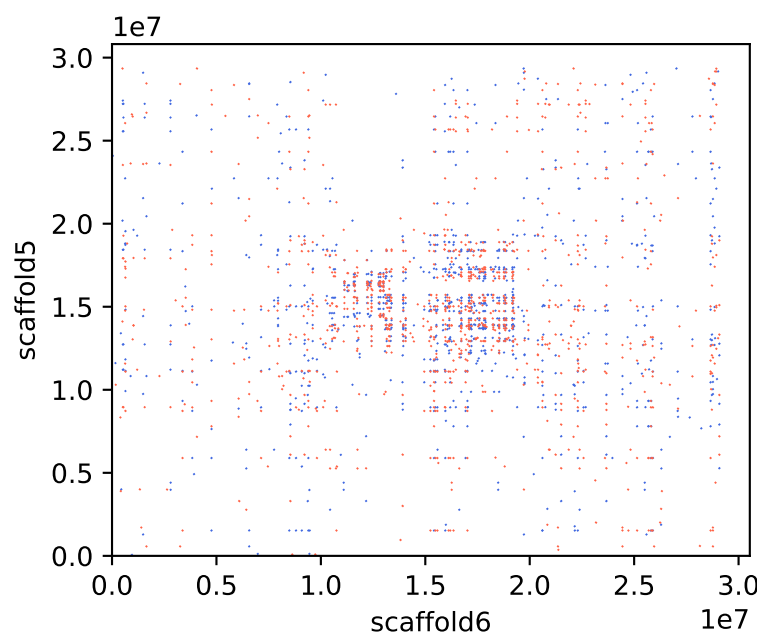

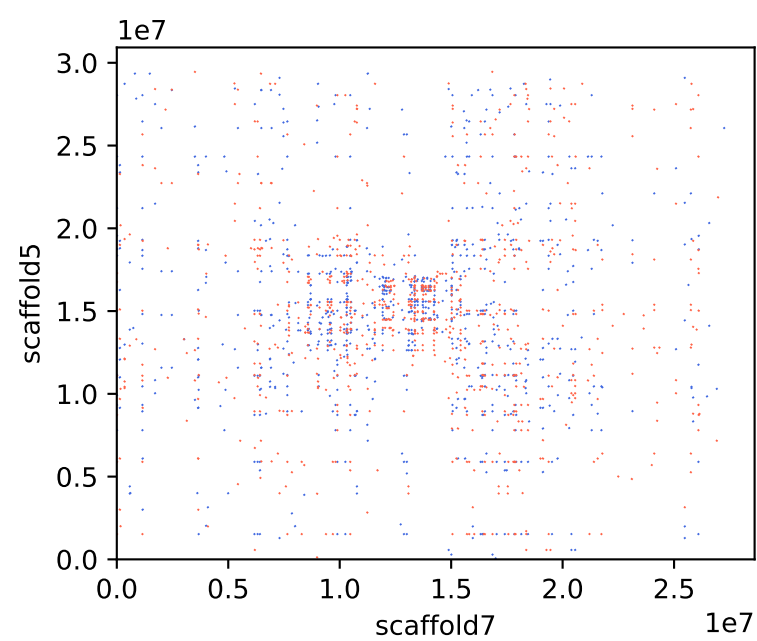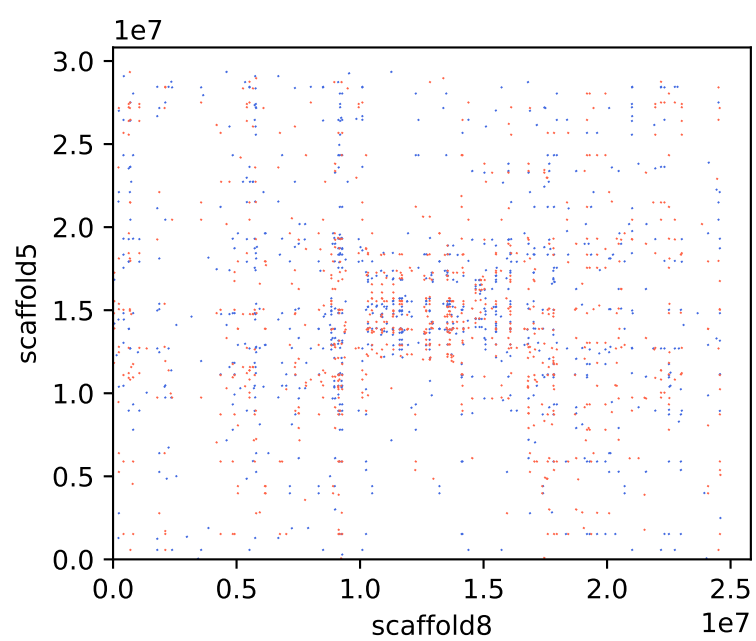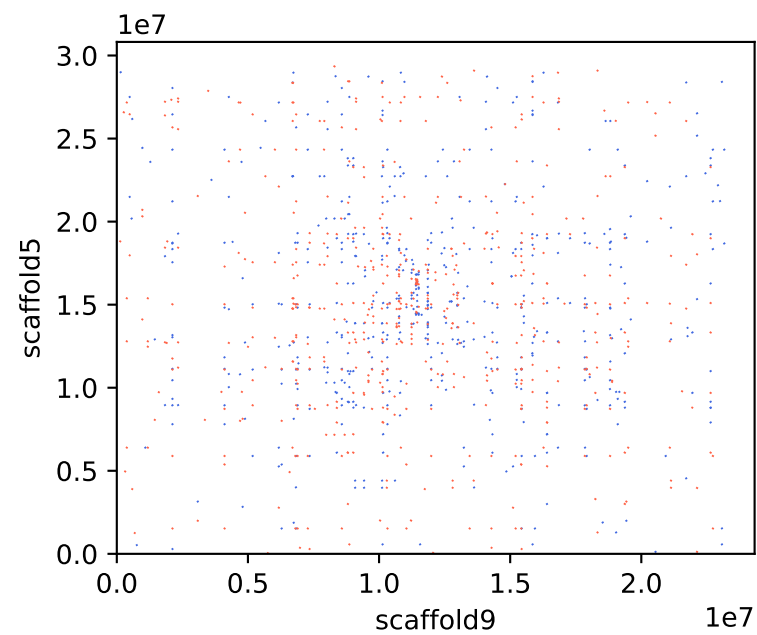

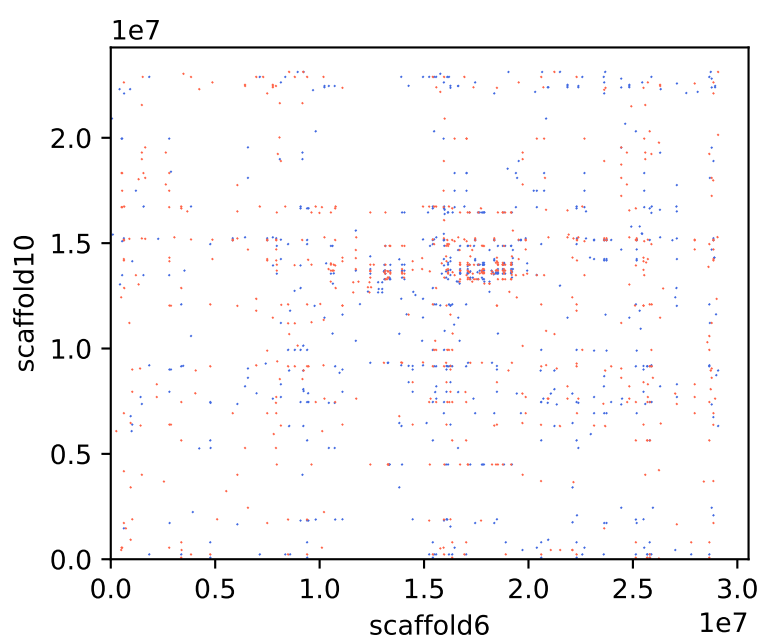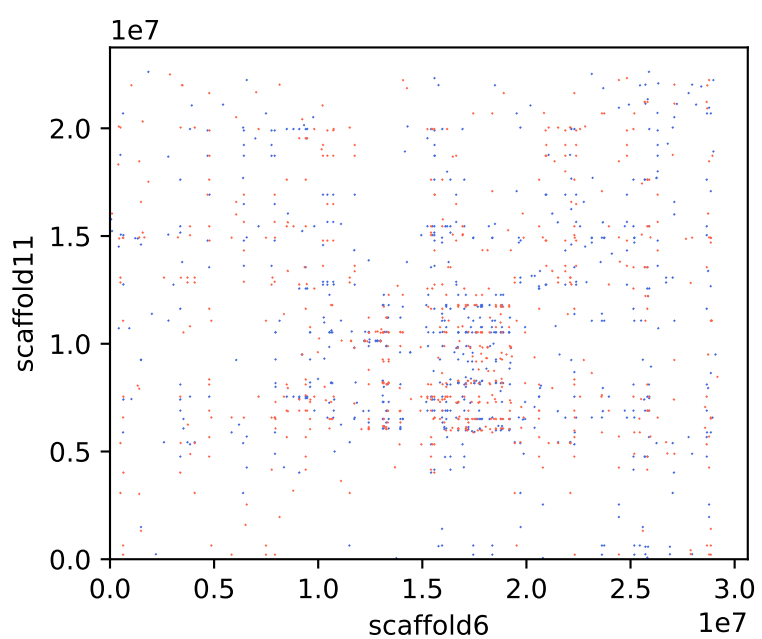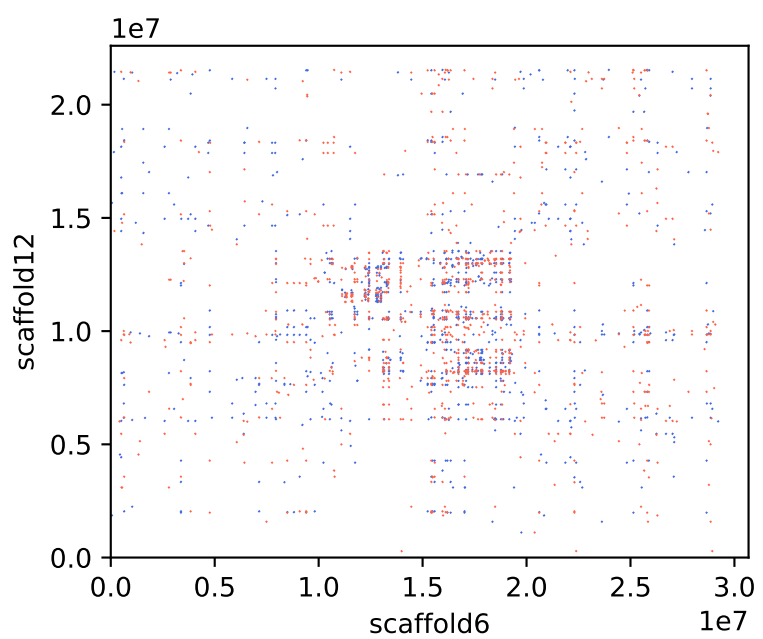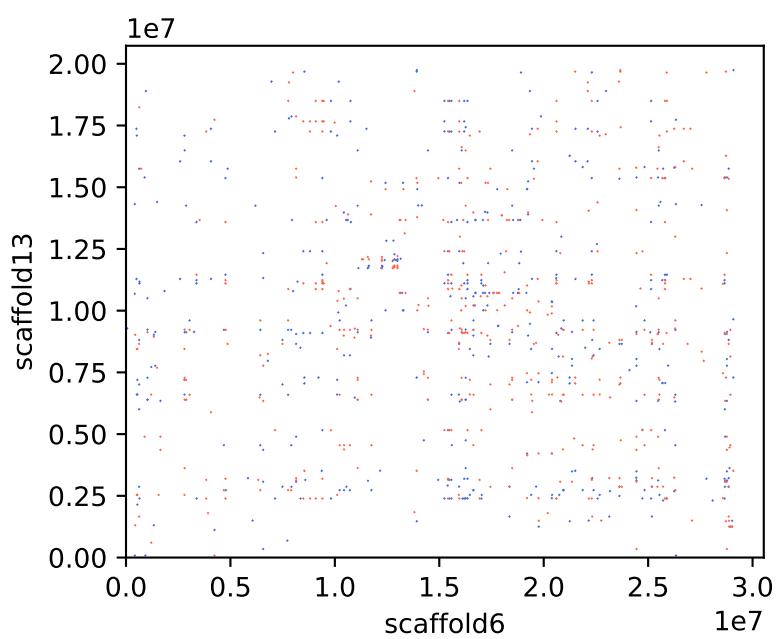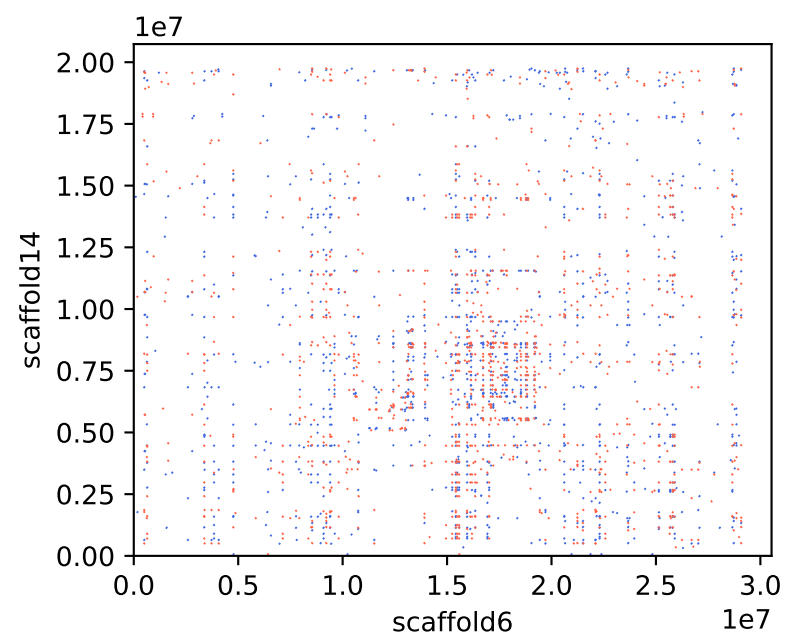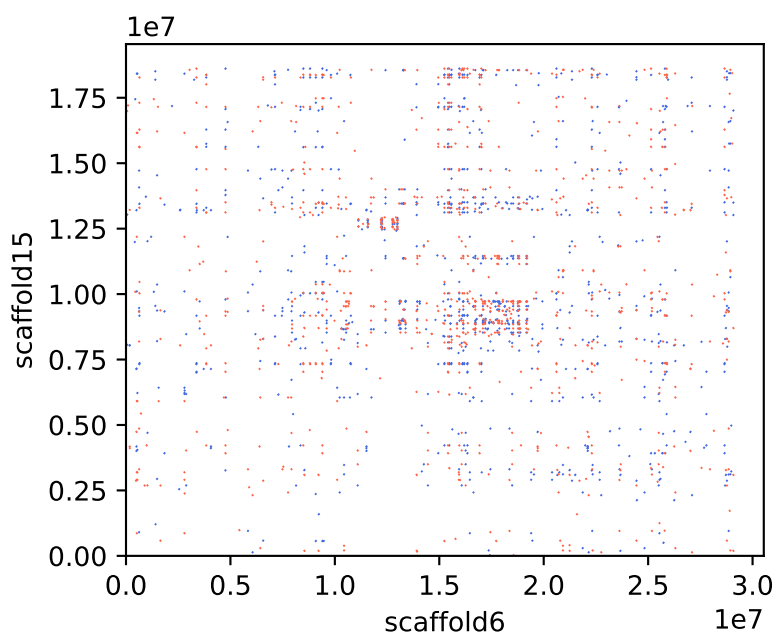

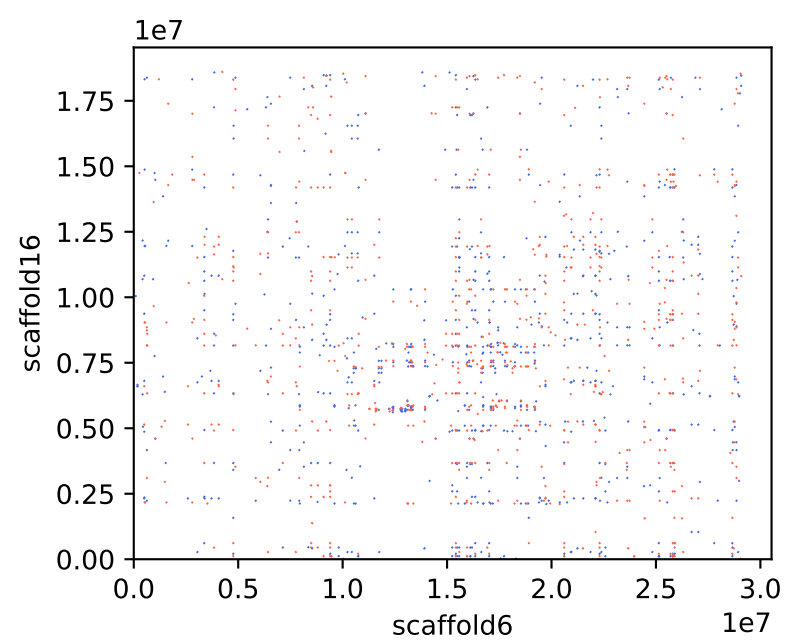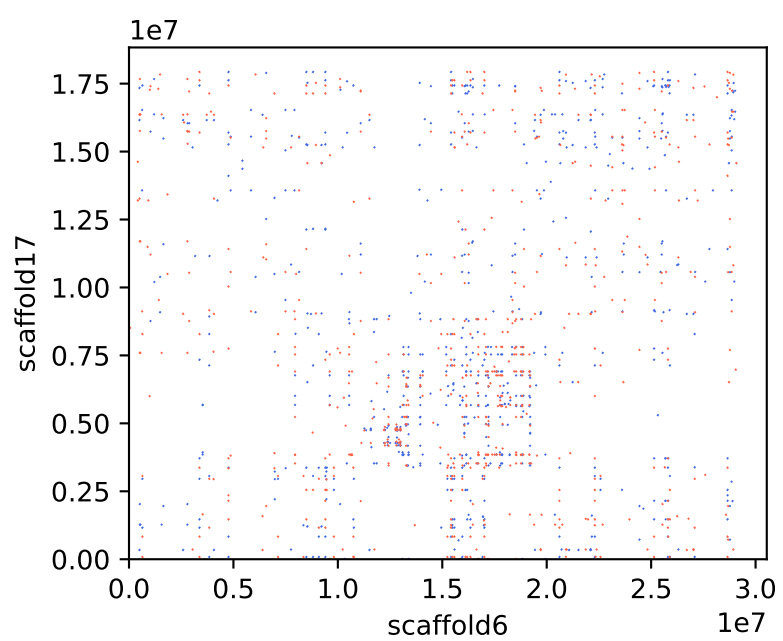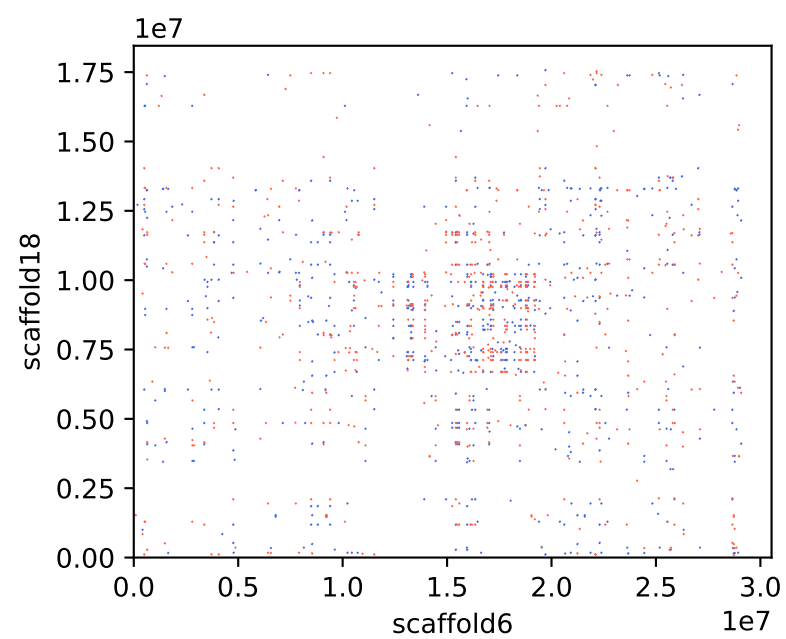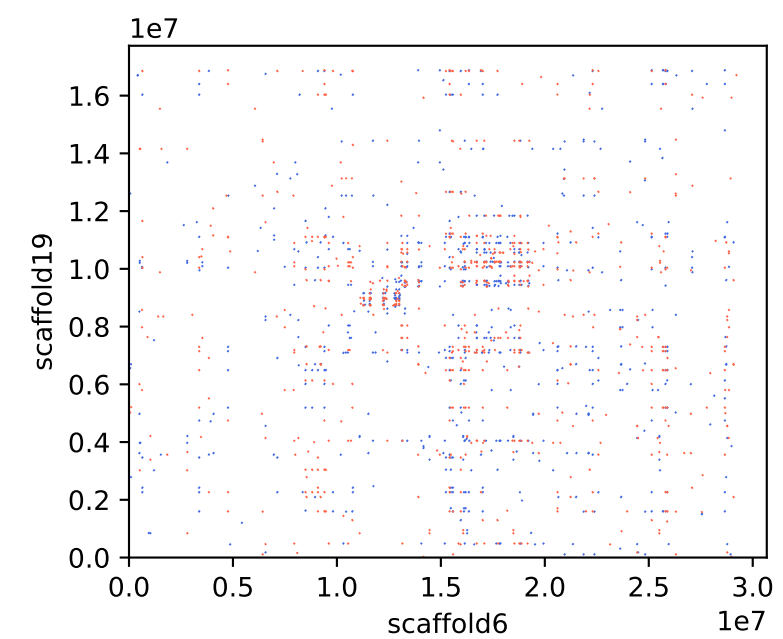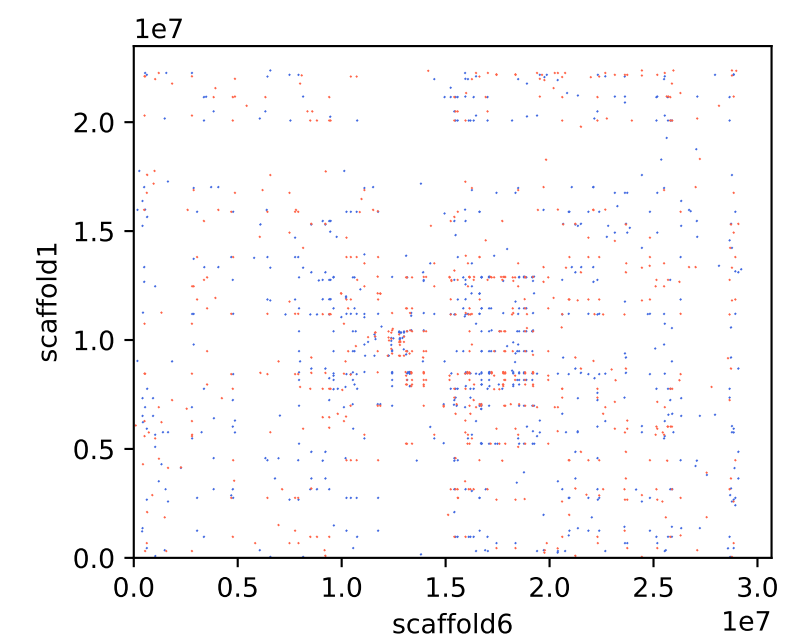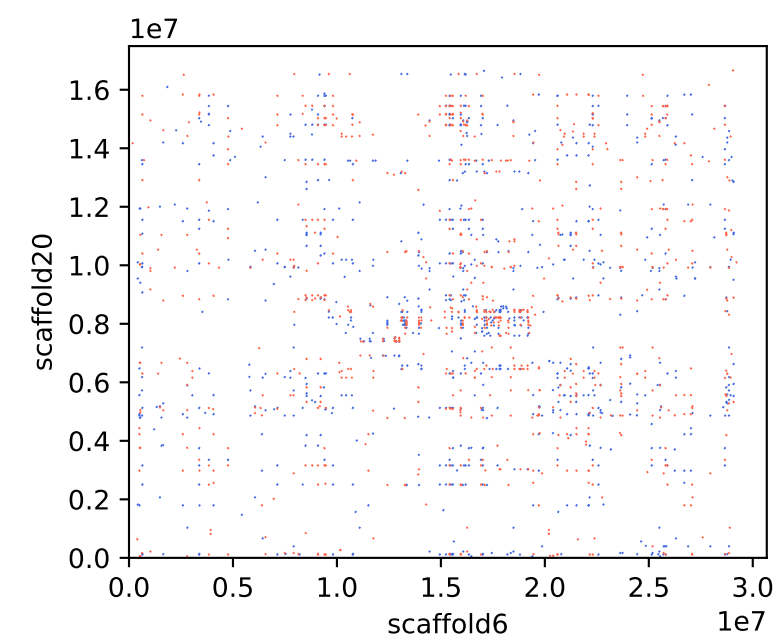

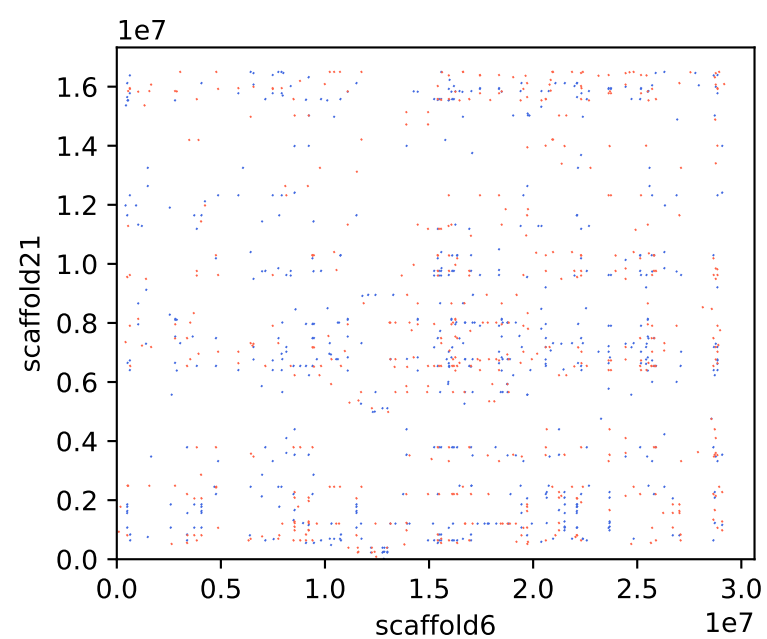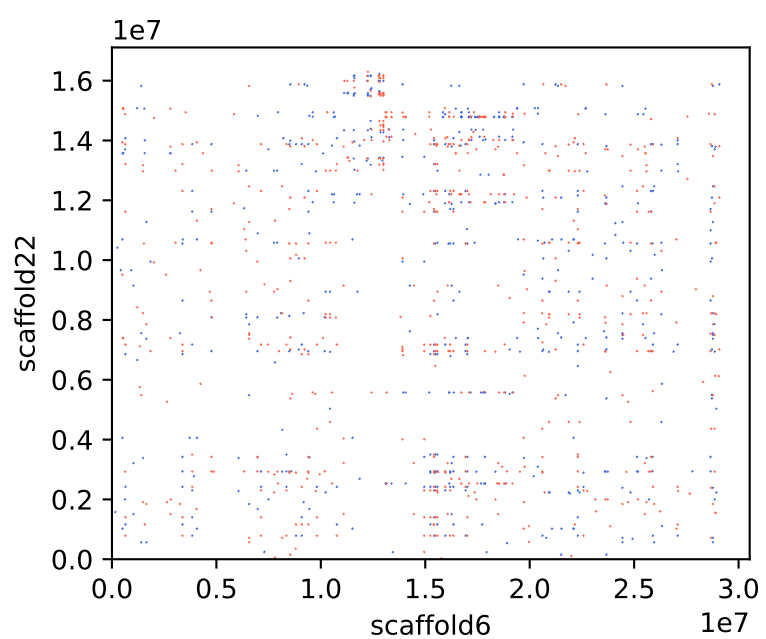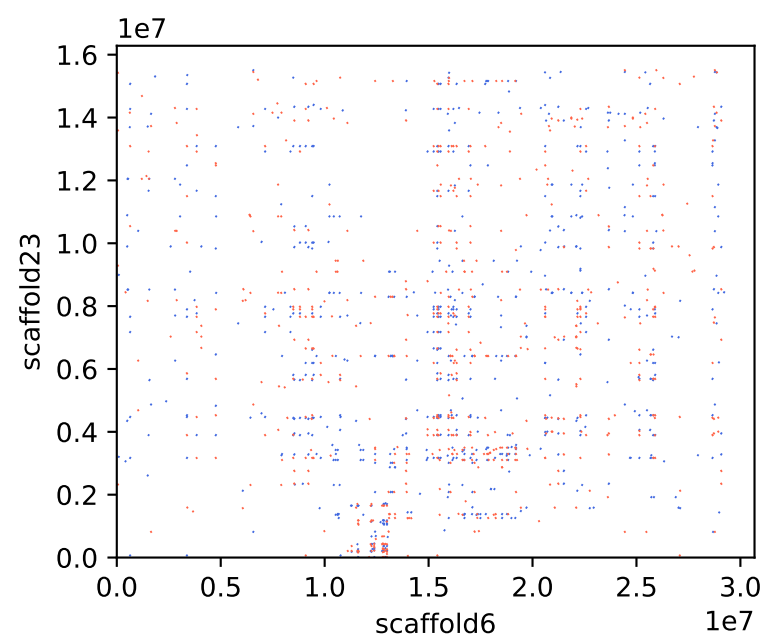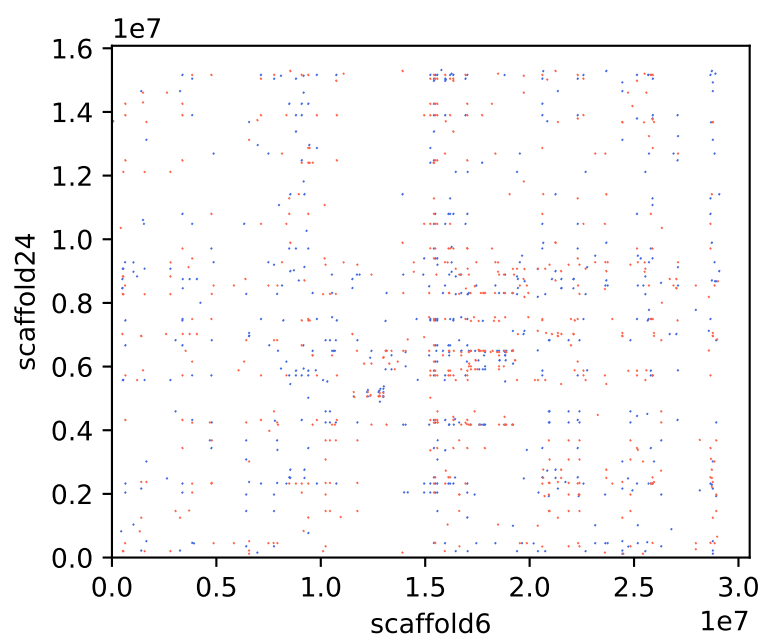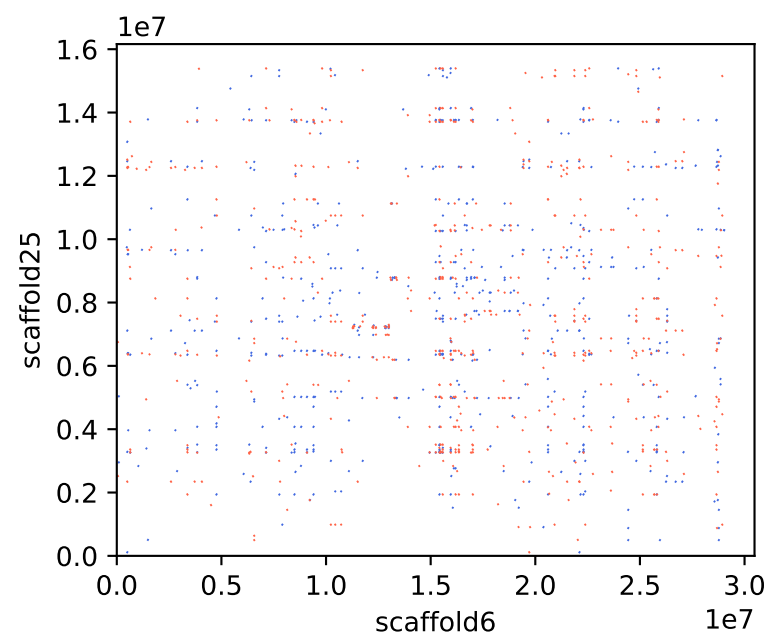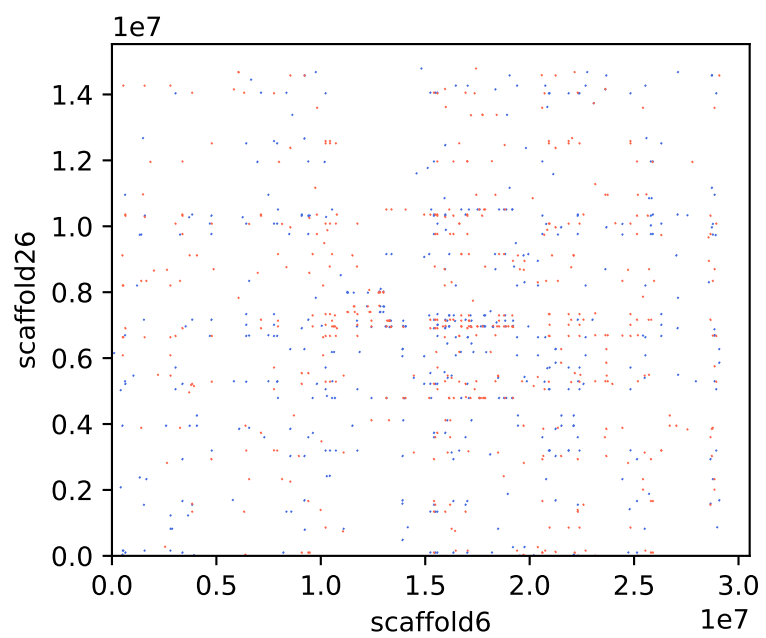

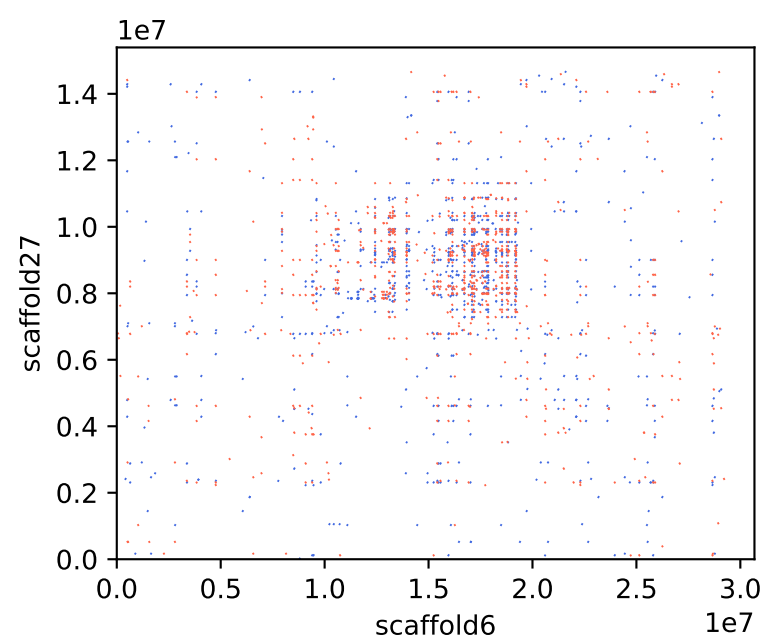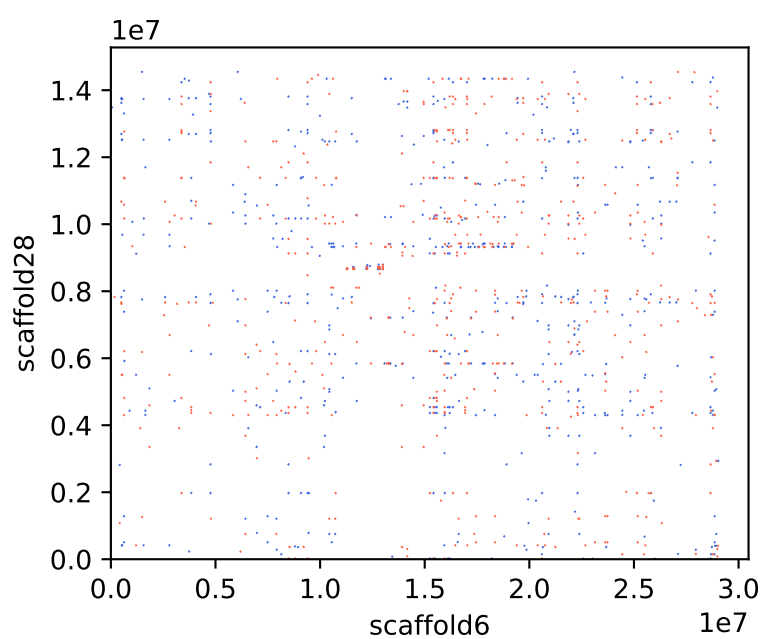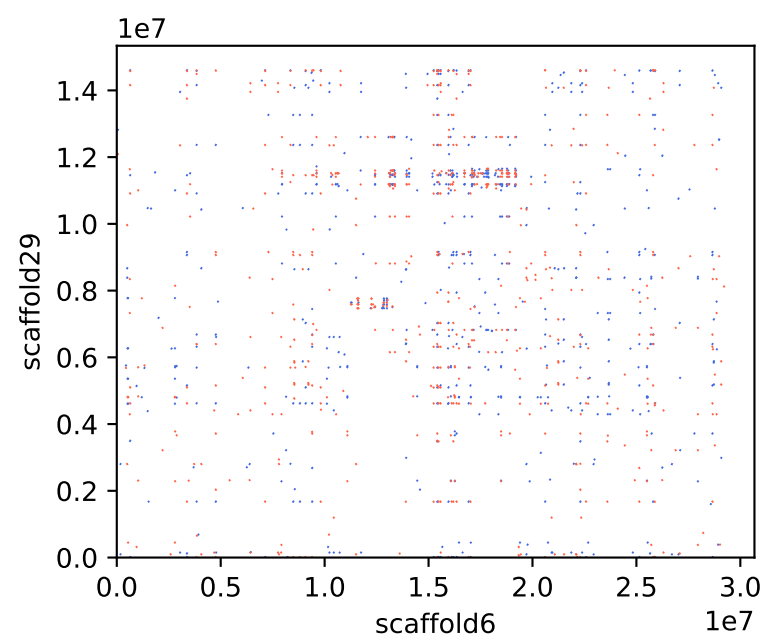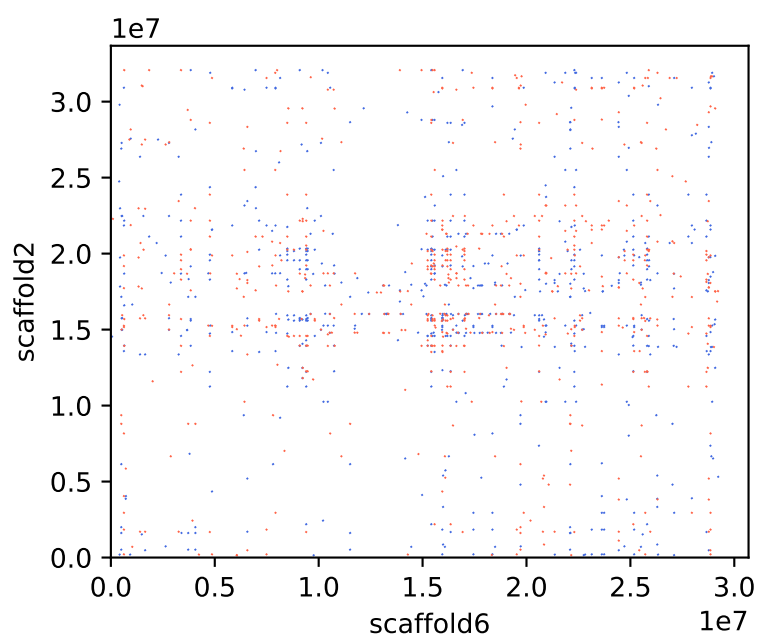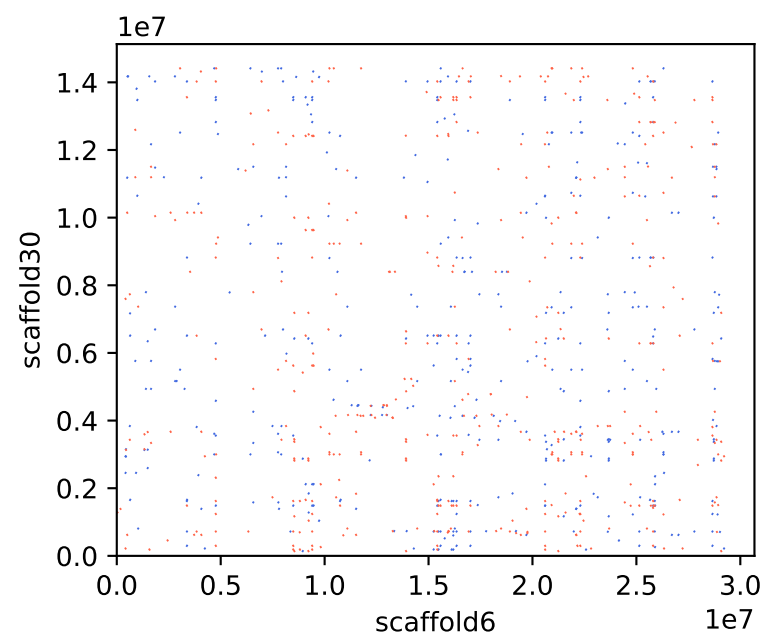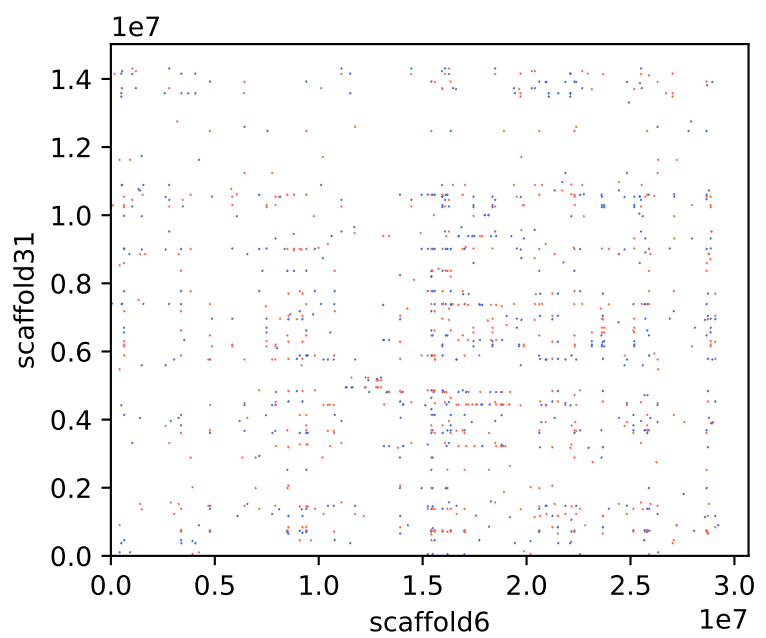

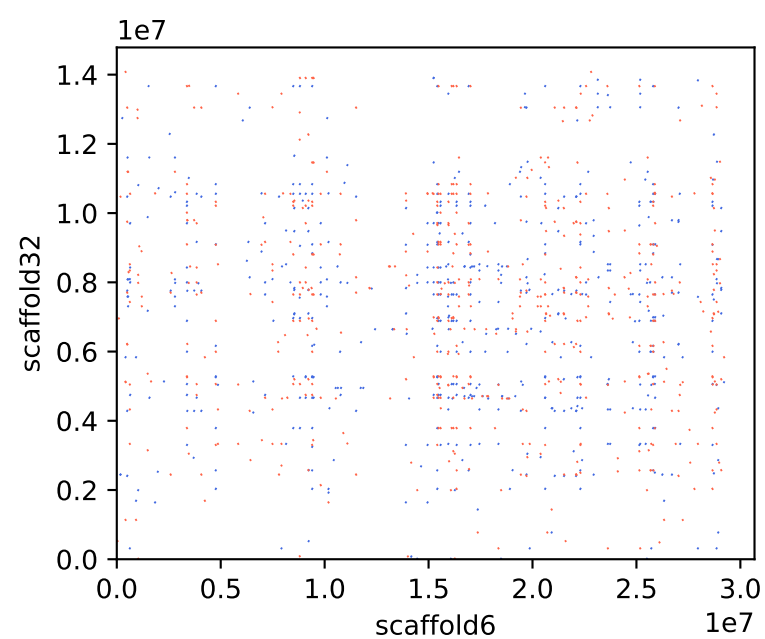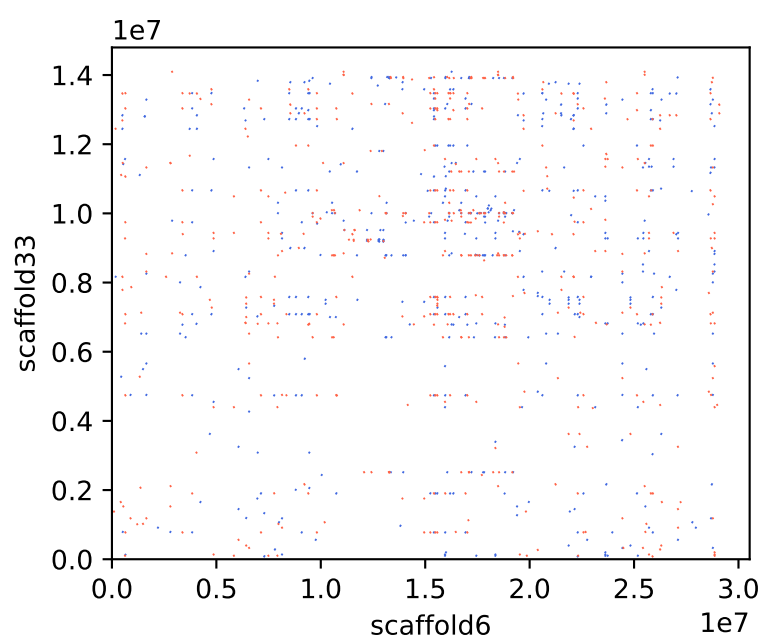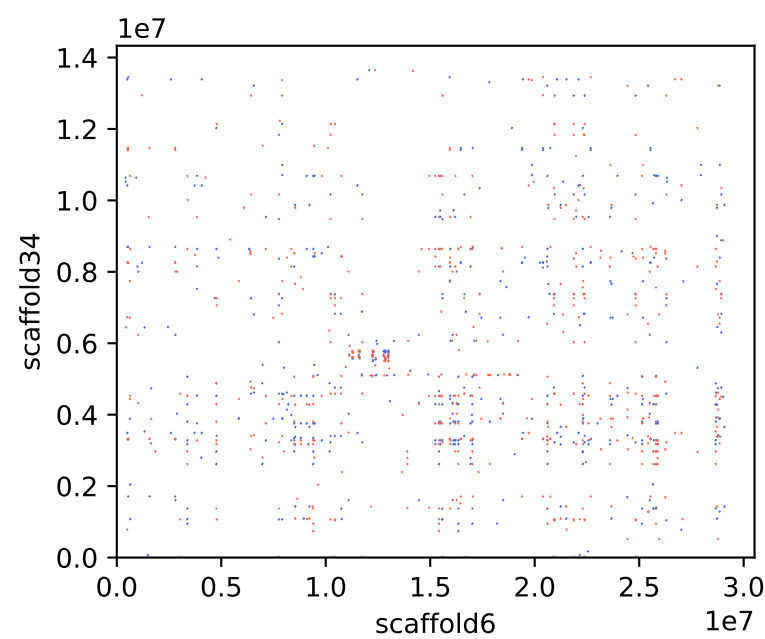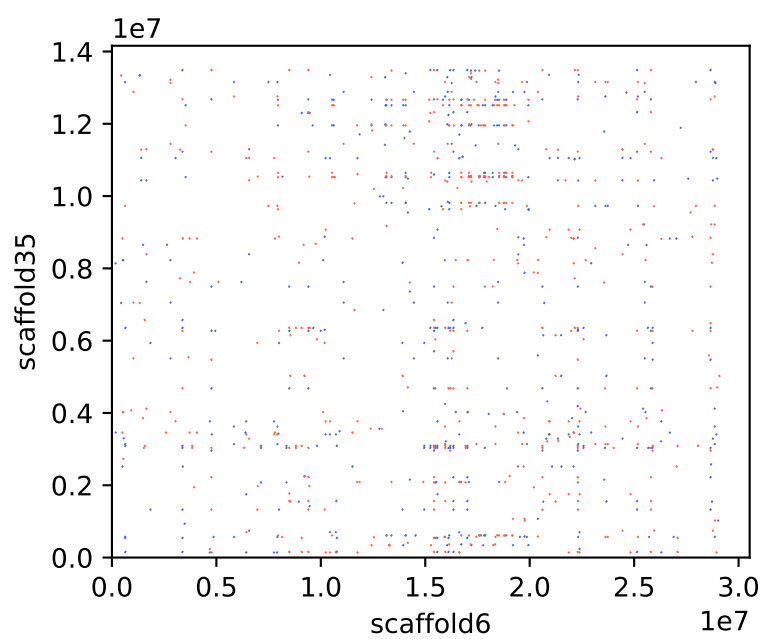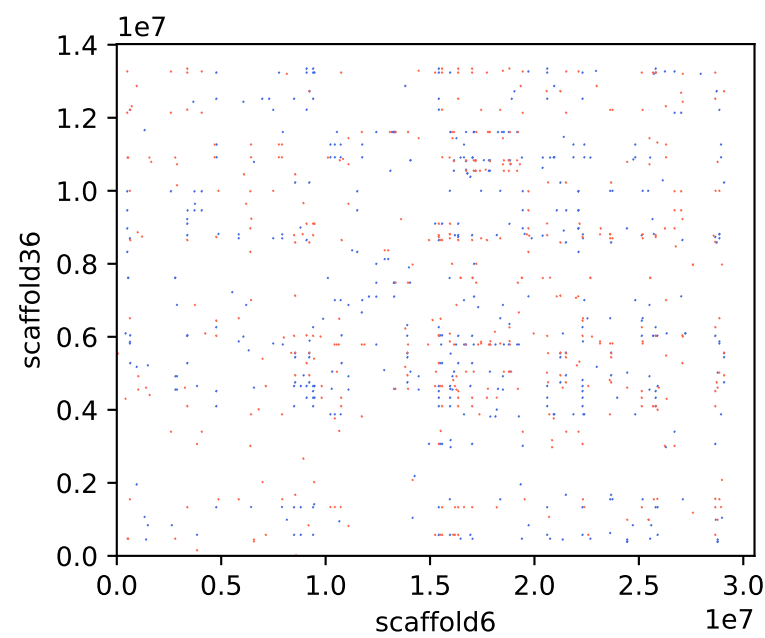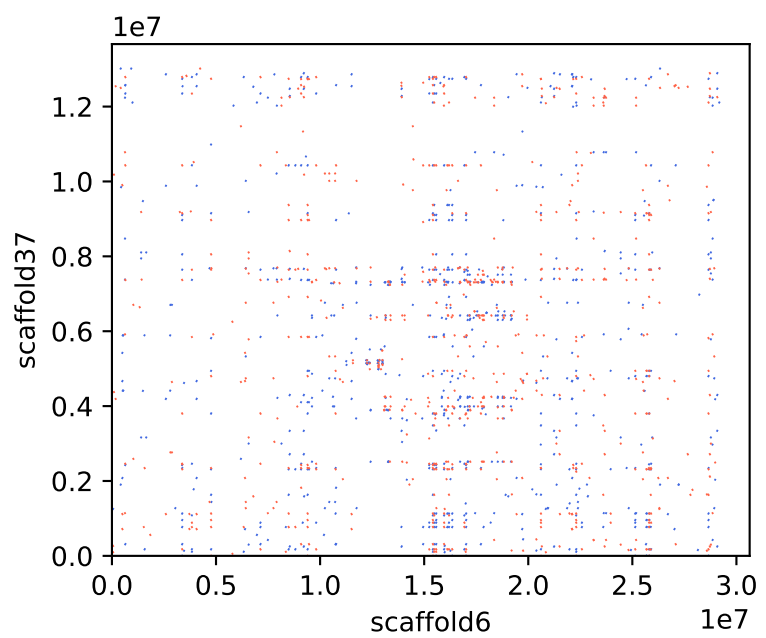

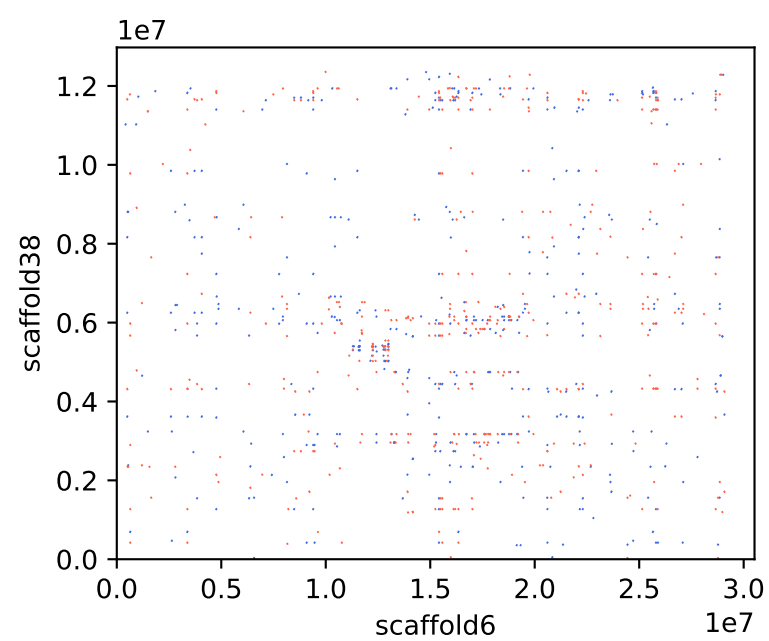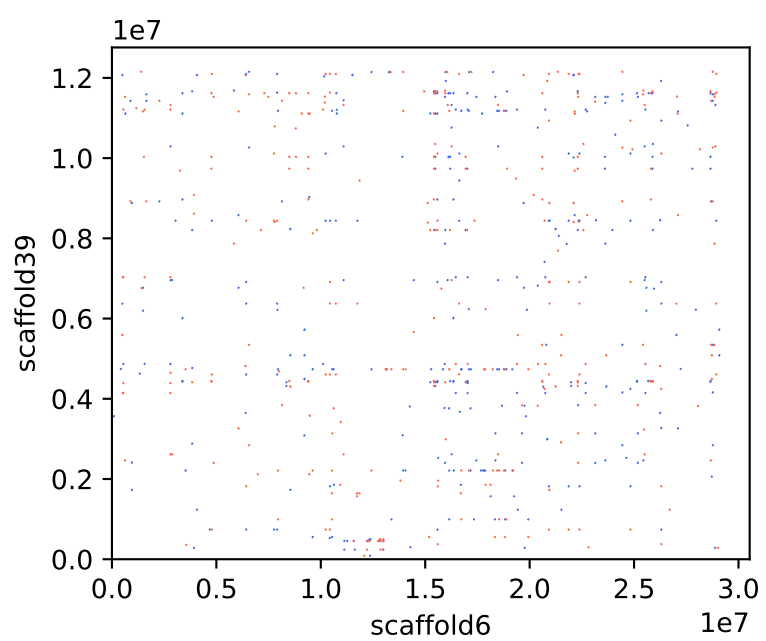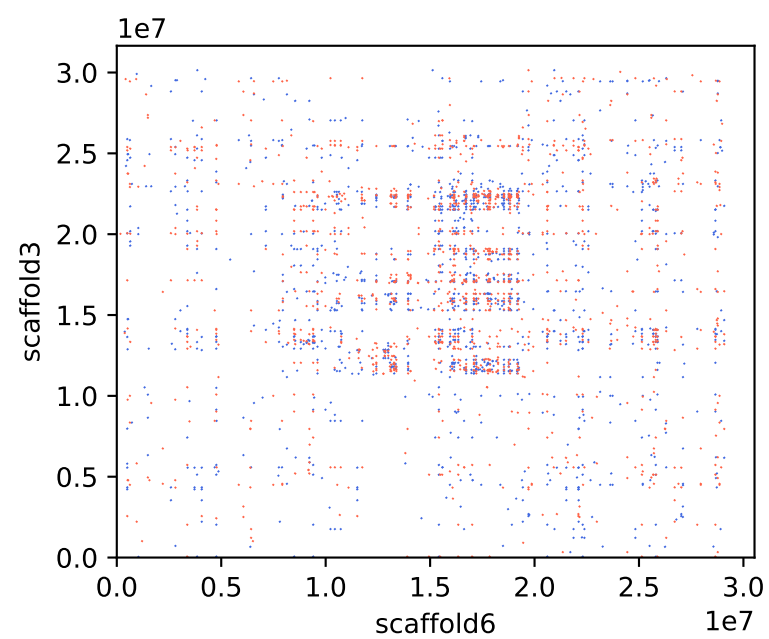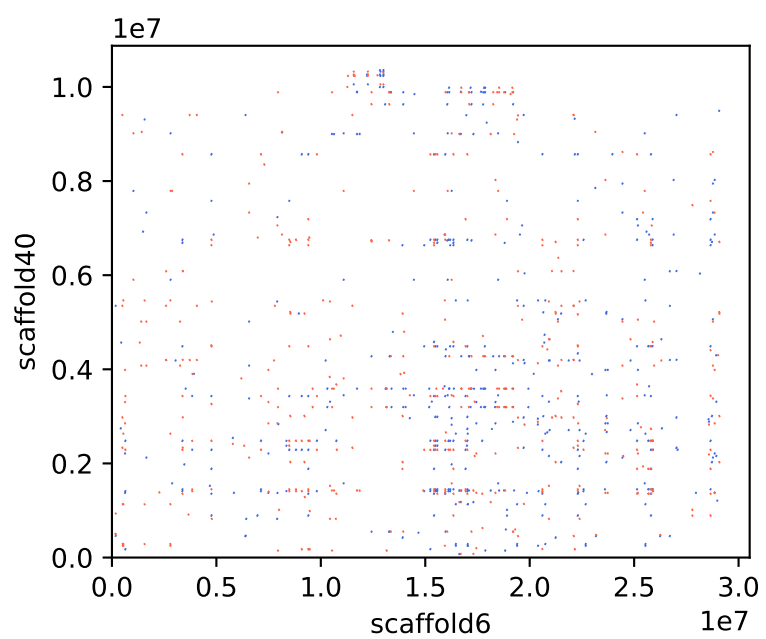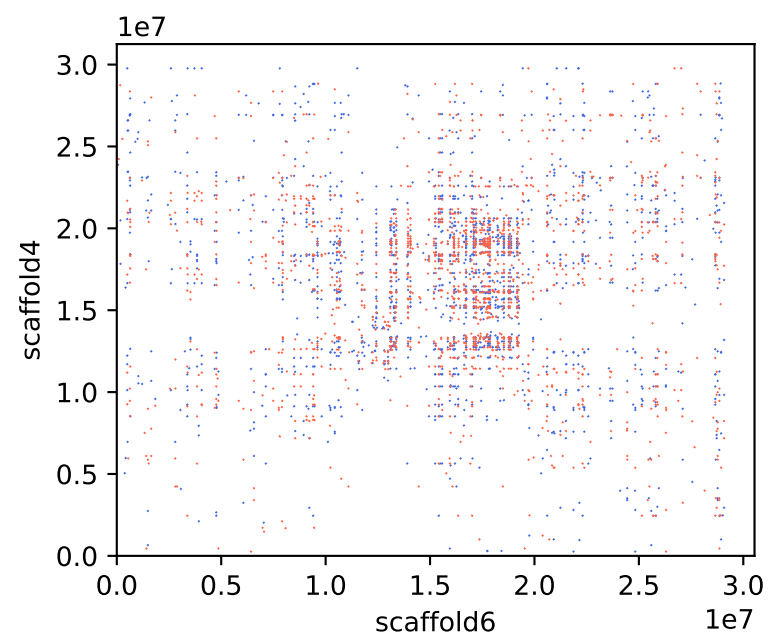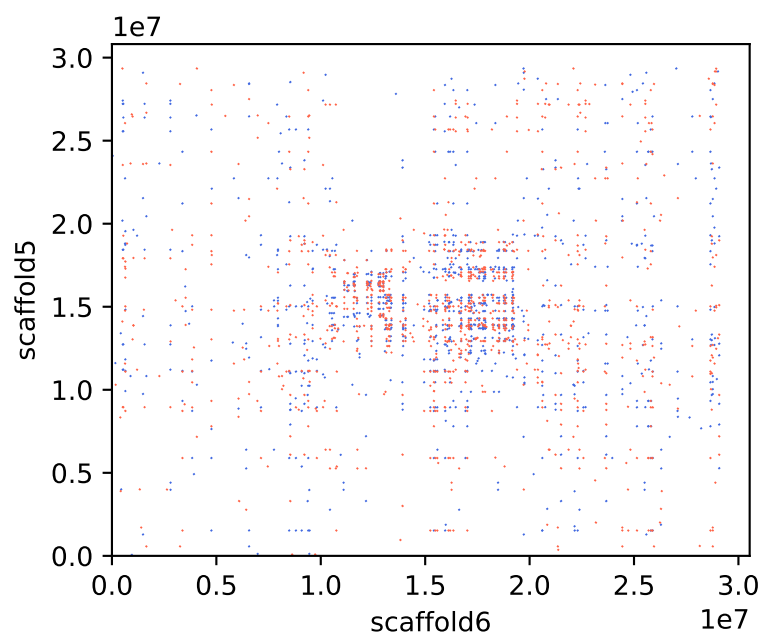

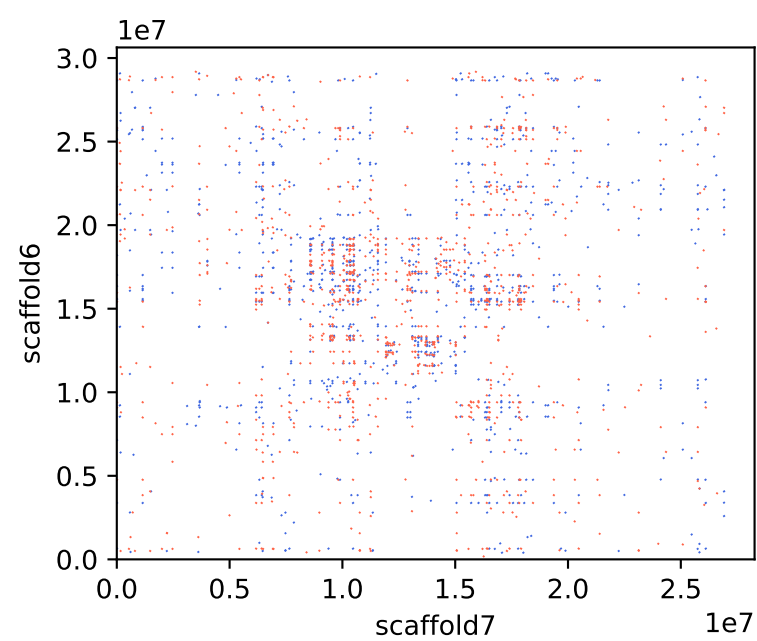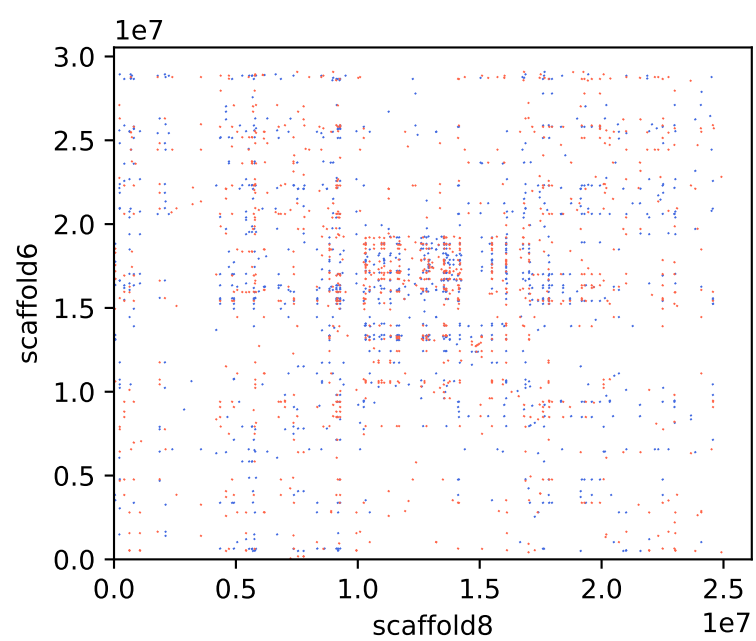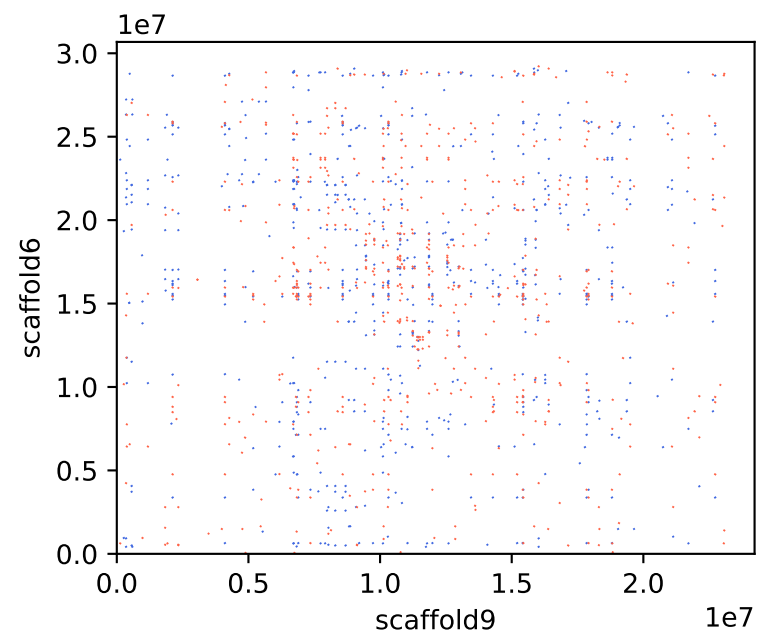

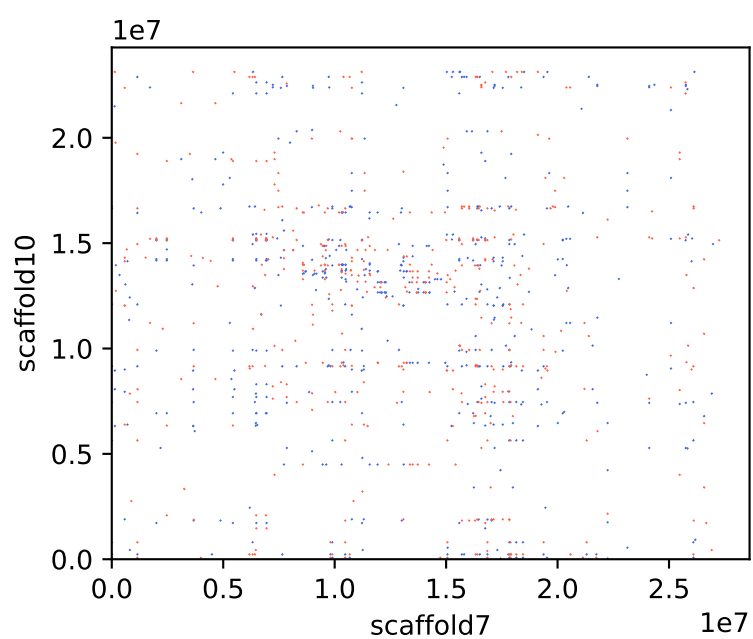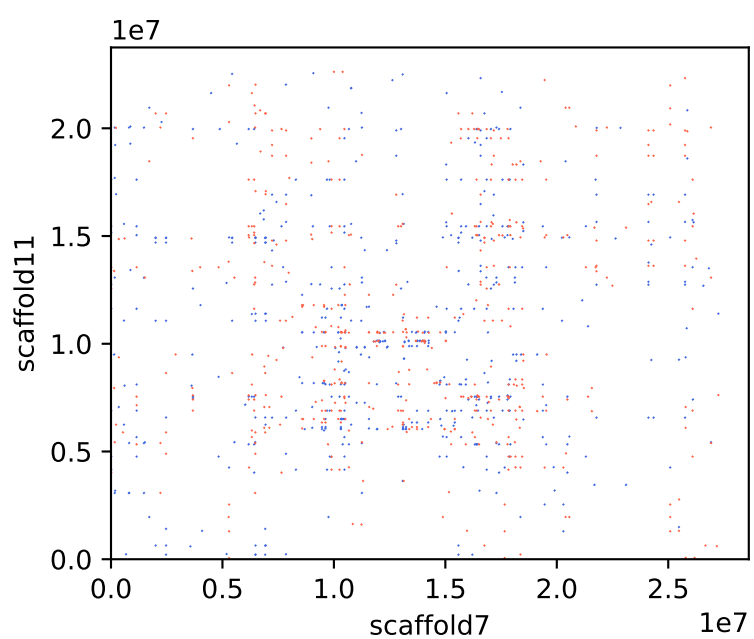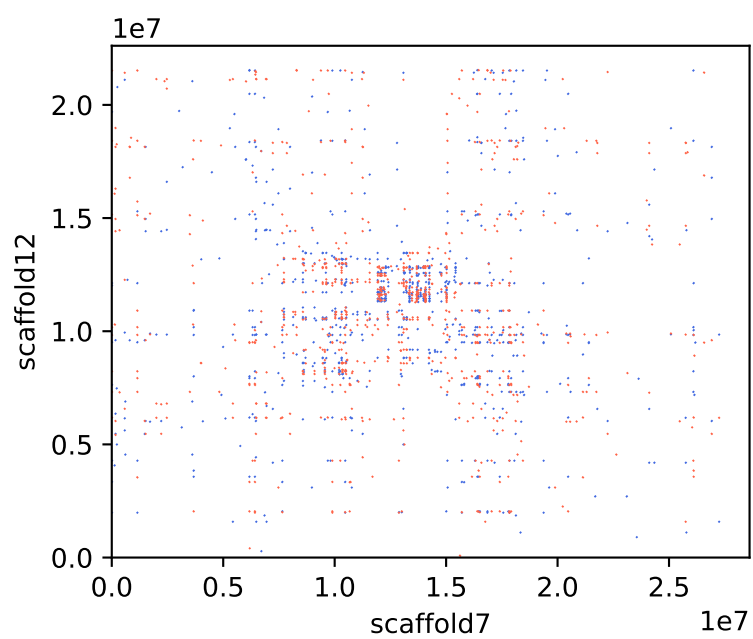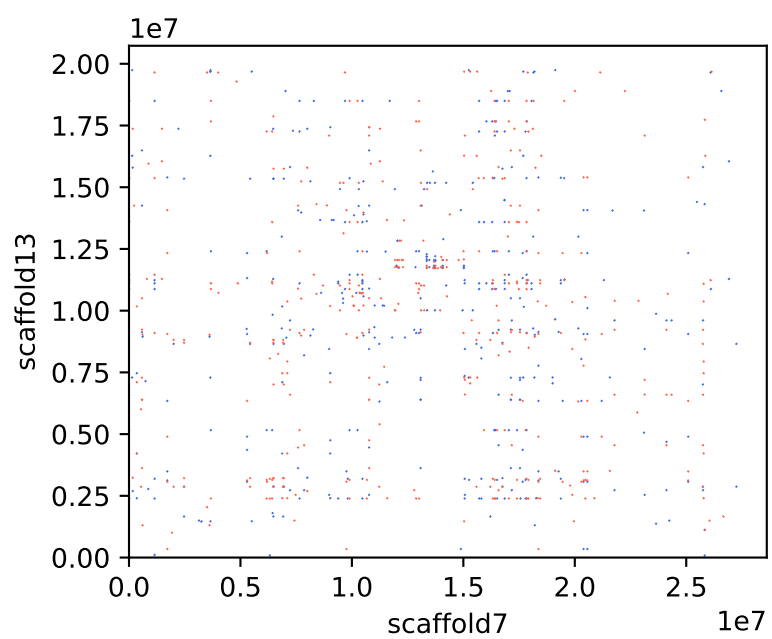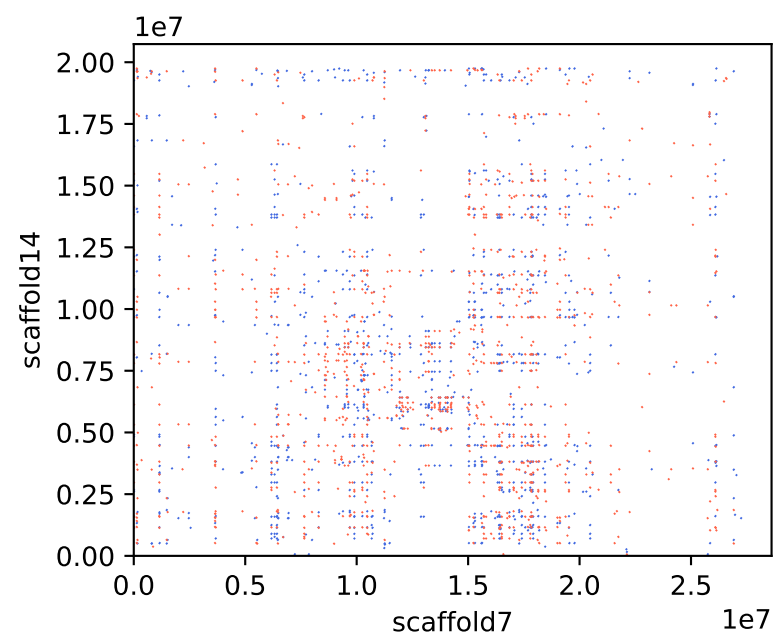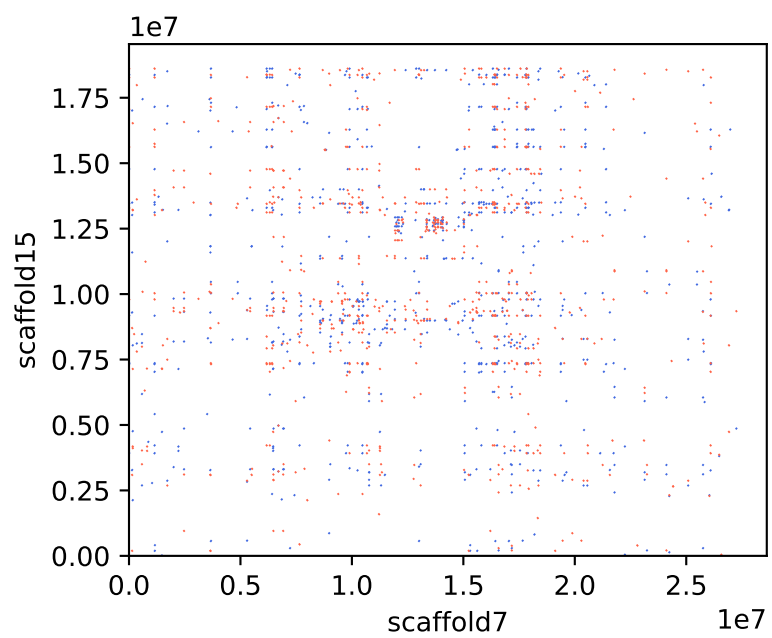

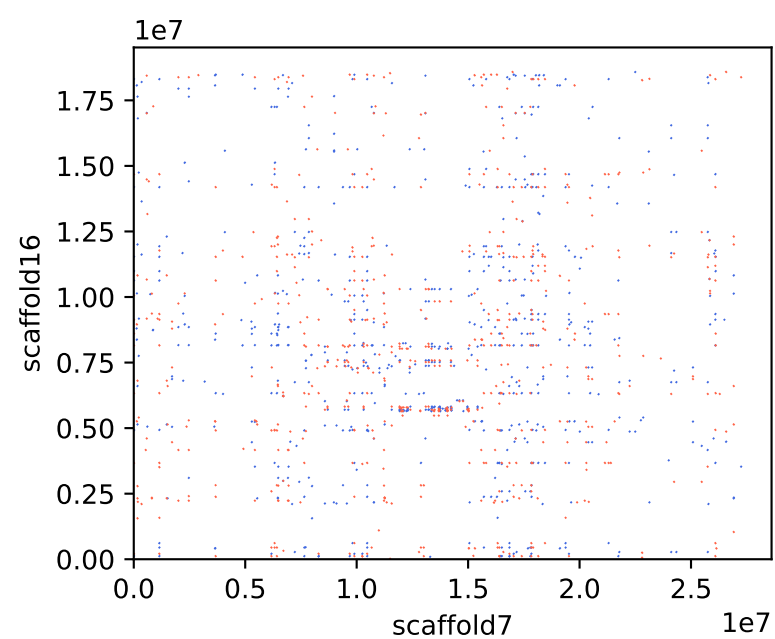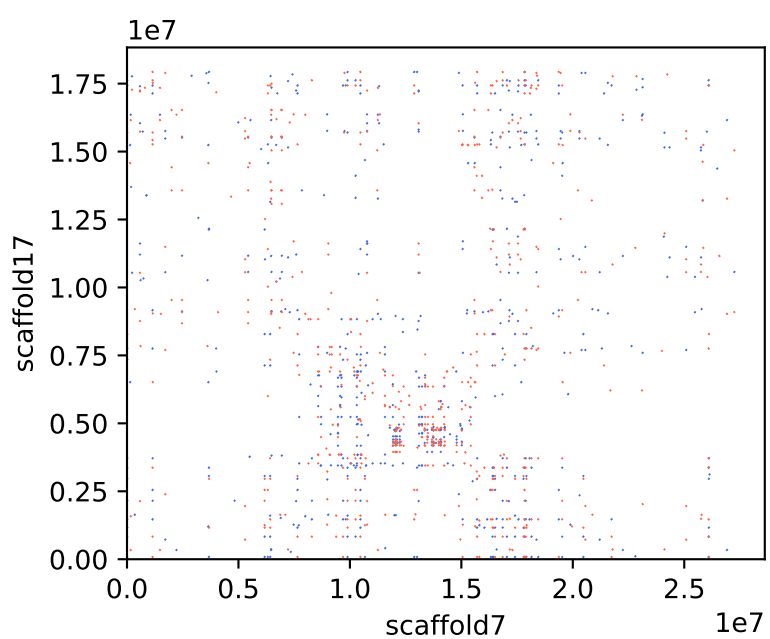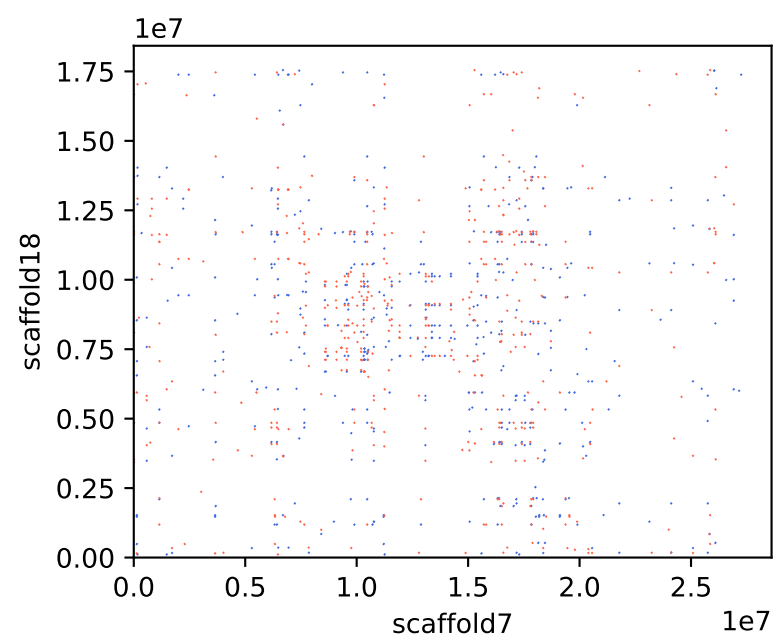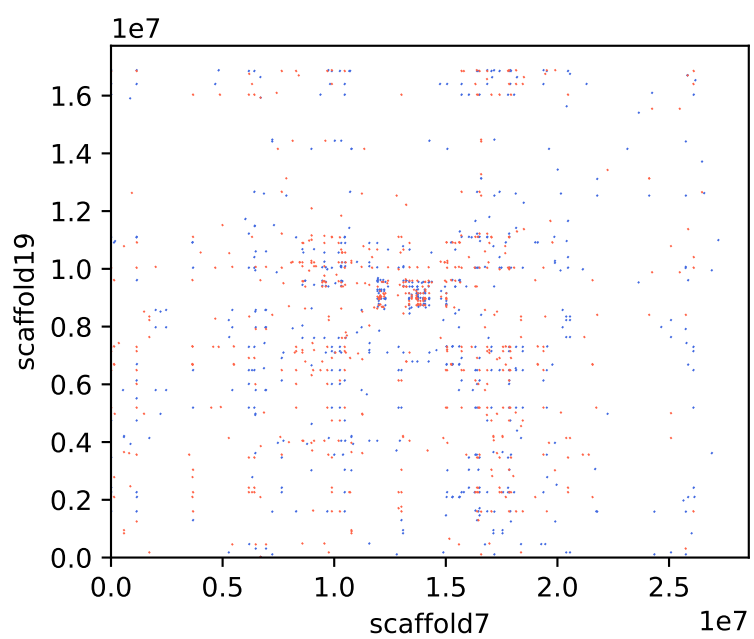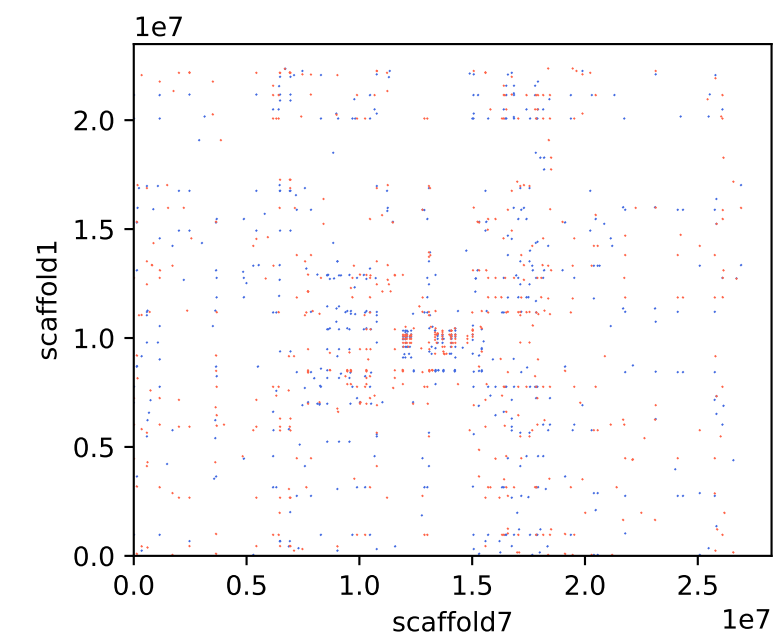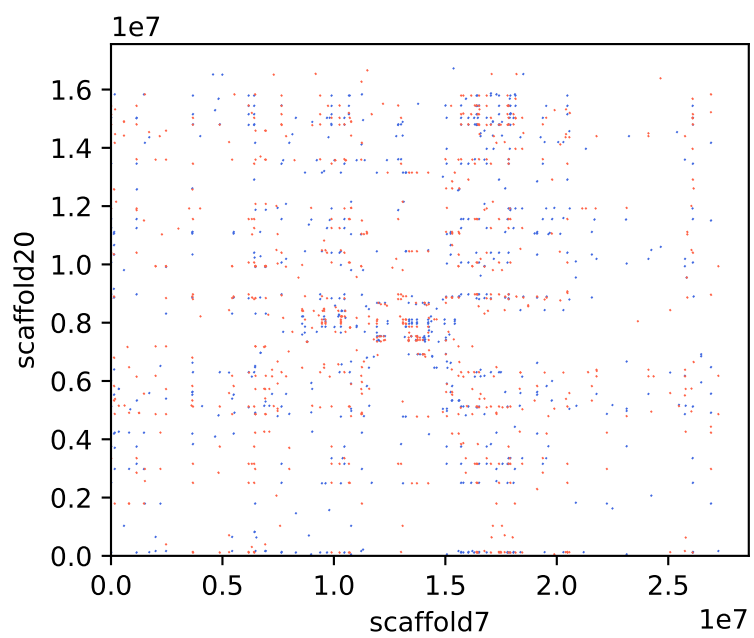

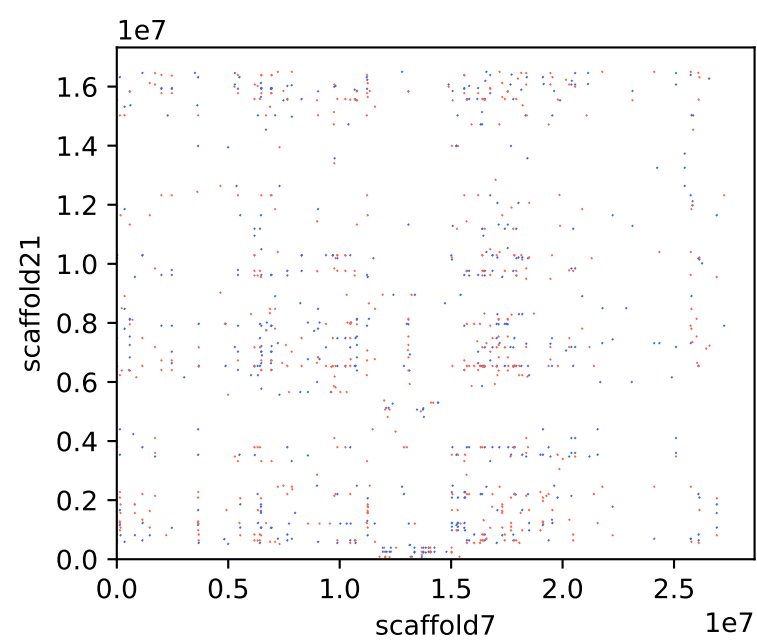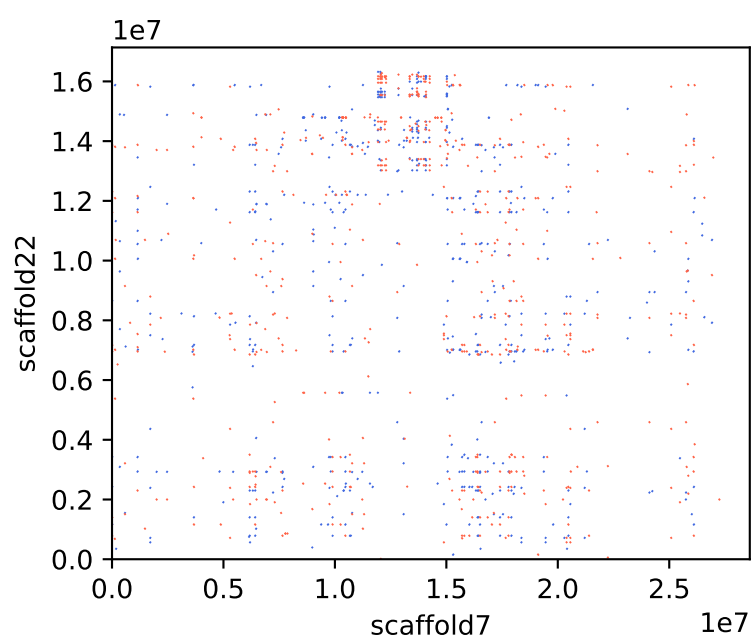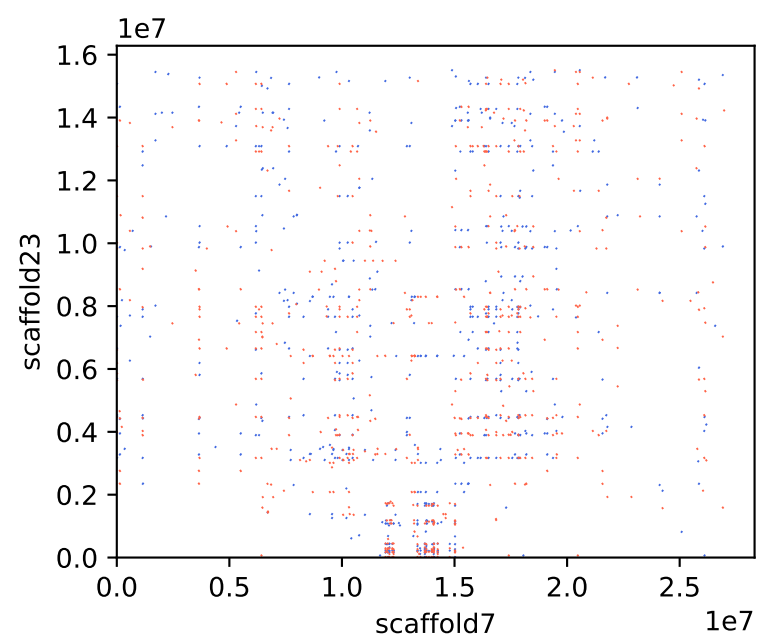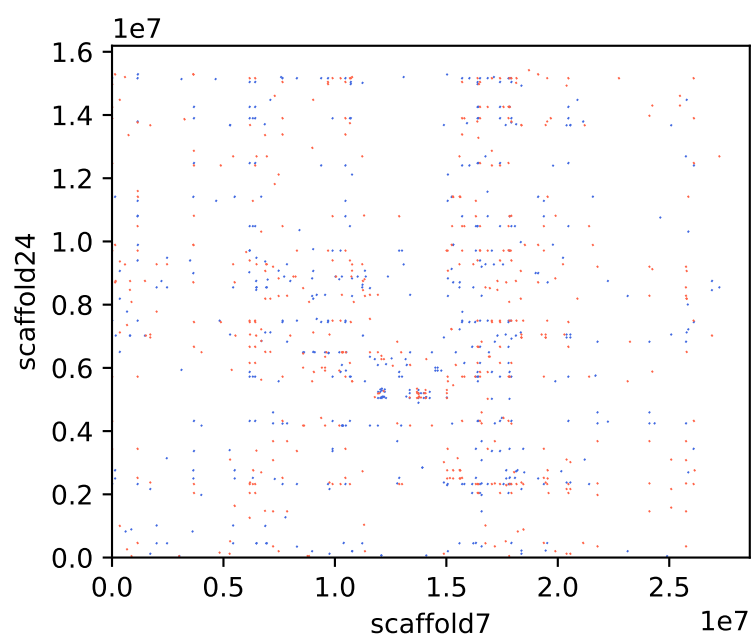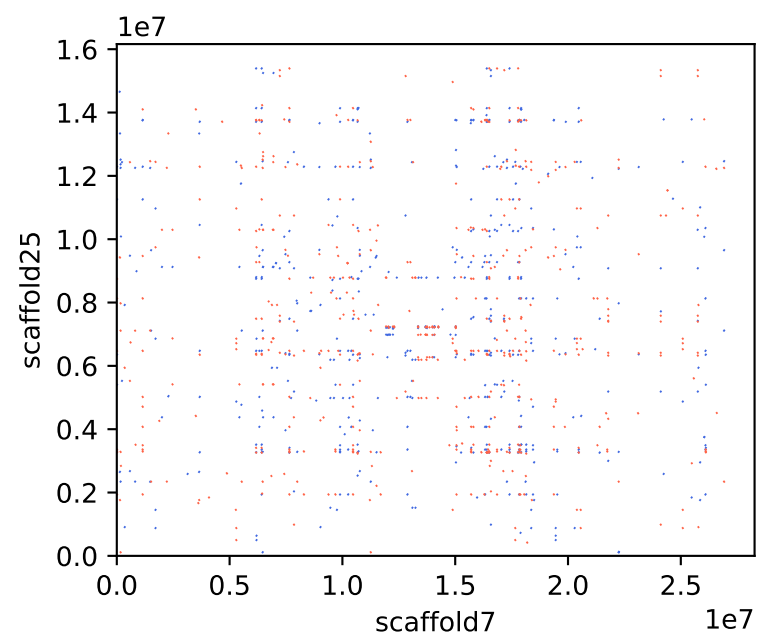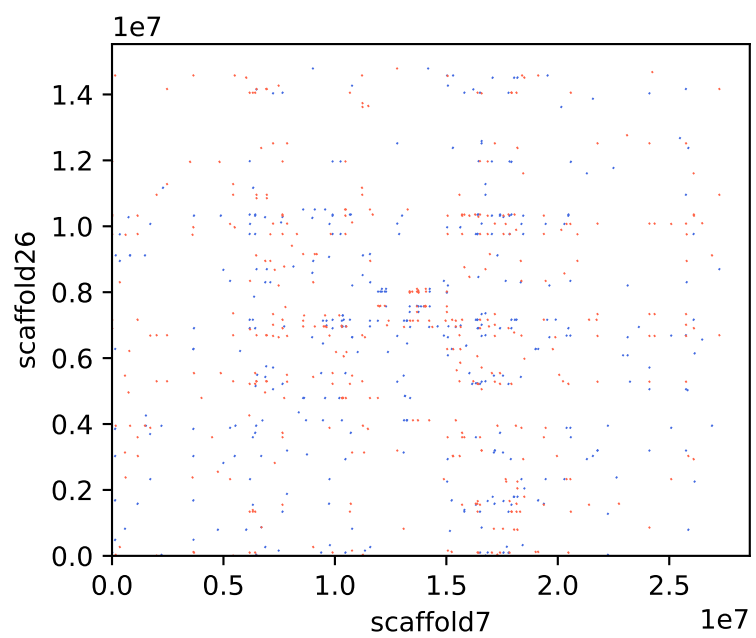

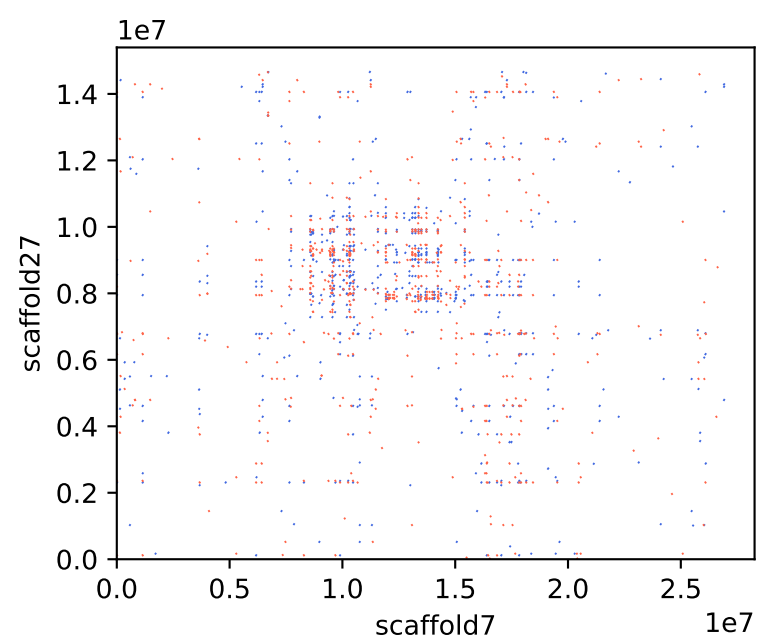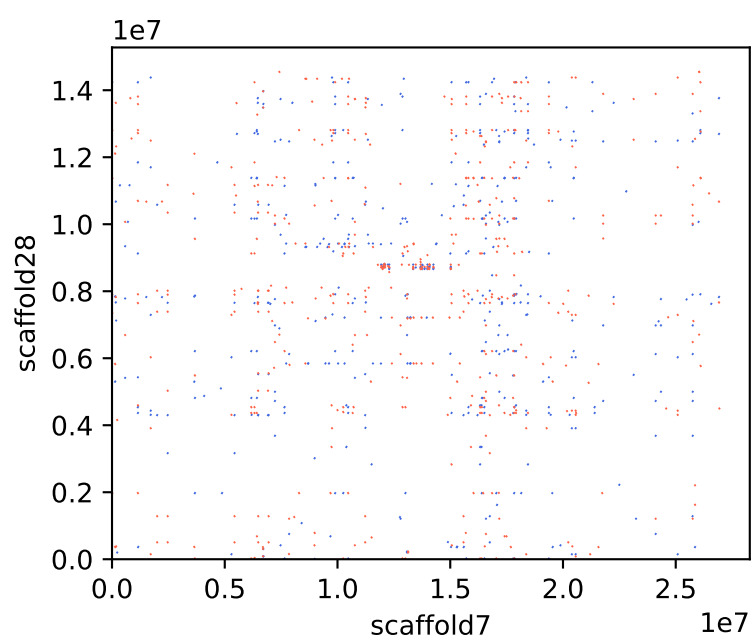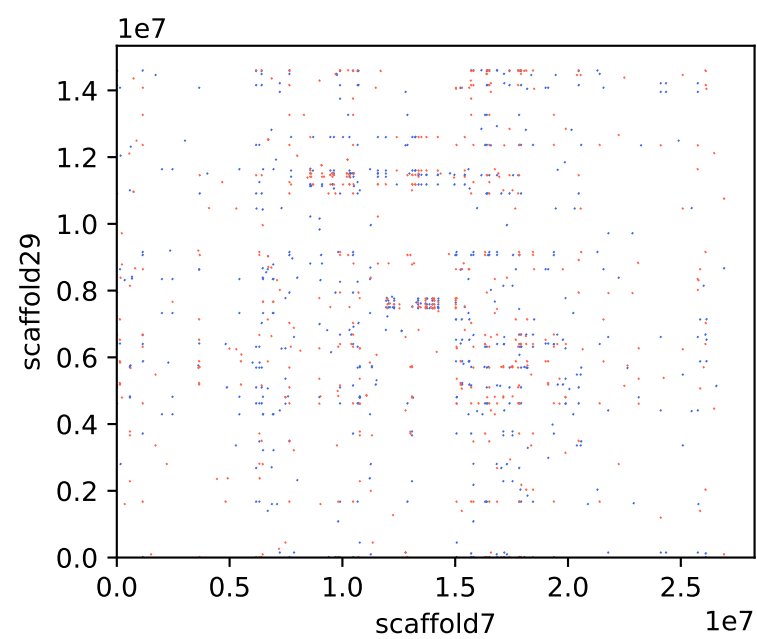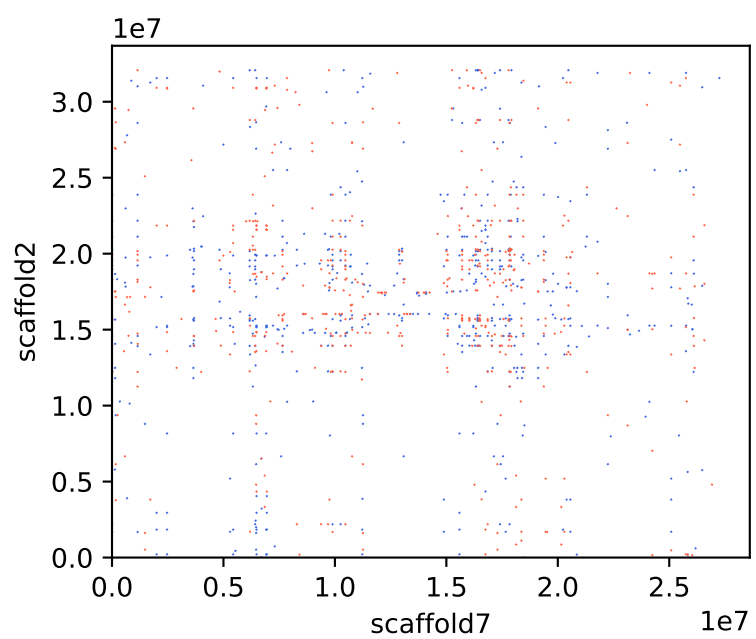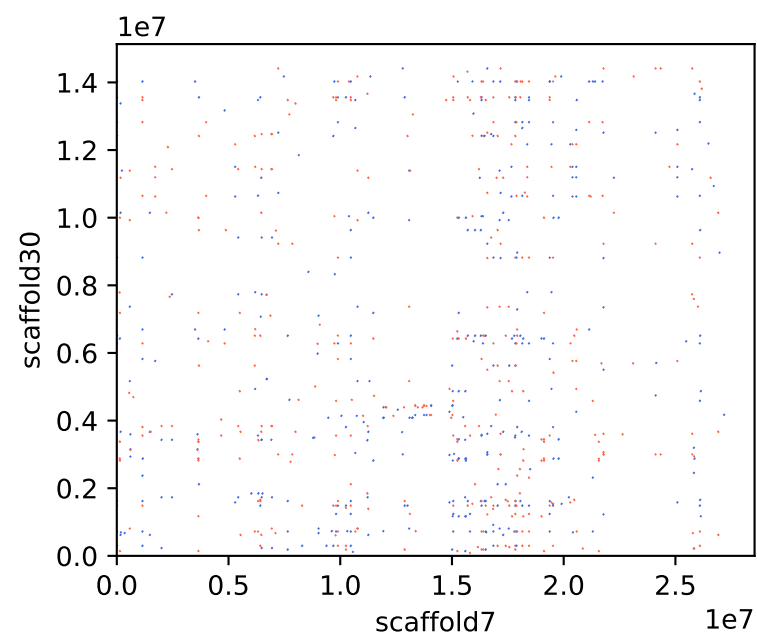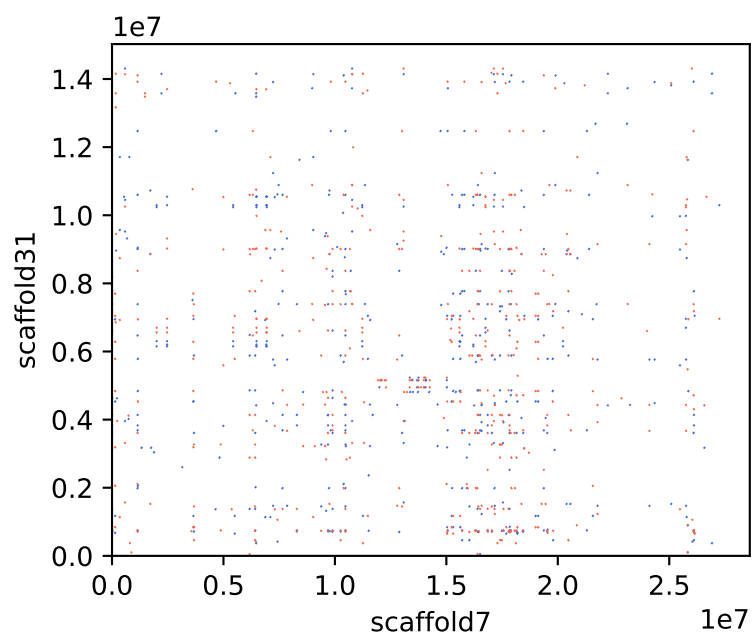

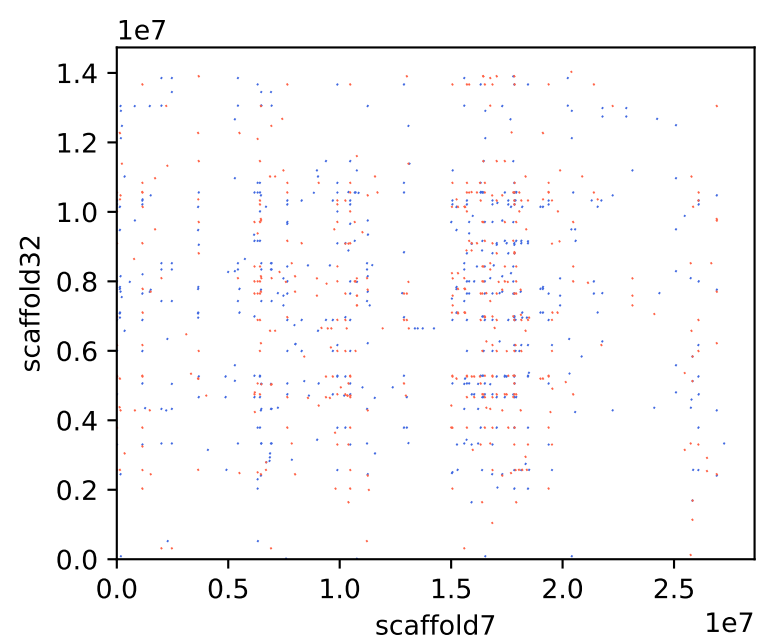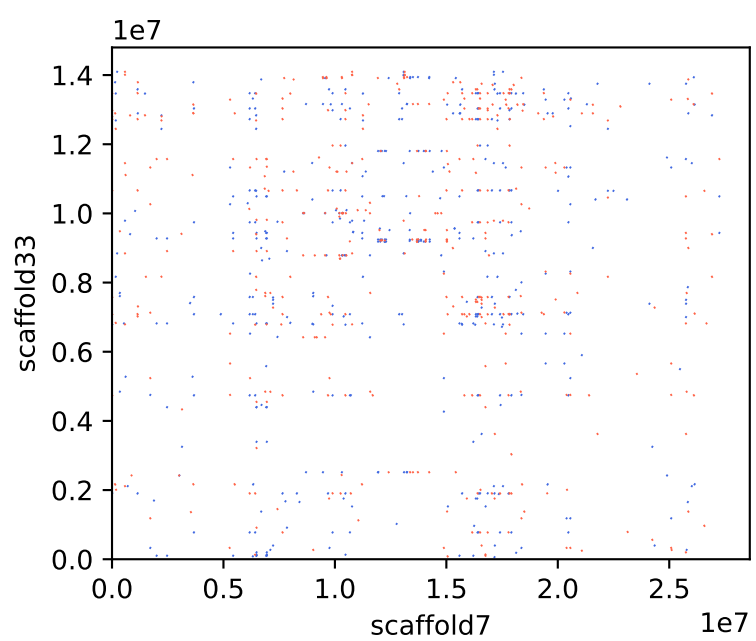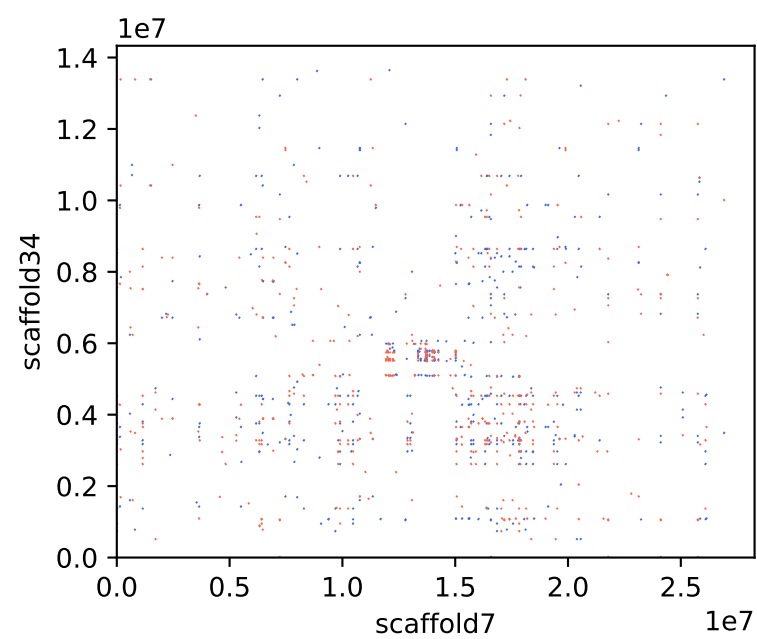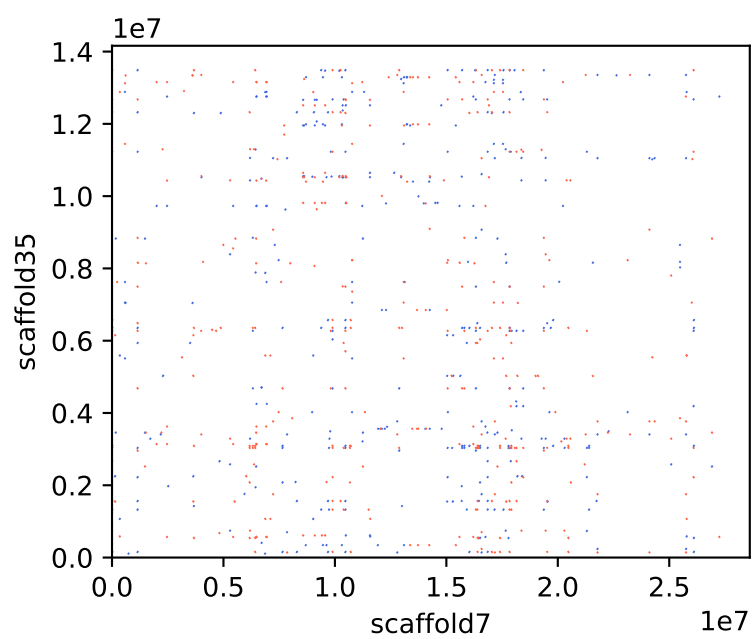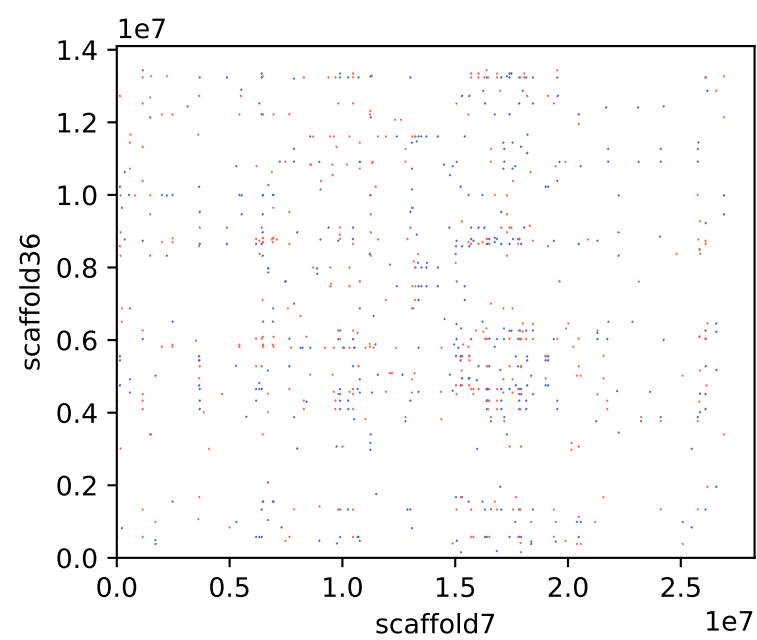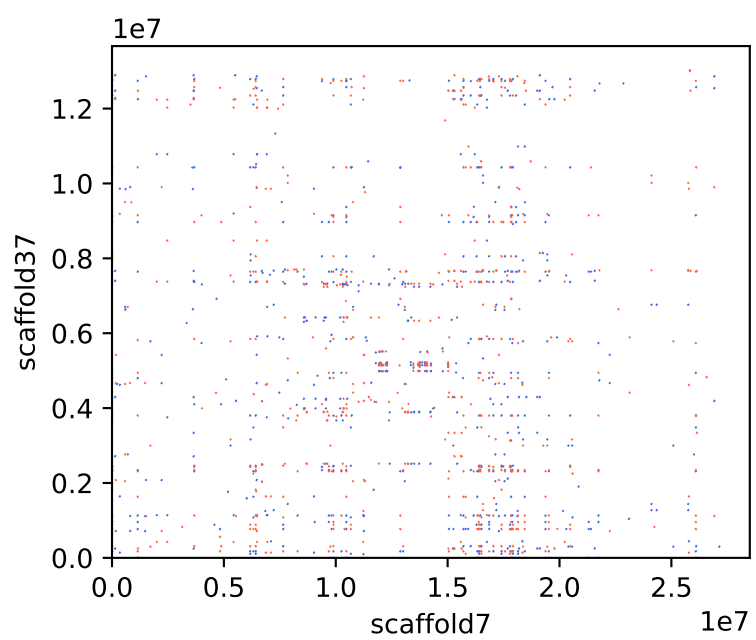

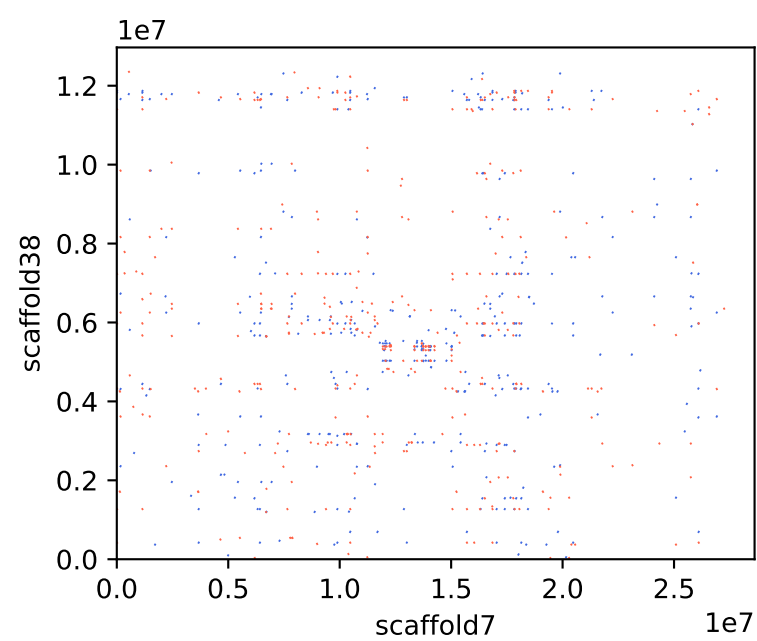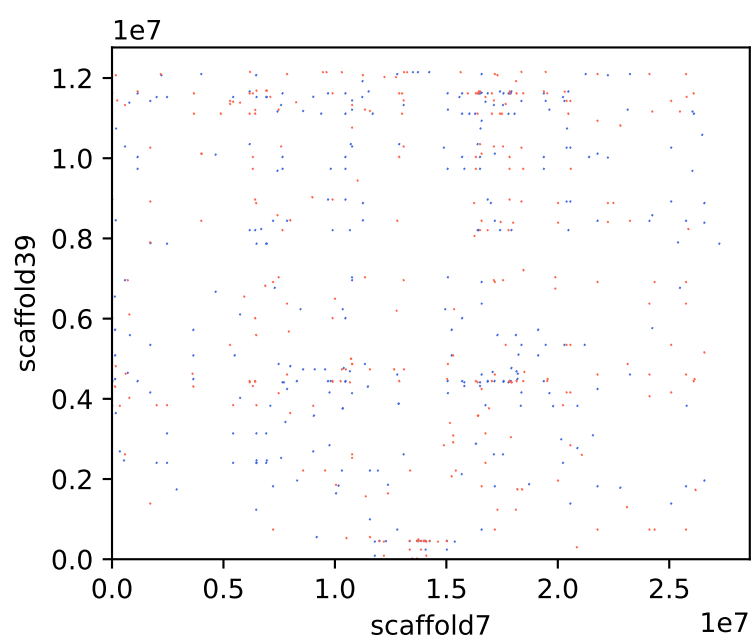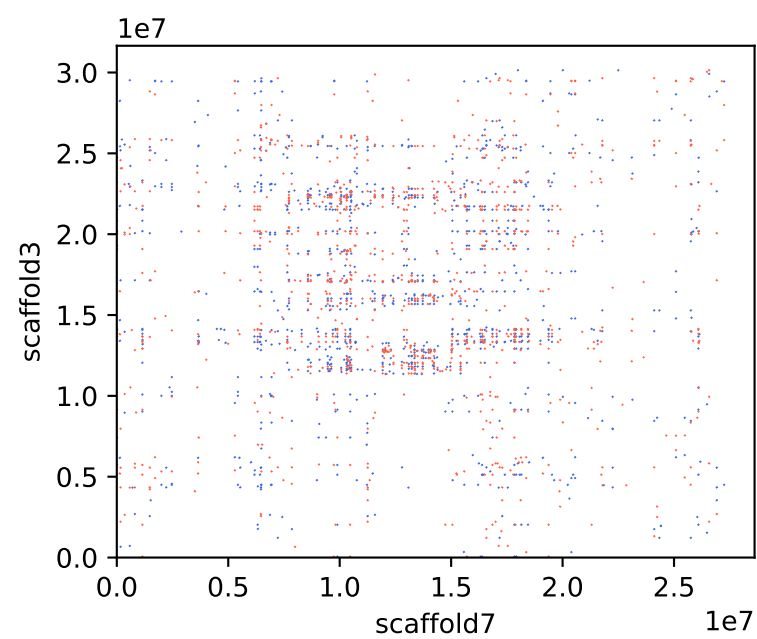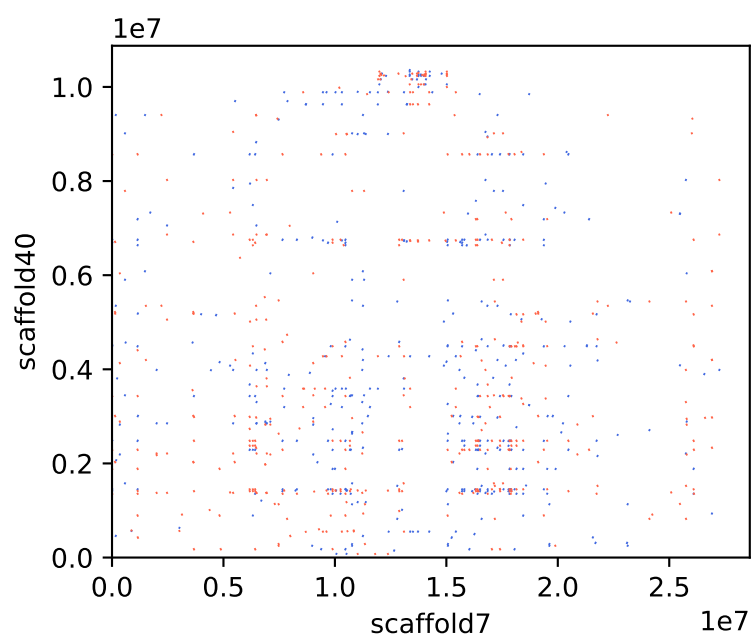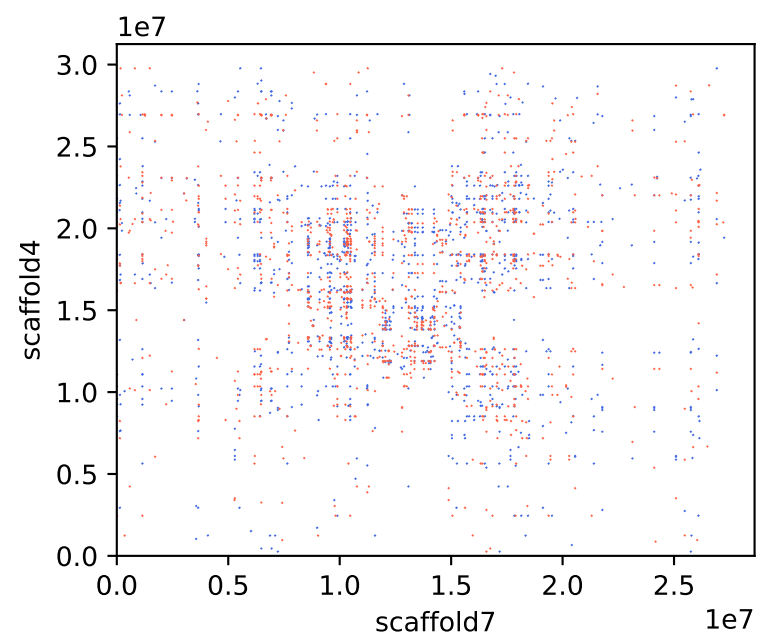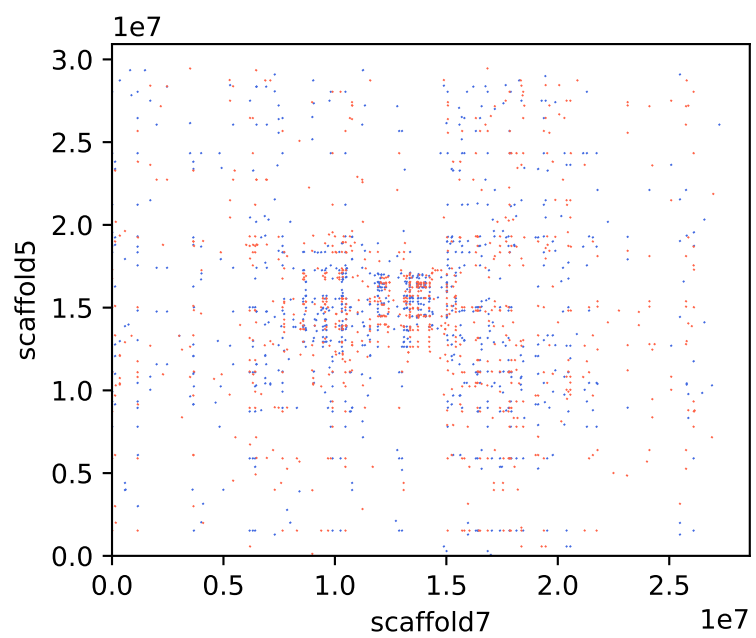

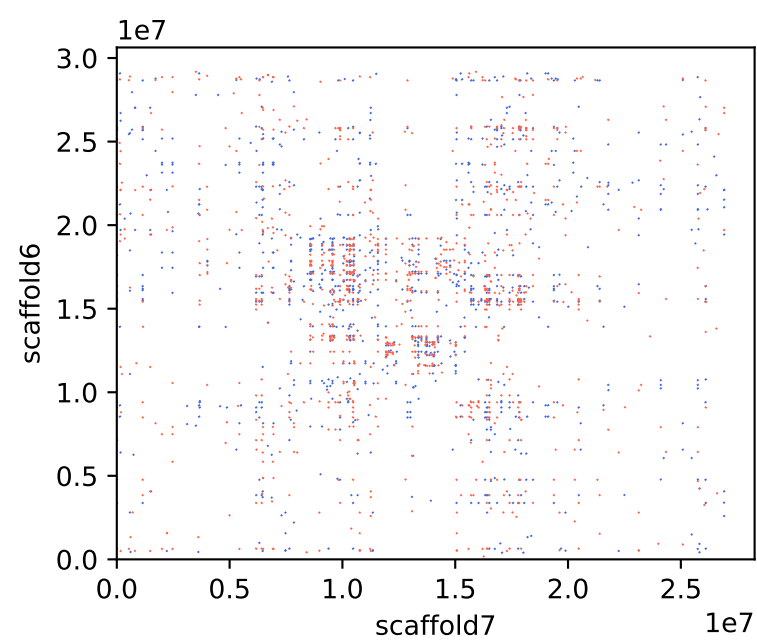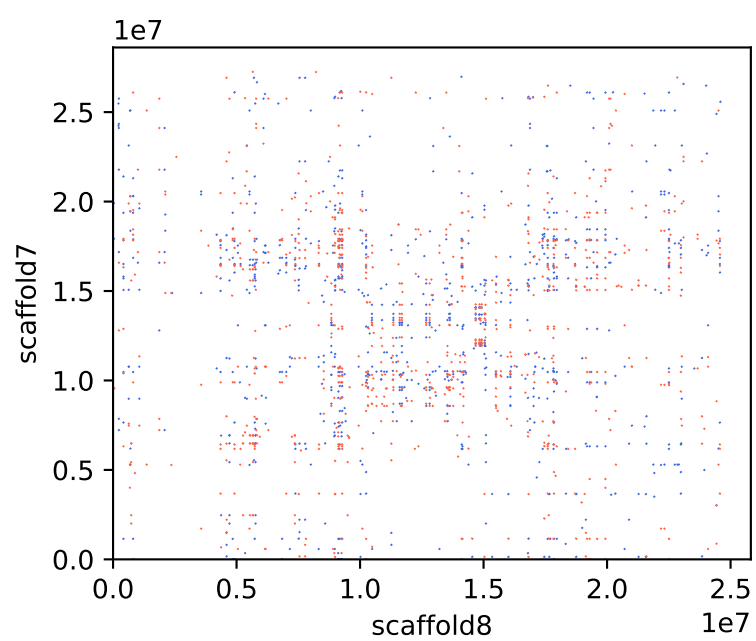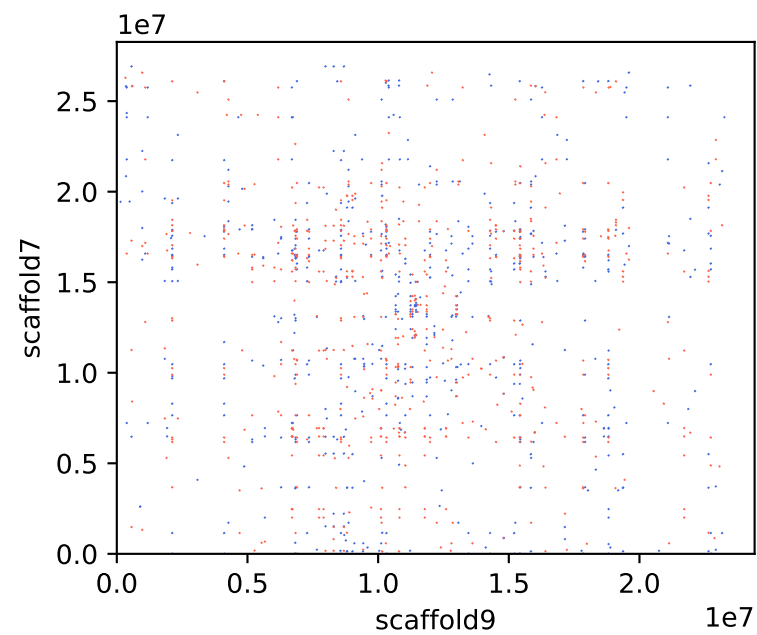

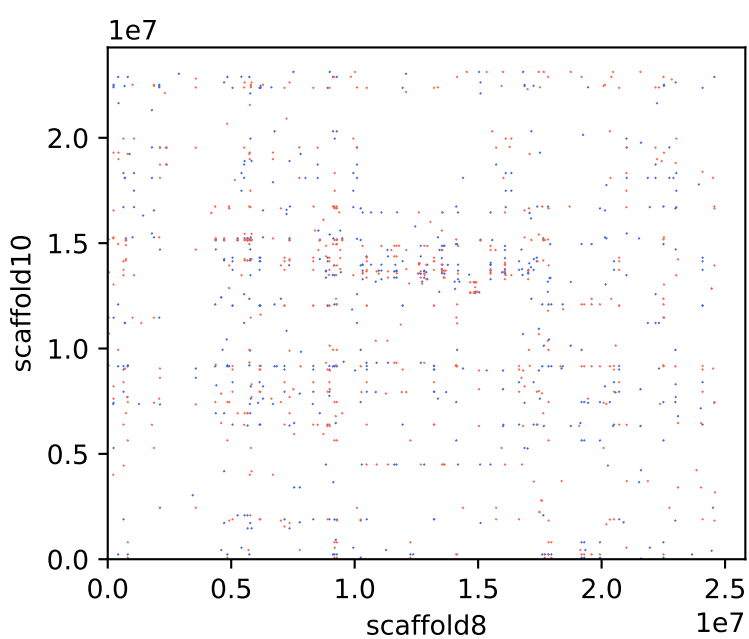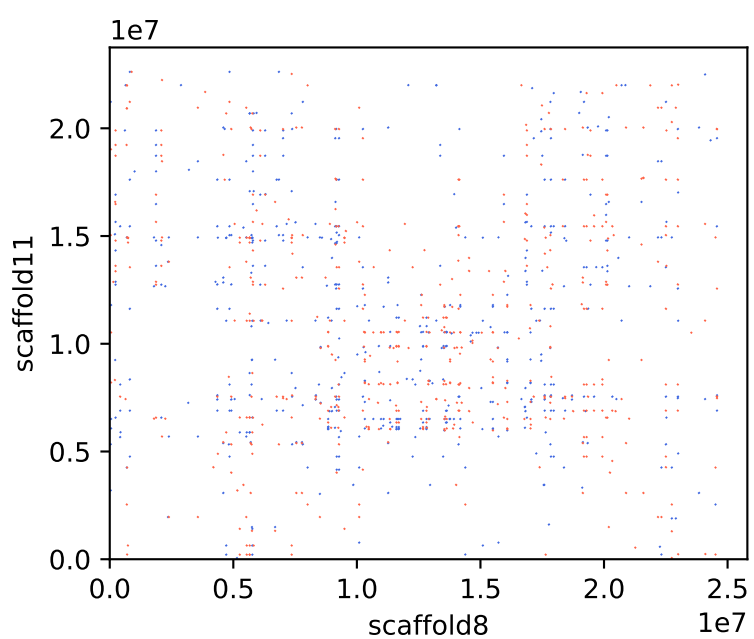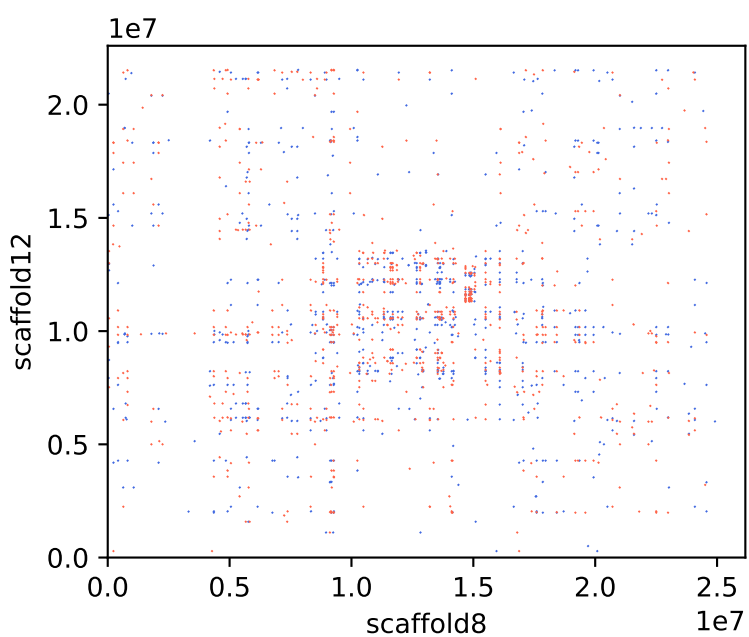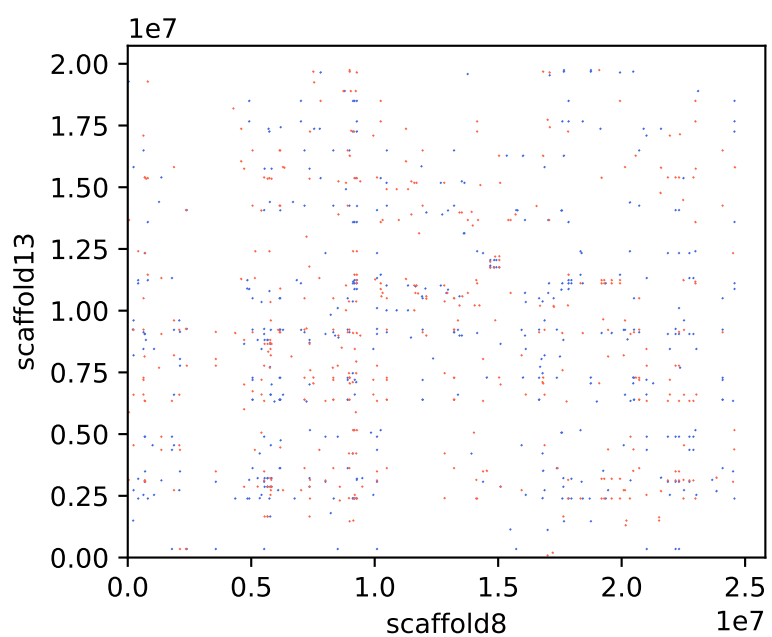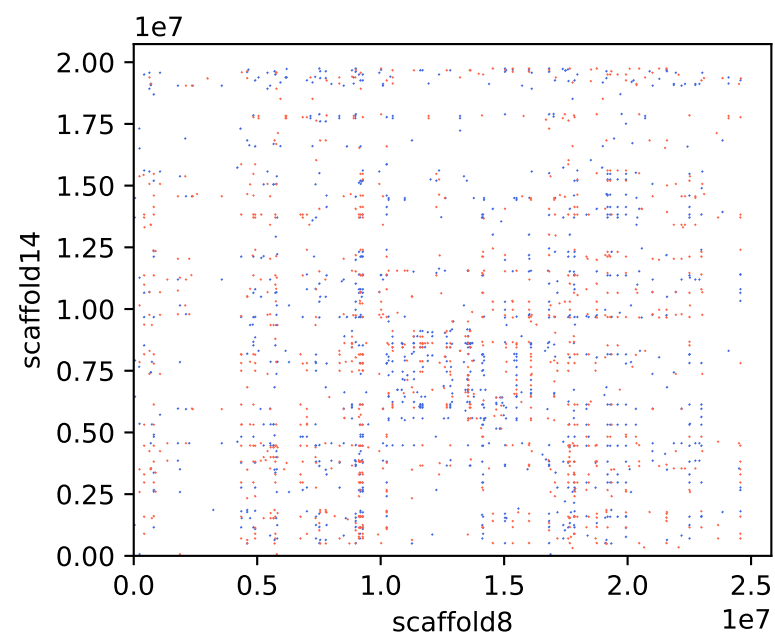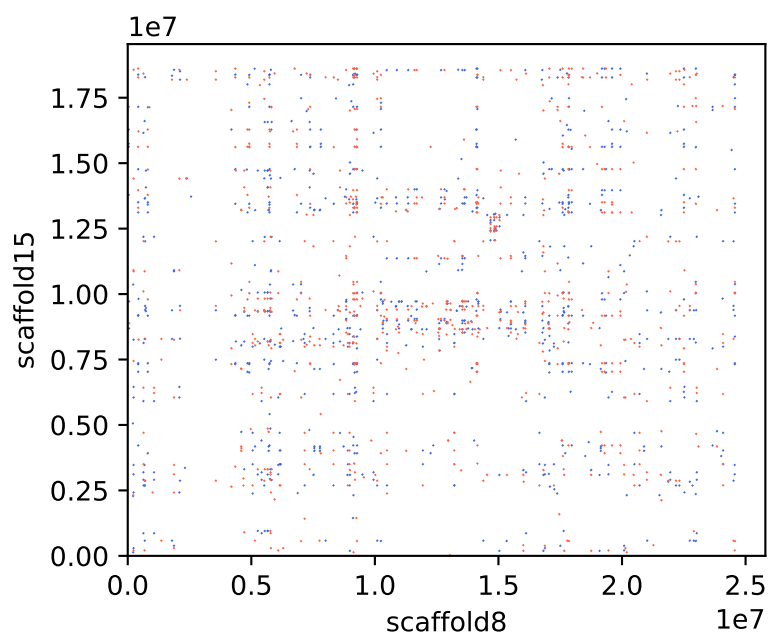

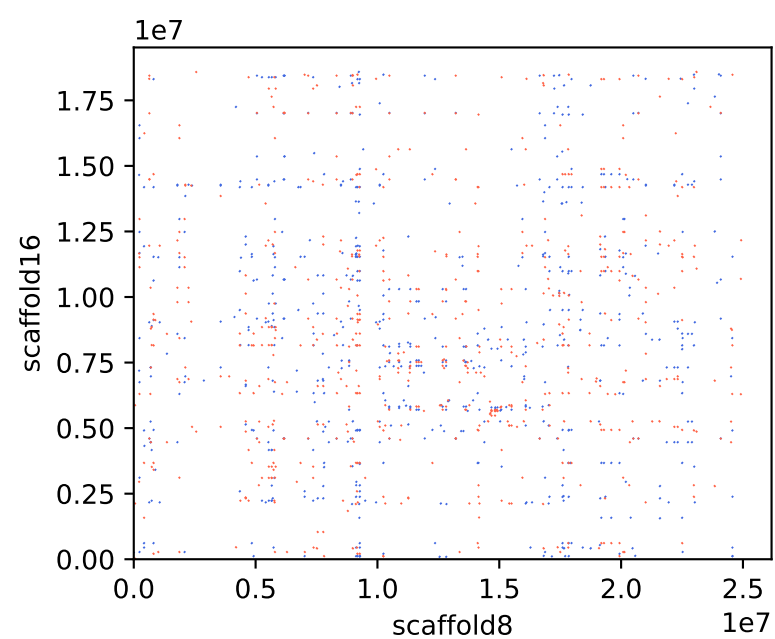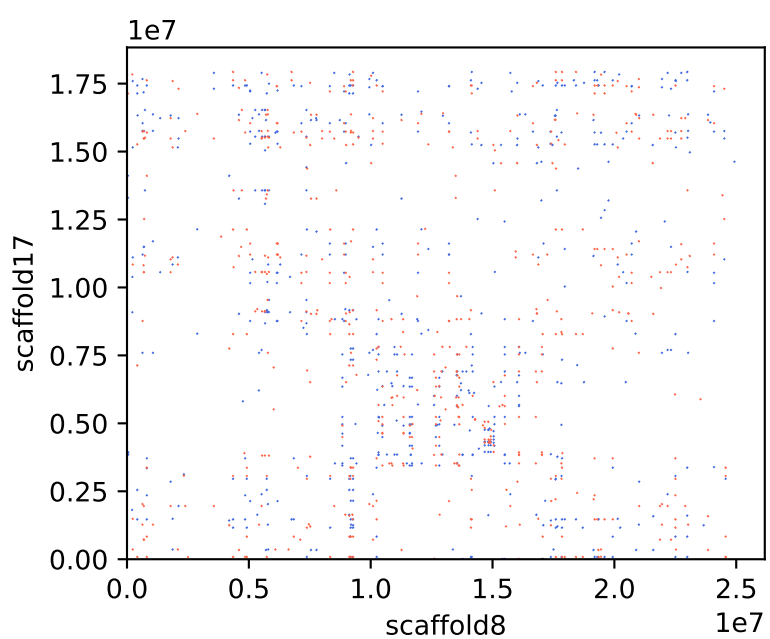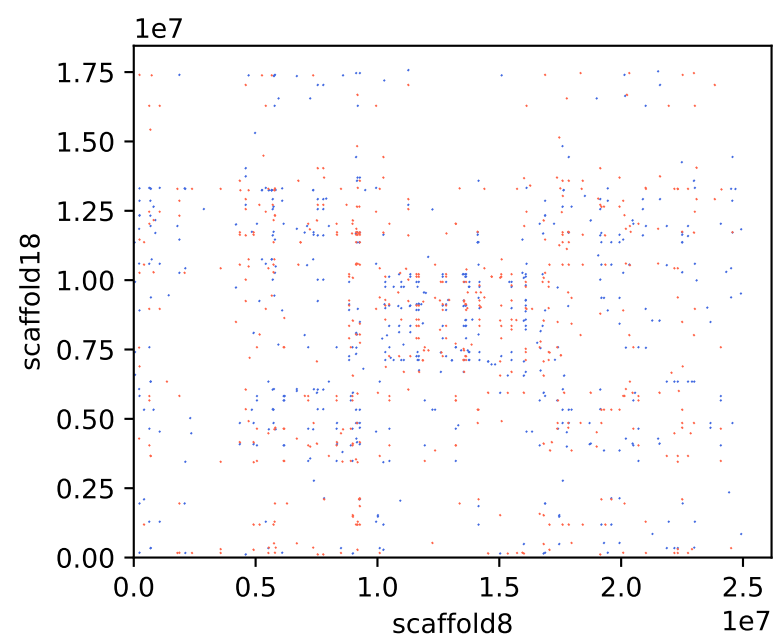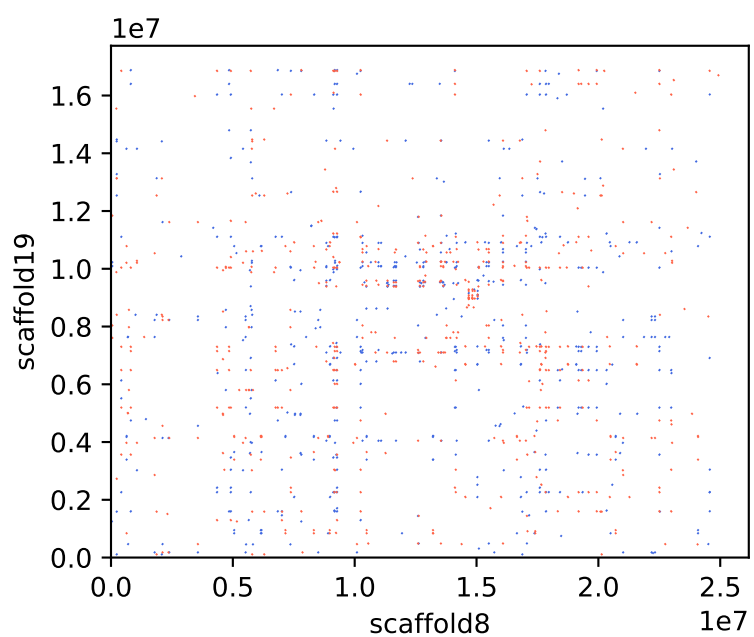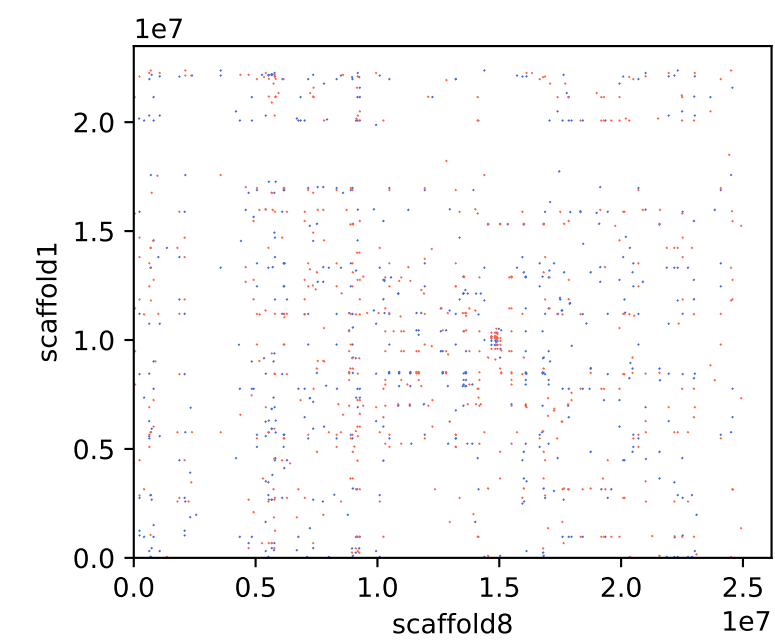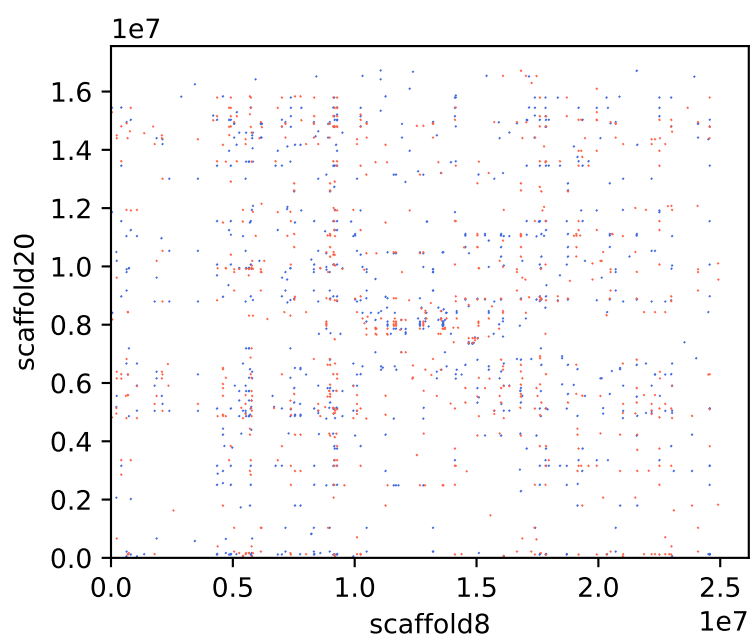

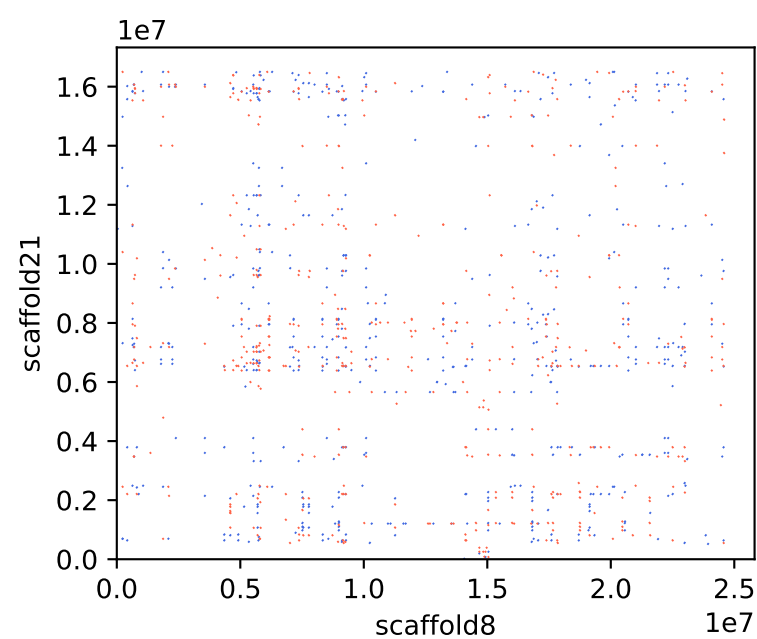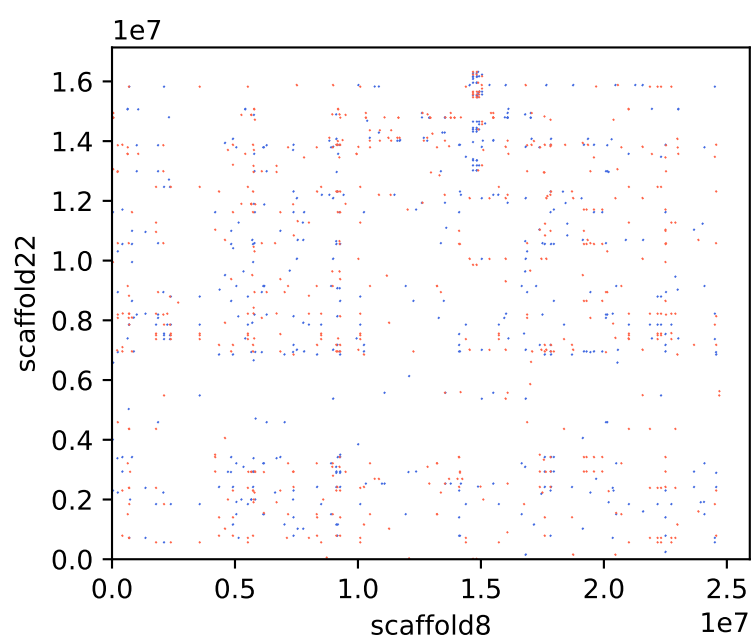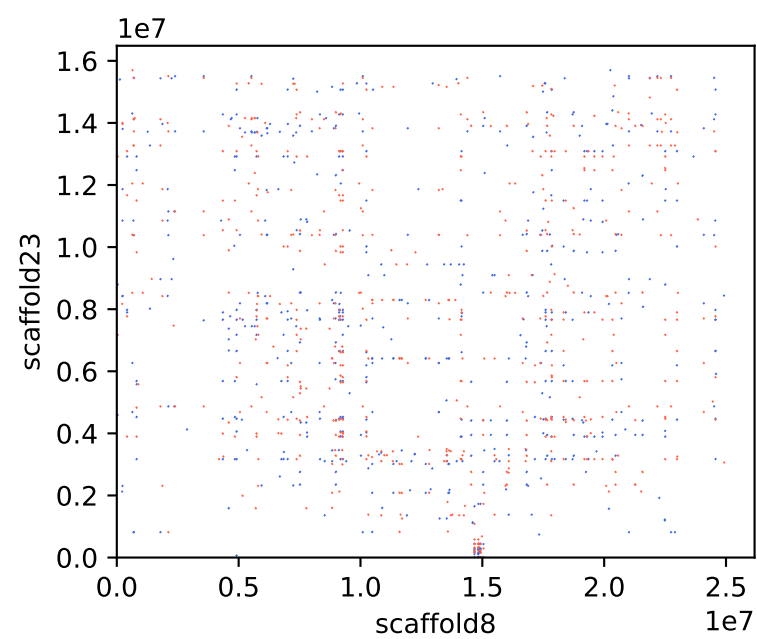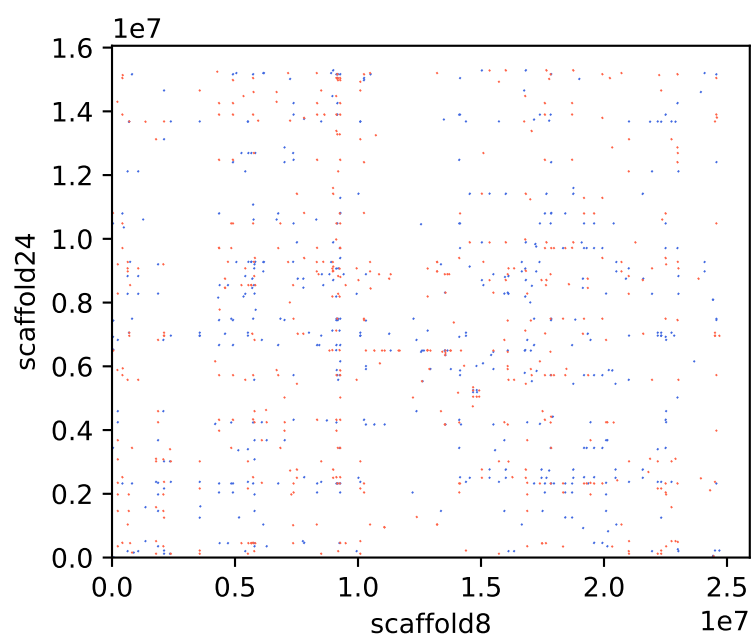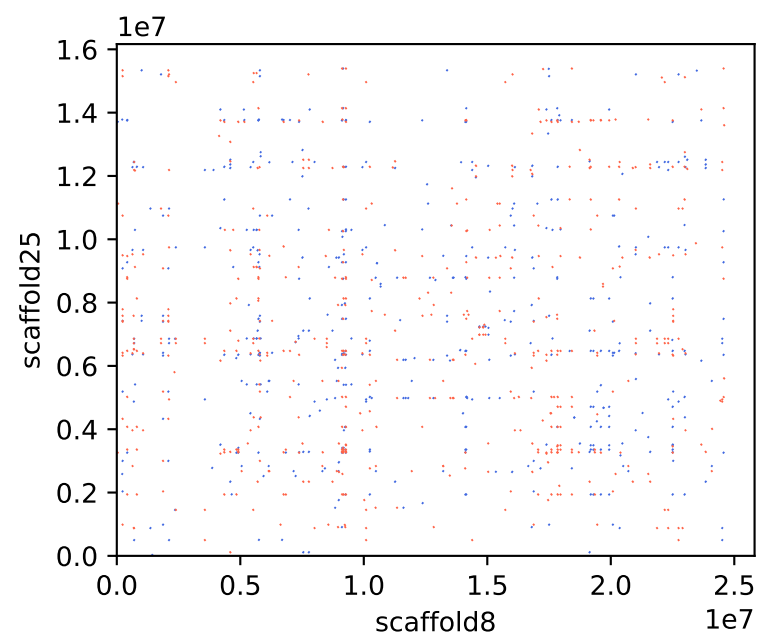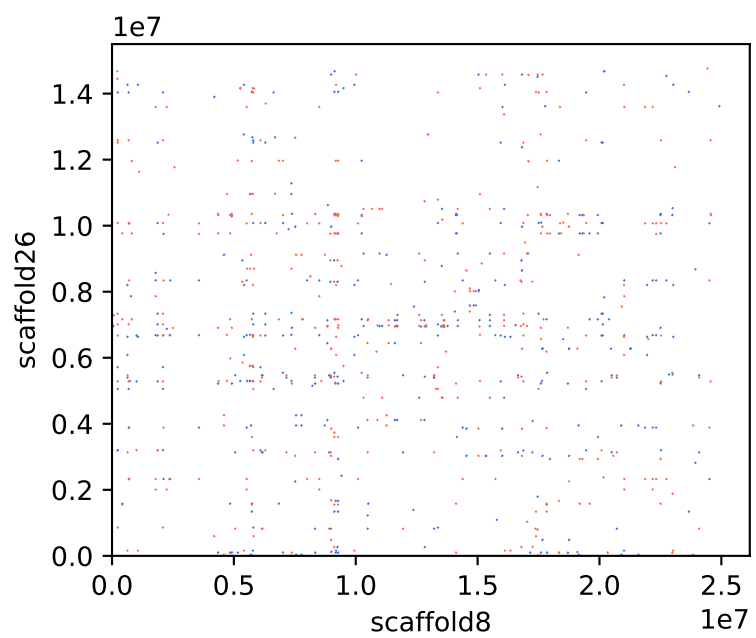

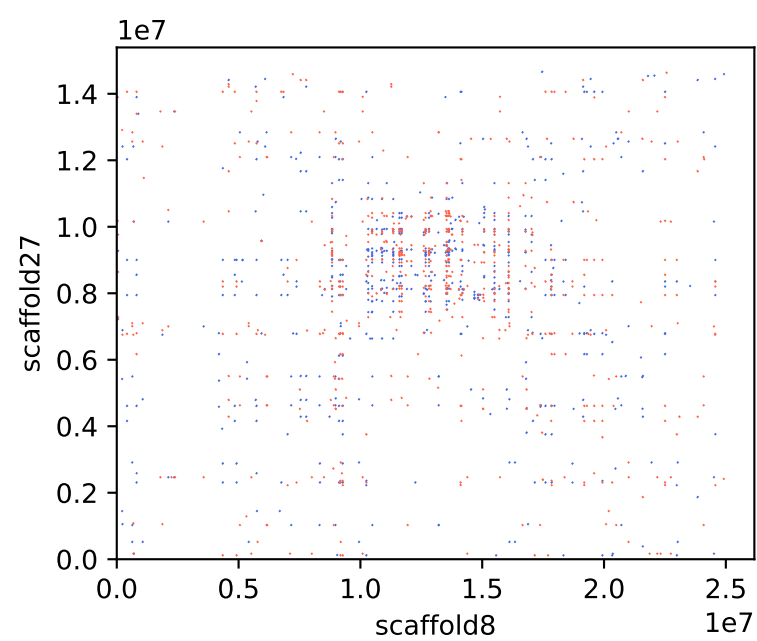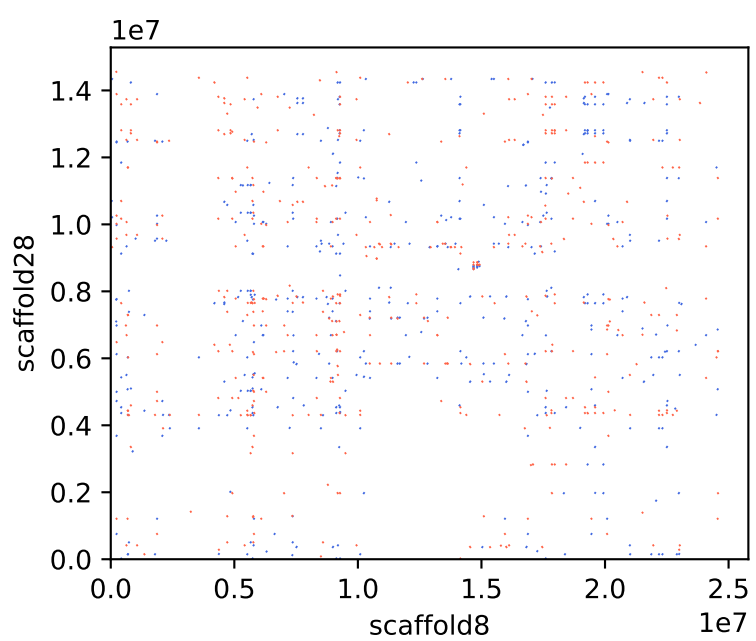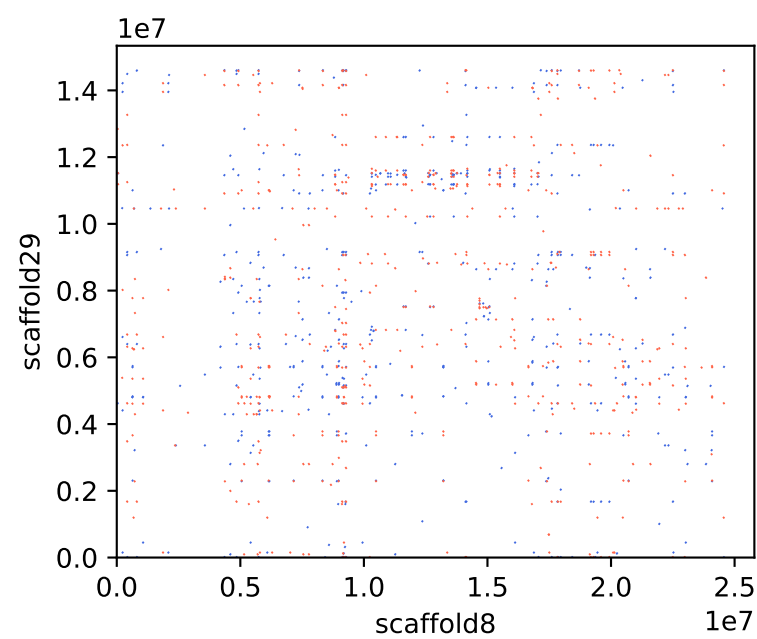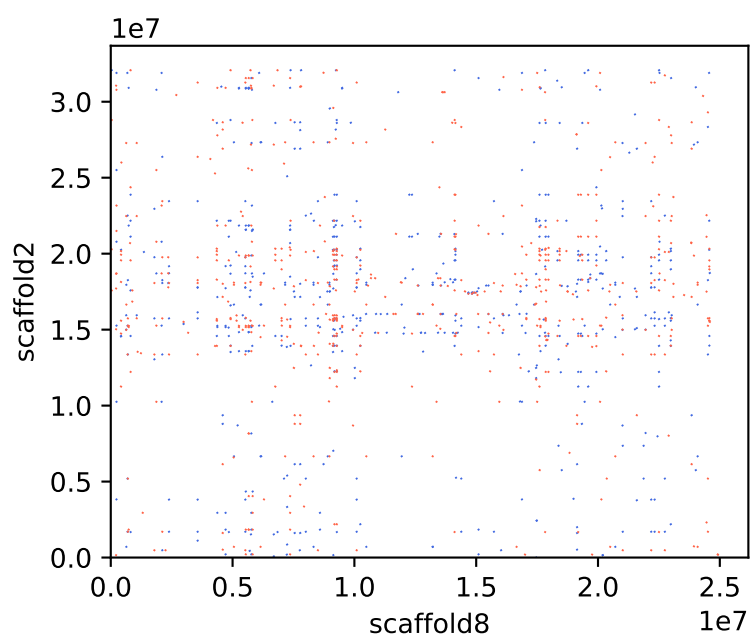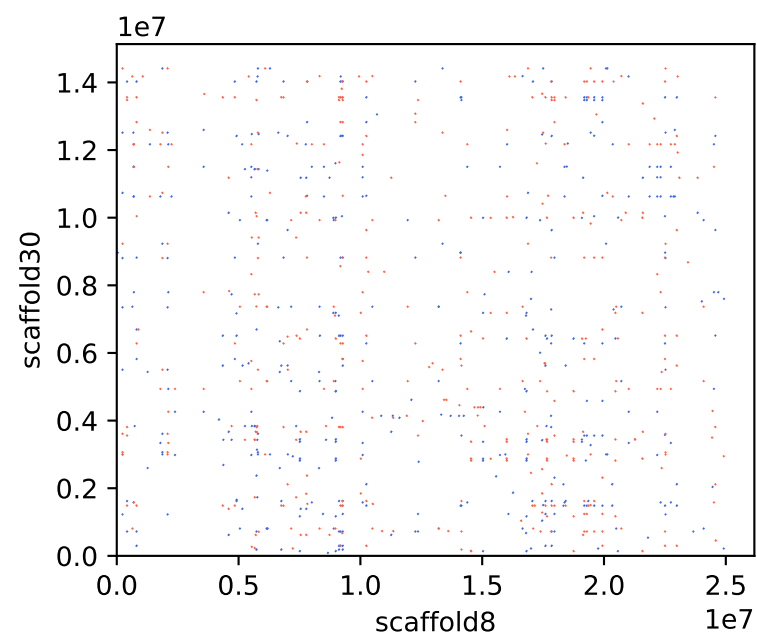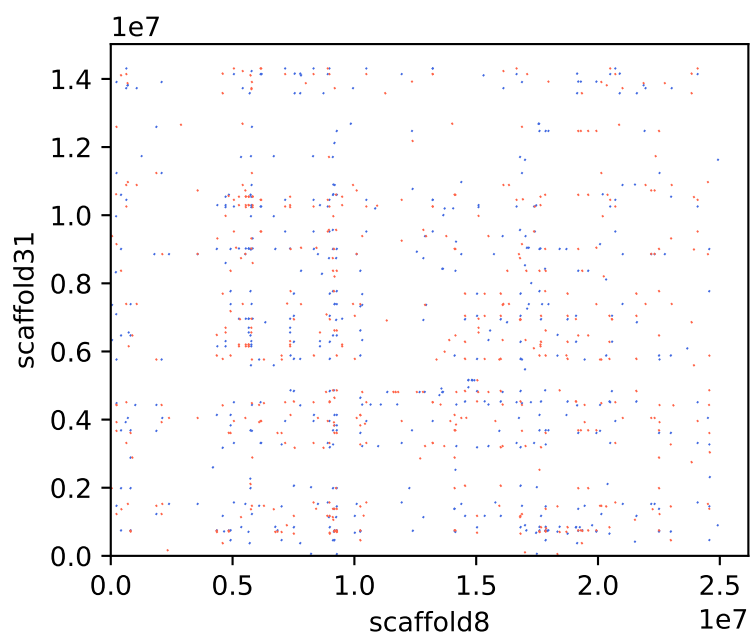

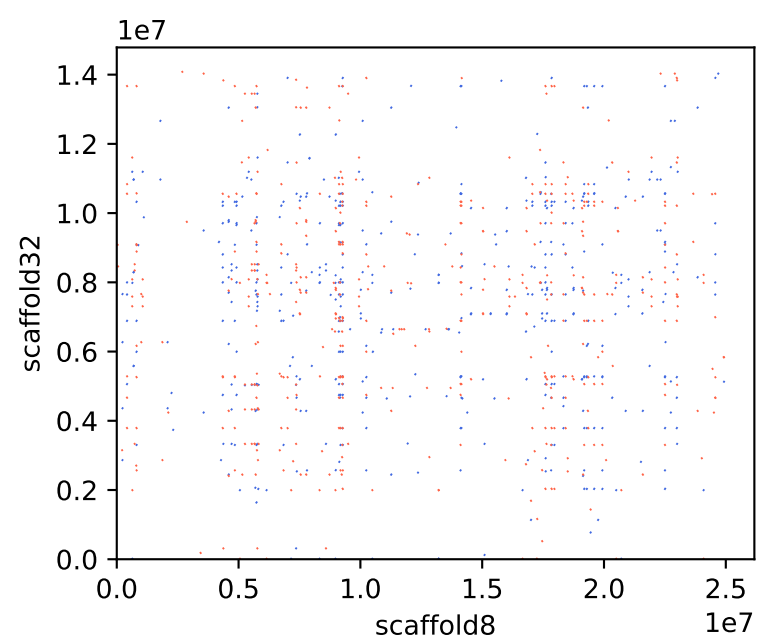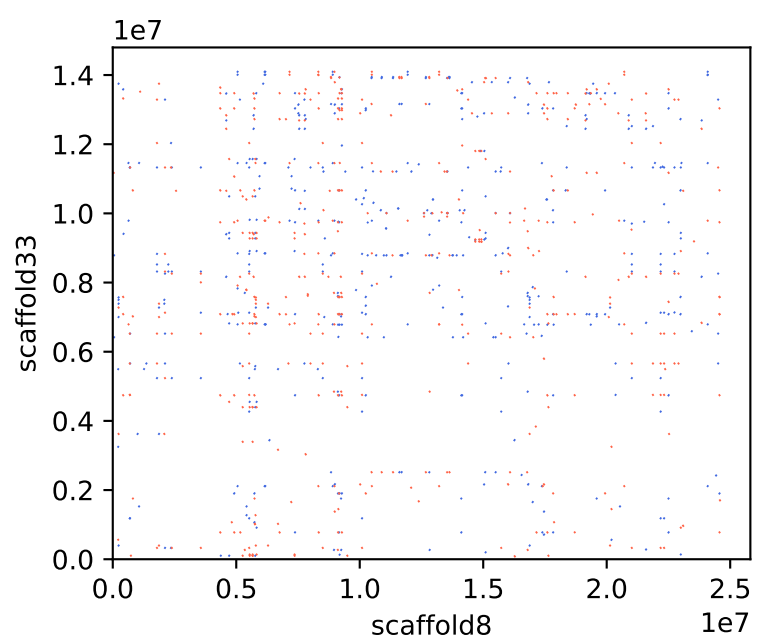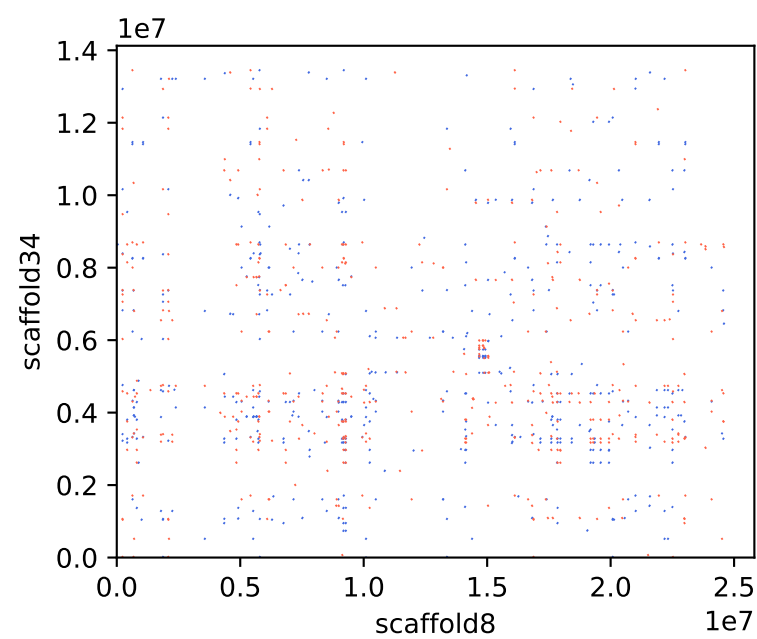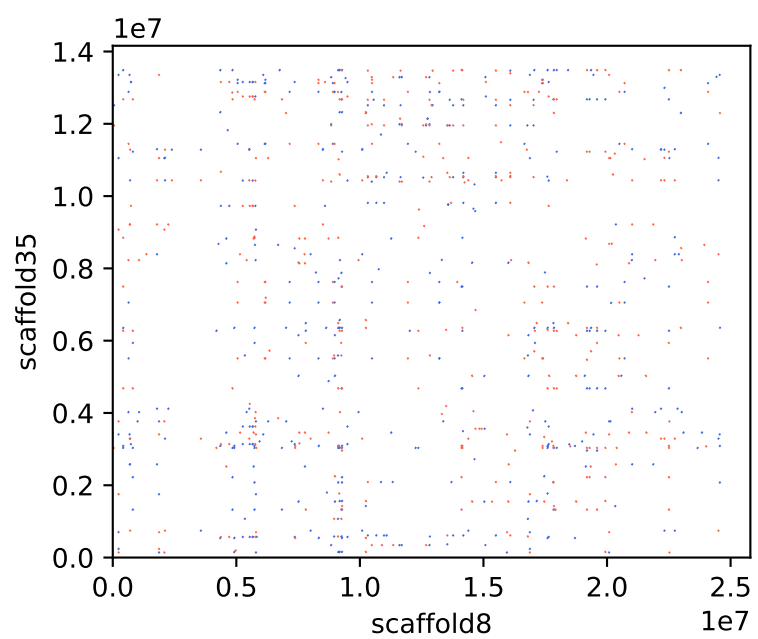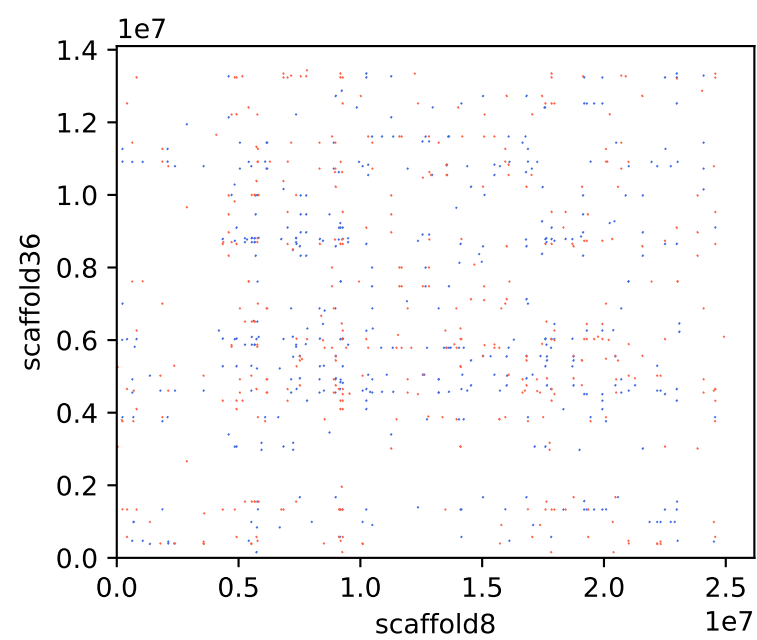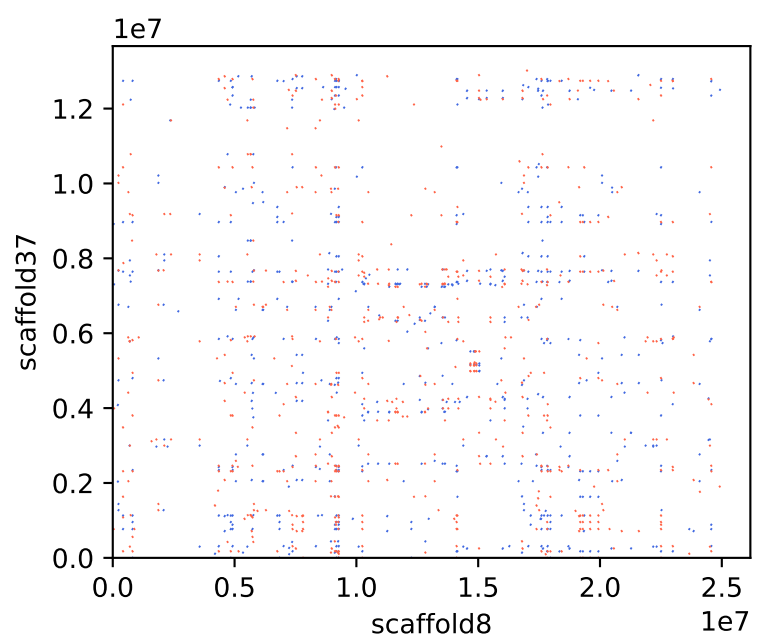

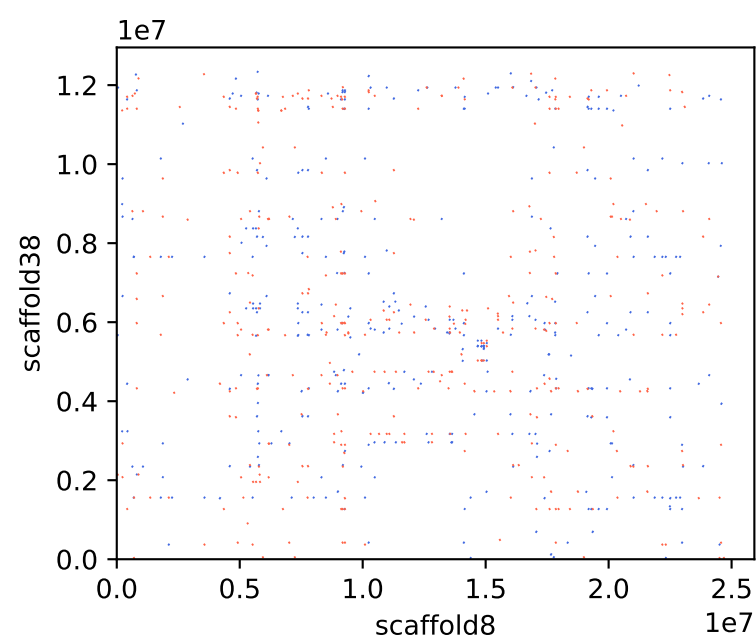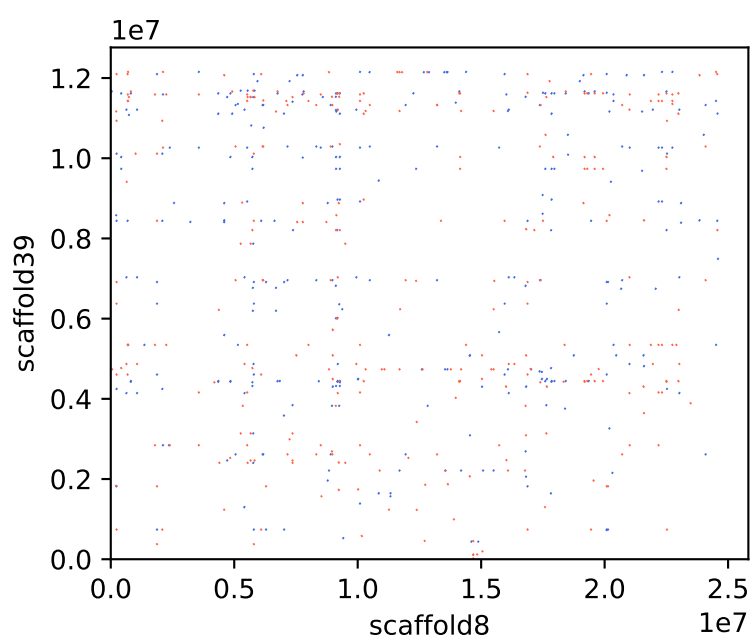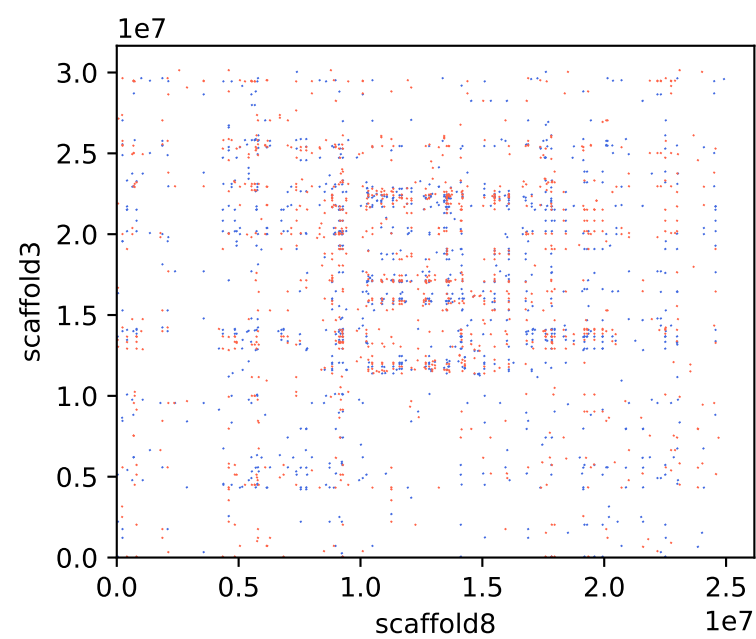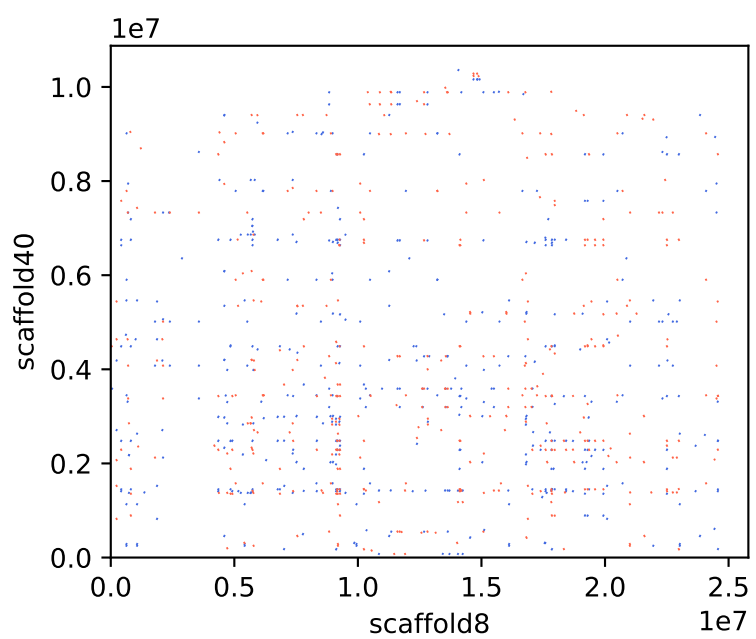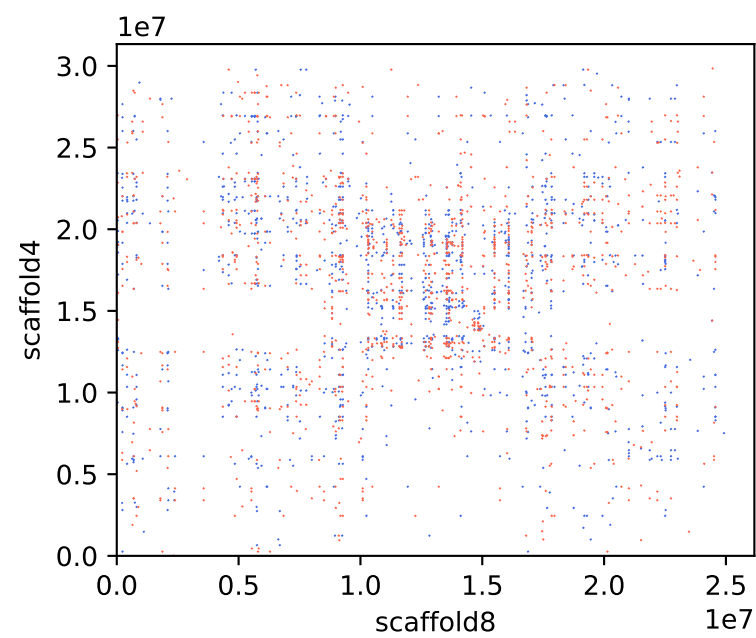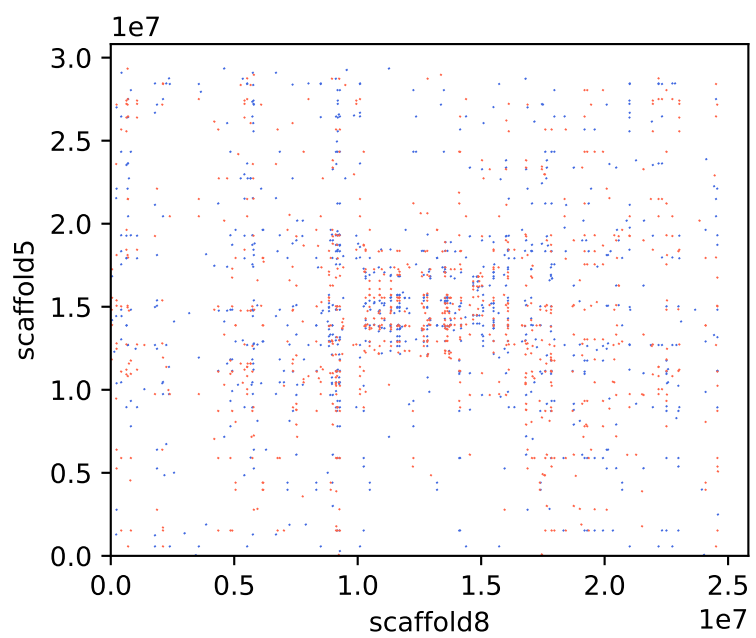

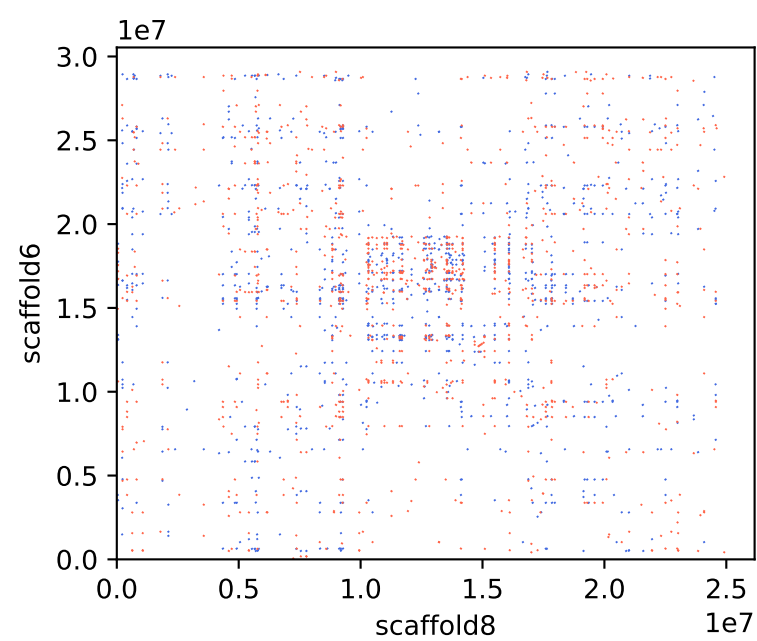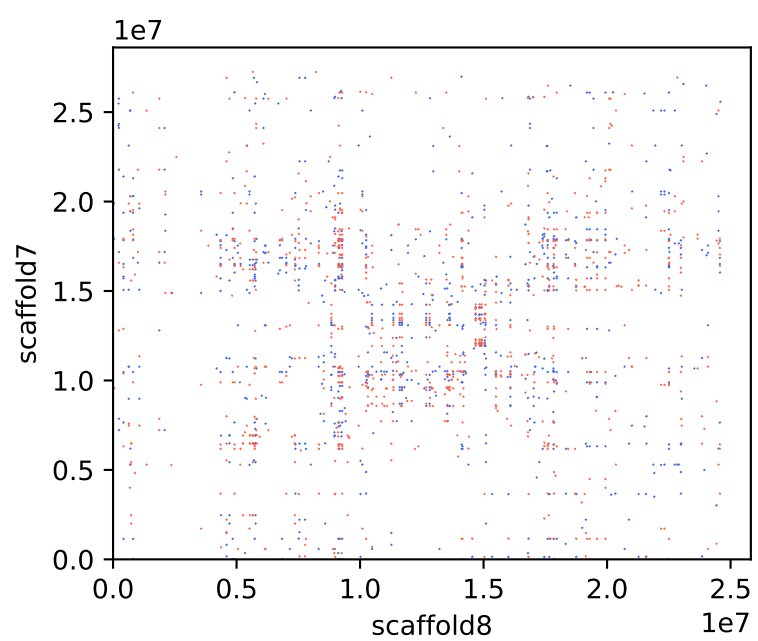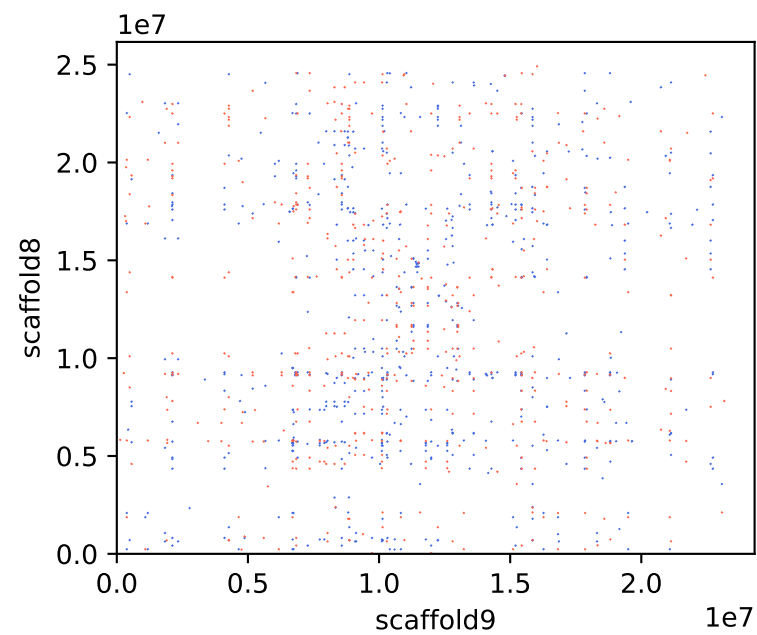

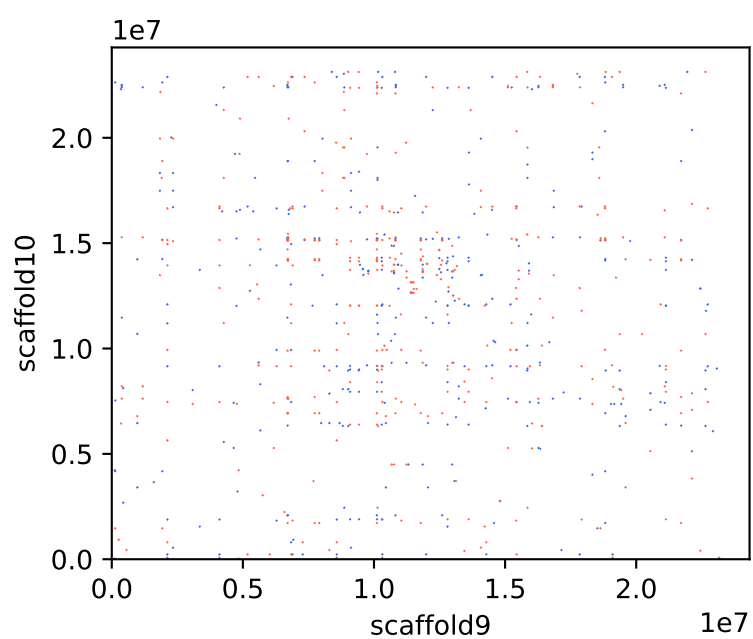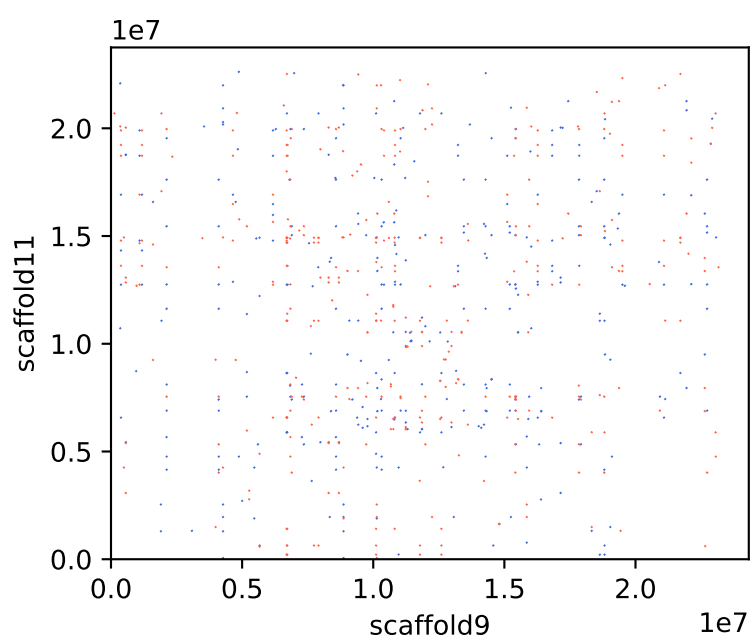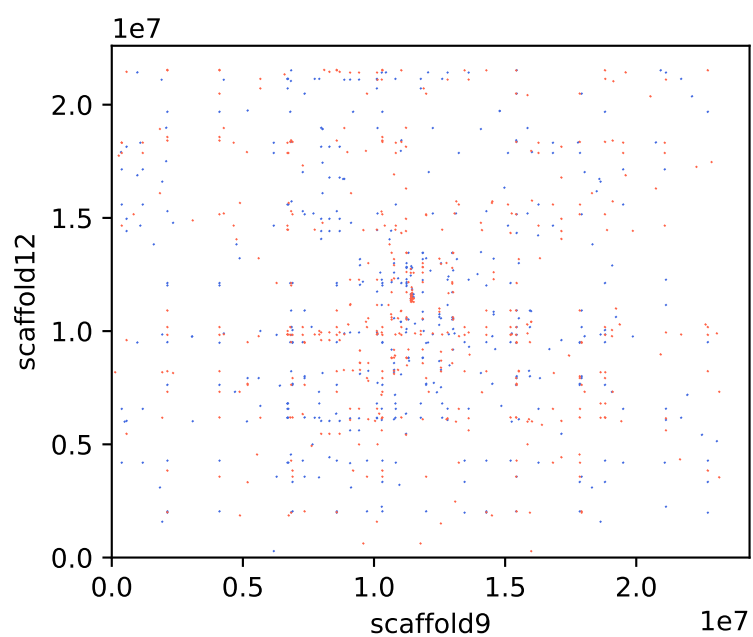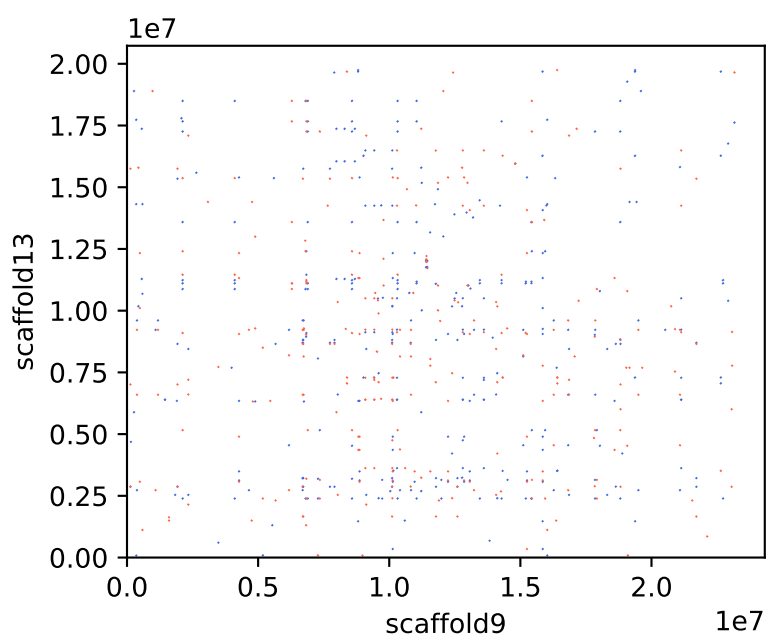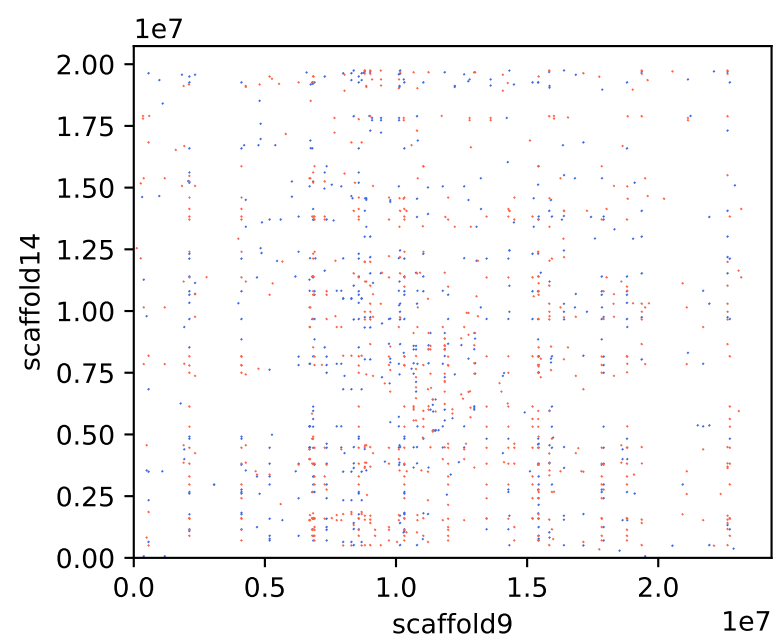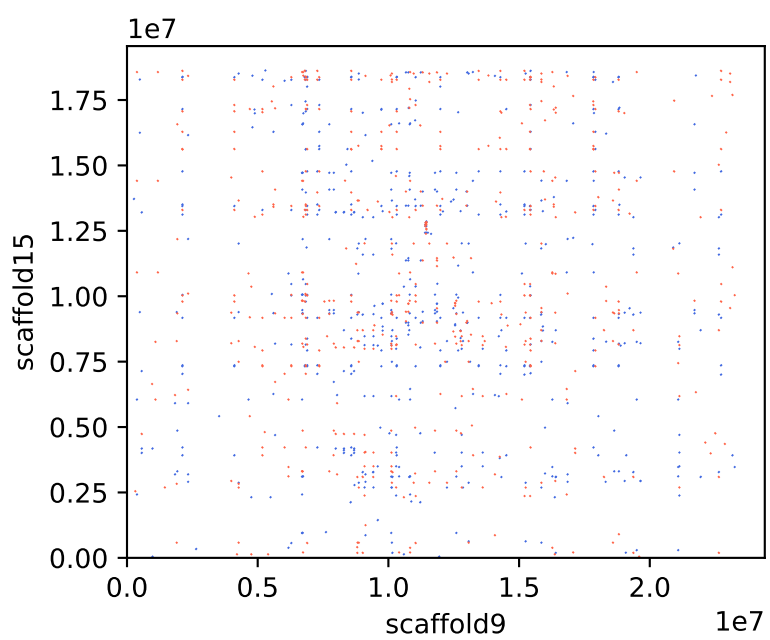

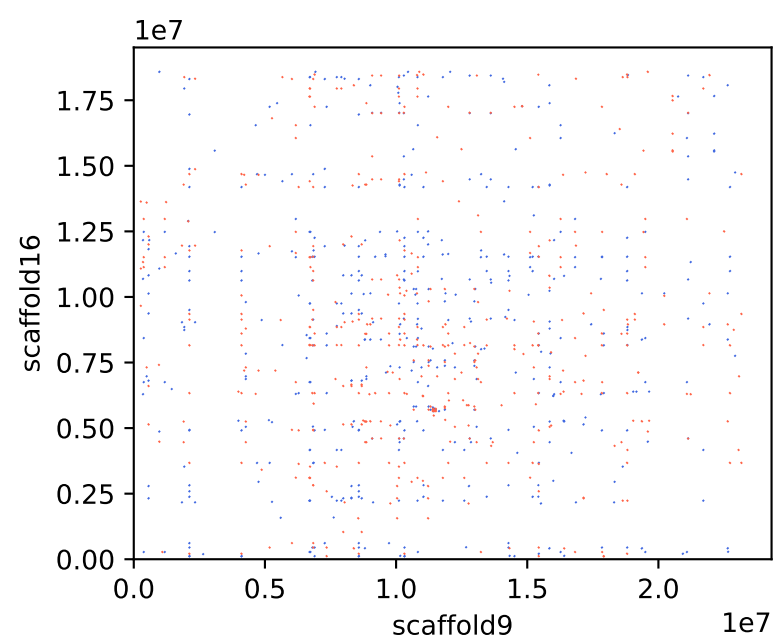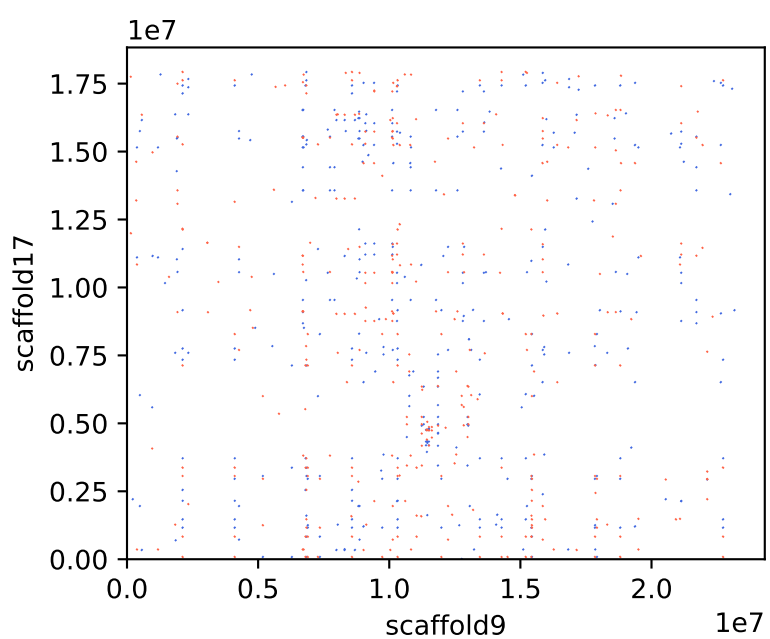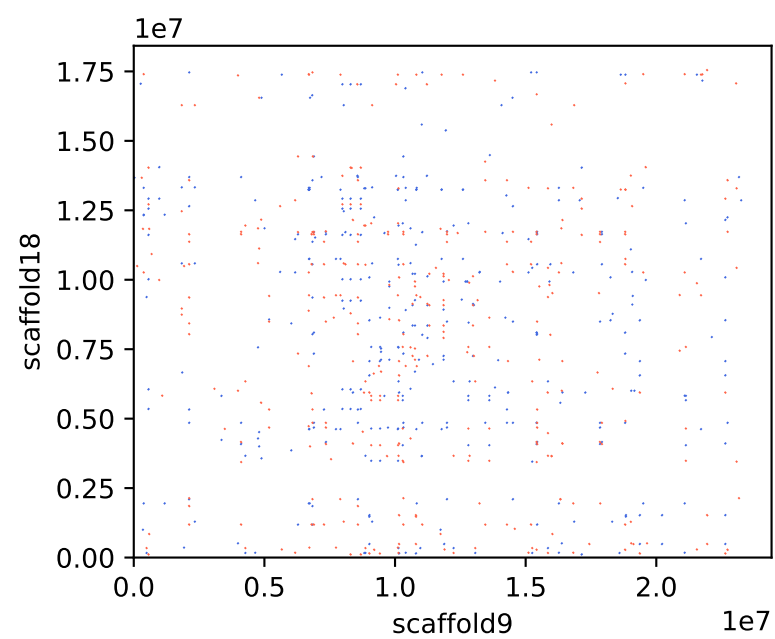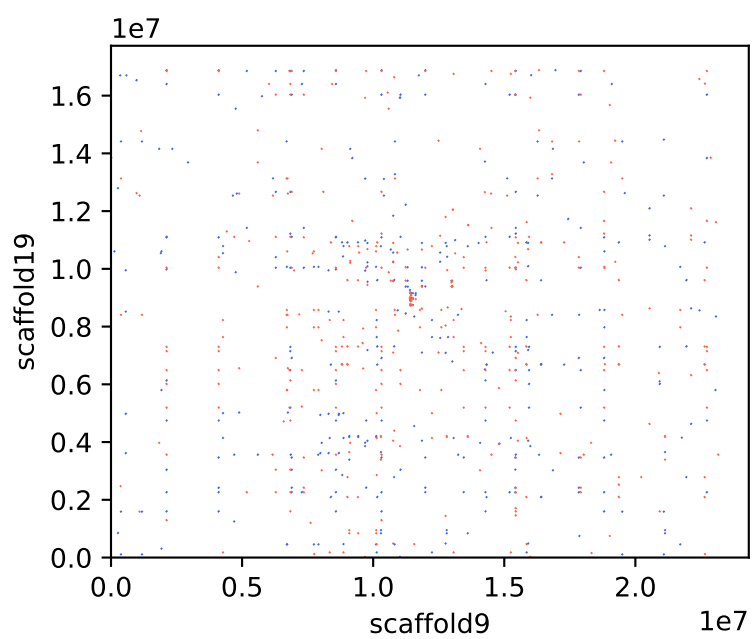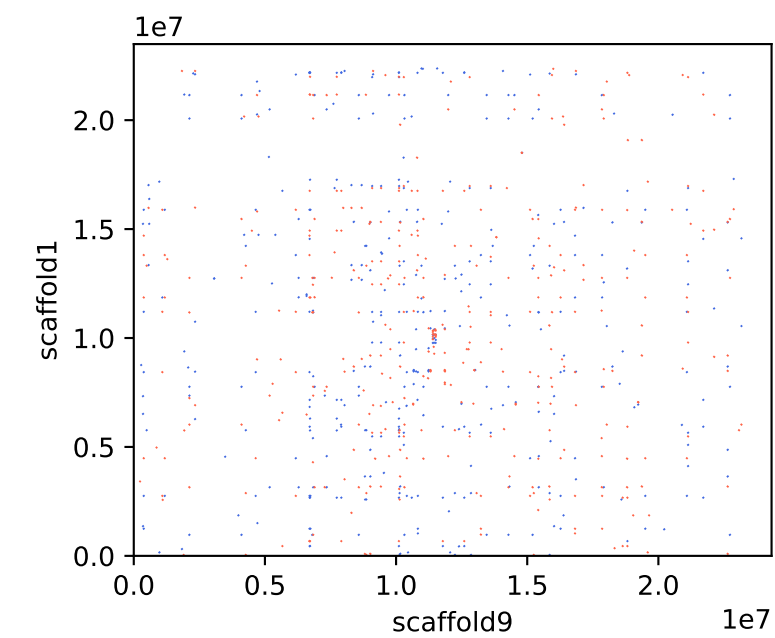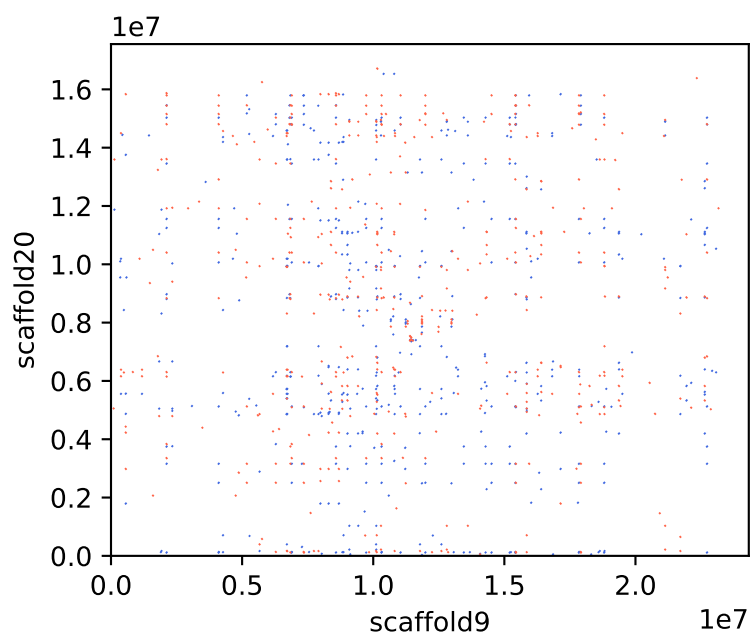

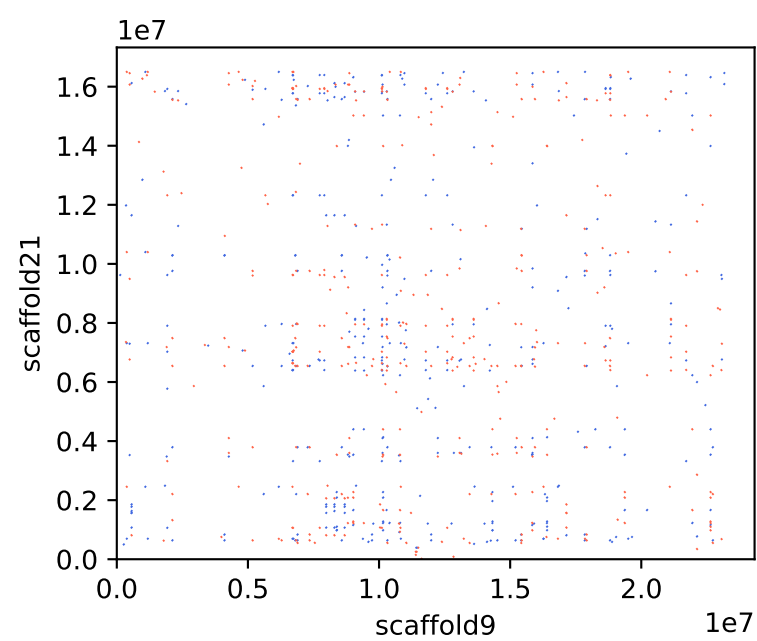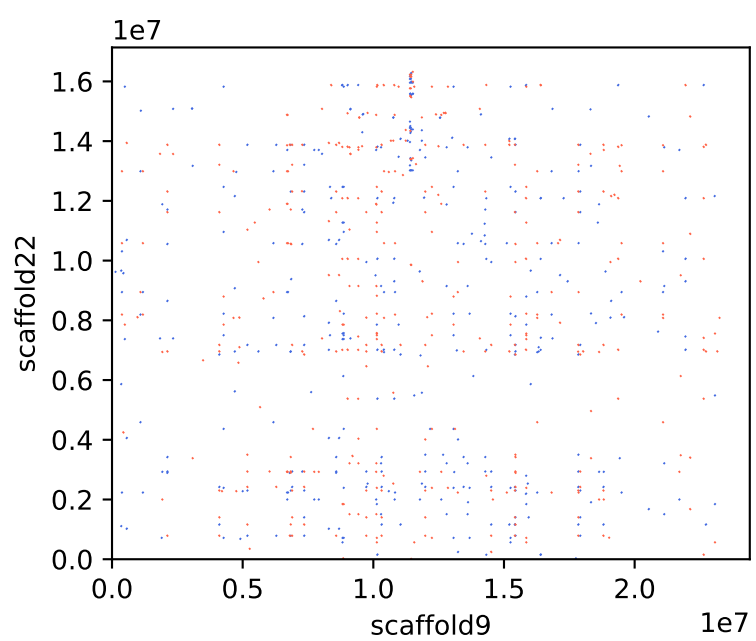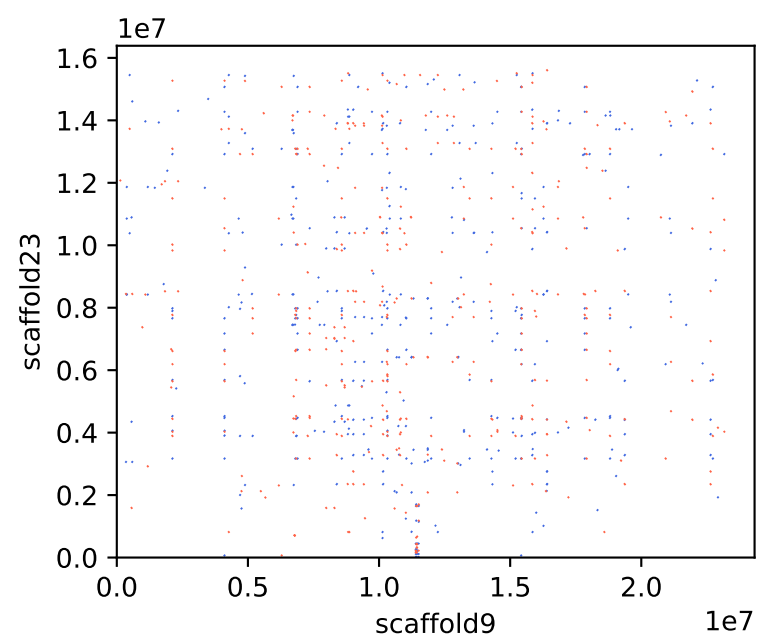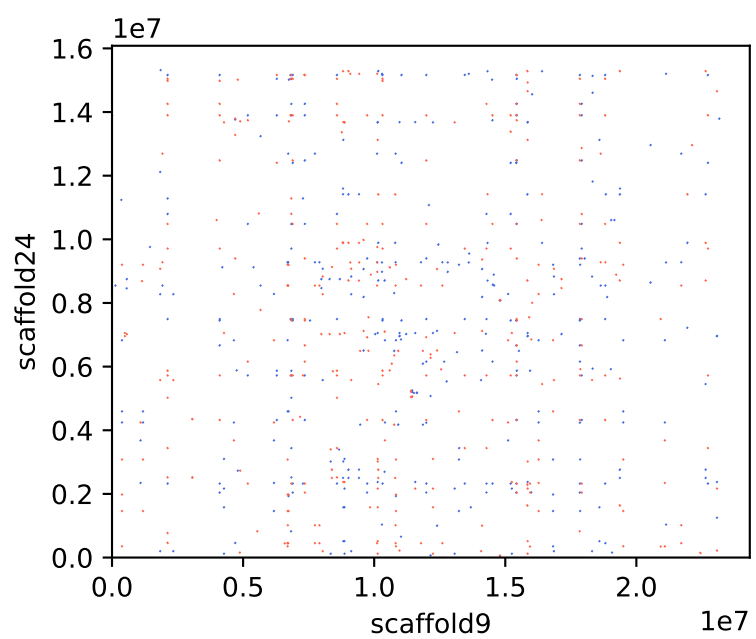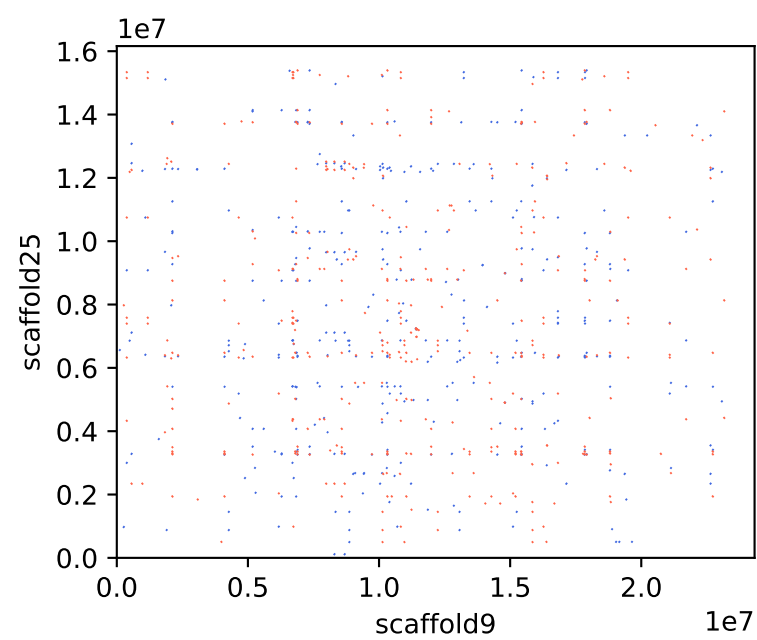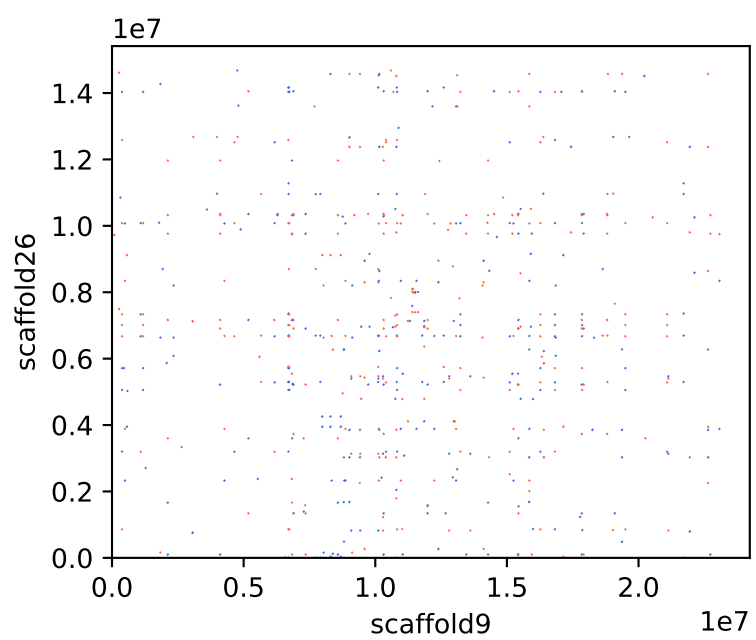

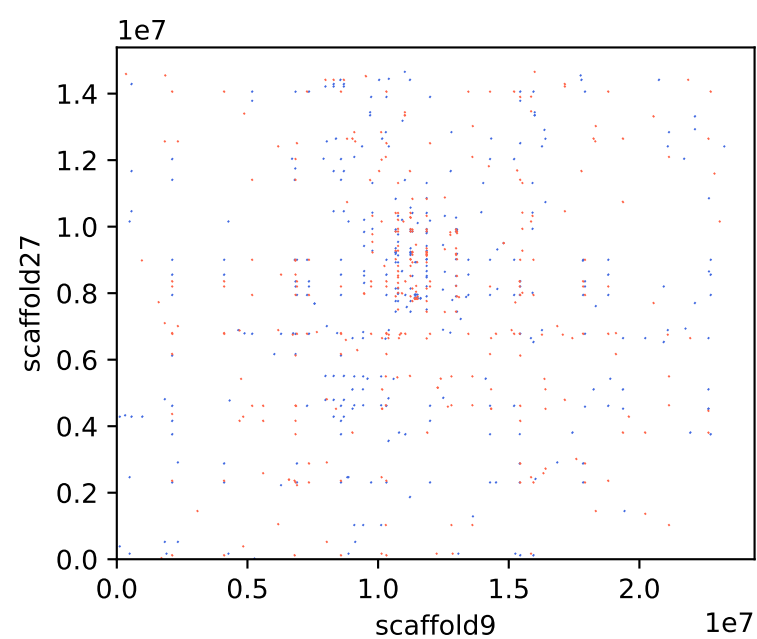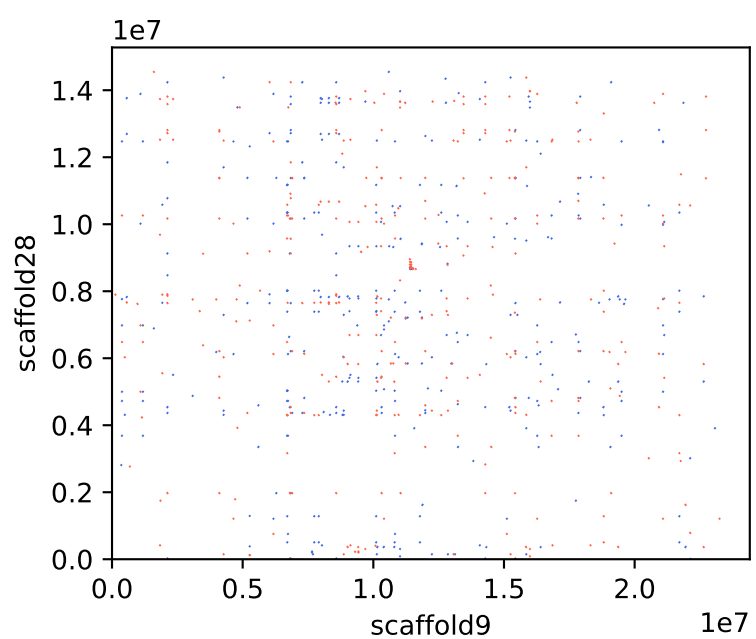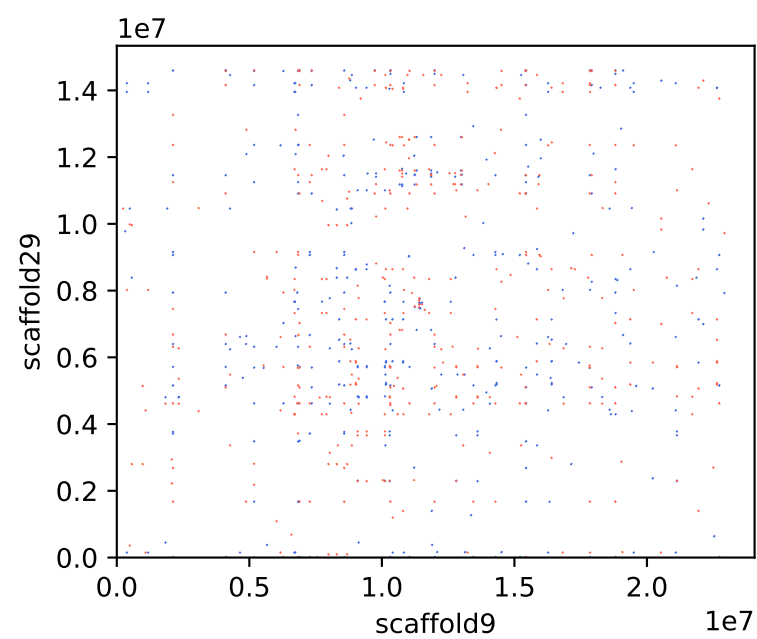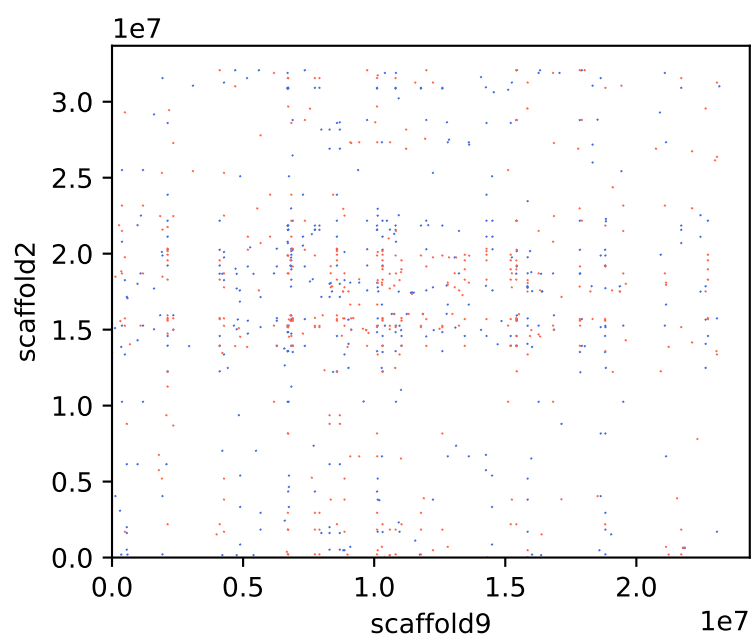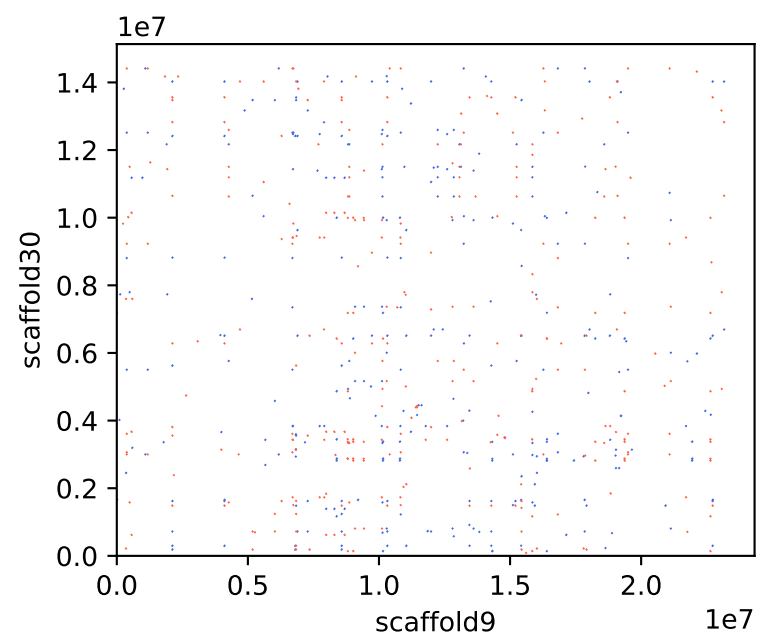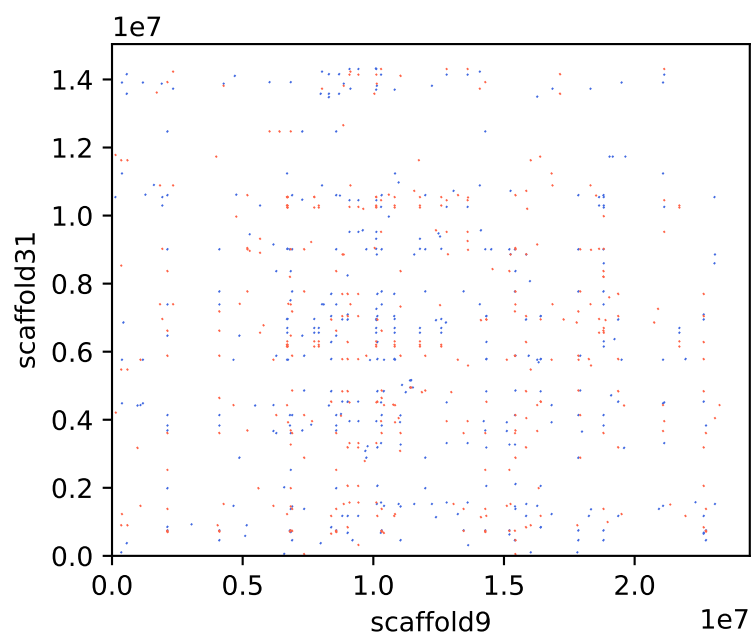

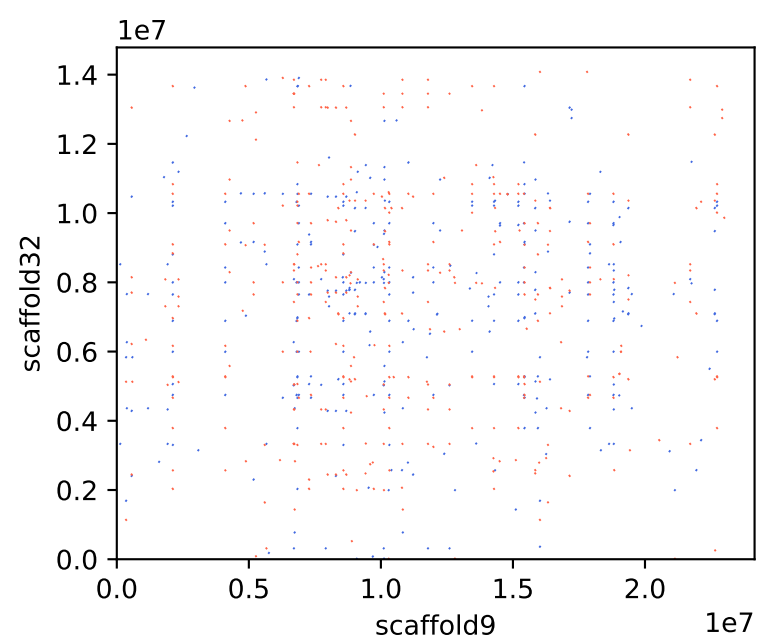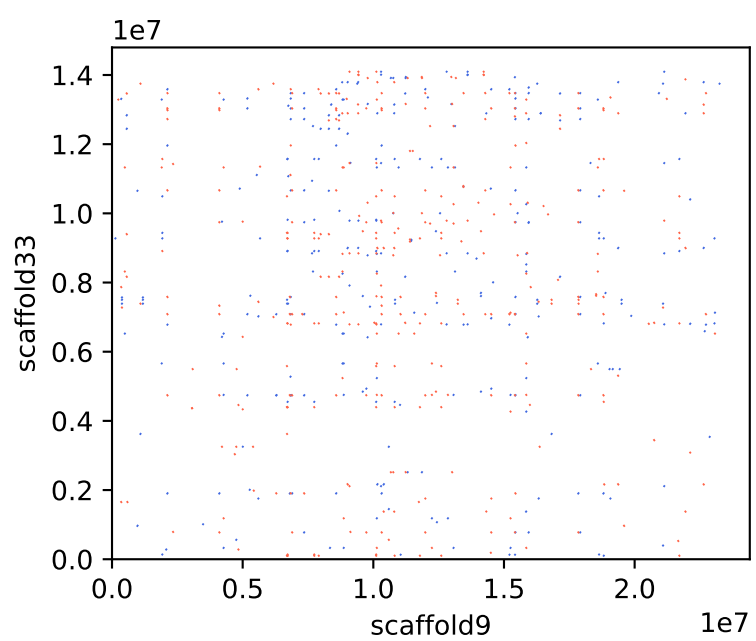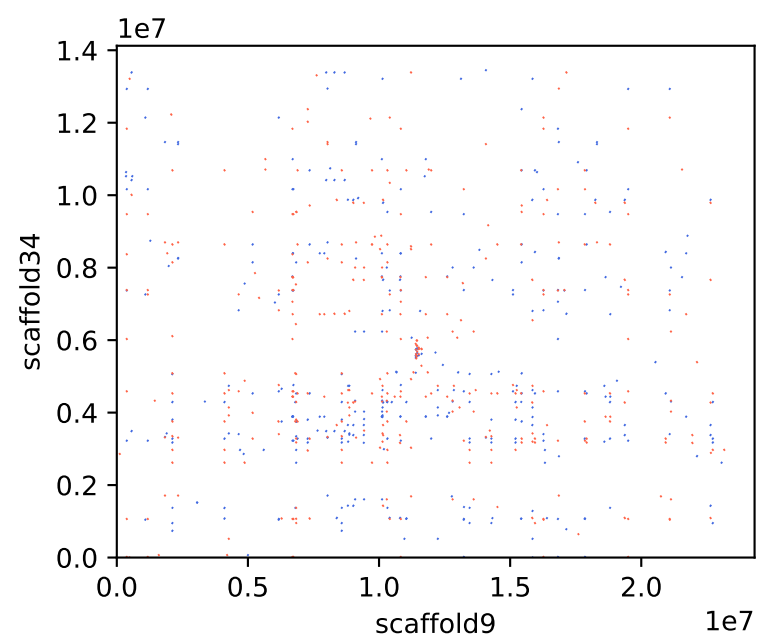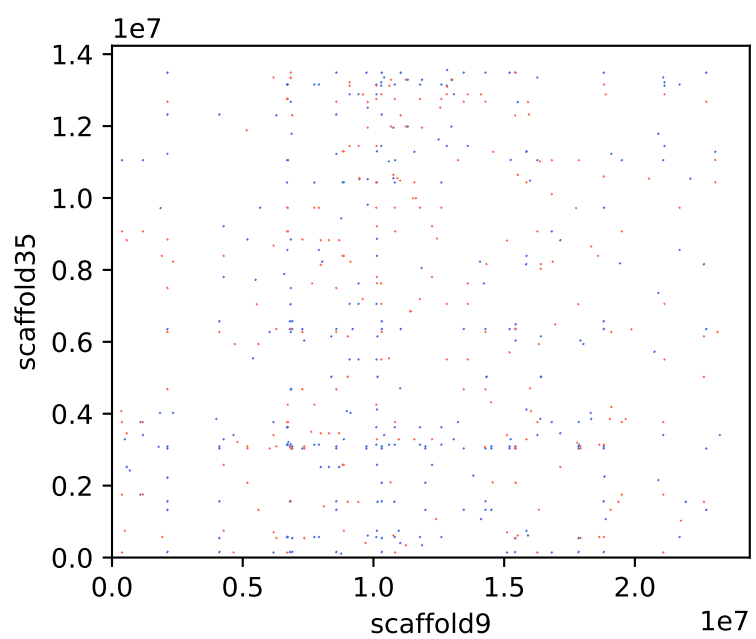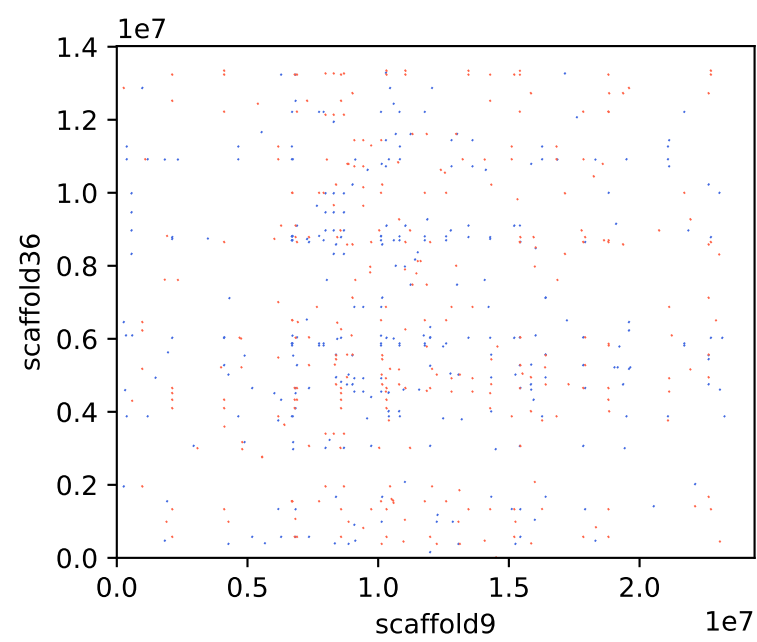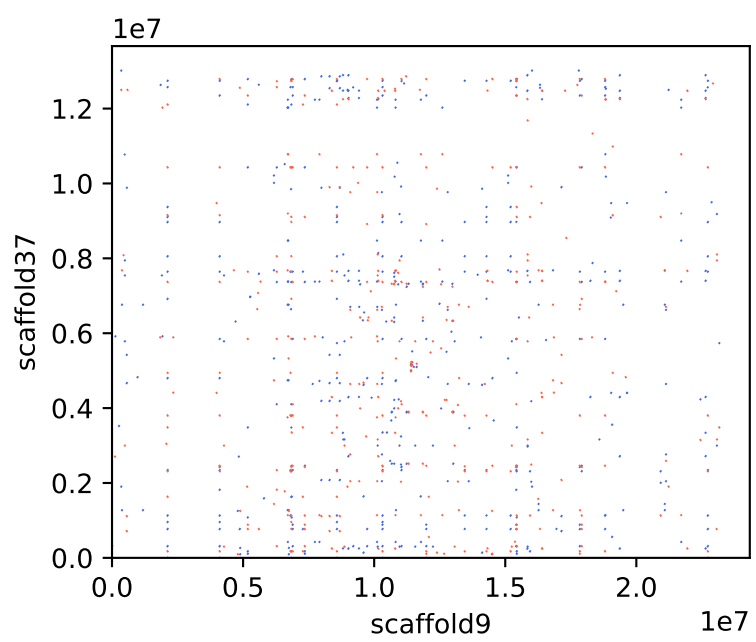

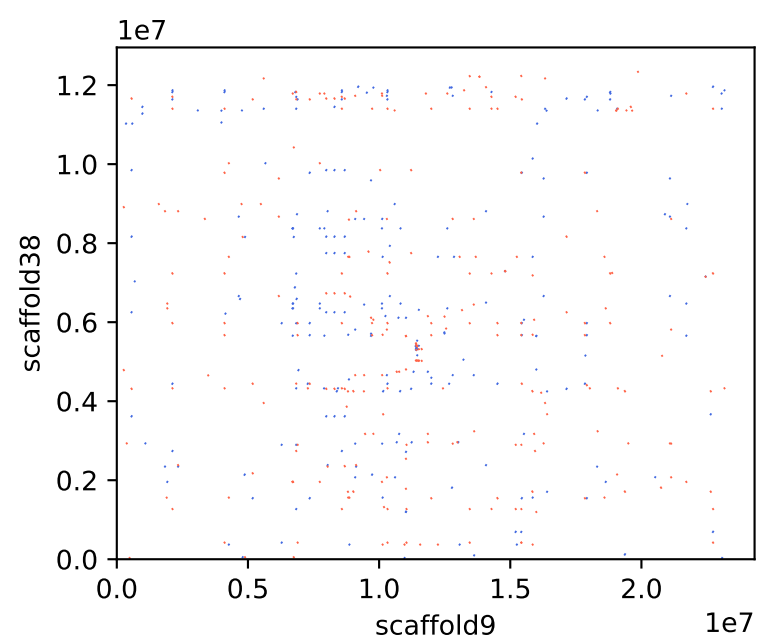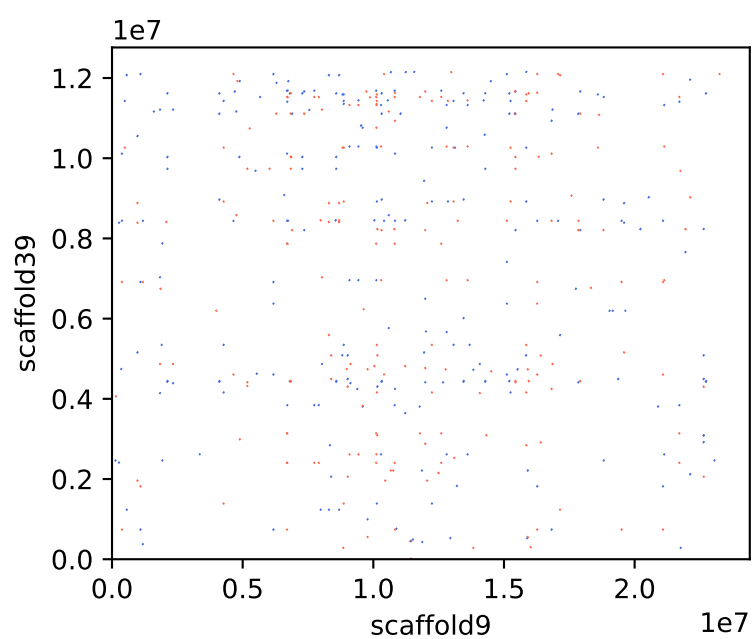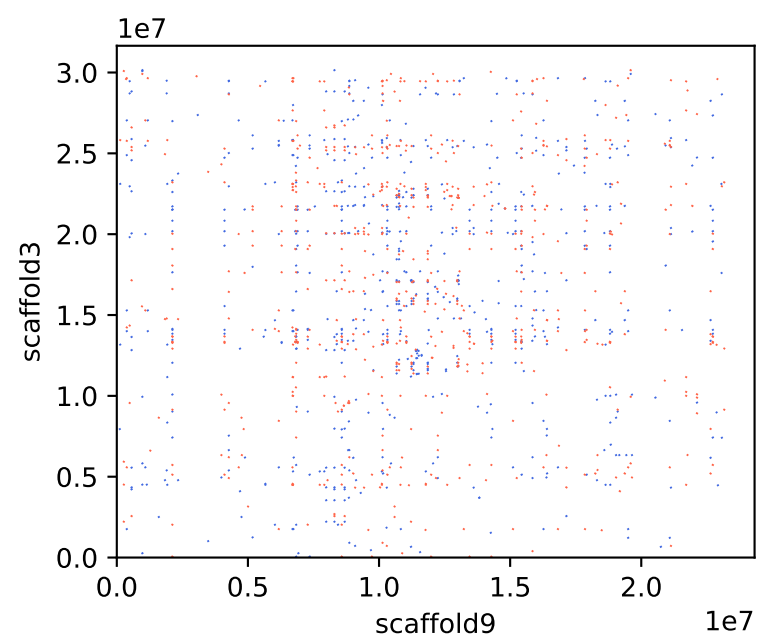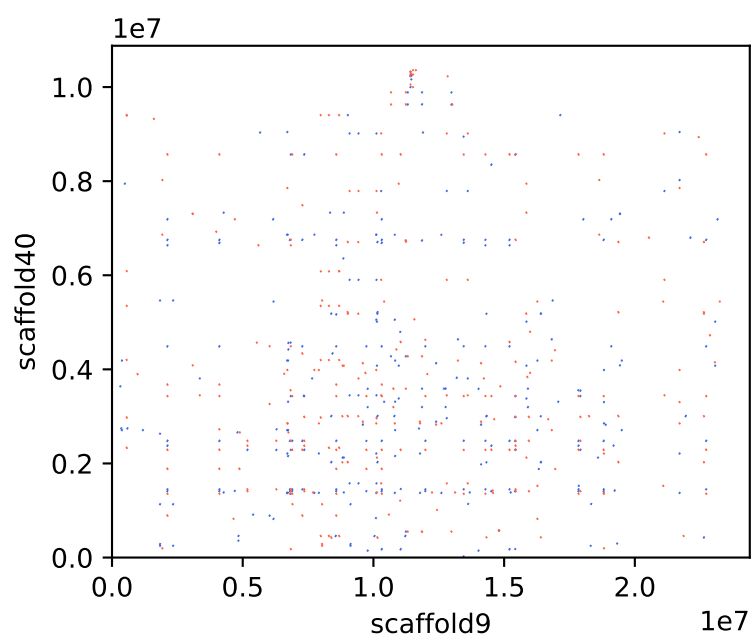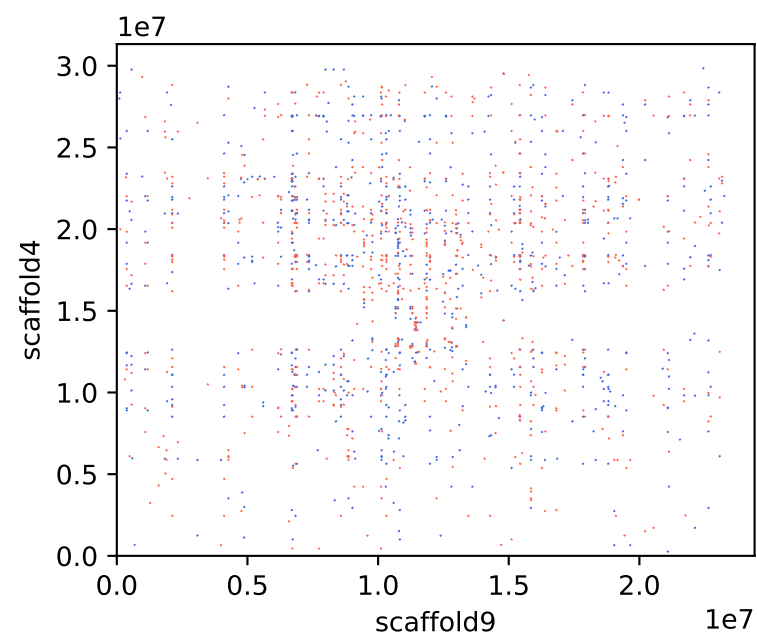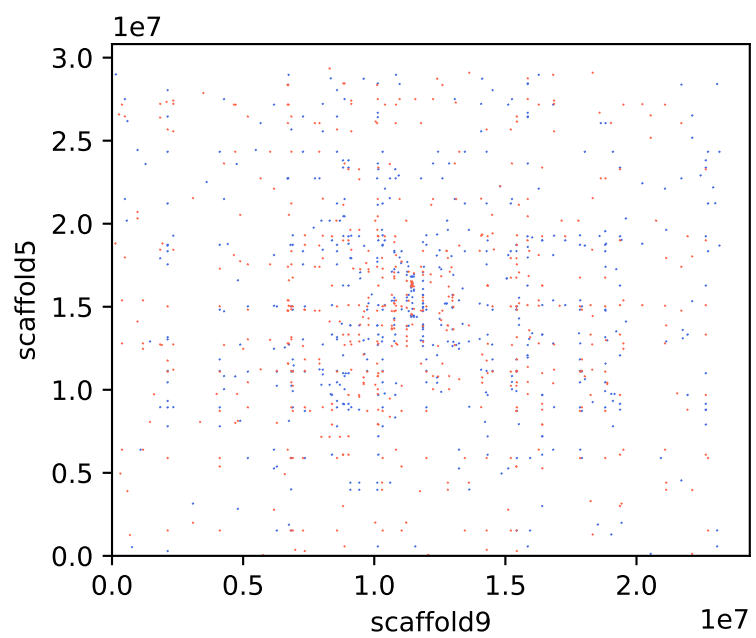

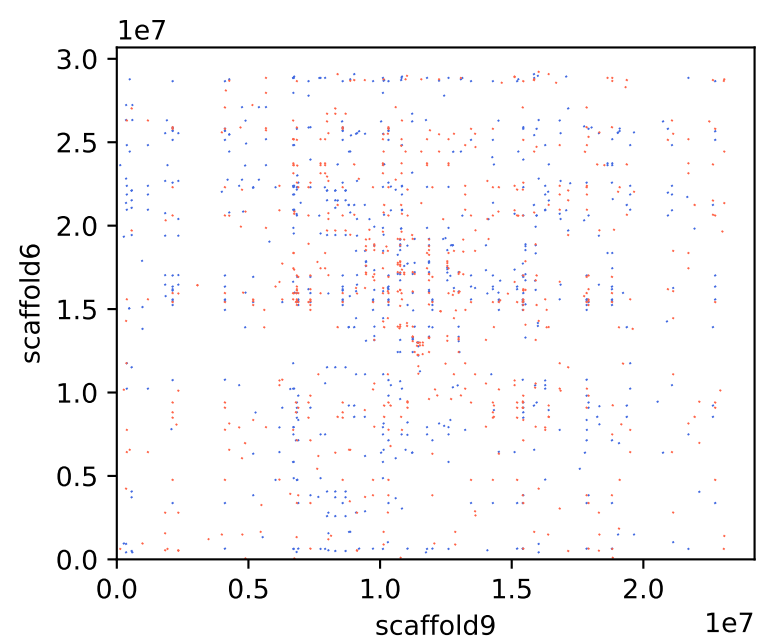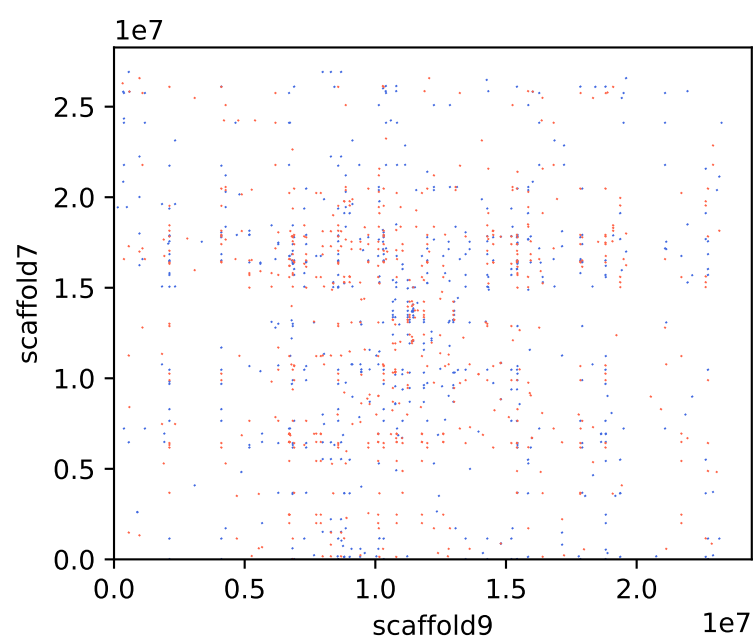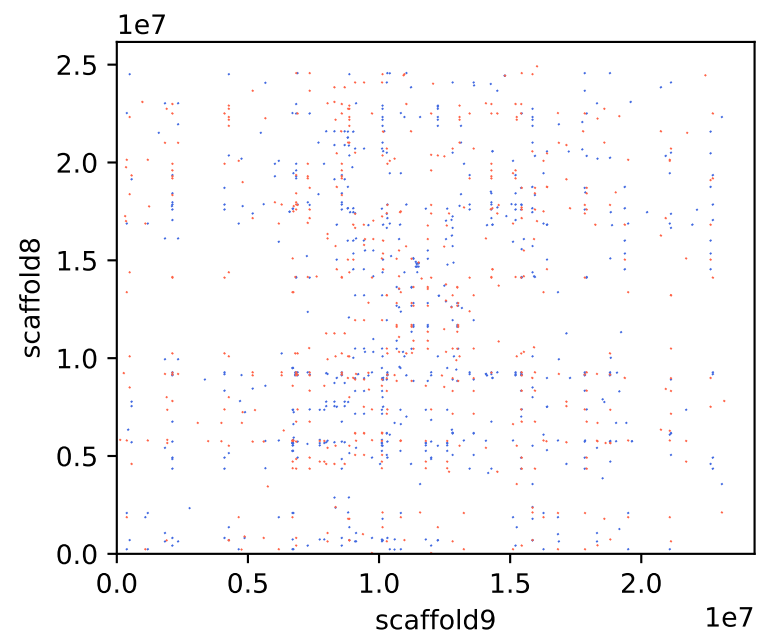

Supplement: S7 File — (PDF) [file pone.0322885.s007.pdf]
