# Supplementary material for: A high quality genome of the common swamp pitcher plant (Nepenthes mirabilis) using PacBio HiFi sequencing
Source: PLoS One. 2025 Jul 10;20(7):e0322885. doi: 10.1371/journal.pone.0322885 (PMC12244726; doi:10.1371/journal.pone.0322885)
Supplement: S5 File — (PDF) [file pone.0322885.s005.pdf]

Supplementary Figure S5. **Plot of the location of genes, tandem repeats and transposons mapped against individual *N. mirabilis* contigs.** Red horizontal bar: contig sequence. Grey horizontal bar: contig coordinates of all-vs-all contig dot plot rectangle hit regions (see manuscript text). Blue dots: location of individual genes. Horizontal lines of purple dots: tandem repeats grouped by period size range (from bottom to top: period 5–20, period 21–50, period 51–100, period 101–200, period 201–500, period >500). Green/yellow/orange/red dots: individual transposons, with each horizontal line of dots corresponding to multiple locations for a single transposon. Only transposons where >=70% of the copies occur within the all-vs-all contig dot plot rectangle hit region for >=10 contigs are shown. Dots are coloured by the number of contigs for which this threshold is true (e.g., in 27 of 33 contigs, >=70% of the copies occur within the rectangle hit region for a given contig). Green is the largest number of contigs (27), and red is the smallest (10); see also Supplementary File S2 Table S12).

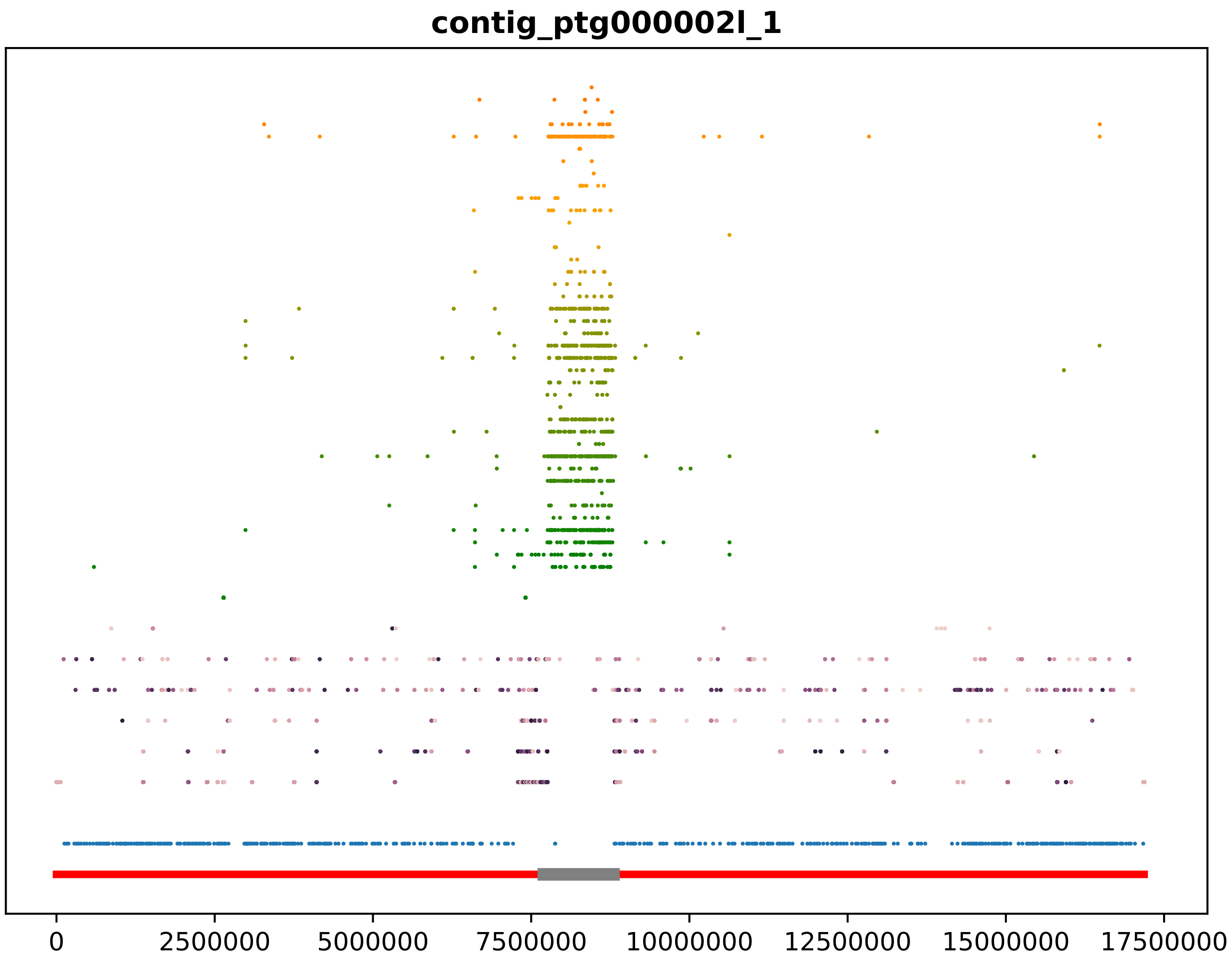

contig_ptg000002l_1
0
2500000
5000000
7500000
10000000
12500000
15000000
17500000

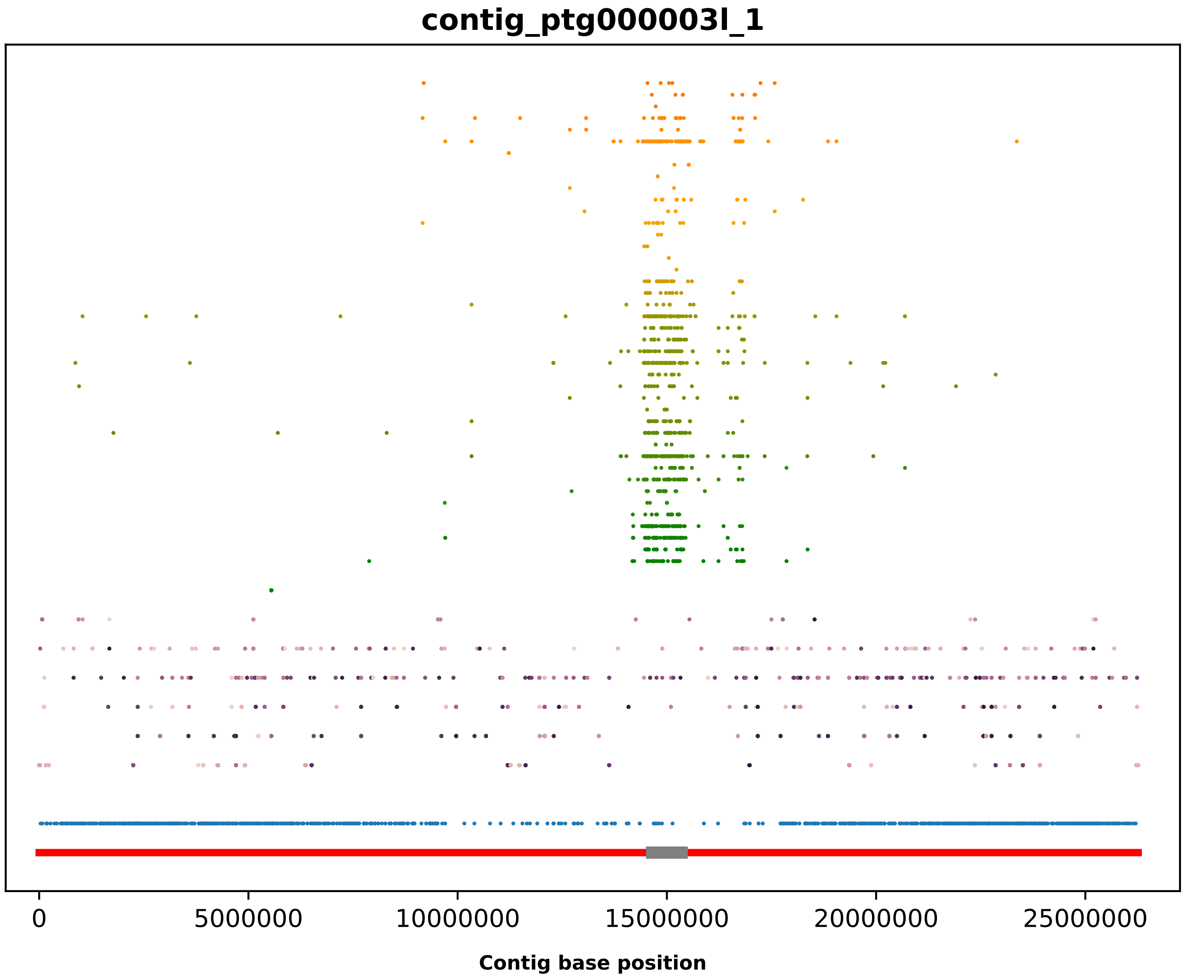

contig_ptg000003l_1
0
5000000
10000000
15000000
20000000
25000000
Contig base position

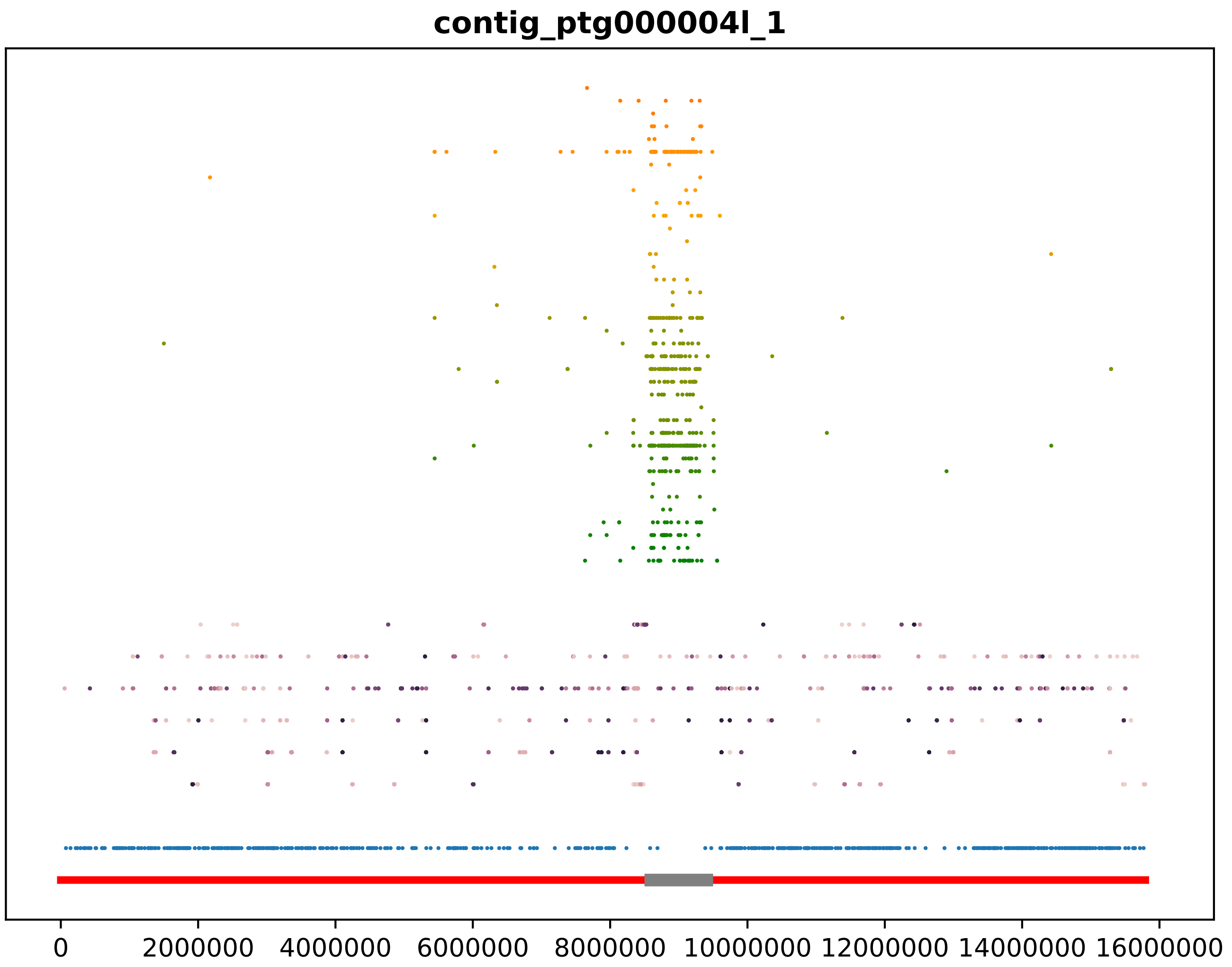
contig_ptg000004l_1
0
2000000
4000000
6000000
8000000
10000000
12000000
14000000
16000000

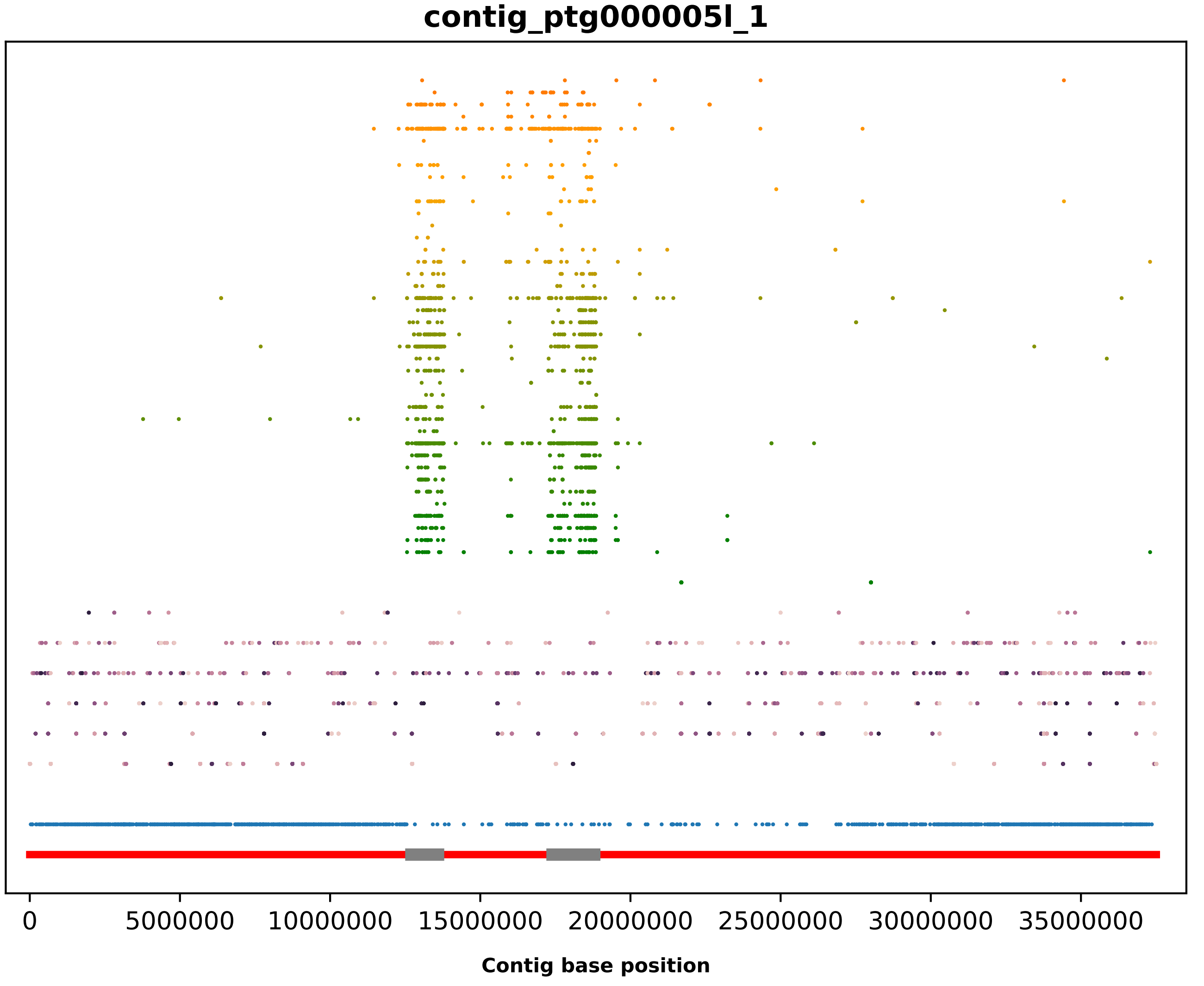
contig_ptg000005l_1
0
5000000
10000000
15000000
20000000
25000000
30000000
35000000
Contig base position

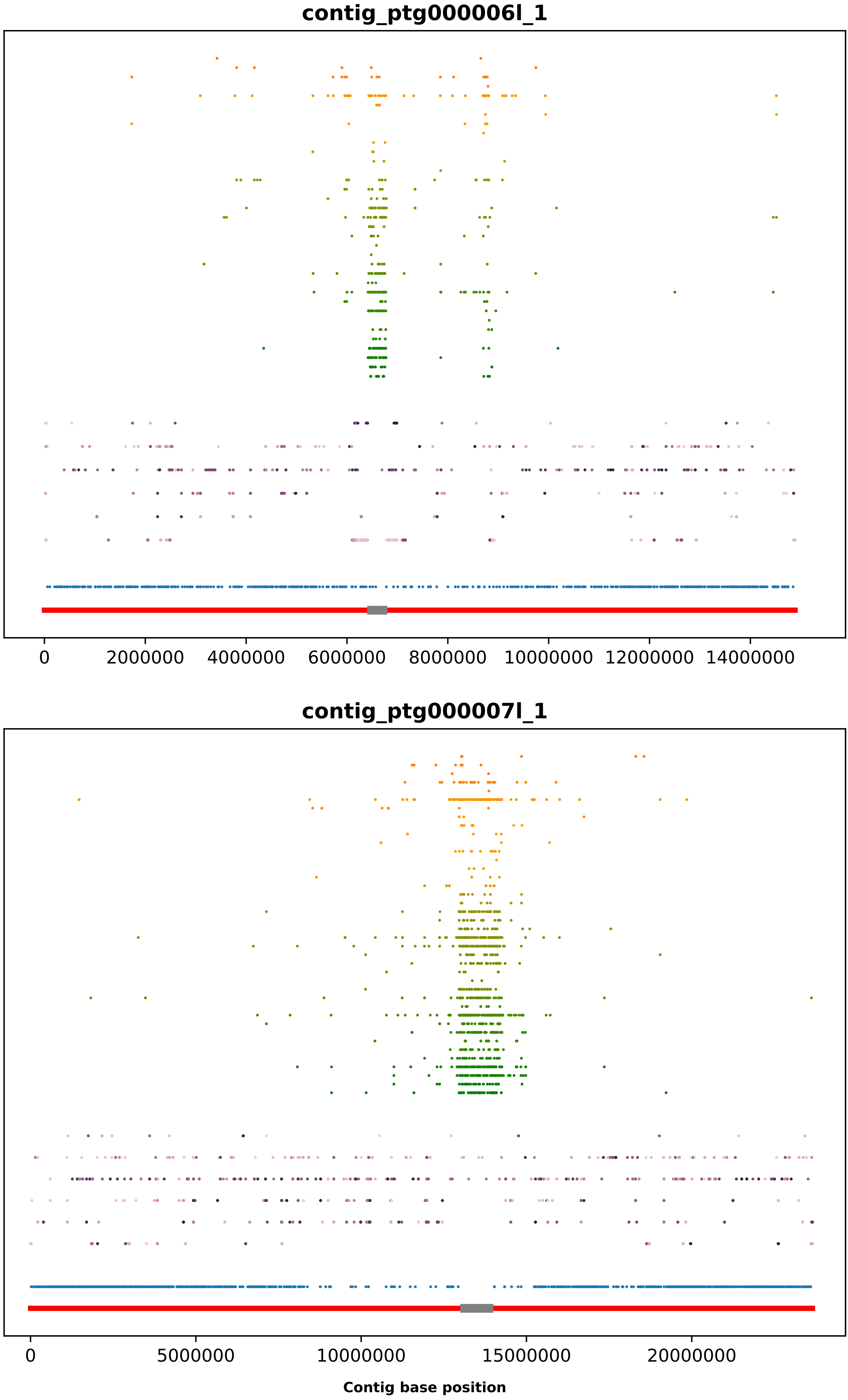

contig_ptg000006l_1
0
2000000
4000000
6000000
8000000
10000000
12000000
14000000
contig_ptg000007l_1
0
5000000
10000000
15000000
20000000
Contig base position

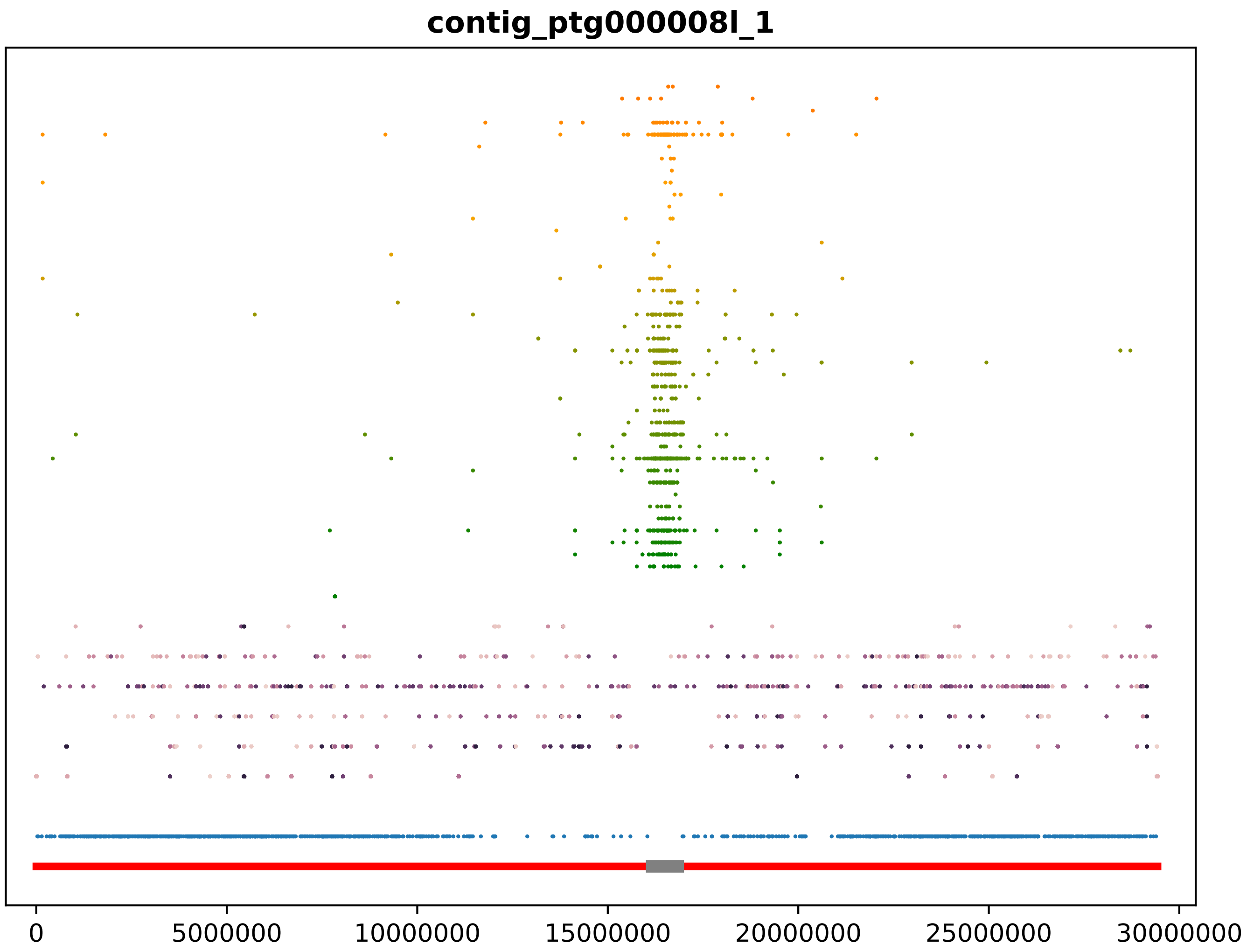
contig_ptg000008l_1
0
5000000
10000000
15000000
20000000
25000000
30000000

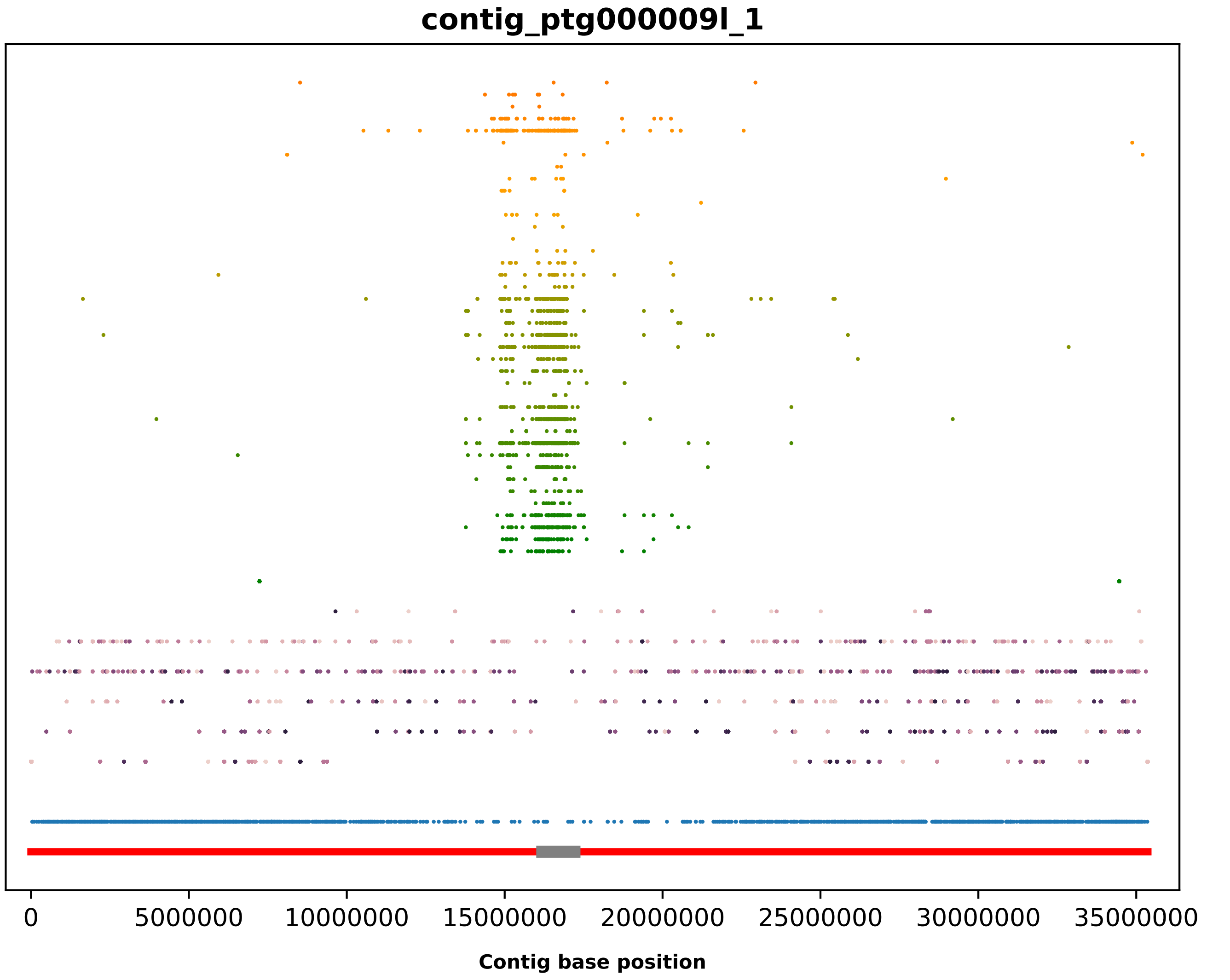
contig_ptg000009l_1
0
5000000
10000000
15000000
20000000
25000000
30000000
35000000
Contig base position

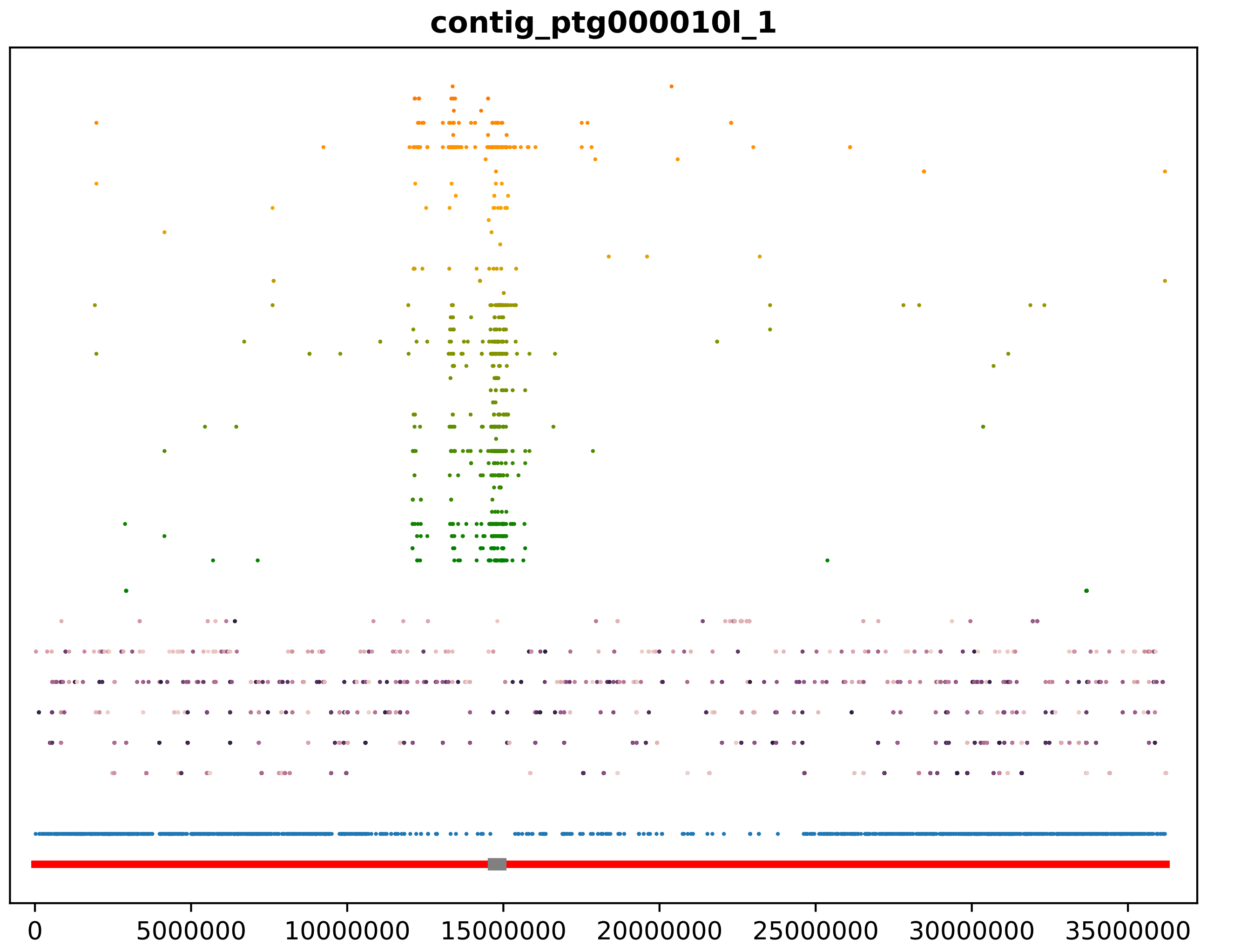

contig_ptg000010l_1
0
5000000
10000000
15000000
20000000
25000000
30000000
35000000

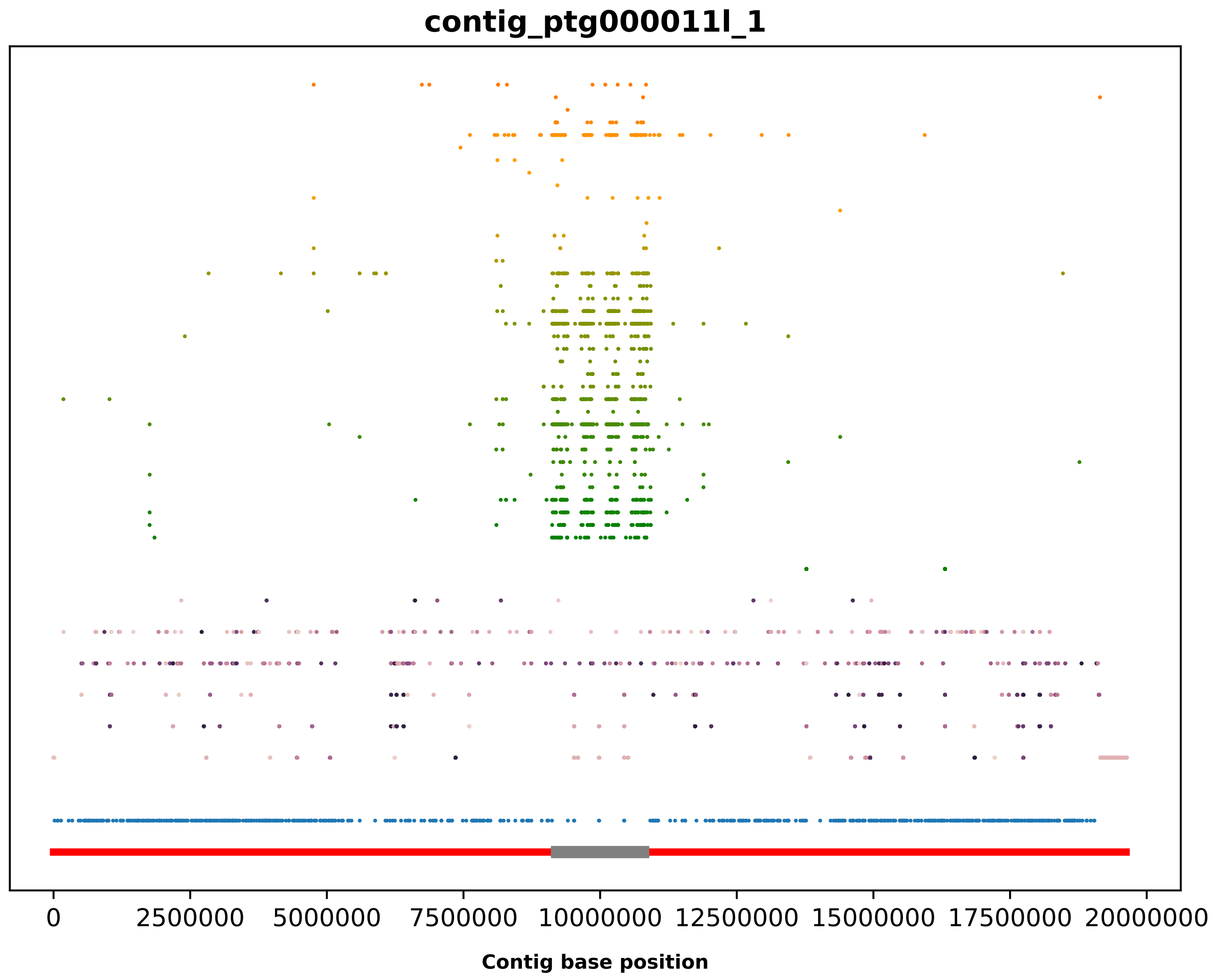

contig_ptg000011l_1
0
2500000
5000000
7500000
10000000
12500000
15000000
17500000
20000000
Contig base position

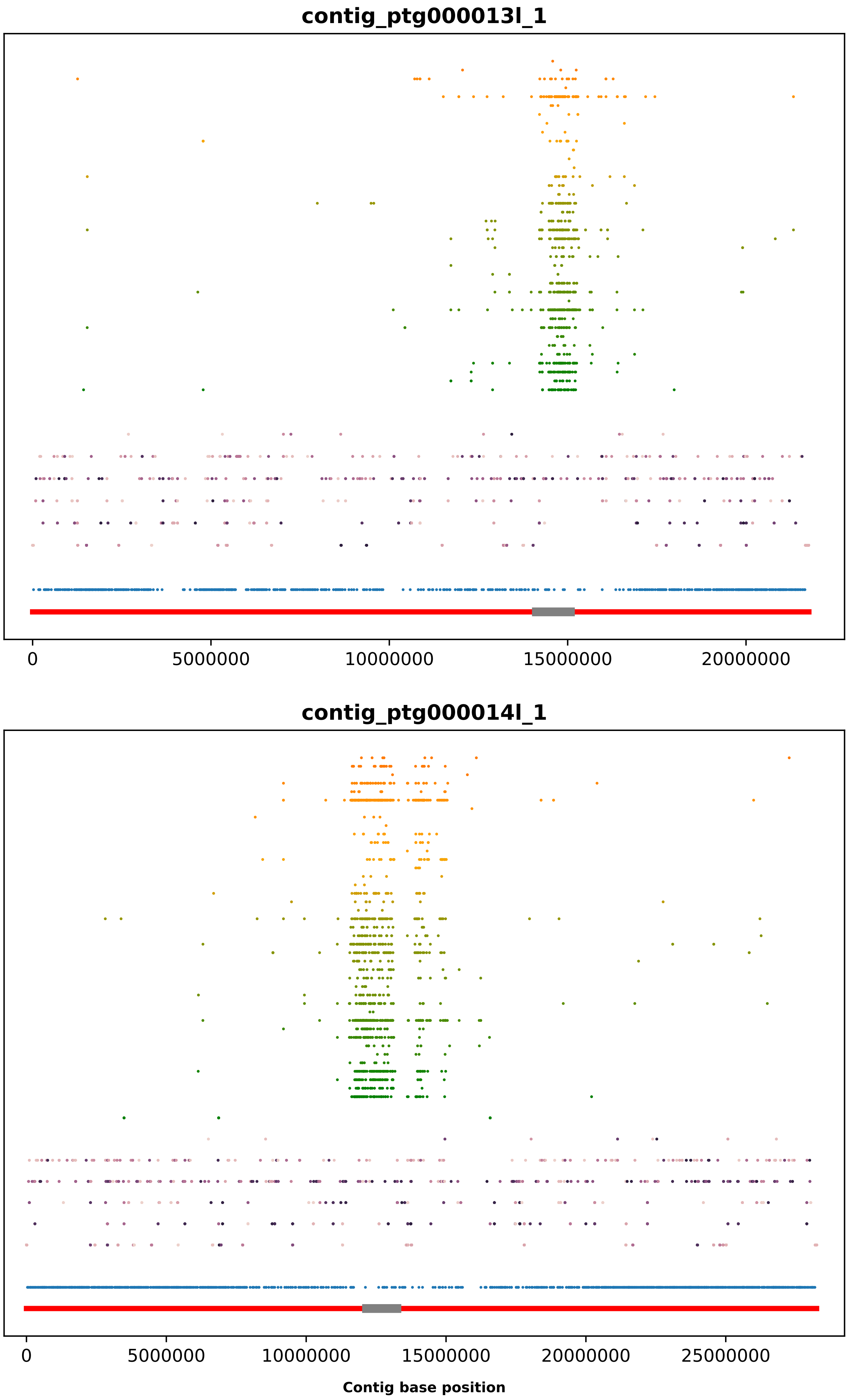

contig_ptg000013l_1
0
5000000
10000000
15000000
20000000
contig_ptg000014l_1
0
5000000
10000000
15000000
20000000
25000000
Contig base position

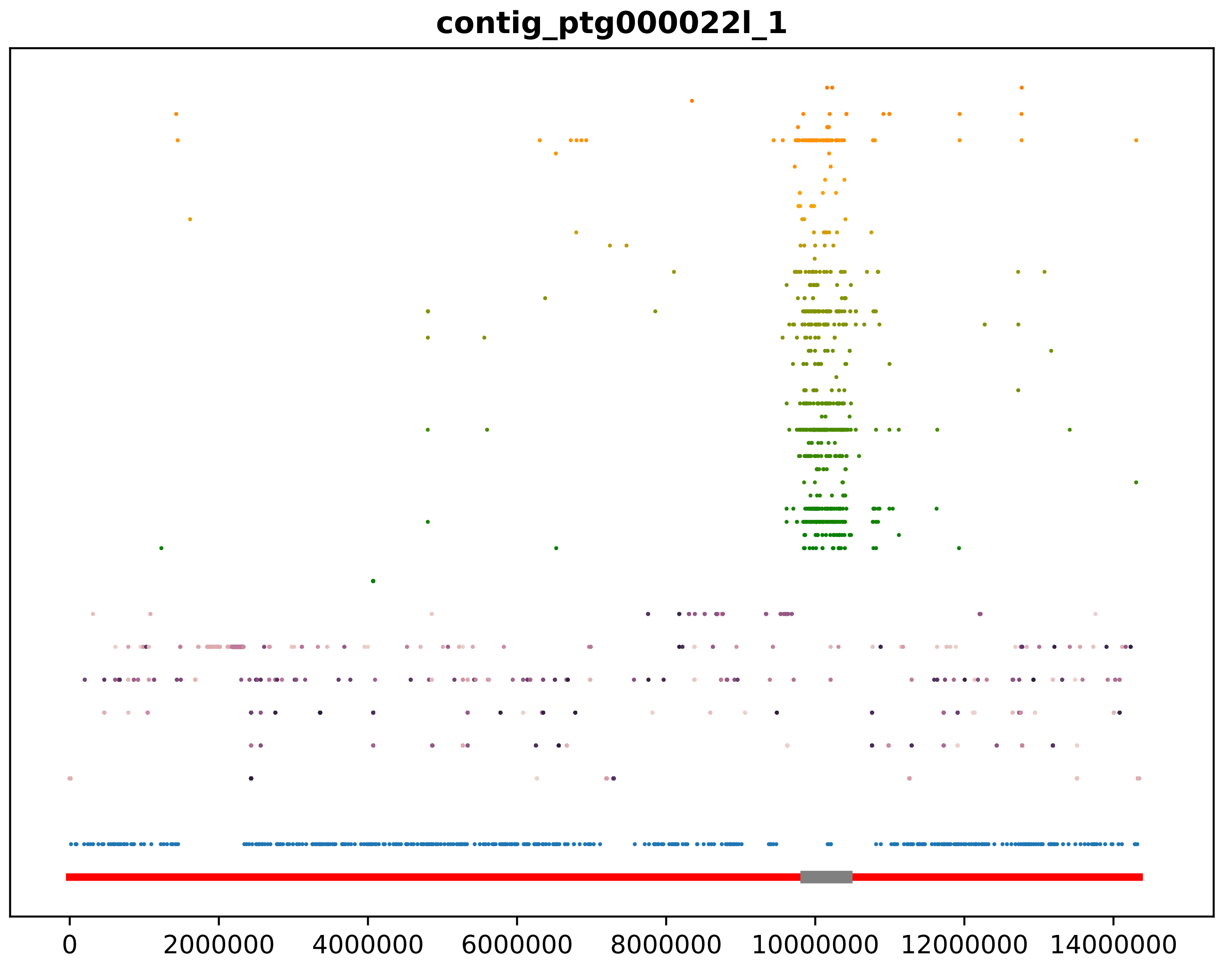

contig_ptg000022l_1
0
2000000
4000000
6000000
8000000
10000000
12000000
14000000

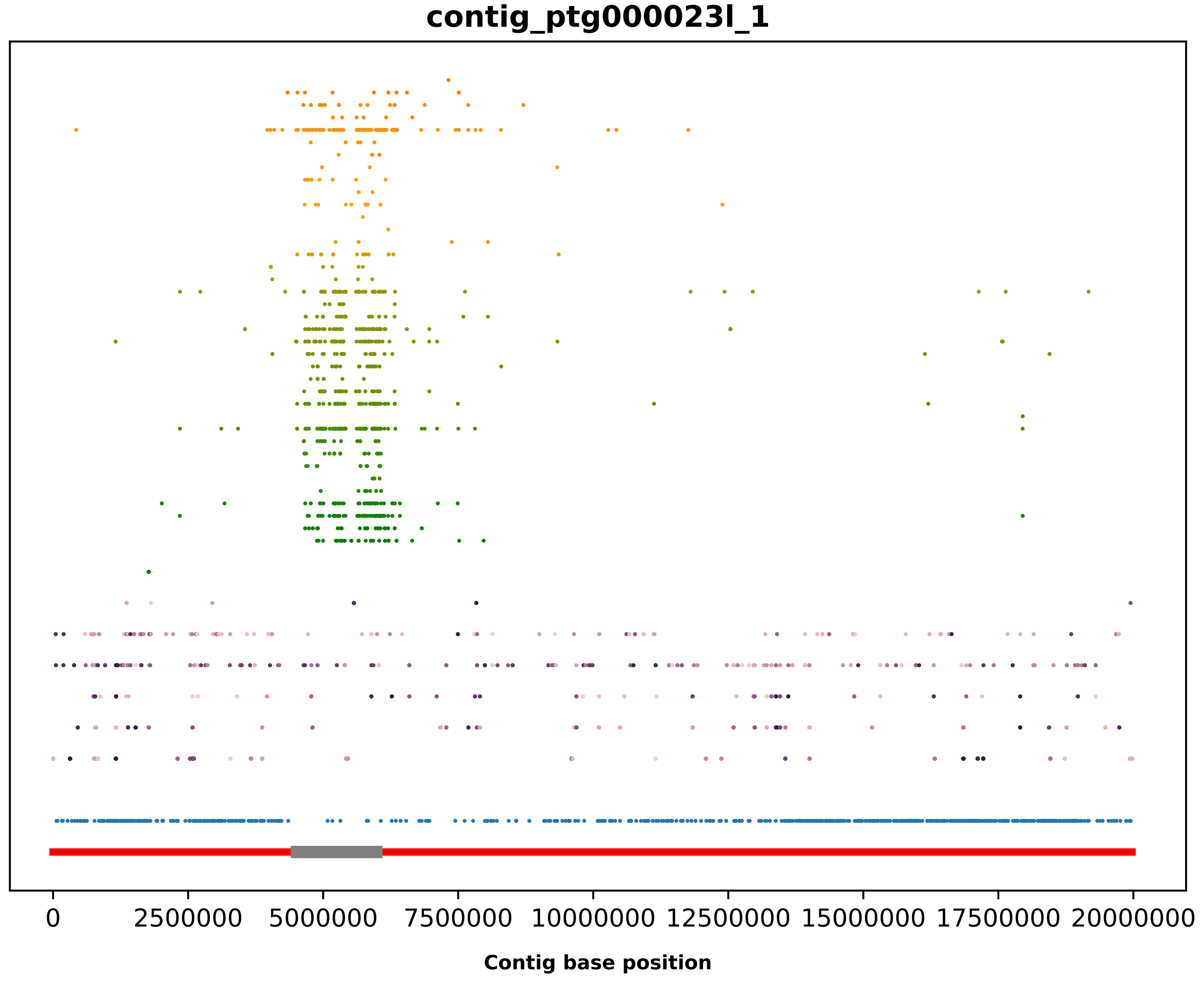

contig_ptg000023l_1
0
2500000
5000000
7500000
10000000
12500000
15000000
17500000
20000000
Contig base position

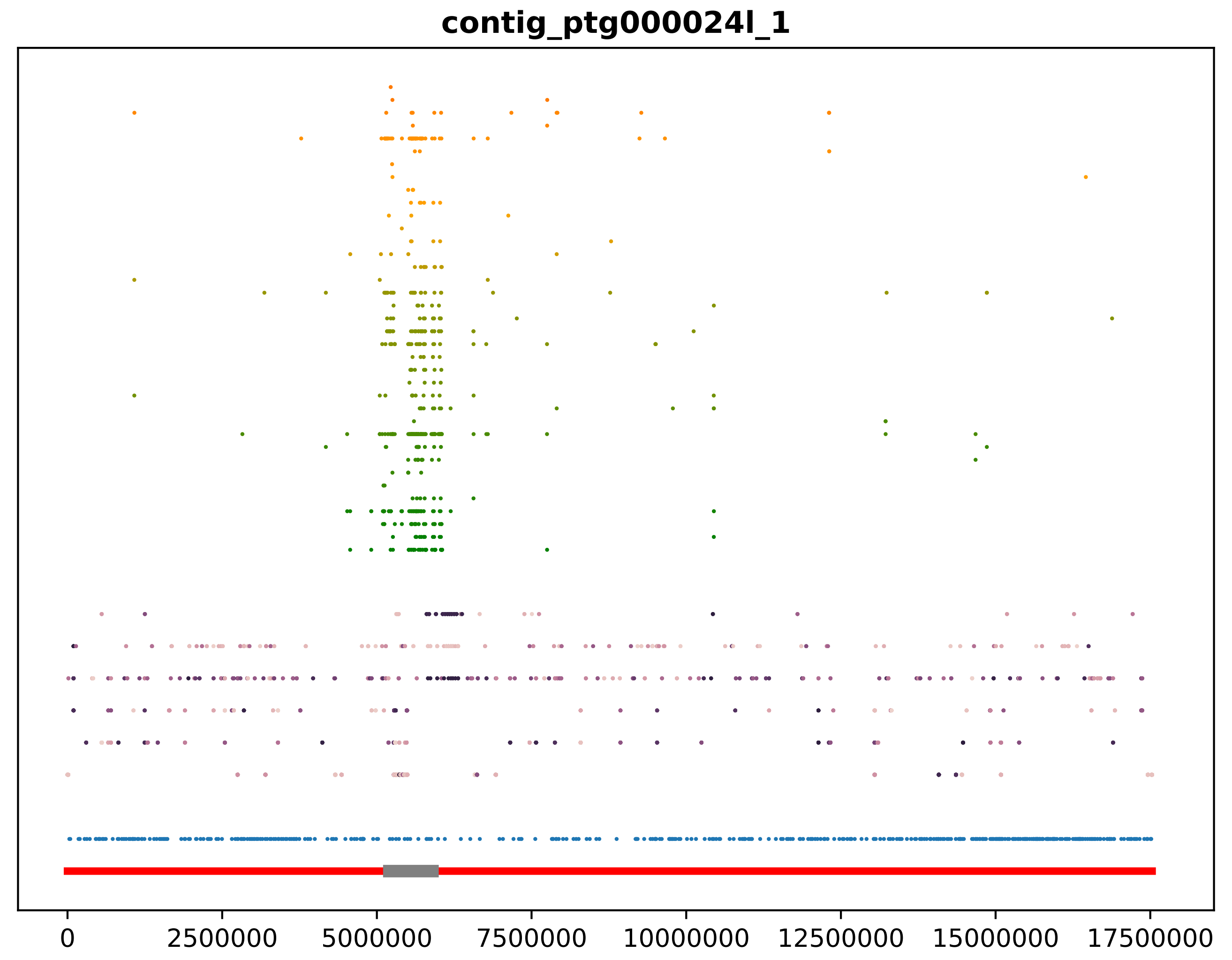

contig_ptg000024l_1
0
2500000
5000000
7500000
10000000
12500000
15000000
17500000

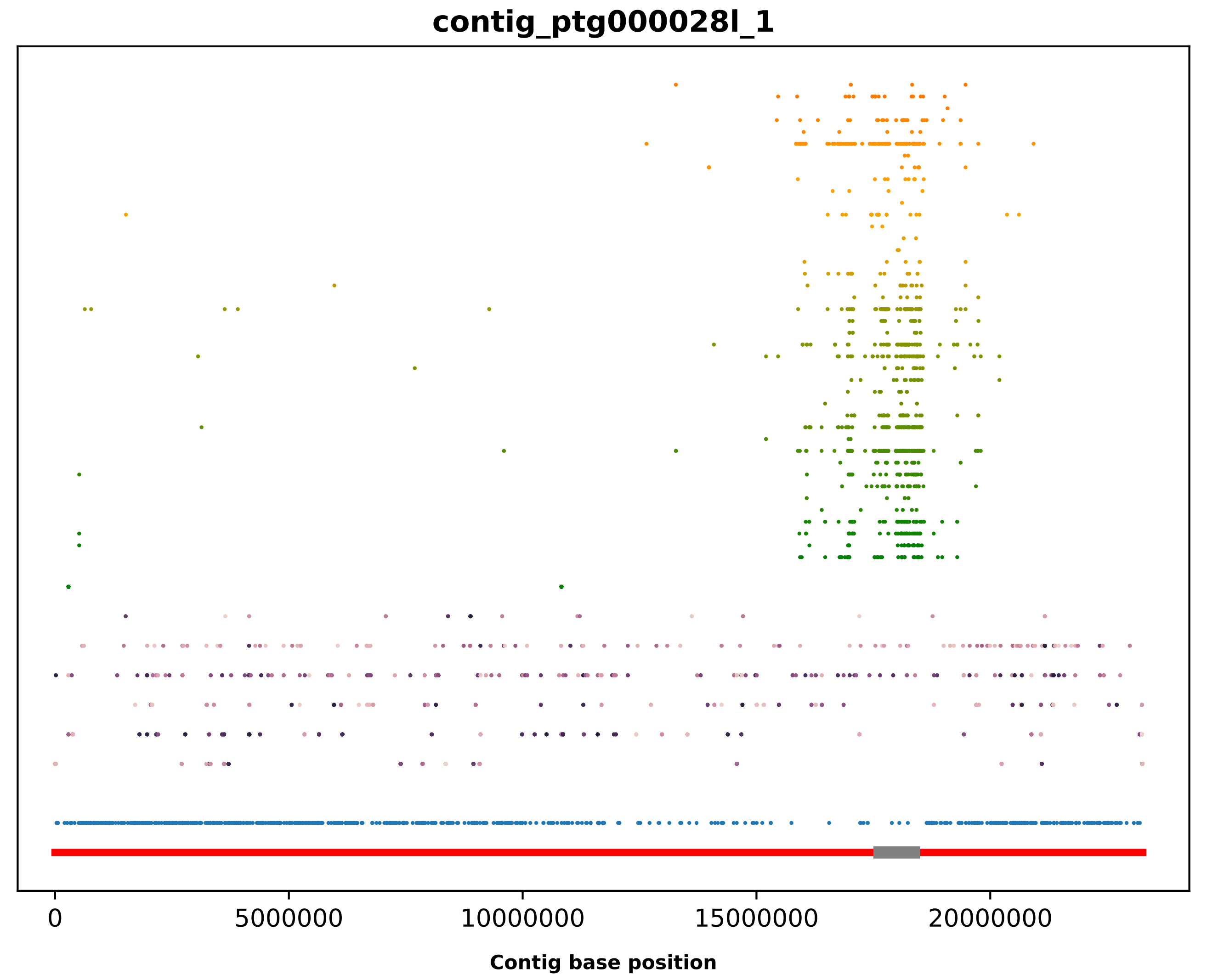

contig_ptg000028l_1
0
5000000
10000000
15000000
20000000
Contig base position

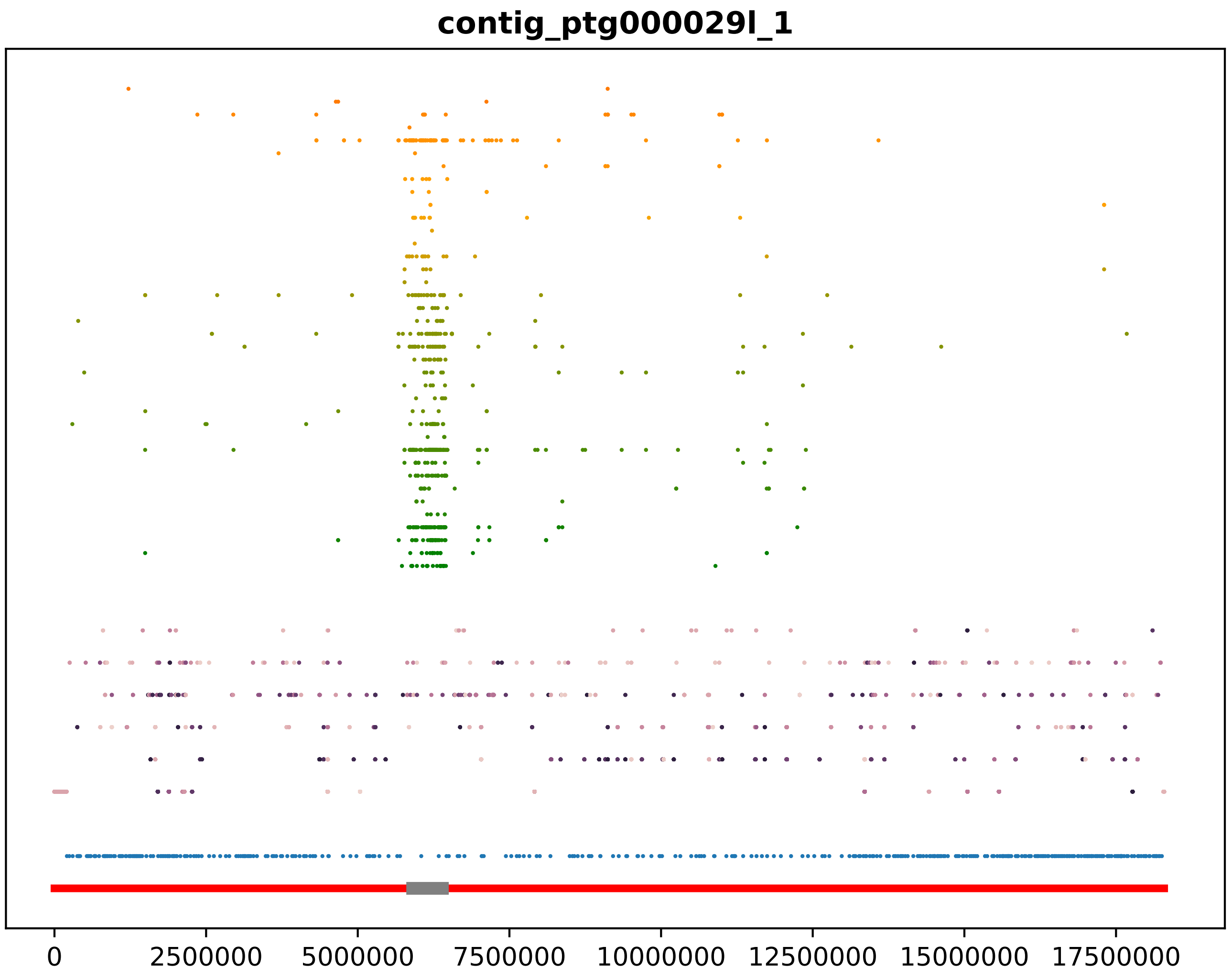
contig_ptg000029l_1
0
2500000
5000000
7500000
10000000
12500000
15000000
17500000

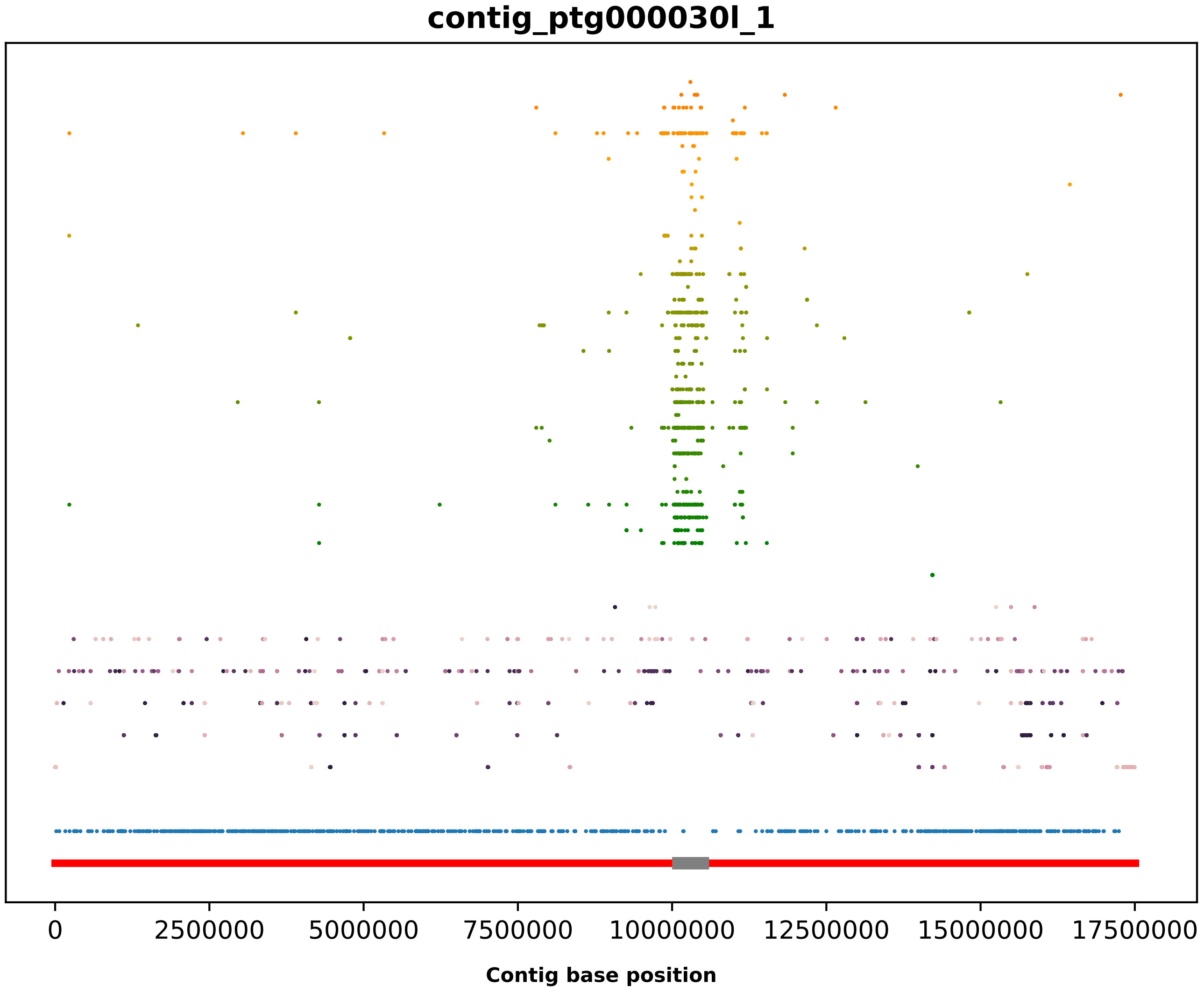
contig_ptg000030l_1
0
2500000
5000000
7500000
10000000
12500000
15000000
17500000
Contig base position

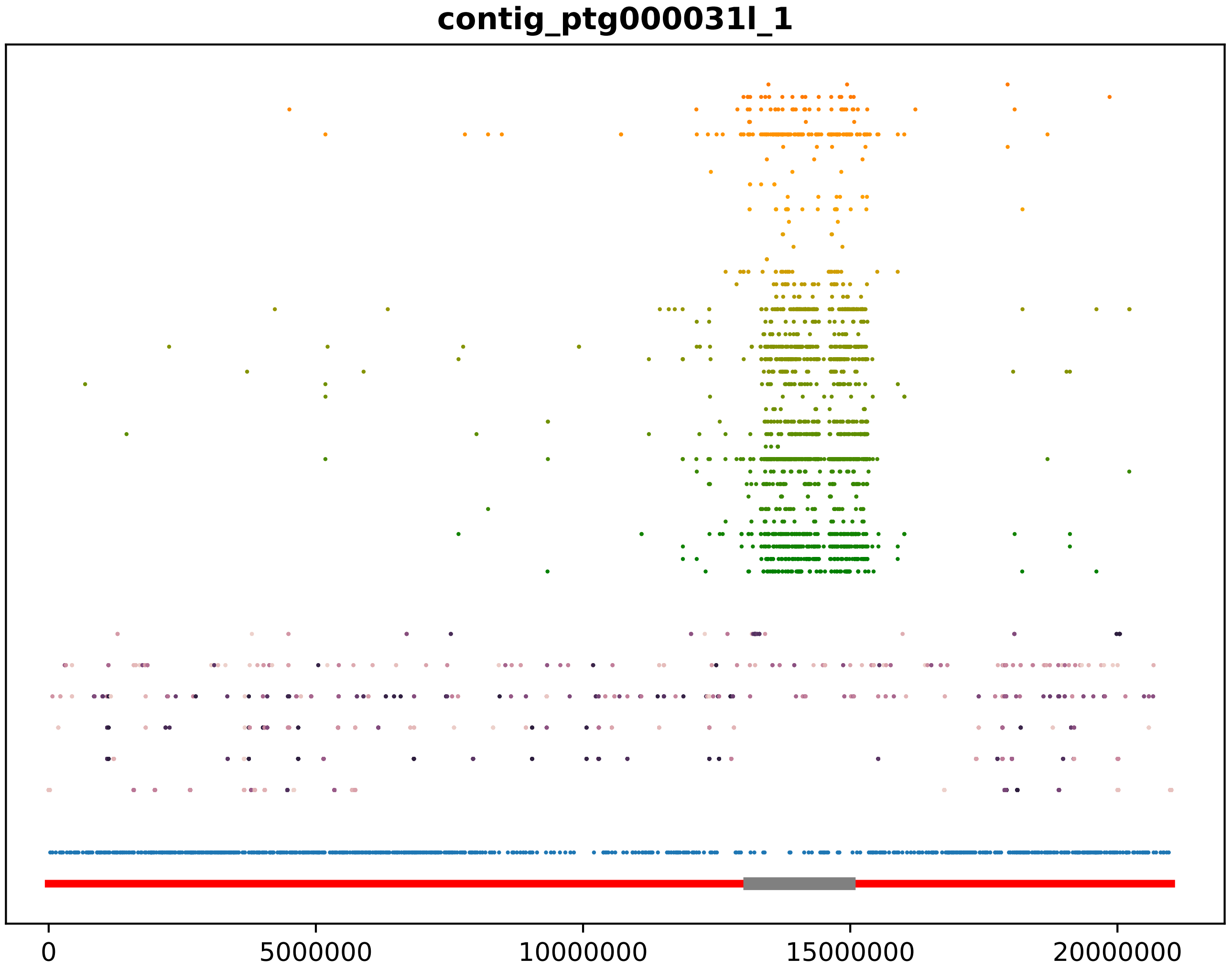
contig_ptg000031l_1
0
5000000
10000000
15000000
20000000

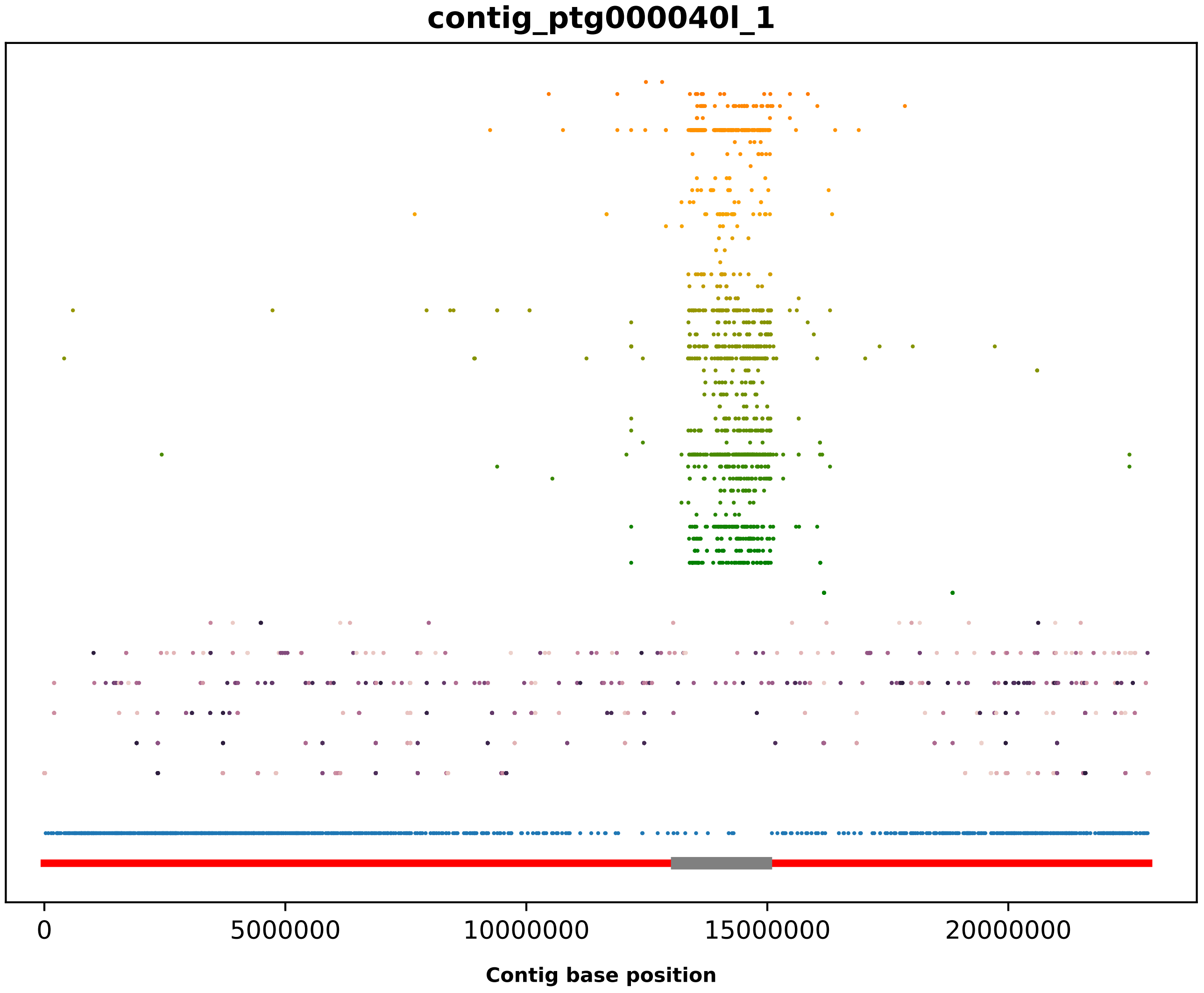
contig_ptg000040l_1
0
5000000
10000000
15000000
20000000
Contig base position

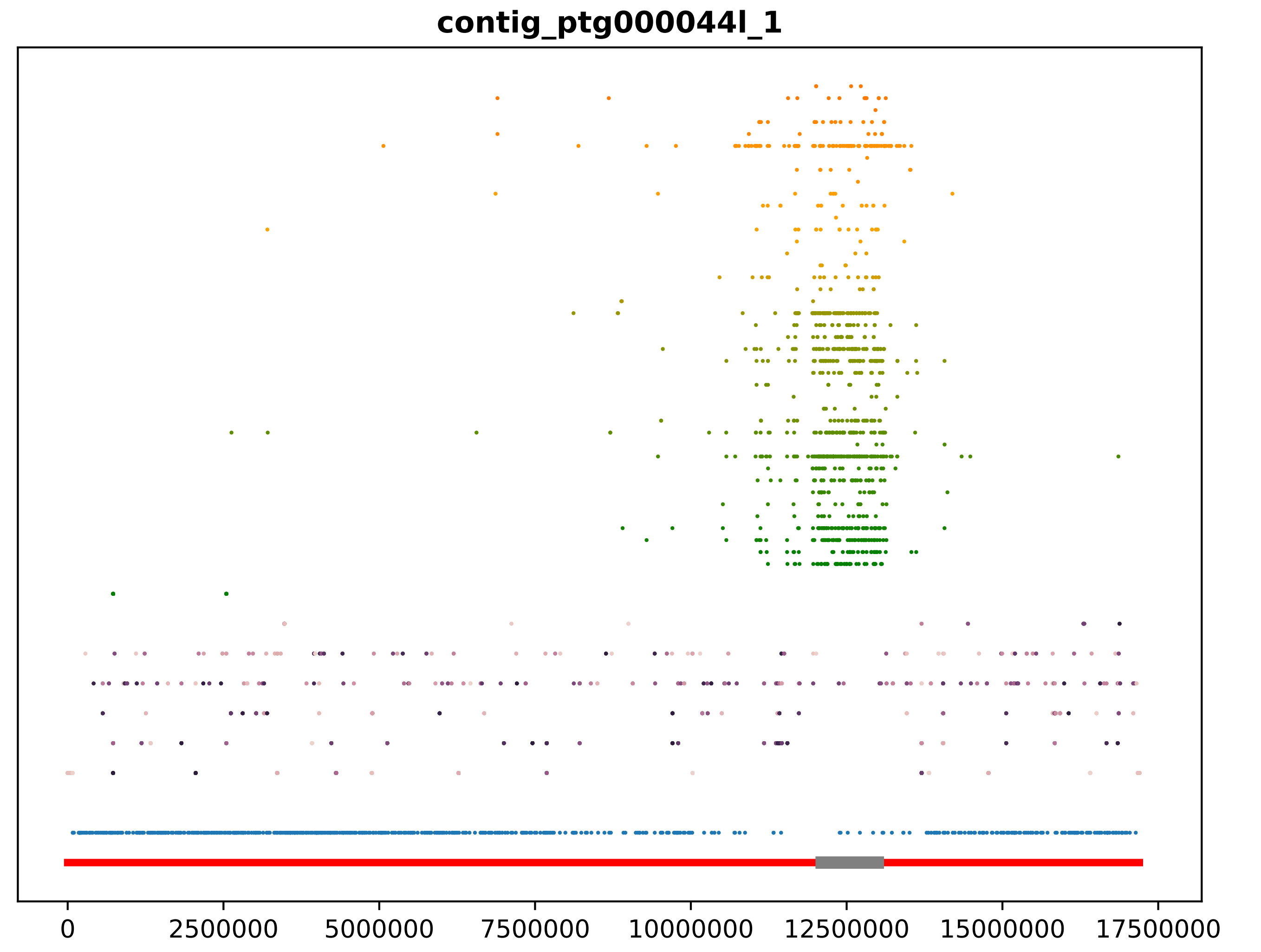
contig_ptg000044l_1
0
2500000
5000000
7500000
10000000
12500000
15000000
17500000

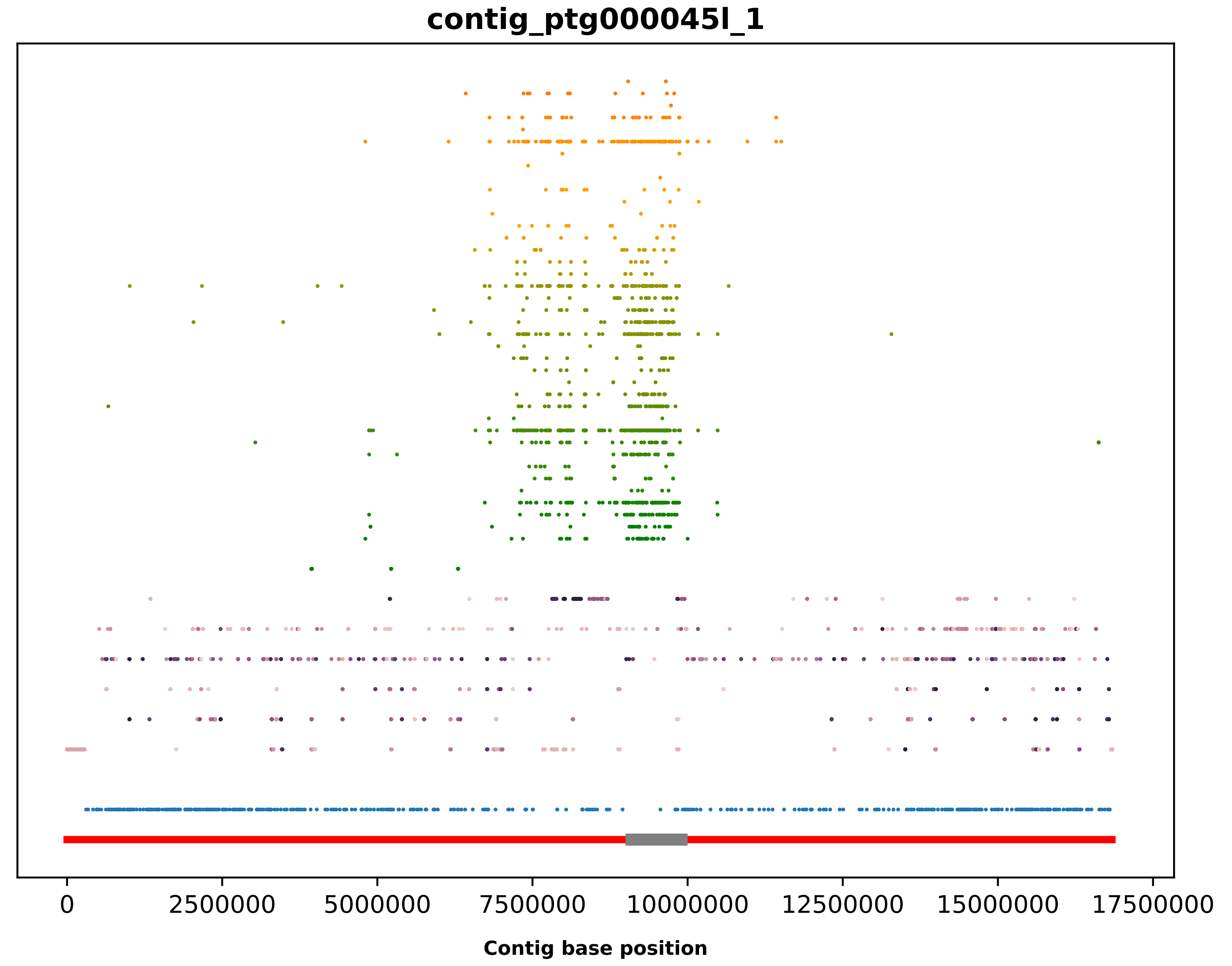
contig_ptg000045l_1
0
2500000
5000000
7500000
10000000
12500000
15000000
17500000
Contig base position

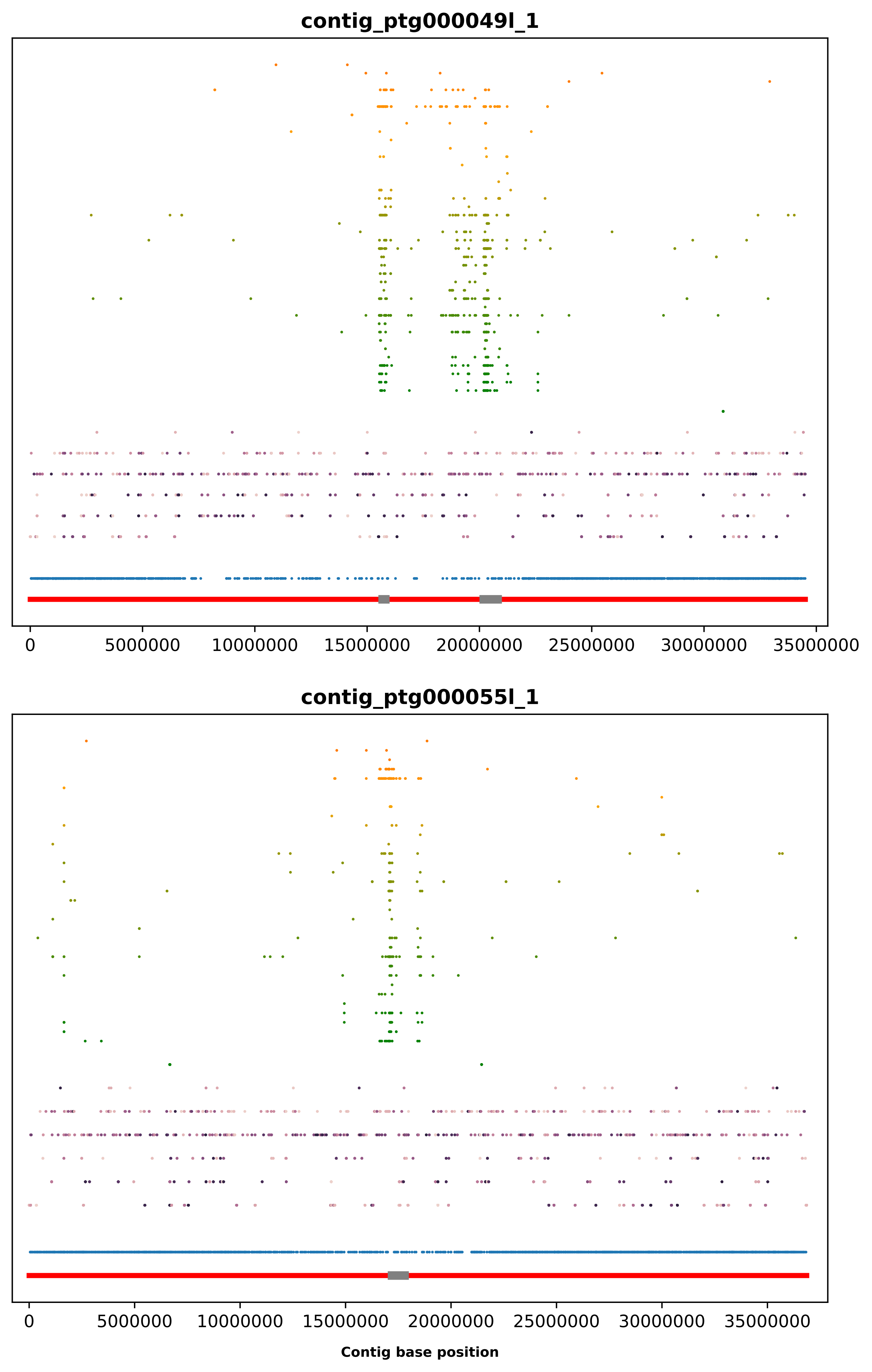
contig_ptg000049l_1
0
5000000
10000000
15000000
20000000
25000000
30000000
35000000
contig_ptg000055l_1
0
5000000
10000000
15000000
20000000
25000000
30000000
35000000
Contig base position

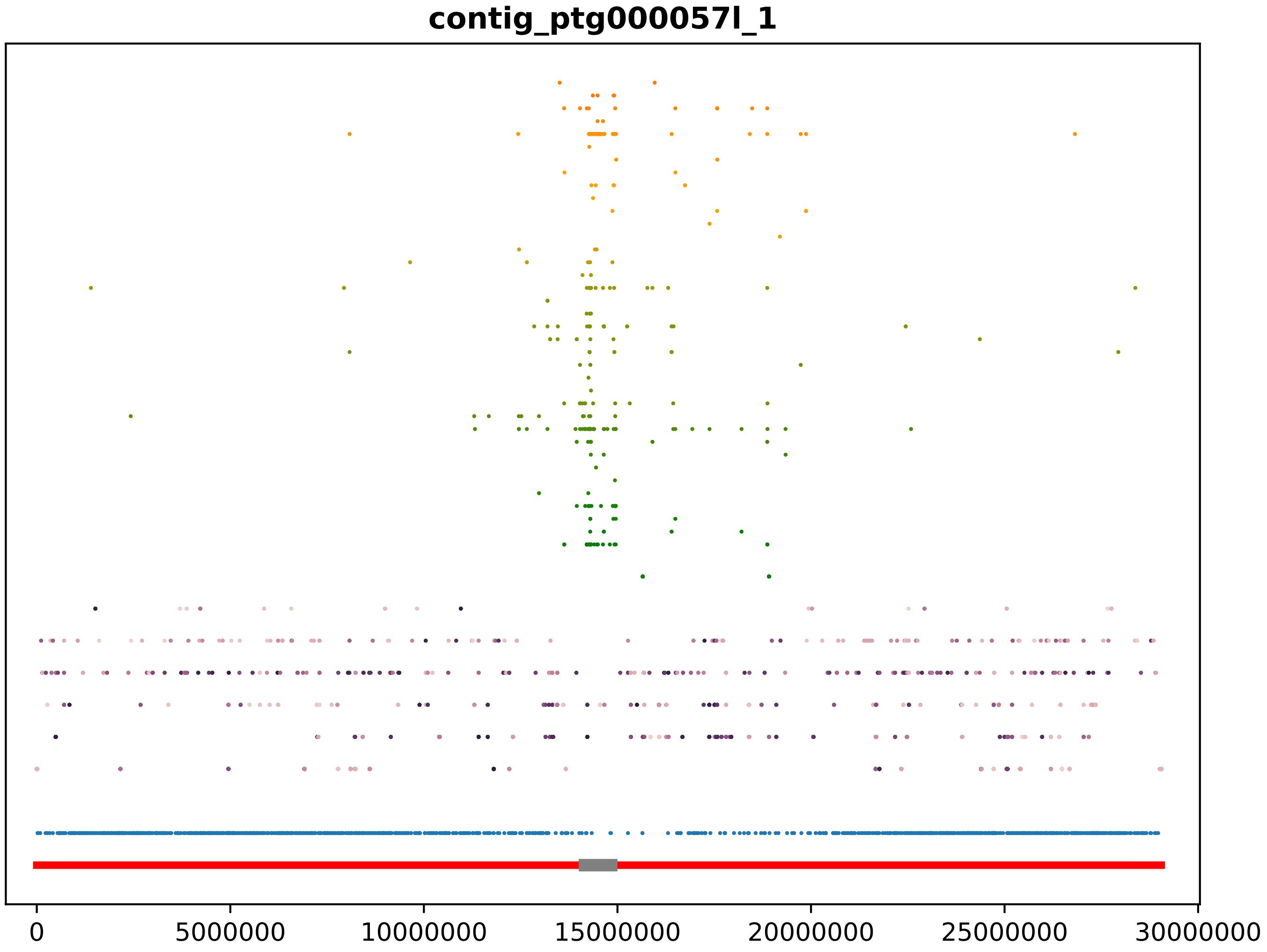
contig_ptg000057l_1
0
5000000
10000000
15000000
20000000
25000000
30000000

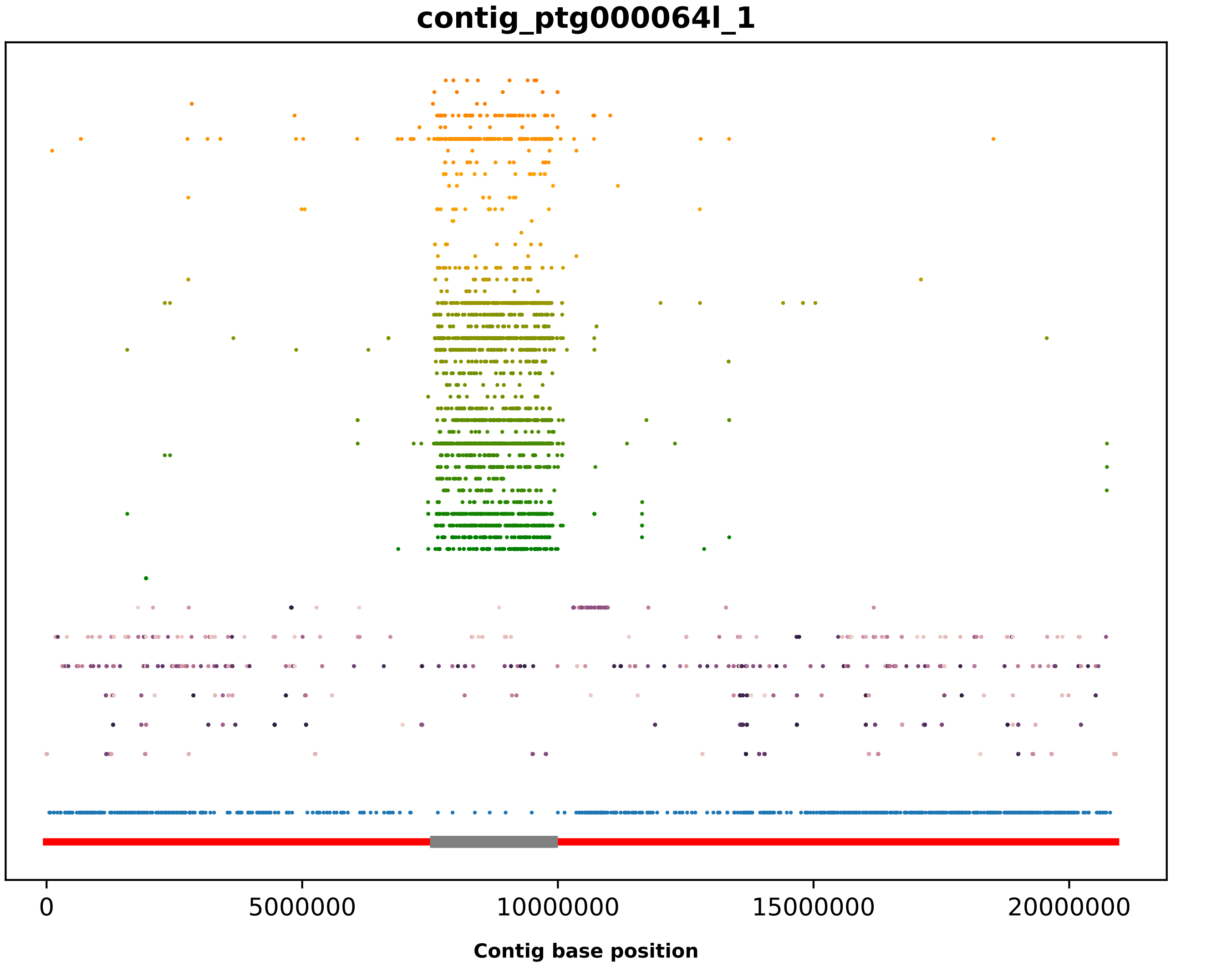
contig_ptg000064l_1
0
5000000
10000000
15000000
20000000
Contig base position

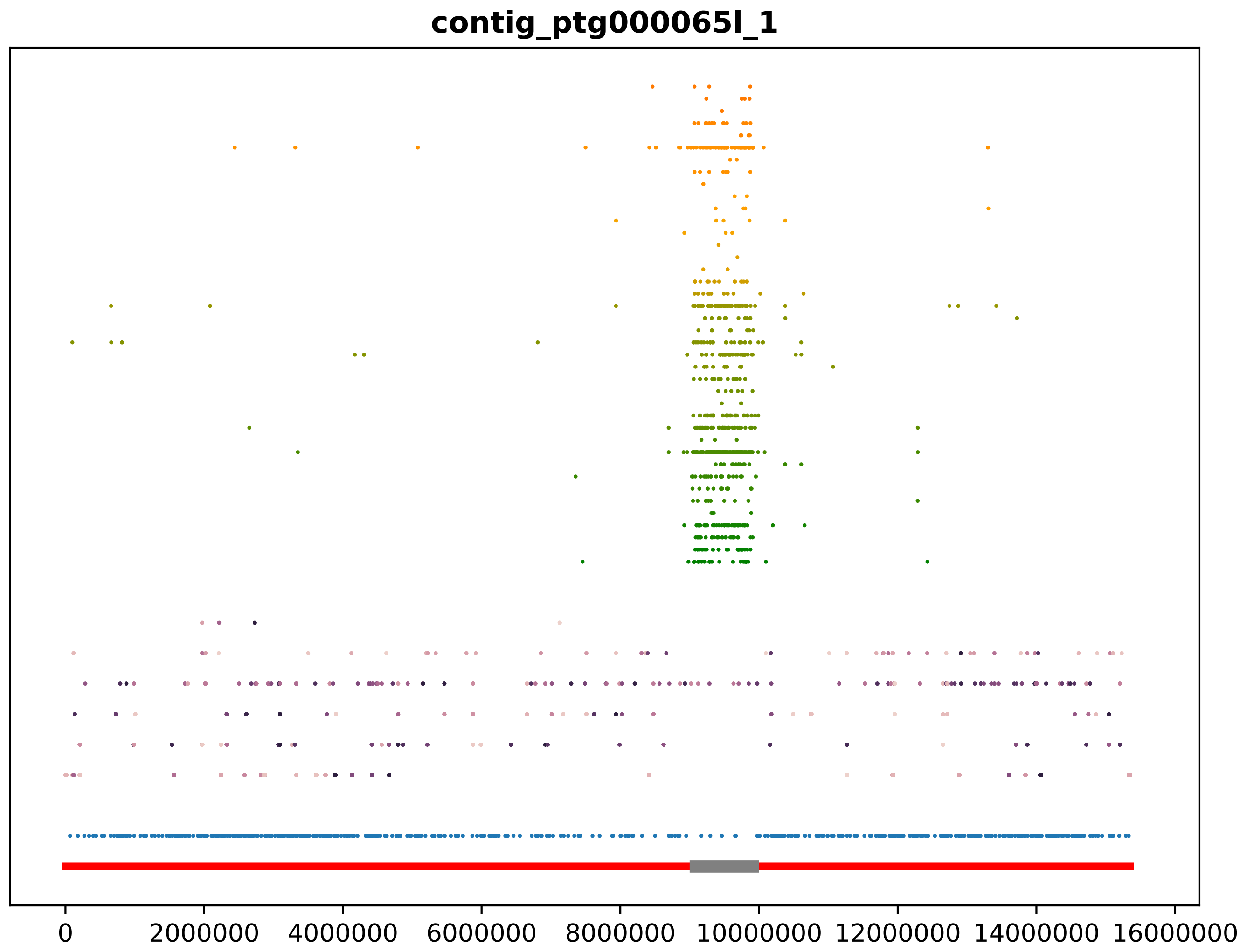
contig_ptg000065l_1
0
2000000
4000000
6000000
8000000
10000000
12000000
14000000
16000000

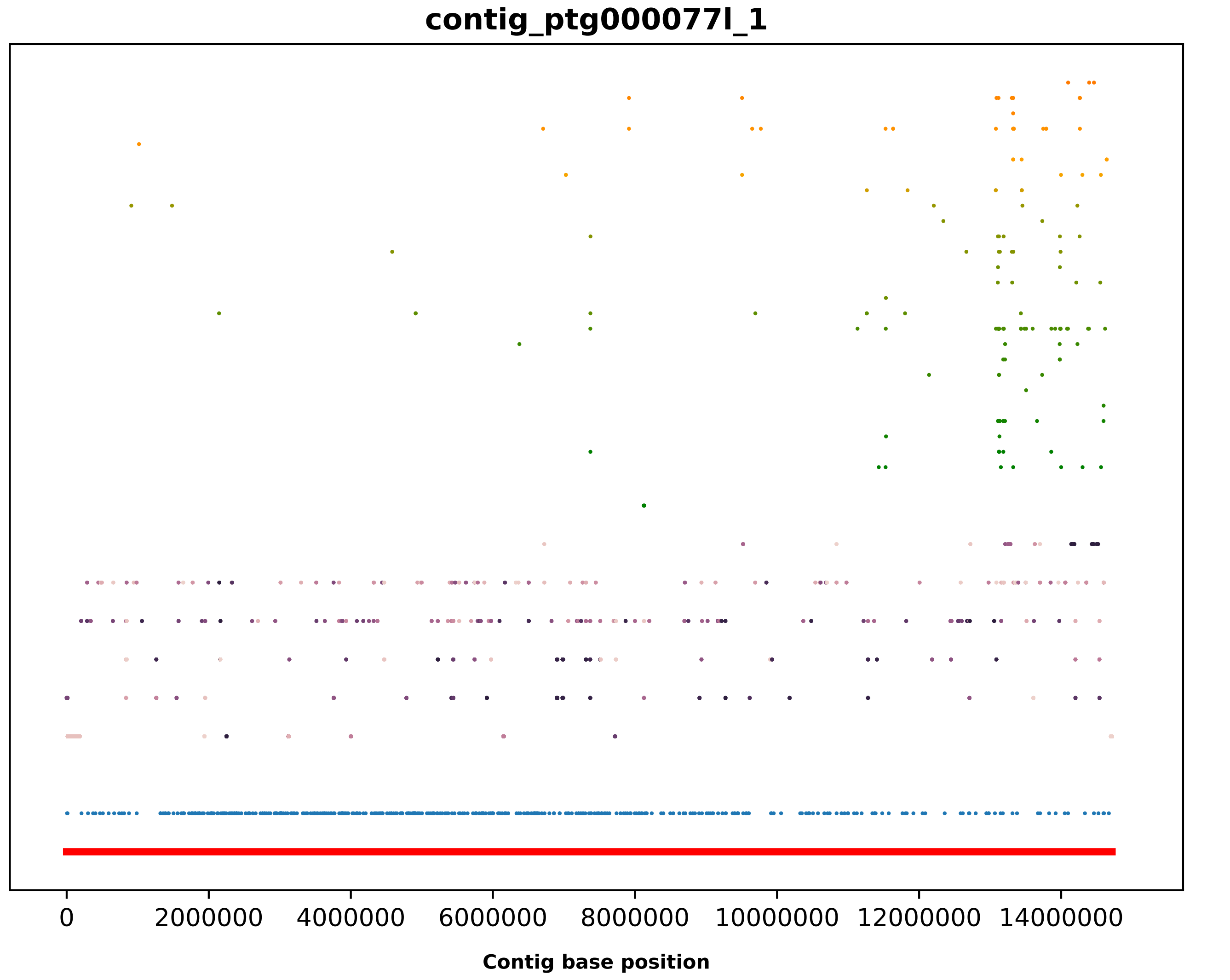
contig_ptg000077l_1
0
2000000
4000000
6000000
8000000
10000000
12000000
14000000
Contig base position

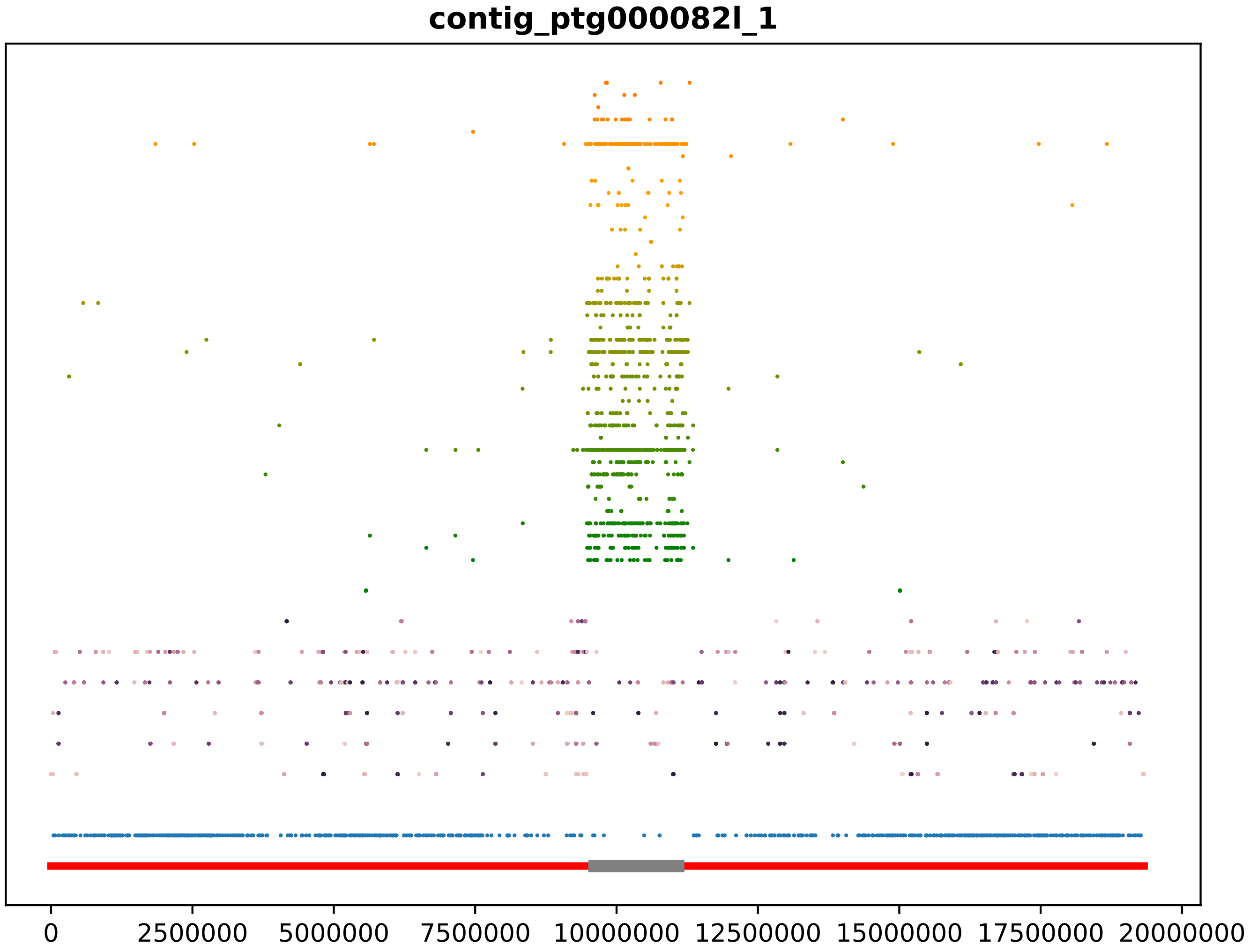
contig_ptg000082l_1
0
2500000
5000000
7500000
10000000
12500000
15000000
17500000
20000000

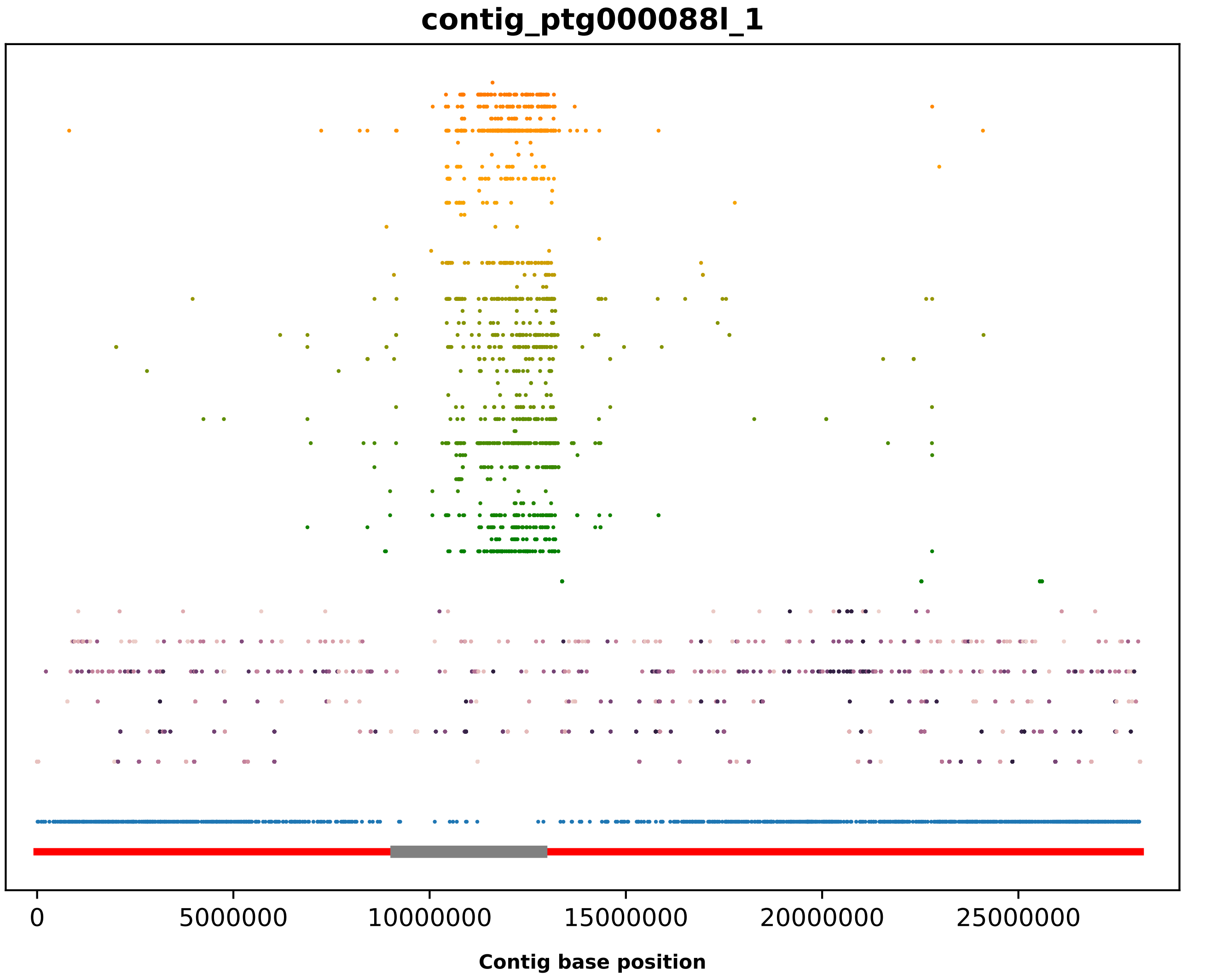
contig_ptg000088l_1
0
5000000
10000000
15000000
20000000
25000000
Contig base position

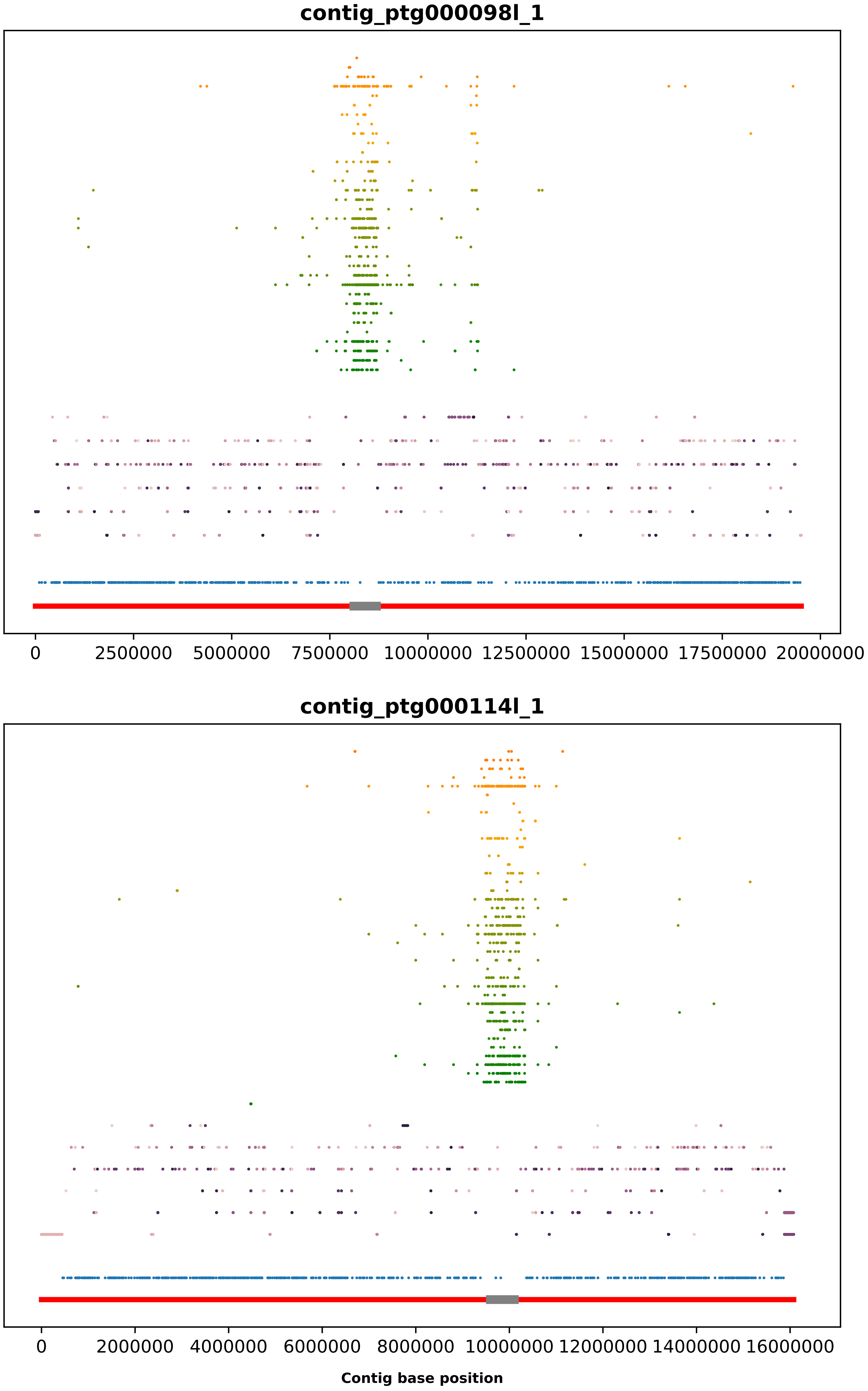

contig_ptg000098l_1
0
2500000
5000000
7500000
10000000
12500000
15000000
17500000
20000000
contig_ptg000114l_1
0
2000000
4000000
6000000
8000000
10000000
12000000
14000000
16000000
Contig base position

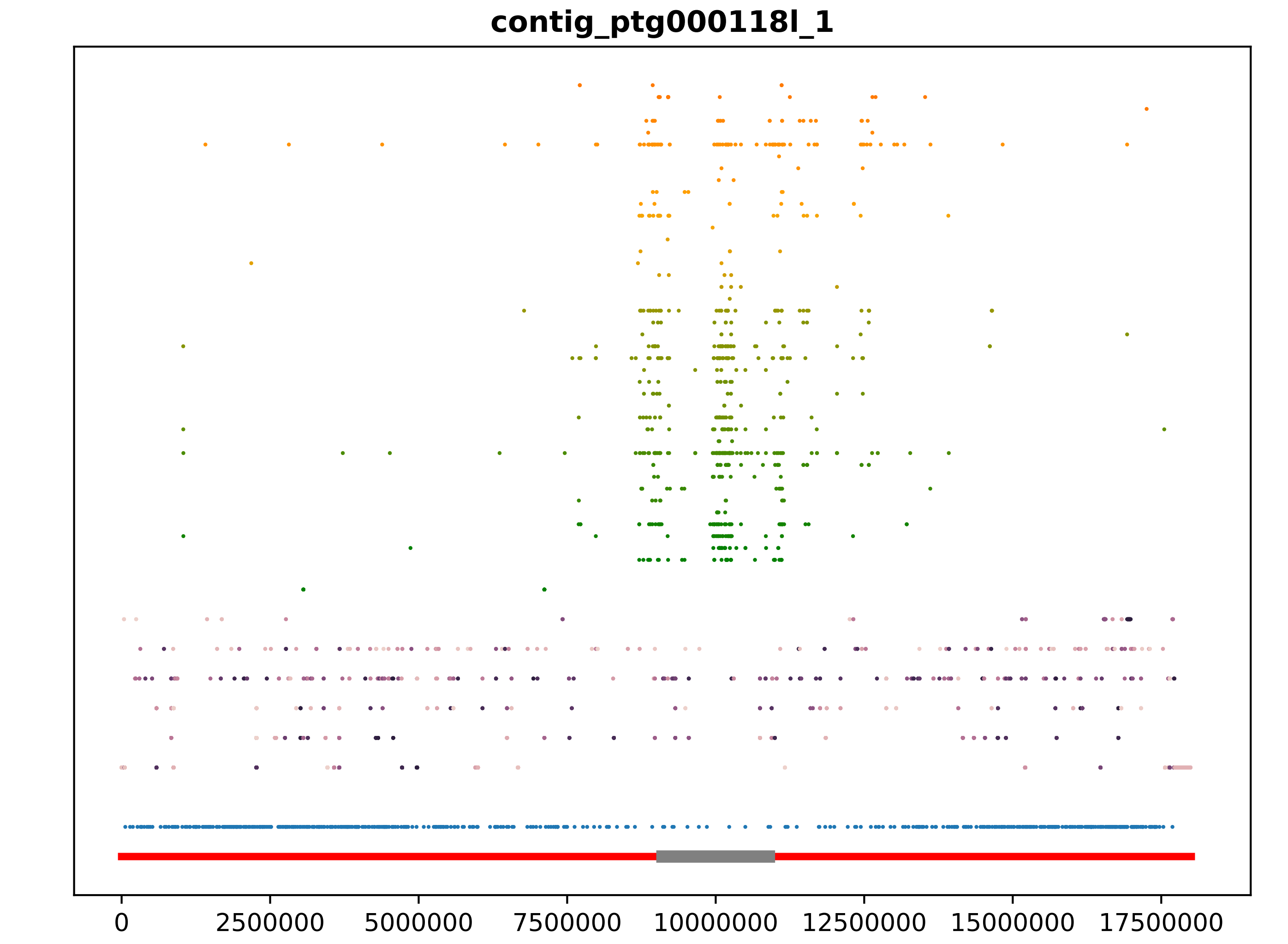
contig_ptg000118l_1
0
2500000
5000000
7500000
10000000
12500000
15000000
17500000

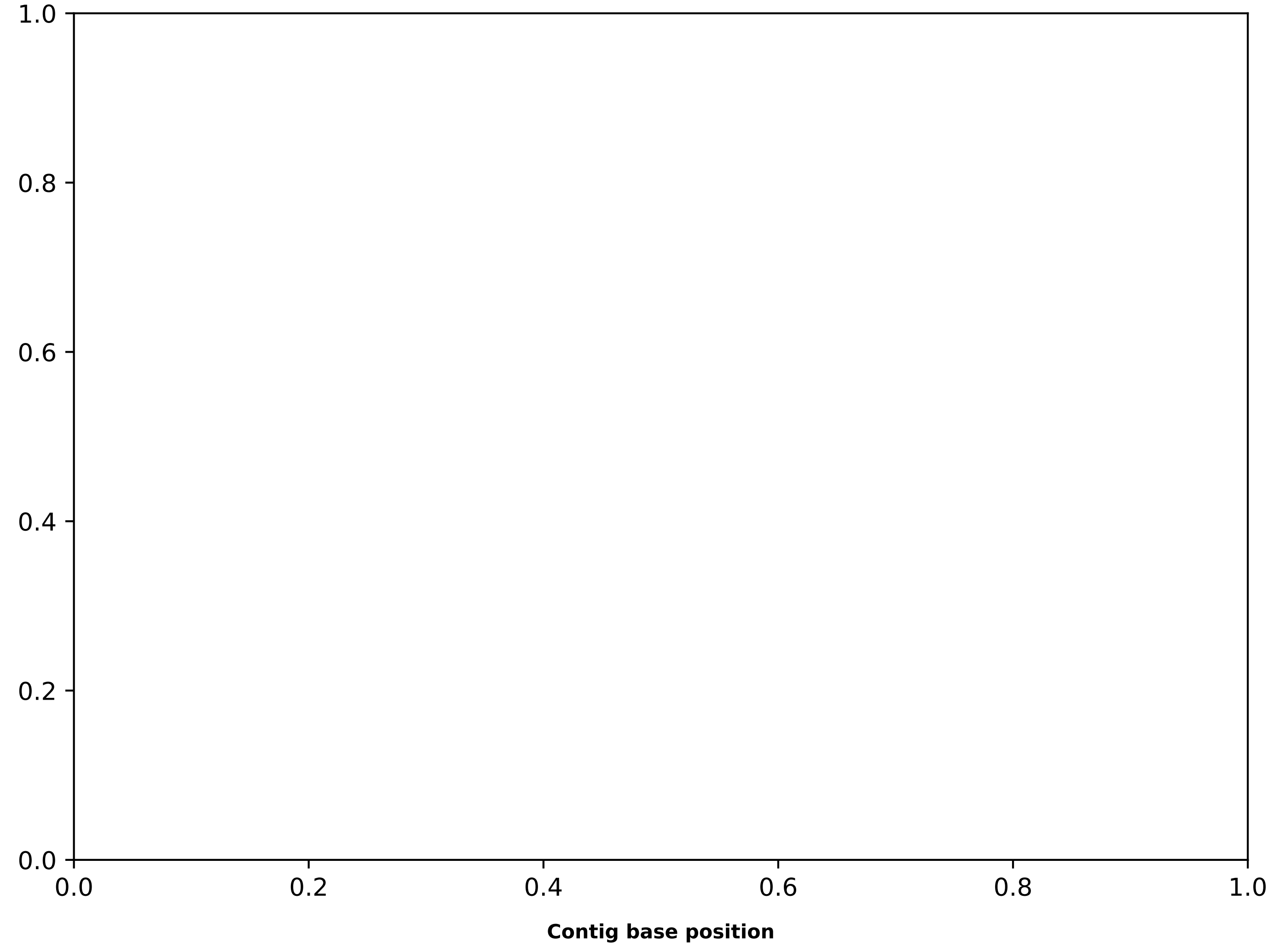
1.0
0.8
0.6
0.4
0.2
0.0
0.0
0.2
0.4
0.6
0.8
1.0
Contig base position

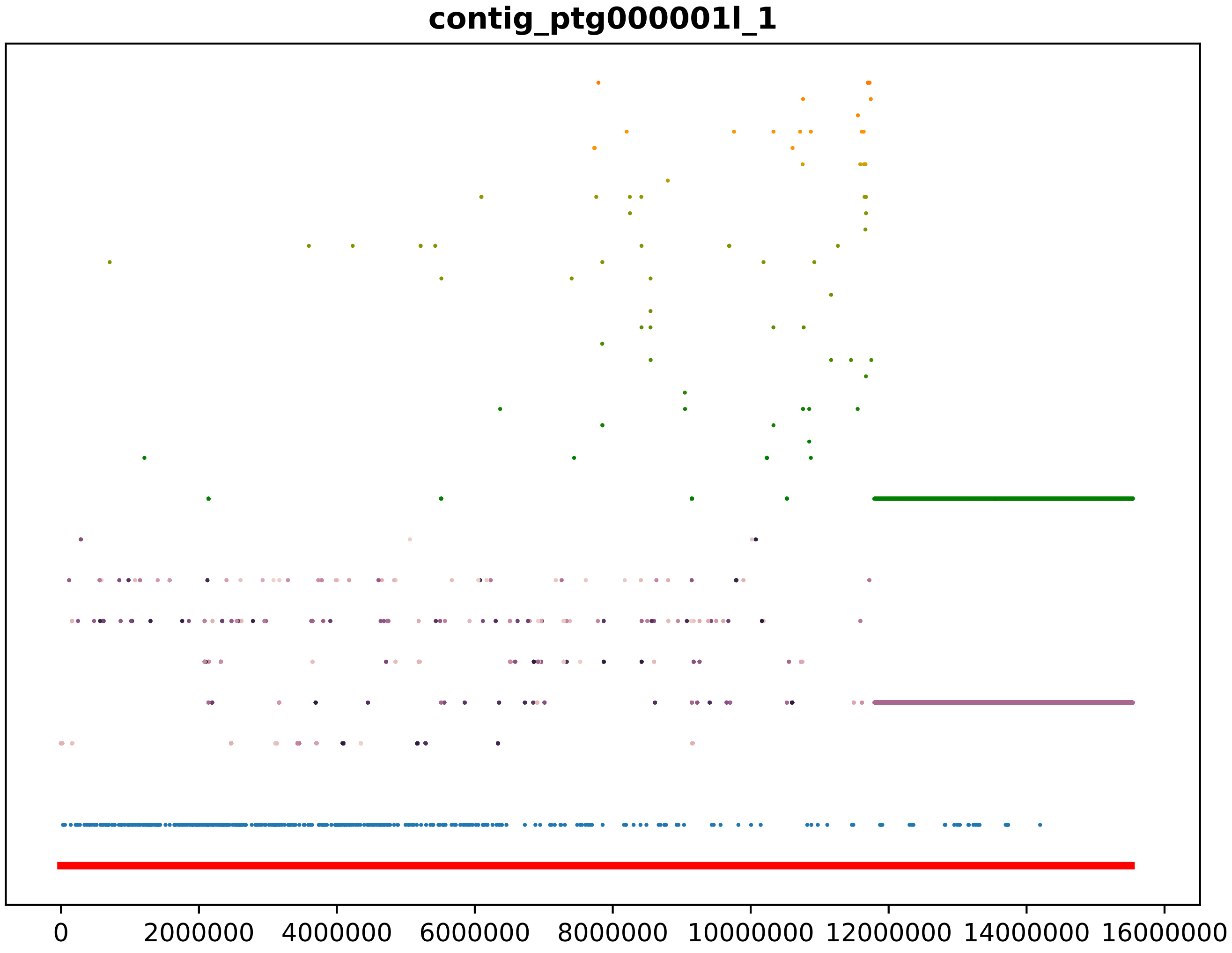

contig_ptg000001l_1
0
2000000
4000000
6000000
8000000
10000000
12000000
14000000
16000000

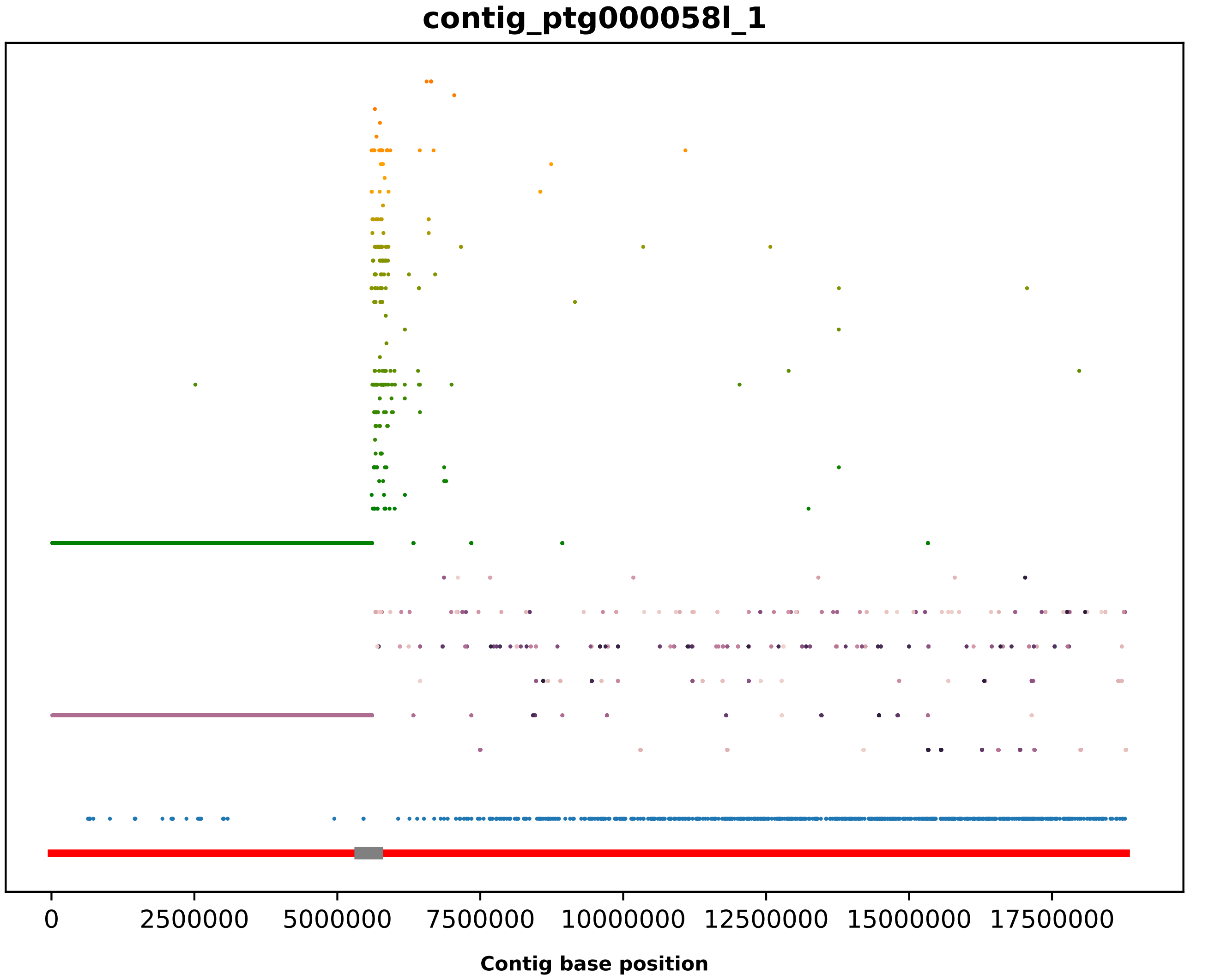

contig_ptg000058l_1
0
2500000
5000000
7500000
10000000
12500000
15000000
17500000
Contig base position

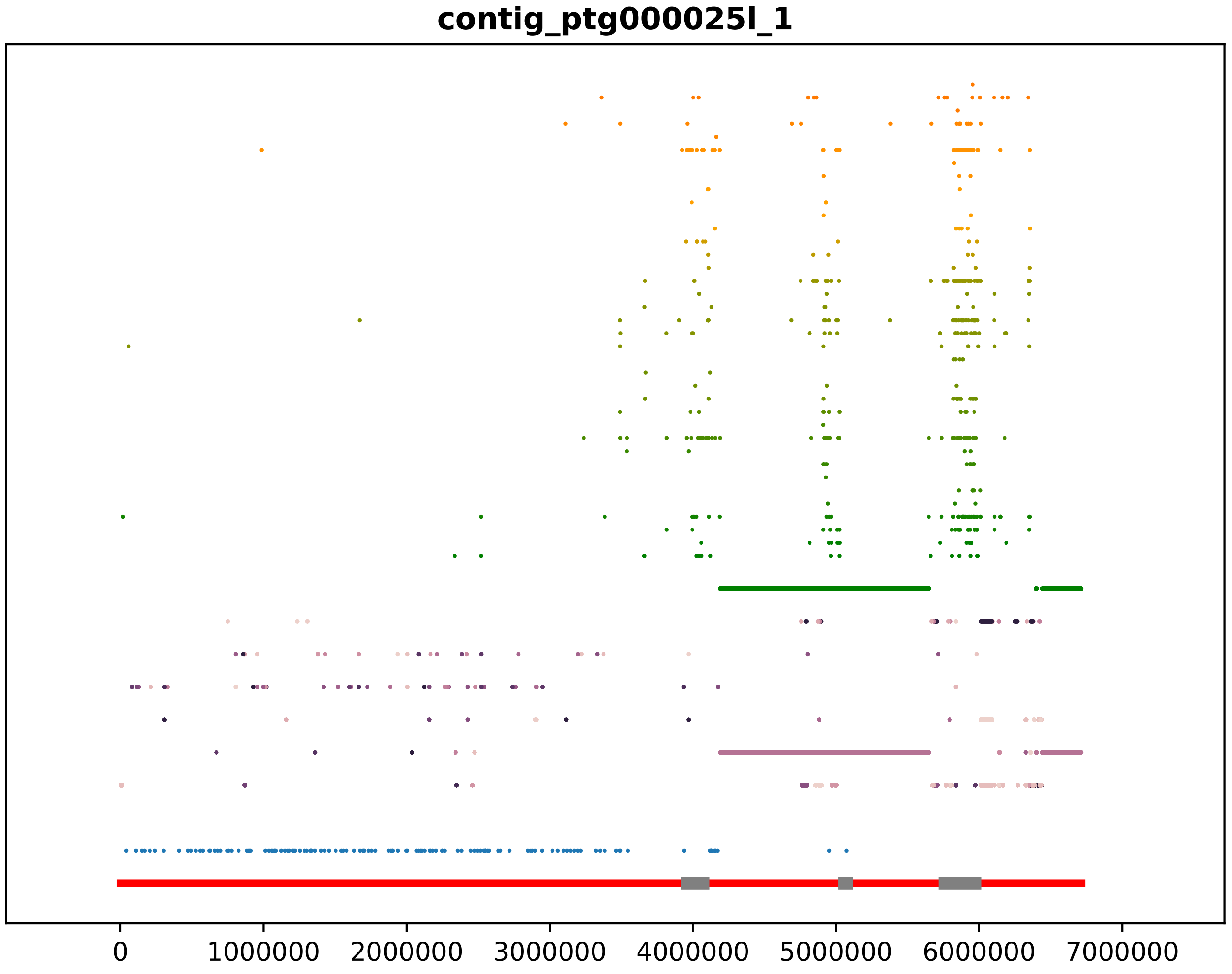
contig_ptg000025l_1
0
1000000
2000000
3000000
4000000
5000000
6000000
7000000

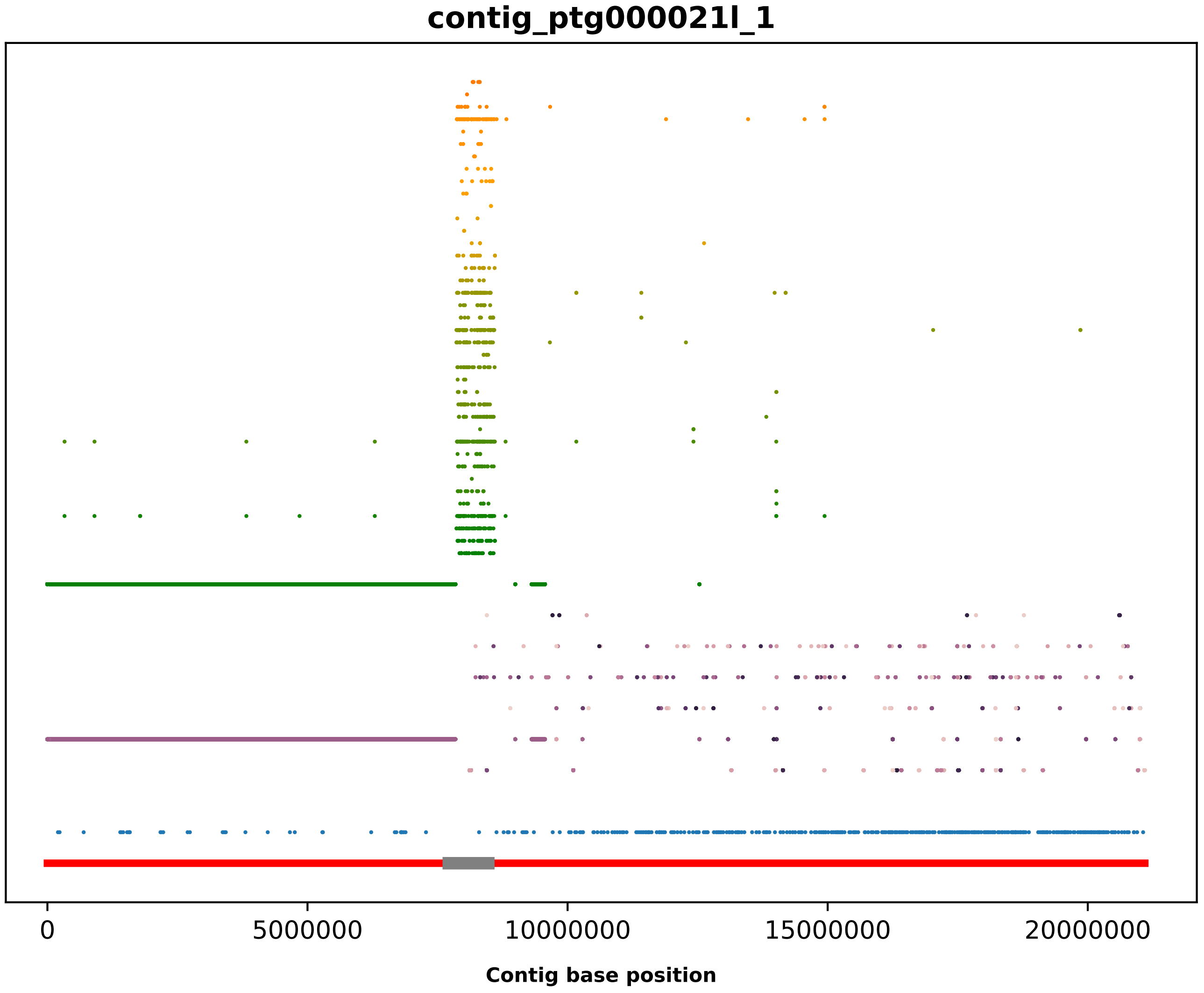
contig_ptg000021l_1
0
5000000
10000000
15000000
20000000
Contig base position

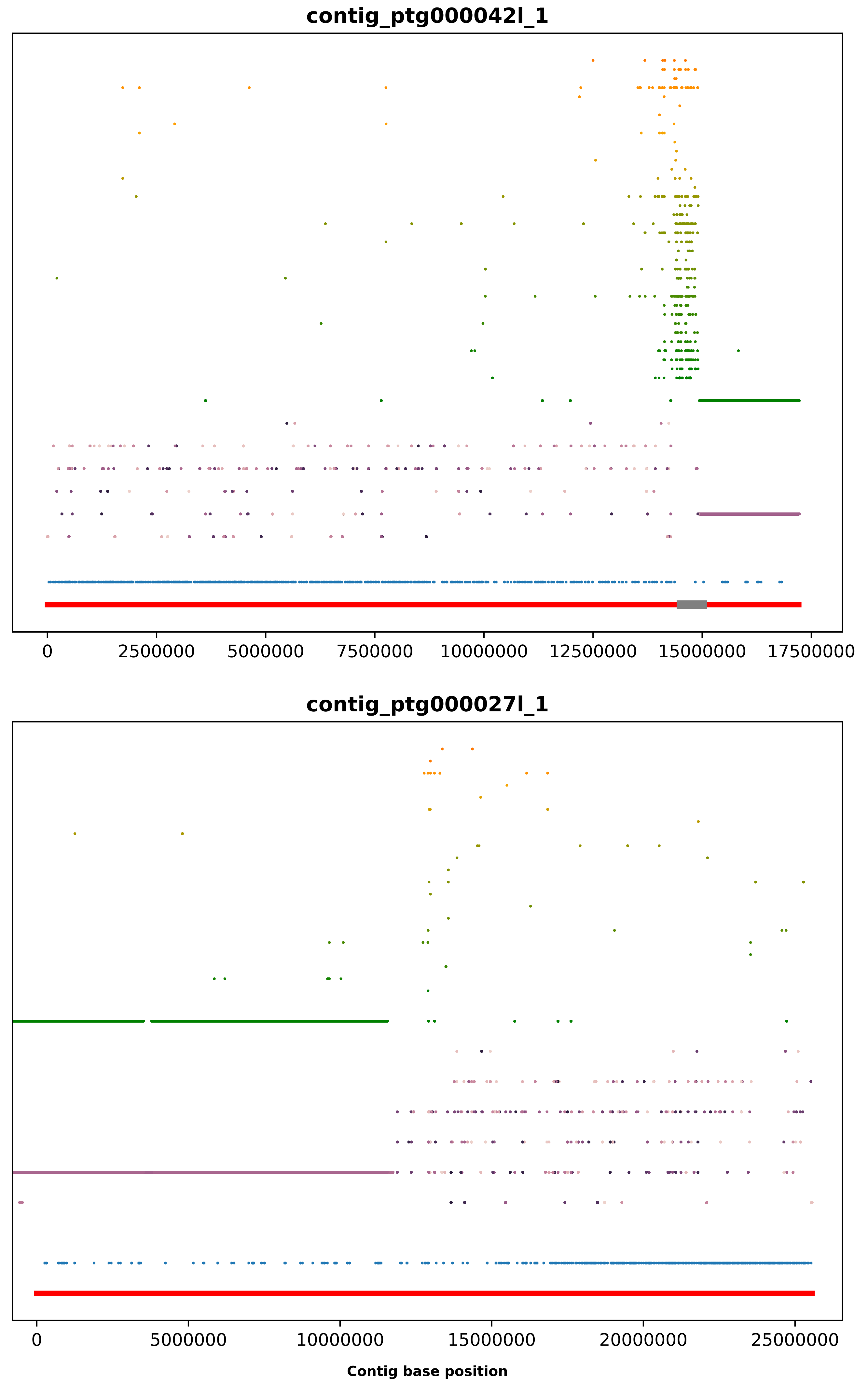

contig_ptg000042l_1
0
2500000
5000000
7500000
10000000
12500000
15000000
17500000
contig_ptg000027l_1
0
5000000
10000000
15000000
20000000
25000000
Contig base position

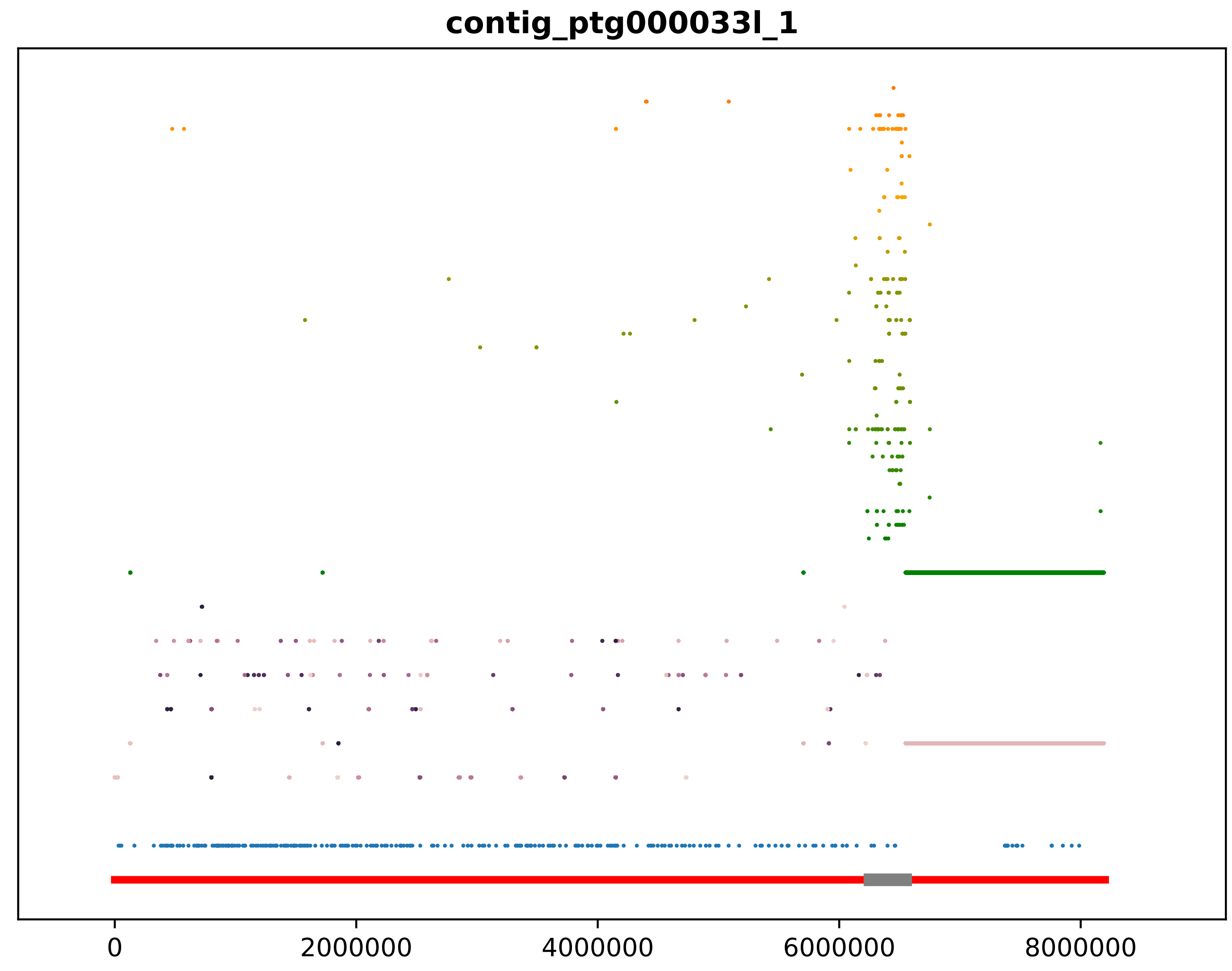
contig_ptg000033l_1
0
2000000
4000000
6000000
8000000

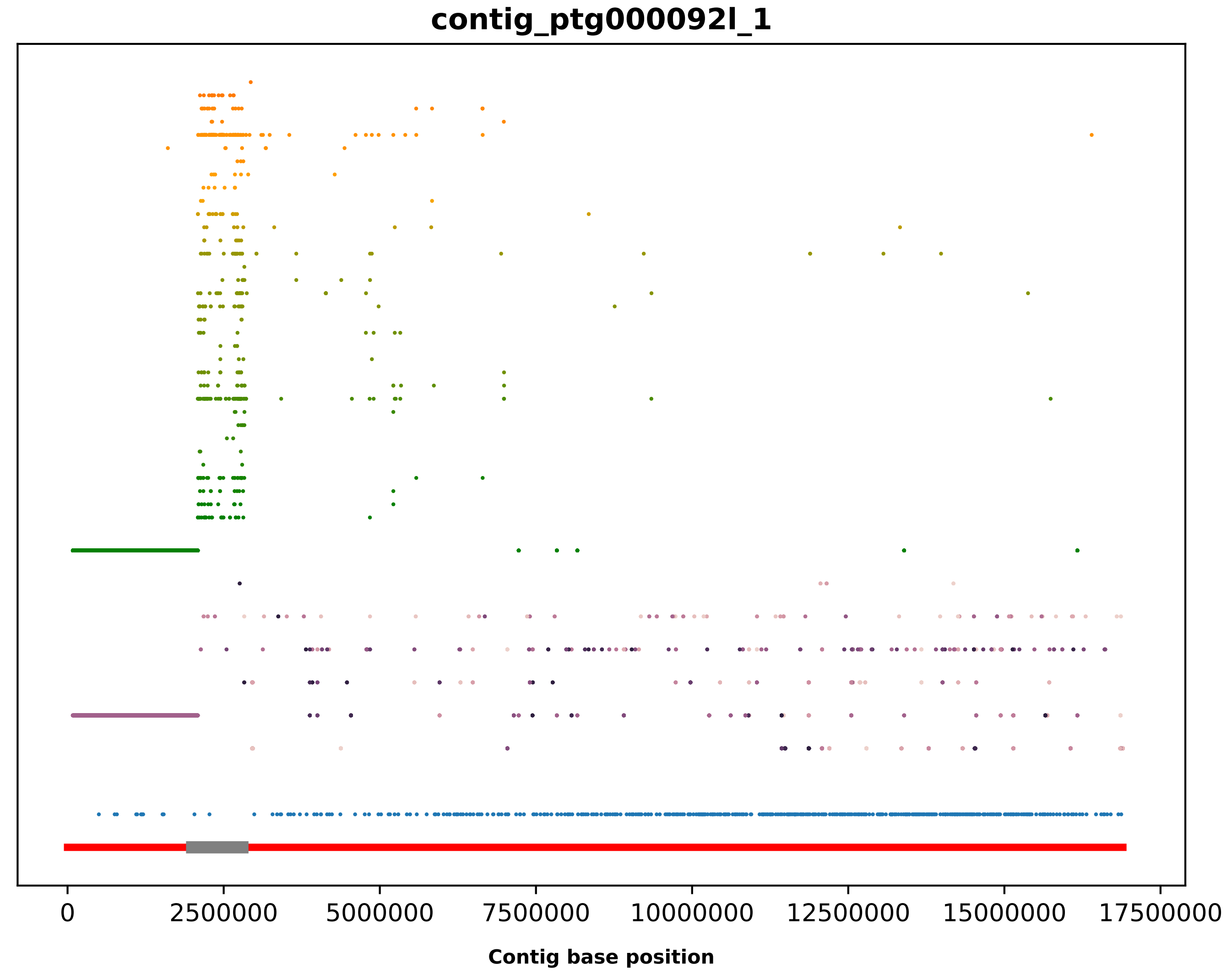
contig_ptg000092l_1
0
2500000
5000000
7500000
10000000
12500000
15000000
17500000
Contig base position

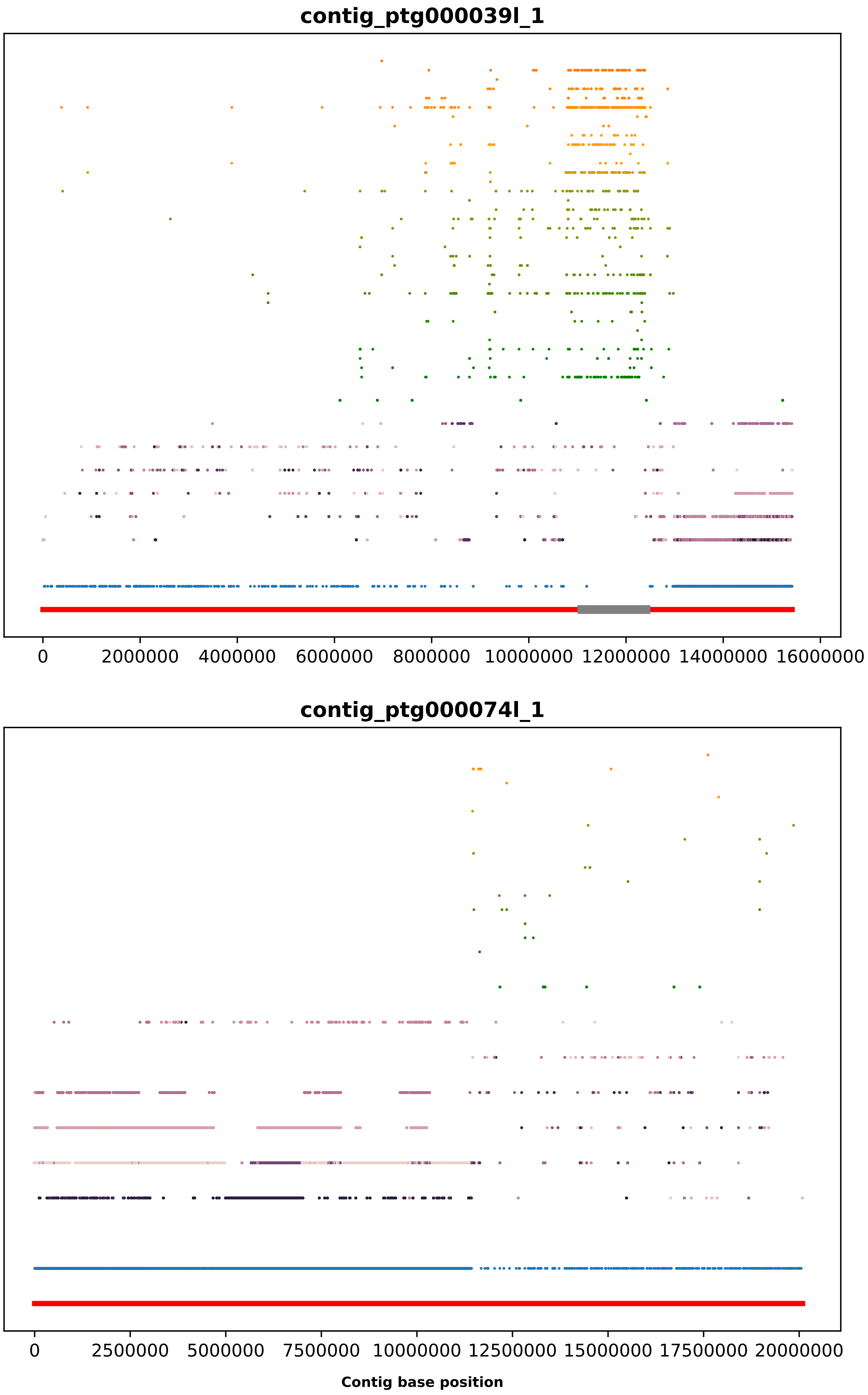
contig_ptg000039l_1
0
2000000
4000000
6000000
8000000
10000000
12000000
14000000
16000000
contig_ptg000074l_1
0
2500000
5000000
7500000
10000000
12500000
15000000
17500000
20000000
Contig base position

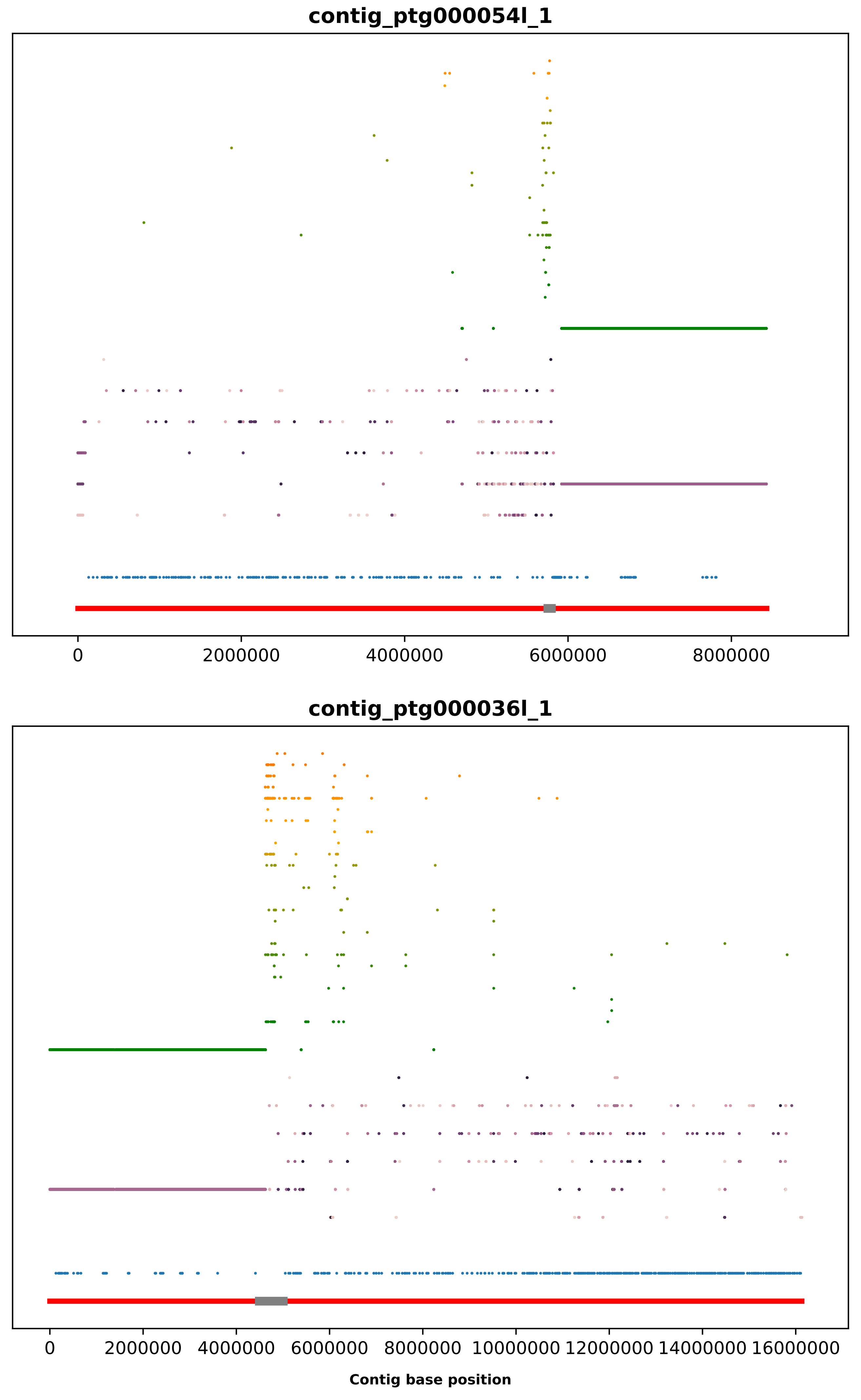

contig_ptg000054l_1
0
2000000
4000000
6000000
8000000
contig_ptg000036l_1
0
2000000
4000000
6000000
8000000
10000000
12000000
14000000
16000000
Contig base position
