# Supplementary material for: A high quality genome of the common swamp pitcher plant (Nepenthes mirabilis) using PacBio HiFi sequencing
Source: PLoS One. 2025 Jul 10;20(7):e0322885. doi: 10.1371/journal.pone.0322885 (PMC12244726; doi:10.1371/journal.pone.0322885)

Supplementary Figure S6. RepeatOBserver plots for all *N. mirabilis* putative full-chromosome contigs. (a) Heatmap of Fourier spectra showing locations of repeats (including their length and how perfectly they repeat) for long repeat lengths 35-2000 bp. **(b)** Plot of the rolling sum of repeat abundance. **(c)** Plot of the Shannon diversity values for repeats, averaged with a rolling window across 250 windows (1.25Mbp region). See the RepeatOBserver manuscript for further details.

ptg000002l_1

a)

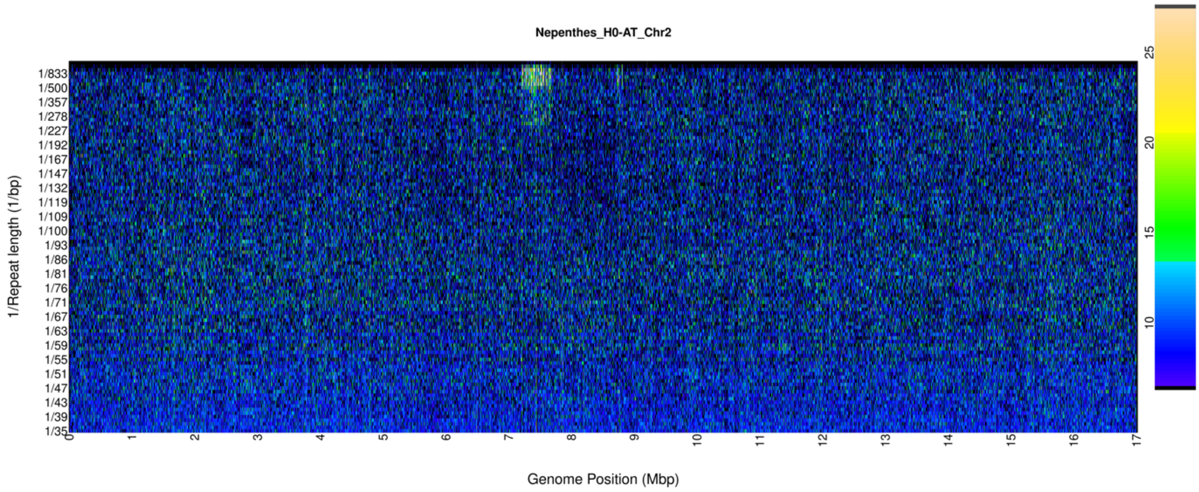

b)

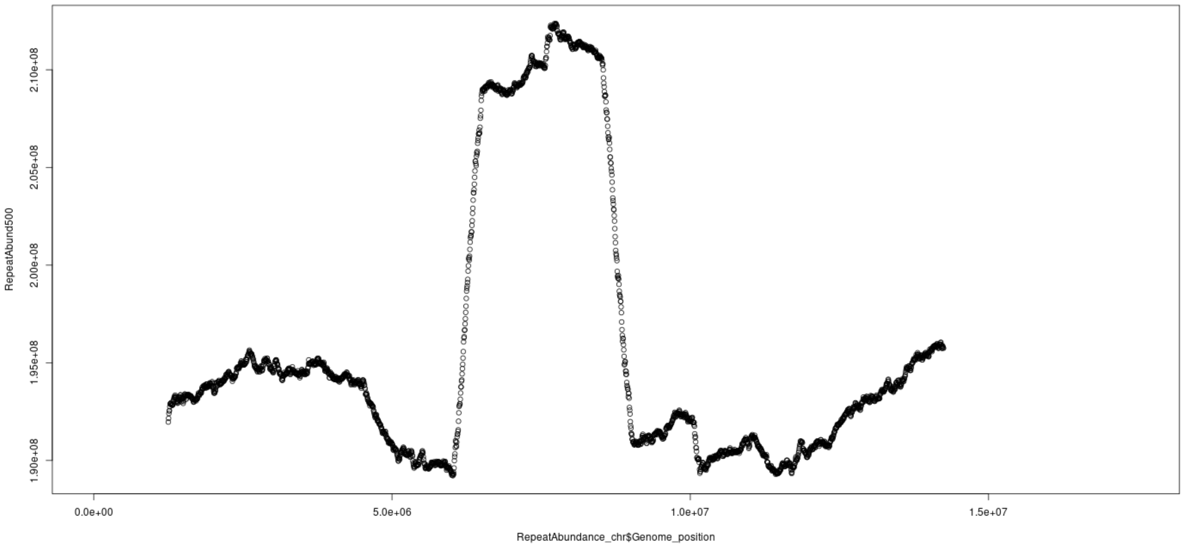

c)

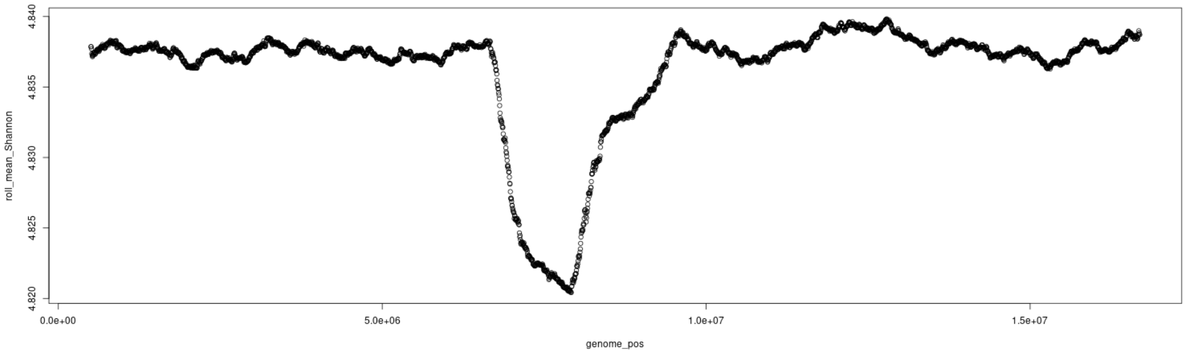

ptg000003l_1

a)

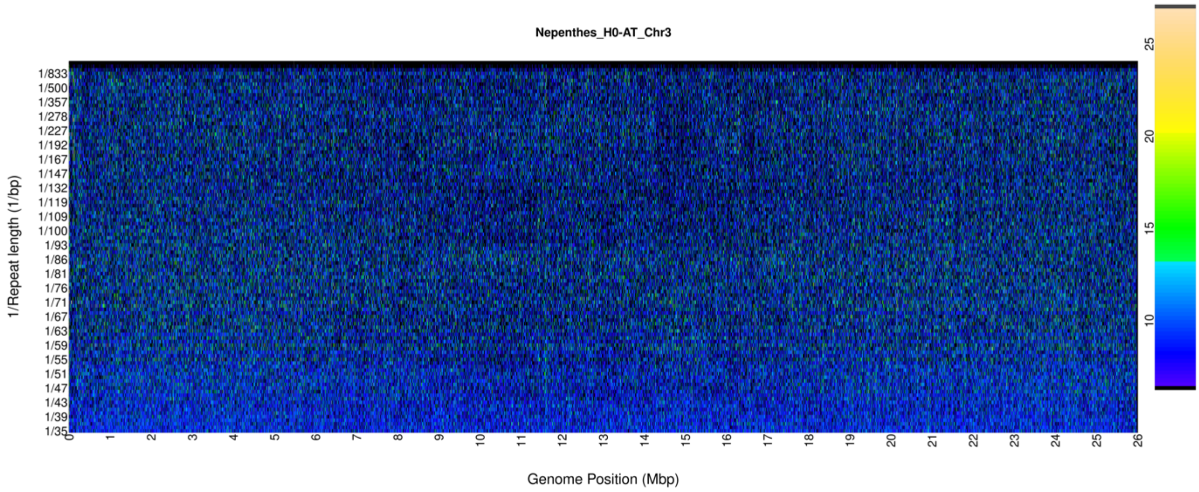

b)

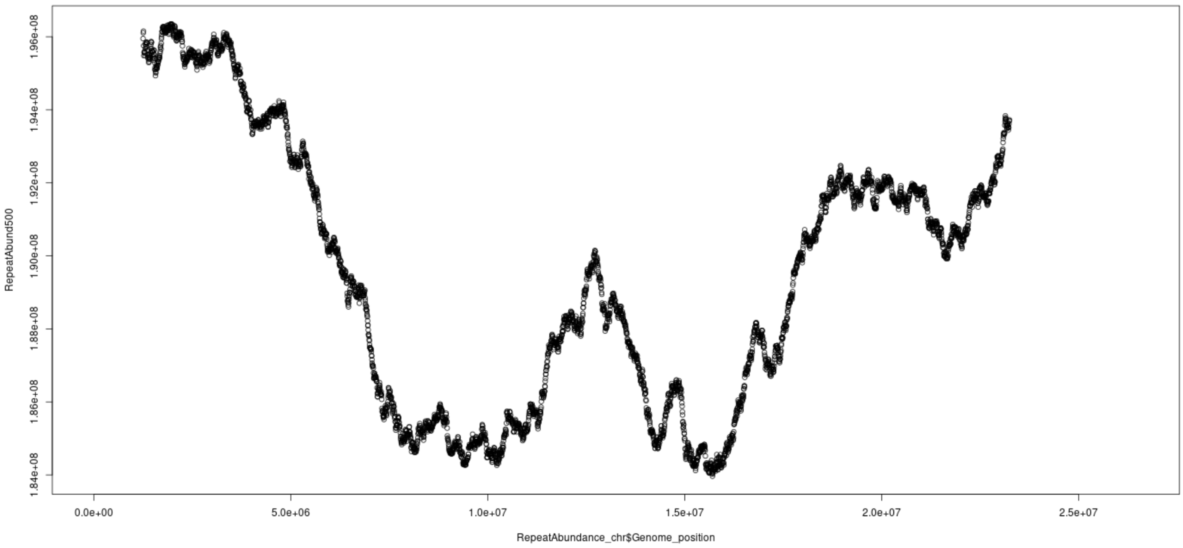

c)

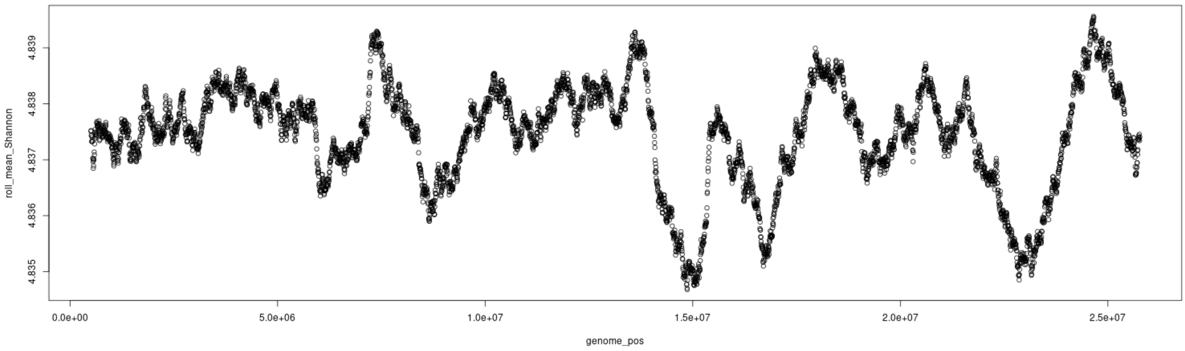

ptg000004l_1

a)

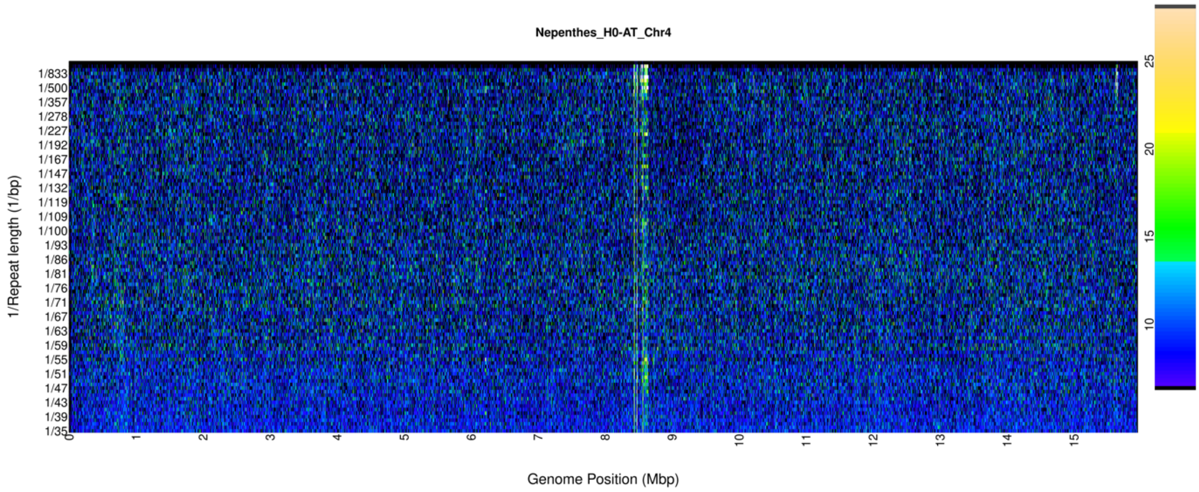

b)

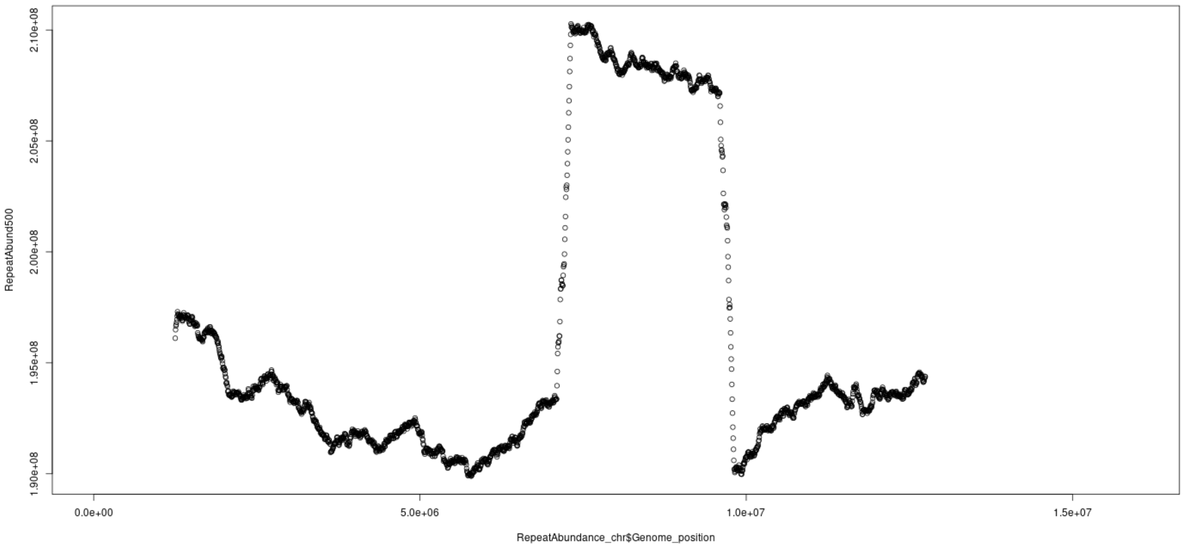

c)

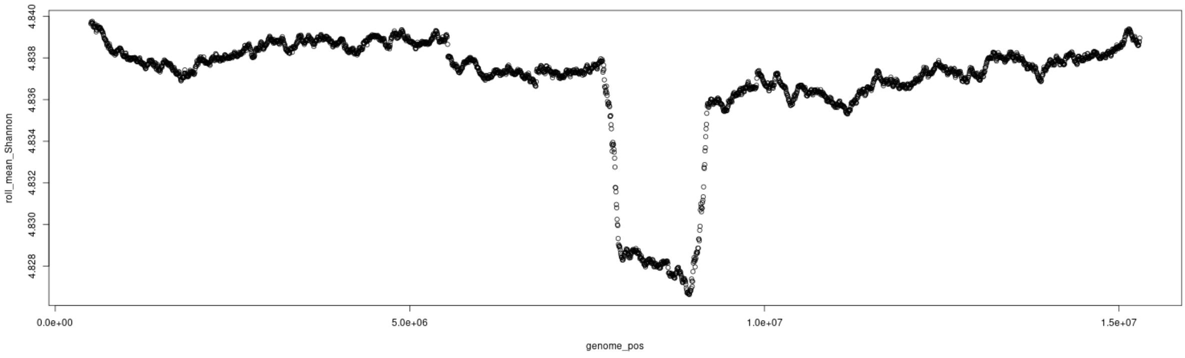

ptg000005l_1

a)

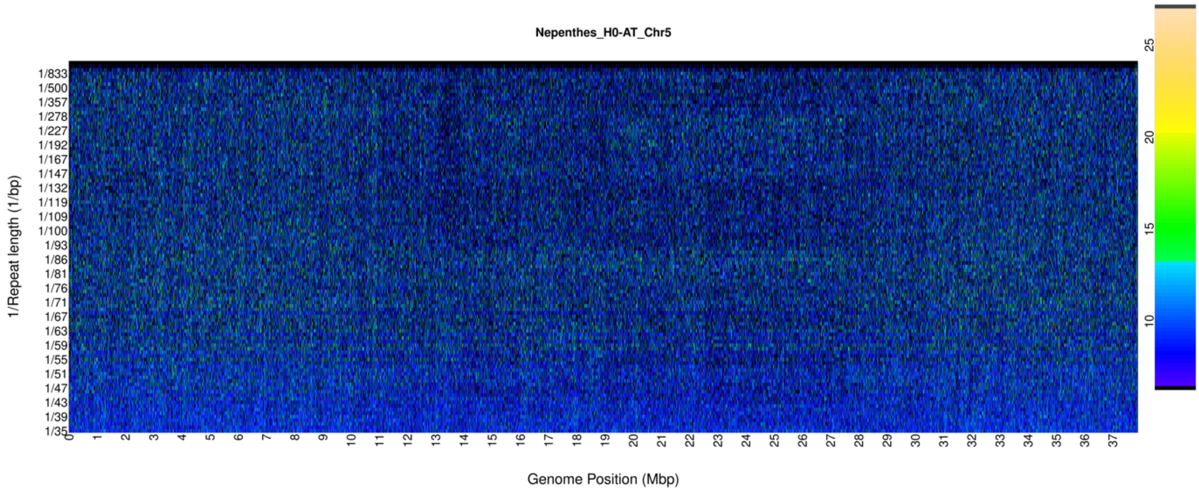

b)

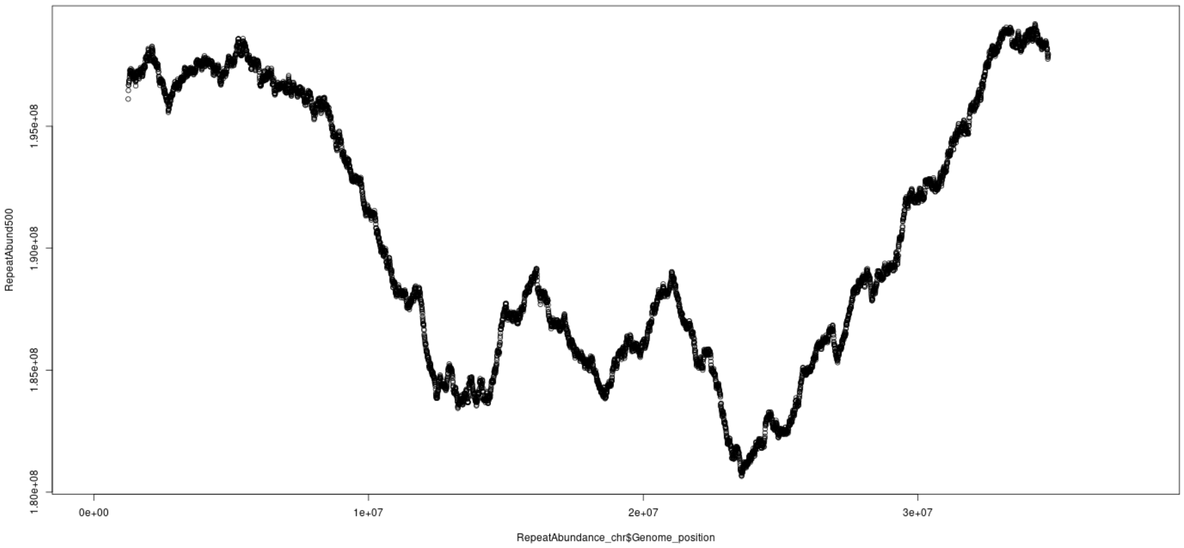

c)

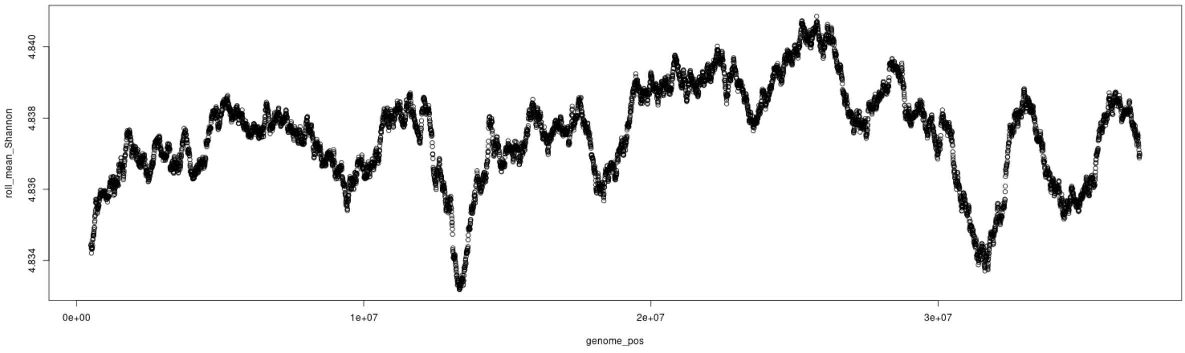

ptg000006l_1

a)

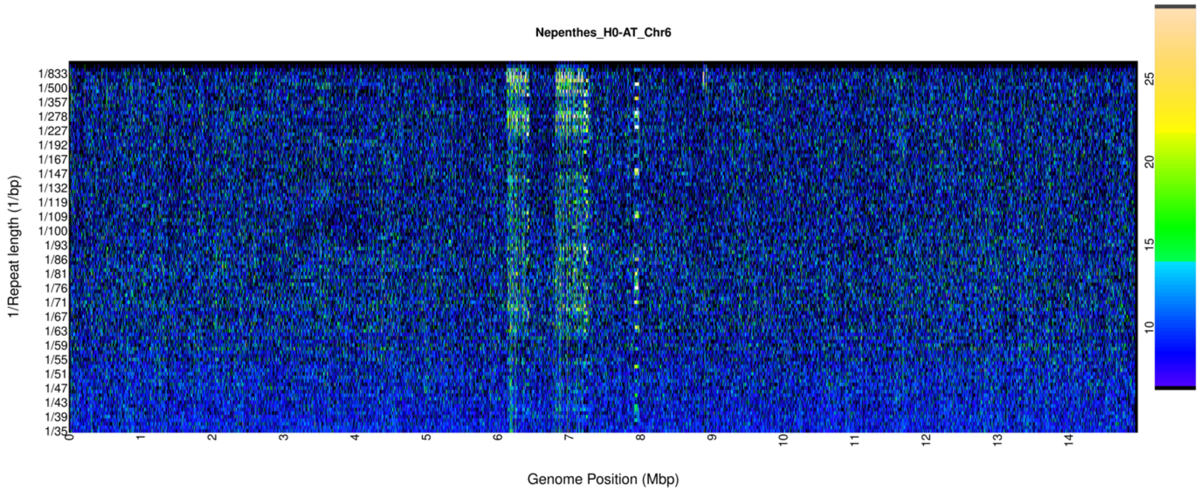

b)

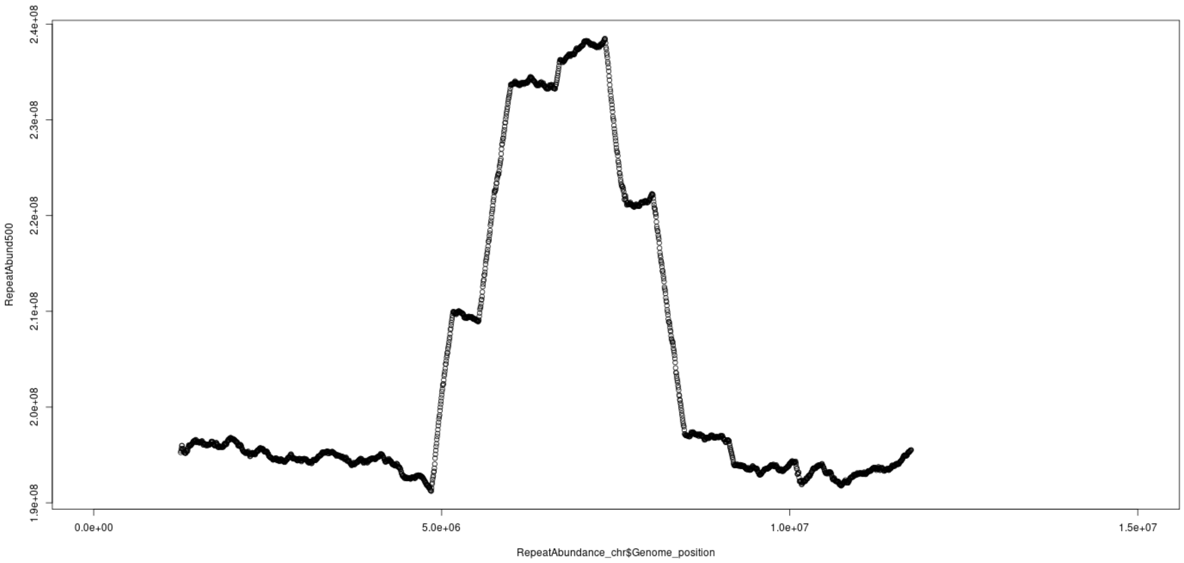

c)

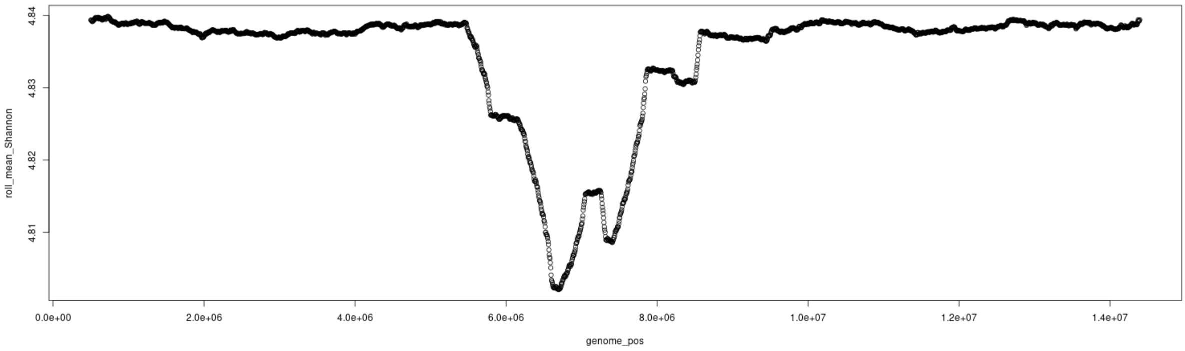

ptg000007l_1

a)

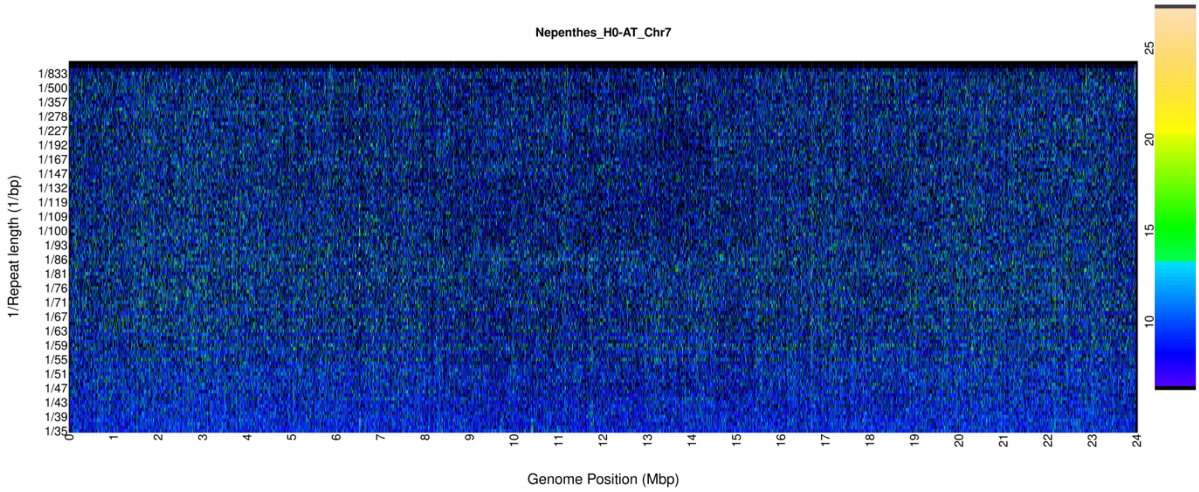

b)

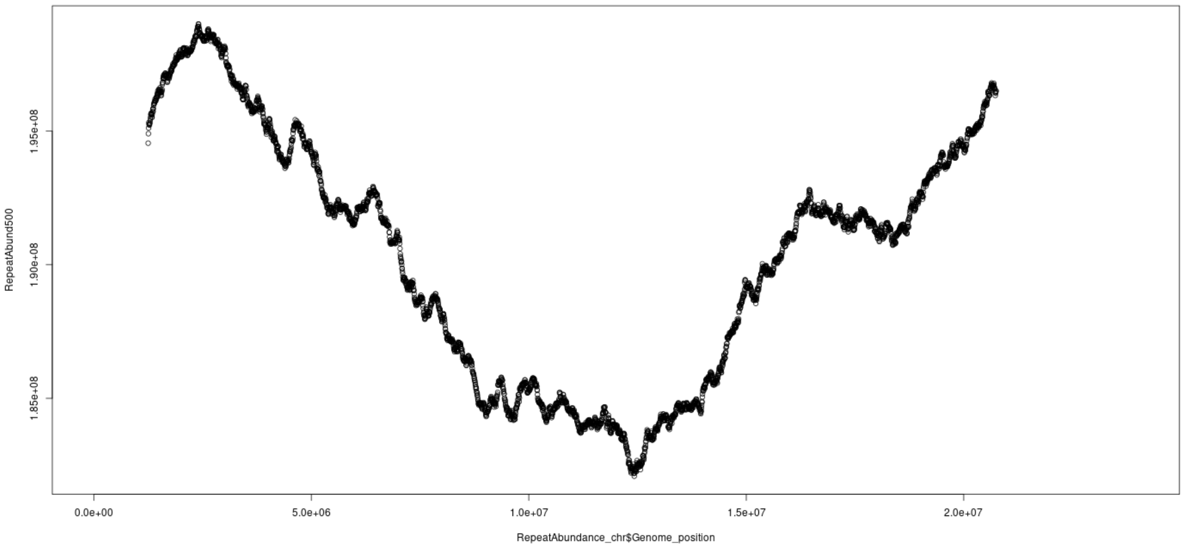

c)

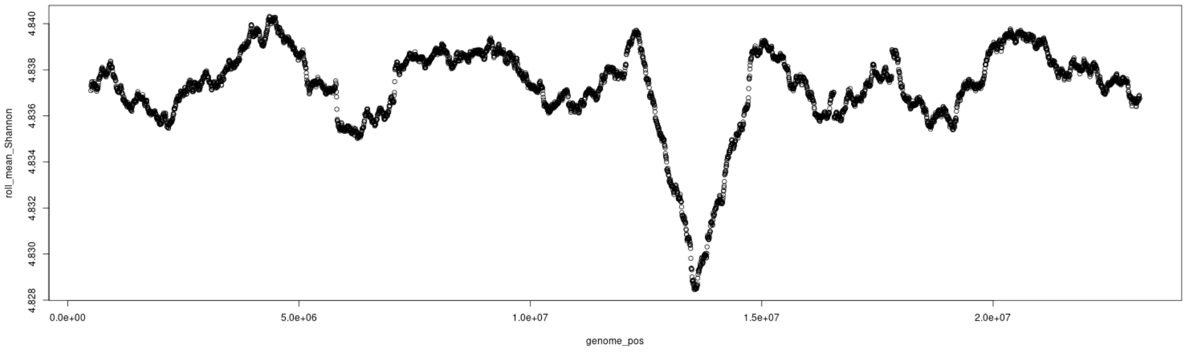

ptg000008l_1

a)

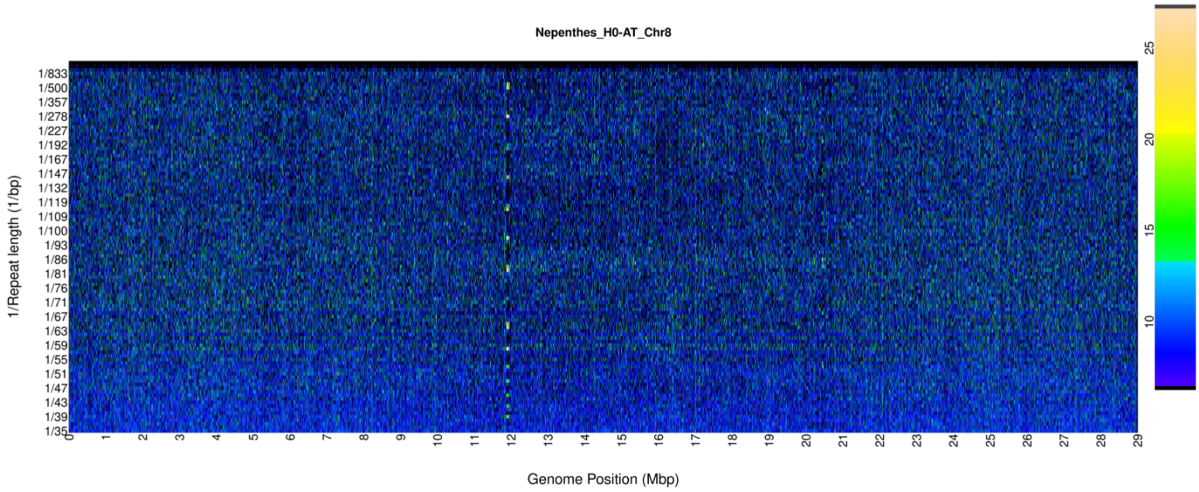

b)

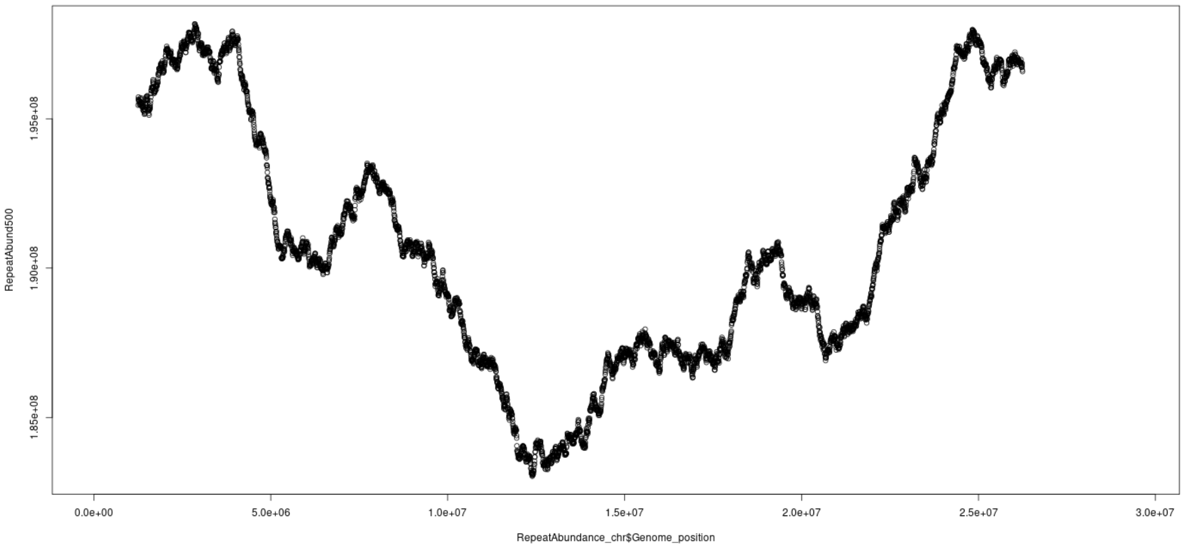

c)

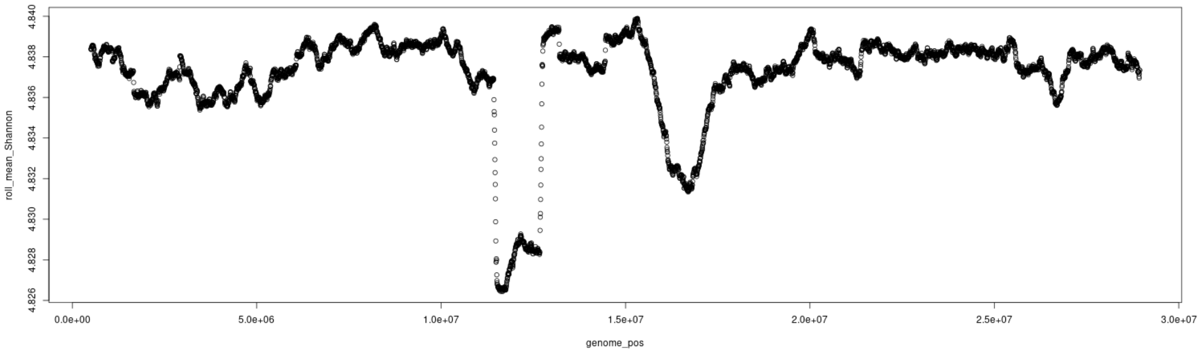

ptg000009l_1

a)

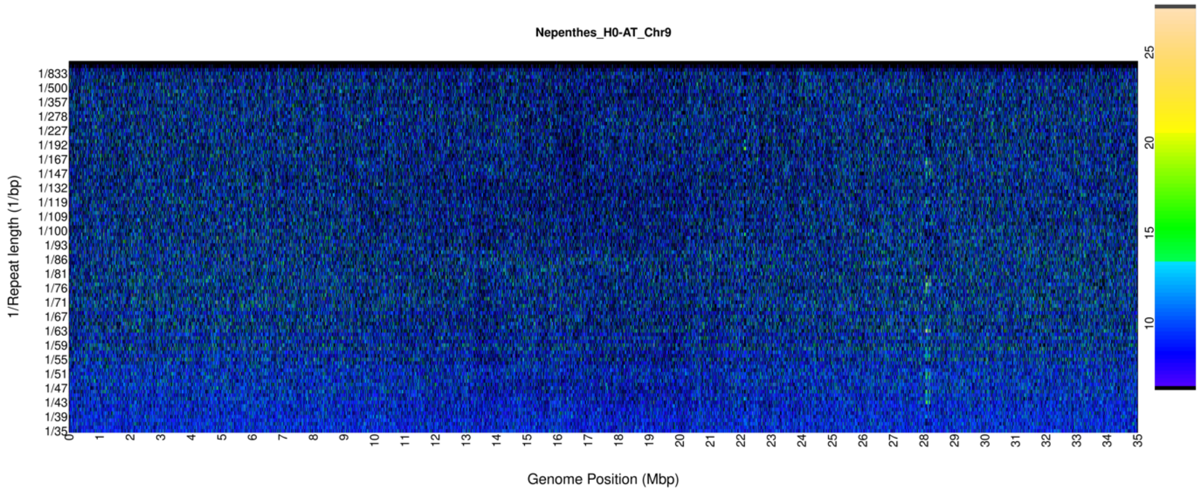

b)

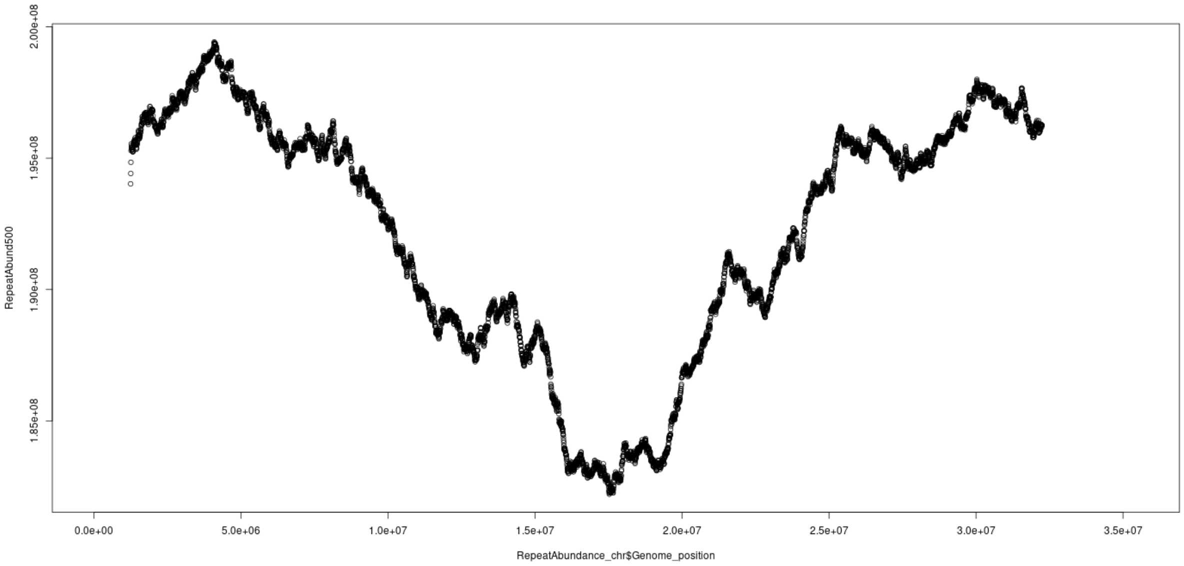

c)

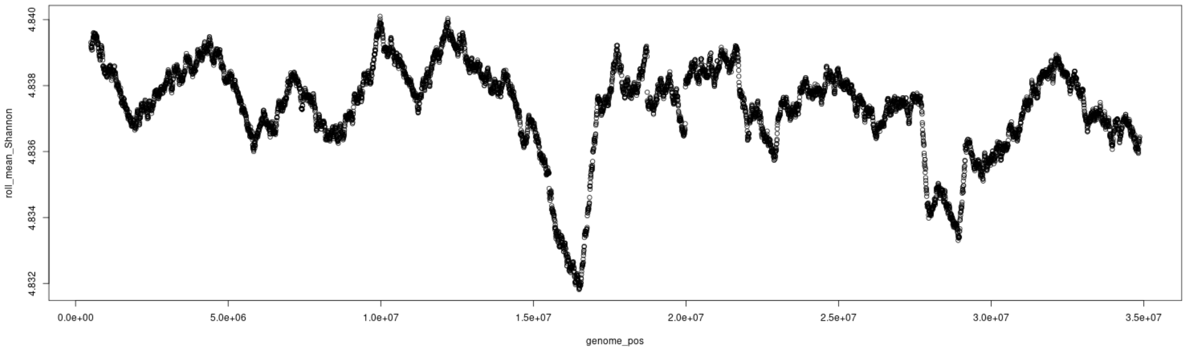

ptg000010l_1

a)

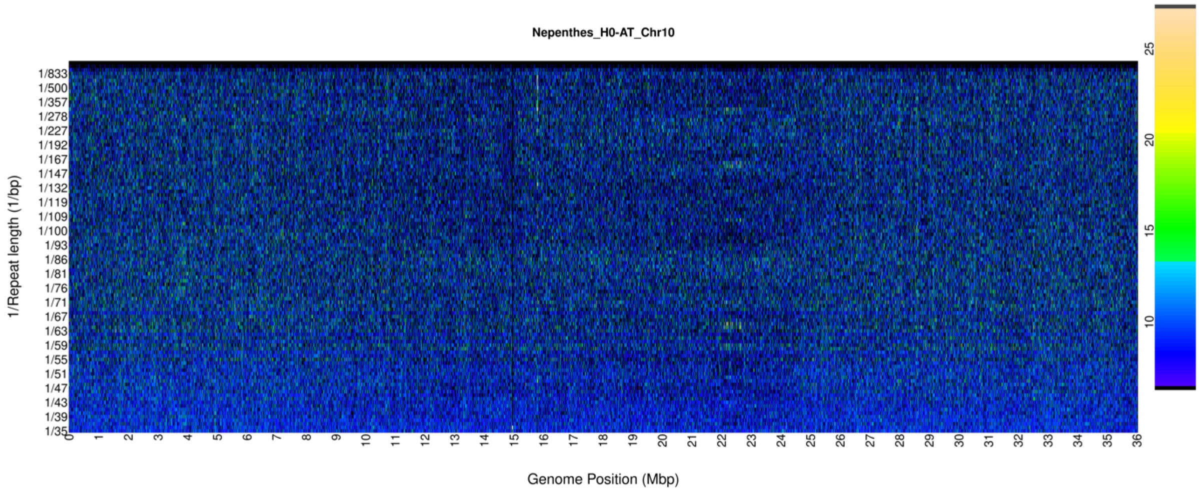

b)

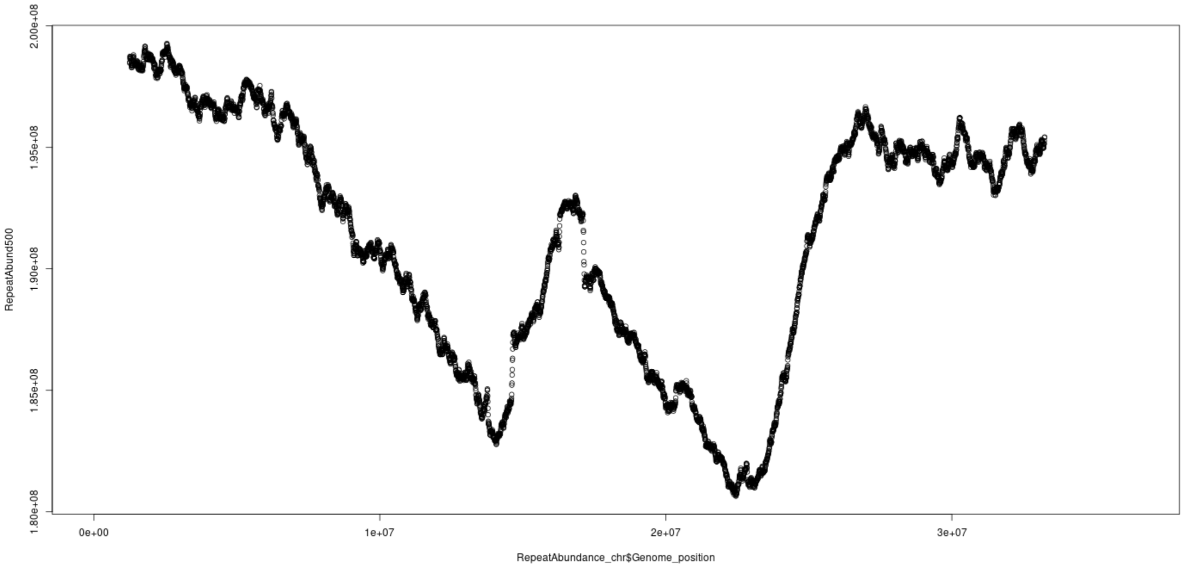

c)

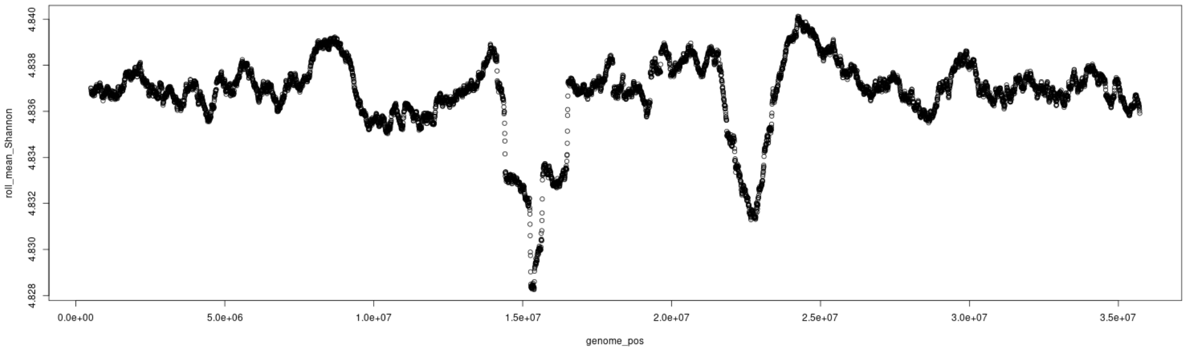

ptg000011l_1

a)

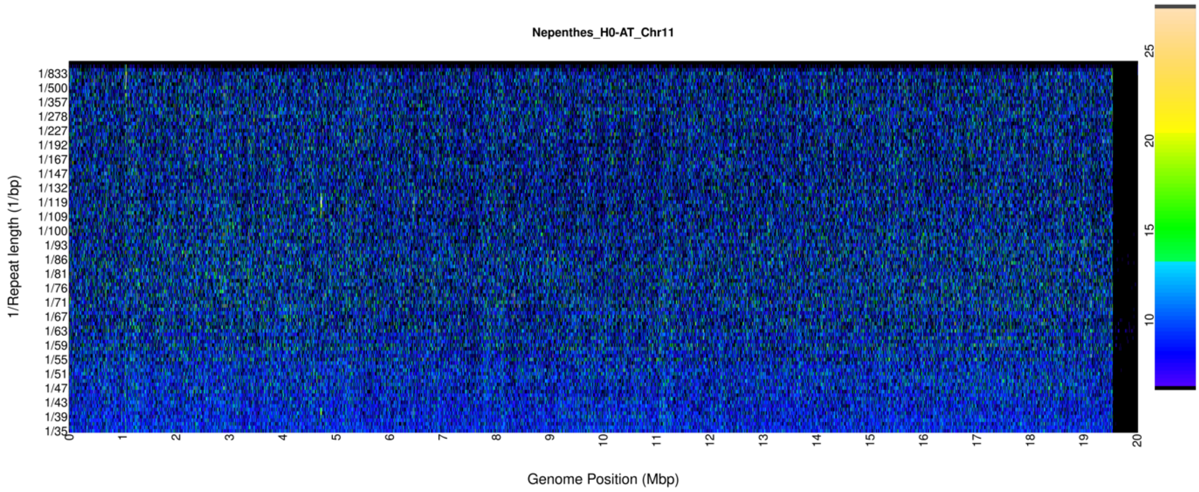

b)

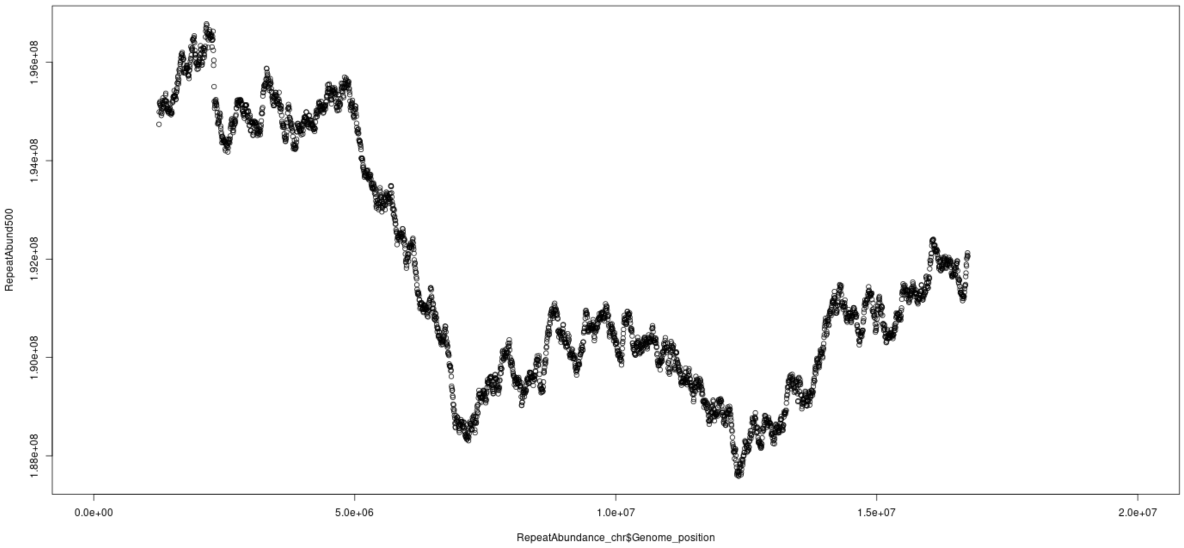

c)

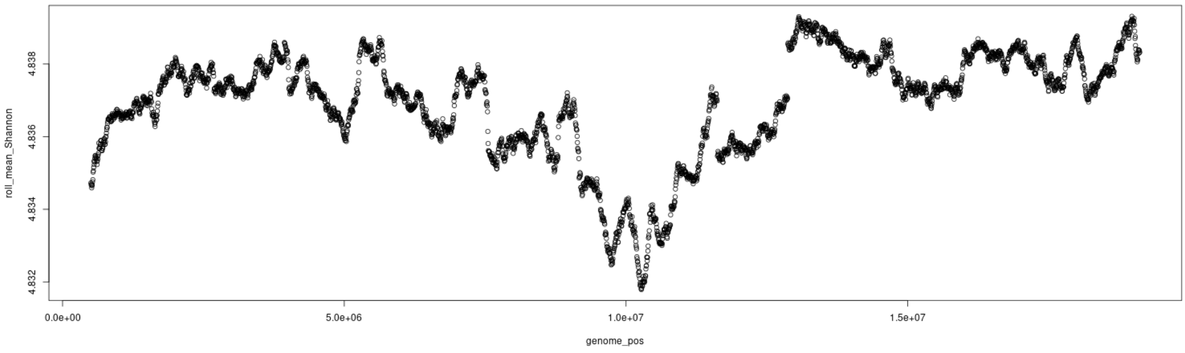

ptg000013l_1

a)

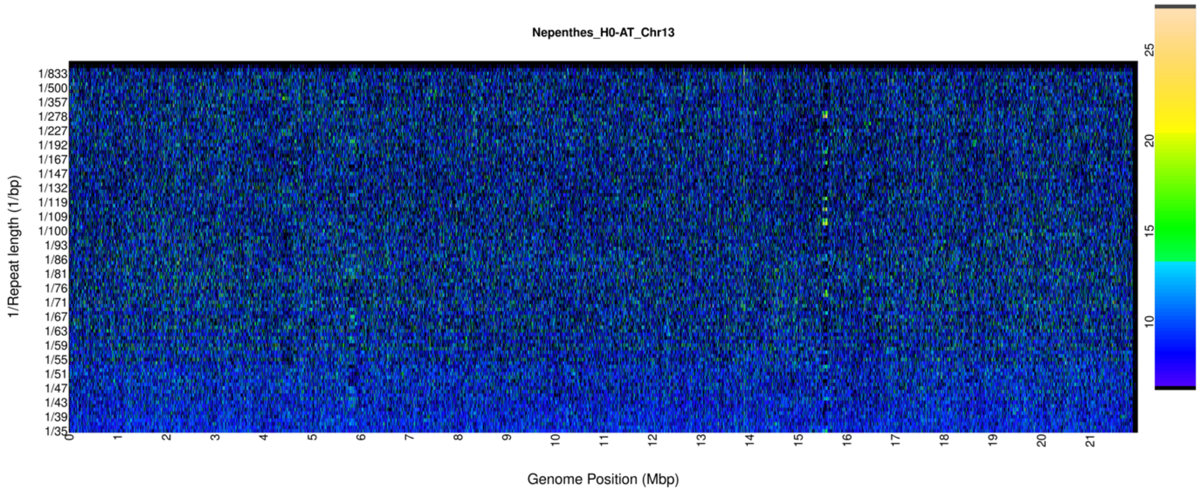

b)

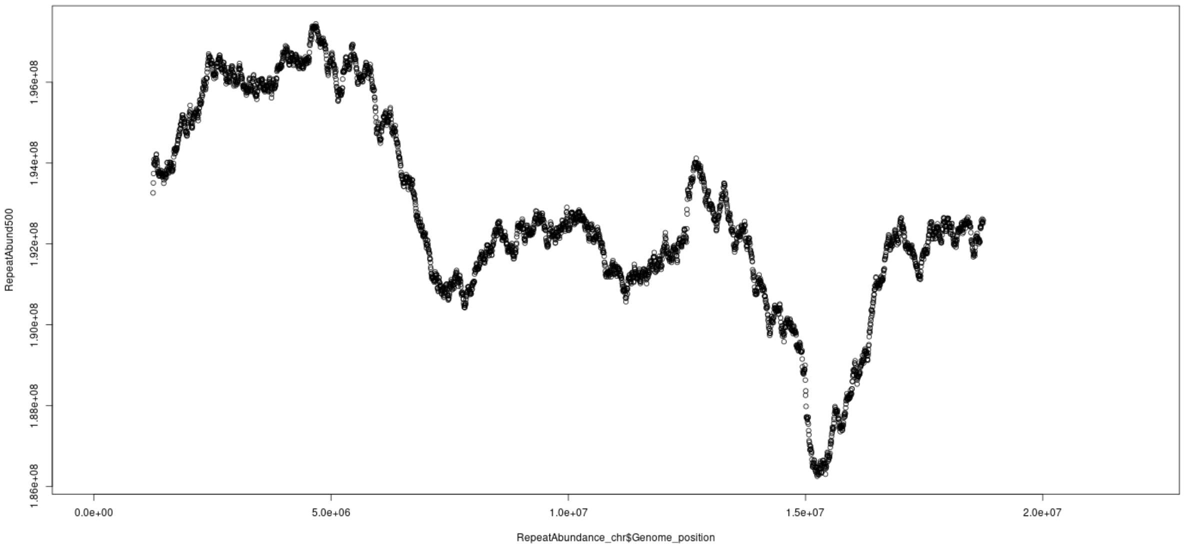

c)

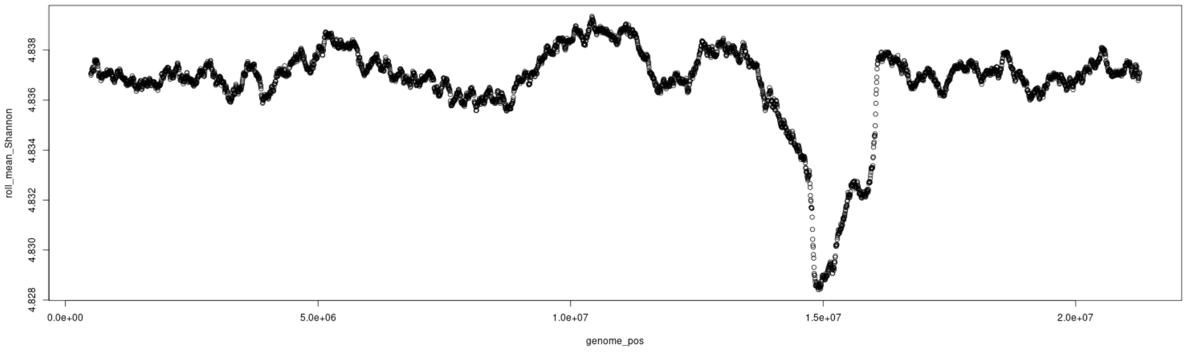

ptg000014l_1

a)

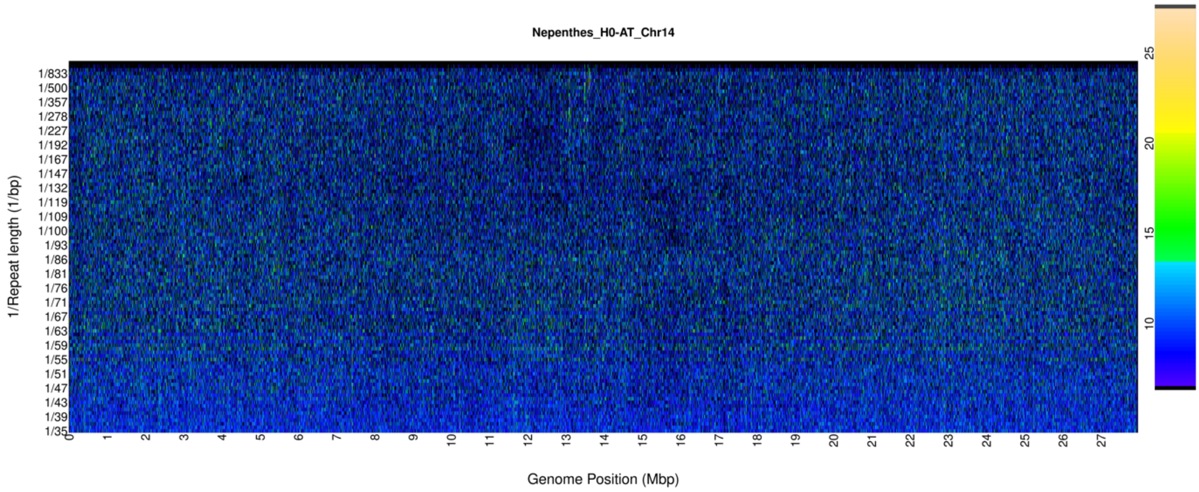

b)

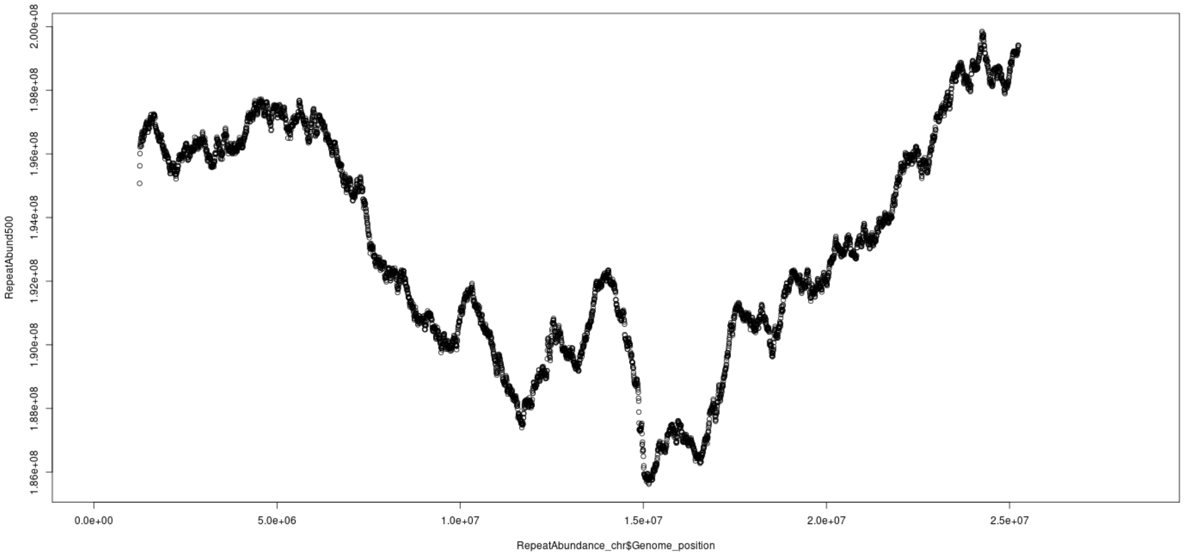

c)

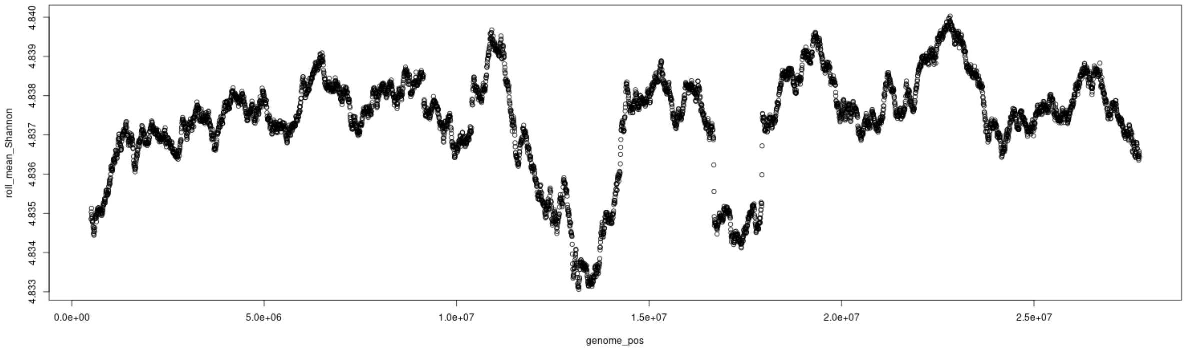

ptg000022l_1

a)

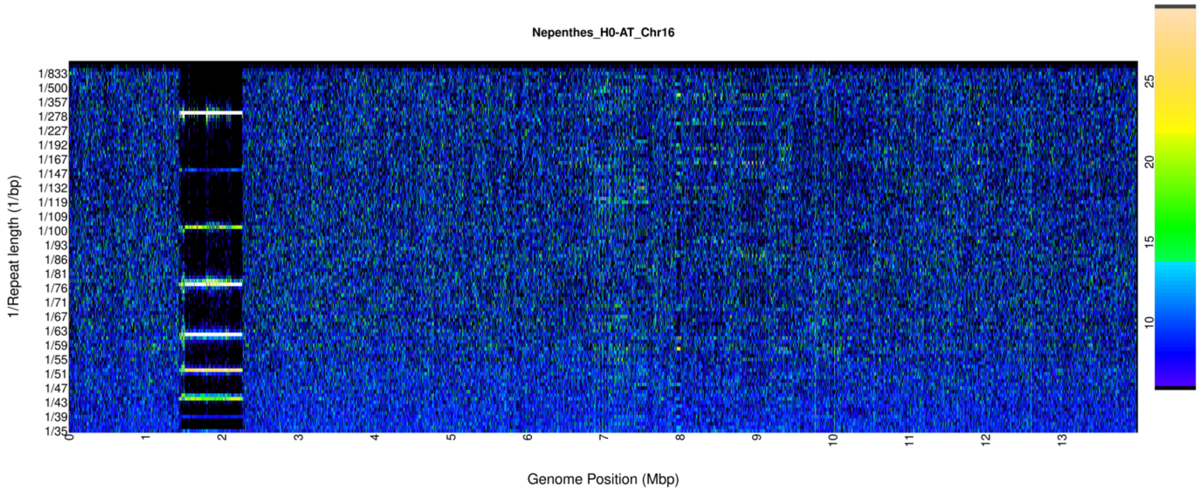

b)

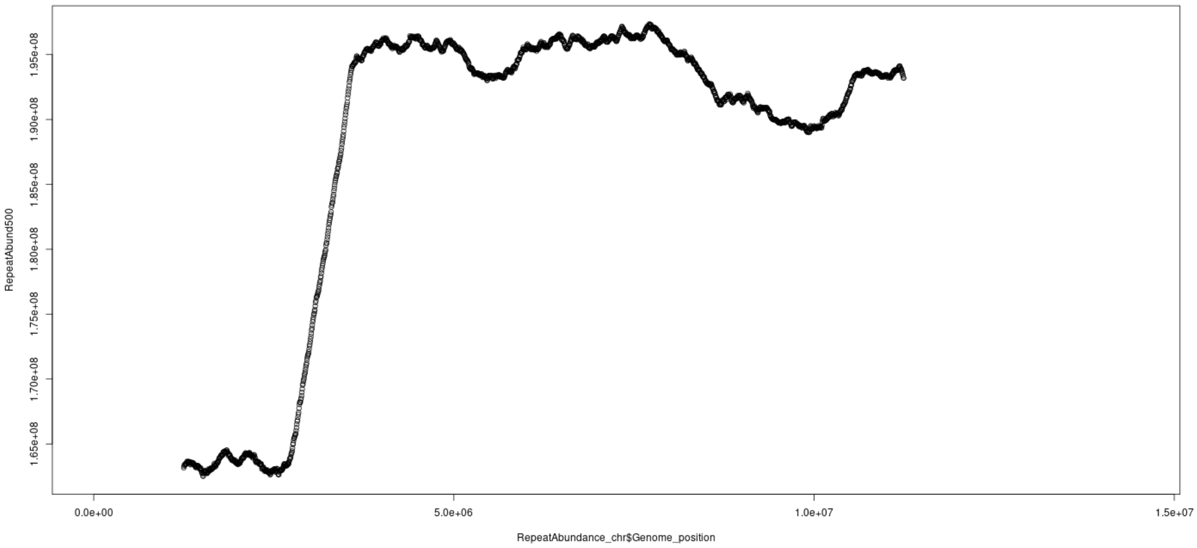

c)

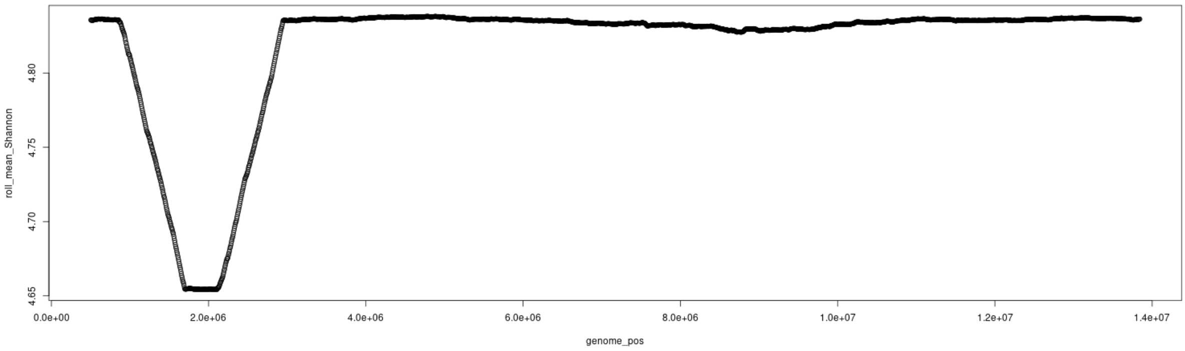

ptg000023l_1

a)

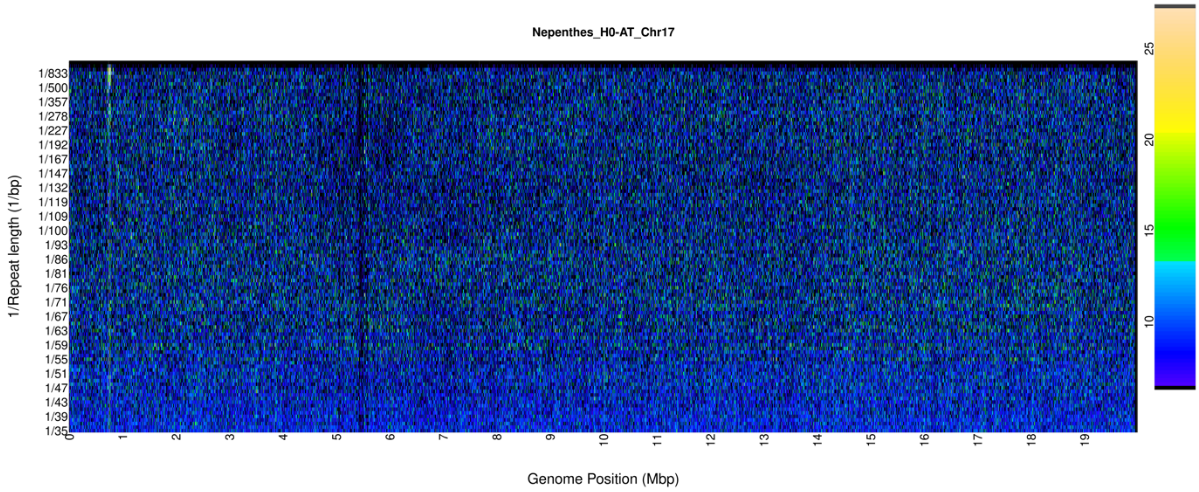

b)

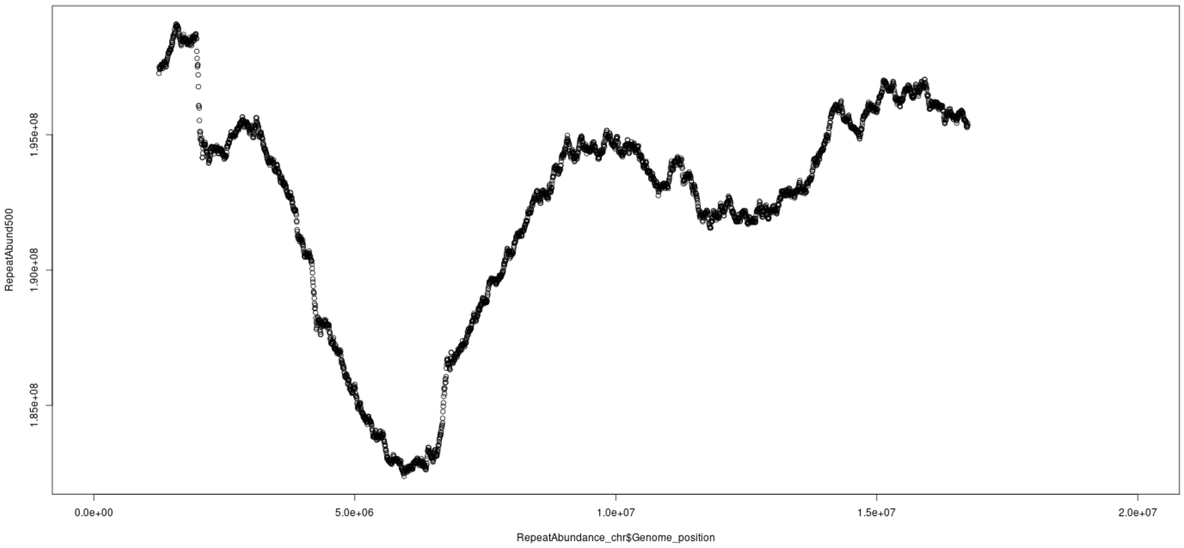

c)

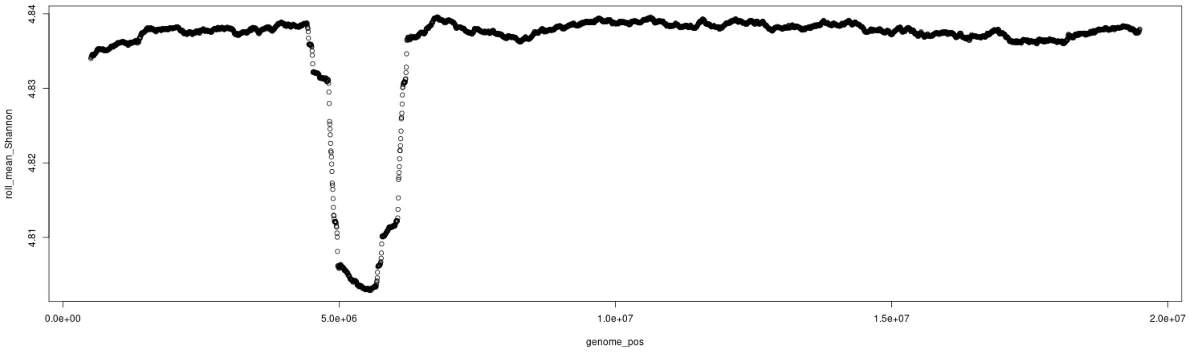

ptg000024l_1

a)

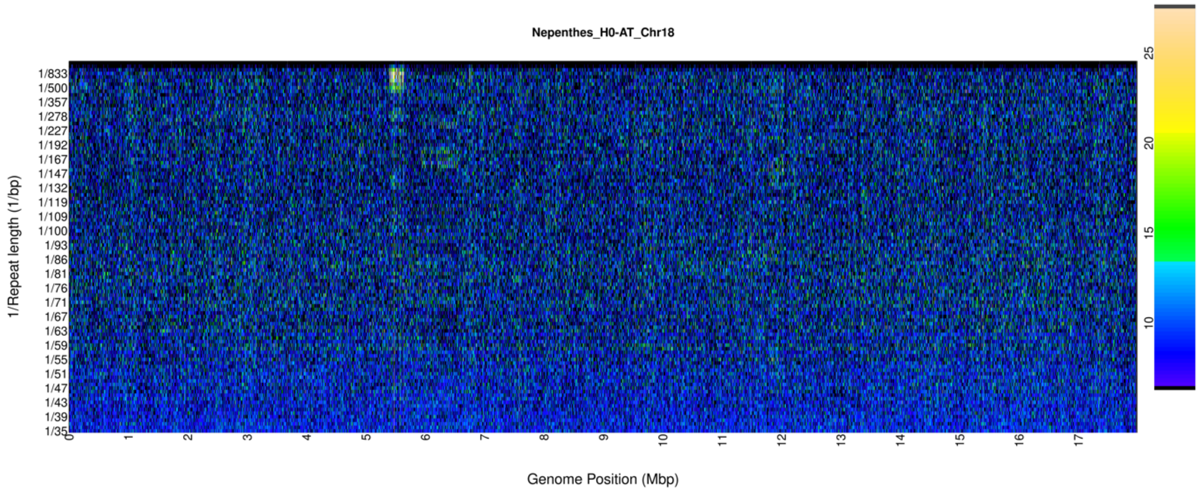

b)

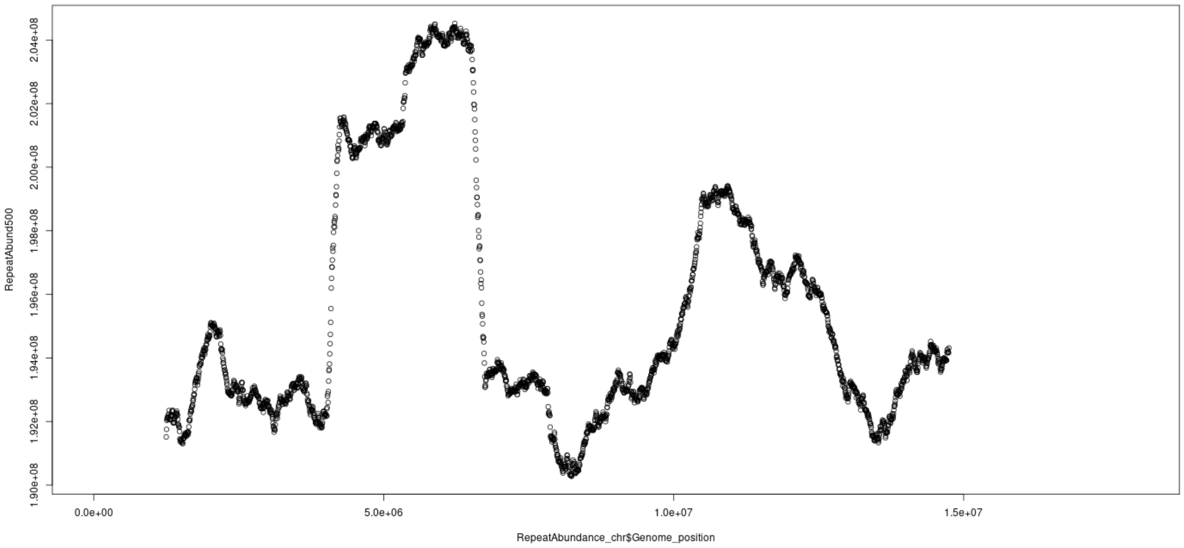

c)

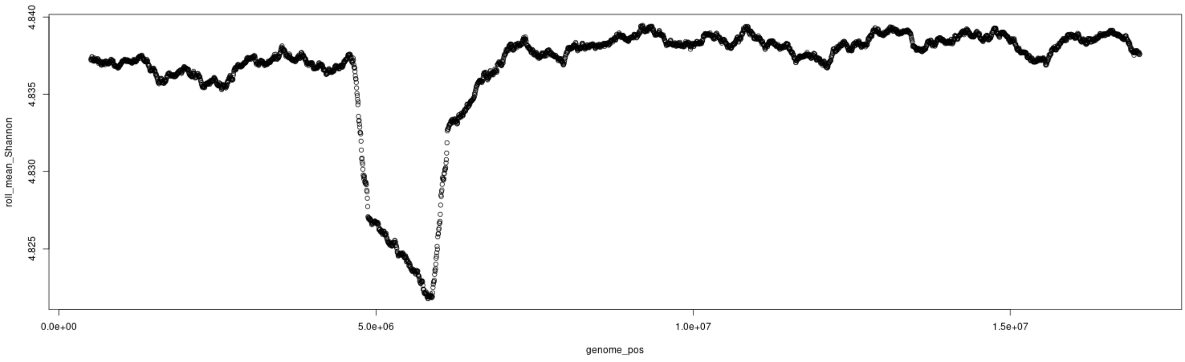

ptg000028l_1

a)

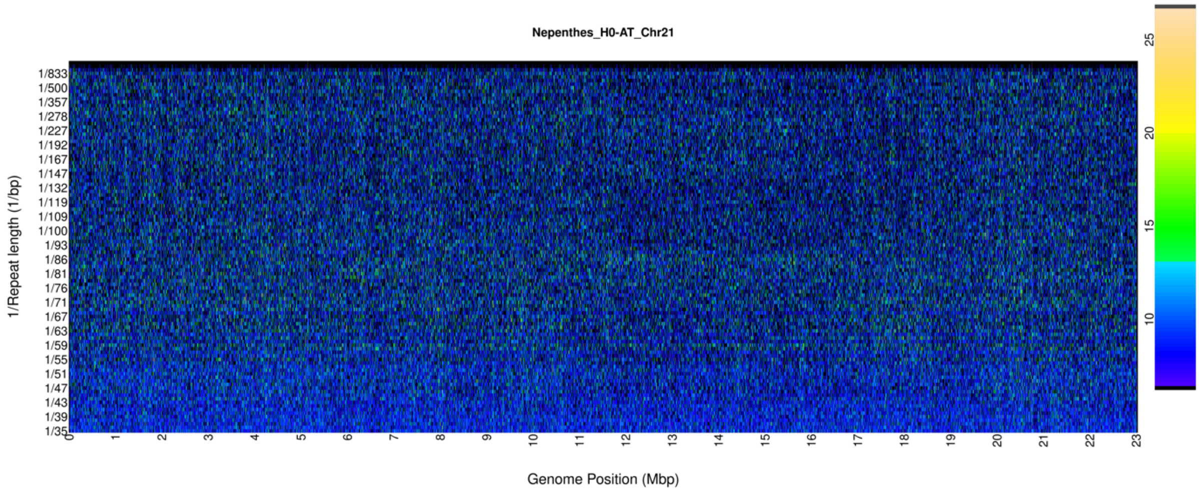

b)

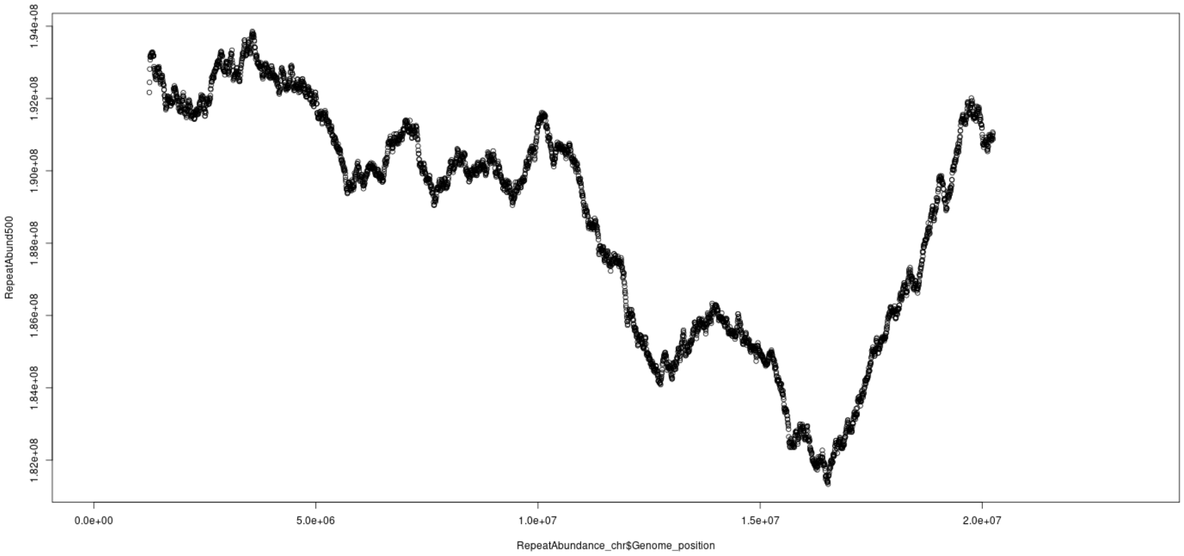

c)

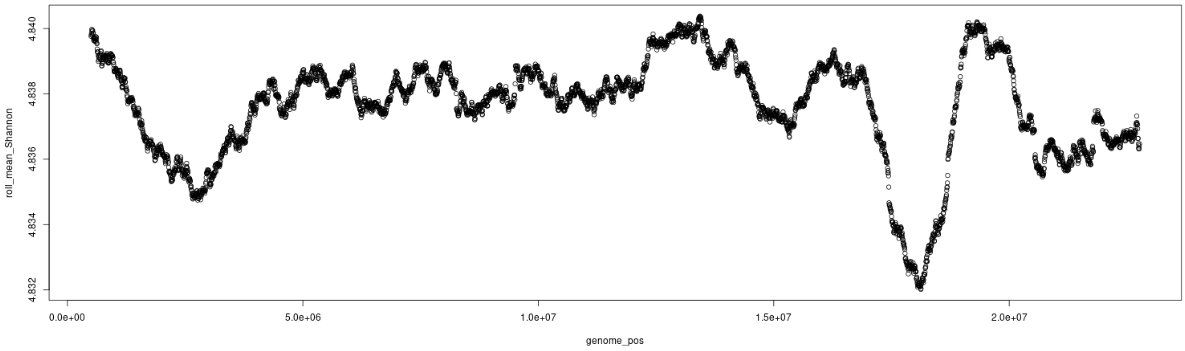

ptg000029l_1

a)

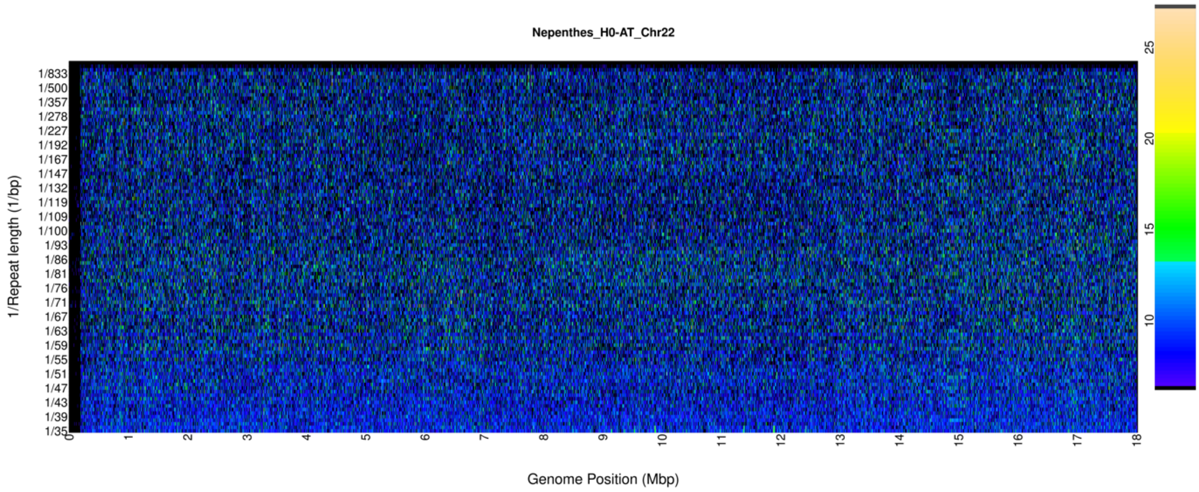

b)

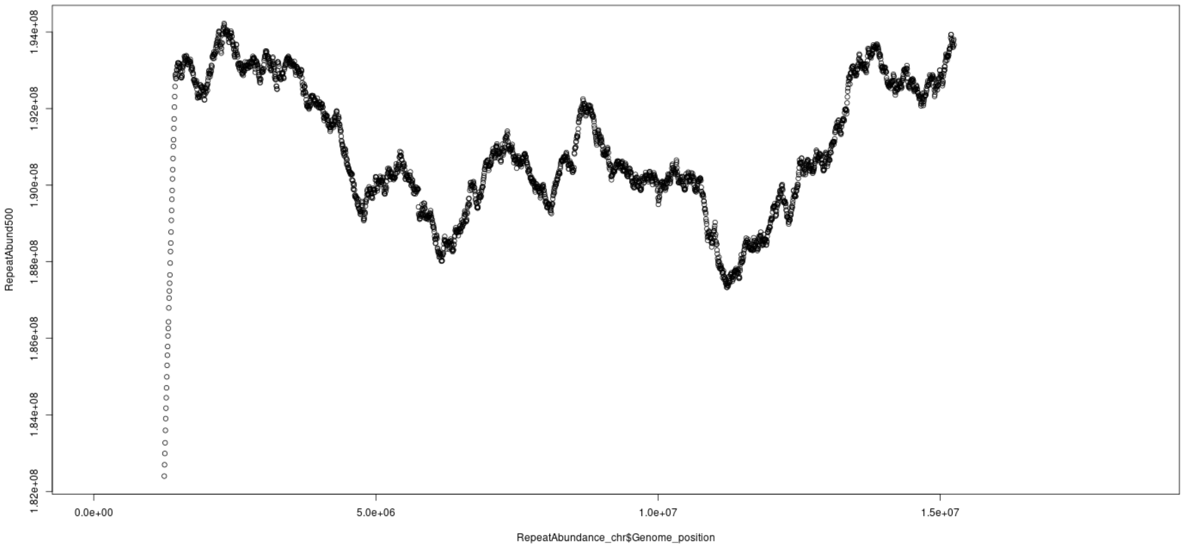

c)

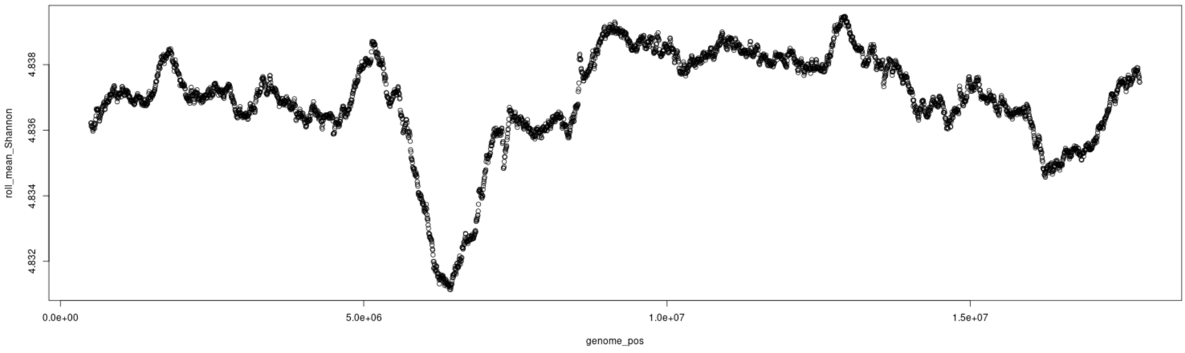

ptg000030l_1

a)

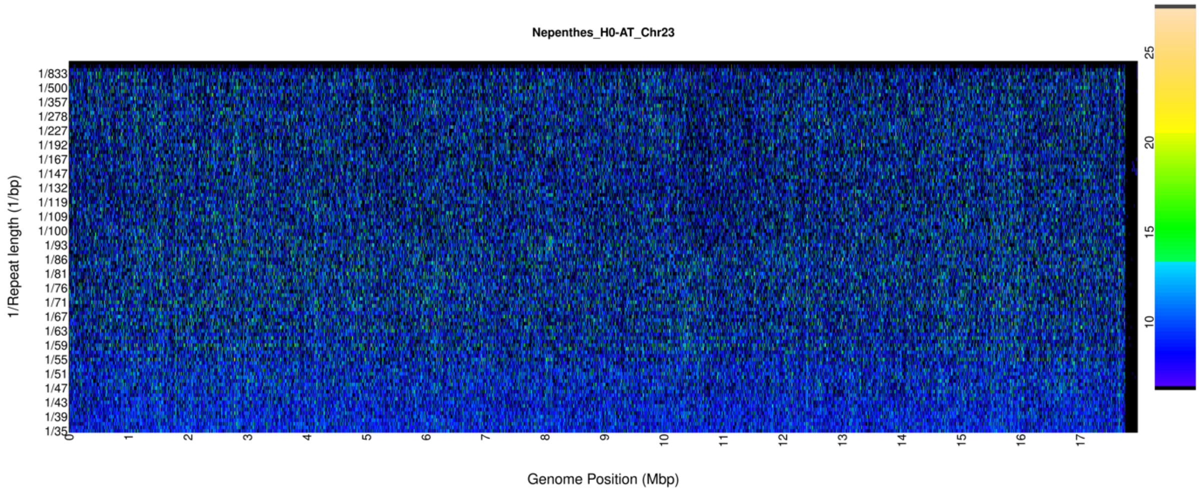

b)

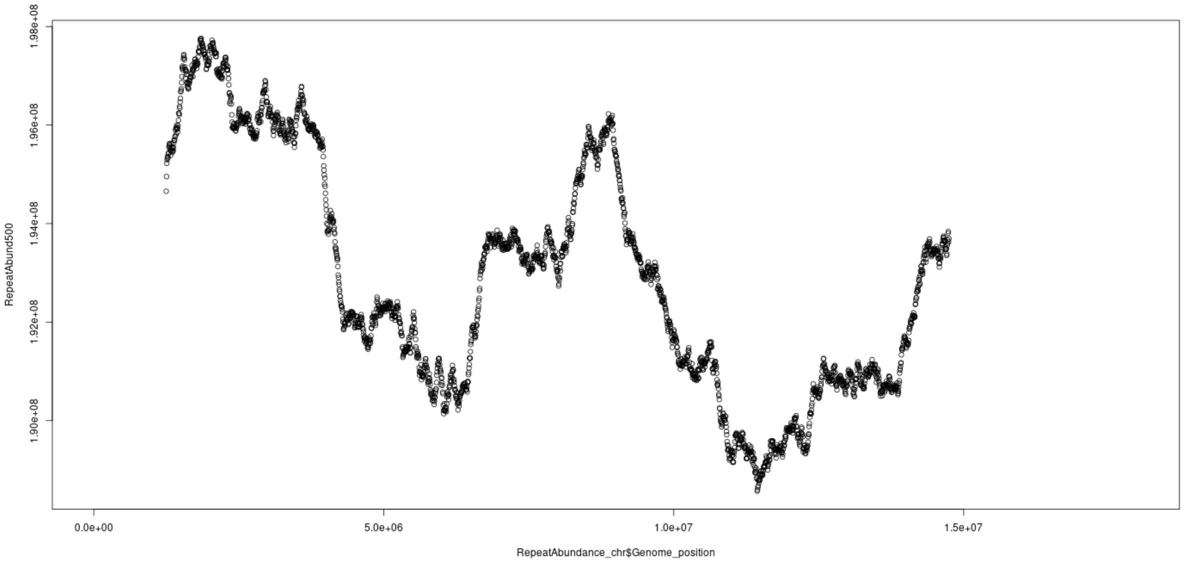

c)

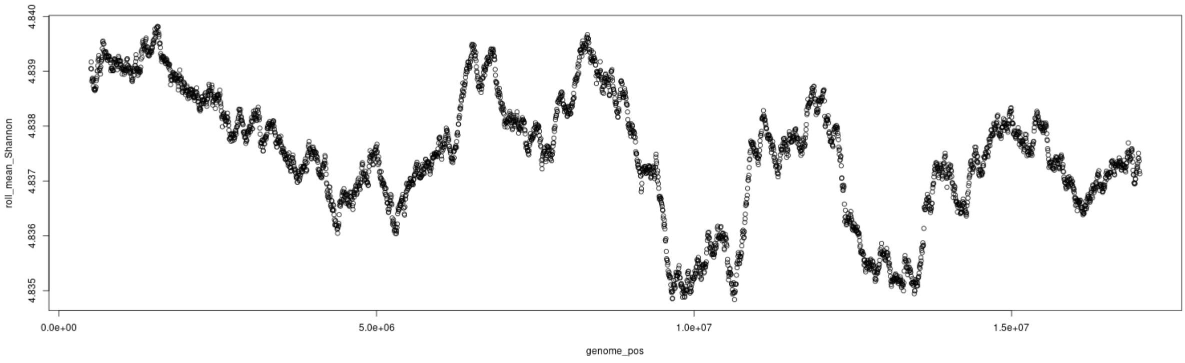

ptg000031l_1

a)

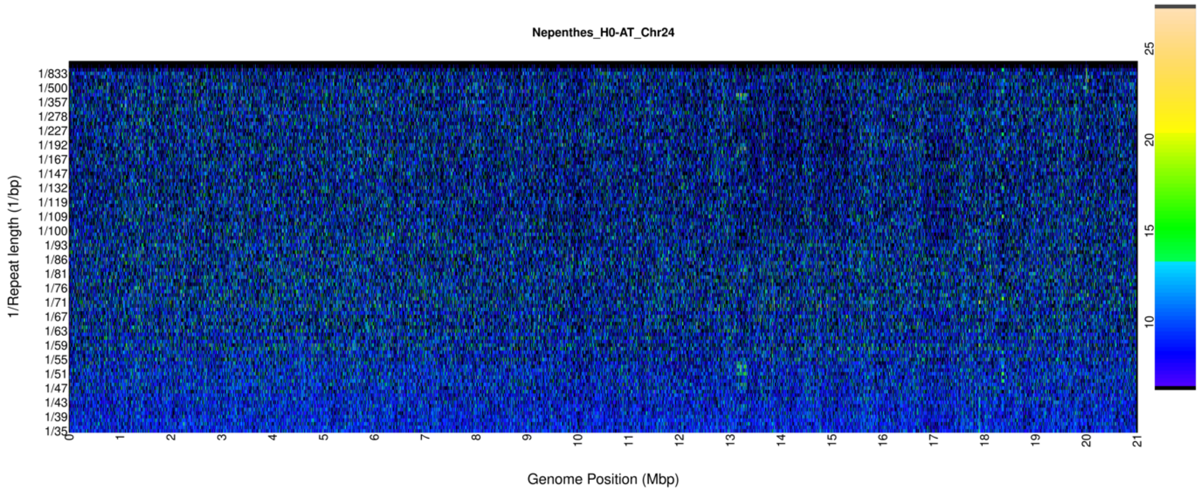

b)

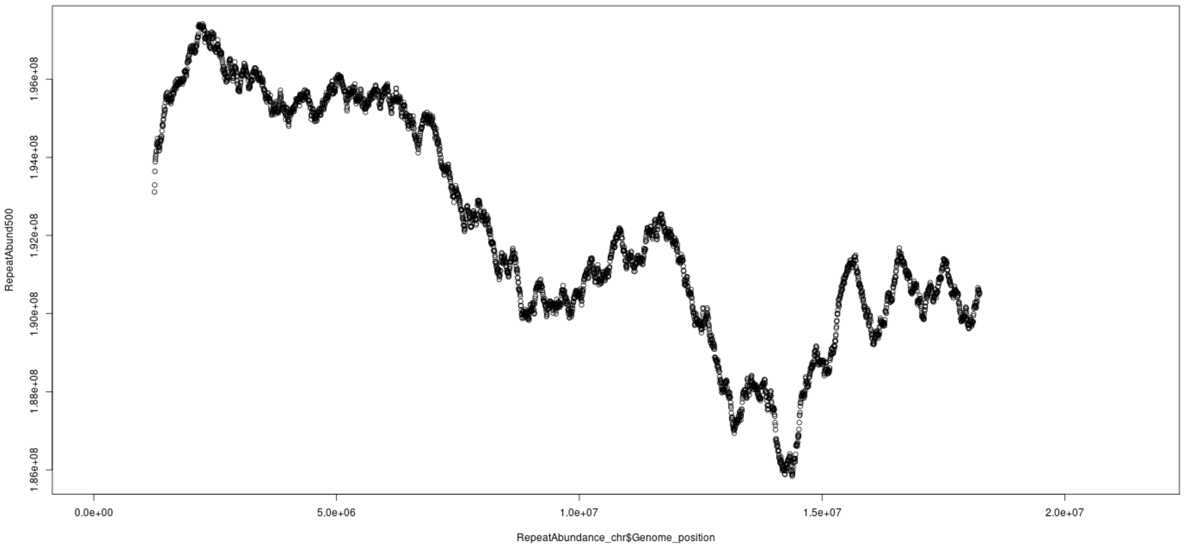

c)

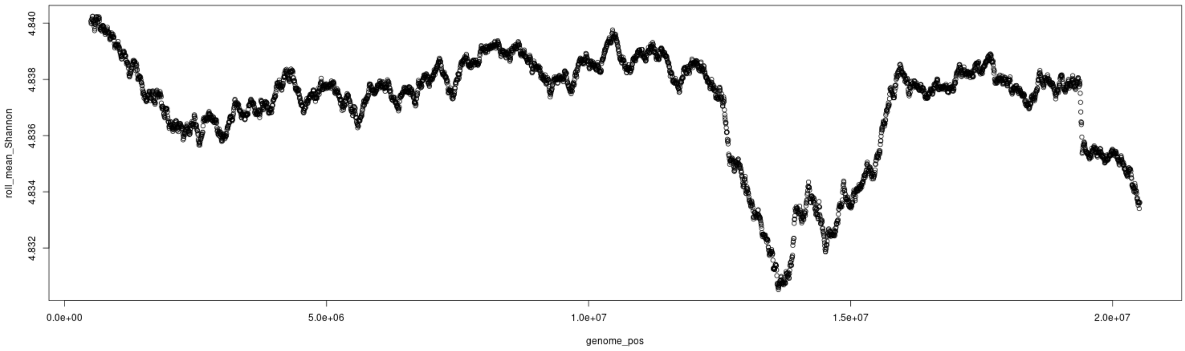

ptg000040l_1

a)

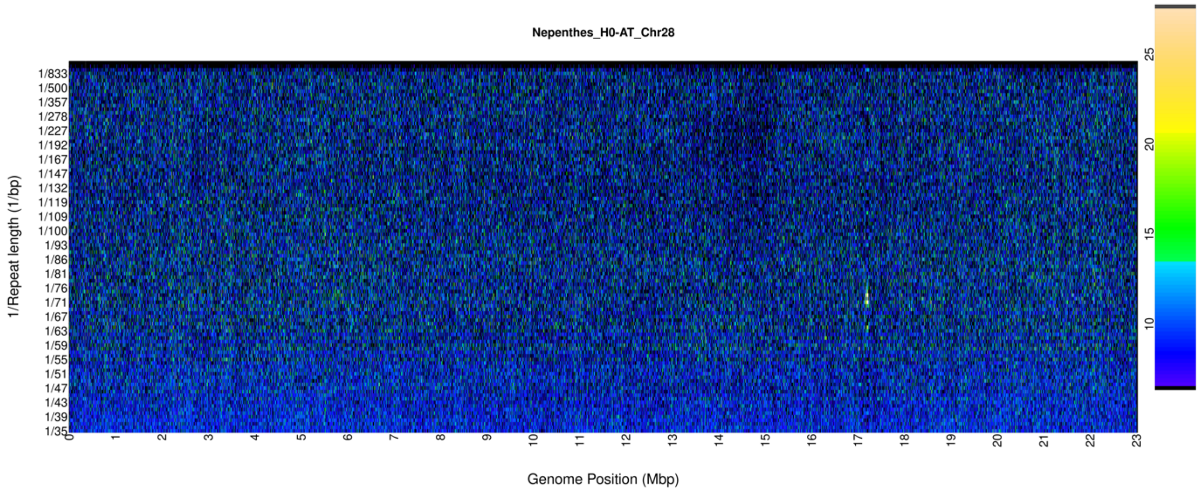

b)

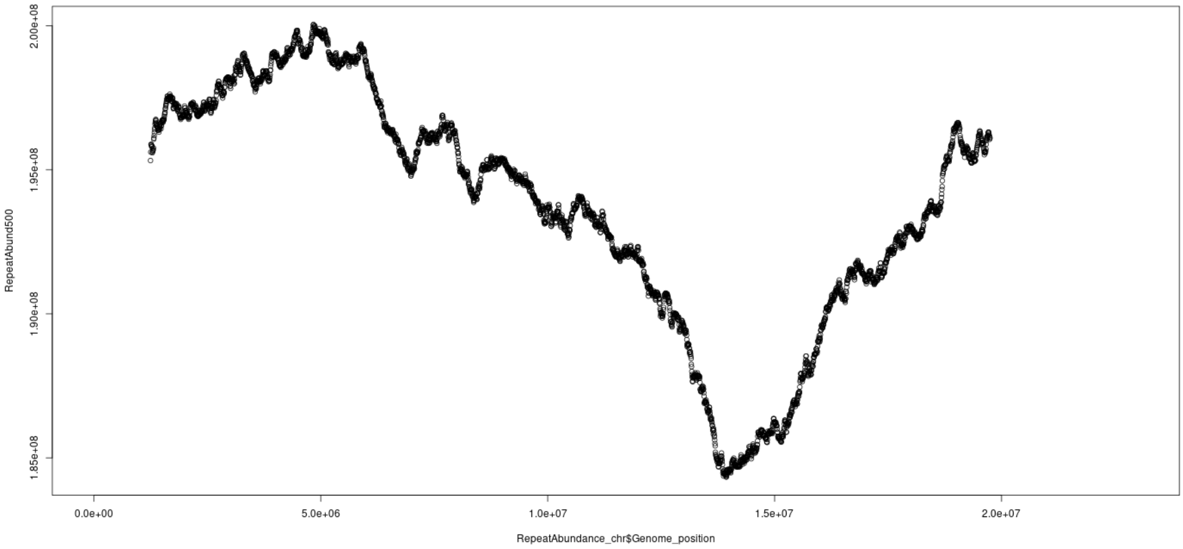

c)

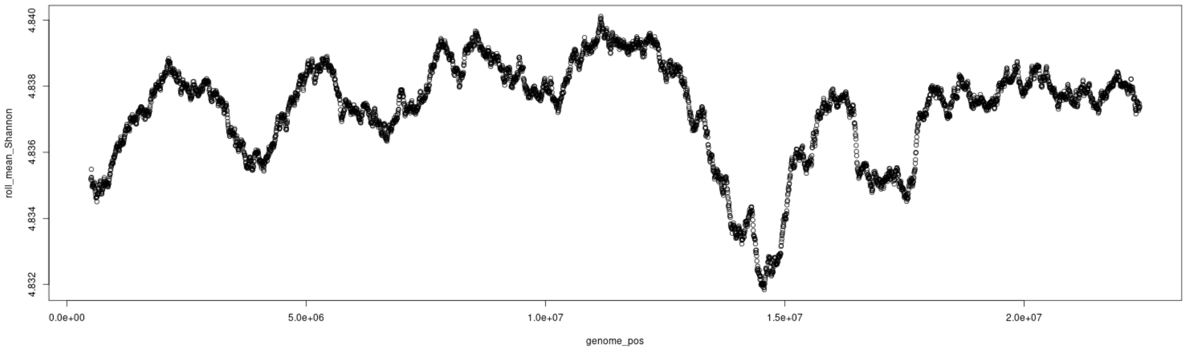

ptg000044l_1

a)

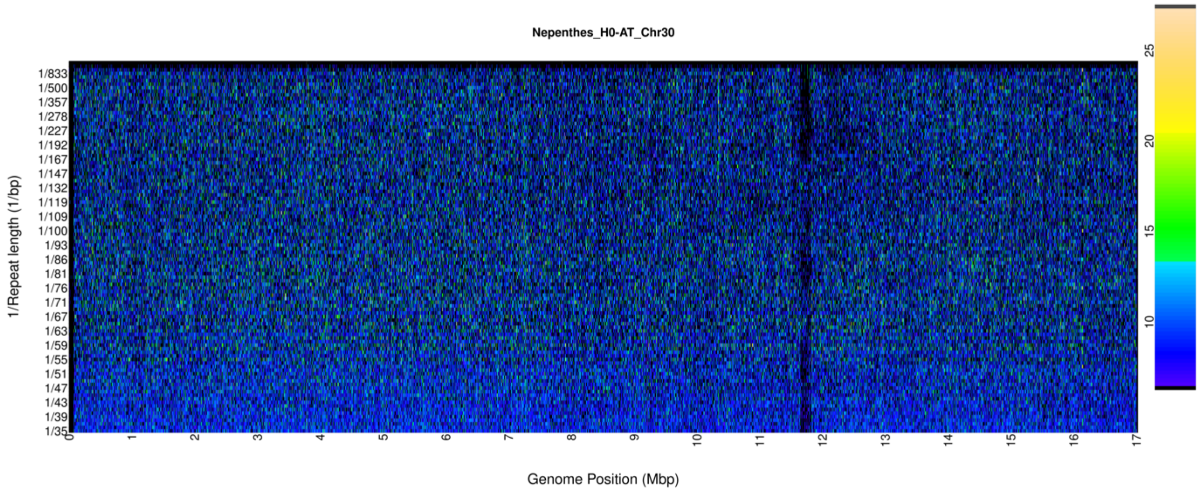

b)

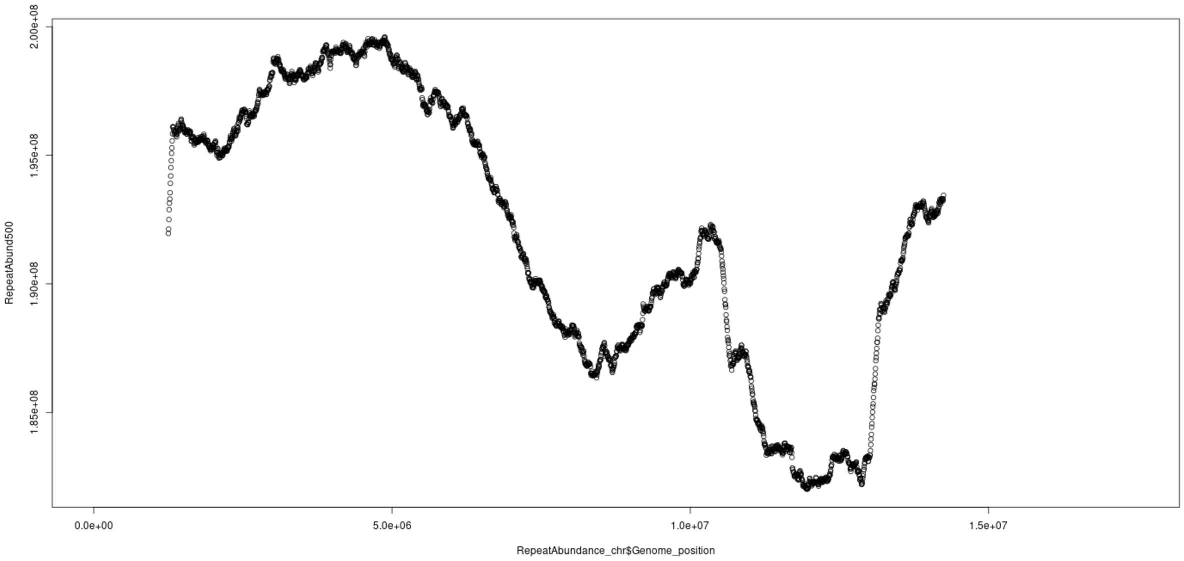

c)

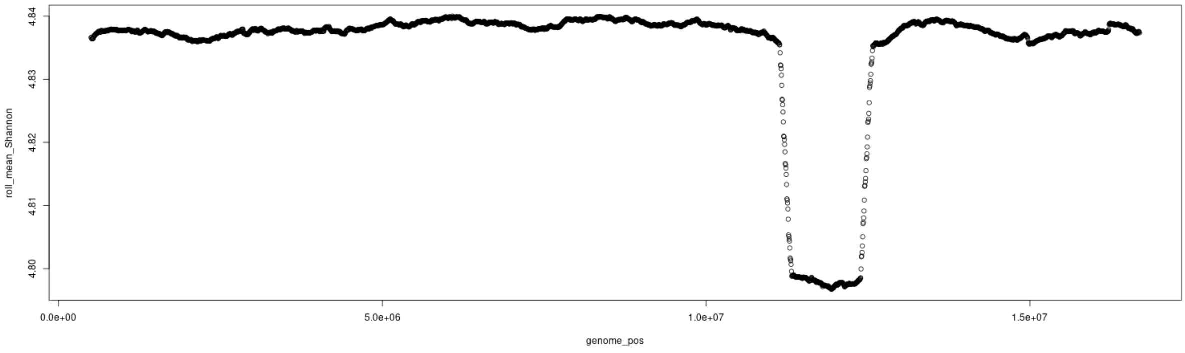

ptg000045l_1

a)

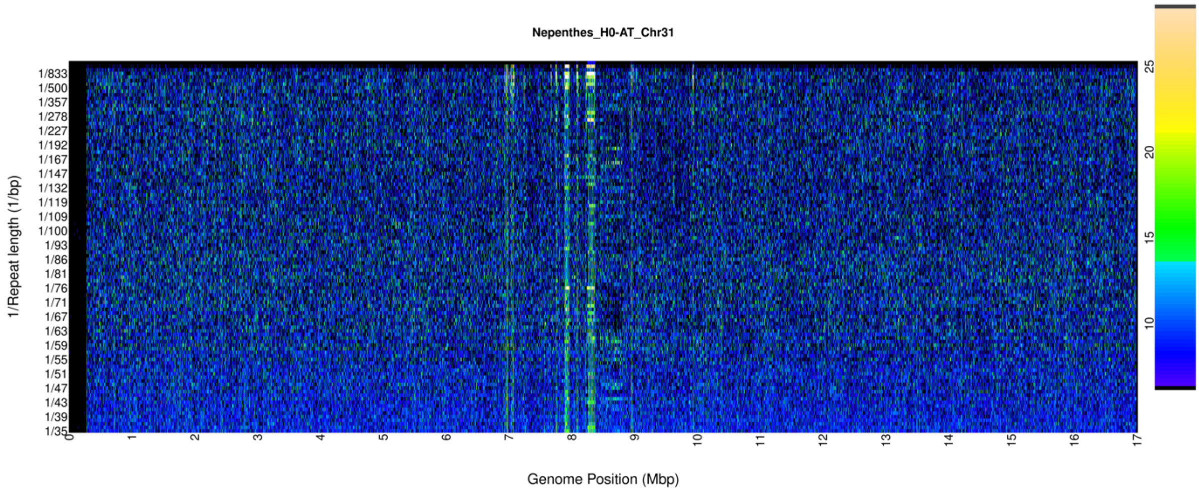

b)

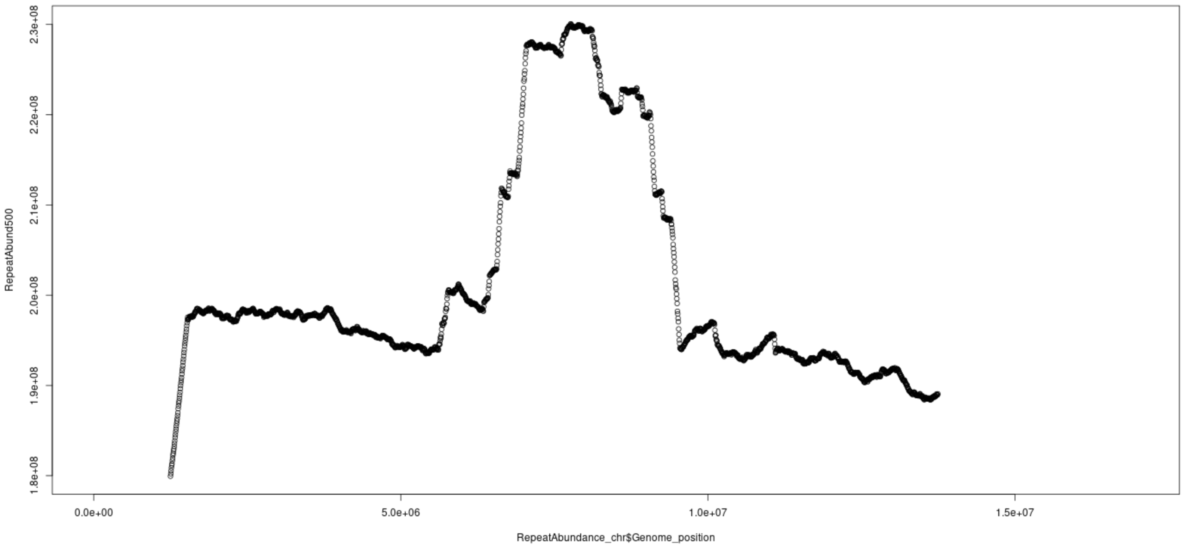

c)

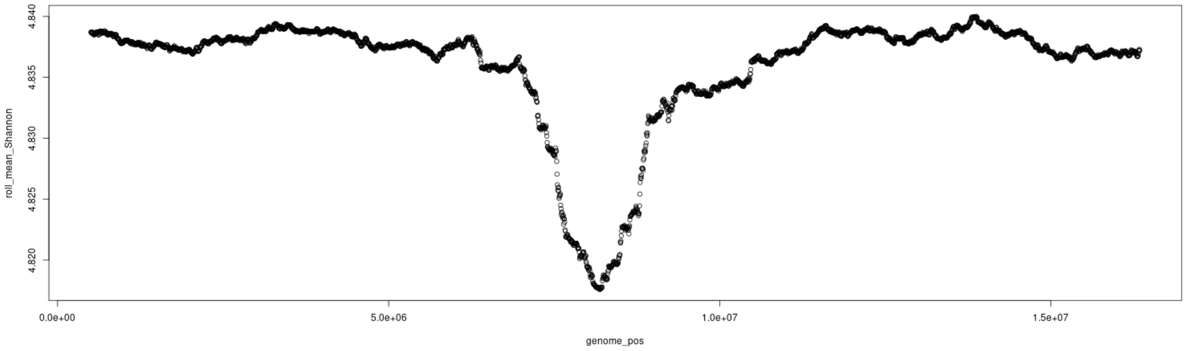

ptg000049l_1

a)

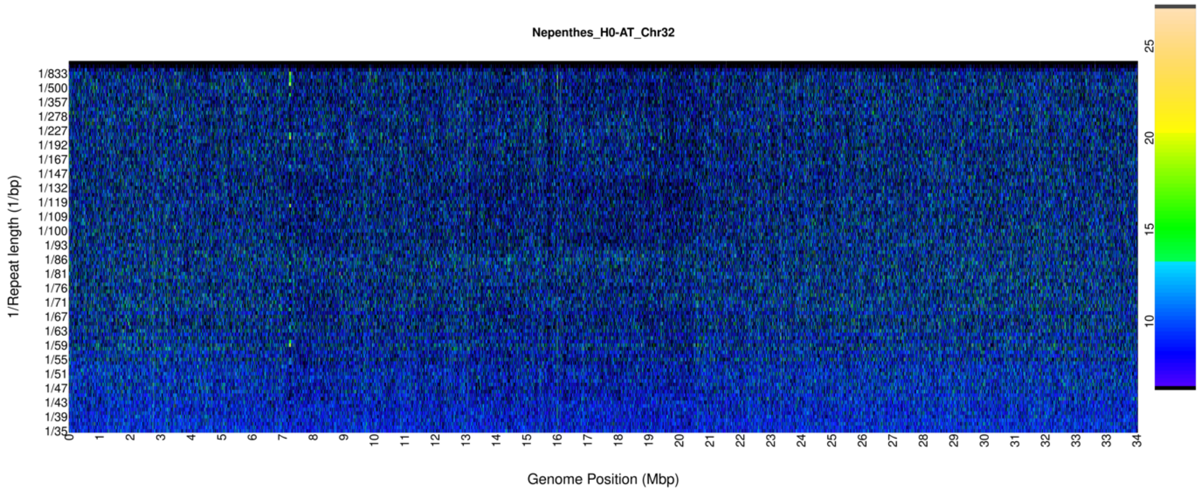

b)

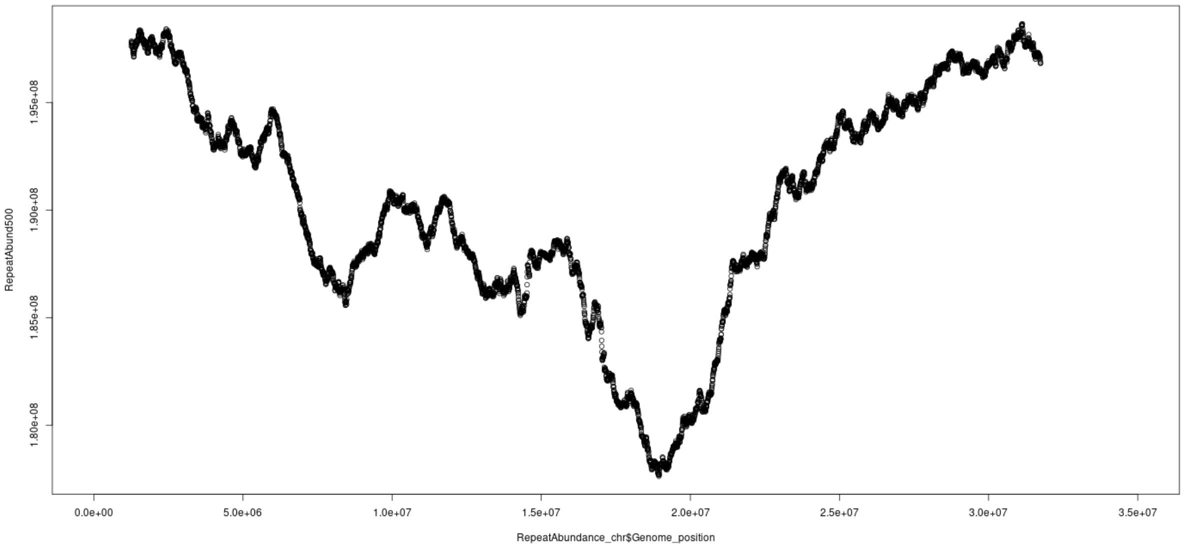

c)

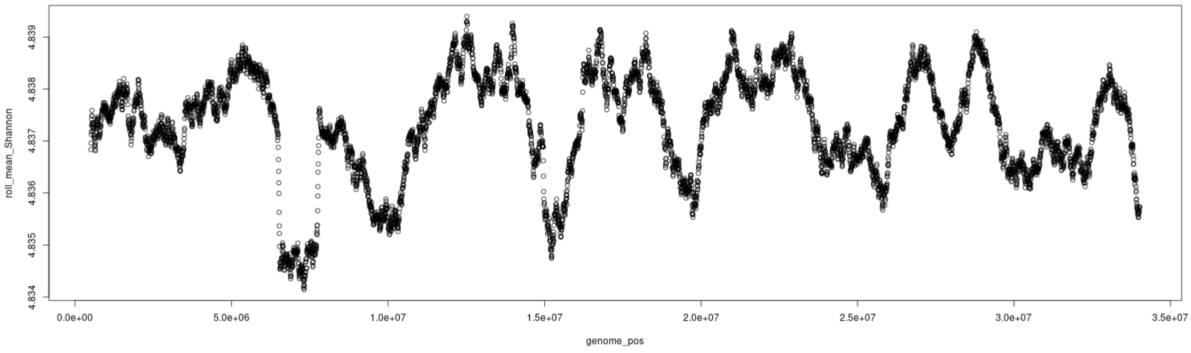

ptg000055l_1

a)

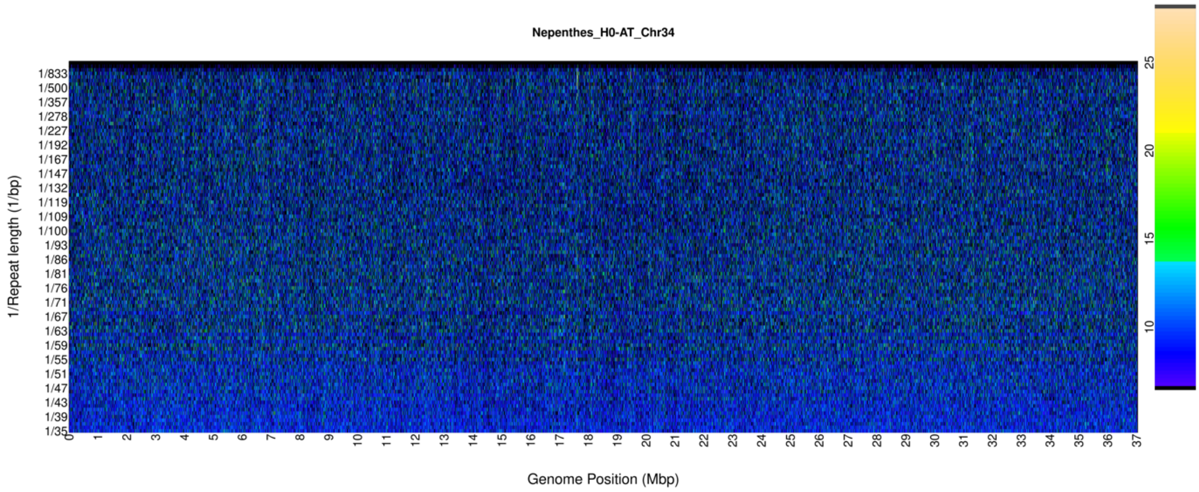

b)

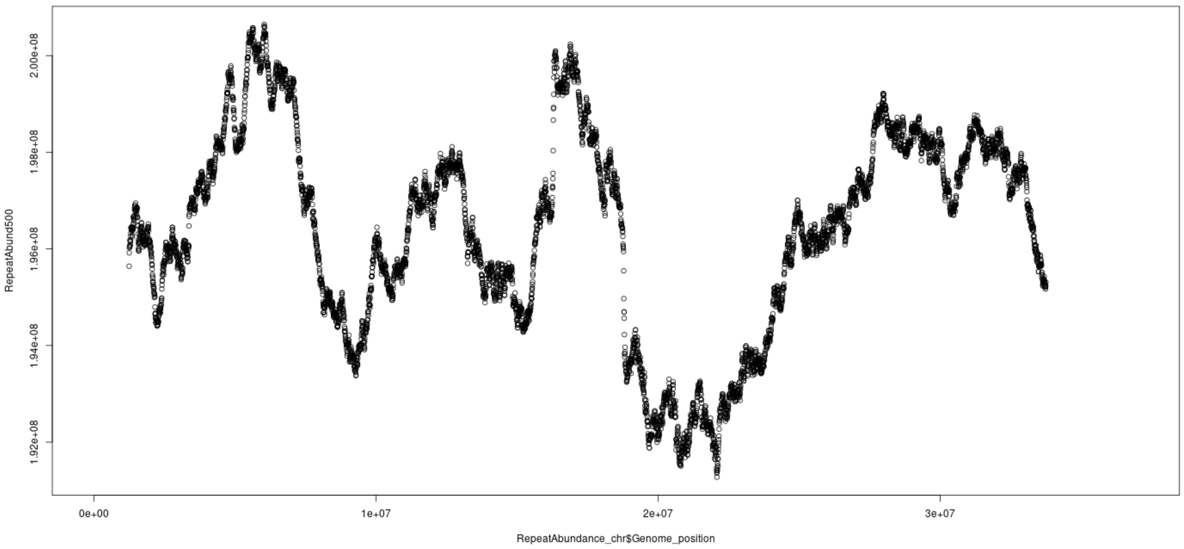

c)

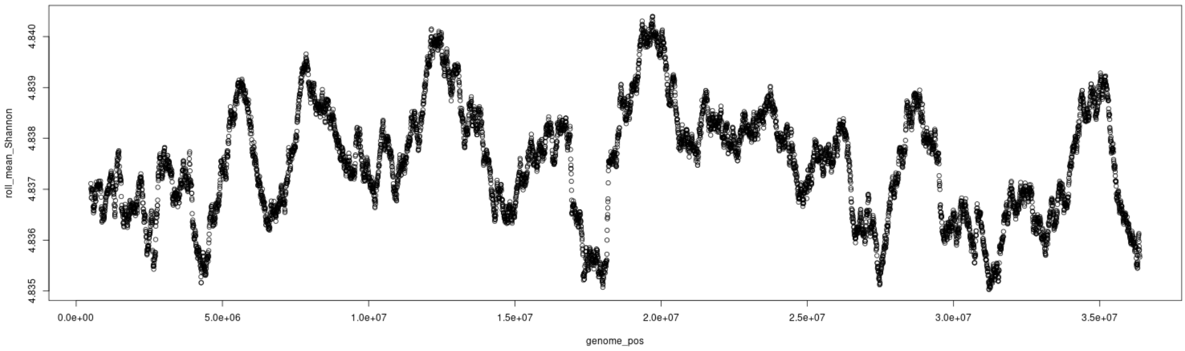

ptg000057l_1

a)

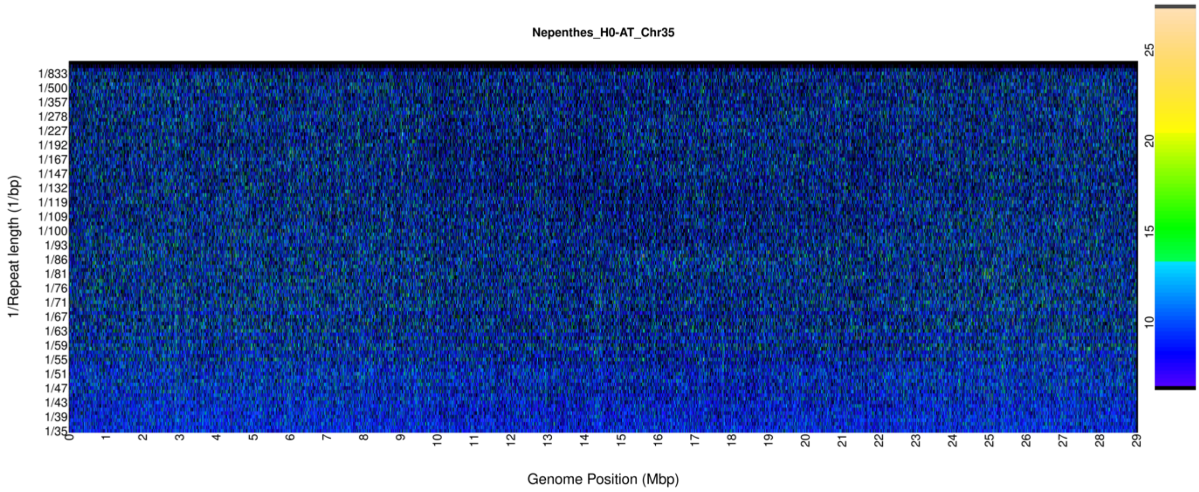

b)

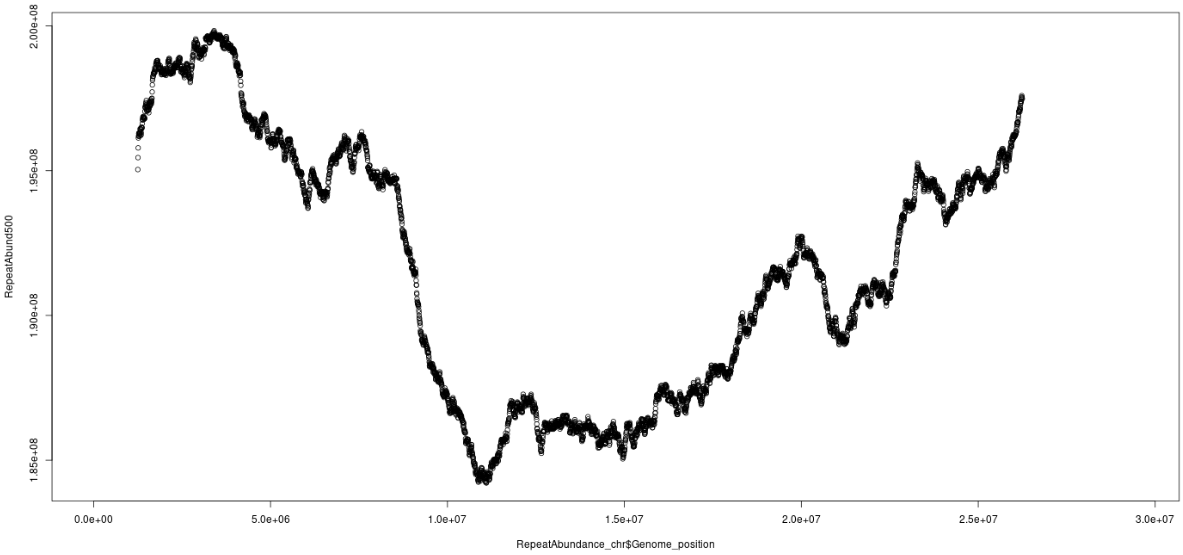

c)

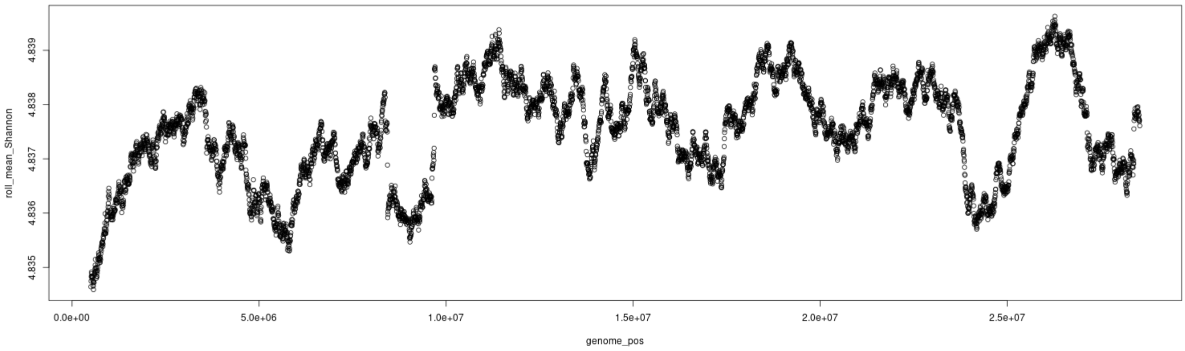

ptg000064l_1

a)

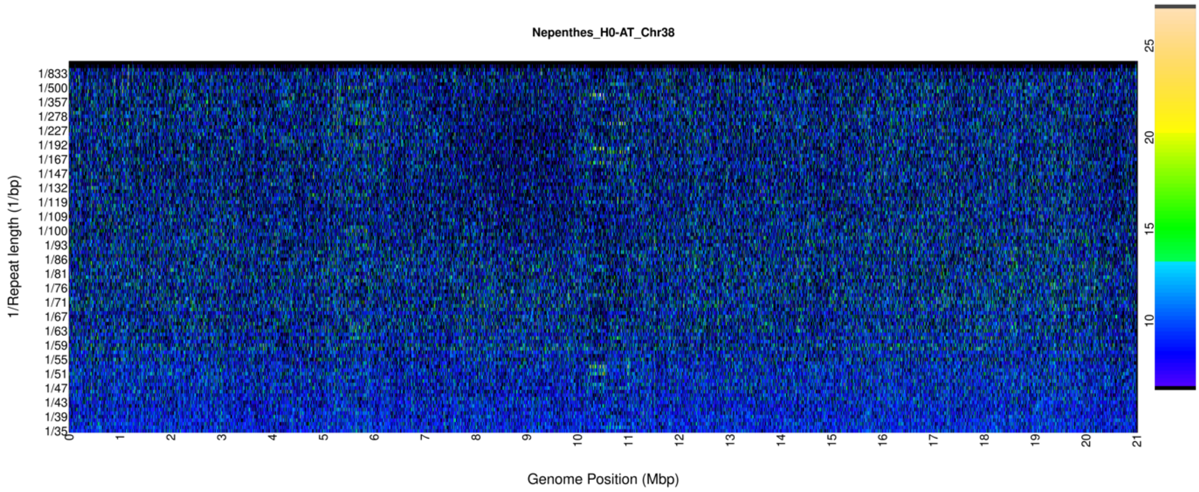

b)

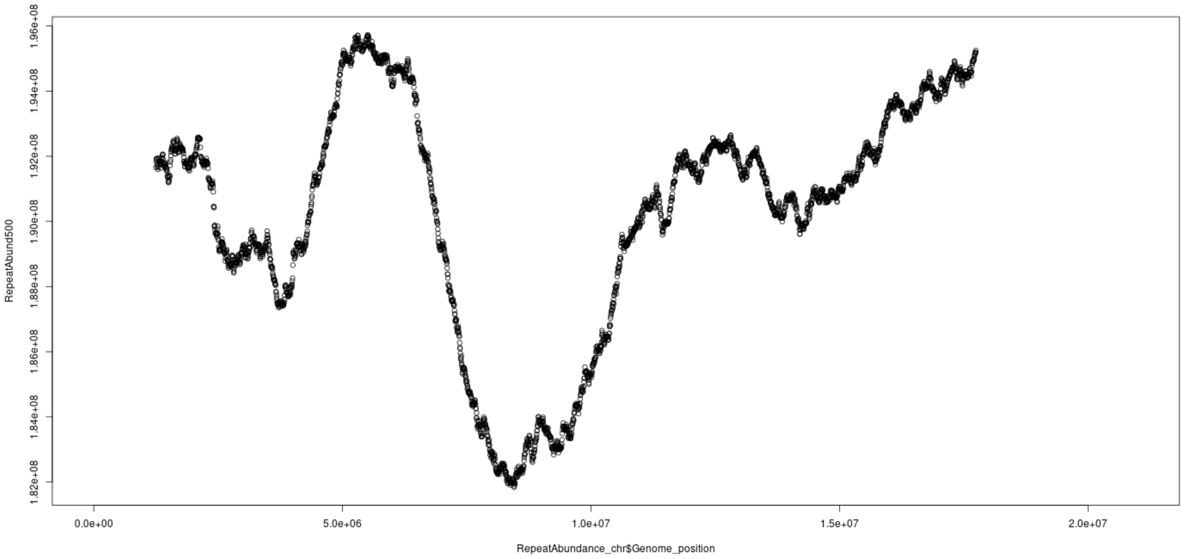

c)

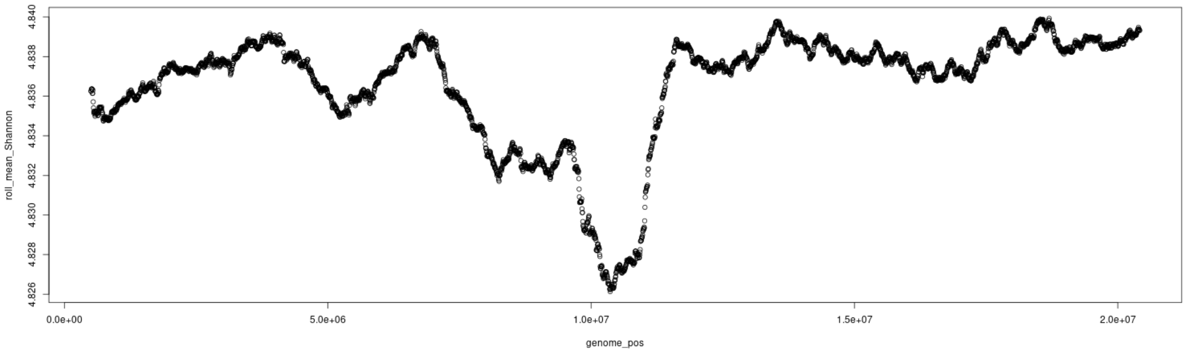

ptg000065l_1

a)

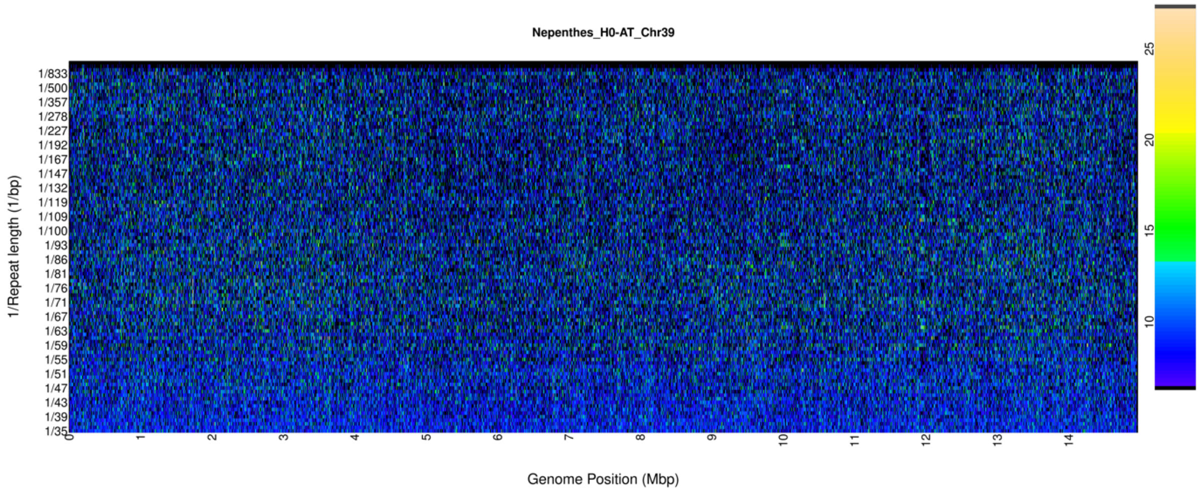

b)

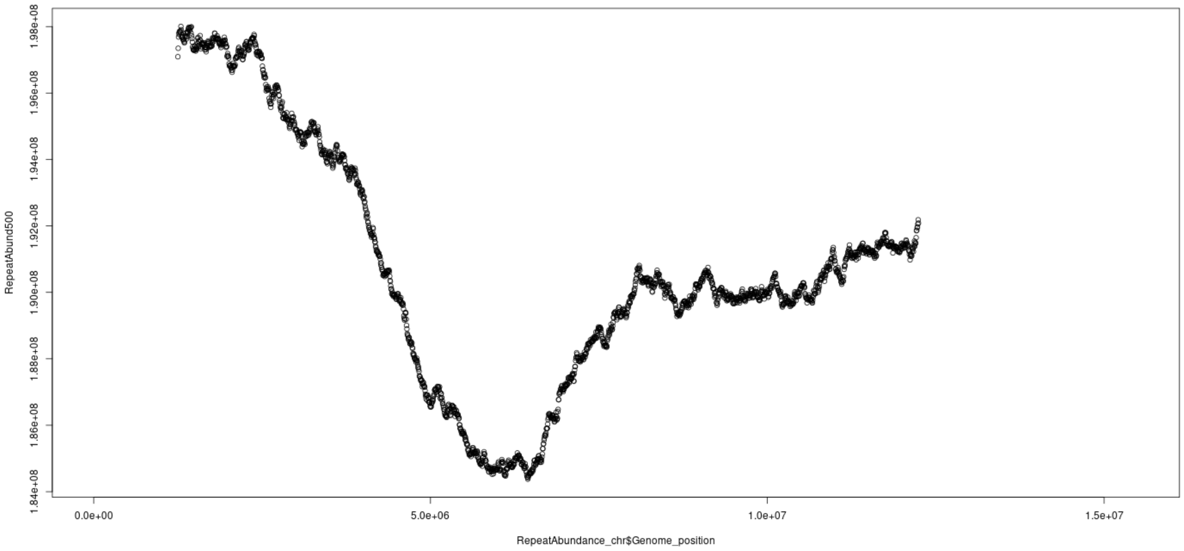

c)

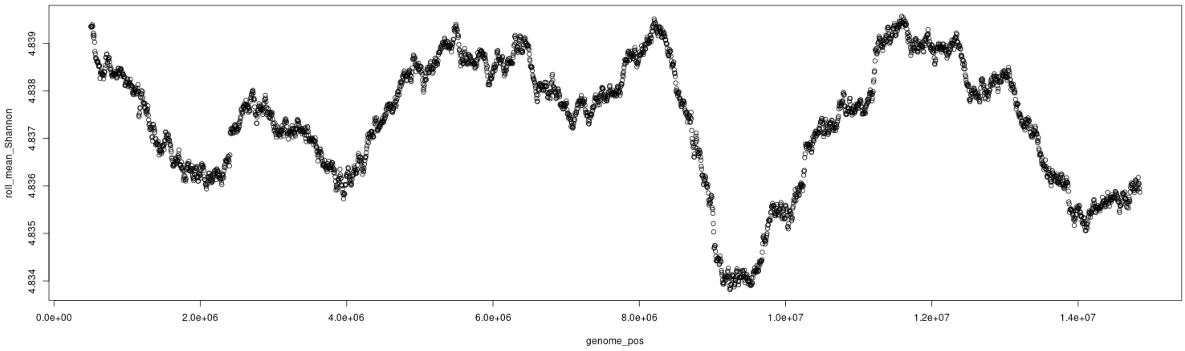

ptg000077l_1

a)

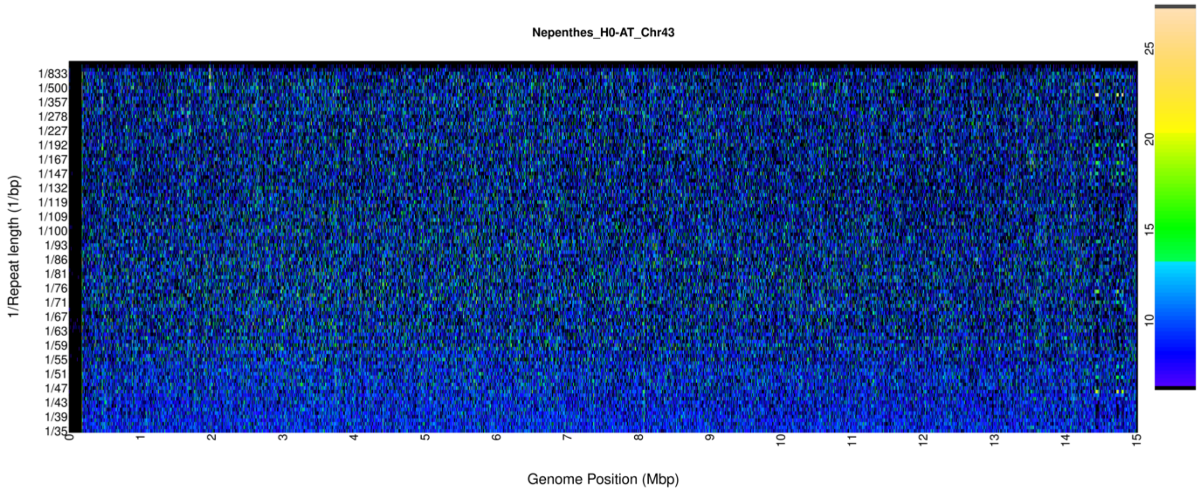

b)

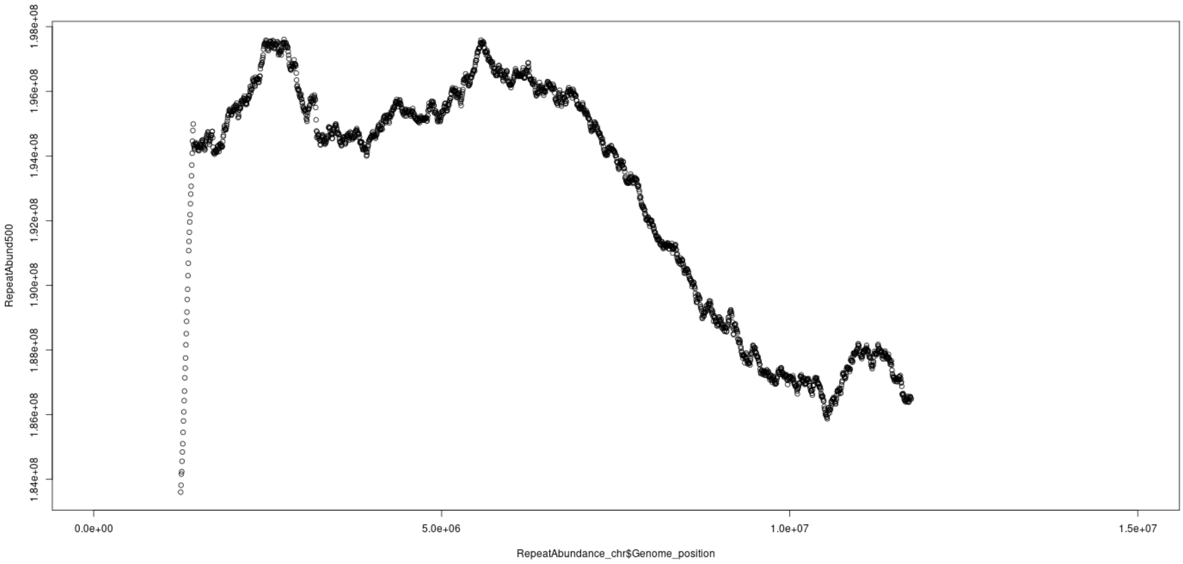

c)

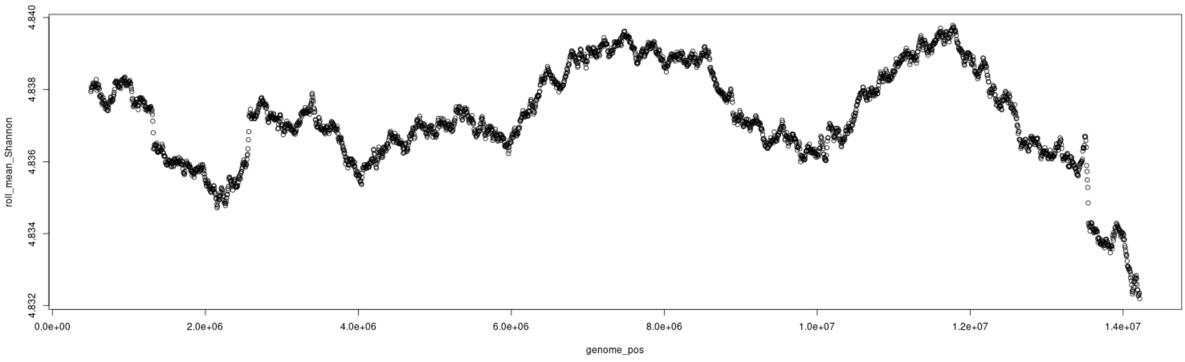

ptg000082l_1

a)

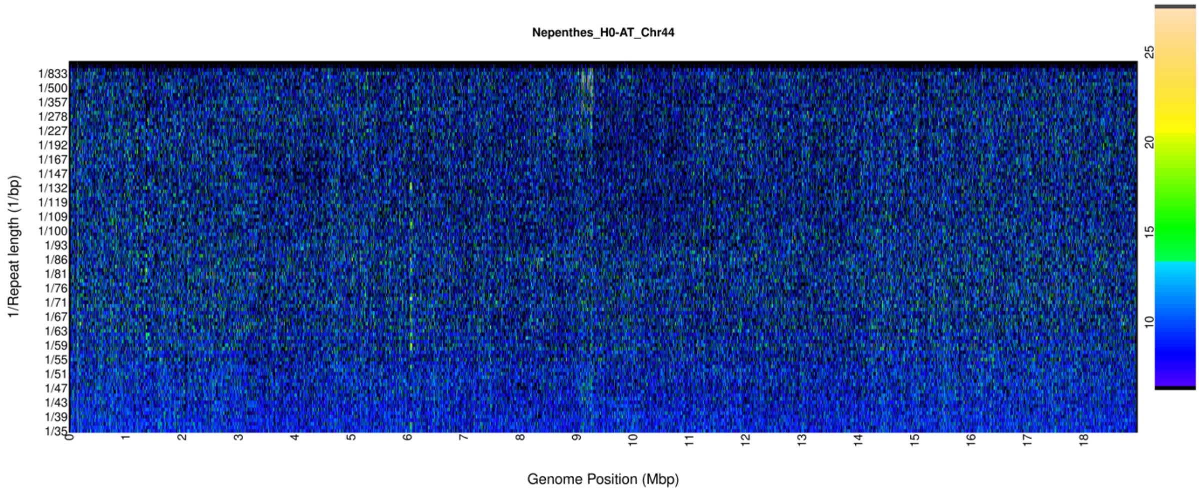

b)

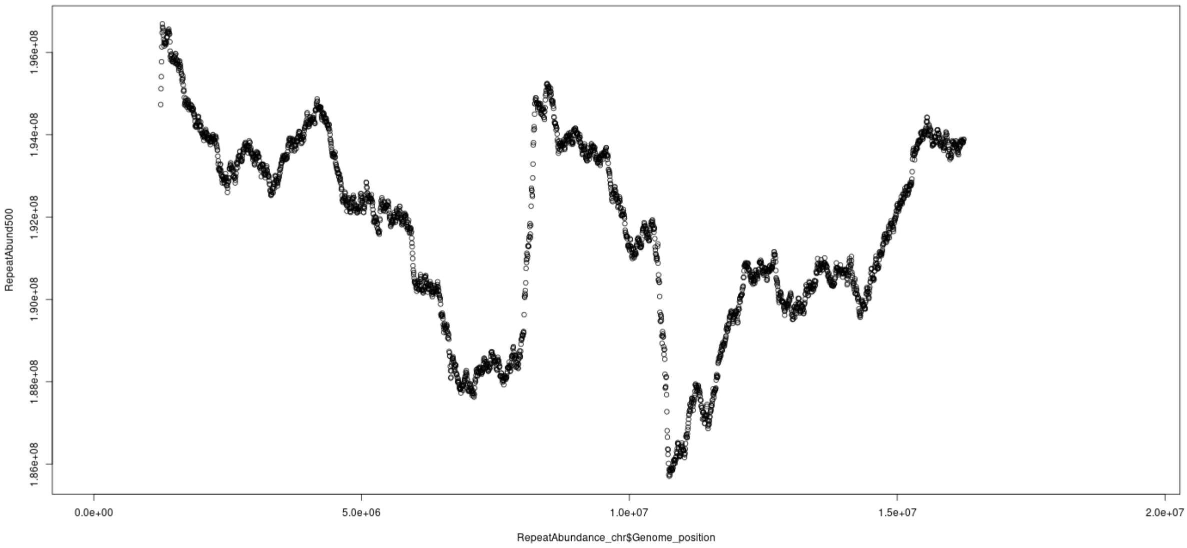

c)

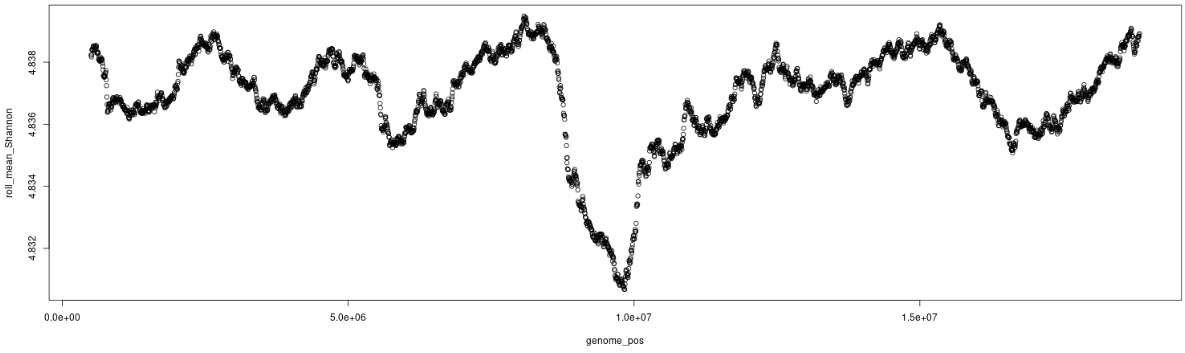

ptg000088l_1

a)

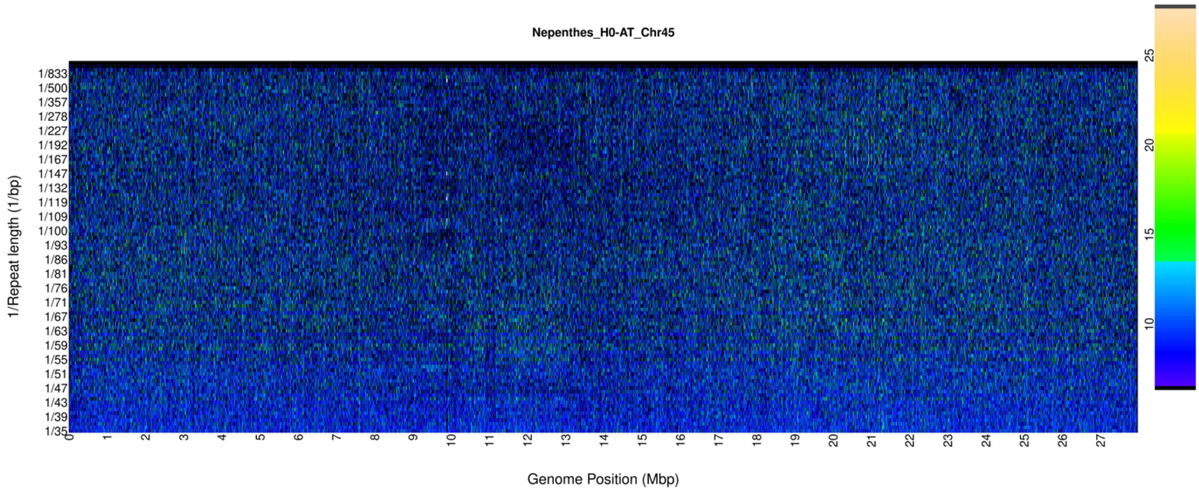

b)

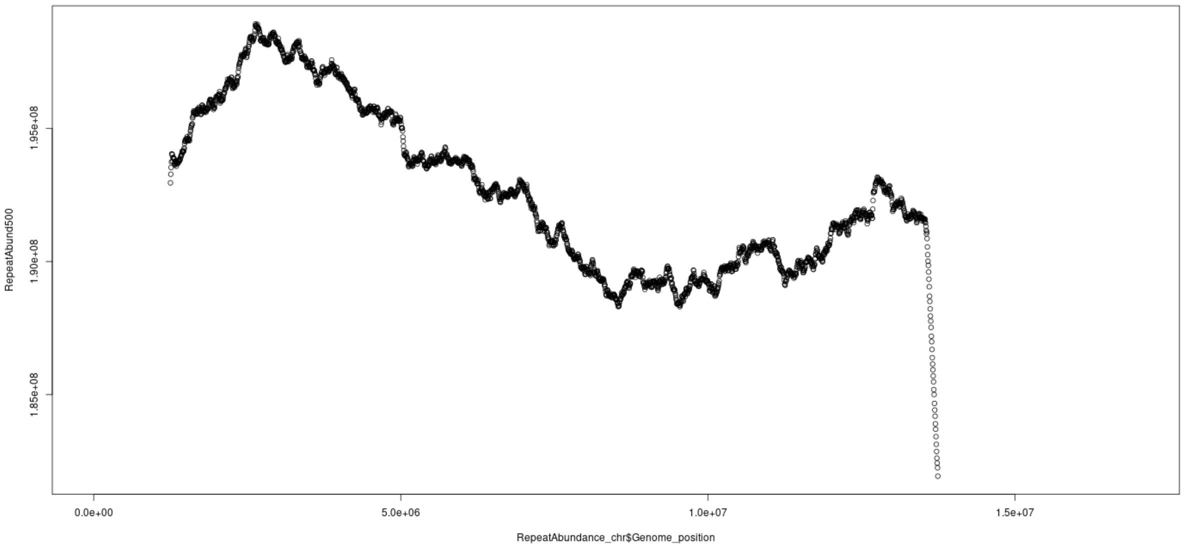

c)

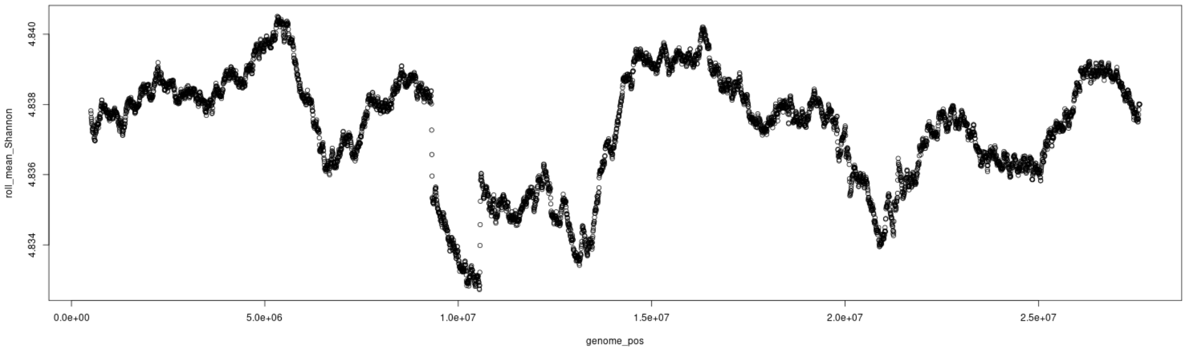

ptg000098l_1

a)

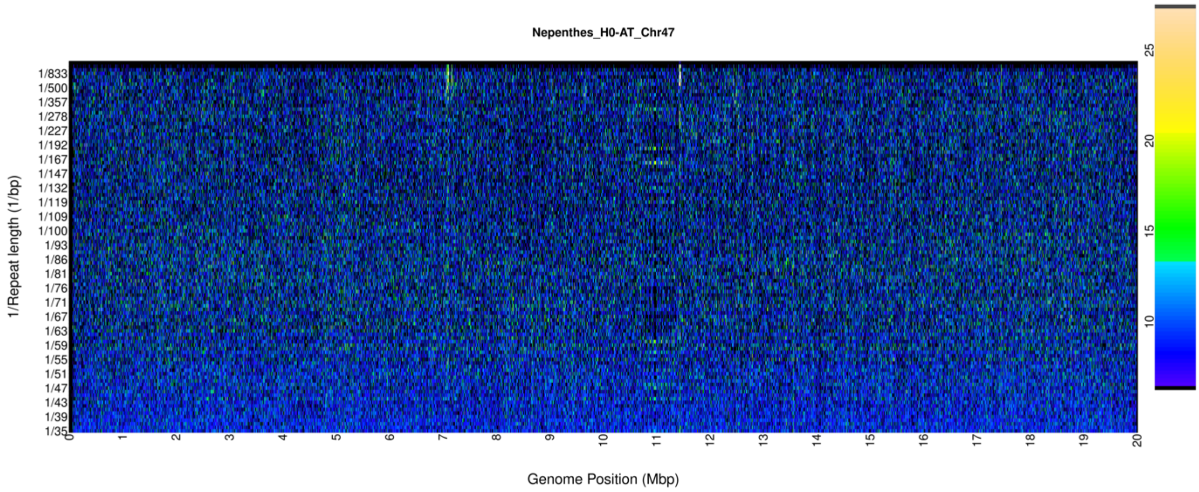

b)

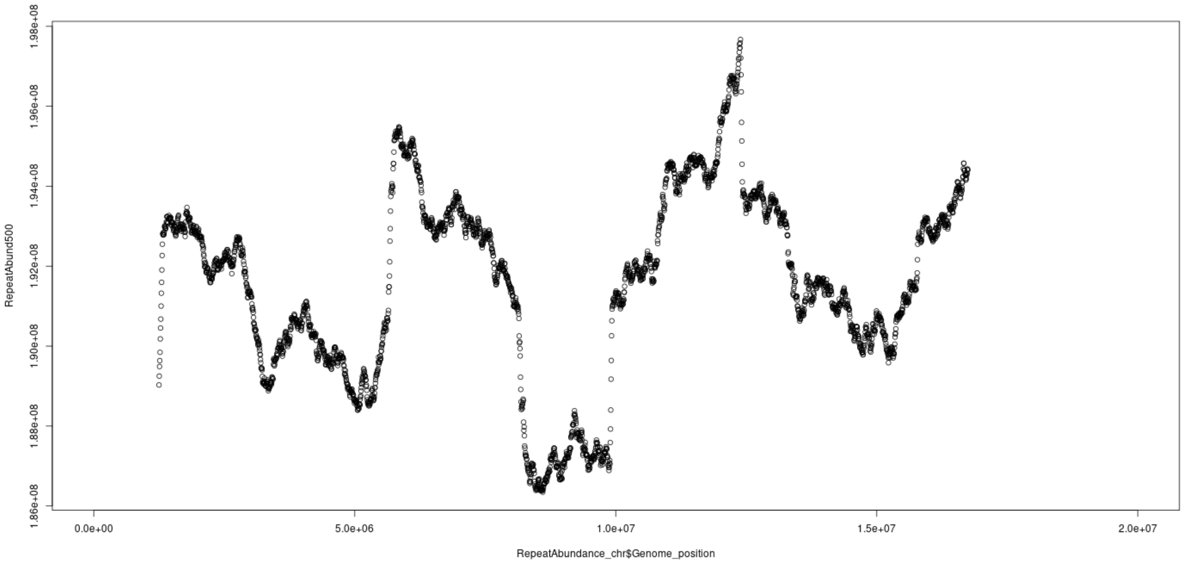

c)

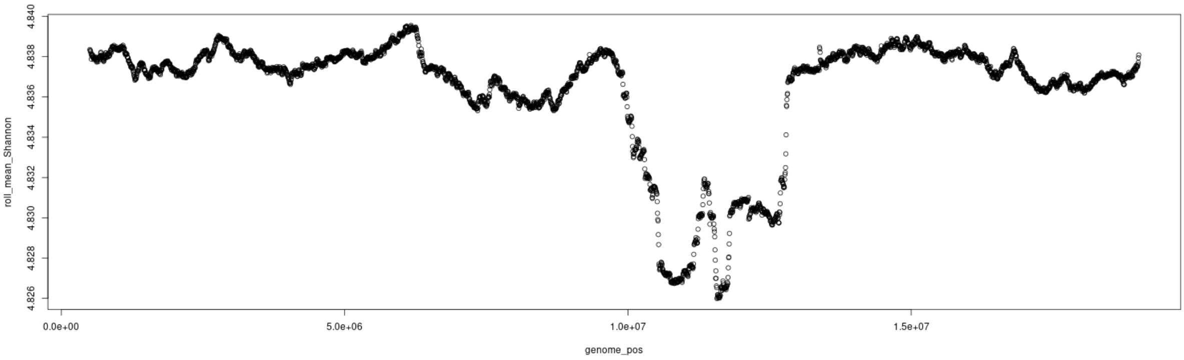

ptg000114l_1

a)

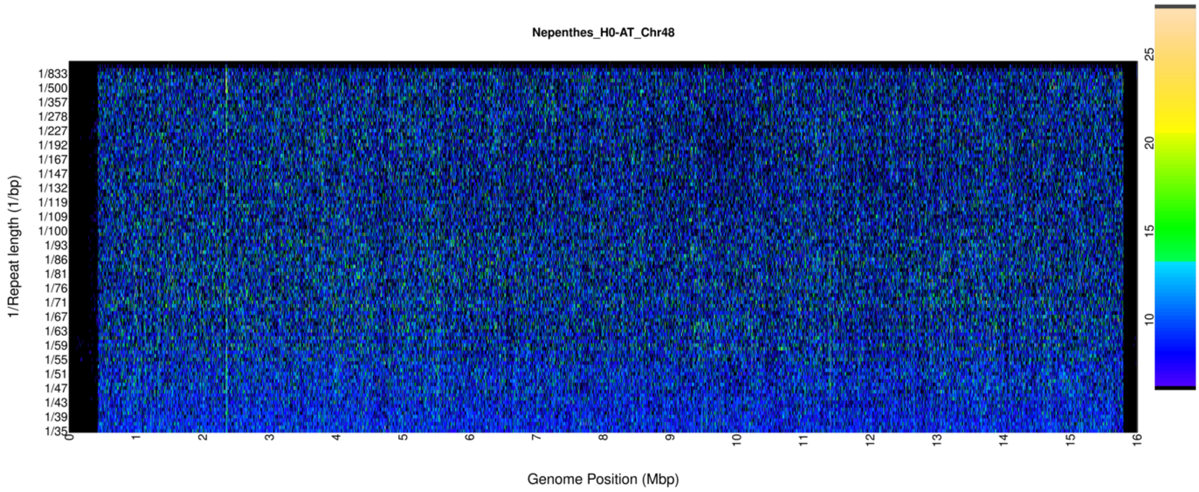

b)

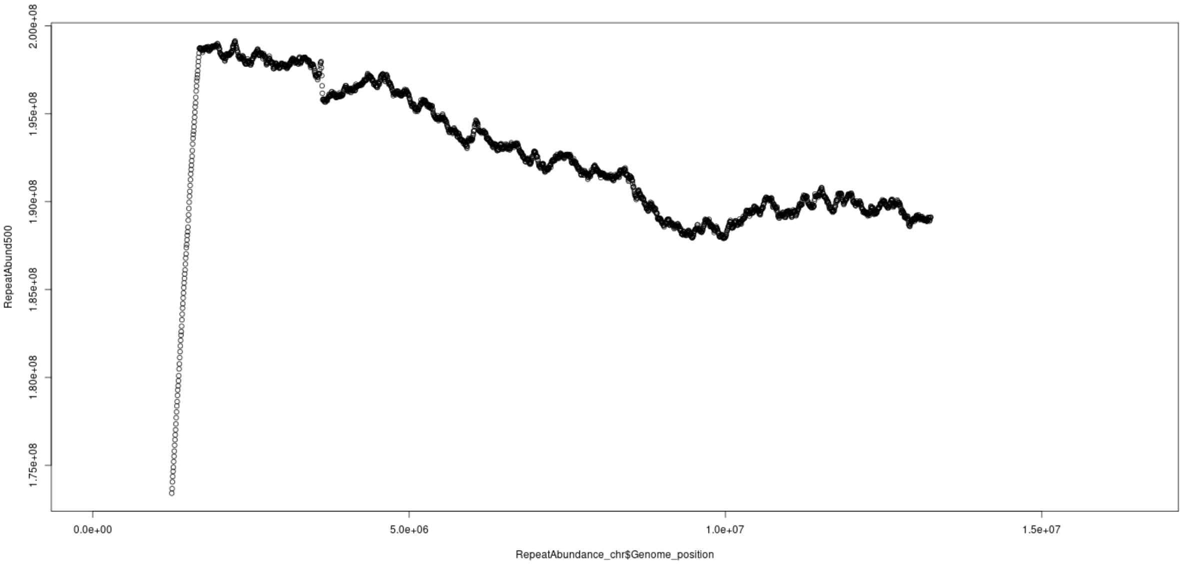

c)

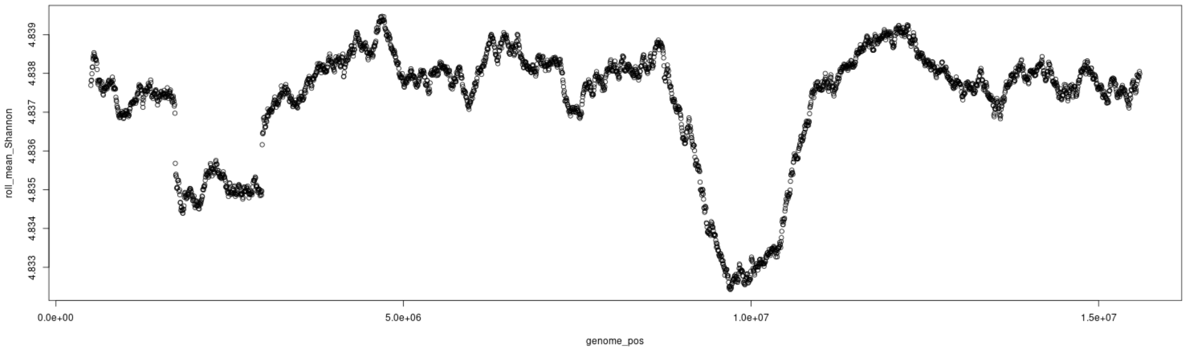

ptg000118l_1

a)

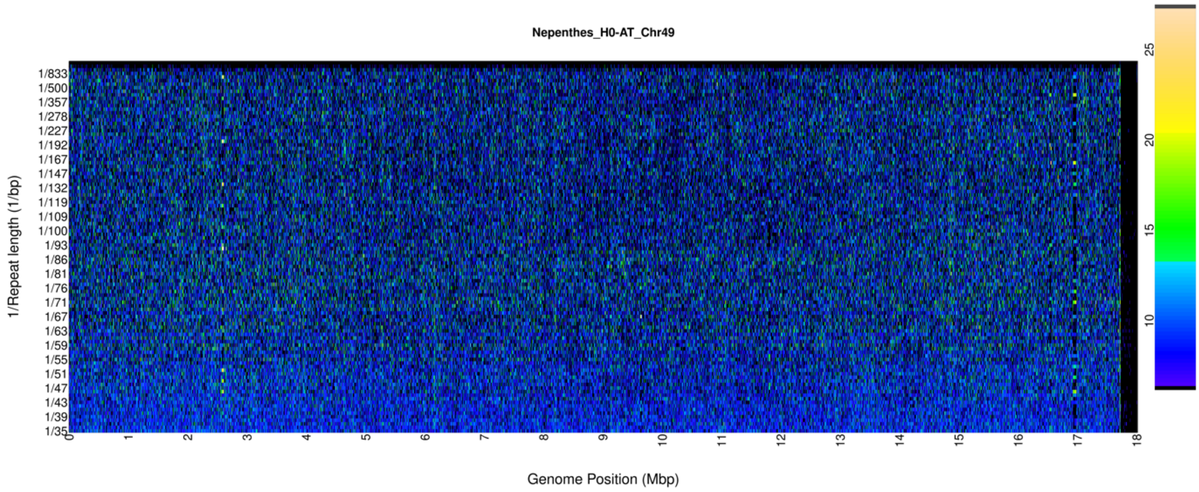

b)

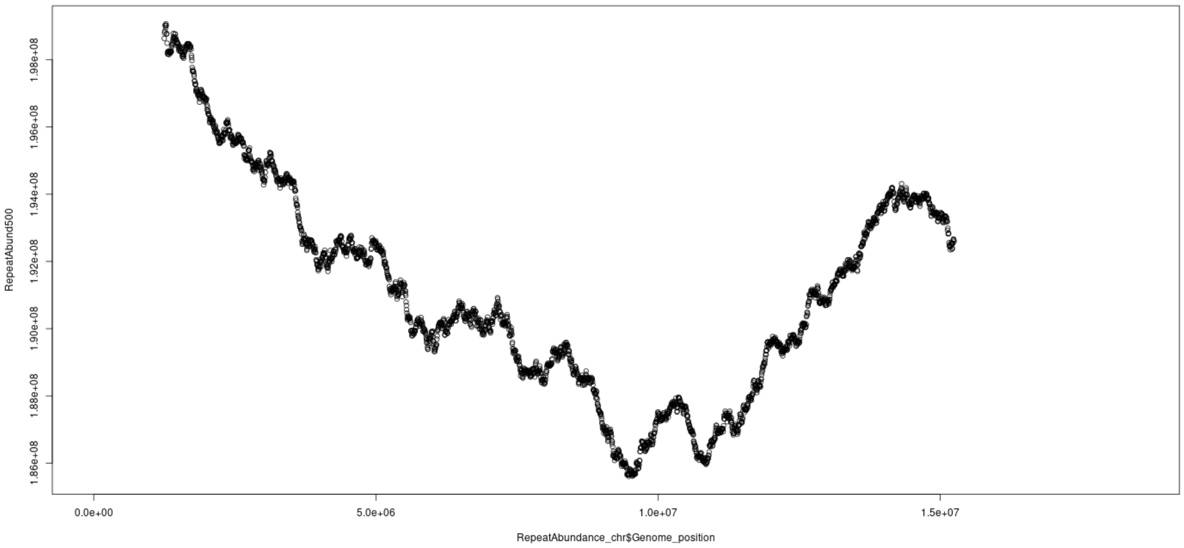

c)

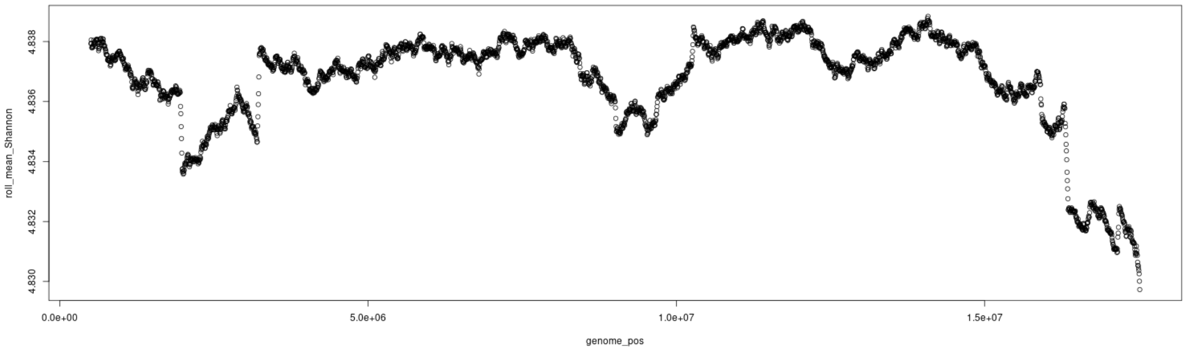

ptg000001l_1 (left contig, shown in reverse complement relative to plots in File S5)

a)

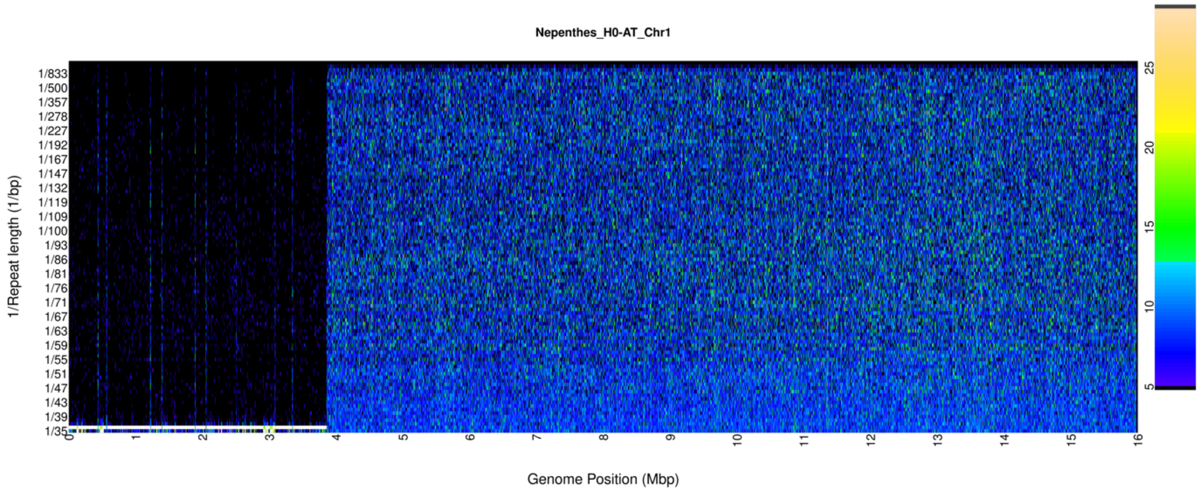

b)

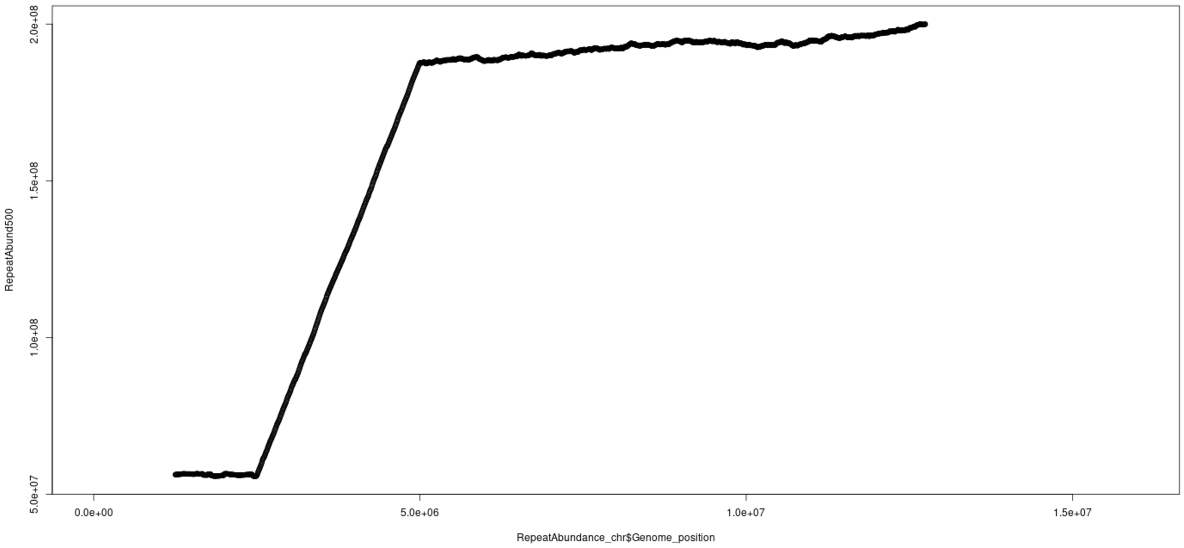

c)

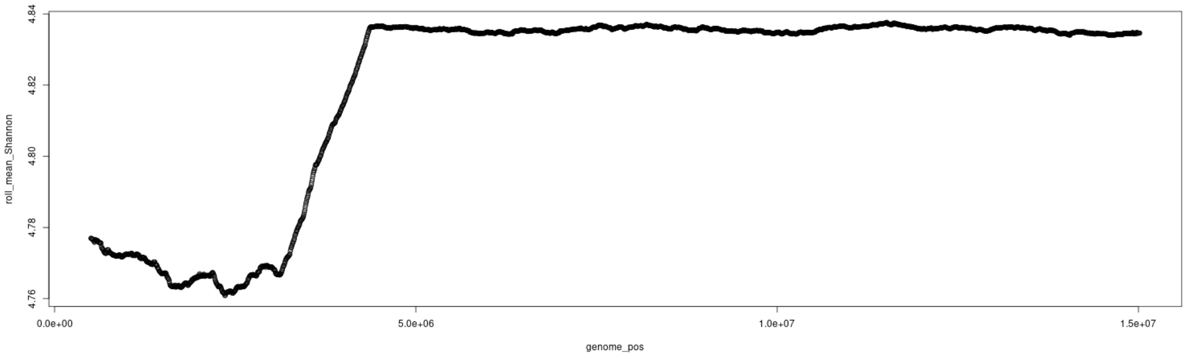

ptg000058l_1 (right contig, shown in reverse complement relative to plots in File S5)

a)

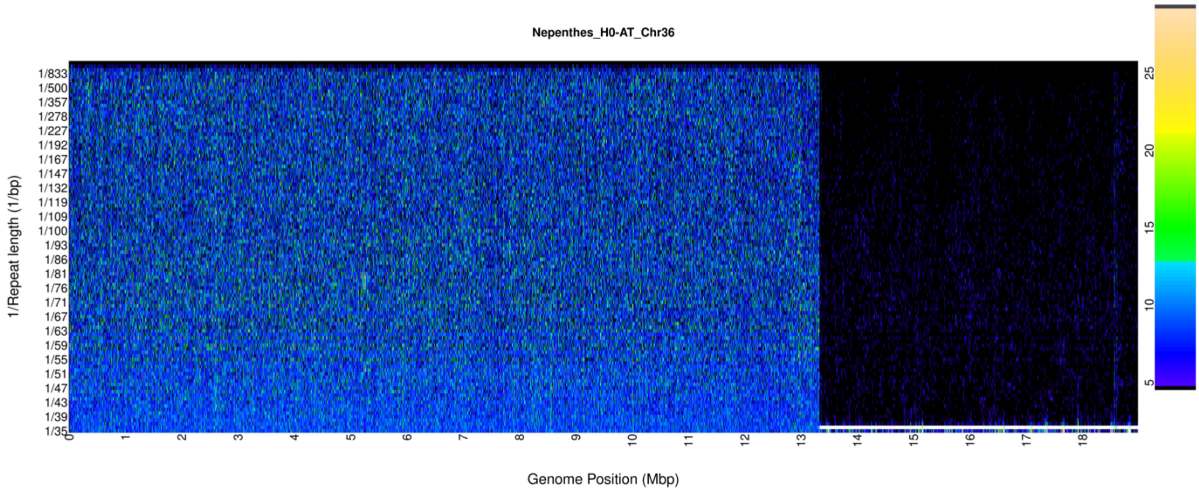

b)

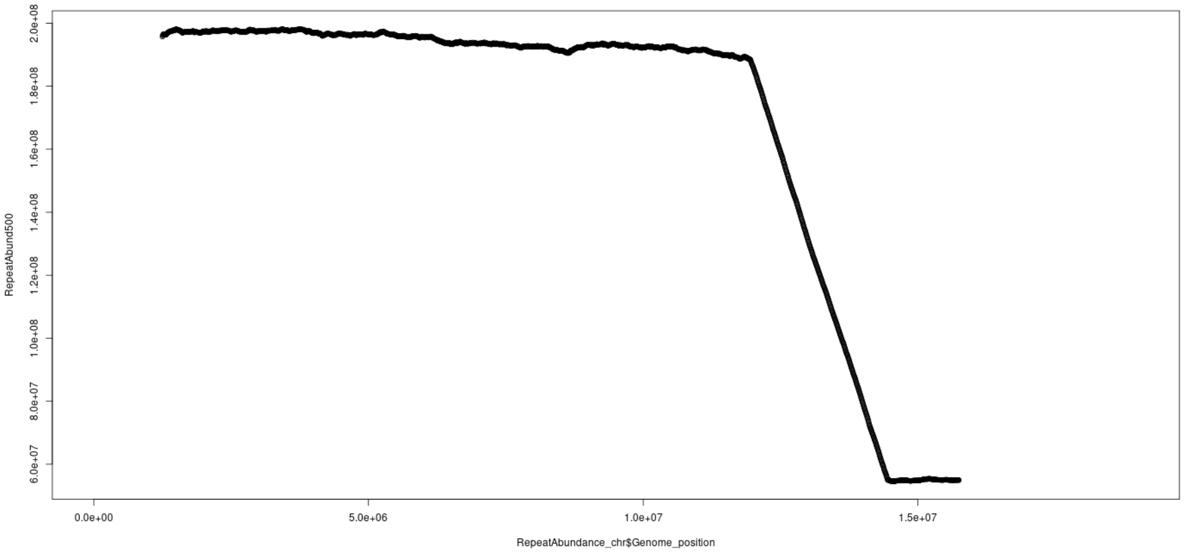

c)

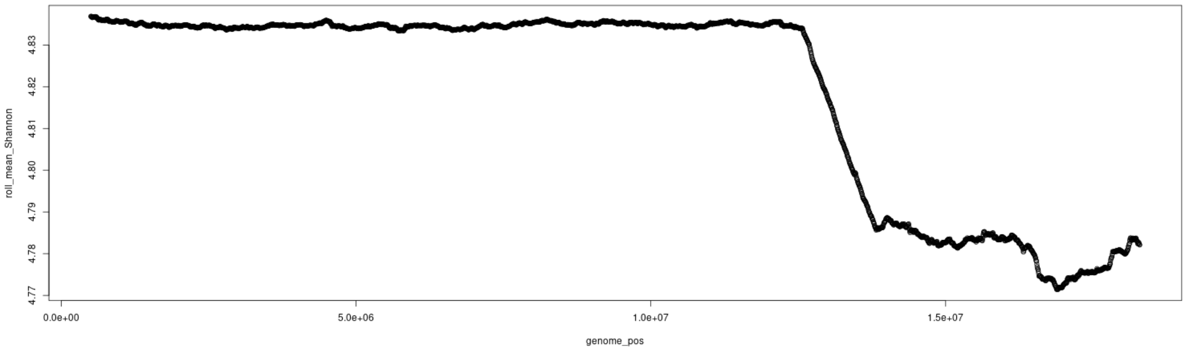

ptg000025l_1 (left contig, shown in reverse complement relative to plots in File S5)

a)

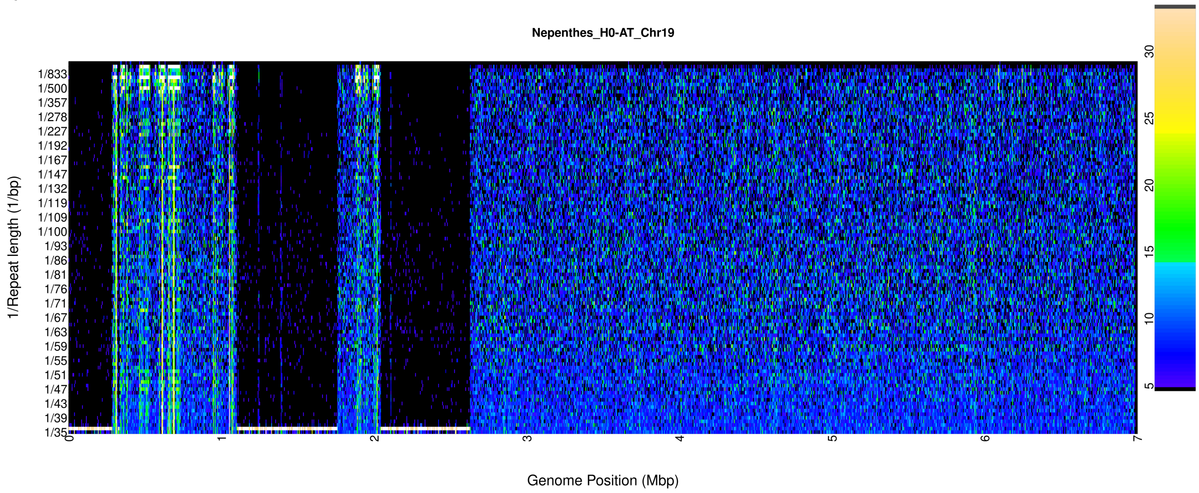

b)

Plot not produced by RepeatOBserverV1 software.

c)

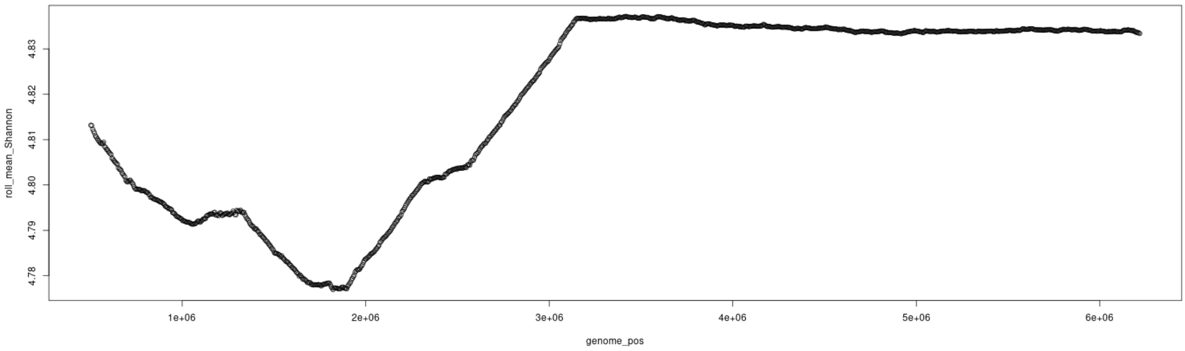

ptg000021l_1 (right contig, shown in reverse complement relative to plots in File S5)

a)

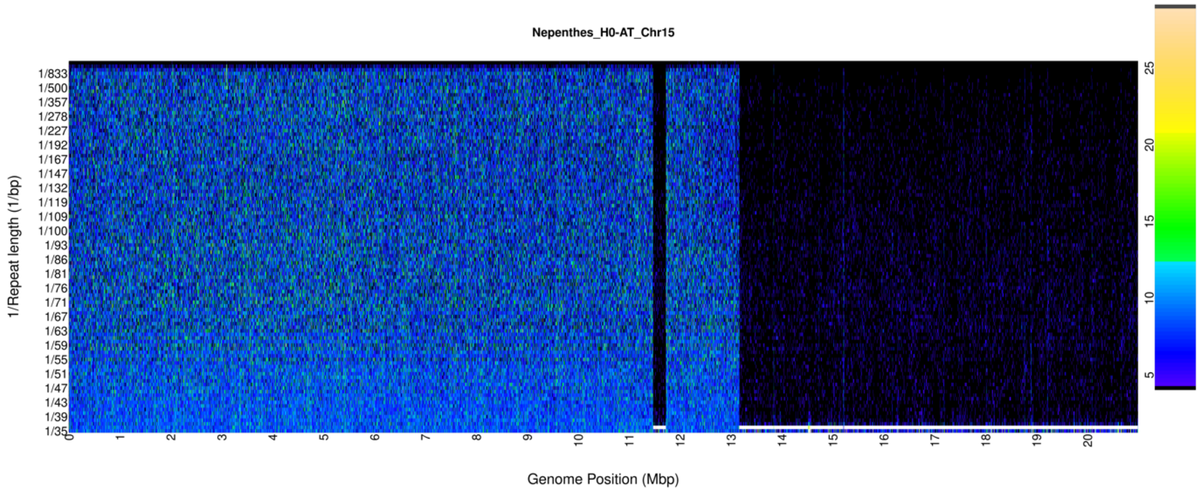

b)

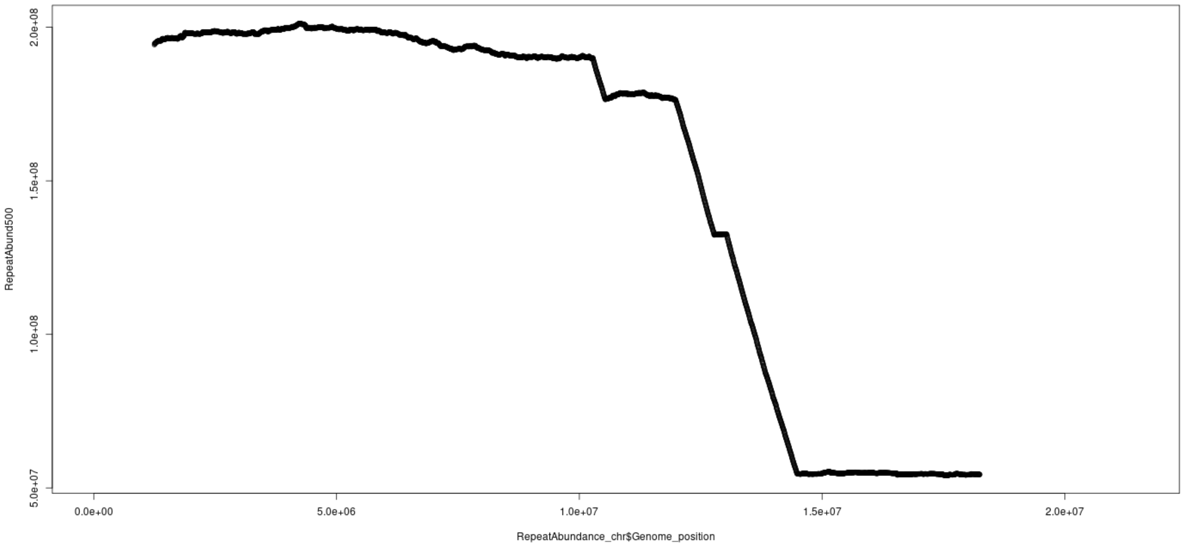

c)

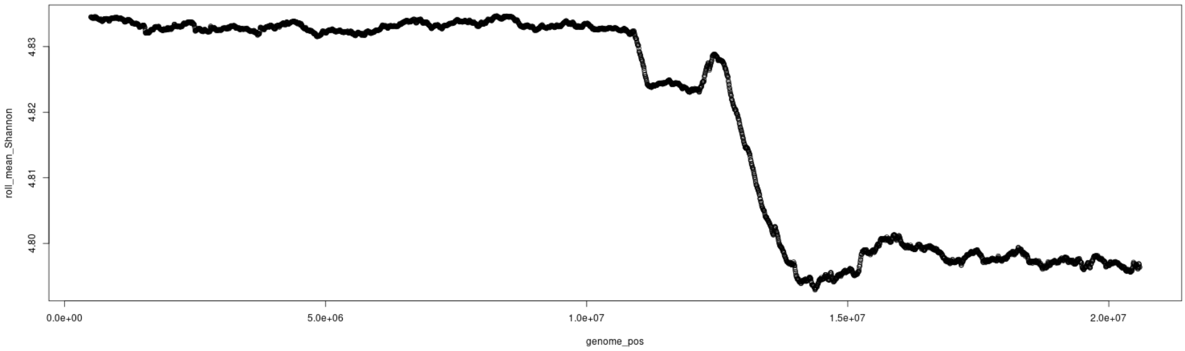

ptg000042l_1 (left contig, shown in reverse complement relative to plots in File S5)

a)

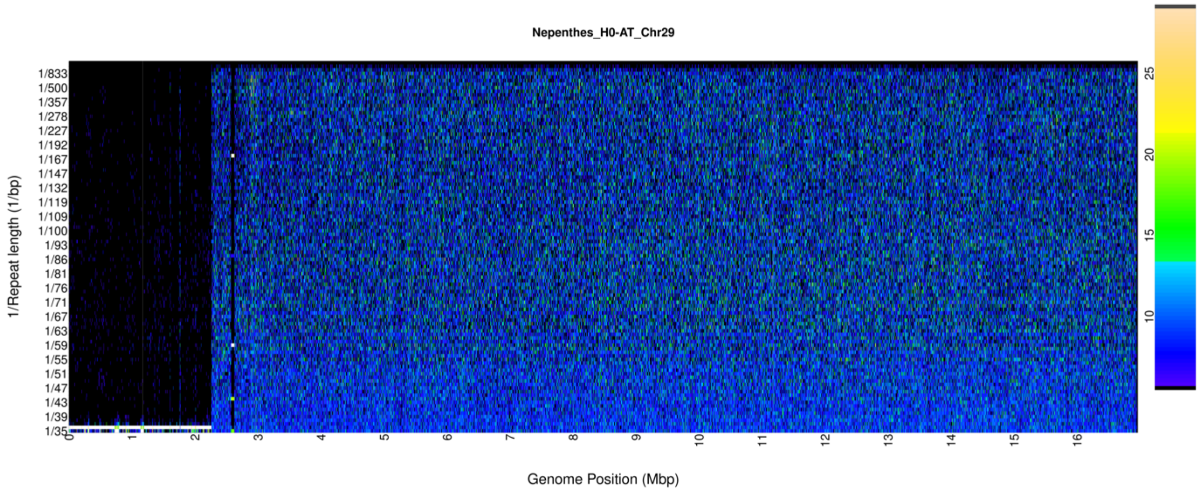

b)

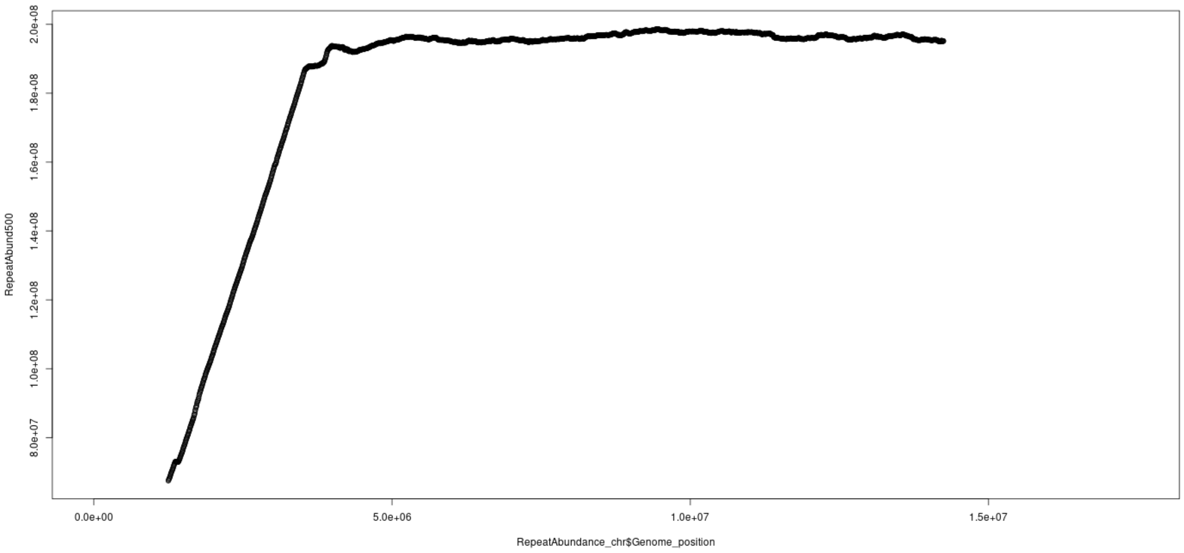

c)

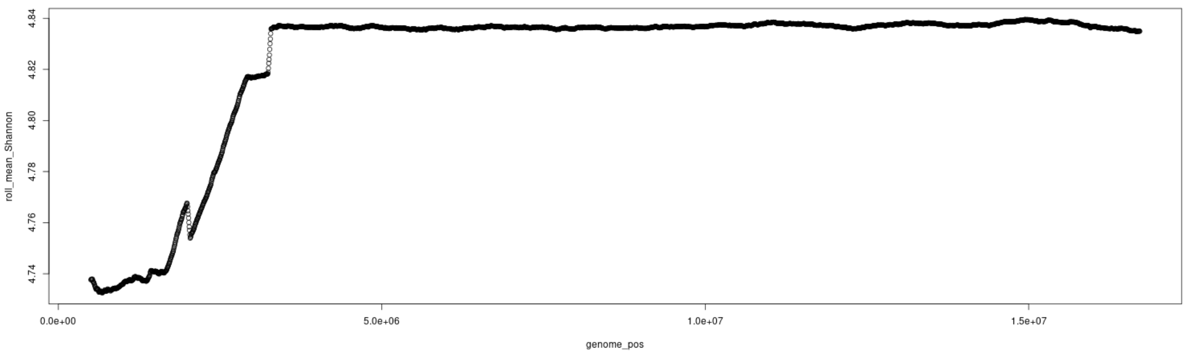

ptg000027l_1 (right contig, shown in reverse complement relative to plots in File S5)

a)

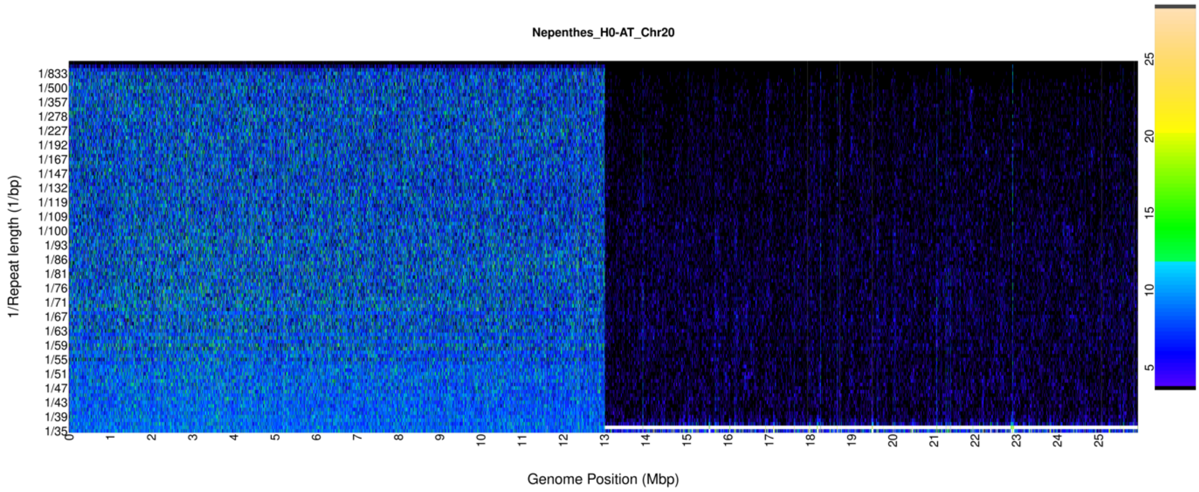

b)

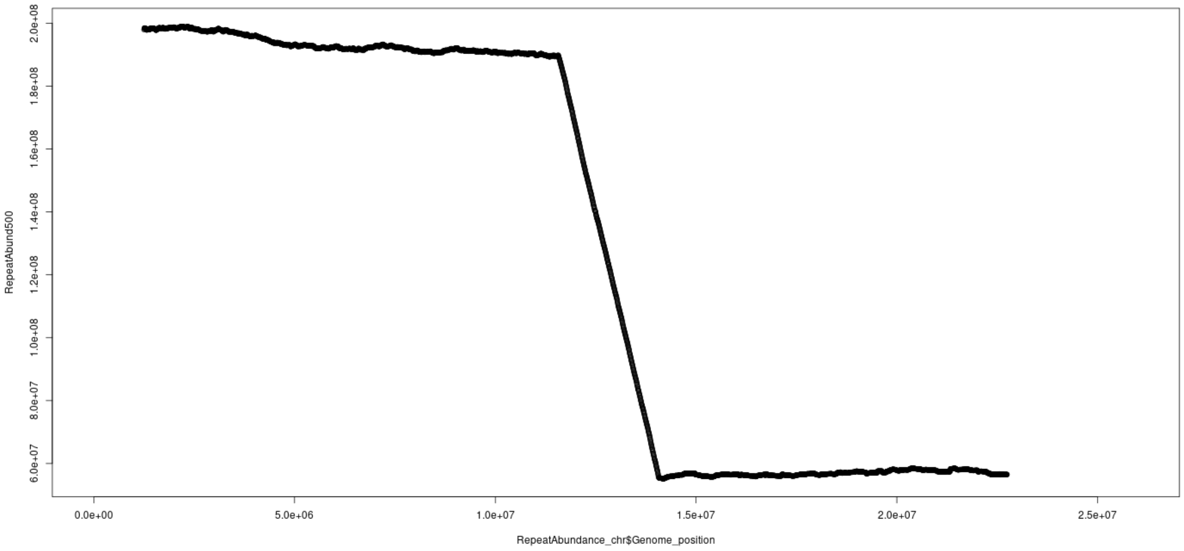

c)

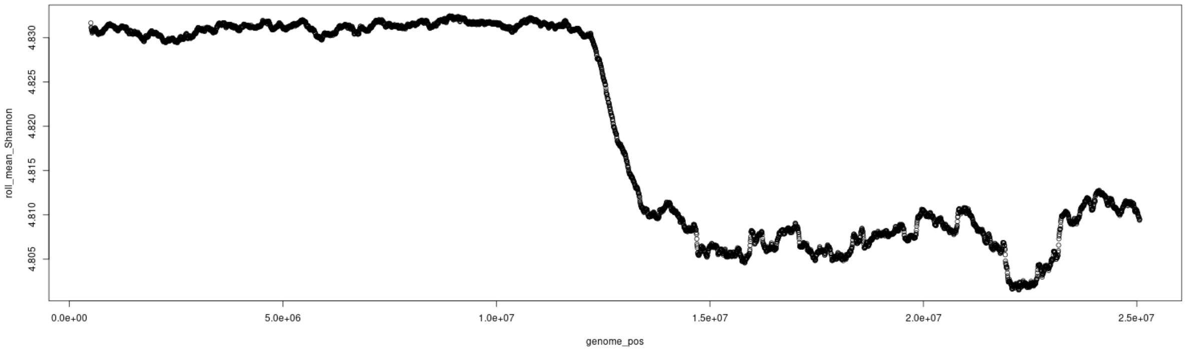

ptg000033l_1 (left contig, shown in reverse complement relative to plots in File S5)

a)

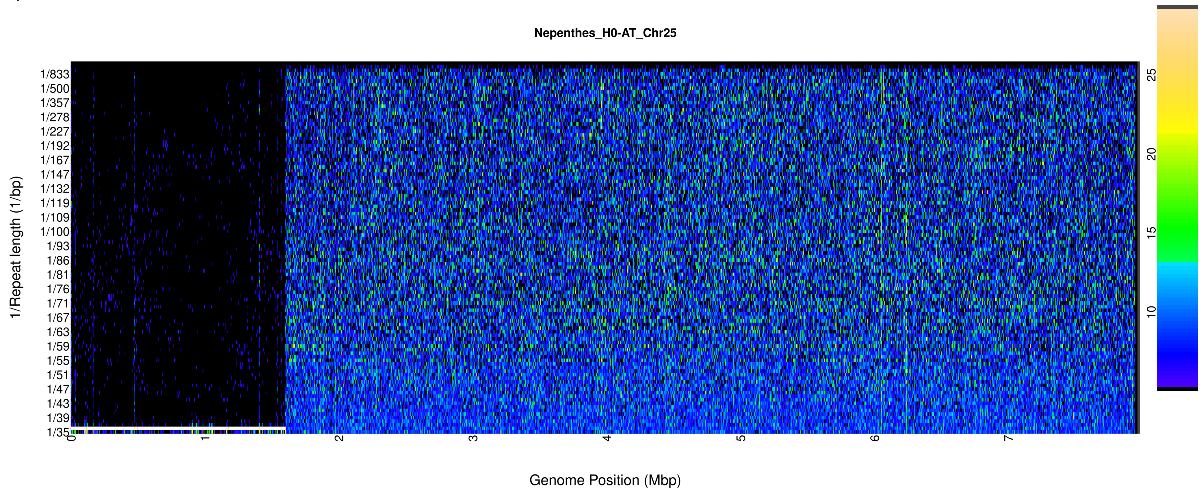

b)

Plot not produced by RepeatOBserverV1 software.

c)

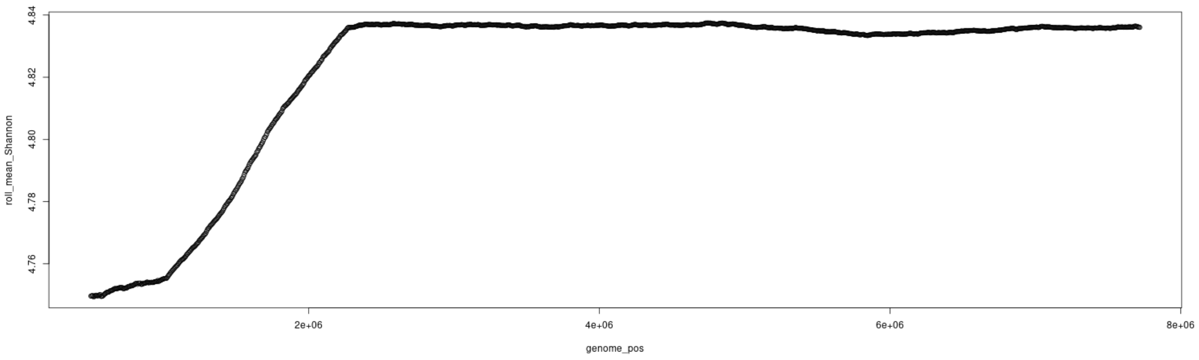

ptg000092l_1 (right contig, shown in reverse complement relative to plots in File S5)

a)

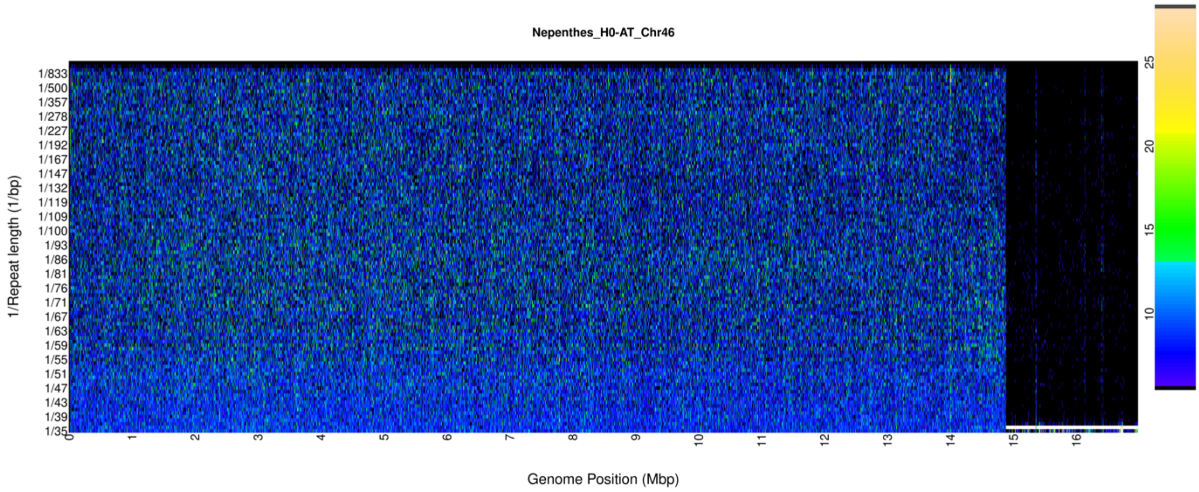

b)

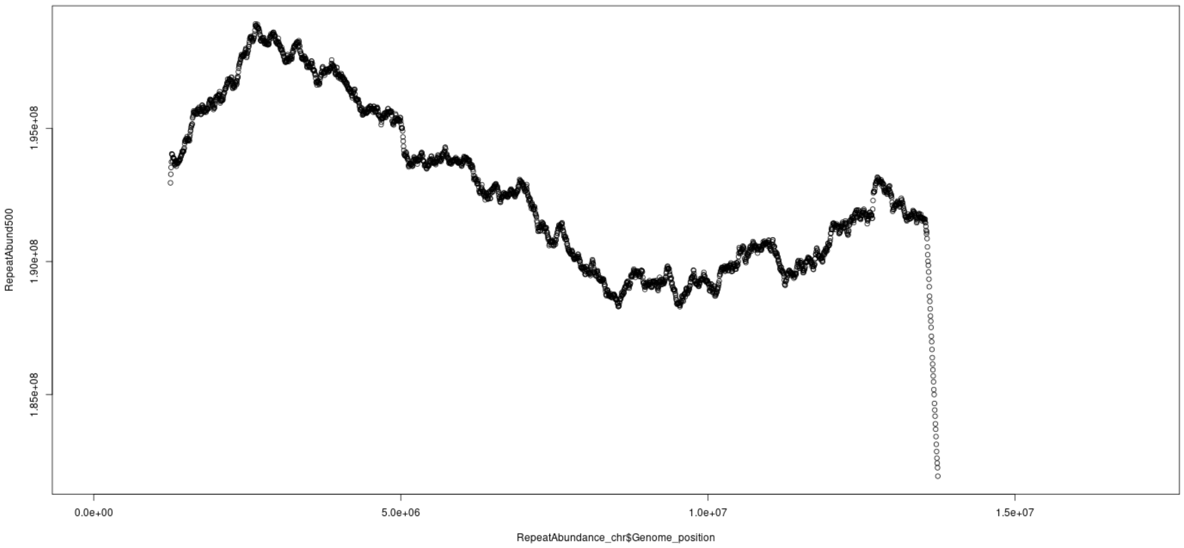

c)

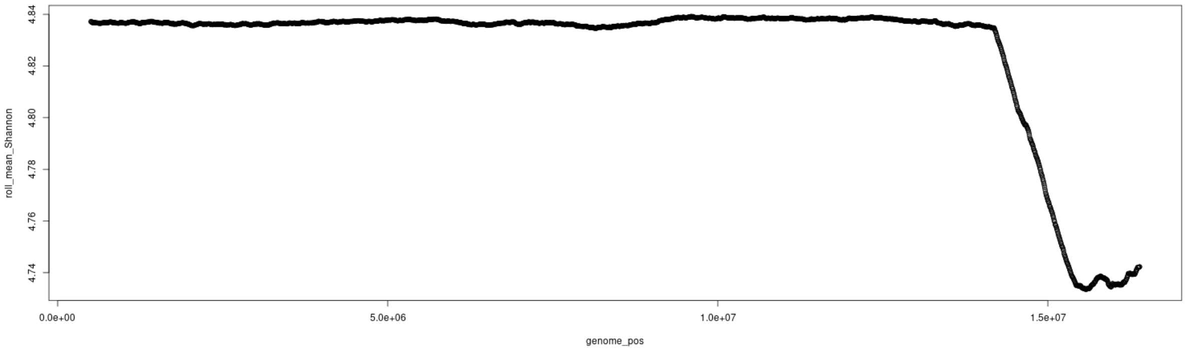

ptg000039l_1 (left contig)

a)

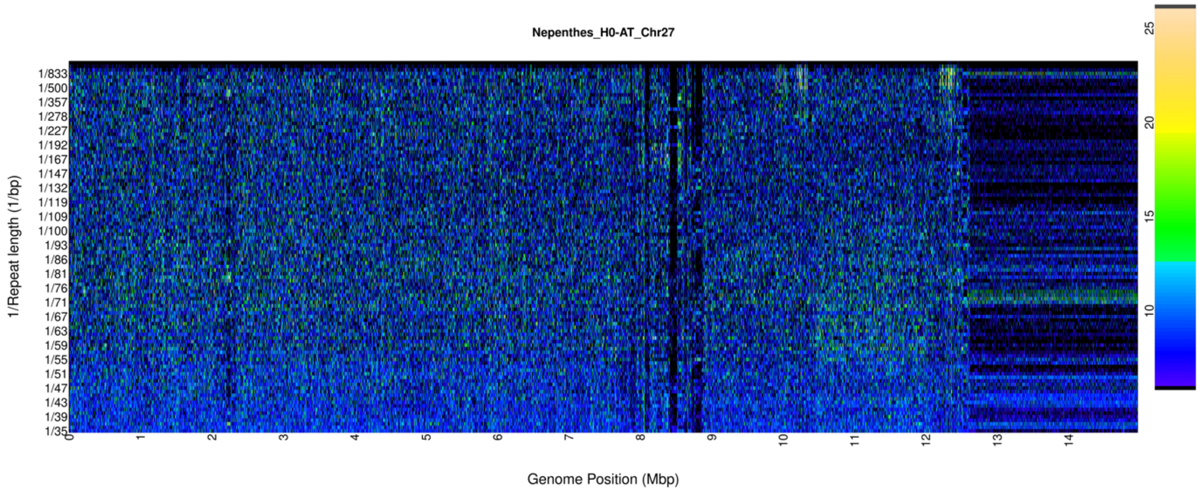

b)

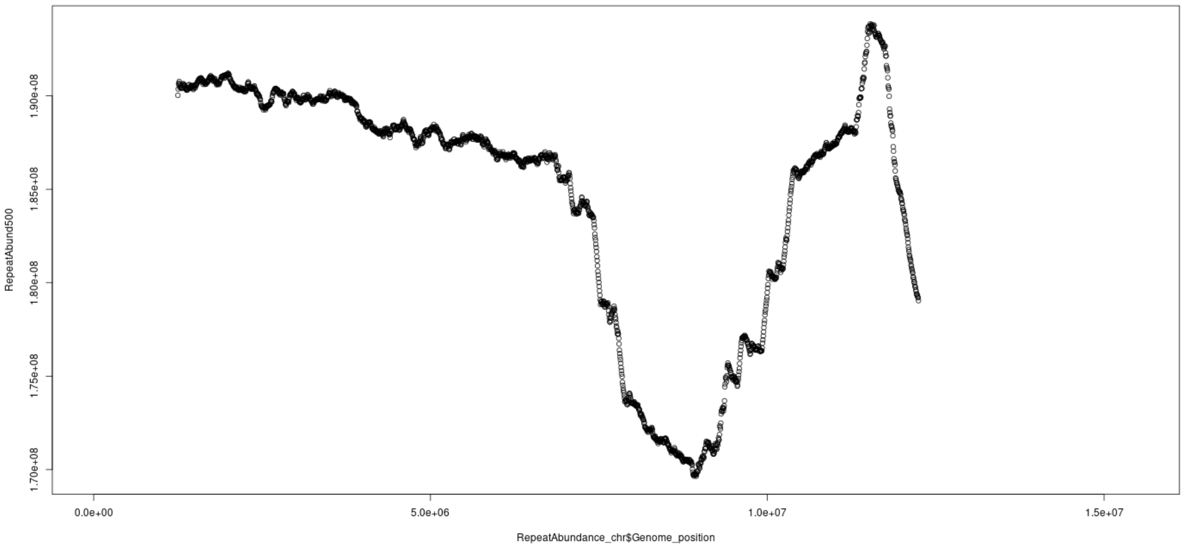

c)

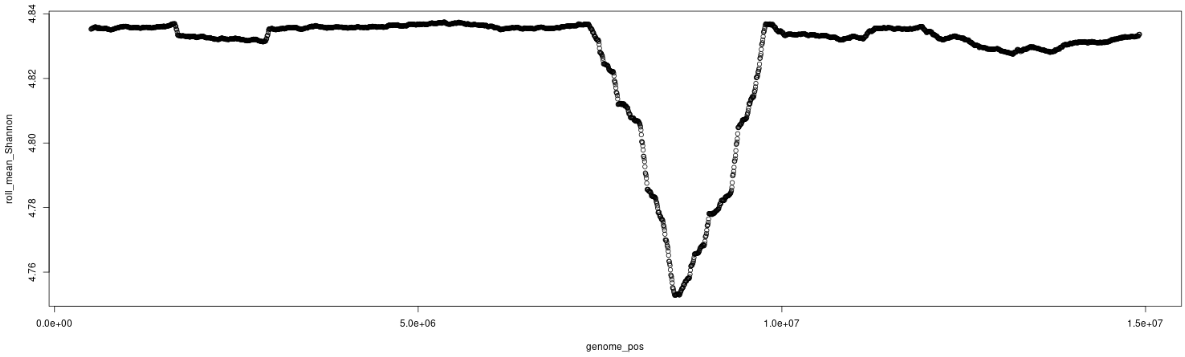

ptg000074l_1 (right contig, shown in reverse complement relative to plots in File S5)

a)

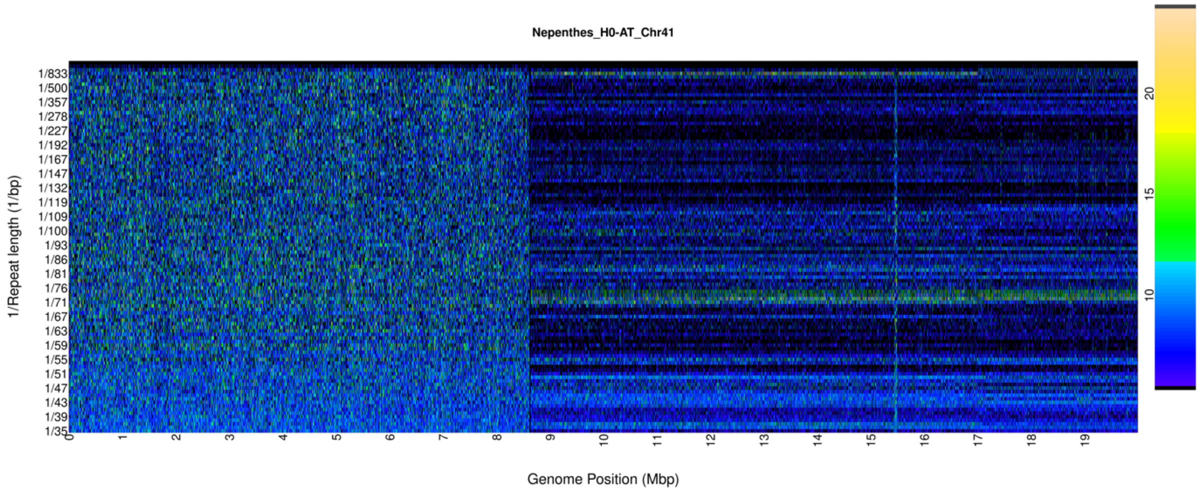

b)

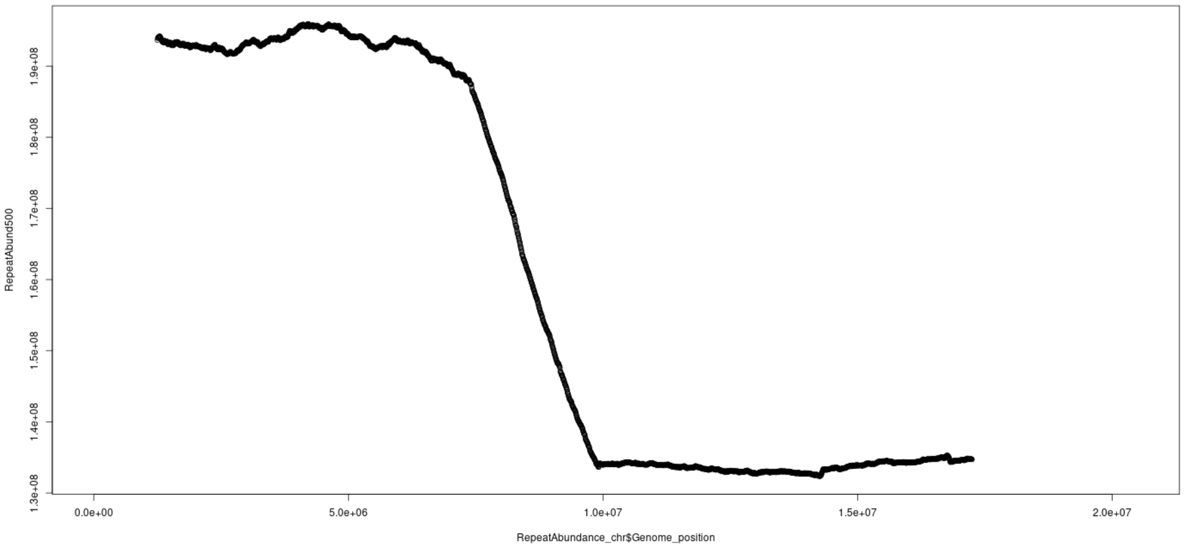

c)

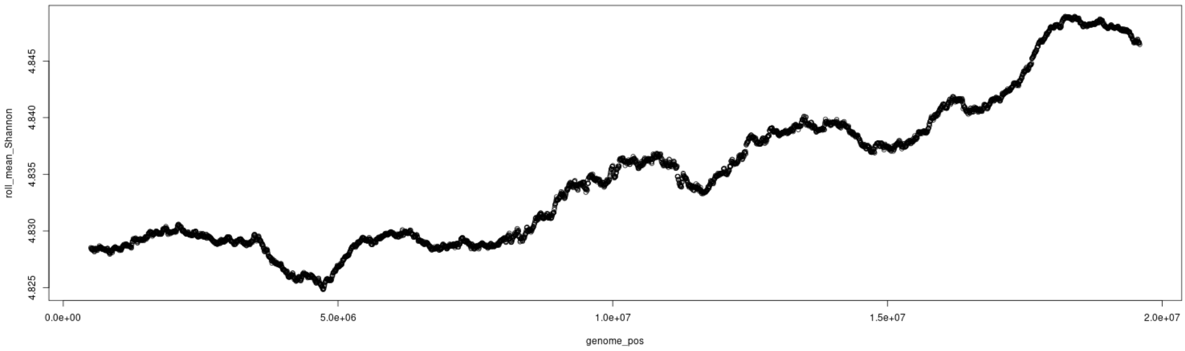

ptg000054l_1

a)

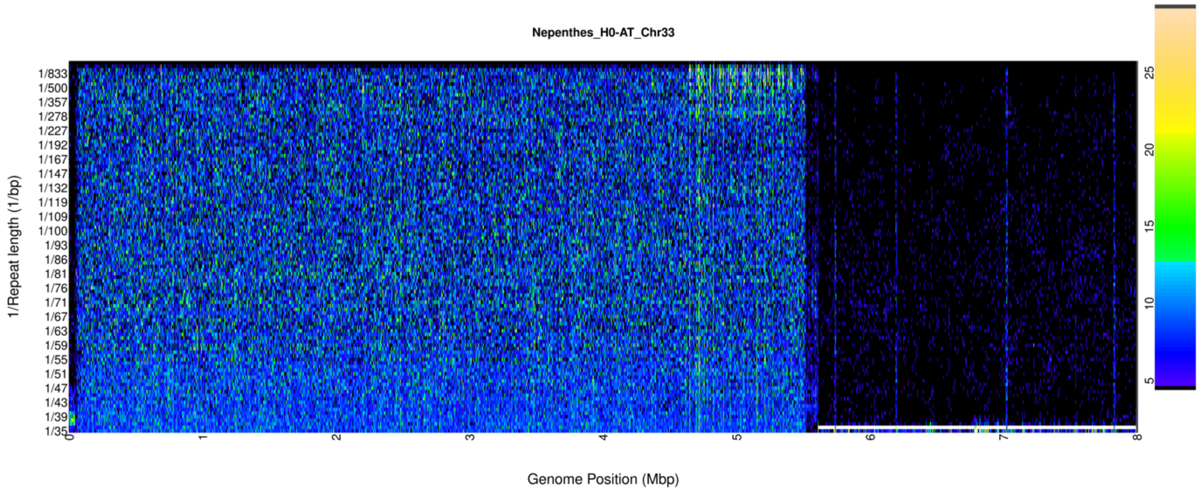

b)

Plot not produced by RepeatOBserverV1 software.

c)

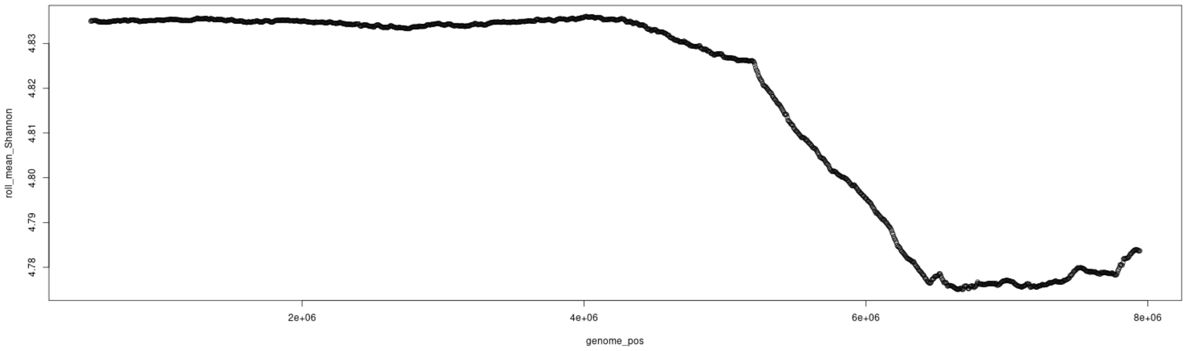

ptg000036l_1

a)

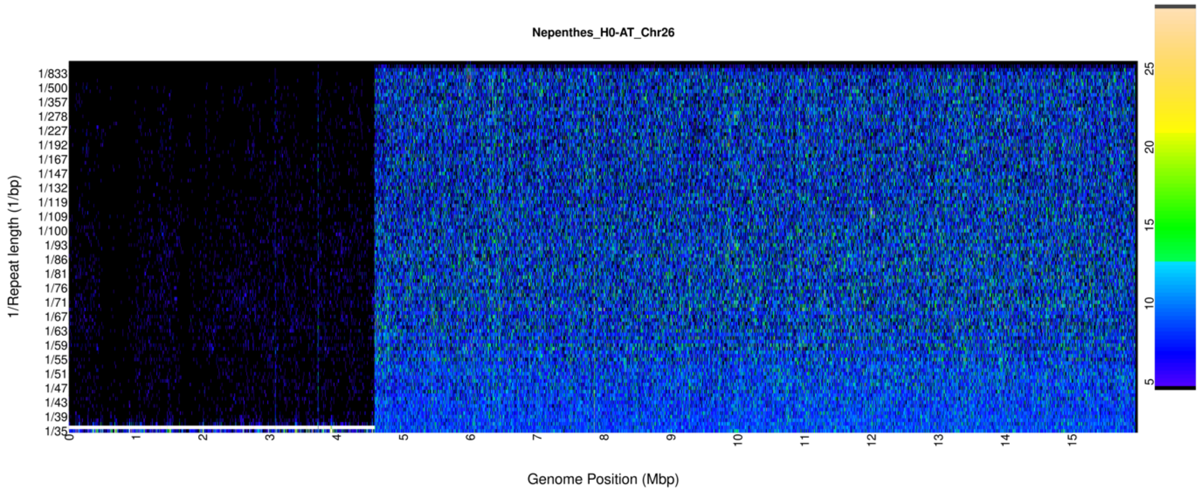

b)

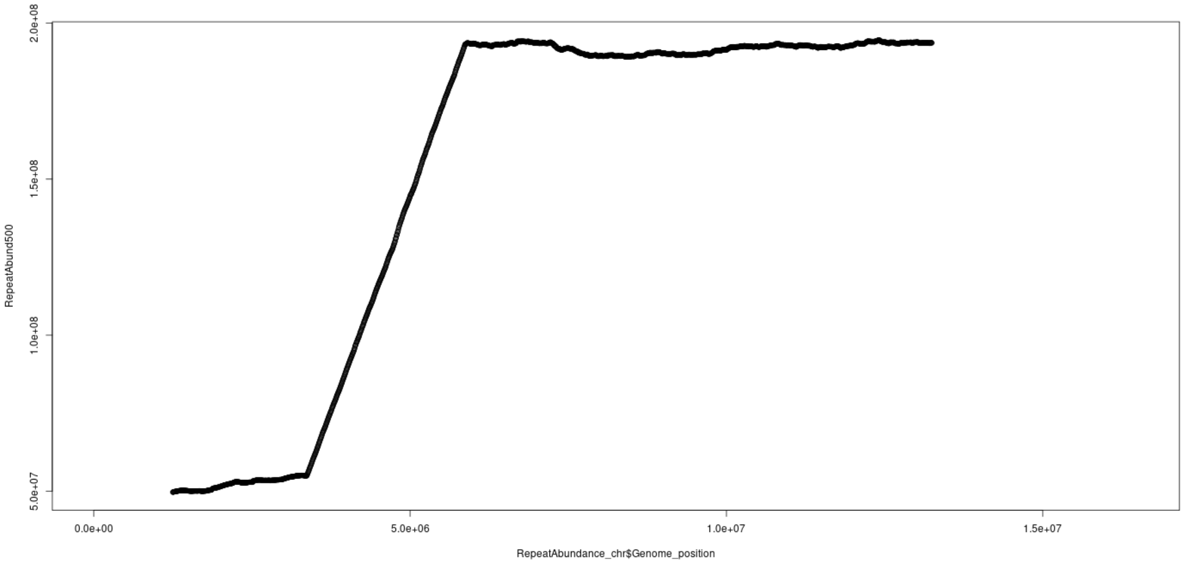

c)

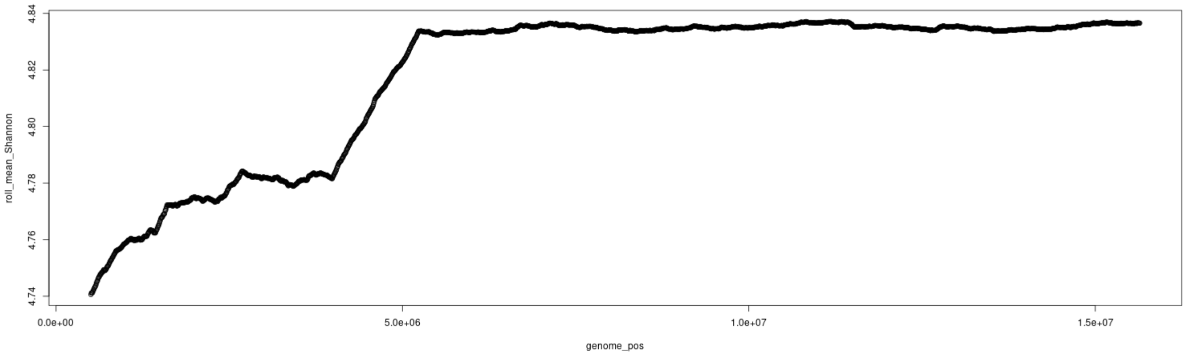

Supplement: S6 File — (PDF) [file pone.0322885.s006.pdf]
